# Lack of Association between* SLC30A8* Variants and Type 2 Diabetes in Mexican American Families

**DOI:** 10.1155/2016/6463214

**Published:** 2016-11-08

**Authors:** Hemant Kulkarni, Manju Mamtani, Juan Manuel Peralta, Vincent Diego, Thomas D. Dyer, Harald Goring, Laura Almasy, Michael C. Mahaney, Sarah Williams-Blangero, Ravindranath Duggirala, Joanne E. Curran, John Blangero

**Affiliations:** South Texas Diabetes and Obesity Institute, University of Texas Rio Grande Valley, Brownsville, TX, USA

## Abstract

*SLC30A8* encodes zinc transporter 8 which is involved in packaging and release of insulin. Evidence for the association of* SLC30A8* variants with type 2 diabetes (T2D) is inconclusive. We interrogated single nucleotide polymorphisms (SNPs) around* SLC30A8* for association with T2D in high-risk, pedigreed individuals from extended Mexican American families. This study of 118 SNPs within 50 kb of the* SLC30A8* locus tested the association with eight T2D-related traits at four levels: (i) each SNP using measured genotype approach (MGA); (ii) interaction of SNPs with age and sex; (iii) combinations of SNPs using Bayesian Quantitative Trait Nucleotide (BQTN) analyses; and (iv) entire gene locus using the gene burden test. Only one SNP (rs7817754) was significantly associated with incident T2D but a summary statistic based on all T2D-related traits identified 11 novel SNPs. Three SNPs and one SNP were weakly but interactively associated with age and sex, respectively. BQTN analyses could not demonstrate any informative combination of SNPs over MGA. Lastly, gene burden test results showed that at best the* SLC30A8* locus could account for only 1-2% of the variability in T2D-related traits. Our results indicate a lack of association of the* SLC30A8* SNPs with T2D in Mexican American families.

## 1. Introduction

Genome-wide association studies in humans and knockout studies in mice have increasingly pointed towards an important role of the ZnT8 zinc transporter in pathogenesis of type 2 diabetes (T2D) [1–6]. Since the publication of the first association reports in 2007, several other studies testing the association of the variants in the* SLC30A8* gene that encodes the ZnT8 transporter and the risk of T2D have been reported. A recent meta-analysis examined evidence for the association of the most intensely scrutinized polymorphism (rs13266634) in* SLC30A8* and found that the risk allele is associated with a 16.5% increased risk of T2D in a dose-dependent manner [7]. Corroborating the human research, studies in knockout mice have also demonstrated that the ZnT8 protein is vital in the process of *β*-cell secretion as well as hepatic clearance of insulin [5, 6].

There is a strong biological basis to the hypothesized association between* SLC30A8* variants and T2D. At the cellular level, a critical step in the release of insulin from *β* cells in the pancreas is proper packaging of proinsulin into the secretory granules [8]. This process is electrochemically facilitated and requires the presence of Zn^2+^ and Ca^2+^ ions which form complexes with hexamers of proinsulin in the secretory granules [9–11]. The Zn^2+^ ions required for this process are transported across electrical gradient by the zinc transporter 8 (ZnT8) protein [12]. This transporter is abundant in *β* cells but has also been observed in *α* cells that orchestrate the release of glucagon [13]. Together, the biological and implied clinical underpinnings place* SLC30A8* at a strategic position in the continued quest for identifying key drug targets to treat T2D.

Despite the perceived importance of this gene in T2D pathogenesis, however, observational evidence in this regard remains inconclusive. First, a large recent study demonstrated that loss-of-function mutations in* SLC30A8* afforded a surprising protection against T2D [14]. As a result, the exact mechanism by which* SLC30A8* may partake in T2D pathogenesis is unknown. Second, Cheng et al. [7] demonstrated in a recently published meta-analysis that the published studies associating rs13266634 with T2D are significantly heterogeneous (*I* ^2^ 62%, *p* < 0.001). This heterogeneity indicates that the results are unlikely to be generalizable. Indeed, ethnic differences among populations explain a substantial degree of this heterogeneity [7]. Third, Rutter and Chimienti [15] argue that factors such as age and hypoxic *β* cell stress can modify the association of* SLC30A8* variants with T2D. In the light of these and other [13, 16] contradictory findings, the role of* SLC30A8* in T2D is far from being well-understood.

Aside from one study [17] that formally tested for association of* SLC308A* variants with T2D-related traits, there is a paucity of data on this potential association in individuals with Mexican American ethnic background. We therefore sought to investigate the specific association of several single nucleotide polymorphisms (SNPs) in and around* SLC308A* in the high-risk settings of Mexican American families. Data for this study come from the high-resolution genotyping of pedigreed individuals recruited in the San Antonio Family Heart Study (SAFHS) [18–20]. Using rich genotyping data and robust statistical techniques suited for family studies, we demonstrate that the* SLC30A8* locus is not associated with differential T2D risk in the study population.

## 2. Materials and Methods

### 2.1. Study Participants

The initial SAFHS cohort consisted of 1,431 individuals from 42 large and extended pedigrees. Of these, high-density genotyping as well as other relevant phenotypic data was available for a maximum of 1,383 individuals. The SAFHS also included a longitudinal arm in which participants were followed up for development of incident T2D. Complete follow-up information was available on 913 individuals for a total follow-up of 11,049.92 person-years. The characteristics of the study population are shown in Table 1. The Institutional Review Board of the University of Texas Health Science Center at San Antonio approved the study. A written informed consent was obtained from all the study participants.

### 2.2. Phenotypic Traits

We included eight (two discrete and six continuous) phenotypic traits related to T2D. The discrete traits were: ever diabetes, defined as either presence of T2D at baseline or* de novo* development of T2D during follow-up, and incident diabetes, new cases of T2D during follow-up. For these traits, T2D was defined using the American Diabetes Association Clinical Practice Recommendations 2004 (fasting plasma glucose level ≥126 mg/dL [7.0 mmol/L], plasma glucose ≥200 mg/dL [11.1 mmol/L] at 2 h after oral glucose challenge, or both) [21]. Also, individuals were considered to have diabetes if they reported use of antidiabetic medication [22]. The six continuous traits were fasting glucose; fasting insulin; 2-hour postprandial glucose; 2-hour postprandial insulin; homeostatic model of assessment-insulin resistance (HOMA-IR); and homeostatic model of assessment, beta (HOMA-*β*). HOMA-IR values were calculated according to the formula (fasting glucose [mmol/L] × fasting insulin [*μ*U/mL]/22.5), while HOMA-*β* was calculated as (20x fasting insulin [*μ*U/mL])/(fasting glucose [mmol/L] − 3.5) [23]. Additional clinical variables measured were age, sex, waist circumference, body mass index, systolic and diastolic blood pressure, fasting and 2-hour plasma glucose, fasting insulin, total serum cholesterol, serum triglycerides, high-density lipoprotein (HDL) cholesterol, and use of lipid-lowering and antihypertensive drugs. Methods used to measure these variables have been described in detail previously [18–20].

### 2.3. High-Density Genotyping

Study participants were previously genotyped for approximately 995,321 SNP markers using several Illumina genotyping arrays, including the HumanHap550v3, HumanExon510Sv1, Human1Mv1, and Human1M-Duov3. Details of the data cleaning and imputing steps for the genotypic data have been detailed elsewhere [24]. We used all the SNPs within the* SLC30A8* gene as well those within 50 kb upstream and downstream of this gene. A total of 118 SNPs were found in this region. Detailed characteristics along with genomic locations of these 118 SNPs are provided in Supplementary Table  1 in Supplementary Material available online at http://dx.doi.org/10.1155/2016/6463214. The variants were annotated using ANNOVAR [25] that used human genome Build 19 and SNP version 138 databases for annotation.

### 2.4. Statistical Analyses

We conducted the genotype-phenotype associations at four levels: each SNP considered one at a time; potential interaction of the SNPs with age and sex; most informative combinations of significantly associated SNPs and association burden associated with the entire locus. All associations were tested under the framework of the variance components that allows partitioning of the total phenotypic variance into components of interest while accounting for kinship among individuals.

#### 2.4.1. Measured Genotype Analyses (MGA)

To evaluate the association of each SNP with T2D-related traits we used the MGA approach. This approach assumes that the likelihood of observing measured genotype of a single locus and the phenotypes within a family is a function of the measured genotypes times the conditional likelihood of the phenotypes [26]. To correct for multiple testing we used the method of Li and Ji [27] since there was a substantial degree of linkage disequilibrium among these SNPs (Supplementary Figure  1). In all association analyses, ever diabetes and incident diabetes were used as discrete traits while all the continuous traits were inverse-normalized to ensure normal distribution with a mean of zero and standard deviation of unity. All association models were also adjusted for age, age_2_, sex, age × sex interaction, age^2^  ×  sex interaction, and top four principal components that captured ancestry-based population admixture. To detect a pattern of associations of the SNPs with all the T2D-related traits, we computed a summary probability score (PS) defined as ∑_*t*_−log_10_⁡*p*, where *t* is the phenotypic trait and *p* is the significance value.

#### 2.4.2. Interactions of SNPs with Age and Sex

Interaction of each SNP with age and sex was conducted using a polygenic modeling approach. In these models, age was binarized based on <45 yrs or ≥45 yrs and sex was used as two nominal categories. Interactive models used SNP dosages multiplied by age and sex, respectively. Statistical significance for interaction terms was evaluated by constraining the interaction term to zero and calculating likelihood ratio statistic as two times the difference in the log-likelihoods of the constrained and unconstrained models. Statistical significance was tested at a liberal global type 1 error rate of 0.2 before applying the correction for multiple testing using the method of Li and Ji [27].

#### 2.4.3. Bayesian Quantitative Trait Nucleotide (BQTN) Analyses

Finding important combinations of key SNPs for the T2D-related traits was facilitated using the Bayesian Quantitative Trait Nucleotide Analyses. The BQTN model developed by Blangero et al. [28] is a Bayesian one that uses the underlying measured genotype model and conducts joint analysis of multiple variants. It evaluates a series of combinations of the candidate SNPs by comparing them to a base model. Typically, if there are *s* SNPs being evaluated then the total number of models tested is 2^*s*^. Selection of the best model is accomplished using the Bayesian Information Criterion (BIC) which is defined for *k*th model as BIC_*k*_ = −∧_*k*0_ + df_*k*_ln⁡*N*_*e*_. In this equation, ∧_*k*0_ is the likelihood ratio test statistic comparing the QTN model with the null model; df_*k*_ are the degrees of freedom and *N*_*e*_ is the effective sample size. The model with least BIC is considered as the best model.

#### 2.4.4. Gene Burden Test

To quantify the contribution of all the SLC30A8-associated SNPs to T2D-related traits, we made use of the gene burden test [29]. For using this procedure, we first transformed gene-specific polymorphism dosages into a covariance matrix and converted this to a gene-specific matrix of empirical kinship coefficients. This matrix was used to extract the contribution to overall phenotypic variance using the following equation: Ω = *σ*_Phenotypic_^2^(2Φ*h*_*r*_^2^ + 2**E** *h*_geff_^2^ + **I** *e* ^2^). In this equation, Ω is the covariance matrix; *σ*_Phenotypic_^2^ is the total phenotypic variance of the trait; Φ and **E** represent matrices of theoretical and empirical kinship expectations, respectively; *h*_*r*_^2^, *h*_geff_^2^, and *e* ^2^ represent the proportion of phenotypic variance explained by residual additive effect of polygenes, a gene-specific effect and a random environmental effect, respectively; and **I** is the identity matrix. The significance of the *h*_geff_^2^ component was tested using a likelihood ratio test statistic that is distributed as a 1/2 : 1/2 mixture of a 1 degree of freedom chi-square and a point mass at zero.

## 3. Results

### 3.1. Study Participants

We included a total of 1,383 individuals from the SAFHS on whom genotypic and phenotypic information was available. The characteristics of the study participants are summarized in Table 1. Briefly, the mean age of the participants was 39 years and the majority of them (60%) were females. At baseline, 15% had T2D, 18% were hypertensive, ~39% were obese, and ~47% had central obesity. During follow-up of 913 individuals who were initially free of T2D and for whom complete follow-up data was available, 149 new cases of T2D were detected. Thus, a total of 357 (~26%) individuals either had T2D at baseline or developed it during follow-up. This trait was dubbed “ever T2D” for the purpose of the present study. The average fasting and 2-hour postprandial insulin values as well as the HOMA-IR and HOMA-*β* values indicated that, even in individuals who did not have T2D at baseline, there was a high likelihood of insulin resistance (Table 1). Approximately 10% and 2% participants were using antihypertensive and lipid-lowering drugs at baseline, respectively.

### 3.2. SNPs around and within* SLC30A8*

We included a total of 118 SNPs contained within a 50 kb region straddling* SLC30A8*. These polymorphisms displayed a wide range of minor allele frequencies from 0.007 to 0.4989 (Supplementary Table  1). In all, 10 (8.47%), 13 (11.02%), and 95 (80.51%) SNPs were found to have a minor allele frequency of <1%, 1–5%, and >5%, respectively. All of the included SNPs had call rates exceeding 99% and none of them were in significant Hardy-Weinberg disequilibrium (defined as *p* < 0.001). The most significant departure from Hardy-Weinberg equilibrium was observed for the rs7000505 SNP with a *p* value of 0.0114. Thus, the SNPs included in this study were fairly common in the Mexican American families and were genotyped with acceptable levels of errors. In the context of* SLC30A8*, only two of the included SNPs (rs1326634 and rs16889462) were in the coding region and resulted in a nonsynonymous change at the level of protein. All other SNPs were either intronic or intergenic. In general, there was strong linkage disequilibrium among the included SNPs (Supplementary Figure  1). Using Li and Ji's method, we estimated that the 118 SNPs represented only 49 independent SNPs conditional on the linkage disequilibrium pattern.

### 3.3. Association of Each* SLC30A8* SNP with T2D-Related Traits

**Figure 1:**
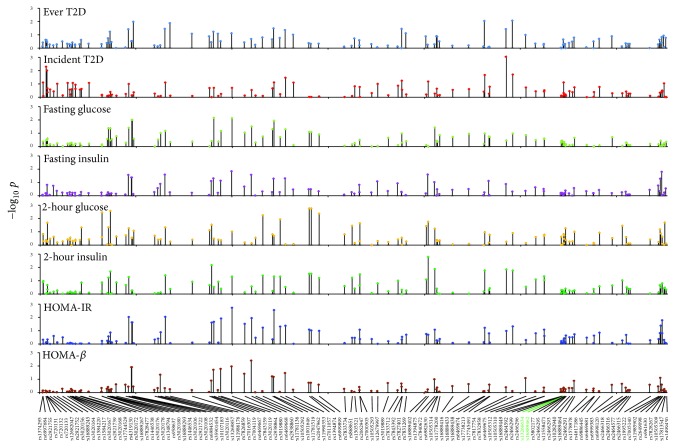
Association of 118 SNPs within and around SLC30A8 with T2D-related traits. Abscissa shows the names and locations of the SNPs on chromosome 8 and the ordinate shows statistical significance. After correcting for 49 independent tests, we need a −log_10_⁡*p* value of 2.99 for statistical significance at a global type I error rate of 0.05 for each trait. Full quantitative results are provided in Supplementary Table  1. Coding variants are indicated in green.

The first step in the association analyses was to interrogate the association of each SNP with phenotypic traits related to T2D. Associations were tested using a measured genotype approach. The results of the analyses are summarized in Figure 1 and detailed in Supplementary Table  1. After correcting for 49 independent tests, we needed a significance value of 0.001 (−log_10_⁡*p* = 3.00) that corresponded with a global type I error rate of 0.05 for each phenotypic trait studied. All the models were adjusted for age, sex, their first and second degree interactions, and the top four principal components to capture potential population admixture.

We observed (Figure 1, Supplementary Table  1) that, aside from the rs7817754 SNP that was significantly associated with incident T2D, no other SNP achieved statistical significance for association with any of the phenotypic traits studied. The most significant association with the other phenotypic traits were as follows: ever T2D, rs7832958 (*p* = 0.0086); fasting glucose, rs6469667 (*p* = 0.0071); fasting insulin, rs2938864 (*p* = 0.0143); 2-hour glucose, rs1394874 (*p* = 0.0018); 2-hour insulin, rs1394875 (*p* = 0.0016); HOMA-IR, rs2938864 (*p* = 0.0019); and HOMA-*β*, rs6469668 (*p* = 0.0040). These results indicated weak and apparently inconsistent associations of the studied SNPs when considered individually with T2D-related traits.

**Figure 2:**
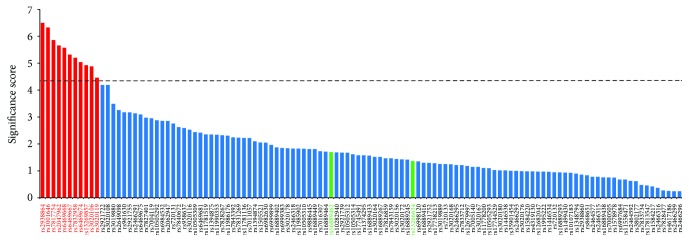
Probability score for the studied SNPs based on strength of association with eight T2D-related traits. Significantly associated SNPs are indicated in red colored bars. Coding variants are labeled in green.

We therefore considered the association of each SNP with all the T2D-related traits using the PS. Given eight traits, 118 SNPs, and a stringent probability criterion of 0.01 we needed a minimum PS of 4.3010 for statistical significance. We observed (Figure 2) that a total of 11 SNPs passed this criterion and showed a somewhat consistent pattern of associations with the T2D-related traits. These SNPs were (in order of significance) rs2938864, rs1001646, rs7817754, rs2047962, rs6469668, rs6469667, rs7832958, rs6469674, rs13269857, rs3020106, and rs3020119. In the rest of the paper, we refer to these 11 SNPs as the most significantly associated (MSA) SNPs. Of note, five (rs2938864, rs7817754, rs6469668, rs6469667, and rs7832958) of the seven top hits enlisted above were included in the list of MSA SNPs. However, the list of MSA SNPs did not include the two coding variants associated with* SLC30A8*.

### 3.4. Interaction of Genetic Variants with Age and Sex

We investigated if the association of SNPs with T2D-related traits was interactively altered by age and sex. The results based on a series of interactive multivariable models are summarized in Table 2 and details are provided in Supplementary Table  2. As is conventional in interactive models, we used a relaxed probability criterion of 0.2 to detect evidence of interaction and corrected it for 49 independent tests for each phenotypic trait. We found that three SNPs (rs7843392, rs11985902, and rs17813547) showed significant interaction with age in regard to ever T2D, incident T2D, and 2-hour glucose, respectively. To understand the direction of these interactions we conducted subgroup analyses within the age and sex strata as shown in Table 2. The rs7843392 SNP was almost significantly associated with a higher risk of ever T2D only in those aged ≥45 years. On the other hand, rs11985902 and rs17813547 polymorphisms were significantly associated with a significantly increased risk of incident T2D and higher values of 2-hour glucose in those aged <45 years. Interestingly, the rs11985902 polymorphism demonstrated a marginally significant reduction in the risk of incident T2D in those aged ≥45 years. Lastly, the rs2062947 polymorphism significantly interacted with sex in the context of ever T2D and fasting glucose. In both instances, the polymorphism was associated with a reduced risk in males. Summarily, these results from interaction analyses demonstrated that only four (3.38%) polymorphisms showed significant interactions with age and sex and none of these four SNPs was included in the MSA SNPs.

### 3.5. Combinatorial Associations of SNPs with T2D-Related Traits: The BQTN Analyses

We conducted the BQTN analyses on the MSA SNPs identified in the previous step. For each T2D-related trait, we thus evaluated a total of 2^11^ = 2,048 multivariable models (total 16,384 models for eight traits) that examined all the combinations of the SNPs. These results are summarized in Table 3 and described in detail in Supplementary Tables  3–10. Our results showed that inclusion of one or more SNPs did not reduce the BIC for ever T2D, incident T2D, fasting glucose, 2-hour glucose, and 2-hour insulin. For fasting glucose, HOMA-IR, and HOMA-*β*, addition of only one SNP (rs2938864, rs2938864, and rs6469667, resp.) was associated with a reduced BIC. However, the probability estimates associated with a reduced BIC indicated that the reduction was not statistically significant. No other combination improved the BIC.

### 3.6. Gene Burden Tests for the* SLC30A8* Locus

As a final step in analyses, we examined if joint associations of the studied variants proffer a significant association of the SLC30A8 as a whole with the T2D-related traits. In order to maximize the association signal, we constructed three genetic kinship matrices that were based on genotyping data from (i) all the 118 SNPs included in this study; (ii) only the two coding variants; and (iii) the MSA SNPs.  Table 4 shows the results of these analyses. As expected, maximum association signal was seen when we used the 11 MSA SNPs. The signal was substantially diminished when the analyses were based on all the 118 SNPs. Best estimates of the variance component that captured the gene burden effects (columns titled *h*_geff_^2^ in Table 4) were seen for incident T2D based on all the 118 SNPs. However, no estimate of the variance component was statistically significant. These analyses demonstrated that the variants within and around the* SLC30A8* gene did not provide an association burden to the locus in relation to any of the eight phenotypic traits studied here.

## 4. Discussion

The current enthusiasm in the putative role of* SLC30A8* in the pathogenesis of T2D is driven by biological plausibility as well as association results from large genetic epidemiologic studies in humans. However, the strength of evidence and the generalizability of the associative observations is currently unclear. Our study in large pedigrees of Mexican American individuals in San Antonio, Texas, failed to demonstrate strong or significant patterns of association between* SLC30A8* SNPs and several phenotypic traits related to T2D. The only other study in Mexican Americans from the Arizona Insulin Resistance Registry also could not find significant association between* SLC30A8* SNPs and T2D [17]. Together, these studies tend to indicate that* SLC30A8* variants are unlikely to be consistently associated with T2D across different ethnic/racial backgrounds. It is instructive in this regard that Cheng et al. [7] as well as Cauchi et al. [16] found ethnic background to be an important contributor to the between-studies heterogeneity in observed association of the coding variant rs1326634 with T2D. Our results therefore proffer a possible but partial explanation for the limited generalizability of the association between* SLC30A8* variants and T2D. Of note, none of the 11 MSA SNPs found to be marginally but significantly associated with T2D-related traits in our studies have been reported to be associated with disease phenotypes in the ClinVar database (http://www.ncbi.nlm.nih.gov/clinvar/).

The overall lack of association needs to be considered in the light of several key aspects related to the* SLC30A8*-T2D nexus. First, Cauchi et al. [16] using data from 32 published studies have shown that the coding variant rs13266634 does not influence the expression of* SLC30A8* in humans. In contrast, data from over 150,000 individuals in the T2D-GENES consortium [14] shows that rare, loss-of-function variants that are associated with a significantly reduced expression of* SLC30A8* are also associated with a reduced risk of T2D across various ethnicities. These confusing findings are difficult to reconcile. Second, Rungby [30] hypothesizes that other factors such as age and sex might confound genotype-phenotype associations. This hypothesis is supported by elegant studies in mice [31–34]. Chang et al. have also recently demonstrated that, in Chinese Han populations, there is a significant interaction of the rs1366634 polymorphism with age [35]. In our study we found a negligible interaction of the* SLC30A8* associations with age and sex. The reasons for these potential interactions and the implication of these interactions in terms of disease pathogenesis are currently unknown. Third, there is evidence to show that* SLC30A8* expression correlates significantly with the secretion of both insulin and glucagon [6, 13, 16, 30]. Since insulin and glucagon have opposite actions on glycemic control, it is conceivable that the association of an upstream regulator (such as* SLC30A8*) with the overall pathogenesis would be difficult to detect since its effects on both insulin and glucagon might partially nullify each other. Fourth, several studies have demonstrated that there are significant gene-gene and gene-environment interactions associated with* SLC30A8* that can all mask association of this gene with T2D [36–42]. In total, these issues make demonstration of association of* SLC30A8* variants with T2D both questionable and difficult to show even if existent.

Before interpreting our results, however, the limitations of our study must be recognized. First, we used data on 118 SNPs available from various Illumina genotyping platforms. It is not known whether this coverage adequately captures the* SLC30A8* locus. However, the fact that the most intensely scrutinized* SLC30A8* polymorphism (rs13266634) was not significantly associated with any T2D-related trait in our study population provides an indirect measure of internal consistency of our results. Second, we do not have data on the expression of* SLC30A8* in *β* cells and on the ZnT8 isoforms and their expression in the study population. Therefore we cannot comment on the functional importance of the 11 MSA SNPs. Third, our BQTN analyses were extensive but not exhaustive since it is practically impossible to test for all the 2^118^ combinations of the included SNPs. We may have thus missed some significant SNP-SNP interactions but our results show that the MSA SNPs were not involved in T2D pathogenesis in a combinatorial fashion.

Our results have important implications in the continued quest to conquer T2D.* SLC30A8* is an attractive drug target since development of inhibitors that will reduce the gene expression is now being pursued as a likely important discovery [14, 42]. However, whether such interventions will work in most scenarios will depend on the generalizability of the results. Our results seem to point towards the possibility that at least the high-risk Mexican American individuals are unlikely to benefit by an intervention that is based on inhibition of* SLC30A8*. Even if our interpretations were to be based on the 11 MSA SNPs, we find that only 1-2% of the variability in T2D-related traits may be attributable to the* SLC30A8* locus (Table 4). These findings beckon that a comprehensive understanding of the role of* SLC30A8* is needed before an interventional leap based on this gene is considered to prevent or treat T2D.

## Supplementary Material

Contains one Supplementary Figure (linkage disequilibrium plot) and 10 Supplementary Tables which provide detailed results from the MGA (Supplementary Tables 1 and 2) and BQTN (Supplementary Tables 3-10, one table for each T2D-related trait) analyses.

## Figures and Tables

**Table 1:** Clinical characteristics of the SAFHS included in this study (*n* = 1, 383).

Characteristic	*N* ^*∗*^	Description^*∗∗*^
Age at enrolment (y)	1,383	39.17 (0.45)
Females	1,383	822 (59.43)
Waist (cm)	1,371	94.71 (0.47)
Central obesity (waist circumference ≥102 cm for males and ≥88 cm for females)	1,371	641 (46.75)
Body mass index (BMI, Kg/m^2^)	1,372	29.24 (0.18)
Obesity (BMI ≥ 30 Kg/m^2^)	1,372	531 (38.70)
Systolic blood pressure (SBP, mmHg)	1,372	120.38 (0.51)
Diastolic blood pressure (DBP, mmHg)	1,372	70.67 (0.28)
Hypertension (SBP ≥ 140 mmHg and/or DBP ≥ 90 mmHg)	1,372	247 (18.00)
Type 2 diabetes		169 (20.58)
Prevalent T2D at baseline visit	1,383	208 (15.04)
Incident T2D detected during follow-up^†^	913	149 (16.32)
Ever T2D	1,383	357 (25.81)
Fasting glucose (mmol/L)^†^	1,171	4.82 (0.02)
2-hour postchallenge glucose (mmol/L)^†^	1,143	5.61 (0.05)
Fasting insulin^†^	1,153	14.11 (0.46)
2-hour postchallenge insulin^†^	1,115	76.86 (2.19)
HOMA-IR^†^	1,153	3.16 (0.13)
HOMA-*β* ^†^	1,153	19.38 (1.67)
Total serum cholesterol (mg/dL)	1,379	189.27 (1.06)
Serum triglycerides (mg/dL)	1,379	150.10 (3.48)
HDL cholesterol (mg/dL)	1,378	50.14 (0.35)
Medication use at baseline		
Antihypertensive medications	1,376	132 (9.59)
Lipid lowering medications	1,376	25 (1.82)

^*∗*^Number of individuals for whom data was available.

^*∗∗*^Numbers indicate mean (SE) for continuous variables and *n* (%) for categorical variables.

^†^These variables are reported only for individuals who did not have T2D at baseline.

**Table 2:** *SLC30A8*-related SNPs showing significant interaction with age or sex.

SNP	Trait	Interaction with	Interaction *p*	Subgroup analyses			
				Group	*N*	*B*	*p*
rs7843392	Ever T2D	Age	0.0017	Age < 45 y	903	−0.45	0.3490
				Age ≥ 45 y	480	1.16	0.0541
rs11985902	Incident T2D	Age	0.0015	Age < 45 y	690	0.20	0.0334
				Age ≥ 45 y	223	−0.28	0.0579
rs17813547	2-hour glucose	Age	0.0024	Age < 45 y	837	0.17	0.0005
				Age ≥ 45 y	305	−0.04	0.5832
rs2062947	Ever T2D	Sex	0.0038	Males	561	−0.47	0.0044
				Females	822	0.08	0.4882
rs2062947	Fasting glucose	Sex	0.0031	Males	481	−0.16	0.0745
				Females	689	0.19	0.0071

**Table 3:** Results of BQTN analyses for potential combinatorial associations of SLC30A8-related SNPS with T2D-related traits.

Characteristic	eT2D	iT2D	FG	FI	2hG	2hI	HOMA-IR	HOMA-*β*
*h*_*r*_^2^	0.65	0.66	0.42	0.42	0.37	0.32	0.40	0.26
BIC of best model compared to the null model	0.00	0.00	0.00	−0.34	0.00	0.00	−1.75	−1.49
Number of models in window	0	0	0	1	0	0	1	1
SNPS in the best model (*p*)								
rs2938864	—	—		−0.06	—	—	−0.08	—
				(0.54)			(0.71)	
rs1001646	—	—	—	—	—	—	—	—
rs7817754	—	—	—	—	—	—	—	—
rs2047962	—	—	—	—	—	—	—	—
rs6469668	—	—	—	—	—	—	—	0.07
								(0.68)
rs6469667	—	—	—	—	—	—	—	—
rs7832958	—	—	—	—	—	—	—	—
rs6469674	—	—	—	—	—	—	—	—
rs13269857	—	—	—	—	—	—	—	—
rs3020106	—	—	—	—	—	—	—	—
rs3020119	—	—	—	—	—	—	—	—

eT2D, ever T2D; iT2D, incident T2D; FG, fasting glucose; FI, fasting insulin; 2hG, 2-hour glucose; 2hI, 2-hour insulin; BIC, Bayesian information criterion.

**Table 4:** Gene burden test for the association of the *SLC30A8* locus with T2D-related traits.

Trait	Based on all 118 SNPs			Based on 2 coding SNPs			Based on 11 MSA SNPs		
	*h*_geff_^2^	SE	*p*	*h*_geff_^2^	SE	*p*	*h*_geff_^2^	SE	*p*
Ever T2D	0.0000	0.0000	1.0000	0.0005	0.0028	0.8498	0.0080	0.0112	0.4774
Incident T2D	0.0000	0.0000	1.0000	0.0010	0.0052	0.8413	0.0192	0.0223	0.3902
Fasting glucose	0.0000	0.0000	1.0000	0.0000	0.0000	1.0000	0.0047	0.0055	0.3946
Fasting insulin	0.0000	0.0000	1.0000	0.0000	0.0000	1.0000	0.0031	0.0043	0.4720
2-hour glucose	0.0093	0.0101	0.3600	0.0006	0.0023	0.8055	0.0039	0.0055	0.4804
2-hour insulin	0.0037	0.0067	0.5791	0.0000	0.0000	1.0000	0.0021	0.0043	0.6288
HOMA-IR	0.0000	0.0000	1.0000	0.0000	0.0000	1.0000	0.0044	0.0051	0.3898
HOMA-*β*	0.0000	0.0000	1.0000	0.0000	0.0000	1.0000	0.0057	0.0057	0.4102

## Abbreviations

BIC: Bayesian information criterion
BQTN: Bayesian Quantitative Trait Nucleotide
HDL: High-density lipoprotein
HOMA-IR: Homeostatic model of assessment-insulin resistance
HOMA-*β*: Homeostatic model of assessment-*β*
MGA: Measured genotype approach
MSA: Most significantly associated
PS: Probability score
SAFHS: San Antonio Family Heart Study
SNP: Single nucleotide polymorphism
T2D: Type 2 diabetes
ZnT8: Zinc transporter 8.
