# Supplementary material for: Lack of Association between SLC30A8 Variants and Type 2 Diabetes in Mexican American Families
Source: J Diabetes Res. 2016 Nov 8;2016:6463214. doi: 10.1155/2016/6463214 (PMC5118530; doi:10.1155/2016/6463214)
Supplement: Supplementary file 1 — Contains one Supplementary Figure (linkage disequilibrium plot) and 10 Supplementary Tables which provide detailed results from the MGA (Supplementary Tables 1 and 2) and BQTN (Supplementary Tables 3-10, one table for each T2D-related trait) analyses. [file 6463214.f1.pdf]

Supplementary Figure 1. Linkage disequilibrium among the studied SNPs (those in green are exonic SNPs)

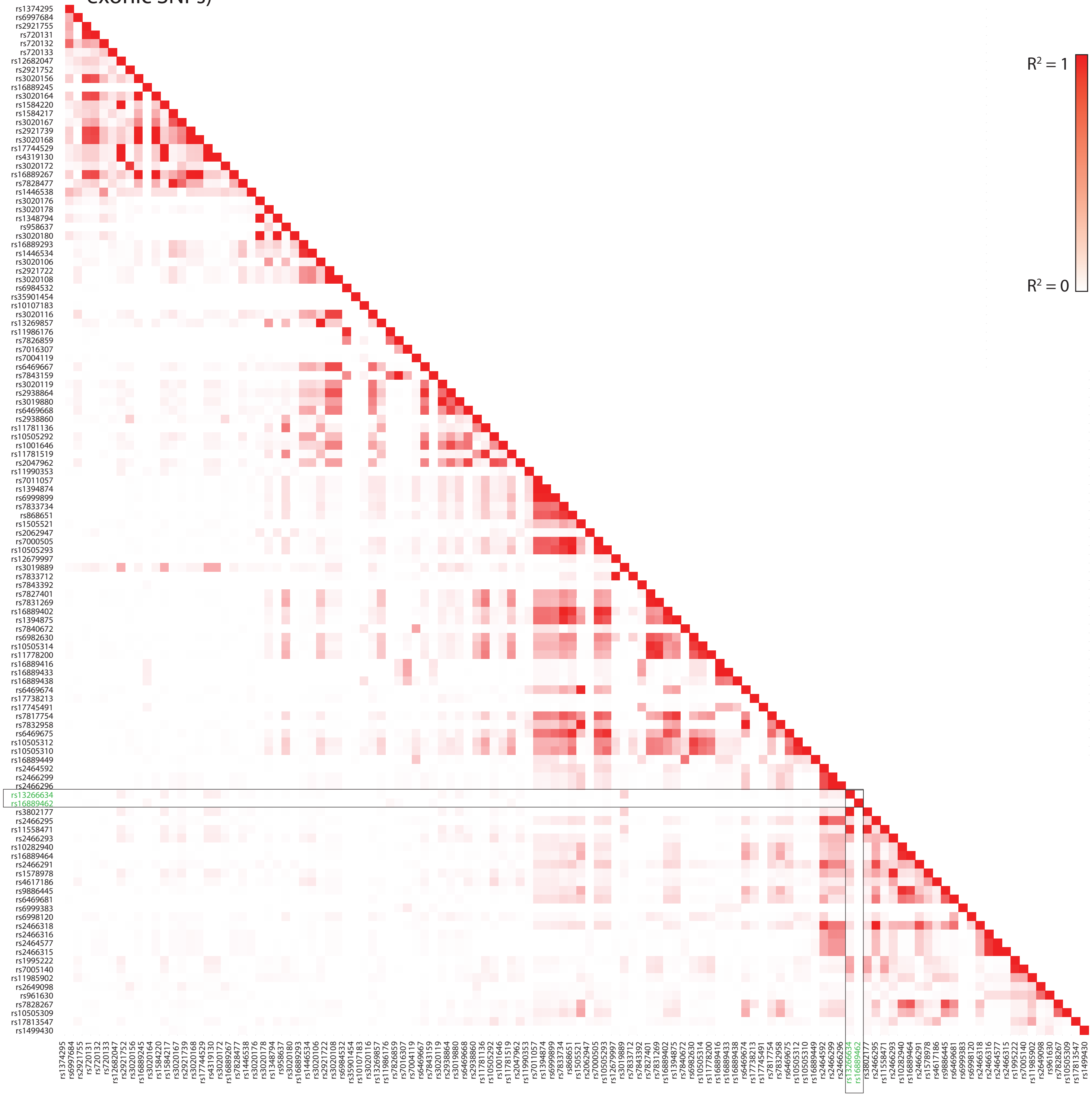

SUPPLEMENTARY TABLE 1

SNP	Basepair	Major allele	Minor allele	MAF	HWE-ChiSq	HWE-P	Ever T2D	
							chi	p(SNP)
rs1374295	116901727	G	T	0.2531	2.27	0.1322	0.115312	0.734176
rs6997684	116901861	C	A	0.4818	0.62	0.4294	0.761496	0.382861
rs2921755	116903318	T	C	0.4906	0.41	0.5228	1.36626	0.242455
rs720131	116903776	G	A	0.4594	0.16	0.6883	1.31373	0.25172
rs720132	116903996	C	T	0.2726	0.11	0.7376	0.06157	0.804032
rs720133	116904042	T	C	0.3390	0.08	0.7757	0.890836	0.345251
rs12682047	116905388	T	C	0.1468	0.12	0.7283	0.38205	0.536508
rs2921752	116907274	T	G	0.0706	2.01	0.1563	0.237182	0.626249
rs3020156	116909116	G	A	0.4840	0.00	0.9699	0.404392	0.52483
rs16889245	116911977	A	G	0.0007	0.00	1.0000	0.999922	0.317329
rs3020164	116914536	T	G	0.4725	0.00	0.9801	0.514232	0.473313
rs1584220	116915698	T	G	0.1435	0.38	0.5387	0.41667	0.518603
rs1584217	116915860	G	T	0.2240	1.99	0.1587	0.011594	0.914253
rs3020167	116916586	C	T	0.3106	2.14	0.1438	0.313302	0.575661
rs2921739	116917219	C	T	0.4800	0.00	0.9693	0.763798	0.382143
rs3020168	116918578	C	T	0.4720	0.00	0.9475	0.264814	0.606832
rs17744529	116918615	G	A	0.1436	0.35	0.5533	0.377944	0.538706
rs4319130	116920945	A	C	0.1436	0.36	0.5483	0.41667	0.518603
rs3020172	116921726	A	G	0.0825	3.53	0.0604	1.356566	0.244134
rs16889267	116925656	C	T	0.4725	0.00	0.9801	0.514232	0.473313
rs7828477	116932347	A	G	0.2278	1.43	0.2312	0.008334	0.927262
rs1446538	116932401	A	G	0.3553	0.10	0.7548	0.447052	0.503738
rs3020176	116935146	A	C	0.1934	0.06	0.8027	0.663408	0.415359
rs3020178	116935759	G	A	0.1988	1.25	0.2630	1.776648	0.182561
rs1348794	116936602	A	G	0.1916	0.07	0.7932	0.629134	0.427673
rs958637	116936747	A	G	0.0498	0.27	0.6001	3.555474	0.059349
rs3020180	116937517	A	G	0.1895	0.26	0.6099	0.856088	0.354836
rs16889293	116939903	C	T	0.2960	0.78	0.3760	0.00034	0.985289
rs1446534	116944800	G	A	0.2750	0.23	0.6340	0.01488	0.902912
rs3020106	116946316	G	A	0.1429	0.06	0.8013	4.294566	0.038234
rs2921722	116947908	G	T	0.4505	0.87	0.3507	1.064782	0.302127
rs3020108	116948147	G	A	0.4504	0.77	0.3812	1.064782	0.302127
rs6984532	116948907	C	T	0.0055	0.05	0.8178	6.554688	0.010461
rs35901454	116959825	G	A	0.0007	0.00	1.0000	0.239834	0.624326
rs10107183	116961569	G	T	0.0047	0.01	0.9425	0.100024	0.751801
rs3020116	116962914	T	G	0.4912	0.46	0.4978	0.248778	0.617937
rs13269857	116965328	G	A	0.1464	0.00	0.9931	4.412568	0.035675
rs11986176	116967932	T	C	0.0065	0.10	0.7574	6.138516	0.013227
rs7826859	116979371	A	G	0.0120	0.12	0.7294	2.985152	0.084031
rs7016307	116988330	A	G	0.0036	0.50	0.4782	2.280554	0.131005
rs7004119	116989423	G	T	0.0211	0.20	0.6511	0.83145	0.361854
rs6469667	116990568	A	G	0.4189	0.03	0.8690	3.572436	0.058746
rs7843159	116992908	G	A	0.0123	0.06	0.8087	2.985152	0.084031

rs3020119	116994332	C	T	0.4149	0.17	0.6797	1.049402	0.305645
rs2938864	117000026	A	G	0.4490	1.08	0.2996	3.117238	0.077468
rs3019880	117006635	C	T	0.4095	0.20	0.6544	0.806318	0.369211
rs6469668	117010246	A	G	0.3810	0.81	0.3695	1.59258	0.206958
rs2938860	117012309	A	G	0.1339	0.58	0.4477	0.32859	0.566491
rs11781136	117016058	A	G	0.0651	0.32	0.5710	1.97553	0.159862
rs10505292	117021088	A	G	0.2959	2.75	0.0971	1.504668	0.219954
rs1001646	117021881	C	T	0.4841	1.57	0.2104	4.553068	0.03286
rs11781519	117025077	T	C	0.0777	0.50	0.4800	1.85079	0.173691
rs2047962	117027832	C	T	0.3771	1.97	0.1606	4.046906	0.044252
rs11990353	117032043	C	A	0.0308	1.01	0.3141	2.749648	0.097276
rs7011057	117040166	G	A	0.1940	0.33	0.5653	0.64431	0.422154
rs1394874	117041250	A	G	0.1942	0.38	0.5366	0.49476	0.481812
rs6999899	117045338	A	C	0.2012	0.17	0.6814	0.534376	0.464773
rs7833734	117058576	G	T	0.1720	3.73	0.0535	0.080776	0.776249
rs868651	117062487	A	G	0.2095	2.92	0.0872	0.167506	0.682338
rs1505521	117063876	C	T	0.0698	0.11	0.7369	1.72059	0.189617
rs2062947	117066457	C	T	0.1049	1.01	0.3140	1.287642	0.256483
rs7000505	117066559	T	G	0.1916	6.40	0.0114	0.229728	0.631726
rs10505293	117072712	G	A	0.2080	3.15	0.0761	0.449146	0.502741
rs12679997	117075816	G	A	0.0207	0.45	0.5044	0.02669	0.870227
rs3019889	117081316	T	C	0.0742	3.63	0.0568	0.207184	0.648984
rs7833712	117086368	C	A	0.0199	0.39	0.5318	0.091036	0.762864
rs7843392	117088354	C	T	0.0076	0.13	0.7230	0	1
rs7827401	117088480	T	C	0.0860	2.58	0.1086	4.392018	0.036108
rs7831269	117090660	C	A	0.0778	1.64	0.2004	3.1225	0.077218
rs16889402	117101065	G	A	0.1794	4.23	0.0396	2.285242	0.13061
rs1394875	117102069	A	G	0.1587	4.93	0.0264	0.329814	0.565769
rs7840672	117105515	G	A	0.0151	0.07	0.7965	0.404302	0.524876
rs6982630	117106584	A	G	0.1092	0.19	0.6671	2.306414	0.128841
rs10505314	117106933	T	C	0.0889	3.02	0.0824	2.230188	0.135337
rs11778200	117108093	A	G	0.0825	1.58	0.2092	1.019508	0.312636
rs16889416	117114869	T	C	0.0105	0.16	0.6905	0.19396	0.659641
rs16889433	117123300	A	C	0.0108	0.16	0.6917	0.229734	0.631721
rs16889438	117131057	C	T	0.0117	0.12	0.7254	0.08945	0.764877
rs6469674	117131504	A	G	0.0742	0.04	0.8470	6.806068	0.009085
rs17738213	117133715	A	G	0.0276	0.65	0.4199	0.038212	0.845018
rs17745491	117134070	G	T	0.0036	0.03	0.8726	0.054922	0.81471
rs7817754	117142742	A	G	0.1530	3.80	0.0511	4.35309	0.036942
rs7832958	117146091	A	G	0.0728	0.04	0.8436	6.907926	0.008581
rs6469675	117152910	A	G	0.2246	2.48	0.1150	2.663478	0.102676
rs10505312	117158022	A	G	0.1226	0.64	0.4225	0.541064	0.461992
rs10505310	117162396	C	T	0.1448	0.03	0.8655	0.305202	0.580639
rs16889449	117162479	C	A	0.0167	0.14	0.7095	0.479714	0.488552
rs2464592	117171312	T	C	0.2617	0.58	0.4446	0.006012	0.938196
rs2466299	117171348	C	T	0.2009	0.10	0.7509	0	1
rs2466296	117171888	C	T	0.2008	0.14	0.7056	0	1

rs13266634	117172544	C	T	0.2270	0.01	0.9324	1.46155	0.226684
rs16889462	117172545	G	A	0.0044	0.01	0.9084	0.332392	0.564254
rs3802177	117172786	C	T	0.2278	0.07	0.7884	0.484076	0.486582
rs2466295	117172802	A	G	0.3038	0.06	0.8131	0.328908	0.566303
rs11558471	117173494	A	G	0.2540	0.00	0.9948	0.463516	0.495985
rs2466293	117173699	T	C	0.4989	5.12	0.0236	0.00028	0.986649
rs10282940	117175587	G	A	0.1168	0.69	0.4077	0.677082	0.410593
rs16889464	117177164	T	C	0.1070	0.02	0.8971	0.965496	0.325806
rs2466291	117178154	C	T	0.3531	0.05	0.8149	1.93311	0.164419
rs1578978	117184564	T	C	0.4700	2.93	0.0872	0.025366	0.873459
rs4617186	117188190	A	G	0.2679	0.61	0.4363	0.000358	0.984904
rs9886445	117188334	G	A	0.1238	0.70	0.4012	0.99381	0.318813
rs6469681	117191195	A	G	0.1842	0.10	0.7462	1.628242	0.201946
rs6999383	117197827	A	G	0.0018	0.03	0.8594	2.294108	0.129866
rs6998120	117203062	A	G	0.0586	0.69	0.4071	0.113756	0.735908
rs2466318	117205068	G	A	0.2990	0.02	0.8997	0.594184	0.440806
rs2466316	117205358	T	C	0.1090	6.20	0.0128	0.109264	0.740984
rs2464577	117206282	C	T	0.1034	0.58	0.4445	0.48847	0.484611
rs2466315	117207144	T	C	0.1034	0.58	0.4445	0.48847	0.484611
rs1995222	117217523	G	A	0.3397	0.97	0.3255	0.000356	0.984946
rs7005140	117221908	A	G	0.3449	0.44	0.5088	0.402472	0.525815
rs11985902	117222649	G	A	0.3737	0.03	0.8657	1.42585	0.232443
rs2649098	117223033	G	A	0.1734	2.26	0.1328	0.757648	0.384066
rs961630	117223771	G	A	0.2679	0.51	0.4739	0.700146	0.402735
rs7828267	117223802	T	C	0.1211	0.19	0.6655	2.082562	0.14899
rs10505309	117224767	T	C	0.0941	0.00	0.9648	2.46172	0.116651
rs17813547	117225125	T	C	0.2272	0.28	0.5960	0.026366	0.87101
rs1499430	117225878	C	A	0.1482	1.85	0.1739	1.881334	0.170183

Association with T2D-related traits u

	Incident T2D			Fasting glucose			Fasting insulin		
**bSNP**	**chi**	**p(SNP)**	**bSNP**	**chi**	**p(SNP)**	**bSNP**	**chi**	**p(SNP)**	**bSNP**
0.023246	3.097598	0.078408	0.162622	0.642838	0.422685	0.031531	0.038138	0.845166	0.009722
0.053584	0.053094	0.817764	-0.01938	0.055432	0.813867	-0.00814	0.002854	0.957395	0.002314
0.070863	7.855828	0.005066	0.222101	0.87135	0.350582	0.032133	0.20746	0.648766	-0.01985
-0.06993	6.662194	0.009848	-0.20552	0.907328	0.340824	-0.03331	0.03028	0.861856	0.007696
-0.01668	1.536598	0.215125	0.111521	0.08419	0.771698	0.01126	0.223448	0.636425	0.023366
0.061873	0.617822	0.431858	0.066743	1.254812	0.262635	0.041115	2.061792	0.151032	-0.0664
-0.05166	1.249138	0.263717	-0.12561	0.165024	0.684573	-0.01926	0.027404	0.868518	0.009909
-0.05885	0.813672	0.367037	-0.13979	0.050422	0.822331	0.015495	1.824838	0.176738	-0.11683
-0.03814	2.72701	0.098664	-0.13082	0.29448	0.587364	-0.01869	0.619578	0.431204	0.034215
-0.9911	0.117724	0.731516	1.250011	0.001644	0.967658	0.000907	0.316454	0.573747	0.533889
-0.04302	2.986888	0.083941	-0.137	0.235264	0.627648	-0.01682	0.231452	0.630449	0.02105
-0.05446	1.217474	0.269857	-0.12465	0.244518	0.620962	-0.02366	0.012388	0.911377	-0.00672
0.007718	0.240338	0.623962	-0.04591	0.211084	0.645919	-0.01863	0.367416	0.544416	0.030917
-0.03664	1.266614	0.260403	-0.09565	0.356366	0.550531	-0.0222	0.009006	0.924394	0.004437
-0.05243	2.972754	0.084678	-0.13634	0.065682	0.797731	-0.00887	0.271174	0.602545	0.022744
-0.03088	2.466732	0.116279	-0.12431	0.222922	0.636822	-0.01636	0.276586	0.598948	0.022998
-0.05162	1.402048	0.236381	-0.13258	0.18627	0.666039	-0.02056	0.000622	0.980103	-0.0015
-0.05446	1.217474	0.269857	-0.12465	0.244518	0.620962	-0.02366	0.012388	0.911377	-0.00672
-0.12846	1.388312	0.23869	-0.16655	0.011332	0.915224	-0.00682	0.405736	0.524142	-0.05128
-0.04304	2.986888	0.083941	-0.137	0.235264	0.627648	-0.01682	0.231452	0.630449	0.02105
0.006415	0.182178	0.669508	-0.03919	0.133278	0.715057	-0.01467	0.315788	0.57415	0.028394
-0.04125	0.128052	0.72046	0.029057	0.01621	0.898688	-0.00452	0.004608	0.945879	-0.00304
-0.06268	0.052422	0.818901	-0.02325	1.185166	0.276307	0.046994	0.047056	0.828268	0.011877
0.099992	0.09292	0.760497	0.02952	4.09776	0.04294	-0.08662	0.363482	0.546579	-0.03257
-0.06103	0.108086	0.742334	-0.03337	0.89074	0.345277	0.040866	0.135588	0.712707	0.020218
0.268325	0.692684	0.405253	0.144013	4.0396	0.044444	-0.1557	0.132554	0.715799	-0.03567
-0.07192	0.208248	0.648144	-0.04643	1.099658	0.294341	0.045684	0.188982	0.663765	0.024029
0.001476	0.0854	0.770109	-0.02458	1.881796	0.17013	-0.05119	0.141568	0.706727	-0.01766
-0.00793	0.317968	0.572831	-0.04776	0.395756	0.529289	-0.0239	0.063282	0.801382	-0.012
0.182984	2.679232	0.101665	0.189266	4.22631	0.039802	-0.10228	4.888618	0.027034	-0.13811
0.061728	0.11932	0.729773	0.026784	6.60973	0.010142	-0.08806	4.136038	0.041979	-0.08763
0.061728	0.11932	0.729773	0.026784	6.60973	0.010142	-0.08806	4.136038	0.041979	-0.08763
-0.8742	0.62151	0.430487	-0.39212	1.24441	0.264623	0.276971	0.103192	0.748032	-0.09967
-0.44936	0.374916	0.540337	3.289441	0.000416	0.983727	-0.00031	0.403304	0.525388	0.593905
-0.11451	0.028214	0.866607	-0.07765	1.030228	0.310105	0.213705	1.232446	0.266932	-0.29216
-0.02945	0.059062	0.807985	-0.01851	2.72449	0.09882	0.055871	2.313648	0.128243	0.064853
0.184195	3.087868	0.078878	0.201818	3.351376	0.067149	-0.08991	4.976302	0.025697	-0.1375
-0.76364	0.082436	0.774023	-0.13064	0.51805	0.471675	0.162467	0.364048	0.546267	-0.17024
-0.44381	0.06265	0.802355	0.089617	0.592644	0.441399	0.124669	0.287334	0.591934	-0.10865
3.711865	1.606894	0.204929	3.720952	0.003378	0.953653	0.001536	0.02738	0.868575	0.05798
0.20081	0.000132	0.990833	0.003368	0.713434	0.398306	-0.09779	3.102686	0.078163	-0.2544
0.115135	1.652262	0.198651	0.100628	7.258496	0.007057	-0.09339	3.867472	0.049231	-0.08571
-0.44383	0.06265	0.802355	0.089617	0.592644	0.441399	0.124694	0.287334	0.591934	-0.10865

-0.06189	0.326446	0.567759	-0.04517	3.33694	0.06774	0.062938	3.202338	0.073533	0.077685
0.106724	1.64902	0.199092	0.099768	7.182448	0.007362	-0.09184	5.995886	0.014339	-0.10549
-0.05493	0.10252	0.748826	-0.02581	2.856774	0.09099	0.058855	1.616228	0.203619	0.055728
0.078032	1.121644	0.289565	0.082629	4.615468	0.031685	-0.0743	4.928528	0.026417	-0.09637
0.051722	0.104156	0.746898	-0.03692	0.441956	0.50618	-0.03408	0.003454	0.953135	0.00378
0.177687	0.061274	0.804493	0.040488	1.780814	0.182049	-0.09387	0.508982	0.47558	-0.06294
0.081805	1.518584	0.217834	0.103422	3.786564	0.051666	-0.07316	0.391364	0.531583	-0.02937
0.128505	3.102496	0.078172	0.137223	6.348338	0.011749	-0.08561	3.783032	0.051775	-0.08308
0.158244	0.28594	0.592834	0.078814	1.632854	0.201309	-0.08249	0.390968	0.531791	-0.05082
0.125105	4.467682	0.034542	0.167439	3.772464	0.052103	-0.06713	3.87326	0.049061	-0.0853
-0.28363	3.076322	0.07944	-0.37618	0.070896	0.790037	0.02611	0.923272	0.336617	0.119099
0.06157	0.004104	0.94892	0.006331	3.010644	0.082719	-0.07446	0.96572	0.32575	-0.05292
0.053887	0.004104	0.94892	0.006331	3.010646	0.082719	-0.07446	0.965722	0.325749	-0.05292
0.055361	0.022248	0.881429	0.014664	2.352644	0.125071	-0.06448	0.544788	0.460455	-0.03901
0.022965	0.00242	0.960765	-0.00523	0.01643	0.898007	-0.00597	0.049552	0.823845	-0.01302
0.030422	0.053446	0.817172	0.022609	0.141708	0.706589	-0.01588	0.375556	0.539991	-0.03251
-0.14878	2.058036	0.151405	-0.21578	0.307736	0.579073	0.036294	0.152808	0.695866	0.032032
0.11706	1.888964	0.169319	0.175361	0.962558	0.326543	0.055432	2.069062	0.150314	-0.10269
0.036942	0.00161	0.967994	-0.0041	0.020156	0.887102	-0.00621	0.359988	0.548513	-0.03302
-0.15919	0.472356	0.491905	0.203159	0.00018	0.989296	0.001834	0.32997	0.565677	-0.09889
-0.03673	2.708234	0.099832	0.5884	0.006114	0.937675	-0.01006	3.005604	0.082977	-0.27773
-0.04937	0.131548	0.716832	-0.05438	1.449462	0.228614	0.07619	0.583574	0.444915	0.061237
-0.0687	2.214576	0.136713	0.541	0.063206	0.801499	-0.033	2.469678	0.116062	-0.25708
-3.7E-05	3.61533	0.057249	3.994874	2.626102	0.10512	0.341013	0.447738	0.503411	0.17606
0.288768	1.15936	0.281598	0.194101	0.419954	0.51696	-0.04869	0.374098	0.540779	0.057702
0.252397	0.248774	0.61794	0.091412	0.671144	0.412653	-0.06405	0.020372	0.886503	0.014029
-0.32865	0.229932	0.631574	-0.14237	0.05547	0.813805	0.028084	0.18865	0.664043	0.06562
-0.09516	3.36444	0.066618	-0.45051	0.000504	0.982089	-0.00203	5.010104	0.0252	0.256594
0.167979	0.379102	0.538084	0.262515	4.284666	0.038458	-0.30709	2.81197	0.093563	-0.31025
0.197828	1.444686	0.229382	0.212767	0.762464	0.382559	-0.06427	0.001824	0.965934	0.003963
0.198309	0.113224	0.736503	0.058934	0.572844	0.449131	-0.05596	0.965206	0.325879	0.091634
0.131786	0.15425	0.694507	-0.06589	2.160714	0.141579	-0.10724	0.100306	0.751463	0.029156
0.135364	1.157376	0.28201	0.505039	1.678518	0.195122	0.219808	0.85218	0.355936	0.195906
0.146761	1.333922	0.248109	0.537991	2.506524	0.113376	0.265396	1.025992	0.311102	0.212645
0.079778	0.749768	0.38655	0.408877	0.215986	0.642116	0.071636	0.017644	0.894327	-0.02558
-0.33963	5.296246	0.021371	-0.39592	2.613914	0.10593	0.122017	0.349046	0.554654	0.055871
-0.03706	0.351852	0.553067	0.137191	1.807726	0.178781	-0.14215	3.128474	0.076936	0.235737
-0.12015	1.939556	0.163717	3.747398	1.646222	0.199474	0.373945	0.018654	0.891363	-0.04975
-0.2928	11.02586	0.000898	-0.66644	0.839328	0.359589	0.072698	2.268414	0.132035	0.150896
-0.34171	5.43025	0.019791	-0.3997	2.578878	0.108298	0.120861	0.48496	0.486184	0.065665
-0.24048	1.731828	0.188178	-0.24544	2.14256	0.143263	0.119802	0.627474	0.428283	0.081964
0.089977	0.829286	0.362479	0.144911	0.9418	0.331815	-0.06741	1.428306	0.232041	-0.10393
0.062908	0.769946	0.380234	0.128037	1.181984	0.276952	-0.07008	1.040842	0.307626	-0.08249
0.17908	1.068082	0.301379	0.420554	1.311334	0.252153	-0.16557	0.564612	0.452408	-0.13555
-0.00584	0.092604	0.760892	-0.02979	0.425188	0.51436	0.027224	0.10079	0.750884	-0.01673
-2.85E-07	0.026308	0.87115	-0.01686	0.730712	0.392653	0.037778	0.25889	0.610884	-0.02834
-2.85E-07	0.026308	0.87115	-0.01686	0.730712	0.392653	0.037778	0.25889	0.610884	-0.02834

0.086513	0.47662	0.489957	0.064914	0.35506	0.551263	-0.02374	0.00868	0.925771	0.004674
0.322492	3.115162	0.077567	3.804443	0.05722	0.810945	0.06526	0.162398	0.686958	-0.14159
0.049739	0.171516	0.678768	0.038999	0.07562	0.783323	-0.01095	0.158734	0.690325	0.01996
-0.03964	0.186628	0.665738	-0.03887	0.506806	0.476525	0.027629	0.043904	0.834032	0.010283
0.047016	0.230824	0.630914	0.043698	0.018144	0.892849	0.005175	0.266934	0.605396	0.024972
-0.00077	0.07024	0.790988	0.021336	0.137186	0.711095	-0.01288	0.69448	0.404645	-0.03664
-0.07878	0.859534	0.353869	-0.11206	0.042438	0.836787	-0.01122	0.19396	0.659641	0.0301
-0.09601	0.658828	0.416974	-0.10127	0.089656	0.764615	-0.01675	0.000864	0.97655	-0.00207
-0.09714	1.783752	0.181689	-0.12243	1.47865	0.223986	0.047895	1.253348	0.262914	0.055602
-0.00991	0.000012	0.997236	0.000186	0.088002	0.766733	-0.01035	0.558246	0.454968	-0.03303
-0.00118	0.0061	0.937747	0.007327	0.000056	0.994029	0.000359	0.224436	0.63568	-0.02397
-0.0941	0.673672	0.411774	-0.09851	0.001822	0.965953	0.002292	0.223998	0.63601	0.032157
-0.10706	1.881526	0.170161	-0.14766	0.041822	0.837959	0.009716	0.804832	0.369653	0.053926
4.289455	0.432194	0.510914	3.183938	1.626918	0.20213	0.442978	0.233618	0.628854	0.209789
-0.04522	0.435348	0.509376	-0.11459	0.009606	0.921924	0.007433	0.35498	0.551307	0.056573
-0.05431	0.781044	0.376822	-0.08098	0.41421	0.51984	0.02539	0.462452	0.49648	0.033912
0.032509	0.063324	0.801317	0.032803	0.3111	0.577006	0.030575	0.030178	0.862087	-0.01199
0.070356	0.36493	0.545781	0.082522	0.063852	0.800508	0.014059	0.064708	0.799204	-0.01781
0.070356	0.36493	0.545781	0.082522	0.063852	0.800508	0.014059	0.064708	0.799204	-0.01781
-0.00114	0.066858	0.795968	-0.02131	0.198176	0.656197	0.01588	0.833522	0.361256	0.041106
0.040379	0.011806	0.913476	-0.00892	0.155388	0.693439	0.013925	1.880794	0.170244	0.060996
-0.07471	0.5506	0.458073	-0.06141	0.009822	0.921054	-0.00357	0.54731	0.459419	0.033633
0.071084	0.587214	0.443499	0.080805	0.785292	0.375528	-0.04053	3.77572	0.052001	-0.11193
0.058425	0.71436	0.398	0.076387	0.094424	0.758626	-0.01193	5.83057	0.01575	-0.11874
-0.13443	1.003696	0.316418	-0.12177	0.008838	0.925101	-0.00506	0.011312	0.915298	0.007708
-0.16809	2.836454	0.092147	-0.23797	0.278758	0.597516	0.032364	0.039158	0.843136	-0.01529
-0.01153	0.004666	0.94554	0.00639	0.017526	0.894679	-0.00547	0.31906	0.572173	0.029514
0.116389	0.000152	0.990163	0.001499	0.558696	0.454786	0.03516	1.171274	0.27914	0.064204

using measured genotype approach

2-hour glucose			2-hour insulin			HOMA-IR			
chi	p(SNP)	bSNP	chi	p(SNP)	bSNP	chi	p(SNP)	bSNP	chi
2.066696	0.150547	0.057671	2.300466	0.129335	0.075747	0.506582	0.476622	0.033848	0.002
0.40846	0.522752	-0.02246	2.595034	0.107199	-0.07004	0.00266	0.958867	0.00217	0.150922
0.631124	0.426943	0.027825	0.002728	0.958345	0.002268	0.09742	0.754948	0.013112	0.123126
0.46813	0.493849	-0.02431	0.035484	0.850585	-0.0083	0.106152	0.744568	-0.01388	0.004122
5.306144	0.02125	0.090868	1.542486	0.214248	0.061202	0.112498	0.737318	0.015827	0.022112
0.280886	0.596121	-0.01988	0.508142	0.475944	-0.03297	0.557108	0.455428	-0.03322	0.04821
0.93805	0.33278	-0.04642	0.06045	0.805786	0.014635	0.000058	0.993924	-0.00044	0.092538
1.370628	0.241704	0.082775	0.173266	0.677225	0.036316	1.332586	0.248346	-0.0955	0.022042
0.224482	0.635646	-0.01664	0.364564	0.545982	0.026207	0.099454	0.752486	0.01317	0.006988
0.373232	0.541248	0.465982	0.003792	0.950898	-0.0578	0.993422	0.318907	-0.50281	0.421142
0.768314	0.380739	-0.03098	0.061532	0.804091	0.010832	0.017408	0.895032	0.005545	0.035314
0.659554	0.416717	-0.03929	0.086276	0.768966	0.017645	0.008982	0.924495	-0.00553	0.059194
1.133486	0.287033	-0.04404	0.158166	0.690851	-0.02035	0.054558	0.815314	0.01156	0.053242
0.000042	0.994829	0.000399	0.049972	0.823112	0.01048	0.0213	0.883965	0.006587	0.376316
0.467936	0.493938	-0.02415	0.051266	0.820875	0.00987	0.002608	0.959271	0.00214	0.169738
0.68338	0.408425	-0.0292	0.055456	0.813828	0.010274	0.003014	0.956218	0.002307	0.038836
0.473672	0.491303	-0.03317	0.18519	0.666951	0.025747	0.001568	0.968414	-0.0023	0.095862
0.659554	0.416717	-0.03929	0.086276	0.768966	0.017645	0.008982	0.924495	-0.00553	0.059194
3.889778	0.048581	0.129602	2.185092	0.139353	0.119973	0.004494	0.946552	0.005131	0.171876
0.768314	0.380739	-0.03098	0.061532	0.804091	0.010832	0.017408	0.895032	0.005545	0.035314
1.266138	0.260492	-0.04611	0.189188	0.663593	-0.02206	0.000846	0.976796	0.001421	0.022502
8.535374	0.003483	0.105421	2.227088	0.135609	0.067144	0.297262	0.585604	0.023387	0.561518
0.015114	0.902155	-0.00543	0.021816	0.882578	-0.00814	0.316558	0.573684	-0.02958	0.400246
3.946028	0.046982	-0.08647	3.571152	0.058791	-0.10211	1.21847	0.269661	-0.05713	0.759386
0.036124	0.84926	-0.00842	0.001084	0.973735	-0.00182	0.205832	0.650054	-0.02395	0.253558
8.976902	0.002734	-0.23516	5.370954	0.020475	-0.22586	2.21452	0.136718	-0.14324	0.078846
0.001454	0.969583	-0.0017	0.01226	0.911834	0.00616	0.091776	0.761931	-0.01611	0.358844
1.460522	0.226847	0.046083	2.219286	0.136296	0.070448	0.248782	0.617934	-0.02265	1.368852
1.74637	0.186334	0.051085	1.57235	0.209866	0.060077	0.049134	0.824577	-0.01022	1.29442
3.681072	0.055033	-0.09711	3.714312	0.053948	-0.12036	6.768908	0.009276	-0.15592	0.830872
1.10059	0.294137	-0.03656	0.355226	0.55117	-0.02573	5.122332	0.02362	-0.09356	6.26129
1.10059	0.294137	-0.03656	0.355226	0.55117	-0.02573	5.122332	0.02362	-0.09356	6.26129
2.970714	0.084784	0.431575	0.000626	0.980039	0.007731	0.025016	0.874327	0.041864	0.865984
1.634076	0.20114	-0.96338	0.015466	0.901028	0.115534	0.531784	0.465858	0.50343	0.228798
0.699624	0.40291	0.177316	0.037474	0.846503	0.050472	0.730326	0.392778	-0.22692	0.546516
0.764496	0.381925	0.030082	0.089962	0.764226	0.012828	2.279494	0.131095	0.061957	4.00768
3.948558	0.046912	-0.09923	4.2064	0.040272	-0.1264	6.804564	0.009092	-0.15449	0.534546
0.334956	0.562755	0.131772	0.639868	0.423759	-0.22545	0.03105	0.860129	0.042447	0.34241
0.695546	0.404284	-0.13632	2.385782	0.122444	-0.30807	0.079428	0.778074	-0.05287	0.206038
3.108246	0.077897	-0.49381	1.507556	0.219512	-0.41839	0.085744	0.769659	-0.10536	0.01371
4.68795	0.030375	-0.25099	7.33305	0.00677	-0.38737	5.06581	0.024402	-0.31289	2.32777
0.955806	0.328246	-0.03452	1.106706	0.292799	-0.04599	5.287264	0.021482	-0.09592	5.48095
0.695546	0.404284	-0.13632	2.385782	0.122444	-0.30805	0.079428	0.778074	-0.05287	0.206038

0.598874	0.439008	0.027148	0.672606	0.412145	0.035536	6.300528	0.01207	0.104565	5.746654
2.744982	0.09756	-0.05772	3.87673	0.04896	-0.08491	9.656734	0.001887	-0.12815	6.487098
0.222412	0.637208	0.016725	0.09895	0.753093	0.013781	4.688066	0.030373	0.091003	4.280536
0.094986	0.757932	-0.01084	1.20327	0.272669	-0.04781	6.417694	0.011299	-0.1066	8.313448
1.11681	0.290606	-0.05533	0.172702	0.677721	-0.02666	0.914254	0.338988	-0.05906	0.00268
7.605184	0.00582	-0.19562	4.185222	0.040778	-0.17897	3.558896	0.059227	-0.16097	0.075574
1.974256	0.159997	0.053295	0.28209	0.595335	0.024921	0.563254	0.452952	-0.03415	3.516204
1.586312	0.207854	-0.04348	3.894288	0.048451	-0.08407	8.927476	0.002809	-0.12238	3.396316
6.338866	0.011812	-0.16427	3.86821	0.049209	-0.15902	3.88216	0.048802	-0.15543	0.020944
0.018232	0.892591	0.004734	1.077558	0.299245	-0.04508	4.115214	0.042499	-0.08554	4.49998
0.039916	0.841645	-0.0197	0.053046	0.817845	0.028198	0.001622	0.967875	0.004859	0.258882
9.764144	0.00178	-0.1352	4.698756	0.030184	-0.11633	3.029296	0.081773	-0.09087	1.76711
9.764144	0.00178	-0.13519	4.698756	0.030184	-0.11633	2.778838	0.095517	-0.08701	1.76711
8.143652	0.004321	-0.12105	3.482192	0.062032	-0.09816	2.660854	0.102846	-0.08373	1.289348
1.992388	0.158091	-0.06662	1.293474	0.255409	-0.0666	0.035946	0.849627	-0.01057	0.401558
4.385196	0.036252	-0.08935	3.505	0.061184	-0.09894	0.892412	0.344824	-0.04816	0.091806
0.13477	0.713537	0.024272	0.736438	0.390804	0.070707	0.892664	0.344756	0.074855	0.017074
0.399724	0.527232	-0.03624	1.398996	0.236892	-0.08391	2.988648	0.08385	-0.11861	0.760534
5.145892	0.023301	-0.10059	4.529016	0.033325	-0.11695	0.621882	0.430349	-0.0417	0.187824
0.000166	0.98972	-0.00176	0.021648	0.883027	-0.02508	0.63241	0.426473	-0.13254	0.09753
0.44777	0.503396	-0.08692	1.959052	0.161615	-0.22673	0.00125	0.971796	-0.00527	0.08011
0.272852	0.601425	-0.03336	0.233228	0.629141	-0.03836	0.243716	0.621535	0.037771	1.696728
0.765894	0.38149	-0.1162	2.709184	0.099772	-0.27285	0.027112	0.869214	0.024923	0.17501
1.13084	0.287596	0.224909	0.4829	0.487112	0.181298	0.519026	0.471257	0.15345	1.567602
0.01205	0.91259	-0.00831	0.000042	0.994829	0.000603	1.122076	0.289472	-0.09777	0.837114
0.36922	0.54343	-0.04789	0.36034	0.548318	-0.05857	1.630716	0.201604	-0.12264	0.261302
4.31882	0.037693	0.247938	3.023982	0.082042	0.259192	1.818468	0.177496	0.197109	0.009728
5.60261	0.017934	0.216653	9.928672	0.001627	0.360544	1.617252	0.203475	0.140431	0.301848
3.572642	0.058739	-0.28055	6.142102	0.0132	-0.45473	2.983998	0.084091	-0.30248	2.704452
0.643886	0.422307	-0.05947	0.70029	0.402686	-0.07728	0.386264	0.53427	-0.05564	0.324468
0.416172	0.518853	-0.04809	0.010364	0.918913	-0.00945	0.2974	0.585517	-0.04953	0.65618
0.56464	0.452397	-0.05532	0.000478	0.982557	0.002002	0.650474	0.419943	-0.07245	0.03386
0.018526	0.891734	0.023194	0.06301	0.8018	0.052582	0.047944	0.82668	0.043677	0.965214
0.046592	0.829103	0.036387	0.002772	0.958011	0.010902	0.09997	0.751866	0.062439	1.437318
0.00008	0.992864	0.001451	0.198646	0.655816	-0.08511	0.113658	0.736017	-0.05985	0.064528
2.567928	0.10905	0.122026	5.705462	0.016912	0.224315	3.520718	0.060606	0.169925	0.079292
0.193104	0.660346	0.046487	0.424824	0.51454	0.085443	1.97502	0.159916	0.180755	0.151322
1.88157	0.170156	0.401554	0.811264	0.367747	0.325267	0.075504	0.783485	-0.08718	1.307244
0.317518	0.573103	0.045123	5.9801	0.014468	0.243656	2.643404	0.103981	0.154704	0.701036
2.328434	0.12703	0.115863	5.677192	0.017187	0.223127	3.924058	0.0476	0.17898	0.081258
0.129462	0.71899	0.029565	1.129956	0.287785	0.109157	0.439942	0.50715	0.066881	3.026808
1.314822	0.251524	-0.08048	3.046946	0.080889	-0.15106	1.994552	0.157866	-0.11877	0.06201
1.599144	0.206025	-0.08212	3.949622	0.046882	-0.15974	2.99295	0.083628	-0.13559	0.055182
1.89059	0.169135	-0.19877	2.861036	0.090749	-0.30215	1.330396	0.248735	-0.1946	0.6383
1.403184	0.236191	0.049928	0.036604	0.848274	0.009989	0.26264	0.608312	0.025909	0.37035
0.002038	0.963992	-0.00202	0.419244	0.517315	-0.03577	0.05677	0.811676	0.012849	0.05634
0.002038	0.963992	-0.00202	0.419244	0.517315	-0.03577	0.05677	0.811676	0.012849	0.05634

2.0459	0.152617	-0.05782	0.2961	0.586338	-0.0273	0.109132	0.741135	-0.01604	0.405368
0.105564	0.745252	-0.08786	0.094346	0.758723	-0.10677	0.433502	0.510276	-0.20405	0.013588
1.718228	0.189921	-0.05289	0.199534	0.655097	-0.02238	0.015444	0.901098	0.00603	0.056998
0.879414	0.348362	0.036834	0.008598	0.926122	0.004512	0.79023	0.374031	0.041893	0.62213
3.68732	0.054828	-0.07477	0.699824	0.402843	-0.04047	0.108442	0.741925	0.015444	0.014638
0.599104	0.43892	0.027467	0.03162	0.858864	0.007842	1.446746	0.229051	-0.05068	0.33919
0.333068	0.563858	0.031848	0.357036	0.550157	0.040738	1.020932	0.312298	0.067042	0.561166
0.384466	0.535223	0.03517	0.20937	0.647262	0.032076	1.033508	0.309336	0.069034	0.544484
2.66141	0.10281	0.064826	0.388586	0.533044	0.030743	1.749532	0.185936	0.06296	2.283488
0.000212	0.988383	-0.00056	0.0195	0.888942	0.006176	0.938376	0.332696	-0.04079	0.255884
0.152696	0.695972	0.01573	0.628872	0.427769	0.039887	0.044118	0.833634	0.010116	0.12705
0.047852	0.826844	0.011923	0.021874	0.882423	0.009998	1.412804	0.234592	0.078272	0.447616
2.420776	0.119736	0.07523	1.219254	0.269507	0.066188	2.17007	0.14072	0.084867	0.070028
1.081464	0.29837	-0.36159	1.431628	0.231499	-0.51196	0.051706	0.820121	0.102026	1.262504
4.383454	0.036289	0.160486	2.806514	0.093882	0.15794	0.371618	0.542124	0.055275	1.512956
1.31785	0.250978	0.045936	0.000346	0.985159	0.000921	1.595296	0.206571	0.060712	0.619154
0.04729	0.827848	-0.01203	0.980756	0.322012	-0.06799	0.015314	0.901513	0.008246	0.063266
0.078438	0.779425	0.015722	0.637984	0.424442	-0.05557	0.002572	0.959553	-0.00343	0.201152
0.07844	0.779423	0.01572	0.637982	0.424443	-0.05556	0.002572	0.959553	-0.00343	0.201152
0.722654	0.395275	-0.03064	0.007426	0.931328	-0.00386	0.401448	0.526342	0.027566	1.2717
0.272	0.601993	-0.01861	0.07965	0.777772	0.012514	0.62045	0.43088	0.033959	0.89137
3.36987	0.066399	0.066879	1.530532	0.216033	0.055944	2.05885	0.151324	0.06222	0.009356
2.189906	0.138918	-0.06872	0.960304	0.32711	-0.0564	4.147426	0.041698	-0.11256	3.113698
1.391326	0.238181	-0.04646	1.958728	0.161649	-0.06871	5.761672	0.01638	-0.11301	1.30004
0.04948	0.823971	0.012065	0.00574	0.939608	0.005107	2.03405	0.15381	0.092655	0.203352
0.01282	0.909852	-0.00698	0.784314	0.375825	0.068047	0.474606	0.490876	0.051103	0.161502
5.373602	0.020444	0.096971	0.909622	0.340215	0.049278	0.089576	0.764717	0.014748	0.35338
0.055138	0.814353	-0.01116	0.488066	0.484791	0.041232	0.02315	0.879068	0.008745	0.075178

HOMA-β	
p(SNP)	bSNP
0.964329	-0.00187
0.697656	-0.01422
0.725669	-0.01289
0.948809	0.002389
0.88179	0.006176
0.826208	-0.00855
0.760975	-0.01535
0.881975	0.010833
0.933379	-0.00307
0.516368	-0.5241
0.850939	-0.00693
0.807774	-0.01238
0.817515	0.00992
0.539582	0.024177
0.680345	-0.01518
0.843774	-0.00727
0.756853	-0.0157
0.807774	-0.01238
0.67845	0.02825
0.850939	-0.00693
0.880759	0.006392
0.453649	0.028344
0.526962	-0.02932
0.383521	0.039826
0.614581	-0.0234
0.778867	0.023182
0.549149	-0.02801
0.242009	0.046437
0.255235	0.045856
0.362021	0.047999
0.01234	0.090873
0.01234	0.090876
0.352069	-0.24882
0.632417	0.383864
0.459745	-0.16514
0.045293	-0.07202
0.464702	0.038032
0.558441	-0.14222
0.649891	-0.0774
0.906789	-0.03386
0.127084	0.185959
0.019225	0.085972
0.649891	-0.0774

0.01652	-0.08781
0.010866	0.092421
0.038551	-0.07661
0.003935	0.105426
0.958713	-0.00278
0.783388	-0.02032
0.060771	0.07454
0.065342	0.066323
0.884932	-0.00985
0.033895	0.077627
0.610889	-0.05324
0.18374	0.060332
0.18374	0.060331
0.256168	0.050617
0.526286	-0.03138
0.761894	-0.01358
0.896038	0.009106
0.383161	-0.05273
0.664734	-0.02018
0.754814	0.0452
0.777148	0.038602
0.192716	-0.08825
0.675697	0.058296
0.210556	-0.28196
0.360223	-0.07273
0.609227	-0.0423
0.921432	-0.01262
0.582726	-0.05315
0.100069	0.259967
0.568935	-0.0446
0.417912	-0.06379
0.854005	-0.01429
0.325877	-0.17667
0.230574	-0.21262
0.799477	-0.04176
0.778259	-0.02255
0.697275	0.043581
0.252894	-0.3567
0.402436	-0.07082
0.7756	-0.02278
0.081899	-0.1516
0.803347	-0.0183
0.81428	0.01603
0.424327	0.122755
0.542813	-0.02701
0.812377	0.011146
0.812377	0.011146

0.52433	0.02701
0.907203	-0.03379
0.811305	0.010137
0.430257	-0.03263
0.903701	-0.00495
0.560297	0.021668
0.453791	0.043448
0.46058	0.043937
0.130757	-0.06341
0.612962	0.018885
0.721511	-0.01523
0.503469	0.038763
0.791296	-0.01348
0.261178	-0.41501
0.218689	-0.09807
0.431362	-0.03307
0.801406	-0.01475
0.653793	-0.02662
0.653793	-0.02662
0.259448	-0.04306
0.345106	-0.03565
0.922944	0.003706
0.077636	0.085422
0.254206	0.047118
0.652029	0.025968
0.687777	0.026355
0.552206	0.025862
0.783942	-0.01376

SUPPLEMENTARY TABLE 2

No	SNP	Ever T2D				Incident T2D			
		age_b	age_p	sex_b	sex_p	age_b	age_p	sex_b	sex_p
1	rs1001646	-0.1196	0.2453	-0.0929	0.3929	-0.1712	0.2530	0.0777	0.5603
2	rs1010718	4.7076	0.0645	4.3590	0.5855	-0.9347	1.0000	4.1068	0.1199
3	rs1028294	-0.1668	0.3076	0.2525	0.1105	-0.2591	0.2760	0.4134	0.0383
4	rs1050529	-0.1086	0.3391	-0.0377	0.7306	-0.2251	0.1617	-0.0746	0.6106
5	rs1050529	-0.0444	0.7294	0.0414	0.7518	0.0465	0.8163	0.3306	0.0595
6	rs1050530	-0.2415	0.1711	-0.0488	0.6957	-0.7281	0.0103	0.0096	0.9293
7	rs1050531	0.0770	0.6052	0.0912	0.5388	0.2544	0.2590	0.4764	0.0199
8	rs1050531	0.0374	0.8121	0.0439	0.7739	0.2148	0.3709	0.3648	0.0960
9	rs1050531	-0.1209	0.5229	0.1792	0.3597	0.0886	0.7558	0.5027	0.0477
10	rs1155847	0.0219	0.8516	-0.1052	0.3912	0.2145	0.2278	-0.4132	0.0127
11	rs1177820	-0.1799	0.3531	0.2213	0.2779	0.1352	0.6330	0.5458	0.0297
12	rs1178113	-0.1541	0.4757	0.1187	0.6245	0.1563	0.6144	0.4560	0.0982
13	rs1178151	-0.0326	0.8708	0.1378	0.4990	0.1684	0.5597	0.5983	0.0219
14	rs1198590	-0.2545	0.0180	0.1110	0.3525	-0.5061	0.0015	0.0685	0.7126
15	rs1198617	0.1527	0.8331	0.5926	0.3778	-0.3390	1.0000	4.4948	0.0510
16	rs1199035	-0.6386	0.0388	-0.1995	0.5063	-0.7403	0.1444	-0.1265	0.8459
17	rs1267999	0.3819	0.3403	-0.6330	0.1921	4.7360	0.0106	-4.9665	0.1062
18	rs1268204	-0.0237	0.8683	-0.1177	0.4117	-0.0924	0.6712	-0.1678	0.4050
19	rs1326663	0.1172	0.3492	-0.0363	0.7957	0.3481	0.0552	-0.2696	0.1225
20	rs1326985	-0.0191	0.8988	-0.1551	0.3258	0.2331	0.2836	0.0327	0.8683
21	rs1348794	0.0314	0.8162	-0.2577	0.0535	0.2380	0.2320	-0.0545	0.7600
22	rs1374295	0.0428	0.7207	0.0777	0.5135	0.2568	0.1558	0.0188	0.9794
23	rs1394874	-0.1631	0.2092	0.0150	0.8841	-0.0864	0.6662	0.2151	0.2089
24	rs1394875	-0.2091	0.1467	0.0685	0.6806	-0.0871	0.6973	0.2806	0.1453
25	rs1446534	-0.0337	0.7670	0.0095	0.9450	-0.0270	0.8694	-0.0328	0.8282
26	rs1446538	0.0619	0.5743	0.2440	0.0230	0.0541	0.7475	0.2273	0.1261
27	rs1499430	0.1240	0.4132	-0.1941	0.2111	0.5130	0.0154	-0.0013	0.9011
28	rs1505521	-0.3458	0.0855	-0.0437	0.7557	-0.5846	0.0786	-0.0884	0.9197
29	rs1578978	-0.1032	0.3313	0.0798	0.4786	-0.0384	0.8069	0.1001	0.4780
30	rs1584217	-0.0110	0.9304	-0.1803	0.1528	-0.2290	0.2328	-0.0723	0.6485
31	rs1584220	-0.0518	0.7202	-0.1307	0.3576	-0.0874	0.6889	-0.2102	0.3033
32	rs1688924	5.9225	0.1630	-1.7808	1.0000	0.8845	0.9594	0.9521	0.8452
33	rs1688926	0.0238	0.8197	-0.1448	0.1700	-0.2553	0.1047	-0.1288	0.3570
34	rs1688929	0.0132	0.9083	-0.0285	0.8066	-0.0636	0.7036	-0.0505	0.7500
35	rs1688940	-0.1297	0.3396	0.0047	0.9872	-0.0050	0.9844	0.1991	0.2833
36	rs1688941	0.6074	0.3148	0.0901	0.9235	-1.1188	0.5444	3.4637	0.2848
37	rs1688943	0.6075	0.3137	0.0804	0.9112	-1.0820	0.5645	3.3236	0.2948
38	rs1688943	0.3989	0.4707	-0.0413	0.7009	-0.9670	0.9956	3.4762	0.2237
39	rs1688944	0.6619	0.1636	-0.5763	0.3283	0.3142	0.7011	-0.3034	0.7976
40	rs1688946	-4.2561	0.2913	-3.4605	0.4135	1.0925	0.9975	1.3100	0.9968
41	rs1688946	-0.2156	0.2009	0.0780	0.6410	-0.5132	0.0484	0.1068	0.4967
42	rs1773821	-0.1651	0.6123	0.4878	0.1290	-0.7534	0.1625	0.8446	0.0517
43	rs1774452	-0.0343	0.8131	-0.1472	0.3078	-0.0116	0.9493	-0.2614	0.2082
44	rs1774549	0.3925	0.7149	-1.7838	0.3268	1.4329	0.9647	1.6131	0.9316

45	rs178135[illegible]	-0.1703	0.1393	-0.0282	0.7561	-0.2572	0.1190	-0.1475	0.3278
46	rs1995222	0.1516	0.1757	0.0545	0.6092	0.3569	0.0298	0.0201	0.8747
47	rs2047962	-0.0316	0.7663	-0.0892	0.4070	-0.1321	0.3760	-0.0641	0.6446
48	rs2062947	0.1349	0.4627	-0.5588	0.0038	0.0667	0.7936	-0.3564	0.1413
49	rs2464577	0.1009	0.5724	0.0406	0.8307	0.0085	0.9733	0.1717	0.4927
50	rs2464592	0.0293	0.8049	-0.1066	0.3754	-0.1385	0.4401	0.0859	0.6125
51	rs2466291	-0.0014	0.9893	-0.0162	0.8840	-0.0513	0.7624	0.2054	0.1815
52	rs2466293	-0.0548	0.5996	0.0108	0.9412	0.0031	0.9825	0.0080	0.9531
53	rs2466295	0.0374	0.7447	0.0036	0.9732	-0.2136	0.2090	0.1504	0.3518
54	rs2466296	-0.0417	0.7522	0.0184	0.8945	-0.1631	0.4037	0.1287	0.4695
55	rs2466299	-0.0307	0.8164	0.0106	0.9403	-0.1538	0.4318	0.1157	0.5160
56	rs2466315	0.1009	0.5724	0.0406	0.8307	0.0085	0.9733	0.1717	0.4927
57	rs2466316	0.1436	0.4111	-0.0058	0.9764	0.0921	0.7153	0.0756	0.7455
58	rs2466318	-0.0033	0.9772	-0.0128	0.9093	-0.2735	0.1146	0.1383	0.3663
59	rs2649098	0.0856	0.5351	-0.0709	0.6108	0.1829	0.3732	-0.0160	0.9367
60	rs2921722	-0.0448	0.6583	-0.0362	0.7129	0.0608	0.6846	0.0690	0.6091
61	rs2921739	0.0474	0.6531	-0.1419	0.1851	-0.2291	0.1453	-0.1073	0.4267
62	rs2921752	0.1911	0.3510	0.2353	0.3169	-0.0880	0.7800	-0.1267	0.6830
63	rs2921755	0.0052	0.9599	-0.0525	0.6226	-0.2266	0.1511	-0.0107	0.9170
64	rs2938860	0.0472	0.7480	0.1380	0.3479	0.1818	0.3997	0.2821	0.1583
65	rs2938864	-0.0770	0.4463	-0.0399	0.7150	-0.0177	0.9043	0.0684	0.6189
66	rs3019880	0.0674	0.5197	-0.0281	0.7865	0.0779	0.6174	-0.1525	0.2849
67	rs3019889	-0.1249	0.4998	0.0565	0.7951	-0.2124	0.4767	-0.4031	0.1422
68	rs3020106	-0.0362	0.8103	-0.1533	0.3343	0.2397	0.2711	0.0523	0.7929
69	rs3020108	-0.0450	0.6572	-0.0364	0.7118	0.0608	0.6846	0.0690	0.6091
70	rs3020116	0.1297	0.2013	0.0153	0.8295	-0.0201	0.8897	-0.1570	0.2501
71	rs3020119	0.0292	0.7776	-0.0339	0.7463	0.0238	0.8757	-0.1975	0.1635
72	rs3020156	0.0579	0.5853	-0.1769	0.1004	-0.2590	0.1048	-0.1415	0.3239
73	rs3020164	0.0243	0.8164	-0.1461	0.1671	-0.2542	0.1068	-0.1282	0.3594
74	rs3020167	0.0430	0.7049	-0.0784	0.4837	-0.2533	0.1390	-0.0371	0.8595
75	rs3020168	0.0400	0.7022	-0.1497	0.1579	-0.2397	0.1280	-0.1546	0.2800
76	rs3020172	0.1754	0.3552	0.2561	0.2140	-0.1564	0.5862	0.0872	0.6791
77	rs3020176	0.0055	0.9676	-0.2314	0.0813	0.2485	0.2126	-0.0473	0.7914
78	rs3020178	0.0464	0.7159	-0.0801	0.5358	0.2346	0.2036	0.0002	0.9254
79	rs3020180	0.0227	0.8665	-0.2580	0.0552	0.2367	0.2374	-0.0600	0.7341
80	rs3590145[illegible]	0.0000	1.0000	-0.1843	1.0000	0.0000	1.0000	1.2503	0.8824
81	rs3802177	0.0817	0.5147	-0.0834	0.5213	0.3217	0.0813	-0.3710	0.0317
82	rs4319130	-0.0501	0.7293	-0.1294	0.3636	-0.0874	0.6889	-0.2102	0.3033
83	rs4617186	-0.2209	0.0700	-0.0891	0.4292	-0.3181	0.0891	-0.0860	0.5895
84	rs6469667	-0.1080	0.2960	-0.0531	0.6175	-0.0531	0.7213	-0.0045	0.9707
85	rs6469668	-0.0667	0.5232	-0.0571	0.5976	-0.0664	0.6490	-0.0743	0.5909
86	rs6469674	-0.2354	0.2237	-0.0010	0.9352	-0.2729	0.3717	0.1193	0.6039
87	rs6469675	-0.0506	0.6870	0.0479	0.7058	0.0381	0.8437	0.3659	0.0279
88	rs6469681	-0.1375	0.3076	0.0182	0.9035	-0.3751	0.0642	0.1742	0.2970
89	rs6982630	-0.0070	0.9677	0.0597	0.7296	0.2796	0.2926	0.3770	0.1209
90	rs6984532	0.1462	0.8612	0.5744	0.4354	-0.2619	1.0000	4.3951	0.1017
91	rs6997684	-0.1358	0.2050	0.0078	0.9632	-0.1095	0.4881	0.0747	0.6111

92	rs6998120	0.1439	0.5136	-0.2744	0.2352	0.0701	0.8396	0.1105	0.6915
93	rs6999383	1.1650	0.9094	1.6044	1.0000	1.4726	0.9404	0.4056	0.9782
94	rs6999899	-0.1037	0.4230	0.0231	0.8468	-0.0795	0.6876	0.2476	0.1467
95	rs7000505	-0.0876	0.5085	0.0137	0.9312	0.0556	0.7900	0.2447	0.1842
96	rs7004119	0.2229	0.5588	0.0565	0.9190	0.2749	0.6393	0.4384	0.4097
97	rs7005140	0.1042	0.3505	0.0283	0.7901	0.3428	0.0348	-0.0585	0.7258
98	rs7011057	-0.1551	0.2328	0.0359	0.7617	-0.0938	0.6373	0.2247	0.1904
99	rs7016307	0.8845	1.0000	0.4158	0.9984	1.6675	0.9066	0.3543	0.9657
100	rs720131	-0.0210	0.8419	-0.0189	0.8505	-0.1840	0.2435	-0.0030	0.9630
101	rs720132	-0.0294	0.8048	0.1038	0.3692	0.0258	0.8853	0.0354	0.8326
102	rs720133	0.1158	0.2976	0.0270	0.8492	0.1710	0.2920	-0.0074	0.9445
103	rs7817754	-0.2461	0.0879	0.1433	0.3751	-0.0981	0.6499	0.3527	0.0610
104	rs7826859	0.6489	0.3764	0.2931	0.7828	-0.1639	1.0000	4.1928	0.0461
105	rs7827401	0.0108	0.9547	0.2479	0.1979	0.2820	0.3219	0.6060	0.0220
106	rs7828267	-0.1954	0.2189	0.1211	0.4270	-0.5927	0.0157	0.0231	0.6657
107	rs7828477	-0.0568	0.6550	-0.1250	0.3300	-0.2611	0.1813	-0.0387	0.8330
108	rs7831269	-0.0672	0.7389	0.3486	0.0862	0.2645	0.3596	0.6560	0.0147
109	rs7832958	-0.2689	0.1662	-0.0304	0.8169	-0.2878	0.3463	0.1004	0.6530
110	rs7833712	0.3987	0.3213	-0.5836	0.2315	4.7176	0.0113	-4.8235	0.1214
111	rs7833734	-0.1663	0.2305	-0.0213	0.8558	0.0080	0.9689	0.2057	0.2815
112	rs7840672	0.3928	0.4012	-0.1904	0.6521	0.1889	0.8122	-0.2350	0.8013
113	rs7843159	0.7255	0.3177	0.3323	0.7319	-0.1518	1.0000	4.0757	0.0420
114	rs7843392	9.6567	0.0017	-9.2169	0.2440	1.1060	0.9980	0.6444	0.9980
115	rs868651	-0.0649	0.6128	0.0365	0.7815	0.0391	0.8418	0.3326	0.0581
116	rs958637	0.0229	0.9270	0.2865	0.2537	-0.1087	0.7626	0.3691	0.2539
117	rs961630	0.1274	0.2911	-0.1497	0.2273	0.2111	0.2304	-0.0537	0.7651
118	rs9886445	-0.2138	0.1811	0.1774	0.2315	-0.5187	0.0340	0.1965	0.2302

Fasting glucose				Fasting insulin				2-hour	
age_b	age_p	sex_b	sex_p	age_b	age_p	sex_b	sex_p	age_b	age_p
0.0381	0.5748	0.0882	0.1488	0.0695	0.4047	-0.0100	0.8997	0.1063	0.1313
-0.0511	0.9465	-0.4980	0.2630	0.2519	0.7864	0.5983	0.2966	0.0177	0.9819
-0.0448	0.6784	-0.0713	0.4624	-0.0075	0.9552	-0.1372	0.2440	-0.1764	0.1186
-0.0031	0.9667	0.1274	0.0616	0.0262	0.7727	-0.0845	0.3237	0.1183	0.1228
0.0471	0.5823	-0.0442	0.5630	0.0790	0.4524	0.0820	0.3723	0.0459	0.6051
-0.0318	0.7981	0.0611	0.5766	0.1406	0.3607	0.1193	0.3385	-0.0347	0.7873
0.1110	0.2579	-0.0641	0.4675	-0.0205	0.8649	-0.0191	0.8539	0.1491	0.1454
0.1456	0.1591	-0.0818	0.4087	-0.0236	0.8526	0.0425	0.7138	0.2035	0.0601
0.2936	0.0165	0.0050	0.7766	-0.0329	0.8266	-0.0822	0.5255	0.0972	0.4485
0.0104	0.8925	-0.0103	0.8880	-0.1103	0.2471	0.0482	0.6015	-0.0186	0.8159
0.2395	0.0549	-0.0048	0.8450	-0.0838	0.5837	-0.0924	0.4593	0.0740	0.5717
0.2185	0.1180	0.0506	0.5529	-0.0658	0.7005	-0.0218	0.8500	0.1040	0.4728
0.1430	0.2697	0.0924	0.3584	-0.1148	0.4707	-0.0238	0.8129	0.1050	0.4340
0.0436	0.5344	-0.0540	0.4240	0.0958	0.2667	-0.0352	0.7091	0.1005	0.1678
-0.5846	0.4884	0.1694	0.5621	0.3970	0.7033	0.8560	0.1636	-0.7747	0.3651
0.0549	0.7957	0.0934	0.6082	0.3600	0.1653	0.0120	0.9726	-0.0243	0.9111
0.1309	0.6009	-0.3419	0.1451	0.0490	0.8732	0.0335	0.9095	0.2974	0.2608
-0.1508	0.1099	0.0354	0.7925	0.0982	0.3971	0.0179	0.8087	-0.2404	0.0130
0.0388	0.6311	-0.0128	0.8806	-0.0860	0.3872	-0.0136	0.8563	-0.1099	0.1872
0.1004	0.2917	-0.0128	0.8955	0.0566	0.6277	0.0999	0.3570	-0.0199	0.8415
-0.1047	0.2405	0.0752	0.3446	0.0089	0.9355	-0.0553	0.5686	-0.0338	0.7172
0.1649	0.0375	-0.0175	0.7737	-0.1261	0.1996	-0.0066	0.9713	-0.0087	0.9158
0.0948	0.2717	-0.0757	0.3176	0.1274	0.2294	0.0194	0.8296	-0.0487	0.5866
0.1544	0.1085	-0.0035	0.9870	0.0782	0.5076	0.0276	0.7756	-0.0197	0.8434
-0.0316	0.6740	0.0764	0.2860	-0.0440	0.6345	-0.0418	0.5965	0.1247	0.1160
0.0191	0.7955	-0.0621	0.3569	-0.0142	0.8746	-0.1307	0.1085	0.0646	0.3959
-0.1536	0.1133	0.0472	0.6395	0.0656	0.5839	0.1114	0.2863	0.0724	0.4694
-0.0792	0.5702	-0.0146	0.9284	0.2306	0.1765	0.2559	0.1031	-0.1246	0.3832
0.1134	0.1089	-0.0433	0.5207	0.0545	0.5310	0.0153	0.8353	0.1378	0.0623
0.0554	0.5169	0.1039	0.1508	-0.0922	0.3826	0.0570	0.5637	0.0539	0.5487
-0.1568	0.0972	0.0388	0.7637	0.0817	0.4824	0.0170	0.8282	-0.2323	0.0168
-0.0643	1.0000	0.2115	1.0000	-0.0710	1.0000	0.2685	1.0000	0.1624	1.0000
-0.0101	0.8853	0.0913	0.1533	0.0568	0.5134	0.0200	0.7413	0.0122	0.8670
-0.0363	0.6304	0.0764	0.2790	0.0030	0.9743	-0.0542	0.5275	0.0770	0.3334
0.1231	0.1777	-0.0528	0.5213	0.1042	0.3531	0.0676	0.5062	0.0479	0.6137
0.2920	0.5412	0.1959	0.6071	0.4827	0.4133	0.1663	0.7757	-0.0179	0.9706
0.3537	0.4560	0.1495	0.7391	0.5038	0.3880	0.1585	0.8058	0.0054	0.9911
0.3646	0.3830	0.2432	0.5477	0.2271	0.6573	0.3836	0.3623	0.5731	0.1746
-0.2185	0.4257	0.1039	0.7597	-0.1709	0.6120	0.6487	0.0309	0.1035	0.7114
-0.0056	0.9911	0.5968	0.2123	-0.6897	0.2805	1.0419	0.0571	0.1509	0.7682
0.0095	0.9331	-0.0617	0.5412	-0.0773	0.5820	-0.0324	0.8026	-0.1149	0.3348
-0.1149	0.5889	-0.4034	0.0426	-0.2332	0.3716	-0.4761	0.0545	-0.3073	0.1584
-0.1567	0.0978	0.0489	0.6829	0.0712	0.5409	0.0170	0.8337	-0.2419	0.0128
0.3045	0.7228	0.4120	0.5636	-0.0267	0.9798	1.0117	0.1799	0.1785	0.8378

-0.0159	0.8320	-0.0004	0.9856	-0.0409	0.6550	0.0724	0.4254	0.2330	0.0024
-0.0361	0.6152	-0.0043	0.9331	-0.0936	0.2867	0.0359	0.6851	-0.0588	0.4250
-0.0145	0.8322	0.0481	0.4507	0.0572	0.4958	0.0001	0.9694	0.0844	0.2342
-0.0718	0.5337	0.3121	0.0031	0.1828	0.2015	0.1204	0.2804	-0.0440	0.7123
-0.2051	0.0752	0.0712	0.5457	0.0141	0.9206	-0.0314	0.8092	-0.0888	0.4577
-0.1657	0.0376	0.0552	0.4838	0.0663	0.4986	0.0117	0.8745	-0.0703	0.3978
-0.1022	0.1745	0.0477	0.4779	0.0474	0.6091	-0.0623	0.4498	-0.0760	0.3305
0.1150	0.0963	0.0229	0.6860	0.1130	0.1843	0.0503	0.4951	0.1550	0.0313
-0.1712	0.0264	0.0230	0.7800	0.0338	0.7225	-0.0121	0.8940	-0.0621	0.4397
-0.1703	0.0516	-0.0113	0.8158	0.0054	0.9603	-0.0459	0.6291	-0.1455	0.1108
-0.1593	0.0691	-0.0238	0.6941	0.0049	0.9637	-0.0397	0.6775	-0.1414	0.1222
-0.2051	0.0752	0.0712	0.5457	0.0141	0.9206	-0.0314	0.8092	-0.0888	0.4577
-0.2100	0.0645	-0.0433	0.5978	0.0116	0.9335	-0.0972	0.4421	-0.1083	0.3578
-0.1433	0.0646	0.0355	0.6155	0.0792	0.4058	-0.0366	0.6735	-0.0664	0.4103
0.0715	0.4301	0.0320	0.6690	-0.0154	0.8894	0.1113	0.2679	-0.0445	0.6369
-0.0025	0.9703	0.0276	0.6601	-0.0135	0.8712	0.0344	0.6668	0.0522	0.4598
-0.0115	0.8704	0.0875	0.1736	0.0733	0.4025	0.0100	0.8176	0.0066	0.9288
-0.0293	0.8316	0.0346	0.7585	0.1731	0.3023	0.0987	0.6159	0.1632	0.2549
-0.0855	0.2232	0.1082	0.1119	0.0523	0.5476	-0.0531	0.5353	-0.0354	0.6274
0.0870	0.3822	0.0843	0.3281	0.0100	0.9344	0.0622	0.5655	0.0618	0.5518
0.0229	0.7322	0.0467	0.4391	-0.0071	0.9315	0.0651	0.3925	0.0547	0.4339
-0.0210	0.7613	-0.0277	0.6529	-0.0723	0.3929	-0.1713	0.0256	-0.1058	0.1425
-0.0630	0.6080	0.0485	0.6825	0.0060	0.9687	0.0279	0.8510	-0.0875	0.4916
0.0774	0.4190	0.0005	0.9803	0.0507	0.6664	0.0912	0.4022	-0.0130	0.8963
-0.0025	0.9703	0.0275	0.6618	-0.0135	0.8709	0.0344	0.6675	0.0522	0.4602
0.0084	0.8998	0.0360	0.5531	-0.0323	0.6956	-0.0776	0.2943	-0.0426	0.5445
-0.0073	0.9159	-0.0165	0.7893	-0.0346	0.6831	-0.1216	0.1136	-0.0909	0.2080
-0.0194	0.7851	0.0979	0.1299	0.0502	0.5681	-0.0229	0.8147	-0.0086	0.9077
-0.0099	0.8876	0.0913	0.1531	0.0567	0.5136	0.0200	0.7413	0.0124	0.8645
0.0597	0.4412	0.0987	0.1401	-0.0060	0.9502	0.0240	0.7748	0.1136	0.1615
-0.0061	0.9307	0.0895	0.1597	0.0605	0.4875	0.0157	0.7808	0.0149	0.8380
0.0382	0.7673	0.0483	0.7067	0.2129	0.1781	-0.0592	0.5779	0.2430	0.0704
-0.1089	0.2216	0.0779	0.3222	0.0018	0.9868	-0.0372	0.7004	-0.0435	0.6399
0.0811	0.3347	0.0465	0.5973	0.0419	0.6855	0.0633	0.5330	0.0609	0.4867
-0.0987	0.2715	0.0726	0.3621	0.0007	0.9951	-0.0704	0.4733	-0.0388	0.6795
0.0000	1.0000	-0.2608	1.0000	0.0000	1.0000	0.2580	1.0000	0.0000	1.0000
0.0061	0.9405	0.0045	0.9511	-0.1371	0.1716	0.0310	0.7679	-0.0999	0.2349
-0.1573	0.0966	0.0389	0.7631	0.0830	0.4758	0.0184	0.8181	-0.2335	0.0163
0.0148	0.8563	-0.0320	0.6651	0.0432	0.6676	-0.0275	0.7635	0.1685	0.0462
0.0182	0.7907	0.0811	0.1922	-0.0245	0.7722	0.0075	0.9341	0.0813	0.2582
-0.0137	0.8389	0.0219	0.7301	0.0375	0.6510	0.0696	0.3504	0.0318	0.6508
-0.1160	0.3955	-0.1070	0.3718	0.2081	0.2147	0.2432	0.0978	-0.1926	0.1680
0.0647	0.4426	-0.0858	0.2541	0.0785	0.4502	0.0874	0.3291	0.1095	0.2107
-0.0595	0.5124	0.0594	0.4739	0.1080	0.3333	-0.0340	0.7578	-0.0304	0.7499
0.2828	0.0129	-0.0489	0.7190	-0.0167	0.9044	-0.0637	0.6027	0.1446	0.2243
-0.3440	0.6985	0.4021	0.3382	0.8352	0.4469	1.2790	0.0867	-0.0118	0.9895
-0.0402	0.5619	-0.0122	0.8416	0.0313	0.7130	-0.0164	0.8353	0.1048	0.1460

-0.1016	0.5064	0.2119	0.1429	0.2874	0.1272	0.0497	0.6013	0.1497	0.3623
-0.1650	0.8606	0.7005	0.2961	1.3111	0.2597	1.3205	0.3239	-0.4619	0.6274
0.0430	0.6165	-0.0326	0.6653	0.1039	0.3244	0.0169	0.8446	-0.0080	0.9284
0.0595	0.4979	-0.0538	0.5073	0.1051	0.3289	0.0726	0.4338	0.0328	0.7183
-0.1603	0.4773	-0.2116	0.3048	-0.0939	0.7331	0.1185	0.6010	-0.1135	0.6236
0.0047	0.9475	-0.0190	0.7714	-0.0502	0.5645	-0.0524	0.5000	-0.0061	0.9336
0.0906	0.2945	-0.0685	0.3665	0.1294	0.2238	0.0264	0.7724	-0.0474	0.5977
-0.2586	1.0000	-0.1563	0.7826	0.0547	1.0000	0.1378	0.8420	-0.2012	1.0000
-0.0715	0.3109	0.0936	0.1694	0.0573	0.5127	-0.0211	0.8395	-0.0061	0.9343
0.1090	0.1758	-0.0759	0.2914	-0.0955	0.3393	-0.0302	0.7319	0.0051	0.9511
-0.0318	0.6618	-0.0217	0.7435	0.0812	0.3605	0.0214	0.7972	0.0528	0.4849
0.0186	0.8467	-0.0572	0.5127	0.0885	0.4539	0.0731	0.4653	-0.0951	0.3402
-0.6568	0.4186	0.0160	0.8021	0.3173	0.7522	0.3780	0.3570	-0.9365	0.2551
0.2698	0.0272	-0.0204	0.9786	0.0136	0.9276	-0.1601	0.2354	0.1015	0.4286
0.0259	0.8122	0.0052	0.9686	0.0097	0.9425	0.0351	0.7742	-0.0513	0.6484
0.0550	0.5225	0.1257	0.0892	-0.0657	0.5364	0.0393	0.6848	0.0252	0.7800
0.1620	0.1963	-0.0293	0.9115	0.0278	0.8569	-0.1564	0.2745	0.1295	0.3275
-0.1223	0.3721	-0.1167	0.3353	0.2203	0.1903	0.2501	0.0917	-0.1978	0.1588
0.1401	0.5913	-0.3389	0.1505	0.1383	0.6648	0.1255	0.7003	0.2652	0.3399
0.1516	0.1033	-0.0367	0.6718	0.0989	0.3857	0.0350	0.7315	0.0108	0.9109
-0.2966	0.2776	-0.0205	0.8355	-0.2028	0.5478	0.8168	0.0075	-0.0297	0.9158
-0.6961	0.3893	0.0113	0.8163	0.1960	0.8446	0.2542	0.5150	-1.0042	0.2196
1.3242	0.0817	-0.0996	0.9470	0.7097	0.4447	-0.0866	0.9669	0.1961	0.8016
0.0748	0.3811	-0.0339	0.6591	0.0566	0.5893	0.0787	0.3936	0.0257	0.7712
0.1149	0.4782	0.1766	0.1867	-0.2119	0.2891	0.1979	0.3044	0.2800	0.1019
-0.0466	0.5522	0.0676	0.3472	-0.0334	0.7274	0.0305	0.7341	-0.1177	0.1469
-0.0562	0.6006	-0.0195	0.8589	-0.0096	0.9422	-0.0873	0.4578	-0.1214	0.2798

glucose		2-hour insulin				HOMA-IR			
sex_b	sex_p	age_b	age_p	sex_b	sex_p	age_b	age_p	sex_b	sex_p
0.0473	0.4467	0.1063	0.2207	0.0364	0.6301	0.0746	0.3383	0.0102	0.8804
0.5210	0.2473	0.1637	0.8654	0.0835	0.9145	0.2372	0.7854	0.4629	0.3947
0.0379	0.6408	0.1315	0.3467	0.0169	0.9252	-0.0284	0.8192	-0.1330	0.2278
0.0120	0.7828	0.1418	0.1349	-0.0047	0.9548	0.0276	0.7452	-0.0559	0.4897
0.0150	0.8432	0.1457	0.1829	0.0498	0.5922	0.0769	0.4339	0.0755	0.3792
0.0920	0.4118	0.1740	0.2734	0.2311	0.0810	0.1140	0.4276	0.1119	0.3406
-0.0179	0.8563	0.0938	0.4577	-0.1400	0.2035	0.0097	0.9318	-0.0140	0.8881
0.0235	0.7493	0.1557	0.2418	-0.0831	0.5061	0.0144	0.9032	0.0397	0.7033
-0.0365	0.8016	0.0559	0.7237	-0.1089	0.4526	0.0239	0.8646	-0.0499	0.7036
-0.0403	0.5878	-0.0692	0.4844	0.0638	0.4955	-0.1031	0.2467	0.0373	0.6676
-0.0605	0.6509	0.0275	0.8650	-0.1241	0.3978	-0.0305	0.8310	-0.0570	0.6387
0.0434	0.6619	-0.0488	0.7848	-0.0134	0.9049	-0.0202	0.8997	0.0121	0.9436
-0.0258	0.8863	-0.0626	0.7072	-0.1042	0.4443	-0.0777	0.6016	0.0133	0.9566
0.0828	0.1853	0.1423	0.1156	0.0389	0.5746	0.0953	0.2374	-0.0479	0.5686
0.0600	0.6852	0.2872	0.7829	0.4870	0.4434	0.1775	0.8548	0.8441	0.1234
0.1150	0.5180	-0.0116	0.9653	-0.1946	0.3850	0.3156	0.1933	0.0268	0.9608
0.1138	0.6946	0.2188	0.5017	-0.1631	0.5575	0.0763	0.7905	-0.0576	0.8289
-0.0487	0.4452	-0.1524	0.2025	-0.1881	0.0703	0.0497	0.6465	0.0168	0.8356
-0.0704	0.3403	-0.1006	0.3297	0.0424	0.6820	-0.0754	0.4168	-0.0155	0.8321
0.0003	0.9975	0.0302	0.8048	0.0957	0.3976	0.0799	0.4636	0.0965	0.3388
0.0306	0.7050	0.1102	0.3402	0.0029	0.9751	-0.0200	0.8451	-0.0401	0.6553
0.0527	0.4784	-0.0838	0.4153	0.0511	0.5488	-0.0860	0.3490	-0.0010	0.9869
0.0545	0.4823	0.0837	0.4485	0.0885	0.3525	0.1238	0.2119	0.0173	0.8373
0.0062	0.9496	0.1143	0.3520	0.0658	0.5223	0.0903	0.4129	0.0405	0.6602
0.0195	0.7102	0.0738	0.4530	-0.0250	0.8244	-0.0400	0.6436	-0.0268	0.7076
-0.0936	0.1861	0.0603	0.5240	-0.1473	0.0888	-0.0192	0.8196	-0.1275	0.0932
0.0440	0.5987	0.1272	0.3061	0.0443	0.6463	0.0260	0.8165	0.1144	0.2502
0.0625	0.5914	0.2362	0.1791	0.3382	0.0343	0.1742	0.2750	0.2263	0.1205
-0.0140	0.8594	0.1005	0.2722	-0.0762	0.3653	0.0782	0.3360	0.0076	0.9003
0.0092	0.8908	-0.1247	0.2617	0.0229	0.8389	-0.0619	0.5304	0.0716	0.4210
-0.0682	0.3382	-0.1472	0.2194	-0.2195	0.0386	0.0338	0.7561	0.0165	0.8501
0.0621	1.0000	-0.0393	1.0000	-0.0485	1.0000	0.2524	1.0000	0.1207	1.0000
-0.0455	0.4975	-0.0543	0.5467	-0.1227	0.1145	0.0506	0.5325	0.0358	0.5746
0.0332	0.6251	0.0531	0.5918	-0.0025	0.9937	-0.0003	0.9964	-0.0381	0.6332
0.0217	0.7987	0.1615	0.1665	0.0278	0.7900	0.1105	0.2920	0.0628	0.5082
0.1213	0.7032	0.6074	0.3061	0.1779	0.7889	0.4549	0.4083	0.1775	0.7306
0.0936	0.7722	0.5549	0.3463	0.2172	0.7100	0.4888	0.3692	0.1600	0.7853
0.9270	0.0078	0.3752	0.4701	0.5394	0.2127	0.2432	0.6111	0.3764	0.3426
0.0045	0.9378	0.0335	0.9220	0.3393	0.2472	-0.1675	0.5945	0.5920	0.0358
-0.9159	0.0560	-0.5668	0.4373	-0.4088	0.5312	-0.5993	0.3152	1.0188	0.0491
0.0334	0.7153	0.1817	0.2179	0.0677	0.6370	-0.0751	0.5669	-0.0404	0.7368
-0.3473	0.0998	-0.0830	0.7606	-0.4453	0.0737	-0.2198	0.3675	-0.4836	0.0363
-0.0607	0.3747	-0.1612	0.1792	-0.2115	0.0449	0.0251	0.8175	0.0182	0.8430
-0.7383	0.2210	0.5185	0.6241	-0.4820	0.4515	-0.0069	0.9945	0.9588	0.1752

0.0477	0.4172	0.0638	0.5058	0.0252	0.7522	-0.0335	0.6959	0.0521	0.5408
-0.1502	0.0246	-0.1268	0.1646	-0.0925	0.2510	-0.0932	0.2570	0.0414	0.6121
0.0335	0.5622	0.1221	0.1630	0.0878	0.2394	0.0559	0.4761	0.0058	0.9053
0.0836	0.4529	0.1150	0.4408	0.1082	0.3679	0.1485	0.2669	0.1607	0.1428
0.0268	0.8283	-0.1350	0.3591	0.0011	0.9734	-0.0218	0.8691	-0.0189	0.8686
0.0411	0.5860	-0.0596	0.5618	0.0877	0.3403	0.0234	0.7980	0.0195	0.8039
0.0777	0.2536	0.0304	0.7530	0.0874	0.3032	0.0202	0.8158	-0.0417	0.5871
0.0288	0.6278	0.1222	0.1733	-0.0179	0.8530	0.1324	0.0960	0.0505	0.4589
0.0733	0.3011	0.0011	0.9912	0.0871	0.3168	-0.0056	0.9499	-0.0100	0.8972
0.0368	0.6764	-0.0535	0.6352	0.0717	0.4724	-0.0364	0.7173	-0.0461	0.5915
0.0307	0.7339	-0.0454	0.6881	0.0783	0.4312	-0.0342	0.7347	-0.0433	0.6154
0.0268	0.8283	-0.1350	0.3591	0.0011	0.9734	-0.0218	0.8691	-0.0189	0.8686
0.0736	0.5164	-0.1341	0.3555	0.0317	0.8451	-0.0290	0.8241	-0.0985	0.3951
0.0932	0.1856	0.0013	0.9898	0.0742	0.3956	0.0407	0.6478	-0.0267	0.7398
0.1286	0.1323	0.0179	0.8789	0.0951	0.3543	-0.0059	0.9545	0.1006	0.2817
0.0619	0.3109	0.0812	0.3526	0.0764	0.3012	-0.0038	0.9609	0.0391	0.5908
-0.0350	0.6020	-0.0456	0.6152	-0.1212	0.1215	0.0630	0.4404	0.0281	0.6325
0.0824	0.6283	0.2414	0.1829	0.0882	0.6917	0.1472	0.3485	0.0933	0.6010
-0.0971	0.1272	-0.0822	0.3657	-0.1426	0.0651	0.0295	0.7162	-0.0261	0.7479
0.1247	0.1655	0.0156	0.9035	-0.0054	0.9663	0.0255	0.8226	0.0781	0.4380
0.0805	0.1943	0.0529	0.5396	0.1025	0.1814	0.0047	0.9508	0.0711	0.3136
-0.1028	0.1033	-0.1799	0.0426	-0.1499	0.0515	-0.0738	0.3510	-0.1595	0.0257
-0.1558	0.2029	-0.1979	0.2066	-0.1240	0.4092	-0.0122	0.9314	0.0377	0.7859
-0.0024	0.9783	0.0334	0.7865	0.0574	0.6144	0.0703	0.5216	0.0907	0.3700
0.0620	0.3107	0.0812	0.3528	0.0763	0.3020	-0.0038	0.9607	0.0390	0.5918
-0.0651	0.2864	-0.1099	0.2063	-0.1498	0.0452	-0.0346	0.6531	-0.0697	0.3100
-0.0866	0.1691	-0.1265	0.1537	-0.1337	0.0854	-0.0376	0.6356	-0.1156	0.1067
-0.0624	0.3397	-0.0599	0.5116	-0.1554	0.0494	0.0420	0.6093	-0.0013	0.9733
-0.0456	0.4975	-0.0543	0.5467	-0.1227	0.1144	0.0506	0.5324	0.0358	0.5744
0.0095	0.8903	-0.0141	0.8894	-0.0134	0.8762	0.0150	0.8671	0.0427	0.5815
-0.0473	0.4831	-0.0530	0.5567	-0.1206	0.1213	0.0546	0.5018	0.0318	0.6104
0.0117	0.9403	0.2994	0.0768	-0.0971	0.4253	0.1988	0.1789	-0.0414	0.6515
0.0336	0.6742	0.1069	0.3541	0.0241	0.8141	-0.0281	0.7837	-0.0233	0.7940
-0.0027	0.9263	0.0529	0.6251	0.0154	0.9116	0.0563	0.5602	0.0669	0.4862
0.0425	0.6008	0.1008	0.3865	0.0158	0.8806	-0.0270	0.7947	-0.0552	0.5458
-0.5611	1.0000	0.0000	1.0000	-0.0434	1.0000	0.0000	1.0000	0.2068	1.0000
-0.0391	0.5960	-0.1490	0.1524	0.0799	0.4277	-0.1286	0.1699	0.0261	0.7937
-0.0691	0.3341	-0.1466	0.2220	-0.2172	0.0410	0.0350	0.7478	0.0177	0.8406
-0.0592	0.4370	-0.0192	0.8550	-0.1680	0.0736	0.0472	0.6160	-0.0316	0.7123
0.0399	0.5134	0.0587	0.5089	0.0589	0.4484	-0.0113	0.8865	0.0237	0.7496
0.0446	0.4756	0.0700	0.4202	0.1091	0.1575	0.0368	0.6339	0.0649	0.3507
0.0807	0.4933	0.1297	0.4521	0.2907	0.0522	0.1434	0.3608	0.1972	0.1498
0.0136	0.8383	0.1965	0.0683	-0.0019	0.9798	0.0769	0.4283	0.0737	0.3771
0.0761	0.3685	0.1013	0.3896	0.0847	0.4141	0.0814	0.4352	-0.0158	0.8845
-0.0395	0.7440	0.0861	0.5573	-0.1724	0.1873	0.0400	0.7590	-0.0505	0.6745
0.8069	0.1169	1.0813	0.3241	1.2656	0.1135	0.5799	0.5706	1.2321	0.0623
-0.0105	0.8786	0.0791	0.3749	0.0032	0.9711	0.0304	0.7019	-0.0185	0.8017

0.0569	0.5888	-0.0133	0.9474	0.1057	0.5481	0.2441	0.1656	0.0941	0.4193
0.4352	0.4204	0.5707	0.6221	1.1450	0.2830	1.0372	0.3384	1.2289	0.2897
0.0334	0.6630	0.1066	0.3320	0.0594	0.5211	0.0932	0.3444	0.0200	0.8070
0.0256	0.7434	0.1569	0.1612	0.0833	0.3789	0.1027	0.3077	0.0666	0.4411
0.1429	0.4514	0.0000	1.0000	0.1711	0.4847	-0.1048	0.6844	0.0915	0.6588
-0.1475	0.0275	-0.0534	0.5551	-0.1086	0.1844	-0.0465	0.5687	-0.0379	0.5983
0.0612	0.4304	0.0842	0.4467	0.0924	0.3322	0.1246	0.2104	0.0250	0.7687
-0.4548	0.4340	-0.1499	1.0000	0.2077	0.7701	0.1299	1.0000	0.0968	0.8810
-0.0662	0.3109	-0.0668	0.4649	-0.1309	0.0933	0.0363	0.6570	-0.0006	0.9705
0.0679	0.3541	-0.0208	0.8411	-0.0182	0.8448	-0.0690	0.4588	-0.0339	0.6812
0.0287	0.6760	0.1737	0.0612	0.1512	0.0736	0.0678	0.4150	0.0089	0.9110
0.0103	0.9476	0.0738	0.5478	0.0905	0.3867	0.0725	0.5111	0.0700	0.4575
0.1392	0.4711	0.2311	0.8175	0.3803	0.3516	0.0968	0.9176	0.3489	0.3362
-0.0688	0.5943	0.0676	0.6683	-0.1805	0.2068	0.0587	0.6749	-0.1194	0.3608
0.0867	0.3749	0.1652	0.2358	0.1786	0.1713	0.0098	0.9378	0.0283	0.8054
0.0127	0.8678	-0.1585	0.1558	0.0292	0.7729	-0.0384	0.6986	0.0629	0.4739
-0.0599	0.6967	0.0906	0.5774	-0.1727	0.2606	0.0526	0.7155	-0.1162	0.3987
0.0639	0.5802	0.1233	0.4753	0.2780	0.0650	0.1539	0.3282	0.2032	0.1414
0.0726	0.8819	0.0988	0.7727	-0.2906	0.3297	0.1593	0.5936	0.0266	0.9591
0.0005	0.9950	0.1183	0.3189	0.0051	0.9559	0.1111	0.2972	0.0391	0.6814
0.2676	0.2899	-0.1354	0.6941	0.5029	0.1110	-0.2112	0.5029	0.7359	0.0103
0.0931	0.5597	0.1194	0.9046	0.2713	0.4830	-0.0221	0.9811	0.2348	0.4886
-0.1594	0.7352	0.6920	0.4699	-0.1506	0.8726	0.8732	0.3150	-0.1115	0.9500
0.0145	0.8498	0.1184	0.2776	0.0506	0.5901	0.0616	0.5299	0.0751	0.3828
0.1201	0.2999	-0.1568	0.4571	0.0815	0.7173	-0.1509	0.4190	0.2207	0.2042
0.0979	0.1990	-0.0819	0.4143	0.0787	0.3989	-0.0373	0.6771	0.0342	0.6836
0.0747	0.3949	0.1493	0.2838	0.0720	0.6036	-0.0278	0.8219	-0.0838	0.4490

HOMA-β			
age_b	age_p	sex_b	sex_p
0.0040	0.9567	-0.0341	0.6088
0.0526	0.9503	0.5123	0.2913
-0.0071	0.9519	0.0643	0.5351
0.0391	0.6253	-0.1287	0.0875
0.0253	0.7868	0.1314	0.1086
-0.0679	0.6166	-0.1163	0.3081
-0.0068	0.9495	0.1440	0.1223
-0.0443	0.6942	0.1740	0.0840
-0.0560	0.6728	0.2069	0.0869
0.1364	0.1045	0.0736	0.3345
-0.0085	0.9500	0.2376	0.0518
-0.0550	0.7165	0.1410	0.3082
0.0111	0.9374	0.0639	0.5988
-0.1256	0.0999	-0.0070	0.8430
0.4527	0.6129	-0.2941	0.4516
-0.1075	0.6421	0.0666	0.7098
-0.1496	0.5837	0.2698	0.2870
-0.0015	0.9884	-0.0618	0.5178
0.0729	0.4056	0.0694	0.3773
0.0394	0.7039	0.0895	0.3502
0.1554	0.1109	-0.0879	0.3186
-0.1086	0.2094	0.0519	0.4858
-0.0680	0.4696	0.1633	0.0466
-0.0015	0.9884	0.1731	0.0633
0.0303	0.7117	-0.0677	0.3871
0.0196	0.8055	0.0123	0.8574
0.0853	0.4197	-0.1236	0.2116
0.0703	0.6428	0.0636	0.6497
-0.1203	0.1187	0.0064	0.9587
-0.0520	0.5748	-0.0548	0.4761
0.0030	0.9766	-0.0661	0.4945
-0.1026	1.0000	-0.1374	1.0000
-0.0700	0.3593	-0.0707	0.2586
0.0259	0.7539	-0.0827	0.2792
-0.0157	0.8745	0.1732	0.0530
-0.3515	0.4915	-0.2553	0.5298
-0.3985	0.4336	-0.2090	0.6401
-0.4005	0.3755	-0.2872	0.5042
-0.0462	0.8762	-0.1913	0.4442
0.0604	0.9144	-0.6777	0.1942
-0.0701	0.5695	0.0554	0.6055
-0.0118	0.9596	0.2647	0.2130
0.0034	0.9739	-0.0760	0.4341
-0.3900	0.6688	-0.5763	0.4525

0.0188	0.8176	-0.0526	0.5082
0.1306	0.0933	0.1181	0.0874
0.0215	0.7726	-0.0682	0.3267
0.1325	0.2975	-0.3130	0.0078
0.0958	0.4459	-0.1562	0.1763
0.0835	0.3375	-0.0339	0.6846
0.0433	0.5989	-0.0387	0.5937
-0.1285	0.0887	-0.0640	0.3231
0.0778	0.3563	-0.0285	0.7193
0.1121	0.2418	-0.0262	0.7868
0.1008	0.2932	-0.0140	0.8989
0.0958	0.4459	-0.1562	0.1763
0.1366	0.2705	-0.0496	0.6936
0.0512	0.5452	-0.0393	0.6021
-0.1135	0.2510	-0.0784	0.3430
0.0605	0.4097	0.0073	0.8607
-0.0700	0.3616	-0.0671	0.2790
-0.0902	0.5493	-0.1572	0.2820
0.0665	0.3855	-0.1006	0.1712
-0.0428	0.6917	0.0077	0.9433
0.0453	0.5349	-0.0110	0.8890
-0.0056	0.9407	0.0085	0.9039
-0.0411	0.7597	0.0216	0.8711
0.0626	0.5489	0.0785	0.4123
0.0606	0.4096	0.0074	0.8591
-0.0516	0.4805	-0.0605	0.3362
-0.0312	0.6786	0.0000	0.9878
-0.0599	0.4371	-0.0803	0.2164
-0.0701	0.3582	-0.0707	0.2584
-0.0928	0.2708	-0.0848	0.2347
-0.0731	0.3380	-0.0699	0.2623
-0.1438	0.3093	-0.1334	0.3505
0.1585	0.1034	-0.0918	0.2900
-0.0291	0.7517	-0.0214	0.8212
0.1513	0.1235	-0.0828	0.3477
0.0000	1.0000	0.2678	1.0000
0.1079	0.2218	0.0491	0.5288
0.0033	0.9743	-0.0668	0.4905
-0.0753	0.4010	0.0112	0.8930
0.0580	0.4379	-0.0315	0.6677
0.0439	0.5492	-0.0435	0.5356
0.0944	0.5249	0.1477	0.2510
0.0534	0.5599	0.1925	0.0158
0.0036	0.9707	-0.0018	0.9845
-0.0663	0.5911	0.2223	0.0443
0.1759	0.8519	-0.5610	0.2653
0.0946	0.2081	0.0304	0.6525

0.0107	0.9486	-0.0091	0.9581
0.0431	0.9655	-0.7589	0.3107
-0.0065	0.9448	0.1124	0.1654
0.0091	0.9246	0.1508	0.0767
0.1200	0.6277	0.1773	0.4283
0.1415	0.0669	0.1525	0.0268
-0.0637	0.4987	0.1561	0.0574
0.0759	1.0000	0.1923	0.7562
0.0449	0.5602	-0.0843	0.2482
-0.0556	0.5268	0.0958	0.2195
-0.0038	0.9615	-0.0180	0.8026
0.0908	0.3846	0.1915	0.0352
0.5713	0.5069	-0.0749	0.6946
-0.0326	0.8058	0.2266	0.0580
-0.1173	0.3217	-0.0848	0.4640
-0.0565	0.5448	-0.0792	0.3192
0.0202	0.8825	0.2145	0.0800
0.0969	0.5153	0.1545	0.2337
-0.1645	0.5629	0.2623	0.2956
-0.0097	0.9240	0.1917	0.0359
0.0021	0.9942	0.0072	0.9778
0.6121	0.4749	-0.0574	0.7307
-1.3739	0.1000	-0.0201	0.7053
0.0276	0.7672	0.1486	0.0692
0.0463	0.7938	0.0366	0.7927
-0.0125	0.8834	-0.1404	0.0637
0.0068	0.9532	-0.0076	0.9388

SUPPLEMENTARY TABLE 3

Model	BIC	Loglike	H2r	SE	bsnp_rs2938864	SE	bsnp_rs1001646
cov0	0	-644.099	0.6506335	0	0	0	0
cov9	2.799	-641.893	0.6568594	0	0	0	0
cov10	2.8645	-641.926	0.6577182	0	0	0	0
cov2	3.1002	-642.044	0.6513084	0	0	0	0.12280941
cov4	3.4645	-642.226	0.6508513	0	0	0	0
cov6	3.7831	-642.386	0.6563746	0	0	0	0
cov7	3.8745	-642.431	0.6505738	0	0	0	0
cov8	3.9674	-642.478	0.6505308	0	0	0	0
cov1	4.1665	-642.577	0.6566502	0	0.10614582	0	0
cov5	5.6132	-643.301	0.6523843	0	0	0	0
cov11	5.9333	-643.461	0.6503208	0	0	0	0
cov3	6.5832	-643.786	0.6481542	0	0	0	0
cov2_7	7.2906	-640.535	0.6523882	0	0	0	0.11822043
cov7_9	7.3647	-640.572	0.6556852	0	0	0	0
cov2_8	7.3781	-640.578	0.652335	0	0	0	0.11830478
cov7_10	7.4187	-640.599	0.6565204	0	0	0	0
cov8_9	7.4528	-640.616	0.6556638	0	0	0	0
cov4_10	7.4547	-640.617	0.656739	0	0	0	0
cov4_9	7.4648	-640.622	0.6558806	0	0	0	0
cov8_10	7.5069	-640.643	0.6564999	0	0	0	0
cov6_7	8.1268	-640.953	0.6570066	0	0	0	0
cov4_7	8.1455	-640.962	0.6515395	0	0	0	0
cov4_8	8.2228	-641.001	0.6514937	0	0	0	0
cov6_8	8.2241	-641.001	0.6569483	0	0	0	0
cov3_9	8.3134	-641.046	0.6522408	0	0	0	0
cov2_9	8.3315	-641.055	0.6559333	0	0	0	0.085569186
cov2_10	8.3452	-641.062	0.6566445	0	0	0	0.08655581
cov3_10	8.352	-641.065	0.6531891	0	0	0	0
cov1_7	8.4513	-641.115	0.6573758	0	0.099163941	0	0
cov1_8	8.5369	-641.158	0.657328	0	0.099297468	0	0
cov6_9	9.0191	-641.399	0.658846	0	0	0	0
cov6_10	9.0289	-641.404	0.6596066	0	0	0	0
cov2_3	9.0953	-641.437	0.6489997	0	0	0	0.13285509
cov5_9	9.1302	-641.454	0.6576566	0	0	0	0
cov5_10	9.1367	-641.458	0.6585357	0	0	0	0
cov1_9	9.157	-641.468	0.6592813	0	0.062341699	0	0
cov1_10	9.1696	-641.474	0.6600707	0	0.063869964	0	0
cov7_11	9.2196	-641.499	0.6510568	0	0	0	0
cov8_11	9.3238	-641.551	0.6509964	0	0	0	0
cov5_7	9.6189	-641.699	0.6533035	0	0	0	0
cov5_8	9.7076	-641.743	0.6532504	0	0	0	0
cov9_11	9.7588	-641.769	0.6565377	0	0	0	0
cov10_11	9.7987	-641.789	0.65733	0	0	0	0
cov3_6	9.8729	-641.826	0.6542969	0	0	0	0
cov9_10	10.0012	-641.89	0.6571005	0	0	0	0

cov2_4	10.0489	-641.914	0.6511971	0	0	0	0.080670476
cov4_5	10.0752	-641.927	0.6486932	0	0	0	0
cov2_6	10.1454	-641.962	0.6530938	0	0	0	0.091047655
cov4_6	10.1675	-641.973	0.6538662	0	0	0	0
cov2_5	10.1946	-641.987	0.6507369	0	0	0	0.14519078
cov2_11	10.2173	-641.998	0.651682	0	0	0	0.1393044
cov1_3	10.29	-642.034	0.6545964	0	0.11512543	0	0
cov1_2	10.3046	-642.042	0.651728	0	0.007887272	0	0.11620718
cov3_4	10.4074	-642.093	0.6495112	0	0	0	0
cov1_4	10.4451	-642.112	0.653267	0	0.042810572	0	0
cov4_11	10.6714	-642.225	0.6508087	0	0	0	0
cov3_7	10.6906	-642.235	0.653323	0	0	0	0
cov3_8	10.8207	-642.3	0.6530821	0	0	0	0
cov5_6	10.9013	-642.34	0.6569116	0	0	0	0
cov6_11	10.9084	-642.343	0.6574478	0	0	0	0
cov7_8	10.9565	-642.368	0.6506712	0	0	0	0
cov1_6	10.9744	-642.376	0.6565346	0	0.019969378	0	0
cov1_5	11.1285	-642.454	0.6582076	0	0.15904414	0	0
cov1_11	11.2829	-642.531	0.6580728	0	0.1278485	0	0
cov3_11	11.9688	-642.874	0.6469306	0	0	0	0
cov3_5	12.3522	-643.065	0.6504844	0	0	0	0
cov4_7_10	12.5577	-639.563	0.6564469	0	0	0	0
cov4_7_9	12.5651	-639.567	0.6556015	0	0	0	0
cov4_8_10	12.6329	-639.601	0.6564169	0	0	0	0
cov4_8_9	12.6404	-639.605	0.6555715	0	0	0	0
cov5_11	12.6742	-643.226	0.6516813	0	0	0	0
cov2_7_9	12.9178	-639.744	0.6557239	0	0	0	0.085102598
cov2_7_10	12.9265	-639.748	0.6564093	0	0	0	0.085946385
cov2_8_9	13.0034	-639.786	0.6556802	0	0	0	0.085164049
cov2_8_10	13.0122	-639.791	0.6563698	0	0	0	0.086008003
cov2_3_10	13.5992	-640.084	0.6532595	0	0	0	0.092468118
cov3_4_10	13.6056	-640.087	0.6538453	0	0	0	0
cov2_3_9	13.6146	-640.092	0.6523969	0	0	0	0.091531155
cov3_4_9	13.6327	-640.101	0.6528844	0	0	0	0
cov6_7_9	13.6867	-640.128	0.658391	0	0	0	0
cov6_7_10	13.6934	-640.131	0.6591368	0	0	0	0
cov5_7_9	13.7384	-640.154	0.6572537	0	0	0	0
cov5_7_10	13.7401	-640.155	0.6581338	0	0	0	0
cov6_8_9	13.7783	-640.174	0.6583523	0	0	0	0
cov6_8_10	13.7851	-640.177	0.6590986	0	0	0	0
cov1_7_9	13.8058	-640.188	0.6587953	0	0.059346555	0	0
cov1_7_10	13.8146	-640.192	0.6595753	0	0.060715052	0	0
cov5_8_9	13.8238	-640.197	0.6572228	0	0	0	0
cov5_8_10	13.8255	-640.197	0.6581009	0	0	0	0
cov1_8_9	13.8906	-640.23	0.6587627	0	0.059468245	0	0
cov1_8_10	13.8993	-640.234	0.659546	0	0.06084196	0	0
cov7_9_11	13.9705	-640.27	0.6555767	0	0	0	0

cov7_10_11	13.9891	-640.279	0.656349	0	0	0	0
cov4_5_9	14.0113	-640.29	0.653505	0	0	0	0
cov4_5_10	14.0301	-640.3	0.6544266	0	0	0	0
cov8_9_11	14.0663	-640.318	0.6555457	0	0	0	0
cov8_10_11	14.0851	-640.327	0.6563057	0	0	0	0
cov2_7_8	14.371	-640.47	0.6524691	0	0	0	0.11833126
cov2_5_7	14.3906	-640.48	0.6515545	0	0	0	0.14014519
cov3_6_10	14.4072	-640.488	0.6560712	0	0	0	0
cov2_6_7	14.4124	-640.491	0.6537545	0	0	0	0.095065444
cov3_6_9	14.4265	-640.498	0.6551801	0	0	0	0
cov7_8_9	14.4305	-640.5	0.6558043	0	0	0	0
cov2_4_7	14.4424	-640.506	0.6523244	0	0	0	0.09821861
cov2_3_7	14.4734	-640.521	0.6529621	0	0	0	0.11575049
cov2_5_8	14.4788	-640.524	0.6515073	0	0	0	0.14016513
cov4_10_11	14.4792	-640.524	0.6571087	0	0	0	0
cov4_9_11	14.4816	-640.525	0.6562087	0	0	0	0
cov7_8_10	14.4848	-640.527	0.6566415	0	0	0	0
cov2_7_11	14.4962	-640.533	0.6523367	0	0	0	0.11482475
cov1_2_7	14.4991	-640.534	0.6522021	0	-0.003255185	0	0.12093802
cov2_6_8	14.5022	-640.536	0.6536932	0	0	0	0.095446783
cov1_4_10	14.5166	-640.543	0.6549183	0	-0.038743626	0	0
cov1_4_9	14.5181	-640.544	0.6539167	0	-0.039995678	0	0
cov2_4_8	14.5271	-640.548	0.6522599	0	0	0	0.097812768
cov1_3_10	14.5401	-640.555	0.6564821	0	0.068039611	0	0
cov3_7_9	14.5472	-640.558	0.6549779	0	0	0	0
cov1_3_9	14.5579	-640.564	0.6555639	0	0.066518594	0	0
cov7_9_10	14.5676	-640.568	0.6559231	0	0	0	0
cov2_3_8	14.5698	-640.57	0.6528034	0	0	0	0.11629666
cov2_8_11	14.5845	-640.577	0.6523028	0	0	0	0.11528643
cov1_2_8	14.5867	-640.578	0.6521645	0	-0.003036446	0	0.12084072
cov3_7_10	14.5984	-640.584	0.6558048	0	0	0	0
cov3_8_9	14.6234	-640.596	0.6548074	0	0	0	0
cov4_6_10	14.6377	-640.603	0.6561783	0	0	0	0
cov4_6_9	14.6443	-640.607	0.6552463	0	0	0	0
cov4_9_10	14.6453	-640.607	0.6564019	0	0	0	0
cov8_9_10	14.6557	-640.613	0.6559014	0	0	0	0
cov2_4_10	14.6638	-640.617	0.6567463	0	0	0	-0.001978858
cov2_4_9	14.674	-640.622	0.6558921	0	0	0	-0.001500367
cov3_8_10	14.6741	-640.622	0.655652	0	0	0	0
cov3_4_7	14.7724	-640.671	0.6545926	0	0	0	0
cov4_6_7	14.8018	-640.686	0.6549906	0	0	0	0
cov4_6_8	14.8875	-640.728	0.6549109	0	0	0	0
cov3_4_8	14.8923	-640.731	0.6544092	0	0	0	0
cov3_5_10	14.893	-640.731	0.6547228	0	0	0	0
cov3_5_9	14.9068	-640.738	0.6537234	0	0	0	0
cov3_10_11	14.9725	-640.771	0.6525391	0	0	0	0
cov3_9_11	14.9752	-640.772	0.6516554	0	0	0	0

cov1_4_7	15.0644	-640.817	0.6545168	0	0.048360034	0	0
cov4_5_7	15.0791	-640.824	0.6496623	0	0	0	0
cov1_4_8	15.1435	-640.856	0.6544919	0	0.048215983	0	0
cov4_5_8	15.1518	-640.861	0.6495985	0	0	0	0
cov4_7_11	15.1584	-640.864	0.6513952	0	0	0	0
cov6_7_8	15.1846	-640.877	0.6571304	0	0	0	0
cov4_7_8	15.2277	-640.898	0.6516279	0	0	0	0
cov4_8_11	15.2428	-640.906	0.6513434	0	0	0	0
cov5_6_7	15.286	-640.928	0.6572995	0	0	0	0
cov3_6_7	15.3004	-640.935	0.6575984	0	0	0	0
cov6_7_11	15.3143	-640.942	0.6564859	0	0	0	0
cov1_6_7	15.3192	-640.944	0.6572135	0	0.01945279	0	0
cov2_9_11	15.3844	-640.977	0.6562724	0	0	0	0.10641295
cov5_6_8	15.3852	-640.977	0.6572342	0	0	0	0
cov2_10_11	15.4066	-640.988	0.6569854	0	0	0	0.10689009
cov3_6_8	15.4091	-640.989	0.6574286	0	0	0	0
cov6_8_11	15.4131	-640.991	0.6564332	0	0	0	0
cov1_6_8	15.4141	-640.992	0.6571643	0	0.020718019	0	0
cov1_4_5	15.4336	-641.001	0.6544879	0	0.16655369	0	0
cov1_2_9	15.4949	-641.032	0.6547607	0	-0.024728958	0	0.10514368
cov3_9_10	15.4992	-641.034	0.6526402	0	0	0	0
cov1_5_7	15.5048	-641.037	0.6584445	0	0.14139671	0	0
cov1_2_10	15.5155	-641.042	0.6555812	0	-0.022724001	0	0.10461517
cov2_9_10	15.53	-641.05	0.6563055	0	0	0	0.08564349
cov1_7_8	15.5317	-641.05	0.6574655	0	0.099258812	0	0
cov2_6_9	15.539	-641.054	0.6560846	0	0	0	0.082396545
cov2_5_9	15.5392	-641.054	0.6558408	0	0	0	0.088514774
cov2_6_10	15.5511	-641.06	0.6568605	0	0	0	0.082224814
cov2_5_10	15.5541	-641.062	0.6565903	0	0	0	0.088156376
cov1_5_8	15.59	-641.08	0.6584015	0	0.14158846	0	0
cov1_3_7	15.6086	-641.089	0.6581024	0	0.095533751	0	0
cov4_5_6	15.6164	-641.093	0.6533606	0	0	0	0
cov1_7_11	15.6327	-641.101	0.6565965	0	0.086602437	0	0
cov1_3_8	15.7071	-641.138	0.6579463	0	0.096164886	0	0
cov1_8_11	15.7213	-641.145	0.6565899	0	0.087419345	0	0
cov2_3_5	15.838	-641.204	0.6471079	0	0	0	0.18185228
cov2_3_6	16.1045	-641.337	0.6509455	0	0	0	0.097953999
cov6_9_11	16.1687	-641.369	0.6595732	0	0	0	0
cov5_6_9	16.1796	-641.374	0.6585597	0	0	0	0
cov6_10_11	16.1806	-641.375	0.6603239	0	0	0	0
cov5_6_10	16.1841	-641.377	0.6593393	0	0	0	0
cov6_9_10	16.2152	-641.392	0.6592024	0	0	0	0
cov1_6_9	16.2222	-641.396	0.6589937	0	0.011765181	0	0
cov1_6_10	16.2312	-641.4	0.6597437	0	0.012583697	0	0
cov5_7_11	16.2767	-641.423	0.6520296	0	0	0	0
cov3_5_7	16.2824	-641.426	0.6565352	0	0	0	0
cov2_3_11	16.2942	-641.432	0.6491945	0	0	0	0.13831401

cov1_2_3	16.2947	-641.432	0.6495809	0	0.011271883	0	0.12344063
cov3_7_11	16.3	-641.435	0.6526626	0	0	0	0
cov1_9_11	16.3012	-641.435	0.6602929	0	0.080728338	0	0
cov7_8_11	16.3035	-641.436	0.651143	0	0	0	0
cov2_3_4	16.3042	-641.437	0.6490119	0	0	0	0.13091973
cov1_5_9	16.3056	-641.437	0.6584523	0	0.028306774	0	0
cov1_5_10	16.3125	-641.441	0.6592769	0	0.028187473	0	0
cov5_9_11	16.315	-641.442	0.6579571	0	0	0	0
cov1_10_11	16.3153	-641.442	0.6615921	0	0.082150344	0	0
cov5_9_10	16.3162	-641.443	0.6580989	0	0	0	0
cov5_10_11	16.3222	-641.446	0.6588312	0	0	0	0
cov1_9_10	16.3528	-641.461	0.6596397	0	0.062615163	0	0
cov5_8_11	16.3759	-641.473	0.6519854	0	0	0	0
cov3_5_8	16.4144	-641.492	0.6563283	0	0	0	0
cov3_8_11	16.4253	-641.497	0.652457	0	0	0	0
cov2_4_5	16.4471	-641.508	0.6486326	0	0	0	0.094448278
cov1_3_5	16.4774	-641.523	0.6568751	0	0.23640577	0	0
cov3_5_6	16.6439	-641.607	0.655074	0	0	0	0
cov5_7_8	16.7031	-641.636	0.6533888	0	0	0	0
cov3_4_6	16.7352	-641.652	0.6530235	0	0	0	0
cov9_10_11	16.9578	-641.764	0.6568305	0	0	0	0
cov2_5_6	16.9756	-641.772	0.6532942	0	0	0	0.11079334
cov1_3_6	17.0549	-641.812	0.6544924	0	0.024618583	0	0
cov3_4_5	17.0741	-641.822	0.647504	0	0	0	0
cov3_6_11	17.0747	-641.822	0.6546791	0	0	0	0
cov2_6_11	17.0836	-641.826	0.6547824	0	0	0	0.1034043
cov4_5_11	17.1038	-641.837	0.6478049	0	0	0	0
cov1_3_4	17.1044	-641.837	0.6527503	0	0.068760086	0	0
cov2_4_6	17.1475	-641.858	0.6526945	0	0	0	0.058434181
cov2_4_11	17.1479	-641.859	0.6516269	0	0	0	0.097234304
cov4_6_11	17.2063	-641.888	0.6554173	0	0	0	0
cov1_2_6	17.2311	-641.9	0.6517482	0	-0.059807725	0	0.11014996
cov1_2_5	17.2391	-641.904	0.6532314	0	0.062394167	0	0.11772179
cov1_2_4	17.2579	-641.914	0.6510791	0	-0.002211702	0	0.082241806
cov1_4_6	17.3384	-641.954	0.6533978	0	-0.031180728	0	0
cov1_2_11	17.3632	-641.966	0.6535689	0	0.032708663	0	0.11961718
cov2_5_11	17.3645	-641.967	0.651039	0	0	0	0.1518973
cov1_3_11	17.4894	-642.029	0.6551315	0	0.12218512	0	0
cov3_4_11	17.5441	-642.057	0.6489695	0	0	0	0
cov1_4_11	17.5558	-642.063	0.6548282	0	0.064943179	0	0
cov3_7_8	17.7842	-642.177	0.6533157	0	0	0	0
cov1_5_6	17.9565	-642.263	0.6579947	0	0.072836755	0	0
cov1_6_11	18.0503	-642.31	0.6581696	0	0.041483413	0	0
cov5_6_11	18.0607	-642.315	0.6576567	0	0	0	0
cov1_5_11	18.252	-642.411	0.6594473	0	0.179108	0	0
cov3_5_11	19.0589	-642.814	0.6481383	0	0	0	0
cov4_5_7_9	19.4321	-639.396	0.6535251	0	0	0	0

cov4_5_7_10	19.4441	-639.402	0.6544426	0	0	0	0
cov4_5_8_9	19.502	-639.431	0.6534875	0	0	0	0
cov4_5_8_10	19.5141	-639.437	0.6544053	0	0	0	0
cov4_7_8_10	19.626	-639.493	0.6565584	0	0	0	0
cov4_7_8_9	19.6333	-639.497	0.6557099	0	0	0	0
cov1_4_7_10	19.6991	-639.53	0.6550617	0	-0.026431191	0	0
cov1_4_7_9	19.701	-639.53	0.6540853	0	-0.027550568	0	0
cov2_4_7_10	19.7224	-639.541	0.6564593	0	0	0	0.024483386
cov2_4_7_9	19.7282	-639.544	0.6556686	0	0	0	0.024908117
cov4_7_9_10	19.7501	-639.555	0.6561062	0	0	0	0
cov4_6_7_10	19.7629	-639.561	0.6562218	0	0	0	0
cov3_4_7_10	19.7651	-639.563	0.6566158	0	0	0	0
cov4_7_10_11	19.7657	-639.563	0.6564721	0	0	0	0
cov4_6_7_9	19.769	-639.564	0.6552998	0	0	0	0
cov3_4_7_9	19.7714	-639.566	0.6558202	0	0	0	0
cov4_7_9_11	19.7725	-639.566	0.6556217	0	0	0	0
cov1_4_8_10	19.7731	-639.567	0.655197	0	-0.026682315	0	0
cov1_4_8_9	19.7751	-639.568	0.6540454	0	-0.027782885	0	0
cov2_4_8_10	19.7996	-639.58	0.6564243	0	0	0	0.023923518
cov2_4_8_9	19.8055	-639.583	0.6556245	0	0	0	0.024352405
cov4_8_9_10	19.8253	-639.593	0.6560696	0	0	0	0
cov4_6_8_10	19.8372	-639.599	0.6561369	0	0	0	0
cov4_8_10_11	19.8402	-639.6	0.656443	0	0	0	0
cov3_4_8_10	19.8421	-639.601	0.6564658	0	0	0	0
cov4_6_8_9	19.8433	-639.602	0.6553658	0	0	0	0
cov4_8_9_11	19.8469	-639.603	0.6555963	0	0	0	0
cov3_4_8_9	19.8492	-639.605	0.6556639	0	0	0	0
cov2_7_8_9	19.9872	-639.674	0.655825	0	0	0	0.085015704
cov2_7_8_10	19.9961	-639.678	0.6565117	0	0	0	0.08594506
cov2_3_7_9	20.0422	-639.701	0.6546944	0	0	0	0.087011184
cov2_3_7_10	20.0444	-639.702	0.6554053	0	0	0	0.087930635
cov1_2_7_9	20.0483	-639.704	0.6540327	0	-0.03241545	0	0.11070754
cov1_2_7_10	20.0653	-639.713	0.6548473	0	-0.030586393	0	0.11020272
cov2_3_8_9	20.108	-639.734	0.6545635	0	0	0	0.087260586
cov2_3_8_10	20.1095	-639.735	0.6552721	0	0	0	0.088188006
cov2_7_9_10	20.1175	-639.739	0.6560377	0	0	0	0.085185673
cov2_5_7_9	20.1229	-639.741	0.6555357	0	0	0	0.089892034
cov2_7_9_11	20.1249	-639.742	0.6557474	0	0	0	0.087629171
cov2_6_7_9	20.1264	-639.743	0.6556144	0	0	0	0.087043423
cov2_5_7_10	20.1335	-639.747	0.6562707	0	0	0	0.089526444
cov2_7_10_11	20.1345	-639.747	0.656432	0	0	0	0.08793152
cov1_2_8_9	20.1351	-639.748	0.6540213	0	-0.032189982	0	0.1105968
cov2_6_7_10	20.1357	-639.748	0.6562757	0	0	0	0.086871121
cov1_2_8_10	20.152	-639.756	0.6548213	0	-0.030368669	0	0.11009618
cov2_8_9_10	20.2032	-639.782	0.656002	0	0	0	0.08524958
cov2_5_8_9	20.2087	-639.784	0.6555132	0	0	0	0.089866808
cov2_8_9_11	20.2099	-639.785	0.6557169	0	0	0	0.0880701

cov2_6_8_9	20.2119	-639.786	0.6555762	0	0	0	0.087393165
cov2_5_8_10	20.2193	-639.79	0.6562663	0	0	0	0.089496544
cov2_8_10_11	20.2196	-639.79	0.6564011	0	0	0	0.08837422
cov2_6_8_10	20.2212	-639.791	0.6563038	0	0	0	0.087226901
cov3_4_5_9	20.2952	-639.828	0.6506874	0	0	0	0
cov3_4_5_10	20.2986	-639.829	0.6517267	0	0	0	0
cov2_3_4_10	20.6489	-640.004	0.6536044	0	0	0	0.050504652
cov2_3_5_9	20.6601	-640.01	0.651209	0	0	0	0.12284169
cov2_3_5_10	20.6612	-640.011	0.6521001	0	0	0	0.12228665
cov2_3_4_9	20.6735	-640.017	0.6526641	0	0	0	0.050858936
cov6_7_8_9	20.7412	-640.051	0.658523	0	0	0	0
cov6_7_8_10	20.7478	-640.054	0.6592725	0	0	0	0
cov1_2_3_10	20.7633	-640.062	0.6520818	0	-0.024508689	0	0.11193794
cov1_2_3_9	20.7709	-640.065	0.6510981	0	-0.026596521	0	0.11257656
cov2_3_10_11	20.7891	-640.075	0.6534751	0	0	0	0.09983303
cov2_3_9_10	20.7919	-640.076	0.6529295	0	0	0	0.091736193
cov1_4_5_7	20.7929	-640.076	0.6548463	0	0.15062763	0	0
cov3_4_9_10	20.796	-640.078	0.653502	0	0	0	0
cov2_3_9_11	20.8003	-640.08	0.6526344	0	0	0	0.0996256
cov1_3_4_10	20.8052	-640.083	0.6533889	0	-0.01039904	0	0
cov3_4_6_10	20.8065	-640.083	0.6541615	0	0	0	0
cov2_3_6_10	20.8068	-640.083	0.6534129	0	0	0	0.08931636
cov3_4_10_11	20.8074	-640.084	0.6539951	0	0	0	0
cov5_7_8_9	20.8075	-640.084	0.6573609	0	0	0	0
cov5_7_8_10	20.8094	-640.085	0.6582418	0	0	0	0
cov2_3_6_9	20.8232	-640.092	0.6524737	0	0	0	0.089520883
cov1_3_4_9	20.8295	-640.095	0.6523467	0	-0.011796257	0	0
cov5_6_7_9	20.8312	-640.096	0.6581159	0	0	0	0
cov5_6_7_10	20.8319	-640.096	0.6589056	0	0	0	0
cov3_4_9_11	20.8321	-640.096	0.653048	0	0	0	0
cov3_6_7_9	20.8324	-640.096	0.6574754	0	0	0	0
cov3_6_7_10	20.8328	-640.096	0.658227	0	0	0	0
cov3_4_6_9	20.8356	-640.098	0.653174	0	0	0	0
cov1_4_5_8	20.8609	-640.11	0.6548032	0	0.150868	0	0
cov6_7_9_11	20.868	-640.114	0.6578734	0	0	0	0
cov6_7_10_11	20.8732	-640.117	0.6586085	0	0	0	0
cov1_7_8_9	20.8742	-640.117	0.6589006	0	0.059318646	0	0
cov4_5_6_7	20.8778	-640.119	0.6538592	0	0	0	0
cov1_7_8_10	20.8832	-640.122	0.6596829	0	0.060694012	0	0
cov6_7_9_10	20.8844	-640.122	0.65874	0	0	0	0
cov1_6_7_9	20.8896	-640.125	0.6585484	0	0.012026928	0	0
cov1_6_7_10	20.8954	-640.128	0.659302	0	0.012774401	0	0
cov5_7_9_11	20.8986	-640.129	0.6568395	0	0	0	0
cov5_7_10_11	20.8993	-640.13	0.6576841	0	0	0	0
cov3_6_8_10	20.905	-640.132	0.6580787	0	0	0	0
cov3_6_8_9	20.9053	-640.133	0.6573211	0	0	0	0
cov5_6_8_9	20.9201	-640.14	0.6580697	0	0	0	0

cov5_6_8_10	20.9208	-640.14	0.65887	0	0	0	0
cov5_7_9_10	20.9263	-640.143	0.6577391	0	0	0	0
cov1_5_7_9	20.9318	-640.146	0.6578464	0	0.019443082	0	0
cov1_5_7_10	20.934	-640.147	0.6586753	0	0.019151521	0	0
cov3_5_7_9	20.9476	-640.154	0.6571971	0	0	0	0
cov3_5_7_10	20.949	-640.154	0.6580369	0	0	0	0
cov1_3_7_9	20.9536	-640.157	0.6578984	0	0.060948635	0	0
cov1_3_7_10	20.9561	-640.158	0.6586703	0	0.062425898	0	0
cov4_5_6_8	20.9588	-640.159	0.6537942	0	0	0	0
cov6_8_9_11	20.9614	-640.161	0.6578495	0	0	0	0
cov6_8_10_11	20.9667	-640.163	0.6585845	0	0	0	0
cov6_8_9_10	20.976	-640.168	0.6587066	0	0	0	0
cov1_6_8_9	20.9799	-640.17	0.6585246	0	0.013220479	0	0
cov1_7_9_11	20.9806	-640.17	0.6580058	0	0.045319269	0	0
cov1_6_8_10	20.9857	-640.173	0.6592913	0	0.013966585	0	0
cov5_8_9_11	20.9874	-640.174	0.6568208	0	0	0	0
cov5_8_10_11	20.9881	-640.174	0.657666	0	0	0	0
cov1_7_10_11	20.9882	-640.174	0.6587754	0	0.046443455	0	0
cov1_7_9_10	21.003	-640.181	0.6591488	0	0.059602993	0	0
cov5_8_9_10	21.0116	-640.186	0.657687	0	0	0	0
cov1_5_8_9	21.0168	-640.188	0.6578196	0	0.019635825	0	0
cov1_5_8_10	21.0191	-640.19	0.6586533	0	0.019354723	0	0
cov1_3_8_9	21.0213	-640.191	0.6577512	0	0.061269586	0	0
cov1_3_8_10	21.0229	-640.191	0.6585376	0	0.062743495	0	0
cov3_5_8_9	21.0309	-640.195	0.6570131	0	0	0	0
cov3_5_8_10	21.0318	-640.196	0.6578637	0	0	0	0
cov7_8_9_11	21.0398	-640.2	0.6557021	0	0	0	0
cov7_8_10_11	21.0586	-640.209	0.6564511	0	0	0	0
cov1_8_9_11	21.0688	-640.214	0.658017	0	0.046162292	0	0
cov1_8_10_11	21.0764	-640.218	0.6587855	0	0.047286762	0	0
cov1_8_9_10	21.0878	-640.224	0.6591221	0	0.059728429	0	0
cov4_5_6_9	21.0884	-640.224	0.6544863	0	0	0	0
cov4_5_6_10	21.1037	-640.232	0.6553162	0	0	0	0
cov1_4_5_9	21.1092	-640.235	0.6548497	0	0.051557969	0	0
cov1_4_5_10	21.1272	-640.244	0.6556594	0	0.051813663	0	0
cov3_7_9_11	21.1278	-640.244	0.6546056	0	0	0	0
cov3_7_10_11	21.1407	-640.25	0.6553597	0	0	0	0
cov7_9_10_11	21.1685	-640.264	0.6559044	0	0	0	0
cov4_5_9_10	21.2049	-640.282	0.653919	0	0	0	0
cov2_4_5_9	21.2071	-640.284	0.6534313	0	0	0	0.013460527
cov3_8_9_11	21.2074	-640.284	0.6544416	0	0	0	0
cov4_5_9_11	21.213	-640.286	0.6536977	0	0	0	0
cov2_4_5_7	21.2162	-640.288	0.6498926	0	0	0	0.10747953
cov3_8_10_11	21.2198	-640.29	0.6551972	0	0	0	0
cov2_4_5_10	21.2267	-640.293	0.6543414	0	0	0	0.013069592
cov4_5_10_11	21.2323	-640.296	0.6546326	0	0	0	0
cov8_9_10_11	21.2643	-640.312	0.6558697	0	0	0	0

cov2_4_5_8	21.2955	-640.328	0.6498346	0	0	0	0.10713311
cov2_5_6_7	21.3228	-640.341	0.6536614	0	0	0	0.11257845
cov1_3_4_5	21.3429	-640.351	0.6535673	0	0.23124028	0	0
cov2_5_6_8	21.4161	-640.388	0.6535681	0	0	0	0.11286569
cov2_5_7_8	21.4698	-640.415	0.6516319	0	0	0	0.1403414
cov1_2_6_7	21.4791	-640.42	0.6521798	0	-0.064448476	0	0.11555328
cov2_6_7_8	21.4865	-640.423	0.6538895	0	0	0	0.094351487
cov2_4_7_8	21.5231	-640.442	0.652396	0	0	0	0.098381073
cov1_2_5_7	21.5293	-640.445	0.653243	0	0.040992615	0	0.1222036
cov2_3_4_7	21.5531	-640.457	0.653554	0	0	0	0.078689749
cov2_3_7_8	21.5566	-640.458	0.6530071	0	0	0	0.11602741
cov2_5_7_11	21.5637	-640.462	0.6512157	0	0	0	0.13354972
cov1_2_6_8	21.5745	-640.467	0.6521379	0	-0.063148971	0	0.11553752
cov2_7_8_11	21.5768	-640.468	0.6524182	0	0	0	0.11500865
cov1_2_7_8	21.5795	-640.47	0.6522879	0	-0.003184977	0	0.12098531
cov2_4_6_7	21.5818	-640.471	0.6535748	0	0	0	0.080472821
cov2_3_5_7	21.5989	-640.479	0.6517105	0	0	0	0.13868308
cov3_6_9_10	21.6007	-640.48	0.6557238	0	0	0	0
cov3_5_6_10	21.6014	-640.481	0.6561602	0	0	0	0
cov1_3_6_10	21.6034	-640.482	0.6562795	0	0.0170919	0	0
cov2_3_6_7	21.6051	-640.483	0.6541515	0	0	0	0.094271117
cov3_7_8_9	21.6091	-640.485	0.6550492	0	0	0	0
cov3_6_10_11	21.6151	-640.488	0.6559268	0	0	0	0
cov2_6_7_11	21.6156	-640.488	0.6540054	0	0	0	0.096924786
cov1_2_5_8	21.6166	-640.488	0.6532069	0	0.041261803	0	0.12210451
cov3_5_6_9	21.6172	-640.489	0.6552995	0	0	0	0
cov1_3_6_9	21.6242	-640.492	0.6553799	0	0.016145675	0	0
cov7_8_9_10	21.6334	-640.497	0.6560429	0	0	0	0
cov3_6_9_11	21.635	-640.497	0.6550651	0	0	0	0
cov1_2_4_7	21.6468	-640.503	0.6518563	0	-0.008071102	0	0.10395374
cov2_4_7_11	21.6503	-640.505	0.6522677	0	0	0	0.096347168
cov2_5_8_11	21.6547	-640.507	0.6511822	0	0	0	0.13383545
cov2_3_4_8	21.6562	-640.508	0.6533766	0	0	0	0.080212431
cov3_7_8_10	21.6603	-640.51	0.6558801	0	0	0	0
cov1_4_9_11	21.6611	-640.51	0.6551046	0	-0.021454915	0	0
cov1_4_10_11	21.6613	-640.511	0.6560238	0	-0.020437108	0	0
cov4_9_10_11	21.663	-640.511	0.6567161	0	0	0	0
cov2_4_10_11	21.6648	-640.512	0.6571431	0	0	0	0.018988536
cov2_4_9_11	21.6649	-640.512	0.6562843	0	0	0	0.019859473
cov2_4_6_8	21.6689	-640.514	0.6534978	0	0	0	0.080363973
cov1_2_4_9	21.6755	-640.518	0.6532207	0	-0.054218476	0	0.030584533
cov1_4_6_10	21.6783	-640.519	0.654908	0	-0.066112846	0	0
cov1_2_4_10	21.6792	-640.52	0.6542375	0	-0.052220085	0	0.029077353
cov2_3_7_11	21.6801	-640.52	0.6529126	0	0	0	0.11288198
cov1_2_3_7	21.6809	-640.52	0.6526949	0	-0.004981495	0	0.11984975
cov1_4_6_9	21.6817	-640.521	0.6539219	0	-0.066761361	0	0
cov4_6_10_11	21.6846	-640.522	0.6573913	0	0	0	0

cov1_3_5_10	21.6853	-640.523	0.6571202	0	0.10595528	0	0
cov4_6_9_11	21.688	-640.524	0.6562215	0	0	0	0
cov2_3_5_8	21.6881	-640.524	0.6515053	0	0	0	0.14018205
cov1_3_5_9	21.6965	-640.528	0.6562889	0	0.10626331	0	0
cov1_4_9_10	21.6976	-640.529	0.6544288	0	-0.040124499	0	0
cov1_2_7_11	21.7006	-640.53	0.6517755	0	-0.009311133	0	0.1202208
cov2_3_6_8	21.7018	-640.531	0.6539809	0	0	0	0.094870216
cov2_6_8_11	21.7043	-640.532	0.6539564	0	0	0	0.097440597
cov1_3_9_10	21.7313	-640.546	0.6561132	0	0.066993969	0	0
cov1_2_4_8	21.7316	-640.546	0.6518085	0	-0.007964039	0	0.10347244
cov2_4_8_11	21.7354	-640.548	0.6522324	0	0	0	0.096286592
cov3_4_5_7	21.7413	-640.551	0.6527012	0	0	0	0
cov3_7_9_10	21.7479	-640.554	0.6552208	0	0	0	0
cov1_3_10_11	21.7483	-640.554	0.6563147	0	0.06553091	0	0
cov4_5_7_11	21.7662	-640.563	0.6482598	0	0	0	0
cov1_3_9_11	21.7665	-640.563	0.6554274	0	0.064494377	0	0
cov3_4_6_7	21.7724	-640.566	0.6560541	0	0	0	0
cov2_3_8_11	21.777	-640.568	0.6527531	0	0	0	0.11372251
cov1_2_3_8	21.7776	-640.569	0.6525609	0	-0.004436239	0	0.11994945
cov1_2_8_11	21.7898	-640.575	0.6517787	0	-0.008466707	0	0.12019502
cov3_8_9_10	21.8236	-640.592	0.6550703	0	0	0	0
cov4_6_9_10	21.8242	-640.592	0.6557619	0	0	0	0
cov2_4_6_10	21.8438	-640.602	0.6560751	0	0	0	0.007172698
cov4_5_8_11	21.8485	-640.604	0.6482285	0	0	0	0
cov2_4_6_9	21.8492	-640.605	0.6551332	0	0	0	0.00834411
cov2_4_9_10	21.8542	-640.607	0.656407	0	0	0	-0.002484504
cov3_4_5_8	21.855	-640.607	0.6525069	0	0	0	0
cov3_4_7_8	21.8676	-640.614	0.6546289	0	0	0	0
cov4_6_7_8	21.8697	-640.615	0.6551259	0	0	0	0
cov3_4_6_8	21.8867	-640.623	0.6558913	0	0	0	0
cov3_4_5_6	21.9021	-640.631	0.6522385	0	0	0	0
cov3_4_7_11	21.934	-640.647	0.6542696	0	0	0	0
cov1_3_4_7	21.9355	-640.648	0.6555095	0	0.021687295	0	0
cov1_4_6_7	21.9926	-640.676	0.654615	0	-0.021712339	0	0
cov4_6_7_11	22.0103	-640.685	0.654892	0	0	0	0
cov3_5_10_11	22.0268	-640.693	0.6538647	0	0	0	0
cov3_5_9_11	22.0432	-640.702	0.6529042	0	0	0	0
cov1_3_4_8	22.0496	-640.705	0.6553834	0	0.022999273	0	0
cov3_4_8_11	22.0538	-640.707	0.6540611	0	0	0	0
cov3_5_9_10	22.0736	-640.717	0.6543266	0	0	0	0
cov1_4_6_8	22.0798	-640.72	0.6545594	0	-0.020789054	0	0
cov4_6_8_11	22.0964	-640.728	0.6548351	0	0	0	0
cov1_4_7_8	22.1458	-640.753	0.6546351	0	0.048442529	0	0
cov3_9_10_11	22.1497	-640.755	0.6521445	0	0	0	0
cov4_5_7_8	22.1595	-640.76	0.6497396	0	0	0	0
cov2_3_5_6	22.1652	-640.763	0.6504393	0	0	0	0.13721139
cov4_7_8_11	22.2413	-640.801	0.6514895	0	0	0	0

cov1_4_7_11	22.2557	-640.808	0.6538939	0	0.03863441	0	0
cov1_4_8_11	22.3371	-640.849	0.6538662	0	0.039135689	0	0
cov5_6_7_8	22.3386	-640.849	0.6574387	0	0	0	0
cov1_2_3_5	22.3523	-640.856	0.6517534	0	0.13467206	0	0.1301741
cov3_6_7_8	22.3628	-640.861	0.657672	0	0	0	0
cov6_7_8_11	22.3747	-640.867	0.6566297	0	0	0	0
cov1_6_7_8	22.3807	-640.87	0.6573082	0	0.017294335	0	0
cov1_5_6_7	22.3866	-640.873	0.6582232	0	0.06139671	0	0
cov5_6_7_11	22.4526	-640.906	0.6566239	0	0	0	0
cov1_5_6_8	22.4808	-640.92	0.6581807	0	0.062752624	0	0
cov1_4_5_6	22.482	-640.921	0.6546098	0	0.10977141	0	0
cov3_5_6_7	22.4869	-640.923	0.6575586	0	0	0	0
cov3_6_7_11	22.4889	-640.924	0.6570809	0	0	0	0
cov1_3_6_7	22.4946	-640.927	0.6577804	0	0.01839509	0	0
cov1_6_7_11	22.5172	-640.939	0.6567352	0	0.012928447	0	0
cov2_6_9_11	22.5419	-640.951	0.6572221	0	0	0	0.093333733
cov5_6_8_11	22.5545	-640.957	0.6565684	0	0	0	0
cov2_6_10_11	22.5581	-640.959	0.6579574	0	0	0	0.093060541
cov1_4_5_11	22.565	-640.962	0.655779	0	0.18562995	0	0
cov2_5_9_11	22.5805	-640.97	0.6565977	0	0	0	0.0997003
cov1_5_7_8	22.5829	-640.971	0.6585449	0	0.14176195	0	0
cov2_9_10_11	22.5855	-640.973	0.6565565	0	0	0	0.10632834
cov3_5_6_8	22.5914	-640.976	0.6573896	0	0	0	0
cov1_2_9_11	22.5932	-640.977	0.6562837	0	-0.003149218	0	0.1081948
cov2_5_10_11	22.5988	-640.979	0.6573827	0	0	0	0.09921365
cov3_6_8_11	22.5989	-640.979	0.656924	0	0	0	0
cov1_3_6_8	22.6006	-640.98	0.6576448	0	0.019976734	0	0
cov1_6_8_11	22.614	-640.987	0.6567306	0	0.014787322	0	0
cov1_2_10_11	22.6158	-640.988	0.656851	0	-0.00135754	0	0.10766458
cov1_2_6_9	22.6161	-640.988	0.6545811	0	-0.06195864	0	0.10212507
cov1_2_6_10	22.6334	-640.997	0.6553852	0	-0.060747857	0	0.10156225
cov1_2_4_5	22.6351	-640.998	0.6548819	0	0.17762234	0	-0.013051614
cov1_2_5_9	22.67	-641.015	0.6542703	0	-0.049154664	0	0.10371612
cov1_5_7_11	22.685	-641.022	0.6576338	0	0.12874454	0	0
cov1_2_5_10	22.6871	-641.024	0.6550903	0	-0.048481618	0	0.10310538
cov1_3_7_8	22.6929	-641.026	0.6581515	0	0.095755004	0	0
cov1_2_9_10	22.695	-641.027	0.6550881	0	-0.024289547	0	0.1048801
cov1_3_5_7	22.7133	-641.037	0.6583915	0	0.14447653	0	0
cov1_7_8_11	22.7136	-641.037	0.6567067	0	0.086755338	0	0
cov2_3_4_5	22.7234	-641.042	0.6462912	0	0	0	0.14388076
cov2_6_9_10	22.7372	-641.049	0.6564251	0	0	0	0.082262202
cov2_5_9_10	22.7384	-641.049	0.6561789	0	0	0	0.087912284
cov2_5_6_9	22.742	-641.051	0.6560589	0	0	0	0.08539563
cov2_5_6_10	22.7558	-641.058	0.6568174	0	0	0	0.084819386
cov2_4_5_6	22.768	-641.064	0.6526338	0	0	0	0.029790269
cov1_5_8_11	22.7733	-641.067	0.65768	0	0.12961155	0	0
cov1_3_7_11	22.7909	-641.075	0.6573359	0	0.083204441	0	0

cov1_3_5_8	22.7948	-641.077	0.6582782	0	0.14883952	0	0
cov4_5_6_11	22.8065	-641.083	0.6538757	0	0	0	0
cov1_3_8_11	22.8923	-641.126	0.6572241	0	0.0845225	0	0
cov2_3_5_11	23.0043	-641.182	0.6464284	0	0	0	0.17657493
cov1_2_3_6	23.1886	-641.274	0.6496153	0	-0.060209449	0	0.117226
cov2_3_6_11	23.212	-641.286	0.6522101	0	0	0	0.10525279
cov3_5_7_11	23.2295	-641.295	0.6548092	0	0	0	0
cov1_3_5_6	23.2711	-641.316	0.6567174	0	0.14723879	0	0
cov5_6_9_11	23.294	-641.327	0.6594117	0	0	0	0
cov5_6_10_11	23.2993	-641.33	0.6602076	0	0	0	0
cov2_3_4_6	23.3097	-641.335	0.6509444	0	0	0	0.10330981
cov1_6_9_11	23.3449	-641.352	0.6601131	0	0.029208859	0	0
cov1_6_10_11	23.3552	-641.358	0.660857	0	0.029888329	0	0
cov3_5_8_11	23.358	-641.359	0.6545663	0	0	0	0
cov5_7_8_11	23.361	-641.36	0.6521125	0	0	0	0
cov6_9_10_11	23.3659	-641.363	0.6599066	0	0	0	0
cov5_6_9_10	23.3711	-641.366	0.6589471	0	0	0	0
cov3_5_7_8	23.3802	-641.37	0.6565635	0	0	0	0
cov1_5_6_9	23.3811	-641.371	0.6582435	0	-0.017501115	0	0
cov1_5_6_10	23.385	-641.372	0.6590473	0	-0.018192252	0	0
cov3_7_8_11	23.3906	-641.375	0.652695	0	0	0	0
cov1_6_9_10	23.4181	-641.389	0.659007	0	0.012103285	0	0
cov1_5_9_11	23.4486	-641.404	0.6594923	0	0.046553445	0	0
cov1_5_10_11	23.457	-641.408	0.6603033	0	0.046241127	0	0
cov1_2_3_11	23.4735	-641.417	0.6505545	0	0.022579608	0	0.12474885
cov1_5_9_10	23.4952	-641.428	0.658849	0	0.026796201	0	0
cov1_9_10_11	23.4978	-641.429	0.6606346	0	0.080880605	0	0
cov5_9_10_11	23.5001	-641.43	0.6584196	0	0	0	0
cov2_3_4_11	23.5022	-641.431	0.6492295	0	0	0	0.1350511
cov1_2_3_4	23.504	-641.432	0.6495864	0	0.011294763	0	0.12349209
cov2_4_5_11	23.6546	-641.507	0.6485315	0	0	0	0.092378905
cov1_3_5_11	23.6864	-641.523	0.6568003	0	0.23525632	0	0
cov3_5_6_11	23.835	-641.597	0.6545279	0	0	0	0
cov3_4_5_11	23.8515	-641.606	0.6452312	0	0	0	0
cov3_4_6_11	23.8975	-641.629	0.6539423	0	0	0	0
cov1_3_4_6	23.9404	-641.65	0.6528754	0	-0.010417303	0	0
cov2_5_6_11	24.0119	-641.686	0.6545465	0	0	0	0.11841341
cov2_4_6_11	24.0896	-641.725	0.6544999	0	0	0	0.070997257
cov1_2_5_6	24.1844	-641.772	0.6531792	0	-0.004933752	0	0.11187145
cov1_2_4_6	24.1993	-641.78	0.6512198	0	-0.06784019	0	0.077330932
cov1_3_6_11	24.2387	-641.799	0.6553228	0	0.03406156	0	0
cov1_2_6_11	24.2508	-641.805	0.6537588	0	-0.03659019	0	0.11359075
cov1_3_4_11	24.2892	-641.825	0.6535853	0	0.078855395	0	0
cov1_2_5_11	24.3064	-641.833	0.6548993	0	0.085600985	0	0.12103341
cov1_2_4_11	24.3301	-641.845	0.6528937	0	0.02171127	0	0.086431021
cov1_4_6_11	24.4124	-641.886	0.6551943	0	-0.009593802	0	0
cov1_5_6_11	25.0419	-642.201	0.6594502	0	0.09250265	0	0

cov4_5_7_8_9	26.498	-639.324	0.6536318	0	0	0	0
cov4_5_7_8_10	26.5102	-639.33	0.6545519	0	0	0	0
cov4_5_6_7_9	26.5377	-639.344	0.6544491	0	0	0	0
cov4_5_6_7_10	26.5474	-639.349	0.6552771	0	0	0	0
cov2_4_5_7_9	26.5532	-639.352	0.6534289	0	0	0	0.034723173
cov2_4_5_7_10	26.5672	-639.359	0.6542825	0	0	0	0.034376582
cov1_4_5_7_9	26.5747	-639.363	0.6546387	0	0.040004645	0	0
cov1_4_5_7_10	26.5868	-639.369	0.6554592	0	0.040047589	0	0
cov4_5_7_9_11	26.5923	-639.371	0.6530597	0	0	0	0
cov4_5_7_10_11	26.603	-639.377	0.6539537	0	0	0	0
cov4_5_6_8_9	26.6103	-639.38	0.6544081	0	0	0	0
cov4_5_6_8_10	26.6202	-639.385	0.6552283	0	0	0	0
cov2_4_5_8_9	26.6255	-639.388	0.653391	0	0	0	0.034237585
cov4_5_7_9_10	26.626	-639.388	0.6539536	0	0	0	0
cov2_4_5_8_10	26.6395	-639.395	0.6542444	0	0	0	0.033891306
cov3_4_5_7_9	26.6412	-639.396	0.6535938	0	0	0	0
cov1_4_5_8_9	26.6437	-639.397	0.6546058	0	0.04028251	0	0
cov3_4_5_7_10	26.6534	-639.402	0.6544802	0	0	0	0
cov1_4_5_8_10	26.6558	-639.403	0.6554263	0	0.040321096	0	0
cov4_5_8_9_11	26.6655	-639.408	0.6530377	0	0	0	0
cov4_5_8_10_11	26.6762	-639.413	0.6537801	0	0	0	0
cov4_5_8_9_10	26.6959	-639.423	0.6539174	0	0	0	0
cov3_4_5_8_9	26.7112	-639.431	0.6534273	0	0	0	0
cov3_4_5_8_10	26.7229	-639.437	0.6543231	0	0	0	0
cov1_2_4_7_9	26.7378	-639.444	0.6528354	0	-0.053183903	0	0.056249647
cov1_2_4_7_10	26.7443	-639.447	0.6538051	0	-0.051355542	0	0.054905529
cov1_4_7_8_10	26.7667	-639.459	0.6551549	0	-0.026517639	0	0
cov1_4_7_8_9	26.7685	-639.46	0.6541839	0	-0.027643881	0	0
cov2_4_7_8_10	26.7913	-639.471	0.6565685	0	0	0	0.024392195
cov2_4_7_8_9	26.7969	-639.474	0.6557646	0	0	0	0.024815644
cov1_2_4_8_9	26.8154	-639.483	0.6528083	0	-0.05315481	0	0.055675872
cov4_7_8_9_10	26.8183	-639.484	0.656208	0	0	0	0
cov1_2_4_8_10	26.8218	-639.486	0.6537724	0	-0.051341143	0	0.054341214
cov4_6_7_8_10	26.8323	-639.491	0.6563418	0	0	0	0
cov4_7_8_10_11	26.8339	-639.492	0.6565798	0	0	0	0
cov3_4_7_8_10	26.8342	-639.492	0.656682	0	0	0	0
cov4_6_7_8_9	26.8385	-639.495	0.6554481	0	0	0	0
cov4_7_8_9_11	26.8405	-639.496	0.6557314	0	0	0	0
cov3_4_7_8_9	26.8407	-639.496	0.6558818	0	0	0	0
cov1_4_6_7_10	26.8594	-639.505	0.6550582	0	-0.054249455	0	0
cov1_4_6_7_9	26.8631	-639.507	0.6540989	0	-0.054852507	0	0
cov1_4_7_9_10	26.8854	-639.518	0.6545757	0	-0.027684649	0	0
cov1_3_4_7_9	26.8883	-639.519	0.654439	0	-0.032624416	0	0
cov1_4_7_10_11	26.8883	-639.519	0.6544063	0	-0.036714618	0	0
cov1_3_4_7_10	26.8904	-639.52	0.6553565	0	-0.030993766	0	0
cov1_4_7_9_11	26.8913	-639.521	0.6534523	0	-0.037572258	0	0
cov2_4_6_7_10	26.9036	-639.527	0.6557528	0	0	0	0.033429598

cov2_4_6_7_9	26.9052	-639.528	0.6548788	0	0	0	0.034464176
cov2_4_7_9_10	26.9165	-639.534	0.6561248	0	0	0	0.024018411
cov2_4_7_10_11	26.9182	-639.534	0.6565417	0	0	0	0.029451782
cov2_4_7_9_11	26.9216	-639.536	0.6557423	0	0	0	0.030247998
cov2_3_4_7_10	26.9299	-639.54	0.6562896	0	0	0	0.02663153
cov2_3_4_7_9	26.9364	-639.543	0.6555194	0	0	0	0.02661118
cov1_4_6_8_10	26.9367	-639.544	0.6550095	0	-0.05354937	0	0
cov1_4_6_8_9	26.9404	-639.545	0.6540671	0	-0.054132632	0	0
cov4_6_7_9_10	26.9538	-639.552	0.6557802	0	0	0	0
cov4_7_9_10_11	26.9575	-639.554	0.6561171	0	0	0	0
cov3_4_7_9_10	26.9576	-639.554	0.6562643	0	0	0	0
cov1_4_8_9_10	26.9594	-639.555	0.6545384	0	-0.027914731	0	0
cov1_4_8_10_11	26.9648	-639.558	0.6544076	0	-0.03627909	0	0
cov1_4_8_9_11	26.9678	-639.559	0.6534547	0	-0.037136082	0	0
cov3_4_6_7_10	26.968	-639.559	0.6563736	0	0	0	0
cov1_3_4_8_9	26.971	-639.561	0.6543141	0	-0.031739734	0	0
cov1_3_4_8_10	26.9721	-639.561	0.6552403	0	-0.030110239	0	0
cov3_4_6_7_9	26.9721	-639.561	0.6555218	0	0	0	0
cov3_4_7_10_11	26.9722	-639.561	0.6566817	0	0	0	0
cov4_6_7_10_11	26.9722	-639.561	0.6562073	0	0	0	0
cov3_4_7_9_11	26.9775	-639.564	0.6558956	0	0	0	0
cov4_6_7_9_11	26.9783	-639.564	0.655313	0	0	0	0
cov2_4_6_8_10	26.9791	-639.565	0.6557012	0	0	0	0.033143416
cov2_4_6_8_9	26.9807	-639.566	0.6548172	0	0	0	0.034181921
cov2_4_8_10_11	26.9936	-639.572	0.6565636	0	0	0	0.02921931
cov2_4_8_9_10	26.9937	-639.572	0.6560975	0	0	0	0.0234615
cov2_4_8_9_11	26.997	-639.574	0.6557152	0	0	0	0.030021071
cov2_3_4_8_10	27.0033	-639.577	0.6561327	0	0	0	0.027682033
cov1_3_4_5_9	27.0049	-639.578	0.6533068	0	0.11615227	0	0
cov1_3_4_5_10	27.0078	-639.579	0.6541307	0	0.11622948	0	0
cov2_3_4_8_9	27.0105	-639.581	0.6553574	0	0	0	0.027670958
cov4_6_8_9_10	27.028	-639.589	0.6557332	0	0	0	0
cov4_8_9_10_11	27.0319	-639.591	0.6560922	0	0	0	0
cov3_4_8_9_10	27.0345	-639.593	0.6561162	0	0	0	0
cov3_4_6_8_10	27.0454	-639.598	0.6562377	0	0	0	0
cov4_6_8_10_11	27.0465	-639.599	0.6561669	0	0	0	0
cov3_4_8_10_11	27.049	-639.6	0.6565352	0	0	0	0
cov3_4_6_8_9	27.0503	-639.6	0.6553729	0	0	0	0
cov4_6_8_9_11	27.0524	-639.602	0.6552841	0	0	0	0
cov3_4_8_9_11	27.055	-639.603	0.6557486	0	0	0	0
cov2_3_7_8_9	27.1051	-639.628	0.654763	0	0	0	0.087075126
cov2_3_7_8_10	27.1072	-639.629	0.6554896	0	0	0	0.087891129
cov3_4_5_6_10	27.1168	-639.634	0.6531951	0	0	0	0
cov1_2_7_8_9	27.1174	-639.634	0.654131	0	-0.032413435	0	0.1107586
cov3_4_5_6_9	27.1184	-639.635	0.6523031	0	0	0	0
cov1_2_7_8_10	27.1346	-639.643	0.6549493	0	-0.030644819	0	0.11016818
cov1_2_3_7_9	27.1769	-639.664	0.6530953	0	-0.031573038	0	0.11190771

cov1_2_6_7_9	27.186	-639.668	0.6538955	0	-0.06613916	0	0.10801936
cov2_7_8_9_10	27.1869	-639.669	0.6561426	0	0	0	0.085121489
cov1_2_3_7_10	27.1876	-639.669	0.6539334	0	-0.029637608	0	0.11139001
cov2_5_7_8_9	27.1922	-639.671	0.6556369	0	0	0	0.089930966
cov2_7_8_9_11	27.1942	-639.672	0.655852	0	0	0	0.087720802
cov2_6_7_8_9	27.1963	-639.673	0.6557551	0	0	0	0.086288424
cov1_2_6_7_10	27.2003	-639.675	0.6546846	0	-0.065010787	0	0.10748305
cov2_5_7_8_10	27.203	-639.677	0.6563689	0	0	0	0.089565167
cov2_7_8_10_11	27.2039	-639.677	0.6565392	0	0	0	0.088021673
cov2_6_7_8_10	27.2054	-639.678	0.6565037	0	0	0	0.086079355
cov1_2_5_7_9	27.208	-639.679	0.653418	0	-0.061931543	0	0.10901045
cov2_3_5_7_9	27.2188	-639.685	0.6539423	0	0	0	0.10173514
cov1_2_5_7_10	27.2209	-639.686	0.6542814	0	-0.061330729	0	0.10836436
cov2_3_5_7_10	27.2264	-639.688	0.6547122	0	0	0	0.10143349
cov2_3_4_5_9	27.2359	-639.693	0.6501132	0	0	0	0.064795813
cov2_3_7_9_10	27.2365	-639.694	0.6550561	0	0	0	0.087195232
cov2_3_4_5_10	27.2425	-639.697	0.6510762	0	0	0	0.064464583
cov1_2_3_8_9	27.2441	-639.697	0.6529873	0	-0.03128109	0	0.11193467
cov1_2_7_9_11	27.245	-639.698	0.653529	0	-0.040171341	0	0.10976323
cov2_3_7_9_11	27.2499	-639.7	0.6547282	0	0	0	0.089127994
cov1_2_7_9_10	27.2502	-639.7	0.6543409	0	-0.032007478	0	0.11045702
cov2_3_6_7_9	27.2512	-639.701	0.6546268	0	0	0	0.088293861
cov2_3_7_10_11	27.2529	-639.702	0.6554393	0	0	0	0.089457356
cov2_3_6_7_10	27.2537	-639.702	0.6553947	0	0	0	0.088141382
cov1_2_3_8_10	27.2541	-639.702	0.6538197	0	-0.029338559	0	0.1114124
cov1_2_7_10_11	27.2612	-639.706	0.6543402	0	-0.038569122	0	0.10923351
cov1_2_6_8_9	27.2769	-639.714	0.653878	0	-0.064899092	0	0.10798566
cov2_3_5_8_9	27.2807	-639.716	0.6537663	0	0	0	0.10286651
cov2_3_5_8_10	27.2879	-639.719	0.6545416	0	0	0	0.10255574
cov1_2_6_8_10	27.2912	-639.721	0.6546653	0	-0.063771995	0	0.10745087
cov1_2_5_8_9	27.2949	-639.723	0.6533972	0	-0.061662104	0	0.10890619
cov2_3_8_9_10	27.3018	-639.726	0.6549253	0	0	0	0.087444988
cov1_2_5_8_10	27.3077	-639.729	0.6542592	0	-0.061063169	0	0.10826078
cov2_3_8_9_11	27.3153	-639.733	0.6546077	0	0	0	0.089691761
cov2_3_6_8_9	27.317	-639.734	0.6544897	0	0	0	0.08867512
cov2_3_8_10_11	27.3177	-639.734	0.655305	0	0	0	0.090018745
cov2_3_6_8_10	27.3188	-639.735	0.6552562	0	0	0	0.088521327
cov2_5_7_9_10	27.3237	-639.737	0.6558703	0	0	0	0.089324291
cov2_7_9_10_11	27.3249	-639.738	0.6560613	0	0	0	0.087536621
cov2_6_7_9_10	27.3263	-639.738	0.6554884	0	0	0	0.086977621
cov2_5_7_9_11	27.3317	-639.741	0.6555803	0	0	0	0.090549898
cov2_5_6_7_9	27.332	-639.741	0.6555755	0	0	0	0.089353235
cov1_2_8_9_11	27.3338	-639.742	0.65356	0	-0.039313076	0	0.10972604
cov2_6_7_9_11	27.3342	-639.742	0.6557175	0	0	0	0.08798453
cov1_2_8_9_10	27.3369	-639.744	0.6543203	0	-0.031788057	0	0.11034878
cov2_5_6_7_10	27.3424	-639.747	0.6563165	0	0	0	0.088788662
cov2_5_7_10_11	27.3424	-639.747	0.6562876	0	0	0	0.090073289

cov2_6_7_10_11	27.3438	-639.747	0.6564508	0	0	0	0.087703681
cov1_2_8_10_11	27.35	-639.75	0.6543566	0	-0.037687071	0	0.10919949
cov2_5_8_9_10	27.4095	-639.78	0.6558331	0	0	0	0.089305587
cov2_8_9_10_11	27.4099	-639.78	0.6560468	0	0	0	0.087984785
cov2_6_8_9_10	27.4118	-639.781	0.6555814	0	0	0	0.087329454
cov2_5_8_9_11	27.417	-639.784	0.6555637	0	0	0	0.090751193
cov2_5_6_8_9	27.418	-639.784	0.6555373	0	0	0	0.089538699
cov2_6_8_9_11	27.4192	-639.785	0.6556832	0	0	0	0.088469998
cov2_5_8_10_11	27.4278	-639.789	0.6562881	0	0	0	0.090270693
cov2_5_6_8_10	27.4284	-639.79	0.6562655	0	0	0	0.088975002
cov2_6_8_10_11	27.4289	-639.79	0.656423	0	0	0	0.088180881
cov3_4_5_9_11	27.4391	-639.795	0.6498694	0	0	0	0
cov3_4_5_10_11	27.4397	-639.795	0.6508559	0	0	0	0
cov3_4_5_9_10	27.4749	-639.813	0.6512232	0	0	0	0
cov1_2_3_4_10	27.7498	-639.95	0.6517407	0	-0.039295185	0	0.07226026
cov2_3_5_6_10	27.7557	-639.953	0.6528897	0	0	0	0.11054635
cov2_3_5_6_9	27.7579	-639.954	0.6520796	0	0	0	0.11121735
cov1_2_3_4_9	27.7639	-639.957	0.6506886	0	-0.041287837	0	0.073598915
cov2_3_4_10_11	27.8146	-639.983	0.6539304	0	0	0	0.057975644
cov2_3_4_9_11	27.8336	-639.992	0.6530379	0	0	0	0.058750634
cov1_4_5_6_7	27.8398	-639.995	0.6549487	0	0.093447739	0	0
cov2_3_4_9_10	27.8429	-639.997	0.6532754	0	0	0	0.049952567
cov2_3_5_9_10	27.8482	-639.999	0.651689	0	0	0	0.12205735
cov1_2_3_5_9	27.852	-640.001	0.6519076	0	0.024163526	0	0.11626096
cov1_2_3_5_10	27.8523	-640.001	0.6527728	0	0.024665108	0	0.11559388
cov2_3_4_6_10	27.8544	-640.002	0.6533411	0	0	0	0.053522433
cov1_4_5_7_8	27.8666	-640.009	0.6549464	0	0.15099452	0	0
cov2_3_5_9_11	27.8688	-640.01	0.6511401	0	0	0	0.12230434
cov2_3_5_10_11	27.8695	-640.01	0.6520005	0	0	0	0.12162356
cov2_3_4_6_9	27.8772	-640.014	0.6523684	0	0	0	0.05451907
cov3_6_7_8_10	27.88	-640.015	0.6583212	0	0	0	0
cov3_6_7_8_9	27.8801	-640.015	0.6575661	0	0	0	0
cov1_2_3_6_10	27.8853	-640.018	0.6519588	0	-0.061667412	0	0.10898515
cov2_4_5_6_7	27.8881	-640.019	0.6527051	0	0	0	0.055951478
cov5_6_7_8_9	27.8915	-640.021	0.6582568	0	0	0	0
cov5_6_7_8_10	27.8924	-640.021	0.6590452	0	0	0	0
cov1_2_3_6_9	27.8957	-640.023	0.6509993	0	-0.063086035	0	0.1096514
cov1_4_5_6_8	27.9135	-640.032	0.654902	0	0.094734315	0	0
cov6_7_8_9_11	27.9255	-640.038	0.658032	0	0	0	0
cov4_5_6_7_8	27.9273	-640.039	0.6540109	0	0	0	0
cov6_7_8_10_11	27.9306	-640.041	0.6587776	0	0	0	0
cov6_7_8_9_10	27.9388	-640.045	0.6588738	0	0	0	0
cov1_6_7_8_9	27.9464	-640.048	0.6586484	0	0.009784249	0	0
cov1_3_4_6_10	27.9474	-640.049	0.6533966	0	-0.042560511	0	0
cov1_2_3_9_10	27.9513	-640.051	0.6516472	0	-0.025829117	0	0.11216346
cov1_6_7_8_10	27.9522	-640.051	0.6594041	0	0.010531697	0	0
cov1_2_4_5_7	27.9579	-640.054	0.6539461	0	0.12309856	0	0.03188168

cov5_7_8_9_11	27.9683	-640.059	0.6569475	0	0	0	0
cov5_7_8_10_11	27.9692	-640.06	0.6577946	0	0	0	0
cov2_4_5_6_8	27.9707	-640.061	0.6526397	0	0	0	0.05572323
cov1_2_3_10_11	27.9712	-640.061	0.652274	0	-0.021978394	0	0.11224064
cov1_3_4_6_9	27.9738	-640.062	0.6523674	0	-0.043405217	0	0
cov1_2_3_9_11	27.9782	-640.064	0.6513352	0	-0.023617304	0	0.11293312
cov2_3_9_10_11	27.9799	-640.065	0.6531312	0	0	0	0.099429399
cov2_3_6_10_11	27.9858	-640.068	0.6540138	0	0	0	0.093387262
cov1_4_5_7_11	27.9921	-640.071	0.6543933	0	0.14311227	0	0
cov1_3_4_9_10	27.9931	-640.072	0.6529656	0	-0.011688036	0	0
cov1_3_4_5_7	27.9933	-640.072	0.6546843	0	0.16089187	0	0
cov3_4_6_10_11	27.9945	-640.073	0.6547171	0	0	0	0
cov5_7_8_9_10	27.9954	-640.073	0.6578279	0	0	0	0
cov3_4_9_10_11	27.9963	-640.073	0.6536508	0	0	0	0
cov3_4_6_9_10	27.9988	-640.075	0.653789	0	0	0	0
cov2_3_6_9_11	27.9992	-640.075	0.6531482	0	0	0	0.093787277
cov2_3_6_9_10	28.0002	-640.075	0.6530552	0	0	0	0.089338306
cov1_5_7_8_9	28.0008	-640.076	0.6579553	0	0.019499102	0	0
cov1_5_7_8_10	28.0032	-640.077	0.6587852	0	0.019232173	0	0
cov3_6_7_10_11	28.0102	-640.08	0.6576456	0	0	0	0
cov3_6_7_9_11	28.0115	-640.081	0.6569097	0	0	0	0
cov3_5_6_7_10	28.0121	-640.081	0.658314	0	0	0	0
cov1_3_4_10_11	28.0129	-640.082	0.6537355	0	-0.007432121	0	0
cov3_5_6_7_9	28.0143	-640.082	0.6575434	0	0	0	0
cov3_5_7_8_9	28.0161	-640.083	0.6572451	0	0	0	0
cov1_3_7_8_9	28.0165	-640.084	0.6579579	0	0.060992887	0	0
cov3_5_7_8_10	28.0175	-640.084	0.6580932	0	0	0	0
cov1_3_7_8_10	28.0188	-640.085	0.6587437	0	0.062470322	0	0
cov3_4_6_9_11	28.0212	-640.086	0.6537459	0	0	0	0
cov5_6_7_9_10	28.0237	-640.087	0.6585216	0	0	0	0
cov3_6_7_9_10	28.025	-640.088	0.657851	0	0	0	0
cov1_5_6_7_10	28.027	-640.089	0.6584874	0	-0.023717487	0	0
cov1_5_6_7_9	28.0271	-640.089	0.6576862	0	-0.022952781	0	0
cov1_2_4_5_8	28.0287	-640.09	0.6539351	0	0.12419764	0	0.030884689
cov5_6_7_9_11	28.0288	-640.09	0.6578114	0	0	0	0
cov5_6_7_10_11	28.0292	-640.09	0.6585374	0	0	0	0
cov1_3_6_7_10	28.0335	-640.092	0.6583951	0	0.013916593	0	0
cov1_3_6_7_9	28.0341	-640.092	0.6576366	0	0.013075987	0	0
cov1_3_4_9_11	28.0366	-640.094	0.6525826	0	-0.008633386	0	0
cov4_5_6_7_11	28.0423	-640.096	0.6530745	0	0	0	0
cov1_7_8_9_11	28.0497	-640.1	0.6581175	0	0.045421108	0	0
cov1_3_4_5_8	28.0531	-640.102	0.6545852	0	0.16508655	0	0
cov1_7_8_10_11	28.0575	-640.104	0.6588896	0	0.046552228	0	0
cov1_4_5_8_11	28.0618	-640.106	0.654386	0	0.14401347	0	0
cov6_7_9_10_11	28.0649	-640.108	0.6582176	0	0	0	0
cov3_4_5_6_7	28.0714	-640.111	0.6541511	0	0	0	0
cov1_7_8_9_10	28.0715	-640.111	0.659255	0	0.059576373	0	0

cov1_6_7_9_11	28.077	-640.114	0.6579345	0	0.002910942	0	0
cov1_6_7_10_11	28.082	-640.116	0.6586802	0	0.003501277	0	0
cov3_6_8_10_11	28.084	-640.117	0.6575106	0	0	0	0
cov3_6_8_9_11	28.0861	-640.118	0.6567757	0	0	0	0
cov1_6_7_9_10	28.087	-640.119	0.6589374	0	0.012259589	0	0
cov5_7_9_10_11	28.0877	-640.119	0.6573008	0	0	0	0
cov3_5_6_8_10	28.0878	-640.119	0.6581568	0	0	0	0
cov3_5_6_8_9	28.0907	-640.121	0.6573758	0	0	0	0
cov3_6_8_9_10	28.0972	-640.124	0.6577046	0	0	0	0
cov3_5_7_10_11	28.1016	-640.126	0.6572648	0	0	0	0
cov3_5_7_9_11	28.1023	-640.126	0.6564541	0	0	0	0
cov1_3_6_8_10	28.1042	-640.127	0.6582621	0	0.014994981	0	0
cov1_3_6_8_9	28.1057	-640.128	0.657497	0	0.014158653	0	0
cov1_5_7_9_11	28.1069	-640.129	0.6570875	0	0.005537966	0	0
cov1_5_7_10_11	28.1078	-640.129	0.6580284	0	0.005048467	0	0
cov5_6_8_9_10	28.1125	-640.132	0.6584753	0	0	0	0
cov1_5_6_8_10	28.1173	-640.134	0.6584627	0	-0.022514439	0	0
cov1_5_6_8_9	28.1174	-640.134	0.6576624	0	-0.021751264	0	0
cov5_6_8_9_11	28.1191	-640.135	0.6577695	0	0	0	0
cov5_6_8_10_11	28.1196	-640.135	0.6585505	0	0	0	0
cov1_5_7_9_10	28.1221	-640.136	0.6582845	0	0.018011873	0	0
cov4_5_6_8_11	28.126	-640.138	0.6530418	0	0	0	0
cov1_3_7_9_11	28.1268	-640.139	0.6570567	0	0.046626378	0	0
cov1_3_7_10_11	28.1279	-640.139	0.6578248	0	0.04781943	0	0
cov1_3_5_7_9	28.1307	-640.141	0.6576207	0	0.0299953	0	0
cov1_3_5_7_10	28.1312	-640.141	0.6584319	0	0.030514488	0	0
cov3_5_7_9_10	28.1351	-640.143	0.6576173	0	0	0	0
cov1_3_7_9_10	28.1458	-640.148	0.6582673	0	0.061332394	0	0
cov6_8_9_10_11	28.1583	-640.154	0.6582106	0	0	0	0
cov3_4_5_6_8	28.1598	-640.155	0.6540032	0	0	0	0
cov1_6_8_9_11	28.1699	-640.16	0.657948	0	0.004701276	0	0
cov1_6_8_10_11	28.175	-640.163	0.658694	0	0.005292422	0	0
cov5_8_9_10_11	28.1764	-640.164	0.6572822	0	0	0	0
cov1_6_8_9_10	28.1772	-640.164	0.6588939	0	0.013538137	0	0
cov1_7_9_10_11	28.1773	-640.164	0.6583623	0	0.045464988	0	0
cov3_5_8_10_11	28.184	-640.167	0.6570892	0	0	0	0
cov3_5_8_9_11	28.1853	-640.168	0.6562726	0	0	0	0
cov3_7_8_9_11	28.1917	-640.171	0.6546834	0	0	0	0
cov1_5_8_9_11	28.1953	-640.173	0.6570636	0	0.006435598	0	0
cov1_5_8_10_11	28.1963	-640.173	0.65791	0	0.005843469	0	0
cov1_3_8_9_11	28.1976	-640.174	0.6569517	0	0.047574833	0	0
cov1_3_8_10_11	28.1979	-640.174	0.6577243	0	0.048766179	0	0
cov3_7_8_10_11	28.2045	-640.178	0.6554258	0	0	0	0
cov1_3_5_8_10	28.2059	-640.178	0.6583217	0	0.034759603	0	0
cov1_3_5_8_9	28.206	-640.178	0.6575264	0	0.034215617	0	0
cov1_5_8_9_10	28.2072	-640.179	0.6582202	0	0.018134157	0	0
cov1_3_8_9_10	28.2127	-640.182	0.6581378	0	0.061655347	0	0

cov3_5_8_9_10	28.2177	-640.184	0.6574438	0	0	0	0
cov7_8_9_10_11	28.2377	-640.194	0.6560174	0	0	0	0
cov4_5_6_9_11	28.2481	-640.199	0.6552056	0	0	0	0
cov1_4_5_9_11	28.2563	-640.203	0.6558963	0	0.069188132	0	0
cov4_5_6_10_11	28.264	-640.207	0.6560466	0	0	0	0
cov1_8_9_10_11	28.2655	-640.208	0.6583801	0	0.046305093	0	0
cov1_4_5_10_11	28.2755	-640.213	0.6567028	0	0.069313638	0	0
cov1_4_5_6_9	28.2802	-640.215	0.6548631	0	0.026530377	0	0
cov4_5_6_9_10	28.2862	-640.218	0.6548329	0	0	0	0
cov2_4_5_7_8	28.2944	-640.222	0.6499746	0	0	0	0.10762558
cov1_4_5_6_10	28.2962	-640.223	0.6556657	0	0.026087701	0	0
cov2_4_5_6_9	28.2964	-640.224	0.6545677	0	0	0	-0.004601991
cov1_2_4_5_9	28.3044	-640.228	0.6553482	0	0.066258582	0	-0.017606362
cov1_4_5_9_10	28.3083	-640.229	0.6551596	0	0.050398808	0	0
cov2_4_5_6_10	28.3113	-640.231	0.6554144	0	0	0	-0.005298712
cov2_4_5_7_11	28.3197	-640.235	0.6491226	0	0	0	0.092297566
cov3_7_9_10_11	28.3212	-640.236	0.6549451	0	0	0	0
cov1_2_4_5_10	28.3213	-640.236	0.6561758	0	0.067042146	0	-0.018291313
cov1_3_4_5_6	28.3516	-640.251	0.6537252	0	0.16889726	0	0
cov2_3_4_5_7	28.3742	-640.262	0.6508903	0	0	0	0.092841067
cov2_5_6_7_8	28.3876	-640.269	0.6537826	0	0	0	0.11205662
cov2_4_5_9_11	28.3981	-640.274	0.653757	0	0	0	0.019137651
cov3_8_9_10_11	28.4002	-640.275	0.6547873	0	0	0	0
cov2_4_5_9_10	28.4024	-640.277	0.6538415	0	0	0	0.012612034
cov2_4_5_8_11	28.4029	-640.277	0.6490752	0	0	0	0.09222871
cov4_5_9_10_11	28.4072	-640.279	0.6537012	0	0	0	0
cov2_4_5_10_11	28.4189	-640.285	0.6546246	0	0	0	0.018567869
cov2_3_4_5_8	28.4666	-640.309	0.6506939	0	0	0	0.094513716
cov2_3_5_6_7	28.4959	-640.323	0.6529638	0	0	0	0.11780728
cov1_2_5_6_7	28.5223	-640.336	0.6531674	0	-0.020087687	0	0.11694667
cov1_2_3_4_5	28.5233	-640.337	0.6528394	0	0.21155619	0	0.025808876
cov2_5_6_7_11	28.5318	-640.341	0.6535995	0	0	0	0.11225051
cov1_2_6_7_8	28.5441	-640.347	0.6522725	0	-0.066507578	0	0.11536142
cov1_3_4_5_11	28.552	-640.351	0.6536387	0	0.23214684	0	0
cov2_3_5_6_8	28.5746	-640.363	0.6527845	0	0	0	0.1189955
cov1_2_5_7_8	28.6074	-640.379	0.6533359	0	0.041327506	0	0.12222
cov1_2_5_6_8	28.6169	-640.384	0.6531329	0	-0.01870886	0	0.11692865
cov2_5_6_8_11	28.6254	-640.388	0.6535574	0	0	0	0.11265758
cov1_2_4_6_7	28.6324	-640.391	0.6518857	0	-0.068284223	0	0.099368281
cov2_3_4_7_8	28.6402	-640.395	0.6535806	0	0	0	0.079539588
cov2_5_7_8_11	28.6431	-640.397	0.6512943	0	0	0	0.13374737
cov2_4_6_7_8	28.6568	-640.404	0.6537113	0	0	0	0.079878217
cov1_2_3_6_7	28.671	-640.411	0.6525633	0	-0.06463787	0	0.11478736
cov2_3_5_7_8	28.6786	-640.415	0.6517366	0	0	0	0.13932912
cov2_3_6_7_8	28.6818	-640.416	0.6542508	0	0	0	0.093545033
cov1_2_6_7_11	28.6868	-640.419	0.6520007	0	-0.066794418	0	0.11528567
cov2_6_7_8_11	28.6886	-640.42	0.6541613	0	0	0	0.096286348

cov3_4_5_7_11	28.7108	-640.431	0.6509716	0	0	0	0
cov1_2_3_5_7	28.7208	-640.436	0.653005	0	0.054286283	0	0.12366365
cov2_3_4_6_7	28.7214	-640.436	0.6543788	0	0	0	0.067495756
cov1_2_4_6_8	28.7246	-640.438	0.6518423	0	-0.067155631	0	0.098946313
cov1_2_4_7_8	28.7276	-640.439	0.6519601	0	-0.007984386	0	0.10404778
cov1_2_5_7_11	28.7304	-640.44	0.6528196	0	0.034808623	0	0.12146516
cov2_4_7_8_11	28.7311	-640.441	0.6523643	0	0	0	0.096529138
cov1_3_5_6_10	28.7353	-640.443	0.6569413	0	0.056456328	0	0
cov1_2_3_4_7	28.7442	-640.447	0.6527434	0	-0.015824075	0	0.088229254
cov1_3_5_6_9	28.749	-640.45	0.6561439	0	0.057221721	0	0
cov2_3_4_7_11	28.7625	-640.457	0.6535437	0	0	0	0.078535758
cov2_3_7_8_11	28.7633	-640.457	0.6529579	0	0	0	0.1132113
cov1_2_3_7_8	28.7641	-640.457	0.6527458	0	-0.004762459	0	0.12001631
cov2_3_4_5_6	28.7703	-640.46	0.6503035	0	0	0	0.076560757
cov2_3_5_7_11	28.7729	-640.462	0.6511485	0	0	0	0.13403157
cov1_2_3_6_8	28.7738	-640.462	0.6524195	0	-0.06321186	0	0.11496418
cov1_2_7_8_11	28.7813	-640.466	0.6518661	0	-0.009123799	0	0.12028012
cov1_2_6_8_11	28.7828	-640.467	0.6519884	0	-0.064956907	0	0.11531141
cov2_4_6_7_11	28.7839	-640.467	0.6539044	0	0	0	0.082293817
cov3_5_6_9_10	28.7948	-640.473	0.6558084	0	0	0	0
cov1_2_3_5_8	28.7962	-640.473	0.6529063	0	0.058352409	0	0.12398626
cov1_3_6_9_10	28.7976	-640.474	0.6559285	0	0.01664051	0	0
cov3_5_6_10_11	28.8042	-640.477	0.6558566	0	0	0	0
cov1_2_4_9_11	28.8058	-640.478	0.6544077	0	-0.035604114	0	0.034289096
cov2_3_6_7_11	28.8081	-640.479	0.6543866	0	0	0	0.096147621
cov3_6_9_10_11	28.8088	-640.48	0.6555921	0	0	0	0
cov3_7_8_9_10	28.8096	-640.48	0.6553028	0	0	0	0
cov1_4_6_10_11	28.8105	-640.481	0.6561266	0	-0.049520861	0	0
cov1_2_4_10_11	28.8119	-640.481	0.6554048	0	-0.033884859	0	0.03274771
cov1_4_6_9_11	28.8123	-640.481	0.6551604	0	-0.050002618	0	0
cov1_3_6_10_11	28.8127	-640.482	0.6562894	0	0.017396946	0	0
cov1_2_5_8_11	28.8192	-640.485	0.6528257	0	0.035698631	0	0.12143966
cov3_5_6_9_11	28.8206	-640.486	0.6550123	0	0	0	0
cov3_4_5_8_11	28.8213	-640.486	0.6507632	0	0	0	0
cov2_3_4_6_8	28.8239	-640.487	0.654199	0	0	0	0.068963124
cov1_3_6_9_11	28.8334	-640.492	0.6554207	0	0.016822946	0	0
cov3_4_5_7_8	28.8342	-640.492	0.6527413	0	0	0	0
cov1_4_9_10_11	28.8417	-640.496	0.6555304	0	-0.021732129	0	0
cov1_2_4_6_9	28.8418	-640.496	0.6532183	0	-0.079834312	0	0.029815059
cov1_2_4_6_10	28.8434	-640.497	0.6542233	0	-0.078514459	0	0.028303922
cov4_5_7_8_11	28.8464	-640.499	0.6483511	0	0	0	0
cov1_2_4_7_11	28.8486	-640.5	0.6514636	0	-0.014006981	0	0.10328007
cov1_2_3_4_8	28.8492	-640.5	0.6526025	0	-0.014959454	0	0.089232054
cov2_4_9_10_11	28.8492	-640.5	0.6567384	0	0	0	0.018740866
cov3_4_6_7_8	28.8546	-640.503	0.6561514	0	0	0	0
cov1_2_4_9_10	28.8591	-640.505	0.6537404	0	-0.053779652	0	0.029389034
cov1_4_6_9_10	28.8616	-640.506	0.6544408	0	-0.066772403	0	0

cov2_3_5_8_11	28.8621	-640.506	0.6509483	0	0	0	0.13549981
cov2_3_4_8_11	28.8655	-640.508	0.6533627	0	0	0	0.080226052
cov4_6_9_10_11	28.8696	-640.51	0.656938	0	0	0	0
cov2_4_6_8_11	28.8698	-640.51	0.653797	0	0	0	0.082308791
cov2_4_6_10_11	28.8738	-640.512	0.6572314	0	0	0	0.01828856
cov2_4_6_9_11	28.8741	-640.512	0.6563085	0	0	0	0.019564103
cov1_3_5_9_10	28.8773	-640.514	0.6567448	0	0.10466124	0	0
cov1_2_3_7_11	28.8826	-640.517	0.6522753	0	-0.010936153	0	0.11914571
cov1_3_5_10_11	28.8912	-640.521	0.6568383	0	0.10248426	0	0
cov1_3_5_9_11	28.903	-640.527	0.6561521	0	0.10303565	0	0
cov2_3_6_8_11	28.9039	-640.527	0.6542588	0	0	0	0.096866086
cov1_3_4_6_7	28.9274	-640.539	0.6554637	0	-0.037958152	0	0
cov1_2_4_8_11	28.9349	-640.543	0.6514395	0	-0.013288661	0	0.10285948
cov1_3_9_10_11	28.9396	-640.545	0.6559631	0	0.064607225	0	0
cov1_2_3_8_11	28.9809	-640.566	0.6521838	0	-0.009764412	0	0.11931932
cov3_4_6_7_11	28.9815	-640.566	0.6561014	0	0	0	0
cov1_3_4_7_8	29.0283	-640.589	0.6555713	0	0.022342195	0	0
cov3_4_7_8_11	29.0284	-640.59	0.6543025	0	0	0	0
cov2_4_6_9_10	29.0303	-640.59	0.6554694	0	0	0	0.007293181
cov1_3_4_6_8	29.0479	-640.599	0.6553322	0	-0.03565342	0	0
cov1_4_6_7_8	29.0568	-640.604	0.6547176	0	-0.02378409	0	0
cov4_6_7_8_11	29.0787	-640.615	0.655055	0	0	0	0
cov3_4_5_6_11	29.0911	-640.621	0.651607	0	0	0	0
cov3_4_6_8_11	29.0957	-640.623	0.6559513	0	0	0	0
cov1_3_4_7_11	29.1314	-640.641	0.6549537	0	0.013470079	0	0
cov1_4_6_7_11	29.1955	-640.673	0.6542312	0	-0.026470824	0	0
cov3_5_9_10_11	29.2104	-640.68	0.6534091	0	0	0	0
cov1_3_4_8_11	29.2475	-640.699	0.6548695	0	0.015426328	0	0
cov1_2_3_5_6	29.2762	-640.713	0.6517207	0	0.065037765	0	0.12428834
cov1_4_6_8_11	29.2841	-640.717	0.6542166	0	-0.025005285	0	0
cov1_4_7_8_11	29.3375	-640.744	0.6539887	0	0.038819977	0	0
cov2_3_5_6_11	29.3734	-640.762	0.6505748	0	0	0	0.13768135
cov1_5_6_7_8	29.4461	-640.798	0.658327	0	0.059470051	0	0
cov5_6_7_8_11	29.508	-640.829	0.6567868	0	0	0	0
cov3_5_6_7_8	29.5428	-640.847	0.657634	0	0	0	0
cov3_6_7_8_11	29.5538	-640.852	0.6571896	0	0	0	0
cov1_2_3_5_11	29.5591	-640.855	0.6520007	0	0.13728256	0	0.13051599
cov1_3_6_7_8	29.5602	-640.855	0.6578334	0	0.016397449	0	0
cov1_6_7_8_11	29.5795	-640.865	0.6568425	0	0.011015968	0	0
cov1_5_6_7_11	29.5836	-640.867	0.6577462	0	0.054746228	0	0
cov1_4_5_6_11	29.5895	-640.87	0.6560088	0	0.12738328	0	0
cov1_3_5_6_7	29.591	-640.871	0.6580975	0	0.068301394	0	0
cov3_5_6_7_11	29.6582	-640.904	0.6568223	0	0	0	0
cov1_3_5_6_8	29.6775	-640.914	0.6579836	0	0.073672864	0	0
cov1_5_6_8_11	29.6798	-640.915	0.6577463	0	0.056677247	0	0
cov1_2_4_5_6	29.6819	-640.916	0.6550669	0	0.12163757	0	-0.014384769
cov1_3_6_7_11	29.6928	-640.922	0.6573076	0	0.011921187	0	0

cov1_2_6_9_11	29.6942	-640.922	0.6560182	0	-0.042626799	0	0.10513494
cov1_2_6_10_11	29.7132	-640.932	0.6568181	0	-0.04154413	0	0.10455886
cov2_6_9_10_11	29.7422	-640.946	0.6575378	0	0	0	0.09316385
cov2_5_6_9_11	29.7513	-640.951	0.65725	0	0	0	0.093316639
cov3_5_6_8_11	29.7632	-640.957	0.6566558	0	0	0	0
cov1_5_7_8_11	29.7637	-640.957	0.6577962	0	0.12923295	0	0
cov2_5_6_10_11	29.7673	-640.959	0.6579328	0	0	0	0.092696944
cov1_2_5_9_11	29.7678	-640.959	0.6556052	0	-0.027754603	0	0.10681789
cov1_2_4_5_11	29.771	-640.961	0.6558234	0	0.19245586	0	-0.008536698
cov2_5_9_10_11	29.7796	-640.965	0.6569463	0	0	0	0.099084946
cov1_2_5_10_11	29.787	-640.969	0.6564317	0	-0.02716631	0	0.10614148
cov1_3_5_7_8	29.7905	-640.971	0.6584728	0	0.14626554	0	0
cov1_2_9_10_11	29.7943	-640.972	0.6564122	0	-0.002842447	0	0.10794441
cov1_2_5_6_9	29.7969	-640.974	0.6541157	0	-0.083185684	0	0.10094275
cov1_3_6_8_11	29.8006	-640.976	0.6571947	0	0.014091479	0	0
cov1_2_5_6_10	29.811	-640.981	0.654946	0	-0.083139534	0	0.10026052
cov1_2_6_9_10	29.8162	-640.983	0.6549102	0	-0.06154677	0	0.10186456
cov2_3_4_5_11	29.8451	-640.998	0.6452559	0	0	0	0.13323289
cov1_2_5_9_10	29.8663	-641.008	0.6545868	0	-0.050135784	0	0.10336044
cov1_3_7_8_11	29.8757	-641.013	0.6573977	0	0.083611715	0	0
cov1_3_5_7_11	29.8933	-641.022	0.6576261	0	0.13217283	0	0
cov2_4_5_6_11	29.9358	-641.043	0.6532781	0	0	0	0.036172997
cov2_5_6_9_10	29.9416	-641.046	0.6563827	0	0	0	0.084919647
cov1_3_5_8_11	29.9775	-641.064	0.6575474	0	0.1371569	0	0
cov3_5_7_8_11	30.3239	-641.237	0.6548027	0	0	0	0
cov1_2_3_6_11	30.3487	-641.25	0.6508128	0	-0.048117149	0	0.11869965
cov1_2_3_4_6	30.3973	-641.274	0.6496359	0	-0.059732323	0	0.1192942
cov2_3_4_6_11	30.42	-641.285	0.6521917	0	0	0	0.10818312
cov1_3_5_6_11	30.4789	-641.315	0.6568919	0	0.14898243	0	0
cov5_6_9_10_11	30.4854	-641.318	0.6598061	0	0	0	0
cov1_5_6_9_11	30.5033	-641.327	0.6594001	0	-0.000160502	0	0
cov1_5_6_10_11	30.5087	-641.33	0.6601863	0	-0.000969223	0	0
cov1_6_9_10_11	30.5417	-641.346	0.660451	0	0.02938663	0	0
cov1_5_6_9_10	30.5714	-641.361	0.6586271	0	-0.018880499	0	0
cov1_5_9_10_11	30.6391	-641.395	0.6598863	0	0.044963555	0	0
cov1_2_3_4_11	30.6827	-641.417	0.6505514	0	0.022372685	0	0.12393715
cov1_3_4_6_11	31.1068	-641.629	0.6539604	0	0.000813382	0	0
cov1_2_5_6_11	31.215	-641.683	0.6549913	0	0.016441566	0	0.11516885
cov1_2_4_6_11	31.2348	-641.693	0.6532163	0	-0.045372624	0	0.081717638
cov4_5_6_7_8_9	33.595	-639.268	0.654596	0	0	0	0
cov4_5_6_7_8_10	33.6049	-639.273	0.6554227	0	0	0	0
cov2_4_5_7_8_9	33.6196	-639.28	0.6535436	0	0	0	0.034638512
cov2_4_5_7_8_10	33.6338	-639.288	0.6543884	0	0	0	0.034312543
cov1_4_5_7_8_9	33.6404	-639.291	0.6547417	0	0.040075775	0	0
cov1_4_5_7_8_10	33.6527	-639.297	0.6555675	0	0.040118703	0	0
cov4_5_7_8_9_11	33.6587	-639.3	0.6531685	0	0	0	0
cov4_5_7_8_10_11	33.6696	-639.305	0.6540483	0	0	0	0

cov4_5_7_8_9_10	33.6918	-639.317	0.6540609	0	0	0	0
cov3_4_5_7_8_9	33.7073	-639.324	0.653652	0	0	0	0
cov2_4_5_6_7_9	33.715	-639.328	0.654111	0	0	0	0.023007238
cov3_4_5_7_8_10	33.7196	-639.33	0.6545548	0	0	0	0
cov2_4_5_6_7_10	33.7265	-639.334	0.6549287	0	0	0	0.022388268
cov3_4_5_6_7_9	33.7324	-639.337	0.6540424	0	0	0	0
cov4_5_6_7_9_11	33.7337	-639.337	0.6540663	0	0	0	0
cov4_5_6_7_9_10	33.7352	-639.338	0.6548089	0	0	0	0
cov3_4_5_6_7_10	33.7401	-639.341	0.6548793	0	0	0	0
cov1_4_5_6_7_9	33.7423	-639.342	0.654658	0	0.013796291	0	0
cov4_5_6_7_10_11	33.7431	-639.342	0.6548951	0	0	0	0
cov2_4_5_7_9_11	33.7446	-639.343	0.6531299	0	0	0	0.029452016
cov2_3_4_5_7_9	33.749	-639.345	0.6529041	0	0	0	0.040869577
cov2_4_5_7_9_10	33.7512	-639.346	0.6538052	0	0	0	0.033947208
cov1_4_5_6_7_10	33.7525	-639.347	0.6554619	0	0.013220708	0	0
cov1_2_4_5_7_9	33.7545	-639.348	0.653945	0	0.017955821	0	0.026072477
cov2_4_5_7_10_11	33.7574	-639.349	0.6539693	0	0	0	0.028936193
cov1_3_4_5_7_9	33.7601	-639.351	0.6543192	0	0.056200194	0	0
cov2_3_4_5_7_10	33.7617	-639.352	0.6537519	0	0	0	0.040767487
cov1_2_4_5_7_10	33.768	-639.355	0.6547899	0	0.018569646	0	0.025447602
cov1_4_5_7_9_11	33.7697	-639.355	0.6541034	0	0.03086713	0	0
cov1_3_4_5_7_10	33.7698	-639.356	0.655129	0	0.056918256	0	0
cov1_4_5_7_9_10	33.7729	-639.357	0.6549729	0	0.038791549	0	0
cov1_4_5_7_10_11	33.7809	-639.361	0.6549111	0	0.030623534	0	0
cov4_5_7_9_10_11	33.7872	-639.364	0.6534766	0	0	0	0
cov2_4_5_6_8_9	33.7886	-639.365	0.6540652	0	0	0	0.0226632
cov3_4_5_6_8_9	33.7958	-639.369	0.653936	0	0	0	0
cov3_4_5_7_9_11	33.7998	-639.371	0.6528464	0	0	0	0
cov2_4_5_6_8_10	33.8002	-639.371	0.6548845	0	0	0	0.022042287
cov3_4_5_6_8_10	33.803	-639.372	0.6547491	0	0	0	0
cov1_2_4_7_8_9	33.8058	-639.374	0.6529369	0	-0.053239406	0	0.056207541
cov4_5_6_8_9_10	33.8078	-639.375	0.6547823	0	0	0	0
cov4_5_6_8_9_11	33.8078	-639.375	0.6540277	0	0	0	0
cov3_4_5_7_10_11	33.8097	-639.375	0.6536785	0	0	0	0
cov1_2_4_7_8_10	33.8125	-639.377	0.653904	0	-0.051418706	0	0.054872762
cov2_3_4_5_8_9	33.8127	-639.377	0.652728	0	0	0	0.042083592
cov1_4_5_6_8_9	33.8141	-639.378	0.654627	0	0.015008765	0	0
cov1_3_4_5_8_9	33.8158	-639.379	0.6542202	0	0.060365378	0	0
cov4_5_6_8_10_11	33.8173	-639.379	0.654855	0	0	0	0
cov2_4_5_8_9_11	33.8187	-639.38	0.6531221	0	0	0	0.029227405
cov2_4_5_8_9_10	33.8235	-639.382	0.6537683	0	0	0	0.033459227
cov1_4_5_6_8_10	33.8244	-639.383	0.6554304	0	0.014436246	0	0
cov1_3_4_5_8_10	33.825	-639.383	0.655033	0	0.061080561	0	0
cov2_3_4_5_8_10	33.8252	-639.383	0.6535807	0	0	0	0.041973195
cov1_2_4_5_8_9	33.8258	-639.384	0.6539395	0	0.01912819	0	0.025023023
cov2_4_5_8_10_11	33.8315	-639.386	0.6539291	0	0	0	0.028699638
cov3_4_5_7_9_10	33.8353	-639.388	0.6539841	0	0	0	0

cov1_2_4_5_8_10	33.8392	-639.39	0.6547812	0	0.019746074	0	0.024401003
cov1_4_5_8_9_11	33.8407	-639.391	0.6541638	0	0.031850796	0	0
cov1_4_5_8_9_10	33.8419	-639.392	0.6549597	0	0.039068679	0	0
cov1_4_5_8_10_11	33.8521	-639.397	0.6549201	0	0.031613089	0	0
cov4_5_8_9_10_11	33.8603	-639.401	0.6534587	0	0	0	0
cov3_4_5_8_9_11	33.8693	-639.405	0.6526768	0	0	0	0
cov3_4_5_8_10_11	33.8788	-639.41	0.6535408	0	0	0	0
cov1_2_4_6_7_9	33.9046	-639.423	0.6528291	0	-0.078756152	0	0.055502662
cov3_4_5_8_9_10	33.9046	-639.423	0.6538237	0	0	0	0
cov1_2_4_6_7_10	33.9092	-639.425	0.6537742	0	-0.077554125	0	0.054176647
cov1_4_6_7_8_10	33.9196	-639.43	0.6551593	0	-0.056436219	0	0
cov1_4_6_7_8_9	33.9232	-639.432	0.6542084	0	-0.057024484	0	0
cov1_2_4_7_9_10	33.9291	-639.435	0.6532968	0	-0.052798853	0	0.055190342
cov1_2_4_7_9_11	33.9345	-639.438	0.6523432	0	-0.060900542	0	0.055286041
cov1_2_4_7_10_11	33.9402	-639.441	0.653295	0	-0.059370569	0	0.053923012
cov1_2_3_4_7_9	33.9463	-639.444	0.6529295	0	-0.053694944	0	0.055022831
cov1_4_7_8_9_10	33.9528	-639.447	0.6546788	0	-0.027752469	0	0
cov1_2_3_4_7_10	33.9533	-639.447	0.6538571	0	-0.051697106	0	0.054141636
cov1_4_7_8_10_11	33.9564	-639.449	0.6545177	0	-0.036693345	0	0
cov1_3_4_7_8_9	33.959	-639.45	0.6545099	0	-0.032271255	0	0
cov1_4_7_8_9_11	33.9592	-639.45	0.6535619	0	-0.037555348	0	0
cov1_3_4_7_8_10	33.961	-639.451	0.6554316	0	-0.030637115	0	0
cov2_4_6_7_8_10	33.9758	-639.459	0.6559048	0	0	0	0.032784363
cov2_4_6_7_8_9	33.9775	-639.459	0.6550215	0	0	0	0.033822303
cov2_4_7_8_9_10	33.9852	-639.463	0.6562324	0	0	0	0.023924489
cov1_2_4_6_8_9	33.9853	-639.463	0.6528009	0	-0.077721072	0	0.054908798
cov2_4_7_8_10_11	33.9867	-639.464	0.6566545	0	0	0	0.029423661
cov1_2_4_6_8_10	33.9898	-639.466	0.6537483	0	-0.076559548	0	0.053605454
cov2_4_7_8_9_11	33.9899	-639.466	0.6558508	0	0	0	0.030227685
cov2_3_4_7_8_10	33.9976	-639.469	0.6563566	0	0	0	0.027081256
cov2_3_4_7_8_9	34.0041	-639.473	0.6555777	0	0	0	0.027156421
cov1_2_4_8_9_10	34.0065	-639.474	0.6532671	0	-0.052772844	0	0.054609307
cov1_2_4_8_9_11	34.0141	-639.478	0.6523548	0	-0.060252859	0	0.054800147
cov1_2_4_8_10_11	34.0196	-639.48	0.6532841	0	-0.058717006	0	0.053420201
cov4_6_7_8_9_10	34.0232	-639.482	0.655928	0	0	0	0
cov1_2_3_4_8_9	34.0247	-639.483	0.6527997	0	-0.053108269	0	0.055788792
cov4_7_8_9_10_11	34.0255	-639.483	0.6562284	0	0	0	0
cov3_4_7_8_9_10	34.0265	-639.484	0.6563288	0	0	0	0
cov1_2_3_4_8_10	34.0309	-639.486	0.6537315	0	-0.051109325	0	0.054894159
cov3_4_6_7_8_10	34.039	-639.49	0.6564773	0	0	0	0
cov3_4_7_8_10_11	34.0414	-639.491	0.6567472	0	0	0	0
cov4_6_7_8_10_11	34.0415	-639.491	0.6563759	0	0	0	0
cov3_4_6_7_8_9	34.0435	-639.492	0.6556223	0	0	0	0
cov3_4_7_8_9_11	34.0468	-639.494	0.6559565	0	0	0	0
cov4_6_7_8_9_11	34.0475	-639.494	0.6554968	0	0	0	0
cov1_4_6_7_9_10	34.0478	-639.495	0.6545946	0	-0.054866281	0	0
cov1_3_4_6_7_10	34.0542	-639.498	0.6553208	0	-0.05741166	0	0

cov1_3_4_6_7_9	34.0542	-639.498	0.6544206	0	-0.058382364	0	0
cov1_4_6_7_10_11	34.0545	-639.498	0.6545104	0	-0.061382501	0	0
cov1_4_6_7_9_11	34.0589	-639.5	0.6535718	0	-0.061785441	0	0
cov1_4_7_9_10_11	34.0751	-639.508	0.6538723	0	-0.037775081	0	0
cov1_3_4_7_9_10	34.0759	-639.509	0.6548795	0	-0.032374291	0	0
cov1_3_4_7_9_11	34.0798	-639.511	0.653825	0	-0.042137486	0	0
cov1_3_4_7_10_11	34.0809	-639.511	0.6547255	0	-0.040791996	0	0
cov2_4_6_7_9_10	34.0943	-639.518	0.6553405	0	0	0	0.033471484
cov2_4_6_7_10_11	34.1105	-639.526	0.6558755	0	0	0	0.034522775
cov2_4_7_9_10_11	34.1109	-639.526	0.6561966	0	0	0	0.029226662
cov2_4_6_7_9_11	34.1115	-639.526	0.6550599	0	0	0	0.035657153
cov2_3_4_6_7_10	34.1126	-639.527	0.6556921	0	0	0	0.034235173
cov2_3_4_6_7_9	34.1144	-639.528	0.6548445	0	0	0	0.034805467
cov2_3_4_7_9_10	34.1241	-639.533	0.655956	0	0	0	0.026118645
cov1_4_6_8_9_10	34.125	-639.533	0.6545546	0	-0.054157502	0	0
cov2_3_4_7_10_11	34.1265	-639.534	0.656412	0	0	0	0.030983168
cov2_3_4_7_9_11	34.1304	-639.536	0.6556623	0	0	0	0.031329625
cov1_4_6_8_10_11	34.1338	-639.538	0.6545091	0	-0.060124772	0	0
cov1_4_6_8_9_11	34.1382	-639.54	0.6535814	0	-0.060525063	0	0
cov1_3_4_6_8_10	34.1384	-639.54	0.6552059	0	-0.055756192	0	0
cov1_3_4_6_8_9	34.1394	-639.54	0.6542968	0	-0.056717631	0	0
cov1_4_8_9_10_11	34.1515	-639.546	0.65395	0	-0.037402934	0	0
cov1_3_4_5_6_9	34.1556	-639.548	0.6533632	0	0.085766014	0	0
cov1_3_4_5_6_10	34.1563	-639.549	0.6541591	0	0.085229582	0	0
cov1_3_4_8_9_10	34.1577	-639.55	0.6547663	0	-0.031483601	0	0
cov3_4_6_7_9_10	34.1585	-639.55	0.6559666	0	0	0	0
cov4_6_7_9_10_11	34.1631	-639.552	0.6558125	0	0	0	0
cov3_4_7_9_10_11	34.164	-639.553	0.6563321	0	0	0	0
cov1_3_4_8_9_11	34.1647	-639.553	0.6537411	0	-0.040648533	0	0
cov1_3_4_8_10_11	34.1648	-639.553	0.6546451	0	-0.039310512	0	0
cov2_4_6_8_9_10	34.1696	-639.555	0.655285	0	0	0	0.03319655
cov3_4_6_7_10_11	34.1772	-639.559	0.6564047	0	0	0	0
cov3_4_6_7_9_11	34.1812	-639.561	0.6555681	0	0	0	0
cov2_4_6_8_10_11	34.1853	-639.563	0.6558924	0	0	0	0.03437072
cov2_3_4_6_8_10	34.1859	-639.564	0.6555368	0	0	0	0.035278536
cov2_4_8_9_10_11	34.1862	-639.564	0.6562059	0	0	0	0.02897639
cov2_4_6_8_9_11	34.1863	-639.564	0.654999	0	0	0	0.035520345
cov2_3_4_6_8_9	34.1885	-639.565	0.6546839	0	0	0	0.035860744
cov1_2_3_4_5_9	34.1946	-639.568	0.6527581	0	0.1002006	0	0.021279046
cov1_3_4_5_9_10	34.1974	-639.569	0.6537013	0	0.11485964	0	0
cov2_3_4_8_9_10	34.1976	-639.569	0.6558026	0	0	0	0.027178417
cov1_2_3_4_5_10	34.1984	-639.57	0.653577	0	0.10068588	0	0.020786348
cov2_3_4_8_10_11	34.1988	-639.57	0.6562631	0	0	0	0.032212433
cov2_3_4_8_9_11	34.2033	-639.572	0.6554982	0	0	0	0.032577213
cov1_3_4_5_9_11	34.2139	-639.578	0.6532313	0	0.11516849	0	0
cov1_3_4_5_10_11	34.2167	-639.579	0.6540301	0	0.11496932	0	0
cov2_3_4_5_6_9	34.2256	-639.583	0.6514988	0	0	0	0.042701541

cov2_3_4_5_6_10	34.2268	-639.584	0.6523822	0	0	0	0.042122903
cov3_4_6_8_9_10	34.2359	-639.589	0.6558256	0	0	0	0
cov4_6_8_9_10_11	34.2372	-639.589	0.6557617	0	0	0	0
cov1_2_3_7_8_9	34.2396	-639.59	0.6531614	0	-0.031544053	0	0.11201063
cov3_4_8_9_10_11	34.2407	-639.591	0.6561862	0	0	0	0
cov1_2_6_7_8_9	34.2463	-639.594	0.6539925	0	-0.068247179	0	0.10784299
cov1_2_3_7_8_10	34.2502	-639.596	0.6540044	0	-0.029602429	0	0.11148801
cov3_4_6_8_10_11	34.2545	-639.598	0.6562937	0	0	0	0
cov3_4_6_8_9_11	34.2593	-639.6	0.6554327	0	0	0	0
cov1_2_6_7_8_10	34.2605	-639.601	0.6547831	0	-0.067098203	0	0.10732849
cov1_2_5_7_8_9	34.2775	-639.609	0.6535198	0	-0.061839962	0	0.10902651
cov2_3_5_7_8_9	34.2798	-639.611	0.653993	0	0	0	0.10221778
cov2_3_5_7_8_10	34.2875	-639.614	0.6547805	0	0	0	0.10182253
cov1_2_5_7_8_10	34.2906	-639.616	0.6543857	0	-0.061232227	0	0.10837884
cov2_3_7_8_9_10	34.2992	-639.62	0.6551289	0	0	0	0.087255124
cov3_4_5_6_9_10	34.3058	-639.624	0.6527669	0	0	0	0
cov1_2_3_6_7_9	34.3121	-639.627	0.6529595	0	-0.065863819	0	0.1091933
cov2_3_7_8_9_11	34.3128	-639.627	0.6547988	0	0	0	0.089256834
cov2_3_6_7_8_9	34.3144	-639.628	0.6547383	0	0	0	0.087545993
cov1_2_7_8_9_11	34.3145	-639.628	0.653641	0	-0.04004363	0	0.10983456
cov2_3_7_8_10_11	34.3156	-639.628	0.6555064	0	0	0	0.089584284
cov2_3_6_7_8_10	34.3164	-639.629	0.6555098	0	0	0	0.087389975
cov3_4_5_6_10_11	34.3179	-639.63	0.652835	0	0	0	0
cov1_2_7_8_9_10	34.3192	-639.63	0.6544409	0	-0.032032132	0	0.11047214
cov1_2_3_6_7_10	34.32	-639.631	0.6537727	0	-0.064606896	0	0.10864459
cov3_4_5_6_9_11	34.3201	-639.631	0.6519625	0	0	0	0
cov1_2_7_8_10_11	34.3309	-639.636	0.6544444	0	-0.038467073	0	0.10926351
cov1_2_5_6_7_9	34.3519	-639.647	0.6533351	0	-0.092322746	0	0.10653542
cov1_2_5_6_7_10	34.3624	-639.652	0.6541914	0	-0.092305437	0	0.1058473
cov1_2_3_7_9_11	34.3726	-639.657	0.6525643	0	-0.03960814	0	0.11094002
cov1_2_3_7_9_10	34.3739	-639.658	0.6534594	0	-0.031024073	0	0.11162717
cov1_2_3_5_7_9	34.3808	-639.661	0.6530123	0	-0.043569188	0	0.11100889
cov1_2_3_6_8_9	34.3823	-639.662	0.6528347	0	-0.064827288	0	0.10928068
cov1_2_3_7_10_11	34.3824	-639.662	0.653392	0	-0.037936466	0	0.11039167
cov1_2_6_7_9_10	34.3878	-639.665	0.6542081	0	-0.065734885	0	0.10776331
cov1_2_6_7_9_11	34.388	-639.665	0.6535259	0	-0.07095463	0	0.10739004
cov1_2_3_6_8_10	34.3895	-639.665	0.6536642	0	-0.06360462	0	0.1087155
cov1_2_3_5_7_10	34.3909	-639.666	0.6538639	0	-0.042353285	0	0.11042457
cov2_5_7_8_9_10	34.393	-639.667	0.6559659	0	0	0	0.089395424
cov2_7_8_9_10_11	34.3942	-639.668	0.6561672	0	0	0	0.087625193
cov2_6_7_8_9_10	34.3961	-639.669	0.6560894	0	0	0	0.086092147
cov2_5_6_7_8_9	34.4008	-639.671	0.6557168	0	0	0	0.088872082
cov2_5_7_8_9_11	34.4009	-639.671	0.6556843	0	0	0	0.090626046
cov1_2_6_7_10_11	34.4018	-639.672	0.6542895	0	-0.069979937	0	0.10683442
cov2_6_7_8_9_11	34.4035	-639.672	0.6558894	0	0	0	0.087340916
cov1_2_5_7_9_11	34.4045	-639.673	0.6529024	0	-0.069745762	0	0.10806627
cov1_2_5_7_9_10	34.4054	-639.673	0.6537735	0	-0.062687246	0	0.10845477

cov2_5_6_7_8_10	34.4113	-639.676	0.6564559	0	0	0	0.088278876
cov2_5_7_8_10_11	34.4119	-639.677	0.6564115	0	0	0	0.090110602
cov2_3_5_6_7_9	34.4125	-639.677	0.654105	0	0	0	0.097999628
cov2_6_7_8_10_11	34.413	-639.677	0.6566149	0	0	0	0.087068797
cov2_3_5_7_9_10	34.416	-639.679	0.6543036	0	0	0	0.10126536
cov1_2_5_7_10_11	34.4166	-639.679	0.6537558	0	-0.069423957	0	0.10738522
cov2_3_5_6_7_10	34.4183	-639.68	0.6548999	0	0	0	0.097638293
cov2_3_4_5_9_10	34.4233	-639.682	0.6506002	0	0	0	0.063940337
cov2_3_5_7_9_11	34.4265	-639.684	0.6538248	0	0	0	0.10086863
cov2_3_4_5_9_11	34.4273	-639.684	0.6496764	0	0	0	0.060610576
cov2_3_4_5_10_11	34.4322	-639.687	0.6506168	0	0	0	0.060083494
cov2_3_5_7_10_11	34.4336	-639.687	0.6545764	0	0	0	0.10045536
cov1_2_3_8_9_10	34.4405	-639.691	0.6533427	0	-0.030720073	0	0.11165079
cov1_2_3_8_9_11	34.4417	-639.691	0.6524805	0	-0.038742031	0	0.11104127
cov1_2_5_6_8_9	34.4428	-639.692	0.6533129	0	-0.091090933	0	0.10650498
cov2_3_7_9_10_11	34.4446	-639.693	0.6550832	0	0	0	0.089065688
cov2_3_6_7_9_10	34.4457	-639.693	0.6550032	0	0	0	0.088170509
cov1_2_7_9_10_11	34.4465	-639.694	0.6538457	0	-0.039841239	0	0.10949988
cov1_2_3_8_10_11	34.4508	-639.696	0.6533052	0	-0.037068876	0	0.1104858
cov1_2_3_5_8_9	34.4509	-639.696	0.6529155	0	-0.039576274	0	0.11131791
cov1_2_5_6_8_10	34.4533	-639.697	0.6541507	0	-0.091089734	0	0.1058371
cov2_3_6_7_9_11	34.4593	-639.7	0.6547275	0	0	0	0.089135615
cov1_2_3_5_8_10	34.4603	-639.701	0.6537731	0	-0.038393005	0	0.11072427
cov2_3_6_7_10_11	34.4621	-639.702	0.6554803	0	0	0	0.088858473
cov2_3_5_6_8_9	34.4725	-639.707	0.6539818	0	0	0	0.099055237
cov2_3_5_8_9_10	34.4775	-639.709	0.6541333	0	0	0	0.10238587
cov2_3_5_6_8_10	34.4779	-639.71	0.6547403	0	0	0	0.098568391
cov1_2_6_8_9_10	34.4787	-639.71	0.6541736	0	-0.064508455	0	0.10772893
cov1_2_6_8_9_11	34.4804	-639.711	0.6535461	0	-0.069186536	0	0.10742418
cov2_3_5_8_9_11	34.4885	-639.715	0.6536519	0	0	0	0.10202784
cov1_2_5_8_9_10	34.4923	-639.717	0.6537852	0	-0.062579746	0	0.1085109
cov1_2_5_8_9_11	34.4935	-639.717	0.6529292	0	-0.068821613	0	0.10803844
cov1_2_6_8_10_11	34.4943	-639.718	0.6543249	0	-0.068207429	0	0.10686686
cov2_3_5_8_10_11	34.4952	-639.718	0.6544097	0	0	0	0.10160567
cov1_2_5_8_10_11	34.5056	-639.723	0.6537755	0	-0.068499085	0	0.10735979
cov2_3_8_9_10_11	34.5095	-639.725	0.6548763	0	0	0	0.089631945
cov2_3_6_8_9_10	34.5109	-639.726	0.6548661	0	0	0	0.0885509
cov2_3_6_8_9_11	34.5247	-639.733	0.6546076	0	0	0	0.089652531
cov2_3_6_8_10_11	34.5269	-639.734	0.6553582	0	0	0	0.089367608
cov2_5_7_9_10_11	34.5325	-639.737	0.6559102	0	0	0	0.089989982
cov2_5_6_7_9_10	34.5329	-639.737	0.6558945	0	0	0	0.088895562
cov2_6_7_9_10_11	34.5343	-639.738	0.6560396	0	0	0	0.087797923
cov1_2_8_9_10_11	34.5353	-639.738	0.6538628	0	-0.038972222	0	0.10947227
cov2_5_6_7_9_11	34.5405	-639.741	0.6556887	0	0	0	0.089855266
cov2_5_6_7_10_11	34.5511	-639.746	0.6563347	0	0	0	0.089283797
cov2_5_8_9_10_11	34.6178	-639.78	0.6558965	0	0	0	0.090195235
cov2_5_6_8_9_10	34.6188	-639.78	0.6557982	0	0	0	0.089072269

cov3_4_5_9_10_11	34.6191	-639.78	0.6503995	0	0	0	0
cov2_6_8_9_10_11	34.6192	-639.78	0.6560045	0	0	0	0.088283752
cov2_5_6_8_9_11	34.626	-639.784	0.6556439	0	0	0	0.090163775
cov2_5_6_8_10_11	34.6367	-639.789	0.6563884	0	0	0	0.089546693
cov1_2_3_4_6_10	34.8958	-639.919	0.6517078	0	-0.070328007	0	0.071763086
cov1_4_5_6_7_8	34.9	-639.921	0.6550637	0	0.091422995	0	0
cov1_2_3_4_6_9	34.9121	-639.927	0.6506584	0	-0.071720287	0	0.073087686
cov1_2_3_4_9_10	34.9367	-639.939	0.6512906	0	-0.040692517	0	0.072402137
cov2_4_5_6_7_8	34.9444	-639.943	0.6528562	0	0	0	0.055027878
cov2_3_5_6_9_10	34.9483	-639.945	0.6525289	0	0	0	0.11059831
cov1_2_3_4_10_11	34.9558	-639.949	0.6520667	0	-0.035504112	0	0.072538565
cov1_2_3_5_6_10	34.9617	-639.951	0.6526651	0	-0.012592272	0	0.112657
cov2_3_5_6_10_11	34.9628	-639.952	0.6530846	0	0	0	0.11117684
cov1_2_3_5_6_9	34.964	-639.953	0.6518237	0	-0.012545442	0	0.11334232
cov2_3_5_6_9_11	34.9646	-639.953	0.6523145	0	0	0	0.11190537
cov1_2_3_4_9_11	34.969	-639.955	0.6510173	0	-0.037059221	0	0.073894866
cov2_3_4_9_10_11	35.0059	-639.974	0.6536082	0	0	0	0.057642774
cov1_2_4_5_6_7	35.0086	-639.975	0.6540446	0	0.067751659	0	0.030514422
cov2_3_4_6_10_11	35.0234	-639.982	0.6540342	0	0	0	0.057067185
cov1_2_4_5_7_8	35.0322	-639.987	0.6540505	0	0.12363138	0	0.031721829
cov1_3_4_5_6_7	35.0342	-639.988	0.6547507	0	0.10558826	0	0
cov1_2_3_5_9_10	35.0416	-639.991	0.6523393	0	0.023109501	0	0.11579051
cov2_3_4_6_9_11	35.0427	-639.992	0.6531577	0	0	0	0.058135584
cov1_4_5_6_7_11	35.0454	-639.993	0.6546762	0	0.089747203	0	0
cov2_3_4_6_9_10	35.047	-639.994	0.6529744	0	0	0	0.053471993
cov2_3_5_9_10_11	35.0568	-639.999	0.651606	0	0	0	0.12149143
cov3_6_7_8_10_11	35.0606	-640.001	0.657767	0	0	0	0
cov1_2_3_5_9_11	35.0612	-640.001	0.651964	0	0.024806795	0	0.11635459
cov1_2_3_5_10_11	35.0616	-640.001	0.6528402	0	0.025076591	0	0.11564648
cov3_6_7_8_9_11	35.0623	-640.002	0.6570274	0	0	0	0
cov1_3_4_5_7_8	35.0645	-640.003	0.6547762	0	0.16258374	0	0
cov3_5_6_7_8_10	35.0656	-640.003	0.6583938	0	0	0	0
cov1_4_5_7_8_11	35.0661	-640.004	0.6544971	0	0.14358612	0	0
cov3_5_6_7_8_9	35.068	-640.005	0.6576172	0	0	0	0
cov3_6_7_8_9_10	35.0723	-640.007	0.6579465	0	0	0	0
cov1_2_3_6_9_10	35.076	-640.009	0.6515444	0	-0.062358323	0	0.10924158
cov2_4_5_6_7_11	35.0833	-640.012	0.6523127	0	0	0	0.052688651
cov1_3_6_7_8_10	35.0835	-640.012	0.6584595	0	0.011615094	0	0
cov5_6_7_8_9_10	35.0841	-640.013	0.6586605	0	0	0	0
cov1_3_6_7_8_9	35.0844	-640.013	0.6576967	0	0.010774508	0	0
cov1_5_6_7_8_10	35.0849	-640.013	0.6585947	0	-0.025716954	0	0
cov1_5_6_7_8_9	35.0849	-640.013	0.6577924	0	-0.024954384	0	0
cov1_2_4_5_6_8	35.0851	-640.013	0.6540292	0	0.069974793	0	0.029452829
cov1_2_3_6_10_11	35.0901	-640.016	0.6522881	0	-0.058012783	0	0.10947148
cov5_6_7_8_9_11	35.0905	-640.016	0.6579565	0	0	0	0
cov5_6_7_8_10_11	35.091	-640.016	0.6587346	0	0	0	0
cov4_5_6_7_8_11	35.0946	-640.018	0.6532444	0	0	0	0

cov1_3_4_5_6_8	35.0969	-640.019	0.6546487	0	0.11057472	0	0
cov2_3_4_5_6_7	35.0969	-640.019	0.6526308	0	0	0	0.056889125
cov1_2_3_6_9_11	35.0997	-640.02	0.651389	0	-0.059052878	0	0.11018667
cov1_4_5_6_8_11	35.1202	-640.031	0.6546696	0	0.091593292	0	0
cov6_7_8_9_10_11	35.1223	-640.032	0.658386	0	0	0	0
cov3_4_5_6_7_8	35.1254	-640.033	0.6542454	0	0	0	0
cov1_6_7_8_9_11	35.1348	-640.038	0.6580491	0	0.000910047	0	0
cov1_3_4_6_9_10	35.1377	-640.039	0.6530012	0	-0.043209526	0	0
cov1_6_7_8_10_11	35.1399	-640.041	0.6587972	0	0.001501029	0	0
cov1_6_7_8_9_10	35.1437	-640.042	0.6590058	0	0.010053691	0	0
cov1_3_4_6_10_11	35.1525	-640.047	0.65372	0	-0.038932694	0	0
cov1_2_3_4_5_7	35.1545	-640.048	0.6537149	0	0.1341194	0	0.033400617
cov5_7_8_9_10_11	35.1573	-640.049	0.657409	0	0	0	0
cov1_2_3_9_10_11	35.1591	-640.05	0.6518223	0	-0.0231496	0	0.11246673
cov1_2_4_5_7_11	35.1593	-640.05	0.6535516	0	0.11703301	0	0.031158073
cov2_4_5_6_8_11	35.1674	-640.054	0.6522685	0	0	0	0.052635578
cov3_5_7_8_10_11	35.1693	-640.055	0.6573155	0	0	0	0
cov3_5_7_8_9_11	35.17	-640.056	0.6565004	0	0	0	0
cov1_5_7_8_9_11	35.1765	-640.059	0.6571524	0	0.00573604	0	0
cov2_3_4_5_6_8	35.1769	-640.059	0.6524557	0	0	0	0.058155594
cov1_5_7_8_10_11	35.1777	-640.059	0.6579726	0	0.005140237	0	0
cov1_3_4_6_9_11	35.178	-640.06	0.6527242	0	-0.039424395	0	0
cov2_3_6_9_10_11	35.1784	-640.06	0.6536512	0	0	0	0.093469562
cov3_4_6_9_10_11	35.1861	-640.064	0.6543369	0	0	0	0
cov1_3_7_8_9_11	35.1901	-640.066	0.6571289	0	0.046760303	0	0
cov1_3_7_8_10_11	35.1911	-640.066	0.6579012	0	0.047957399	0	0
cov1_5_7_8_9_10	35.1911	-640.066	0.6583524	0	0.018020918	0	0
cov1_3_4_5_7_11	35.1921	-640.067	0.6542178	0	0.15345507	0	0
cov1_3_5_7_8_9	35.1966	-640.069	0.6576977	0	0.031553116	0	0
cov1_3_5_7_8_10	35.1971	-640.069	0.6585129	0	0.032023885	0	0
cov1_3_4_9_10_11	35.2006	-640.071	0.6531781	0	-0.008868237	0	0
cov3_5_6_7_10_11	35.2031	-640.072	0.6578371	0	0	0	0
cov3_6_7_9_10_11	35.2031	-640.072	0.6572863	0	0	0	0
cov3_5_7_8_9_10	35.2033	-640.072	0.6576716	0	0	0	0
cov3_5_6_7_9_10	35.2045	-640.073	0.6579419	0	0	0	0
cov3_5_6_7_9_11	35.2061	-640.074	0.6570722	0	0	0	0
cov1_3_7_8_9_10	35.2084	-640.075	0.6583395	0	0.061373741	0	0
cov1_5_6_7_10_11	35.2132	-640.077	0.6578381	0	-0.033176566	0	0
cov1_5_6_7_9_11	35.2142	-640.078	0.6570471	0	-0.03220483	0	0
cov1_2_3_4_5_8	35.2157	-640.078	0.6536403	0	0.13875176	0	0.032881956
cov1_5_6_7_9_10	35.2181	-640.08	0.6580781	0	-0.024306617	0	0
cov1_3_6_7_10_11	35.2188	-640.08	0.6577352	0	0.004383689	0	0
cov1_3_5_6_7_10	35.2189	-640.08	0.6582044	0	-0.011116515	0	0
cov1_3_6_7_9_11	35.2203	-640.081	0.6569864	0	0.003729457	0	0
cov1_3_5_6_7_9	35.2211	-640.081	0.6574177	0	-0.011089913	0	0
cov5_6_7_9_10_11	35.2214	-640.081	0.6581971	0	0	0	0
cov1_3_6_7_9_10	35.2262	-640.084	0.6580212	0	0.013446792	0	0

cov1_2_4_5_8_11	35.2315	-640.086	0.6535796	0	0.11872808	0	0.030207429
cov3_4_5_6_7_11	35.243	-640.092	0.6533569	0	0	0	0
cov1_7_8_9_10_11	35.2463	-640.094	0.6584745	0	0.04556682	0	0
cov1_3_4_5_8_11	35.2534	-640.097	0.6541516	0	0.15823675	0	0
cov1_6_7_9_10_11	35.2739	-640.108	0.6582923	0	0.003091592	0	0
cov3_6_8_9_10_11	35.277	-640.109	0.6571529	0	0	0	0
cov3_5_6_8_10_11	35.2793	-640.11	0.6576809	0	0	0	0
cov3_5_6_8_9_10	35.2802	-640.111	0.6577856	0	0	0	0
cov3_5_6_8_9_11	35.283	-640.112	0.6569154	0	0	0	0
cov3_5_7_9_10_11	35.2899	-640.116	0.656881	0	0	0	0
cov1_3_6_8_10_11	35.292	-640.117	0.6576448	0	0.006000412	0	0
cov1_3_6_8_9_11	35.2944	-640.118	0.6568797	0	0.005349743	0	0
cov1_3_5_6_8_10	35.2964	-640.119	0.6580928	0	-0.006132633	0	0
cov1_5_7_9_10_11	35.2965	-640.119	0.6574329	0	0.003871796	0	0
cov1_3_6_8_9_10	35.2971	-640.119	0.6578898	0	0.014528007	0	0
cov1_3_5_6_8_9	35.2993	-640.12	0.6573085	0	-0.006086148	0	0
cov1_3_5_7_10_11	35.3041	-640.123	0.6575881	0	0.016555786	0	0
cov1_3_5_7_9_11	35.305	-640.123	0.6567917	0	0.016338396	0	0
cov1_5_6_8_10_11	35.3062	-640.124	0.6578517	0	-0.031375402	0	0
cov1_5_6_8_9_11	35.3071	-640.124	0.6570615	0	-0.030405903	0	0
cov1_5_6_8_9_10	35.3083	-640.125	0.6580585	0	-0.023108263	0	0
cov5_6_8_9_10_11	35.3116	-640.126	0.6581857	0	0	0	0
cov1_3_7_9_10_11	35.3182	-640.13	0.6574351	0	0.046860354	0	0
cov1_3_5_7_9_10	35.32	-640.131	0.6580182	0	0.028981409	0	0
cov3_4_5_6_8_11	35.3318	-640.137	0.6532098	0	0	0	0
cov1_6_8_9_10_11	35.3668	-640.154	0.6583021	0	0.004882237	0	0
cov3_5_8_9_10_11	35.3722	-640.157	0.6567029	0	0	0	0
cov1_3_5_8_10_11	35.3817	-640.161	0.6575122	0	0.021441349	0	0
cov1_3_5_8_9_11	35.3833	-640.162	0.6567159	0	0.021251934	0	0
cov3_7_8_9_10_11	35.3849	-640.163	0.6550204	0	0	0	0
cov1_5_8_9_10_11	35.385	-640.163	0.6574477	0	0.004778247	0	0
cov1_3_8_9_10_11	35.3883	-640.165	0.657337	0	0.047811082	0	0
cov1_3_5_8_9_10	35.3949	-640.168	0.6579097	0	0.033242641	0	0
cov2_4_5_7_8_11	35.3981	-640.17	0.6492014	0	0	0	0.092465639
cov1_4_5_6_9_11	35.4164	-640.179	0.6559956	0	0.042261272	0	0
cov1_4_5_6_10_11	35.4334	-640.187	0.6567775	0	0.04174641	0	0
cov4_5_6_9_10_11	35.4457	-640.194	0.6555602	0	0	0	0
cov1_4_5_9_10_11	35.4561	-640.199	0.6561926	0	0.067975852	0	0
cov2_3_4_5_7_8	35.457	-640.199	0.6509232	0	0	0	0.093637458
cov2_4_5_6_9_11	35.4572	-640.199	0.6551859	0	0	0	0.002207431
cov1_2_4_5_9_11	35.4574	-640.199	0.6562258	0	0.079801395	0	-0.013588886
cov2_4_5_6_10_11	35.4732	-640.207	0.6560301	0	0	0	0.001436133
cov1_2_4_5_6_9	35.4746	-640.208	0.6554833	0	0.041306853	0	-0.018140284
cov1_2_4_5_10_11	35.4757	-640.208	0.6570405	0	0.080459206	0	-0.014320573
cov1_4_5_6_9_10	35.4795	-640.21	0.6551731	0	0.025416874	0	0
cov1_2_4_5_6_10	35.4894	-640.215	0.6562036	0	0.041476608	0	-0.018849727
cov2_4_5_6_9_10	35.4938	-640.218	0.6549304	0	0	0	-0.005230567

cov2_3_4_5_7_11	35.5	-640.221	0.6499784	0	0	0	0.082582427
cov1_2_4_5_9_10	35.5031	-640.222	0.655681	0	0.065326242	0	-0.017914235
cov1_2_3_4_5_6	35.534	-640.238	0.6529856	0	0.15017851	0	0.024943159
cov2_3_5_6_7_8	35.5533	-640.247	0.6530453	0	0	0	0.11781145
cov1_3_4_5_6_11	35.5578	-640.25	0.6539857	0	0.17142078	0	0
cov1_2_5_6_7_8	35.5852	-640.263	0.653266	0	-0.021862424	0	0.11680602
cov2_3_4_5_8_11	35.5928	-640.267	0.6497749	0	0	0	0.084284009
cov2_4_5_9_10_11	35.5935	-640.267	0.6541178	0	0	0	0.018288596
cov2_5_6_7_8_11	35.5967	-640.269	0.6537454	0	0	0	0.11183411
cov1_2_4_6_7_8	35.6979	-640.32	0.6519967	0	-0.070334275	0	0.099297929
cov2_3_5_6_7_11	35.7036	-640.322	0.6528212	0	0	0	0.11719221
cov1_2_3_5_6_7	35.7047	-640.323	0.6528824	0	-0.005104958	0	0.11863331
cov1_2_5_6_7_11	35.7296	-640.335	0.6529654	0	-0.022622892	0	0.11663289
cov1_2_3_4_5_11	35.732	-640.337	0.6531023	0	0.21291869	0	0.026052657
cov1_2_3_6_7_8	35.7388	-640.34	0.6526166	0	-0.066592035	0	0.11472036
cov1_2_6_7_8_11	35.752	-640.347	0.6520903	0	-0.068587134	0	0.11514733
cov1_2_3_4_6_7	35.7574	-640.349	0.652677	0	-0.07153952	0	0.085949424
cov2_3_5_6_8_11	35.7826	-640.362	0.6526483	0	0	0	0.11842509
cov1_2_3_5_6_8	35.784	-640.363	0.6527817	0	-0.000188157	0	0.1190268
cov1_2_3_5_7_8	35.7951	-640.368	0.6530749	0	0.055997338	0	0.1239058
cov3_4_5_7_8_11	35.8006	-640.371	0.6509974	0	0	0	0
cov2_3_4_6_7_8	35.8032	-640.372	0.6544649	0	0	0	0.067648883
cov1_2_5_7_8_11	35.8087	-640.375	0.6529205	0	0.03528193	0	0.12152543
cov1_2_5_6_8_11	35.825	-640.383	0.6529681	0	-0.020710689	0	0.11667344
cov1_2_3_4_7_8	35.832	-640.387	0.6527908	0	-0.015428053	0	0.088895354
cov1_2_4_6_7_11	35.8401	-640.391	0.6517005	0	-0.070582353	0	0.0991156
cov2_3_4_7_8_11	35.8496	-640.395	0.6535898	0	0	0	0.079297374
cov2_3_5_7_8_11	35.8519	-640.397	0.6511655	0	0	0	0.13466874
cov2_4_6_7_8_11	35.8577	-640.399	0.6540064	0	0	0	0.081826477
cov1_2_3_4_6_8	35.868	-640.405	0.652525	0	-0.069799151	0	0.087060504
cov1_2_3_6_7_11	35.8786	-640.41	0.6523688	0	-0.066956627	0	0.11449651
cov2_3_6_7_8_11	35.8837	-640.412	0.6545143	0	0	0	0.095677451
cov1_2_3_5_7_11	35.9215	-640.431	0.6525687	0	0.048101466	0	0.12292541
cov2_3_4_6_7_11	35.9224	-640.432	0.6546677	0	0	0	0.069333279
cov1_2_4_7_8_11	35.9296	-640.435	0.6515221	0	-0.013817604	0	0.10338315
cov1_3_5_6_9_10	35.9306	-640.436	0.656605	0	0.055762882	0	0
cov1_2_4_6_8_11	35.933	-640.437	0.6516904	0	-0.068912781	0	0.098731184
cov1_3_5_6_10_11	35.9443	-640.443	0.6568547	0	0.055528526	0	0
cov1_2_3_4_7_11	35.9467	-640.444	0.6523447	0	-0.021437102	0	0.087633567
cov1_3_5_6_9_11	35.9581	-640.45	0.6560796	0	0.056521853	0	0
cov1_2_4_6_9_11	35.9592	-640.45	0.654492	0	-0.063366927	0	0.033707586
cov1_2_4_6_10_11	35.9631	-640.452	0.6554722	0	-0.062248518	0	0.032184211
cov1_2_3_7_8_11	35.9662	-640.454	0.6523317	0	-0.010606807	0	0.11931607
cov2_3_4_5_6_11	35.9775	-640.459	0.6500998	0	0	0	0.075542524
cov1_2_3_6_8_11	35.9821	-640.462	0.6522739	0	-0.065008304	0	0.11473869
cov1_2_4_9_10_11	35.991	-640.466	0.6548963	0	-0.035370293	0	0.03308409
cov1_4_6_9_10_11	35.9936	-640.467	0.655648	0	-0.050135177	0	0

cov3_5_6_9_10_11	35.9979	-640.47	0.6555203	0	0	0	0
cov1_2_3_5_8_11	35.9983	-640.47	0.652508	0	0.052723725	0	0.12331132
cov1_3_4_6_7_8	36.0052	-640.473	0.6555306	0	-0.039340817	0	0
cov1_3_6_9_10_11	36.0069	-640.474	0.6559509	0	0.017017377	0	0
cov2_3_4_6_8_11	36.0239	-640.483	0.6545024	0	0	0	0.0709099
cov1_2_4_6_9_10	36.0257	-640.483	0.6537327	0	-0.079335619	0	0.028616783
cov1_2_3_4_8_11	36.0531	-640.497	0.652247	0	-0.019943549	0	0.088705794
cov2_4_6_9_10_11	36.0585	-640.5	0.6567931	0	0	0	0.018426874
cov3_4_6_7_8_11	36.0635	-640.502	0.6562107	0	0	0	0
cov1_3_5_9_10_11	36.0835	-640.512	0.6564894	0	0.10131036	0	0
cov1_3_4_6_7_11	36.1318	-640.537	0.6551413	0	-0.04202781	0	0
cov1_3_4_7_8_11	36.2246	-640.583	0.6550226	0	0.014250365	0	0
cov1_3_4_6_8_11	36.2536	-640.597	0.6550462	0	-0.039194292	0	0
cov1_4_6_7_8_11	36.2602	-640.601	0.6543503	0	-0.028329377	0	0
cov1_2_3_5_6_11	36.4762	-640.709	0.6521731	0	0.06899689	0	0.1248521
cov1_5_6_7_8_11	36.6439	-640.793	0.657866	0	0.053053263	0	0
cov1_3_5_6_7_8	36.6479	-640.795	0.6581731	0	0.067960899	0	0
cov3_5_6_7_8_11	36.7156	-640.828	0.6569111	0	0	0	0
cov1_3_6_7_8_11	36.7592	-640.85	0.6573763	0	0.010168381	0	0
cov1_3_5_6_7_11	36.7878	-640.865	0.6576248	0	0.061750717	0	0
cov1_2_4_5_6_11	36.795	-640.868	0.6562247	0	0.13466571	0	-0.009264955
cov1_2_5_6_9_11	36.875	-640.908	0.6555754	0	-0.063823083	0	0.1039522
cov1_3_5_6_8_11	36.8761	-640.909	0.6575337	0	0.067644314	0	0
cov1_2_5_6_10_11	36.891	-640.916	0.6563897	0	-0.063864725	0	0.1032598
cov1_2_6_9_10_11	36.8954	-640.918	0.6563234	0	-0.042304072	0	0.10487754
cov2_5_6_9_10_11	36.9515	-640.946	0.6575457	0	0	0	0.092840215
cov1_2_5_9_10_11	36.9652	-640.953	0.6559434	0	-0.028779844	0	0.10642423
cov1_3_5_7_8_11	36.9709	-640.956	0.6577049	0	0.13414326	0	0
cov1_2_5_6_9_10	36.9934	-640.967	0.6544669	0	-0.084078512	0	0.10055635
cov1_2_3_4_6_11	37.558	-641.25	0.6508712	0	-0.047949178	0	0.11977487
cov1_5_6_9_10_11	37.6947	-641.318	0.6597816	0	-0.001622268	0	0
cov2_4_5_6_7_8_9	40.7749	-639.253	0.654266	0	0	0	0.022139807
cov3_4_5_6_7_8_9	40.7849	-639.258	0.6541662	0	0	0	0
cov2_4_5_6_7_8_10	40.7865	-639.259	0.6550851	0	0	0	0.021488912
cov3_4_5_6_7_8_10	40.7925	-639.262	0.6549846	0	0	0	0
cov4_5_6_7_8_9_11	40.7925	-639.262	0.6542326	0	0	0	0
cov4_5_6_7_8_9_10	40.7927	-639.262	0.6549592	0	0	0	0
cov1_4_5_6_7_8_9	40.801	-639.266	0.6547728	0	0.011711678	0	0
cov4_5_6_7_8_10_11	40.802	-639.267	0.6550477	0	0	0	0
cov1_4_5_6_7_8_10	40.8112	-639.272	0.6555782	0	0.011144747	0	0
cov2_4_5_7_8_9_11	40.8113	-639.272	0.6532376	0	0	0	0.029407465
cov2_3_4_5_7_8_9	40.8125	-639.272	0.6529572	0	0	0	0.041465769
cov2_4_5_7_8_9_10	40.8176	-639.275	0.6539101	0	0	0	0.033878611
cov1_2_4_5_7_8_9	40.8208	-639.276	0.6540555	0	0.018206116	0	0.025873568
cov1_3_4_5_7_8_9	40.8216	-639.277	0.6544115	0	0.057579038	0	0
cov2_4_5_7_8_10_11	40.8243	-639.278	0.6540718	0	0	0	0.02890478
cov2_3_4_5_7_8_10	40.8254	-639.279	0.6538111	0	0	0	0.041282923

cov1_3_4_5_7_8_10	40.8313	-639.282	0.6552159	0	0.058357491	0	0
cov1_2_4_5_7_8_10	40.8344	-639.283	0.6548997	0	0.01883253	0	0.025248403
cov1_4_5_7_8_9_11	40.8357	-639.284	0.6542162	0	0.031056089	0	0
cov1_4_5_7_8_9_10	40.8386	-639.285	0.6550823	0	0.038851012	0	0
cov1_4_5_7_8_10_11	40.8472	-639.29	0.6550273	0	0.030818826	0	0
cov4_5_7_8_9_10_11	40.8536	-639.293	0.6535997	0	0	0	0
cov3_4_5_7_8_9_11	40.865	-639.298	0.6528987	0	0	0	0
cov3_4_5_7_8_10_11	40.875	-639.303	0.6537516	0	0	0	0
cov2_3_4_5_6_7_9	40.8912	-639.312	0.6534238	0	0	0	0.030235744
cov2_3_4_5_6_7_10	40.9003	-639.316	0.6542111	0	0	0	0.029826416
cov3_4_5_7_8_9_10	40.9011	-639.317	0.6540484	0	0	0	0
cov2_4_5_6_7_9_10	40.9142	-639.323	0.6545214	0	0	0	0.022529908
cov2_4_5_6_7_9_11	40.9178	-639.325	0.6538649	0	0	0	0.020863153
cov1_2_4_5_6_7_9	40.9234	-639.328	0.6539616	0	-0.007381048	0	0.025517419
cov1_3_4_5_6_7_9	40.9236	-639.328	0.654325	0	0.030062541	0	0
cov3_4_5_6_7_9_11	40.9237	-639.328	0.6535769	0	0	0	0
cov3_4_5_6_7_9_10	40.9284	-639.33	0.6544652	0	0	0	0
cov2_4_5_6_7_10_11	40.9289	-639.33	0.6546817	0	0	0	0.020168807
cov3_4_5_6_7_10_11	40.9307	-639.331	0.6543609	0	0	0	0
cov1_3_4_5_6_7_10	40.9312	-639.332	0.6551138	0	0.030128557	0	0
cov4_5_6_7_9_10_11	40.9313	-639.332	0.6544304	0	0	0	0
cov2_3_4_5_7_9_11	40.9348	-639.333	0.6524631	0	0	0	0.03600828
cov1_2_3_4_5_7_9	40.9349	-639.333	0.6535528	0	0.033935134	0	0.028281218
cov1_2_4_5_6_7_10	40.9349	-639.333	0.6547934	0	-0.007371028	0	0.024882296
cov1_4_5_6_7_9_10	40.9406	-639.336	0.6549938	0	0.012633994	0	0
cov1_4_5_6_7_9_11	40.9418	-639.337	0.6542216	0	0.007517269	0	0
cov2_4_5_7_9_10_11	40.9427	-639.337	0.6535089	0	0	0	0.0286782
cov2_3_4_5_7_9_10	40.9457	-639.339	0.6532713	0	0	0	0.040345295
cov1_2_3_4_5_7_10	40.946	-639.339	0.654393	0	0.035050986	0	0.02775287
cov2_3_4_5_7_10_11	40.946	-639.339	0.6532684	0	0	0	0.035730087
cov1_4_5_6_7_10_11	40.9514	-639.342	0.6550114	0	0.006746454	0	0
cov2_3_4_5_6_8_9	40.9514	-639.342	0.6532647	0	0	0	0.031198953
cov1_2_4_5_7_9_11	40.9516	-639.342	0.6534656	0	0.010285449	0	0.025157675
cov1_2_4_5_7_9_10	40.9534	-639.343	0.6542802	0	0.017031576	0	0.025769427
cov1_3_4_5_7_9_11	40.9542	-639.343	0.6537657	0	0.047083102	0	0
cov1_3_4_5_7_9_10	40.9571	-639.344	0.6546667	0	0.055326826	0	0
cov2_3_4_5_6_8_10	40.96	-639.346	0.6540819	0	0	0	0.030800174
cov1_3_4_5_7_10_11	40.9631	-639.348	0.6545613	0	0.047516365	0	0
cov1_2_4_5_7_10_11	40.9643	-639.348	0.6542939	0	0.010634742	0	0.024503751
cov1_2_4_6_7_8_9	40.9656	-639.349	0.6529338	0	-0.080856313	0	0.055392562
cov1_4_5_7_9_10_11	40.9675	-639.35	0.654432	0	0.029509747	0	0
cov1_2_4_6_7_8_10	40.9703	-639.351	0.6538812	0	-0.079651475	0	0.054065029
cov1_3_4_5_6_8_9	40.9807	-639.356	0.6542246	0	0.034787566	0	0
cov3_4_5_6_8_9_11	40.9877	-639.36	0.653421	0	0	0	0
cov1_3_4_5_6_8_10	40.9878	-639.36	0.6550168	0	0.034840861	0	0
cov2_4_5_6_8_9_10	40.9878	-639.36	0.6544166	0	0	0	0.022069065
cov3_4_5_6_8_9_10	40.9913	-639.362	0.6543281	0	0	0	0

cov1_2_3_4_5_8_9	40.9919	-639.362	0.6533833	0	0.03887017	0	0.027781974
cov2_4_5_6_8_9_11	40.9924	-639.362	0.6538368	0	0	0	0.020683909
cov3_4_5_7_9_10_11	40.9939	-639.363	0.6532373	0	0	0	0
cov3_4_5_6_8_10_11	40.9941	-639.363	0.6542284	0	0	0	0
cov1_2_4_7_8_9_10	40.9971	-639.364	0.6533946	0	-0.05286404	0	0.055131428
cov1_2_4_5_6_8_9	40.9975	-639.365	0.6539588	0	-0.005252493	0	0.024448101
cov2_3_4_5_8_9_11	40.9991	-639.365	0.6522763	0	0	0	0.037264424
cov1_2_3_4_5_8_10	41.0024	-639.367	0.6543003	0	0.039693593	0	0.027225154
cov1_2_4_7_8_9_11	41.0029	-639.367	0.6524368	0	-0.060858109	0	0.055266781
cov2_4_5_6_8_10_11	41.0035	-639.368	0.6546449	0	0	0	0.019986434
cov4_5_6_8_9_10_11	41.0054	-639.369	0.6544021	0	0	0	0
cov1_2_4_7_8_10_11	41.0087	-639.37	0.653393	0	-0.059355845	0	0.053844228
cov1_2_4_5_6_8_10	41.009	-639.37	0.6547817	0	-0.005271764	0	0.023829511
cov2_3_4_5_8_9_10	41.0091	-639.371	0.6531047	0	0	0	0.041547215
cov2_3_4_5_8_10_11	41.0099	-639.371	0.6530997	0	0	0	0.03698376
cov1_3_4_5_8_9_11	41.0118	-639.372	0.6536966	0	0.05186543	0	0
cov1_4_5_6_8_9_10	41.0124	-639.372	0.6549622	0	0.013849682	0	0
cov1_3_4_5_8_9_10	41.0125	-639.372	0.6545676	0	0.059487978	0	0
cov1_2_3_4_7_8_9	41.0148	-639.373	0.6529916	0	-0.05350736	0	0.055516921
cov1_4_5_6_8_9_11	41.0152	-639.374	0.6542254	0	0.009304763	0	0
cov2_4_5_8_9_10_11	41.0166	-639.374	0.6534822	0	0	0	0.028436045
cov1_3_4_5_8_10_11	41.0203	-639.376	0.6544967	0	0.052275485	0	0
cov1_2_3_4_7_8_10	41.0218	-639.377	0.6539238	0	-0.051501603	0	0.054629885
cov1_2_4_5_8_9_10	41.0247	-639.378	0.6542804	0	0.018185073	0	0.024729492
cov1_2_4_5_8_9_11	41.0248	-639.378	0.6535006	0	0.012083609	0	0.024173746
cov1_4_5_6_8_10_11	41.025	-639.378	0.6550175	0	0.008542284	0	0
cov1_2_4_5_8_10_11	41.0375	-639.385	0.6543275	0	0.012433104	0	0.023520855
cov1_4_5_8_9_10_11	41.0386	-639.385	0.654447	0	0.030498683	0	0
cov3_4_5_8_9_10_11	41.0629	-639.397	0.6530737	0	0	0	0
cov1_2_4_6_7_9_10	41.0961	-639.414	0.6532867	0	-0.078311894	0	0.054450381
cov1_2_4_6_7_9_11	41.1055	-639.419	0.6524184	0	-0.083915614	0	0.054758017
cov1_4_6_7_8_9_10	41.1079	-639.42	0.6547037	0	-0.057043872	0	0
cov1_2_4_6_7_10_11	41.1096	-639.421	0.6533641	0	-0.082889515	0	0.053391611
cov1_2_3_4_6_7_9	41.1136	-639.423	0.6528851	0	-0.078931473	0	0.054776374
cov1_4_6_7_8_10_11	41.1155	-639.424	0.6546353	0	-0.063348768	0	0
cov1_3_4_6_7_8_10	41.1174	-639.425	0.6553969	0	-0.059148654	0	0
cov1_3_4_6_7_8_9	41.1177	-639.425	0.6544926	0	-0.06011478	0	0
cov1_2_3_4_6_7_10	41.1185	-639.425	0.6537967	0	-0.077621212	0	0.053900179
cov1_4_6_7_8_9_11	41.1198	-639.426	0.6536966	0	-0.06375357	0	0
cov1_2_4_7_9_10_11	41.1253	-639.429	0.6527833	0	-0.060664054	0	0.054205209
cov1_2_3_4_7_9_10	41.138	-639.435	0.653357	0	-0.053180634	0	0.05429994
cov1_4_7_8_9_10_11	41.1429	-639.437	0.6540554	0	-0.037841946	0	0
cov1_2_3_4_7_9_11	41.1431	-639.438	0.652421	0	-0.061355357	0	0.054151696
cov1_3_4_7_8_9_10	41.1463	-639.439	0.6549536	0	-0.032020955	0	0
cov1_2_3_4_7_10_11	41.1492	-639.441	0.6533306	0	-0.059643826	0	0.053230803
cov1_3_4_7_8_9_11	41.1509	-639.441	0.6539003	0	-0.041692415	0	0
cov1_3_4_7_8_10_11	41.1518	-639.442	0.6548048	0	-0.040344416	0	0

cov2_4_6_7_8_9_10	41.1665	-639.449	0.6554891	0	0	0	0.032838119
cov1_2_4_6_8_9_10	41.1766	-639.454	0.6532598	0	-0.077321876	0	0.053878842
cov2_4_7_8_9_10_11	41.1791	-639.456	0.6563056	0	0	0	0.029202696
cov2_4_6_7_8_10_11	41.182	-639.457	0.6560633	0	0	0	0.034025022
cov2_4_6_7_8_9_11	41.1831	-639.457	0.655195	0	0	0	0.0351731
cov2_3_4_6_7_8_10	41.1841	-639.458	0.655796	0	0	0	0.034198668
cov2_3_4_6_7_8_9	41.1864	-639.459	0.6549473	0	0	0	0.034787512
cov1_2_4_6_8_9_11	41.1877	-639.46	0.6524212	0	-0.082396655	0	0.054245257
cov2_3_4_7_8_9_10	41.1916	-639.462	0.6560169	0	0	0	0.026650329
cov1_2_4_6_8_10_11	41.1917	-639.462	0.6533627	0	-0.081406371	0	0.052891744
cov2_3_4_7_8_10_11	41.194	-639.463	0.6564757	0	0	0	0.031546011
cov1_2_3_4_6_8_9	41.1944	-639.463	0.6527525	0	-0.077623322	0	0.055575697
cov2_3_4_7_8_9_11	41.1981	-639.465	0.655708	0	0	0	0.031902541
cov1_2_3_4_6_8_10	41.1985	-639.465	0.6536675	0	-0.076316481	0	0.05468691
cov1_2_4_8_9_10_11	41.2048	-639.468	0.6527972	0	-0.060010217	0	0.053700182
cov1_2_3_4_8_9_10	41.2158	-639.474	0.6532347	0	-0.052585319	0	0.055050895
cov1_2_3_4_8_9_11	41.2234	-639.478	0.6523148	0	-0.060200005	0	0.055036652
cov1_2_3_4_8_10_11	41.2287	-639.48	0.6532341	0	-0.058478913	0	0.054040876
cov3_4_6_7_8_9_10	41.2296	-639.481	0.6560683	0	0	0	0
cov4_6_7_8_9_10_11	41.2324	-639.482	0.6559695	0	0	0	0
cov3_4_7_8_9_10_11	41.233	-639.482	0.6563963	0	0	0	0
cov1_3_4_6_7_9_10	41.2419	-639.487	0.6548637	0	-0.058114118	0	0
cov1_4_6_7_9_10_11	41.2432	-639.488	0.6540581	0	-0.061911661	0	0
cov3_4_6_7_8_10_11	41.2482	-639.49	0.6565388	0	0	0	0
cov1_3_4_6_7_10_11	41.2501	-639.491	0.6547924	0	-0.06425046	0	0
cov1_3_4_6_7_9_11	41.2509	-639.491	0.6539056	0	-0.065022678	0	0
cov3_4_6_7_8_9_11	41.2524	-639.492	0.65569	0	0	0	0
cov1_3_4_7_9_10_11	41.2668	-639.499	0.6542636	0	-0.042033134	0	0
cov2_4_6_7_9_10_11	41.3009	-639.516	0.6554803	0	0	0	0.034629803
cov2_3_4_6_7_9_10	41.3033	-639.518	0.6552802	0	0	0	0.034184636
cov2_3_4_7_9_10_11	41.3192	-639.526	0.6560711	0	0	0	0.030699358
cov2_3_4_6_7_10_11	41.3195	-639.526	0.655833	0	0	0	0.035263837
cov2_3_4_6_7_9_11	41.3208	-639.526	0.654958	0	0	0	0.035929715
cov1_4_6_8_9_10_11	41.3224	-639.527	0.6540647	0	-0.060662588	0	0
cov1_3_4_6_8_9_10	41.3262	-639.529	0.6547488	0	-0.056452964	0	0
cov1_3_4_6_8_10_11	41.3361	-639.534	0.65471	0	-0.062103326	0	0
cov1_3_4_6_8_9_11	41.3378	-639.535	0.6538257	0	-0.062856249	0	0
cov1_2_3_4_5_6_9	41.346	-639.539	0.6527814	0	0.07023956	0	0.020930906
cov1_2_3_4_5_6_10	41.3476	-639.54	0.6535999	0	0.070119606	0	0.020435523
cov1_3_4_5_6_9_10	41.3483	-639.54	0.6537505	0	0.084502939	0	0
cov1_3_4_8_9_10_11	41.3509	-639.541	0.6541839	0	-0.040545971	0	0
cov1_3_4_5_6_9_11	41.3649	-639.548	0.6534003	0	0.086062278	0	0
cov1_2_3_6_7_8_9	41.3652	-639.549	0.6530166	0	-0.068000787	0	0.10912563
cov1_3_4_5_6_10_11	41.3657	-639.549	0.6541703	0	0.085341226	0	0
cov3_4_6_7_9_10_11	41.3677	-639.55	0.6560265	0	0	0	0
cov1_2_3_6_7_8_10	41.3728	-639.552	0.6538378	0	-0.066772904	0	0.10857245
cov2_4_6_8_9_10_11	41.3756	-639.554	0.6554446	0	0	0	0.03447675

cov2_3_4_6_8_9_10	41.3768	-639.554	0.6551423	0	0	0	0.035113945
cov1_2_3_4_5_9_10	41.3877	-639.56	0.6531482	0	0.099128116	0	0.021005379
cov2_3_4_8_9_10_11	41.3915	-639.562	0.6559242	0	0	0	0.031927959
cov2_3_4_6_8_10_11	41.3923	-639.562	0.6556954	0	0	0	0.036412455
cov2_3_4_6_8_9_11	41.3942	-639.563	0.6548655	0	0	0	0.037110971
cov1_2_3_4_5_9_11	41.4039	-639.568	0.6527099	0	0.099792431	0	0.021206042
cov1_3_4_5_9_10_11	41.4064	-639.569	0.6536123	0	0.11371894	0	0
cov1_2_3_4_5_10_11	41.4076	-639.57	0.6535269	0	0.10002408	0	0.020667977
cov1_2_5_6_7_8_9	41.4129	-639.572	0.6534343	0	-0.094184244	0	0.10638956
cov2_3_4_5_6_9_10	41.4161	-639.574	0.651949	0	0	0	0.04205998
cov1_2_5_6_7_8_10	41.4235	-639.578	0.6542724	0	-0.094177162	0	0.10572253
cov2_3_4_5_6_9_11	41.4336	-639.583	0.6513627	0	0	0	0.041911519
cov2_3_4_5_6_10_11	41.4345	-639.583	0.6522296	0	0	0	0.041237648
cov1_2_3_7_8_9_11	41.4355	-639.584	0.6526334	0	-0.03949596	0	0.11105713
cov1_2_3_7_8_9_10	41.4364	-639.584	0.6535248	0	-0.030984588	0	0.11173397
cov1_2_3_5_7_8_9	41.4447	-639.588	0.6530869	0	-0.042137981	0	0.1112151
cov3_4_6_8_9_10_11	41.445	-639.588	0.6558761	0	0	0	0
cov1_2_3_7_8_10_11	41.4453	-639.589	0.6534604	0	-0.037820337	0	0.11050289
cov1_2_6_7_8_9_10	41.448	-639.59	0.6542965	0	-0.067857817	0	0.10757534
cov1_2_6_7_8_9_11	41.4489	-639.59	0.6536373	0	-0.072838449	0	0.10726226
cov1_2_3_5_7_8_10	41.4548	-639.593	0.6539414	0	-0.040916274	0	0.11062732
cov1_2_6_7_8_10_11	41.4627	-639.597	0.6544186	0	-0.071857459	0	0.10670408
cov2_3_5_6_7_8_9	41.4684	-639.6	0.6542429	0	0	0	0.097975342
cov2_3_5_6_7_8_10	41.4741	-639.603	0.654985	0	0	0	0.097570242
cov1_2_5_7_8_9_11	41.4744	-639.603	0.6530168	0	-0.069536517	0	0.10809512
cov1_2_5_7_8_9_10	41.4749	-639.603	0.6538769	0	-0.062746706	0	0.10863107
cov2_3_5_7_8_9_10	41.4769	-639.604	0.6543665	0	0	0	0.1016676
cov1_2_5_7_8_10_11	41.4867	-639.609	0.6538672	0	-0.069207237	0	0.10741369
cov2_3_5_7_8_9_11	41.4874	-639.61	0.6538718	0	0	0	0.10132632
cov2_3_5_7_8_10_11	41.4946	-639.613	0.6546278	0	0	0	0.10090893
cov2_3_7_8_9_10_11	41.5072	-639.62	0.6551571	0	0	0	0.089196004
cov3_4_5_6_9_10_11	41.5073	-639.62	0.652407	0	0	0	0
cov2_3_6_7_8_9_10	41.5085	-639.62	0.6551196	0	0	0	0.087419768
cov1_2_3_6_7_9_10	41.509	-639.62	0.6533209	0	-0.065317058	0	0.10891062
cov1_2_3_6_7_9_11	41.5134	-639.623	0.652565	0	-0.070868464	0	0.10854238
cov1_2_7_8_9_10_11	41.516	-639.624	0.6539508	0	-0.039722419	0	0.10956685
cov1_2_3_5_6_7_9	41.5187	-639.625	0.6528988	0	-0.073891029	0	0.10859079
cov1_2_3_6_7_10_11	41.5208	-639.626	0.6533605	0	-0.069820019	0	0.10796957
cov2_3_6_7_8_9_11	41.522	-639.627	0.6548585	0	0	0	0.088544463
cov2_3_6_7_8_10_11	41.5244	-639.628	0.6556147	0	0	0	0.088263294
cov1_2_3_5_6_7_10	41.5262	-639.629	0.6537259	0	-0.073299795	0	0.10798283
cov1_2_5_6_7_9_10	41.5496	-639.641	0.6536876	0	-0.093171517	0	0.10615054
cov1_2_5_6_7_9_11	41.5536	-639.643	0.6529578	0	-0.097311985	0	0.10588963
cov1_2_5_6_7_10_11	41.5636	-639.648	0.6537925	0	-0.09749799	0	0.10519285
cov1_2_3_7_9_10_11	41.5692	-639.651	0.6529196	0	-0.039182999	0	0.11064245
cov1_2_3_5_7_9_11	41.5767	-639.654	0.6524882	0	-0.051414874	0	0.11005323
cov1_2_3_5_7_9_10	41.5768	-639.654	0.6533794	0	-0.044155971	0	0.1106322

cov1_2_3_6_8_9_10	41.5786	-639.655	0.6532083	0	-0.064305486	0	0.10899048
cov1_2_3_6_8_9_11	41.5851	-639.658	0.6524711	0	-0.069382855	0	0.10868846
cov1_2_3_5_7_10_11	41.5858	-639.659	0.6533149	0	-0.050497066	0	0.10942206
cov1_2_6_7_9_10_11	41.5896	-639.661	0.6538339	0	-0.07063949	0	0.1071171
cov1_2_3_5_6_8_9	41.5908	-639.661	0.652802	0	-0.069272667	0	0.10894798
cov1_2_3_6_8_10_11	41.5917	-639.662	0.6532797	0	-0.068336225	0	0.10811011
cov1_2_3_5_6_8_10	41.5977	-639.665	0.6536278	0	-0.068705381	0	0.10833691
cov1_2_5_7_9_10_11	41.6016	-639.667	0.6532612	0	-0.070789142	0	0.10765001
cov2_5_6_7_8_9_10	41.6017	-639.667	0.6560349	0	0	0	0.088445499
cov2_5_7_8_9_10_11	41.6017	-639.667	0.6560147	0	0	0	0.090065811
cov2_6_7_8_9_10_11	41.6035	-639.668	0.6562038	0	0	0	0.087165633
cov2_5_6_7_8_9_11	41.6089	-639.67	0.6558139	0	0	0	0.089469885
cov2_3_5_6_7_9_10	41.61	-639.671	0.6545283	0	0	0	0.097676637
cov2_3_4_5_9_10_11	41.6142	-639.673	0.6501615	0	0	0	0.059699243
cov2_5_6_7_8_10_11	41.6196	-639.676	0.656546	0	0	0	0.088845312
cov2_3_5_6_7_9_11	41.6217	-639.677	0.654114	0	0	0	0.09796352
cov2_3_5_7_9_10_11	41.6237	-639.678	0.6541845	0	0	0	0.10037731
cov2_3_5_6_7_10_11	41.6275	-639.68	0.6548522	0	0	0	0.097429697
cov1_2_3_8_9_10_11	41.6377	-639.685	0.6528582	0	-0.038270602	0	0.11068843
cov1_2_5_6_8_9_10	41.6404	-639.686	0.6536685	0	-0.091936691	0	0.10611841
cov1_2_5_6_8_9_11	41.646	-639.689	0.6529756	0	-0.095541007	0	0.10592416
cov1_2_3_5_8_9_10	41.6465	-639.689	0.6532863	0	-0.040164156	0	0.11093629
cov1_2_3_5_8_9_11	41.6485	-639.69	0.6524205	0	-0.046850168	0	0.11043753
cov2_3_6_7_9_10_11	41.654	-639.693	0.6550913	0	0	0	0.08894748
cov1_2_5_6_8_10_11	41.6561	-639.694	0.6537914	0	-0.095736608	0	0.10523303
cov1_2_3_5_8_10_11	41.6572	-639.695	0.6532552	0	-0.045934974	0	0.10981528
cov2_3_5_6_8_9_10	41.6697	-639.701	0.6543401	0	0	0	0.098629462
cov2_3_5_6_8_9_11	41.6818	-639.707	0.6539569	0	0	0	0.098944997
cov1_2_6_8_9_10_11	41.6819	-639.707	0.6538589	0	-0.068857644	0	0.10715923
cov2_3_5_8_9_10_11	41.6852	-639.709	0.6540177	0	0	0	0.10152758
cov2_3_5_6_8_10_11	41.6872	-639.71	0.6547029	0	0	0	0.09840574
cov1_2_5_8_9_10_11	41.6905	-639.711	0.6532825	0	-0.069862369	0	0.10762328
cov2_3_6_8_9_10_11	41.7188	-639.725	0.6549723	0	0	0	0.089452704
cov2_5_6_7_9_10_11	41.7414	-639.737	0.65598	0	0	0	0.089388864
cov2_5_6_8_9_10_11	41.8269	-639.779	0.6559475	0	0	0	0.08969494
cov1_2_4_5_6_7_8	42.0695	-639.901	0.6541612	0	0.065997202	0	0.030274193
cov1_2_3_4_6_9_10	42.0852	-639.909	0.6512714	0	-0.071061968	0	0.071901131
cov1_3_4_5_6_7_8	42.0906	-639.911	0.654842	0	0.1050301	0	0
cov1_2_3_4_6_10_11	42.0985	-639.915	0.6521088	0	-0.065907369	0	0.072129485
cov1_4_5_6_7_8_11	42.106	-639.919	0.654804	0	0.087960446	0	0
cov1_2_3_4_6_9_11	42.1136	-639.923	0.6511183	0	-0.06694308	0	0.073432547
cov2_4_5_6_7_8_11	42.1409	-639.936	0.6524846	0	0	0	0.051879707
cov1_2_3_4_9_10_11	42.1424	-639.937	0.6515891	0	-0.036790566	0	0.07268446
cov2_3_4_5_6_7_8	42.1522	-639.942	0.6527269	0	0	0	0.056712504
cov1_2_3_5_6_9_10	42.1538	-639.943	0.6522592	0	-0.013506161	0	0.11287686
cov2_3_5_6_9_10_11	42.1552	-639.944	0.6527133	0	0	0	0.11126004
cov1_2_3_5_6_10_11	42.1697	-639.951	0.652848	0	-0.010956436	0	0.11288048

cov1_2_3_5_6_9_11	42.1716	-639.952	0.6520161	0	-0.010746697	0	0.11360134
cov1_2_3_4_5_6_7	42.1984	-639.965	0.6537763	0	0.08015424	0	0.032346168
cov2_3_4_6_9_10_11	42.215	-639.973	0.6536748	0	0	0	0.057059843
cov1_2_4_5_6_7_11	42.2154	-639.974	0.6538248	0	0.065000938	0	0.030125315
cov1_2_3_4_5_7_8	42.2258	-639.979	0.6538059	0	0.13584688	0	0.033371318
cov1_2_4_5_7_8_11	42.2338	-639.983	0.6536622	0	0.11766393	0	0.031006171
cov1_3_4_5_6_7_11	42.2397	-639.986	0.654469	0	0.10186916	0	0
cov1_2_3_5_9_10_11	42.2508	-639.991	0.6523811	0	0.023618989	0	0.11585782
cov3_6_7_8_9_10_11	42.2535	-639.993	0.6574086	0	0	0	0
cov3_5_6_7_8_10_11	42.2576	-639.995	0.6579259	0	0	0	0
cov3_5_6_7_8_9_10	42.258	-639.995	0.6580224	0	0	0	0
cov3_5_6_7_8_9_11	42.2608	-639.996	0.6571739	0	0	0	0
cov1_2_3_4_5_6_8	42.2625	-639.997	0.6536916	0	0.085582386	0	0.031825662
cov1_3_4_5_7_8_11	42.2636	-639.998	0.6543021	0	0.15527494	0	0
cov1_3_6_7_8_10_11	42.2697	-640.001	0.6578119	0	0.002295057	0	0
cov1_3_6_7_8_9_11	42.2716	-640.002	0.6570593	0	0.001640763	0	0
cov1_3_5_6_7_8_10	42.2722	-640.002	0.6582759	0	-0.011596793	0	0
cov1_5_6_7_8_10_11	42.2722	-640.002	0.6579723	0	-0.034959805	0	0
cov1_5_6_7_8_9_11	42.2731	-640.003	0.6571689	0	-0.03396444	0	0
cov1_3_5_6_7_8_9	42.2746	-640.003	0.6574901	0	-0.011563964	0	0
cov1_5_6_7_8_9_10	42.2759	-640.004	0.6581898	0	-0.026343591	0	0
cov1_3_6_7_8_9_10	42.2762	-640.004	0.6580852	0	0.011144011	0	0
cov1_2_3_6_9_10_11	42.2806	-640.006	0.6518862	0	-0.058618466	0	0.10973052
cov5_6_7_8_9_10_11	42.2832	-640.008	0.658369	0	0	0	0
cov2_3_4_5_6_7_11	42.2916	-640.012	0.652191	0	0	0	0.054037453
cov1_2_4_5_6_8_11	42.2927	-640.012	0.6538467	0	0.067706669	0	0.029123415
cov3_4_5_6_7_8_11	42.2984	-640.015	0.6534642	0	0	0	0
cov1_3_4_5_6_8_11	42.3033	-640.018	0.6544027	0	0.10737409	0	0
cov1_6_7_8_9_10_11	42.3317	-640.032	0.6584087	0	0.001028109	0	0
cov1_3_4_6_9_10_11	42.3427	-640.037	0.6533164	0	-0.039514943	0	0
cov1_2_3_4_5_7_11	42.3555	-640.044	0.6534315	0	0.12810072	0	0.032649854
cov3_5_7_8_9_10_11	42.3574	-640.045	0.6569338	0	0	0	0
cov1_5_7_8_9_10_11	42.3661	-640.049	0.6575477	0	0.00406218	0	0
cov1_3_5_7_8_10_11	42.3703	-640.051	0.6576719	0	0.018217798	0	0
cov1_3_5_7_8_9_11	42.3713	-640.052	0.6568743	0	0.017958901	0	0
cov2_3_4_5_6_8_11	42.3722	-640.052	0.6520247	0	0	0	0.055372257
cov1_3_7_8_9_10_11	42.3813	-640.057	0.6575105	0	0.046905694	0	0
cov1_3_5_7_8_9_10	42.3857	-640.059	0.6580924	0	0.030532115	0	0
cov3_5_6_7_9_10_11	42.396	-640.064	0.6574746	0	0	0	0
cov1_3_5_6_7_10_11	42.4045	-640.068	0.6575504	0	-0.020475765	0	0
cov1_5_6_7_9_10_11	42.4046	-640.068	0.6574424	0	-0.033703378	0	0
cov1_3_5_6_7_9_11	42.4076	-640.07	0.6567645	0	-0.020242025	0	0
cov1_3_5_6_7_9_10	42.4109	-640.071	0.657821	0	-0.012020515	0	0
cov1_3_6_7_9_10_11	42.4118	-640.072	0.6573724	0	0.003977046	0	0
cov1_2_3_4_5_8_11	42.4181	-640.075	0.6532676	0	0.13323845	0	0.032213572
cov3_5_6_8_9_10_11	42.4723	-640.102	0.6573002	0	0	0	0
cov1_3_5_6_8_10_11	42.4844	-640.108	0.6574624	0	-0.014952337	0	0

cov1_3_6_8_9_10_11	42.4851	-640.109	0.6572692	0	0.005596293	0	0
cov1_3_5_6_8_9_11	42.4881	-640.11	0.656694	0	-0.014702903	0	0
cov1_3_5_6_8_9_10	42.4885	-640.11	0.6577101	0	-0.007026737	0	0
cov1_3_5_7_9_10_11	42.4935	-640.113	0.6571856	0	0.01516277	0	0
cov1_5_6_8_9_10_11	42.4975	-640.115	0.6574585	0	-0.031915884	0	0
cov1_3_5_8_9_10_11	42.5714	-640.152	0.6571133	0	0.020059934	0	0
cov2_3_4_5_7_8_11	42.582	-640.157	0.6500023	0	0	0	0.083349913
cov1_4_5_6_9_10_11	42.6164	-640.174	0.6562902	0	0.041109732	0	0
cov1_2_4_5_6_9_11	42.6171	-640.175	0.6563424	0	0.052984136	0	-0.013849548
cov1_2_4_5_6_10_11	42.6332	-640.183	0.6571426	0	0.052987299	0	-0.014561527
cov2_4_5_6_9_10_11	42.6549	-640.193	0.6555412	0	0	0	0.001560903
cov1_2_4_5_9_10_11	42.6567	-640.194	0.6565437	0	0.078805759	0	-0.013900145
cov1_2_4_5_6_9_10	42.6733	-640.203	0.6557162	0	0.040501211	0	-0.018466098
cov1_2_3_4_5_6_11	42.7389	-640.235	0.6532847	0	0.15268841	0	0.02555701
cov2_3_5_6_7_8_11	42.7613	-640.247	0.6529159	0	0	0	0.11719362
cov1_2_3_5_6_7_8	42.762	-640.247	0.6529565	0	-0.005503618	0	0.11866541
cov1_2_5_6_7_8_11	42.7928	-640.262	0.6530777	0	-0.024172942	0	0.11651086
cov1_2_3_4_6_7_8	42.8306	-640.281	0.6527226	0	-0.073257114	0	0.086536231
cov1_2_4_6_7_8_11	42.9059	-640.319	0.6518161	0	-0.072410798	0	0.099027523
cov1_2_3_5_6_7_11	42.9119	-640.322	0.6526682	0	-0.007666179	0	0.11830783
cov1_2_3_6_7_8_11	42.9467	-640.339	0.6524385	0	-0.068650915	0	0.11451736
cov1_2_3_4_6_7_11	42.9652	-640.349	0.6524975	0	-0.073755569	0	0.085676031
cov1_2_3_5_6_8_11	42.9918	-640.362	0.652605	0	-0.002280608	0	0.11875967
cov1_2_3_5_7_8_11	42.9959	-640.364	0.6526438	0	0.049905444	0	0.12317403
cov2_3_4_6_7_8_11	43.003	-640.367	0.6547754	0	0	0	0.069617595
cov1_2_3_4_7_8_11	43.0348	-640.383	0.6524001	0	-0.020919135	0	0.088312748
cov1_2_3_4_6_8_11	43.0765	-640.404	0.6523825	0	-0.071483945	0	0.086859245
cov1_3_5_6_9_10_11	43.1396	-640.436	0.6565307	0	0.054905451	0	0
cov1_2_4_6_9_10_11	43.1449	-640.438	0.6549799	0	-0.063029914	0	0.0325151
cov1_3_4_6_7_8_11	43.2102	-640.471	0.6552187	0	-0.043201179	0	0
cov1_3_5_6_7_8_11	43.8455	-640.789	0.6577087	0	0.0615865	0	0
cov1_2_5_6_9_10_11	44.0729	-640.902	0.6559131	0	-0.064780174	0	0.10357744
cov2_3_4_5_6_7_8_9	47.9447	-639.234	0.6535231	0	0	0	0.030070758
cov2_3_4_5_6_7_8_10	47.9535	-639.238	0.6543342	0	0	0	0.029675843
cov2_4_5_6_7_8_9_10	47.9741	-639.248	0.6546154	0	0	0	0.021550108
cov1_3_4_5_6_7_8_9	47.977	-639.25	0.6544133	0	0.029356235	0	0
cov3_4_5_6_7_8_9_11	47.9773	-639.25	0.6536673	0	0	0	0
cov2_4_5_6_7_8_9_11	47.9785	-639.251	0.6540346	0	0	0	0.020128106
cov3_4_5_6_7_8_9_10	47.9809	-639.252	0.6545362	0	0	0	0
cov1_2_4_5_6_7_8_9	47.9827	-639.253	0.6540794	0	-0.009203976	0	0.025271551
cov3_4_5_6_7_8_10_11	47.9841	-639.253	0.6544696	0	0	0	0
cov1_3_4_5_6_7_8_10	47.9845	-639.254	0.6552081	0	0.0293803	0	0
cov2_4_5_6_7_8_10_11	47.9896	-639.256	0.6548409	0	0	0	0.019434522
cov4_5_6_7_8_9_10_11	47.9902	-639.256	0.6545955	0	0	0	0
cov1_2_4_5_6_7_8_10	47.9943	-639.258	0.6549157	0	-0.009228816	0	0.024629448
cov1_2_3_4_5_7_8_9	47.9965	-639.26	0.6536379	0	0.035382756	0	0.028297053
cov2_3_4_5_7_8_9_11	47.9981	-639.26	0.6524944	0	0	0	0.036499483

cov1_4_5_6_7_8_9_10	47.9993	-639.261	0.6551056	0	0.010580902	0	0
cov1_4_5_6_7_8_9_11	48.0011	-639.262	0.6543459	0	0.005670302	0	0
cov1_2_3_4_5_7_8_10	48.0076	-639.265	0.6544707	0	0.036574638	0	0.027755824
cov2_3_4_5_7_8_9_10	48.0092	-639.266	0.6533333	0	0	0	0.040854424
cov2_3_4_5_7_8_10_11	48.0093	-639.266	0.6533223	0	0	0	0.036295386
cov2_4_5_7_8_9_10_11	48.0093	-639.266	0.6536152	0	0	0	0.028647955
cov1_4_5_6_7_8_10_11	48.0108	-639.267	0.6551404	0	0.00490426	0	0
cov1_3_4_5_7_8_9_11	48.016	-639.269	0.6538532	0	0.048612544	0	0
cov1_2_4_5_7_8_9_11	48.0181	-639.27	0.6535835	0	0.010650615	0	0.024982291
cov1_3_4_5_7_8_9_10	48.0184	-639.271	0.6547516	0	0.056706346	0	0
cov1_2_4_5_7_8_9_10	48.0196	-639.271	0.6543921	0	0.017291401	0	0.025585516
cov1_3_4_5_7_8_10_11	48.0248	-639.274	0.6546515	0	0.049043204	0	0
cov1_2_4_5_7_8_10_11	48.031	-639.277	0.6544149	0	0.01100598	0	0.024325661
cov1_4_5_7_8_9_10_11	48.0335	-639.278	0.6545662	0	0.029690642	0	0
cov3_4_5_7_8_9_10_11	48.059	-639.291	0.6532957	0	0	0	0
cov2_3_4_5_6_7_9_10	48.0888	-639.306	0.6537848	0	0	0	0.029801149
cov2_3_4_5_6_7_9_11	48.0911	-639.307	0.6530901	0	0	0	0.02799893
cov1_2_3_4_5_6_7_9	48.0994	-639.311	0.6535496	0	0.008421691	0	0.027866542
cov2_3_4_5_6_7_10_11	48.0994	-639.311	0.6538976	0	0	0	0.027530293
cov1_2_3_4_5_6_7_10	48.1084	-639.315	0.6543605	0	0.008947732	0	0.027331941
cov2_4_5_6_7_9_10_11	48.1169	-639.32	0.6542182	0	0	0	0.020254098
cov3_4_5_6_7_9_10_11	48.1196	-639.321	0.653925	0	0	0	0
cov1_3_4_5_6_7_9_10	48.1206	-639.322	0.6546708	0	0.029215543	0	0
cov1_2_4_5_6_7_9_10	48.1224	-639.322	0.6543155	0	-0.008261763	0	0.025216869
cov1_3_4_5_6_7_9_11	48.1226	-639.323	0.6538994	0	0.02380367	0	0
cov1_2_4_5_6_7_9_11	48.1245	-639.324	0.6535723	0	-0.012512177	0	0.024791638
cov1_3_4_5_6_7_10_11	48.1296	-639.326	0.6546491	0	0.023640967	0	0
cov2_3_4_5_7_9_10_11	48.1313	-639.327	0.6528191	0	0	0	0.035449963
cov1_2_3_4_5_7_9_11	48.1314	-639.327	0.6530566	0	0.026241946	0	0.02736948
cov1_2_3_4_5_7_9_10	48.1326	-639.328	0.6539	0	0.033307317	0	0.028003464
cov1_2_4_5_6_7_10_11	48.1355	-639.329	0.6543841	0	-0.012714003	0	0.024145762
cov1_4_5_6_7_9_10_11	48.1398	-639.331	0.6545405	0	0.006233979	0	0
cov1_2_3_4_5_7_10_11	48.1416	-639.332	0.6538709	0	0.027158707	0	0.026800235
cov2_3_4_5_6_8_9_10	48.1485	-639.336	0.6536347	0	0	0	0.030721887
cov1_2_4_5_7_9_10_11	48.1501	-639.336	0.6538263	0	0.009158907	0	0.024888295
cov1_3_4_5_7_9_10_11	48.1508	-639.337	0.6541077	0	0.046070204	0	0
cov2_3_4_5_6_8_9_11	48.1518	-639.337	0.6529385	0	0	0	0.029022149
cov1_2_4_6_7_8_9_10	48.1571	-639.34	0.653393	0	-0.080409991	0	0.054337385
cov1_2_3_4_5_6_8_9	48.1578	-639.34	0.6534665	0	0.01358733	0	0.027340611
cov2_3_4_5_6_8_10_11	48.1598	-639.341	0.6537387	0	0	0	0.028540235
cov1_2_3_4_5_6_8_10	48.1662	-639.344	0.6542788	0	0.014069235	0	0.026822893
cov1_2_4_6_7_8_9_11	48.1672	-639.345	0.6525382	0	-0.085819177	0	0.054673023
cov1_2_4_6_7_8_10_11	48.1713	-639.347	0.6534841	0	-0.084817084	0	0.05329193
cov1_2_3_4_6_7_8_9	48.175	-639.349	0.652947	0	-0.080899107	0	0.055214354
cov1_3_4_5_6_8_9_10	48.1774	-639.35	0.6545815	0	0.033927309	0	0
cov1_2_3_4_6_7_8_10	48.1796	-639.351	0.6538586	0	-0.07954945	0	0.054385203
cov1_3_4_5_6_8_9_11	48.1812	-639.352	0.6538037	0	0.029016362	0	0

cov3_4_5_6_8_9_10_11	48.183	-639.353	0.65379	0	0	0	0
cov1_3_4_5_6_8_10_11	48.1878	-639.355	0.6545916	0	0.02886868	0	0
cov1_2_3_4_5_8_9_10	48.1893	-639.356	0.6538224	0	0.037859991	0	0.027501698
cov1_2_3_4_5_8_9_11	48.1901	-639.356	0.6530018	0	0.031388698	0	0.026914059
cov2_4_5_6_8_9_10_11	48.1914	-639.357	0.6541877	0	0	0	0.02006696
cov1_2_4_7_8_9_10_11	48.1936	-639.358	0.6528921	0	-0.060615828	0	0.054171111
cov2_3_4_5_8_9_10_11	48.1951	-639.359	0.6526598	0	0	0	0.036697948
cov1_2_4_5_6_8_9_10	48.1964	-639.36	0.6542955	0	-0.006133775	0	0.024149483
cov1_2_3_4_5_8_10_11	48.1998	-639.361	0.6538211	0	0.032282228	0	0.026342533
cov1_2_4_5_6_8_9_11	48.2001	-639.361	0.6536055	0	-0.009882043	0	0.023787062
cov1_2_3_4_7_8_9_10	48.2063	-639.364	0.653423	0	-0.052987986	0	0.05478975
cov1_3_4_5_8_9_10_11	48.2081	-639.365	0.654046	0	0.05084116	0	0
cov1_2_4_5_6_8_10_11	48.2112	-639.367	0.6544194	0	-0.010034872	0	0.023114464
cov1_2_3_4_7_8_9_11	48.2119	-639.367	0.6524871	0	-0.061072523	0	0.054658436
cov1_4_5_6_8_9_10_11	48.2133	-639.368	0.6545471	0	0.008023639	0	0
cov1_2_3_4_7_8_10_11	48.218	-639.37	0.6534015	0	-0.059355676	0	0.053733555
cov1_2_4_5_8_9_10_11	48.2233	-639.373	0.6538364	0	0.011030705	0	0.023855323
cov1_2_4_6_7_9_10_11	48.2967	-639.41	0.6528719	0	-0.08358551	0	0.053675725
cov1_4_6_7_8_9_10_11	48.3041	-639.413	0.6541874	0	-0.063887155	0	0
cov1_3_4_6_7_8_9_10	48.305	-639.414	0.6549356	0	-0.059883003	0	0
cov1_2_3_4_6_7_9_10	48.3054	-639.414	0.6533148	0	-0.078409565	0	0.054054378
cov1_3_4_6_7_8_10_11	48.314	-639.418	0.6548962	0	-0.065847583	0	0
cov1_2_3_4_6_7_9_11	48.3146	-639.419	0.6524727	0	-0.084070715	0	0.054071648
cov1_3_4_6_7_8_9_11	48.3151	-639.419	0.6539939	0	-0.066555156	0	0
cov1_2_3_4_6_7_10_11	48.3189	-639.421	0.6533876	0	-0.082954246	0	0.053166771
cov1_2_3_4_7_9_10_11	48.3343	-639.428	0.6528445	0	-0.060984255	0	0.053400408
cov1_3_4_7_8_9_10_11	48.3376	-639.43	0.65434	0	-0.041588409	0	0
cov2_4_6_7_8_9_10_11	48.3724	-639.447	0.65565	0	0	0	0.034174269
cov2_3_4_6_7_8_9_10	48.375	-639.449	0.6553944	0	0	0	0.03405718
cov1_2_4_6_8_9_10_11	48.3787	-639.451	0.6528928	0	-0.082091015	0	0.053156696
cov1_2_3_4_6_8_9_10	48.3854	-639.454	0.6531887	0	-0.077104692	0	0.054843844
cov2_3_4_7_8_9_10_11	48.3866	-639.455	0.6561343	0	0	0	0.031263045
cov2_3_4_6_7_8_10_11	48.3904	-639.456	0.6559579	0	0	0	0.035358232
cov2_3_4_6_7_8_9_11	48.392	-639.457	0.6551251	0	0	0	0.036048676
cov1_2_3_4_6_8_9_11	48.3968	-639.46	0.6523758	0	-0.082280961	0	0.054936842
cov1_2_3_4_6_8_10_11	48.4003	-639.461	0.6532763	0	-0.081173913	0	0.05401497
cov1_2_3_4_8_9_10_11	48.414	-639.468	0.6528675	0	-0.059554993	0	0.054080911
cov1_3_4_6_7_9_10_11	48.438	-639.48	0.6543432	0	-0.064878536	0	0
cov3_4_6_7_8_9_10_11	48.4387	-639.481	0.6561273	0	0	0	0
cov2_3_4_6_7_9_10_11	48.51	-639.516	0.6554379	0	0	0	0.035246756
cov1_3_4_6_8_9_10_11	48.5241	-639.523	0.6542629	0	-0.062728006	0	0
cov1_2_3_4_5_6_9_10	48.5392	-639.531	0.6532143	0	0.069099633	0	0.020701791
cov1_2_3_4_5_6_9_11	48.5551	-639.539	0.6528482	0	0.070848358	0	0.021072013
cov1_2_3_4_5_6_10_11	48.5568	-639.54	0.65365	0	0.07054138	0	0.02054541
cov1_3_4_5_6_9_10_11	48.5576	-639.54	0.6537677	0	0.084681912	0	0
cov1_2_3_6_7_8_9_10	48.5618	-639.542	0.6533884	0	-0.067542601	0	0.10876216
cov1_2_3_6_7_8_9_11	48.5671	-639.545	0.6526302	0	-0.072852334	0	0.10849753

cov1_2_3_5_6_7_8_9	48.5728	-639.548	0.6529713	0	-0.074441124	0	0.10860634
cov1_2_3_6_7_8_10_11	48.5742	-639.548	0.6534388	0	-0.071802949	0	0.10791997
cov1_2_3_5_6_7_8_10	48.5801	-639.551	0.6537977	0	-0.07383421	0	0.10803
cov2_3_4_6_8_9_10_11	48.5828	-639.553	0.6552923	0	0	0	0.036411172
cov1_2_3_4_5_9_10_11	48.5969	-639.56	0.6531126	0	0.098589325	0	0.020908784
cov1_2_5_6_7_8_9_10	48.6106	-639.567	0.6537889	0	-0.095025582	0	0.10600515
cov1_2_5_6_7_8_9_11	48.6152	-639.569	0.6530614	0	-0.09896414	0	0.10577114
cov2_3_4_5_6_9_10_11	48.624	-639.573	0.6518079	0	0	0	0.041224597
cov1_2_5_6_7_8_10_11	48.6253	-639.574	0.6538889	0	-0.099150545	0	0.10507727
cov1_2_3_7_8_9_10_11	48.6319	-639.577	0.6529943	0	-0.039067258	0	0.11075285
cov1_2_3_5_7_8_9_10	48.6406	-639.582	0.6534578	0	-0.042785162	0	0.11074985
cov1_2_3_5_7_8_9_11	48.6408	-639.582	0.6525621	0	-0.049951301	0	0.11020885
cov1_2_3_5_7_8_10_11	48.65	-639.586	0.6533981	0	-0.048959622	0	0.10965549
cov1_2_6_7_8_9_10_11	48.6504	-639.586	0.6539484	0	-0.072514819	0	0.10699614
cov2_3_5_6_7_8_9_10	48.6659	-639.594	0.6545876	0	0	0	0.097575544
cov1_2_5_7_8_9_10_11	48.6714	-639.597	0.6533713	0	-0.070578579	0	0.10767902
cov2_3_5_6_7_8_9_11	48.6777	-639.6	0.6542064	0	0	0	0.097948189
cov2_3_5_6_7_8_10_11	48.6833	-639.603	0.6549495	0	0	0	0.097413874
cov2_3_5_7_8_9_10_11	48.6844	-639.604	0.6542339	0	0	0	0.10082652
cov1_2_3_6_7_9_10_11	48.71	-639.616	0.6529173	0	-0.070453737	0	0.10824271
cov1_2_3_5_6_7_9_10	48.7148	-639.619	0.6532669	0	-0.07443944	0	0.10821933
cov2_3_6_7_8_9_10_11	48.7163	-639.619	0.6552286	0	0	0	0.088347239
cov1_2_3_5_6_7_9_11	48.7201	-639.621	0.6524976	0	-0.078930352	0	0.10794039
cov1_2_3_5_6_7_10_11	48.7269	-639.625	0.6533106	0	-0.078536378	0	0.10730552
cov1_2_5_6_7_9_10_11	48.751	-639.637	0.653297	0	-0.098279453	0	0.10548725
cov1_2_3_5_7_9_10_11	48.7722	-639.647	0.6528453	0	-0.052149841	0	0.10966158
cov1_2_3_6_8_9_10_11	48.781	-639.652	0.6528382	0	-0.068965804	0	0.10838252
cov1_2_3_5_6_8_9_10	48.7866	-639.655	0.6531742	0	-0.069833394	0	0.10857169
cov1_2_3_5_6_8_9_11	48.7936	-639.658	0.6524364	0	-0.073823506	0	0.10835917
cov1_2_3_5_6_8_10_11	48.7999	-639.661	0.6532496	0	-0.073450213	0	0.10772116
cov2_5_6_7_8_9_10_11	48.81	-639.666	0.6561327	0	0	0	0.088961187
cov2_3_5_6_7_9_10_11	48.8192	-639.671	0.6544638	0	0	0	0.097521413
cov1_2_5_6_8_9_10_11	48.8434	-639.683	0.6533188	0	-0.096505523	0	0.10552255
cov1_2_3_5_8_9_10_11	48.8438	-639.683	0.6527875	0	-0.047576354	0	0.11003453
cov2_3_5_6_8_9_10_11	48.879	-639.701	0.6542957	0	0	0	0.098481966
cov1_2_3_4_5_6_7_8	49.2549	-639.889	0.6538677	0	0.079631891	0	0.03232588
cov1_2_4_5_6_7_8_11	49.2767	-639.9	0.653956	0	0.063438502	0	0.029905097
cov1_2_3_4_6_9_10_11	49.2876	-639.905	0.6516829	0	-0.066564884	0	0.072278663
cov1_3_4_5_6_7_8_11	49.2964	-639.91	0.6545854	0	0.10147017	0	0
cov2_3_4_5_6_7_8_11	49.3477	-639.935	0.6523007	0	0	0	0.053929471
cov1_2_3_5_6_9_10_11	49.3617	-639.942	0.6524362	0	-0.011829877	0	0.11311901
cov1_2_3_4_5_6_7_11	49.4051	-639.964	0.6535496	0	0.077381381	0	0.031950458
cov1_2_3_4_5_7_8_11	49.427	-639.975	0.6534029	0	0.12990203	0	0.032690473
cov3_5_6_7_8_9_10_11	49.4506	-639.987	0.6575682	0	0	0	0
cov1_3_5_6_7_8_10_11	49.4586	-639.991	0.6576215	0	-0.020762464	0	0
cov1_3_5_6_7_8_9_11	49.462	-639.992	0.6568503	0	-0.020491674	0	0
cov1_3_6_7_8_9_10_11	49.4628	-639.993	0.657446	0	0.001890165	0	0

cov1_5_6_7_8_9_10_11	49.4635	-639.993	0.6575632	0	-0.035490604	0	0
cov1_3_5_6_7_8_9_10	49.4642	-639.993	0.6578929	0	-0.012557744	0	0
cov1_2_3_4_5_6_8_11	49.4699	-639.996	0.6534996	0	0.083250411	0	0.03148757
cov1_3_5_7_8_9_10_11	49.5597	-640.041	0.6572823	0	0.016747654	0	0
cov1_3_5_6_7_9_10_11	49.5967	-640.06	0.6571596	0	-0.021305973	0	0
cov1_3_5_6_8_9_10_11	49.6768	-640.1	0.6570876	0	-0.015775887	0	0
cov1_2_4_5_6_9_10_11	49.8167	-640.17	0.6566461	0	0.052080462	0	-0.014168694
cov1_2_3_5_6_7_8_11	49.9694	-640.246	0.652757	0	-0.00788036	0	0.11836264
cov1_2_3_4_6_7_8_11	50.0387	-640.281	0.6525529	0	-0.075215204	0	0.086351049
cov2_3_4_5_6_7_8_9_10	55.1421	-639.228	0.6538836	0	0	0	0.029620163
cov2_3_4_5_6_7_8_9_11	55.1452	-639.229	0.653197	0	0	0	0.027876577
cov1_2_3_4_5_6_7_8_9	55.1531	-639.233	0.6536379	0	0.007707505	0	0.027815254
cov2_3_4_5_6_7_8_10_11	55.1534	-639.233	0.653993	0	0	0	0.027396171
cov1_2_3_4_5_6_7_8_10	55.1618	-639.238	0.6544507	0	0.008268523	0	0.027308635
cov3_4_5_6_7_8_9_10_11	55.173	-639.243	0.6540339	0	0	0	0
cov1_3_4_5_6_7_8_9_10	55.1739	-639.244	0.6547618	0	0.028457861	0	0
cov1_3_4_5_6_7_8_9_11	55.1766	-639.245	0.6539703	0	0.023256311	0	0
cov2_4_5_6_7_8_9_10_11	55.1776	-639.245	0.6543826	0	0	0	0.019520748
cov1_2_4_5_6_7_8_9_10	55.1817	-639.247	0.6544165	0	-0.010083081	0	0.024971388
cov1_3_4_5_6_7_8_10_11	55.1835	-639.248	0.6547552	0	0.023088689	0	0
cov1_2_4_5_6_7_8_9_11	55.1844	-639.249	0.6537123	0	-0.01413546	0	0.024564539
cov1_2_3_4_5_7_8_9_11	55.1932	-639.253	0.6531455	0	0.027770373	0	0.027394074
cov1_2_3_4_5_7_8_9_10	55.194	-639.254	0.6539852	0	0.034746648	0	0.028025211
cov2_3_4_5_7_8_9_10_11	55.1945	-639.254	0.6528749	0	0	0	0.035940679
cov1_2_4_5_6_7_8_10_11	55.1955	-639.254	0.6545181	0	-0.014336888	0	0.023922952
cov1_4_5_6_7_8_9_10_11	55.1991	-639.256	0.6546795	0	0.004389477	0	0
cov1_2_3_4_5_7_8_10_11	55.2034	-639.258	0.6539633	0	0.028684648	0	0.026820688
cov1_3_4_5_7_8_9_10_11	55.2124	-639.263	0.6541967	0	0.0475953	0	0
cov1_2_4_5_7_8_9_10_11	55.2166	-639.265	0.6539194	0	0.009594735	0	0.024660559
cov2_3_4_5_6_7_9_10_11	55.2884	-639.301	0.6534479	0	0	0	0.027518089
cov1_2_3_4_5_6_7_9_10	55.2971	-639.305	0.6538969	0	0.007807121	0	0.027595962
cov1_2_3_4_5_6_7_9_11	55.3003	-639.307	0.6531537	0	0.003249593	0	0.027150012
cov1_2_3_4_5_6_7_10_11	55.3086	-639.311	0.6539545	0	0.003602616	0	0.026583311
cov1_3_4_5_6_7_9_10_11	55.3192	-639.316	0.6542117	0	0.022797927	0	0
cov1_2_4_5_6_7_9_10_11	55.3232	-639.318	0.6539065	0	-0.013499253	0	0.024473567
cov1_2_3_4_5_7_9_10_11	55.3286	-639.321	0.6534124	0	0.025489524	0	0.027072046
cov2_3_4_5_6_8_9_10_11	55.3487	-639.331	0.6533047	0	0	0	0.028530351
cov1_2_3_4_5_6_8_9_10	55.3552	-639.334	0.6538174	0	0.012959868	0	0.027067582
cov1_2_4_6_7_8_9_10_11	55.3583	-639.336	0.6529927	0	-0.085489261	0	0.053589884
cov1_2_3_4_5_6_8_9_11	55.36	-639.337	0.6530975	0	0.008891662	0	0.02667272
cov1_2_3_4_6_7_8_9_10	55.3664	-639.34	0.6533811	0	-0.080378232	0	0.054483855
cov1_2_3_4_5_6_8_10_11	55.3679	-639.341	0.6538967	0	0.009207913	0	0.02611209
cov1_2_3_4_6_7_8_9_11	55.3765	-639.345	0.6525465	0	-0.085806639	0	0.054590475
cov1_3_4_5_6_8_9_10_11	55.3776	-639.345	0.6541482	0	0.028034021	0	0
cov1_2_3_4_6_7_8_10_11	55.3805	-639.347	0.6534458	0	-0.084696043	0	0.053676817
cov1_2_3_4_5_8_9_10_11	55.387	-639.35	0.6533534	0	0.030613538	0	0.026620149
cov1_2_4_5_6_8_9_10_11	55.3988	-639.356	0.6539524	0	-0.010845745	0	0.023447219

cov1_2_3_4_7_8_9_10_11	55.4029	-639.358	0.6529143	0	-0.060696799	0	0.053906729
cov1_3_4_6_7_8_9_10_11	55.5019	-639.408	0.6544316	0	-0.066433946	0	0
cov1_2_3_4_6_7_9_10_11	55.5059	-639.41	0.6528979	0	-0.083668098	0	0.053325242
cov2_3_4_6_7_8_9_10_11	55.581	-639.447	0.6555556	0	0	0	0.035356377
cov1_2_3_4_6_8_9_10_11	55.5874	-639.45	0.6528074	0	-0.081882498	0	0.054175965
cov1_2_3_4_5_6_9_10_11	55.7484	-639.531	0.6532357	0	0.069710018	0	0.020774657
cov1_2_3_6_7_8_9_10_11	55.7634	-639.538	0.6529982	0	-0.072490138	0	0.10812263
cov1_2_3_5_6_7_8_9_10	55.7688	-639.541	0.6533419	0	-0.074994302	0	0.1082322
cov1_2_3_5_6_7_8_9_11	55.7747	-639.544	0.6525827	0	-0.079262535	0	0.10801444
cov1_2_3_5_6_7_8_10_11	55.7814	-639.547	0.6533973	0	-0.078876339	0	0.10737785
cov1_2_5_6_7_8_9_10_11	55.8126	-639.563	0.653414	0	-0.099921219	0	0.10536692
cov1_2_3_5_7_8_9_10_11	55.8362	-639.575	0.6529311	0	-0.050680674	0	0.10980446
cov2_3_5_6_7_8_9_10_11	55.8752	-639.594	0.6545584	0	0	0	0.097508408
cov1_2_3_5_6_7_9_10_11	55.9158	-639.615	0.6528619	0	-0.079595669	0	0.10754938
cov1_2_3_5_6_8_9_10_11	55.989	-639.651	0.6528045	0	-0.074501399	0	0.1079637
cov1_2_3_4_5_6_7_8_11	56.4619	-639.888	0.6536539	0	0.077028441	0	0.031951998
cov1_3_5_6_7_8_9_10_11	56.651	-639.982	0.6572502	0	-0.021629003	0	0
cov2_3_4_5_6_7_8_9_10_11	62.3424	-639.223	0.6535583	0	0	0	0.027415742
cov1_2_3_4_5_6_7_8_9_10	62.3507	-639.227	0.6539867	0	0.007117086	0	0.027557411
cov1_2_3_4_5_6_7_8_9_11	62.3544	-639.229	0.6532489	0	0.002770557	0	0.027139178
cov1_2_3_4_5_6_7_8_10_11	62.3626	-639.233	0.6540495	0	0.003100234	0	0.026578123
cov1_3_4_5_6_7_8_9_10_11	62.3732	-639.239	0.6543142	0	0.022283795	0	0
cov1_2_4_5_6_7_8_9_10_11	62.3831	-639.243	0.6540385	0	-0.015125285	0	0.024251345
cov1_2_3_4_5_7_8_9_10_11	62.3903	-639.247	0.6534905	0	0.027000199	0	0.027101847
cov1_2_3_4_5_6_7_9_10_11	62.4976	-639.301	0.6534915	0	0.002536186	0	0.026851956
cov1_2_3_4_5_6_8_9_10_11	62.5571	-639.33	0.6534451	0	0.008151133	0	0.026382467
cov1_2_3_4_6_7_8_9_10_11	62.5676	-639.336	0.6529755	0	-0.085404609	0	0.053838447
cov1_2_3_5_6_7_8_9_10_11	62.9703	-639.537	0.6529501	0	-0.079959183	0	0.10758604
cov1_2_3_4_5_6_7_8_9_10_11	69.5517	-639.223	0.6535942	0	0.002027582	0	0.026834886

SE	bsnp_rs7817754	SE	bsnp_rs2047962	SE	bsnp_rs6469668	SE	bsnp_rs6469667	SE	bsnp_rs7832958
0	0	0	0	0	0	0	0	0	0
0	0	0	0	0	0	0	0	0	0
0	0	0	0	0	0	0	0	0	0
0	0	0	0	0	0	0	0	0	0
0	0	0	0.12134786	0	0	0	0	0	0
0	0	0	0	0	0	0	0.11351511	0	0
0	0	0	0	0	0	0	0	0	-0.19912259
0	0	0	0	0	0	0	0	0	0
0	0	0	0	0	0	0	0	0	0
0	0	0	0	0	0.07880759	0	0	0	0
0	0	0	0	0	0	0	0	0	0
0	-0.065478699	0	0	0	0	0	0	0	0
0	0	0	0	0	0	0	0	0	-0.18952228
0	0	0	0	0	0	0	0	0	-0.17818961
0	0	0	0	0	0	0	0	0	0
0	0	0	0	0	0	0	0	0	-0.17852715
0	0	0	0	0	0	0	0	0	0
0	0	0	0.10304511	0	0	0	0	0	0
0	0	0	0.10177147	0	0	0	0	0	0
0	0	0	0	0	0	0	0	0	0
0	0	0	0	0	0	0	0.10589231	0	-0.18508054
0	0	0	0.10853588	0	0	0	0	0	-0.174822
0	0	0	0.10881085	0	0	0	0	0	0
0	0	0	0	0	0	0	0.1058177	0	0
0	-0.11106046	0	0	0	0	0	0	0	0
0	0	0	0	0	0	0	0	0	0
0	0	0	0	0	0	0	0	0	0
0	-0.11207696	0	0	0	0	0	0	0	0
0	0	0	0	0	0	0	0	0	-0.18660413
0	0	0	0	0	0	0	0	0	0
0	0	0	0	0	0	0	0.068693155	0	0
0	0	0	0	0	0	0	0.070154891	0	0
0	-0.092320752	0	0	0	0	0	0	0	0
0	0	0	0	0	0.059362929	0	0	0	0
0	0	0	0	0	0.061172517	0	0	0	0
0	0	0	0	0	0	0	0	0	0
0	0	0	0	0	0	0	0	0	0
0	0	0	0	0	0	0	0	0	-0.21758932
0	0	0	0	0	0	0	0	0	0
0	0	0	0	0	0.075686758	0	0	0	-0.19506792
0	0	0	0	0	0.075790467	0	0	0	0
0	0	0	0	0	0	0	0	0	0
0	0	0	0	0	0	0	0	0	0
0	-0.08857597	0	0	0	0	0	0.1227199	0	0
0	0	0	0	0	0	0	0	0	0

0	0	0	0.054160708	0	0	0	0	0	0
0	0	0	0.20244427	0	-0.094142727	0	0	0	0
0	0	0	0	0	0	0	0.040662124	0	0
0	0	0	0.07880823	0	0	0	0.060336303	0	0
0	0	0	0	0	-0.031371418	0	0	0	0
0	0	0	0	0	0	0	0	0	0
0	-0.087152721	0	0	0	0	0	0	0	0
0	0	0	0	0	0	0	0	0	0
0	-0.04319391	0	0.11663237	0	0	0	0	0	0
0	0	0	0.089054311	0	0	0	0	0	0
0	0	0	0.11923221	0	0	0	0	0	0
0	0.071676354	0	0	0	0	0	0	0	-0.26386767
0	0.067969439	0	0	0	0	0	0	0	0
0	0	0	0	0	-0.030366226	0	0.1369026	0	0
0	0	0	0	0	0	0	0.13184286	0	0
0	0	0	0	0	0	0	0	0	-0.62645399
0	0	0	0	0	0	0	0.095160856	0	0
0	0	0	0	0	-0.062531634	0	0	0	0
0	0	0	0	0	0	0	0	0	0
0	-0.0922658	0	0	0	0	0	0	0	0
0	-0.056931203	0	0	0	0.075171868	0	0	0	0
0	0	0	0.092353609	0	0	0	0	0	-0.16009011
0	0	0	0.091159754	0	0	0	0	0	-0.16021161
0	0	0	0.092632625	0	0	0	0	0	0
0	0	0	0.091438609	0	0	0	0	0	0
0	0	0	0	0	0.05734763	0	0	0	0
0	0	0	0	0	0	0	0	0	-0.1773854
0	0	0	0	0	0	0	0	0	-0.17753693
0	0	0	0	0	0	0	0	0	0
0	0	0	0	0	0	0	0	0	0
0	-0.11968738	0	0	0	0	0	0	0	0
0	-0.089493165	0	0.090664647	0	0	0	0	0	0
0	-0.11865273	0	0	0	0	0	0	0	0
0	-0.088691858	0	0.089352769	0	0	0	0	0	0
0	0	0	0	0	0	0	0.065171573	0	-0.17479138
0	0	0	0	0	0	0	0.066480092	0	-0.17487103
0	0	0	0	0	0.058017127	0	0	0	-0.17664238
0	0	0	0	0	0.059659928	0	0	0	-0.17676042
0	0	0	0	0	0	0	0.06504333	0	0
0	0	0	0	0	0	0	0.066355169	0	0
0	0	0	0	0	0	0	0	0	-0.1752528
0	0	0	0	0	0	0	0	0	-0.17534789
0	0	0	0	0	0.058110339	0	0	0	0
0	0	0	0	0	0.059753961	0	0	0	0
0	0	0	0	0	0	0	0	0	0
0	0	0	0	0	0	0	0	0	0
0	0	0	0	0	0	0	0	0	-0.19230221

0	0	0	0	0	0	0	0	0	-0.19287183
0	0	0	0.1868977	0	-0.099101883	0	0	0	0
0	0	0	0.18631057	0	-0.096840845	0	0	0	0
0	0	0	0	0	0	0	0	0	0
0	0	0	0	0	0	0	0	0	0
0	0	0	0	0	0	0	0	0	-0.62644739
0	0	0	0	0	-0.030675119	0	0	0	-0.18939468
0	-0.11569845	0	0	0	0	0	0.073875539	0	0
0	0	0	0	0	0	0	0.029744557	0	-0.18749585
0	-0.11463199	0	0	0	0	0	0.072441872	0	0
0	0	0	0	0	0	0	0	0	-0.6264962
0	0	0	0.025838758	0	0	0	0	0	-0.18536087
0	0.019213306	0	0	0	0	0	0	0	-0.20708414
0	0	0	0	0	-0.030583762	0	0	0	0
0	0	0	0.12085799	0	0	0	0	0	0
0	0	0	0.11987571	0	0	0	0	0	0
0	0	0	0	0	0	0	0	0	-0.62651018
0	0	0	0	0	0	0	0	0	-0.19093406
0	0	0	0	0	0	0	0	0	-0.18970475
0	0	0	0	0	0	0	0.029365556	0	0
0	0	0	0.13037632	0	0	0	0	0	0
0	0	0	0.12982482	0	0	0	0	0	0
0	0	0	0.026470461	0	0	0	0	0	0
0	-0.11591656	0	0	0	0	0	0	0	0
0	-0.020554159	0	0	0	0	0	0	0	-0.15883585
0	-0.11481324	0	0	0	0	0	0	0	0
0	0	0	0	0	0	0	0	0	-0.17815669
0	0.015622632	0	0	0	0	0	0	0	0
0	0	0	0	0	0	0	0	0	0
0	0	0	0	0	0	0	0	0	0
0	-0.021681982	0	0	0	0	0	0	0	-0.1581028
0	-0.024602119	0	0	0	0	0	0	0	0
0	0	0	0.11326692	0	0	0	-0.015696938	0	0
0	0	0	0.11261578	0	0	0	-0.016766993	0	0
0	0	0	0.10233803	0	0	0	0	0	0
0	0	0	0	0	0	0	0	0	0
0	0	0	0.10460852	0	0	0	0	0	0
0	0	0	0.10294938	0	0	0	0	0	0
0	-0.02576805	0	0	0	0	0	0	0	0
0	0.087435654	0	0.11233106	0	0	0	0	0	-0.25283145
0	0	0	0.063881802	0	0	0	0.063142532	0	-0.17645527
0	0	0	0.064534722	0	0	0	0.062644204	0	0
0	0.083946026	0	0.11251071	0	0	0	0	0	0
0	-0.10369479	0	0	0	0.052073852	0	0	0	0
0	-0.10286966	0	0	0	0.05013721	0	0	0	0
0	-0.12382076	0	0	0	0	0	0	0	0
0	-0.12235193	0	0	0	0	0	0	0	0

0	0	0	0.071881595	0	0	0	0	0	-0.17691928
0	0	0	0.16498703	0	-0.064763326	0	0	0	-0.16555821
0	0	0	0.072270446	0	0	0	0	0	0
0	0	0	0.16570219	0	-0.065276528	0	0	0	0
0	0	0	0.088234167	0	0	0	0	0	-0.18680191
0	0	0	0	0	0	0	0.10641833	0	-0.62665368
0	0	0	0.10854584	0	0	0	0	0	-0.62633652
0	0	0	0.088891343	0	0	0	0	0	0
0	0	0	0	0	-0.022623882	0	0.12335693	0	-0.18398835
0	0.022400751	0	0	0	0	0	0.10275452	0	-0.20570762
0	0	0	0	0	0	0	0.0960246	0	-0.18934013
0	0	0	0	0	0	0	0.088008574	0	-0.18498045
0	0	0	0	0	0	0	0	0	0
0	0	0	0	0	-0.022174645	0	0.12294102	0	0
0	0	0	0	0	0	0	0	0	0
0	0.018419526	0	0	0	0	0	0.10322048	0	0
0	0	0	0	0	0	0	0.096299141	0	0
0	0	0	0	0	0	0	0.086773742	0	0
0	0	0	0.20824525	0	-0.24703768	0	0	0	0
0	0	0	0	0	0	0	0	0	0
0	-0.11175102	0	0	0	0	0	0	0	0
0	0	0	0	0	-0.049827487	0	0	0	-0.18397633
0	0	0	0	0	0	0	0	0	0
0	0	0	0	0	0	0	0	0	0
0	0	0	0	0	0	0	0	0	-0.62642278
0	0	0	0	0	0	0	0.004445999	0	0
0	0	0	0	0	-0.003795614	0	0	0	0
0	0	0	0	0	0	0	0.006031885	0	0
0	0	0	0	0	-0.002057527	0	0	0	0
0	0	0	0	0	-0.049895504	0	0	0	0
0	0.026992318	0	0	0	0	0	0	0	-0.21144036
0	0	0	0.19327846	0	-0.18813411	0	0.12777021	0	0
0	0	0	0	0	0	0	0	0	-0.1917913
0	0.023335466	0	0	0	0	0	0	0	0
0	0	0	0	0	0	0	0	0	0
0	-0.10947488	0	0	0	-0.066013412	0	0	0	0
0	-0.093779384	0	0	0	0	0	0.044925504	0	0
0	0	0	0	0	0	0	0.084385302	0	0
0	0	0	0	0	0.024009638	0	0.047427364	0	0
0	0	0	0	0	0	0	0.085604271	0	0
0	0	0	0	0	0.025377566	0	0.047701788	0	0
0	0	0	0	0	0	0	0.068931798	0	0
0	0	0	0	0	0	0	0.057965187	0	0
0	0	0	0	0	0	0	0.05867645	0	0
0	0	0	0	0	0.033162511	0	0	0	-0.21096968
0	0.084730994	0	0	0	0.079855733	0	0	0	-0.27131406
0	-0.090711478	0	0	0	0	0	0	0	0

0	-0.092546631	0	0	0	0	0	0	0	0
0	0.041978885	0	0	0	0	0	0	0	-0.25449292
0	0	0	0	0	0	0	0	0	0
0	0	0	0	0	0	0	0	0	-0.62615937
0	-0.091484517	0	0.002372151	0	0	0	0	0	0
0	0	0	0	0	0.035530949	0	0	0	0
0	0	0	0	0	0.037323564	0	0	0	0
0	0	0	0	0	0.068034068	0	0	0	0
0	0	0	0	0	0	0	0	0	0
0	0	0	0	0	0.060095279	0	0	0	0
0	0	0	0	0	0.069771651	0	0	0	0
0	0	0	0	0	0	0	0	0	0
0	0	0	0	0	0.03369093	0	0	0	0
0	0.081072758	0	0	0	0.079810053	0	0	0	0
0	0.038280153	0	0	0	0	0	0	0	0
0	0	0	0.13833673	0	-0.11097845	0	0	0	0
0	-0.1249678	0	0	0	-0.13901403	0	0	0	0
0	-0.10668932	0	0	0	-0.069935715	0	0.1783342	0	0
0	0	0	0	0	0.075705318	0	0	0	-0.6264222
0	-0.071140849	0	0.054167671	0	0	0	0.084411092	0	0
0	0	0	0	0	0	0	0	0	0
0	0	0	0	0	-0.065388636	0	0.075281504	0	0
0	-0.088973579	0	0	0	0	0	0.10013555	0	0
0	-0.038524762	0	0.1946081	0	-0.089919419	0	0	0	0
0	-0.087141533	0	0	0	0	0	0.12817381	0	0
0	0	0	0	0	0	0	0.064657175	0	0
0	0	0	0.20367393	0	-0.11888356	0	0	0	0
0	-0.066622281	0	0.062253405	0	0	0	0	0	0
0	0	0	0.048842601	0	0	0	0.033773572	0	0
0	0	0	0.05609242	0	0	0	0	0	0
0	0	0	0.083524183	0	0	0	0.083522234	0	0
0	0	0	0	0	0	0	0.080371236	0	0
0	0	0	0	0	-0.06597733	0	0	0	0
0	0	0	0.054522169	0	0	0	0	0	0
0	0	0	0.085139946	0	0	0	0.084739615	0	0
0	0	0	0	0	0	0	0	0	0
0	0	0	0	0	-0.024595922	0	0	0	0
0	-0.085458351	0	0	0	0	0	0	0	0
0	-0.052602798	0	0.10320845	0	0	0	0	0	0
0	0	0	0.089328699	0	0	0	0	0	0
0	0.071033455	0	0	0	0	0	0	0	-0.627443
0	0	0	0	0	-0.059957302	0	0.092756848	0	0
0	0	0	0	0	0	0	0.1002115	0	0
0	0	0	0	0	-0.024745313	0	0.14712899	0	0
0	0	0	0	0	-0.061626876	0	0	0	0
0	-0.081928177	0	0	0	0.030675477	0	0	0	0
0	0	0	0.15385749	0	-0.072207161	0	0	0	-0.14968722

0	0	0	0.15325354	0	-0.07007212	0	0	0	-0.1499262
0	0	0	0.15462722	0	-0.072779086	0	0	0	0
0	0	0	0.15401714	0	-0.070637003	0	0	0	0
0	0	0	0.092300446	0	0	0	0	0	-0.62615454
0	0	0	0.091129246	0	0	0	0	0	-0.62638628
0	0	0	0.11115568	0	0	0	0	0	-0.15769612
0	0	0	0.11065071	0	0	0	0	0	-0.15769487
0	0	0	0.072737222	0	0	0	0	0	-0.16373587
0	0	0	0.07127299	0	0	0	0	0	-0.16391728
0	0	0	0.091684028	0	0	0	0	0	-0.16003147
0	0	0	0.096417894	0	0	0	-0.006207822	0	-0.15963513
0	0.005661388	0	0.092787899	0	0	0	0	0	-0.16533572
0	0	0	0.093987193	0	0	0	0	0	-0.15896413
0	0	0	0.095830418	0	0	0	-0.007169754	0	-0.15965239
0	0.006955928	0	0.091708984	0	0	0	0	0	-0.16665117
0	0	0	0.09315538	0	0	0	0	0	-0.15882056
0	0	0	0.11158081	0	0	0	0	0	0
0	0	0	0.1110886	0	0	0	0	0	0
0	0	0	0.073470596	0	0	0	0	0	0
0	0	0	0.07200344	0	0	0	0	0	0
0	0	0	0.091967672	0	0	0	0	0	0
0	0	0	0.097115477	0	0	0	-0.006834874	0	0
0	0	0	0.094661452	0	0	0	0	0	0
0	0.001646961	0	0.092760073	0	0	0	0	0	0
0	0	0	0.09649515	0	0	0	-0.007768634	0	0
0	0	0	0.093822951	0	0	0	0	0	0
0	0.002956903	0	0.091673988	0	0	0	0	0	0
0	0	0	0	0	0	0	0	0	-0.62617491
0	0	0	0	0	0	0	0	0	-0.62649535
0	-0.036813738	0	0	0	0	0	0	0	-0.14269583
0	-0.038326408	0	0	0	0	0	0	0	-0.14138064
0	0	0	0	0	0	0	0	0	-0.17871808
0	0	0	0	0	0	0	0	0	-0.17880716
0	-0.040744724	0	0	0	0	0	0	0	0
0	-0.042281975	0	0	0	0	0	0	0	0
0	0	0	0	0	0	0	0	0	-0.17734353
0	0	0	0	0	-0.006183451	0	0	0	-0.1775109
0	0	0	0	0	0	0	0	0	-0.17625887
0	0	0	0	0	0	0	-0.002728699	0	-0.17751332
0	0	0	0	0	-0.004609881	0	0	0	-0.17761814
0	0	0	0	0	0	0	0	0	-0.17664036
0	0	0	0	0	0	0	0	0	0
0	0	0	0	0	0	0	-0.00128783	0	-0.17756598
0	0	0	0	0	0	0	0	0	0
0	0	0	0	0	0	0	0	0	0
0	0	0	0	0	-0.006074146	0	0	0	0
0	0	0	0	0	0	0	0	0	0

0	0	0	0	0	0	0	-0.003117674	0	0
0	0	0	0	0	-0.004497789	0	0	0	0
0	0	0	0	0	0	0	0	0	0
0	0	0	0	0	0	0	-0.001686907	0	0
0	-0.083891295	0	0.16764675	0	-0.090355356	0	0	0	0
0	-0.0846827	0	0.16676832	0	-0.087741998	0	0	0	0
0	-0.10415585	0	0.048699411	0	0	0	0	0	0
0	-0.12771971	0	0	0	-0.039787212	0	0	0	0
0	-0.12819117	0	0	0	-0.037804442	0	0	0	0
0	-0.10350288	0	0.047208815	0	0	0	0	0	0
0	0	0	0	0	0	0	0.065669952	0	-0.6266241
0	0	0	0	0	0	0	0.066941161	0	-0.62643226
0	-0.11989694	0	0	0	0	0	0	0	0
0	-0.11888927	0	0	0	0	0	0	0	0
0	-0.11753753	0	0	0	0	0	0	0	0
0	-0.11947178	0	0	0	0	0	0	0	0
0	0	0	0.17313163	0	-0.20534792	0	0	0	-0.1522379
0	-0.089501738	0	0.089957071	0	0	0	0	0	0
0	-0.11629904	0	0	0	0	0	0	0	0
0	-0.086984499	0	0.098345056	0	0	0	0	0	0
0	-0.091504394	0	0.084424962	0	0	0	0.009161509	0	0
0	-0.11965398	0	0	0	0	0	0.004399632	0	0
0	-0.086771841	0	0.094873666	0	0	0	0	0	0
0	0	0	0	0	0.05798216	0	0	0	-0.62649246
0	0	0	0	0	0.059628879	0	0	0	-0.62649371
0	-0.1186025	0	0	0	0	0	0.002817841	0	0
0	-0.085846822	0	0.098022914	0	0	0	0	0	0
0	0	0	0	0	0.027757257	0	0.040585641	0	-0.17533195
0	0	0	0	0	0.029085124	0	0.040738155	0	-0.17542435
0	-0.08558062	0	0.094158946	0	0	0	0	0	0
0	-0.031825184	0	0	0	0	0	0.066806435	0	-0.1447451
0	-0.033464218	0	0	0	0	0	0.068209329	0	-0.14325894
0	-0.090455827	0	0.083919357	0	0	0	0.008026952	0	0
0	0	0	0.17388631	0	-0.20611407	0	0	0	0
0	0	0	0	0	0	0	0.053909642	0	-0.1796278
0	0	0	0	0	0	0	0.054914877	0	-0.17982819
0	0	0	0	0	0	0	0	0	-0.62648236
0	0	0	0.15879532	0	-0.1534897	0	0.11802221	0	-0.15612624
0	0	0	0	0	0	0	0	0	-0.62647906
0	0	0	0	0	0	0	0.06539774	0	-0.17473132
0	0	0	0	0	0	0	0.054214009	0	-0.17476585
0	0	0	0	0	0	0	0.054828522	0	-0.1748415
0	0	0	0	0	0.045344758	0	0	0	-0.18241181
0	0	0	0	0	0.046799495	0	0	0	-0.18257856
0	-0.037744976	0	0	0	0	0	0.06834629	0	0
0	-0.036080574	0	0	0	0	0	0.066936095	0	0
0	0	0	0	0	0.028320706	0	0.039959204	0	0

0	0	0	0	0	0.029647631	0	0.040113662	0	0
0	0	0	0	0	0.058695026	0	0	0	-0.17655652
0	0	0	0	0	0.04165501	0	0	0	-0.17611804
0	0	0	0	0	0.043462463	0	0	0	-0.17623873
0	-0.001768739	0	0	0	0.057869702	0	0	0	-0.17496944
0	-0.002862245	0	0	0	0.059430551	0	0	0	-0.17407077
0	-0.031317818	0	0	0	0	0	0	0	-0.14567369
0	-0.032997478	0	0	0	0	0	0	0	-0.14416831
0	0	0	0.15966558	0	-0.15385367	0	0.1176809	0	0
0	0	0	0	0	0	0	0.054137306	0	0
0	0	0	0	0	0	0	0.055146175	0	0
0	0	0	0	0	0	0	0.065240954	0	0
0	0	0	0	0	0	0	0.052999057	0	0
0	0	0	0	0	0	0	0	0	-0.18100943
0	0	0	0	0	0	0	0.053620285	0	0
0	0	0	0	0	0.045894585	0	0	0	0
0	0	0	0	0	0.047352609	0	0	0	0
0	0	0	0	0	0	0	0	0	-0.18119864
0	0	0	0	0	0	0	0	0	-0.17519193
0	0	0	0	0	0.058787075	0	0	0	0
0	0	0	0	0	0.041585865	0	0	0	0
0	0	0	0	0	0.043371182	0	0	0	0
0	-0.035284233	0	0	0	0	0	0	0	0
0	-0.036988147	0	0	0	0	0	0	0	0
0	-0.005950865	0	0	0	0.057613493	0	0	0	0
0	-0.007047479	0	0	0	0.059186319	0	0	0	0
0	0	0	0	0	0	0	0	0	-0.62641039
0	0	0	0	0	0	0	0	0	-0.62656309
0	0	0	0	0	0	0	0	0	0
0	0	0	0	0	0	0	0	0	0
0	0	0	0	0	0	0	0	0	0
0	0	0	0.18584131	0	-0.13033246	0	0.043122085	0	0
0	0	0	0.18531127	0	-0.12875109	0	0.043665528	0	0
0	0	0	0.19086266	0	-0.14584576	0	0	0	0
0	0	0	0.19036723	0	-0.14410834	0	0	0	0
0	-0.028762061	0	0	0	0	0	0	0	-0.16555952
0	-0.030378414	0	0	0	0	0	0	0	-0.16462679
0	0	0	0	0	0	0	0	0	-0.19232887
0	0	0	0.18656856	0	-0.098245502	0	0	0	0
0	0	0	0.17826005	0	-0.10138039	0	0	0	0
0	-0.03283892	0	0	0	0	0	0	0	0
0	0	0	0.18641827	0	-0.0938412	0	0	0	0
0	0	0	0.089780464	0	-0.082273128	0	0	0	-0.17466834
0	-0.034474218	0	0	0	0	0	0	0	0
0	0	0	0.17792148	0	-0.099106065	0	0	0	0
0	0	0	0.18582503	0	-0.091726516	0	0	0	0
0	0	0	0	0	0	0	0	0	0

0	0	0	0.090808886	0	-0.082786169	0	0	0	0
0	0	0	0	0	-0.058284599	0	0.060786977	0	-0.18518813
0	-0.10565141	0	0.18903489	0	-0.29468816	0	0	0	0
0	0	0	0	0	-0.057944834	0	0.060214789	0	0
0	0	0	0	0	-0.030801957	0	0	0	-0.62631951
0	0	0	0	0	0	0	0.072580042	0	-0.18829239
0	0	0	0	0	0	0	0.030883011	0	-0.62650103
0	0	0	0.02579525	0	0	0	0	0	-0.62642101
0	0	0	0	0	-0.05346614	0	0	0	-0.18697209
0	0.042098919	0	0.044068508	0	0	0	0	0	-0.22087421
0	0.0184934	0	0	0	0	0	0	0	-0.62690606
0	0	0	0	0	-0.037397631	0	0	0	-0.19411194
0	0	0	0	0	0	0	0.071324363	0	0
0	0	0	0	0	0	0	0	0	-0.6264577
0	0	0	0	0	0	0	0	0	-0.6263056
0	0	0	0.021750679	0	0	0	0.026860693	0	-0.18418583
0	0.004115706	0	0	0	-0.029369156	0	0	0	-0.19316329
0	-0.11549514	0	0	0	0	0	0.072861281	0	0
0	-0.11852976	0	0	0	-0.013870667	0	0.086226924	0	0
0	-0.11590161	0	0	0	0	0	0.058299032	0	0
0	0.015365886	0	0	0	0	0	0.028224428	0	-0.20164258
0	-0.021636196	0	0	0	0	0	0	0	-0.6267987
0	-0.11637917	0	0	0	0	0	0.071400656	0	0
0	0	0	0	0	0	0	0.033665498	0	-0.18518919
0	0	0	0	0	-0.053523792	0	0	0	0
0	-0.11781179	0	0	0	-0.015366765	0	0.086139167	0	0
0	-0.11481333	0	0	0	0	0	0.057745016	0	0
0	0	0	0	0	0	0	0	0	-0.62650544
0	-0.11516548	0	0	0	0	0	0.070492873	0	0
0	0	0	0.027136622	0	0	0	0	0	-0.18561325
0	0	0	0.025454288	0	0	0	0	0	-0.18631651
0	0	0	0	0	-0.037027702	0	0	0	0
0	0.037873113	0	0.042918513	0	0	0	0	0	0
0	-0.022771253	0	0	0	0	0	0	0	-0.62679877
0	0	0	0.12986499	0	0	0	0	0	0
0	0	0	0.13040594	0	0	0	0	0	0
0	0	0	0.12038341	0	0	0	0	0	0
0	0	0	0.10841124	0	0	0	0	0	0
0	0	0	0.10689914	0	0	0	0	0	0
0	0	0	0.022470301	0	0	0	0.026396488	0	0
0	0	0	0.11571336	0	0	0	0	0	0
0	0	0	0.12779422	0	0	0	0.033627911	0	0
0	0	0	0.1169025	0	0	0	0	0	0
0	0.018882366	0	0	0	0	0	0	0	-0.20799213
0	0.019688816	0	0	0	0	0	0	0	-0.20780334
0	0	0	0.12729099	0	0	0	0.032957024	0	0
0	0	0	0.1177854	0	0	0	0.007042477	0	0

0	-0.1243571	0	0	0	-0.03935538	0	0	0	0
0	0	0	0.11731389	0	0	0	0.006443576	0	0
0	-4.74E-05	0	0	0	-0.030598955	0	0	0	0
0	-0.12373526	0	0	0	-0.041173942	0	0	0	0
0	0	0	0.13049362	0	0	0	0	0	0
0	0	0	0	0	0	0	0	0	-0.19245468
0	0.011689845	0	0	0	0	0	0.028175009	0	0
0	0	0	0	0	0	0	0.033586737	0	0
0	-0.11568954	0	0	0	0	0	0	0	0
0	0	0	0.027750638	0	0	0	0	0	0
0	0	0	0.026157321	0	0	0	0	0	0
0	0.084839464	0	0.16493063	0	-0.060488277	0	0	0	-0.24185925
0	-0.021487087	0	0	0	0	0	0	0	-0.15791891
0	-0.11655187	0	0	0	0	0	0	0	0
0	0	0	0.16358862	0	-0.10494935	0	0	0	-0.18132426
0	-0.11532275	0	0	0	0	0	0	0	0
0	0.062164104	0	0.080657679	0	0	0	0.043168357	0	-0.23142378
0	0.015317111	0	0	0	0	0	0	0	0
0	0.016039867	0	0	0	0	0	0	0	0
0	0	0	0	0	0	0	0	0	0
0	-0.025576547	0	0	0	0	0	0	0	0
0	0	0	0.11328446	0	0	0	-0.016919555	0	0
0	0	0	0.10926888	0	0	0	-0.018264156	0	0
0	0	0	0.16437525	0	-0.10512476	0	0	0	0
0	0	0	0.10798188	0	0	0	-0.019762287	0	0
0	0	0	0.10429887	0	0	0	0	0	0
0	0.081372831	0	0.16577713	0	-0.061259551	0	0	0	0
0	0.086808493	0	0.1123484	0	0	0	0	0	-0.62720964
0	0	0	0.063310106	0	0	0	0.064044716	0	-0.6264955
0	0.058235984	0	0.080387273	0	0	0	0.043783261	0	0
0	-0.086026724	0	0.1735034	0	-0.2040494	0	0.1622495	0	0
0	0.079356546	0	0.10144373	0	0	0	0	0	-0.25185536
0	0.074873174	0	0.095327348	0	0	0	0	0	-0.24258992
0	0	0	0.068347819	0	0	0	0.080116546	0	-0.17595266
0	0	0	0.063480615	0	0	0	0.061522191	0	-0.17732261
0	-0.11231339	0	0	0	0.035041037	0	0	0	0
0	-0.11133831	0	0	0	0.033443539	0	0	0	0
0	0.07067058	0	0.09447409	0	0	0	0	0	0
0	0.075820249	0	0.10160061	0	0	0	0	0	0
0	-0.10365887	0	0	0	0.051010138	0	0	0	0
0	0	0	0.068817499	0	0	0	0.078889893	0	0
0	0	0	0.064221491	0	0	0	0.061395749	0	0
0	0	0	0.071819679	0	0	0	0	0	-0.62608784
0	-0.1233359	0	0	0	0	0	0	0	0
0	0	0	0.1651496	0	-0.064917236	0	0	0	-0.62617792
0	-0.12727445	0	0	0	-0.12126463	0	0.11031692	0	0
0	0	0	0.088280454	0	0	0	0	0	-0.62618844

0	0	0	0.071349695	0	0	0	0	0	-0.18117414
0	0	0	0.071783986	0	0	0	0	0	0
0	0	0	0	0	-0.023691512	0	0.12479342	0	-0.62673065
0	-0.13312647	0	0	0	-0.14793546	0	0	0	0
0	0.021417749	0	0	0	0	0	0.10350505	0	-0.62759588
0	0	0	0	0	0	0	0.097112428	0	-0.62642153
0	0	0	0	0	0	0	0.090580833	0	-0.62663109
0	0	0	0	0	-0.047571147	0	0.086211533	0	-0.18250913
0	0	0	0	0	-0.027741098	0	0.11311204	0	-0.1898922
0	0	0	0	0	-0.047686107	0	0.084986211	0	0
0	0	0	0.20189618	0	-0.23968939	0	0.060798413	0	0
0	0.012490036	0	0	0	-0.017484216	0	0.11764361	0	-0.19573987
0	0.022093851	0	0	0	0	0	0.093158153	0	-0.20959967
0	0.021840887	0	0	0	0	0	0.085923181	0	-0.2051119
0	0	0	0	0	0	0	0.086341099	0	-0.1883339
0	0	0	0	0	0	0	0.025991135	0	0
0	0	0	0	0	-0.027110218	0	0.11301057	0	0
0	0	0	0	0	0	0	0.027397707	0	0
0	0	0	0.20795242	0	-0.24586932	0	0	0	0
0	0	0	0	0	0.011760359	0	0	0	0
0	0	0	0	0	-0.050135412	0	0	0	-0.62629858
0	0	0	0	0	0	0	0	0	0
0	0.00761215	0	0	0	-0.01901882	0	0.11943259	0	0
0	0	0	0	0	0	0	0	0	0
0	0	0	0	0	0.013409854	0	0	0	0
0	0.018134975	0	0	0	0	0	0.093920454	0	0
0	0.017893757	0	0	0	0	0	0.084928884	0	0
0	0	0	0	0	0	0	0.085230037	0	0
0	0	0	0	0	0	0	0	0	0
0	0	0	0	0	0	0	0.045530843	0	0
0	0	0	0	0	0	0	0.046384919	0	0
0	0	0	0.21748197	0	-0.25485338	0	0	0	0
0	0	0	0	0	0.026726863	0	0	0	0
0	0	0	0	0	-0.050008831	0	0	0	-0.18926013
0	0	0	0	0	0.028232229	0	0	0	0
0	0.026103832	0	0	0	0	0	0	0	-0.62659036
0	0	0	0	0	0	0	0	0	0
0	-0.004358879	0	0	0	-0.052769972	0	0	0	-0.17980893
0	0	0	0	0	0	0	0	0	-0.62609291
0	-0.090527107	0	0.086290059	0	-0.10974023	0	0	0	0
0	0	0	0	0	0	0	0.004772114	0	0
0	0	0	0	0	-0.002896954	0	0	0	0
0	0	0	0	0	-0.009265186	0	0.010319012	0	0
0	0	0	0	0	-0.007994788	0	0.01109554	0	0
0	0	0	0.17418956	0	-0.18195046	0	0.11207529	0	0
0	0	0	0	0	-0.05006766	0	0	0	0
0	0.026766508	0	0	0	0	0	0	0	-0.21633843

0	-0.010256404	0	0	0	-0.056826155	0	0	0	0
0	0	0	0.19237266	0	-0.18389963	0	0.13418432	0	0
0	0.023085834	0	0	0	0	0	0	0	0
0	-0.11552763	0	0	0	-0.07541661	0	0	0	0
0	-0.093870016	0	0	0	0	0	0.08486439	0	0
0	-0.088735365	0	0	0	0	0	0.059703554	0	0
0	0.062249754	0	0	0	0.047334274	0	0	0	-0.26285083
0	-0.12624559	0	0	0	-0.13707981	0	0.096781057	0	0
0	0	0	0	0	0.032480924	0	0.060356928	0	0
0	0	0	0	0	0.033819211	0	0.060587184	0	0
0	-0.096539119	0	-0.007710943	0	0	0	0.046131615	0	0
0	0	0	0	0	0	0	0.062426726	0	0
0	0	0	0	0	0	0	0.063105602	0	0
0	0.058468754	0	0	0	0.047061891	0	0	0	0
0	0	0	0	0	0.033199145	0	0	0	-0.62652407
0	0	0	0	0	0	0	0.084481707	0	0
0	0	0	0	0	0.025210611	0	0.046647857	0	0
0	0.083774924	0	0	0	0.079805237	0	0	0	-0.62677224
0	0	0	0	0	0.032389597	0	0.055957163	0	0
0	0	0	0	0	0.034122107	0	0.056560424	0	0
0	0.041325089	0	0	0	0	0	0	0	-0.627371
0	0	0	0	0	0	0	0.057928174	0	0
0	0	0	0	0	0.035859449	0	0	0	0
0	0	0	0	0	0.037635479	0	0	0	0
0	-0.089604766	0	0	0	0	0	0	0	0
0	0	0	0	0	0.037452766	0	0	0	0
0	0	0	0	0	0	0	0	0	0
0	0	0	0	0	0.068911445	0	0	0	0
0	-0.089076307	0	0.004379903	0	0	0	0	0	0
0	-0.092577881	0	-8.62E-05	0	0	0	0	0	0
0	0	0	0.13988226	0	-0.11339095	0	0	0	0
0	-0.12540281	0	0	0	-0.13934481	0	0	0	0
0	-0.11031325	0	0	0	-0.074841658	0	0.1732898	0	0
0	-0.062041924	0	0.19194519	0	-0.12896464	0	0	0	0
0	-0.066087456	0	0.058485543	0	0	0	0.095288676	0	0
0	-0.070181467	0	0.056623911	0	0	0	0.092232522	0	0
0	0	0	0	0	-0.057212054	0	0.090396791	0	0
0	0	0	0.048393644	0	0	0	0.057675352	0	0
0	0	0	0	0	-0.063727778	0	0.077672844	0	0
0	0	0	0.052951348	0	0	0	0.078254294	0	0
0	-0.086290985	0	0	0	0	0	0.10225009	0	0
0	0	0	0	0	0	0	0.085972907	0	0
0	-0.063489089	0	0.063647353	0	0	0	0	0	0
0	0	0	0	0	-0.064933389	0	0	0	0
0	0	0	0.053079668	0	0	0	0	0	0
0	0	0	0.085237407	0	0	0	0.089853809	0	0
0	0	0	0	0	-0.058718554	0	0.097660633	0	0

0	0	0	0.15402666	0	-0.072379207	0	0	0	-0.62638585
0	0	0	0.15341036	0	-0.070264284	0	0	0	-0.62624505
0	0	0	0.15321901	0	-0.10019736	0	0.038283881	0	-0.14859464
0	0	0	0.15268502	0	-0.098603411	0	0.038672312	0	-0.14879181
0	0	0	0.13054777	0	-0.077270733	0	0	0	-0.15412705
0	0	0	0.1302146	0	-0.075240851	0	0	0	-0.15430806
0	0	0	0.1573025	0	-0.10877991	0	0	0	-0.14799994
0	0	0	0.1567666	0	-0.10691286	0	0	0	-0.14822415
0	0	0	0.15384984	0	-0.084870491	0	0	0	-0.15547202
0	0	0	0.15326405	0	-0.082978164	0	0	0	-0.15577706
0	0	0	0.15402658	0	-0.10042232	0	0.037773969	0	0
0	0	0	0.15348991	0	-0.098814007	0	0.038156807	0	0
0	0	0	0.13164717	0	-0.077771687	0	0	0	0
0	0	0	0.15353836	0	-0.071364357	0	0	0	-0.14966567
0	0	0	0.13131243	0	-0.075737215	0	0	0	0
0	0.002078891	0	0.15390561	0	-0.072074398	0	0	0	-0.15163027
0	0	0	0.1580766	0	-0.10958595	0	0	0	0
0	0.001195607	0	0.15328244	0	-0.069999967	0	0	0	-0.15104456
0	0	0	0.15754163	0	-0.10771292	0	0	0	0
0	0	0	0.15462541	0	-0.085016007	0	0	0	0
0	0	0	0.15385673	0	-0.082787518	0	0	0	0
0	0	0	0.1543037	0	-0.071931949	0	0	0	0
0	-0.001895897	0	0.1545779	0	-0.072896415	0	0	0	0
0	-0.00278809	0	0.15394366	0	-0.070802852	0	0	0	0
0	0	0	0.083892075	0	0	0	0	0	-0.16371858
0	0	0	0.084915808	0	0	0	0	0	-0.16360018
0	0	0	0.11120378	0	0	0	0	0	-0.62626469
0	0	0	0.11069517	0	0	0	0	0	-0.62626364
0	0	0	0.072811259	0	0	0	0	0	-0.6262919
0	0	0	0.071349497	0	0	0	0	0	-0.62628944
0	0	0	0.084613083	0	0	0	0	0	0
0	0	0	0.091658857	0	0	0	0	0	-0.62638116
0	0	0	0.085635383	0	0	0	0	0	0
0	0	0	0.095807807	0	0	0	-0.005346203	0	-0.62605358
0	0	0	0.094048599	0	0	0	0	0	-0.62638482
0	0.004545789	0	0.092659951	0	0	0	0	0	-0.62674683
0	0	0	0.095200122	0	0	0	-0.006287381	0	-0.62607479
0	0	0	0.093196734	0	0	0	0	0	-0.62626426
0	0.005847131	0	0.091574721	0	0	0	0	0	-0.62675549
0	0	0	0.10855527	0	0	0	0.03413963	0	-0.15781208
0	0	0	0.10809258	0	0	0	0.033568925	0	-0.15782564
0	0	0	0.11127383	0	0	0	0	0	-0.15751317
0	0.019930177	0	0.11581051	0	0	0	0	0	-0.17569257
0	0	0	0.11059234	0	0	0	0	0	-0.1621884
0	0.018049857	0	0.115788	0	0	0	0	0	-0.17400137
0	0	0	0.11009623	0	0	0	0	0	-0.16209298
0	0	0	0.07731268	0	0	0	-0.017901933	0	-0.16369161

0	0	0	0.076192714	0	0	0	-0.019242925	0	-0.16383939
0	0	0	0.072477939	0	0	0	0	0	-0.1636151
0	0	0	0.074357979	0	0	0	0	0	-0.16060456
0	0	0	0.073051336	0	0	0	0	0	-0.16051214
0	-0.005959048	0	0.070559414	0	0	0	0	0	-0.15853271
0	-0.004692069	0	0.069548088	0	0	0	0	0	-0.15982179
0	0	0	0.10910621	0	0	0	0.032981676	0	0
0	0	0	0.10863432	0	0	0	0.032395144	0	0
0	0	0	0.096458835	0	0	0	-0.007319983	0	-0.15946666
0	0	0	0.093647483	0	0	0	0	0	-0.15867339
0	0.005546268	0	0.092114932	0	0	0	0	0	-0.16517262
0	0	0	0.11171505	0	0	0	0	0	0
0	0	0	0.11107889	0	0	0	0	0	0
0	0	0	0.11057747	0	0	0	0	0	0
0	0.008712022	0	0.098343136	0	0	0	-0.008110992	0	-0.16753695
0	0.015521515	0	0.11512315	0	0	0	0	0	0
0	0.013629034	0	0.11510654	0	0	0	0	0	0
0	0.010500827	0	0.09815628	0	0	0	-0.009465324	0	-0.16920132
0	0.006947591	0	0.095042879	0	0	0	0	0	-0.16504064
0	0	0	0.096496772	0	0	0	-0.00596931	0	-0.15947476
0	0.008520974	0	0.094460559	0	0	0	0	0	-0.16627728
0	0	0	0.095953783	0	0	0	-0.006696161	0	-0.15939442
0	0	0	0.078168019	0	0	0	-0.018428146	0	0
0	0	0	0.077040736	0	0	0	-0.01977151	0	0
0	0	0	0.075199609	0	0	0	0	0	0
0	0	0	0.073204548	0	0	0	0	0	0
0	0	0	0.073873157	0	0	0	0	0	0
0	-0.010394719	0	0.069653254	0	0	0	0	0	0
0	-0.10634092	0	0.1714439	0	-0.19332017	0	0	0	0
0	-0.1069477	0	0.17076687	0	-0.19137903	0	0	0	0
0	-0.00911896	0	0.068629474	0	0	0	0	0	0
0	0	0	0.097136051	0	0	0	-0.007940154	0	0
0	0	0	0.094317825	0	0	0	0	0	0
0	0.001531085	0	0.092087375	0	0	0	0	0	0
0	0.004625908	0	0.098138368	0	0	0	-0.007857109	0	0
0	0	0	0.097274131	0	0	0	-0.006231368	0	0
0	0.002997408	0	0.095119283	0	0	0	0	0	0
0	0.006434532	0	0.097952354	0	0	0	-0.009215252	0	0
0	0	0	0.096740317	0	0	0	-0.006957496	0	0
0	0.004598162	0	0.094546102	0	0	0	0	0	0
0	-0.03783621	0	0	0	0	0	0	0	-0.62669413
0	-0.039587668	0	0	0	0	0	0	0	-0.62620528
0	-0.098489658	0	0.16194741	0	-0.1421194	0	0.076371701	0	0
0	0	0	0	0	0	0	0	0	-0.62652111
0	-0.097742447	0	0.16271081	0	-0.14398811	0	0.075974186	0	0
0	0	0	0	0	0	0	0	0	-0.62613911
0	-0.035912559	0	0	0	0	0	0	0	-0.1448323

0	0	0	0	0	0	0	0.04117954	0	-0.17829424
0	0	0	0	0	0	0	0	0	-0.62625659
0	-0.037403256	0	0	0	0	0	0	0	-0.14349098
0	0	0	0	0	-0.006237159	0	0	0	-0.62636811
0	0	0	0	0	0	0	0	0	-0.62649035
0	0	0	0	0	0	0	-0.001612726	0	-0.62650171
0	0	0	0	0	0	0	0.041935909	0	-0.17836496
0	0	0	0	0	-0.00465896	0	0	0	-0.62636705
0	0	0	0	0	0	0	0	0	-0.62649769
0	0	0	0	0	0	0	-0.000198661	0	-0.62635678
0	0	0	0	0	0.032269567	0	0	0	-0.17931126
0	-0.045527608	0	0	0	-0.01841317	0	0	0	-0.13482899
0	0	0	0	0	0.033731681	0	0	0	-0.17941357
0	-0.046200962	0	0	0	-0.016846952	0	0	0	-0.13429127
0	-0.10228176	0	0.12179398	0	-0.099367755	0	0	0	0
0	-0.038087464	0	0	0	0	0	0	0	-0.14143641
0	-0.10288059	0	0.12121021	0	-0.096934583	0	0	0	0
0	-0.039880886	0	0	0	0	0	0	0	0
0	0	0	0	0	0	0	0	0	-0.18218031
0	-0.036684003	0	0	0	0	0	0	0	-0.14186431
0	0	0	0	0	0	0	0	0	-0.17865682
0	-0.036747506	0	0	0	0	0	-0.001805905	0	-0.14283707
0	-0.038220171	0	0	0	0	0	0	0	-0.14079884
0	-0.038315093	0	0	0	0	0	-0.000295214	0	-0.14140461
0	-0.04140035	0	0	0	0	0	0	0	0
0	0	0	0	0	0	0	0	0	-0.1823826
0	0	0	0	0	0	0	0.039944551	0	0
0	-0.049946237	0	0	0	-0.019512455	0	0	0	0
0	-0.050627061	0	0	0	-0.017930293	0	0	0	0
0	0	0	0	0	0	0	0.040702581	0	0
0	0	0	0	0	0.032213676	0	0	0	0
0	-0.042050522	0	0	0	0	0	0	0	0
0	0	0	0	0	0.033676778	0	0	0	0
0	-0.040594185	0	0	0	0	0	0	0	0
0	-0.040670616	0	0	0	0	0	-0.001990201	0	0
0	-0.042155146	0	0	0	0	0	0	0	0
0	-0.042262436	0	0	0	0	0	-0.000467017	0	0
0	0	0	0	0	-0.005346922	0	0	0	-0.17744464
0	0	0	0	0	0	0	0	0	-0.17629025
0	0	0	0	0	0	0	-0.002483326	0	-0.17745857
0	0	0	0	0	-0.00523962	0	0	0	-0.17686977
0	0	0	0	0	-0.007144863	0	0.001805	0	-0.17744322
0	0	0	0	0	0	0	0	0	0
0	0	0	0	0	0	0	-0.000731473	0	-0.1763655
0	0	0	0	0	0	0	0	0	0
0	0	0	0	0	-0.005929871	0	0.002462651	0	-0.17753357
0	0	0	0	0	-0.00382981	0	0	0	-0.17708766

0	0	0	0	0	0	0	0.000476171	0	-0.17657891
0	0	0	0	0	0	0	0	0	0
0	0	0	0	0	-0.005239554	0	0	0	0
0	0	0	0	0	0	0	0	0	0
0	0	0	0	0	0	0	-0.002926215	0	0
0	0	0	0	0	-0.004808107	0	0	0	0
0	0	0	0	0	-0.006658188	0	0.001098868	0	0
0	0	0	0	0	0	0	-0.00082354	0	0
0	0	0	0	0	-0.003391098	0	0	0	0
0	0	0	0	0	-0.005430529	0	0.00175522	0	0
0	0	0	0	0	0	0	0.000392308	0	0
0	-0.091986683	0	0.16731644	0	-0.10589753	0	0	0	0
0	-0.092942717	0	0.16644272	0	-0.10366829	0	0	0	0
0	-0.08461515	0	0.16704098	0	-0.089116663	0	0	0	0
0	-0.10098028	0	0.059657185	0	0	0	0	0	0
0	-0.13340443	0	0	0	-0.062625049	0	0.043695883	0	0
0	-0.13287918	0	0	0	-0.064101095	0	0.043142707	0	0
0	-0.10017184	0	0.058723487	0	0	0	0	0	0
0	-0.099504817	0	0.053006559	0	0	0	0	0	0
0	-0.09857396	0	0.051803807	0	0	0	0	0	0
0	0	0	0.16682905	0	-0.19805619	0	0.061217001	0	-0.15240364
0	-0.10398874	0	0.048523149	0	0	0	0	0	0
0	-0.12811783	0	0	0	-0.03858715	0	0	0	0
0	-0.1311288	0	0	0	-0.055727652	0	0	0	0
0	-0.13163829	0	0	0	-0.054157046	0	0	0	0
0	-0.1035535	0	0.050722685	0	0	0	-0.006649947	0	0
0	0	0	0.17332146	0	-0.20583441	0	0	0	-0.62617614
0	-0.12843379	0	0	0	-0.041014223	0	0	0	0
0	-0.12907279	0	0	0	-0.039316269	0	0	0	0
0	-0.10277976	0	0.049655743	0	0	0	-0.008093355	0	0
0	-0.034724087	0	0	0	0	0	0.068816456	0	-0.62687549
0	-0.033070241	0	0	0	0	0	0.067410574	0	-0.62688295
0	-0.11980456	0	0	0	0	0	0.045311438	0	0
0	0	0	0.12123021	0	-0.14023434	0	0.08815111	0	-0.16332697
0	0	0	0	0	0.026582249	0	0.042178687	0	-0.62658854
0	0	0	0	0	0.027906637	0	0.042335602	0	-0.62658491
0	-0.11880979	0	0	0	0	0	0.04460543	0	0
0	0	0	0.16775841	0	-0.19901552	0	0.060115192	0	0
0	0	0	0	0	0	0	0.055022543	0	-0.62662852
0	0	0	0.15892494	0	-0.15479524	0	0.11941091	0	-0.62631977
0	0	0	0	0	0	0	0.055958933	0	-0.6263118
0	0	0	0	0	0	0	0.065900858	0	-0.62661261
0	0	0	0	0	0	0	0.056817363	0	-0.62661832
0	-0.087989189	0	0.094932613	0	0	0	0.039907749	0	0
0	-0.1196639	0	0	0	0	0	0	0	0
0	0	0	0	0	0	0	0.057431326	0	-0.62662782
0	0	0	0.14933684	0	-0.18486431	0	0	0	-0.15742847

0	0	0	0	0	0.045396466	0	0	0	-0.62651889
0	0	0	0	0	0.046855287	0	0	0	-0.62651744
0	0	0	0.12224044	0	-0.14061814	0	0.087904256	0	0
0	-0.11919937	0	0	0	0	0	0	0	0
0	-0.086866657	0	0.094640326	0	0	0	0.039323357	0	0
0	-0.11807062	0	0	0	0	0	0	0	0
0	-0.11721316	0	0	0	0	0	0	0	0
0	-0.11660363	0	0	0	0	0	0.012915244	0	0
0	0	0	0.17249329	0	-0.20488263	0	0	0	-0.15548669
0	-0.086681885	0	0.098544093	0	0	0	0	0	0
0	-0.014459518	0	0.17370947	0	-0.21564697	0	0	0	-0.13831325
0	-0.088021846	0	0.087218328	0	0	0	0.01674507	0	0
0	0	0	0	0	0.058663827	0	0	0	-0.62647276
0	-0.086533114	0	0.094522881	0	0	0	0	0	0
0	-0.0912716	0	0.084506005	0	0	0	0.008053081	0	0
0	-0.11546029	0	0	0	0	0	0.011720295	0	0
0	-0.11944637	0	0	0	0	0	0.003364629	0	0
0	0	0	0	0	0.041542123	0	0	0	-0.62625754
0	0	0	0	0	0.04336988	0	0	0	-0.62635453
0	-0.034055474	0	0	0	0	0	0.056209776	0	-0.14785569
0	-0.032367247	0	0	0	0	0	0.055153365	0	-0.14924878
0	-0.02366859	0	0	0	0.020727481	0	0.04936108	0	-0.15290458
0	-0.08618217	0	0.098459381	0	0	0	0	0	0
0	-0.022396035	0	0	0	0.019770431	0	0.048815306	0	-0.15402854
0	-0.002940847	0	0	0	0.057725988	0	0	0	-0.62685784
0	-0.032340522	0	0	0	0	0	0	0	-0.62670103
0	-0.00403006	0	0	0	0.059299387	0	0	0	-0.62684703
0	-0.034037064	0	0	0	0	0	0	0	-0.62669214
0	-0.086800563	0	0.086878969	0	0	0	0.015991473	0	0
0	0	0	0	0	0.028925638	0	0.039823546	0	-0.175284
0	-0.033187692	0	0	0	0	0	0.067148453	0	-0.14338616
0	0	0	0	0	0.04048006	0	0.052288988	0	-0.1756942
0	0	0	0	0	0.038729312	0	0.051782153	0	-0.17559204
0	0	0	0.1508301	0	-0.18626267	0	0	0	0
0	0	0	0	0	0.024806182	0	0.035722197	0	-0.17834185
0	0	0	0	0	0.025945511	0	0.035907383	0	-0.17884488
0	-0.033809501	0	0	0	0	0	0.055535881	0	-0.14289741
0	-0.032136027	0	0	0	0	0	0.054911129	0	-0.14442143
0	-0.084866259	0	0.098392456	0	0	0	0	0	0
0	0	0	0.15888984	0	-0.15880292	0	0.10752777	0	-0.16215223
0	0	0	0	0	0	0	0	0	-0.62651352
0	-0.020022243	0	0.17466148	0	-0.22036459	0	0	0	0
0	0	0	0	0	0	0	0	0	-0.62650972
0	0	0	0.17331082	0	-0.20569269	0	0	0	0
0	0	0	0	0	0	0	0.054005983	0	-0.17963395
0	0.017029368	0	0.15918792	0	-0.14678591	0	0.11020613	0	-0.17206924
0	0	0	0	0	0	0	0	0	-0.62647924

0	0	0	0	0	0	0	0.051754067	0	-0.17940897
0	0	0	0	0	0	0	0.052319896	0	-0.17956414
0	-0.038299512	0	0	0	0	0	0.056660823	0	0
0	-0.036583124	0	0	0	0	0	0.055600517	0	0
0	0	0	0	0	0	0	0.054287526	0	-0.17468647
0	0	0	0	0	0.046155476	0	0	0	-0.18226629
0	-0.028494777	0	0	0	0.01949118	0	0.050611808	0	0
0	-0.027206798	0	0	0	0.018530103	0	0.050060943	0	0
0	-0.037466719	0	0	0	0	0	0.067287489	0	0
0	-0.011097234	0	0	0	0.044605157	0	0	0	-0.17274086
0	-0.009847729	0	0	0	0.04336925	0	0	0	-0.17368683
0	-0.038042653	0	0	0	0	0	0.054687293	0	0
0	-0.036349072	0	0	0	0	0	0.054053326	0	0
0	0	0	0	0	0.041586289	0	0	0	-0.18184322
0	0	0	0	0	0.04332685	0	0	0	-0.18215031
0	0	0	0	0	0.029493888	0	0.039192422	0	0
0	0	0	0	0	0.040474742	0	0.051071093	0	0
0	0	0	0	0	0.038722862	0	0.050564707	0	0
0	0	0	0	0	0.025524866	0	0.035405914	0	0
0	0	0	0	0	0.026806887	0	0.035476869	0	0
0	0	0	0	0	0.04346015	0	0	0	-0.17605741
0	0	0	0.15975952	0	-0.15898572	0	0.10749266	0	0
0	-0.031715822	0	0	0	0	0	0	0	-0.15119215
0	-0.03343659	0	0	0	0	0	0	0	-0.14975181
0	-0.015638863	0	0	0	0.031477027	0	0	0	-0.16113994
0	-0.016927958	0	0	0	0.032497223	0	0	0	-0.16002026
0	-0.003069167	0	0	0	0.058445385	0	0	0	-0.17367778
0	-0.032696744	0	0	0	0	0	0	0	-0.14430275
0	0	0	0	0	0	0	0.054227617	0	0
0	0.012350809	0	0.15996176	0	-0.14896461	0	0.11197038	0	0
0	0	0	0	0	0	0	0.050654922	0	0
0	0	0	0	0	0	0	0.051222231	0	0
0	0	0	0	0	0.046706613	0	0	0	0
0	0	0	0	0	0	0	0.053001326	0	0
0	0	0	0	0	0	0	0	0	-0.1809967
0	-0.015320501	0	0	0	0.044305229	0	0	0	0
0	-0.014058189	0	0	0	0.043058477	0	0	0	0
0	-0.030166983	0	0	0	0	0	0	0	-0.6261548
0	0	0	0	0	0.041529398	0	0	0	0
0	0	0	0	0	0.043371066	0	0	0	0
0	-0.035716636	0	0	0	0	0	0	0	0
0	-0.037453578	0	0	0	0	0	0	0	0
0	-0.031459677	0	0	0	0	0	0	0	-0.62683987
0	-0.022972023	0	0	0	0.028507696	0	0	0	0
0	-0.021665431	0	0	0	0.027499892	0	0	0	0
0	0	0	0	0	0.043490426	0	0	0	0
0	-0.036692	0	0	0	0	0	0	0	0

0	-0.00725558	0	0	0	0.058199871	0	0	0	0
0	0	0	0	0	0	0	0	0	-0.62655734
0	0	0	0.18431678	0	-0.12293105	0	0.052542484	0	0
0	0	0	0.19070749	0	-0.14535375	0	0	0	0
0	0	0	0.18378416	0	-0.12137531	0	0.053030959	0	0
0	0	0	0	0	0	0	0	0	0
0	0	0	0.1902204	0	-0.14366784	0	0	0	0
0	0	0	0.18819894	0	-0.14497985	0	0.030131958	0	0
0	0	0	0.18557	0	-0.12913589	0	0.042490018	0	0
0	0	0	0.089916244	0	-0.082482312	0	0	0	-0.62624696
0	0	0	0.18764743	0	-0.14324581	0	0.030931179	0	0
0	0	0	0.18874744	0	-0.13095316	0	0.045055796	0	0
0	0	0	0.20329221	0	-0.15619476	0	0	0	0
0	0	0	0.19049979	0	-0.14409636	0	0	0	0
0	0	0	0.18865605	0	-0.12944792	0	0.045879566	0	0
0	0	0	0.099729726	0	-0.099686981	0	0	0	-0.18123473
0	-0.030082606	0	0	0	0	0	0	0	-0.16435882
0	0	0	0.20328135	0	-0.1548254	0	0	0	0
0	-0.10738216	0	0.18173879	0	-0.28735459	0	0.06789795	0	0
0	0.030647743	0	0.10000863	0	-0.078383149	0	0	0	-0.20101696
0	0	0	0	0	-0.059164505	0	0.062435106	0	-0.62645832
0	0	0	0.17381552	0	-0.093691927	0	0	0	0
0	-0.034179746	0	0	0	0	0	0	0	0
0	0	0	0.17847296	0	-0.1004091	0	0	0	0
0	0	0	0.10057362	0	-0.099864982	0	0	0	0
0	0	0	0.186006	0	-0.092619909	0	0	0	0
0	0	0	0.17361957	0	-0.091640688	0	0	0	0
0	0.02638018	0	0.099640965	0	-0.079439733	0	0	0	0
0	-0.026689898	0	0	0	-0.070967721	0	0.070129845	0	-0.16010479
0	0	0	0	0	-0.051527959	0	0.0705188	0	-0.18569629
0	-0.10923709	0	0.17009445	0	-0.28083452	0	0	0	0
0	0	0	0	0	-0.058635418	0	0.060071177	0	-0.18569406
0	0	0	0	0	0	0	0.075065517	0	-0.62623154
0	-0.10529203	0	0.18908595	0	-0.29446018	0	0	0	0
0	-0.031574304	0	0	0	-0.073010885	0	0.071401204	0	0
0	0	0	0	0	-0.053806543	0	0	0	-0.62616468
0	0	0	0	0	-0.051631371	0	0.069273789	0	0
0	0	0	0	0	-0.058145259	0	0.059776647	0	0
0	0	0	0.025944911	0	0	0	0.071690792	0	-0.18440228
0	0.041011067	0	0.043438382	0	0	0	0	0	-0.62695722
0	0	0	0	0	-0.037516619	0	0	0	-0.62624681
0	0	0	0.021525546	0	0	0	0.027991293	0	-0.6263318
0	0.015713386	0	0	0	0	0	0.071166201	0	-0.20275551
0	0.002806111	0	0	0	-0.029957772	0	0	0	-0.62626548
0	0.014079836	0	0	0	0	0	0.029412823	0	-0.62606864
0	0	0	0	0	0	0	0.071980967	0	-0.18960758
0	0	0	0	0	0	0	0.035092988	0	-0.62621742

0	0.06329288	0	0.16391095	0	-0.090751577	0	0	0	-0.23395557
0	-0.020518891	0	0	0	-0.067353105	0	0	0	-0.16739765
0	0.036227072	0	0.038319153	0	0	0	0.02106688	0	-0.21499951
0	0	0	0.026595497	0	0	0	0.070484118	0	0
0	0	0	0.027076066	0	0	0	0	0	-0.62618995
0	0	0	0	0	-0.053545913	0	0	0	-0.18980876
0	0	0	0.025409413	0	0	0	0	0	-0.62628125
0	-0.12520639	0	0	0	-0.043296979	0	0.060988756	0	0
0	0.045746166	0	0.048210298	0	0	0	0	0	-0.2244508
0	-0.12462584	0	0	0	-0.045092673	0	0.06055724	0	0
0	0.042041936	0	0.044017029	0	0	0	0	0	-0.22092087
0	0.018165155	0	0	0	0	0	0	0	-0.62691625
0	0.018953605	0	0	0	0	0	0	0	-0.62692179
0	-0.10395157	0	0.12022486	0	-0.19150579	0	0.12921618	0	0
0	-0.001650374	0	0	0	-0.038025864	0	0	0	-0.19267597
0	0.011833744	0	0	0	0	0	0.070170001	0	0
0	0	0	0	0	0	0	0	0	-0.62631647
0	0	0	0	0	0	0	0.070838192	0	0
0	0	0	0.022060554	0	0	0	0.031074967	0	-0.1816229
0	-0.11833842	0	0	0	-0.013953187	0	0.085281102	0	0
0	-0.02636748	0	0	0	-0.071389257	0	0	0	0
0	-0.11569623	0	0	0	0	0	0.057725532	0	0
0	-0.12068401	0	0	0	-0.016921732	0	0.083376153	0	0
0	0	0	0.11406001	0	0	0	0	0	0
0	0.015393973	0	0	0	0	0	0.032173162	0	-0.199318
0	-0.11613605	0	0	0	0	0	0.070535405	0	0
0	-0.022806499	0	0	0	0	0	0	0	-0.62640952
0	0	0	0.12749674	0	0	0	0.037940382	0	0
0	0	0	0.11523523	0	0	0	0	0	0
0	0	0	0.12700867	0	0	0	0.037316774	0	0
0	-0.11580961	0	0	0	0	0	0.058380185	0	0
0	0	0	0	0	-0.053596445	0	0	0	0
0	-0.11986483	0	0	0	-0.018279583	0	0.083431036	0	0
0	0.059681877	0	0.16474939	0	-0.091727805	0	0	0	0
0	0.031890538	0	0.037112114	0	0	0	0.021210518	0	0
0	-0.11461567	0	0	0	0	0	0.057915177	0	0
0	0.083774897	0	0.16515127	0	-0.060774726	0	0	0	-0.62624484
0	0	0	0.13052294	0	0	0	0	0	0
0	0	0	0.11361362	0	0	0	0.03198293	0	0
0	0	0	0.11474446	0	0	0	0.032748895	0	0
0	0	0	0.16378717	0	-0.10512124	0	0	0	-0.62626093
0	0	0	0.0270786	0	0	0	0	0	-0.18832273
0	0.041292645	0	0.04683415	0	0	0	0	0	0
0	0	0	0.10809863	0	0	0	0	0	0
0	0.060297927	0	0.079618884	0	0	0	0.044552942	0	-0.62644407
0	0	0	0.11687567	0	0	0	0	0	0
0	0	0	0.12796083	0	0	0	0.032812028	0	0

0	-0.005753279	0	0	0	-0.039265203	0	0	0	0
0	0.037871399	0	0.042940253	0	0	0	0	0	0
0	0	0	0.11782072	0	0	0	0.005883436	0	0
0	0	0	0.022790246	0	0	0	0.030918413	0	0
0	0	0	0.10792479	0	0	0	0.002171048	0	0
0	0	0	0.10668816	0	0	0	0.000925104	0	0
0	-0.12407929	0	0	0	-0.039074593	0	0	0	0
0	0.01960575	0	0	0	0	0	0	0	-0.21043191
0	-0.12562723	0	0	0	-0.040202942	0	0	0	0
0	-0.1248814	0	0	0	-0.041885924	0	0	0	0
0	0.011724522	0	0	0	0	0	0.032411525	0	0
0	0.067864302	0	0.089986446	0	0	0	0.071030107	0	-0.23556639
0	0	0	0.027703549	0	0	0	0	0	0
0	-0.11629316	0	0	0	0	0	0	0	0
0	0.01594767	0	0	0	0	0	0	0	0
0	0.062306102	0	0.080889444	0	0	0	0.043897775	0	-0.23113088
0	0.073870256	0	0.09489944	0	0	0	0	0	-0.62703388
0	0.078637144	0	0.10137334	0	0	0	0	0	-0.62716698
0	0	0	0.10925194	0	0	0	-0.0195595	0	0
0	0.063451769	0	0.08914323	0	0	0	0.069971683	0	0
0	0	0	0.068186897	0	0	0	0.082661855	0	-0.62640638
0	0	0	0.06301808	0	0	0	0.062871999	0	-0.62650314
0	-0.089859566	0	0.17359645	0	-0.20932055	0	0.15690105	0	0
0	0.058413914	0	0.080679597	0	0	0	0.044707439	0	0
0	0.074398384	0	0.094714622	0	0	0	0	0	-0.24583925
0	0	0	0.068081794	0	0	0	0.078876744	0	-0.17851825
0	-0.11213183	0	0	0	0.034339111	0	0	0	0
0	0.070210589	0	0.09390585	0	0	0	0	0	0
0	-0.13391336	0	0	0	-0.14604587	0	0.080684248	0	0
0	0	0	0.068586108	0	0	0	0.07778681	0	0
0	0	0	0.071295546	0	0	0	0	0	-0.62609729
0	-0.12641157	0	0	0	-0.12018184	0	0.11137869	0	0
0	0	0	0	0	-0.047848816	0	0.088766855	0	-0.62649265
0	0	0	0	0	-0.028707225	0	0.11481076	0	-0.62630079
0	0.010073825	0	0	0	-0.019677698	0	0.12037202	0	-0.62722879
0	0.020627208	0	0	0	0	0	0.094404875	0	-0.62639348
0	-0.13204437	0	0	0	-0.14711496	0	0	0	0
0	0.020837579	0	0	0	0	0	0.088578357	0	-0.6271552
0	0	0	0	0	0	0	0.088953462	0	-0.62664932
0	0	0	0	0	-0.047743929	0	0.084463363	0	-0.18599363
0	0	0	0.20110964	0	-0.23786858	0	0.06544886	0	0
0	-0.010751727	0	0	0	-0.054806516	0	0.086959234	0	-0.17222214
0	0.008314972	0	0	0	-0.024079681	0	0.10978306	0	-0.1974374
0	-0.017175071	0	0	0	-0.059253892	0	0.086307544	0	0
0	0	0	0	0	-0.047844239	0	0.083350937	0	0
0	0	0	0.21205064	0	-0.24827007	0	0.061156329	0	0
0	0.021796401	0	0	0	0	0	0.084263217	0	-0.208398

0	0	0	0	0	0	0	0.05069899	0	0
0	0	0	0	0	0	0	0.051519386	0	0
0	0	0	0	0	0	0	0.026182311	0	0
0	0	0	0	0	5.88E-05	0	0.025957177	0	0
0	0.0034782	0	0	0	-0.025566188	0	0.11160403	0	0
0	0	0	0	0	-0.050316693	0	0	0	-0.62632205
0	0	0	0	0	0.001273629	0	0.026680288	0	0
0	0	0	0	0	0.026925615	0	0	0	0
0	0	0	0.21400112	0	-0.25103983	0	0	0	0
0	0	0	0	0	0.012678243	0	0	0	0
0	0	0	0	0	0.028351305	0	0	0	0
0	-0.006370328	0	0	0	-0.054502973	0	0	0	-0.62619738
0	0	0	0	0	0	0	0	0	0
0	0	0	0	0	0.024451094	0	0.044131871	0	0
0	0.017840805	0	0	0	0	0	0.083397346	0	0
0	0	0	0	0	0.025894603	0	0.044903873	0	0
0	0	0	0	0	0	0	0.045560463	0	0
0	-0.097769511	0	0.093087324	0	-0.12674177	0	0	0	0
0	0	0	0	0	0.028338732	0	0	0	0
0	0.026022222	0	0	0	0	0	0	0	-0.62694457
0	-0.004925962	0	0	0	-0.053338124	0	0	0	-0.18456929
0	0	0	0.16871904	0	-0.17421204	0	0.11843174	0	0
0	0	0	0	0	-0.008187632	0	0.009942748	0	0
0	-0.010852061	0	0	0	-0.05740214	0	0	0	0
0	0.061411576	0	0	0	0.047145884	0	0	0	-0.62725308
0	-0.090167067	0	0	0	0	0	0.087627697	0	0
0	-0.095011748	0	-0.003198973	0	0	0	0.08504465	0	0
0	-0.090333714	0	-0.00432856	0	0	0	0.060233729	0	0
0	-0.12540294	0	0	0	-0.13642131	0	0.097316452	0	0
0	0	0	0	0	0.033689621	0	0.059580597	0	0
0	0	0	0	0	0.032551732	0	0.060427143	0	0
0	0	0	0	0	0.034249588	0	0.0609983	0	0
0	0	0	0	0	0	0	0.062389128	0	0
0	0	0	0	0	0.03429113	0	0.055822818	0	0
0	0	0	0	0	0.037722433	0	0	0	0
0	-0.089146526	0	0.001256247	0	0	0	0	0	0
0	-0.066124763	0	0.058324977	0	0	0	0.094758923	0	0
0	0	0	0	0	-0.062355545	0	0.083156499	0	0
0	0	0	0.051223206	0	0	0	0.083723423	0	0
0	0	0	0.15336199	0	-0.10155346	0	0.039881218	0	-0.6263276
0	0	0	0.15282878	0	-0.099965003	0	0.040272662	0	-0.62632824
0	0	0	0.13077681	0	-0.077479633	0	0	0	-0.62622123
0	0	0	0.13044557	0	-0.075422812	0	0	0	-0.62626161
0	0	0	0.15744548	0	-0.10900316	0	0	0	-0.62625262
0	0	0	0.15691103	0	-0.10712754	0	0	0	-0.62623521
0	0	0	0.15401385	0	-0.08500203	0	0	0	-0.62627532
0	0	0	0.15342919	0	-0.083112705	0	0	0	-0.6262748

0	0	0	0.15369672	0	-0.071562917	0	0	0	-0.62624821
0	0.000971015	0	0.15404646	0	-0.072320684	0	0	0	-0.62672036
0	0	0	0.1379381	0	-0.096419959	0	0.0285226	0	-0.15182136
0	-0.000179866	0	0.15341606	0	-0.070276565	0	0	0	-0.62610328
0	0	0	0.13782225	0	-0.095004972	0	0.029224197	0	-0.15193194
0	-0.016800242	0	0.15272524	0	-0.10577076	0	0.044458358	0	-0.13267577
0	0	0	0.15330114	0	-0.10346369	0	0.033121331	0	-0.151954
0	0	0	0.15294987	0	-0.098991911	0	0.037637975	0	-0.14859346
0	-0.017941995	0	0.1521583	0	-0.10451158	0	0.045220826	0	-0.13182219
0	0	0	0.15451525	0	-0.10787208	0	0.031534513	0	-0.14820625
0	0	0	0.15276851	0	-0.10192262	0	0.033420215	0	-0.1522037
0	0	0	0.13408151	0	-0.084568251	0	0	0	-0.15714064
0	-0.016273583	0	0.12605195	0	-0.079213385	0	0	0	-0.13969036
0	0	0	0.13078581	0	-0.076427243	0	0	0	-0.15401016
0	0	0	0.15393599	0	-0.10599966	0	0.032217729	0	-0.14841796
0	0	0	0.1378959	0	-0.092418428	0	0	0	-0.15227145
0	0	0	0.13387054	0	-0.082798036	0	0	0	-0.15742381
0	-0.023822433	0	0.15814151	0	-0.1251009	0	0	0	-0.12504131
0	-0.017026852	0	0.12551945	0	-0.077222942	0	0	0	-0.13919969
0	0	0	0.13782837	0	-0.09098398	0	0	0	-0.1523935
0	0	0	0.15650086	0	-0.10804813	0	0	0	-0.15187653
0	-0.024957284	0	0.1576308	0	-0.12391179	0	0	0	-0.12416037
0	0	0	0.15689887	0	-0.10692987	0	0	0	-0.14803605
0	0	0	0.15593906	0	-0.10612432	0	0	0	-0.15220044
0	0	0	0.15355673	0	-0.083942721	0	0	0	-0.15539286
0	0	0	0.13897028	0	-0.09667886	0	0.0281402	0	0
0	-0.021368421	0	0.15337531	0	-0.10757574	0	0.045718922	0	0
0	-0.005739617	0	0.15371901	0	-0.085916587	0	0	0	-0.15041377
0	0	0	0.13885435	0	-0.095259009	0	0.028842722	0	0
0	-0.02255018	0	0.15281474	0	-0.1063128	0	0.046482521	0	0
0	0	0	0.083982266	0	0	0	0	0	-0.62629895
0	0	0	0.15375275	0	-0.099204769	0	0.037124508	0	0
0	0	0	0.15410527	0	-0.10348578	0	0.032896334	0	0
0	-0.006790138	0	0.15306906	0	-0.084189176	0	0	0	-0.1497868
0	0	0	0.085002593	0	0	0	0	0	-0.62629348
0	-0.02072865	0	0.12588395	0	-0.080234275	0	0	0	0
0	0	0	0.155427	0	-0.10875879	0	0.030430537	0	0
0	-0.029547527	0	0.15909393	0	-0.12982138	0	0	0	0
0	0	0	0.15357144	0	-0.10193914	0	0.033186986	0	0
0	0	0	0.13500843	0	-0.084709093	0	0	0	0
0	0	0	0.13188591	0	-0.076925889	0	0	0	0
0	0	0	0.15484865	0	-0.1068881	0	0.031113059	0	0
0	-0.030712287	0	0.15857905	0	-0.12862862	0	0	0	0
0	-0.021489858	0	0.12534955	0	-0.07822762	0	0	0	0
0	0	0	0.1394707	0	-0.093906281	0	0	0	0
0	0	0	0.13480691	0	-0.082946105	0	0	0	0
0	0.000973477	0	0.15356182	0	-0.071305435	0	0	0	-0.15057611

0	0	0	0.13939663	0	-0.092465873	0	0	0	0
0	0	0	0.15733528	0	-0.10891957	0	0	0	0
0	0	0	0.15768811	0	-0.10775069	0	0	0	0
0	0	0	0.15679394	0	-0.10701456	0	0	0	0
0	0	0	0.15431358	0	-0.084061845	0	0	0	0
0	-0.009752158	0	0.15439264	0	-0.086795295	0	0	0	0
0	-0.010794994	0	0.15377762	0	-0.085067718	0	0	0	0
0	0	0	0.081816496	0	0	0	0.031866418	0	-0.16376318
0	-0.003015747	0	0.15422752	0	-0.072113432	0	0	0	0
0	0	0	0.082789003	0	0	0	0.032540356	0	-0.16363307
0	0	0	0.10841506	0	0	0	0.036739282	0	-0.62635273
0	0	0	0.10794764	0	0	0	0.036156014	0	-0.62634075
0	0	0	0.084911693	0	0	0	0	0	-0.16343856
0	0	0	0.083897452	0	0	0	0	0	-0.16718743
0	0	0	0.084924708	0	0	0	0	0	-0.16719055
0	0.004204481	0	0.085564256	0	0	0	0	0	-0.16738312
0	0	0	0.11132912	0	0	0	0	0	-0.62641305
0	0.002686737	0	0.085971072	0	0	0	0	0	-0.16594014
0	0	0	0.11065233	0	0	0	0	0	-0.62628662
0	0.018700586	0	0.11547948	0	0	0	0	0	-0.62682645
0	0	0	0.11015154	0	0	0	0	0	-0.62629066
0	0.01681275	0	0.1154573	0	0	0	0	0	-0.62681544
0	0	0	0.077106333	0	0	0	-0.016771521	0	-0.62624877
0	0	0	0.075978814	0	0	0	-0.018114781	0	-0.62625018
0	0	0	0.072550842	0	0	0	0	0	-0.62629092
0	0	0	0.082646134	0	0	0	0.030639111	0	0
0	0	0	0.074453313	0	0	0	0	0	-0.62627927
0	0	0	0.083616769	0	0	0	0.031344846	0	0
0	0	0	0.073138762	0	0	0	0	0	-0.62627787
0	-0.007553493	0	0.070027318	0	0	0	0	0	-0.6261119
0	-0.005983504	0	0.069039826	0	0	0	0	0	-0.62676239
0	0	0	0.085643833	0	0	0	0	0	0
0	0	0	0.0846194	0	0	0	0	0	0
0	0	0	0.085654906	0	0	0	0	0	0
0	0	0	0.095845591	0	0	0	-0.006412176	0	-0.62625959
0	-0.00038431	0	0.084459612	0	0	0	0	0	0
0	0	0	0.093691522	0	0	0	0	0	-0.62625213
0	0.004429131	0	0.091982572	0	0	0	0	0	-0.62674919
0	-0.001908628	0	0.084879926	0	0	0	0	0	0
0	0.007128606	0	0.097341123	0	0	0	-0.006761984	0	-0.62675117
0	0.00582092	0	0.094891947	0	0	0	0	0	-0.626751
0	0	0	0.095981169	0	0	0	-0.004668142	0	-0.62611619
0	0.008926905	0	0.097153142	0	0	0	-0.008118549	0	-0.6267627
0	0.007400675	0	0.094305286	0	0	0	0	0	-0.62675961
0	0	0	0.095450764	0	0	0	-0.00536597	0	-0.62625324
0	0	0	0.1087249	0	0	0	0.033425213	0	-0.157636
0	0.016226054	0	0.11281252	0	0	0	0.032978981	0	-0.17246395

0	0.018124757	0	0.11288849	0	0	0	0.032235351	0	-0.17417908
0	0	0	0.1082339	0	0	0	0.032213996	0	-0.16160476
0	0	0	0.10776898	0	0	0	0.031685614	0	-0.16152881
0	0	0	0.11085346	0	0	0	0	0	-0.16210549
0	0.018464679	0	0.11601455	0	0	0	0	0	-0.17419046
0	0.019327841	0	0.11512055	0	0	0	0	0	-0.17938253
0	0.017415768	0	0.11508259	0	0	0	0	0	-0.17778253
0	0	0	0.07732026	0	0	0	-0.019026989	0	-0.16355434
0	0	0	0.077447394	0	0	0	-0.015402716	0	-0.1622138
0	0	0	0.074174496	0	0	0	0	0	-0.16031859
0	0	0	0.076353293	0	0	0	-0.016467557	0	-0.16220673
0	-0.002655301	0	0.076273077	0	0	0	-0.017605498	0	-0.16136721
0	-0.001121829	0	0.07574881	0	0	0	-0.019117732	0	-0.16286315
0	-0.005824368	0	0.070354573	0	0	0	0	0	-0.15852686
0	0	0	0.10927103	0	0	0	0.03226014	0	0
0	-0.004600438	0	0.072639429	0	0	0	0	0	-0.1566708
0	-0.003211973	0	0.071837993	0	0	0	0	0	-0.15776385
0	0	0	0.10880908	0	0	0	0.031163097	0	0
0	0	0	0.10833868	0	0	0	0.030631193	0	0
0	0.011695878	0	0.11218727	0	0	0	0.03205719	0	0
0	0.013612228	0	0.11225867	0	0	0	0.031303815	0	0
0	0	0	0.11120458	0	0	0	0	0	0
0	-0.10727195	0	0.16803065	0	-0.19272165	0	0.037287107	0	0
0	-0.1078725	0	0.1673131	0	-0.19076578	0	0.037938814	0	0
0	0.01403657	0	0.11533098	0	0	0	0	0	0
0	0.009035475	0	0.098440314	0	0	0	-0.009292364	0	-0.16768557
0	0	0	0.096553007	0	0	0	-0.006945296	0	-0.15926016
0	0.007033465	0	0.094711926	0	0	0	0	0	-0.16482708
0	0.014923992	0	0.11447119	0	0	0	0	0	0
0	0.012999451	0	0.11443853	0	0	0	0	0	0
0	0	0	0.0781697	0	0	0	-0.019563991	0	0
0	0.008815814	0	0.098510176	0	0	0	-0.007584111	0	-0.16732635
0	0.010656019	0	0.098411003	0	0	0	-0.008665744	0	-0.16887743
0	0	0	0.0783108	0	0	0	-0.015629573	0	0
0	-0.00701564	0	0.075391414	0	0	0	-0.017629936	0	0
0	0	0	0.074978925	0	0	0	0	0	0
0	0	0	0.077204511	0	0	0	-0.016714935	0	0
0	-0.005466843	0	0.074858188	0	0	0	-0.019150401	0	0
0	-0.10930053	0	0.15587388	0	-0.18214784	0	0	0	0
0	-0.10663779	0	0.17092949	0	-0.19122492	0	0	0	0
0	-0.010284204	0	0.069436095	0	0	0	0	0	0
0	-0.10983525	0	0.15554544	0	-0.18048156	0	0	0	0
0	-0.008960947	0	0.071823309	0	0	0	0	0	0
0	-0.007569463	0	0.071002909	0	0	0	0	0	0
0	-0.10670954	0	0.17138803	0	-0.19351769	0	0	0	0
0	-0.1074166	0	0.1706945	0	-0.19162246	0	0	0	0
0	-0.10719201	0	0.13345734	0	-0.13958128	0	0.061291286	0	0

0	-0.10777059	0	0.1331114	0	-0.13787584	0	0.06193756	0	0
0	0.00495077	0	0.098238332	0	0	0	-0.009035765	0	0
0	0	0	0.09733238	0	0	0	-0.007211941	0	0
0	-0.036941017	0	0	0	0	0	0	0	-0.62671718
0	0.003082344	0	0.094788519	0	0	0	0	0	0
0	0	0	0	0	0	0	0.043718584	0	-0.62636957
0	-0.038442961	0	0	0	0	0	0	0	-0.62671194
0	0.004783959	0	0.098363699	0	0	0	-0.007110972	0	0
0	0.006629343	0	0.098272099	0	0	0	-0.008202951	0	0
0	0	0	0	0	0	0	0.044491876	0	-0.62646722
0	0	0	0	0	0.03217726	0	0	0	-0.6263638
0	-0.046789025	0	0	0	-0.018861631	0	0	0	-0.62667637
0	-0.047743977	0	0	0	-0.017365541	0	0	0	-0.62607745
0	0	0	0	0	0.033639604	0	0	0	-0.62635813
0	-0.039122277	0	0	0	0	0	0	0	-0.62667784
0	-0.098238997	0	0.1622313	0	-0.14242389	0	0.075276639	0	0
0	-0.03645273	0	0	0	0	0	0.041883348	0	-0.14389869
0	-0.037700471	0	0	0	0	0	0	0	-0.62669407
0	-0.037811164	0	0	0	0	0	-0.000599963	0	-0.62669717
0	0	0	0	0	0	0	0	0	-0.62654275
0	-0.039251928	0	0	0	0	0	0	0	-0.62668946
0	-0.039393143	0	0	0	0	0	0.000915071	0	-0.62669159
0	-0.10089663	0	0.16204239	0	-0.14561998	0	0.073170251	0	0
0	0	0	0	0	0	0	0	0	-0.62637515
0	-0.037932254	0	0	0	0	0	0.042621985	0	-0.14255101
0	-0.10006077	0	0.16279351	0	-0.14734909	0	0.07291058	0	0
0	0	0	0	0	0	0	0	0	-0.62639026
0	0	0	0	0	0.030211622	0	0.039417896	0	-0.1788686
0	0	0	0	0	0.031603632	0	0.040119099	0	-0.17895187
0	-0.036128415	0	0	0	0	0	0	0	-0.14824539
0	-0.037085439	0	0	0	0	0	0	0	-0.14364483
0	-0.029442405	0	0	0	0.01295	0	0	0	-0.15117253
0	-0.040593916	0	0	0	0	0	0.040999711	0	0
0	-0.037641448	0	0	0	0	0	0	0	-0.14700322
0	0	0	0	0	0	0	0.041208064	0	-0.17822691
0	0	0	0	0	0	0	0.039830054	0	-0.18097229
0	-0.042101399	0	0	0	0	0	0.041782447	0	0
0	-0.030573706	0	0	0	0.013759408	0	0	0	-0.1501881
0	0	0	0	0	-0.005377896	0	0	0	-0.62652078
0	0	0	0	0	0	0	0	0	-0.62649416
0	0	0	0	0	0	0	-0.001346482	0	-0.62625353
0	0	0	0	0	-0.008102928	0	0.003535471	0	-0.62636062
0	0	0	0	0	-0.005238904	0	0	0	-0.62636674
0	0	0	0	0	0	0	0.040540202	0	-0.18113389
0	0	0	0	0	0	0	0.000709166	0	-0.62626001
0	0	0	0	0	0.032305252	0	0	0	-0.18281667
0	0	0	0	0	0.033798044	0	0	0	-0.17918943

0	0	0	0	0	-0.00691886	0	0.004166529	0	-0.62611743
0	0	0	0	0	-0.003849412	0	0	0	-0.62625239
0	-0.051133371	0	0	0	-0.028628874	0	0.016935253	0	-0.12877797
0	0	0	0	0	0	0	0.001916216	0	-0.62636295
0	-0.046310036	0	0	0	-0.017632546	0	0	0	-0.13402102
0	0	0	0	0	0.033794466	0	0	0	-0.18302736
0	-0.052022171	0	0	0	-0.027856036	0	0.017677639	0	-0.12822738
0	-0.10267141	0	0.12185383	0	-0.098159552	0	0	0	0
0	-0.046583859	0	0	0	-0.02033377	0	0	0	-0.13495147
0	-0.10546911	0	0.12458039	0	-0.10718497	0	0	0	0
0	-0.10621737	0	0.12413046	0	-0.10512232	0	0	0	0
0	-0.047395931	0	0	0	-0.01901809	0	0	0	-0.13442338
0	-0.041081311	0	0	0	0	0	0	0	0
0	-0.040112759	0	0	0	0	0	0	0	0
0	0	0	0	0	0.030220443	0	0.038180498	0	0
0	-0.037962802	0	0	0	0	0	0	0	-0.14070116
0	-0.038034524	0	0	0	0	0	-0.001388523	0	-0.14154212
0	0	0	0	0	0	0	0	0	-0.18218668
0	-0.041649246	0	0	0	0	0	0	0	0
0	-0.035432028	0	0	0	0.008954897	0	0	0	0
0	0	0	0	0	0.031635256	0	0.038865356	0	0
0	-0.036683199	0	0	0	0	0	-1.57E-05	0	-0.14186698
0	-0.03656172	0	0	0	0.009795249	0	0	0	0
0	-0.038254131	0	0	0	0	0	0.001230488	0	-0.14058561
0	-0.055859682	0	0	0	-0.030545307	0	0.01779039	0	0
0	-0.050739312	0	0	0	-0.018714899	0	0	0	0
0	-0.056819094	0	0	0	-0.029550706	0	0.018635531	0	0
0	0	0	0	0	0	0	0.039977628	0	0
0	0	0	0	0	0	0	0.038718914	0	0
0	-0.050972646	0	0	0	-0.021373582	0	0	0	0
0	0	0	0	0	0.033790171	0	0	0	0
0	0	0	0	0	0.032248257	0	0	0	0
0	0	0	0	0	0	0	0.039430047	0	0
0	-0.051793152	0	0	0	-0.0200435	0	0	0	0
0	0	0	0	0	0.0337357	0	0	0	0
0	-0.041880194	0	0	0	0	0	0	0	0
0	-0.041981375	0	0	0	0	0	-0.001559049	0	0
0	-0.040606095	0	0	0	0	0	9.15E-05	0	0
0	-0.042190942	0	0	0	0	0	0.001340088	0	0
0	0	0	0	0	-0.004398492	0	0	0	-0.17680099
0	0	0	0	0	-0.006129529	0	0.001450111	0	-0.17739832
0	0	0	0	0	0	0	-0.000539953	0	-0.17636795
0	0	0	0	0	0	0	0	0	0
0	0	0	0	0	-0.006530429	0	0.002911897	0	-0.17659994
0	0	0	0	0	-0.005463393	0	0.003526846	0	-0.17677461
0	0	0	0	0	-0.003963994	0	0	0	0
0	0	0	0	0	-0.005625811	0	0.000740847	0	0

0	-0.092700902	0	0.16670796	0	-0.10463429	0	0	0	0
0	0	0	0	0	0	0	-0.000632299	0	0
0	0	0	0	0	-0.005902227	0	0.002471321	0	0
0	0	0	0	0	-0.004718445	0	0.003001769	0	0
0	-0.10187441	0	0.056602166	0	0	0	0.038768538	0	0
0	0	0	0.16676893	0	-0.19831397	0	0.06378541	0	-0.62614382
0	-0.10106558	0	0.055713172	0	0	0	0.038118778	0	0
0	-0.10069469	0	0.059805354	0	0	0	0	0	0
0	0	0	0.12202366	0	-0.14168024	0	0.090045963	0	-0.62641264
0	-0.13320192	0	0	0	-0.062723345	0	0.042706571	0	0
0	-0.099842208	0	0.05994241	0	0	0	0	0	0
0	-0.13219093	0	0	0	-0.057003317	0	0.048531053	0	0
0	-0.13220469	0	0	0	-0.061114048	0	0.045157834	0	0
0	-0.13168115	0	0	0	-0.058522437	0	0.047932633	0	0
0	-0.13156965	0	0	0	-0.062432209	0	0.044714054	0	0
0	-0.098876998	0	0.059073886	0	0	0	0	0	0
0	-0.099201713	0	0.052921392	0	0	0	0	0	0
0	0	0	0.14412429	0	-0.17855409	0	0.060540454	0	-0.15737378
0	-0.099516988	0	0.052370973	0	0	0	0.002826375	0	0
0	0	0	0.14967514	0	-0.18548259	0	0	0	-0.62618444
0	-0.018724167	0	0.16745029	0	-0.21123958	0	0.062449684	0	-0.1343811
0	-0.13136525	0	0	0	-0.053874747	0	0	0	0
0	-0.098475887	0	0.051472826	0	0	0	0.001766129	0	0
0	0	0	0.16653776	0	-0.19788612	0	0.060300041	0	-0.15437039
0	-0.10330873	0	0.050844653	0	0	0	-0.007744097	0	0
0	-0.12887104	0	0	0	-0.039873833	0	0	0	0
0	-0.035272888	0	0	0	0	0	0.057416957	0	-0.62687259
0	-0.13086119	0	0	0	-0.055536092	0	0	0	0
0	-0.13150237	0	0	0	-0.054078289	0	0	0	0
0	-0.033573682	0	0	0	0	0	0.056356918	0	-0.62688475
0	-0.016227188	0	0.17396434	0	-0.21746068	0	0	0	-0.62627498
0	-0.025972731	0	0	0	0.018568694	0	0.052001021	0	-0.62689185
0	0	0	0.17269405	0	-0.2053761	0	0	0	-0.62619836
0	-0.024694132	0	0	0	0.017616802	0	0.051447464	0	-0.62689742
0	-0.034445874	0	0	0	0	0	0.067758373	0	-0.62687505
0	-0.11959194	0	0	0	0	0	0.044635479	0	0
0	0	0	0.1234727	0	-0.14404501	0	0.083867883	0	-0.16637484
0	-0.034987557	0	0	0	0	0	0.058304753	0	-0.6268576
0	0	0	0	0	0.027747333	0	0.041414878	0	-0.62658757
0	-0.033304533	0	0	0	0	0	0.057672874	0	-0.62686438
0	0	0	0	0	0.040234167	0	0.054869342	0	-0.62647216
0	0	0	0	0	0.038483609	0	0.054360347	0	-0.62647459
0	0	0	0.14585618	0	-0.18019832	0	0.059413496	0	0
0	-0.11856817	0	0	0	0	0	0.046320001	0	0
0	0	0	0	0	0.023774698	0	0.037611193	0	-0.62660158
0	0	0	0	0	0.025050975	0	0.03768767	0	-0.62659578
0	0	0	0.1590472	0	-0.1598345	0	0.10930138	0	-0.6266059

0	-0.024659285	0	0.16852443	0	-0.21634596	0	0.061911554	0	0
0	-0.003104603	0	0.12052862	0	-0.14123407	0	0.08907638	0	-0.16053967
0	-0.1174444	0	0	0	0	0	0.045661187	0	0
0	0	0	0.16750922	0	-0.19887146	0	0.059308779	0	0
0	0	0	0	0	0	0	0.055109101	0	-0.62663234
0	0.014670464	0	0.15929743	0	-0.14903512	0	0.11290096	0	-0.62704656
0	0	0	0	0	0	0	0.054400941	0	-0.62664403
0	-0.087695563	0	0.09517096	0	0	0	0.039210831	0	0
0	0	0	0	0	0	0	0.054968337	0	-0.62664461
0	0	0	0	0	0	0	0.056799564	0	-0.62661995
0	-0.086656467	0	0.09535233	0	0	0	0.040846052	0	0
0	-0.017377894	0	0.1489048	0	-0.19627038	0	0	0	-0.14094735
0	0	0	0	0	0.046205536	0	0	0	-0.62652403
0	-0.11893657	0	0	0	0	0	0	0	0
0	0	0	0.14930999	0	-0.18491627	0	0	0	-0.16020458
0	0	0	0.12436667	0	-0.14422467	0	0.083834611	0	0
0	-0.012335007	0	0	0	0.044352026	0	0	0	-0.62686199
0	-0.01107783	0	0	0	0.043116414	0	0	0	-0.62686039
0	0	0	0	0	0.041499772	0	0	0	-0.62638475
0	-0.008071591	0	0.12041435	0	-0.14324054	0	0.090341726	0	0
0	0	0	0	0	0.043333712	0	0	0	-0.62639201
0	-0.085396736	0	0.095120234	0	0	0	0.040351859	0	0
0	-0.11635624	0	0	0	0	0	0.011964502	0	0
0	-0.087726857	0	0.087356569	0	0	0	0.015740235	0	0
0	-0.032757908	0	0	0	0	0	0	0	-0.62674425
0	-0.034487628	0	0	0	0	0	0	0	-0.62673336
0	0	0	0	0	0.043460236	0	0	0	-0.6263503
0	-0.014771373	0	0.17307617	0	-0.21539583	0	0	0	-0.14132173
0	-0.017458509	0	0	0	0.030028929	0	0	0	-0.6267931
0	-0.018914729	0	0	0	0.031006385	0	0	0	-0.62643525
0	-0.085809568	0	0.098860038	0	0	0	0	0	0
0	-0.026375889	0	0	0	0.015962893	0	0.044216872	0	-0.15431957
0	-0.033777478	0	0	0	0	0	0.055316585	0	-0.14792438
0	-0.004239685	0	0	0	0.058305346	0	0	0	-0.62685373
0	-0.023427012	0	0	0	0.020658408	0	0.048370642	0	-0.15299692
0	-0.02504291	0	0	0	0.015119413	0	0.043787287	0	-0.15542736
0	-0.033735934	0	0	0	0	0	0	0	-0.62669387
0	0	0	0	0	0.040595018	0	0.049745689	0	-0.18046464
0	0	0	0	0	0.038815503	0	0.049294936	0	-0.18026971
0	-0.022933489	0	0.15024175	0	-0.20130612	0	0	0	0
0	0	0	0	0	0.040585492	0	0.051654334	0	-0.1755636
0	-0.034126555	0	0	0	0	0	0.052965074	0	-0.14745659
0	-0.020310276	0	0	0	0.027256286	0	0.053549179	0	-0.15622554
0	-0.032425488	0	0	0	0	0	0.052395571	0	-0.14891226
0	-0.019029933	0	0	0	0.026278439	0	0.052979916	0	-0.15735869
0	0	0	0	0	0.025944143	0	0.034974531	0	-0.17843087
0	-0.033524261	0	0	0	0	0	0.054920566	0	-0.14303619

0	0	0	0.15083649	0	-0.18632868	0	0	0	0
0	0.012824193	0	0.15917447	0	-0.15337443	0	0.10237711	0	-0.17373392
0	0	0	0	0	0	0	0	0	-0.62651559
0	-0.020340994	0	0.17407835	0	-0.22015456	0	0	0	0
0	0	0	0	0	0	0	0.051709952	0	-0.17939621
0	-0.038019855	0	0	0	0	0	0.055767361	0	0
0	-0.03116138	0	0	0	0.014802695	0	0.045524817	0	0
0	-0.028252349	0	0	0	0.019422882	0	0.049620925	0	0
0	-0.029809661	0	0	0	0.013944171	0	0.045102238	0	0
0	-0.01113456	0	0	0	0.043953022	0	0	0	-0.17239345
0	-0.038350686	0	0	0	0	0	0.052211459	0	0
0	-0.036646544	0	0	0	0	0	0.051641163	0	0
0	-0.026663086	0	0	0	0.023093765	0	0.052919713	0	0
0	0	0	0	0	0.043519115	0	0	0	-0.18187621
0	-0.037760448	0	0	0	0	0	0.054072961	0	0
0	-0.025383389	0	0	0	0.022098165	0	0.052349779	0	0
0	-0.017593198	0	0	0	0.032032818	0	0	0	-0.16530288
0	-0.016282222	0	0	0	0.030994801	0	0	0	-0.1663316
0	0	0	0	0	0.040585306	0	0.048643719	0	0
0	0	0	0	0	0.038806224	0	0.048193003	0	0
0	0	0	0	0	0.040595005	0	0.050425662	0	0
0	0	0	0	0	0.026698907	0	0.03464989	0	0
0	-0.033136735	0	0	0	0	0	0	0	-0.14983886
0	-0.016412979	0	0	0	0.032924178	0	0	0	-0.16036236
0	0.00819823	0	0.15994975	0	-0.15550004	0	0.10417373	0	0
0	0	0	0	0	0	0	0.05061099	0	0
0	-0.015360193	0	0	0	0.043649648	0	0	0	0
0	-0.023672242	0	0	0	0.028022903	0	0	0	0
0	-0.022366682	0	0	0	0.026962918	0	0	0	0
0	-0.031494756	0	0	0	0	0	0	0	-0.62617297
0	0	0	0	0	0.043452799	0	0	0	0
0	-0.037158403	0	0	0	0	0	0	0	0
0	-0.022469372	0	0	0	0.028924006	0	0	0	0
0	0	0	0.099843796	0	-0.099860191	0	0	0	-0.62627933
0	0	0	0.18765694	0	-0.14432075	0	0.034339416	0	0
0	0	0	0.18712078	0	-0.1426374	0	0.035098342	0	0
0	0	0	0.18403318	0	-0.12171467	0	0.051910783	0	0
0	0	0	0.19035431	0	-0.14365824	0	0	0	0
0	0.02918352	0	0.099652874	0	-0.078809122	0	0	0	-0.62609783
0	0	0	0.18288369	0	-0.12249353	0	0.051781793	0	0
0	0	0	0.20030685	0	-0.15336241	0	0	0	0
0	0	0	0.18285815	0	-0.12109697	0	0.05254423	0	0
0	0	0	0.20097444	0	-0.15555467	0	0.030453052	0	0
0	0	0	0.20033996	0	-0.15207977	0	0	0	0
0	0	0	0.18784389	0	-0.14324387	0	0.030086054	0	0
0	0	0	0.20092322	0	-0.15426321	0	0.031275383	0	0
0	0	0	0.18886995	0	-0.12982304	0	0.04467787	0	0

0	0.023437672	0	0.10657308	0	-0.095015742	0	0	0	-0.20075215
0	0	0	0.20314578	0	-0.15458512	0	0	0	0
0	-0.11084518	0	0.16346966	0	-0.27402152	0	0.067585592	0	0
0	-0.028911956	0	0	0	-0.073058831	0	0.072789757	0	-0.6269071
0	-0.10615778	0	0.18182172	0	-0.28647743	0	0.068655062	0	0
0	0	0	0	0	-0.051802116	0	0.073051894	0	-0.62645881
0	0.019148367	0	0.10618202	0	-0.096044037	0	0	0	0
0	0	0	0.17404717	0	-0.092737406	0	0	0	0
0	0	0	0	0	-0.059397476	0	0.061946661	0	-0.62645192
0	0	0	0.025792234	0	0	0	0.074226689	0	-0.62630113
0	-0.027358077	0	0	0	-0.072065707	0	0.06873565	0	-0.16065141
0	-0.025238333	0	0	0	-0.068559472	0	0.072093712	0	-0.16160084
0	0	0	0	0	-0.051594342	0	0.069856903	0	-0.18712743
0	-0.10867826	0	0.16997904	0	-0.28033454	0	0	0	0
0	0.0145514	0	0	0	0	0	0.073763774	0	-0.62672098
0	0	0	0	0	0	0	0.074536013	0	-0.62647642
0	0.039861609	0	0.044346214	0	0	0	0.067509979	0	-0.21829929
0	-0.032189275	0	0	0	-0.074026901	0	0.070101385	0	0
0	-0.031521659	0	0	0	-0.072923186	0	0.071473797	0	0
0	-0.022233491	0	0	0	-0.069003988	0	0	0	-0.62674382
0	0.06212316	0	0.16412632	0	-0.091225949	0	0	0	-0.62633994
0	0.034738493	0	0.037288189	0	0	0	0.0224866	0	-0.62700112
0	0	0	0	0	-0.053858045	0	0	0	-0.62631692
0	0	0	0	0	-0.051679588	0	0.068722989	0	0
0	0.044584036	0	0.047482199	0	0	0	0	0	-0.62698698
0	0	0	0.025892555	0	0	0	0.071120263	0	-0.18566948
0	0.040614497	0	0.043353826	0	0	0	0	0	-0.62624948
0	-0.002896949	0	0	0	-0.038668447	0	0	0	-0.6264951
0	0	0	0.021853976	0	0	0	0.032542477	0	-0.62632726
0	0.035168152	0	0.042915578	0	0	0	0.066533571	0	0
0	0.015709825	0	0	0	0	0	0.070553235	0	-0.20405074
0	0.014481754	0	0	0	0	0	0.033762903	0	-0.62696995
0	-0.020745574	0	0	0	-0.067587956	0	0	0	-0.1700804
0	0.036502911	0	0.038774416	0	0	0	0.025532101	0	-0.21250673
0	0	0	0.02701671	0	0	0	0	0	-0.62628226
0	-0.12494574	0	0	0	-0.043009224	0	0.060406392	0	0
0	0	0	0.026583615	0	0	0	0.070008974	0	0
0	-0.12562777	0	0	0	-0.04356527	0	0.060698927	0	0
0	0.045602904	0	0.048089987	0	0	0	0	0	-0.22690082
0	-0.12495279	0	0	0	-0.045310521	0	0.060334249	0	0
0	0	0	0.11151498	0	0	0	0.036606086	0	0
0	0	0	0.11263482	0	0	0	0.037303725	0	0
0	0.018923437	0	0	0	0	0	0	0	-0.62698138
0	-0.10496453	0	0.12096466	0	-0.19339769	0	0.12789525	0	0
0	0.011825982	0	0	0	0	0	0.069689078	0	0
0	0	0	0.11520187	0	0	0	0	0	0
0	0	0	0.12767367	0	0	0	0.037149555	0	0

0	-0.12044039	0	0	0	-0.016922126	0	0.082511027	0	0
0	-0.026601641	0	0	0	-0.07161856	0	0	0	0
0	0.066494769	0	0.089318813	0	0	0	0.073630545	0	-0.6272351
0	-0.11558391	0	0	0	0	0	0.057812042	0	0
0	0.032172601	0	0.037588428	0	0	0	0.025940716	0	0
0	0	0	0.11478321	0	0	0	0.031908139	0	0
0	0.041147447	0	0.046725592	0	0	0	0	0	0
0	0	0	0.10789189	0	0	0	0.000969019	0	0
0	0.060844563	0	0.079995264	0	0	0	0.04570455	0	-0.62711494
0	-0.12531029	0	0	0	-0.039904211	0	0	0	0
0	0.067679346	0	0.089689112	0	0	0	0.069964938	0	-0.23763937
0	0.073394352	0	0.094292856	0	0	0	0	0	-0.6270644
0	0.063284635	0	0.088889248	0	0	0	0.069039294	0	0
0	0	0	0.067934948	0	0	0	0.081467299	0	-0.62641083
0	-0.13184374	0	0	0	-0.14444777	0	0.081934004	0	0
0	0	0	0	0	-0.04801452	0	0.087064484	0	-0.62650877
0	-0.013105967	0	0	0	-0.056803627	0	0.089737825	0	-0.62663814
0	0.005992828	0	0	0	-0.0261321	0	0.11265142	0	-0.62722755
0	0.020790453	0	0	0	0	0	0.08696606	0	-0.62717833
0	-0.010994492	0	0	0	-0.055167339	0	0.085218269	0	-0.17549975
0	0	0	0.20767032	0	-0.24343616	0	0.06555106	0	0
0	0	0	0	0	0.024409576	0	0.049300123	0	0
0	-0.017428222	0	0	0	-0.05958159	0	0.084670056	0	0
0	0	0	0	0	0.0257804	0	0.05004425	0	0
0	0	0	0	0	0	0	0.050692776	0	0
0	0	0	0	0	0.001093633	0	0.025569057	0	0
0	0	0	0	0	0.028471725	0	0	0	0
0	-0.006669738	0	0	0	-0.055004383	0	0	0	-0.62682069
0	0	0	0	0	0.026053988	0	0.044063781	0	0
0	-0.09069274	0	-0.00153861	0	0	0	0.087678584	0	0
0	0	0	0	0	0.034402266	0	0.060276813	0	0
0	0	0	0.13868122	0	-0.097915284	0	0.030481187	0	-0.62632467
0	-0.019008274	0	0.15280809	0	-0.10797629	0	0.04707139	0	-0.62675513
0	0	0	0.13855187	0	-0.09653027	0	0.031156103	0	-0.62613819
0	-0.020175144	0	0.1522246	0	-0.10669976	0	0.047833306	0	-0.62674854
0	0	0	0.15344049	0	-0.10463188	0	0.034986113	0	-0.62633098
0	0	0	0.15308421	0	-0.10037718	0	0.039217859	0	-0.62620757
0	0	0	0.15445052	0	-0.10807965	0	0.034128816	0	-0.62620341
0	0	0	0.15291043	0	-0.10310037	0	0.035291079	0	-0.62632952
0	0	0	0.15387036	0	-0.10620228	0	0.034815761	0	-0.62622544
0	0	0	0.13428435	0	-0.084718574	0	0	0	-0.62619572
0	-0.017586441	0	0.1258413	0	-0.0795458	0	0	0	-0.62668199
0	0	0	0.13102073	0	-0.076615789	0	0	0	-0.62625492
0	0	0	0.13821538	0	-0.09281934	0	0	0	-0.62617389
0	-0.025766774	0	0.15833855	0	-0.12671062	0	0	0	-0.62604167
0	0	0	0.1340823	0	-0.082932548	0	0	0	-0.62627457
0	-0.018593797	0	0.12528142	0	-0.077612838	0	0	0	-0.62617561

0	-0.026650073	0	0.1578507	0	-0.12544011	0	0	0	-0.62658974
0	0	0	0.13814345	0	-0.091380118	0	0	0	-0.62617466
0	0	0	0.15667068	0	-0.10830035	0	0	0	-0.62625531
0	0	0	0.15706599	0	-0.10718179	0	0	0	-0.62616049
0	0	0	0.15610869	0	-0.10637698	0	0	0	-0.62625277
0	0	0	0.15368664	0	-0.084080054	0	0	0	-0.62614295
0	-0.006911612	0	0.15386235	0	-0.08627217	0	0	0	-0.6267257
0	-0.007979584	0	0.1532433	0	-0.084544118	0	0	0	-0.62671863
0	-0.026413445	0	0.13237022	0	-0.1040383	0	0.035191208	0	-0.12785427
0	-0.027380989	0	0.13213342	0	-0.10289206	0	0.036114606	0	-0.1270477
0	-0.000393585	0	0.1536757	0	-0.0716175	0	0	0	-0.62622398
0	0	0	0.13804695	0	-0.095221582	0	0.027942869	0	-0.15152893
0	0	0	0.1394205	0	-0.099105135	0	0.025735119	0	-0.1539287
0	0	0	0.13557756	0	-0.091903395	0	0.031069232	0	-0.15237961
0	-0.025807177	0	0.15530167	0	-0.12552662	0	0.033082458	0	-0.1233442
0	-0.019469029	0	0.152771	0	-0.11052908	0	0.039397926	0	-0.13414367
0	-0.017629571	0	0.15237958	0	-0.10472227	0	0.044070428	0	-0.1319341
0	0	0	0.13935425	0	-0.097782304	0	0.026331102	0	-0.15411105
0	-0.020699425	0	0.15217651	0	-0.10935034	0	0.040023327	0	-0.13325035
0	-0.026964886	0	0.15473123	0	-0.12432801	0	0.03380556	0	-0.12243872
0	0	0	0.15302998	0	-0.10224841	0	0.032485249	0	-0.15195193
0	-0.019556077	0	0.12923055	0	-0.088058499	0	0	0	-0.14027254
0	-0.026251334	0	0.1371886	0	-0.10902959	0	0	0	-0.12732876
0	0	0	0.13545698	0	-0.090482415	0	0.031778265	0	-0.1524939
0	0	0	0.15414331	0	-0.10605308	0	0.031479616	0	-0.14823897
0	0	0	0.15399887	0	-0.10732423	0	0.029943324	0	-0.15141939
0	0	0	0.13431947	0	-0.083725378	0	0	0	-0.15702755
0	-0.017043943	0	0.12608662	0	-0.078413108	0	0	0	-0.13888329
0	-0.027307371	0	0.13706494	0	-0.10812008	0	0	0	-0.12644414
0	-0.020441743	0	0.12882551	0	-0.08639833	0	0	0	-0.1397782
0	0	0	0.15340158	0	-0.10541224	0	0.03058024	0	-0.15172155
0	-0.031261801	0	0.13236709	0	-0.10577444	0	0.036156916	0	0
0	0	0	0.13784701	0	-0.092332597	0	0	0	-0.1557041
0	0	0	0.13774658	0	-0.090824438	0	0	0	-0.15225301
0	-0.024211264	0	0.15734521	0	-0.12463297	0	0	0	-0.12864383
0	-0.024423841	0	0.15775958	0	-0.12358369	0	0	0	-0.12450147
0	-0.032269809	0	0.13205299	0	-0.10455723	0	0.03706339	0	0
0	-0.025364906	0	0.15680751	0	-0.12340669	0	0	0	-0.12785738
0	0	0	0.13777002	0	-0.090864814	0	0	0	-0.1559294
0	0	0	0.081747749	0	0	0	0.034439619	0	-0.62637041
0	0	0	0.15610176	0	-0.10617308	0	0	0	-0.15196175
0	0	0	0.082719877	0	0	0	0.035115737	0	-0.62636641
0	-0.031707363	0	0.15633189	0	-0.13040962	0	0.032563977	0	0
0	-0.024032311	0	0.15340629	0	-0.11225497	0	0.040715925	0	0
0	-0.03286764	0	0.15576543	0	-0.1292098	0	0.033298052	0	0
0	0	0	0.13910742	0	-0.095644988	0	0.027791361	0	0
0	-0.022281248	0	0.15306603	0	-0.10659699	0	0.045390148	0	0

0	-0.03217044	0	0.13856625	0	-0.11438029	0	0	0	0
0	0	0	0.14034029	0	-0.099154919	0	0.025554208	0	0
0	-0.006839543	0	0.15337479	0	-0.085138987	0	0	0	-0.14936134
0	-0.025276551	0	0.15281576	0	-0.11106504	0	0.041355139	0	0
0	0	0	0.085006787	0	0	0	0	0	-0.62629438
0	0	0	0.13729317	0	-0.093463788	0	0.029950385	0	0
0	-0.023999869	0	0.12903858	0	-0.089007195	0	0	0	0
0	-0.03303677	0	0.13840974	0	-0.11319378	0	0	0	0
0	0	0	0.083987511	0	0	0	0	0	-0.62631263
0	0	0	0.14027726	0	-0.097826403	0	0.026150252	0	0
0	0	0	0.15383376	0	-0.10226483	0	0.032247728	0	0
0	0	0	0.08500024	0	0	0	0	0	-0.62613817
0	0	0	0.13717075	0	-0.09202211	0	0.030660252	0	0
0	-0.021511562	0	0.12591981	0	-0.079419971	0	0	0	0
0	-0.024888284	0	0.12862671	0	-0.087324507	0	0	0	0
0	-0.029952525	0	0.15835274	0	-0.12941983	0	0	0	0
0	0	0	0.15505705	0	-0.10694286	0	0.030373575	0	0
0	-0.030153057	0	0.15871058	0	-0.12828975	0	0	0	0
0	0.00287471	0	0.085011775	0	0	0	0	0	-0.62681479
0	0	0	0.15496409	0	-0.10826346	0	0.028957246	0	0
0	0	0	0.13527091	0	-0.083889363	0	0	0	0
0	-0.031101027	0	0.15781281	0	-0.12816781	0	0	0	0
0	0.001350569	0	0.085423064	0	0	0	0	0	-0.62680045
0	0	0	0.13935525	0	-0.092387535	0	0	0	0
0	0	0	0.13943762	0	-0.093838358	0	0	0	0
0	0	0	0.15436588	0	-0.1063553	0	0.029590695	0	0
0	0	0	0.13935949	0	-0.09237087	0	0	0	0
0	0	0	0.15695662	0	-0.1070642	0	0	0	0
0	-0.010866227	0	0.15404079	0	-0.08600245	0	0	0	0
0	0	0	0.0828424	0	0	0	0.031781972	0	-0.16348623
0	0	0	0.081913161	0	0	0	0.030406592	0	-0.16660982
0	0	0	0.10858103	0	0	0	0.03601747	0	-0.62633576
0	0	0	0.082897216	0	0	0	0.03101668	0	-0.16657281
0	0.002502545	0	0.082822821	0	0	0	0.031705031	0	-0.16594381
0	0	0	0.10810109	0	0	0	0.034847703	0	-0.62635809
0	0.014773647	0	0.11222422	0	0	0	0.035690815	0	-0.6269113
0	0.016680689	0	0.11229776	0	0	0	0.034940383	0	-0.62692426
0	0.000962373	0	0.083171456	0	0	0	0.032480154	0	-0.16447388
0	0	0	0.10763574	0	0	0	0.034317996	0	-0.62636379
0	0	0	0.084933757	0	0	0	0	0	-0.16697946
0	0.003074821	0	0.086131164	0	0	0	0	0	-0.16611873
0	0	0	0.11076554	0	0	0	0	0	-0.62618699
0	0.003943072	0	0.085463135	0	0	0	0	0	-0.17061562
0	0.017226882	0	0.11568485	0	0	0	0	0	-0.62682076
0	0.002405321	0	0.085874117	0	0	0	0	0	-0.1692803
0	0.018092847	0	0.11479329	0	0	0	0	0	-0.62685627
0	0.016172274	0	0.1147552	0	0	0	0	0	-0.62684578

0	0	0	0.077107101	0	0	0	-0.017904435	0	-0.62626245
0	0	0	0.083673587	0	0	0	0.030586955	0	0
0	0	0	0.074266244	0	0	0	0	0	-0.62627849
0	0	0	0.077249079	0	0	0	-0.013940571	0	-0.62625126
0	0	0	0.07614467	0	0	0	-0.015023493	0	-0.62624508
0	-0.004168596	0	0.075352547	0	0	0	-0.016234508	0	-0.62672399
0	-0.002620304	0	0.074833814	0	0	0	-0.017737586	0	-0.62675237
0	0	0	0.082730419	0	0	0	0.029326279	0	0
0	-0.007105994	0	0.069817343	0	0	0	0	0	-0.62678682
0	0	0	0.083722725	0	0	0	0.02994172	0	0
0	-0.005883029	0	0.072147694	0	0	0	0	0	-0.62674106
0	-0.002197823	0	0.081754556	0	0	0	0.030824979	0	0
0	-0.004514835	0	0.071334306	0	0	0	0	0	-0.6267441
0	-0.003751128	0	0.08211425	0	0	0	0.031609211	0	0
0	0	0	0.085663235	0	0	0	0	0	0
0	-0.001526219	0	0.085039641	0	0	0	0	0	0
0	-0.000703471	0	0.08431264	0	0	0	0	0	0
0	-0.002198266	0	0.084803073	0	0	0	0	0	0
0	0.007541018	0	0.097428818	0	0	0	-0.007943821	0	-0.62686651
0	0	0	0.096053944	0	0	0	-0.005616877	0	-0.62625957
0	0.00590669	0	0.094559547	0	0	0	0	0	-0.6267522
0	0.016663464	0	0.11309601	0	0	0	0.032211415	0	-0.17268059
0	0	0	0.10839409	0	0	0	0.031490471	0	-0.1613723
0	0.006976357	0	0.09757423	0	0	0	-0.005965135	0	-0.6259875
0	0.015789588	0	0.11237981	0	0	0	0.031129893	0	-0.17575976
0	0.017715139	0	0.11246515	0	0	0	0.030442007	0	-0.17739601
0	0.009147161	0	0.097517274	0	0	0	-0.006969317	0	-0.62676515
0	0.017835618	0	0.11531596	0	0	0	0	0	-0.1779217
0	0	0	0.077454963	0	0	0	-0.016425925	0	-0.16200721
0	-0.002337552	0	0.076408402	0	0	0	-0.018774981	0	-0.16150397
0	-0.004406127	0	0.072526	0	0	0	0	0	-0.15654731
0	-0.00246292	0	0.076476613	0	0	0	-0.015154111	0	-0.16007846
0	-0.001017644	0	0.075955687	0	0	0	-0.016347494	0	-0.16132772
0	0	0	0.10897517	0	0	0	0.030461439	0	0
0	0.012126115	0	0.11246833	0	0	0	0.031288377	0	0
0	0.011272123	0	0.11178442	0	0	0	0.030311953	0	0
0	0.013212158	0	0.11186605	0	0	0	0.029597954	0	0
0	-0.11017575	0	0.15272964	0	-0.18173041	0	0.037089624	0	0
0	-0.11070715	0	0.15237355	0	-0.18008148	0	0.037749943	0	0
0	-0.10756515	0	0.16752461	0	-0.19062659	0	0.037247572	0	0
0	0.013412781	0	0.11467169	0	0	0	0	0	0
0	-0.10712949	0	0.16803834	0	-0.19263249	0	0.037382377	0	0
0	-0.037582779	0	0	0	0	0	0.044607407	0	-0.62683862
0	-0.10782136	0	0.16731756	0	-0.19073765	0	0.037973228	0	0
0	0.009172156	0	0.098650377	0	0	0	-0.0086432	0	-0.1674276
0	-0.039083485	0	0	0	0	0	0.045374407	0	-0.62683045
0	0	0	0.07831759	0	0	0	-0.016656343	0	0

0	-0.006614731	0	0.075666441	0	0	0	-0.018796948	0	0
0	-0.10955045	0	0.15555755	0	-0.18021577	0	0	0	0
0	-0.008771526	0	0.071710346	0	0	0	0	0	0
0	-0.006804237	0	0.0756134	0	0	0	-0.014921036	0	0
0	-0.005241461	0	0.075107137	0	0	0	-0.016166129	0	0
0	-0.10946053	0	0.15589581	0	-0.18227405	0	0	0	0
0	-0.10706387	0	0.17086489	0	-0.19144934	0	0	0	0
0	-0.11009854	0	0.15559212	0	-0.18069377	0	0	0	0
0	0	0	0	0	0.029984236	0	0.041977734	0	-0.62641736
0	-0.10751288	0	0.13343596	0	-0.13818174	0	0.060864396	0	0
0	0	0	0	0	0.031397164	0	0.042659552	0	-0.62644167
0	-0.10799212	0	0.13403399	0	-0.14107654	0	0.060273422	0	0
0	-0.10866665	0	0.1337569	0	-0.13954432	0	0.060792041	0	0
0	-0.037167198	0	0	0	0	0	0	0	-0.62674909
0	-0.038121506	0	0	0	0	0	0	0	-0.62670908
0	-0.031239707	0	0	0	0.011511196	0	0	0	-0.62675191
0	0.005119238	0	0.098513241	0	0	0	-0.008168445	0	0
0	-0.038693748	0	0	0	0	0	0	0	-0.62673832
0	0	0	0	0	0	0	0.043717185	0	-0.6261885
0	0	0	0	0	0	0	0.042430028	0	-0.62647231
0	-0.032376146	0	0	0	0.012322618	0	0	0	-0.62675103
0	0	0	0	0	0	0	0.043141681	0	-0.62647404
0	-0.053471445	0	0	0	-0.030961143	0	0.019403816	0	-0.62606383
0	-0.054155573	0	0	0	-0.029901385	0	0.020295201	0	-0.62669234
0	0	0	0	0	0.032214719	0	0	0	-0.6263802
0	0	0	0	0	0.033728506	0	0	0	-0.62636579
0	-0.047781175	0	0	0	-0.018140857	0	0	0	-0.62623924
0	0	0	0	0	0.033702095	0	0	0	-0.62638
0	-0.047874959	0	0	0	-0.020835922	0	0	0	-0.62668398
0	-0.048691381	0	0	0	-0.019512292	0	0	0	-0.62667851
0	-0.038988161	0	0	0	0	0	0	0	-0.6266797
0	-0.10059614	0	0.16231092	0	-0.14582494	0	0.072168285	0	0
0	-0.039114412	0	0	0	0	0	-0.000175833	0	-0.62669007
0	-0.037625394	0	0	0	0	0	0.041901007	0	-0.14271333
0	-0.036592642	0	0	0	0	0	0.040454341	0	-0.14656346
0	0	0	0	0	0	0	0	0	-0.62654271
0	-0.031812354	0	0	0	0.00924772	0	0.041235891	0	-0.14845619
0	-0.038103518	0	0	0	0	0	0.041161808	0	-0.14528629
0	-0.037736329	0	0	0	0	0	0.001531433	0	-0.6266994
0	-0.039319627	0	0	0	0	0	0.002781507	0	-0.62669569
0	-0.0329615	0	0	0	0.010001944	0	0.041952877	0	-0.14743171
0	0	0	0	0	0.031754284	0	0.039349436	0	-0.1788181
0	0	0	0	0	0.030305751	0	0.038035939	0	-0.18159197
0	0	0	0	0	0.031735911	0	0.038673116	0	-0.18177874
0	-0.03733024	0	0	0	0	0	0	0	-0.14709256
0	-0.029736524	0	0	0	0.012785399	0	0	0	-0.15449361
0	-0.030036852	0	0	0	0.014200272	0	0	0	-0.15055639

0	-0.041800823	0	0	0	0	0	0.041052128	0	0
0	-0.04074591	0	0	0	0	0	0.039712794	0	0
0	-0.030894696	0	0	0	0.013623947	0	0	0	-0.15359393
0	0	0	0	0	0	0	0.039835268	0	-0.18096674
0	-0.038041494	0	0	0	0.005123222	0	0.040634656	0	0
0	-0.042276693	0	0	0	0	0	0.04042001	0	0
0	-0.039189257	0	0	0	0.005897388	0	0.041357205	0	0
0	0	0	0	0	0.033881891	0	0	0	-0.18281878
0	0	0	0	0	-0.00706468	0	0.003200474	0	-0.62652209
0	0	0	0	0	-0.004396638	0	0	0	-0.62636581
0	0	0	0	0	0	0	0.000902428	0	-0.62636201
0	0	0	0	0	-0.007375841	0	0.004847918	0	-0.62636256
0	-0.051729346	0	0	0	-0.027888903	0	0.016598033	0	-0.1284107
0	-0.10590286	0	0.12467077	0	-0.1060511	0	0	0	0
0	0	0	0	0	-0.006239651	0	0.005392817	0	-0.62627605
0	-0.051237349	0	0	0	-0.029133783	0	0.016480729	0	-0.12921739
0	-0.047393348	0	0	0	-0.019589273	0	0	0	-0.13413857
0	-0.052245264	0	0	0	-0.028249964	0	0.017205223	0	-0.12843079
0	-0.04131663	0	0	0	0	0	0	0	0
0	0	0	0	0	0.031760761	0	0.038112775	0	0
0	0	0	0	0	0.030304785	0	0.036921858	0	0
0	-0.036040322	0	0	0	0.010213965	0	0	0	0
0	-0.035746098	0	0	0	0.008785903	0	0	0	0
0	-0.037966709	0	0	0	0	0	0.000252051	0	-0.14065998
0	0	0	0	0	0.03174774	0	0.037551835	0	0
0	-0.036896165	0	0	0	0.009627213	0	0	0	0
0	-0.056573786	0	0	0	-0.029635489	0	0.017584841	0	0
0	-0.055976865	0	0	0	-0.030753816	0	0.017540869	0	0
0	0	0	0	0	0	0	0.038705228	0	0
0	-0.051791968	0	0	0	-0.020616386	0	0	0	0
0	-0.056993182	0	0	0	-0.029858284	0	0.018265226	0	0
0	0	0	0	0	0.03382293	0	0	0	0
0	-0.041903947	0	0	0	0	0	0.000359448	0	0
0	0	0	0	0	-0.005526068	0	0.002532593	0	-0.17656323
0	0	0	0	0	-0.004895149	0	0.002089855	0	0
0	0	0	0.14428938	0	-0.1789705	0	0.063118517	0	-0.62631717
0	-0.10158463	0	0.05680053	0	0	0	0.038031839	0	0
0	-0.020685452	0	0.16746898	0	-0.21300174	0	0.065302268	0	-0.62673733
0	-0.10025146	0	0.056944925	0	0	0	0.039948614	0	0
0	0	0	0.16650465	0	-0.19812405	0	0.062935077	0	-0.62632356
0	-0.0992398	0	0.056150088	0	0	0	0.0393819	0	0
0	0	0	0.12416448	0	-0.14534836	0	0.085922203	0	-0.62631589
0	-0.099493233	0	0.060120881	0	0	0	0	0	0
0	-0.005605794	0	0.12073953	0	-0.14353781	0	0.091733515	0	-0.62639262
0	-0.13191751	0	0	0	-0.056698104	0	0.047855965	0	0
0	-0.13195259	0	0	0	-0.061136965	0	0.04422451	0	0
0	-0.13141029	0	0	0	-0.056548306	0	0.04906025	0	0

0	-0.13079804	0	0	0	-0.057970403	0	0.048517138	0	0
0	-0.021530486	0	0.14347895	0	-0.19253659	0	0.061923754	0	-0.13694767
0	-0.099216221	0	0.052515099	0	0	0	0.001816397	0	0
0	0	0	0.14417659	0	-0.17866254	0	0.059786333	0	-0.15894497
0	-0.019218045	0	0.14918262	0	-0.19813365	0	0	0	-0.62613458
0	0	0	0.1496535	0	-0.1855368	0	0	0	-0.62620047
0	-0.018850975	0	0.1671552	0	-0.21115314	0	0.061517551	0	-0.13627861
0	-0.13116454	0	0	0	-0.05374498	0	0	0	0
0	-0.03499565	0	0	0	0	0	0.056522316	0	-0.62687827
0	-0.028603233	0	0	0	0.013949779	0	0.04698802	0	-0.62689357
0	-0.025724044	0	0	0	0.018503167	0	0.051012112	0	-0.62689251
0	-0.027578536	0	0	0	0.013016245	0	0.046510877	0	-0.62621467
0	-0.027446219	0	0.14494591	0	-0.19796505	0	0.061377054	0	0
0	-0.016377729	0	0.17335108	0	-0.21717872	0	0	0	-0.62658862
0	-0.035306149	0	0	0	0	0	0.055771398	0	-0.6268857
0	-0.033595628	0	0	0	0	0	0.055192606	0	-0.62689653
0	-0.02246273	0	0	0	0.025415476	0	0.056402141	0	-0.62692662
0	0	0	0	0	0.040302877	0	0.052326941	0	-0.62625483
0	0	0	0	0	0.038570021	0	0.051918439	0	-0.62649234
0	-0.021182081	0	0	0	0.024432783	0	0.055832064	0	-0.62693297
0	0	0	0	0	0.040316208	0	0.054195535	0	-0.62625954
0	-0.034703413	0	0	0	0	0	0.057689022	0	-0.62685603
0	-0.11833009	0	0	0	0	0	0.045662074	0	0
0	0	0	0	0	0.024865518	0	0.036797812	0	-0.62622947
0	-0.004741092	0	0.12246126	0	-0.14567539	0	0.085169964	0	-0.16219878
0	0	0	0.14590298	0	-0.18029376	0	0.058776859	0	0
0	0.010411793	0	0.15926526	0	-0.15553281	0	0.105157	0	-0.62670836
0	-0.024783595	0	0.16827554	0	-0.21629502	0	0.061085136	0	0
0	0	0	0	0	0	0	0.054303441	0	-0.62635996
0	-0.086322292	0	0.095613869	0	0	0	0.040161806	0	0
0	-0.01762321	0	0.14888838	0	-0.19647465	0	0	0	-0.14353988
0	-0.012686799	0	0	0	0.043612261	0	0	0	-0.62617925
0	0	0	0	0	0.043420848	0	0	0	-0.62637049
0	-0.019434514	0	0	0	0.030571735	0	0	0	-0.62681983
0	-0.01828273	0	0	0	0.029481384	0	0	0	-0.62648618
0	-0.009664581	0	0.12230175	0	-0.14756836	0	0.086525415	0	0
0	-0.034455748	0	0	0	0	0	0	0	-0.62606056
0	-0.018169681	0	0	0	0.031482024	0	0	0	-0.62684992
0	-0.026102722	0	0	0	0.015974362	0	0.04331492	0	-0.15439222
0	-0.020676996	0	0	0	0.027108759	0	0.050999928	0	-0.16071482
0	0	0	0	0	0.040718126	0	0.049121752	0	-0.18029694
0	-0.019394068	0	0	0	0.026128253	0	0.050485518	0	-0.16174964
0	-0.019789959	0	0	0	0.027720443	0	0.052880866	0	-0.15658794
0	-0.033843023	0	0	0	0	0	0.052373724	0	-0.14756432
0	-0.023154302	0	0.15022212	0	-0.20150739	0	0	0	0
0	-0.030893962	0	0	0	0.014799756	0	0.04463651	0	0
0	-0.027059214	0	0	0	0.022950359	0	0.050473127	0	0

0	-0.038086743	0	0	0	0	0	0.051628802	0	0
0	-0.025759638	0	0	0	0.021944971	0	0.049952932	0	0
0	-0.026152198	0	0	0	0.023549531	0	0.052259406	0	0
0	-0.017081414	0	0	0	0.032455978	0	0	0	-0.16559238
0	0	0	0	0	0.040709881	0	0.048047833	0	0
0	-0.023168738	0	0	0	0.028441759	0	0	0	0
0	0.021975779	0	0.10624328	0	-0.095511024	0	0	0	-0.62628718
0	0	0	0.18731697	0	-0.14264657	0	0.034274128	0	0
0	0	0	0.19743254	0	-0.15247379	0	0.034448933	0	0
0	0	0	0.19739884	0	-0.15117955	0	0.03520802	0	0
0	0	0	0.18302824	0	-0.12141332	0	0.051380983	0	0
0	0	0	0.2001766	0	-0.15181998	0	0	0	0
0	0	0	0.20084221	0	-0.15404208	0	0.030448744	0	0
0	-0.10947688	0	0.16311679	0	-0.27264777	0	0.068481978	0	0
0	-0.02969285	0	0	0	-0.074103889	0	0.071475274	0	-0.62654247
0	-0.027480603	0	0	0	-0.070484125	0	0.074892174	0	-0.62664142
0	0	0	0	0	-0.051858512	0	0.072421794	0	-0.62646046
0	0.038196104	0	0.043375502	0	0	0	0.070115551	0	-0.62687851
0	0	0	0.02576719	0	0	0	0.073656021	0	-0.62625954
0	-0.025308155	0	0	0	-0.068668761	0	0.07139733	0	-0.16301608
0	0.014734383	0	0	0	0	0	0.073243881	0	-0.62712684
0	0.039847727	0	0.044320435	0	0	0	0.066931114	0	-0.2195269
0	-0.031588477	0	0	0	-0.073018129	0	0.070892923	0	0
0	-0.022470135	0	0	0	-0.069248768	0	0	0	-0.62676656
0	0.035027123	0	0.037775675	0	0	0	0.027275606	0	-0.6269911
0	0.0444359	0	0.047359031	0	0	0	0	0	-0.62701012
0	0.035148144	0	0.04289624	0	0	0	0.066077151	0	0
0	-0.12534056	0	0	0	-0.043267767	0	0.060133616	0	0
0	0	0	0.11266037	0	0	0	0.036467017	0	0
0	0.066320399	0	0.089046277	0	0	0	0.072628467	0	-0.62724967
0	-0.013489899	0	0	0	-0.057185412	0	0.088015452	0	-0.62630225
0	0	0	0	0	0.025954728	0	0.049207696	0	0
0	-0.028536117	0	0.13261347	0	-0.10623476	0	0.037858593	0	-0.62672425
0	-0.029539258	0	0.13229623	0	-0.10503162	0	0.038762009	0	-0.62671234
0	0	0	0.13881995	0	-0.096887565	0	0.030135597	0	-0.6263066
0	-0.027764844	0	0.15529616	0	-0.12718889	0	0.035962821	0	-0.62667689
0	-0.021622355	0	0.15282895	0	-0.11257351	0	0.042133913	0	-0.62675175
0	0	0	0.14007384	0	-0.10043289	0	0.027856339	0	-0.62631558
0	-0.020143823	0	0.15247082	0	-0.10705464	0	0.046656455	0	-0.62628162
0	0	0	0.13573096	0	-0.092273954	0	0.033668825	0	-0.62631312
0	-0.022853636	0	0.15224837	0	-0.11139901	0	0.042771048	0	-0.62675288
0	-0.029069696	0	0.15471799	0	-0.12604352	0	0.03665625	0	-0.62636061
0	0	0	0.14001112	0	-0.099111953	0	0.028451377	0	-0.62631522
0	0	0	0.1531718	0	-0.10342264	0	0.034356168	0	-0.62632825
0	0	0	0.13560706	0	-0.090855863	0	0.034363791	0	-0.62617124
0	-0.028064068	0	0.13742823	0	-0.11063017	0	0	0	-0.62638544
0	-0.021068433	0	0.12900828	0	-0.08851071	0	0	0	-0.62630624

0	0	0	0.15407731	0	-0.10621513	0	0.034100445	0	-0.62643495
0	0	0	0.15395971	0	-0.10753021	0	0.032596626	0	-0.62631714
0	-0.029148225	0	0.1373114	0	-0.10977715	0	0	0	-0.6263818
0	-0.018641566	0	0.12585583	0	-0.07881136	0	0	0	-0.6260857
0	-0.02178305	0	0.12863011	0	-0.086776233	0	0	0	-0.62669251
0	0	0	0.13452877	0	-0.083860883	0	0	0	-0.62627579
0	0	0	0.15336222	0	-0.10562025	0	0.033231215	0	-0.62631655
0	-0.025915425	0	0.15757906	0	-0.12618922	0	0	0	-0.62662064
0	0	0	0.13817868	0	-0.092727249	0	0	0	-0.62625704
0	-0.02637214	0	0.15796028	0	-0.12518255	0	0	0	-0.62604702
0	0	0	0.13806981	0	-0.091211573	0	0	0	-0.62624999
0	-0.02707076	0	0.15703799	0	-0.12495372	0	0	0	-0.62661529
0	0	0	0.13810119	0	-0.091260229	0	0	0	-0.62625377
0	0	0	0.15626481	0	-0.10647309	0	0	0	-0.62611891
0	-0.008271015	0	0.15348856	0	-0.085564051	0	0	0	-0.62623648
0	-0.027112696	0	0.13236907	0	-0.10312818	0	0.034983556	0	-0.12713203
0	-0.027684228	0	0.1338985	0	-0.10766108	0	0.032132219	0	-0.12925566
0	-0.028186193	0	0.13469131	0	-0.1097071	0	0.03272624	0	-0.1256112
0	-0.028728338	0	0.13363492	0	-0.10657676	0	0.032914809	0	-0.12849482
0	-0.02928947	0	0.1345237	0	-0.10886602	0	0.033472911	0	-0.12466704
0	0	0	0.13956739	0	-0.098094501	0	0.025359129	0	-0.15386937
0	-0.020375158	0	0.15244154	0	-0.10955153	0	0.038999665	0	-0.13329902
0	-0.026406362	0	0.15492766	0	-0.12401801	0	0.033060032	0	-0.12280086
0	0	0	0.13541551	0	-0.090314271	0	0.031023561	0	-0.15235074
0	-0.02601258	0	0.15476997	0	-0.12512063	0	0.031471479	0	-0.12642275
0	0	0	0.13564536	0	-0.091857278	0	0.029630625	0	-0.15519574
0	-0.027196923	0	0.15420155	0	-0.12390548	0	0.032144483	0	-0.12559671
0	-0.020366443	0	0.12929046	0	-0.087305526	0	0	0	-0.13945217
0	-0.026535492	0	0.13713138	0	-0.10912206	0	0	0	-0.13057925
0	-0.026811162	0	0.13701993	0	-0.10771161	0	0	0	-0.12676939
0	0	0	0.13552432	0	-0.090393648	0	0.030287054	0	-0.15539885
0	0	0	0.15361024	0	-0.10546338	0	0.029853611	0	-0.15150811
0	-0.027636016	0	0.13701093	0	-0.10825578	0	0	0	-0.12975826
0	-0.031970306	0	0.13236052	0	-0.10484496	0	0.03596665	0	0
0	0	0	0.13763357	0	-0.090639333	0	0	0	-0.1557232
0	-0.024829503	0	0.15694763	0	-0.12308571	0	0	0	-0.1281407
0	-0.032486198	0	0.13384857	0	-0.10927554	0	0.033198035	0	0
0	0	0	0.082777054	0	0	0	0.034355031	0	-0.6263621
0	-0.034070834	0	0.13611754	0	-0.11491195	0	0.032200602	0	0
0	-0.033546706	0	0.13359078	0	-0.10818453	0	0.03398237	0	0
0	-0.035184466	0	0.13591501	0	-0.11399833	0	0.032947795	0	0
0	0	0	0.081844297	0	0	0	0.03302267	0	-0.62637447
0	0	0	0.082832329	0	0	0	0.033633661	0	-0.62620127
0	0.000775183	0	0.081993053	0	0	0	0.034397683	0	-0.62652139
0	-0.032320141	0	0.15594411	0	-0.12888963	0	0.032552383	0	0
0	-0.000590687	0	0.082360221	0	0	0	0.035212257	0	-0.62689238
0	-0.031946201	0	0.15585789	0	-0.13006004	0	0.03105683	0	0

0	-0.024950856	0	0.15308575	0	-0.11126743	0	0.040324873	0	0
0	-0.033115111	0	0.15527027	0	-0.12882955	0	0.031740216	0	0
0	-0.032518565	0	0.13833501	0	-0.11269787	0	0	0	0
0	-0.032260354	0	0.13848512	0	-0.1141879	0	0	0	0
0	0	0	0.1404907	0	-0.098139254	0	0.025178114	0	0
0	0	0	0.085028875	0	0	0	0	0	-0.62630357
0	-0.024819286	0	0.12910059	0	-0.088228085	0	0	0	0
0	0	0	0.13714938	0	-0.091880752	0	0.029901988	0	0
0	-0.033354604	0	0.13835942	0	-0.11329169	0	0	0	0
0	0	0	0.13736278	0	-0.093428408	0	0.028626544	0	0
0	0.0017394	0	0.085585842	0	0	0	0	0	-0.62680351
0	-0.030574773	0	0.15795443	0	-0.12785423	0	0	0	0
0	0	0	0.13725235	0	-0.091995723	0	0.029284879	0	0
0	0.002609593	0	0.084909359	0	0	0	0	0	-0.62684118
0	0	0	0.1545761	0	-0.10640663	0	0.02886778	0	0
0	0.001063633	0	0.085324889	0	0	0	0	0	-0.62683074
0	0	0	0.13928553	0	-0.092222833	0	0	0	0
0	0	0	0.0829534	0	0	0	0.030285581	0	-0.16639608
0	0	0	0.10826982	0	0	0	0.034148992	0	-0.62635995
0	0.015259531	0	0.11247389	0	0	0	0.034887186	0	-0.62694841
0	0.001377369	0	0.083392688	0	0	0	0.031692133	0	-0.16468692
0	0.014093668	0	0.1117843	0	0	0	0.03383751	0	-0.62645423
0	0.002367734	0	0.082865121	0	0	0	0.030256101	0	-0.16866762
0	0.016341872	0	0.11188757	0	0	0	0.033189884	0	-0.62710713
0	0.000816075	0	0.083225273	0	0	0	0.030971637	0	-0.16728907
0	0.002796155	0	0.086036365	0	0	0	0	0	-0.1694068
0	0.016590826	0	0.1149903	0	0	0	0	0	-0.6268484
0	0	0	0.077272089	0	0	0	-0.01493274	0	-0.62639018
0	-0.004116641	0	0.075465458	0	0	0	-0.017461713	0	-0.62608833
0	0	0	0.083773316	0	0	0	0.029237314	0	0
0	-0.00333278	0	0.082337058	0	0	0	0.030821793	0	0
0	-0.005689464	0	0.072036366	0	0	0	0	0	-0.62673674
0	-0.003955528	0	0.075579808	0	0	0	-0.01346735	0	-0.62672713
0	-0.00240131	0	0.075080758	0	0	0	-0.014706092	0	-0.62672771
0	-0.00233554	0	0.081791699	0	0	0	0.029488247	0	0
0	-0.003905269	0	0.082158908	0	0	0	0.030211455	0	0
0	-0.001653935	0	0.085026312	0	0	0	0	0	0
0	0.016228753	0	0.1126667	0	0	0	0.030385873	0	-0.17594048
0	0.00744255	0	0.097728619	0	0	0	-0.006995664	0	-0.62633015
0	-0.002098206	0	0.07664951	0	0	0	-0.016266553	0	-0.16020297
0	0.011706531	0	0.11207098	0	0	0	0.029568033	0	0
0	-0.11042447	0	0.15231671	0	-0.1796367	0	0.037053083	0	0
0	-0.10986758	0	0.15265595	0	-0.18147177	0	0.037306342	0	0
0	-0.11048865	0	0.15231163	0	-0.17988385	0	0.037908944	0	0
0	-0.10748303	0	0.16753363	0	-0.1905827	0	0.037300956	0	0
0	-0.039046855	0	0	0	0	0	0.044604379	0	-0.62632393
0	-0.037733813	0	0	0	0	0	0.043240053	0	-0.6268554

0	-0.034028555	0	0	0	0.007422482	0	0.044067256	0	-0.62660335
0	-0.039252919	0	0	0	0	0	0.043953625	0	-0.62685233
0	-0.035047431	0	0	0	0.008214529	0	0.044809134	0	-0.62684977
0	-0.006449142	0	0.075761745	0	0	0	-0.015978603	0	0
0	-0.10977604	0	0.15559157	0	-0.18039791	0	0	0	0
0	0	0	0	0	0.031524073	0	0.041907207	0	-0.62645213
0	0	0	0	0	0.030081714	0	0.040639086	0	-0.62645392
0	-0.10835918	0	0.13404615	0	-0.13975815	0	0.05978718	0	0
0	0	0	0	0	0.03151689	0	0.041268584	0	-0.62645486
0	-0.038377162	0	0	0	0	0	0	0	-0.6267407
0	-0.032129473	0	0	0	0.012683836	0	0	0	-0.62614902
0	-0.031749983	0	0	0	0.01128577	0	0	0	-0.62637173
0	-0.032702519	0	0	0	0.012172395	0	0	0	-0.626775
0	0	0	0	0	0	0	0.042424623	0	-0.62647691
0	-0.054103077	0	0	0	-0.030033184	0	0.01920951	0	-0.62631209
0	0	0	0	0	0.0337907	0	0	0	-0.62637758
0	-0.053311766	0	0	0	-0.031079358	0	0.01921769	0	-0.62670224
0	-0.054322075	0	0	0	-0.03019539	0	0.019941217	0	-0.62669515
0	-0.048689753	0	0	0	-0.020090388	0	0	0	-0.62668582
0	-0.03779602	0	0	0	0	0	0.040466368	0	-0.14540873
0	-0.032411381	0	0	0	0.010488039	0	0.041196413	0	-0.14782921
0	-0.039034223	0	0	0	0	0	0.00180084	0	-0.62669514
0	-0.031962816	0	0	0	0.009252362	0	0.039827876	0	-0.15111183
0	-0.033120665	0	0	0	0.010025101	0	0.04049	0	-0.15018025
0	0	0	0	0	0.031876563	0	0.037936766	0	-0.18160521
0	-0.030350347	0	0	0	0.014060103	0	0	0	-0.15391826
0	-0.041971132	0	0	0	0	0	0.039724203	0	0
0	-0.038645061	0	0	0	0.006379601	0	0.040600361	0	0
0	-0.038197858	0	0	0	0.005114769	0	0.039339517	0	0
0	-0.03935663	0	0	0	0.005904715	0	0.040004051	0	0
0	0	0	0	0	-0.006414488	0	0.004441623	0	-0.62615277
0	-0.051968132	0	0	0	-0.02827707	0	0.016236686	0	-0.12849717
0	0	0	0	0	0.031871734	0	0.03682239	0	0
0	-0.03637151	0	0	0	0.010059039	0	0	0	0
0	-0.056685102	0	0	0	-0.029848671	0	0.017287534	0	0
0	-0.023640857	0	0.14353952	0	-0.19439219	0	0.064743013	0	-0.62646624
0	0	0	0.14433919	0	-0.17907588	0	0.062408277	0	-0.62632805
0	-0.099934514	0	0.057148295	0	0	0	0.039231654	0	0
0	-0.021016375	0	0.16718002	0	-0.21297467	0	0.064377936	0	-0.62641763
0	-0.007231523	0	0.1226139	0	-0.14785884	0	0.087925556	0	-0.62621062
0	-0.1310979	0	0	0	-0.056198415	0	0.04839643	0	0
0	-0.021602434	0	0.14352824	0	-0.19269591	0	0.061156987	0	-0.13849067
0	-0.019337711	0	0.14916031	0	-0.19831208	0	0	0	-0.62634698
0	-0.028322732	0	0	0	0.013953848	0	0.046085696	0	-0.62689424
0	-0.022886189	0	0	0	0.025250555	0	0.053866674	0	-0.62686334
0	-0.021551073	0	0	0	0.024276626	0	0.053379212	0	-0.62696025
0	-0.035022387	0	0	0	0	0	0.055176088	0	-0.62688973

0	0	0	0	0	0.040448241	0	0.051718477	0	-0.62637681
0	-0.022238801	0	0	0	0.025801017	0	0.055685736	0	-0.62638439
0	-0.027518242	0	0.14499308	0	-0.19811055	0	0.060717611	0	0
0	-0.019248842	0	0	0	0.030905247	0	0	0	-0.62607902
0	-0.020176844	0	0	0	0.027580841	0	0.050354668	0	-0.16102872
0	-0.026549118	0	0	0	0.023404749	0	0.049834438	0	0
0	0	0	0.19732114	0	-0.1509847	0	0.034382897	0	0
0	-0.027552282	0	0	0	-0.070589853	0	0.074239544	0	-0.6266462
0	0.038323638	0	0.043370851	0	0	0	0.069621637	0	-0.62716988
0	-0.029233374	0	0.1326134	0	-0.10531889	0	0.037659476	0	-0.62672007
0	-0.029712965	0	0.13406161	0	-0.10974379	0	0.034885823	0	-0.62678426
0	-0.030376329	0	0.13470718	0	-0.11146609	0	0.03554888	0	-0.62623939
0	-0.030888537	0	0.13379705	0	-0.10869224	0	0.035673346	0	-0.62665272
0	-0.031376364	0	0.1345572	0	-0.11060809	0	0.036323869	0	-0.62644711
0	-0.022528923	0	0.15251348	0	-0.11159786	0	0.041748333	0	-0.62674501
0	-0.028530114	0	0.15489988	0	-0.12572006	0	0.035902926	0	-0.62633916
0	-0.027993626	0	0.15479107	0	-0.12680517	0	0.034384722	0	-0.62669538
0	0	0	0.14022735	0	-0.099421173	0	0.027486065	0	-0.62632196
0	0	0	0.13558844	0	-0.09069229	0	0.033620297	0	-0.62631421
0	-0.029288828	0	0.15419359	0	-0.12562328	0	0.035030499	0	-0.62643237
0	0	0	0.13579861	0	-0.092242008	0	0.032268614	0	-0.62634357
0	-0.028357208	0	0.13737379	0	-0.11073461	0	0	0	-0.62641272
0	-0.028626924	0	0.13725239	0	-0.10929661	0	0	0	-0.62638973
0	-0.021946621	0	0.12907814	0	-0.087754241	0	0	0	-0.62623225
0	0	0	0.1356805	0	-0.090772183	0	0.032928261	0	-0.62632491
0	0	0	0.15357529	0	-0.10567846	0	0.032515052	0	-0.62630686
0	-0.029461494	0	0.13725051	0	-0.10986262	0	0	0	-0.6264033
0	-0.026536433	0	0.15717965	0	-0.12463538	0	0	0	-0.62661196
0	0	0	0.13802655	0	-0.091105288	0	0	0	-0.62624845
0	-0.028404199	0	0.13391792	0	-0.10678622	0	0.031891945	0	-0.12854373
0	-0.028748639	0	0.13452194	0	-0.10838663	0	0.032707748	0	-0.12505696
0	-0.0283294	0	0.13474535	0	-0.10973637	0	0.03126179	0	-0.12835478
0	-0.029445223	0	0.13458288	0	-0.10889069	0	0.031961206	0	-0.12749384
0	-0.026647564	0	0.15439103	0	-0.12358317	0	0.031418179	0	-0.12591811
0	0	0	0.13550215	0	-0.090252768	0	0.029554248	0	-0.15523038
0	-0.02711386	0	0.13696227	0	-0.10778676	0	0	0	-0.13006553
0	-0.033210608	0	0.13387157	0	-0.10839084	0	0.032957204	0	0
0	-0.034638204	0	0.13594399	0	-0.11357341	0	0.032186301	0	0
0	0	0	0.082885626	0	0	0	0.032902611	0	-0.62637441
0	-0.034224936	0	0.13618337	0	-0.11497179	0	0.030846598	0	0
0	-0.000350531	0	0.082565113	0	0	0	0.034386561	0	-0.62652842
0	-0.035340615	0	0.13600968	0	-0.11409327	0	0.031542616	0	0
0	0.000821491	0	0.082049259	0	0	0	0.033027366	0	-0.62691532
0	-0.032566133	0	0.15546327	0	-0.12850645	0	0.031009967	0	0
0	-0.000741109	0	0.082410042	0	0	0	0.033747867	0	-0.62690786
0	-0.032842159	0	0.13830427	0	-0.11281967	0	0	0	0
0	0	0	0.13723329	0	-0.091819325	0	0.028562515	0	0

0	0.001450171	0	0.08549018	0	0	0	0	0	-0.62681706
0	0.014778953	0	0.11208184	0	0	0	0.033126477	0	-0.62693674
0	0.001227486	0	0.083442721	0	0	0	0.030206522	0	-0.16746467
0	-0.00359893	0	0.075728083	0	0	0	-0.014523465	0	-0.62672589
0	-0.003492301	0	0.082379311	0	0	0	0.029449893	0	0
0	-0.11017505	0	0.15236723	0	-0.17960976	0	0.037227975	0	0
0	-0.039198846	0	0	0	0	0	0.043210947	0	-0.62636133
0	-0.034638419	0	0	0	0.008669357	0	0.044030884	0	-0.62658309
0	-0.034051454	0	0	0	0.007451763	0	0.042723562	0	-0.62687438
0	-0.035210532	0	0	0	0.008230288	0	0.043387937	0	-0.62687027
0	0	0	0	0	0.031641411	0	0.040540461	0	-0.62645325
0	-0.032421567	0	0	0	0.012552789	0	0	0	-0.6262373
0	-0.054041341	0	0	0	-0.030214833	0	0.018970367	0	-0.62669996
0	-0.032572127	0	0	0	0.010508208	0	0.039757127	0	-0.15053771
0	-0.038812359	0	0	0	0.006385597	0	0.039272362	0	0
0	-0.023706781	0	0.14358675	0	-0.19454793	0	0.06401552	0	-0.62648374
0	-0.022631065	0	0	0	0.025662141	0	0.053195516	0	-0.62639983
0	-0.030478045	0	0.13410619	0	-0.10884823	0	0.034666965	0	-0.62672219
0	-0.03087587	0	0.13455031	0	-0.11014014	0	0.035547658	0	-0.62635475
0	-0.030424052	0	0.13479248	0	-0.1115104	0	0.03415425	0	-0.62646138
0	-0.031539735	0	0.13462225	0	-0.1106447	0	0.034849642	0	-0.62645832
0	-0.0286087	0	0.15440166	0	-0.12526067	0	0.034338024	0	-0.62669797
0	0	0	0.13565837	0	-0.090630312	0	0.03219528	0	-0.62632402
0	-0.028943382	0	0.13719512	0	-0.10938306	0	0	0	-0.62640177
0	-0.028907009	0	0.13458355	0	-0.1084146	0	0.031220701	0	-0.12783862
0	-0.034803442	0	0.13600773	0	-0.11361665	0	0.030803164	0	0
0	-0.000327299	0	0.082630695	0	0	0	0.032984248	0	-0.62691039
0	-0.034798806	0	0	0	0.008681827	0	0.042631979	0	-0.62659977
0	-0.031042359	0	0.13461586	0	-0.11017907	0	0.034098862	0	-0.62636464

SE	bsnp_rs6469674	SE	bsnp_rs13269857	SE	bsnp_rs3020106	SE	bsnp_rs3020119	SE
0	0	0	0	0	0	0	0	0
0	0	0	0.18543255	0	0	0	0	0
0	0	0	0	0	0.18527184	0	0	0
0	0	0	0	0	0	0	0	0
0	0	0	0	0	0	0	0	0
0	0	0	0	0	0	0	0	0
0	0	0	0	0	0	0	0	0
0	-0.19611356	0	0	0	0	0	0	0
0	0	0	0	0	0	0	0	0
0	0	0	0	0	0	0	0	0
0	0	0	0	0	0	0	-0.068743798	0
0	0	0	0	0	0	0	0	0
0	0	0	0	0	0	0	0	0
0	0	0	0.17132422	0	0	0	0	0
0	-0.18654683	0	0	0	0	0	0	0
0	0	0	0	0	0.1712007	0	0	0
0	-0.17500733	0	0.17144609	0	0	0	0	0
0	0	0	0	0	0.16187236	0	0	0
0	0	0	0.16087938	0	0	0	0	0
0	-0.1753491	0	0	0	0.171321	0	0	0
0	0	0	0	0	0	0	0	0
0	0	0	0	0	0	0	0	0
0	-0.17192507	0	0	0	0	0	0	0
0	-0.18171557	0	0	0	0	0	0	0
0	0	0	0.21238449	0	0	0	0	0
0	0	0	0.13571864	0	0	0	0	0
0	0	0	0	0	0.13564761	0	0	0
0	0	0	0	0	0.21332082	0	0	0
0	0	0	0	0	0	0	0	0
0	-0.18364505	0	0	0	0	0	0	0
0	0	0	0.14000914	0	0	0	0	0
0	0	0	0	0	0.13968765	0	0	0
0	0	0	0	0	0	0	0	0
0	0	0	0.17202934	0	0	0	0	0
0	0	0	0	0	0.17260481	0	0	0
0	0	0	0.14626076	0	0	0	0	0
0	0	0	0	0	0.14592393	0	0	0
0	0	0	0	0	0	0	-0.083873198	0
0	-0.21441195	0	0	0	0	0	-0.083615869	0
0	0	0	0	0	0	0	0	0
0	-0.19213182	0	0	0	0	0	0	0
0	0	0	0.17107339	0	0	0	-0.031980927	0
0	0	0	0	0	0.17061748	0	-0.033474938	0
0	0	0	0	0	0	0	0	0
0	0	0	0.14133624	0	0.045018179	0	0	0

0	0	0	0	0	0	0	0	0
0	0	0	0	0	0	0	0	0
0	0	0	0	0	0	0	0	0
0	0	0	0	0	0	0	0	0
0	0	0	0	0	0	0	0	0
0	0	0	0	0	0	0	0.024838021	0
0	0	0	0	0	0	0	0	0
0	0	0	0	0	0	0	0	0
0	0	0	0	0	0	0	0	0
0	0	0	0	0	0	0	0	0
0	0	0	0	0	0	0	-0.003662686	0
0	0	0	0	0	0	0	0	0
0	-0.25721841	0	0	0	0	0	0	0
0	0	0	0	0	0	0	0	0
0	0	0	0	0	0	0	0.025329696	0
0	0.42756158	0	0	0	0	0	0	0
0	0	0	0	0	0	0	0	0
0	0	0	0	0	0	0	0	0
0	0	0	0	0	0	0	0.028515315	0
0	0	0	0	0	0	0	-0.084600756	0
0	0	0	0	0	0	0	0	0
0	0	0	0	0	0.15153413	0	0	0
0	0	0	0.15062916	0	0	0	0	0
0	-0.15702657	0	0	0	0.15160042	0	0	0
0	-0.15714527	0	0.15069299	0	0	0	0	0
0	0	0	0	0	0	0	-0.031422343	0
0	0	0	0.12194672	0	0	0	0	0
0	0	0	0	0	0.12198914	0	0	0
0	-0.17428736	0	0.12204602	0	0	0	0	0
0	-0.17442235	0	0	0	0.1220755	0	0	0
0	0	0	0	0	0.16232476	0	0	0
0	0	0	0	0	0.18738491	0	0	0
0	0	0	0.16118121	0	0	0	0	0
0	0	0	0.18571735	0	0	0	0	0
0	0	0	0.12848585	0	0	0	0	0
0	0	0	0	0	0.12825901	0	0	0
0	0	0	0.15829086	0	0	0	0	0
0	0	0	0	0	0.15891299	0	0	0
0	-0.17143219	0	0.12870441	0	0	0	0	0
0	-0.17151074	0	0	0	0.12847327	0	0	0
0	0	0	0.13426461	0	0	0	0	0
0	0	0	0	0	0.13402757	0	0	0
0	-0.17353396	0	0.15838969	0	0	0	0	0
0	-0.17365587	0	0	0	0.15901226	0	0	0
0	-0.17215561	0	0.13431364	0	0	0	0	0
0	-0.17223925	0	0	0	0.13406735	0	0	0
0	0	0	0.14758056	0	0	0	-0.050487314	0

0	0	0	0	0	0.1474413	0	-0.051763555	0
0	0	0	0.16257116	0	0	0	0	0
0	0	0	0	0	0.16284754	0	0	0
0	-0.18899182	0	0.14784561	0	0	0	-0.050162629	0
0	-0.18956431	0	0	0	0.14770102	0	-0.051442601	0
0	0.4371302	0	0	0	0	0	0	0
0	0	0	0	0	0	0	0	0
0	0	0	0	0	0.16628027	0	0	0
0	0	0	0	0	0	0	0	0
0	0	0	0.16542816	0	0	0	0	0
0	0.44862464	0	0.17170315	0	0	0	0	0
0	0	0	0	0	0	0	0	0
0	0	0	0	0	0	0	0	0
0	-0.1863948	0	0	0	0	0	0	0
0	0	0	0	0	0.17221706	0	0.032765348	0
0	0	0	0.17159291	0	0	0	0.033486565	0
0	0.44828356	0	0	0	0.17157748	0	0	0
0	0	0	0	0	0	0	-0.005076673	0
0	0	0	0	0	0	0	0	0
0	-0.1844422	0	0	0	0	0	0	0
0	0	0	0	0	0.17965582	0	0	0
0	0	0	0.17936928	0	0	0	0	0
0	-0.18231967	0	0	0	0	0	0	0
0	0	0	0	0	0.17241425	0	0	0
0	0	0	0.1777689	0	0	0	0	0
0	0	0	0.17157181	0	0	0	0	0
0	0	0	0.12952658	0	0.042671538	0	0	0
0	-0.2007674	0	0	0	0	0	0	0
0	-0.18780396	0	0	0	0	0	-0.004512628	0
0	-0.18671928	0	0	0	0	0	0	0
0	0	0	0	0	0.17815442	0	0	0
0	-0.1519241	0	0.17918462	0	0	0	0	0
0	0	0	0	0	0.16979529	0	0	0
0	0	0	0.16939942	0	0	0	0	0
0	0	0	0.072074097	0	0.090516442	0	0	0
0	-0.17497424	0	0.12963156	0	0.042688299	0	0	0
0	0	0	0	0	0.1626553	0	0	0
0	0	0	0.16147088	0	0	0	0	0
0	-0.15116437	0	0	0	0.17960228	0	0	0
0	0	0	0	0	0	0	0	0
0	0	0	0	0	0	0	0	0
0	-0.17326612	0	0	0	0	0	0	0
0	-0.24650208	0	0	0	0	0	0	0
0	0	0	0	0	0.20067557	0	0	0
0	0	0	0.19933639	0	0	0	0	0
0	0	0	0	0	0.19453084	0	-0.049820292	0
0	0	0	0.19356488	0	0	0	-0.048206696	0

0	0	0	0	0	0	0	0	0
0	0	0	0	0	0	0	0	0
0	-0.17400024	0	0	0	0	0	0	0
0	-0.1626287	0	0	0	0	0	0	0
0	0	0	0	0	0	0	-0.033618175	0
0	0.4418885	0	0	0	0	0	0	0
0	0.45172986	0	0	0	0	0	0	0
0	-0.18361781	0	0	0	0	0	-0.033007182	0
0	0	0	0	0	0	0	0	0
0	0	0	0	0	0	0	0	0
0	0	0	0	0	0	0	-0.013388351	0
0	0	0	0	0	0	0	0	0
0	0	0	0.13814868	0	0	0	0.032400717	0
0	-0.18056555	0	0	0	0	0	0	0
0	0	0	0	0	0.13775667	0	0.031493254	0
0	-0.19862135	0	0	0	0	0	0	0
0	-0.18585162	0	0	0	0	0	-0.012916143	0
0	-0.18170452	0	0	0	0	0	0	0
0	0	0	0	0	0	0	0	0
0	0	0	0.13989547	0	0	0	0	0
0	0	0	0.13168418	0	0.082532901	0	0	0
0	0	0	0	0	0	0	0	0
0	0	0	0	0	0.13929526	0	0	0
0	0	0	0.081867911	0	0.054925878	0	0	0
0	0.44003109	0	0	0	0	0	0	0
0	0	0	0.13461955	0	0	0	0	0
0	0	0	0.13487309	0	0	0	0	0
0	0	0	0	0	0.13421674	0	0	0
0	0	0	0	0	0.13515893	0	0	0
0	-0.18098905	0	0	0	0	0	0	0
0	0	0	0	0	0	0	0	0
0	0	0	0	0	0	0	0	0
0	0	0	0	0	0	0	-0.016270847	0
0	-0.20502842	0	0	0	0	0	0	0
0	-0.18852491	0	0	0	0	0	-0.015388125	0
0	0	0	0	0	0	0	0	0
0	0	0	0	0	0	0	0	0
0	0	0	0.13921313	0	0	0	0.021356713	0
0	0	0	0.14861454	0	0	0	0	0
0	0	0	0	0	0.13883211	0	0.02100309	0
0	0	0	0	0	0.14898303	0	0	0
0	0	0	0.080116232	0	0.060988201	0	0	0
0	0	0	0.13970362	0	0	0	0	0
0	0	0	0	0	0.13938683	0	0	0
0	0	0	0	0	0	0	-0.061853829	0
0	0	0	0	0	0	0	0	0
0	0	0	0	0	0	0	0.008498236	0

0	0	0	0	0	0	0	0	0
0	0	0	0	0	0	0	-0.079308021	0
0	0	0	0.14541072	0	0	0	0.02386756	0
0	0.40894037	0	0	0	0	0	-0.083817132	0
0	0	0	0	0	0	0	0	0
0	0	0	0.15959162	0	0	0	0	0
0	0	0	0	0	0.16014385	0	0	0
0	0	0	0.17606798	0	0	0	0.013320034	0
0	0	0	0	0	0.14484629	0	0.023609654	0
0	0	0	0.089559819	0	0.083966414	0	0	0
0	0	0	0	0	0.17655448	0	0.013116311	0
0	0	0	0.085447906	0	0.061910119	0	0	0
0	-0.20776361	0	0	0	0	0	-0.061248918	0
0	-0.26478055	0	0	0	0	0	0	0
0	-0.24792751	0	0	0	0	0	-0.079430821	0
0	0	0	0	0	0	0	0	0
0	0	0	0	0	0	0	0	0
0	0	0	0	0	0	0	0	0
0	0.43156018	0	0	0	0	0	0	0
0	0	0	0	0	0	0	0	0
0	0	0	0.11793785	0	0.054143528	0	-0.032205471	0
0	0	0	0	0	0	0	0	0
0	0	0	0	0	0	0	0	0
0	0	0	0	0	0	0	0	0
0	0	0	0	0	0	0	0.007748549	0
0	0	0	0	0	0	0	0.046828501	0
0	0	0	0	0	0	0	-0.034755878	0
0	0	0	0	0	0	0	0	0
0	0	0	0	0	0	0	0	0
0	0	0	0	0	0	0	0.027209898	0
0	0	0	0	0	0	0	0.036434941	0
0	0	0	0	0	0	0	0	0
0	0	0	0	0	0	0	0	0
0	0	0	0	0	0	0	0	0
0	0	0	0	0	0	0	0	0
0	0	0	0	0	0	0	0.036463777	0
0	0	0	0	0	0	0	0.017310652	0
0	0	0	0	0	0	0	0.009518523	0
0	0	0	0	0	0	0	-0.021482461	0
0	0	0	0	0	0	0	0.029387395	0
0	0.36425604	0	0	0	0	0	0	0
0	0	0	0	0	0	0	0	0
0	0	0	0	0	0	0	0.034319965	0
0	0	0	0	0	0	0	0.0201263	0
0	0	0	0	0	0	0	0.027403036	0
0	0	0	0	0	0	0	-0.062868231	0
0	0	0	0.15253768	0	0	0	0	0

0	0	0	0	0	0.1529016	0	0	0
0	-0.14656001	0	0.15262698	0	0	0	0	0
0	-0.14680598	0	0	0	0.15298689	0	0	0
0	0.46644318	0	0	0	0.15191617	0	0	0
0	0.46646697	0	0.15101448	0	0	0	0	0
0	0	0	0	0	0.16378446	0	0	0
0	0	0	0.16348233	0	0	0	0	0
0	0	0	0	0	0.14164161	0	0	0
0	0	0	0.14064032	0	0	0	0	0
0	0	0	0.068901527	0	0.083314031	0	0	0
0	0	0	0	0	0.15469428	0	0	0
0	0	0	0	0	0.1496023	0	0	0
0	0	0	0	0	0.15250612	0	0.00287036	0
0	0	0	0.15430584	0	0	0	0	0
0	0	0	0.14829338	0	0	0	0	0
0	0	0	0.15185	0	0	0	0.003531335	0
0	-0.15461242	0	0	0	0.16394101	0	0	0
0	-0.1546166	0	0.1636655	0	0	0	0	0
0	-0.16057796	0	0	0	0.14193489	0	0	0
0	-0.16075024	0	0.14092704	0	0	0	0	0
0	-0.15696759	0	0.068745593	0	0.083535155	0	0	0
0	-0.15653229	0	0	0	0.15507177	0	0	0
0	-0.15563391	0	0	0	0.15281162	0	0.003563859	0
0	-0.15854718	0	0	0	0.15103876	0	0	0
0	-0.15659169	0	0.15465101	0	0	0	0	0
0	-0.15549817	0	0.15214011	0	0	0	0.004211782	0
0	-0.15987319	0	0.14969829	0	0	0	0	0
0	0.44921715	0	0.12238952	0	0	0	0	0
0	0.44923523	0	0	0	0.12241811	0	0	0
0	0	0	0.13245035	0	0	0	0	0
0	0	0	0	0	0.13320434	0	0	0
0	0	0	0.12737426	0	0	0	0	0
0	0	0	0	0	0.12684074	0	0	0
0	-0.13602669	0	0.13369655	0	0	0	0	0
0	-0.13469049	0	0	0	0.13447524	0	0	0
0	0	0	0.071072833	0	0.051923034	0	0	0
0	0	0	0.12059049	0	0	0	0	0
0	0	0	0.12235111	0	0	0	0.003938399	0
0	0	0	0.12263535	0	0	0	0	0
0	0	0	0	0	0.12090367	0	0	0
0	0	0	0	0	0.12227156	0	0.003082871	0
0	-0.17557788	0	0.12741684	0	0	0	0	0
0	0	0	0	0	0.12229115	0	0	0
0	-0.17567766	0	0	0	0.12688505	0	0	0
0	-0.17423768	0	0.071115079	0	0.051961294	0	0	0
0	-0.17440008	0	0.12070158	0	0	0	0	0
0	-0.17298934	0	0.12249005	0	0	0	0.004526678	0

0	-0.17445986	0	0.12277846	0	0	0	0	0
0	-0.1745054	0	0	0	0.12103861	0	0	0
0	-0.17337322	0	0	0	0.1224046	0	0.003670104	0
0	-0.17451605	0	0	0	0.12245519	0	0	0
0	0	0	0.18582226	0	0	0	0	0
0	0	0	0	0	0.18680953	0	0	0
0	0	0	0	0	0.17148997	0	0	0
0	0	0	0.15411575	0	0	0	0	0
0	0	0	0	0	0.15511963	0	0	0
0	0	0	0.16980813	0	0	0	0	0
0	0.45216219	0	0.12860833	0	0	0	0	0
0	0.45196978	0	0	0	0.12838698	0	0	0
0	0	0	0	0	0.16632004	0	0	0
0	0	0	0.16573577	0	0	0	0	0
0	0	0	0	0	0.16262086	0	0.011598918	0
0	0	0	0.067948996	0	0.095263233	0	0	0
0	0	0	0	0	0	0	0	0
0	0	0	0.072923723	0	0.11517369	0	0	0
0	0	0	0.16163455	0	0	0	0.012799408	0
0	0	0	0	0	0.19145481	0	0	0
0	0	0	0	0	0.18332282	0	0	0
0	0	0	0	0	0.16125947	0	0	0
0	0	0	0	0	0.18884005	0	0.007055325	0
0	0.45011726	0	0.15868608	0	0	0	0	0
0	0.44999598	0	0	0	0.15930399	0	0	0
0	0	0	0.16047895	0	0	0	0	0
0	0	0	0.1903859	0	0	0	0	0
0	0	0	0.13837498	0	0	0	0	0
0	0	0	0	0	0.13885901	0	0	0
0	0	0	0.18743764	0	0	0	0.008089844	0
0	0	0	0.13742278	0	0	0	0	0
0	0	0	0	0	0.13790801	0	0	0
0	0	0	0.18212405	0	0	0	0	0
0	-0.14923509	0	0	0	0	0	0	0
0	0	0	0.12875082	0	0	0	-0.015183683	0
0	0	0	0	0	0.12858645	0	-0.015573263	0
0	0.45150455	0	0.13470101	0	0	0	0	0
0	0	0	0	0	0	0	0	0
0	0.4514076	0	0	0	0.13445825	0	0	0
0	0	0	0.07183515	0	0.05768604	0	0	0
0	0	0	0.12817405	0	0	0	0	0
0	0	0	0	0	0.12795979	0	0	0
0	0	0	0.15199958	0	0	0	-0.019391628	0
0	0	0	0	0	0.1525568	0	-0.019578976	0
0	-0.13598054	0	0	0	0.13935599	0	0	0
0	-0.1374893	0	0.1388349	0	0	0	0	0
0	-0.17209381	0	0.13878833	0	0	0	0	0

0	-0.17218915	0	0	0	0.13927409	0	0	0
0	0	0	0.080943733	0	0.078789465	0	0	0
0	0	0	0.14979903	0	0	0	0	0
0	0	0	0	0	0.15049661	0	0	0
0	0	0	0.15887568	0	0	0	0	0
0	0	0	0	0	0.15988577	0	0	0
0	0	0	0.14311694	0	0	0	0	0
0	0	0	0	0	0.14359111	0	0	0
0	-0.15268934	0	0	0	0	0	0	0
0	-0.1761345	0	0.12895918	0	0	0	-0.014697476	0
0	-0.17633399	0	0	0	0.12878869	0	-0.015086975	0
0	-0.17141346	0	0.07423834	0	0.055489309	0	0	0
0	-0.17145658	0	0.1283584	0	0	0	0	0
0	0	0	0.13452536	0	0	0	-0.018080344	0
0	-0.17153511	0	0	0	0.12815146	0	0	0
0	-0.17906753	0	0.15231969	0	0	0	-0.018697545	0
0	-0.17923829	0	0	0	0.15287721	0	-0.018885181	0
0	0	0	0	0	0.1343332	0	-0.018383614	0
0	0	0	0.076629328	0	0.058677378	0	0	0
0	-0.17345451	0	0.080905022	0	0.078927669	0	0	0
0	-0.17300807	0	0.14981355	0	0	0	0	0
0	-0.17311847	0	0	0	0.1505162	0	0	0
0	-0.13894318	0	0.14430093	0	0	0	0	0
0	-0.13741695	0	0	0	0.14482197	0	0	0
0	-0.16796311	0	0.16038714	0	0	0	0	0
0	-0.1670568	0	0	0	0.16141245	0	0	0
0	0.43447717	0	0.14802899	0	0	0	-0.050295994	0
0	0.43401394	0	0	0	0.14788073	0	-0.051588566	0
0	-0.17757578	0	0.13454811	0	0	0	-0.017161007	0
0	-0.17776813	0	0	0	0.13435431	0	-0.017464296	0
0	-0.17207701	0	0.076662266	0	0.058685398	0	0	0
0	0	0	0.14129313	0	0	0	0	0
0	0	0	0	0	0.14122909	0	0	0
0	0	0	0.13970178	0	0	0	0	0
0	0	0	0	0	0.13970455	0	0	0
0	0	0	0.15603584	0	0	0	-0.051714706	0
0	0	0	0	0	0.15658677	0	-0.053082357	0
0	0	0	0.091460983	0	0.057162515	0	-0.050727429	0
0	0	0	0.097344763	0	0.066492404	0	0	0
0	0	0	0.15732405	0	0	0	0	0
0	-0.15857638	0	0.15751458	0	0	0	-0.051576054	0
0	0	0	0.16486204	0	0	0	0.007462415	0
0	0	0	0	0	0	0	0	0
0	-0.15762518	0	0	0	0.15810038	0	-0.052954596	0
0	0	0	0	0	0.15769331	0	0	0
0	0	0	0	0	0.16505982	0	0.007203787	0
0	-0.18901805	0	0.091839136	0	0.05705855	0	-0.050405355	0

0	-0.17157103	0	0	0	0	0	0	0
0	0	0	0	0	0	0	0	0
0	0	0	0	0	0	0	0	0
0	-0.18198008	0	0	0	0	0	0	0
0	0.43718695	0	0	0	0	0	0	0
0	0	0	0	0	0	0	0	0
0	0.43929753	0	0	0	0	0	0	0
0	0.44125457	0	0	0	0	0	0	0
0	0	0	0	0	0	0	0	0
0	0	0	0	0	0	0	0	0
0	0.42061846	0	0	0	0	0	0	0
0	0	0	0	0	0	0	-0.017043326	0
0	-0.18504425	0	0	0	0	0	0	0
0	0.4357538	0	0	0	0	0	-0.004937183	0
0	0.43686008	0	0	0	0	0	0	0
0	0	0	0	0	0	0	0	0
0	0	0	0	0	0	0	0	0
0	0	0	0.066902601	0	0.10049134	0	0	0
0	0	0	0	0	0.16181103	0	0	0
0	0	0	0	0	0.16591598	0	0	0
0	0	0	0	0	0	0	0	0
0	0.46925417	0	0.1785957	0	0	0	0	0
0	0	0	0	0	0.16657658	0	-0.003396652	0
0	0	0	0	0	0	0	0.00734338	0
0	-0.18395354	0	0	0	0	0	0	0
0	0	0	0.16058511	0	0	0	0	0
0	0	0	0.16504632	0	0	0	0	0
0	0.44865706	0	0.1297999	0	0.042781437	0	0	0
0	0	0	0.16564854	0	0	0	-0.002677236	0
0	0	0	0	0	0	0	0	0
0	0	0	0	0	0	0	-0.003244255	0
0	-0.19089006	0	0	0	0	0	-0.016349759	0
0	-0.21414677	0	0	0	0	0	0	0
0	0.46999017	0	0	0	0.17899265	0	0	0
0	0	0	0.1785279	0	0	0	0.024130296	0
0	0	0	0	0	0.17877911	0	0.023811179	0
0	0	0	0.084224438	0	0.089026459	0	0.033402392	0
0	0	0	0	0	0.16616301	0	0.037480848	0
0	0	0	0.16534226	0	0	0	0.038411582	0
0	-0.18106385	0	0	0	0	0	0	0
0	0	0	0.17387184	0	0	0	0	0
0	0	0	0	0	0.17524007	0	0	0
0	0	0	0	0	0.17428038	0	0	0
0	0	0	0	0	0	0	-0.004352135	0
0	0	0	0	0	0	0	0	0
0	0	0	0.1749916	0	0	0	0	0
0	0	0	0	0	0.16953643	0	0.035555354	0

0	0	0	0	0	0.15926825	0	0	0
0	0	0	0.16902679	0	0	0	0.036210648	0
0	-0.18635155	0	0	0	0	0	0	0
0	0	0	0.15800468	0	0	0	0	0
0	0	0	0.089065945	0	0.092100983	0	0	0
0	0	0	0	0	0	0	-0.008634964	0
0	-0.1951662	0	0	0	0	0	0	0
0	-0.18196041	0	0	0	0	0	0.007895877	0
0	0	0	0.071697609	0	0.10184445	0	0	0
0	-0.18256735	0	0	0	0	0	0	0
0	-0.18310017	0	0	0	0	0	-0.002644272	0
0	0	0	0	0	0	0	0	0
0	0	0	0.12907575	0	0.050008169	0	0	0
0	0	0	0	0	0.17268516	0	-0.003286749	0
0	0	0	0	0	0	0	-0.060254465	0
0	0	0	0.17178052	0	0	0	-0.00265289	0
0	0	0	0	0	0	0	0	0
0	-0.20157142	0	0	0	0	0	-0.003909015	0
0	-0.20139894	0	0	0	0	0	0	0
0	-0.18916972	0	0	0	0	0	-0.007746087	0
0	-0.15097265	0	0.12910734	0	0.051424992	0	0	0
0	0	0	0.079802822	0	0.091402168	0	0	0
0	0	0	0	0	0.16824335	0	0	0
0	-0.17810706	0	0	0	0	0	-0.059679556	0
0	0	0	0.16763351	0	0	0	0	0
0	0	0	0.072491324	0	0.091087753	0	0	0
0	-0.23547443	0	0	0	0	0	0	0
0	0.37502733	0	0	0	0	0	0	0
0	0.45025302	0	0	0	0	0	0	0
0	-0.2246156	0	0	0	0	0	0	0
0	0	0	0	0	0	0	0	0
0	0	0	0	0	0	0	-0.017425381	0
0	0	0	0	0	0	0	0	0
0	0	0	0	0	0	0	0	0
0	0	0	0	0	0	0	-0.00256414	0
0	0	0	0	0	0.19542188	0	-0.024893858	0
0	0	0	0.19411777	0	0	0	-0.024467266	0
0	-0.23571825	0	0	0	0	0	0	0
0	-0.24550356	0	0	0	0	0	-0.017450624	0
0	0	0	0.090616724	0	0.11095035	0	0	0
0	-0.17272404	0	0	0	0	0	0	0
0	-0.17393237	0	0	0	0	0	-0.001983339	0
0	0.44947381	0	0	0	0	0	0	0
0	0	0	0.095240304	0	0.10035653	0	-0.048741921	0
0	0.46091451	0	0	0	0	0	0	0
0	0	0	0	0	0	0	0	0
0	0.43970658	0	0	0	0	0	-0.033535509	0

0	0	0	0	0	0	0	-0.013088588	0
0	-0.17793524	0	0	0	0	0	-0.012212319	0
0	0.44309108	0	0	0	0	0	0	0
0	0	0	0	0	0	0	0	0
0	0.42278617	0	0	0	0	0	0	0
0	0.43774974	0	0	0	0	0	-0.012549254	0
0	0.44192268	0	0	0	0	0	0	0
0	0	0	0	0	0	0	0	0
0	0	0	0	0	0	0	-0.019281064	0
0	-0.17920379	0	0	0	0	0	0	0
0	0	0	0	0	0	0	0	0
0	0	0	0	0	0	0	0	0
0	0	0	0	0	0	0	-0.013085496	0
0	0	0	0	0	0	0	0	0
0	0	0	0	0	0	0	-0.010405095	0
0	0	0	0.13235023	0	0	0	0.04089959	0
0	-0.18628096	0	0	0	0	0	-0.018653358	0
0	0	0	0	0	0.13179998	0	0.040508567	0
0	0	0	0	0	0	0	0.02613169	0
0	0	0	0.14105579	0	0	0	0.03615778	0
0	0.44260918	0	0	0	0	0	0	0
0	0	0	0.091145151	0	0.047937978	0	0.032147694	0
0	-0.18771496	0	0	0	0	0	0	0
0	0	0	0.13861821	0	0	0	0.031311701	0
0	0	0	0	0	0.14123109	0	0.035770795	0
0	-0.20241967	0	0	0	0	0	-0.012674595	0
0	-0.19814003	0	0	0	0	0	0	0
0	-0.18474433	0	0	0	0	0	-0.009499676	0
0	0	0	0	0	0.13794368	0	0.031020847	0
0	0	0	0.13493526	0	0	0	0	0
0	0	0	0	0	0.13433524	0	0	0
0	0	0	0	0	0	0	0	0
0	0	0	0.15002381	0	0	0	0	0
0	0	0	0	0	0	0	-0.016589635	0
0	0	0	0	0	0.15016947	0	0	0
0	0.41619812	0	0	0	0	0	0	0
0	0	0	0.090383759	0	0.050438412	0	0	0
0	0	0	0	0	0	0	0	0
0	0.43471072	0	0	0	0	0	-0.016102768	0
0	0	0	0	0	0	0	0	0
0	0	0	0.080011522	0	0.0556258	0	0	0
0	0	0	0.082661377	0	0.053468045	0	0	0
0	0	0	0.13110591	0	0	0	0	0
0	0	0	0	0	0.13110386	0	0	0
0	0	0	0	0	0	0	0	0
0	-0.18596738	0	0	0	0	0	-0.015705031	0
0	0	0	0	0	0	0	-0.016011852	0

0	-0.17121932	0	0	0	0	0	0	0
0	0	0	0	0	0	0	0.012540501	0
0	-0.20959512	0	0	0	0	0	-0.015125643	0
0	0	0	0	0	0	0	-0.019080245	0
0	0	0	0	0	0	0	0	0
0	0	0	0	0	0	0	0.029234651	0
0	0	0	0	0	0	0	-0.045663805	0
0	0	0	0	0	0	0	0	0
0	0	0	0.15060883	0	0	0	0.027788492	0
0	0	0	0	0	0.15095347	0	0.027662684	0
0	0	0	0	0	0	0	0	0
0	0	0	0.13820546	0	0	0	0.027683465	0
0	0	0	0	0	0.13785361	0	0.02747465	0
0	-0.25628695	0	0	0	0	0	-0.045976135	0
0	0.41574588	0	0	0	0	0	-0.061806161	0
0	0	0	0.08174348	0	0.05852544	0	0.021172398	0
0	0	0	0.079009479	0	0.071303753	0	0	0
0	0.3565036	0	0	0	0	0	0	0
0	0	0	0.15208129	0	0	0	0	0
0	0	0	0	0	0.15262584	0	0	0
0	0.37352917	0	0	0	0	0	-0.079329303	0
0	0	0	0.075362328	0	0.065503262	0	0	0
0	0	0	0.15885367	0	0	0	0.024094588	0
0	0	0	0	0	0.15937019	0	0.023820071	0
0	0	0	0	0	0	0	0.016739997	0
0	0	0	0.08615937	0	0.075478737	0	0	0
0	0	0	0.086358425	0	0.060123184	0	0.023721494	0
0	0	0	0.094091518	0	0.083576725	0	0.013557504	0
0	0	0	0	0	0	0	0.008980543	0
0	0	0	0	0	0	0	0	0
0	0	0	0	0	0	0	-0.003908776	0
0	0	0	0	0	0	0	-0.001939419	0
0	0	0	0	0	0	0	-0.012717634	0
0	0	0	0	0	0	0	-0.058401107	0
0	0	0	0	0	0	0	0.019790984	0
0	0	0	0	0	0	0	0	0
0	0	0	0	0	0	0	0.038056236	0
0	0	0	0	0	0	0	0.046515175	0
0	0	0	0	0	0	0	0	0
0	0	0	0	0	0	0	0	0
0	0	0	0	0	0	0	0.015309616	0
0	0	0	0	0	0	0	0.04106455	0
0	0	0	0	0	0	0	0.015018689	0
0	0	0	0	0	0	0	0.035381676	0
0	0	0	0	0	0	0	0.034800657	0
0	0	0	0	0	0	0	0.034589945	0
0	0	0	0	0	0	0	0.033089132	0

0	0.47700407	0	0.15293358	0	0	0	0	0
0	0.47669112	0	0	0	0.15329273	0	0	0
0	0	0	0.13374061	0	0	0	0	0
0	0	0	0	0	0.13384887	0	0	0
0	0	0	0.13877551	0	0	0	0	0
0	0	0	0	0	0.13912323	0	0	0
0	0	0	0.13492726	0	0	0	0	0
0	0	0	0	0	0.13515901	0	0	0
0	0	0	0.14622545	0	0	0	-0.019408436	0
0	0	0	0	0	0.14649921	0	-0.019660076	0
0	-0.14533999	0	0.13408919	0	0	0	0	0
0	-0.14553949	0	0	0	0.13419434	0	0	0
0	-0.15092041	0	0.13904996	0	0	0	0	0
0	0	0	0.087397918	0	0.066397834	0	0	0
0	-0.15110984	0	0	0	0.13940157	0	0	0
0	0	0	0.15183846	0	0	0	0	0
0	-0.14487778	0	0.13489078	0	0	0	0	0
0	0	0	0	0	0.15249268	0	0	0
0	-0.14509961	0	0	0	0.13511191	0	0	0
0	-0.15212159	0	0.14652125	0	0	0	-0.018753094	0
0	-0.15235905	0	0	0	0.14683614	0	-0.018695618	0
0	-0.14654057	0	0.087477491	0	0.066405332	0	0	0
0	-0.1447949	0	0.15326524	0	0	0	0	0
0	-0.14421516	0	0	0	0.15394138	0	0	0
0	0	0	0.15285856	0	0	0	0	0
0	0	0	0	0	0.15310542	0	0	0
0	0.46891692	0	0	0	0.16422933	0	0	0
0	0.46890762	0	0.16393712	0	0	0	0	0
0	0.46290038	0	0	0	0.14208049	0	0	0
0	0.46272295	0	0.14108494	0	0	0	0	0
0	-0.16053787	0	0.15313647	0	0	0	0	0
0	0.46664546	0	0.069130796	0	0.083472943	0	0	0
0	-0.16041909	0	0	0	0.15339168	0	0	0
0	0.4668346	0	0	0	0.15463435	0	0	0
0	0.46776968	0	0	0	0.15294353	0	0.003053289	0
0	0.46268783	0	0	0	0.15047296	0	0	0
0	0.46675835	0	0.15423383	0	0	0	0	0
0	0.46783586	0	0.15227524	0	0	0	0.003691552	0
0	0.46137171	0	0.14915644	0	0	0	0	0
0	0	0	0	0	0.15930662	0	0	0
0	0	0	0.15903869	0	0	0	0	0
0	0	0	0.08050911	0	0.084651138	0	0	0
0	0	0	0.15915868	0	0	0	0	0
0	0	0	0	0	0.16385802	0	-0.013816267	0
0	0	0	0	0	0.15973511	0	0	0
0	0	0	0.16352985	0	0	0	-0.013483313	0
0	0	0	0	0	0.14712465	0	0	0

0	0	0	0.14665842	0	0	0	0	0
0	0	0	0.065143116	0	0.077333878	0	0	0
0	0	0	0	0	0.14295484	0	0.009826513	0
0	0	0	0.14215524	0	0	0	0.010650084	0
0	0	0	0	0	0.14280517	0	0	0
0	0	0	0.14153451	0	0	0	0	0
0	-0.15459316	0	0	0	0.15964853	0	0	0
0	-0.15461333	0	0.15937332	0	0	0	0	0
0	0	0	0.072151281	0	0.083818674	0	0	0
0	0	0	0.070113453	0	0.083286977	0	0.003460873	0
0	0	0	0.068646614	0	0.081673881	0	0	0
0	-0.15442677	0	0.080543234	0	0.084793792	0	0	0
0	-0.15879291	0	0	0	0.16402735	0	-0.01292425	0
0	-0.15869258	0	0.16369939	0	0	0	-0.012585853	0
0	0	0	0	0	0.15268705	0	0	0
0	-0.16857171	0	0.16028384	0	0	0	0	0
0	-0.16687955	0	0	0	0.16089594	0	0	0
0	0	0	0.15194953	0	0	0	0	0
0	0	0	0	0	0.15044725	0	0.003784764	0
0	0	0	0	0	0.15470603	0	0.000400398	0
0	0	0	0.14936182	0	0	0	0.004638113	0
0	0	0	0.15431916	0	0	0	0.00076151	0
0	-0.16059131	0	0	0	0.1475676	0	0	0
0	-0.160757	0	0.14708838	0	0	0	0	0
0	-0.15719839	0	0	0	0.14335209	0	0.010482937	0
0	-0.16045153	0	0.065144395	0	0.077621551	0	0	0
0	-0.15716683	0	0.14252814	0	0	0	0.011291868	0
0	-0.15153289	0	0	0	0.14396406	0	0	0
0	0	0	0.14037072	0	0	0	0	0
0	0	0	0	0	0.14106532	0	0	0
0	-0.15282651	0	0.14266103	0	0	0	0	0
0	-0.15638075	0	0.07260058	0	0.083751885	0	0	0
0	-0.15534271	0	0.070296147	0	0.083409626	0	0.004153189	0
0	-0.15838073	0	0.068670616	0	0.083088124	0	0	0
0	-0.16072241	0	0	0	0.15401825	0	0	0
0	-0.15618979	0	0	0	0.15510325	0	0.000977345	0
0	-0.15824615	0	0	0	0.15192084	0	0.003960157	0
0	-0.16240396	0	0.15324565	0	0	0	0	0
0	-0.15609298	0	0.1547273	0	0	0	0.001356233	0
0	-0.15949861	0	0.15081341	0	0	0	0.004834443	0
0	0.48520829	0	0.13326369	0	0	0	0	0
0	0.48640801	0	0	0	0.13402235	0	0	0
0	0	0	0	0	0.15279335	0	0	0
0	0.4480544	0	0.12781106	0	0	0	0	0
0	0	0	0.15204428	0	0	0	0	0
0	0.44776205	0	0	0	0.12727383	0	0	0
0	0	0	0.13745784	0	0	0	0	0

0	0	0	0.12292391	0	0	0	0	0
0	0.44929311	0	0.071381868	0	0.05204123	0	0	0
0	0	0	0	0	0.13762782	0	0	0
0	0.44917417	0	0.1209932	0	0	0	0	0
0	0.45054146	0	0.12279191	0	0	0	0.00411477	0
0	0.44929183	0	0.12279966	0	0	0	0	0
0	0	0	0	0	0.12238817	0	0	0
0	0.44906398	0	0	0	0.12130232	0	0	0
0	0.45015717	0	0	0	0.12270661	0	0.003253116	0
0	0.44914306	0	0	0	0.12247488	0	0	0
0	0	0	0.13952672	0	0	0	0	0
0	0	0	0.13081248	0	0	0	0	0
0	0	0	0	0	0.1397604	0	0	0
0	0	0	0	0	0.13149403	0	0	0
0	0	0	0.16561658	0	0	0	0	0
0	0	0	0.068622089	0	0.065478129	0	0	0
0	0	0	0	0	0.16651143	0	0	0
0	-0.13809499	0	0.1386551	0	0	0	0	0
0	0	0	0.12759677	0	0	0	-0.010988604	0
0	0	0	0.13273735	0	0	0	0.003307744	0
0	0	0	0.082309489	0	0.045913399	0	0	0
0	0	0	0.13287361	0	0	0	0	0
0	0	0	0	0	0.13338909	0	0.002379953	0
0	0	0	0	0	0.13327084	0	0	0
0	-0.13672958	0	0	0	0.13886473	0	0	0
0	0	0	0	0	0.12709326	0	-0.011310505	0
0	-0.17501817	0	0.1231092	0	0	0	0	0
0	-0.12773701	0	0.13194219	0	0	0	0	0
0	-0.12719673	0	0	0	0.13264783	0	0	0
0	-0.17508885	0	0	0	0.1225699	0	0	0
0	-0.17617932	0	0.13954849	0	0	0	0	0
0	-0.1347305	0	0.06882096	0	0.066550424	0	0	0
0	-0.17628602	0	0	0	0.13978353	0	0	0
0	-0.13507643	0	0.13401341	0	0	0	0.003793174	0
0	-0.13619533	0	0.13415615	0	0	0	0	0
0	-0.13399481	0	0	0	0.13469338	0	0.002850149	0
0	-0.1347319	0	0	0	0.13457925	0	0	0
0	0	0	0.072739409	0	0.049012798	0	0	0
0	0	0	0.072218354	0	0.051120927	0	0.003663955	0
0	0	0	0.070571519	0	0.052978662	0	0	0
0	0	0	0.12101184	0	0	0	0.00216353	0
0	0	0	0.11992963	0	0	0	0	0
0	-0.17875396	0	0.12763813	0	0	0	-0.010068223	0
0	0	0	0.122505	0	0	0	0.003680401	0
0	-0.17552347	0	0.082282468	0	0.045984822	0	0	0
0	0	0	0	0	0.12000871	0	0	0
0	0	0	0	0	0.12125201	0	0.001794505	0

0	0	0	0	0	0.12217708	0	0.003267616	0
0	-0.17895765	0	0	0	0.12711095	0	-0.010395089	0
0	-0.17433954	0	0.072713336	0	0.049142203	0	0	0
0	-0.17300837	0	0.072083669	0	0.051399041	0	0.004259555	0
0	-0.1743427	0	0.070710863	0	0.05310142	0	0	0
0	-0.17354467	0	0.12126075	0	0	0	0.00289897	0
0	-0.17435789	0	0.12029871	0	0	0	0	0
0	-0.17311194	0	0.12266303	0	0	0	0.004235854	0
0	-0.17376734	0	0	0	0.12149804	0	0.002523844	0
0	-0.17444451	0	0	0	0.12037304	0	0	0
0	-0.17331929	0	0	0	0.12232642	0	0.003807088	0
0	0	0	0.18091101	0	0	0	-0.02321467	0
0	0	0	0	0	0.1818497	0	-0.023684061	0
0	0	0	0.095726831	0	0.092028765	0	0	0
0	0	0	0	0	0.17999149	0	0	0
0	0	0	0	0	0.13991857	0	0	0
0	0	0	0.1390771	0	0	0	0	0
0	0	0	0.17899891	0	0	0	0	0
0	0	0	0	0	0.17276309	0	0.017619893	0
0	0	0	0.17130422	0	0	0	0.018737035	0
0	0	0	0	0	0	0	0	0
0	0	0	0.065320066	0	0.10699434	0	0	0
0	0	0	0.078441807	0	0.077562443	0	0	0
0	0	0	0.14717385	0	0	0	0	0
0	0	0	0	0	0.14800839	0	0	0
0	0	0	0	0	0.17348895	0	0	0
0	0.4742828	0	0	0	0	0	0	0
0	0	0	0.15381538	0	0	0	-0.0023722	0
0	0	0	0	0	0.15475739	0	-0.002922148	0
0	0	0	0.17227967	0	0	0	0	0
0	0.48511183	0	0	0	0.13849248	0	0	0
0	0.48361416	0	0.13798618	0	0	0	0	0
0	0	0	0	0	0.16145487	0	0	0
0	0	0	0	0	0	0	0	0
0	0.45157858	0	0.13804184	0	0	0	0	0
0	0.45148517	0	0	0	0.1385189	0	0	0
0	0	0	0.16085316	0	0	0	0	0
0	-0.14914946	0	0	0	0	0	0	0
0	0.44758225	0	0.12885749	0	0	0	-0.014311483	0
0	0.47066512	0	0	0	0	0	0	0
0	0.44727434	0	0	0	0.12868614	0	-0.014739923	0
0	0.45222398	0	0.07175179	0	0.057899304	0	0	0
0	0.45215796	0	0.12835285	0	0	0	0	0
0	0	0	0	0	0.18634768	0	0	0
0	0	0	0.077050282	0	0.090490658	0	0	0
0	0.45208077	0	0	0	0.1281376	0	0	0
0	0	0	0	0	0	0	0	0

0	0.44440915	0	0.15243546	0	0	0	-0.019234918	0
0	0.44423895	0	0	0	0.15298804	0	-0.019424722	0
0	-0.1599391	0	0	0	0	0	0	0
0	0	0	0	0	0.16600656	0	0.003646863	0
0	0	0	0.18529176	0	0	0	0	0
0	0	0	0.16537891	0	0	0	0.004295994	0
0	0	0	0.071738982	0	0.091835695	0	0.012176295	0
0	0	0	0	0	0.15961408	0	0.016024651	0
0	0	0	0	0	0	0	-0.009815449	0
0	0	0	0.077815949	0	0.11490285	0	0	0
0	0	0	0	0	0	0	0	0
0	0	0	0	0	0.18270951	0	0.013347599	0
0	0.45019947	0	0.081337442	0	0.078798462	0	0	0
0	0	0	0.07569948	0	0.11401199	0	0.007696879	0
0	0	0	0.069274322	0	0.11521835	0	0	0
0	0	0	0.15884531	0	0	0	0.01678461	0
0	0	0	0.066558812	0	0.095822456	0	0	0
0	0.45050206	0	0.15018204	0	0	0	0	0
0	0.45043944	0	0	0	0.15087204	0	0	0
0	0	0	0	0	0.13841643	0	-0.016202111	0
0	0	0	0.13784762	0	0	0	-0.015751513	0
0	0	0	0	0	0.14264535	0	0	0
0	0	0	0	0	0.19103662	0	0.004025166	0
0	0	0	0.14181997	0	0	0	0	0
0	0.45319901	0	0.15978507	0	0	0	0	0
0	0.48224126	0	0.14393038	0	0	0	0	0
0	0.45409929	0	0	0	0.16079106	0	0	0
0	0.48375883	0	0	0	0.14442226	0	0	0
0	0	0	0.1815452	0	0	0	0.014054974	0
0	0	0	0.070388894	0	0.069645417	0	0	0
0	0	0	0.069631556	0	0.069416367	0	0	0
0	0	0	0	0	0.14358091	0	0	0
0	0	0	0.14289158	0	0	0	0	0
0	-0.15423533	0	0	0	0	0	0	0
0	0	0	0.13752729	0	0	0	-0.010054204	0
0	0	0	0	0	0.13798094	0	-0.010231191	0
0	0	0	0	0	0.13768055	0	0	0
0	0	0	0.13717001	0	0	0	0	0
0	0	0	0.19010074	0	0	0	0.004534858	0
0	0	0	0	0	0	0	-0.019753263	0
0	0.44582366	0	0.1349586	0	0	0	-0.017884509	0
0	-0.13002441	0	0	0	0	0	0	0
0	0.4456326	0	0	0	0.13476101	0	-0.018187736	0
0	-0.15218126	0	0	0	0	0	-0.008951504	0
0	0	0	0.070466292	0	0.059333123	0	-0.015372199	0
0	0	0	0	0	0	0	0	0
0	0.45156546	0	0.077005028	0	0.058741427	0	0	0

0	0	0	0.12866439	0	0	0	-0.014513801	0
0	0	0	0	0	0.12848797	0	-0.014766934	0
0	-0.14049801	0	0	0	0.13984226	0	-0.015771067	0
0	-0.14191621	0	0.13923901	0	0	0	-0.015313976	0
0	0	0	0.066414373	0	0.062775889	0	0	0
0	0	0	0.077040031	0	0.076440116	0	-0.019156797	0
0	-0.14512946	0	0	0	0.14379561	0	0	0
0	-0.14626718	0	0.1429497	0	0	0	0	0
0	-0.1361085	0	0.069534575	0	0.070957522	0	0	0
0	0	0	0	0	0.15567808	0	-0.021567386	0
0	0	0	0.15471881	0	0	0	-0.021163811	0
0	-0.13572181	0	0	0	0.13908764	0	0	0
0	-0.13725825	0	0.13853837	0	0	0	0	0
0	0	0	0.15004859	0	0	0	-0.018009219	0
0	0	0	0	0	0.15068676	0	-0.018358253	0
0	-0.17204883	0	0.070666777	0	0.069785036	0	0	0
0	-0.17239934	0	0	0	0.14376144	0	0	0
0	-0.17229535	0	0.14307284	0	0	0	0	0
0	-0.17506011	0	0.13795617	0	0	0	-0.009471209	0
0	-0.17520386	0	0	0	0.13843726	0	-0.009625965	0
0	0	0	0.077327765	0	0.074443656	0	0	0
0	-0.15853642	0	0	0	0	0	-0.019139533	0
0	0	0	0.14348603	0	0	0	-0.018495395	0
0	0	0	0	0	0.14403017	0	-0.018844895	0
0	0	0	0.15042528	0	0	0	0	0
0	0	0	0	0	0.15125354	0	0	0
0	0	0	0.081120627	0	0.079659326	0	0	0
0	0	0	0.074435755	0	0.070307028	0	0	0
0	-0.17613315	0	0.070666357	0	0.059361185	0	-0.014888189	0
0	-0.16422798	0	0	0	0	0	0	0
0	-0.17579832	0	0.12881807	0	0	0	-0.013617791	0
0	-0.17595422	0	0	0	0.12863846	0	-0.013870824	0
0	-0.1789207	0	0.077198388	0	0.076604774	0	-0.018462277	0
0	-0.17138423	0	0.067945871	0	0.061427223	0	0	0
0	0	0	0.075562499	0	0.0600311	0	-0.018231123	0
0	-0.16570837	0	0	0	0.1571913	0	-0.021647968	0
0	-0.16665968	0	0.1562061	0	0	0	-0.021243383	0
0	0.46237001	0	0.1568994	0	0	0	-0.051622882	0
0	-0.17841493	0	0.15003984	0	0	0	-0.017089715	0
0	-0.17863046	0	0	0	0.1508009	0	-0.017420861	0
0	-0.14413844	0	0.14467254	0	0	0	-0.017684701	0
0	-0.14269118	0	0	0	0.14525119	0	-0.018040118	0
0	0.46351059	0	0	0	0.15746433	0	-0.052974438	0
0	-0.15120135	0	0	0	0.1515564	0	0	0
0	-0.15234025	0	0.15071884	0	0	0	0	0
0	-0.17297568	0	0.077250496	0	0.074590325	0	0	0
0	-0.13754324	0	0.07417246	0	0.071798268	0	0	0

0	-0.16665923	0	0.081292653	0	0.081017181	0	0	0
0	0.43456442	0	0.091995191	0	0.057086948	0	-0.050543906	0
0	0	0	0.14280954	0	0	0	0.020161425	0
0	0	0	0.13902073	0	0	0	0.023359708	0
0	0	0	0	0	0.14272864	0	0.020042878	0
0	-0.1775816	0	0.075703521	0	0.059912005	0	-0.017330022	0
0	0	0	0	0	0.13897034	0	0.023128202	0
0	0	0	0.13594408	0	0	0	0	0
0	0	0	0.085823699	0	0.056871255	0	0	0
0	0.45188204	0	0	0	0	0	0	0
0	0	0	0	0	0.13589024	0	0	0
0	0	0	0.14212835	0	0	0	0	0
0	0	0	0.14003223	0	0	0	0	0
0	0	0	0.087804639	0	0.053436442	0	0	0
0	0	0	0	0	0.14221704	0	0	0
0	0	0	0	0	0	0	-0.029769012	0
0	0	0	0.090024049	0	0.067645618	0	-0.052056412	0
0	0	0	0	0	0.14010145	0	0	0
0	0	0	0	0	0	0	0	0
0	0	0	0	0	0	0	0	0
0	0.4416738	0	0	0	0	0	0	0
0	0	0	0.15886909	0	0	0	0.012275538	0
0	-0.15736099	0	0.090141222	0	0.069038997	0	-0.051925296	0
0	0	0	0.095971965	0	0.062879208	0	0	0
0	-0.17798322	0	0	0	0	0	-0.029209264	0
0	0	0	0.082249444	0	0.083929516	0	0.00761868	0
0	0	0	0	0	0.15917206	0	0.011867479	0
0	-0.1941763	0	0	0	0	0	0	0
0	0	0	0	0	0	0	0	0
0	0	0	0	0	0	0	0	0
0	0	0	0	0	0	0	0	0
0	0	0	0	0	0	0	-0.001689635	0
0	0.43838445	0	0	0	0	0	0	0
0	0	0	0	0	0	0	0.001551717	0
0	-0.15237638	0	0	0	0	0	0	0
0	0.4395395	0	0	0	0	0	0	0
0	-0.18242457	0	0	0	0	0	0	0
0	-0.18229378	0	0	0	0	0	-0.001035566	0
0	0	0	0	0	0	0	0	0
0	0.4070893	0	0	0	0	0	0	0
0	0.43246145	0	0	0	0	0	-0.016971277	0
0	0.44245595	0	0	0	0	0	0	0
0	0	0	0	0	0	0	0	0
0	0.43459545	0	0	0	0	0	0	0
0	0.42614658	0	0	0	0	0	0	0
0	0	0	0	0	0	0	-0.004048986	0
0	0.44163154	0	0	0	0	0	0.007982472	0

0	0	0	0	0	0	0	-0.043734817	0
0	0	0	0	0	0	0	0	0
0	0	0	0	0	0	0	0	0
0	-0.18108673	0	0	0	0	0	0	0
0	0.44084746	0	0	0	0	0	0	0
0	0	0	0	0	0	0	-0.008910319	0
0	0.44023898	0	0	0	0	0	-0.003131268	0
0	0	0	0	0	0.15115903	0	0	0
0	0	0	0	0	0	0	0	0
0	0	0	0.14987752	0	0	0	0	0
0	0	0	0	0	0	0	-0.000303724	0
0	0.41973469	0	0	0	0	0	-0.004240698	0
0	0.41992426	0	0	0	0	0	0	0
0	0	0	0	0	0	0	0	0
0	0	0	0	0	0	0	-0.017311831	0
0	-0.19589538	0	0	0	0	0	0	0
0	0.43417392	0	0	0	0	0	-0.008452656	0
0	-0.18605641	0	0	0	0	0	-0.003160226	0
0	0	0	0	0	0	0	0.007974499	0
0	0	0	0.067209588	0	0.095692814	0	0	0
0	-0.1588897	0	0	0	0	0	0	0
0	0	0	0.065383647	0	0.10163028	0	0	0
0	0	0	0	0	0.16149722	0	-0.007640688	0
0	0	0	0.1722937	0	0	0	0.026475101	0
0	0	0	0	0	0	0	0.007388979	0
0	0	0	0.066516472	0	0.10114974	0	-0.003202833	0
0	0.4700831	0	0.12932689	0	0.050606908	0	0	0
0	0	0	0	0	0.1736993	0	0.026145648	0
0	0	0	0	0	0.17264103	0	0.026060908	0
0	0	0	0.17348428	0	0	0	0.026422327	0
0	0	0	0	0	0.16586704	0	0.000490775	0
0	-0.18649321	0	0	0	0	0	-0.008018318	0
0	0	0	0.1602668	0	0	0	-0.007286545	0
0	-0.22751362	0	0	0	0	0	-0.044032276	0
0	-0.20810121	0	0	0	0	0	0	0
0	0	0	0.16493836	0	0	0	0.00108027	0
0	0.38568125	0	0	0	0	0	0	0
0	0	0	0.086874063	0	0.093425582	0	0.023903076	0
0	0	0	0.16977127	0	0	0	0	0
0	0	0	0	0	0.17012598	0	0	0
0	0.44523871	0	0	0	0	0	-0.0602341	0
0	0	0	0	0	0	0	-0.008484866	0
0	-0.21748176	0	0	0	0	0	0	0
0	0	0	0.084402848	0	0.082855156	0	0.038056319	0
0	0.3969358	0	0	0	0	0	0	0
0	0	0	0.090468499	0	0.085303447	0	0	0
0	0	0	0.085322961	0	0.091478453	0	0	0

0	-0.18599666	0	0	0	0	0	-0.017338886	0
0	-0.21412809	0	0	0	0	0	4.69E-05	0
0	0	0	0.084673384	0	0.086305866	0	0.03573247	0
0	-0.17832786	0	0	0	0	0	0.008536866	0
0	0	0	0	0	0.16556008	0	0.038167934	0
0	0	0	0.16507817	0	0	0	0.038701744	0
0	0	0	0.06974606	0	0.090721022	0	0	0
0	0	0	0	0	0	0	-0.008503198	0
0	0	0	0	0	0.15944984	0	-0.005614754	0
0	0	0	0.15818435	0	0	0	-0.005138701	0
0	-0.19270156	0	0	0	0	0	0.007922351	0
0	0	0	0	0	0	0	0	0
0	-0.18498664	0	0	0	0	0	-0.007609775	0
0	0	0	0.071493745	0	0.10230374	0	-0.003128676	0
0	-0.20372347	0	0	0	0	0	-0.007612735	0
0	0	0	0	0	0	0	0.001227499	0
0	0.38542866	0	0	0	0	0	0	0
0	0.37600401	0	0	0	0	0	-0.017524224	0
0	0	0	0.080921883	0	0.088718043	0	0	0
0	-0.22825955	0	0	0	0	0	0	0
0	0.45077114	0	0	0	0	0	0	0
0	0.44962099	0	0	0	0	0	-0.001823639	0
0	0	0	0	0	0	0	-0.013475783	0
0	-0.22424175	0	0	0	0	0	0.001553161	0
0	0	0	0	0	0	0	-0.011293128	0
0	0	0	0	0	0	0	-0.00786234	0
0	0	0	0.087379566	0	0.10897113	0	-0.024434511	0
0	-0.23867936	0	0	0	0	0	-0.010415454	0
0	0	0	0	0	0	0	0	0
0	-0.17499144	0	0	0	0	0	-0.006970521	0
0	0.44528654	0	0	0	0	0	-0.012941677	0
0	0	0	0	0	0	0	0.003266785	0
0	0.44434132	0	0	0	0	0	0	0
0	0.43717251	0	0	0	0	0	-0.018627002	0
0	0.43403622	0	0	0	0	0	0	0
0	0.41881741	0	0	0	0	0	-0.012273288	0
0	0	0	0	0	0	0	0.004872658	0
0	0.42317172	0	0	0	0	0	0	0
0	0.43870025	0	0	0	0	0	-0.010008384	0
0	0	0	0	0	0	0	-0.010853195	0
0	0	0	0	0	0	0	0.030025104	0
0	0	0	0	0	0	0	0	0
0	0	0	0	0	0	0	-0.018383604	0
0	-0.16282602	0	0	0	0	0	0	0
0	-0.18237621	0	0	0	0	0	-0.009951539	0
0	0	0	0	0	0	0	0	0
0	0	0	0	0	0	0	-0.010340005	0

0	0	0	0.13295152	0	0	0	0.034195472	0
0	0	0	0	0	0.13229376	0	0.033972773	0
0	0	0	0.083146766	0	0.050145744	0	0.040702998	0
0	0	0	0.13237462	0	0	0	0.040908066	0
0	-0.18943281	0	0	0	0	0	-0.018279665	0
0	0.43739756	0	0	0	0	0	-0.016413781	0
0	0	0	0	0	0.13229084	0	0.040681528	0
0	0	0	0.14879229	0	0	0	0.03137453	0
0	0	0	0	0	0	0	0.025535943	0
0	0	0	0.088595355	0	0.053731746	0	0.036170344	0
0	0	0	0	0	0.14884697	0	0.031074853	0
0	0.44864463	0	0	0	0	0	0	0
0	0	0	0.091789135	0	0.047696858	0	0.031162805	0
0	0	0	0.14434643	0	0	0	0	0
0	-0.20110666	0	0	0	0	0	-0.009433746	0
0	0	0	0	0	0.14445899	0	0	0
0	0	0	0.085158097	0	0.050706692	0	0	0
0	0	0	0	0	0	0	-0.027570037	0
0	0	0	0.091068235	0	0.060665447	0	0	0
0	0.4114862	0	0	0	0	0	-0.015806828	0
0	0	0	0	0	0	0	-0.016659427	0
0	0	0	0	0	0	0	0.01899745	0
0	0	0	0.080357364	0	0.052113401	0	0	0
0	-0.17567714	0	0	0	0	0	-0.01587048	0
0	0.36514458	0	0	0	0	0	-0.045917812	0
0	0	0	0	0	0	0	0.021358341	0
0	0	0	0	0	0	0	0	0
0	0	0	0	0	0	0	0.028941613	0
0	0	0	0	0	0	0	0.003780456	0
0	0	0	0.080733164	0	0.07158241	0	0.027803414	0
0	0	0	0.15063279	0	0	0	0.027767325	0
0	0	0	0	0	0.15114112	0	0.027538708	0
0	0	0	0.079748914	0	0.059522348	0	0.02753308	0
0	0	0	0.08020525	0	0.073908993	0	0	0
0	0	0	0.087345328	0	0.073506217	0	0.023937016	0
0	0	0	0	0	0	0	0.01680225	0
0	0	0	0	0	0	0	0.019938305	0
0	0	0	0	0	0	0	0.039860834	0
0	0	0	0	0	0	0	0.03935642	0
0	0.47816353	0	0.13338807	0	0	0	0	0
0	0.47796635	0	0	0	0.13348813	0	0	0
0	0.47246108	0	0.13921515	0	0	0	0	0
0	0.47232618	0	0	0	0.13955877	0	0	0
0	0.47861843	0	0.13530335	0	0	0	0	0
0	0.47838431	0	0	0	0.13552171	0	0	0
0	0.47120221	0	0.14665459	0	0	0	-0.019285496	0
0	0.47089268	0	0	0	0.14692221	0	-0.019544484	0

0	0.47695553	0	0.087773942	0	0.066418451	0	0	0	
0	0.47638755	0	0.15270642	0	0	0	0	0	
0	0	0	0.12942703	0	0	0	0	0	
0	0.47681492	0	0	0	0.15337342	0	0	0	
0	0	0	0	0	0.12954585	0	0	0	
0	0	0	0.13634485	0	0	0	0	0	
0	0	0	0.1327733	0	0	0	-0.010788002	0	
0	0	0	0.077467323	0	0.057690327	0	0	0	
0	0	0	0	0	0.13674973	0	0	0	
0	0	0	0.13098558	0	0	0	0	0	
0	0	0	0	0	0.13288188	0	-0.010951689	0	
0	0	0	0.13685552	0	0	0	-0.012359392	0	
0	0	0	0.14181248	0	0	0	0	0	
0	0	0	0.083617016	0	0.056544174	0	0	0	
0	0	0	0	0	0.13117258	0	0	0	
0	0	0	0.13430362	0	0	0	0	0	
0	0	0	0	0	0.13715924	0	-0.012758501	0	
0	0	0	0.1358115	0	0	0	0	0	
0	0	0	0	0	0.1423793	0	0	0	
0	0	0	0	0	0.13447454	0	0	0	
0	0	0	0.13515143	0	0	0	-0.01169805	0	
0	0	0	0	0	0.13619581	0	0	0	
0	0	0	0.080258927	0	0.056267404	0	0	0	
0	0	0	0	0	0.13541962	0	-0.012018524	0	
0	0	0	0.083444552	0	0.064054067	0	-0.019213213	0	
0	-0.14854246	0	0.12983906	0	0	0	0	0	
0	-0.12519145	0	0.13738822	0	0	0	0	0	
0	0	0	0.14781891	0	0	0	-0.020445316	0	
0	-0.14865425	0	0	0	0.12995839	0	0	0	
0	-0.12425397	0	0	0	0.13781757	0	0	0	
0	0.46292523	0	0.15332151	0	0	0	0	0	
0	-0.14533405	0	0.077627891	0	0.057879928	0	0	0	
0	-0.14853165	0	0.13315392	0	0	0	-0.010183129	0	
0	0	0	0	0	0.14841905	0	-0.020917882	0	
0	0.46304388	0	0	0	0.15356599	0	0	0	
0	-0.13259945	0	0.14292487	0	0	0	0	0	
0	-0.14494904	0	0.13108635	0	0	0	0	0	
0	-0.11650144	0	0.13601232	0	0	0	0	0	
0	-0.1487636	0	0	0	0.13328319	0	-0.01033782	0	
0	-0.15377913	0	0.13721883	0	0	0	-0.01175438	0	
0	-0.15080406	0	0.083740157	0	0.056699963	0	0	0	
0	-0.14516225	0	0	0	0.13126883	0	0	0	
0	-0.11558789	0	0	0	0.13643126	0	0	0	
0	-0.13210652	0	0	0	0.14351622	0	0	0	
0	-0.14895279	0	0.13428702	0	0	0	0	0	
0	-0.15405257	0	0	0	0.13752985	0	-0.012153319	0	
0	0	0	0.087363838	0	0.066098716	0	0	0	

0	-0.14907438	0	0	0	0.1344562	0	0	0
0	-0.14848853	0	0.13506089	0	0	0	-0.010813302	0
0	-0.14490497	0	0.080230892	0	0.056254137	0	0	0
0	-0.14876148	0	0	0	0.13535513	0	-0.011123202	0
0	-0.15204124	0	0.083690723	0	0.064113937	0	-0.018546285	0
0	-0.14355994	0	0.14923221	0	0	0	-0.020525691	0
0	-0.14292836	0	0	0	0.14987691	0	-0.020968338	0
0	0	0	0.14877982	0	0	0	0	0
0	-0.14373062	0	0.087621359	0	0.067295713	0	0	0
0	0	0	0	0	0.14899205	0	0	0
0	0.46888568	0	0	0	0.15943537	0	0	0
0	0.46886253	0	0.15916657	0	0	0	0	0
0	0	0	0.082899021	0	0.071581044	0	0	0
0	0	0	0.15306824	0	0	0	-0.010979557	0
0	0	0	0	0	0.15335996	0	-0.011362561	0
0	0	0	0.15217772	0	0	0	0	0
0	0.46917358	0	0.080885236	0	0.084722307	0	0	0
0	0	0	0	0	0.15265558	0	0	0
0	0.46448549	0	0	0	0.16429864	0	-0.013659847	0
0	0.45246501	0	0.15993936	0	0	0	0	0
0	0.46458615	0	0.16397673	0	0	0	-0.013320597	0
0	0.45415398	0	0	0	0.16052278	0	0	0
0	0.46290136	0	0	0	0.1472176	0	0	0
0	0.46274401	0	0.14674481	0	0	0	0	0
0	0.46302241	0	0.065467922	0	0.077453063	0	0	0
0	-0.16044151	0	0.14922429	0	0	0	0	0
0	0.46605487	0	0	0	0.14342094	0	0.009978835	0
0	-0.160329	0	0	0	0.1494392	0	0	0
0	0.46615053	0	0.14261389	0	0	0	0.010805978	0
0	0.46939209	0	0	0	0.143562	0	0	0
0	0.46827859	0	0.14227978	0	0	0	0	0
0	-0.16025902	0	0.082799712	0	0.071969059	0	0	0
0	-0.16371023	0	0.15332797	0	0	0	-0.010085617	0
0	-0.16371287	0	0	0	0.15362324	0	-0.010476233	0
0	0.46705674	0	0.072061065	0	0.083845193	0	0	0
0	-0.16020375	0	0.15319862	0	0	0	0	0
0	0.46798606	0	0.070478517	0	0.083363462	0	0.003633012	0
0	0.46285409	0	0.068995959	0	0.082198955	0	0	0
0	-0.1587596	0	0	0	0.15371317	0	0	0
0	0.46081121	0	0	0	0.1530645	0	0	0
0	0.46297591	0	0	0	0.15130809	0	0.00378025	0
0	0.4672039	0	0	0	0.15466085	0	0.001088051	0
0	0.45914111	0	0.15231083	0	0	0	0	0
0	0.4617409	0	0.15021507	0	0	0	0.004637864	0
0	0.4673481	0	0.15427685	0	0	0	0.001471596	0
0	0	0	0.076774978	0	0.08394068	0	0	0
0	0	0	0	0	0.15582206	0	0	0

0	0	0	0.15527854	0	0	0	0	0
0	0	0	0	0	0.15962536	0	-0.011687648	0
0	0	0	0.15932445	0	0	0	-0.011392818	0
0	0	0	0.079479706	0	0.085438928	0	-0.013720789	0
0	0	0	0.081917621	0	0.079123408	0	0	0
0	0	0	0.15932861	0	0	0	-0.013009735	0
0	0	0	0	0	0.15994556	0	-0.013386036	0
0	0	0	0.072396313	0	0.076000783	0	0	0
0	0	0	0	0	0.1469824	0	0.004645549	0
0	0	0	0.068227328	0	0.075681068	0	0.01035245	0
0	0	0	0.14650631	0	0	0	0.005133935	0
0	0	0	0	0	0.14755268	0	0	0
0	0	0	0.14683043	0	0	0	0	0
0	0	0	0.064820258	0	0.07879169	0	0	0
0	-0.15441755	0	0.076944803	0	0.08411168	0	0	0
0	0	0	0	0	0.14383043	0	0.009586832	0
0	0	0	0.14274233	0	0	0	0.010485211	0
0	-0.15809578	0	0	0	0.15994176	0	-0.010827808	0
0	-0.15801644	0	0.15964074	0	0	0	-0.010527552	0
0	-0.16511863	0	0	0	0.15713741	0	0	0
0	-0.16684631	0	0.15655605	0	0	0	0	0
0	-0.1585415	0	0.080079075	0	0.085307043	0	-0.012755611	0
0	0	0	0.13571574	0	0	0	0	0
0	0	0	0	0	0.13639631	0	0	0
0	-0.16705806	0	0.08160104	0	0.080596581	0	0	0
0	0	0	0.072764578	0	0.081131615	0	0	0
0	0	0	0.07201289	0	0.083973236	0	0.000600055	0
0	0	0	0.07023633	0	0.081079007	0	0.004384565	0
0	-0.17198545	0	0.16044612	0	0	0	-0.012197375	0
0	-0.17038199	0	0	0	0.16109944	0	-0.012580057	0
0	-0.16045511	0	0.072567192	0	0.076276367	0	0	0
0	0	0	0	0	0.1526842	0	0.000886194	0
0	0	0	0.15195095	0	0	0	0.001347957	0
0	-0.15894005	0	0	0	0.14741663	0	0.005201889	0
0	-0.15448663	0	0	0	0.1486964	0	0	0
0	-0.15692251	0	0.068284502	0	0.07601668	0	0.010965331	0
0	-0.15894048	0	0.14695263	0	0	0	0.005697264	0
0	-0.15599771	0	0.14794323	0	0	0	0	0
0	0	0	0.13999027	0	0	0	0	0
0	0	0	0.073591166	0	0.068747426	0	0	0
0	-0.15150655	0	0.064898932	0	0.079875481	0	0	0
0	0	0	0	0	0.14065141	0	0	0
0	-0.14961899	0	0	0	0.14502917	0	0.009989315	0
0	-0.15071949	0	0.14391279	0	0	0	0.010894626	0
0	0	0	0.14042206	0	0	0	-0.001599542	0
0	0	0	0	0	0.14113952	0	-0.002035057	0
0	0	0	0.14529712	0	0	0	0	0

0	0	0	0	0	0.14599604	0	0	0
0	-0.16087382	0	0.072704581	0	0.082517222	0	0	0
0	-0.15597652	0	0.072481439	0	0.083903388	0	0.001178097	0
0	0.48309311	0	0.13828203	0	0	0	0	0
0	-0.15802823	0	0.070341834	0	0.08244926	0	0.004561701	0
0	0.44848791	0	0.12311001	0	0	0	0	0
0	0.4844377	0	0	0	0.13845913	0	0	0
0	-0.16043586	0	0	0	0.15398577	0	0.001242268	0
0	-0.1619915	0	0.1532471	0	0	0	0.00170399	0
0	0.44846143	0	0	0	0.12257416	0	0	0
0	0.44737423	0	0.13991158	0	0	0	0	0
0	0.49326526	0	0.13157587	0	0	0	0	0
0	0.4936445	0	0	0	0.13226422	0	0	0
0	0.44726682	0	0	0	0.14013967	0	0	0
0	0.48648128	0	0.069207356	0	0.065722496	0	0	0
0	0	0	0.075131138	0	0.078893971	0	0	0
0	0	0	0.13309584	0	0	0	0	0
0	0.48605882	0	0.13355866	0	0	0	0.003440071	0
0	0.48515375	0	0.13341822	0	0	0	0	0
0	0.44463493	0	0.12802163	0	0	0	-0.010796835	0
0	0.487131	0	0	0	0.13421692	0	0.002500078	0
0	0.48659757	0	0	0	0.13383623	0	0	0
0	0	0	0	0	0.15243021	0	-0.00858168	0
0	0.44803731	0	0.082656739	0	0.046005059	0	0	0
0	0	0	0	0	0.13325395	0	0	0
0	0	0	0.15167824	0	0	0	-0.00824318	0
0	0.44436255	0	0	0	0.1275187	0	-0.011131662	0
0	0	0	0.13449392	0	0	0	0	0
0	0	0	0	0	0.13468848	0	0	0
0	0	0	0.13773887	0	0	0	-0.011407474	0
0	0	0	0.079897984	0	0.058975627	0	0	0
0	0	0	0.14052164	0	0	0	0	0
0	-0.13685403	0	0.13442684	0	0	0	0	0
0	0	0	0	0	0.13794063	0	-0.011773406	0
0	0	0	0.077406149	0	0.046349488	0	0	0
0	0	0	0.12323247	0	0	0	-0.008399923	0
0	-0.1354779	0	0	0	0.13463335	0	0	0
0	0	0	0	0	0.14093221	0	0	0
0	0.4493146	0	0.073024838	0	0.049127224	0	0	0
0	0.45051132	0	0.072583278	0	0.051198494	0	0.003837145	0
0	0.44922715	0	0.071937176	0	0.051814472	0	0	0
0	0.44929254	0	0.11972394	0	0	0	0	0
0	0.4498494	0	0.12143934	0	0	0	0.002309268	0
0	0	0	0	0	0.12272715	0	-0.008666925	0
0	0.45049244	0	0.12265866	0	0	0	0.004356914	0
0	0	0	0.13975534	0	0	0	-0.011043266	0
0	0	0	0.083239323	0	0.057930418	0	0	0

0	0.44912202	0	0	0	0.11979746	0	0	0
0	0.44956408	0	0	0	0.12167006	0	0.001916404	0
0	0	0	0.12581532	0	0	0	0	0
0	0.45035896	0	0	0	0.12232249	0	0.003906726	0
0	0	0	0.073757006	0	0.058619793	0	0	0
0	0	0	0	0	0.14003466	0	-0.011397345	0
0	0	0	0	0	0.12640247	0	0	0
0	0	0	0.088654761	0	0.078900033	0	0	0
0	0	0	0.13031654	0	0	0	-0.003753437	0
0	0	0	0.16428392	0	0	0	-0.012539827	0
0	0	0	0	0	0.16515672	0	-0.013100228	0
0	0	0	0	0	0.13094806	0	-0.00423034	0
0	-0.13688316	0	0.079641109	0	0.06046087	0	0	0
0	-0.14121798	0	0.13891944	0	0	0	-0.010591777	0
0	-0.17559917	0	0.13467843	0	0	0	0	0
0	0	0	0.069863775	0	0.064477481	0	0.002940527	0
0	0	0	0.069505537	0	0.06491611	0	0	0
0	0	0	0.081581373	0	0.046877389	0	-0.011130029	0
0	-0.13995517	0	0	0	0.13917099	0	-0.01097026	0
0	-0.14244271	0	0.14077361	0	0	0	0	0
0	-0.17568625	0	0	0	0.13488097	0	0	0
0	0	0	0.13274012	0	0	0	0.003302504	0
0	-0.14146058	0	0	0	0.14124319	0	0	0
0	0	0	0	0	0.13314425	0	0.002811383	0
0	-0.12154806	0	0.12681724	0	0	0	0	0
0	-0.1269177	0	0.07390094	0	0.059631388	0	0	0
0	-0.12070559	0	0	0	0.1273013	0	0	0
0	-0.17494927	0	0.077663667	0	0.046289863	0	0	0
0	-0.17740139	0	0.12338456	0	0	0	-0.007508782	0
0	-0.12785082	0	0.13146239	0	0	0	-0.003636018	0
0	-0.17616492	0	0.079785073	0	0.06142548	0	0	0
0	-0.17937125	0	0.13975556	0	0	0	-0.010119103	0
0	-0.17755586	0	0	0	0.12287601	0	-0.007773666	0
0	-0.12732025	0	0	0	0.13211669	0	-0.004115626	0
0	-0.17958856	0	0	0	0.14003354	0	-0.01047395	0
0	-0.13395578	0	0.06816075	0	0.067454197	0	0.003402911	0
0	-0.13487023	0	0.069411795	0	0.066315973	0	0	0
0	-0.13504113	0	0.13398443	0	0	0	0.003833106	0
0	-0.13376044	0	0	0	0.1344301	0	0.003325416	0
0	0	0	0.073069493	0	0.04910794	0	0.002182433	0
0	0	0	0.07258003	0	0.048640507	0	0	0
0	0	0	0.072373412	0	0.051078483	0	0.003472062	0
0	-0.17874039	0	0.081677318	0	0.046803103	0	-0.010206031	0
0	0	0	0.12006545	0	0	0	0.002752623	0
0	0	0	0	0	0.11992811	0	0.002456862	0
0	-0.1734981	0	0.072911968	0	0.049515883	0	0.002923685	0
0	-0.17429359	0	0.070991507	0	0.050616239	0	0	0

0	0	0	0.091097002	0	0.091758282	0	-0.023175216	0
0	-0.17311324	0	0.07263461	0	0.050973548	0	0.004028428	0
0	-0.1733003	0	0.12045397	0	0	0	0.003400272	0
0	-0.17345631	0	0	0	0.12057434	0	0.003144916	0
0	0	0	0	0	0.1751172	0	0	0
0	0.47418132	0	0	0	0	0	0	0
0	0	0	0.17414689	0	0	0	0	0
0	0	0	0.079168569	0	0.10210624	0	0	0
0	0.46365289	0	0	0	0	0	0	0
0	0	0	0.067910013	0	0.073098704	0	0	0
0	0	0	0	0	0.17956799	0	0.005564917	0
0	0	0	0	0	0.14183527	0	0	0
0	0	0	0	0	0.13992819	0	0.004509322	0
0	0	0	0.14100839	0	0	0	0	0
0	0	0	0.13916067	0	0	0	0.004939035	0
0	0	0	0.1785579	0	0	0	0.006233587	0
0	0	0	0.070792337	0	0.10288975	0	0.018174168	0
0	0	0	0	0	0	0	0	0
0	0	0	0	0	0.17198195	0	0.018515223	0
0	0.46913197	0	0	0	0	0	0	0
0	0	0	0	0	0	0	0	0
0	0	0	0.074563464	0	0.074738545	0	0	0
0	0	0	0.17082297	0	0	0	0.01931341	0
0	0	0	0	0	0	0	-0.005952974	0
0	0	0	0.068372922	0	0.10629123	0	0	0
0	0	0	0.078004689	0	0.077685649	0	-0.002496287	0
0	0.48072488	0	0	0	0.13897083	0	-0.015347723	0
0	0	0	0.14713914	0	0	0	0.001191625	0
0	0	0	0	0	0.14795998	0	0.000701573	0
0	0.47932357	0	0.13838451	0	0	0	-0.014896669	0
0	0.4899404	0	0	0	0	0	0	0
0	0.47647805	0	0	0	0.14270496	0	0	0
0	0.4710971	0	0	0	0	0	-0.009661092	0
0	0.47535068	0	0.14187993	0	0	0	0	0
0	0.48498322	0	0.069399712	0	0.070227783	0	0	0
0	0	0	0.072002806	0	0.090666118	0	0	0
0	0	0	0	0	0	0	-0.011328272	0
0	0.4853537	0	0	0	0.13829162	0	0	0
0	0.45162416	0	0.070433236	0	0.06925968	0	0	0
0	0.48382661	0	0.13776822	0	0	0	0	0
0	0.45114577	0	0	0	0.14364362	0	0	0
0	0.45125092	0	0.14295649	0	0	0	0	0
0	-0.15392504	0	0	0	0	0	0	0
0	0	0	0	0	0.16077441	0	0.006477636	0
0	0.44860455	0	0.13720636	0	0	0	-0.009470587	0
0	0.44846416	0	0	0	0.13767966	0	-0.009622458	0
0	0.46502753	0	0	0	0	0	-0.019081674	0

0	-0.12550059	0	0	0	0	0	0	0
0	0	0	0	0	0	0	0	0
0	0	0	0.16015739	0	0	0	0.007081821	0
0	-0.15082726	0	0	0	0	0	-0.005091636	0
0	0.44758087	0	0.070370113	0	0.05955795	0	-0.014509268	0
0	0.45751513	0	0	0	0	0	0	0
0	0.44764265	0	0.1288304	0	0	0	-0.014121418	0
0	0	0	0.07331719	0	0.11431731	0	0	0
0	0.44748737	0	0	0	0.12865256	0	-0.014375113	0
0	0.45221873	0	0.070905887	0	0.058488048	0	0	0
0	0	0	0	0	0.18581307	0	0.006275999	0
0	0	0	0	0	0	0	0	0
0	0.44455528	0	0.07742967	0	0.07648752	0	-0.019002307	0
0	0	0	0.07684898	0	0.090373046	0	0.003835198	0
0	0	0	0	0	0	0	-0.00873896	0
0	-0.16282797	0	0	0	0	0	-0.010736638	0
0	0.45544458	0	0	0	0.15655128	0	-0.021707145	0
0	0.45450304	0	0.15558992	0	0	0	-0.021292882	0
0	0.4449022	0	0.15041126	0	0	0	-0.017828957	0
0	-0.15271074	0	0	0	0	0	0	0
0	0.44464556	0	0	0	0.15116276	0	-0.018155724	0
0	0	0	0.18472254	0	0	0	0.00689721	0
0	0	0	0.068266454	0	0.092476779	0	0.01624111	0
0	0	0	0.070712158	0	0.11318306	0	0.013570439	0
0	0.47681383	0	0.14431315	0	0	0	-0.018344606	0
0	0.47825387	0	0	0	0.14486464	0	-0.018697441	0
0	0.45059613	0	0.077941011	0	0.074259226	0	0	0
0	0	0	0	0	0	0	-0.009998903	0
0	0.46762609	0	0.15089657	0	0	0	0	0
0	0.46864733	0	0	0	0.15172928	0	0	0
0	0	0	0.07834078	0	0.11410332	0	0.0040352	0
0	0	0	0	0	0.14195217	0	-0.012790593	0
0	0	0	0.068026393	0	0.071497285	0	-0.016008805	0
0	0.45449606	0	0.081551609	0	0.080135854	0	0	0
0	0	0	0.069301001	0	0.074460162	0	0	0
0	0	0	0.14112759	0	0	0	-0.012512913	0
0	0.4836281	0	0.074712244	0	0.070866421	0	0	0
0	0	0	0	0	0.14415646	0	-0.014906024	0
0	0	0	0.14341663	0	0	0	-0.014615458	0
0	-0.13256117	0	0	0	0	0	0	0
0	0	0	0.072189592	0	0.07270017	0	0	0
0	0	0	0	0	0.13831321	0	-0.015192524	0
0	0	0	0	0	0.1443236	0	0	0
0	0	0	0.13775258	0	0	0	-0.014892676	0
0	0	0	0.1434927	0	0	0	0	0
0	0	0	0.069606965	0	0.069558731	0	-0.010089991	0
0	0	0	0.068472276	0	0.070336181	0	0	0

0	-0.15674276	0	0	0	0	0	-0.007900107	0
0	0	0	0	0	0	0	-0.018363893	0
0	0.44583693	0	0.07591897	0	0.060109571	0	-0.018035896	0
0	-0.13275993	0	0	0	0	0	-0.009239455	0
0	0	0	0.070191767	0	0.059537733	0	-0.014666793	0
0	-0.14056887	0	0.068024474	0	0.072924775	0	-0.015578112	0
0	-0.14654865	0	0	0	0.14311738	0	-0.01262021	0
0	-0.14522434	0	0.069240166	0	0.075670581	0	0	0
0	-0.14765978	0	0.1422578	0	0	0	-0.012340214	0
0	0	0	0.077149045	0	0.079463468	0	-0.021151548	0
0	-0.14001574	0	0	0	0.13969719	0	-0.014400008	0
0	-0.1414699	0	0.13909522	0	0	0	-0.014087809	0
0	-0.14692813	0	0	0	0.14472926	0	0	0
0	0	0	0.076475849	0	0.075610711	0	-0.018194593	0
0	-0.13585406	0	0.068292183	0	0.071918584	0	0	0
0	-0.14805553	0	0.14387075	0	0	0	0	0
0	0	0	0	0	0.15157703	0	-0.018599074	0
0	0	0	0.15068834	0	0	0	-0.018242778	0
0	-0.176869	0	0	0	0.14430303	0	-0.014013384	0
0	-0.17667268	0	0.14356695	0	0	0	-0.013722498	0
0	-0.17226014	0	0.072095005	0	0.072987921	0	0	0
0	-0.17501311	0	0.069861237	0	0.069758129	0	-0.00945328	0
0	0	0	0.073292801	0	0.071869025	0	-0.018693174	0
0	0	0	0.076197092	0	0.076331473	0	0	0
0	-0.16592257	0	0	0	0	0	-0.018251161	0
0	-0.17578523	0	0.070347692	0	0.059535532	0	-0.013769689	0
0	-0.16535462	0	0.077497602	0	0.080630485	0	-0.021231954	0
0	-0.15617027	0	0	0	0.15186506	0	-0.017805193	0
0	-0.15718779	0	0.15094886	0	0	0	-0.017445198	0
0	0.46359143	0	0.090320098	0	0.068231037	0	-0.051973618	0
0	-0.17844238	0	0.076549877	0	0.075534926	0	-0.017274083	0
0	-0.14277037	0	0.073082126	0	0.073297986	0	-0.017888181	0
0	-0.15152637	0	0.075770965	0	0.077053838	0	0	0
0	0.44533967	0	0	0	0	0	-0.029711496	0
0	0	0	0.13467018	0	0	0	0.025470559	0
0	0	0	0	0	0.13457485	0	0.02529013	0
0	0	0	0.086976249	0	0.05724305	0	0.020183879	0
0	0	0	0.08873849	0	0.051775797	0	0.023241782	0
0	0.42671723	0	0	0	0	0	0	0
0	0	0	0.1424331	0	0	0	0.020528495	0
0	0	0	0.13930055	0	0	0	0.022386206	0
0	0	0	0	0	0.14247853	0	0.020284703	0
0	0	0	0.13635498	0	0	0	0	0
0	0	0	0	0	0.13931253	0	0.022100672	0
0	0	0	0.084372638	0	0.053107162	0	0	0
0	0	0	0	0	0.1362642	0	0	0
0	0	0	0.085794088	0	0.057873886	0	0	0

0	0	0	0	0	0	0	-0.026881364	0
0	0	0	0.086967771	0	0.054650466	0	0	0
0	0	0	0	0	0	0	0	0
0	0.46919115	0	0	0	0	0	0	0
0	0	0	0	0	0	0	0.005444472	0
0	0.44112149	0	0	0	0	0	0	0
0	-0.19386341	0	0	0	0	0	-0.026834644	0
0	0	0	0.097652536	0	0.062744931	0	0.012255098	0
0	0.44131637	0	0	0	0	0	-0.001130718	0
0	0.44227704	0	0	0	0	0	0	0
0	0	0	0	0	0	0	-0.003842144	0
0	0	0	0	0	0	0	0	0
0	0	0	0	0	0	0	-0.004461577	0
0	0	0	0	0	0	0	0.002574262	0
0	0.42533926	0	0	0	0	0	0	0
0	0.4373248	0	0	0	0	0	-0.00368837	0
0	0	0	0	0	0	0	0	0
0	-0.15289472	0	0	0	0	0	-0.003568239	0
0	-0.152431	0	0	0	0	0	0	0
0	0.4611647	0	0	0	0	0	0	0
0	0.39368999	0	0	0	0	0	-0.04401833	0
0	0.41340513	0	0	0	0	0	0	0
0	0.43684848	0	0	0	0	0	-0.008731892	0
0	-0.18356579	0	0	0	0	0	-0.003573429	0
0	0.40360521	0	0	0	0	0	0	0
0	0	0	0	0	0	0	-0.003961585	0
0	0.40682068	0	0	0	0	0	-0.000258795	0
0	0.4351631	0	0	0	0	0	-0.017459184	0
0	0.44520238	0	0	0	0	0	0.008609389	0
0	-0.21088975	0	0	0	0	0	0	0
0	0	0	0	0	0	0	-0.004042659	0
0	0.42886921	0	0	0	0	0	0.008043843	0
0	0	0	0	0	0	0	-0.009121082	0
0	0	0	0	0	0	0	0.008524698	0
0	0.43826632	0	0	0	0	0	-0.0083112	0
0	0	0	0.062974254	0	0.089345746	0	0	0
0	-0.18207398	0	0	0	0	0	-0.003081701	0
0	0	0	0	0	0.15125807	0	-0.001885046	0
0	0	0	0	0	0	0	-0.00806356	0
0	0	0	0.14995271	0	0	0	-0.001441264	0
0	0	0	0.16745278	0	0	0	0.028676874	0
0	0	0	0	0	0.16776183	0	0.028314907	0
0	0.41737659	0	0	0	0	0	-0.008320768	0
0	0	0	0	0	0	0	-0.004419429	0
0	-0.19689294	0	0	0	0	0	-0.003141211	0
0	0	0	0.091517754	0	0.082623061	0	0.026194168	0
0	0	0	0.081605271	0	0.093653848	0	0.026189944	0

0	0	0	0.066448852	0	0.096139538	0	-0.007445305	0
0	-0.16129849	0	0	0	0	0	-0.008329257	0
0	0.39301093	0	0	0	0	0	0	0
0	0	0	0.06548176	0	0.10147405	0	0.000595267	0
0	-0.20545183	0	0	0	0	0	0.0090207	0
0	0	0	0.086655054	0	0.085009061	0	0	0
0	-0.21963397	0	0	0	0	0	-0.007169108	0
0	0	0	0.082765365	0	0.084227497	0	0.038355306	0
0	0.39757547	0	0	0	0	0	0.00184049	0
0	0	0	0.069340253	0	0.091291146	0	-0.005445064	0
0	0	0	0	0	0	0	-0.006823191	0
0	0.38225304	0	0	0	0	0	-0.011091806	0
0	-0.23004759	0	0	0	0	0	-0.005944657	0
0	0.44831852	0	0	0	0	0	-0.007503235	0
0	0	0	0	0	0	0	0.009388026	0
0	0.44098248	0	0	0	0	0	-0.010471188	0
0	0.4570226	0	0	0	0	0	0	0
0	0.43235065	0	0	0	0	0	-0.017990869	0
0	0.42001993	0	0	0	0	0	-0.00993054	0
0	0	0	0	0	0	0	-0.010933694	0
0	0	0	0	0	0	0	0.029371168	0
0	0	0	0.14234718	0	0	0	0.034185272	0
0	-0.16582507	0	0	0	0	0	-0.010134768	0
0	0	0	0	0	0.14237748	0	0.03393797	0
0	0	0	0.086026577	0	0.047809923	0	0.03405938	0
0	0	0	0.083124225	0	0.05057569	0	0.040850718	0
0	0	0	0.092541854	0	0.057904855	0	0.031216051	0
0	0.44413073	0	0	0	0	0	-0.016504353	0
0	0	0	0.086032128	0	0.060028056	0	0	0
0	0	0	0	0	0	0	0.021309952	0
0	0	0	0.080872625	0	0.071757541	0	0.027594303	0
0	0.47504751	0	0.12924849	0	0	0	0	0
0	0.49646415	0	0.13637573	0	0	0	0	0
0	0.4748477	0	0	0	0.12936452	0	0	0
0	0.4973741	0	0	0	0.13678009	0	0	0
0	0.47498241	0	0.13248069	0	0	0	-0.010182226	0
0	0.4780965	0	0.077362229	0	0.057436598	0	0	0
0	0.47841116	0	0.13105461	0	0	0	0	0
0	0.47473437	0	0	0	0.13257471	0	-0.010347775	0
0	0.4782239	0	0	0	0.13123633	0	0	0
0	0.4695008	0	0.13730258	0	0	0	-0.012263688	0
0	0.48841222	0	0.14254942	0	0	0	0	0
0	0.4726287	0	0.083994845	0	0.0566065	0	0	0
0	0.47435087	0	0.13468274	0	0	0	0	0
0	0.50332591	0	0.13626989	0	0	0	0	0
0	0.46924972	0	0	0	0.13760922	0	-0.012653287	0
0	0.48877355	0	0	0	0.14312312	0	0	0

0	0.50434073	0	0	0	0.13665584	0	0	0
0	0.47423266	0	0	0	0.13484696	0	0	0
0	0.47479437	0	0.1355238	0	0	0	-0.011540817	0
0	0.47853474	0	0.080575877	0	0.056343533	0	0	0
0	0.47446648	0	0	0	0.13578444	0	-0.011862587	0
0	0.47121833	0	0.083867887	0	0.06406638	0	-0.019092573	0
0	0.47762073	0	0.14865854	0	0	0	-0.02057854	0
0	0.47825156	0	0	0	0.14926743	0	-0.021023006	0
0	0	0	0.13217271	0	0	0	0	0
0	0	0	0	0	0.13250791	0	0	0
0	0.4773138	0	0.087792754	0	0.066553383	0	0	0
0	0	0	0.077695553	0	0.0531045	0	0	0
0	0	0	0.12914222	0	0	0	-0.007716961	0
0	0	0	0.13042961	0	0	0	0	0
0	0	0	0.13174744	0	0	0	0	0
0	0	0	0.13563481	0	0	0	-0.012674077	0
0	0	0	0.076120244	0	0.061863668	0	0	0
0	0	0	0	0	0.12926826	0	-0.007983404	0
0	0	0	0	0	0.136045	0	-0.012950935	0
0	0	0	0	0	0.13211416	0	0	0
0	0	0	0.076559114	0	0.057632788	0	-0.010768726	0
0	0	0	0.14019015	0	0	0	-0.014316509	0
0	0	0	0.13522329	0	0	0	0	0
0	0	0	0	0	0.13055317	0	0	0
0	0	0	0.076672262	0	0.055916828	0	0	0
0	0	0	0.13137148	0	0	0	-0.009736866	0
0	0	0	0.081687371	0	0.056551481	0	-0.012360435	0
0	0	0	0.083677326	0	0.059739049	0	0	0
0	0	0	0	0	0.13558096	0	0	0
0	0	0	0	0	0.14075013	0	-0.014807802	0
0	0	0	0	0	0.13159905	0	-0.010007541	0
0	-0.12024557	0	0.13305595	0	0	0	0	0
0	0	0	0.13453103	0	0	0	-0.010816869	0
0	0	0	0.081374374	0	0.054495277	0	0	0
0	0	0	0.13605486	0	0	0	-0.01201466	0
0	0	0	0.078301852	0	0.059227233	0	0	0
0	-0.11943009	0	0	0	0.13344449	0	0	0
0	0	0	0	0	0.13648686	0	-0.012355995	0
0	0	0	0	0	0.13474509	0	-0.011159278	0
0	0.46296193	0	0.14893612	0	0	0	0	0
0	0	0	0.079565622	0	0.057222947	0	-0.011852139	0
0	0.46308855	0	0	0	0.14914731	0	0	0
0	-0.11451396	0	0.13202171	0	0	0	0	0
0	-0.12656145	0	0.1366803	0	0	0	-0.012495952	0
0	-0.11360465	0	0	0	0.13240219	0	0	0
0	-0.14846105	0	0.077622149	0	0.053648248	0	0	0
0	-0.12431364	0	0.076439224	0	0.062587294	0	0	0

0	-0.11842746	0	0.13533554	0	0	0	0	0
0	-0.15049042	0	0.12957767	0	0	0	-0.007133584	0
0	0	0	0.083540613	0	0.065902227	0	-0.020431948	0
0	-0.12566583	0	0	0	0.13711466	0	-0.012773804	0
0	0.46320161	0	0.083080456	0	0.071863833	0	0	0
0	-0.14893016	0	0.13055446	0	0	0	0	0
0	-0.13314746	0	0.14132211	0	0	0	-0.014185543	0
0	-0.11780136	0	0	0	0.13579146	0	0	0
0	0.45950675	0	0.15353196	0	0	0	-0.010813722	0
0	-0.15067577	0	0	0	0.12970294	0	-0.007404568	0
0	-0.14850984	0	0.076861689	0	0.057738726	0	-0.010153931	0
0	0.45941291	0	0	0	0.15381626	0	-0.011178113	0
0	-0.14904357	0	0	0	0.1306829	0	0	0
0	-0.13178112	0	0.083828354	0	0.060730085	0	0	0
0	-0.1326587	0	0	0	0.14190447	0	-0.014676343	0
0	-0.11981132	0	0.13623728	0	0	0	-0.011243474	0
0	-0.14498273	0	0.076849126	0	0.055838906	0	0	0
0	-0.11595496	0	0.07786788	0	0.059875672	0	0	0
0	0.46079643	0	0.15290565	0	0	0	0	0
0	-0.14786828	0	0.13143889	0	0	0	-0.008874959	0
0	-0.15365499	0	0.082076799	0	0.05652883	0	-0.011761377	0
0	-0.11903948	0	0	0	0.13669342	0	-0.011586201	0
0	0.46223726	0	0	0	0.15339013	0	0	0
0	-0.14897733	0	0.081844076	0	0.054021443	0	0	0
0	-0.15209108	0	0.13449322	0	0	0	-0.009947638	0
0	-0.14816912	0	0	0	0.13165824	0	-0.009146282	0
0	-0.15232021	0	0	0	0.13470474	0	-0.010290194	0
0	-0.14852026	0	0.079601823	0	0.057123018	0	-0.010958007	0
0	-0.14249839	0	0.083795799	0	0.067087984	0	-0.020512139	0
0	0	0	0.079204775	0	0.071202108	0	0	0
0	0	0	0.14914192	0	0	0	-0.00899696	0
0	0.46905296	0	0.076881636	0	0.083962383	0	0	0
0	0	0	0	0	0.14938781	0	-0.009322416	0
0	0	0	0.14839505	0	0	0	0	0
0	0.46520296	0	0	0	0.15974293	0	-0.011343365	0
0	0.45597849	0	0	0	0.15633482	0	0	0
0	0.45426173	0	0.15577816	0	0	0	0	0
0	0	0	0	0	0.14883668	0	0	0
0	0.46528251	0	0.159444	0	0	0	-0.011047308	0
0	0	0	0.082091138	0	0.072631531	0	-0.011183091	0
0	0	0	0.083012187	0	0.070958183	0	0	0
0	0.46467315	0	0.080087305	0	0.085575734	0	-0.013511787	0
0	0	0	0.1524326	0	0	0	-0.010932938	0
0	0.45396957	0	0.082154277	0	0.079677784	0	0	0
0	0	0	0	0	0.15295414	0	-0.011334004	0
0	0.44884046	0	0.16011113	0	0	0	-0.012856021	0
0	0.45043855	0	0	0	0.16073554	0	-0.013236654	0

0	0.4630444	0	0.072280001	0	0.076207198	0	0	0
0	-0.16018124	0	0.079323437	0	0.071534533	0	0	0
0	0.46634992	0	0.068593539	0	0.075777633	0	0.01050723	0
0	0.46456722	0	0	0	0.14706004	0	0.00527466	0
0	0.46456656	0	0.14659701	0	0	0	0.005771401	0
0	0.46688492	0	0	0	0.14793557	0	0	0
0	0.46538165	0	0.14719907	0	0	0	0	0
0	-0.16303132	0	0.14954459	0	0	0	-0.008145482	0
0	0.4696031	0	0.065289002	0	0.079061065	0	0	0
0	-0.16300196	0	0	0	0.14980512	0	-0.008485973	0
0	0.47142366	0	0	0	0.14458556	0	0.00966678	0
0	-0.1585478	0	0.1495594	0	0	0	0	0
0	0.47034077	0	0.14349076	0	0	0	0.010571707	0
0	-0.15706758	0	0	0	0.15003768	0	0	0
0	-0.16349879	0	0.082146829	0	0.072839649	0	-0.010295919	0
0	-0.15893169	0	0.082692009	0	0.072331816	0	0	0
0	-0.16307742	0	0.15341219	0	0	0	-0.010098946	0
0	-0.16182741	0	0	0	0.15398776	0	-0.010524465	0
0	0.4606867	0	0.07259627	0	0.081658021	0	0	0
0	0.46749855	0	0.072179086	0	0.083759884	0	0.001301491	0
0	0.46319264	0	0.070574382	0	0.081606727	0	0.004383721	0
0	0	0	0.078181391	0	0.078979712	0	0	0
0	0	0	0.076325281	0	0.084698459	0	-0.011581775	0
0	0.46096306	0	0	0	0.15306088	0	0.001457157	0
0	0	0	0	0	0.15622243	0	-0.011365582	0
0	0	0	0.15564039	0	0	0	-0.011040227	0
0	0.45960889	0	0.15231311	0	0	0	0.001965024	0
0	0	0	0.081075982	0	0.080159001	0	-0.013214928	0
0	0	0	0.072837491	0	0.075421905	0	0.004863819	0
0	0	0	0.072231016	0	0.076546204	0	0	0
0	0	0	0.068045964	0	0.076688509	0	0.010120085	0
0	0	0	0	0	0.14738374	0	0.004600522	0
0	0	0	0.14670948	0	0	0	0.005117708	0
0	-0.15788457	0	0.07648884	0	0.084849505	0	-0.010719085	0
0	-0.16532914	0	0.078023188	0	0.080450325	0	0	0
0	-0.16816067	0	0	0	0.15751445	0	-0.010586706	0
0	-0.16980922	0	0.15688197	0	0	0	-0.010257566	0
0	0	0	0.13536813	0	0	0	0	0
0	0	0	0	0	0.13601447	0	0	0
0	0	0	0.069269382	0	0.068413061	0	0	0
0	-0.17051085	0	0.080801894	0	0.081585146	0	-0.012407548	0
0	0	0	0.13568602	0	0	0	0.000616856	0
0	0.48427527	0	0.13367548	0	0	0	0	0
0	0	0	0	0	0.13638403	0	0.000225595	0
0	0	0	0.072866434	0	0.081042852	0	0.00110926	0
0	0.48563972	0	0	0	0.13386087	0	0	0
0	-0.15873676	0	0.073115204	0	0.075575094	0	0.005417764	0

0	-0.15482545	0	0.071125134	0	0.078727103	0	0	0
0	0	0	0.074270722	0	0.067662934	0	0	0
0	-0.14948615	0	0.068066639	0	0.077870328	0	0.010523257	0
0	-0.15306322	0	0	0	0.14851071	0	0.00507608	0
0	-0.15441175	0	0.14776529	0	0	0	0.005607999	0
0	0	0	0.14001655	0	0	0	-0.000754845	0
0	0	0	0.07344758	0	0.068954712	0	-0.001848454	0
0	0	0	0	0	0.14069917	0	-0.001209813	0
0	0.44793156	0	0.13457778	0	0	0	0	0
0	0	0	0.074399565	0	0.07283718	0	0	0
0	0.44785957	0	0	0	0.13477642	0	0	0
0	0	0	0.14526845	0	0	0	-0.003468076	0
0	0	0	0	0	0.14597696	0	-0.003880401	0
0	0.47974422	0	0.13856559	0	0	0	-0.011257163	0
0	0.48428804	0	0.08017273	0	0.059537771	0	0	0
0	0.47752215	0	0.14098663	0	0	0	0	0
0	-0.16052163	0	0.072842589	0	0.082378314	0	0.001461191	0
0	0.48099693	0	0	0	0.13878893	0	-0.011631632	0
0	0.44850625	0	0.077818571	0	0.046154391	0	0	0
0	0.44597008	0	0.12340924	0	0	0	-0.008034673	0
0	0.47851272	0	0	0	0.14140057	0	0	0
0	0.44581539	0	0	0	0.12290248	0	-0.008300656	0
0	0.49984521	0	0.12599108	0	0	0	0	0
0	0.50079517	0	0	0	0.12645047	0	0	0
0	0.44393668	0	0.14013645	0	0	0	-0.010859719	0
0	0.44742474	0	0.083347051	0	0.058218136	0	0	0
0	0.49397557	0	0.074188067	0	0.058954802	0	0	0
0	0.4437207	0	0	0	0.14040908	0	-0.011216113	0
0	0.49314233	0	0.13106644	0	0	0	-0.003825333	0
0	0.49366884	0	0	0	0.13170205	0	-0.004305779	0
0	0.48722617	0	0.070157882	0	0.065017558	0	0.003071982	0
0	0	0	0.074375903	0	0.079288377	0	-0.00836028	0
0	0.48646016	0	0.069298452	0	0.065682509	0	0	0
0	0	0	0.075176514	0	0.059326055	0	0	0
0	0	0	0.13344752	0	0	0	-0.008771614	0
0	0.44465465	0	0.081945048	0	0.046945406	0	-0.01093335	0
0	0	0	0.13533522	0	0	0	0	0
0	0	0	0	0	0.13366029	0	-0.009093214	0
0	0.48630856	0	0.13326067	0	0	0	0.00395777	0
0	0.4875992	0	0	0	0.13368192	0	0.003465008	0
0	0	0	0	0	0.13572525	0	0	0
0	0	0	0.078545485	0	0.057594619	0	0	0
0	0	0	0.13484956	0	0	0	-0.008552601	0
0	0	0	0	0	0.13508706	0	-0.008859863	0
0	0	0	0.079125134	0	0.060061165	0	-0.011597199	0
0	0	0	0.14076244	0	0	0	-0.011351416	0
0	0	0	0.080644919	0	0.061649162	0	0	0

0	-0.13563593	0	0.07500779	0	0.06087475	0	0	0
0	-0.13923751	0	0.13475932	0	0	0	-0.007966981	0
0	0	0	0	0	0.1412402	0	-0.011737982	0
0	0	0	0.07716497	0	0.046938629	0	-0.008542995	0
0	-0.13935282	0	0.13567954	0	0	0	0	0
0	-0.13795089	0	0	0	0.13501154	0	-0.008296902	0
0	-0.13833233	0	0	0	0.13609819	0	0	0
0	0	0	0.082317174	0	0.059122169	0	-0.011214085	0
0	0.44942987	0	0.072265665	0	0.048730541	0	0	0
0	0.44991767	0	0.073443147	0	0.049163344	0	0.002328863	0
0	0.45056049	0	0.072333401	0	0.051266114	0	0.004127641	0
0	0.45030528	0	0.11988423	0	0	0	0.003284479	0
0	0	0	0.069715635	0	0.05784711	0	0	0
0	0	0	0.086687456	0	0.079534193	0	-0.012679893	0
0	0.45010198	0	0	0	0.11993372	0	0.003016484	0
0	0	0	0.12591301	0	0	0	-0.001001841	0
0	0	0	0.073071044	0	0.058803073	0	-0.003834028	0
0	0	0	0	0	0.12636586	0	-0.001340196	0
0	-0.14009297	0	0.079446929	0	0.060921051	0	-0.010802991	0
0	-0.17555155	0	0.078672755	0	0.057652679	0	0	0
0	-0.17803316	0	0.13499664	0	0	0	-0.007667369	0
0	-0.14180702	0	0.08022593	0	0.062339594	0	0	0
0	-0.14547361	0	0.14100063	0	0	0	-0.010552285	0
0	0	0	0.06984514	0	0.0644427	0	0.003030618	0
0	-0.17822067	0	0	0	0.13523826	0	-0.007975269	0
0	-0.14458967	0	0	0	0.14148646	0	-0.010929235	0
0	-0.12080967	0	0.069814593	0	0.058625191	0	0	0
0	-0.12165677	0	0.12679557	0	0	0	-0.000709273	0
0	-0.17738911	0	0.077431994	0	0.046817255	0	-0.007647889	0
0	-0.12703062	0	0.07324096	0	0.059805385	0	-0.003718275	0
0	-0.12086598	0	0	0	0.12727263	0	-0.001048411	0
0	-0.17937697	0	0.082310276	0	0.059128032	0	-0.010290538	0
0	-0.13383655	0	0.069765293	0	0.065809295	0	0.00354112	0
0	0	0	0.072591361	0	0.048758273	0	0.002695011	0
0	-0.17327039	0	0.072806114	0	0.048940602	0	0.003343369	0
0	0.46935327	0	0	0	0	0	0	0
0	0	0	0.07475284	0	0.10167459	0	0	0
0	0.49449521	0	0	0	0	0	0	0
0	0	0	0	0	0.17437986	0	0.007912639	0
0	0.4724169	0	0	0	0	0	-0.005612553	0
0	0	0	0.17341075	0	0	0	0.008553274	0
0	0.46067899	0	0	0	0	0	-0.010831541	0
0	0	0	0.079587065	0	0.10126306	0	0.005759648	0
0	0.4686789	0	0	0	0	0	0	0
0	0	0	0.068930426	0	0.074187151	0	0	0
0	0	0	0.068476902	0	0.072592215	0	0.004745897	0
0	0	0	0	0	0.14162496	0	0.003576496	0

0	0	0	0.1407944	0	0	0	0.004005536	0
0	0	0	0	0	0	0	0	0
0	0	0	0.070322373	0	0.10284998	0	0.0187419	0
0	0	0	0	0	0	0	-0.0049465	0
0	0.48732837	0	0	0	0	0	0	0
0	0.46641092	0	0	0	0	0	-0.00858886	0
0	0	0	0	0	0	0	-0.006112654	0
0	0	0	0.074561881	0	0.074703612	0	0.000902328	0
0	0.48065434	0	0.067926385	0	0.072148836	0	-0.015155593	0
0	0.47508751	0	0	0	0.14203768	0	-0.012400361	0
0	0.47638295	0	0.06912174	0	0.074694967	0	0	0
0	0.47382338	0	0.141205	0	0	0	-0.012143585	0
0	-0.12798786	0	0	0	0	0	0	0
0	0.48709305	0	0	0	0	0	-0.009867148	0
0	0.4809157	0	0	0	0.13891481	0	-0.014840835	0
0	0.47945623	0	0.1383405	0	0	0	-0.014542576	0
0	0.47303235	0	0	0	0.1444698	0	0	0
0	0.44637994	0	0	0	0.14421073	0	-0.014541539	0
0	0.44669757	0	0.14346973	0	0	0	-0.014238338	0
0	0.47190533	0	0.14363154	0	0	0	0	0
0	0.45117307	0	0.072241949	0	0.072730903	0	0	0
0	0.48521743	0	0.06844915	0	0.070968323	0	0	0
0	0	0	0.072215522	0	0.089769564	0	0.006613339	0
0	0.44845755	0	0.069659089	0	0.069201156	0	-0.0094827	0
0	0	0	0	0	0	0	-0.011624268	0
0	-0.15522628	0	0	0	0	0	-0.004111077	0
0	0.45575987	0	0	0	0	0	-0.018003147	0
0	-0.12712822	0	0	0	0	0	-0.005324245	0
0	0.44751	0	0.070218445	0	0.059676025	0	-0.014300344	0
0	0	0	0.073706305	0	0.11337713	0	0.006398534	0
0	0	0	0	0	0	0	-0.008912582	0
0	0.45560858	0	0.077575753	0	0.079920478	0	-0.021312315	0
0	0.44488037	0	0.076900624	0	0.075558431	0	-0.018026757	0
0	0.46354327	0	0	0	0.15204961	0	-0.01845531	0
0	0.46242827	0	0.15115714	0	0	0	-0.018116603	0
0	-0.1543339	0	0	0	0	0	-0.011340401	0
0	0.47802152	0	0.074253657	0	0.071740708	0	-0.018578341	0
0	0.46841536	0	0.076451686	0	0.076540365	0	0	0
0	0	0	0.068134401	0	0.074935197	0	-0.012597151	0
0	0	0	0	0	0.14490445	0	-0.015128505	0
0	0	0	0.071344646	0	0.074113394	0	-0.014807636	0
0	0	0	0.14403601	0	0	0	-0.014809872	0
0	0	0	0.070275799	0	0.075311488	0	0	0
0	0	0	0.067777148	0	0.071650763	0	-0.015095314	0
0	-0.13492818	0	0	0	0	0	-0.008161394	0
0	-0.14661927	0	0.068600378	0	0.075613245	0	-0.012429197	0
0	-0.15111733	0	0	0	0.14529787	0	-0.014319605	0

0	-0.14010269	0	0.067648655	0	0.073154051	0	-0.014291408	0
0	-0.15215949	0	0.14438902	0	0	0	-0.014012072	0
0	-0.14728408	0	0.069778159	0	0.076209711	0	0	0
0	0	0	0.075110658	0	0.077722009	0	-0.018442517	0
0	-0.17669028	0	0.071522209	0	0.074073308	0	-0.013895767	0
0	-0.15644799	0	0.074656137	0	0.078458066	0	-0.017647958	0
0	0.42706387	0	0	0	0	0	-0.027012261	0
0	0	0	0.084927605	0	0.051232173	0	0.025349031	0
0	0	0	0.13494538	0	0	0	0.024484539	0
0	0	0	0	0	0.13490324	0	0.024251746	0
0	0	0	0.087003135	0	0.056945317	0	0.02044665	0
0	0	0	0.087978603	0	0.052858367	0	0.022246615	0
0	0	0	0.083378671	0	0.054438492	0	0	0
0	0	0	0	0	0	0	0.006441411	0
0	0.46859344	0	0	0	0	0	-0.003485082	0
0	0.46750907	0	0	0	0	0	0	0
0	0.43980184	0	0	0	0	0	-0.004101277	0
0	0.41019775	0	0	0	0	0	0	0
0	0.44110085	0	0	0	0	0	-0.003605498	0
0	0	0	0	0	0	0	-0.004624768	0
0	0.42427675	0	0	0	0	0	-0.003647538	0
0	0	0	0	0	0	0	-0.003866889	0
0	-0.15358096	0	0	0	0	0	-0.003802019	0
0	0.45854493	0	0	0	0	0	-0.008967169	0
0	0.41607547	0	0	0	0	0	0.009152944	0
0	0.40122907	0	0	0	0	0	-0.0078602	0
0	-0.21182014	0	0	0	0	0	-0.002961479	0
0	0	0	0.063002292	0	0.089406369	0	-0.001761321	0
0	0	0	0.087454419	0	0.081852367	0	0.028392559	0
0	0.39105869	0	0	0	0	0	-0.00643135	0
0	0.45380681	0	0	0	0	0	-0.010610631	0
0	0	0	0.086855026	0	0.057138982	0	0.03402178	0
0	0.50122756	0	0.13222435	0	0	0	0	0
0	0.50203715	0	0	0	0.13259316	0	0	0
0	0.47512007	0	0.077359159	0	0.053311973	0	0	0
0	0.50549629	0	0.13187852	0	0	0	0	0
0	0.49509122	0	0.13568054	0	0	0	-0.012282903	0
0	0.47306167	0	0.12898317	0	0	0	-0.007231975	0
0	0.49718672	0	0.076300266	0	0.061729078	0	0	0
0	0.47434902	0	0.13050308	0	0	0	0	0
0	0.49597647	0	0	0	0.13609731	0	-0.012564387	0
0	0.50632474	0	0	0	0.1322419	0	0	0
0	0.47287827	0	0	0	0.12910362	0	-0.00750226	0
0	0.47498464	0	0.076564936	0	0.057323423	0	-0.010163036	0
0	0.47415666	0	0	0	0.13063722	0	0	0
0	0.50114693	0	0.13568211	0	0	0	0	0
0	0.48776437	0	0.14091643	0	0	0	-0.014423433	0

0	0.47852242	0	0.076736084	0	0.055903285	0	0	0
0	0.47537186	0	0.13142578	0	0	0	-0.00939739	0
0	0.50206235	0	0	0	0.13601606	0	0	0
0	0.48907301	0	0.084067181	0	0.060113612	0	0	0
0	0.48833017	0	0	0	0.14148408	0	-0.01486525	0
0	0.46964752	0	0.0820567	0	0.056633219	0	-0.012256605	0
0	0.47506996	0	0	0	0.13164948	0	-0.009669161	0
0	0.49992629	0	0.13651037	0	0	0	-0.011889748	0
0	0.47100287	0	0.13490722	0	0	0	-0.010663872	0
0	0.50387406	0	0.07848732	0	0.059506768	0	0	0
0	0.47440194	0	0.08172504	0	0.054523832	0	0	0
0	0.50070751	0	0	0	0.13694545	0	-0.012233347	0
0	0.47077419	0	0	0	0.13511462	0	-0.011007546	0
0	0.47461895	0	0.079894805	0	0.057278261	0	-0.011697674	0
0	0.47854536	0	0.083949264	0	0.066346127	0	-0.020590575	0
0	0	0	0.075982183	0	0.057801535	0	0	0
0	0	0	0.13195473	0	0	0	-0.009341459	0
0	0	0	0.13120998	0	0	0	0	0
0	0	0	0	0	0.13233309	0	-0.00966698	0
0	0	0	0	0	0.13150933	0	0	0
0	0	0	0.076817064	0	0.053754628	0	-0.007789377	0
0	0	0	0.075336366	0	0.061949913	0	-0.012738331	0
0	0	0	0.074399725	0	0.059059863	0	0	0
0	0	0	0.07805126	0	0.053936323	0	0	0
0	0	0	0.13214936	0	0	0	-0.009943284	0
0	0	0	0.13079771	0	0	0	-0.008888564	0
0	0	0	0	0	0.13254526	0	-0.010268767	0
0	0	0	0.08142033	0	0.060388272	0	-0.014405186	0
0	0	0	0.13546647	0	0	0	-0.011090115	0
0	0	0	0.079401692	0	0.05748857	0	0	0
0	0	0	0	0	0.13096956	0	-0.009179934	0
0	0	0	0.076254904	0	0.056751762	0	-0.009903179	0
0	0	0	0	0	0.13584602	0	-0.011449435	0
0	-0.1195071	0	0.075848838	0	0.058839444	0	0	0
0	0	0	0.080055246	0	0.056091869	0	-0.010941633	0
0	0	0	0.077514884	0	0.060285378	0	-0.012187365	0
0	-0.12160642	0	0.13284731	0	0	0	-0.009043167	0
0	0.46323613	0	0.079300014	0	0.071264113	0	0	0
0	-0.11669667	0	0.13149562	0	0	0	0	0
0	-0.12083325	0	0	0	0.13325558	0	-0.009380178	0
0	-0.11575931	0	0	0	0.13182823	0	0	0
0	0.46022205	0	0.14928714	0	0	0	-0.008653239	0
0	0.46014795	0	0	0	0.14954363	0	-0.008993815	0
0	0.46241876	0	0.14884073	0	0	0	0	0
0	-0.11395903	0	0.074060717	0	0.05968847	0	0	0
0	0.46398619	0	0	0	0.14929081	0	0	0
0	-0.11730923	0	0.13239379	0	0	0	-0.009205848	0

0	-0.12571796	0	0.075336122	0	0.063024004	0	-0.012564108	0
0	-0.11647907	0	0	0	0.13281185	0	-0.009493099	0
0	-0.11816419	0	0.07879639	0	0.05833272	0	0	0
0	-0.12168444	0	0.13566112	0	0	0	-0.01032645	0
0	-0.15042943	0	0.077030451	0	0.053981777	0	-0.007206567	0
0	0.45972113	0	0.082465401	0	0.072718937	0	-0.011017909	0
0	-0.13231681	0	0.081494713	0	0.061464104	0	-0.014270467	0
0	-0.14891324	0	0.077972358	0	0.054146065	0	0	0
0	-0.12087453	0	0	0	0.13606257	0	-0.010691337	0
0	-0.15147249	0	0.13088769	0	0	0	-0.008055769	0
0	0.46205916	0	0.083254666	0	0.071452572	0	0	0
0	-0.11930989	0	0.077138726	0	0.060862296	0	-0.011417182	0
0	-0.15166099	0	0	0	0.13105098	0	-0.008336192	0
0	0.45762509	0	0.15315917	0	0	0	-0.010770792	0
0	-0.14795497	0	0.076449492	0	0.056620171	0	-0.00904102	0
0	0.45896146	0	0	0	0.15368853	0	-0.011175079	0
0	-0.15212587	0	0.080792998	0	0.05529515	0	-0.010107759	0
0	0	0	0.078805194	0	0.071989243	0	-0.009208187	0
0	0.46541061	0	0.076420542	0	0.084719938	0	-0.011236786	0
0	0.45577865	0	0.07807023	0	0.07957147	0	0	0
0	0	0	0.079320994	0	0.070867203	0	0	0
0	0.45268069	0	0	0	0.15673302	0	-0.011048471	0
0	0	0	0.14877806	0	0	0	-0.008979334	0
0	0.45116109	0	0.15612953	0	0	0	-0.010713182	0
0	0	0	0	0	0.14926744	0	-0.009304244	0
0	0	0	0.082283305	0	0.071970148	0	-0.01114899	0
0	0.45030516	0	0.081335911	0	0.080693997	0	-0.013062714	0
0	0.46485598	0	0.072775976	0	0.075558451	0	0.005510399	0
0	0.46654257	0	0.071966141	0	0.077186366	0	0	0
0	-0.16279838	0	0.079002303	0	0.072200797	0	-0.008359857	0
0	-0.15728583	0	0.079132612	0	0.07225254	0	0	0
0	0.47155308	0	0.068351723	0	0.077142957	0	0.010205527	0
0	0.46833953	0	0	0	0.14774634	0	0.005213588	0
0	0.46699626	0	0.1470151	0	0	0	0.005740755	0
0	-0.16100371	0	0.14991134	0	0	0	-0.008168749	0
0	-0.15961759	0	0	0	0.15042972	0	-0.008520932	0
0	-0.16184672	0	0.079582094	0	0.07559868	0	-0.010198259	0
0	0	0	0.077688683	0	0.079861111	0	-0.011248669	0
0	0.46094544	0	0.072930897	0	0.081334447	0	0.001692676	0
0	0	0	0.072917662	0	0.07570494	0	0.004810605	0
0	-0.16833751	0	0.077505707	0	0.081333843	0	-0.010469876	0
0	0	0	0.067445478	0	0.069951967	0	0	0
0	0	0	0.13529339	0	0	0	0.001449325	0
0	0	0	0	0	0.13595425	0	0.001040834	0
0	0	0	0.069261178	0	0.068402861	0	0.000361493	0
0	0.4853534	0	0.075169018	0	0.05994766	0	0	0
0	0.48172783	0	0.13403131	0	0	0	-0.00843822	0

0	0.48060698	0	0.13547631	0	0	0	0	0
0	0.4830107	0	0	0	0.1342556	0	-0.008755949	0
0	0.48169102	0	0	0	0.13587198	0	0	0
0	-0.15316341	0	0.072644572	0	0.077079236	0	0.005299669	0
0	0	0	0.074200283	0	0.067771031	0	-0.001010815	0
0	0.44799904	0	0.07863968	0	0.057578764	0	0	0
0	0.4453323	0	0.13491641	0	0	0	-0.008197441	0
0	0	0	0.074069651	0	0.073145307	0	-0.003663544	0
0	0.44515094	0	0	0	0.13515757	0	-0.008501236	0
0	0.48090286	0	0.079409481	0	0.060611626	0	-0.011447956	0
0	0.47799143	0	0.080853129	0	0.061921261	0	0	0
0	0.47416158	0	0.14122983	0	0	0	-0.011238144	0
0	0.47516043	0	0	0	0.14169248	0	-0.011582417	0
0	0.44599189	0	0.077406315	0	0.046870179	0	-0.008172977	0
0	0.50059706	0	0.069702181	0	0.057891901	0	0	0
0	0.44392988	0	0.082636791	0	0.059183407	0	-0.011031286	0
0	0.4998583	0	0.12596503	0	0	0	-0.00063146	0
0	0.50064307	0	0	0	0.12642294	0	-0.000969106	0
0	0.49395539	0	0.073459265	0	0.059169178	0	-0.003910865	0
0	0	0	0.074753031	0	0.060143061	0	-0.008966405	0
0	0	0	0.075828779	0	0.061274293	0	0	0
0	0.48752376	0	0.06965381	0	0.06516837	0	0.003679895	0
0	0	0	0.13570419	0	0	0	-0.008780453	0
0	0	0	0	0	0.1361377	0	-0.00910031	0
0	0	0	0.078161363	0	0.058362993	0	-0.008737484	0
0	0	0	0.079898624	0	0.062672992	0	-0.011545077	0
0	-0.13806981	0	0.074633542	0	0.061611798	0	-0.008170052	0
0	-0.13872439	0	0.075392464	0	0.062076142	0	0	0
0	-0.14173948	0	0.1360143	0	0	0	-0.007976186	0
0	-0.14081059	0	0	0	0.13647477	0	-0.008298729	0
0	0.45023006	0	0.072576337	0	0.048581606	0	0.003218776	0
0	0	0	0.069842903	0	0.057666624	0	-0.001124794	0
0	-0.17804763	0	0.078347703	0	0.058322004	0	-0.007847409	0
0	-0.14488295	0	0.079460461	0	0.063364555	0	-0.010743678	0
0	-0.12095769	0	0.070534753	0	0.057891965	0	-0.000811989	0
0	0.49188897	0	0	0	0	0	0	0
0	0.46788976	0	0	0	0	0	-0.004611235	0
0	0	0	0.075195914	0	0.1004839	0	0.00805146	0
0	0.49263431	0	0	0	0	0	-0.005791689	0
0	0.46702297	0	0	0	0	0	-0.011282041	0
0	0	0	0.069171913	0	0.073735177	0	0.003712333	0
0	0	0	0	0	0	0	-0.005070445	0
0	0.48481202	0	0	0	0	0	-0.008789488	0
0	0.47501476	0	0.068011106	0	0.075129158	0	-0.012210642	0
0	0.46869135	0	0	0	0.1450477	0	-0.014790232	0
0	0.46763956	0	0.14416441	0	0	0	-0.0144545	0
0	0.4808099	0	0.06780577	0	0.072218455	0	-0.014743177	0

0	0.44664845	0	0.071436309	0	0.07406299	0	-0.014462152	0
0	0.4725366	0	0.07008767	0	0.075654537	0	0	0
0	-0.12927966	0	0	0	0	0	-0.004297455	0
0	0.46305453	0	0.07515153	0	0.078161227	0	-0.018321814	0
0	0	0	0.069567138	0	0.076603262	0	-0.015016891	0
0	-0.15144068	0	0.069161596	0	0.077380181	0	-0.014218101	0
0	0	0	0.084202678	0	0.052247025	0	0.024335236	0
0	0.46619916	0	0	0	0	0	-0.004285562	0
0	0.40918914	0	0	0	0	0	-0.003467387	0
0	0.5019494	0	0.075806445	0	0.058032455	0	0	0
0	0.49988991	0	0.13200497	0	0	0	-0.009009261	0
0	0.50313849	0	0.13134502	0	0	0	0	0
0	0.5007034	0	0	0	0.13240499	0	-0.009368765	0
0	0.50414074	0	0	0	0.13165086	0	0	0
0	0.49592715	0	0.075205788	0	0.062130194	0	-0.012350028	0
0	0.50596412	0	0.074270641	0	0.059324048	0	0	0
0	0.50253146	0	0.13226836	0	0	0	-0.009658979	0
0	0.47312957	0	0.076818409	0	0.053592488	0	-0.007301419	0
0	0.4743677	0	0.077921936	0	0.054142673	0	0	0
0	0.50329179	0	0	0	0.13267395	0	-0.009976781	0
0	0.47165405	0	0.13085316	0	0	0	-0.008555114	0
0	0.49795543	0	0.13592295	0	0	0	-0.010976731	0
0	0.50170392	0	0.07953161	0	0.057830392	0	0	0
0	0.48853831	0	0.081797675	0	0.060758551	0	-0.014485261	0
0	0.47143827	0	0	0	0.13102586	0	-0.008848012	0
0	0.47528106	0	0.076374252	0	0.056683833	0	-0.009556263	0
0	0.49877499	0	0	0	0.13630491	0	-0.011338818	0
0	0.50043051	0	0.077727241	0	0.060534662	0	-0.012062624	0
0	0.47096454	0	0.081033323	0	0.055471072	0	-0.010826501	0
0	0	0	0.075171575	0	0.058409696	0	-0.009462846	0
0	0	0	0.075459142	0	0.057423908	0	0	0
0	0	0	0.13159327	0	0	0	-0.009075516	0
0	0	0	0	0	0.13193244	0	-0.009377815	0
0	0	0	0.073942891	0	0.059949247	0	-0.010153042	0
0	0	0	0.077433521	0	0.054956136	0	-0.009055186	0
0	0	0	0.078534779	0	0.058636898	0	-0.011267013	0
0	-0.12089122	0	0.075184829	0	0.059311918	0	-0.009165233	0
0	-0.11613942	0	0.075063551	0	0.058125109	0	0	0
0	0.46043536	0	0.07889768	0	0.072042122	0	-0.008865181	0
0	-0.11917532	0	0.13184366	0	0	0	-0.008314316	0
0	0.46368281	0	0.079254662	0	0.071384356	0	0	0
0	-0.11832011	0	0	0	0.13221109	0	-0.008623178	0
0	0.4599025	0	0.14920938	0	0	0	-0.008632621	0
0	-0.11680632	0	0.073570128	0	0.060584608	0	-0.00938381	0
0	0.46128055	0	0	0	0.14970485	0	-0.008981777	0
0	-0.12117075	0	0.07818255	0	0.059201211	0	-0.010509742	0
0	-0.1514734	0	0.077591403	0	0.054876352	0	-0.008191466	0

0	0.45884173	0	0.082517115	0	0.072474432	0	-0.010982646	0
0	0.45260737	0	0.077647414	0	0.080397476	0	-0.010921918	0
0	0	0	0.078911286	0	0.071688786	0	-0.009198987	0
0	0.4682362	0	0.072525635	0	0.076429212	0	0.005436553	0
0	-0.15978645	0	0.078728427	0	0.073036608	0	-0.008393481	0
0	0	0	0.070015417	0	0.067228836	0	0.001188375	0
0	0.4827638	0	0.074750986	0	0.060748186	0	-0.00865279	0
0	0.48122603	0	0.075687062	0	0.061564023	0	0	0
0	0.47812562	0	0.1358287	0	0	0	-0.008437262	0
0	0.47905447	0	0	0	0.13627082	0	-0.008758879	0
0	0.44532394	0	0.078252014	0	0.058336775	0	-0.008374121	0
0	0.47471444	0	0.080047296	0	0.063002482	0	-0.011418484	0
0	0.50057196	0	0.06962386	0	0.057939222	0	-0.0007537	0
0	0	0	0.075392796	0	0.06210947	0	-0.008973578	0
0	-0.1411626	0	0.075003421	0	0.06282971	0	-0.008172102	0
0	0.49044916	0	0	0	0	0	-0.004758241	0
0	0.46821755	0	0.06945184	0	0.076860666	0	-0.01469012	0
0	0.50058442	0	0.075027587	0	0.058615741	0	-0.009102386	0
0	0.50372991	0	0.075353104	0	0.057668081	0	0	0
0	0.50056551	0	0.13171037	0	0	0	-0.00876944	0
0	0.50142207	0	0	0	0.13206163	0	-0.009077449	0
0	0.50303174	0	0.073835542	0	0.060179349	0	-0.009835272	0
0	0.4716068	0	0.077522881	0	0.054924626	0	-0.008722561	0
0	0.49846774	0	0.078787318	0	0.058848383	0	-0.011155965	0
0	0	0	0.075019452	0	0.05827353	0	-0.0092548	0
0	-0.11866522	0	0.074646653	0	0.058919777	0	-0.008497721	0
0	0.46111079	0	0.078844834	0	0.07219193	0	-0.008853759	0
0	0.47863075	0	0.07527804	0	0.062357644	0	-0.008645588	0
0	0.50105588	0	0.074897681	0	0.058520619	0	-0.008961249	0

SUPPLEMENTARY TABLE 4

Model	BIC	Loglike	H2r	SE	bsnp_rs2938864	SE	bsnp_rs1001646
cov0	0	-361.944	0.6608045	0	0	0	0
cov4	2.7186	-359.906	0.6654512	0	0	0	0
cov7	3.1998	-360.146	0.6433935	0	0	0	0
cov3	3.2282	-360.161	0.6423395	0	0	0	0
cov8	3.3258	-360.209	0.6437329	0	0	0	0
cov9	3.7979	-360.445	0.663795	0	0	0	0
cov2	4.085	-360.589	0.6625308	0	0	0	0.12871133
cov10	4.1421	-360.617	0.6645435	0	0	0	0
cov3_9	4.5877	-357.443	0.6350964	0	0	0	0
cov3_10	4.997	-357.648	0.6363289	0	0	0	0
cov6	5.4115	-361.252	0.6659444	0	0	0	0
cov1	5.4437	-361.268	0.6663568	0	0.090644777	0	0
cov5	5.8512	-361.472	0.6655897	0	0	0	0
cov2_3	6.1647	-358.232	0.6451598	0	0	0	0.15616198
cov11	6.4733	-361.783	0.6607402	0	0	0	0
cov4_7	6.6297	-358.464	0.65184	0	0	0	0
cov4_8	6.7366	-358.517	0.6521338	0	0	0	0
cov4_5	6.8464	-358.572	0.6555898	0	0	0	0
cov3_4	6.9971	-358.648	0.6521941	0	0	0	0
cov7_9	7.311	-358.805	0.6459777	0	0	0	0
cov8_9	7.4392	-358.869	0.6463696	0	0	0	0
cov2_7	7.4503	-358.874	0.6474389	0	0	0	0.12509183
cov4_9	7.522	-358.91	0.6670117	0	0	0	0
cov2_8	7.5727	-358.936	0.647753	0	0	0	0.1251831
cov7_10	7.63	-358.964	0.6466505	0	0	0	0
cov4_10	7.7318	-359.015	0.6678278	0	0	0	0
cov8_10	7.7583	-359.028	0.6470393	0	0	0	0
cov1_3	7.8074	-359.053	0.6488819	0	0.11821102	0	0
cov3_6	7.8784	-359.088	0.6490949	0	0	0	0
cov1_7	8.7685	-359.533	0.6503342	0	0.086733157	0	0
cov6_7	8.7934	-359.546	0.650083	0	0	0	0
cov3_11	8.8529	-359.576	0.6401217	0	0	0	0
cov1_8	8.891	-359.595	0.6506594	0	0.086856981	0	0
cov6_8	8.9293	-359.614	0.6504077	0	0	0	0
cov5_7	9.03	-359.664	0.6497425	0	0	0	0
cov4_11	9.0633	-359.681	0.667757	0	0	0	0
cov5_8	9.1539	-359.726	0.6500576	0	0	0	0
cov1_4	9.2655	-359.782	0.6634852	0	-0.057746464	0	0
cov7_11	9.266	-359.782	0.6429829	0	0	0	0
cov3_7	9.3096	-359.804	0.6393079	0	0	0	0
cov3_8	9.3762	-359.837	0.6394425	0	0	0	0
cov3_5	9.379	-359.839	0.6485275	0	0	0	0
cov4_6	9.3866	-359.842	0.6644448	0	0	0	0
cov8_11	9.4035	-359.851	0.6433228	0	0	0	0
cov2_4	9.5112	-359.905	0.6653684	0	0	0	0.00574008

cov2_9	9.5746	-359.936	0.6645454	0	0	0	0.08697783
cov3_4_9	9.7708	-356.637	0.6440412	0	0	0	0
cov2_10	9.7888	-360.044	0.6651264	0	0	0	0.091829699
cov7_8	9.884	-360.091	0.6435538	0	0	0	0
cov3_4_10	10.057	-356.78	0.645534	0	0	0	0
cov5_9	10.1098	-360.204	0.6669494	0	0	0	0
cov2_3_9	10.1263	-356.815	0.6395299	0	0	0	0.097296784
cov2_11	10.3526	-360.325	0.6641696	0	0	0	0.17993027
cov9_10	10.3597	-360.329	0.6614119	0	0	0	0
cov6_9	10.3902	-360.344	0.6656709	0	0	0	0
cov2_3_10	10.3904	-356.947	0.6408311	0	0	0	0.1021785
cov5_10	10.3924	-360.345	0.667869	0	0	0	0
cov1_9	10.3997	-360.349	0.6659986	0	0.038189026	0	0
cov9_11	10.5925	-360.445	0.6637949	0	0	0	0
cov1_2	10.6625	-360.48	0.6590576	0	-0.070179301	0	0.1887273
cov6_10	10.6666	-360.482	0.6666027	0	0	0	0
cov1_10	10.6755	-360.487	0.6669452	0	0.044125235	0	0
cov2_5	10.7276	-360.513	0.6600312	0	0	0	0.16326696
cov2_6	10.8042	-360.551	0.6610154	0	0	0	0.15870794
cov10_11	10.9334	-360.616	0.6645281	0	0	0	0
cov1_3_9	11.0771	-357.29	0.6391884	0	0.048445861	0	0
cov3_6_9	11.1219	-357.313	0.6388512	0	0	0	0
cov3_9_11	11.1785	-357.341	0.6347731	0	0	0	0
cov3_5_9	11.2434	-357.374	0.6383981	0	0	0	0
cov3_9_10	11.2613	-357.383	0.6337963	0	0	0	0
cov3_7_9	11.3786	-357.441	0.6349873	0	0	0	0
cov3_8_9	11.3819	-357.443	0.6350545	0	0	0	0
cov1_3_10	11.4005	-357.452	0.6408126	0	0.05445291	0	0
cov3_6_10	11.4545	-357.479	0.6404908	0	0	0	0
cov4_5_9	11.4942	-357.499	0.6568549	0	0	0	0
cov3_10_11	11.5327	-357.518	0.6359961	0	0	0	0
cov3_4_5	11.5917	-357.548	0.6443221	0	0	0	0
cov4_7_9	11.5943	-357.549	0.652698	0	0	0	0
cov3_5_10	11.6112	-357.557	0.6400567	0	0	0	0
cov4_5_7	11.6702	-357.587	0.645406	0	0	0	0
cov4_8_9	11.7047	-357.604	0.653026	0	0	0	0
cov2_3_5	11.7193	-357.612	0.6333588	0	0	0	0.26408561
cov4_5_10	11.7506	-357.627	0.6578409	0	0	0	0
cov4_5_8	11.7581	-357.631	0.6456594	0	0	0	0
cov3_7_10	11.7851	-357.644	0.6361828	0	0	0	0
cov3_8_10	11.7901	-357.647	0.6362559	0	0	0	0
cov4_7_10	11.7974	-357.651	0.6535151	0	0	0	0
cov4_8_10	11.9075	-357.706	0.6538514	0	0	0	0
cov1_11	11.9813	-361.14	0.6696195	0	0.13763	0	0
cov6_11	12.0104	-361.154	0.6683656	0	0	0	0
cov1_6	12.1688	-361.234	0.6663019	0	0.039968784	0	0
cov5_6	12.2047	-361.252	0.6660238	0	0	0	0

cov1_5	12.2337	-361.266	0.666264	0	0.099775998	0	0
cov1_4_9	12.4347	-357.969	0.6594409	0	-0.18439968	0	0
cov5_11	12.6333	-361.466	0.6661846	0	0	0	0
cov2_3_7	12.7264	-358.115	0.6434028	0	0	0	0.14868216
cov2_3_8	12.7623	-358.133	0.6435449	0	0	0	0.14933282
cov1_4_10	12.795	-358.149	0.6611078	0	-0.17620886	0	0
cov1_2_3	12.8419	-358.173	0.6430154	0	-0.051957903	0	0.20031182
cov2_3_11	12.8827	-358.193	0.646491	0	0	0	0.17523411
cov2_3_6	12.9188	-358.211	0.6440418	0	0	0	0.17806173
cov4_6_9	12.9239	-358.214	0.6632591	0	0	0	0
cov2_3_4	12.9376	-358.221	0.6463181	0	0	0	0.1375102
cov4_9_11	13.0428	-358.273	0.6704138	0	0	0	0
cov3_4_7	13.0578	-358.281	0.6493184	0	0	0	0
cov1_4_5	13.0683	-358.286	0.6555772	0	0.11831559	0	0
cov2_7_9	13.1115	-358.308	0.6483819	0	0	0	0.086175722
cov3_4_8	13.1236	-358.314	0.6494505	0	0	0	0
cov2_8_9	13.2366	-358.37	0.6487445	0	0	0	0.086313965
cov4_5_6	13.2453	-358.375	0.6549853	0	0	0	0
cov4_6_10	13.25	-358.377	0.6646031	0	0	0	0
cov1_4_7	13.2767	-358.39	0.6504514	0	-0.044946829	0	0
cov1_3_5	13.2768	-358.39	0.6394846	0	0.29945226	0	0
cov2_7_10	13.3069	-358.405	0.6489982	0	0	0	0.090856249
cov4_7_8	13.3133	-358.409	0.6519565	0	0	0	0
cov4_10_11	13.3262	-358.415	0.6712971	0	0	0	0
cov4_6_7	13.341	-358.422	0.6509918	0	0	0	0
cov4_7_11	13.3576	-358.431	0.6531929	0	0	0	0
cov1_4_8	13.381	-358.442	0.6507273	0	-0.045315544	0	0
cov2_4_7	13.3846	-358.444	0.6513603	0	0	0	0.026183146
cov2_8_10	13.4319	-358.468	0.6493515	0	0	0	0.090994195
cov4_6_8	13.442	-358.473	0.6512438	0	0	0	0
cov4_8_11	13.4586	-358.481	0.653539	0	0	0	0
cov2_4_8	13.4934	-358.499	0.6516697	0	0	0	0.025523354
cov2_4_5	13.5519	-358.528	0.6544388	0	0	0	0.039329989
cov5_7_9	13.5875	-358.546	0.6503611	0	0	0	0
cov4_5_11	13.6378	-358.571	0.6552185	0	0	0	0
cov5_8_9	13.714	-358.609	0.6507292	0	0	0	0
cov3_4_6	13.7265	-358.615	0.6521701	0	0	0	0
cov2_4_9	13.7425	-358.623	0.6681173	0	0	0	-0.1140194
cov1_3_4	13.752	-358.628	0.6521471	0	0.02552868	0	0
cov3_4_11	13.7917	-358.648	0.6522107	0	0	0	0
cov5_7_10	13.8462	-358.675	0.6512559	0	0	0	0
cov7_9_10	13.867	-358.685	0.6435869	0	0	0	0
cov1_7_9	13.9246	-358.714	0.6488373	0	0.037141288	0	0
cov6_7_9	13.9379	-358.721	0.6485096	0	0	0	0
cov5_8_10	13.9727	-358.738	0.6516207	0	0	0	0
cov7_8_9	13.9827	-358.743	0.6461075	0	0	0	0
cov8_9_10	13.9948	-358.749	0.643973	0	0	0	0

cov7_9_11	14.0031	-358.753	0.6457358	0	0	0	0
cov1_2_7	14.0105	-358.757	0.6436398	0	-0.073061636	0	0.18741934
cov2_4_10	14.0187	-358.761	0.6691042	0	0	0	-0.10715373
cov1_8_9	14.0511	-358.777	0.6492259	0	0.037326167	0	0
cov6_8_9	14.0699	-358.787	0.6488709	0	0	0	0
cov2_6_7	14.1311	-358.817	0.6452756	0	0	0	0.16192796
cov2_7_8	14.1325	-358.818	0.6475621	0	0	0	0.1252708
cov1_2_8	14.134	-358.819	0.6439683	0	-0.072892176	0	0.18737063
cov8_9_11	14.1359	-358.82	0.6461288	0	0	0	0
cov2_5_7	14.1426	-358.823	0.6451827	0	0	0	0.15360933
cov3_5_6	14.1456	-358.825	0.6446287	0	0	0	0
cov2_7_11	14.1479	-358.826	0.6486674	0	0	0	0.1475998
cov1_7_10	14.18	-358.842	0.6498542	0	0.042874193	0	0
cov4_9_10	14.1936	-358.849	0.6651826	0	0	0	0
cov6_7_10	14.1972	-358.85	0.6494945	0	0	0	0
cov2_6_8	14.2474	-358.876	0.6455195	0	0	0	0.16297711
cov2_8_11	14.264	-358.884	0.6490193	0	0	0	0.1484157
cov2_5_8	14.2653	-358.885	0.6455084	0	0	0	0.15367024
cov7_10_11	14.2839	-358.894	0.6463455	0	0	0	0
cov1_3_7	14.2862	-358.895	0.6466685	0	0.10917605	0	0
cov7_8_10	14.3024	-358.903	0.6467829	0	0	0	0
cov1_8_10	14.3063	-358.905	0.6502273	0	0.0430583	0	0
cov1_3_8	14.3285	-358.916	0.6468247	0	0.1098749	0	0
cov6_8_10	14.3299	-358.917	0.6498566	0	0	0	0
cov3_6_7	14.3512	-358.927	0.6467268	0	0	0	0
cov3_4_5_9	14.379	-355.544	0.6364391	0	0	0	0
cov3_6_8	14.4006	-358.952	0.6469065	0	0	0	0
cov8_10_11	14.4177	-358.961	0.6467253	0	0	0	0
cov1_3_6	14.5481	-359.026	0.6492006	0	0.073439398	0	0
cov1_3_11	14.5964	-359.05	0.6495255	0	0.12504503	0	0
cov3_6_11	14.6726	-359.088	0.6489385	0	0	0	0
cov3_4_5_10	14.7115	-355.71	0.6381465	0	0	0	0
cov3_7_11	15.0006	-359.252	0.6376574	0	0	0	0
cov3_8_11	15.0641	-359.284	0.6377728	0	0	0	0
cov3_5_7	15.3256	-359.415	0.6460317	0	0	0	0
cov3_5_8	15.3985	-359.451	0.6461349	0	0	0	0
cov1_7_8	15.452	-359.478	0.6504755	0	0.0868697	0	0
cov6_7_8	15.4607	-359.482	0.650263	0	0	0	0
cov1_6_7	15.5287	-359.516	0.6504652	0	0.050769968	0	0
cov1_5_7	15.5609	-359.532	0.6504226	0	0.080389059	0	0
cov5_6_7	15.5617	-359.533	0.6504668	0	0	0	0
cov1_7_11	15.5629	-359.533	0.6504629	0	0.088139689	0	0
cov6_7_11	15.5859	-359.545	0.6497161	0	0	0	0
cov3_5_11	15.6459	-359.575	0.6406394	0	0	0	0
cov1_6_8	15.6567	-359.58	0.6507938	0	0.053966155	0	0
cov1_5_8	15.6835	-359.594	0.6507458	0	0.080596358	0	0
cov1_8_11	15.6848	-359.594	0.6508984	0	0.08946542	0	0

cov5_6_8	15.6949	-359.599	0.6507999	0	0	0	0
cov2_4_11	15.7048	-359.604	0.6676366	0	0	0	0.056984309
cov5_7_8	15.7165	-359.61	0.6498727	0	0	0	0
cov6_8_11	15.7222	-359.613	0.6500866	0	0	0	0
cov2_9_11	15.7286	-359.616	0.666074	0	0	0	0.14159855
cov1_3_4_5	15.7299	-356.22	0.6355794	0	0.28834588	0	0
cov5_7_11	15.7578	-359.631	0.6479272	0	0	0	0
cov1_2_4	15.8274	-359.666	0.6605709	0	-0.10581839	0	0.082470422
cov1_4_11	15.8491	-359.676	0.6670228	0	-0.014086462	0	0
cov4_6_11	15.856	-359.68	0.6680054	0	0	0	0
cov5_8_11	15.8867	-359.695	0.648317	0	0	0	0
cov1_2_9	15.91	-359.707	0.6594194	0	-0.10350811	0	0.17104288
cov7_8_11	15.9525	-359.728	0.6430969	0	0	0	0
cov2_10_11	15.9661	-359.735	0.6666601	0	0	0	0.14568113
cov3_7_8	15.9766	-359.74	0.6394032	0	0	0	0
cov1_3_4_9	16.0426	-356.376	0.6420091	0	-0.10295052	0	0
cov1_4_6	16.0438	-359.774	0.6633452	0	-0.081564894	0	0
cov2_4_6	16.0852	-359.794	0.6631046	0	0	0	0.049573566
cov2_6_9	16.1099	-359.807	0.6619416	0	0	0	0.13994839
cov3_4_6_9	16.1486	-356.429	0.6426741	0	0	0	0
cov1_2_10	16.1623	-359.833	0.6603482	0	-0.098940942	0	0.17252537
cov2_9_10	16.1777	-359.841	0.6622966	0	0	0	0.085308192
cov2_3_5_9	16.3134	-356.511	0.6321854	0	0	0	0.18029988
cov2_6_10	16.3514	-359.928	0.6627307	0	0	0	0.14207287
cov2_5_9	16.3568	-359.93	0.6638305	0	0	0	0.098194915
cov1_3_4_10	16.401	-356.555	0.6438797	0	-0.095206387	0	0
cov3_4_9_11	16.4149	-356.562	0.6467228	0	0	0	0
cov4_5_7_9	16.4876	-356.598	0.646147	0	0	0	0
cov3_4_6_10	16.4918	-356.6	0.6444235	0	0	0	0
cov3_4_9_10	16.4958	-356.602	0.6428815	0	0	0	0
cov1_2_3_9	16.5166	-356.613	0.6349837	0	-0.097711389	0	0.17645865
cov5_9_11	16.5257	-360.015	0.6700498	0	0	0	0
cov3_4_7_9	16.5397	-356.624	0.6438634	0	0	0	0
cov3_4_8_9	16.5512	-356.63	0.6439146	0	0	0	0
cov2_3_4_9	16.5593	-356.634	0.6437213	0	0	0	0.012826685
cov2_5_10	16.5682	-360.036	0.6643122	0	0	0	0.10434344
cov2_3_5_10	16.5731	-356.641	0.6333907	0	0	0	0.18567272
cov4_5_8_9	16.5771	-356.643	0.6464461	0	0	0	0
cov2_3_6_9	16.6279	-356.669	0.6361894	0	0	0	0.1535394
cov1_5_9	16.7229	-360.113	0.6659154	0	-0.084775757	0	0
cov5_9_10	16.7235	-360.114	0.6646909	0	0	0	0
cov3_4_10_11	16.7237	-356.716	0.6480368	0	0	0	0
cov4_5_7_10	16.7279	-356.719	0.6471064	0	0	0	0
cov3_4_5_6	16.7951	-356.752	0.639485	0	0	0	0
cov4_5_8_10	16.8175	-356.763	0.6474019	0	0	0	0
cov1_2_3_10	16.8177	-356.763	0.6366262	0	-0.092938456	0	0.17777168
cov3_4_7_10	16.8194	-356.764	0.6453096	0	0	0	0

cov2_3_9_11	16.827	-356.768	0.6409357	0	0	0	0.118155
cov3_4_8_10	16.8325	-356.771	0.6453597	0	0	0	0
cov2_3_9_10	16.8359	-356.773	0.6383193	0	0	0	0.096031353
cov2_3_4_10	16.8377	-356.773	0.6450152	0	0	0	0.0193469
cov5_6_9	16.8469	-360.175	0.6667988	0	0	0	0
cov5_10_11	16.8478	-360.176	0.6708813	0	0	0	0
cov2_3_8_9	16.9182	-356.814	0.6396364	0	0	0	0.0975839
cov1_4_7_9	16.919	-356.814	0.6464092	0	-0.16439888	0	0
cov2_3_6_10	16.9208	-356.815	0.6377382	0	0	0	0.15571751
cov2_3_7_9	16.9208	-356.815	0.6395491	0	0	0	0.097363384
cov1_9_11	16.9693	-360.237	0.6688929	0	0.082606816	0	0
cov6_9_10	16.9709	-360.237	0.6632901	0	0	0	0
cov1_9_10	16.9833	-360.244	0.6635973	0	0.036023386	0	0
cov6_9_11	16.993	-360.248	0.6677557	0	0	0	0
cov1_4_8_9	17.0212	-356.865	0.6467407	0	-0.16484957	0	0
cov1_5_10	17.0615	-360.283	0.6672011	0	-0.070396501	0	0
cov2_3_10_11	17.101	-356.905	0.6421341	0	0	0	0.12199069
cov2_5_11	17.1238	-360.314	0.6629355	0	0	0	0.19045403
cov1_2_11	17.1329	-360.318	0.6629955	0	-0.020775663	0	0.19288884
cov2_6_11	17.1446	-360.324	0.6645419	0	0	0	0.17555802
cov9_10_11	17.1536	-360.329	0.6614113	0	0	0	0
cov5_6_10	17.1539	-360.329	0.6678242	0	0	0	0
cov2_3_4_5	17.1608	-356.935	0.6351586	0	0	0	0.16580706
cov1_6_9	17.1795	-360.342	0.665885	0	0.015127275	0	0
cov2_3_8_10	17.1835	-356.946	0.6409187	0	0	0	0.10240417
cov2_3_7_10	17.185	-356.947	0.6408337	0	0	0	0.10217122
cov4_6_7_9	17.2421	-356.976	0.6490324	0	0	0	0
cov1_10_11	17.2468	-360.375	0.6698507	0	0.08841336	0	0
cov1_4_7_10	17.2526	-356.981	0.6479994	0	-0.15635136	0	0
cov6_10_11	17.2756	-360.39	0.6686524	0	0	0	0
cov4_6_8_9	17.3299	-357.02	0.6492965	0	0	0	0
cov1_4_8_10	17.355	-357.032	0.6483293	0	-0.15679685	0	0
cov1_2_6	17.4404	-360.472	0.6590195	0	-0.093345738	0	0.18660172
cov1_2_5	17.4449	-360.474	0.6588188	0	-0.055671676	0	0.18929867
cov1_6_10	17.4527	-360.478	0.6668548	0	0.019120451	0	0
cov2_5_6	17.5151	-360.509	0.6598017	0	0	0	0.16950438
cov4_6_7_10	17.5489	-357.129	0.6503551	0	0	0	0
cov4_6_8_10	17.6376	-357.173	0.6506274	0	0	0	0
cov1_3_5_9	17.7653	-357.237	0.6374451	0	0.11296235	0	0
cov1_3_9_10	17.7717	-357.24	0.6378739	0	0.046827216	0	0
cov3_6_9_10	17.8119	-357.261	0.6375177	0	0	0	0
cov3_4_5_11	17.8479	-357.279	0.6360347	0	0	0	0
cov1_3_9_11	17.8673	-357.288	0.6385851	0	0.042148696	0	0
cov1_3_7_9	17.8711	-357.29	0.63914	0	0.048304468	0	0
cov1_3_6_9	17.8712	-357.29	0.63925	0	0.0445903	0	0
cov1_3_8_9	17.8714	-357.29	0.6392219	0	0.048543785	0	0
cov3_9_10_11	17.8727	-357.291	0.6336377	0	0	0	0

cov4_7_9_11	17.8925	-357.301	0.6563118	0	0	0	0
cov3_6_9_11	17.8995	-357.304	0.6377772	0	0	0	0
cov3_5_6_9	17.9042	-357.307	0.6383197	0	0	0	0
cov3_6_7_9	17.9157	-357.312	0.6387905	0	0	0	0
cov3_6_8_9	17.9163	-357.313	0.6388713	0	0	0	0
cov3_5_9_10	17.9357	-357.322	0.636986	0	0	0	0
cov3_7_9_11	17.9652	-357.337	0.6346814	0	0	0	0
cov3_5_9_11	17.9671	-357.338	0.635819	0	0	0	0
cov3_8_9_11	17.9709	-357.34	0.6347462	0	0	0	0
cov4_8_9_11	17.9862	-357.348	0.6567046	0	0	0	0
cov3_7_8_9	18.01	-357.36	0.6350418	0	0	0	0
cov3_4_5_7	18.0196	-357.364	0.6426186	0	0	0	0
cov3_5_7_9	18.0234	-357.366	0.6383863	0	0	0	0
cov3_5_8_9	18.0318	-357.37	0.6383782	0	0	0	0
cov2_4_5_9	18.0442	-357.377	0.658675	0	0	0	-0.075434382
cov3_7_9_10	18.049	-357.379	0.6336344	0	0	0	0
cov1_4_5_7	18.0503	-357.38	0.6457318	0	0.10130254	0	0
cov3_8_9_10	18.0542	-357.382	0.633715	0	0	0	0
cov1_3_5_10	18.0618	-357.385	0.6387364	0	0.12657239	0	0
cov3_4_5_8	18.0648	-357.387	0.6427243	0	0	0	0
cov2_4_7_9	18.0846	-357.397	0.6538857	0	0	0	-0.084041451
cov4_5_9_10	18.1117	-357.41	0.6543772	0	0	0	0
cov4_5_9_11	18.1207	-357.415	0.6594801	0	0	0	0
cov1_2_3_5	18.1312	-357.42	0.632506	0	0.13298277	0	0.21089966
cov1_4_5_8	18.1356	-357.422	0.6459896	0	0.10162193	0	0
cov4_7_10_11	18.1416	-357.425	0.6570613	0	0	0	0
cov2_4_8_9	18.189	-357.449	0.6542355	0	0	0	-0.084841729
cov1_3_10_11	18.1903	-357.45	0.640188	0	0.047826261	0	0
cov1_3_7_10	18.1937	-357.451	0.6407292	0	0.054224004	0	0
cov4_5_6_7	18.194	-357.452	0.6453445	0	0	0	0
cov1_3_6_10	18.1943	-357.452	0.6408551	0	0.049027947	0	0
cov1_3_8_10	18.1951	-357.452	0.6408279	0	0.054475365	0	0
cov3_6_10_11	18.2296	-357.469	0.6393448	0	0	0	0
cov3_5_6_10	18.2329	-357.471	0.6398654	0	0	0	0
cov4_8_10_11	18.236	-357.473	0.6574527	0	0	0	0
cov3_6_7_10	18.2472	-357.478	0.6403945	0	0	0	0
cov4_5_6_9	18.2488	-357.479	0.6571076	0	0	0	0
cov3_6_8_10	18.2491	-357.479	0.6404921	0	0	0	0
cov1_4_5_9	18.2523	-357.481	0.6566797	0	-0.038295198	0	0
cov4_7_9_10	18.2536	-357.481	0.6507318	0	0	0	0
cov4_7_8_9	18.2675	-357.488	0.6528259	0	0	0	0
cov2_3_5_6	18.2741	-357.492	0.6335438	0	0	0	0.23137207
cov4_5_6_8	18.2881	-357.499	0.6456211	0	0	0	0
cov2_4_5_7	18.3148	-357.512	0.6438085	0	0	0	0.051402571
cov3_7_10_11	18.3151	-357.512	0.6358129	0	0	0	0
cov3_5_10_11	18.3187	-357.514	0.6371849	0	0	0	0
cov3_8_10_11	18.3226	-357.516	0.6358829	0	0	0	0

cov2_4_7_10	18.3359	-357.523	0.6547803	0	0	0	-0.077041073
cov2_4_5_10	18.347	-357.528	0.6596538	0	0	0	-0.06792108
cov4_5_7_8	18.347	-357.528	0.6455119	0	0	0	0
cov4_5_7_11	18.3617	-357.535	0.6428935	0	0	0	0
cov4_8_9_10	18.3639	-357.537	0.6510667	0	0	0	0
cov3_5_7_10	18.3842	-357.547	0.6400056	0	0	0	0
cov3_5_8_10	18.3948	-357.552	0.6400198	0	0	0	0
cov4_5_10_11	18.4025	-357.556	0.6602617	0	0	0	0
cov2_4_5_8	18.4055	-357.557	0.6440813	0	0	0	0.050910843
cov3_7_8_10	18.4174	-357.563	0.6362434	0	0	0	0
cov2_4_8_10	18.4406	-357.575	0.6551277	0	0	0	-0.077838854
cov4_5_8_11	18.4549	-357.582	0.6432174	0	0	0	0
cov2_3_5_11	18.4609	-357.585	0.6310698	0	0	0	0.25719495
cov4_7_8_10	18.4711	-357.59	0.6536455	0	0	0	0
cov2_3_5_7	18.5088	-357.609	0.6333766	0	0	0	0.26007187
cov2_3_5_8	18.5129	-357.611	0.6333687	0	0	0	0.26232631
cov4_5_6_10	18.5249	-357.617	0.6580773	0	0	0	0
cov1_4_5_10	18.531	-357.62	0.6578084	0	-0.023903594	0	0
cov1_6_11	18.6741	-361.089	0.6698006	0	0.079161466	0	0
cov1_5_11	18.7725	-361.138	0.6695167	0	0.14548078	0	0
cov5_6_11	18.7896	-361.147	0.6688023	0	0	0	0
cov1_5_6	18.9576	-361.231	0.6662076	0	0.049785277	0	0
cov1_4_9_11	19.0655	-357.887	0.66253	0	-0.14530577	0	0
cov1_4_9_10	19.1102	-357.91	0.6576325	0	-0.18423374	0	0
cov1_4_6_9	19.2119	-357.961	0.6597018	0	-0.16062604	0	0
cov1_2_4_9	19.2276	-357.968	0.6596457	0	-0.18094206	0	-0.007303271
cov4_6_9_11	19.3594	-358.034	0.6665063	0	0	0	0
cov1_2_3_7	19.3782	-358.044	0.6408237	0	-0.057423327	0	0.19704132
cov2_3_7_8	19.3844	-358.047	0.6434712	0	0	0	0.14893057
cov1_2_3_8	19.4166	-358.063	0.6410005	0	-0.056933287	0	0.19728999
cov2_3_4_7	19.4208	-358.065	0.6456797	0	0	0	0.1048485
cov1_4_10_11	19.4284	-358.069	0.6641373	0	-0.13736673	0	0
cov1_2_7_9	19.4415	-358.075	0.6428857	0	-0.10431676	0	0.17070083
cov2_3_6_7	19.4634	-358.086	0.6419614	0	0	0	0.17459848
cov2_3_4_8	19.4651	-358.087	0.6457192	0	0	0	0.10736995
cov2_3_7_11	19.4722	-358.091	0.6445125	0	0	0	0.16439756
cov2_3_6_8	19.4994	-358.104	0.642103	0	0	0	0.17522268
cov2_3_8_11	19.5059	-358.108	0.6446864	0	0	0	0.16540589
cov1_2_8_9	19.5685	-358.139	0.6432696	0	-0.10411234	0	0.1706753
cov1_4_6_10	19.5749	-358.142	0.6613521	0	-0.15441533	0	0
cov1_2_3_4	19.5814	-358.145	0.6445574	0	-0.060573118	0	0.17698157
cov1_2_4_10	19.5891	-358.149	0.6612072	0	-0.17449851	0	-0.0036174
cov2_6_7_9	19.5978	-358.153	0.6449634	0	0	0	0.1439215
cov4_6_9_10	19.605	-358.157	0.66151	0	0	0	0
cov1_2_3_11	19.6218	-358.165	0.6441296	0	-0.041294061	0	0.20092962
cov1_2_3_6	19.6273	-358.168	0.6431276	0	-0.069247116	0	0.19879412
cov2_3_4_11	19.6387	-358.174	0.6482324	0	0	0	0.15228534

cov2_4_6_9	19.6512	-358.18	0.6640296	0	0	0	-0.04369494
cov2_3_6_11	19.6698	-358.189	0.6457463	0	0	0	0.18281682
cov1_2_7_10	19.6733	-358.191	0.643847	0	-0.099958075	0	0.17218592
cov2_3_4_6	19.6769	-358.193	0.6453868	0	0	0	0.15756198
cov4_6_10_11	19.6972	-358.203	0.6677915	0	0	0	0
cov4_9_10_11	19.6993	-358.204	0.6685365	0	0	0	0
cov2_4_9_11	19.7003	-358.205	0.6704445	0	0	0	-0.059877409
cov2_7_9_10	19.7094	-358.209	0.6460899	0	0	0	0.084454323
cov2_6_8_9	19.7131	-358.211	0.6452479	0	0	0	0.14494621
cov1_4_5_11	19.7221	-358.216	0.6582248	0	0.15220973	0	0
cov1_2_4_5	19.7251	-358.217	0.6577763	0	0.17981816	0	-0.071386715
cov3_4_7_8	19.7297	-358.219	0.6493871	0	0	0	0
cov1_2_4_7	19.7315	-358.22	0.6466758	0	-0.10262882	0	0.10003775
cov2_7_9_11	19.7517	-358.23	0.6498563	0	0	0	0.11353334
cov2_7_8_9	19.7853	-358.247	0.6484873	0	0	0	0.086496122
cov1_2_8_10	19.8001	-358.255	0.6442226	0	-0.099750175	0	0.17216327
cov2_6_7_10	19.8215	-358.265	0.6458098	0	0	0	0.14603026
cov2_8_9_10	19.8341	-358.272	0.6464445	0	0	0	0.084589373
cov1_2_4_8	19.8401	-358.275	0.6469877	0	-0.10265904	0	0.099412106
cov1_3_4_7	19.8446	-358.277	0.6491938	0	-0.011946897	0	0
cov3_4_7_11	19.8501	-358.28	0.6496755	0	0	0	0
cov3_4_6_7	19.8523	-358.281	0.6493125	0	0	0	0
cov1_4_5_6	19.8581	-358.284	0.6554711	0	0.1050896	0	0
cov2_8_9_11	19.8685	-358.289	0.6502532	0	0	0	0.11438499
cov2_5_7_9	19.9025	-358.306	0.6479731	0	0	0	0.092241022
cov1_3_4_8	19.9129	-358.311	0.6493502	0	-0.00986364	0	0
cov3_4_8_11	19.916	-358.313	0.6497967	0	0	0	0
cov3_4_6_8	19.9181	-358.314	0.6494504	0	0	0	0
cov2_4_6_7	19.9344	-358.322	0.6486872	0	0	0	0.072195559
cov2_6_8_10	19.9372	-358.323	0.6460917	0	0	0	0.14706058
cov2_7_10_11	19.9588	-358.334	0.650432	0	0	0	0.11727961
cov1_4_7_8	19.9605	-358.335	0.6505693	0	-0.044792272	0	0
cov4_5_6_11	19.9753	-358.342	0.6566743	0	0	0	0
cov2_7_8_10	19.9815	-358.345	0.649108	0	0	0	0.091175611
cov2_4_6_10	19.9905	-358.35	0.6653401	0	0	0	-0.039174623
cov1_3_5_6	20.0014	-358.355	0.6399869	0	0.24951508	0	0
cov2_4_10_11	20.0106	-358.36	0.6714253	0	0	0	-0.05371427
cov2_5_8_9	20.0276	-358.368	0.6483328	0	0	0	0.092376764
cov4_6_7_8	20.0303	-358.37	0.6511358	0	0	0	0
cov2_4_5_6	20.0304	-358.37	0.6554061	0	0	0	-0.016088046
cov2_4_7_11	20.0307	-358.37	0.6531528	0	0	0	0.050908162
cov1_3_5_8	20.0336	-358.371	0.6391851	0	0.32579051	0	0
cov2_4_6_8	20.0342	-358.372	0.6489273	0	0	0	0.072410703
cov4_7_8_11	20.0403	-358.375	0.6533144	0	0	0	0
cov1_3_5_7	20.0487	-358.379	0.6392494	0	0.32002076	0	0
cov1_3_5_11	20.0655	-358.387	0.6387863	0	0.29391154	0	0
cov1_4_6_7	20.0661	-358.388	0.6504274	0	-0.058497546	0	0

cov2_4_7_8	20.0678	-358.388	0.6514731	0	0	0	0.026418896
cov1_4_7_11	20.071	-358.39	0.6506233	0	-0.043238782	0	0
cov2_8_10_11	20.0758	-358.393	0.6508225	0	0	0	0.11813491
cov2_5_7_10	20.0962	-358.403	0.6484853	0	0	0	0.098251086
cov1_5_7_9	20.121	-358.415	0.6487311	0	-0.10229977	0	0
cov4_6_7_11	20.1238	-358.416	0.6519366	0	0	0	0
cov2_4_8_11	20.1318	-358.42	0.6535046	0	0	0	0.050912767
cov1_4_6_8	20.1722	-358.441	0.6507152	0	-0.05625425	0	0
cov1_4_8_11	20.1747	-358.442	0.6510174	0	-0.04249447	0	0
cov5_7_9_10	20.1977	-358.453	0.6480633	0	0	0	0
cov2_5_8_10	20.2213	-358.465	0.6488401	0	0	0	0.098391142
cov4_6_8_11	20.2234	-358.466	0.652243	0	0	0	0
cov1_5_8_9	20.25	-358.48	0.6491157	0	-0.10179969	0	0
cov5_6_7_9	20.2559	-358.483	0.6496881	0	0	0	0
cov5_7_8_9	20.262	-358.486	0.6504679	0	0	0	0
cov5_8_9_10	20.3239	-358.517	0.6484147	0	0	0	0
cov5_7_9_11	20.3346	-358.522	0.6518862	0	0	0	0
cov2_4_5_11	20.3399	-358.525	0.6548847	0	0	0	0.044513253
cov5_6_8_9	20.3752	-358.542	0.6500166	0	0	0	0
cov2_4_9_10	20.4298	-358.569	0.6664375	0	0	0	-0.11255691
cov1_5_7_10	20.446	-358.578	0.6500971	0	-0.0882734	0	0
cov5_8_9_11	20.4564	-358.583	0.6523165	0	0	0	0
cov3_4_6_11	20.4977	-358.603	0.6535648	0	0	0	0
cov1_7_9_10	20.502	-358.606	0.646382	0	0.034936733	0	0
cov6_7_9_10	20.5113	-358.61	0.6460654	0	0	0	0
cov1_3_4_6	20.5207	-358.615	0.6521746	0	-0.004381822	0	0
cov5_7_8_10	20.5213	-358.615	0.651363	0	0	0	0
cov1_3_4_11	20.5283	-358.619	0.6534183	0	0.03709382	0	0
cov7_8_9_10	20.5396	-358.624	0.6437211	0	0	0	0
cov5_6_7_10	20.5512	-358.63	0.6507969	0	0	0	0
cov1_5_8_10	20.5747	-358.642	0.6504743	0	-0.087767945	0	0
cov7_9_10_11	20.5758	-358.643	0.6434271	0	0	0	0
cov1_7_8_9	20.5977	-358.653	0.6489564	0	0.037379508	0	0
cov6_7_8_9	20.6039	-358.657	0.6486846	0	0	0	0
cov5_7_10_11	20.6067	-358.658	0.6525739	0	0	0	0
cov1_8_9_10	20.628	-358.669	0.6467651	0	0.035119333	0	0
cov6_8_9_10	20.6426	-358.676	0.6464215	0	0	0	0
cov5_6_8_10	20.6717	-358.69	0.6511286	0	0	0	0
cov7_8_9_11	20.6766	-358.693	0.6458646	0	0	0	0
cov1_2_7_8	20.6923	-358.701	0.6437599	0	-0.072987289	0	0.18764433
cov8_9_10_11	20.7078	-358.708	0.6438128	0	0	0	0
cov1_6_7_9	20.7163	-358.713	0.6488486	0	0.026731484	0	0
cov1_7_9_11	20.7192	-358.714	0.6487982	0	0.036694891	0	0
cov5_8_10_11	20.7293	-358.719	0.6530115	0	0	0	0
cov6_7_9_11	20.7308	-358.72	0.6482068	0	0	0	0
cov1_2_7_11	20.8001	-358.755	0.6443038	0	-0.066608266	0	0.18788322
cov1_2_6_7	20.802	-358.756	0.6436716	0	-0.083123801	0	0.18653111

cov1_2_5_7	20.8051	-358.757	0.643638	0	-0.072970527	0	0.18742316
cov2_6_7_8	20.8208	-358.765	0.6454627	0	0	0	0.16088285
cov2_5_7_8	20.8232	-358.766	0.6452911	0	0	0	0.15399965
cov1_3_4_5_9	20.8264	-355.37	0.6349733	0	0.12787477	0	0
cov2_7_8_11	20.8286	-358.769	0.6487978	0	0	0	0.14794712
cov1_6_8_9	20.8441	-358.777	0.6492337	0	0.029895753	0	0
cov1_8_9_11	20.8456	-358.777	0.6493021	0	0.038161807	0	0
cov1_7_8_10	20.8539	-358.782	0.6499658	0	0.043117957	0	0
cov6_7_8_10	20.863	-358.786	0.6496494	0	0	0	0
cov6_8_9_11	20.8633	-358.786	0.6486138	0	0	0	0
cov3_5_6_7	20.8678	-358.788	0.6441571	0	0	0	0
cov3_5_6_11	20.8818	-358.795	0.6421859	0	0	0	0
cov2_5_7_11	20.8911	-358.8	0.6466094	0	0	0	0.16314954
cov3_5_6_8	20.8941	-358.802	0.6442522	0	0	0	0
cov2_6_7_11	20.8953	-358.802	0.6466495	0	0	0	0.16615716
cov2_5_6_7	20.8975	-358.803	0.6445355	0	0	0	0.16820816
cov1_2_8_11	20.9217	-358.815	0.6447503	0	-0.065281738	0	0.18792126
cov1_2_6_8	20.927	-358.818	0.643994	0	-0.080108909	0	0.18673351
cov1_2_5_8	20.9286	-358.819	0.6439652	0	-0.072730962	0	0.18737777
cov1_3_7_8	20.9475	-358.828	0.646754	0	0.10940445	0	0
cov7_8_10_11	20.9585	-358.834	0.6464731	0	0	0	0
cov1_6_7_10	20.9705	-358.84	0.6498597	0	0.030605918	0	0
cov1_7_10_11	20.9746	-358.842	0.6497808	0	0.042146646	0	0
cov3_6_7_8	20.9877	-358.848	0.6468797	0	0	0	0
cov6_7_10_11	20.9895	-358.849	0.6491335	0	0	0	0
cov2_5_8_11	21.0089	-358.859	0.6470029	0	0	0	0.16370123
cov2_6_8_11	21.0093	-358.859	0.6469484	0	0	0	0.16734176
cov2_5_6_8	21.0158	-358.862	0.6448135	0	0	0	0.16904606
cov1_3_6_7	21.037	-358.873	0.6469591	0	0.068779281	0	0
cov3_4_5_9_10	21.0661	-355.49	0.6347972	0	0	0	0
cov3_4_5_9_11	21.072	-355.493	0.6331808	0	0	0	0
cov3_4_5_6_9	21.0738	-355.494	0.6358628	0	0	0	0
cov1_3_4_5_10	21.0799	-355.497	0.6361936	0	0.14136039	0	0
cov1_3_7_11	21.0802	-358.895	0.646438	0	0.10680396	0	0
cov1_3_6_8	21.0811	-358.895	0.647123	0	0.070350536	0	0
cov2_3_4_5_9	21.0974	-355.506	0.6347055	0	0	0	0.045798003
cov1_6_8_10	21.0985	-358.904	0.6502382	0	0.03377689	0	0
cov1_8_10_11	21.1008	-358.905	0.650277	0	0.043610692	0	0
cov6_8_10_11	21.1227	-358.916	0.6495386	0	0	0	0
cov1_3_8_11	21.1229	-358.916	0.6466677	0	0.10827305	0	0
cov3_6_7_11	21.1338	-358.921	0.6457689	0	0	0	0
cov3_4_5_8_9	21.1501	-355.532	0.636526	0	0	0	0
cov3_4_5_7_9	21.1608	-355.538	0.6365075	0	0	0	0
cov3_6_8_11	21.1843	-358.947	0.6459912	0	0	0	0
cov1_3_6_11	21.3325	-359.021	0.6500957	0	0.080595936	0	0
cov3_4_5_6_10	21.3726	-355.644	0.6373663	0	0	0	0
cov3_4_5_10_11	21.3862	-355.65	0.63458	0	0	0	0

cov2_3_4_5_10	21.4048	-355.66	0.6360674	0	0	0	0.052804972
cov3_4_5_8_10	21.49	-355.702	0.638248	0	0	0	0
cov3_4_5_7_10	21.4986	-355.707	0.6382154	0	0	0	0
cov3_7_8_11	21.6653	-359.187	0.6377434	0	0	0	0
cov3_5_7_11	21.7635	-359.236	0.6398479	0	0	0	0
cov3_5_8_11	21.8293	-359.269	0.6398812	0	0	0	0
cov3_5_7_8	21.9971	-359.353	0.6460981	0	0	0	0
cov1_6_7_8	22.2047	-359.457	0.6506053	0	0.047323794	0	0
cov1_3_4_5_8	22.2295	-356.072	0.6346523	0	0.36308191	0	0
cov5_6_7_8	22.2331	-359.471	0.6505934	0	0	0	0
cov1_5_7_8	22.2447	-359.477	0.6505555	0	0.0809217	0	0
cov1_7_8_11	22.2463	-359.478	0.6506249	0	0.08852691	0	0
cov6_7_8_11	22.2541	-359.482	0.6499698	0	0	0	0
cov1_3_4_5_7	22.2722	-356.093	0.6347101	0	0.35798725	0	0
cov2_9_10_11	22.3043	-359.507	0.6637162	0	0	0	0.14102988
cov1_5_6_7	22.3217	-359.515	0.6505381	0	0.045639056	0	0
cov1_6_7_11	22.3218	-359.515	0.6508052	0	0.053545539	0	0
cov1_5_7_11	22.3553	-359.532	0.6505524	0	0.081799419	0	0
cov5_6_7_11	22.356	-359.533	0.6502875	0	0	0	0
cov1_2_5_9	22.3621	-359.536	0.6600849	0	-0.20215148	0	0.16288494
cov1_2_4_11	22.3754	-359.542	0.6642655	0	-0.062123053	0	0.088734592
cov1_2_9_11	22.419	-359.564	0.6629095	0	-0.056797337	0	0.17523538
cov5_7_8_11	22.4447	-359.577	0.6480664	0	0	0	0
cov1_6_8_11	22.4489	-359.579	0.6512355	0	0.057528943	0	0
cov1_5_6_8	22.4497	-359.579	0.6508654	0	0.04884654	0	0
cov1_5_8_11	22.4773	-359.593	0.6509848	0	0.083202693	0	0
cov2_4_6_11	22.4787	-359.594	0.6666721	0	0	0	0.067302175
cov2_5_9_11	22.4829	-359.596	0.6676978	0	0	0	0.12512103
cov5_6_8_11	22.4893	-359.599	0.6507059	0	0	0	0
cov2_6_9_11	22.4918	-359.601	0.6648936	0	0	0	0.15622588
cov1_2_9_10	22.4933	-359.601	0.6568922	0	-0.1058536	0	0.17125801
cov1_3_4_5_11	22.5104	-356.212	0.6344396	0	0.27977137	0	0
cov1_3_4_5_6	22.5135	-356.214	0.6356094	0	0.26847932	0	0
cov1_2_3_4_5	22.5221	-356.218	0.6352506	0	0.28090845	0	0.009643579
cov1_4_6_11	22.6116	-359.66	0.6669844	0	-0.045805974	0	0
cov1_2_4_6	22.6142	-359.662	0.6605028	0	-0.12165167	0	0.081229936
cov1_2_3_4_9	22.6231	-356.269	0.6390828	0	-0.13742025	0	0.08599352
cov1_2_5_10	22.6699	-359.69	0.6612383	0	-0.18919608	0	0.16495372
cov1_2_10_11	22.6721	-359.691	0.6638012	0	-0.05226501	0	0.17672612
cov1_2_6_9	22.7044	-359.707	0.6594191	0	-0.10523289	0	0.17091043
cov2_6_9_10	22.7046	-359.707	0.6595742	0	0	0	0.13917331
cov2_5_10_11	22.7278	-359.718	0.6681843	0	0	0	0.13076476
cov1_3_4_7_9	22.7364	-356.325	0.6414145	0	-0.11253884	0	0
cov2_6_10_11	22.7373	-359.723	0.6656646	0	0	0	0.15832751
cov1_3_4_8_9	22.7621	-356.338	0.6414993	0	-0.11114842	0	0
cov1_3_4_9_10	22.7637	-356.339	0.6407884	0	-0.10334692	0	0
cov2_3_4_6_9	22.8197	-356.367	0.6407239	0	0	0	0.062632158

cov1_3_4_6_9	22.8214	-356.368	0.6420348	0	-0.080333675	0	0
cov1_3_4_9_11	22.8371	-356.376	0.6420674	0	-0.10241663	0	0
cov2_5_6_9	22.8425	-359.776	0.6630044	0	0	0	0.12799704
cov3_4_6_7_9	22.8632	-356.389	0.6422842	0	0	0	0
cov3_4_6_9_10	22.8738	-356.394	0.6415088	0	0	0	0
cov3_4_6_8_9	22.8832	-356.399	0.6423332	0	0	0	0
cov3_4_6_9_11	22.9349	-356.425	0.6435019	0	0	0	0
cov2_5_9_10	22.951	-359.83	0.6612742	0	0	0	0.10008743
cov1_2_6_10	22.9565	-359.833	0.6603303	0	-0.10232298	0	0.17223864
cov1_2_3_4_10	22.9626	-356.439	0.6406732	0	-0.13123506	0	0.089700273
cov2_3_5_9_10	22.9911	-356.453	0.6304837	0	0	0	0.18121591
cov2_3_5_8_9	23.0034	-356.459	0.6319637	0	0	0	0.19416596
cov2_3_5_7_9	23.0285	-356.472	0.6319875	0	0	0	0.19247676
cov1_3_4_7_10	23.0888	-356.502	0.643236	0	-0.10511184	0	0
cov2_5_6_10	23.1005	-359.905	0.6637236	0	0	0	0.13179685
cov2_3_5_6_9	23.1029	-356.509	0.6321443	0	0	0	0.18375387
cov4_5_7_9_10	23.1043	-356.509	0.643658	0	0	0	0
cov1_2_3_5_9	23.1049	-356.51	0.6322013	0	-0.013425304	0	0.18402288
cov2_3_5_9_11	23.1054	-356.51	0.6317005	0	0	0	0.17915751
cov1_3_4_8_10	23.1153	-356.515	0.643325	0	-0.10371486	0	0
cov3_4_9_10_11	23.132	-356.523	0.6455717	0	0	0	0
cov5_9_10_11	23.1385	-359.924	0.6678444	0	0	0	0
cov2_3_4_6_10	23.1444	-356.529	0.6422857	0	0	0	0.067167
cov2_4_5_7_9	23.1503	-356.532	0.64761	0	0	0	-0.055856265
cov4_5_7_8_9	23.1527	-356.534	0.6462634	0	0	0	0
cov2_3_4_9_11	23.1759	-356.545	0.6462718	0	0	0	0.031153291
cov3_4_7_8_9	23.1799	-356.547	0.6438882	0	0	0	0
cov1_3_4_6_10	23.182	-356.548	0.6439026	0	-0.074248987	0	0
cov3_4_7_9_11	23.1862	-356.55	0.6465244	0	0	0	0
cov4_5_8_9_10	23.1935	-356.554	0.643954	0	0	0	0
cov1_3_4_10_11	23.1956	-356.555	0.6438866	0	-0.095144052	0	0
cov3_4_8_9_11	23.197	-356.556	0.6465748	0	0	0	0
cov4_5_6_7_9	23.1982	-356.556	0.6461295	0	0	0	0
cov1_4_5_7_9	23.2	-356.557	0.6455897	0	-0.057860346	0	0
cov3_4_6_7_10	23.2	-356.557	0.643977	0	0	0	0
cov1_2_3_9_10	23.2139	-356.564	0.6335847	0	-0.099267654	0	0.17640037
cov3_4_6_8_10	23.221	-356.568	0.6440317	0	0	0	0
cov2_4_5_8_9	23.2368	-356.576	0.647925	0	0	0	-0.056480288
cov3_4_7_9_10	23.2593	-356.587	0.642634	0	0	0	0
cov4_5_7_9_11	23.2653	-356.59	0.6471665	0	0	0	0
cov3_4_8_9_10	23.2721	-356.593	0.6426929	0	0	0	0
cov2_3_5_8_10	23.2725	-356.594	0.6332411	0	0	0	0.19887877
cov3_4_6_10_11	23.2801	-356.597	0.6451349	0	0	0	0
cov2_3_4_9_10	23.2838	-356.599	0.6425347	0	0	0	0.013362967
cov4_5_6_8_9	23.2838	-356.599	0.6464164	0	0	0	0
cov1_5_9_11	23.2857	-359.997	0.6691359	0	-0.040693165	0	0
cov1_4_5_8_9	23.2913	-356.603	0.6459009	0	-0.057212129	0	0

cov2_3_5_7_10	23.2964	-356.605	0.6332555	0	0	0	0.19718575
cov1_2_3_6_9	23.3034	-356.609	0.6348852	0	-0.081860871	0	0.17771682
cov2_3_4_5_6	23.3082	-356.611	0.6355561	0	0	0	0.092854469
cov1_2_3_8_9	23.3097	-356.612	0.6350796	0	-0.097584586	0	0.17656826
cov1_2_3_9_11	23.3108	-356.613	0.6348115	0	-0.099343669	0	0.17634847
cov1_2_3_7_9	23.3111	-356.613	0.6349745	0	-0.097724063	0	0.17644836
cov5_6_9_11	23.3195	-360.014	0.6699762	0	0	0	0
cov2_3_6_9_10	23.3319	-356.623	0.6348874	0	0	0	0.15281553
cov2_3_4_7_9	23.3327	-356.624	0.6437081	0	0	0	0.00668204
cov2_3_4_8_9	23.3433	-356.629	0.6437095	0	0	0	0.008497065
cov4_5_8_9_11	23.3523	-356.633	0.6475362	0	0	0	0
cov1_5_9_10	23.3537	-360.031	0.6638478	0	-0.080791064	0	0
cov2_3_5_10_11	23.3628	-356.639	0.6327152	0	0	0	0.18406939
cov2_3_5_6_10	23.3665	-356.641	0.6333803	0	0	0	0.18730561
cov1_2_3_5_10	23.3676	-356.641	0.6333948	0	-0.001315273	0	0.18603896
cov4_5_7_8_10	23.3938	-356.654	0.647226	0	0	0	0
cov2_3_6_9_11	23.4178	-356.666	0.636764	0	0	0	0.15517285
cov2_3_6_8_9	23.4211	-356.668	0.6362772	0	0	0	0.15364403
cov2_3_6_7_9	23.4225	-356.669	0.6361905	0	0	0	0.15354132
cov2_4_5_7_10	23.4239	-356.669	0.6484916	0	0	0	-0.048309048
cov3_4_7_8_10	23.4607	-356.688	0.645335	0	0	0	0
cov4_5_6_7_10	23.4677	-356.691	0.6471941	0	0	0	0
cov5_6_9_10	23.4694	-360.089	0.6646242	0	0	0	0
cov2_3_4_10_11	23.471	-356.693	0.6474594	0	0	0	0.036993302
cov1_4_5_7_10	23.4754	-356.695	0.6468325	0	-0.043751046	0	0
cov3_4_7_10_11	23.4883	-356.701	0.6477841	0	0	0	0
cov3_4_5_6_11	23.4927	-356.704	0.6363028	0	0	0	0
cov3_4_8_10_11	23.5008	-356.708	0.6478452	0	0	0	0
cov2_4_5_8_10	23.5109	-356.713	0.648806	0	0	0	-0.04892684
cov4_5_7_10_11	23.5131	-356.714	0.6478849	0	0	0	0
cov1_5_6_9	23.5147	-360.112	0.665862	0	-0.093817356	0	0
cov2_3_9_10_11	23.5271	-356.721	0.6397265	0	0	0	0.11788331
cov1_9_10_11	23.5414	-360.125	0.6665491	0	0.081526805	0	0
cov4_5_6_8_10	23.5542	-356.734	0.6474811	0	0	0	0
cov2_3_7_8_9	23.5547	-356.735	0.6395673	0	0	0	0.097107014
cov6_9_10_11	23.5586	-360.134	0.6653984	0	0	0	0
cov1_4_5_8_10	23.5664	-356.74	0.6471269	0	-0.043106551	0	0
cov1_4_7_9_10	23.5832	-356.749	0.6444627	0	-0.1641419	0	0
cov3_4_5_6_8	23.5874	-356.751	0.6395395	0	0	0	0
cov1_4_7_8_9	23.5877	-356.751	0.6465307	0	-0.16450622	0	0
cov3_4_5_6_7	23.5897	-356.752	0.6394783	0	0	0	0
cov4_5_8_10_11	23.6008	-356.758	0.6482506	0	0	0	0
cov1_2_3_6_10	23.6058	-356.76	0.6365024	0	-0.078453298	0	0.1789276
cov2_3_4_7_10	23.6083	-356.761	0.6449838	0	0	0	0.012803242
cov1_2_3_8_10	23.6117	-356.763	0.6366921	0	-0.092849685	0	0.17784085
cov1_2_3_10_11	23.6119	-356.763	0.6364414	0	-0.094788743	0	0.17764335
cov1_2_3_7_10	23.612	-356.763	0.6365799	0	-0.093002967	0	0.17772357

cov2_3_8_9_11	23.6131	-356.764	0.6411965	0	0	0	0.1195033
cov2_3_7_9_11	23.6189	-356.767	0.6410824	0	0	0	0.11892394
cov2_3_4_8_10	23.6197	-356.767	0.6449916	0	0	0	0.01462279
cov1_5_10_11	23.6279	-360.169	0.6703561	0	-0.026425589	0	0
cov2_3_8_9_10	23.6295	-356.772	0.6384034	0	0	0	0.0962163
cov2_3_7_9_10	23.6304	-356.772	0.6382975	0	0	0	0.095978345
cov5_6_10_11	23.6419	-360.176	0.6709346	0	0	0	0
cov1_4_8_9_10	23.6854	-356.8	0.6447948	0	-0.16459219	0	0
cov1_4_6_7_9	23.6866	-356.801	0.6466123	0	-0.13459639	0	0
cov1_2_4_7_9	23.7056	-356.81	0.6459101	0	-0.17182573	0	0.015995678
cov2_3_6_10_11	23.7113	-356.813	0.6382662	0	0	0	0.15723215
cov1_4_7_9_11	23.7136	-356.814	0.6464052	0	-0.16439579	0	0
cov2_3_6_8_10	23.7147	-356.815	0.6378006	0	0	0	0.15578845
cov2_3_6_7_10	23.7152	-356.815	0.6377073	0	0	0	0.15567144
cov1_6_9_11	23.7336	-360.221	0.6688805	0	0.05120408	0	0
cov1_6_9_10	23.762	-360.236	0.6634684	0	0.012152713	0	0
cov2_3_4_5_11	23.7715	-356.843	0.6310271	0	0	0	0.14641532
cov1_4_6_8_9	23.7843	-356.849	0.6469482	0	-0.13266591	0	0
cov1_2_4_8_9	23.8084	-356.862	0.6462664	0	-0.1719447	0	0.015273996
cov1_4_8_9_11	23.8156	-356.865	0.6468662	0	-0.16366646	0	0
cov2_3_7_8_10	23.8194	-356.867	0.6408443	0	0	0	0.1018851
cov1_5_6_10	23.8516	-360.28	0.6671264	0	-0.081875864	0	0
cov2_3_8_10_11	23.8898	-356.902	0.6423937	0	0	0	0.12311222
cov1_2_6_11	23.8919	-360.301	0.663084	0	-0.053081109	0	0.18992662
cov2_3_7_10_11	23.8943	-356.904	0.6420236	0	0	0	0.12241857
cov2_5_6_11	23.9016	-360.305	0.6634755	0	0	0	0.1822083
cov4_6_7_9_10	23.9113	-356.913	0.6471376	0	0	0	0
cov1_2_5_11	23.9172	-360.313	0.6627149	0	-0.00762556	0	0.19339568
cov2_3_4_5_7	23.9206	-356.918	0.6352521	0	0	0	0.15384183
cov1_4_7_8_10	23.9222	-356.918	0.648126	0	-0.15645468	0	0
cov2_3_4_5_8	23.934	-356.924	0.6352289	0	0	0	0.15646461
cov4_6_7_8_9	23.9362	-356.925	0.6491757	0	0	0	0
cov4_6_8_9_10	23.9992	-356.957	0.6474024	0	0	0	0
cov1_6_10_11	24.0074	-360.358	0.6698036	0	0.055186648	0	0
cov4_6_7_9_11	24.0098	-356.962	0.6503773	0	0	0	0
cov1_4_6_7_10	24.0236	-356.969	0.6481894	0	-0.12846254	0	0
cov2_4_6_7_9	24.0281	-356.971	0.6493907	0	0	0	-0.015692212
cov1_2_4_7_10	24.0347	-356.975	0.6473622	0	-0.16562179	0	0.019977269
cov1_4_7_10_11	24.0472	-356.981	0.6479492	0	-0.15684209	0	0
cov4_6_8_9_11	24.0957	-357.005	0.6506942	0	0	0	0
cov2_4_6_8_9	24.1159	-357.015	0.649658	0	0	0	-0.015733368
cov1_4_6_8_10	24.1218	-357.018	0.6485229	0	-0.12651683	0	0
cov1_2_4_8_10	24.138	-357.026	0.647716	0	-0.16573509	0	0.019255112
cov1_4_8_10_11	24.1496	-357.032	0.6484013	0	-0.15610374	0	0
cov1_2_5_6	24.222	-360.466	0.6587831	0	-0.078920575	0	0.18713636
cov4_6_7_8_10	24.2426	-357.079	0.6505023	0	0	0	0
cov4_6_7_10_11	24.3202	-357.117	0.6516099	0	0	0	0

cov2_4_6_7_10	24.3393	-357.127	0.6506248	0	0	0	-0.010931578
cov3_4_5_7_11	24.39	-357.152	0.6355776	0	0	0	0
cov4_6_8_10_11	24.407	-357.161	0.651933	0	0	0	0
cov3_4_5_8_11	24.4277	-357.171	0.6356095	0	0	0	0
cov2_4_6_8_10	24.428	-357.171	0.6508929	0	0	0	-0.010970212
cov1_3_5_9_10	24.4466	-357.181	0.6359135	0	0.11528512	0	0
cov1_3_7_8_9	24.5042	-357.209	0.6391804	0	0.048203373	0	0
cov1_3_5_8_9	24.5085	-357.212	0.6372023	0	0.14295158	0	0
cov1_3_5_7_9	24.5275	-357.221	0.6372496	0	0.13701875	0	0
cov3_6_7_8_9	24.5367	-357.226	0.6389136	0	0	0	0
cov4_7_9_10_11	24.5433	-357.229	0.6543401	0	0	0	0
cov1_3_5_9_11	24.5492	-357.232	0.6364633	0	0.10536728	0	0
cov1_3_5_6_9	24.5572	-357.236	0.6375045	0	0.10403704	0	0
cov4_7_8_9_11	24.5617	-357.238	0.6564475	0	0	0	0
cov1_3_9_10_11	24.5634	-357.239	0.6373864	0	0.04174076	0	0
cov1_3_7_9_10	24.5642	-357.239	0.637755	0	0.046539702	0	0
cov1_3_6_9_10	24.5657	-357.24	0.6378993	0	0.042356314	0	0
cov1_3_8_9_10	24.5663	-357.24	0.6378551	0	0.046795842	0	0
cov2_4_7_9_11	24.5777	-357.246	0.6563939	0	0	0	-0.053599878
cov3_5_6_9_10	24.5886	-357.252	0.6368313	0	0	0	0
cov3_6_9_10_11	24.5934	-357.254	0.6365972	0	0	0	0
cov3_7_8_9_11	24.5976	-357.256	0.634733	0	0	0	0
cov3_6_7_9_10	24.6038	-357.259	0.6373928	0	0	0	0
cov3_6_8_9_10	24.6064	-357.26	0.6374982	0	0	0	0
cov4_8_9_10_11	24.6369	-357.276	0.6547312	0	0	0	0
cov3_7_9_10_11	24.6555	-357.285	0.6334354	0	0	0	0
cov3_5_7_8_9	24.6582	-357.286	0.6383934	0	0	0	0
cov1_3_7_9_11	24.6606	-357.288	0.63846	0	0.041380596	0	0
cov1_3_6_9_11	24.6617	-357.288	0.6386165	0	0.039948049	0	0
cov1_3_8_9_11	24.6618	-357.288	0.6386077	0	0.042284936	0	0
cov3_8_9_10_11	24.6628	-357.289	0.6335131	0	0	0	0
cov3_5_9_10_11	24.663	-357.289	0.6344863	0	0	0	0
cov1_3_6_7_9	24.6653	-357.29	0.6391684	0	0.044481581	0	0
cov1_3_6_8_9	24.6655	-357.29	0.6392526	0	0.044655966	0	0
cov3_5_6_9_11	24.6701	-357.292	0.6366318	0	0	0	0
cov2_4_8_9_11	24.6706	-357.293	0.6567852	0	0	0	-0.053792242
cov2_4_5_9_10	24.6784	-357.296	0.6563196	0	0	0	-0.072921875
cov3_7_8_9_10	24.6817	-357.298	0.6336968	0	0	0	0
cov3_4_5_7_8	24.6848	-357.3	0.6426846	0	0	0	0
cov3_6_7_9_11	24.6917	-357.303	0.6376164	0	0	0	0
cov3_6_8_9_11	24.694	-357.304	0.6377587	0	0	0	0
cov3_5_6_8_9	24.6947	-357.305	0.6383376	0	0	0	0
cov3_5_6_7_9	24.6985	-357.307	0.6383238	0	0	0	0
cov3_5_7_9_10	24.7113	-357.313	0.63692	0	0	0	0
cov3_5_8_9_10	24.7212	-357.318	0.6369396	0	0	0	0
cov1_4_5_7_8	24.7235	-357.319	0.6458419	0	0.10178468	0	0
cov4_5_9_10_11	24.7388	-357.327	0.6569893	0	0	0	0

cov3_5_7_9_11	24.7512	-357.333	0.6358721	0	0	0	0
cov2_4_7_9_10	24.7562	-357.335	0.6520096	0	0	0	-0.082370949
cov2_4_7_8_9	24.7565	-357.336	0.6540199	0	0	0	-0.084074292
cov3_5_8_9_11	24.7581	-357.336	0.6358495	0	0	0	0
cov2_4_5_9_11	24.7716	-357.343	0.6599883	0	0	0	-0.061352707
cov1_3_5_8_10	24.8013	-357.358	0.6384701	0	0.15754036	0	0
cov4_7_8_10_11	24.8116	-357.363	0.657201	0	0	0	0
cov1_2_4_5_9	24.8181	-357.366	0.6593303	0	0.036487106	0	-0.09245174
cov1_3_5_7_10	24.821	-357.368	0.6385199	0	0.15165966	0	0
cov1_2_3_5_8	24.823	-357.369	0.6320513	0	0.17295151	0	0.21541628
cov1_3_7_8_10	24.8278	-357.371	0.6407715	0	0.054171432	0	0
cov1_2_4_5_7	24.8285	-357.372	0.646615	0	0.12305728	0	-0.025017284
cov2_4_5_6_9	24.8382	-357.376	0.6587039	0	0	0	-0.077164675
cov1_4_5_6_7	24.8435	-357.379	0.6457025	0	0.094256526	0	0
cov1_3_5_10_11	24.8444	-357.379	0.6376986	0	0.11841132	0	0
cov1_4_5_7_11	24.8444	-357.379	0.6459355	0	0.10343544	0	0
cov1_2_3_5_7	24.8491	-357.382	0.6321099	0	0.16779741	0	0.21481997
cov2_4_7_10_11	24.8505	-357.383	0.6572204	0	0	0	-0.047458845
cov1_3_5_6_10	24.8523	-357.383	0.6388084	0	0.11550611	0	0
cov4_5_6_7_8	24.8543	-357.384	0.6454619	0	0	0	0
cov2_4_8_9_10	24.8608	-357.388	0.6523676	0	0	0	-0.083177508
cov3_6_7_8_10	24.8678	-357.391	0.6405207	0	0	0	0
cov4_5_6_9_10	24.8738	-357.394	0.6546661	0	0	0	0
cov1_4_5_9_10	24.8777	-357.396	0.6542942	0	-0.033995774	0	0
cov1_2_3_5_6	24.9101	-357.412	0.632725	0	0.11079029	0	0.20896125
cov4_5_6_9_11	24.9116	-357.413	0.6593609	0	0	0	0
cov1_2_4_5_8	24.9118	-357.413	0.6469252	0	0.12466388	0	-0.026493956
cov1_4_5_9_11	24.9147	-357.415	0.6593355	0	-0.005269438	0	0
cov1_2_3_5_11	24.924	-357.419	0.6320949	0	0.13006051	0	0.21075462
cov4_7_8_9_10	24.9273	-357.421	0.6508589	0	0	0	0
cov1_4_5_8_11	24.9291	-357.422	0.6462971	0	0.10483042	0	0
cov1_4_5_6_8	24.9295	-357.422	0.6459713	0	0.096761561	0	0
cov2_4_8_10_11	24.9442	-357.429	0.6576096	0	0	0	-0.047647387
cov3_7_8_10_11	24.9484	-357.432	0.6358699	0	0	0	0
cov2_4_5_6_7	24.9802	-357.447	0.6448637	0	0	0	0.01530502
cov4_5_6_7_11	24.9815	-357.448	0.6446108	0	0	0	0
cov1_3_7_10_11	24.9824	-357.449	0.6400049	0	0.046762019	0	0
cov1_3_6_10_11	24.9844	-357.45	0.6402386	0	0.044196927	0	0
cov1_3_8_10_11	24.9848	-357.45	0.6401593	0	0.047669515	0	0
cov1_3_6_7_10	24.9875	-357.451	0.6407742	0	0.048818005	0	0
cov1_3_6_8_10	24.9889	-357.452	0.6408628	0	0.049039487	0	0
cov2_4_5_7_8	24.9905	-357.453	0.6439097	0	0	0	0.051649242
cov3_5_6_10_11	24.9937	-357.454	0.6380386	0	0	0	0
cov2_4_7_8_10	25.0084	-357.462	0.654917	0	0	0	-0.077074722
cov3_6_7_10_11	25.02	-357.467	0.6391258	0	0	0	0
cov3_5_7_8_10	25.0202	-357.467	0.6400226	0	0	0	0
cov3_6_8_10_11	25.0238	-357.469	0.6392739	0	0	0	0

cov3_5_6_8_10	25.0244	-357.469	0.6398886	0	0	0	0
cov3_5_6_7_10	25.0274	-357.471	0.639868	0	0	0	0
cov1_4_5_6_9	25.0372	-357.476	0.6569142	0	-0.020990838	0	0
cov4_5_7_8_11	25.0386	-357.477	0.6430077	0	0	0	0
cov2_3_5_6_8	25.044	-357.479	0.6335575	0	0	0	0.23660328
cov2_3_5_6_7	25.0566	-357.486	0.6335478	0	0	0	0.23511604
cov2_3_5_6_11	25.0588	-357.487	0.6325277	0	0	0	0.23018461
cov2_4_5_6_8	25.0743	-357.494	0.6451429	0	0	0	0.015247094
cov4_5_6_8_11	25.0767	-357.496	0.6449531	0	0	0	0
cov2_4_5_7_11	25.0795	-357.497	0.6426297	0	0	0	0.040652963
cov2_4_5_10_11	25.0831	-357.499	0.6608967	0	0	0	-0.054717779
cov3_5_7_10_11	25.0973	-357.506	0.6372302	0	0	0	0
cov1_2_4_5_10	25.1032	-357.509	0.6604503	0	0.049660168	0	-0.09110394
cov3_5_8_10_11	25.1063	-357.51	0.6372174	0	0	0	0
cov2_4_5_6_10	25.1377	-357.526	0.659689	0	0	0	-0.072621094
cov2_3_5_7_8	25.1531	-357.534	0.633378	0	0	0	0.26094749
cov2_4_5_8_11	25.1728	-357.544	0.6429574	0	0	0	0.040633866
cov1_4_5_10_11	25.1954	-357.555	0.6604129	0	0.00901597	0	0
cov4_5_6_10_11	25.197	-357.556	0.6602733	0	0	0	0
cov2_3_5_7_11	25.2518	-357.583	0.6311155	0	0	0	0.25390661
cov2_3_5_8_11	25.2551	-357.585	0.6310849	0	0	0	0.25610622
cov1_4_5_6_10	25.3183	-357.616	0.6580143	0	-0.009001349	0	0
cov1_5_6_11	25.464	-361.087	0.6696572	0	0.087896485	0	0
cov1_4_9_10_11	25.7338	-357.824	0.6607564	0	-0.14425517	0	0
cov1_2_5_7_9	25.7902	-357.852	0.6436098	0	-0.21729871	0	0.16090659
cov1_4_6_9_11	25.8526	-357.884	0.6626294	0	-0.13055216	0	0
cov1_2_4_9_11	25.8601	-357.887	0.6625615	0	-0.14485267	0	-0.001101043
cov1_4_6_9_10	25.8886	-357.902	0.6579021	0	-0.16137167	0	0
cov1_2_4_9_10	25.9039	-357.909	0.6577852	0	-0.18180266	0	-0.005136172
cov1_2_5_8_9	25.9193	-357.917	0.6439952	0	-0.21682116	0	0.16091632
cov1_2_3_4_7	25.9816	-357.948	0.6431895	0	-0.076335445	0	0.14982399
cov1_2_4_6_9	26.0052	-357.96	0.659895	0	-0.15797219	0	-0.006244835
cov1_2_7_9_10	26.0198	-357.967	0.6402944	0	-0.10661261	0	0.17081573
cov4_6_9_10_11	26.0287	-357.972	0.6647499	0	0	0	0
cov1_2_3_4_8	26.0331	-357.974	0.6432873	0	-0.075143679	0	0.15173058
cov1_2_3_7_8	26.0362	-357.975	0.6408972	0	-0.05737109	0	0.19725973
cov2_3_4_7_8	26.0831	-357.999	0.6456986	0	0	0	0.10602033
cov1_2_5_7_10	26.0843	-357.999	0.6448491	0	-0.20474212	0	0.16301379
cov2_3_4_6_7	26.1094	-358.012	0.6442962	0	0	0	0.12982728
cov1_2_7_8_9	26.1137	-358.014	0.6429905	0	-0.10427609	0	0.17097487
cov2_3_6_7_8	26.1283	-358.021	0.6421189	0	0	0	0.17323177
cov2_4_6_9_11	26.1291	-358.022	0.6668094	0	0	0	-0.026918258
cov2_3_7_8_11	26.13	-358.022	0.6445842	0	0	0	0.16468871
cov2_3_4_7_11	26.1439	-358.029	0.6472621	0	0	0	0.118812
cov1_2_8_9_10	26.1464	-358.03	0.6406738	0	-0.10640994	0	0.17079347
cov2_3_4_6_8	26.1554	-358.035	0.6443503	0	0	0	0.13206898
cov1_2_3_6_7	26.1668	-358.041	0.6409172	0	-0.07138132	0	0.19583667

cov1_2_3_7_11	26.1719	-358.043	0.6411247	0	-0.054660416	0	0.19722798
cov2_3_4_8_11	26.1863	-358.05	0.6473765	0	0	0	0.1215343
cov2_6_7_9_10	26.1866	-358.051	0.6425316	0	0	0	0.14308157
cov1_2_3_6_8	26.2058	-358.06	0.6410926	0	-0.07018253	0	0.19615048
cov1_2_3_8_11	26.2097	-358.062	0.6413834	0	-0.053433439	0	0.19752524
cov1_2_5_8_10	26.2131	-358.064	0.6452496	0	-0.20426673	0	0.16303991
cov1_4_6_10_11	26.2172	-358.066	0.6641969	0	-0.12449632	0	0
cov1_2_4_10_11	26.2227	-358.069	0.6640727	0	-0.138492	0	0.002644213
cov1_2_6_7_9	26.2331	-358.074	0.6428867	0	-0.09458377	0	0.17149369
cov1_2_7_9_11	26.2337	-358.074	0.6433397	0	-0.099817044	0	0.1710435
cov2_3_6_7_11	26.2426	-358.079	0.6430546	0	0	0	0.17758562
cov2_5_6_7_9	26.2679	-358.091	0.6463549	0	0	0	0.1268662
cov2_3_6_8_11	26.2771	-358.096	0.6432508	0	0	0	0.17833624
cov2_6_7_8_9	26.2841	-358.099	0.6451592	0	0	0	0.14282694
cov2_6_8_9_10	26.3014	-358.108	0.6428109	0	0	0	0.14410612
cov2_7_9_10_11	26.3363	-358.125	0.6475631	0	0	0	0.11294497
cov2_4_6_9_10	26.3373	-358.126	0.6623088	0	0	0	-0.042088375
cov1_2_7_8_10	26.3464	-358.13	0.6439414	0	-0.099870902	0	0.17251563
cov1_2_3_4_11	26.3576	-358.136	0.6458698	0	-0.048943852	0	0.17688419
cov1_2_6_8_9	26.3582	-358.136	0.6432647	0	-0.091592038	0	0.17169776
cov1_2_8_9_11	26.3592	-358.137	0.6438578	0	-0.098383673	0	0.17111188
cov2_4_9_10_11	26.3676	-358.141	0.6686604	0	0	0	-0.057554747
cov1_2_3_4_6	26.3681	-358.141	0.6446287	0	-0.076575026	0	0.17581341
cov2_6_7_9_11	26.3686	-358.142	0.6461195	0	0	0	0.14770298
cov1_2_4_6_10	26.3693	-358.142	0.6614263	0	-0.15327789	0	-0.002657965
cov2_5_6_8_9	26.3786	-358.147	0.6466667	0	0	0	0.1275632
cov2_7_8_9_10	26.3839	-358.149	0.6461989	0	0	0	0.084784725
cov1_2_3_6_11	26.4044	-358.159	0.6443695	0	-0.060167053	0	0.19924229
cov1_2_4_5_11	26.407	-358.161	0.6598901	0	0.20401795	0	-0.064053167
cov1_2_4_7_8	26.4143	-358.164	0.6467901	0	-0.10260411	0	0.10025206
cov2_3_4_6_11	26.4199	-358.167	0.6473907	0	0	0	0.16056094
cov2_7_8_9_11	26.4234	-358.169	0.6500067	0	0	0	0.11376577
cov2_8_9_10_11	26.4524	-358.183	0.6479523	0	0	0	0.11379394
cov1_2_7_10_11	26.4657	-358.19	0.644275	0	-0.095643861	0	0.17252099
cov1_2_6_7_10	26.4658	-358.19	0.6438427	0	-0.091821857	0	0.17285276
cov2_4_6_10_11	26.4746	-358.195	0.6680668	0	0	0	-0.022395555
cov2_6_8_9_11	26.4818	-358.198	0.6464589	0	0	0	0.14887288
cov2_5_7_9_10	26.4951	-358.205	0.6453902	0	0	0	0.094068308
cov1_4_5_6_11	26.5041	-358.209	0.6581844	0	0.13188568	0	0
cov2_6_7_8_10	26.508	-358.211	0.6460074	0	0	0	0.14493851
cov1_2_4_5_6	26.51	-358.212	0.6576875	0	0.16224136	0	-0.072873043
cov2_5_6_7_10	26.5152	-358.215	0.6471833	0	0	0	0.13063019
cov1_3_4_7_8	26.5175	-358.216	0.6492724	0	-0.011045414	0	0
cov3_4_7_8_11	26.5222	-358.218	0.6497261	0	0	0	0
cov1_2_4_7_11	26.5238	-358.219	0.6471519	0	-0.098145308	0	0.10047529
cov3_4_6_7_8	26.5242	-358.219	0.649397	0	0	0	0
cov1_2_4_6_7	26.5255	-358.22	0.646677	0	-0.10722961	0	0.099687818

cov2_5_7_9_11	26.5371	-358.226	0.6507297	0	0	0	0.10571749
cov2_5_7_8_9	26.5762	-358.245	0.6480774	0	0	0	0.092536525
cov1_2_6_8_10	26.591	-358.253	0.6442148	0	-0.088824557	0	0.17305594
cov1_2_8_10_11	26.5911	-358.253	0.6447728	0	-0.094217405	0	0.17259312
cov2_6_7_10_11	26.5935	-358.254	0.6469342	0	0	0	0.14972666
cov2_5_8_9_10	26.6198	-358.267	0.6457456	0	0	0	0.094206696
cov2_5_6_8_10	26.6268	-358.271	0.6474785	0	0	0	0.13132682
cov1_3_4_6_7	26.6283	-358.271	0.6491913	0	-0.031151774	0	0
cov2_4_6_7_8	26.6288	-358.272	0.6488534	0	0	0	0.071334749
cov1_2_4_8_11	26.631	-358.273	0.6475764	0	-0.097104256	0	0.099960321
cov2_7_8_10_11	26.6313	-358.273	0.6505823	0	0	0	0.11751363
cov1_2_4_6_8	26.6345	-358.275	0.6469887	0	-0.10471398	0	0.099253576
cov1_3_4_7_11	26.6392	-358.277	0.6491717	0	-0.012159523	0	0
cov3_4_6_7_11	26.6443	-358.279	0.649786	0	0	0	0
cov2_5_8_9_11	26.6531	-358.284	0.6511601	0	0	0	0.10623253
cov1_3_5_7_8	26.6939	-358.304	0.6392844	0	0.31987365	0	0
cov2_4_6_7_11	26.6974	-358.306	0.6501332	0	0	0	0.0764853
cov1_3_4_6_8	26.6979	-358.306	0.6493549	0	-0.027813471	0	0
cov2_6_8_10_11	26.7072	-358.311	0.6472742	0	0	0	0.15090156
cov1_3_4_8_11	26.7074	-358.311	0.6494132	0	-0.00927003	0	0
cov3_4_6_8_11	26.7098	-358.312	0.64996	0	0	0	0
cov2_4_7_8_11	26.7123	-358.313	0.6532707	0	0	0	0.051282621
cov1_4_6_7_8	26.7468	-358.331	0.6505472	0	-0.062011142	0	0
cov2_5_7_10_11	26.7476	-358.331	0.6511391	0	0	0	0.11108746
cov1_3_5_6_8	26.7494	-358.332	0.6397393	0	0.27559042	0	0
cov1_5_7_9_10	26.7518	-358.333	0.6466514	0	-0.098324369	0	0
cov1_4_7_8_11	26.7547	-358.335	0.6507645	0	-0.042870188	0	0
cov1_3_5_6_7	26.7676	-358.341	0.6397909	0	0.27041943	0	0
cov2_4_5_6_11	26.7683	-358.341	0.6568067	0	0	0	-0.006803386
cov2_5_7_8_10	26.7706	-358.343	0.6485815	0	0	0	0.098605927
cov1_3_5_6_11	26.7936	-358.354	0.6395412	0	0.24701355	0	0
cov2_4_6_8_11	26.795	-358.355	0.6504256	0	0	0	0.076836133
cov1_5_7_8_9	26.7959	-358.355	0.6488876	0	-0.10202698	0	0
cov4_6_7_8_11	26.811	-358.363	0.6521567	0	0	0	0
cov1_3_5_8_11	26.8253	-358.37	0.6387061	0	0.32100605	0	0
cov1_3_5_7_11	26.8398	-358.377	0.6387241	0	0.31476019	0	0
cov1_4_6_7_11	26.86	-358.387	0.6506924	0	-0.056513322	0	0
cov2_5_8_10_11	26.8641	-358.389	0.6515667	0	0	0	0.11160871
cov5_7_8_9_10	26.8728	-358.394	0.648163	0	0	0	0
cov5_6_7_9_10	26.8793	-358.397	0.6475185	0	0	0	0
cov1_5_8_9_10	26.8804	-358.397	0.6470316	0	-0.097820449	0	0
cov1_5_6_7_9	26.9148	-358.415	0.6487339	0	-0.097565692	0	0
cov1_5_7_9_11	26.9155	-358.415	0.6486645	0	-0.10299284	0	0
cov5_6_7_8_9	26.9406	-358.428	0.6498501	0	0	0	0
cov5_7_9_10_11	26.9447	-358.43	0.6496005	0	0	0	0
cov1_4_6_8_11	26.9654	-358.44	0.6510796	0	-0.053543416	0	0
cov5_6_8_9_10	26.9987	-358.457	0.6478479	0	0	0	0

cov5_7_8_9_11	27.0081	-358.461	0.6520077	0	0	0	0
cov5_6_7_9_11	27.042	-358.478	0.650454	0	0	0	0
cov1_5_6_8_9	27.0427	-358.479	0.6491155	0	-0.094358668	0	0
cov1_5_8_9_11	27.0446	-358.48	0.6491718	0	-0.101215	0	0
cov5_8_9_10_11	27.0661	-358.49	0.6500352	0	0	0	0
cov1_5_7_8_10	27.1215	-358.518	0.6502065	0	-0.087973059	0	0
cov5_6_8_9_11	27.16	-358.537	0.6508429	0	0	0	0
cov1_7_8_9_10	27.1757	-358.545	0.6465012	0	0.035182617	0	0
cov6_7_8_9_10	27.1785	-358.547	0.6463067	0	0	0	0
cov5_6_7_8_10	27.2351	-358.575	0.6509577	0	0	0	0
cov1_5_6_7_10	27.2404	-358.577	0.6500905	0	-0.085907506	0	0
cov1_5_7_10_11	27.2404	-358.578	0.6499831	0	-0.089404921	0	0
cov7_8_9_10_11	27.2501	-358.582	0.6435632	0	0	0	0
cov5_7_8_10_11	27.2811	-358.598	0.6526969	0	0	0	0
cov1_3_4_6_11	27.2917	-358.603	0.6536246	0	0.005727736	0	0
cov1_6_7_9_10	27.2932	-358.604	0.6463834	0	0.023720378	0	0
cov1_7_9_10_11	27.2965	-358.606	0.6464438	0	0.035625092	0	0
cov6_7_9_10_11	27.3053	-358.61	0.6458881	0	0	0	0
cov1_3_4_5_8_9	27.3193	-355.22	0.6343252	0	0.20285009	0	0
cov5_6_7_10_11	27.3397	-358.627	0.651446	0	0	0	0
cov1_3_4_5_7_9	27.3651	-355.243	0.6343686	0	0.19759302	0	0
cov1_5_6_8_10	27.3685	-358.642	0.6504682	0	-0.082694872	0	0
cov1_5_8_10_11	27.3693	-358.642	0.650484	0	-0.087596053	0	0
cov1_6_7_8_9	27.3868	-358.651	0.648969	0	0.023045214	0	0
cov1_7_8_9_11	27.3923	-358.653	0.648938	0	0.037207334	0	0
cov6_7_8_9_11	27.3976	-358.656	0.6484661	0	0	0	0
cov1_6_8_9_10	27.4208	-358.668	0.6467701	0	0.026885605	0	0
cov1_8_9_10_11	27.4222	-358.668	0.6469418	0	0.037084054	0	0
cov6_8_9_10_11	27.4368	-358.676	0.646288	0	0	0	0
cov5_6_8_10_11	27.459	-358.687	0.6518361	0	0	0	0
cov3_5_6_7_8	27.4734	-358.694	0.6441849	0	0	0	0
cov1_2_6_7_8	27.4813	-358.698	0.6438112	0	-0.086549958	0	0.18638768
cov1_2_7_8_11	27.4815	-358.698	0.6444505	0	-0.066280068	0	0.18813584
cov1_2_5_7_8	27.4868	-358.701	0.6437683	0	-0.072513914	0	0.18766981
cov1_3_4_5_9_10	27.5001	-355.31	0.6331588	0	0.1304133	0	0
cov1_6_7_9_11	27.5109	-358.713	0.6488637	0	0.02689269	0	0
cov1_3_4_5_8_10	27.5635	-355.342	0.6355081	0	0.21720211	0	0
cov2_5_7_8_11	27.5709	-358.743	0.6467269	0	0	0	0.16362089
cov2_6_7_8_11	27.5819	-358.748	0.6469055	0	0	0	0.16529033
cov2_5_6_7_8	27.5839	-358.749	0.6446924	0	0	0	0.16743858
cov1_2_6_7_11	27.5906	-358.753	0.6444139	0	-0.077658201	0	0.18689684
cov1_2_5_7_11	27.5947	-358.755	0.6443023	0	-0.066522	0	0.18788741
cov1_2_5_6_7	27.5966	-358.756	0.6436647	0	-0.082808834	0	0.18654421
cov3_5_6_7_11	27.6003	-358.757	0.6416384	0	0	0	0
cov1_3_4_5_9_11	27.6022	-355.361	0.633619	0	0.11782356	0	0
cov1_3_4_5_7_10	27.6098	-355.365	0.6355509	0	0.21200536	0	0
cov1_3_4_5_6_9	27.6165	-355.368	0.6350049	0	0.1396272	0	0

cov1_2_3_4_5_9	27.6175	-355.369	0.6353515	0	0.13637326	0	-0.011782488
cov3_5_6_8_11	27.6271	-358.771	0.6417394	0	0	0	0
cov1_6_8_9_11	27.6385	-358.777	0.6493547	0	0.030901029	0	0
cov1_6_7_8_10	27.6415	-358.778	0.6499829	0	0.026939217	0	0
cov1_7_8_10_11	27.6485	-358.782	0.6499225	0	0.042658468	0	0
cov6_7_8_10_11	27.6562	-358.785	0.6493934	0	0	0	0
cov2_5_6_7_11	27.6685	-358.792	0.645832	0	0	0	0.17122122
cov1_3_6_7_8	27.6877	-358.801	0.6470854	0	0.064340538	0	0
cov1_2_6_8_11	27.7137	-358.814	0.6448417	0	-0.073917032	0	0.18715004
cov1_2_5_8_11	27.7163	-358.815	0.6447452	0	-0.065119412	0	0.18792612
cov1_2_5_6_8	27.7216	-358.818	0.6439875	0	-0.079789104	0	0.18674645
cov1_3_7_8_11	27.7415	-358.828	0.6465301	0	0.10710263	0	0
cov3_4_5_6_9_10	27.7538	-355.437	0.6341248	0	0	0	0
cov3_4_5_9_10_11	27.763	-355.442	0.6316153	0	0	0	0
cov1_6_7_10_11	27.7651	-358.84	0.6498595	0	0.030646161	0	0
cov3_6_7_8_11	27.7727	-358.844	0.6460355	0	0	0	0
cov2_3_4_5_9_10	27.7806	-355.45	0.632962	0	0	0	0.047006082
cov3_4_5_6_8_9	27.7823	-355.451	0.6358723	0	0	0	0
cov2_5_6_8_11	27.7844	-358.849	0.6461748	0	0	0	0.17219098
cov3_4_5_7_8_9	27.7969	-355.458	0.6365208	0	0	0	0
cov3_4_5_6_7_9	27.8074	-355.464	0.635871	0	0	0	0
cov3_4_5_6_9_11	27.8113	-355.466	0.633457	0	0	0	0
cov2_3_4_5_9_11	27.8224	-355.471	0.6322854	0	0	0	0.035804354
cov1_3_6_7_11	27.8316	-358.873	0.6469702	0	0.068850298	0	0
cov2_3_4_5_8_9	27.835	-355.477	0.6344458	0	0	0	0.057254438
cov3_4_5_8_9_11	27.8354	-355.478	0.6331414	0	0	0	0
cov2_3_4_5_6_9	27.8405	-355.48	0.6348362	0	0	0	0.030052807
cov3_4_5_8_9_10	27.8422	-355.481	0.6349214	0	0	0	0
cov3_4_5_7_9_11	27.848	-355.484	0.6331511	0	0	0	0
cov3_4_5_7_9_10	27.8516	-355.486	0.6348841	0	0	0	0
cov2_3_4_5_7_9	27.853	-355.486	0.634487	0	0	0	0.055325634
cov1_3_4_5_10_11	27.8542	-355.487	0.6348017	0	0.13081878	0	0
cov1_3_4_5_6_10	27.8714	-355.496	0.636219	0	0.15101195	0	0
cov1_2_3_4_5_10	27.8719	-355.496	0.6365194	0	0.14869905	0	-0.010175664
cov1_3_6_8_11	27.8756	-358.895	0.6472089	0	0.070999495	0	0
cov1_6_8_10_11	27.893	-358.904	0.6503454	0	0.034654213	0	0
cov3_4_5_6_8_10	28.0859	-355.603	0.6374221	0	0	0	0
cov3_4_5_6_10_11	28.1035	-355.612	0.6348338	0	0	0	0
cov3_4_5_6_7_10	28.1104	-355.615	0.6374113	0	0	0	0
cov2_3_4_5_10_11	28.1199	-355.62	0.6334731	0	0	0	0.042038587
cov2_3_4_5_6_10	28.1302	-355.625	0.6361091	0	0	0	0.034514224
cov3_4_5_7_8_10	28.1353	-355.628	0.6382411	0	0	0	0
cov2_3_4_5_8_10	28.1493	-355.635	0.635866	0	0	0	0.063548593
cov3_4_5_8_10_11	28.1574	-355.639	0.6345616	0	0	0	0
cov2_3_4_5_7_10	28.1661	-355.643	0.6359002	0	0	0	0.061612929
cov3_4_5_7_10_11	28.1682	-355.644	0.6345797	0	0	0	0
cov3_5_7_8_11	28.4299	-359.172	0.6398711	0	0	0	0

cov1_2_5_9_11	28.8674	-359.391	0.6638464	0	-0.15579254	0	0.16707047
cov1_3_4_5_7_8	28.9218	-356.021	0.6347422	0	0.35785188	0	0
cov1_2_5_9_10	28.9868	-359.451	0.6578178	0	-0.19842982	0	0.16349339
cov1_2_9_10_11	28.9898	-359.452	0.6604212	0	-0.058140888	0	0.17550664
cov1_6_7_8_11	28.9974	-359.456	0.6509822	0	0.050444658	0	0
cov1_5_6_7_8	28.9979	-359.456	0.6506605	0	0.042533406	0	0
cov1_3_4_5_6_8	29.0041	-356.062	0.6347337	0	0.33743733	0	0
cov1_3_4_5_8_11	29.0212	-356.071	0.6341478	0	0.35826395	0	0
cov1_2_3_4_5_8	29.0229	-356.071	0.6344233	0	0.35760206	0	0.006928715
cov5_6_7_8_11	29.0277	-359.471	0.6505161	0	0	0	0
cov1_5_7_8_11	29.039	-359.477	0.6507081	0	0.082571207	0	0
cov1_3_4_5_6_7	29.0493	-356.085	0.6347855	0	0.33381847	0	0
cov1_3_4_5_7_11	29.0635	-356.092	0.6341679	0	0.35279158	0	0
cov1_2_3_4_5_7	29.0656	-356.093	0.6344781	0	0.35242315	0	0.007030336
cov2_6_9_10_11	29.0672	-359.491	0.6625206	0	0	0	0.15577617
cov2_5_9_10_11	29.0706	-359.493	0.665173	0	0	0	0.12718383
cov1_5_6_7_11	29.1149	-359.515	0.6508782	0	0.04842009	0	0
cov1_2_4_6_11	29.1501	-359.532	0.6642722	0	-0.086205992	0	0.086942489
cov1_2_5_6_9	29.1547	-359.535	0.6601375	0	-0.19489189	0	0.16349768
cov2_5_6_9_11	29.1653	-359.54	0.6667754	0	0	0	0.14054683
cov1_2_5_10_11	29.1776	-359.546	0.6649372	0	-0.14281608	0	0.16916685
cov1_2_6_9_11	29.2092	-359.562	0.6628948	0	-0.068010475	0	0.17429129
cov1_5_6_8_11	29.242	-359.578	0.6513051	0	0.0524211	0	0
cov1_2_6_9_10	29.2877	-359.601	0.656878	0	-0.10830699	0	0.17104943
cov1_3_4_5_6_11	29.2965	-356.208	0.6345723	0	0.26294143	0	0
cov1_2_3_4_5_11	29.3032	-356.212	0.6341797	0	0.27349057	0	0.008363634
cov1_2_3_4_5_6	29.3064	-356.213	0.6353298	0	0.26271551	0	0.008204964
cov1_2_3_4_9_10	29.3389	-356.229	0.6377534	0	-0.13831465	0	0.087115735
cov1_2_3_4_7_9	29.3603	-356.24	0.6388325	0	-0.14157903	0	0.077953786
cov1_2_3_4_8_9	29.3794	-356.25	0.6388795	0	-0.14077259	0	0.079453479
cov1_3_4_7_8_9	29.38	-356.25	0.6414467	0	-0.11209689	0	0
cov1_2_3_4_6_9	29.397	-356.258	0.639073	0	-0.11191931	0	0.087039329
cov1_2_3_4_9_11	29.4175	-356.269	0.6392184	0	-0.13621597	0	0.086031142
cov2_5_6_10_11	29.4356	-359.675	0.6674513	0	0	0	0.14429448
cov1_3_4_7_9_10	29.4462	-356.283	0.640041	0	-0.113481	0	0
cov2_5_6_9_10	29.4545	-359.685	0.6606028	0	0	0	0.12898992
cov1_2_6_10_11	29.461	-359.688	0.6638037	0	-0.065093635	0	0.1756441
cov1_2_5_6_10	29.4635	-359.689	0.6612775	0	-0.18412145	0	0.16538226
cov1_3_4_8_9_10	29.4735	-356.297	0.6401424	0	-0.11208062	0	0
cov1_3_4_6_7_9	29.5127	-356.316	0.6414586	0	-0.088267892	0	0
cov3_4_6_7_8_9	29.5267	-356.323	0.6423418	0	0	0	0
cov1_3_4_7_9_11	29.529	-356.325	0.6409556	0	-0.11676109	0	0
cov1_3_4_6_8_9	29.5379	-356.329	0.6415392	0	-0.086550532	0	0
cov2_3_4_6_9_10	29.5422	-356.331	0.6394978	0	0	0	0.06332985
cov1_3_4_6_9_10	29.5434	-356.332	0.6408244	0	-0.08139198	0	0
cov1_3_4_8_9_11	29.5553	-356.338	0.6411171	0	-0.11464779	0	0
cov1_3_4_9_10_11	29.558	-356.339	0.6409452	0	-0.10192248	0	0

cov2_3_4_6_7_9	29.5669	-356.343	0.6406289	0	0	0	0.054792569
cov3_4_6_7_9_10	29.579	-356.349	0.6409935	0	0	0	0
cov2_3_4_6_8_9	29.5818	-356.351	0.640639	0	0	0	0.05625827
cov2_3_4_6_9_11	29.5992	-356.36	0.6418059	0	0	0	0.064595109
cov3_4_6_8_9_10	29.6002	-356.36	0.6410557	0	0	0	0
cov1_3_4_6_9_11	29.6159	-356.368	0.6419482	0	-0.08092527	0	0
cov3_4_6_7_9_11	29.6547	-356.387	0.6428022	0	0	0	0
cov3_4_6_9_10_11	29.658	-356.389	0.6424209	0	0	0	0
cov2_3_5_7_8_9	29.6608	-356.39	0.6319916	0	0	0	0.19231805
cov3_4_6_8_9_11	29.6742	-356.397	0.6428926	0	0	0	0
cov2_3_5_8_9_10	29.6923	-356.406	0.6303697	0	0	0	0.19428499
cov1_2_3_4_7_10	29.6967	-356.408	0.6405098	0	-0.13550701	0	0.081500981
cov2_3_5_7_9_10	29.7159	-356.418	0.630382	0	0	0	0.19259302
cov1_2_3_4_8_10	29.7164	-356.418	0.6405618	0	-0.13469335	0	0.082995617
cov1_3_4_7_8_10	29.7333	-356.427	0.6432743	0	-0.10466074	0	0
cov1_2_3_4_6_10	29.7389	-356.429	0.6407844	0	-0.10724585	0	0.090690875
cov1_2_3_4_10_11	29.7571	-356.439	0.6408848	0	-0.1303928	0	0.089748047
cov4_5_7_8_9_10	29.77	-356.445	0.6437778	0	0	0	0
cov2_4_5_7_9_10	29.779	-356.449	0.6451831	0	0	0	-0.053329607
cov2_3_5_6_9_10	29.7819	-356.451	0.6304576	0	0	0	0.18420682
cov1_2_3_5_9_10	29.7836	-356.452	0.6305039	0	-0.011244837	0	0.18433362
cov2_3_5_9_10_11	29.784	-356.452	0.6300849	0	0	0	0.18026346
cov1_2_3_5_8_9	29.7875	-356.454	0.6318777	0	0.027762373	0	0.18869455
cov2_3_5_8_9_11	29.7948	-356.457	0.6314131	0	0	0	0.19292265
cov2_3_5_6_8_9	29.7971	-356.459	0.6319776	0	0	0	0.19308798
cov2_4_5_7_8_9	29.8149	-356.467	0.647737	0	0	0	-0.05587194
cov1_2_3_5_7_9	29.8162	-356.468	0.6319206	0	0.022443121	0	0.18808
cov2_3_5_7_9_11	29.8199	-356.47	0.6314482	0	0	0	0.1912536
cov2_3_5_6_7_9	29.8229	-356.471	0.6319953	0	0	0	0.19197611
cov4_5_6_7_9_10	29.8257	-356.473	0.6437396	0	0	0	0
cov3_4_7_8_9_11	29.8273	-356.474	0.6465349	0	0	0	0
cov1_4_5_7_9_10	29.8285	-356.474	0.6432524	0	-0.053648757	0	0
cov3_4_6_7_8_10	29.8629	-356.491	0.6440382	0	0	0	0
cov1_4_5_7_8_9	29.8654	-356.493	0.6457133	0	-0.057693542	0	0
cov2_4_5_8_9_10	29.8654	-356.493	0.645498	0	0	0	-0.053949103
cov1_3_4_6_7_10	29.8675	-356.494	0.6432763	0	-0.082479169	0	0
cov4_5_6_7_8_9	29.8723	-356.496	0.6462481	0	0	0	0
cov1_3_4_7_10_11	29.8808	-356.5	0.6427176	0	-0.10992717	0	0
cov4_5_7_9_10_11	29.8821	-356.501	0.6446863	0	0	0	0
cov2_3_4_6_7_10	29.8887	-356.504	0.6421568	0	0	0	0.05910468
cov2_3_4_9_10_11	29.8906	-356.505	0.6450806	0	0	0	0.03227512
cov2_3_5_6_9_11	29.8925	-356.506	0.6314341	0	0	0	0.18297233
cov1_3_4_6_8_10	29.8934	-356.507	0.6433607	0	-0.080732205	0	0
cov1_2_3_5_9_11	29.8947	-356.507	0.6315151	0	-0.018277077	0	0.18374326
cov1_2_3_5_6_9	29.8971	-356.509	0.632158	0	-0.005429653	0	0.18464819
cov3_4_7_9_10_11	29.898	-356.509	0.6452975	0	0	0	0
cov3_4_7_8_9_10	29.9005	-356.51	0.6426614	0	0	0	0

cov2_3_4_6_8_10	29.9041	-356.512	0.6421723	0	0	0	0.060582473
cov1_5_9_10_11	29.9067	-359.911	0.6671	0	-0.035617844	0	0
cov1_3_4_8_10_11	29.908	-356.514	0.6428835	0	-0.10781363	0	0
cov3_4_8_9_10_11	29.9101	-356.515	0.6453653	0	0	0	0
cov4_5_6_8_9_10	29.9114	-356.516	0.6440268	0	0	0	0
cov1_4_5_8_9_10	29.9194	-356.52	0.6435585	0	-0.052996386	0	0
cov2_4_5_6_7_9	29.9249	-356.522	0.6472796	0	0	0	-0.044894949
cov2_3_4_6_10_11	29.9258	-356.523	0.6432833	0	0	0	0.069027612
cov2_3_5_7_8_10	29.9293	-356.525	0.6332622	0	0	0	0.1970274
cov4_5_7_8_9_11	29.93	-356.525	0.6472942	0	0	0	0
cov5_6_9_10_11	29.9331	-359.924	0.667831	0	0	0	0
cov1_2_4_5_7_9	29.9389	-356.529	0.6471484	0	-0.019912797	0	-0.046412879
cov1_2_3_7_8_9	29.9427	-356.531	0.6349771	0	-0.09807009	0	0.17639714
cov2_4_5_7_9_11	29.9446	-356.532	0.6477233	0	0	0	-0.05505667
cov2_3_4_7_9_11	29.9588	-356.539	0.6461888	0	0	0	0.026128835
cov2_3_4_8_9_11	29.9661	-356.543	0.646221	0	0	0	0.028099518
cov4_5_8_9_10_11	29.969	-356.544	0.6450538	0	0	0	0
cov2_3_4_7_8_9	29.9726	-356.546	0.6437131	0	0	0	0.007442389
cov1_3_4_6_10_11	29.9764	-356.548	0.6437738	0	-0.075139248	0	0
cov1_4_5_6_7_9	29.9765	-356.548	0.645797	0	-0.034199553	0	0
cov4_5_6_7_9_11	29.9922	-356.556	0.6463303	0	0	0	0
cov3_4_6_7_10_11	29.9926	-356.556	0.6443817	0	0	0	0
cov1_4_5_7_9_11	29.9945	-356.557	0.6456397	0	-0.057361109	0	0
cov1_2_3_6_9_10	30.0013	-356.561	0.6334872	0	-0.084001888	0	0.17761529
cov1_2_3_7_9_10	30.0078	-356.564	0.6334999	0	-0.099365802	0	0.17632858
cov1_2_3_8_9_10	30.0082	-356.564	0.6336219	0	-0.09920835	0	0.17644836
cov1_2_3_9_10_11	30.0084	-356.564	0.6335232	0	-0.099775476	0	0.17636868
cov2_4_5_6_8_9	30.01	-356.565	0.6475772	0	0	0	-0.045107229
cov3_4_6_8_10_11	30.0133	-356.567	0.6444757	0	0	0	0
cov1_2_4_5_8_9	30.0265	-356.573	0.6475098	0	-0.018025084	0	-0.047932562
cov2_4_5_8_9_11	30.0308	-356.575	0.648084	0	0	0	-0.055257881
cov1_2_3_5_8_10	30.0444	-356.582	0.633047	0	0.040780407	0	0.19079342
cov2_3_4_5_6_11	30.0448	-356.582	0.6332495	0	0	0	0.087081832
cov2_3_4_7_9_10	30.0524	-356.586	0.6424745	0	0	0	0.006575711
cov2_3_5_8_10_11	30.0613	-356.591	0.6324928	0	0	0	0.19718708
cov2_3_5_6_8_10	30.0631	-356.592	0.6332547	0	0	0	0.19651221
cov2_3_4_8_9_10	30.0643	-356.592	0.6424896	0	0	0	0.008389205
cov1_4_5_6_8_9	30.0645	-356.592	0.6461161	0	-0.031559138	0	0
cov1_5_6_9_11	30.0693	-359.992	0.6690965	0	-0.057794743	0	0
cov2_3_6_7_8_9	30.0728	-356.596	0.6362947	0	0	0	0.151771
cov1_2_3_5_7_10	30.0738	-356.597	0.6330931	0	0.035515929	0	0.19018692
cov4_5_6_8_9_11	30.0775	-356.599	0.6466658	0	0	0	0
cov2_3_4_5_6_8	30.0809	-356.6	0.6355195	0	0	0	0.097771744
cov2_3_5_7_10_11	30.0853	-356.603	0.632521	0	0	0	0.19551602
cov1_4_5_8_9_11	30.0856	-356.603	0.6460658	0	-0.055549974	0	0
cov2_3_5_6_7_10	30.0887	-356.604	0.6332676	0	0	0	0.19539263
cov2_4_5_7_8_10	30.0894	-356.605	0.648622	0	0	0	-0.048334656

cov2_3_4_5_6_7	30.0931	-356.607	0.6355265	0	0	0	0.09621966
cov1_2_3_6_8_9	30.0967	-356.608	0.6349781	0	-0.081828326	0	0.17781361
cov1_2_3_6_9_11	30.0971	-356.609	0.6346049	0	-0.083858287	0	0.1775902
cov1_2_3_6_7_9	30.098	-356.609	0.6348756	0	-0.081872242	0	0.17770501
cov1_2_3_8_9_11	30.1042	-356.612	0.6349569	0	-0.098693048	0	0.17648905
cov1_2_3_7_9_11	30.1054	-356.613	0.6347833	0	-0.09946993	0	0.17632217
cov2_3_6_9_10_11	30.1196	-356.62	0.6355775	0	0	0	0.15478175
cov2_3_6_7_9_10	30.1261	-356.623	0.634834	0	0	0	0.15273994
cov2_3_6_8_9_10	30.1262	-356.623	0.6349353	0	0	0	0.15286299
cov3_4_7_8_10_11	30.1304	-356.625	0.6478008	0	0	0	0
cov4_5_6_7_8_10	30.1408	-356.63	0.6473094	0	0	0	0
cov1_4_5_7_8_10	30.1415	-356.631	0.6469488	0	-0.043596325	0	0
cov1_5_6_9_10	30.1448	-360.03	0.6637745	0	-0.091039282	0	0
cov2_3_5_6_10_11	30.1546	-356.637	0.632566	0	0	0	0.18639039
cov1_2_3_5_10_11	30.1567	-356.638	0.6326594	0	-0.006605472	0	0.18572918
cov1_2_3_5_6_10	30.1608	-356.64	0.6333623	0	0.004786387	0	0.18651475
cov4_5_7_8_10_11	30.1787	-356.649	0.6480158	0	0	0	0
cov3_4_5_6_7_8	30.1908	-356.655	0.6394549	0	0	0	0
cov2_3_7_8_9_10	30.2038	-356.662	0.6383233	0	0	0	0.095721205
cov2_4_5_6_7_10	30.2077	-356.664	0.6482801	0	0	0	-0.040263169
cov2_3_6_8_9_11	30.2099	-356.665	0.6369496	0	0	0	0.15550269
cov2_3_6_7_9_11	30.2122	-356.666	0.6368071	0	0	0	0.15525909
cov1_2_4_5_7_10	30.2177	-356.669	0.6483419	0	-0.007049826	0	-0.044962642
cov2_4_5_7_10_11	30.2184	-356.669	0.6484691	0	0	0	-0.048480448
cov1_2_3_7_8_10	30.2444	-356.682	0.6366054	0	-0.093093696	0	0.17792416
cov2_3_4_7_8_10	30.2489	-356.684	0.6449946	0	0	0	0.013566222
cov2_3_4_7_10_11	30.251	-356.686	0.6473509	0	0	0	0.031414532
cov1_4_7_8_9_10	30.2525	-356.686	0.6445888	0	-0.16424997	0	0
cov2_3_7_8_9_11	30.2536	-356.687	0.6410881	0	0	0	0.11858799
cov1_4_5_6_7_10	30.2552	-356.688	0.6470136	0	-0.022435665	0	0
cov2_3_4_8_10_11	30.2594	-356.69	0.647389	0	0	0	0.03338886
cov4_5_6_7_10_11	30.2622	-356.691	0.6472783	0	0	0	0
cov1_4_5_7_10_11	30.27	-356.695	0.6468433	0	-0.043577094	0	0
cov3_4_5_6_8_11	30.2857	-356.703	0.6363563	0	0	0	0
cov3_4_5_6_7_11	30.2871	-356.704	0.6362881	0	0	0	0
cov2_4_5_6_8_10	30.2936	-356.707	0.6485787	0	0	0	-0.040473195
cov1_6_9_10_11	30.3037	-360.109	0.6664954	0	0.049098061	0	0
cov1_2_4_5_8_10	30.3051	-356.713	0.6486966	0	-0.005163123	0	-0.046476655
cov2_4_5_8_10_11	30.3055	-356.713	0.6488365	0	0	0	-0.048674126
cov2_3_8_9_10_11	30.3164	-356.718	0.6399576	0	0	0	0.11895443
cov2_3_7_9_10_11	30.3206	-356.72	0.63983	0	0	0	0.11836731
cov1_4_5_6_8_10	30.3433	-356.732	0.6473274	0	-0.019791949	0	0
cov4_5_6_8_10_11	30.3485	-356.734	0.6476226	0	0	0	0
cov1_4_6_7_9_10	30.3525	-356.736	0.6446747	0	-0.13527902	0	0
cov1_4_5_8_10_11	30.3608	-356.74	0.6472565	0	-0.041769249	0	0
cov1_4_6_7_8_9	30.3616	-356.741	0.6467101	0	-0.13835234	0	0
cov2_3_6_7_8_10	30.3652	-356.743	0.637827	0	0	0	0.15409255

cov1_2_4_7_9_10	30.3673	-356.744	0.6438638	0	-0.17264918	0	0.018313472
cov1_2_4_7_8_9	30.3743	-356.747	0.646033	0	-0.17191479	0	0.016086988
cov1_4_7_9_10_11	30.3777	-356.749	0.6445447	0	-0.163345	0	0
cov1_4_7_8_9_11	30.3823	-356.751	0.6465528	0	-0.16429104	0	0
cov1_2_3_6_10_11	30.3995	-356.76	0.6362423	0	-0.080544379	0	0.17878452
cov1_2_3_6_8_10	30.3998	-356.76	0.6365987	0	-0.078409701	0	0.17898252
cov1_2_3_6_7_10	30.4001	-356.76	0.6364899	0	-0.078497542	0	0.17887271
cov1_2_3_7_10_11	30.406	-356.763	0.6363431	0	-0.095182013	0	0.17756496
cov1_2_3_8_10_11	30.406	-356.763	0.6365285	0	-0.094394258	0	0.17772545
cov1_5_6_10_11	30.4083	-360.161	0.6703055	0	-0.045810103	0	0
cov1_4_6_8_9_10	30.4503	-356.785	0.6450112	0	-0.13334268	0	0
cov1_2_4_8_9_10	30.4703	-356.795	0.6442204	0	-0.17276791	0	0.01759302
cov1_2_4_6_7_9	30.4718	-356.796	0.6460818	0	-0.1418701	0	0.017326168
cov1_4_8_9_10_11	30.4795	-356.8	0.6449982	0	-0.162611	0	0
cov1_4_6_7_9_11	30.4808	-356.8	0.6464143	0	-0.13614532	0	0
cov1_2_4_7_9_11	30.5001	-356.81	0.6459576	0	-0.17133532	0	0.016053646
cov2_3_6_8_10_11	30.5047	-356.812	0.6384018	0	0	0	0.15746443
cov2_3_6_7_10_11	30.5059	-356.813	0.6382544	0	0	0	0.15720797
cov2_3_7_8_10_11	30.5294	-356.825	0.6422819	0	0	0	0.12220028
cov2_3_4_5_7_11	30.5366	-356.828	0.631213	0	0	0	0.13570179
cov2_3_4_5_8_11	30.5488	-356.834	0.6311685	0	0	0	0.13826823
cov1_2_4_6_8_9	30.5701	-356.845	0.6464364	0	-0.13968013	0	0.016739458
cov2_3_4_5_7_8	30.5719	-356.846	0.6352534	0	0	0	0.15500665
cov1_4_6_8_9_11	30.5788	-356.849	0.6468426	0	-0.13345195	0	0
cov1_2_4_8_9_11	30.6027	-356.861	0.6464446	0	-0.17033823	0	0.015456678
cov4_6_7_8_9_10	30.6059	-356.863	0.6472812	0	0	0	0
cov1_2_5_6_11	30.6752	-360.295	0.6627931	0	-0.039754999	0	0.19041327
cov4_6_7_9_10_11	30.676	-356.898	0.6485451	0	0	0	0
cov1_4_6_7_8_10	30.699	-356.909	0.6482874	0	-0.13216756	0	0
cov2_4_6_7_9_10	30.6992	-356.91	0.6474761	0	0	0	-0.013917163
cov4_6_7_8_9_11	30.7011	-356.911	0.6505886	0	0	0	0
cov1_2_4_7_8_10	30.7044	-356.912	0.647494	0	-0.16575519	0	0.020007322
cov1_4_7_8_10_11	30.7168	-356.918	0.6480978	0	-0.15673176	0	0
cov2_4_6_7_8_9	30.7212	-356.921	0.6495593	0	0	0	-0.016562068
cov4_6_8_9_10_11	30.7617	-356.941	0.6488621	0	0	0	0
cov2_4_6_8_9_10	30.787	-356.953	0.6477426	0	0	0	-0.013955323
cov2_4_6_7_9_11	30.7996	-356.96	0.6505627	0	0	0	-0.011908814
cov1_2_4_6_7_10	30.8041	-356.962	0.6475234	0	-0.1373774	0	0.021207994
cov1_4_6_7_10_11	30.8176	-356.969	0.6479474	0	-0.13030238	0	0
cov1_2_4_7_10_11	30.8293	-356.975	0.6473788	0	-0.16546004	0	0.019992678
cov2_4_6_8_9_11	30.8855	-357.003	0.6508793	0	0	0	-0.01180984
cov1_2_4_6_8_10	30.9031	-357.012	0.647877	0	-0.13516518	0	0.020617954
cov1_4_6_8_10_11	30.9161	-357.018	0.6483809	0	-0.12758931	0	0
cov1_2_4_8_10_11	30.9324	-357.026	0.6478485	0	-0.16446242	0	0.019404872
cov4_6_7_8_10_11	31.0112	-357.066	0.6518249	0	0	0	0
cov2_4_6_7_8_10	31.0323	-357.076	0.6507951	0	0	0	-0.01180427
cov3_4_5_7_8_11	31.0499	-357.085	0.6355997	0	0	0	0

cov2_4_6_7_10_11	31.113	-357.116	0.6517345	0	0	0	-0.007284774
cov1_3_5_7_8_9	31.1609	-357.14	0.6372765	0	0.13653103	0	0
cov1_3_5_8_9_10	31.1965	-357.158	0.6357435	0	0.14316916	0	0
cov1_3_7_8_9_10	31.1984	-357.159	0.6378061	0	0.04646123	0	0
cov2_4_6_8_10_11	31.1998	-357.16	0.652052	0	0	0	-0.007181398
cov4_7_8_9_10_11	31.2131	-357.167	0.6544787	0	0	0	0
cov1_3_5_7_9_10	31.214	-357.167	0.6357786	0	0.13724116	0	0
cov3_6_7_8_9_10	31.2266	-357.173	0.6375275	0	0	0	0
cov1_3_5_9_10_11	31.2328	-357.176	0.6350515	0	0.10849894	0	0
cov1_3_5_6_9_10	31.2379	-357.179	0.6359734	0	0.10541212	0	0
cov2_4_7_9_10_11	31.2381	-357.179	0.6545014	0	0	0	-0.051257776
cov2_4_7_8_9_11	31.2468	-357.183	0.6565319	0	0	0	-0.053490657
cov3_7_8_9_10_11	31.2891	-357.205	0.6334949	0	0	0	0
cov1_3_7_8_9_11	31.2937	-357.207	0.6384987	0	0.041263039	0	0
cov1_3_6_7_8_9	31.2966	-357.208	0.6392478	0	0.039224118	0	0
cov1_3_5_8_9_11	31.2973	-357.209	0.6364856	0	0.13607225	0	0
cov1_3_5_6_8_9	31.2983	-357.209	0.6372908	0	0.13163409	0	0
cov3_5_6_7_8_9	31.314	-357.217	0.6383442	0	0	0	0
cov1_3_5_7_9_11	31.3154	-357.218	0.636483	0	0.12961471	0	0
cov3_6_7_8_9_11	31.3158	-357.218	0.6378455	0	0	0	0
cov1_3_5_6_7_9	31.318	-357.219	0.6373278	0	0.12659738	0	0
cov2_4_8_9_10_11	31.3309	-357.225	0.654892	0	0	0	-0.051447227
cov1_3_5_6_9_11	31.3422	-357.231	0.6365543	0	0.098678804	0	0
cov3_5_7_8_9_10	31.3471	-357.234	0.6369477	0	0	0	0
cov1_3_7_9_10_11	31.3549	-357.237	0.637171	0	0.040526965	0	0
cov1_3_6_9_10_11	31.3577	-357.239	0.6374284	0	0.038666563	0	0
cov1_3_8_9_10_11	31.3578	-357.239	0.6373333	0	0.041444823	0	0
cov3_5_6_9_10_11	31.358	-357.239	0.6352656	0	0	0	0
cov1_3_6_7_9_10	31.3582	-357.239	0.6377886	0	0.042093485	0	0
cov1_3_6_8_9_10	31.3603	-357.24	0.6378877	0	0.042341176	0	0
cov3_5_6_8_9_10	31.3808	-357.25	0.6368579	0	0	0	0
cov3_5_6_7_9_10	31.3832	-357.252	0.6368314	0	0	0	0
cov3_6_7_9_10_11	31.3833	-357.252	0.6363502	0	0	0	0
cov3_5_7_8_9_11	31.3845	-357.252	0.6358607	0	0	0	0
cov3_6_8_9_10_11	31.3874	-357.254	0.6365063	0	0	0	0
cov2_4_5_9_10_11	31.4031	-357.262	0.6576555	0	0	0	-0.058530596
cov2_4_7_8_9_10	31.4287	-357.274	0.6521569	0	0	0	-0.082418548
cov3_5_7_9_10_11	31.4432	-357.282	0.6345107	0	0	0	0
cov1_2_4_5_9_10	31.4483	-357.284	0.6569975	0	0.039915934	0	-0.091507378
cov3_5_8_9_10_11	31.4516	-357.286	0.6345018	0	0	0	0
cov1_3_5_7_8_10	31.4551	-357.288	0.6385468	0	0.15110334	0	0
cov1_3_6_7_9_11	31.4551	-357.288	0.6384899	0	0.039336027	0	0
cov1_3_6_8_9_11	31.4562	-357.288	0.6386411	0	0.040050145	0	0
cov3_5_6_8_9_11	31.4614	-357.291	0.6366704	0	0	0	0
cov3_5_6_7_9_11	31.4646	-357.292	0.6366373	0	0	0	0
cov2_4_5_6_9_10	31.4717	-357.296	0.6563449	0	0	0	-0.075598367
cov1_2_3_5_7_8	31.4921	-357.306	0.6321199	0	0.16764049	0	0.21485075

cov1_2_4_5_7_8	31.5015	-357.311	0.6467326	0	0.12366492	0	-0.025124161
cov1_4_5_6_7_8	31.5147	-357.317	0.6457971	0	0.090774228	0	0
cov1_2_4_5_9_11	31.5159	-357.318	0.6613286	0	0.059301155	0	-0.085500947
cov1_4_5_7_8_11	31.5175	-357.319	0.6460613	0	0.10414452	0	0
cov2_4_7_8_10_11	31.5204	-357.32	0.6573655	0	0	0	-0.047380531
cov4_5_6_9_10_11	31.5318	-357.326	0.6569605	0	0	0	0
cov1_4_5_9_10_11	31.5334	-357.327	0.6569884	0	-3.98E-05	0	0
cov2_4_5_6_9_11	31.5588	-357.339	0.6601623	0	0	0	-0.066933834
cov1_3_5_6_8_10	31.5892	-357.355	0.6385756	0	0.14411064	0	0
cov1_3_5_8_10_11	31.5892	-357.355	0.6377062	0	0.1500755	0	0
cov1_2_3_5_6_8	31.5958	-357.358	0.6323371	0	0.14795598	0	0.21326258
cov1_3_5_7_10_11	31.6079	-357.364	0.637705	0	0.14367732	0	0
cov1_2_4_5_6_9	31.6083	-357.364	0.6594612	0	0.047472959	0	-0.091578985
cov1_3_5_6_7_10	31.6098	-357.365	0.6386148	0	0.1391211	0	0
cov1_3_7_8_10_11	31.6165	-357.368	0.6400487	0	0.046705682	0	0
cov1_2_3_5_8_11	31.6176	-357.369	0.6320307	0	0.17276826	0	0.2154049
cov1_3_6_7_8_10	31.6194	-357.37	0.6408602	0	0.043591036	0	0
cov1_2_4_5_6_7	31.6209	-357.37	0.6466021	0	0.11463699	0	-0.025773959
cov1_2_4_5_7_11	31.6229	-357.371	0.6467391	0	0.12428094	0	-0.024845805
cov1_2_3_5_6_7	31.6238	-357.372	0.6323786	0	0.14375339	0	0.21274244
cov1_3_5_6_10_11	31.6362	-357.378	0.6378161	0	0.10979874	0	0
cov1_4_5_6_7_11	31.6374	-357.379	0.6459715	0	0.096250351	0	0
cov3_5_6_7_8_10	31.6414	-357.381	0.6398822	0	0	0	0
cov2_4_5_6_7_8	31.6423	-357.381	0.6450347	0	0	0	0.013655413
cov4_5_6_7_8_11	31.6432	-357.382	0.6448061	0	0	0	0
cov1_2_3_5_7_11	31.6436	-357.382	0.6320423	0	0.16719119	0	0.21478236
cov3_6_7_8_10_11	31.644	-357.382	0.6393584	0	0	0	0
cov1_4_5_6_9_10	31.6639	-357.392	0.6545248	0	-0.017910119	0	0
cov2_3_5_6_7_8	31.6767	-357.398	0.6335674	0	0	0	0.23395013
cov1_2_3_5_6_11	31.7039	-357.412	0.6324385	0	0.10934254	0	0.20891024
cov1_2_4_5_6_8	31.705	-357.413	0.6469198	0	0.11813583	0	-0.027095063
cov1_2_4_5_8_11	31.7058	-357.413	0.6471411	0	0.12679526	0	-0.02619042
cov1_4_5_6_9_11	31.7059	-357.413	0.6594242	0	0.004374324	0	0
cov1_4_5_6_8_11	31.7227	-357.421	0.64631	0	0.09945089	0	0
cov3_5_7_8_10_11	31.7317	-357.426	0.6372254	0	0	0	0
cov2_4_5_7_8_11	31.7554	-357.438	0.6427398	0	0	0	0.040960113
cov2_4_5_6_7_11	31.7702	-357.445	0.644334	0	0	0	0.013049828
cov1_3_6_7_10_11	31.7767	-357.448	0.6400669	0	0.043346562	0	0
cov1_3_6_8_10_11	31.779	-357.449	0.6402132	0	0.044094304	0	0
cov3_5_6_8_10_11	31.7859	-357.453	0.6380761	0	0	0	0
cov3_5_6_7_10_11	31.7882	-357.454	0.638038	0	0	0	0
cov1_2_4_5_10_11	31.8025	-357.461	0.6624047	0	0.072434313	0	-0.084189623
cov2_3_5_6_8_11	31.8303	-357.475	0.6326206	0	0	0	0.23534344
cov2_3_5_6_7_11	31.8424	-357.481	0.632588	0	0	0	0.23382164
cov2_4_5_6_10_11	31.8625	-357.491	0.6611118	0	0	0	-0.062645504
cov2_4_5_6_8_11	31.8652	-357.493	0.6446658	0	0	0	0.013232868
cov2_3_5_7_8_11	31.8949	-357.507	0.6310904	0	0	0	0.25471128

cov1_2_4_5_6_10	31.895	-357.507	0.6605558	0	0.05849377	0	-0.090407952
cov1_4_5_6_10_11	31.9881	-357.554	0.6605168	0	0.016393737	0	0
cov1_2_5_7_9_10	32.4157	-357.768	0.6413362	0	-0.2135534	0	0.16144827
cov1_2_5_7_8_9	32.464	-357.792	0.6437056	0	-0.2171301	0	0.16113088
cov1_4_6_9_10_11	32.522	-357.821	0.660855	0	-0.13057274	0	0
cov1_2_4_9_10_11	32.5284	-357.824	0.6607242	0	-0.14479844	0	0.001275279
cov1_2_5_8_9_10	32.5443	-357.832	0.6417105	0	-0.21307375	0	0.16145755
cov1_2_5_6_7_9	32.5707	-357.845	0.6436497	0	-0.19775232	0	0.16247656
cov1_2_5_7_9_11	32.5828	-357.851	0.6440425	0	-0.2131761	0	0.16122656
cov1_2_3_4_7_8	32.6459	-357.883	0.6432229	0	-0.075909085	0	0.15088155
cov1_2_4_6_9_11	32.6472	-357.884	0.66264	0	-0.13033548	0	-0.000550581
cov1_2_4_6_9_10	32.6827	-357.901	0.6580209	0	-0.15960518	0	-0.00412856
cov1_2_7_8_9_10	32.6928	-357.906	0.6404014	0	-0.10654827	0	0.17111321
cov1_2_5_6_8_9	32.6958	-357.908	0.6440225	0	-0.19473335	0	0.16268945
cov1_2_5_8_9_11	32.7105	-357.915	0.6445498	0	-0.21146863	0	0.16133357
cov1_2_5_7_8_10	32.7588	-357.939	0.6449467	0	-0.20457912	0	0.16323281
cov1_2_3_4_6_7	32.773	-357.946	0.6432208	0	-0.086562402	0	0.14921095
cov1_2_3_4_7_11	32.7754	-357.948	0.6434855	0	-0.073647937	0	0.15002241
cov2_3_4_6_7_8	32.7821	-357.951	0.6443865	0	0	0	0.12972491
cov2_4_6_9_10_11	32.8022	-357.961	0.6650649	0	0	0	-0.024858709
cov2_3_4_7_8_11	32.8063	-357.963	0.6473244	0	0	0	0.12003202
cov1_2_7_9_10_11	32.8108	-357.965	0.6408506	0	-0.10107609	0	0.17123548
cov1_2_6_7_9_10	32.8119	-357.966	0.6402986	0	-0.097629823	0	0.17154814
cov1_2_3_6_7_8	32.8206	-357.97	0.6410227	0	-0.075662371	0	0.19568204
cov1_2_3_4_6_8	32.8249	-357.972	0.6433188	0	-0.084481638	0	0.15119638
cov1_2_3_4_8_11	32.8262	-357.973	0.6436692	0	-0.071682157	0	0.15198145
cov1_2_3_7_8_11	32.8298	-357.975	0.6412066	0	-0.054537047	0	0.19745079
cov1_2_5_6_7_10	32.8676	-357.994	0.6448897	0	-0.18731865	0	0.16441315
cov2_6_7_8_9_10	32.8738	-357.997	0.6427331	0	0	0	0.14200105
cov1_2_5_7_10_11	32.8772	-357.999	0.6452477	0	-0.20089274	0	0.16331582
cov2_5_6_7_9_10	32.8817	-358.001	0.6439629	0	0	0	0.12778956
cov2_3_4_6_7_11	32.8853	-358.003	0.645522	0	0	0	0.13261
cov2_3_6_7_8_11	32.9054	-358.013	0.6432869	0	0	0	0.17639961
cov1_2_7_8_9_11	32.9057	-358.013	0.64347	0	-0.099526528	0	0.17133128
cov1_2_6_7_8_9	32.9071	-358.014	0.6429874	0	-0.098093633	0	0.17143605
cov2_3_4_6_8_11	32.9299	-358.025	0.6456254	0	0	0	0.13495431
cov1_2_8_9_10_11	32.9356	-358.028	0.641351	0	-0.099654146	0	0.17130849
cov1_2_6_8_9_10	32.9367	-358.028	0.6406736	0	-0.094642061	0	0.17175296
cov2_6_7_9_10_11	32.9527	-358.036	0.6437833	0	0	0	0.14720956
cov1_2_3_6_7_11	32.9598	-358.04	0.6413229	0	-0.068582006	0	0.19600851
cov2_5_6_7_8_9	32.9612	-358.041	0.6465074	0	0	0	0.12624152
cov2_5_6_8_9_10	32.9924	-358.056	0.6442565	0	0	0	0.12848258
cov1_2_5_6_8_10	32.9929	-358.056	0.6452556	0	-0.18428196	0	0.16462201
cov1_2_3_6_8_11	32.9981	-358.059	0.6415801	0	-0.066851276	0	0.19635641
cov1_2_5_8_10_11	33.0048	-358.062	0.6457478	0	-0.19915909	0	0.16342148
cov2_7_8_9_10_11	33.0086	-358.064	0.6477138	0	0	0	0.11317369
cov1_2_4_6_10_11	33.0115	-358.066	0.6641489	0	-0.12567183	0	0.003119379

cov2_5_6_7_9_11	33.0208	-358.07	0.6480904	0	0	0	0.13052321
cov1_2_6_7_9_11	33.0259	-358.073	0.6432856	0	-0.091570761	0	0.17171794
cov2_6_7_8_9_11	33.0522	-358.086	0.6463829	0	0	0	0.14679538
cov2_6_8_9_10_11	33.0652	-358.093	0.6441186	0	0	0	0.14837202
cov2_5_7_9_10_11	33.1268	-358.123	0.6481817	0	0	0	0.10768775
cov2_5_6_8_9_11	33.1285	-358.124	0.6484124	0	0	0	0.1313404
cov1_2_7_8_10_11	33.1385	-358.129	0.6444071	0	-0.095351943	0	0.17280647
cov1_2_6_7_8_10	33.1403	-358.13	0.6439682	0	-0.095335606	0	0.17276276
cov1_2_3_4_6_11	33.1413	-358.131	0.6460595	0	-0.066838444	0	0.17550695
cov1_2_6_8_9_11	33.1499	-358.135	0.6437723	0	-0.087809002	0	0.17198188
cov2_5_7_8_9_10	33.1693	-358.145	0.6454976	0	0	0	0.094376643
cov1_2_4_5_6_11	33.1836	-358.152	0.6599106	0	0.18098581	0	-0.065762012
cov1_2_4_7_8_11	33.2062	-358.163	0.6472884	0	-0.097903446	0	0.10071168
cov1_2_4_6_7_8	33.2069	-358.163	0.6467939	0	-0.11065836	0	0.099629256
cov2_5_6_7_8_10	33.208	-358.164	0.6473342	0	0	0	0.13000216
cov2_5_7_8_9_11	33.2089	-358.164	0.6508742	0	0	0	0.10601277
cov2_5_8_9_10_11	33.2423	-358.181	0.6486078	0	0	0	0.10820495
cov1_2_6_7_10_11	33.2587	-358.189	0.6442298	0	-0.088875331	0	0.1730736
cov2_5_6_7_10_11	33.2719	-358.196	0.6488045	0	0	0	0.13415918
cov2_6_7_8_10_11	33.2773	-358.199	0.6471974	0	0	0	0.14882108
cov1_3_4_6_7_8	33.2961	-358.208	0.649272	0	-0.034344773	0	0
cov1_3_4_7_8_11	33.3121	-358.216	0.6492721	0	-0.011144253	0	0
cov3_4_6_7_8_11	33.3153	-358.218	0.6499488	0	0	0	0
cov1_2_4_6_7_11	33.3173	-358.219	0.6471904	0	-0.10354341	0	0.10007178
cov2_5_6_8_10_11	33.3806	-358.25	0.649163	0	0	0	0.13497149
cov1_2_6_8_10_11	33.3828	-358.251	0.6447067	0	-0.085107462	0	0.17333667
cov2_4_6_7_8_11	33.3889	-358.254	0.6503653	0	0	0	0.075801723
cov1_3_5_6_7_8	33.3976	-358.259	0.6398752	0	0.26540897	0	0
cov2_5_7_8_10_11	33.4203	-358.27	0.6512822	0	0	0	0.11136819
cov1_3_4_6_7_11	33.4228	-358.271	0.6492972	0	-0.030390206	0	0
cov1_2_4_6_8_11	33.4252	-358.273	0.6476014	0	-0.10034707	0	0.099715471
cov1_5_7_8_9_10	33.4272	-358.274	0.6467687	0	-0.0980255	0	0
cov1_3_5_7_8_11	33.4848	-358.302	0.6387406	0	0.31445595	0	0
cov1_3_4_6_8_11	33.4921	-358.306	0.649542	0	-0.02647771	0	0
cov1_4_6_7_8_11	33.5404	-358.33	0.650855	0	-0.059647802	0	0
cov1_3_5_6_8_11	33.5435	-358.332	0.6395549	0	0.27422227	0	0
cov1_5_6_7_9_10	33.546	-358.333	0.6466563	0	-0.094817202	0	0
cov1_5_7_9_10_11	33.5464	-358.333	0.6466766	0	-0.098031943	0	0
cov1_3_5_6_7_11	33.5615	-358.341	0.6395447	0	0.26863688	0	0
cov5_6_7_8_9_10	33.5641	-358.342	0.6476791	0	0	0	0
cov1_5_6_7_8_9	33.5904	-358.355	0.6488359	0	-0.10068546	0	0
cov1_5_7_8_9_11	33.5904	-358.355	0.6488057	0	-0.1024254	0	0
cov5_7_8_9_10_11	33.6189	-358.369	0.6497561	0	0	0	0
cov5_6_7_9_10_11	33.664	-358.392	0.6483471	0	0	0	0
cov1_5_6_8_9_10	33.6737	-358.397	0.6470385	0	-0.091613046	0	0
cov1_5_8_9_10_11	33.6747	-358.397	0.6471828	0	-0.096232102	0	0
cov1_5_6_7_9_11	33.7093	-358.415	0.6486329	0	-0.098379414	0	0

cov5_6_7_8_9_11	33.7252	-358.423	0.6506816	0	0	0	0
cov5_6_8_9_10_11	33.7819	-358.451	0.6487358	0	0	0	0
cov1_5_6_8_9_11	33.8373	-358.479	0.6491224	0	-0.094310761	0	0
cov1_5_7_8_10_11	33.916	-358.518	0.6501258	0	-0.088840367	0	0
cov1_5_6_7_8_10	33.9161	-358.518	0.6502054	0	-0.089036529	0	0
cov1_6_7_8_9_10	33.9642	-358.542	0.6465129	0	0.020068027	0	0
cov1_7_8_9_10_11	33.9702	-358.545	0.6465846	0	0.036147289	0	0
cov6_7_8_9_10_11	33.9729	-358.546	0.6461501	0	0	0	0
cov1_3_4_5_7_8_9	34.0043	-355.165	0.634393	0	0.19698415	0	0
cov1_3_4_5_8_9_10	34.0091	-355.167	0.632674	0	0.20309434	0	0
cov5_6_7_8_10_11	34.0223	-358.571	0.6516733	0	0	0	0
cov1_5_6_7_10_11	34.0348	-358.577	0.6499665	0	-0.086934647	0	0
cov1_3_4_5_7_9_10	34.0536	-355.189	0.6327053	0	0.19783839	0	0
cov1_6_7_9_10_11	34.0875	-358.604	0.6465173	0	0.024808333	0	0
cov1_2_3_4_5_8_9	34.1085	-355.217	0.6347757	0	0.21367406	0	-0.014606555
cov1_3_4_5_8_9_11	34.1089	-355.217	0.6336366	0	0.19643166	0	0
cov1_3_4_5_6_8_9	34.113	-355.219	0.6343296	0	0.20780288	0	0
cov1_3_4_5_7_9_11	34.154	-355.24	0.6336374	0	0.19075707	0	0
cov1_2_3_4_5_7_9	34.1544	-355.24	0.6348158	0	0.20833023	0	-0.014477278
cov1_3_4_5_6_7_9	34.1581	-355.242	0.6343744	0	0.20413887	0	0
cov1_5_6_8_10_11	34.163	-358.642	0.6504488	0	-0.082852921	0	0
cov1_6_7_8_9_11	34.1815	-358.651	0.6490872	0	0.023405	0	0
cov3_5_6_7_8_11	34.2088	-358.664	0.6417259	0	0	0	0
cov1_6_8_9_10_11	34.2147	-358.667	0.6469991	0	0.028790422	0	0
cov1_3_4_5_7_8_10	34.2498	-355.288	0.6355809	0	0.21140248	0	0
cov1_2_6_7_8_11	34.269	-358.695	0.6446017	0	-0.080747868	0	0.18677739
cov1_2_5_6_7_8	34.2758	-358.698	0.6437964	0	-0.085801335	0	0.1864067
cov1_2_5_7_8_11	34.2761	-358.698	0.6444452	0	-0.065888085	0	0.18807892
cov1_3_4_5_9_10_11	34.2788	-355.302	0.631934	0	0.12112855	0	0
cov1_3_4_5_6_9_10	34.2908	-355.308	0.6331929	0	0.14116679	0	0
cov1_2_3_4_5_9_10	34.2913	-355.308	0.6335473	0	0.13869483	0	-0.011518722
cov1_3_4_5_8_10_11	34.3524	-355.339	0.6347779	0	0.2103056	0	0
cov2_5_6_7_8_11	34.3525	-358.736	0.6460507	0	0	0	0.17058229
cov1_2_3_4_5_8_10	34.3538	-355.34	0.6359133	0	0.22688641	0	-0.013068206
cov1_3_4_5_6_8_10	34.3578	-355.342	0.6355109	0	0.22013139	0	0
cov1_2_5_6_7_11	34.3851	-358.753	0.6444067	0	-0.077309891	0	0.1869113
cov1_3_4_5_6_9_11	34.3898	-355.358	0.6335667	0	0.13178092	0	0
cov1_2_3_4_5_9_11	34.3923	-355.359	0.634019	0	0.12720681	0	-0.013408079
cov1_3_4_5_7_10_11	34.398	-355.362	0.6347816	0	0.2046902	0	0
cov1_2_3_4_5_7_10	34.4002	-355.363	0.6359531	0	0.22160016	0	-0.012939263
cov1_3_4_5_6_7_10	34.4037	-355.365	0.635556	0	0.21651738	0	0
cov1_2_3_4_5_6_9	34.408	-355.367	0.6353544	0	0.14697939	0	-0.011005737
cov3_4_5_6_7_8_9	34.4262	-355.376	0.635853	0	0	0	0
cov1_6_7_8_10_11	34.4361	-358.778	0.6500335	0	0.027404909	0	0
cov3_4_5_6_8_9_10	34.4707	-355.398	0.6342137	0	0	0	0
cov1_3_6_7_8_11	34.4823	-358.801	0.6471411	0	0.064653992	0	0
cov3_4_5_7_8_9_11	34.4831	-355.404	0.6331546	0	0	0	0

cov3_4_5_7_8_9_10	34.4882	-355.407	0.6349061	0	0	0	0
cov2_3_4_5_7_8_9	34.4889	-355.407	0.6344991	0	0	0	0.055324561
cov3_4_5_6_7_9_10	34.4944	-355.41	0.6341992	0	0	0	0
cov3_4_5_6_9_10_11	34.496	-355.411	0.6318464	0	0	0	0
cov1_2_5_6_8_11	34.5083	-358.814	0.64484	0	-0.073557848	0	0.18716569
cov2_3_4_5_9_10_11	34.5098	-355.418	0.6306407	0	0	0	0.037284262
cov2_3_4_5_6_9_10	34.5194	-355.422	0.6330565	0	0	0	0.030633664
cov3_4_5_6_8_9_11	34.5249	-355.425	0.633565	0	0	0	0
cov2_3_4_5_8_9_10	34.5256	-355.426	0.6327913	0	0	0	0.057631228
cov2_3_4_5_6_8_9	34.5308	-355.428	0.6345501	0	0	0	0.039105155
cov3_4_5_8_9_10_11	34.5323	-355.429	0.6316387	0	0	0	0
cov2_3_4_5_7_9_10	34.5423	-355.434	0.6328192	0	0	0	0.055706476
cov3_4_5_7_9_10_11	34.5435	-355.434	0.6316388	0	0	0	0
cov3_4_5_6_7_9_11	34.5489	-355.437	0.6335421	0	0	0	0
cov2_3_4_5_6_7_9	34.5584	-355.442	0.6345731	0	0	0	0.03801224
cov2_3_4_5_8_9_11	34.5586	-355.442	0.6319779	0	0	0	0.047279274
cov2_3_4_5_7_9_11	34.5769	-355.451	0.6320277	0	0	0	0.045367934
cov2_3_4_5_6_9_11	34.5869	-355.456	0.6327666	0	0	0	0.024951197
cov1_3_4_5_6_10_11	34.6436	-355.484	0.6347516	0	0.14286125	0	0
cov1_2_3_4_5_10_11	34.6452	-355.485	0.6351529	0	0.13911955	0	-0.011867115
cov1_2_3_4_5_6_10	34.6637	-355.495	0.6365238	0	0.15738573	0	-0.009542866
cov3_4_5_6_7_8_10	34.7279	-355.527	0.6373955	0	0	0	0
cov2_3_4_5_7_8_10	34.8025	-355.564	0.6359244	0	0	0	0.061667061
cov3_4_5_7_8_10_11	34.8037	-355.565	0.6345877	0	0	0	0
cov3_4_5_6_8_10_11	34.8219	-355.574	0.6349724	0	0	0	0
cov2_3_4_5_6_8_10	34.8235	-355.574	0.6359128	0	0	0	0.043470466
cov3_4_5_6_7_10_11	34.8453	-355.585	0.6349454	0	0	0	0
cov2_3_4_5_6_7_10	34.8508	-355.588	0.635941	0	0	0	0.042397204
cov2_3_4_5_8_10_11	34.8629	-355.594	0.6332205	0	0	0	0.052821541
cov2_3_4_5_6_10_11	34.8723	-355.599	0.6339976	0	0	0	0.029160694
cov2_3_4_5_7_10_11	34.88	-355.603	0.6332641	0	0	0	0.050905866
cov1_2_5_9_10_11	35.481	-359.301	0.6616051	0	-0.15101714	0	0.16775427
cov1_2_5_6_9_11	35.6618	-359.391	0.6638402	0	-0.15763457	0	0.16691611
cov1_3_4_5_6_7_8	35.6914	-356.008	0.6348328	0	0.32894856	0	0
cov1_3_4_5_7_8_11	35.7128	-356.019	0.6341869	0	0.35241993	0	0
cov1_2_3_4_5_7_8	35.715	-356.02	0.6344976	0	0.35200889	0	0.007352521
cov2_5_6_9_10_11	35.7653	-359.443	0.6643798	0	0	0	0.14171631
cov1_2_6_9_10_11	35.7793	-359.45	0.6603966	0	-0.070320708	0	0.17447845
cov1_2_5_6_9_10	35.7799	-359.45	0.6578675	0	-0.19231004	0	0.16401188
cov1_5_6_7_8_11	35.7907	-359.455	0.6510487	0	0.045681886	0	0
cov1_3_4_5_6_8_11	35.7973	-356.061	0.6343818	0	0.33490638	0	0
cov1_2_3_4_5_6_8	35.7981	-356.062	0.634572	0	0.3339088	0	0.004881692
cov1_2_3_4_5_8_11	35.8148	-356.07	0.6339526	0	0.35337758	0	0.006374982
cov1_3_4_5_6_7_11	35.8421	-356.084	0.6343837	0	0.33090875	0	0
cov1_2_3_4_5_6_7	35.8432	-356.084	0.6346155	0	0.33011062	0	0.005126193
cov1_2_3_4_5_7_11	35.857	-356.091	0.6339714	0	0.34785335	0	0.006436923
cov1_2_5_6_10_11	35.9715	-359.546	0.6649234	0	-0.14673345	0	0.16883939

cov1_2_3_4_7_8_9	36.0008	-356.163	0.6388434	0	-0.14144996	0	0.078708962
cov1_2_3_4_7_9_10	36.0675	-356.196	0.6373911	0	-0.14281841	0	0.078571264
cov1_2_3_4_8_9_10	36.0881	-356.207	0.6374548	0	-0.14199897	0	0.080062294
cov1_2_3_4_5_6_11	36.0897	-356.208	0.6343407	0	0.25798202	0	0.007194648
cov1_3_4_7_8_9_10	36.0908	-356.208	0.6400827	0	-0.11303267	0	0
cov1_2_3_4_6_9_10	36.1138	-356.22	0.6377558	0	-0.11344897	0	0.088130723
cov1_2_3_4_6_7_9	36.1325	-356.229	0.6388406	0	-0.11510775	0	0.078920414
cov1_2_3_4_9_10_11	36.1329	-356.229	0.6379908	0	-0.13618764	0	0.08718661
cov1_2_3_4_6_8_9	36.1512	-356.238	0.6388823	0	-0.11408026	0	0.080367819
cov1_2_3_4_7_9_11	36.1542	-356.24	0.6385697	0	-0.14394724	0	0.077739749
cov1_3_4_6_7_8_9	36.1625	-356.244	0.6414869	0	-0.092342766	0	0
cov1_3_4_7_8_9_11	36.1727	-356.249	0.6409905	0	-0.11629402	0	0
cov1_2_3_4_8_9_11	36.1737	-356.25	0.6386947	0	-0.14242961	0	0.079305202
cov1_2_3_4_6_9_11	36.1916	-356.258	0.6390432	0	-0.11212819	0	0.087035337
cov1_3_4_6_7_9_10	36.2234	-356.274	0.6400968	0	-0.089818224	0	0
cov2_3_4_6_7_8_9	36.2316	-356.278	0.6406987	0	0	0	0.054424079
cov1_3_4_7_9_10_11	36.2394	-356.282	0.6396666	0	-0.11695589	0	0
cov3_4_6_7_8_9_10	36.2433	-356.284	0.6410574	0	0	0	0
cov1_3_4_6_8_9_10	36.2501	-356.288	0.6401915	0	-0.088051835	0	0
cov1_3_4_8_9_10_11	36.2672	-356.296	0.6398514	0	-0.11484128	0	0
cov2_3_4_6_7_9_10	36.2821	-356.304	0.6393154	0	0	0	0.054957909
cov2_3_4_6_8_9_10	36.2982	-356.312	0.6393379	0	0	0	0.056426751
cov1_3_4_6_7_9_11	36.3035	-356.314	0.6408222	0	-0.092779562	0	0
cov3_4_6_7_8_9_11	36.3172	-356.321	0.6429348	0	0	0	0
cov2_3_4_6_9_10_11	36.3187	-356.322	0.6406542	0	0	0	0.065503547
cov1_3_4_6_8_9_11	36.3295	-356.327	0.6409737	0	-0.090524399	0	0
cov1_2_3_4_7_8_10	36.338	-356.332	0.640527	0	-0.13537083	0	0.082251416
cov1_3_4_6_9_10_11	36.338	-356.332	0.6408415	0	-0.081273032	0	0
cov2_3_5_7_8_9_10	36.3488	-356.337	0.6303889	0	0	0	0.1924366
cov2_3_4_6_7_9_11	36.353	-356.339	0.6414416	0	0	0	0.0567634
cov2_3_4_6_8_9_11	36.3669	-356.346	0.6415006	0	0	0	0.058340159
cov3_4_6_7_9_10_11	36.3693	-356.347	0.6415926	0	0	0	0
cov3_4_6_8_9_10_11	36.3899	-356.358	0.6416938	0	0	0	0
cov2_4_5_7_8_9_10	36.4442	-356.385	0.6453141	0	0	0	-0.053353968
cov1_2_3_5_7_8_9	36.4489	-356.387	0.6319252	0	0.02188578	0	0.18803552
cov2_3_5_7_8_9_11	36.4521	-356.389	0.6314347	0	0	0	0.19106071
cov2_3_5_6_7_8_9	36.4542	-356.39	0.632009	0	0	0	0.19099983
cov1_2_3_4_6_7_10	36.4715	-356.398	0.6405159	0	-0.11057261	0	0.0823994
cov1_2_3_5_8_9_10	36.4763	-356.401	0.6302866	0	0.027887819	0	0.18877271
cov2_3_5_8_9_10_11	36.4846	-356.405	0.6299087	0	0	0	0.19322928
cov2_3_5_6_8_9_10	36.4858	-356.406	0.6303839	0	0	0	0.19306117
cov1_2_3_4_7_10_11	36.4904	-356.408	0.6402064	0	-0.13835009	0	0.081243433
cov1_2_3_4_6_8_10	36.4907	-356.408	0.6405624	0	-0.10951841	0	0.083846057
cov1_4_5_7_8_9_10	36.4944	-356.41	0.6433792	0	-0.053466878	0	0
cov4_5_6_7_8_9_10	36.4998	-356.413	0.6438551	0	0	0	0
cov1_2_3_5_7_9_10	36.5036	-356.414	0.6303153	0	0.022570319	0	0.18816018
cov2_3_5_7_9_10_11	36.5083	-356.417	0.6299311	0	0	0	0.19155911

cov2_3_5_6_7_9_10	36.5102	-356.418	0.6303894	0	0	0	0.19194088
cov1_2_3_4_8_10_11	36.5104	-356.418	0.640327	0	-0.13682632	0	0.082802726
cov1_3_4_6_7_8_10	36.5177	-356.422	0.6433097	0	-0.086534232	0	0
cov1_3_4_7_8_10_11	36.5253	-356.425	0.6427716	0	-0.10951767	0	0
cov1_2_3_4_6_10_11	36.5334	-356.429	0.6407196	0	-0.10769029	0	0.090676175
cov3_4_7_8_9_10_11	36.5401	-356.433	0.6453184	0	0	0	0
cov4_5_7_8_9_10_11	36.5474	-356.436	0.6448171	0	0	0	0
cov2_3_4_6_7_8_10	36.553	-356.439	0.6422291	0	0	0	0.058736285
cov2_4_5_6_7_9_10	36.5571	-356.441	0.6449118	0	0	0	-0.043380399
cov1_2_4_5_7_9_10	36.5695	-356.447	0.6448167	0	-0.016465233	0	-0.045534715
cov2_3_5_6_9_10_11	36.573	-356.449	0.6298691	0	0	0	0.18354711
cov2_4_5_7_9_10_11	36.5731	-356.449	0.6453191	0	0	0	-0.052274848
cov1_2_3_5_9_10_11	36.5749	-356.45	0.6299422	0	-0.015303253	0	0.18409971
cov1_2_3_5_6_9_10	36.5763	-356.451	0.6304691	0	-0.004149159	0	0.18489054
cov1_2_3_5_6_8_9	36.5814	-356.453	0.6318505	0	0.031733599	0	0.18899767
cov1_2_3_5_8_9_11	36.5814	-356.453	0.6316133	0	0.025442588	0	0.18853535
cov2_3_5_6_8_9_11	36.5891	-356.457	0.6314601	0	0	0	0.19242303
cov2_4_5_6_7_8_9	36.5945	-356.46	0.6474468	0	0	0	-0.04634114
cov2_3_4_7_8_9_11	36.5987	-356.462	0.6461944	0	0	0	0.026868383
cov1_2_4_5_7_8_9	36.6037	-356.465	0.6472828	0	-0.019577083	0	-0.046583392
cov1_4_5_6_7_9_10	36.6068	-356.466	0.6434641	0	-0.031221314	0	0
cov2_4_5_7_8_9_11	36.6092	-356.467	0.6478497	0	0	0	-0.055007887
cov1_2_3_5_6_7_9	36.6098	-356.468	0.6318867	0	0.027539179	0	0.18847101
cov1_2_3_5_7_9_11	36.6098	-356.468	0.6316037	0	0.019656002	0	0.18789047
cov2_3_5_6_7_9_11	36.6145	-356.47	0.6314471	0	0	0	0.19127235
cov4_5_6_7_9_10_11	36.6194	-356.472	0.6439978	0	0	0	0
cov1_4_5_7_9_10_11	36.6228	-356.474	0.6433918	0	-0.052218276	0	0
cov1_2_3_7_8_9_10	36.6405	-356.483	0.6335395	0	-0.099525027	0	0.17645679
cov2_4_5_6_8_9_10	36.6422	-356.484	0.6452094	0	0	0	-0.043589928
cov1_4_5_6_7_8_9	36.6471	-356.486	0.6458858	0	-0.037687004	0	0
cov3_4_6_7_8_10_11	36.6548	-356.49	0.6445166	0	0	0	0
cov1_2_4_5_8_9_10	36.6568	-356.491	0.6451746	0	-0.014581712	0	-0.047046962
cov1_3_4_6_7_10_11	36.6576	-356.492	0.6425913	0	-0.087379056	0	0
cov2_4_5_8_9_10_11	36.6592	-356.492	0.6456881	0	0	0	-0.052473034
cov1_4_5_7_8_9_11	36.6599	-356.493	0.6457917	0	-0.056935463	0	0
cov4_5_6_7_8_9_11	36.6659	-356.496	0.646516	0	0	0	0
cov2_3_4_7_9_10_11	36.6699	-356.498	0.6449477	0	0	0	0.026557575
cov1_2_3_7_8_9_11	36.6742	-356.5	0.6347432	0	-0.10023946	0	0.17635366
cov2_3_4_6_7_10_11	36.6763	-356.501	0.642887	0	0	0	0.06090091
cov2_3_4_8_9_10_11	36.6785	-356.502	0.644993	0	0	0	0.028523677
cov1_3_4_6_8_10_11	36.6845	-356.505	0.6427463	0	-0.085091545	0	0
cov1_2_3_5_6_9_11	36.6861	-356.506	0.6314007	0	-0.009358815	0	0.18444971
cov1_5_6_9_10_11	36.6885	-359.904	0.6670533	0	-0.054104392	0	0
cov2_3_4_6_8_10_11	36.691	-356.508	0.6429507	0	0	0	0.062488491
cov2_3_4_7_8_9_10	36.6932	-356.509	0.642488	0	0	0	0.007336069
cov1_4_5_6_8_9_10	36.6946	-356.51	0.6437803	0	-0.028580618	0	0
cov2_3_4_5_6_7_8	36.7034	-356.514	0.635557	0	0	0	0.094643789

cov4_5_6_8_9_10_11	36.7046	-356.515	0.6443426	0	0	0	0
cov1_2_3_5_7_8_10	36.7072	-356.516	0.6331028	0	0.034963783	0	0.19014246
cov1_4_5_8_9_10_11	36.7133	-356.519	0.6438097	0	-0.050403429	0	0
cov2_3_5_7_8_10_11	36.7179	-356.522	0.6325103	0	0	0	0.19532316
cov2_3_5_6_7_8_10	36.7189	-356.522	0.6332801	0	0	0	0.19440724
cov2_4_5_6_7_9_11	36.7189	-356.522	0.6470994	0	0	0	-0.045642113
cov1_2_4_5_6_7_9	36.7195	-356.522	0.6472722	0	-0.000413123	0	-0.044766133
cov1_2_4_5_7_9_11	36.7334	-356.529	0.6470571	0	-0.020791604	0	-0.046539626
cov1_2_3_6_7_8_9	36.7335	-356.529	0.6349093	0	-0.086954651	0	0.17729785
cov1_4_5_6_7_9_11	36.7709	-356.548	0.645683	0	-0.035112928	0	0
cov2_3_6_7_8_9_10	36.7773	-356.551	0.6349743	0	0	0	0.15113228
cov1_2_3_6_7_9_10	36.7952	-356.56	0.6334115	0	-0.084093407	0	0.17754148
cov1_2_3_6_8_9_10	36.7956	-356.561	0.6335295	0	-0.083983338	0	0.17765276
cov1_2_3_6_9_10_11	36.7956	-356.561	0.6333344	0	-0.085102531	0	0.1775455
cov1_2_3_7_9_10_11	36.8023	-356.564	0.6333998	0	-0.10028683	0	0.17626227
cov1_2_3_8_9_10_11	36.8028	-356.564	0.6335907	0	-0.099495387	0	0.17642764
cov2_4_5_6_8_9_11	36.8043	-356.565	0.6474488	0	0	0	-0.045636118
cov1_2_4_5_6_8_9	36.8045	-356.565	0.6476441	0	0.00315165	0	-0.046090836
cov1_2_4_5_8_9_11	36.8211	-356.573	0.647521	0	-0.01792144	0	-0.047916736
cov2_3_4_5_6_8_11	36.8211	-356.573	0.6332767	0	0	0	0.091737509
cov2_3_4_5_6_7_11	36.832	-356.579	0.633263	0	0	0	0.090117775
cov1_2_3_5_8_10_11	36.838	-356.582	0.6327401	0	0.038029582	0	0.19060344
cov1_2_3_5_6_8_10	36.8388	-356.582	0.6330351	0	0.042899634	0	0.19095404
cov2_3_5_6_8_10_11	36.8538	-356.59	0.6326234	0	0	0	0.19569033
cov1_4_5_6_8_9_11	36.8591	-356.592	0.6460986	0	-0.03169816	0	0
cov2_3_6_7_8_9_11	36.8615	-356.593	0.6369679	0	0	0	0.15366139
cov1_2_3_5_7_10_11	36.8671	-356.596	0.632733	0	0.032299263	0	0.18996556
cov1_2_3_5_6_7_10	36.868	-356.597	0.6330715	0	0.038755227	0	0.19043529
cov2_4_5_6_7_8_10	36.8767	-356.601	0.648446	0	0	0	-0.041711576
cov2_3_5_6_7_10_11	36.879	-356.602	0.6326061	0	0	0	0.19453177
cov1_2_4_5_7_8_10	36.8833	-356.604	0.6484789	0	-0.006717362	0	-0.045133268
cov2_4_5_7_8_10_11	36.8839	-356.605	0.6486089	0	0	0	-0.048434211
cov1_2_3_6_8_9_11	36.8907	-356.608	0.6347459	0	-0.083459994	0	0.17769842
cov3_4_5_6_7_8_11	36.8915	-356.608	0.636326	0	0	0	0
cov1_2_3_6_7_9_11	36.8917	-356.609	0.6345679	0	-0.083988995	0	0.17755797
cov2_3_4_7_8_10_11	36.8917	-356.609	0.6473609	0	0	0	0.032158359
cov2_3_6_8_9_10_11	36.9133	-356.619	0.6356917	0	0	0	0.15498656
cov2_3_6_7_9_10_11	36.9141	-356.62	0.6355378	0	0	0	0.15472555
cov1_4_5_6_7_8_10	36.926	-356.626	0.6471052	0	-0.025911995	0	0
cov4_5_6_7_8_10_11	36.9351	-356.63	0.647461	0	0	0	0
cov1_4_5_7_8_10_11	36.9361	-356.631	0.6469848	0	-0.043224339	0	0
cov1_2_3_5_6_10_11	36.9492	-356.637	0.6325671	0	0.000588031	0	0.18629729
cov2_3_7_8_9_10_11	36.9559	-356.641	0.6398395	0	0	0	0.11804308
cov1_2_4_5_6_7_10	37.0011	-356.663	0.648456	0	0.010360563	0	-0.043498748
cov2_4_5_6_7_10_11	37.0012	-356.663	0.6480294	0	0	0	-0.041307797
cov1_2_4_5_7_10_11	37.0121	-356.669	0.6482251	0	-0.008193499	0	-0.045127388
cov1_4_6_7_8_9_10	37.0278	-356.677	0.6447747	0	-0.13902623	0	0

cov1_2_3_6_7_8_10	37.0361	-356.681	0.6365439	0	-0.083342559	0	0.17869701
cov1_2_4_7_8_9_10	37.0367	-356.681	0.6439957	0	-0.17278816	0	0.01834477
cov1_2_3_7_8_10_11	37.0385	-356.682	0.6363788	0	-0.095353355	0	0.17768916
cov1_4_7_8_9_10_11	37.047	-356.686	0.6446929	0	-0.16323776	0	0
cov1_4_5_6_7_10_11	37.0496	-356.688	0.6468864	0	-0.023496117	0	0
cov1_2_4_5_6_8_10	37.0862	-356.706	0.648813	0	0.013925366	0	-0.044821792
cov2_4_5_6_8_10_11	37.0876	-356.706	0.6483804	0	0	0	-0.041297185
cov1_2_4_5_8_10_11	37.0997	-356.713	0.6486857	0	-0.005318414	0	-0.046497805
cov1_2_4_6_7_9_10	37.1351	-356.73	0.6440436	0	-0.1435187	0	0.019592449
cov1_4_5_6_8_10_11	37.1379	-356.732	0.6472934	0	-0.0200673	0	0
cov1_2_4_6_7_8_9	37.147	-356.736	0.6461852	0	-0.14554961	0	0.017198795
cov1_4_6_7_9_10_11	37.147	-356.736	0.6445572	0	-0.13616755	0	0
cov2_3_6_7_8_10_11	37.1549	-356.74	0.6384171	0	0	0	0.15559189
cov1_4_6_7_8_9_11	37.1559	-356.741	0.6465502	0	-0.13955309	0	0
cov1_2_4_7_9_10_11	37.1617	-356.744	0.6440031	0	-0.1713185	0	0.018468683
cov1_2_4_7_8_9_11	37.1689	-356.747	0.6461121	0	-0.17126623	0	0.016112836
cov2_3_4_5_7_8_11	37.1861	-356.756	0.6311938	0	0	0	0.13677692
cov1_2_3_6_7_10_11	37.1935	-356.759	0.6361445	0	-0.080885886	0	0.17870377
cov1_2_3_6_8_10_11	37.1937	-356.76	0.6363229	0	-0.080306246	0	0.17884807
cov1_2_4_6_8_9_10	37.2336	-356.78	0.6444002	0	-0.14132007	0	0.019005316
cov1_4_6_8_9_10_11	37.2449	-356.785	0.6449941	0	-0.13347147	0	0
cov1_2_4_8_9_10_11	37.2642	-356.795	0.6444758	0	-0.17032806	0	0.017880536
cov1_2_4_6_7_9_11	37.2662	-356.796	0.6459317	0	-0.14296634	0	0.017175858
cov1_2_4_6_8_9_11	37.3647	-356.845	0.6463834	0	-0.14006317	0	0.016686634
cov4_6_7_8_9_10_11	37.3675	-356.846	0.6487587	0	0	0	0
cov2_4_6_7_8_9_10	37.3928	-356.859	0.6476484	0	0	0	-0.014787686
cov2_4_6_7_9_10_11	37.4673	-356.896	0.6487126	0	0	0	-0.009835206
cov1_2_4_6_7_8_10	37.4797	-356.903	0.647631	0	-0.14103694	0	0.021078872
cov2_4_6_7_8_9_11	37.4903	-356.908	0.6507923	0	0	0	-0.012599026
cov1_4_6_7_8_10_11	37.4931	-356.909	0.6480949	0	-0.13369021	0	0
cov1_2_4_7_8_10_11	37.4989	-356.912	0.6475323	0	-0.16538852	0	0.020050846
cov2_4_6_8_9_10_11	37.5531	-356.939	0.6490289	0	0	0	-0.009734067
cov1_2_4_6_7_10_11	37.5984	-356.962	0.6473507	0	-0.13865229	0	0.021029853
cov1_2_4_6_8_10_11	37.6976	-357.012	0.6477999	0	-0.13572838	0	0.020538207
cov2_4_6_7_8_10_11	37.8036	-357.065	0.6519652	0	0	0	-0.007976827
cov1_3_5_7_8_9_10	37.848	-357.087	0.6358094	0	0.13667796	0	0
cov2_4_7_8_9_10_11	37.9078	-357.117	0.6546429	0	0	0	-0.051151497
cov1_3_5_6_7_8_9	37.9473	-357.136	0.6373839	0	0.12156148	0	0
cov1_3_5_7_8_9_11	37.9485	-357.137	0.6364812	0	0.12882687	0	0
cov1_3_5_6_8_9_10	37.9856	-357.156	0.6358287	0	0.13116787	0	0
cov1_3_5_8_9_10_11	37.9866	-357.156	0.6351223	0	0.1371391	0	0
cov1_3_7_8_9_10_11	37.9892	-357.157	0.6372182	0	0.040421185	0	0
cov1_3_6_7_8_9_10	37.9905	-357.158	0.6378744	0	0.036880748	0	0
cov3_5_6_7_8_9_10	37.9991	-357.162	0.6368478	0	0	0	0
cov1_3_5_7_9_10_11	38.0034	-357.164	0.6351071	0	0.13069269	0	0
cov1_3_5_6_7_9_10	38.004	-357.165	0.6358552	0	0.12608912	0	0
cov3_6_7_8_9_10_11	38.0088	-357.167	0.6365832	0	0	0	0

cov1_3_5_6_9_10_11	38.0252	-357.175	0.6351523	0	0.10069326	0	0
cov3_5_7_8_9_10_11	38.0776	-357.201	0.6345033	0	0	0	0
cov3_5_6_7_8_9_11	38.0816	-357.204	0.6367004	0	0	0	0
cov1_3_6_7_8_9_11	38.0868	-357.206	0.6386007	0	0.034328357	0	0
cov1_3_5_6_8_9_11	38.0883	-357.207	0.6366327	0	0.12679972	0	0
cov1_3_5_6_7_9_11	38.1071	-357.216	0.6366121	0	0.12127679	0	0
cov1_2_4_5_9_10_11	38.1401	-357.233	0.6593424	0	0.063760125	0	-0.084364189
cov1_3_6_7_9_10_11	38.1493	-357.237	0.637211	0	0.037688646	0	0
cov3_5_6_8_9_10_11	38.1508	-357.238	0.6353074	0	0	0	0
cov1_3_6_8_9_10_11	38.1521	-357.239	0.6373763	0	0.038465165	0	0
cov3_5_6_7_9_10_11	38.1525	-357.239	0.6352631	0	0	0	0
cov2_4_5_6_9_10_11	38.1875	-357.256	0.657858	0	0	0	-0.065025414
cov1_3_5_6_7_8_10	38.2391	-357.282	0.6386759	0	0.13408883	0	0
cov1_2_4_5_6_9_10	38.2393	-357.282	0.6571223	0	0.049833817	0	-0.090724652
cov1_3_5_7_8_10_11	38.2416	-357.284	0.6377091	0	0.14289104	0	0
cov1_2_3_5_6_7_8	38.2587	-357.292	0.6324401	0	0.13904481	0	0.21238463
cov1_2_3_5_7_8_11	38.2866	-357.306	0.632041	0	0.16685878	0	0.21482449
cov1_2_4_5_6_7_8	38.2914	-357.308	0.6467185	0	0.11150841	0	-0.026222729
cov1_2_4_5_7_8_11	38.2959	-357.311	0.6468693	0	0.12507452	0	-0.024884688
cov1_4_5_6_7_8_11	38.3083	-357.317	0.6460864	0	0.093082569	0	0
cov1_2_4_5_6_9_11	38.3093	-357.317	0.6611822	0	0.064643061	0	-0.085133826
cov1_4_5_6_9_10_11	38.3255	-357.325	0.6570721	0	0.008232067	0	0
cov1_3_5_6_8_10_11	38.3785	-357.352	0.6378839	0	0.13893019	0	0
cov1_2_3_5_6_8_11	38.3902	-357.358	0.6324932	0	0.1489607	0	0.21332124
cov1_3_5_6_7_10_11	38.3982	-357.362	0.6378666	0	0.13345965	0	0
cov3_5_6_7_8_10_11	38.404	-357.365	0.6381036	0	0	0	0
cov1_3_6_7_8_10_11	38.409	-357.367	0.6401744	0	0.038358681	0	0
cov1_2_4_5_6_7_11	38.4151	-357.37	0.6466483	0	0.11605221	0	-0.025504717
cov1_2_3_5_6_7_11	38.4183	-357.372	0.6324721	0	0.14438112	0	0.21277696
cov2_4_5_6_7_8_11	38.4332	-357.379	0.6445593	0	0	0	0.011642476
cov2_3_5_6_7_8_11	38.4633	-357.394	0.6326519	0	0	0	0.23272179
cov1_2_4_5_6_8_11	38.4988	-357.412	0.6472054	0	0.12005964	0	-0.026788239
cov1_2_4_5_6_10_11	38.5966	-357.461	0.662474	0	0.075707055	0	-0.083962915
cov1_2_5_7_8_9_10	39.0901	-357.708	0.6414366	0	-0.21339422	0	0.1616624
cov1_2_5_6_7_9_10	39.1978	-357.762	0.6413867	0	-0.19516835	0	0.16292428
cov1_2_5_7_9_10_11	39.2073	-357.766	0.6418639	0	-0.20847466	0	0.16184094
cov1_2_5_6_7_8_9	39.2487	-357.787	0.6437376	0	-0.20076655	0	0.16241614
cov1_2_5_7_8_9_11	39.2564	-357.791	0.6441675	0	-0.21273793	0	0.16147436
cov1_2_4_6_9_10_11	39.3164	-357.821	0.6608101	0	-0.13127326	0	0.001783398
cov1_2_5_6_8_9_10	39.3227	-357.824	0.6417572	0	-0.19215069	0	0.16313821
cov1_2_5_8_9_10_11	39.3343	-357.83	0.6423638	0	-0.20675138	0	0.16194859
cov1_2_5_6_7_9_11	39.3644	-357.845	0.6439443	0	-0.19555569	0	0.16264517
cov1_2_3_4_6_7_8	39.434	-357.88	0.6432732	0	-0.090320148	0	0.15000081
cov1_2_3_4_7_8_11	39.4395	-357.882	0.6435285	0	-0.073130935	0	0.15108302
cov1_2_7_8_9_10_11	39.4834	-357.904	0.6409835	0	-0.10078374	0	0.17152584
cov1_2_6_7_8_9_10	39.4864	-357.906	0.6404008	0	-0.10112356	0	0.17149518
cov1_2_5_6_8_9_11	39.4887	-357.907	0.644425	0	-0.19173619	0	0.1629225

cov1_2_5_6_7_8_10	39.5459	-357.936	0.6449793	0	-0.19031495	0	0.16434999
cov1_2_5_7_8_10_11	39.5515	-357.938	0.6453796	0	-0.20046817	0	0.16354747
cov2_3_4_6_7_8_11	39.5557	-357.941	0.6456828	0	0	0	0.13265715
cov1_2_3_4_6_7_11	39.5662	-357.946	0.6435932	0	-0.083988795	0	0.14941133
cov2_5_6_7_8_9_10	39.5751	-357.95	0.6441147	0	0	0	0.12716512
cov1_2_6_7_9_10_11	39.6035	-357.964	0.6408058	0	-0.093785341	0	0.17183195
cov1_2_3_6_7_8_11	39.6133	-357.969	0.6414704	0	-0.072572433	0	0.19590567
cov1_2_3_4_6_8_11	39.6175	-357.971	0.6437844	0	-0.081361029	0	0.15143091
cov2_5_6_7_9_10_11	39.6309	-357.978	0.6457252	0	0	0	0.13162117
cov2_6_7_8_9_10_11	39.6369	-357.981	0.6440471	0	0	0	0.14630534
cov1_2_5_6_7_10_11	39.6614	-357.993	0.6451632	0	-0.18522017	0	0.16457162
cov1_2_6_7_8_9_11	39.6994	-358.012	0.6434393	0	-0.09474982	0	0.17166431
cov2_5_6_7_8_9_11	39.7112	-358.018	0.648265	0	0	0	0.1300176
cov1_2_6_8_9_10_11	39.727	-358.026	0.6412856	0	-0.090035653	0	0.17209577
cov2_5_6_8_9_10_11	39.7385	-358.032	0.6460841	0	0	0	0.13242837
cov1_2_5_6_8_10_11	39.7859	-358.056	0.6456392	0	-0.1813877	0	0.16484725
cov2_5_7_8_9_10_11	39.7992	-358.062	0.6483252	0	0	0	0.10796924
cov1_2_6_7_8_10_11	39.9327	-358.129	0.6443832	0	-0.092024198	0	0.17303409
cov2_5_6_7_8_10_11	39.9619	-358.144	0.6490115	0	0	0	0.13364203
cov1_2_4_6_7_8_11	39.9982	-358.162	0.6473542	0	-0.10665035	0	0.10004967
cov1_3_4_6_7_8_11	40.0905	-358.208	0.6494177	0	-0.033301685	0	0
cov1_3_5_6_7_8_11	40.1915	-358.258	0.6396439	0	0.26365877	0	0
cov1_5_6_7_8_9_10	40.2218	-358.274	0.6467664	0	-0.097932386	0	0
cov1_5_7_8_9_10_11	40.2218	-358.274	0.6468228	0	-0.097475788	0	0
cov1_5_6_7_9_10_11	40.3406	-358.333	0.64666	0	-0.094786416	0	0
cov5_6_7_8_9_10_11	40.3471	-358.336	0.6485745	0	0	0	0
cov1_5_6_7_8_9_11	40.3849	-358.355	0.6487957	0	-0.1011044	0	0
cov1_5_6_8_9_10_11	40.4681	-358.397	0.6471457	0	-0.090722202	0	0
cov1_3_4_5_7_8_9_10	40.6933	-355.112	0.6327335	0	0.19723192	0	0
cov1_5_6_7_8_10_11	40.7106	-358.518	0.6501303	0	-0.08964998	0	0
cov1_6_7_8_9_10_11	40.7584	-358.542	0.6466854	0	0.021504088	0	0
cov1_3_4_5_7_8_9_11	40.7929	-355.162	0.6336393	0	0.18992925	0	0
cov1_2_3_4_5_7_8_9	40.7939	-355.162	0.6348315	0	0.20749328	0	-0.014203201
cov1_2_3_4_5_8_9_10	40.7985	-355.165	0.6331283	0	0.2137168	0	-0.014337727
cov1_3_4_5_6_7_8_9	40.7987	-355.165	0.6343952	0	0.19920549	0	0
cov1_3_4_5_8_9_10_11	40.7998	-355.165	0.6320784	0	0.19747724	0	0
cov1_3_4_5_6_8_9_10	40.803	-355.167	0.6326799	0	0.20738895	0	0
cov1_2_3_4_5_7_9_10	40.8431	-355.187	0.6331513	0	0.20837181	0	-0.014201918
cov1_3_4_5_7_9_10_11	40.8438	-355.187	0.6320689	0	0.19180618	0	0
cov1_3_4_5_6_7_9_10	40.8469	-355.189	0.632713	0	0.20369973	0	0
cov1_2_3_4_5_8_9_11	40.8974	-355.214	0.6340743	0	0.20748544	0	-0.015446436
cov1_3_4_5_6_8_9_11	40.9019	-355.216	0.633595	0	0.20248537	0	0
cov1_2_3_4_5_6_8_9	40.9024	-355.217	0.6347697	0	0.21763077	0	-0.014285942
cov1_2_3_4_5_7_9_11	40.9427	-355.237	0.6340746	0	0.20175723	0	-0.015360755
cov1_3_4_5_6_7_9_11	40.9463	-355.239	0.6335872	0	0.19840107	0	0
cov1_2_3_4_5_6_7_9	40.9478	-355.239	0.6348033	0	0.2138011	0	-0.014039582
cov1_3_4_5_7_8_10_11	41.0376	-355.284	0.6347882	0	0.20386886	0	0

cov1_2_3_4_5_7_8_10	41.0404	-355.286	0.6359737	0	0.22077193	0	-0.012664961
cov1_3_4_5_6_7_8_10	41.0444	-355.288	0.6355812	0	0.21159509	0	0
cov1_2_5_6_7_8_11	41.0636	-358.695	0.6445861	0	-0.079961882	0	0.18679697
cov1_3_4_5_6_9_10_11	41.0675	-355.299	0.6318876	0	0.13396911	0	0
cov1_2_3_4_5_9_10_11	41.0691	-355.3	0.6323241	0	0.13025027	0	-0.01303856
cov1_2_3_4_5_6_9_10	41.0825	-355.307	0.6335408	0	0.1484478	0	-0.010825524
cov3_4_5_6_7_8_9_10	41.1136	-355.322	0.6341827	0	0	0	0
cov1_2_3_4_5_8_10_11	41.1421	-355.336	0.6351772	0	0.2202919	0	-0.013960992
cov1_3_4_5_6_8_10_11	41.1462	-355.339	0.6347505	0	0.21453943	0	0
cov1_2_3_4_5_6_8_10	41.1482	-355.34	0.63591	0	0.22900506	0	-0.012897214
cov3_4_5_6_7_8_9_11	41.1699	-355.35	0.6335747	0	0	0	0
cov2_3_4_5_7_8_9_10	41.1787	-355.355	0.6328348	0	0	0	0.055705278
cov3_4_5_7_8_9_10_11	41.1791	-355.355	0.6316448	0	0	0	0
cov2_3_4_5_6_7_8_9	41.1801	-355.355	0.6346056	0	0	0	0.036690886
cov1_2_3_4_5_6_9_11	41.1805	-355.356	0.6339363	0	0.13997344	0	-0.012530915
cov1_2_3_4_5_7_10_11	41.1878	-355.359	0.6351784	0	0.21462377	0	-0.013875268
cov1_3_4_5_6_7_10_11	41.1912	-355.361	0.634744	0	0.21050826	0	0
cov1_2_3_4_5_6_7_10	41.1942	-355.363	0.6359482	0	0.22523024	0	-0.012649697
cov2_3_4_5_7_8_9_11	41.2119	-355.371	0.6320268	0	0	0	0.04530983
cov3_4_5_6_8_9_10_11	41.2174	-355.374	0.6320263	0	0	0	0
cov2_3_4_5_6_8_9_10	41.2189	-355.375	0.6328707	0	0	0	0.03922695
cov3_4_5_6_7_9_10_11	41.2402	-355.386	0.6319914	0	0	0	0
cov2_3_4_5_6_7_9_10	41.2452	-355.388	0.6328917	0	0	0	0.038159455
cov2_3_4_5_8_9_10_11	41.2535	-355.392	0.6304246	0	0	0	0.047932763
cov2_3_4_5_6_9_10_11	41.2704	-355.401	0.6311159	0	0	0	0.02575849
cov2_3_4_5_7_9_10_11	41.2705	-355.401	0.6304617	0	0	0	0.046027324
cov2_3_4_5_6_8_9_11	41.2852	-355.408	0.6326499	0	0	0	0.034069859
cov2_3_4_5_6_7_9_11	41.3115	-355.421	0.6326561	0	0	0	0.032932188
cov1_2_3_4_5_6_10_11	41.435	-355.483	0.6350832	0	0.15012355	0	-0.011114093
cov3_4_5_6_7_8_10_11	41.4651	-355.498	0.6349748	0	0	0	0
cov2_3_4_5_6_7_8_10	41.4716	-355.501	0.6359628	0	0	0	0.041037957
cov2_3_4_5_7_8_10_11	41.5155	-355.523	0.6332669	0	0	0	0.050848169
cov2_3_4_5_6_8_10_11	41.5735	-355.552	0.6339162	0	0	0	0.038132204
cov2_3_4_5_6_7_10_11	41.5995	-355.565	0.6339177	0	0	0	0.036983676
cov1_2_5_6_9_10_11	42.2752	-359.3	0.661592	0	-0.15415842	0	0.16749083
cov1_3_4_5_6_7_8_11	42.4842	-356.008	0.6344392	0	0.32609846	0	0
cov1_2_3_4_5_6_7_8	42.4853	-356.008	0.6346627	0	0.3252494	0	0.005084139
cov1_2_3_4_5_7_8_11	42.5062	-356.018	0.6339724	0	0.34726943	0	0.006738086
cov1_2_3_4_5_6_8_11	42.5914	-356.061	0.6342401	0	0.3316841	0	0.004556875
cov1_2_3_4_5_6_7_11	42.6361	-356.083	0.6342353	0	0.32754543	0	0.004755416
cov1_2_3_4_7_8_9_10	42.709	-356.12	0.6374115	0	-0.14268041	0	0.079322329
cov1_2_3_4_6_7_8_9	42.78	-356.155	0.6388499	0	-0.11944649	0	0.079478598
cov1_2_3_4_7_8_9_11	42.7947	-356.163	0.6385836	0	-0.14378905	0	0.078499398
cov1_2_3_4_6_7_9_10	42.8407	-356.186	0.6374107	0	-0.11693823	0	0.079513477
cov1_2_3_4_6_8_9_10	42.8609	-356.196	0.6374687	0	-0.11585487	0	0.080954572
cov1_2_3_4_7_9_10_11	42.8617	-356.196	0.6372168	0	-0.14441173	0	0.078417893
cov1_3_4_6_7_8_9_10	42.874	-356.202	0.6401298	0	-0.093867895	0	0

cov1_2_3_4_8_9_10_11	42.8826	-356.207	0.6373549	0	-0.1429062	0	0.079980384
cov1_3_4_7_8_9_10_11	42.8841	-356.207	0.6397108	0	-0.11648735	0	0
cov1_2_3_4_6_9_10_11	42.9083	-356.22	0.6378338	0	-0.11291669	0	0.088146998
cov1_2_3_4_6_7_9_11	42.9252	-356.228	0.6383848	0	-0.11824763	0	0.078585326
cov1_2_3_4_6_8_9_11	42.9444	-356.238	0.6385004	0	-0.11668791	0	0.080090117
cov2_3_4_6_7_8_9_10	42.9477	-356.239	0.6394054	0	0	0	0.054591717
cov1_3_4_6_7_8_9_11	42.9537	-356.242	0.6408829	0	-0.096606226	0	0
cov1_3_4_6_7_9_10_11	43.0152	-356.273	0.6395493	0	-0.093724142	0	0
cov2_3_4_6_7_8_9_11	43.0162	-356.274	0.6415792	0	0	0	0.056552688
cov3_4_6_7_8_9_10_11	43.0325	-356.282	0.641732	0	0	0	0
cov1_3_4_6_8_9_10_11	43.0425	-356.287	0.6397165	0	-0.091422518	0	0
cov2_3_4_6_7_9_10_11	43.0663	-356.299	0.6402016	0	0	0	0.057148895
cov2_3_4_6_8_9_10_11	43.0813	-356.306	0.6402721	0	0	0	0.058727293
cov1_2_3_4_6_7_8_10	43.1193	-356.325	0.6405312	0	-0.11489205	0	0.082958438
cov1_2_3_4_7_8_10_11	43.1316	-356.331	0.6402174	0	-0.13818105	0	0.081998082
cov1_2_3_5_7_8_9_10	43.1368	-356.334	0.6303243	0	0.022013703	0	0.18811631
cov2_3_5_7_8_9_10_11	43.1411	-356.336	0.6299205	0	0	0	0.19136746
cov2_3_5_6_7_8_9_10	43.1419	-356.336	0.6304054	0	0	0	0.19096531
cov2_4_5_6_7_8_9_10	43.2267	-356.379	0.6450779	0	0	0	-0.044826208
cov1_2_4_5_7_8_9_10	43.2349	-356.383	0.6449673	0	-0.01612448	0	-0.045689384
cov2_4_5_7_8_9_10_11	43.2383	-356.385	0.6454585	0	0	0	-0.052228223
cov1_2_3_5_7_8_9_11	43.2423	-356.387	0.6315961	0	0.018882467	0	0.18783034
cov1_2_3_5_6_7_8_9	43.2435	-356.387	0.6319198	0	0.022787825	0	0.18811367
cov2_3_5_6_7_8_9_11	43.2462	-356.389	0.6315007	0	0	0	0.19034893
cov1_2_3_4_6_7_10_11	43.2637	-356.397	0.640022	0	-0.11400497	0	0.082027638
cov1_2_3_5_6_8_9_10	43.2704	-356.401	0.6302649	0	0.031217808	0	0.18902777
cov1_2_3_5_8_9_10_11	43.2706	-356.401	0.6301155	0	0.02637597	0	0.18867038
cov1_4_5_6_7_8_9_10	43.2776	-356.404	0.6435531	0	-0.034706789	0	0
cov2_3_5_6_8_9_10_11	43.2787	-356.405	0.6299822	0	0	0	0.1925298
cov1_2_3_4_6_8_10_11	43.2836	-356.407	0.6401414	0	-0.11242341	0	0.083533174
cov1_4_5_7_8_9_10_11	43.2887	-356.41	0.6435368	0	-0.051859412	0	0
cov4_5_6_7_8_9_10_11	43.2929	-356.412	0.6441798	0	0	0	0
cov1_2_3_5_6_7_9_10	43.2974	-356.414	0.6302883	0	0.026984513	0	0.18849919
cov1_2_3_5_7_9_10_11	43.2977	-356.414	0.630094	0	0.020596344	0	0.18802603
cov2_3_5_6_7_9_10_11	43.3028	-356.417	0.6299509	0	0	0	0.19136858
cov1_3_4_6_7_8_10_11	43.3082	-356.42	0.6426577	0	-0.091182143	0	0
cov2_3_4_7_8_9_10_11	43.3108	-356.421	0.6449606	0	0	0	0.027297048
cov2_3_4_6_7_8_10_11	43.3393	-356.435	0.6430271	0	0	0	0.060691603
cov2_4_5_6_7_9_10_11	43.3514	-356.441	0.6447979	0	0	0	-0.043856606
cov1_2_4_5_6_7_9_10	43.3516	-356.441	0.6449465	0	0.001941651	0	-0.043984824
cov1_2_4_5_7_9_10_11	43.3641	-356.447	0.6448144	0	-0.016489625	0	-0.045538166
cov1_2_3_5_6_9_10_11	43.367	-356.449	0.6298281	0	-0.007457297	0	0.18472375
cov1_2_3_5_6_8_9_11	43.3751	-356.453	0.631547	0	0.029680251	0	0.18886124
cov2_4_5_6_7_8_9_11	43.3888	-356.46	0.6473163	0	0	0	-0.046881071
cov1_2_4_5_6_7_8_9	43.3889	-356.46	0.6473815	0	-0.003573537	0	-0.045225153
cov1_2_4_5_7_8_9_11	43.3982	-356.465	0.6472089	0	-0.020290054	0	-0.046686573
cov1_4_5_6_7_9_10_11	43.4014	-356.466	0.6434481	0	-0.0313439	0	0

cov1_2_3_5_6_7_9_11	43.4031	-356.467	0.6315248	0	0.0250489	0	0.18830827
cov1_2_3_6_7_8_9_10	43.4316	-356.481	0.633479	0	-0.089006873	0	0.17728117
cov1_2_3_7_8_9_10_11	43.4349	-356.483	0.6334381	0	-0.10045685	0	0.17639063
cov1_2_4_5_6_8_9_10	43.4364	-356.484	0.6453065	0	0.005501362	0	-0.045303921
cov2_4_5_6_8_9_10_11	43.4367	-356.484	0.6451474	0	0	0	-0.043848882
cov1_4_5_6_7_8_9_11	43.4416	-356.486	0.6458157	0	-0.038246822	0	0
cov2_3_4_5_6_7_8_11	43.4441	-356.487	0.6333337	0	0	0	0.088657746
cov1_2_4_5_8_9_10_11	43.4513	-356.491	0.6452727	0	-0.013623506	0	-0.046906966
cov1_2_3_6_7_8_9_11	43.4663	-356.499	0.634622	0	-0.091638288	0	0.17703841
cov1_4_5_6_8_9_10_11	43.4891	-356.51	0.6438606	0	-0.027933358	0	0
cov1_2_3_5_7_8_10_11	43.5002	-356.516	0.6327184	0	0.031528827	0	0.18990832
cov1_2_3_5_6_7_8_10	43.5017	-356.516	0.6331095	0	0.034008034	0	0.19007756
cov2_3_5_6_7_8_10_11	43.5098	-356.52	0.6326612	0	0	0	0.19360114
cov2_3_6_7_8_9_10_11	43.5106	-356.521	0.6357887	0	0	0	0.15238891
cov1_2_4_5_6_7_9_11	43.5135	-356.522	0.6470495	0	-0.002092632	0	-0.045036327
cov1_2_3_6_7_9_10_11	43.5894	-356.56	0.6332023	0	-0.085542176	0	0.17744035
cov1_2_3_6_8_9_10_11	43.59	-356.56	0.633392	0	-0.084952511	0	0.17758397
cov1_2_4_5_6_8_9_11	43.5988	-356.565	0.6474993	0	0.002137986	0	-0.046255072
cov1_2_3_5_6_8_10_11	43.6323	-356.582	0.6327002	0	0.040599296	0	0.19080063
cov1_2_3_5_6_7_10_11	43.661	-356.596	0.6326762	0	0.03602277	0	0.19025277
cov2_4_5_6_7_8_10_11	43.6706	-356.601	0.6482461	0	0	0	-0.04254728
cov1_2_4_5_6_7_8_10	43.6707	-356.601	0.6485677	0	0.007208478	0	-0.043956675
cov1_2_4_5_7_8_10_11	43.6777	-356.604	0.648379	0	-0.007695377	0	-0.045274376
cov1_4_5_6_7_8_10_11	43.7205	-356.626	0.6470192	0	-0.026614165	0	0
cov1_2_4_5_6_7_10_11	43.795	-356.663	0.6482227	0	0.008569492	0	-0.043787345
cov1_2_4_6_7_8_9_10	43.8107	-356.671	0.6441494	0	-0.14718814	0	0.019462947
cov1_4_6_7_8_9_10_11	43.8224	-356.677	0.6447025	0	-0.13956759	0	0
cov1_2_3_6_7_8_10_11	43.8298	-356.68	0.6362431	0	-0.085582454	0	0.17846016
cov1_2_4_7_8_9_10_11	43.831	-356.681	0.6441563	0	-0.17125326	0	0.018524238
cov1_2_4_5_6_8_10_11	43.8805	-356.706	0.6486663	0	0.012807259	0	-0.045005346
cov1_2_4_6_7_9_10_11	43.9297	-356.73	0.644052	0	-0.14391556	0	0.019400999
cov1_2_4_6_7_8_9_11	43.9415	-356.736	0.6460796	0	-0.14631871	0	0.017092682
cov1_2_4_6_8_9_10_11	44.0282	-356.78	0.6444294	0	-0.14102497	0	0.019047487
cov2_4_6_7_8_9_10_11	44.1583	-356.845	0.6489457	0	0	0	-0.010517796
cov1_2_4_6_7_8_10_11	44.2741	-356.902	0.6475023	0	-0.14198412	0	0.020945547
cov1_3_5_6_7_8_9_10	44.6336	-357.082	0.6359128	0	0.12105902	0	0
cov1_3_5_7_8_9_10_11	44.6371	-357.084	0.6351098	0	0.12990349	0	0
cov1_3_5_6_7_8_9_11	44.7365	-357.134	0.636675	0	0.11629427	0	0
cov3_5_6_7_8_9_10_11	44.7699	-357.15	0.635327	0	0	0	0
cov1_3_5_6_8_9_10_11	44.7769	-357.154	0.6352764	0	0.12704486	0	0
cov1_3_6_7_8_9_10_11	44.782	-357.156	0.6373259	0	0.032728438	0	0
cov1_3_5_6_7_9_10_11	44.7945	-357.163	0.6352437	0	0.12149315	0	0
cov1_2_4_5_6_9_10_11	44.9339	-357.232	0.6590653	0	0.067667808	0	-0.084057654
cov1_3_5_6_7_8_10_11	45.0276	-357.279	0.6379328	0	0.12847964	0	0
cov1_2_3_5_6_7_8_11	45.0532	-357.292	0.632541	0	0.13972677	0	0.21242301
cov1_2_4_5_6_7_8_11	45.0854	-357.308	0.6469324	0	0.11310448	0	-0.025970646
cov1_2_5_6_7_8_9_10	45.8761	-357.703	0.6414723	0	-0.19816359	0	0.16288256

cov1_2_5_7_8_9_10_11	45.8814	-357.706	0.6419904	0	-0.20804426	0	0.16208984
cov1_2_5_6_7_9_10_11	45.9907	-357.761	0.6417849	0	-0.19215868	0	0.16315396
cov1_2_5_6_7_8_9_11	46.0421	-357.786	0.6440838	0	-0.19818916	0	0.16262544
cov1_2_5_6_8_9_10_11	46.1146	-357.823	0.642264	0	-0.18834575	0	0.16342977
cov1_2_3_4_6_7_8_11	46.227	-357.879	0.643685	0	-0.087483268	0	0.15022051
cov1_2_6_7_8_9_10_11	46.2775	-357.904	0.6409585	0	-0.096942402	0	0.17179308
cov2_5_6_7_8_9_10_11	46.3213	-357.926	0.6459327	0	0	0	0.13110461
cov1_2_5_6_7_8_10_11	46.3394	-357.935	0.6453046	0	-0.18786151	0	0.16453407
cov1_5_6_7_8_9_10_11	47.0164	-358.274	0.6468217	0	-0.09750961	0	0
cov1_2_3_4_5_7_8_9_10	47.483	-355.11	0.6331703	0	0.20753807	0	-0.013928104
cov1_3_4_5_7_8_9_10_11	47.4832	-355.11	0.6320738	0	0.19097977	0	0
cov1_3_4_5_6_7_8_9_10	47.4878	-355.112	0.6327359	0	0.19875882	0	0
cov1_2_3_4_5_7_8_9_11	47.5818	-355.159	0.6340687	0	0.20072648	0	-0.015109455
cov1_3_4_5_6_7_8_9_11	47.587	-355.162	0.6336159	0	0.19352251	0	0
cov1_2_3_4_5_6_7_8_9	47.5884	-355.162	0.6348296	0	0.20888081	0	-0.014085838
cov1_2_3_4_5_8_9_10_11	47.5887	-355.162	0.6325134	0	0.20826558	0	-0.015078114
cov1_2_3_4_5_6_8_9_10	47.5926	-355.164	0.6331201	0	0.21706605	0	-0.014066775
cov1_3_4_5_6_8_9_10_11	47.5933	-355.165	0.6320459	0	0.20273936	0	0
cov1_2_3_4_5_7_9_10_11	47.6327	-355.184	0.6325018	0	0.20253735	0	-0.014985774
cov1_3_4_5_6_7_9_10_11	47.6365	-355.186	0.6320281	0	0.1986225	0	0
cov1_2_3_4_5_6_7_9_10	47.6367	-355.186	0.6331467	0	0.21319846	0	-0.013816056
cov1_2_3_4_5_6_8_9_11	47.6908	-355.214	0.6340265	0	0.21257696	0	-0.015050442
cov1_2_3_4_5_6_7_9_11	47.7353	-355.236	0.6340145	0	0.20836914	0	-0.014854422
cov1_2_3_4_5_7_8_10_11	47.8276	-355.282	0.6351776	0	0.2135996	0	-0.013625102
cov1_3_4_5_6_7_8_10_11	47.8321	-355.284	0.6347771	0	0.20563693	0	0
cov1_2_3_4_5_6_7_8_10	47.835	-355.286	0.6359737	0	0.22033163	0	-0.012665369
cov1_2_3_4_5_6_9_10_11	47.8583	-355.297	0.6322554	0	0.14196685	0	-0.012234821
cov3_4_5_6_7_8_9_10_11	47.8614	-355.299	0.6320235	0	0	0	0
cov2_3_4_5_6_7_8_9_10	47.8673	-355.302	0.6329159	0	0	0	0.03680729
cov2_3_4_5_7_8_9_10_11	47.9061	-355.321	0.6304642	0	0	0	0.045968336
cov2_3_4_5_6_7_8_9_11	47.9347	-355.335	0.6327163	0	0	0	0.031680423
cov1_2_3_4_5_6_8_10_11	47.9361	-355.336	0.6351456	0	0.22372268	0	-0.013696364
cov2_3_4_5_6_8_9_10_11	47.977	-355.357	0.6310851	0	0	0	0.034422324
cov1_2_3_4_5_6_7_10_11	47.9813	-355.359	0.635136	0	0.21956561	0	-0.013498499
cov2_3_4_5_6_7_9_10_11	48.0021	-355.369	0.6310793	0	0	0	0.033280323
cov2_3_4_5_6_7_8_10_11	48.2218	-355.479	0.6339777	0	0	0	0.035726996
cov1_2_3_4_5_6_7_8_11	49.2783	-356.007	0.634291	0	0.32273601	0	0.004721612
cov1_2_3_4_6_7_8_9_10	49.489	-356.113	0.6374271	0	-0.12124462	0	0.080069329
cov1_2_3_4_7_8_9_10_11	49.5033	-356.12	0.6372366	0	-0.14426954	0	0.079178081
cov1_2_3_4_6_7_8_9_11	49.5729	-356.155	0.6384293	0	-0.12234001	0	0.079171419
cov1_2_3_4_6_7_9_10_11	49.634	-356.185	0.6370468	0	-0.11946518	0	0.079240599
cov1_2_3_4_6_8_9_10_11	49.6546	-356.195	0.6371828	0	-0.11785818	0	0.08073949
cov1_3_4_6_7_8_9_10_11	49.6661	-356.201	0.6396161	0	-0.097523008	0	0
cov2_3_4_6_7_8_9_10_11	49.7303	-356.233	0.6403472	0	0	0	0.056951114
cov1_2_3_4_6_7_8_10_11	49.9118	-356.324	0.6400712	0	-0.11808156	0	0.082612403
cov1_2_3_5_7_8_9_10_11	49.9308	-356.334	0.6300781	0	0.019822127	0	0.18797112
cov1_2_3_5_6_7_8_9_10	49.9314	-356.334	0.630323	0	0.022238064	0	0.18814351

cov2_3_5_6_7_8_9_10_11	49.9348	-356.336	0.6300041	0	0	0	0.19044654
cov2_4_5_6_7_8_9_10_11	50.0212	-356.379	0.6450159	0	0	0	-0.045067354
cov1_2_4_5_6_7_8_9_10	50.0213	-356.379	0.6450563	0	-0.001217181	0	-0.044442895
cov1_2_4_5_7_8_9_10_11	50.0294	-356.383	0.6449682	0	-0.015997756	0	-0.045685209
cov1_2_3_5_6_7_8_9_11	50.0368	-356.387	0.6315625	0	0.020349865	0	0.18795412
cov1_2_3_5_6_8_9_10_11	50.0646	-356.4	0.6300645	0	0.029839921	0	0.188938
cov1_4_5_6_7_8_9_10_11	50.0722	-356.404	0.6435812	0	-0.034479298	0	0
cov1_2_3_5_6_7_9_10_11	50.0913	-356.414	0.6300272	0	0.025182306	0	0.18838046
cov1_2_4_5_6_7_9_10_11	50.146	-356.441	0.644825	0	0.000983776	0	-0.044138474
cov1_2_4_5_6_7_8_9_11	50.1831	-356.46	0.6471989	0	-0.00494634	0	-0.045448003
cov1_2_3_6_7_8_9_10_11	50.2259	-356.481	0.6333105	0	-0.090226585	0	0.17720203
cov1_2_4_5_6_8_9_10_11	50.231	-356.484	0.6452679	0	0.005203323	0	-0.045353034
cov1_2_3_5_6_7_8_10_11	50.2948	-356.516	0.6327216	0	0.031324407	0	0.18990009
cov1_2_4_5_6_7_8_10_11	50.4649	-356.601	0.6483741	0	0.00572454	0	-0.044197866
cov1_2_4_6_7_8_9_10_11	50.6053	-356.671	0.6441373	0	-0.14727607	0	0.019451644
cov1_3_5_6_7_8_9_10_11	51.4242	-357.08	0.635315	0	0.11651099	0	0
cov1_2_5_6_7_8_9_10_11	52.6686	-357.702	0.6419251	0	-0.1948079	0	0.16311937
cov1_2_3_4_5_7_8_9_10_11	54.2724	-355.107	0.6324996	0	0.20150957	0	-0.014734677
cov1_3_4_5_6_7_8_9_10_11	54.2775	-355.11	0.6320577	0	0.1937485	0	0
cov1_2_3_4_5_6_7_8_9_10	54.2776	-355.11	0.6331776	0	0.20832308	0	-0.013830689
cov1_2_3_4_5_6_7_8_9_11	54.376	-355.159	0.6340439	0	0.20349285	0	-0.014892645
cov1_2_3_4_5_6_8_9_10_11	54.3824	-355.162	0.6324753	0	0.21262449	0	-0.014740525
cov1_2_3_4_5_6_7_9_10_11	54.4258	-355.184	0.6324528	0	0.20837945	0	-0.014539502
cov1_2_3_4_5_6_7_8_10_11	54.6221	-355.282	0.6351682	0	0.21469552	0	-0.01353588
cov2_3_4_5_6_7_8_9_10_11	54.6256	-355.284	0.631141	0	0	0	0.032028398
cov1_2_3_4_6_7_8_9_10_11	56.2825	-356.112	0.6370969	0	-0.12353203	0	0.079821202
cov1_2_3_5_6_7_8_9_10_11	56.7254	-356.334	0.630069	0	0.020483256	0	0.18802976
cov1_2_4_5_6_7_8_9_10_11	56.8158	-356.379	0.6449702	0	-0.00187449	0	-0.04454872
cov1_2_3_4_5_6_7_8_9_10_11	61.0668	-355.107	0.6324831	0	0.20350973	0	-0.014576554

SE	bsnp_rs7817754	SE	bsnp_rs2047962	SE	bsnp_rs6469668	SE	bsnp_rs6469667	SE	bsnp_rs7832958
0	0	0	0	0	0	0	0	0	0
0	0	0	0.1605498	0	0	0	0	0	0
0	0	0	0	0	0	0	0	0	-0.26492087
0	-0.19722774	0	0	0	0	0	0	0	0
0	0	0	0	0	0	0	0	0	0
0	0	0	0	0	0	0	0	0	0
0	0	0	0	0	0	0	0	0	0
0	0	0	0	0	0	0	0	0	0
0	-0.26790192	0	0	0	0	0	0	0	0
0	-0.266837	0	0	0	0	0	0	0	0
0	0	0	0	0	0	0	0.092541539	0	0
0	0	0	0	0	0	0	0	0	0
0	0	0	0	0	0.076118222	0	0	0	0
0	-0.22962797	0	0	0	0	0	0	0	0
0	0	0	0	0	0	0	0	0	0
0	0	0	0.14702117	0	0	0	0	0	-0.23807745
0	0	0	0.14742472	0	0	0	0	0	0
0	0	0	0.39579418	0	-0.26599209	0	0	0	0
0	-0.16764464	0	0.14050612	0	0	0	0	0	0
0	0	0	0	0	0	0	0	0	-0.25331183
0	0	0	0	0	0	0	0	0	0
0	0	0	0	0	0	0	0	0	-0.25778657
0	0	0	0.14179356	0	0	0	0	0	0
0	0	0	0	0	0	0	0	0	0
0	0	0	0	0	0	0	0	0	-0.25426799
0	0	0	0.14444295	0	0	0	0	0	0
0	0	0	0	0	0	0	0	0	0
0	-0.2227934	0	0	0	0	0	0	0	0
0	-0.21971385	0	0	0	0	0	0.11701076	0	0
0	0	0	0	0	0	0	0	0	-0.25942765
0	0	0	0	0	0	0	0.086607661	0	-0.25781237
0	-0.22703969	0	0	0	0	0	0	0	0
0	0	0	0	0	0	0	0	0	0
0	0	0	0	0	0	0	0.08624908	0	0
0	0	0	0	0	0.07724819	0	0	0	-0.26493758
0	0	0	0.19604188	0	0	0	0	0	0
0	0	0	0	0	0.077334473	0	0	0	0
0	0	0	0.2039757	0	0	0	0	0	0
0	0	0	0	0	0	0	0	0	-0.28222212
0	-0.11753947	0	0	0	0	0	0	0	-0.15877921
0	-0.12199552	0	0	0	0	0	0	0	0
0	-0.18925013	0	0	0	0.063398271	0	0	0	0
0	0	0	0.18872487	0	0	0	-0.039565673	0	0
0	0	0	0	0	0	0	0	0	0
0	0	0	0.15588512	0	0	0	0	0	0

0	0	0	0	0	0	0	0	0	0
0	-0.2383023	0	0.10540645	0	0	0	0	0	0
0	0	0	0	0	0	0	0	0	0
0	0	0	0	0	0	0	0	0	-0.6273492
0	-0.23650083	0	0.10898804	0	0	0	0	0	0
0	0	0	0	0	0.055400239	0	0	0	0
0	-0.27292914	0	0	0	0	0	0	0	0
0	0	0	0	0	0	0	0	0	0
0	0	0	0	0	0	0	0	0	0
0	0	0	0	0	0	0	0.039515821	0	0
0	-0.27222972	0	0	0	0	0	0	0	0
0	0	0	0	0	0.058645157	0	0	0	0
0	0	0	0	0	0	0	0	0	0
0	0	0	0	0	0	0	0	0	0
0	0	0	0	0	0	0	0	0	0
0	0	0	0	0	0	0	0.045369012	0	0
0	0	0	0	0	0	0	0	0	0
0	0	0	0	0	-0.046330714	0	0	0	0
0	0	0	0	0	0	0	-0.037177561	0	0
0	0	0	0	0	0	0	0	0	0
0	-0.2704059	0	0	0	0	0	0	0	0
0	-0.26907949	0	0	0	0	0	0.045152019	0	0
0	-0.27697784	0	0	0	0	0	0	0	0
0	-0.26231189	0	0	0	0.030141877	0	0	0	0
0	-0.265821	0	0	0	0	0	0	0	0
0	-0.26100579	0	0	0	0	0	0	0	-0.012204343
0	-0.26574776	0	0	0	0	0	0	0	0
0	-0.26972288	0	0	0	0	0	0	0	0
0	-0.26820219	0	0	0	0	0	0.051057686	0	0
0	0	0	0.38302198	0	-0.27385034	0	0	0	0
0	-0.27710305	0	0	0	0	0	0	0	0
0	-0.15270238	0	0.35783227	0	-0.24365075	0	0	0	0
0	0	0	0.1291841	0	0	0	0	0	-0.23143446
0	-0.26059283	0	0	0	0.034236325	0	0	0	0
0	0	0	0.34398009	0	-0.22036686	0	0	0	-0.20194794
0	0	0	0.12962314	0	0	0	0	0	0
0	-0.26837481	0	0	0	-0.13947555	0	0	0	0
0	0	0	0.38372363	0	-0.27139076	0	0	0	0
0	0	0	0.34539772	0	-0.22153349	0	0	0	0
0	-0.2576239	0	0	0	0	0	0	0	-0.016252477
0	-0.26242437	0	0	0	0	0	0	0	0
0	0	0	0.13176262	0	0	0	0	0	-0.2317221
0	0	0	0.13220063	0	0	0	0	0	0
0	0	0	0	0	0	0	0	0	0
0	0	0	0	0	0	0	0.13023119	0	0
0	0	0	0	0	0	0	0.055187398	0	0
0	0	0	0	0	0.005047628	0	0.088452253	0	0

0	0	0	0	0	-0.010604302	0	0	0	0
0	0	0	0.27147802	0	0	0	0	0	0
0	0	0	0	0	0.08396188	0	0	0	0
0	-0.18109685	0	0	0	0	0	0	0	-0.092442385
0	-0.18536163	0	0	0	0	0	0	0	0
0	0	0	0.26922522	0	0	0	0	0	0
0	-0.22756772	0	0	0	0	0	0	0	0
0	-0.22331055	0	0	0	0	0	0	0	0
0	-0.22888019	0	0	0	0	0	-0.027308301	0	0
0	0	0	0.24181253	0	0	0	-0.15076878	0	0
0	-0.22116801	0	0.022419718	0	0	0	0	0	0
0	0	0	0.20032214	0	0	0	0	0	0
0	-0.0865851	0	0.14105243	0	0	0	0	0	-0.16103974
0	0	0	0.40175571	0	-0.37388289	0	0	0	0
0	0	0	0	0	0	0	0	0	-0.25170219
0	-0.090930858	0	0.14107728	0	0	0	0	0	0
0	0	0	0	0	0	0	0	0	0
0	0	0	0.39430276	0	-0.33199289	0	0.083760047	0	0
0	0	0	0.24060694	0	0	0	-0.14394989	0	0
0	0	0	0.18106896	0	0	0	0	0	-0.23477542
0	-0.28509489	0	0	0	-0.20254247	0	0	0	0
0	0	0	0	0	0	0	0	0	-0.25238151
0	0	0	0.14706778	0	0	0	0	0	-0.62714506
0	0	0	0.20159384	0	0	0	0	0	0
0	0	0	0.17014899	0	0	0	-0.032288621	0	-0.23658173
0	0	0	0.16167624	0	0	0	0	0	-0.22889614
0	0	0	0.1817334	0	0	0	0	0	0
0	0	0	0.12554711	0	0	0	0	0	-0.24049504
0	0	0	0	0	0	0	0	0	0
0	0	0	0.17131771	0	0	0	-0.033383929	0	0
0	0	0	0.16268156	0	0	0	0	0	0
0	0	0	0.12649689	0	0	0	0	0	0
0	0	0	0.37072326	0	-0.27373746	0	0	0	0
0	0	0	0	0	0.057610266	0	0	0	-0.25415992
0	0	0	0.39642901	0	-0.27055138	0	0	0	0
0	0	0	0	0	0.057704979	0	0	0	0
0	-0.17847792	0	0.1175735	0	0	0	0.030539705	0	0
0	0	0	0.22931249	0	0	0	0	0	0
0	-0.17754735	0	0.12017516	0	0	0	0	0	0
0	-0.16754242	0	0.1406393	0	0	0	0	0	0
0	0	0	0	0	0.060683103	0	0	0	-0.25502716
0	0	0	0	0	0	0	0	0	-0.25356194
0	0	0	0	0	0	0	0	0	-0.25246848
0	0	0	0	0	0	0	0.036099074	0	-0.25182105
0	0	0	0	0	0.060778294	0	0	0	0
0	0	0	0	0	0	0	0	0	-0.6286062
0	0	0	0	0	0	0	0	0	0

0	0	0	0	0	0	0	0	0	-0.26109372
0	0	0	0	0	0	0	0	0	-0.25871789
0	0	0	0.22713246	0	0	0	0	0	0
0	0	0	0	0	0	0	0	0	0
0	0	0	0	0	0	0	0.03569556	0	0
0	0	0	0	0	0	0	-0.045768271	0	-0.25934791
0	0	0	0	0	0	0	0	0	-0.62738972
0	0	0	0	0	0	0	0	0	0
0	0	0	0	0	0	0	0	0	0
0	0	0	0	0	-0.038209816	0	0	0	-0.25620949
0	-0.24825765	0	0	0	-0.10231425	0	0.20248246	0	0
0	0	0	0	0	0	0	0	0	-0.24765551
0	0	0	0	0	0	0	0	0	-0.25318715
0	0	0	0.13966088	0	0	0	0	0	0
0	0	0	0	0	0	0	0.04177373	0	-0.25244208
0	0	0	0	0	0	0	-0.046958963	0	0
0	0	0	0	0	0	0	0	0	0
0	0	0	0	0	-0.038167937	0	0	0	0
0	0	0	0	0	0	0	0	0	-0.26325719
0	-0.16621282	0	0	0	0	0	0	0	-0.10770564
0	0	0	0	0	0	0	0	0	-0.6285863
0	0	0	0	0	0	0	0	0	0
0	-0.1705805	0	0	0	0	0	0	0	0
0	0	0	0	0	0	0	0.04137243	0	0
0	-0.1629644	0	0	0	0	0	0.10785632	0	-0.10877055
0	-0.2228268	0	0.3223987	0	-0.24353084	0	0	0	0
0	-0.16789035	0	0	0	0	0	0.10845599	0	0
0	0	0	0	0	0	0	0	0	0
0	-0.22248881	0	0	0	0	0	0.048729571	0	0
0	-0.22112076	0	0	0	0	0	0	0	0
0	-0.22017281	0	0	0	0	0	0.11539586	0	0
0	-0.22089888	0	0.323676	0	-0.24076522	0	0	0	0
0	-0.1499818	0	0	0	0	0	0	0	-0.15122116
0	-0.15447251	0	0	0	0	0	0	0	0
0	-0.10111594	0	0	0	0.070053762	0	0	0	-0.17362301
0	-0.1056927	0	0	0	0.069772976	0	0	0	0
0	0	0	0	0	0	0	0	0	-0.62735414
0	0	0	0	0	0	0	0.087555683	0	-0.62873176
0	0	0	0	0	0	0	0.039139795	0	-0.25848378
0	0	0	0	0	0.007354727	0	0	0	-0.25982586
0	0	0	0	0	0.021739391	0	0.068935517	0	-0.25924181
0	0	0	0	0	0	0	0	0	-0.25886751
0	0	0	0	0	0	0	0.08248407	0	-0.25956708
0	-0.2253326	0	0	0	0.004575271	0	0	0	0
0	0	0	0	0	0	0	0.035801553	0	0
0	0	0	0	0	0.007258572	0	0	0	0
0	0	0	0	0	0	0	0	0	0

0	0	0	0	0	0.022832309	0	0.06769145	0	0
0	0	0	0.16031376	0	0	0	0	0	0
0	0	0	0	0	0.077283415	0	0	0	-0.6273491
0	0	0	0	0	0	0	0.082640234	0	0
0	0	0	0	0	0	0	0	0	0
0	-0.2461352	0	0.34934319	0	-0.49250579	0	0	0	0
0	0	0	0	0	0.058717901	0	0	0	-0.27203695
0	0	0	0.17321267	0	0	0	0	0	0
0	0	0	0.20258018	0	0	0	0	0	0
0	0	0	0.19345304	0	0	0	0.005948648	0	0
0	0	0	0	0	0.059527197	0	0	0	0
0	0	0	0	0	0	0	0	0	0
0	0	0	0	0	0	0	0	0	-0.62863436
0	0	0	0	0	0	0	0	0	0
0	-0.11834617	0	0	0	0	0	0	0	-0.62736896
0	-0.211286	0	0.18192326	0	0	0	0	0	0
0	0	0	0.20279454	0	0	0	0.026838941	0	0
0	0	0	0.16619123	0	0	0	-0.064412848	0	0
0	0	0	0	0	0	0	-0.070241075	0	0
0	-0.21915768	0	0.16496656	0	0	0	-0.085554146	0	0
0	0	0	0	0	0	0	0	0	0
0	0	0	0	0	0	0	0	0	0
0	-0.29559962	0	0	0	-0.10002642	0	0	0	0
0	0	0	0	0	0	0	-0.066355341	0	0
0	0	0	0	0	-0.01365703	0	0	0	0
0	-0.21144728	0	0.18014626	0	0	0	0	0	0
0	-0.22167992	0	0.12950474	0	0	0	0	0	0
0	0	0	0.33360948	0	-0.22969357	0	0	0	-0.19329936
0	-0.21875027	0	0.16447941	0	0	0	-0.079262695	0	0
0	-0.2371199	0	0.10395628	0	0	0	0	0	0
0	-0.27190072	0	0	0	0	0	0	0	0
0	0	0	0	0	0.098684094	0	0	0	0
0	-0.21972694	0	0.10646001	0	0	0	0	0	-0.032280222
0	-0.22459232	0	0.10619568	0	0	0	0	0	0
0	-0.24191368	0	0.094985849	0	0	0	0	0	0
0	0	0	0	0	-0.015218083	0	0	0	0
0	-0.29477543	0	0	0	-0.10054357	0	0	0	0
0	0	0	0.33511346	0	-0.23092847	0	0	0	0
0	-0.27369876	0	0	0	0	0	-0.07485717	0	0
0	0	0	0	0	0.12524036	0	0	0	0
0	0	0	0	0	0.05252724	0	0	0	0
0	-0.22112092	0	0.13128904	0	0	0	0	0	0
0	0	0	0.33419714	0	-0.22724945	0	0	0	-0.19411232
0	-0.20759877	0	0.34174196	0	-0.37771489	0	0.18033195	0	0
0	0	0	0.33569499	0	-0.22847845	0	0	0	0
0	-0.27122017	0	0	0	0	0	0	0	0
0	-0.21564421	0	0.11010739	0	0	0	0	0	-0.036157261

0	-0.26604629	0	0	0	0	0	0	0	0
0	-0.22055528	0	0.10985706	0	0	0	0	0	0
0	-0.27108102	0	0	0	0	0	0	0	0
0	-0.24195219	0	0.093210739	0	0	0	0	0	0
0	0	0	0	0	0.084612684	0	-0.038504144	0	0
0	0	0	0	0	0.099791839	0	0	0	0
0	-0.27882096	0	0	0	0	0	0	0	0
0	0	0	0.24576871	0	0	0	0	0	-0.21544083
0	-0.27297344	0	0	0	0	0	-0.070977293	0	0
0	-0.27424307	0	0	0	0	0	0	0	0.002304578
0	0	0	0	0	0	0	0	0	0
0	0	0	0	0	0	0	0.037670668	0	0
0	0	0	0	0	0	0	0	0	0
0	0	0	0	0	0	0	0.076872241	0	0
0	0	0	0.24651361	0	0	0	0	0	0
0	0	0	0	0	0.11696489	0	0	0	0
0	-0.26565409	0	0	0	0	0	0	0	0
0	0	0	0	0	-0.01927034	0	0	0	0
0	0	0	0	0	0	0	0	0	0
0	0	0	0	0	0	0	0.007551518	0	0
0	0	0	0	0	0	0	0	0	0
0	0	0	0	0	0.080889549	0	-0.029154356	0	0
0	-0.21582088	0	0.23596367	0	-0.26644212	0	0	0	0
0	0	0	0	0	0	0	0.025630507	0	0
0	-0.27668547	0	0	0	0	0	0	0	0
0	-0.2720573	0	0	0	0	0	0	0	-0.000302901
0	0	0	0.22108828	0	0	0	-0.1375574	0	-0.22171348
0	0	0	0	0	0	0	0	0	0
0	0	0	0.24339523	0	0	0	0	0	-0.21659206
0	0	0	0	0	0	0	0.082203885	0	0
0	0	0	0.22228168	0	0	0	-0.13879338	0	0
0	0	0	0.24413705	0	0	0	0	0	0
0	0	0	0	0	0	0	0.027188341	0	0
0	0	0	0	0	-0.017410037	0	0	0	0
0	0	0	0	0	0	0	0.027778978	0	0
0	0	0	0	0	-0.040226461	0	-0.013377498	0	0
0	0	0	0.21977501	0	0	0	-0.13084182	0	-0.2225154
0	0	0	0.22098739	0	0	0	-0.13208861	0	0
0	-0.2857476	0	0	0	-0.065133739	0	0	0	0
0	-0.2684165	0	0	0	0	0	0	0	0
0	-0.26709553	0	0	0	0	0	0.043762343	0	0
0	-0.18961773	0	0.35815554	0	-0.30367218	0	0	0	0
0	-0.27204481	0	0	0	0	0	0	0	0
0	-0.26767508	0	0	0	0	0	0	0	-0.004807134
0	-0.27031602	0	0	0	0	0	0.004284922	0	0
0	-0.2723321	0	0	0	0	0	0	0	0
0	-0.27459394	0	0	0	0	0	0	0	0

0	0	0	0.16865206	0	0	0	0	0	-0.20359849
0	-0.27246384	0	0	0	0	0	0.033767312	0	0
0	-0.27260419	0	0	0	-0.016835692	0	0.060729691	0	0
0	-0.26573987	0	0	0	0	0	0.044990349	0	-0.005895063
0	-0.27069491	0	0	0	0	0	0.045233999	0	0
0	-0.2607678	0	0	0	0.028125695	0	0	0	0
0	-0.2669658	0	0	0	0	0	0	0	-0.017846411
0	-0.27377798	0	0	0	0.008828416	0	0	0	0
0	-0.27171266	0	0	0	0	0	0	0	0
0	0	0	0.16969736	0	0	0	0	0	0
0	-0.26208321	0	0	0	0	0	0	0	-0.62666725
0	-0.095613065	0	0.34227816	0	-0.22581521	0	0	0	-0.11608725
0	-0.24816995	0	0	0	0.031690953	0	0	0	-0.024491706
0	-0.25318212	0	0	0	0.031149352	0	0	0	0
0	0	0	0.42899662	0	-0.26044316	0	0	0	0
0	-0.25630374	0	0	0	0	0	0	0	-0.016787422
0	0	0	0.35093232	0	-0.31438574	0	0	0	-0.19415679
0	-0.26109603	0	0	0	0	0	0	0	0
0	-0.28676813	0	0	0	-0.073019873	0	0	0	0
0	-0.09968874	0	0.34339105	0	-0.22704906	0	0	0	0
0	0	0	0.19433773	0	0	0	0	0	-0.22189828
0	0	0	0.38325195	0	-0.27688653	0	0	0	0
0	0	0	0.37715065	0	-0.23981005	0	0	0	0
0	-0.29435274	0	0	0	-0.21675183	0	0	0	0
0	0	0	0.35237654	0	-0.31585534	0	0	0	0
0	0	0	0.16955882	0	0	0	0	0	-0.2053087
0	0	0	0.19538417	0	0	0	0	0	0
0	-0.2714644	0	0	0	0	0	0	0	0
0	-0.26545255	0	0	0	0	0	0	0	-0.007492525
0	0	0	0.34429541	0	-0.27685334	0	0.069838403	0	-0.19610446
0	-0.26959806	0	0	0	0	0	0.006025319	0	0
0	-0.27015085	0	0	0	0	0	0	0	0
0	-0.27186476	0	0	0	0	0	0.038825938	0	0
0	-0.2722095	0	0	0	-0.019379482	0	0.068973581	0	0
0	0	0	0.17060519	0	0	0	0	0	0
0	-0.26321538	0	0	0	0	0	0.050809255	0	-0.008772185
0	0	0	0.38247776	0	-0.24905853	0	-0.032074129	0	0
0	-0.26825147	0	0	0	0	0	0.051060331	0	0
0	0	0	0.3798144	0	-0.23958494	0	0	0	0
0	0	0	0.12689689	0	0	0	0	0	-0.23200245
0	0	0	0.12918949	0	0	0	0	0	-0.62729647
0	-0.2814726	0	0	0	-0.17972375	0	0.079675326	0	0
0	0	0	0.34583621	0	-0.27747358	0	0.069032224	0	0
0	0	0	0.31013897	0	-0.22961886	0	0	0	-0.20525915
0	-0.2646524	0	0	0	0	0	0	0	-0.022158942
0	-0.27332529	0	0	0	0.010539308	0	0	0	0
0	-0.26946057	0	0	0	0	0	0	0	0

0	0	0	0.19180479	0	0	0	0	0	-0.22297861
0	0	0	0.42512476	0	-0.25903226	0	0	0	0
0	0	0	0.34434217	0	-0.22064473	0	0	0	-0.62705089
0	0	0	0.34551059	0	-0.24476576	0	0	0	-0.21064363
0	0	0	0.12733807	0	0	0	0	0	0
0	-0.2432765	0	0	0	0.036065791	0	0	0	-0.029911388
0	-0.2483495	0	0	0	0.035539496	0	0	0	0
0	0	0	0.37838157	0	-0.23995238	0	0	0	0
0	0	0	0.31190778	0	-0.23071719	0	0	0	0
0	-0.25874095	0	0	0	0	0	0	0	-0.62667787
0	0	0	0.1928509	0	0	0	0	0	0
0	0	0	0.34691843	0	-0.24530279	0	0	0	0
0	-0.27812821	0	0	0	-0.15348355	0	0	0	0
0	0	0	0.13176825	0	0	0	0	0	-0.62728691
0	-0.25953759	0	0	0	-0.13585209	0	0	0	-0.014861041
0	-0.26451176	0	0	0	-0.13788566	0	0	0	0
0	0	0	0.38332888	0	-0.25362041	0	-0.022847161	0	0
0	0	0	0.38172153	0	-0.24988876	0	0	0	0
0	0	0	0	0	0	0	0.067117222	0	0
0	0	0	0	0	-0.009226999	0	0	0	0
0	0	0	0	0	0.016746791	0	0.11870062	0	0
0	0	0	0	0	-0.011937949	0	0.055688376	0	0
0	0	0	0.2700389	0	0	0	0	0	0
0	0	0	0.26931176	0	0	0	0	0	0
0	0	0	0.27340984	0	0	0	-0.028136524	0	0
0	0	0	0.27464671	0	0	0	0	0	0
0	0	0	0.24714898	0	0	0	-0.10357408	0	0
0	-0.17615387	0	0	0	0	0	0	0	-0.097648589
0	-0.18190199	0	0	0	0	0	0	0	-0.62681268
0	-0.18051886	0	0	0	0	0	0	0	0
0	-0.15143971	0	0.050771667	0	0	0	0	0	-0.11266586
0	0	0	0.26775535	0	0	0	0	0	0
0	0	0	0	0	0	0	0	0	-0.2523184
0	-0.1782779	0	0	0	0	0	-0.032691979	0	-0.096093807
0	-0.15706268	0	0.048615606	0	0	0	0	0	0
0	-0.17866534	0	0	0	0	0	0	0	-0.087405622
0	-0.18239836	0	0	0	0	0	-0.032686848	0	0
0	-0.18282604	0	0	0	0	0	0	0	0
0	0	0	0	0	0	0	0	0	0
0	0	0	0.27101409	0	0	0	-0.025800516	0	0
0	-0.21331122	0	0.036827875	0	0	0	0	0	0
0	0	0	0.27080235	0	0	0	0	0	0
0	0	0	0	0	0	0	-0.076716941	0	-0.25365378
0	0	0	0.23949073	0	0	0	-0.15028987	0	0
0	-0.22478517	0	0	0	0	0	0	0	0
0	-0.2274391	0	0	0	0	0	0.020215091	0	0
0	-0.21109227	0	0.030374662	0	0	0	0	0	0

0	0	0	0.26430224	0	0	0	-0.13416937	0	0
0	-0.22393131	0	0	0	0	0	-0.01316784	0	0
0	0	0	0	0	0	0	0	0	-0.25304219
0	-0.21750627	0	0.029724765	0	0	0	-0.032587076	0	0
0	0	0	0.24581372	0	0	0	-0.097402857	0	0
0	0	0	0.19842089	0	0	0	0	0	0
0	0	0	0.23874937	0	0	0	0	0	0
0	0	0	0	0	0	0	0	0	-0.25194443
0	0	0	0	0	0	0	-0.077903499	0	0
0	0	0	0.39866412	0	-0.3700543	0	0	0	0
0	0	0	0.45040987	0	-0.41590555	0	0	0	0
0	-0.087481644	0	0.14097423	0	0	0	0	0	-0.6270906
0	0	0	0.14285726	0	0	0	0	0	-0.23960518
0	0	0	0	0	0	0	0	0	-0.23870107
0	0	0	0	0	0	0	0	0	-0.62857395
0	0	0	0	0	0	0	0	0	0
0	0	0	0	0	0	0	-0.073004462	0	-0.25428867
0	0	0	0	0	0	0	0	0	0
0	0	0	0.1438035	0	0	0	0	0	0
0	-0.079111297	0	0.15060133	0	0	0	0	0	-0.1668258
0	-0.083978407	0	0.14414909	0	0	0	0	0	-0.16153466
0	-0.085923633	0	0.14188185	0	0	0	-0.001097907	0	-0.16156953
0	0	0	0.40082472	0	-0.37336211	0	0.014625505	0	0
0	0	0	0	0	0	0	0	0	0
0	0	0	0	0	-0.007386162	0	0	0	-0.25148434
0	-0.08479117	0	0.14896212	0	0	0	0	0	0
0	-0.088413215	0	0.1440664	0	0	0	0	0	0
0	-0.090919015	0	0.14109194	0	0	0	-1.93E-05	0	0
0	0	0	0.13662419	0	0	0	-0.068010208	0	-0.24147733
0	0	0	0	0	0	0	-0.074189798	0	0
0	0	0	0	0	0	0	0	0	-0.23991821
0	0	0	0.18107334	0	0	0	0	0	-0.62715993
0	0	0	0.3908574	0	-0.32252731	0	0.10071584	0	0
0	0	0	0	0	0	0	0	0	-0.62859717
0	0	0	0.26085579	0	0	0	-0.12906346	0	0
0	-0.28518016	0	0	0	-0.20386733	0	0.055658773	0	0
0	0	0	0.23618843	0	0	0	0	0	0
0	0	0	0	0	-0.007385082	0	0	0	0
0	0	0	0.16941186	0	0	0	-0.031065388	0	-0.62713314
0	0	0	0.40441678	0	-0.33645372	0	0.093434359	0	0
0	0	0	0.12963984	0	0	0	0	0	-0.22744141
0	-0.31705258	0	0	0	-0.22763323	0	0	0	0
0	0	0	0.13769338	0	0	0	-0.069218871	0	0
0	0	0	0.16179699	0	0	0	0	0	-0.62709496
0	-0.31009537	0	0	0	-0.22212886	0	0	0	0.035933819
0	-0.28740379	0	0	0	-0.20432067	0	0	0	0
0	0	0	0.18042504	0	0	0	0.01524073	0	-0.23448618

0	0	0	0.12550182	0	0	0	0	0	-0.62721783
0	0	0	0.18107447	0	0	0	0	0	-0.23408808
0	0	0	0	0	0	0	0	0	0
0	0	0	0	0	-0.008994244	0	0	0	-0.25212006
0	0	0	0	0	0.14190133	0	0	0	-0.2577684
0	0	0	0.17139458	0	0	0	-0.023354535	0	-0.23220811
0	0	0	0.13065329	0	0	0	0	0	0
0	0	0	0.18124005	0	0	0	0.012291562	0	0
0	0	0	0.18175803	0	0	0	0	0	0
0	0	0	0	0	0.054705712	0	0	0	-0.25433961
0	0	0	0	0	-0.008996654	0	0	0	0
0	0	0	0.17262144	0	0	0	-0.023940655	0	0
0	0	0	0	0	0.14158528	0	0	0	0
0	0	0	0	0	0.10139357	0	-0.057574326	0	-0.25724398
0	0	0	0	0	0.057865302	0	0	0	-0.62858485
0	0	0	0	0	0.054800021	0	0	0	0
0	0	0	0	0	0.07328172	0	0	0	-0.24723771
0	0	0	0.36641534	0	-0.26750827	0	0	0	0
0	0	0	0	0	0.10269846	0	-0.059169783	0	0
0	0	0	0.22619508	0	0	0	0	0	0
0	0	0	0	0	0.13381137	0	0	0	-0.25820393
0	0	0	0	0	0.074127472	0	0	0	0
0	-0.17358362	0	0.12084122	0	0	0	0.041311319	0	0
0	0	0	0	0	0	0	0	0	-0.25275852
0	0	0	0	0	0	0	0.03421427	0	-0.25214004
0	-0.17797243	0	0.11853733	0	0	0	0.033904525	0	0
0	0	0	0	0	0.060952366	0	0	0	-0.62869954
0	-0.17404386	0	0.12136954	0	0	0	0	0	0
0	0	0	0	0	0	0	0	0	-0.62852862
0	0	0	0	0	0.09776494	0	-0.048486433	0	-0.25765805
0	0	0	0	0	0.13349087	0	0	0	0
0	0	0	0	0	0	0	0	0	-0.26068945
0	0	0	0	0	0	0	0	0	-0.62858854
0	0	0	0	0	0	0	0.03707055	0	-0.6287684
0	0	0	0	0	0.074010966	0	0	0	-0.2492004
0	0	0	0	0	0	0	0	0	0
0	0	0	0	0	0	0	0.033805414	0	0
0	0	0	0	0	0.099079731	0	-0.050085356	0	0
0	0	0	0	0	0	0	0	0	-0.62865192
0	0	0	0	0	0	0	0	0	-0.62745567
0	0	0	0	0	0	0	0	0	0
0	0	0	0	0	0	0	0.011556284	0	-0.25222744
0	0	0	0	0	0	0	0	0	-0.25264553
0	0	0	0	0	0.074860392	0	0	0	0
0	0	0	0	0	0	0	0.032480898	0	-0.25337221
0	0	0	0	0	0	0	0	0	-0.25594423
0	0	0	0	0	0	0	0.011768491	0	-0.25845391

0	0	0	0	0	-0.000109105	0	0	0	-0.25871208
0	0	0	0	0	0	0	-0.044161442	0	-0.62727631
0	0	0	0	0	-0.038363359	0	0	0	-0.62745046
0	-0.24916298	0	0.32599778	0	-0.35440531	0	0	0	0
0	0	0	0	0	0	0	0	0	-0.62733253
0	0	0	0	0	0	0	0.008249092	0	0
0	0	0	0	0	0	0	0	0	0
0	0	0	0	0	0	0	0	0	-0.62858493
0	0	0	0	0	0	0	0.042751086	0	-0.62948806
0	0	0	0	0	0	0	0.032605862	0	0
0	-0.21284224	0	0	0	-0.083387797	0	0.18184973	0	-0.057729718
0	-0.25727932	0	0	0	-0.11281478	0	0.18980604	0	0
0	0	0	0	0	-0.028827552	0	0	0	-0.24914104
0	-0.22004699	0	0	0	-0.086990619	0	0.18575837	0	0
0	0	0	0	0	0	0	-0.033303923	0	-0.2524931
0	0	0	0	0	-0.023533461	0	-0.031752975	0	-0.25788619
0	0	0	0	0	0	0	0	0	0
0	0	0	0	0	0	0	0.008441823	0	0
0	0	0	0	0	-0.000194028	0	0	0	0
0	-0.16704068	0	0	0	0	0	0	0	-0.62686569
0	0	0	0	0	0	0	0	0	-0.6285804
0	0	0	0	0	0	0	0.01360888	0	-0.2528993
0	0	0	0	0	0	0	0	0	-0.25347348
0	-0.16433449	0	0	0	0	0	0.10894471	0	-0.62727802
0	0	0	0	0	0	0	0.037440059	0	-0.25429259
0	0	0	0	0	-0.028320082	0	0	0	0
0	0	0	0	0	0	0	-0.034002992	0	0
0	0	0	0	0	-0.02271207	0	-0.033443685	0	0
0	-0.16682704	0	0	0	0	0	0.044097331	0	-0.10609388
0	-0.22112612	0	0.322854	0	-0.24597404	0	0	0	0
0	-0.23661049	0	0.32379192	0	-0.27035512	0	0	0	0
0	-0.23212175	0	0.32100504	0	-0.28297078	0	0.052450754	0	0
0	-0.24990023	0	0.32766359	0	-0.36391125	0	0	0	0
0	-0.16633845	0	0	0	0	0	0	0	-0.10854447
0	-0.17142525	0	0	0	0	0	0.043189388	0	0
0	-0.23536896	0	0.2909045	0	-0.2498523	0	0	0	0
0	0	0	0	0	0	0	0.010300344	0	0
0	0	0	0	0	0	0	0	0	0
0	0	0	0	0	0	0	0.037570177	0	0
0	-0.17067138	0	0	0	0	0	0	0	0
0	-0.16389678	0	0	0	0	0	0.098170941	0	-0.11209431
0	-0.24040468	0	0.32558427	0	-0.24823883	0	0	0	0
0	-0.23587853	0	0.32477415	0	-0.24703767	0	0	0	0.023176946
0	-0.16875277	0	0	0	0	0	0.099233635	0	0
0	-0.22018444	0	0	0	0	0	0.051125101	0	0
0	-0.23157134	0	0.32208275	0	-0.28659956	0	0.060624656	0	0
0	-0.23589795	0	0.32514487	0	-0.26994295	0	0	0	0

0	-0.23532805	0	0.28734763	0	-0.24821823	0	0	0	0
0	-0.23551796	0	0.32629927	0	-0.24460315	0	0	0	0
0	-0.230941	0	0.3254844	0	-0.24339933	0	0	0	0.017801127
0	-0.15091424	0	0	0	0	0	0	0	-0.62710589
0	-0.13945173	0	0	0	0.020501046	0	0	0	-0.15688904
0	-0.14435781	0	0	0	0.019727648	0	0	0	0
0	-0.1020793	0	0	0	0.069959395	0	0	0	-0.62723369
0	0	0	0	0	0	0	0.043475853	0	-0.62836803
0	-0.33540723	0	0.36482691	0	-0.57704432	0	0	0	0
0	0	0	0	0	0.020355435	0	0.071179224	0	-0.62884837
0	0	0	0	0	0.00704122	0	0	0	-0.62741144
0	0	0	0	0	0	0	0	0	-0.62735308
0	0	0	0	0	0	0	0.084499064	0	-0.62888311
0	-0.32944831	0	0.36381705	0	-0.57129357	0	0	0	0.12220603
0	0	0	0	0	0	0	0	0	0
0	0	0	0	0	0.006278316	0	0.038830021	0	-0.25882976
0	0	0	0	0	0	0	0.040122062	0	-0.25699556
0	0	0	0	0	0.007357531	0	0	0	-0.25926339
0	0	0	0	0	0.021314242	0	0.067612733	0	-0.25991987
0	0	0	0	0	0.10732167	0	0	0	0
0	0	0	0.16938252	0	0	0	0	0	0
0	0	0	0	0	0	0	0	0	0
0	0	0	0	0	0.058824328	0	0	0	-0.62741268
0	0	0	0	0	0	0	0.037103058	0	0
0	0	0	0	0	0.006266382	0	0.035490691	0	0
0	0	0	0	0	0.007264665	0	0	0	0
0	0	0	0.16343497	0	0	0	-0.021942268	0	0
0	0	0	0	0	0.02662972	0	0	0	0
0	0	0	0	0	0.022575953	0	0.066905293	0	0
0	0	0	0	0	0	0	-0.027133137	0	0
0	0	0	0	0	0	0	0	0	0
0	-0.24975778	0	0.34970129	0	-0.49562123	0	0	0	0
0	-0.24637562	0	0.34796884	0	-0.49189629	0	0.022153013	0	0
0	-0.24735581	0	0.34247478	0	-0.48741475	0	0	0	0
0	0	0	0.2008584	0	0	0	0.037857295	0	0
0	0	0	0.17284364	0	0	0	0.018664656	0	0
0	-0.22655457	0	0.13773158	0	0	0	0	0	0
0	0	0	0	0	0.098575154	0	0	0	0
0	0	0	0	0	0	0	0	0	0
0	0	0	0	0	0	0	0.00203491	0	0
0	0	0	0	0	0	0	-0.071421758	0	0
0	0	0	0	0	0.024090084	0	0	0	0
0	-0.17125519	0	0.19119032	0	0	0	0	0	-0.065460957
0	0	0	0	0	0	0	-0.023387144	0	0
0	-0.17701651	0	0.18987708	0	0	0	0	0	0
0	-0.20996727	0	0.18075076	0	0	0	0	0	0
0	-0.23243886	0	0.12764294	0	0	0	-0.10496134	0	0

0	-0.21122568	0	0.18376795	0	0	0	-0.026802897	0	0
0	-0.21112068	0	0.18196908	0	0	0	0	0	0
0	0	0	0	0	0.038594874	0	-0.096464763	0	0
0	-0.1842192	0	0.17177164	0	0	0	-0.092547304	0	-0.058017604
0	-0.21798376	0	0.16353296	0	0	0	-0.085545702	0	0
0	-0.18901873	0	0.17100535	0	0	0	-0.091799552	0	0
0	-0.21618883	0	0.16684477	0	0	0	-0.079005439	0	0
0	0	0	0	0	-0.018032192	0	0	0	0
0	0	0	0	0	0	0	0.004019889	0	0
0	-0.22735023	0	0.13396861	0	0	0	0	0	0
0	-0.29402661	0	0	0	-0.10286596	0	0	0	0
0	-0.33772254	0	0	0	-0.11435587	0	0	0	0
0	-0.33261784	0	0	0	-0.11258635	0	0	0	0.060034445
0	-0.17004328	0	0.18964625	0	0	0	0	0	-0.067524761
0	0	0	0	0	0.033189748	0	-0.088852879	0	0
0	-0.29407696	0	0	0	-0.092750521	0	-0.012623904	0	0
0	0	0	0.3336624	0	-0.23258813	0	0	0	-0.19340294
0	-0.29351238	0	0	0	-0.091398073	0	0	0	0
0	-0.29761611	0	0	0	-0.10329912	0	0	0	0
0	-0.17583921	0	0.18833711	0	0	0	0	0	0
0	-0.21999711	0	0.1286147	0	0	0	0	0	0
0	0	0	0	0	0.095861348	0	0	0	0
0	-0.23297863	0	0.12435437	0	0	0	-0.10012682	0	0
0	0	0	0.36909862	0	-0.22099714	0	0	0	-0.18829234
0	0	0	0.33397425	0	-0.23002599	0	0	0	-0.62719616
0	-0.22836023	0	0.1072157	0	0	0	0	0	0
0	-0.22096136	0	0.10619997	0	0	0	0	0	-0.62667542
0	-0.21140515	0	0.18186003	0	0	0	-0.024829139	0	0
0	-0.20411458	0	0.13031649	0	0	0	0	0	-0.030768321
0	0	0	0.33516548	0	-0.23382413	0	0	0	0
0	-0.21142811	0	0.18015136	0	0	0	0	0	0
0	-0.20890476	0	0.13011128	0	0	0	0	0	0
0	0	0	0.33203456	0	-0.19272471	0	-0.04685888	0	-0.19605507
0	0	0	0.32803426	0	-0.17726583	0	0	0	-0.19634072
0	-0.18227393	0	0.17149151	0	0	0	-0.086543676	0	-0.060430092
0	-0.26988855	0	0	0	0	0	0	0	0
0	-0.18713969	0	0.17073407	0	0	0	-0.085796847	0	0
0	0	0	0.37097675	0	-0.22212086	0	0	0	0
0	-0.21660402	0	0.10505894	0	0	0	0	0	-0.035579175
0	0	0	0.33269044	0	-0.21952334	0	0	0	-0.18925776
0	-0.22150792	0	0.10480841	0	0	0	0	0	0
0	-0.33508402	0	0	0	-0.11411283	0	0	0	0
0	-0.21613131	0	0.16612519	0	0	0	-0.073513238	0	0
0	-0.24087616	0	0.093097571	0	0	0	0	0	0
0	0	0	0.33342713	0	-0.19306893	0	-0.047906664	0	0
0	0	0	0	0	0.12622863	0	0	0	0
0	0	0	0.32961036	0	-0.17909551	0	0	0	0

0	-0.32993502	0	0	0	-0.11235025	0	0	0	0.056908194
0	-0.27225913	0	0	0	0	0	-0.018760682	0	0
0	-0.22890173	0	0.2774472	0	-0.35665443	0	0.1348498	0	0
0	-0.27616143	0	0	0	0	0	0	0	0
0	-0.27235605	0	0	0	0	0	0	0	0
0	-0.27149489	0	0	0	0	0	0	0	-0.00071429
0	0	0	0	0	0.10150329	0	-0.004652018	0	0
0	-0.27179011	0	0	0	0	0	-0.075593651	0	0
0	-0.22277796	0	0.10096585	0	0	0	0	0	-0.030245202
0	-0.22845323	0	0.09920948	0	0	0	0	0	0
0	0	0	0.33411748	0	-0.22003865	0	0	0	0
0	0	0	0	0	0.11922919	0	0	0	0
0	-0.29757095	0	0	0	-0.10507149	0	0	0	0
0	-0.29406369	0	0	0	-0.097098599	0	-0.005952676	0	0
0	-0.29457195	0	0	0	-0.099695476	0	0	0	0
0	0	0	0.33455783	0	-0.22757093	0	0	0	-0.62718244
0	-0.27190632	0	0	0	0	0	-0.070009564	0	0
0	-0.2779452	0	0	0	0	0	-0.074719254	0	0
0	-0.27375294	0	0	0	0	0	-0.074856189	0	9.47E-05
0	0	0	0.3648946	0	-0.21952306	0	0	0	-0.1898191
0	-0.21691407	0	0.10985681	0	0	0	0	0	-0.62668048
0	0	0	0.33291451	0	-0.19721971	0	-0.03783923	0	-0.19637059
0	0	0	0	0	0.079506913	0	-0.035465591	0	0
0	-0.2290141	0	0.10476201	0	0	0	0	0	0
0	0	0	0.32997113	0	-0.18739737	0	0	0	-0.196443
0	-0.20107764	0	0.13218763	0	0	0	0	0	-0.0349855
0	-0.21919199	0	0.34343681	0	-0.39274068	0	0.16390982	0	0
0	-0.20592295	0	0.13198803	0	0	0	0	0	0
0	0	0	0.36676756	0	-0.22064262	0	0	0	0
0	0	0	0.33351376	0	-0.21962851	0	0	0	-0.19111079
0	0	0	0	0	0.12425475	0	0.011123871	0	0
0	-0.26374853	0	0	0	0	0	0	0	0
0	0	0	0	0	0	0	0	0	0
0	0	0	0.33431621	0	-0.19756588	0	-0.038886818	0	0
0	-0.27400882	0	0	0	0	0	0	0	-0.62250512
0	0	0	0	0	0	0	0.076439731	0	0
0	0	0	0.33154398	0	-0.18922129	0	0	0	0
0	0	0	0.24338885	0	0	0	0	0	-0.21600278
0	-0.21380798	0	0.3427986	0	-0.3820182	0	0.1840205	0	0
0	0	0	0.24591892	0	0	0	0	0	-0.62730953
0	-0.2070645	0	0.34165089	0	-0.37734924	0	0.18001985	0	-0.000890106
0	0	0	0.33493805	0	-0.22013953	0	0	0	0
0	-0.27154673	0	0	0	0	0	-0.017152971	0	0
0	-0.22150364	0	0.099544721	0	0	0	0	0	-0.032244776
0	-0.27398577	0	0	0	0	0	0	0	0
0	-0.27174058	0	0	0	0	0	0	0	0
0	-0.26927482	0	0	0	0	0	0	0	-0.003409633

0	-0.27636385	0	0	0	0	0	0	0	0
0	-0.27189719	0	0	0	0	0	0	0	0.010549792
0	-0.22721576	0	0.097793181	0	0	0	0	0	0
0	0	0	0	0	0.11776066	0	0	0	0
0	-0.27469958	0	0	0	0	0	0	0	0
0	-0.2700601	0	0	0	0	0	0	0	-0.001786467
0	0	0	0	0	0.097354637	0	0.003998584	0	0
0	0	0	0.24413323	0	0	0	0	0	0
0	0	0	0.2481259	0	0	0	-0.035182635	0	-0.21584034
0	0	0	0.2386489	0	0	0	0	0	-0.21650741
0	-0.27130406	0	0	0	0	0	-0.06650432	0	0
0	0	0	0.24576548	0	0	0	0	0	-0.21544174
0	-0.27579812	0	0	0	0	0	-0.07088271	0	0
0	-0.27154255	0	0	0	0	0	-0.071013211	0	-0.002503532
0	0	0	0	0	0	0	0.03687191	0	0
0	0	0	0	0	0	0	0.026520736	0	0
0	-0.23099004	0	0.25046158	0	-0.30052771	0	0	0	0
0	0	0	0.24902651	0	0	0	-0.037969968	0	0
0	0	0	0.23971978	0	0	0	0	0	0
0	0	0	0.24652169	0	0	0	0	0	0
0	-0.27194488	0	0	0	0	0	0	0	-0.62522081
0	0	0	0	0	0.11569171	0	0.014142065	0	0
0	-0.27423888	0	0	0	0	0	0	0	0
0	0	0	0	0	0	0	0.039890405	0	0
0	-0.26954829	0	0	0	0	0	0	0	0.007154989
0	0	0	0	0	-0.027225638	0	0.021737492	0	0
0	0	0	0.21855375	0	0	0	-0.1369939	0	-0.22227996
0	0	0	0	0	-0.015949623	0	0	0	0
0	-0.19199263	0	0.23951946	0	-0.25885226	0	0	0	-0.038882885
0	0	0	0.24354891	0	0	0	0	0	-0.6273019
0	-0.1972773	0	0.23875809	0	-0.26052697	0	0	0	0
0	0	0	0.22025589	0	0	0	-0.13629356	0	-0.62681632
0	0	0	0.21974795	0	0	0	-0.13822939	0	0
0	0	0	0	0	0	0	0.039024962	0	0
0	0	0	0.22317175	0	0	0	-0.12442374	0	-0.21498028
0	0	0	0.24561736	0	0	0	-0.032925342	0	-0.2169679
0	0	0	0.22934691	0	0	0	-0.13173518	0	-0.22037543
0	0	0	0.23446424	0	0	0	0	0	-0.21792868
0	0	0	0.24339279	0	0	0	0	0	-0.21679047
0	0	0	0.22442334	0	0	0	-0.12515082	0	0
0	0	0	0.23055936	0	0	0	-0.13295114	0	0
0	0	0	0.24651837	0	0	0	-0.035725628	0	0
0	0	0	0.23553365	0	0	0	0	0	0
0	0	0	0.24414131	0	0	0	0	0	0
0	0	0	0	0	-0.017989042	0	0.027855416	0	0
0	0	0	0.21896778	0	0	0	-0.12956967	0	-0.62688486
0	0	0	0.22171616	0	0	0	-0.11856736	0	-0.21626779

0	0	0	0.22556368	0	0	0	-0.12679326	0	-0.22159866
0	-0.13779886	0	0.34512113	0	-0.28259582	0	0	0	-0.097430112
0	0	0	0.22297446	0	0	0	-0.11929687	0	0
0	-0.14218789	0	0.34623801	0	-0.28431375	0	0	0	0
0	0	0	0.22678312	0	0	0	-0.1280129	0	0
0	-0.28457288	0	0	0	-0.069225308	0	0	0	0
0	-0.2688144	0	0	0	0	0	0	0	-0.62643037
0	-0.32292402	0	0	0	-0.0938299	0	0	0	0
0	-0.31556786	0	0	0	-0.088133421	0	0	0	0.042911536
0	-0.2669481	0	0	0	0	0	0.045987079	0	-0.6268068
0	0	0	0.16663837	0	0	0	0	0	-0.20393103
0	-0.28886182	0	0	0	-0.067430608	0	0	0	0
0	-0.28576275	0	0	0	-0.066121523	0	0.011003803	0	0
0	0	0	0.16878677	0	0	0	0	0	-0.62715328
0	-0.26975781	0	0	0	0	0	0	0	0
0	-0.2631344	0	0	0	0	0	0	0	-0.009269619
0	-0.26831638	0	0	0	0	0	0.004964344	0	0
0	-0.26784834	0	0	0	0	0	0	0	0
0	0	0	0.20345403	0	0	0	0	0	-0.20235533
0	-0.27129052	0	0	0	-0.020337915	0	0.062541327	0	0
0	-0.27009663	0	0	0	0	0	0.03380642	0	0
0	-0.26805258	0	0	0	0	0	0	0	-0.62668381
0	-0.26119082	0	0	0	0	0	0.043463732	0	-0.010388534
0	-0.26619105	0	0	0	0	0	0.043713618	0	0
0	0	0	0.16768076	0	0	0	0	0	0
0	-0.26233762	0	0	0	0	0	0	0	-0.021825328
0	-0.24952833	0	0	0	0.03138583	0	0	0	-0.62654684
0	-0.26810914	0	0	0	0	0	0	0	-0.007182698
0	-0.27195496	0	0	0	0	0	0.002602325	0	0
0	-0.27274788	0	0	0	0	0	0	0	0
0	-0.26713288	0	0	0	0	0	0	0	0
0	-0.27195992	0	0	0	0.007389015	0	0	0	0
0	-0.267595	0	0	0	0	0	0.004248021	0	-0.004796112
0	-0.27227728	0	0	0	0	0	0.004321242	0	0
0	-0.27881003	0	0	0	-0.024557414	0	0.052432765	0	0
0	0	0	0.20461575	0	0	0	0	0	0
0	0	0	0.42758787	0	-0.26368525	0	0	0	0
0	-0.25741233	0	0	0	0	0	0	0	-0.6266847
0	-0.09641102	0	0.34250616	0	-0.22609152	0	0	0	-0.62691829
0	-0.2670013	0	0	0	0	0	0.032852076	0	-0.00997691
0	-0.271862	0	0	0	0	0	0.033662278	0	0
0	-0.28129091	0	0	0	-0.020996644	0	0.064983068	0	0
0	-0.27469778	0	0	0	-0.017818378	0	0.061730221	0	0.003333437
0	-0.24454201	0	0	0	0.029853457	0	0	0	-0.028042629
0	-0.24959659	0	0	0	0.029324027	0	0	0	0
0	0	0	0.35129879	0	-0.31511414	0	0	0	-0.62689381
0	0	0	0.37737036	0	-0.24286708	0	0	0	0

0	-0.26142202	0	0	0	0.01071961	0	0	0	-0.020863872
0	0	0	0.19086489	0	0	0	0	0	-0.2226353
0	0	0	0.1944553	0	0	0	0	0	-0.62729495
0	-0.26658053	0	0	0	0.009941129	0	0	0	0
0	0	0	0.41650191	0	-0.24010968	0	0	0	0
0	-0.32535297	0	0	0	-0.10267032	0	0	0	0
0	0	0	0.16969537	0	0	0	0	0	-0.62714162
0	0	0	0.44240893	0	-0.29006266	0	0	0	0
0	-0.3180214	0	0	0	-0.097020323	0	0	0	0.044931695
0	-0.34742885	0	0	0	-0.25846969	0	0	0	0
0	-0.26647203	0	0	0	0	0	0	0	-0.62663989
0	0	0	0.36891838	0	-0.3300841	0	0	0	-0.19081104
0	0	0	0.43012417	0	-0.26329488	0	0.004086099	0	0
0	0	0	0.35048167	0	-0.31417265	0	0.007813122	0	-0.19404549
0	-0.29009059	0	0	0	-0.075411799	0	0	0	0
0	0	0	0.35095822	0	-0.31440635	0	0	0	-0.1932893
0	-0.34064553	0	0	0	-0.25306377	0	0	0	0.066286816
0	0	0	0.2004829	0	0	0	0	0	-0.20422795
0	-0.28678046	0	0	0	-0.074258557	0	0.013650502	0	0
0	0	0	0.34469695	0	-0.2789845	0	0.072081674	0	-0.62715821
0	0	0	0.19191793	0	0	0	0	0	0
0	-0.26446622	0	0	0	0	0	0.051806048	0	-0.62683146
0	0	0	0.38274783	0	-0.25442063	0	-0.028971451	0	0
0	0	0	0.38039087	0	-0.24639431	0	0	0	0
0	-0.29436075	0	0	0	-0.2172755	0	0.02645383	0	0
0	0	0	0.37733081	0	-0.23367685	0	-0.01037903	0	0
0	0	0	0.37141178	0	-0.33247455	0	0	0	0
0	0	0	0.37690466	0	-0.23601851	0	0	0	0
0	-0.29565255	0	0	0	-0.21775223	0	0	0	0
0	0	0	0.12690928	0	0	0	0	0	-0.62728674
0	0	0	0.35241198	0	-0.31588458	0	0	0	0
0	0	0	0.35207518	0	-0.31571889	0	0.005392618	0	0
0	0	0	0.20164377	0	0	0	0	0	0
0	-0.26578196	0	0	0	0	0	0	0	-0.62669161
0	0	0	0.33415911	0	-0.2720841	0	0.060541653	0	-0.19789269
0	0	0	0.3447186	0	-0.27922718	0	0.063777917	0	-0.19921058
0	-0.26594603	0	0	0	0	0	0	0	-0.010020312
0	-0.27130904	0	0	0	0	0	0.004294436	0	0
0	-0.27063054	0	0	0	0	0	0	0	0
0	-0.26533625	0	0	0	0	0	0.006005761	0	-0.007473589
0	-0.27007045	0	0	0	0	0	0.006038313	0	0
0	0	0	0.31036875	0	-0.22998527	0	0	0	-0.626942
0	-0.27895995	0	0	0	-0.027720305	0	0.059896546	0	0
0	0	0	0.19192965	0	0	0	0	0	-0.62729919
0	-0.26465409	0	0	0	0	0	0.037629357	0	-0.013115587
0	-0.24456492	0	0	0	0.035809349	0	0	0	-0.62675107
0	-0.26959334	0	0	0	0	0	0.038433694	0	0

0	-0.2797754	0	0	0	-0.0229751	0	0.072659432	0	0
0	-0.27310215	0	0	0	-0.019795137	0	0.069397964	0	0.001419347
0	0	0	0.38091011	0	-0.2388399	0	-0.021000938	0	0
0	0	0	0.34589813	0	-0.24501177	0	0	0	-0.62716385
0	-0.30359987	0	0	0	-0.19307304	0	0.089555239	0	0
0	-0.29701214	0	0	0	-0.1890017	0	0.086439447	0	0.024278773
0	-0.28508592	0	0	0	-0.18369687	0	0.075068428	0	0
0	0	0	0.3357295	0	-0.27270588	0	0.059759809	0	0
0	0	0	0.34622599	0	-0.27963699	0	0.063455305	0	0
0	0	0	0.3181132	0	-0.24219909	0	0	0	-0.20978096
0	0	0	0.4134435	0	-0.24003914	0	0	0	0
0	-0.25802921	0	0	0	0.012844817	0	0	0	-0.025745859
0	0	0	0.44340443	0	-0.29948523	0	0	0	0
0	-0.26324613	0	0	0	0.012072438	0	0	0	0
0	0	0	0.42818904	0	-0.26682779	0	0.011115213	0	0
0	-0.26072485	0	0	0	-0.13666596	0	0	0	-0.62636608
0	0	0	0.31954255	0	-0.24273678	0	0	0	0
0	0	0	0.37886368	0	-0.24650853	0	0	0	0
0	0	0	0.37839835	0	-0.23883935	0	-0.001854742	0	0
0	-0.27054838	0	0	0	-0.15024904	0	0	0	-0.012527308
0	-0.27562359	0	0	0	-0.15241031	0	0	0	0
0	0	0	0.38265654	0	-0.24921794	0	-0.0180977	0	0
0	0	0	0	0	-0.010735816	0	0.067572989	0	0
0	0	0	0.2677511	0	0	0	0	0	0
0	0	0	0	0	0.1232054	0	0	0	-0.25668442
0	0	0	0.27135639	0	0	0	-0.018602285	0	0
0	0	0	0.27052608	0	0	0	0	0	0
0	0	0	0.27118974	0	0	0	-0.027067075	0	0
0	0	0	0.27154609	0	0	0	0	0	0
0	0	0	0	0	0.12290563	0	0	0	0
0	-0.13178385	0	0.073086348	0	0	0	0	0	-0.1285819
0	0	0	0.27610178	0	0	0	-0.027792309	0	0
0	0	0	0	0	0	0	0	0	-0.25256277
0	0	0	0.24476087	0	0	0	-0.10224234	0	0
0	-0.13788877	0	0.070510466	0	0	0	0	0	0
0	-0.17707335	0	0	0	0	0	0	0	-0.62668583
0	-0.15302163	0	0.049701698	0	0	0	0	0	-0.6267036
0	0	0	0	0	0.11465888	0	0	0	-0.2571262
0	-0.13983667	0	0.063781734	0	0	0	-0.045686107	0	-0.12306875
0	0	0	0	0	0	0	0	0	-0.62850367
0	-0.17930748	0	0	0	0	0	-0.030672372	0	-0.62656044
0	0	0	0.26077085	0	0	0	-0.095565417	0	0
0	-0.1794491	0	0	0	0	0	0	0	-0.62679301
0	-0.14489207	0	0.056839846	0	0	0	0	0	-0.10900776
0	0	0	0	0	0	0	0	0	0
0	-0.14533112	0	0.061607888	0	0	0	-0.045343703	0	0
0	-0.17634789	0	0	0	0	0	0.016344446	0	-0.097111875

0	-0.17600286	0	0	0	0	0	0	0	-0.096592806
0	-0.1504167	0	0.054755778	0	0	0	0	0	0
0	0	0	0	0	0	0	-0.077885377	0	-0.25391745
0	-0.18078889	0	0	0	0	0	0.015526753	0	0
0	-0.18030493	0	0	0	0	0	0	0	0
0	0	0	0	0	0.11434997	0	0	0	0
0	0	0	0.26892371	0	0	0	-0.016253749	0	0
0	0	0	0.2665984	0	0	0	0	0	0
0	0	0	0	0	0	0	-0.011527074	0	-0.25254407
0	0	0	0	0	0	0	0	0	-0.25039123
0	-0.17750006	0	0	0	0	0	-0.023894958	0	-0.091909266
0	0	0	0	0	0.055239142	0	-0.11439003	0	-0.25632506
0	-0.1815949	0	0	0	0	0	-0.023464399	0	0
0	0	0	0	0	0	0	-0.075109633	0	-0.62730175
0	0	0	0	0	0	0	-0.079071914	0	0
0	0	0	0	0	0	0	0	0	-0.23837443
0	0	0	0.26119262	0	0	0	-0.13430245	0	0
0	0	0	0	0	0	0	0	0	-0.62867613
0	-0.20970159	0	0.038120339	0	0	0	0	0	0
0	0	0	0	0	0	0	-0.014829734	0	0
0	0	0	0	0	0	0	0	0	0
0	0	0	0.23542889	0	0	0	0	0	0
0	-0.2133438	0	0.036431795	0	0	0	0.018820367	0	0
0	0	0	0	0	0	0	-0.065616883	0	-0.24758272
0	0	0	0.2721624	0	0	0	-0.02565816	0	0
0	0	0	0	0	0.056244711	0	-0.11623383	0	0
0	0	0	0	0	0	0	0	0	-0.62865496
0	-0.22435719	0	0	0	0	0	0.023323221	0	0
0	0	0	0.44261396	0	-0.40813111	0	0	0	0
0	0	0	0.1427926	0	0	0	0	0	-0.62706458
0	-0.21089142	0	0.032939105	0	0	0	-0.017732142	0	0
0	0	0	0	0	0	0	0	0	-0.62743813
0	0	0	0	0	0	0	0	0	0
0	0	0	0	0	0	0	0	0	-0.25119546
0	0	0	0	0	0	0	-0.009639643	0	-0.25323272
0	0	0	0.2571949	0	0	0	-0.09075762	0	0
0	0	0	0	0	0	0	-0.066294195	0	0
0	0	0	0	0	-0.011732782	0	0	0	-0.25159875
0	0	0	0.39703717	0	-0.36907415	0	0.023751326	0	0
0	0	0	0	0	0	0	-0.071398541	0	-0.62730792
0	0	0	0.45011314	0	-0.41604824	0	0.020851296	0	0
0	0	0	0	0	0.049901727	0	-0.10697363	0	-0.25669583
0	-0.080497945	0	0.14990291	0	0	0	0	0	-0.62720352
0	-0.085003034	0	0.14391461	0	0	0	0	0	-0.62709253
0	0	0	0.14271413	0	0	0	0	0	-0.23770305
0	-0.088149612	0	0.14012013	0	0	0	0.001209691	0	-0.62711662
0	0	0	0.1427579	0	0	0	0.005405528	0	-0.23949088

0	0	0	0	0	0.012816329	0	0	0	-0.23779334
0	0	0	0	0	-0.007361835	0	0	0	-0.62838721
0	0	0	0	0	0	0	-0.012945412	0	0
0	0	0	0	0	0	0	0	0	0
0	0	0	0	0	0	0	-0.062177069	0	-0.24838429
0	0	0	0	0	-0.011736614	0	0	0	0
0	0	0	0	0	0.050918407	0	-0.10882946	0	0
0	-0.080270517	0	0.14923387	0	0	0	0.022145382	0	-0.16541272
0	0	0	0.13629996	0	0	0	-0.066435285	0	-0.62683568
0	0	0	0.14361948	0	0	0	0	0	0
0	0	0	0	0	0	0	0	0	-0.62742581
0	0	0	0.14376168	0	0	0	0.002414562	0	0
0	-0.079126629	0	0.15059485	0	0	0	0	0	-0.16690075
0	-0.085022584	0	0.14277377	0	0	0	0.002798685	0	-0.16029791
0	0	0	0	0	0.013362944	0	0	0	0
0	-0.30979743	0	0	0	-0.22228412	0	0	0	-0.58909353
0	0	0	0.13669577	0	0	0	-0.055338275	0	-0.23448467
0	-0.086018334	0	0.14763921	0	0	0	0.020758905	0	0
0	0	0	0	0	0	0	-0.062853877	0	0
0	-0.084744836	0	0.14898188	0	0	0	0	0	0
0	-0.089964789	0	0.14203518	0	0	0	0.004131876	0	0
0	0	0	0.12959807	0	0	0	0	0	-0.62714674
0	0	0	0.18023668	0	0	0	0.019373661	0	-0.62706121
0	0	0	0	0	0.010143567	0	0	0	-0.23920209
0	-0.32065901	0	0	0	-0.23177205	0	0.05916807	0	0
0	0	0	0	0	0.13589778	0	0	0	-0.25780568
0	0	0	0.18108024	0	0	0	0	0	-0.62715578
0	-0.31309343	0	0	0	-0.22575833	0	0.057939034	0	0.04018517
0	0	0	0.39525307	0	-0.32476541	0	0.10416527	0	0
0	0	0	0	0	-0.008901275	0	0	0	-0.62862072
0	-0.28662244	0	0	0	-0.20494911	0	0.054545637	0	0
0	0	0	0.13775662	0	0	0	-0.05608252	0	0
0	0	0	0	0	0.14184931	0	0	0	-0.62833143
0	0	0	0.17072771	0	0	0	-0.021386995	0	-0.62706625
0	-0.31760028	0	0	0	-0.22803812	0	0	0	0
0	-0.31066167	0	0	0	-0.22254918	0	0	0	0.034195473
0	0	0	0.18040468	0	0	0	0.015951221	0	-0.23341939
0	0	0	0	0	0.010684472	0	0	0	0
0	0	0	0	0	0.054974412	0	0	0	-0.6283788
0	0	0	0	0	0.096289355	0	-0.054532455	0	-0.2572583
0	0	0	0	0	0.13557469	0	0	0	0
0	0	0	0	0	0.14244517	0	-0.00584774	0	-0.25791479
0	0	0	0	0	0.14190469	0	0	0	-0.25804297
0	0	0	0	0	0.09962288	0	-0.055165733	0	-0.62735605
0	0	0	0	0	0.070408779	0	0	0	-0.24741074
0	0	0	0.18120927	0	0	0	0.013290654	0	0
0	0	0	0	0	0.097595587	0	-0.056129105	0	0

0	0	0	0	0	0.073684408	0	0	0	-0.62852602
0	0	0	0	0	0.10395528	0	-0.051562833	0	-0.25377342
0	0	0	0	0	0.14244347	0	-0.00919614	0	0
0	0	0	0	0	0.14159862	0	0	0	0
0	0	0	0	0	0.071252887	0	0	0	0
0	0	0	0	0	0.13384299	0	0	0	-0.62851969
0	0	0	0	0	0.10543318	0	-0.052679452	0	0
0	0	0	0	0	0	0	0	0	-0.62864104
0	0	0	0	0	0	0	0.035038193	0	-0.62763226
0	0	0	0	0	0.095984013	0	-0.046063956	0	-0.62737962
0	0	0	0	0	0.13409614	0	-0.002929659	0	-0.25827552
0	0	0	0	0	0.13382885	0	0	0	-0.25864919
0	0	0	0	0	0	0	0	0	-0.62854279
0	0	0	0	0	0.074418793	0	0	0	-0.62856293
0	-0.1740177	0	0.1197307	0	0	0	0.037414845	0	0
0	0	0	0	0	0	0	0.012445885	0	-0.25249808
0	0	0	0	0	0	0	0	0	-0.25248481
0	0	0	0	0	0	0	0.032072434	0	-0.25305984
0	-0.33928125	0	0.34167109	0	-0.43940793	0	0	0	0
0	0	0	0	0	0.099927845	0	-0.043370504	0	-0.25472418
0	-0.33294979	0	0.34061089	0	-0.43342748	0	0	0	0.12305225
0	0	0	0	0	0.13408847	0	-0.006278091	0	0
0	0	0	0	0	0.13349172	0	0	0	0
0	0	0	0	0	0	0	0.015940523	0	-0.62858958
0	0	0	0	0	0	0	0	0	-0.62863417
0	0	0	0	0	0	0	0.034478608	0	-0.62879424
0	0	0	0	0	0	0	0.009137905	0	0
0	0	0	0	0	0	0	0	0	0
0	0	0	0	0	0	0	0.032196248	0	0
0	0	0	0	0	0.1014167	0	-0.044489639	0	0
0	-0.21699494	0	0	0	-0.087984977	0	0.18729634	0	-0.62743596
0	0	0	0	0	0	0	0.015853925	0	-0.62728568
0	0	0	0	0	0	0	0	0	-0.62746348
0	0	0	0	0	-0.000435166	0	0	0	-0.62747505
0	-0.24783985	0	0.32658917	0	-0.35924282	0	0	0	0
0	0	0	0	0	0	0	0.011616293	0	-0.25213903
0	-0.3415561	0	0.34348086	0	-0.44992773	0	0	0	0
0	0	0	0	0	-0.028906515	0	0	0	-0.62742544
0	0	0	0	0	0	0	-0.031049753	0	-0.62727897
0	0	0	0	0	-0.024827108	0	-0.029344803	0	-0.62713011
0	0	0	0	0	0	0	0.013761574	0	-0.25532799
0	0	0	0	0	-0.000104092	0	0	0	-0.25593948
0	0	0	0	0	-0.000396288	0	0.011785747	0	-0.25843294
0	-0.22123128	0	0	0	-0.093694786	0	0.16822381	0	-0.059185941
0	-0.25335432	0	0.32635139	0	-0.35785428	0	0	0	0
0	-0.33525769	0	0.34243718	0	-0.44401503	0	0	0	0.12523971
0	-0.24902698	0	0.32671572	0	-0.35376607	0	-0.014401033	0	0

0	-0.2476913	0	0.33433776	0	-0.36015294	0	0	0	0
0	-0.22833604	0	0	0	-0.097220861	0	0.17214136	0	0
0	0	0	0	0	0	0	0.00861917	0	0
0	0	0	0	0	0	0	0.017981434	0	-0.6285846
0	0	0	0	0	0	0	0	0	-0.62862791
0	0	0	0	0	0	0	0.039438314	0	-0.62881227
0	0	0	0	0	-0.020686744	0	-0.022367898	0	-0.2519648
0	-0.16781473	0	0	0	0	0	0.049291952	0	-0.62703913
0	0	0	0	0	0	0	0.010773305	0	0
0	0	0	0	0	-0.000192641	0	0	0	0
0	0	0	0	0	-0.000402059	0	0.008459177	0	0
0	-0.16716412	0	0	0	0	0	0	0	-0.62687307
0	-0.23068316	0	0.32143353	0	-0.28692836	0	0.054338191	0	0
0	-0.2346623	0	0.32423788	0	-0.27225881	0	0	0	0
0	0	0	0	0	0	0	0.013628034	0	-0.2528768
0	-0.16514971	0	0	0	0	0	0.10030364	0	-0.62733642
0	-0.23394336	0	0.2905824	0	-0.25255249	0	0	0	0
0	-0.27191344	0	0.32708884	0	-0.30748133	0	0.072130224	0	0
0	0	0	0	0	-0.01975523	0	-0.023560016	0	0
0	-0.23575202	0	0.32446494	0	-0.24704023	0	0	0	-0.60155686
0	-0.26570977	0	0.32618094	0	-0.30338133	0	0.068679429	0	0.054614449
0	-0.24082808	0	0.32241249	0	-0.29514096	0	0.040742159	0	0
0	-0.24435737	0	0.29896224	0	-0.2712525	0	0	0	0
0	-0.16682427	0	0	0	0	0	0.044121051	0	-0.10605959
0	-0.26707666	0	0.28820745	0	-0.2590822	0	0	0	0
0	-0.25748393	0	0.32752601	0	-0.27687647	0	0	0	0
0	-0.23830656	0	0.30063664	0	-0.2784038	0	0.040872443	0	0
0	-0.23679628	0	0.3256851	0	-0.25011849	0	0	0	0
0	-0.25280808	0	0.32672378	0	-0.27542481	0	0	0	0.028035476
0	-0.23223431	0	0.32486762	0	-0.24890897	0	0	0	0.019703174
0	-0.26178089	0	0.28868386	0	-0.25753339	0	0	0	0.042249978
0	-0.25426189	0	0.32801939	0	-0.3674089	0	0	0	0
0	-0.24979441	0	0.32824977	0	-0.36337488	0	-0.011825682	0	0
0	-0.248629	0	0.33486902	0	-0.36886983	0	0	0	0
0	-0.17138083	0	0	0	0	0	0.043422543	0	0
0	0	0	0	0	0	0	0.010624129	0	0
0	-0.27030867	0	0.32796054	0	-0.31027743	0	0.079682111	0	0
0	-0.24078449	0	0.32353676	0	-0.29937185	0	0.048167354	0	0
0	-0.26403278	0	0.32704713	0	-0.30617069	0	0.076225387	0	0.052717193
0	-0.24494077	0	0.29598517	0	-0.27107987	0	0	0	0
0	-0.23855122	0	0.29870951	0	-0.28139925	0	0.047318827	0	0
0	-0.23081727	0	0.32518154	0	-0.24339746	0	0	0	-0.6069448
0	-0.26514338	0	0.28477802	0	-0.2567765	0	0	0	0
0	-0.254077	0	0.32835056	0	-0.27555033	0	0	0	0
0	-0.25981024	0	0.28526157	0	-0.25523955	0	0	0	0.039075851
0	-0.24934805	0	0.32753237	0	-0.27408521	0	0	0	0.023195914
0	-0.14066911	0	0	0	0.020053143	0	0	0	-0.62719139

0	0	0	0	0	0.10799645	0	0	0	0
0	-0.32905452	0	0.36355104	0	-0.57117259	0	0	0	-0.50288349
0	0	0	0	0	0.1010318	0	0	0	0
0	0	0	0	0	0	0	0	0	0
0	0	0	0	0	0	0	0.044564739	0	-0.62857866
0	0	0	0	0	0.005892257	0	0.043027066	0	-0.62855372
0	-0.33718513	0	0.36323811	0	-0.57757618	0	0.029941943	0	0
0	-0.33597563	0	0.36480049	0	-0.57744054	0	0	0	0
0	-0.3361309	0	0.35986675	0	-0.57323122	0	0	0	0
0	0	0	0	0	0.020147833	0	0.070537527	0	-0.62905601
0	0	0	0	0	0.007038411	0	0	0	-0.62739508
0	-0.3308789	0	0.36229139	0	-0.57156804	0	0.027987675	0	0.12392769
0	-0.33003215	0	0.36378286	0	-0.57169427	0	0	0	0.12046559
0	-0.3301766	0	0.35878261	0	-0.56741972	0	0	0	0.121965
0	0	0	0	0	0	0	-0.027307651	0	0
0	0	0	0	0	0.022377063	0	0	0	0
0	0	0	0	0	0.006259438	0	0.039810609	0	-0.25734535
0	0	0	0.16866904	0	0	0	0.029930106	0	0
0	0	0	0	0	0.10808253	0	-0.009459667	0	0
0	0	0	0	0	0.054516968	0	-0.060591342	0	0
0	0	0	0	0	0.099029951	0	0	0	0
0	0	0	0	0	0	0	0.014057354	0	0
0	0	0	0	0	0.006240076	0	0.036789818	0	0
0	0	0	0	0	0	0	0.002916936	0	0
0	-0.24965052	0	0.34845445	0	-0.49480889	0	0.019597396	0	0
0	-0.25074169	0	0.3437357	0	-0.49114171	0	0	0	0
0	-0.24740768	0	0.34216138	0	-0.48758014	0	0.021525091	0	0
0	-0.22535012	0	0.13597523	0	0	0	0	0	0
0	-0.19440734	0	0.14898811	0	0	0	0	0	-0.05019854
0	-0.20050312	0	0.14688045	0	0	0	0	0	0
0	-0.17278544	0	0.19065683	0	0	0	0	0	-0.6268732
0	-0.22664029	0	0.1393287	0	0	0	-0.0307382	0	0
0	-0.22618366	0	0.13781698	0	0	0	0	0	0
0	0	0	0	0	0.048759383	0	-0.053295193	0	0
0	-0.16759152	0	0.19043435	0	0	0	0	0	-0.069164856
0	0	0	0	0	0.032921123	0	-0.093729551	0	0
0	0	0	0	0	0	0	0.016081585	0	0
0	0	0	0	0	0.099119777	0	-0.006622762	0	0
0	-0.17340071	0	0.18912794	0	0	0	0	0	0
0	-0.17064282	0	0.19331205	0	0	0	-0.028908584	0	-0.066312127
0	-0.1862778	0	0.16998005	0	0	0	-0.090343679	0	-0.62639687
0	-0.17159543	0	0.19106152	0	0	0	0	0	-0.066920272
0	-0.17618649	0	0.19207909	0	0	0	-0.029357355	0	0
0	-0.23136605	0	0.12578598	0	0	0	-0.10518951	0	0
0	-0.20992026	0	0.18254722	0	0	0	-0.026011777	0	0
0	-0.17731459	0	0.18976587	0	0	0	0	0	0
0	-0.20952888	0	0.18086635	0	0	0	0	0	0

0	-0.20328821	0	0.13768077	0	0	0	-0.10804928	0	-0.045588342
0	-0.18091029	0	0.17063487	0	0	0	-0.092937963	0	-0.061452404
0	-0.20843346	0	0.13598803	0	0	0	-0.10769022	0	0
0	-0.22880417	0	0.12902527	0	0	0	-0.096652804	0	0
0	-0.18573893	0	0.16988959	0	0	0	-0.092202838	0	0
0	-0.21146287	0	0.18371732	0	0	0	-0.027019671	0	0
0	-0.18325139	0	0.17275954	0	0	0	-0.088305677	0	-0.05657489
0	-0.21463219	0	0.16561442	0	0	0	-0.078183649	0	0
0	-0.33242491	0	0	0	-0.11275406	0	0	0	-0.56477364
0	-0.18799507	0	0.17206287	0	0	0	-0.087231585	0	0
0	-0.33394223	0	0	0	-0.11628552	0	0	0	0
0	-0.19421566	0	0.1454403	0	0	0	0	0	-0.051598615
0	-0.32879464	0	0	0	-0.11452613	0	0	0	0.056317805
0	-0.2003404	0	0.14333846	0	0	0	0	0	0
0	-0.17160322	0	0.18911436	0	0	0	0	0	-0.62687274
0	-0.22743028	0	0.1354402	0	0	0	-0.028869901	0	0
0	-0.227086	0	0.13398179	0	0	0	0	0	0
0	0	0	0.33403152	0	-0.23291464	0	0	0	-0.62719518
0	0	0	0.36747632	0	-0.2241189	0	0	0	-0.1886406
0	-0.29271775	0	0	0	-0.096550716	0	-0.010935205	0	0
0	-0.29228545	0	0	0	-0.095631052	0	0	0	0
0	-0.2957039	0	0	0	-0.10557263	0	0	0	0
0	-0.34884102	0	0	0	-0.13450067	0	0	0	0
0	-0.34013115	0	0	0	-0.11808527	0	0	0	0
0	-0.33958302	0	0	0	-0.11786542	0	0.005385021	0	0
0	0	0	0.36948908	0	-0.22135323	0	0	0	-0.62704784
0	-0.34168258	0	0	0	-0.1288948	0	0	0	0.069068938
0	-0.33497448	0	0	0	-0.11624813	0	0	0	0.060244205
0	-0.33346047	0	0	0	-0.11420054	0	0.002483457	0	0.060922599
0	0	0	0.33217381	0	-0.19794091	0	-0.043781691	0	-0.1959787
0	-0.20538107	0	0.12998674	0	0	0	0	0	-0.62666701
0	0	0	0.32846312	0	-0.18385303	0	0	0	-0.19622909
0	-0.18439093	0	0.16969816	0	0	0	-0.084335162	0	-0.62636543
0	0	0	0.3283926	0	-0.17774316	0	0	0	-0.62706701
0	0	0	0.3693561	0	-0.22524655	0	0	0	0
0	-0.16949314	0	0.1916271	0	0	0	-0.026945925	0	-0.068302402
0	0	0	0.33243955	0	-0.19506656	0	-0.044288135	0	-0.6269169
0	-0.17044802	0	0.1894995	0	0	0	0	0	-0.069165281
0	0	0	0.33273279	0	-0.22245478	0	0	0	-0.18938377
0	-0.2028544	0	0.134647	0	0	0	-0.10331073	0	-0.047013013
0	-0.2268769	0	0.10551456	0	0	0	0	0	0
0	-0.29659449	0	0	0	-0.095640574	0	-0.015764302	0	0
0	-0.17508448	0	0.19039841	0	0	0	-0.027421346	0	0
0	-0.29562641	0	0	0	-0.092935207	0	0	0	0
0	-0.29350267	0	0	0	-0.090550677	0	-0.01038728	0	0
0	-0.20044377	0	0.12947871	0	0	0	0	0	-0.034161854
0	-0.21786431	0	0.10480936	0	0	0	0	0	-0.62668436

0	-0.20805723	0	0.13295127	0	0	0	-0.10295388	0	0
0	0	0	0	0	0.12003307	0	0	0	0
0	-0.17620228	0	0.18820809	0	0	0	0	0	0
0	-0.20527542	0	0.129282	0	0	0	0	0	0
0	0	0	0.33356983	0	-0.19828783	0	-0.044829273	0	0
0	0	0	0.33003874	0	-0.18568893	0	0	0	0
0	0	0	0.36125902	0	-0.20252538	0	-0.025559863	0	-0.1908048
0	-0.22957118	0	0.12561712	0	0	0	-0.092359854	0	0
0	-0.32976015	0	0	0	-0.11251428	0	0	0	-0.56788462
0	0	0	0.3330412	0	-0.21972856	0	0	0	-0.62717996
0	0	0	0	0	0.09649263	0	-0.001037017	0	0
0	0	0	0.36117768	0	-0.2044196	0	0	0	-0.19020044
0	-0.27156418	0	0	0	0	0	0	0	-0.62565701
0	0	0	0.36847321	0	-0.21984024	0	0	0	-0.18785802
0	-0.21435734	0	0.11140645	0	0	0	0	0	-0.022630523
0	-0.21985887	0	0.1097714	0	0	0	0	0	0
0	0	0	0.33415821	0	-0.22297224	0	0	0	0
0	-0.22444129	0	0.10010248	0	0	0	0	0	-0.62650524
0	-0.21176374	0	0.18178585	0	0	0	-0.025155953	0	0
0	0	0	0.32934053	0	-0.17595547	0	-0.028835853	0	-0.19679359
0	0	0	0.33190517	0	-0.19194332	0	-0.045317811	0	-0.1951713
0	-0.18149602	0	0.17226691	0	0	0	-0.083207733	0	-0.059322391
0	0	0	0.32804162	0	-0.17727495	0	0	0	-0.19613897
0	-0.27024235	0	0	0	0	0	-0.018061696	0	0
0	-0.26695319	0	0	0	0	0	0	0	-0.005150202
0	-0.2716791	0	0	0	0	0	0	0	0
0	-0.27003431	0	0	0	0	0	0	0	0
0	0	0	0.36280896	0	-0.20296174	0	-0.026472595	0	0
0	-0.18630043	0	0.17157616	0	0	0	-0.082139371	0	0
0	0	0	0.36381602	0	-0.20712117	0	0	0	0
0	0	0	0.37002309	0	-0.22035741	0	0	0	0
0	-0.35142274	0	0	0	-0.14376822	0	0	0	0
0	-0.23666414	0	0.28277813	0	-0.36970396	0	0.12484829	0	0
0	-0.21960099	0	0.099653103	0	0	0	0	0	-0.033582186
0	-0.33835068	0	0	0	-0.11913658	0	0	0	0
0	-0.33914606	0	0	0	-0.12180728	0	0.011773131	0	0
0	-0.22531339	0	0.097911756	0	0	0	0	0	0
0	0	0	0.33097192	0	-0.17760866	0	-0.031290169	0	0
0	0	0	0	0	0.12425971	0	0.02233887	0	0
0	-0.27354447	0	0	0	0	0	-0.072872148	0	-0.62476218
0	-0.34429754	0	0	0	-0.13821824	0	0	0	0.071239272
0	0	0	0.33326434	0	-0.19207978	0	-0.045892749	0	0
0	-0.24974969	0	0.27738628	0	-0.36918587	0	0.14415005	0	0
0	-0.33315214	0	0	0	-0.1173033	0	0	0	0.057229142
0	0	0	0.32963726	0	-0.17912823	0	0	0	0
0	-0.33296306	0	0	0	-0.1181353	0	0.008868702	0	0.060098429
0	0	0	0.36527931	0	-0.21987606	0	0	0	-0.62701018

0	-0.24282073	0	0.2773719	0	-0.3649004	0	0.14087409	0	0.02183316
0	-0.27638276	0	0	0	0	0	-0.018652601	0	0
0	-0.27300338	0	0	0	0	0	-0.019495137	0	0
0	-0.27183193	0	0	0	0	0	-0.018761492	0	-0.000750645
0	-0.27623951	0	0	0	0	0	0	0	0
0	-0.27161179	0	0	0	0	0	0	0	-0.001359882
0	-0.26959365	0	0	0	0	0	-0.069732395	0	0
0	-0.26941429	0	0	0	0	0	-0.075657466	0	-0.004162395
0	-0.27365489	0	0	0	0	0	-0.075530335	0	0
0	-0.20238076	0	0.1318618	0	0	0	0	0	-0.62667405
0	0	0	0.33331952	0	-0.19958259	0	-0.035258837	0	-0.62692356
0	0	0	0.33032819	0	-0.18785639	0	0	0	-0.62705206
0	0	0	0	0	0.11809295	0	0.012622642	0	0
0	-0.29696525	0	0	0	-0.10039619	0	-0.009592719	0	0
0	-0.29686098	0	0	0	-0.10131687	0	0	0	0
0	-0.29456805	0	0	0	-0.099041969	0	-0.00792791	0	0
0	0	0	0.33386068	0	-0.21982508	0	0	0	-0.627172
0	-0.20898108	0	0.34131647	0	-0.38051462	0	0.18376735	0	-0.6234402
0	-0.269545	0	0	0	0	0	0	0	-3.1691672
0	0	0	0.35913628	0	-0.20590768	0	-0.018768669	0	-0.19167147
0	-0.27735081	0	0	0	0	0	-0.069266085	0	0
0	-0.27319834	0	0	0	0	0	-0.06984663	0	0.002354152
0	0	0	0.36208718	0	-0.2136357	0	0	0	-0.19049837
0	0	0	0.36502784	0	-0.21976992	0	0	0	-0.18991129
0	-0.27020483	0	0	0	0	0	0	0	-0.62724135
0	-0.22319414	0	0.098685134	0	0	0	0	0	-0.62650398
0	-0.2133805	0	0.10940778	0	0	0	0	0	-0.025195759
0	0	0	0.24354577	0	0	0	0	0	-0.62730069
0	-0.27167097	0	0	0	0	0	0	0	-0.61430075
0	0	0	0.33114933	0	-0.18617033	0	-0.026013282	0	-0.19686308
0	-0.21892375	0	0.10777382	0	0	0	0	0	0
0	0	0	0.3328613	0	-0.19688836	0	-0.037178857	0	-0.19599353
0	0	0	0.32997633	0	-0.18739692	0	0	0	-0.19638059
0	-0.22431494	0	0.34430753	0	-0.39626406	0	0.16702957	0	0
0	-0.21769924	0	0.34318097	0	-0.39171475	0	0.16301074	0	-0.002511044
0	0	0	0.36069059	0	-0.20633946	0	-0.019686293	0	0
0	0	0	0	0	0	0	0.038079743	0	0
0	0	0	0.36471417	0	-0.21633233	0	0	0	0
0	0	0	0.36657065	0	-0.22028066	0	0	0	0
0	-0.27194782	0	0	0	0	0	0	0	0
0	-0.26743192	0	0	0	0	0	0	0	0.006618195
0	0	0	0.33277739	0	-0.1878269	0	-0.028464271	0	0
0	0	0	0.33423077	0	-0.19702113	0	-0.037777126	0	0
0	0	0	0.24569011	0	0	0	-0.034075537	0	-0.21638412
0	0	0	0.33156481	0	-0.18925213	0	0	0	0
0	0	0	0.24795861	0	0	0	-0.030871128	0	-0.6270477
0	-0.27223729	0	0	0	0	0	-0.068872293	0	-0.62719422

0	0	0	0.23522145	0	0	0	0	0	-0.21722385
0	0	0	0.2388172	0	0	0	0	0	-0.6273728
0	0	0	0.24339236	0	0	0	0	0	-0.21567958
0	0	0	0.24592032	0	0	0	0	0	-0.6273114
0	-0.27234886	0	0	0	0	0	-0.017932596	0	0
0	-0.27420118	0	0	0	0	0	-0.017088325	0	0
0	-0.26959779	0	0	0	0	0	-0.017160944	0	-0.003431231
0	-0.26942902	0	0	0	0	0	0	0	-0.004205815
0	-0.27409862	0	0	0	0	0	0	0	0
0	0	0	0	0	0.11548104	0	0.025368047	0	0
0	0	0	0.24659285	0	0	0	-0.036869846	0	0
0	0	0	0.23629195	0	0	0	0	0	0
0	0	0	0.2404796	0	0	0	-0.036096864	0	-0.21699922
0	0	0	0.24414124	0	0	0	0	0	0
0	0	0	0.24814677	0	0	0	-0.035743047	0	-0.21666725
0	0	0	0.23862999	0	0	0	0	0	-0.21630011
0	-0.27523295	0	0	0	0	0	-0.065974773	0	0
0	-0.27102056	0	0	0	0	0	-0.066541729	0	-0.000519336
0	-0.26950251	0	0	0	0	0	0	0	-0.6175469
0	-0.20882619	0	0.25352138	0	-0.29306171	0	0	0	-0.035818146
0	-0.21417813	0	0.25280345	0	-0.29484898	0	0	0	0
0	0	0	0.24164349	0	0	0	-0.038871044	0	0
0	-0.19341343	0	0.23891101	0	-0.25935729	0	0	0	-0.62642748
0	0	0	0.24904053	0	0	0	-0.038262906	0	0
0	0	0	0.23964626	0	0	0	0	0	0
0	0	0	0.21774073	0	0	0	-0.13571633	0	-0.62686633
0	0	0	0	0	-0.016737031	0	0.040476747	0	0
0	0	0	0.22071255	0	0	0	-0.12309222	0	-0.21516963
0	0	0	0.24546377	0	0	0	-0.028599999	0	-0.62720147
0	0	0	0.22589119	0	0	0	-0.13182943	0	-0.2210938
0	0	0	0.22243559	0	0	0	-0.12242625	0	-0.62687977
0	0	0	0.23461331	0	0	0	0	0	-0.62715995
0	0	0	0.24354617	0	0	0	0	0	-0.62730602
0	0	0	0.22898591	0	0	0	-0.13013514	0	-0.62672605
0	0	0	0.22196405	0	0	0	-0.12381968	0	0
0	0	0	0.22710427	0	0	0	-0.13304836	0	0
0	0	0	0.22930902	0	0	0	-0.12082381	0	-0.21438889
0	0	0	0.23621386	0	0	0	-0.034022188	0	-0.21839238
0	0	0	0.24565014	0	0	0	-0.033591956	0	-0.21794783
0	0	0	0.23445837	0	0	0	0	0	-0.21786154
0	0	0	0.23051042	0	0	0	-0.12157719	0	0
0	0	0	0.23737944	0	0	0	-0.036814123	0	0
0	0	0	0.24653855	0	0	0	-0.036125371	0	0
0	0	0	0.23547189	0	0	0	0	0	0
0	0	0	0.22098169	0	0	0	-0.11656797	0	-0.62689168
0	0	0	0.22521172	0	0	0	-0.12519292	0	-0.62673744
0	-0.13883898	0	0.34534926	0	-0.28321563	0	0	0	-0.62685142

0	0	0	0.22548573	0	0	0	-0.116372	0	-0.21591022
0	-0.31509994	0	0	0	-0.087892708	0	0	0	-0.58203969
0	-0.31928575	0	0	0	-0.095860482	0	0	0	0
0	-0.26428926	0	0	0	0	0	0	0	-0.6264829
0	0	0	0.22669329	0	0	0	-0.11713196	0	0
0	0	0	0.16677428	0	0	0	0	0	-0.62713859
0	-0.3119042	0	0	0	-0.090180925	0	0	0	0.039323786
0	-0.26244575	0	0	0	0	0	0.044463737	0	-0.62681014
0	-0.28735058	0	0	0	-0.071222407	0	0	0	0
0	-0.28458573	0	0	0	-0.070330027	0	0.012179442	0	0
0	0	0	0.19998378	0	0	0	0	0	-0.20274381
0	0	0	0.20358282	0	0	0	0	0	-0.62718265
0	-0.26345602	0	0	0	0	0	0	0	-0.62669378
0	-0.26926152	0	0	0	0	0	0	0	-0.62644324
0	-0.26865799	0	0	0	0	0	0.01001037	0	-0.62645772
0	-0.32369888	0	0	0	-0.094351042	0	0	0	0
0	-0.323802	0	0	0	-0.095814825	0	0.014804924	0	0
0	-0.27626645	0	0	0	-0.021342128	0	0.065786462	0	-0.61951349
0	-0.31635172	0	0	0	-0.088655222	0	0	0	0.040478215
0	-0.26823407	0	0	0	0	0	0.034850087	0	-0.62658468
0	-0.31624783	0	0	0	-0.089850807	0	0.013504494	0	0.043874515
0	0	0	0.2011409	0	0	0	0	0	0
0	-0.2887363	0	0	0	-0.068106361	0	0.008658859	0	0
0	-0.24596281	0	0	0	0.029528713	0	0	0	-0.62652073
0	-0.26353722	0	0	0	0	0	0	0	-0.011311332
0	-0.26963057	0	0	0	0	0	0.003634968	0	0
0	-0.26822803	0	0	0	0	0	0	0	0
0	-0.27712241	0	0	0	-0.027513728	0	0.054724813	0	0
0	-0.2630397	0	0	0	0	0	0.004938196	0	-0.009257775
0	-0.26778506	0	0	0	0	0	0.004949696	0	0
0	-0.27793961	0	0	0	-0.023493429	0	0.065777354	0	0
0	-0.27132664	0	0	0	-0.020354399	0	0.062558087	0	5.74E-05
0	-0.26239631	0	0	0	0	0	0.03252156	0	-0.014012652
0	-0.26292132	0	0	0	0.010186509	0	0	0	-0.62655798
0	-0.26730694	0	0	0	0	0	0.033321886	0	0
0	0	0	0.41480959	0	-0.24294829	0	0	0	0
0	0	0	0.1910004	0	0	0	0	0	-0.62729442
0	-0.25743181	0	0	0	0.009570819	0	0	0	-0.024481652
0	0	0	0.44224099	0	-0.29612924	0	0	0	0
0	-0.26263066	0	0	0	0.008802266	0	0	0	0
0	-0.31764028	0	0	0	-0.096819325	0	0	0	-0.58005244
0	-0.26805612	0	0	0	0	0	0.002424432	0	-0.007127615
0	-0.27270568	0	0	0	0	0	0.002653902	0	0
0	-0.28647478	0	0	0	-0.028169623	0	0.056348746	0	0
0	-0.27998237	0	0	0	-0.025099173	0	0.053016839	0	0.001885862
0	0	0	0.42933066	0	-0.2681278	0	0.006351797	0	0
0	-0.34025566	0	0	0	-0.25318557	0	0	0	-0.55872708

0	0	0	0.36938514	0	-0.33091484	0	0	0	-0.62686323
0	0	0	0.35059967	0	-0.31480009	0	0.012253712	0	-0.6269265
0	0	0	0.43519689	0	-0.28319981	0	0	0	0
0	0	0	0.35135026	0	-0.31509673	0	0	0	-0.62706527
0	0	0	0.20061371	0	0	0	0	0	-0.62705133
0	0	0	0.37746408	0	-0.23880144	0	-0.006831519	0	0
0	0	0	0.37736824	0	-0.24283819	0	0	0	0
0	0	0	0.41986978	0	-0.24956666	0	0.015888605	0	0
0	-0.32637437	0	0	0	-0.10502689	0	0.017555222	0	0
0	-0.32619782	0	0	0	-0.10320095	0	0	0	0
0	-0.34906354	0	0	0	-0.26034648	0	0.031251912	0	0
0	-0.3188781	0	0	0	-0.097550718	0	0	0	0.042339912
0	0	0	0.44254356	0	-0.28907456	0	-0.014183223	0	0
0	-0.31882342	0	0	0	-0.099089919	0	0.016239578	0	0.04607704
0	-0.26700799	0	0	0	0	0	0	0	-0.62667227
0	-0.34745031	0	0	0	-0.25848555	0	0	0	0
0	-0.26643036	0	0	0	0	0	0.011742689	0	-0.62646238
0	0	0	0.36888617	0	-0.33028967	0	0.010065044	0	-0.19056763
0	0	0	0.36881142	0	-0.32998981	0	0	0	-0.1902768
0	-0.34194042	0	0	0	-0.2546439	0	0.029809414	0	0.068166532
0	-0.28992226	0	0	0	-0.076293993	0	0.01116085	0	0
0	0	0	0.35052243	0	-0.31422378	0	0.008545239	0	-0.19292185
0	-0.27471802	0	0	0	-0.02335548	0	0.073440717	0	-0.62141327
0	0	0	0.33568114	0	-0.27474121	0	0.063790133	0	-0.62711905
0	0	0	0.34507397	0	-0.28109196	0	0.066646621	0	-0.6271605
0	-0.34071246	0	0	0	-0.25311356	0	0	0	0.066069443
0	-0.26593011	0	0	0	0	0	0.039624555	0	-0.62660425
0	0	0	0.38140257	0	-0.24566797	0	-0.019540761	0	0
0	-0.29837662	0	0	0	-0.19168931	0	0.090403653	0	-0.59882926
0	-0.29525311	0	0	0	-0.21794918	0	0.025789326	0	0
0	0	0	0.37140755	0	-0.33265307	0	0.007821513	0	0
0	0	0	0.37121949	0	-0.33230556	0	0	0	0
0	0	0	0.37759724	0	-0.2356317	0	-0.012428661	0	0
0	0	0	0.35205937	0	-0.3157256	0	0.006378064	0	0
0	-0.25957312	0	0	0	0.012314403	0	0	0	-0.62655868
0	0	0	0.31830625	0	-0.24249494	0	0	0	-0.62697163
0	0	0	0.33599843	0	-0.27472497	0	0.056952551	0	-0.2001742
0	-0.26585619	0	0	0	0	0	0.004046451	0	-0.009922362
0	-0.27055878	0	0	0	0	0	0.004245086	0	0
0	-0.28547977	0	0	0	-0.030772007	0	0.063205286	0	0
0	-0.27891047	0	0	0	-0.02769759	0	0.059871871	0	-7.93E-05
0	0	0	0.43626368	0	-0.29281049	0	0	0	0
0	-0.30621137	0	0	0	-0.19629656	0	0.085029245	0	0
0	-0.29972163	0	0	0	-0.19233439	0	0.081784846	0	0.023180371
0	0	0	0.41823178	0	-0.25355745	0	0.022628711	0	0
0	0	0	0.33737333	0	-0.27505907	0	0.056531104	0	0
0	-0.27186345	0	0	0	-0.15123375	0	0	0	-0.62636988

0	0	0	0.44351424	0	-0.29867708	0	-0.011408099	0	0
0	0	0	0.37938781	0	-0.24617773	0	-0.009529929	0	0
0	0	0	0	0	0.11694846	0	0	0	-0.25668875
0	0	0	0	0	0.1231538	0	0	0	-0.62879546
0	0	0	0.26898463	0	0	0	-0.017259091	0	0
0	0	0	0.26719438	0	0	0	0	0	0
0	0	0	0	0	0.11664296	0	0	0	0
0	0	0	0	0	0.12537014	0	-0.025489036	0	-0.25727144
0	0	0	0	0	0.12315471	0	0	0	-0.2549319
0	-0.13341186	0	0.07189364	0	0	0	0	0	-0.62704134
0	0	0	0.27159262	0	0	0	-0.01857813	0	0
0	0	0	0.27296886	0	0	0	-0.026843557	0	0
0	0	0	0	0	0	0	0	0	-0.62876026
0	0	0	0	0	0.12536461	0	-0.028821883	0	0
0	0	0	0	0	0.12285119	0	0	0	0
0	0	0	0	0	0.114611	0	0	0	-0.62858015
0	-0.13220581	0	0.072643429	0	0	0	0.012111374	0	-0.1279809
0	-0.13164417	0	0.073076222	0	0	0	0	0	-0.12754748
0	-0.14190802	0	0.06212241	0	0	0	-0.043417907	0	-0.62662177
0	0	0	0.25737036	0	0	0	-0.094860975	0	0
0	-0.14639145	0	0.055769125	0	0	0	0	0	-0.62688671
0	0	0	0	0	0	0	0	0	-0.25018847
0	0	0	0	0	0	0	-0.010635153	0	-0.25276964
0	-0.17737715	0	0	0	0	0	0.021460058	0	-0.62673794
0	-0.13835781	0	0.070069616	0	0	0	0.01107839	0	0
0	-0.13769124	0	0.070503686	0	0	0	0	0	0
0	-0.1769152	0	0	0	0	0	0	0	-0.62668156
0	0	0	0	0	0.11662141	0	-0.022745913	0	-0.25765537
0	0	0	0	0	0	0	-0.076279299	0	-0.62728811
0	0	0	0	0	0.11459377	0	0	0	-0.25549942
0	0	0	0	0	0.049597516	0	-0.11162339	0	-0.25630632
0	-0.13854015	0	0.064525782	0	0	0	-0.036123577	0	-0.1187552
0	-0.17848119	0	0	0	0	0	-0.021260021	0	-0.62656534
0	0	0	0	0	0	0	0	0	-0.62846707
0	0	0	0	0	0	0	-0.007301056	0	-0.62851796
0	-0.14401063	0	0.062361796	0	0	0	-0.03540858	0	0
0	0	0	0	0	0	0	0	0	0
0	0	0	0	0	0	0	-0.013935567	0	0
0	0	0	0	0	0	0	-0.06573348	0	-0.24726691
0	-0.17616217	0	0	0	0	0	0.01735355	0	-0.095671945
0	0	0	0	0	0.05376385	0	-0.11185547	0	-0.6269767
0	0	0	0	0	0.050609062	0	-0.11347629	0	0
0	0	0	0	0	0.11660969	0	-0.026086602	0	0
0	-0.18055521	0	0	0	0	0	0.016757539	0	0
0	0	0	0	0	0.11426517	0	0	0	0
0	0	0	0	0	0	0	0	0	-0.62741604
0	0	0	0.26756272	0	0	0	-0.016424062	0	0

0	0	0	0	0	0.059609213	0	-0.10248344	0	-0.24843409
0	0	0	0	0	0	0	-0.010413601	0	-0.25082883
0	0	0	0	0	0	0	-0.063354519	0	-0.62730799
0	0	0	0	0	0	0	-0.066408029	0	0
0	0	0	0	0	0.008618237	0	0	0	-0.23777086
0	0	0	0	0	0.060730284	0	-0.10386684	0	0
0	0	0	0	0	0	0	0	0	-0.62845765
0	0	0	0	0	0	0	-0.005433112	0	-0.62841199
0	-0.2094394	0	0.037752453	0	0	0	0.022197288	0	0
0	0	0	0	0	0	0	-0.013384652	0	0
0	0	0	0	0	-0.011687106	0	0	0	-0.6284167
0	0	0	0.44186754	0	-0.40798055	0	0.028503399	0	0
0	0	0	0.14263998	0	0	0	0	0	-0.62705388
0	0	0	0.14261452	0	0	0	0.009500987	0	-0.62707124
0	0	0	0	0	0.048419755	0	-0.1044315	0	-0.62698879
0	0	0	0	0	0.012872409	0	0	0	-0.62751267
0	0	0	0	0	0.009157815	0	0	0	0
0	0	0	0	0	0	0	-0.008548025	0	-0.25155782
0	0	0	0	0	0.054020046	0	-0.095588358	0	-0.24916569
0	0	0	0	0	0	0	-0.059918933	0	-0.62730881
0	-0.081979707	0	0.14819845	0	0	0	0.02697744	0	-0.62726773
0	-0.080498859	0	0.14990596	0	0	0	0	0	-0.62720832
0	-0.087107385	0	0.14114233	0	0	0	0.005718424	0	-0.62712835
0	0	0	0.14257866	0	0	0	0.006757997	0	-0.23741038
0	0	0	0	0	0.055152022	0	-0.096962679	0	0
0	0	0	0	0	0	0	-0.011522968	0	0
0	0	0	0.13636045	0	0	0	-0.053153519	0	-0.62684206
0	-0.31296687	0	0	0	-0.22624495	0	0.063623451	0	-0.58440549
0	0	0	0	0	0.010192204	0	0	0	-0.62745787
0	-0.080211337	0	0.14924815	0	0	0	0.022418127	0	-0.1650447
0	0	0	0.14354188	0	0	0	0.004065644	0	0
0	0	0	0	0	0.13592757	0	0	0	-0.62851811
0	-0.31029021	0	0	0	-0.22268216	0	0	0	-0.59087088
0	-0.085909838	0	0.14766478	0	0	0	0.021248552	0	0
0	0	0	0.1802292	0	0	0	0.020257369	0	-0.62725085
0	-0.3208347	0	0	0	-0.23188988	0	0.058660746	0	0
0	0	0	0	0	0.13630659	0	-0.004336332	0	-0.25791402
0	0	0	0	0	0.13588918	0	0	0	-0.25769234
0	-0.3133193	0	0	0	-0.22591475	0	0.057268469	0	0.039353874
0	0	0	0	0	0.094520148	0	-0.052119905	0	-0.62736439
0	0	0	0	0	0.14204998	0	-0.001604063	0	-0.62877932
0	0	0	0	0	0.1419145	0	0	0	-0.62850062
0	0	0	0	0	0.070547723	0	0	0	-0.62745911
0	0	0	0	0	0.0990453	0	-0.047984718	0	-0.25348719
0	0	0	0	0	0.13630413	0	-0.007682427	0	0
0	0	0	0	0	0.13556088	0	0	0	0
0	0	0	0	0	0.14247673	0	-0.006133966	0	-0.25833602

0	0	0	0	0	0.10238634	0	-0.048608587	0	-0.62736051
0	0	0	0	0	0.10052045	0	-0.049098257	0	0
0	0	0	0	0	0.14244176	0	-0.009176914	0	0
0	0	0	0	0	0.13384504	0	0	0	-0.62849981
0	0	0	0	0	0.1336685	0	0.001299605	0	-0.6284997
0	0	0	0	0	0	0	0.016832428	0	-0.62880556
0	0	0	0	0	0	0	0	0	-0.62866251
0	0	0	0	0	0	0	0.034062873	0	-0.62879632
0	-0.33253956	0	0.34026736	0	-0.43291355	0	0	0	-0.50203347
0	-0.33555111	0	0.34184197	0	-0.4415794	0	0	0	0
0	0	0	0	0	0.098349957	0	-0.040401468	0	-0.62738146
0	0	0	0	0	0.13414165	0	-0.003291153	0	-0.25880687
0	-0.32919266	0	0.34077614	0	-0.43560656	0	0	0	0.11946141
0	0	0	0	0	0	0	0.01281761	0	-0.2519291
0	-0.3377796	0	0.35206157	0	-0.44685745	0	0	0	0
0	-0.34002661	0	0.34160777	0	-0.43985443	0	0	0	0
0	-0.33890771	0	0.34193147	0	-0.43883342	0	-0.006384459	0	0
0	-0.33370233	0	0.34053637	0	-0.43386576	0	0	0	0.12076689
0	-0.33147368	0	0.35091199	0	-0.44082114	0	0	0	0.12354836
0	-0.33252964	0	0.34096364	0	-0.43273976	0	-0.00836376	0	0.12253144
0	0	0	0	0	0.13409587	0	-0.006335044	0	0
0	0	0	0	0	0	0	0.015995962	0	-0.6281732
0	-0.22517165	0	0	0	-0.098026606	0	0.17392169	0	-0.62736851
0	0	0	0	0	0	0	0.00984416	0	0
0	-0.3348576	0	0.34209216	0	-0.44349732	0	0	0	-0.49983768
0	0	0	0	0	0	0	0.01800601	0	-0.62728055
0	0	0	0	0	-0.000895165	0	0.015887186	0	-0.62726168
0	0	0	0	0	-0.000485004	0	0	0	-0.627219
0	-0.2517001	0	0.32692274	0	-0.36235515	0	0	0	0
0	-0.24771118	0	0.32725871	0	-0.35859471	0	-0.013265804	0	0
0	-0.24640339	0	0.33473848	0	-0.3648326	0	0	0	0
0	-0.34236385	0	0.34340489	0	-0.45037075	0	0	0	0
0	0	0	0	0	-0.021864461	0	-0.019461988	0	-0.62713229
0	-0.34021687	0	0.3527804	0	-0.45658955	0	0	0	0
0	-0.34134038	0	0.34363433	0	-0.44958819	0	-0.003772275	0	0
0	0	0	0	0	-0.000437421	0	0.013780611	0	-0.25530456
0	-0.25347336	0	0.32727387	0	-0.35729639	0	-0.017945964	0	0
0	-0.2517901	0	0.33586013	0	-0.36448897	0	0	0	0
0	-0.33606865	0	0.34234932	0	-0.44444186	0	0	0	0.12281781
0	-0.33394181	0	0.35164666	0	-0.45061814	0	0	0	0.12568567
0	-0.33497224	0	0.34267773	0	-0.44353365	0	-0.005765501	0	0.12488205
0	-0.24766081	0	0.33446778	0	-0.35916646	0	-0.013678538	0	0
0	-0.26730714	0	0.32593592	0	-0.30668367	0	0.072637915	0	-0.56831629
0	0	0	0	0	0	0	0.018174804	0	-0.62859332
0	-0.26853062	0	0.32721578	0	-0.31007192	0	0.072945692	0	0
0	-0.16794869	0	0	0	0	0	0.049383868	0	-0.62682975
0	-0.25275548	0	0.32640363	0	-0.27555356	0	0	0	-0.59669694

0	-0.23211266	0	0.32456434	0	-0.24891605	0	0	0	-0.60504539
0	-0.26165316	0	0.28834906	0	-0.25753968	0	0	0	-0.58250029
0	-0.2623076	0	0.32630341	0	-0.30600464	0	0.069525923	0	0.051386731
0	-0.23904535	0	0.32279234	0	-0.29851411	0	0.043064128	0	0
0	0	0	0	0	-0.000455387	0	0.010794525	0	0
0	-0.24267399	0	0.29841936	0	-0.27325453	0	0	0	0
0	-0.23696783	0	0.30070051	0	-0.28231769	0	0.042545007	0	0
0	-0.27906146	0	0.32824266	0	-0.31841387	0	0.060424945	0	0
0	-0.26354648	0	0.28811142	0	-0.26108731	0	0	0	0
0	-0.28452183	0	0.30127006	0	-0.30433088	0	0.059319194	0	0
0	-0.25354098	0	0.32760177	0	-0.27809257	0	0	0	0
0	-0.25822309	0	0.28857939	0	-0.25954629	0	0	0	0.038809211
0	-0.24883654	0	0.32677159	0	-0.27662914	0	0	0	0.024477747
0	-0.27297803	0	0.32735558	0	-0.31445814	0	0.056892201	0	0.052769376
0	-0.278125	0	0.3011271	0	-0.30041055	0	0.056276551	0	0.062003525
0	-0.27651082	0	0.29631493	0	-0.28079666	0	0	0	0
0	-0.27117891	0	0.29677528	0	-0.27919628	0	0	0	0.042795278
0	-0.24532056	0	0.30539728	0	-0.29043796	0	0.031996146	0	0
0	-0.25437662	0	0.32880747	0	-0.36691068	0	-0.015497757	0	0
0	-0.25288254	0	0.33643018	0	-0.37327313	0	0	0	0
0	-0.24860959	0	0.33497371	0	-0.36805254	0	-0.011201182	0	0
0	-0.26566724	0	0.32677708	0	-0.30950956	0	0.080177883	0	-0.57023599
0	-0.25962788	0	0.28495964	0	-0.2552006	0	0	0	-0.58564296
0	-0.24929901	0	0.32723403	0	-0.27422559	0	0	0	-0.60152434
0	-0.2779818	0	0.329161	0	-0.32184971	0	0.06719225	0	0
0	-0.284338	0	0.29923226	0	-0.30681312	0	0.065421216	0	0
0	-0.27182996	0	0.3282678	0	-0.31788506	0	0.06365944	0	0.050852862
0	-0.27786318	0	0.29906005	0	-0.30284923	0	0.062337056	0	0.060935905
0	-0.27530249	0	0.29346575	0	-0.27999664	0	0	0	0
0	-0.2460089	0	0.30364408	0	-0.29390016	0	0.037950588	0	0
0	-0.26993011	0	0.2939321	0	-0.2784037	0	0	0	0.039766005
0	0	0	0	0	0.10149495	0	0	0	0
0	0	0	0	0	0.1077914	0	0.002543563	0	0
0	-0.3307373	0	0.36172062	0	-0.57149167	0	0.033427638	0	-0.50082352
0	-0.3297453	0	0.36349674	0	-0.57161787	0	0	0	-0.50476891
0	-0.32979903	0	0.35826073	0	-0.56709005	0	0	0	-0.50312372
0	0	0	0	0	0.048843349	0	-0.057259473	0	0
0	0	0	0	0	0	0	0.015270613	0	0
0	0	0	0	0	0.10168616	0	-0.007986393	0	0
0	0	0	0	0	0.005861175	0	0.044154096	0	-0.62847933
0	-0.33752508	0	0.36327028	0	-0.5778371	0	0.028993869	0	0
0	-0.33767266	0	0.35976251	0	-0.57488233	0	0.029555069	0	0
0	-0.33662427	0	0.36023754	0	-0.57392077	0	0	0	0
0	-0.33125537	0	0.36232529	0	-0.57185494	0	0.026917497	0	0.12257614
0	-0.33138967	0	0.35864118	0	-0.56873882	0	0.027584026	0	0.12372623
0	-0.3306813	0	0.35917501	0	-0.56813609	0	0	0	0.12029391
0	0	0	0	0	0.098584509	0	0.005417726	0	0

0	-0.19620312	0	0.14806118	0	0	0	0	0	-0.62663235
0	-0.19078476	0	0.14793636	0	0	0	0	0	-0.053907159
0	-0.19692441	0	0.14584034	0	0	0	0	0	0
0	-0.25050515	0	0.3433551	0	-0.49098198	0	0.019089733	0	0
0	-0.16915191	0	0.18990733	0	0	0	0	0	-0.62688639
0	-0.22544442	0	0.13754623	0	0	0	-0.029969058	0	0
0	-0.19400268	0	0.15082775	0	0	0	-0.031979065	0	-0.050936634
0	-0.22469129	0	0.1361191	0	0	0	0	0	0
0	-0.19985006	0	0.14882312	0	0	0	-0.032285856	0	0
0	-0.19454427	0	0.14902544	0	0	0	0	0	-0.051088327
0	-0.17233289	0	0.19239017	0	0	0	-0.023558761	0	-0.62660756
0	-0.17313411	0	0.19052477	0	0	0	0	0	-0.62688345
0	-0.20060751	0	0.14690419	0	0	0	0	0	0
0	-0.2267249	0	0.13931469	0	0	0	-0.030812768	0	0
0	-0.1670159	0	0.19250654	0	0	0	-0.028174142	0	-0.069971681
0	-0.20515784	0	0.13615216	0	0	0	-0.10574838	0	-0.62627306
0	-0.16788285	0	0.19033125	0	0	0	0	0	-0.07035349
0	-0.18293082	0	0.16886746	0	0	0	-0.090725017	0	-0.62649074
0	-0.17261034	0	0.19128321	0	0	0	-0.028670082	0	0
0	-0.17364	0	0.18904289	0	0	0	0	0	0
0	-0.1999679	0	0.13646449	0	0	0	-0.10851699	0	-0.049046767
0	-0.20514185	0	0.13479084	0	0	0	-0.10817194	0	0
0	-0.17108226	0	0.19324991	0	0	0	-0.030546674	0	-0.068393554
0	-0.18519744	0	0.17109955	0	0	0	-0.085483894	0	-0.62635949
0	-0.22735428	0	0.12726002	0	0	0	-0.096072316	0	0
0	-0.17658825	0	0.19202255	0	0	0	-0.030826096	0	0
0	-0.19604064	0	0.14451367	0	0	0	0	0	-0.62661768
0	-0.20987223	0	0.18255701	0	0	0	-0.02596795	0	0
0	-0.32861807	0	0	0	-0.11468997	0	0	0	-0.56847383
0	-0.20236551	0	0.13809675	0	0	0	-0.10156158	0	-0.042734758
0	-0.20747399	0	0.13641765	0	0	0	-0.10081783	0	0
0	-0.17975246	0	0.17178112	0	0	0	-0.087956981	0	-0.059781665
0	-0.18452359	0	0.17110004	0	0	0	-0.086899129	0	0
0	0	0	0.367871	0	-0.22447598	0	0	0	-0.627021
0	-0.34127578	0	0	0	-0.12868688	0	0	0	-0.55594016
0	-0.33485723	0	0	0	-0.11653278	0	0	0	-0.56454995
0	-0.33466584	0	0	0	-0.11704363	0	0.006560899	0	-0.56239177
0	-0.19385551	0	0.14718155	0	0	0	-0.030106197	0	-0.052276718
0	-0.34508169	0	0	0	-0.13651014	0	0	0	0
0	-0.33599154	0	0	0	-0.11943371	0	0	0	0
0	-0.33604142	0	0	0	-0.12026304	0	0.006101122	0	0
0	-0.19438594	0	0.14549207	0	0	0	0	0	-0.052655594
0	-0.19974412	0	0.14517896	0	0	0	-0.030434724	0	0
0	0	0	0.32883611	0	-0.18430463	0	0	0	-0.62711883
0	0	0	0.33258163	0	-0.20029071	0	-0.041209726	0	-0.62691287
0	-0.33789575	0	0	0	-0.1309204	0	0	0	0.065386752
0	-0.33079893	0	0	0	-0.11760741	0	0	0	0.056509058

0	-0.32989641	0	0	0	-0.11663041	0	0.003233863	0	0.057477902
0	-0.20048154	0	0.14337135	0	0	0	0	0	0
0	-0.17120912	0	0.1907057	0	0	0	-0.021610702	0	-0.62661513
0	-0.17218112	0	0.1889349	0	0	0	0	0	-0.62664102
0	-0.22760986	0	0.13540884	0	0	0	-0.029035665	0	0
0	-0.20173921	0	0.12916723	0	0	0	0	0	-0.62670624
0	0	0	0.33308402	0	-0.22265845	0	0	0	-0.6271678
0	-0.20475635	0	0.13311591	0	0	0	-0.10100938	0	-0.62627914
0	0	0	0.36036316	0	-0.20728185	0	-0.023258131	0	-0.19092058
0	0	0	0.36093711	0	-0.21039655	0	0	0	-0.19021606
0	-0.29484079	0	0	0	-0.098955941	0	-0.013590075	0	0
0	0	0	0.36665154	0	-0.2225967	0	0	0	-0.18806944
0	-0.29404682	0	0	0	-0.096881246	0	0	0	0
0	-0.29227996	0	0	0	-0.094867969	0	-0.00922679	0	0
0	-0.34855342	0	0	0	-0.13384128	0	-0.005431228	0	0
0	-0.34910461	0	0	0	-0.13466484	0	0	0	0
0	-0.34097611	0	0	0	-0.11973555	0	0.002934103	0	0
0	0	0	0.36264404	0	-0.20527434	0	-0.022210901	0	-0.62694214
0	-0.21596053	0	0.11055508	0	0	0	0	0	-0.62647882
0	0	0	0.3616796	0	-0.20503254	0	0	0	-0.62701242
0	0	0	0.32970301	0	-0.18257913	0	-0.027354816	0	-0.1966575
0	0	0	0.36880444	0	-0.22009866	0	0	0	-0.62701631
0	-0.34137972	0	0	0	-0.1281042	0	-0.006906964	0	0.068635197
0	-0.34198203	0	0	0	-0.12907895	0	0	0	0.068077309
0	-0.33494552	0	0	0	-0.11618968	0	-0.00010483	0	0.060208238
0	0	0	0.33200429	0	-0.19693685	0	-0.041785718	0	-0.19483688
0	0	0	0.32848548	0	-0.18388679	0	0	0	-0.195651
0	-0.26813076	0	0	0	0	0	0	0	-0.62645727
0	0	0	0.3619143	0	-0.2077183	0	-0.024173851	0	0
0	0	0	0.32947722	0	-0.1766238	0	-0.024359872	0	-0.6269666
0	-0.18346423	0	0.17061079	0	0	0	-0.080377472	0	-0.62641175
0	0	0	0.36356777	0	-0.21309534	0	0	0	0
0	-0.16999271	0	0.19156347	0	0	0	-0.028721779	0	-0.070532336
0	0	0	0.36819726	0	-0.22311595	0	0	0	0
0	0	0	0.32844171	0	-0.1777215	0	0	0	-0.62713989
0	0	0	0.33228047	0	-0.19399927	0	-0.042198141	0	-0.62707841
0	-0.21081154	0	0.11026127	0	0	0	0	0	-0.025925852
0	-0.27108454	0	0	0	0	0	0	0	-3.1491663
0	-0.20199157	0	0.13500913	0	0	0	-0.097439211	0	-0.044470479
0	-0.21634654	0	0.10863953	0	0	0	0	0	0
0	-0.1755444	0	0.19033893	0	0	0	-0.029034929	0	0
0	-0.29580913	0	0	0	-0.09208409	0	-0.012162427	0	0
0	0	0	0	0	0.11787617	0	0.024174829	0	0
0	-0.20715437	0	0.13332837	0	0	0	-0.096704	0	0
0	-0.22128593	0	0.0988008	0	0	0	0	0	-0.62651618
0	0	0	0.33133603	0	-0.18423824	0	-0.029805551	0	0
0	-0.24409192	0	0.2780663	0	-0.36814822	0	0.14514537	0	-0.60118501

0	0	0	0.33336275	0	-0.1970675	0	-0.042378672	0	0
0	-0.34390318	0	0	0	-0.13800768	0	0	0	-0.55376086
0	0	0	0.33007536	0	-0.18574656	0	0	0	0
0	-0.33304552	0	0	0	-0.1175834	0	0	0	-0.56755198
0	-0.33417977	0	0	0	-0.12098831	0	0.012955244	0	-0.56320371
0	0	0	0.36187675	0	-0.20348347	0	-0.026764323	0	-0.19160989
0	0	0	0.36114285	0	-0.20229482	0	-0.025403299	0	-0.19082897
0	0	0	0.36125282	0	-0.2044746	0	0	0	-0.19057992
0	-0.27179772	0	0	0	0	0	-0.013195929	0	-0.62567413
0	0	0	0.32933609	0	-0.17591713	0	-0.029158974	0	-0.19727519
0	-0.2704638	0	0	0	0	0	-0.073553666	0	-0.62622124
0	-0.26729084	0	0	0	0	0	-0.018070534	0	-0.005173086
0	-0.27190226	0	0	0	0	0	-0.018017753	0	0
0	-0.27065833	0	0	0	0	0	-0.018470198	0	0
0	-0.26701877	0	0	0	0	0	0	0	-0.00548764
0	-0.27170077	0	0	0	0	0	0	0	0
0	0	0	0.36324636	0	-0.20363812	0	-0.027332675	0	0
0	0	0	0.36369426	0	-0.2047231	0	-0.027665549	0	0
0	0	0	0.36380606	0	-0.20711342	0	0	0	0
0	-0.25549106	0	0.282567	0	-0.38078095	0	0.13368067	0	0
0	-0.24867176	0	0.28261477	0	-0.37666966	0	0.13031868	0	0.019055354
0	-0.3517406	0	0	0	-0.14395258	0	0	0	0
0	-0.35127402	0	0	0	-0.14341952	0	-0.002894081	0	0
0	-0.34086226	0	0	0	-0.12407705	0	0.008767884	0	0
0	0	0	0.33097139	0	-0.17760284	0	-0.031340821	0	0
0	-0.27290677	0	0	0	0	0	-0.067377324	0	-0.62220523
0	-0.34464576	0	0	0	-0.13841422	0	0	0	0.070106429
0	-0.34410984	0	0	0	-0.13771611	0	-0.004386388	0	0.070967672
0	0	0	0.36052599	0	-0.20866111	0	-0.015411802	0	-0.62695833
0	-0.3347724	0	0	0	-0.12052358	0	0.005729275	0	0.059257212
0	0	0	0.36258281	0	-0.21423603	0	0	0	-0.62699933
0	0	0	0.36535745	0	-0.22001969	0	0	0	-0.62701404
0	-0.27654782	0	0	0	0	0	-0.019250138	0	0
0	-0.21953192	0	0.34280759	0	-0.39466555	0	0.16700259	0	-0.62521115
0	-0.27203131	0	0	0	0	0	-0.019537717	0	-0.001777779
0	-0.21503221	0	0.10855719	0	0	0	0	0	-0.62647181
0	-0.2729282	0	0	0	0	0	-0.069284023	0	0
0	-0.26872912	0	0	0	0	0	-0.069844707	0	-0.001577154
0	0	0	0.33128592	0	-0.1868328	0	-0.021541085	0	-0.62697579
0	0	0	0.3332226	0	-0.19899318	0	-0.034076394	0	-0.6269278
0	0	0	0.3303342	0	-0.18786515	0	0	0	-0.62705271
0	-0.29701418	0	0	0	-0.10061987	0	-0.009819565	0	0
0	-0.26720684	0	0	0	0	0	0	0	-0.61821921
0	0	0	0.36205731	0	-0.21171071	0	-0.022694296	0	-0.19106269
0	0	0	0.35999705	0	-0.20723266	0	-0.020449927	0	-0.19278791
0	0	0	0.36218366	0	-0.21370037	0	0	0	-0.19098866
0	0	0	0.24552863	0	0	0	-0.029773803	0	-0.62704514

0	-0.27038805	0	0	0	0	0	-0.011437124	0	-0.62724753
0	0	0	0.23537446	0	0	0	0	0	-0.62716506
0	-0.27060759	0	0	0	0	0	0	0	-0.62645001
0	0	0	0.24355186	0	0	0	0	0	-0.6273113
0	0	0	0.3311437	0	-0.18612324	0	-0.026372606	0	-0.19738306
0	0	0	0.36460494	0	-0.21413705	0	-0.02495296	0	0
0	0	0	0.3613698	0	-0.207382	0	-0.021022984	0	0
0	0	0	0.36472652	0	-0.21634399	0	0	0	0
0	0	0	0.23702486	0	0	0	-0.035096003	0	-0.21769553
0	0	0	0.33277611	0	-0.18781364	0	-0.028564372	0	0
0	0	0	0.24039047	0	0	0	-0.031795114	0	-0.62703402
0	0	0	0.24570706	0	0	0	-0.034398562	0	-0.21685884
0	-0.27079956	0	0	0	0	0	-0.064083145	0	-0.6251255
0	0	0	0.24797906	0	0	0	-0.031311587	0	-0.6270546
0	0	0	0.23515833	0	0	0	0	0	-0.216665
0	0	0	0.23876273	0	0	0	0	0	-0.62717079
0	-0.21034089	0	0.25297735	0	-0.29374376	0	0	0	-0.62641905
0	-0.26983499	0	0	0	0	0	-0.018037986	0	-0.004577576
0	-0.27439676	0	0	0	0	0	-0.017800375	0	0
0	0	0	0.23819214	0	0	0	-0.037880261	0	0
0	0	0	0.24659542	0	0	0	-0.036917856	0	0
0	0	0	0.2361734	0	0	0	0	0	0
0	0	0	0.24056412	0	0	0	-0.036508784	0	-0.2176097
0	0	0	0.24167321	0	0	0	-0.039019573	0	0
0	0	0	0.21998113	0	0	0	-0.12109802	0	-0.62687963
0	0	0	0.22553962	0	0	0	-0.13023506	0	-0.62672398
0	0	0	0.22579284	0	0	0	-0.12012451	0	-0.21468324
0	0	0	0.23612798	0	0	0	-0.029737689	0	-0.62703338
0	0	0	0.22893256	0	0	0	-0.1186303	0	-0.62673027
0	0	0	0.24547971	0	0	0	-0.029175479	0	-0.62705176
0	0	0	0.23459679	0	0	0	0	0	-0.62716457
0	0	0	0.22699227	0	0	0	-0.12088032	0	0
0	0	0	0.23631655	0	0	0	-0.03450225	0	-0.21910028
0	0	0	0.23742824	0	0	0	-0.037031498	0	0
0	0	0	0.22511423	0	0	0	-0.11417961	0	-0.62673803
0	-0.3115049	0	0	0	-0.089972954	0	0	0	-0.58565921
0	0	0	0.20012104	0	0	0	0	0	-0.62718258
0	-0.31611862	0	0	0	-0.090372752	0	0.019253207	0	-0.58070282
0	-0.31597408	0	0	0	-0.088469568	0	0	0	-0.58458454
0	-0.32020442	0	0	0	-0.097970196	0	0.015697163	0	0
0	-0.31997954	0	0	0	-0.096297451	0	0	0	0
0	-0.26471668	0	0	0	0	0	0	0	-0.62646268
0	-0.26413345	0	0	0	0	0	0.010665091	0	-0.62647686
0	-0.27293743	0	0	0	-0.023885826	0	0.066575677	0	-0.62279519
0	-0.31261441	0	0	0	-0.090622874	0	0	0	0.037190912
0	-0.31261841	0	0	0	-0.092021653	0	0.014447106	0	0.040340877
0	-0.26353723	0	0	0	0	0	0.034551591	0	-0.62680444

0	-0.28719864	0	0	0	-0.072019285	0	0.010112033	0	0
0	-0.25884696	0	0	0	0.009084959	0	0	0	-0.62674847
0	-0.28141916	0	0	0	-0.028416357	0	0.057273893	0	-0.6209349
0	-0.26910522	0	0	0	0	0	0.008258022	0	-0.62646431
0	-0.32439893	0	0	0	-0.096048955	0	0.013017257	0	0
0	-0.31687261	0	0	0	-0.090087097	0	0.011579627	0	0.041501633
0	0	0	0.43487948	0	-0.28928161	0	0	0	0
0	-0.26346045	0	0	0	0	0	0.003365044	0	-0.011237341
0	-0.28284397	0	0	0	-0.030185568	0	0.05762969	0	0
0	-0.26816849	0	0	0	0	0	0.003537637	0	0
0	-0.27632734	0	0	0	-0.02714917	0	0.054332483	0	-0.00127775
0	0	0	0.41872008	0	-0.25402342	0	0.018593437	0	0
0	-0.31870011	0	0	0	-0.099610163	0	0.021984216	0	-0.57849757
0	0	0	0.44236732	0	-0.29522686	0	-0.012808554	0	0
0	-0.31851215	0	0	0	-0.097360374	0	0	0	-0.58271508
0	-0.34177028	0	0	0	-0.25506217	0	0.035413198	0	-0.55649638
0	-0.34033192	0	0	0	-0.25323097	0	0	0	-0.55903818
0	0	0	0.36934387	0	-0.33122832	0	0.014573395	0	-0.62689828
0	0	0	0.3692675	0	-0.33076243	0	0	0	-0.62757146
0	0	0	0.35058641	0	-0.31480701	0	0.013086687	0	-0.62691714
0	0	0	0.43535247	0	-0.28277032	0	-0.007252943	0	0
0	0	0	0.37796694	0	-0.24249483	0	-0.010679577	0	0
0	-0.32702002	0	0	0	-0.10524486	0	0.015643691	0	0
0	-0.34892397	0	0	0	-0.26025086	0	0.031643019	0	0
0	-0.31949939	0	0	0	-0.099308811	0	0.01418974	0	0.043582052
0	-0.28035821	0	0	0	-0.031038318	0	0.064141587	0	-0.62289288
0	-0.26693756	0	0	0	0	0	0.009863605	0	-0.62646765
0	0	0	0.36872262	0	-0.33016657	0	0.010562196	0	-0.18954309
0	-0.34185883	0	0	0	-0.25458886	0	0.030043501	0	0.068473041
0	0	0	0.33732442	0	-0.27709517	0	0.060564644	0	-0.62711878
0	-0.30095172	0	0	0	-0.19486239	0	0.085961809	0	-0.59988466
0	0	0	0.37117672	0	-0.33246823	0	0.00858267	0	0
0	0	0	0.43635925	0	-0.2925348	0	-0.00448489	0	0
0	0	0	0	0	0.11689841	0	0	0	-0.62852822
0	0	0	0	0	0.11901319	0	-0.023993363	0	-0.25724072
0	0	0	0	0	0.11686215	0	0	0	-0.25453655
0	0	0	0	0	0.12494418	0	-0.02135457	0	-0.62849164
0	0	0	0	0	0.12310213	0	0	0	-0.62872617
0	0	0	0.26821222	0	0	0	-0.017334774	0	0
0	0	0	0	0	0.11900346	0	-0.027322639	0	0
0	0	0	0	0	0.1165368	0	0	0	0
0	0	0	0	0	0.1252679	0	-0.024692445	0	-0.25605989
0	-0.13418413	0	0.071220479	0	0	0	0.017078161	0	-0.62683759
0	-0.13326083	0	0.071885449	0	0	0	0	0	-0.62703432
0	0	0	0	0	0	0	0	0	-0.62845588
0	0	0	0	0	0	0	-0.006417845	0	-0.62852447
0	0	0	0	0	0.12522359	0	-0.027704127	0	0

0	0	0	0	0	0.11619543	0	-0.018626322	0	-0.62847346
0	0	0	0	0	0.11453232	0	0	0	-0.62863927
0	-0.14054807	0	0.062891814	0	0	0	-0.03328655	0	-0.62663051
0	-0.13206365	0	0.07259669	0	0	0	0.013044306	0	-0.12664073
0	0	0	0	0	0.048122652	0	-0.10908955	0	-0.62697787
0	0	0	0	0	0	0	-0.009205379	0	-0.25057557
0	-0.17713443	0	0	0	0	0	0.022606218	0	-0.62692127
0	-0.13816115	0	0.070021537	0	0	0	0.01224018	0	0
0	0	0	0	0	0.054085674	0	-0.099176309	0	-0.24804734
0	0	0	0	0	0	0	-0.063476685	0	-0.62729954
0	0	0	0	0	0.11651075	0	-0.021997026	0	-0.25650916
0	0	0	0	0	0	0	-0.006049278	0	-0.62840201
0	0	0	0	0	0.058241992	0	-0.099502748	0	-0.62697405
0	0	0	0	0	0	0	-0.012166512	0	0
0	0	0	0	0	0.05520804	0	-0.10054364	0	0
0	0	0	0	0	0.11645406	0	-0.025006348	0	0
0	0	0	0	0	0.008665563	0	0	0	-0.62745997
0	0	0	0	0	0	0	-0.004191166	0	-0.62844452
0	0	0	0	0	0.052652495	0	-0.092586215	0	-0.62698681
0	0	0	0.14241668	0	0	0	0.010994357	0	-0.62706398
0	-0.081897098	0	0.1482217	0	0	0	0.027358133	0	-0.62728188
0	-0.31319397	0	0	0	-0.226388	0	0.062991063	0	-0.58520975
0	0	0	0	0	0.13590368	0	-9.36E-05	0	-0.62854798
0	0	0	0	0	0.13591613	0	0	0	-0.6284874
0	0	0	0	0	0.13630512	0	-0.004324779	0	-0.25789796
0	0	0	0	0	0.097474273	0	-0.04502097	0	-0.62738098
0	0	0	0	0	0.14203696	0	-0.001752711	0	-0.62845634
0	0	0	0	0	0.13626062	0	-0.007356401	0	0
0	-0.32878858	0	0.34043606	0	-0.43508885	0	0	0	-0.50561474
0	0	0	0	0	0.13370227	0	0.001084411	0	-0.62848365
0	0	0	0	0	0	0	0.017344546	0	-0.62853793
0	-0.33330876	0	0.34019143	0	-0.43336082	0	0	0	-0.50438952
0	-0.33107708	0	0.35034578	0	-0.44012957	0	0	0	-0.50153793
0	-0.33408562	0	0.3520275	0	-0.44887499	0	0	0	0
0	-0.33238374	0	0.3403945	0	-0.43267386	0	-0.002867267	0	-0.50220105
0	-0.33621654	0	0.34178803	0	-0.4419544	0	0	0	0
0	-0.33523359	0	0.34206763	0	-0.4410813	0	-0.00553324	0	0
0	-0.32775526	0	0.35087152	0	-0.44284888	0	0	0	0.11995634
0	-0.32987016	0	0.34071238	0	-0.43597519	0	0	0	0.11746103
0	-0.32880037	0	0.34109399	0	-0.4349984	0	-0.007483604	0	0.11898839
0	-0.33848172	0	0.35259173	0	-0.44775828	0	0	0	0
0	-0.33958678	0	0.34194327	0	-0.43913242	0	-0.008358501	0	0
0	-0.33749658	0	0.35205322	0	-0.44620727	0	-0.005405983	0	0
0	-0.3321763	0	0.35146197	0	-0.44173319	0	0	0	0.12116307
0	-0.33323452	0	0.34097096	0	-0.43303935	0	-0.010454735	0	0.11993399
0	-0.33114406	0	0.35091171	0	-0.43998549	0	-0.0074086	0	0.12306805
0	-0.33568867	0	0.34200349	0	-0.44393511	0	0	0	-0.50232729

0	-0.33355662	0	0.35108018	0	-0.44992481	0	0	0	-0.49939066
0	-0.33483042	0	0.34210965	0	-0.44346797	0	-0.000275808	0	-0.49984421
0	0	0	0	0	-0.000941548	0	0.01804073	0	-0.62725107
0	-0.25182038	0	0.32776769	0	-0.36182591	0	-0.016528448	0	0
0	-0.25018967	0	0.33615085	0	-0.368792	0	0	0	0
0	-0.24637989	0	0.33486408	0	-0.36394427	0	-0.012532642	0	0
0	-0.26389707	0	0.3260618	0	-0.30929634	0	0.073477252	0	-0.57154409
0	-0.34097303	0	0.35333644	0	-0.45750881	0	0	0	0
0	-0.34206317	0	0.34363927	0	-0.44986333	0	-0.005845798	0	0
0	-0.34006851	0	0.35277616	0	-0.45624207	0	-0.002891257	0	0
0	-0.27441197	0	0.32709081	0	-0.3175118	0	0.061068037	0	-0.57013077
0	-0.25809781	0	0.28824934	0	-0.25954751	0	0	0	-0.58593303
0	-0.2488028	0	0.32649606	0	-0.27676047	0	0	0	-0.60025214
0	-0.27924712	0	0.30170932	0	-0.30371927	0	0.060586432	0	-0.56123558
0	-0.2520096	0	0.33611629	0	-0.3635203	0	-0.017203561	0	0
0	-0.33469598	0	0.35222193	0	-0.45154414	0	0	0	0.12318213
0	-0.33572285	0	0.34267909	0	-0.44381102	0	-0.007954662	0	0.12219198
0	-0.33372988	0	0.3516469	0	-0.45006574	0	-0.004908215	0	0.12537377
0	-0.27110254	0	0.29648882	0	-0.2793303	0	0	0	-0.58194884
0	-0.27542901	0	0.3283354	0	-0.32050404	0	0.061675272	0	0
0	-0.28113159	0	0.30134918	0	-0.30693147	0	0.060079404	0	0
0	-0.26932415	0	0.3274422	0	-0.31657532	0	0.05817606	0	0.049653027
0	-0.27469771	0	0.30116793	0	-0.30299984	0	0.057028832	0	0.058733489
0	-0.27274056	0	0.29599708	0	-0.28210522	0	0	0	0
0	-0.24366505	0	0.30524989	0	-0.29369897	0	0.034050102	0	0
0	-0.26737944	0	0.2964483	0	-0.28051151	0	0	0	0.039380292
0	-0.28924223	0	0.30561869	0	-0.31443531	0	0.050567266	0	0
0	-0.28292908	0	0.30550873	0	-0.31065395	0	0.047376882	0	0.059318467
0	-0.25308033	0	0.33664992	0	-0.37242282	0	-0.014838702	0	0
0	-0.27327077	0	0.32800039	0	-0.32095023	0	0.067845266	0	-0.57203415
0	-0.27901071	0	0.29967884	0	-0.30620951	0	0.066696437	0	-0.56226406
0	-0.26986284	0	0.29364264	0	-0.27853096	0	0	0	-0.58496847
0	-0.28936604	0	0.30381997	0	-0.31742284	0	0.056156243	0	0
0	-0.28299369	0	0.30371672	0	-0.31363913	0	0.052967959	0	0.058208353
0	0	0	0	0	0.10113853	0	0.004344295	0	0
0	-0.33110249	0	0.36175514	0	-0.57177281	0	0.032379549	0	-0.50214395
0	-0.33123178	0	0.35807531	0	-0.5686566	0	0.033033433	0	-0.50101627
0	-0.33032137	0	0.35866714	0	-0.56783159	0	0	0	-0.50485573
0	-0.33797065	0	0.36002491	0	-0.57531446	0	0.028658243	0	0
0	-0.33172045	0	0.35893881	0	-0.56922473	0	0.026569117	0	0.12242365
0	-0.19260425	0	0.14701846	0	0	0	0	0	-0.62664698
0	-0.19579656	0	0.14962527	0	0	0	-0.026569111	0	-0.62653832
0	-0.19634201	0	0.14809672	0	0	0	0	0	-0.62664105
0	-0.19041112	0	0.14974192	0	0	0	-0.031254998	0	-0.054605967
0	-0.19630314	0	0.14775061	0	0	0	-0.031614124	0	0
0	-0.19085256	0	0.14798193	0	0	0	0	0	-0.054544913
0	-0.16872768	0	0.19159157	0	0	0	-0.022848269	0	-0.62662621

0	-0.19698451	0	0.14585482	0	0	0	0	0	0
0	-0.16945344	0	0.18980236	0	0	0	0	0	-0.62688954
0	-0.2252278	0	0.1375832	0	0	0	-0.029771003	0	0
0	-0.19422492	0	0.15095926	0	0	0	-0.033143364	0	-0.052506998
0	-0.20004513	0	0.14893096	0	0	0	-0.033274676	0	0
0	-0.20186555	0	0.1349569	0	0	0	-0.10622598	0	-0.62636372
0	-0.17275383	0	0.19233118	0	0	0	-0.025116206	0	-0.62662165
0	-0.16741245	0	0.19246102	0	0	0	-0.029592536	0	-0.071758687
0	-0.20416392	0	0.13659135	0	0	0	-0.098702062	0	-0.62627527
0	-0.18171545	0	0.17013294	0	0	0	-0.085149337	0	-0.6263634
0	-0.17296568	0	0.19124215	0	0	0	-0.029920772	0	0
0	-0.19890875	0	0.13690258	0	0	0	-0.10132314	0	-0.045919371
0	-0.20404583	0	0.13523984	0	0	0	-0.10059828	0	0
0	-0.1956795	0	0.1459772	0	0	0	-0.02471274	0	-0.62653256
0	-0.1962128	0	0.14456066	0	0	0	0	0	-0.62663601
0	-0.33750063	0	0	0	-0.13070836	0	0	0	-0.55961116
0	-0.33069021	0	0	0	-0.11788681	0	0	0	-0.56827327
0	-0.33110002	0	0	0	-0.11946185	0	0.007302496	0	-0.56583665
0	0	0	0.36174918	0	-0.21003171	0	-0.019913125	0	-0.62693972
0	0	0	0.36144942	0	-0.21099373	0	0	0	-0.62694422
0	0	0	0.36698848	0	-0.2228509	0	0	0	-0.62701779
0	-0.34159854	0	0	0	-0.12888264	0	0	0	-0.55700794
0	-0.34121126	0	0	0	-0.1285401	0	-0.001250556	0	-0.55601292
0	-0.33604306	0	0	0	-0.11888467	0	0.004155171	0	-0.56305879
0	-0.19411361	0	0.14733114	0	0	0	-0.031382808	0	-0.053975851
0	-0.34484503	0	0	0	-0.13595714	0	-0.004553251	0	0
0	-0.34525784	0	0	0	-0.1366123	0	0	0	0
0	0	0	0.32984198	0	-0.18324482	0	-0.022883814	0	-0.62696388
0	-0.33715934	0	0	0	-0.12174509	0	0.004119452	0	0
0	-0.19997416	0	0.14530374	0	0	0	-0.031537636	0	0
0	0	0	0.32884731	0	-0.18435949	0	0	0	-0.62705718
0	0	0	0.33236886	0	-0.19903647	0	-0.038690546	0	-0.62691487
0	-0.3376392	0	0	0	-0.13023503	0	-0.005980512	0	0.065016932
0	-0.33811334	0	0	0	-0.13104391	0	0	0	0.064691648
0	-0.33111721	0	0	0	-0.11823648	0	0.001118105	0	0.056907916
0	-0.17169142	0	0.19064391	0	0	0	-0.023312983	0	-0.62662236
0	-0.21245262	0	0.10941902	0	0	0	0	0	-0.62647978
0	-0.203821	0	0.13350191	0	0	0	-0.094580222	0	-0.62628302
0	0	0	0.36075819	0	-0.2078887	0	-0.024028859	0	-0.19143379
0	0	0	0.36090993	0	-0.20836833	0	-0.023993542	0	-0.19080731
0	0	0	0.360939	0	-0.21039778	0	0	0	-0.19022663
0	-0.29421702	0	0	0	-0.096115035	0	-0.010726114	0	0
0	-0.34881079	0	0	0	-0.13393246	0	-0.006209557	0	0
0	0	0	0.36308858	0	-0.2059646	0	-0.023087288	0	-0.62693888
0	0	0	0.36162416	0	-0.20326752	0	-0.020845653	0	-0.62692863
0	0	0	0.36174127	0	-0.20507731	0	0	0	-0.62701581
0	0	0	0.32970224	0	-0.18257266	0	-0.027399677	0	-0.19672424

0	-0.34167711	0	0	0	-0.12820169	0	-0.007831812	0	0.067443831
0	-0.26836171	0	0	0	0	0	-0.01242604	0	-0.62639314
0	-0.26819626	0	0	0	0	0	0	0	-0.62646415
0	0	0	0.3634579	0	-0.21079464	0	-0.026252151	0	0
0	0	0	0.36212901	0	-0.20804704	0	-0.02459535	0	0
0	0	0	0.32947552	0	-0.17659984	0	-0.024560308	0	-0.62697008
0	-0.24983199	0	0.28322035	0	-0.37969842	0	0.13474425	0	-0.60391689
0	0	0	0.36348322	0	-0.21303781	0	0	0	0
0	-0.27132666	0	0	0	0	0	-0.010841082	0	-3.1253714
0	0	0	0.3313379	0	-0.18426836	0	-0.029569248	0	0
0	-0.34427046	0	0	0	-0.13821431	0	0	0	-0.55496873
0	-0.34394702	0	0	0	-0.13814909	0	0.001264729	0	-0.55367704
0	-0.33588217	0	0	0	-0.12322667	0	0.009997934	0	-0.56399965
0	-0.26819918	0	0	0	0	0	-0.066056417	0	-3.1338714
0	0	0	0.36132622	0	-0.20237501	0	-0.026048292	0	-0.19178328
0	-0.26743599	0	0	0	0	0	-0.018605005	0	-0.005870437
0	-0.27200587	0	0	0	0	0	-0.018376987	0	0
0	0	0	0.36380712	0	-0.204771	0	-0.02806315	0	0
0	-0.35156683	0	0	0	-0.1435072	0	-0.003765724	0	0
0	-0.34444343	0	0	0	-0.13781138	0	-0.005400924	0	0.069679276
0	0	0	0.36121113	0	-0.20971822	0	-0.016763876	0	-0.62695534
0	0	0	0.36253616	0	-0.21267863	0	-0.018144282	0	-0.62692563
0	0	0	0.36266673	0	-0.21429172	0	0	0	-0.62700689
0	0	0	0.33128017	0	-0.18680136	0	-0.021794224	0	-0.62696605
0	0	0	0.36225105	0	-0.2117833	0	-0.023381228	0	-0.1920742
0	0	0	0.23694184	0	0	0	-0.030805953	0	-0.62703642
0	0	0	0.24553905	0	0	0	-0.029973156	0	-0.62705206
0	-0.27087655	0	0	0	0	0	-0.012305849	0	-0.62639938
0	0	0	0.23530062	0	0	0	0	0	-0.6271616
0	0	0	0.36472948	0	-0.21418487	0	-0.025395321	0	0
0	0	0	0.23712229	0	0	0	-0.035240629	0	-0.21776885
0	0	0	0.24045036	0	0	0	-0.032088343	0	-0.62703458
0	0	0	0.2381676	0	0	0	-0.037765551	0	0
0	0	0	0.22543081	0	0	0	-0.11792856	0	-0.62674612
0	0	0	0.23620494	0	0	0	-0.030099683	0	-0.62703613
0	-0.31248867	0	0	0	-0.092536123	0	0.020189357	0	-0.58423196
0	-0.31224185	0	0	0	-0.090427525	0	0	0	-0.58786228
0	-0.3167338	0	0	0	-0.090606034	0	0.017349876	0	-0.58304873
0	-0.27779916	0	0	0	-0.030478254	0	0.058560984	0	-0.62422986
0	-0.32072466	0	0	0	-0.098147501	0	0.014168735	0	0
0	-0.26450531	0	0	0	0	0	0.009172801	0	-0.62652232
0	-0.31317454	0	0	0	-0.092205984	0	0.012776815	0	0.038307241
0	0	0	0.43490056	0	-0.28882405	0	-0.005604327	0	0
0	-0.31936572	0	0	0	-0.099826755	0	0.019958218	0	-0.58096553
0	-0.34168321	0	0	0	-0.25500226	0	0.035667254	0	-0.55616393
0	0	0	0.36915368	0	-0.33107572	0	0.015198774	0	-0.62689145
0	0	0	0	0	0.11861268	0	-0.019850142	0	-0.62876133

0	0	0	0	0	0.11682205	0	0	0	-0.62872054
0	0	0	0	0	0.11885392	0	-0.022899193	0	-0.25558397
0	0	0	0	0	0.1248335	0	-0.02039458	0	-0.62866082
0	0	0	0	0	0.11879965	0	-0.025896157	0	0
0	-0.13402249	0	0.071170032	0	0	0	0.018119282	0	-0.62683268
0	0	0	0	0	0	0	-0.004839131	0	-0.62841725
0	0	0	0	0	0.052720363	0	-0.096179243	0	-0.62697508
0	0	0	0	0	0.1160595	0	-0.017722899	0	-0.6284239
0	0	0	0	0	0.13587433	0	5.74E-05	0	-0.62847966
0	-0.3273651	0	0.35030956	0	-0.44215492	0	0	0	-0.5051225
0	-0.3294847	0	0.34037124	0	-0.43546712	0	0	0	-0.50768557
0	-0.32867803	0	0.34052566	0	-0.43492099	0	-0.001978087	0	-0.50572545
0	-0.33179587	0	0.35091183	0	-0.44106441	0	0	0	-0.50399141
0	-0.33307768	0	0.34040355	0	-0.43296824	0	-0.004935952	0	-0.50477024
0	-0.33098675	0	0.35035148	0	-0.43991516	0	-0.00190229	0	-0.50165494
0	-0.33472064	0	0.35250026	0	-0.44965709	0	0	0	0
0	-0.33385178	0	0.35202442	0	-0.4483307	0	-0.004570699	0	0
0	-0.33584202	0	0.34208097	0	-0.44132458	0	-0.007267274	0	0
0	-0.32839417	0	0.3513612	0	-0.44363913	0	0	0	0.11785695
0	-0.32946448	0	0.34110091	0	-0.43523733	0	-0.009322121	0	0.116729
0	-0.32747241	0	0.35087224	0	-0.44211421	0	-0.006529953	0	0.11954127
0	-0.3381308	0	0.35260816	0	-0.44691288	0	-0.007422565	0	0
0	-0.33180143	0	0.35149758	0	-0.44072036	0	-0.009536695	0	0.12039086
0	-0.33432853	0	0.35167109	0	-0.45087363	0	0	0	-0.50196312
0	-0.3355684	0	0.34211042	0	-0.44373686	0	-0.002442171	0	-0.50250618
0	-0.33354263	0	0.35108456	0	-0.4499605	0	0.000605006	0	-0.49932042
0	-0.25039852	0	0.33638838	0	-0.36788957	0	-0.015801877	0	0
0	-0.27074889	0	0.32718153	0	-0.3196204	0	0.062343367	0	-0.57325069
0	-0.27583294	0	0.30178974	0	-0.30634166	0	0.061372067	0	-0.56447972
0	-0.26730677	0	0.29616744	0	-0.28064127	0	0	0	-0.58535587
0	-0.28397083	0	0.30604787	0	-0.31380438	0	0.051870465	0	-0.5638464
0	-0.34074219	0	0.35334823	0	-0.45693958	0	-0.00499753	0	0
0	-0.28566767	0	0.30550385	0	-0.31650558	0	0.051709806	0	0
0	-0.33442333	0	0.35224823	0	-0.45078611	0	-0.007123645	0	0.12261064
0	-0.27933287	0	0.30539096	0	-0.31275092	0	0.048552274	0	0.056234148
0	-0.28404205	0	0.30425533	0	-0.31679594	0	0.057471191	0	-0.56494532
0	-0.33155411	0	0.35836747	0	-0.56913118	0	0.032040425	0	-0.50229052
0	-0.19223333	0	0.14854819	0	0	0	-0.025874988	0	-0.62654647
0	-0.19270519	0	0.14704506	0	0	0	0	0	-0.62665005
0	-0.19601804	0	0.14974833	0	0	0	-0.027652335	0	-0.62654186
0	-0.1906013	0	0.14985421	0	0	0	-0.032198362	0	-0.055861822
0	-0.1964607	0	0.14783762	0	0	0	-0.032377751	0	0
0	-0.16910663	0	0.19154739	0	0	0	-0.024192251	0	-0.62663149
0	-0.20071323	0	0.13541782	0	0	0	-0.098465814	0	-0.62631952
0	-0.19592609	0	0.14611505	0	0	0	-0.0259116	0	-0.62653542
0	-0.33773107	0	0	0	-0.13083978	0	0	0	-0.56038261
0	-0.33747154	0	0	0	-0.13066425	0	-0.000330946	0	-0.5596263

0	-0.3322044	0	0	0	-0.12091933	0	0.005372702	0	-0.56635869
0	0	0	0.36199121	0	-0.21035499	0	-0.020326536	0	-0.62698992
0	0	0	0.36139243	0	-0.20933694	0	-0.019442107	0	-0.62692559
0	0	0	0.36142684	0	-0.21099158	0	0	0	-0.62700519
0	-0.34150113	0	0	0	-0.12863909	0	-0.002154791	0	-0.55717366
0	-0.34502343	0	0	0	-0.13601245	0	-0.005077592	0	0
0	0	0	0.32984267	0	-0.18325535	0	-0.02280186	0	-0.62696427
0	-0.33786246	0	0	0	-0.13030096	0	-0.006653445	0	0.064163297
0	0	0	0.36101529	0	-0.20841609	0	-0.024360247	0	-0.19134719
0	0	0	0.3617768	0	-0.20333426	0	-0.02138039	0	-0.6269315
0	-0.2684743	0	0	0	0	0	-0.012875043	0	-0.62641195
0	0	0	0.36349186	0	-0.21080807	0	-0.026370538	0	0
0	-0.3442716	0	0	0	-0.1382425	0	0.00026984	0	-0.55493991
0	0	0	0.36269848	0	-0.21273959	0	-0.018721437	0	-0.62692835
0	0	0	0.23695008	0	0	0	-0.030839305	0	-0.62703895
0	-0.31302907	0	0	0	-0.092713321	0	0.018544907	0	-0.58624158
0	0	0	0	0	0.11841017	0	-0.018615698	0	-0.6284116
0	-0.32801758	0	0.35081624	0	-0.44296733	0	0	0	-0.50728579
0	-0.32930762	0	0.34053609	0	-0.43516215	0	-0.003809944	0	-0.50797336
0	-0.32727682	0	0.35034091	0	-0.44202688	0	-0.000990813	0	-0.5051397
0	-0.33163348	0	0.3509331	0	-0.44063951	0	-0.004010171	0	-0.50430986
0	-0.33442691	0	0.35251578	0	-0.44893381	0	-0.00635262	0	0
0	-0.32807212	0	0.35139435	0	-0.4427436	0	-0.008425478	0	0.1171835
0	-0.33426116	0	0.35168216	0	-0.45070244	0	-0.001603424	0	-0.50208538
0	-0.28037061	0	0.30593318	0	-0.31589331	0	0.053039525	0	-0.56693147
0	-0.19240905	0	0.14864822	0	0	0	-0.026738267	0	-0.62655129
0	-0.33768529	0	0	0	-0.13072723	0	-0.000984956	0	-0.56045446
0	0	0	0.36146675	0	-0.20936418	0	-0.019698124	0	-0.62693011
0	-0.32790227	0	0.35083236	0	-0.44266032	0	-0.002904208	0	-0.50750953

SE	bsnp_rs6469674	SE	bsnp_rs13269857	SE	bsnp_rs3020106	SE	bsnp_rs3020119	SE
0	0	0	0	0	0	0	0	0
0	0	0	0	0	0	0	0	0
0	0	0	0	0	0	0	0	0
0	0	0	0	0	0	0	0	0
0	-0.25977237	0	0	0	0	0	0	0
0	0	0	0.19949644	0	0	0	0	0
0	0	0	0	0	0	0	0	0
0	0	0	0	0	0.188965	0	0	0
0	0	0	0.28113426	0	0	0	0	0
0	0	0	0	0	0.27248051	0	0	0
0	0	0	0	0	0	0	0	0
0	0	0	0	0	0	0	0	0
0	0	0	0	0	0	0	0	0
0	0	0	0	0	0	0	0	0
0	0	0	0	0	0	0	-0.044968887	0
0	0	0	0	0	0	0	0	0
0	-0.23320124	0	0	0	0	0	0	0
0	0	0	0	0	0	0	0	0
0	0	0	0	0	0	0	0	0
0	0	0	0.18974061	0	0	0	0	0
0	-0.24787614	0	0.18966631	0	0	0	0	0
0	0	0	0	0	0	0	0	0
0	0	0	0.16557141	0	0	0	0	0
0	-0.2526942	0	0	0	0	0	0	0
0	0	0	0	0	0.17928452	0	0	0
0	0	0	0	0	0.1572566	0	0	0
0	-0.24884547	0	0	0	0.17919873	0	0	0
0	0	0	0	0	0	0	0	0
0	0	0	0	0	0	0	0	0
0	0	0	0	0	0	0	0	0
0	0	0	0	0	0	0	0	0
0	0	0	0	0	0	0	-0.089272191	0
0	-0.25435673	0	0	0	0	0	0	0
0	-0.25219366	0	0	0	0	0	0	0
0	0	0	0	0	0	0	0	0
0	0	0	0	0	0	0	0.064063314	0
0	-0.25990378	0	0	0	0	0	0	0
0	0	0	0	0	0	0	0	0
0	0	0	0	0	0	0	-0.068718836	0
0	0	0	0	0	0	0	0	0
0	-0.15034719	0	0	0	0	0	0	0
0	0	0	0	0	0	0	0	0
0	0	0	0	0	0	0	0	0
0	-0.27679829	0	0	0	0	0	-0.068163571	0
0	0	0	0	0	0	0	0	0

0	0	0	0.14558089	0	0	0	0	0
0	0	0	0.24853902	0	0	0	0	0
0	0	0	0	0	0.13323205	0	0	0
0	0.36285897	0	0	0	0	0	0	0
0	0	0	0	0	0.24069458	0	0	0
0	0	0	0.18618344	0	0	0	0	0
0	0	0	0.22221339	0	0	0	0	0
0	0	0	0	0	0	0	0.077646895	0
0	0	0	0.54836551	0	-0.35560338	0	0	0
0	0	0	0.1738086	0	0	0	0	0
0	0	0	0	0	0.21204761	0	0	0
0	0	0	0	0	0.17620236	0	0	0
0	0	0	0.17456584	0	0	0	0	0
0	0	0	0.19949267	0	0	0	-7.99E-06	0
0	0	0	0	0	0	0	0	0
0	0	0	0	0	0.16006965	0	0	0
0	0	0	0	0	0.16085655	0	0	0
0	0	0	0	0	0	0	0	0
0	0	0	0	0	0	0	0	0
0	0	0	0	0	0.18678759	0	-0.004757047	0
0	0	0	0.25022283	0	0	0	0	0
0	0	0	0.25209353	0	0	0	0	0
0	0	0	0.26550365	0	0	0	-0.038815624	0
0	0	0	0.2727952	0	0	0	0	0
0	0	0	0.54161255	0	-0.26575631	0	0	0
0	0	0	0.27844717	0	0	0	0	0
0	-0.003822369	0	0.28029002	0	0	0	0	0
0	0	0	0	0	0.23863526	0	0	0
0	0	0	0	0	0.24033508	0	0	0
0	0	0	0.17253378	0	0	0	0	0
0	0	0	0	0	0.25583608	0	-0.043543757	0
0	0	0	0	0	0	0	0	0
0	0	0	0.15937298	0	0	0	0	0
0	0	0	0	0	0.26372949	0	0	0
0	0	0	0	0	0	0	0	0
0	-0.22629317	0	0.15923093	0	0	0	0	0
0	0	0	0	0	0	0	0	0
0	0	0	0	0	0.1625686	0	0	0
0	-0.19702344	0	0	0	0	0	0	0
0	0	0	0	0	0.26882489	0	0	0
0	-0.007801751	0	0	0	0.27072049	0	0	0
0	0	0	0	0	0.15088167	0	0	0
0	-0.22658594	0	0	0	0.15072894	0	0	0
0	0	0	0	0	0	0	0.06238664	0
0	0	0	0	0	0	0	0.051701989	0
0	0	0	0	0	0	0	0	0
0	0	0	0	0	0	0	0	0

0	0	0	0	0	0	0	0	0
0	0	0	0.25725092	0	0	0	0	0
0	0	0	0	0	0	0	0.011892046	0
0	0	0	0	0	0	0	0	0
0	-0.084546012	0	0	0	0	0	0	0
0	0	0	0	0	0.24425647	0	0	0
0	0	0	0	0	0	0	0	0
0	0	0	0	0	0	0	0.030297948	0
0	0	0	0	0	0	0	0	0
0	0	0	0.24161418	0	0	0	0	0
0	0	0	0	0	0	0	0	0
0	0	0	0.20563527	0	0	0	0.11275002	0
0	0	0	0	0	0	0	0	0
0	0	0	0	0	0	0	0	0
0	0	0	0.13629957	0	0	0	0	0
0	-0.15283004	0	0	0	0	0	0	0
0	-0.24639053	0	0.13613908	0	0	0	0	0
0	0	0	0	0	0	0	0	0
0	0	0	0	0	0.22960619	0	0	0
0	0	0	0	0	0	0	0	0
0	0	0	0	0	0	0	0	0
0	0	0	0	0	0.12411156	0	0	0
0	0.3894762	0	0	0	0	0	0	0
0	0	0	0	0	0.19547028	0	0.10927741	0
0	0	0	0	0	0	0	0	0
0	0	0	0	0	0	0	0.02553203	0
0	-0.2299167	0	0	0	0	0	0	0
0	0	0	0	0	0	0	0	0
0	-0.24708754	0	0	0	0.12394279	0	0	0
0	-0.23188915	0	0	0	0	0	0	0
0	-0.22372221	0	0	0	0	0	0.02660027	0
0	-0.23552022	0	0	0	0	0	0	0
0	0	0	0	0	0	0	0	0
0	0	0	0.17580122	0	0	0	0	0
0	0	0	0	0	0	0	-0.006101087	0
0	-0.24883548	0	0.17570179	0	0	0	0	0
0	0	0	0	0	0	0	0	0
0	0	0	0.21678995	0	0	0	0	0
0	0	0	0	0	0	0	0	0
0	0	0	0	0	0	0	0.000219407	0
0	0	0	0	0	0.16595806	0	0	0
0	0	0	0.55007	0	-0.3670863	0	0	0
0	0	0	0.16552905	0	0	0	0	0
0	0	0	0.16634064	0	0	0	0	0
0	-0.2497183	0	0	0	0.16585003	0	0	0
0	0.37537218	0	0.19027914	0	0	0	0	0
0	-0.24815145	0	0.55036412	0	-0.36746306	0	0	0

0	0	0	0.1765675	0	0	0	-0.0273524	0
0	0	0	0	0	0	0	0	0
0	0	0	0	0	0.20548941	0	0	0
0	-0.24710417	0	0.16533359	0	0	0	0	0
0	-0.24622742	0	0.16652716	0	0	0	0	0
0	0	0	0	0	0	0	0	0
0	0.36996158	0	0	0	0	0	0	0
0	-0.25358356	0	0	0	0	0	0	0
0	-0.25544481	0	0.17678409	0	0	0	-0.026725655	0
0	0	0	0	0	0	0	0	0
0	0	0	0	0	0	0	0	0
0	0	0	0	0	0	0	0.034142779	0
0	0	0	0	0	0.15200009	0	0	0
0	0	0	0.42147411	0	-0.26025248	0	0	0
0	0	0	0	0	0.15274512	0	0	0
0	-0.25456867	0	0	0	0	0	0	0
0	-0.24231649	0	0	0	0	0	0.035239816	0
0	-0.25107709	0	0	0	0	0	0	0
0	0	0	0	0	0.16439252	0	-0.031894296	0
0	0	0	0	0	0	0	0	0
0	0.37438788	0	0	0	0.17983182	0	0	0
0	-0.24784468	0	0	0	0.15179707	0	0	0
0	-0.099665222	0	0	0	0	0	0	0
0	-0.24683473	0	0	0	0.15291669	0	0	0
0	0	0	0	0	0	0	0	0
0	0	0	0.24845847	0	0	0	0	0
0	-0.099561207	0	0	0	0	0	0	0
0	-0.25762398	0	0	0	0.16458992	0	-0.031273885	0
0	0	0	0	0	0	0	0	0
0	0	0	0	0	0	0	0.009378412	0
0	0	0	0	0	0	0	-0.002292355	0
0	0	0	0	0	0.23918243	0	0	0
0	0	0	0	0	0	0	-0.086722698	0
0	-0.1428186	0	0	0	0	0	-0.086852717	0
0	0	0	0	0	0	0	0	0
0	-0.16508126	0	0	0	0	0	0	0
0	0.36830491	0	0	0	0	0	0	0
0	0.37101099	0	0	0	0	0	0	0
0	0	0	0	0	0	0	0	0
0	0	0	0	0	0	0	0	0
0	0	0	0	0	0	0	0	0
0	0	0	0	0	0	0	0.001858	0
0	0	0	0	0	0	0	-0.00560178	0
0	0	0	0	0	0	0	-0.085851722	0
0	-0.25325827	0	0	0	0	0	0	0
0	-0.25475426	0	0	0	0	0	0	0
0	-0.2533272	0	0	0	0	0	0.00344593	0

0	-0.25384107	0	0	0	0	0	0	0
0	0	0	0	0	0	0	0.083089212	0
0	0.36277764	0	0	0	0	0	0	0
0	-0.25374007	0	0	0	0	0	-0.004899328	0
0	0	0	0.15209948	0	0	0	0.085651206	0
0	0	0	0	0	0	0	0	0
0	0	0	0	0	0	0	-0.02815217	0
0	0	0	0	0	0	0	0	0
0	0	0	0	0	0	0	0.05673679	0
0	0	0	0	0	0	0	0.067037684	0
0	-0.26664759	0	0	0	0	0	-0.027058365	0
0	0	0	0.16097467	0	0	0	0	0
0	0.34646477	0	0	0	0	0	-0.06848627	0
0	0	0	0	0	0.13888623	0	0.08405238	0
0	0.46964456	0	0	0	0	0	0	0
0	0	0	0.2910361	0	0	0	0	0
0	0	0	0	0	0	0	0	0
0	0	0	0	0	0	0	0	0
0	0	0	0.15846451	0	0	0	0	0
0	0	0	0.28564025	0	0	0	0	0
0	0	0	0	0	0.1471912	0	0	0
0	0	0	0.45953928	0	-0.31910885	0	0	0
0	0	0	0.20001728	0	0	0	0	0
0	0	0	0	0	0.14503564	0	0	0
0	0	0	0.14196299	0	0	0	0	0
0	0	0	0	0	0.27952894	0	0	0
0	0	0	0.25775797	0	0	0	0.041671657	0
0	0	0	0.16612591	0	0	0	0	0
0	0	0	0	0	0.27484087	0	0	0
0	0	0	0.44700169	0	-0.20202735	0	0	0
0	0	0	0.23652591	0	0	0	0	0
0	0	0	0.20857422	0	0	0	0.068789351	0
0	0	0	0.24097702	0	0	0	0	0
0	-0.023869124	0	0.24292563	0	0	0	0	0
0	0	0	0.24395989	0	0	0	0	0
0	0	0	0	0	0.12900869	0	0	0
0	0	0	0	0	0.18872278	0	0	0
0	-0.18808274	0	0.1660616	0	0	0	0	0
0	0	0	0.23630096	0	0	0	0	0
0	0	0	0.22533788	0	0	0	0	0
0	0	0	0.49635545	0	-0.31540514	0	0	0
0	0	0	0	0	0.24886552	0	0.038366204	0
0	0	0	0	0	0.1563176	0	0	0
0	0	0	0	0	0	0	0	0
0	-0.18891369	0	0	0	0.15624509	0	0	0
0	0	0	0	0	0.22497732	0	0	0
0	0	0	0	0	0.23208612	0	0	0

0	0	0	0.22289106	0	0	0	0.033490815	0
0	-0.027694914	0	0	0	0.23407551	0	0	0
0	0	0	0.43874755	0	-0.22026264	0	0	0
0	0	0	0	0	0.23379996	0	0	0
0	0	0	0.20438808	0	0	0	0	0
0	0	0	0	0	0.19727614	0	0.06504361	0
0	0.010374371	0	0.22434578	0	0	0	0	0
0	0	0	0.2410492	0	0	0	0	0
0	0	0	0	0	0.22503591	0	0	0
0	0	0	0.22268272	0	0	0	0	0
0	0	0	0.17312233	0	0	0	0.058389004	0
0	0	0	0.5084369	0	-0.3399108	0	0	0
0	0	0	0.50754518	0	-0.33800755	0	0	0
0	0	0	0.17367408	0	0	0	0.05115683	0
0	-0.2102427	0	0.24115566	0	0	0	0	0
0	0	0	0	0	0.20880988	0	0	0
0	0	0	0	0	0.21232904	0	0.031679683	0
0	0	0	0	0	0	0	0.071802922	0
0	0	0	0	0	0	0	0.070335416	0
0	0	0	0	0	0	0	0.080258697	0
0	0	0	0.55070107	0	-0.35686961	0	0.002304983	0
0	0	0	0	0	0.19005557	0	0	0
0	0	0	0	0	0	0	0	0
0	0	0	0.17295603	0	0	0	0	0
0	0.007813	0	0	0	0.21369198	0	0	0
0	0	0	0	0	0.21198422	0	0	0
0	0	0	0.22859278	0	0	0	0	0
0	0	0	0	0	0.15920793	0	0.058175108	0
0	0	0	0	0	0.22806779	0	0	0
0	0	0	0	0	0.15960227	0	0.050324998	0
0	-0.21732585	0	0.22909843	0	0	0	0	0
0	-0.21141115	0	0	0	0.22817053	0	0	0
0	0	0	0	0	0	0	0	0
0	0	0	0	0	0	0	0	0
0	0	0	0	0	0.15908801	0	0	0
0	0	0	0	0	0	0	0	0
0	0	0	0	0	0.21653519	0	0	0
0	-0.21812227	0	0	0	0.21703802	0	0	0
0	0	0	0.22734656	0	0	0	0	0
0	0	0	0.48749236	0	-0.24106303	0	0	0
0	0	0	0.49432892	0	-0.24627489	0	0	0
0	0	0	0	0	0	0	-0.087252955	0
0	0	0	0.2508933	0	0	0	-0.008388182	0
0	0	0	0.24925253	0	0	0	0	0
0	0	0	0.24991101	0	0	0	0	0
0	0.00339736	0	0.25091435	0	0	0	0	0
0	0	0	0.50340099	0	-0.24183784	0	-0.036861158	0

0	0	0	0.18586963	0	0	0	0.073230519	0
0	0	0	0.25313145	0	0	0	-0.015737742	0
0	0	0	0.2467791	0	0	0	0	0
0	0	0	0.25089821	0	0	0	0	0
0	0.002856045	0	0.25267497	0	0	0	0	0
0	0	0	0.51380103	0	-0.24530872	0	0	0
0	0	0	0.26151128	0	0	0	-0.03924413	0
0	0	0	0.26582677	0	0	0	-0.032175658	0
0	-0.009381372	0	0.26345967	0	0	0	-0.039022494	0
0	-0.19819808	0	0.18619225	0	0	0	0.074414872	0
0	0.61600465	0	0.27946674	0	0	0	0	0
0	0	0	0	0	0	0	0	0
0	0	0	0.2669437	0	0	0	0	0
0	-0.015839434	0	0.26899597	0	0	0	0	0
0	0	0	0.2060122	0	0	0	0	0
0	0	0	0.54198344	0	-0.26990674	0	0	0
0	0	0	0	0	0	0	0	0
0	-0.008351911	0	0.54180743	0	-0.26783835	0	0	0
0	0	0	0	0	0.21281256	0	0	0
0	-0.10810253	0	0	0	0	0	0	0
0	0	0	0.19710595	0	0	0	0	0
0	0	0	0.47747382	0	-0.31021405	0	0	0
0	0	0	0.1879074	0	0	0	0.046261728	0
0	0	0	0	0	0	0	0	0
0	-0.18918593	0	0	0	0	0	0	0
0	0	0	0	0	0.17571271	0	0.06962578	0
0	-0.21671613	0	0.19733311	0	0	0	0	0
0	0	0	0	0	0.23939371	0	-0.008820308	0
0	0	0	0	0	0.23709257	0	0	0
0	0	0	0	0	0	0	0	0
0	0	0	0	0	0.23821144	0	0	0
0	0.000752938	0	0	0	0.23879164	0	0	0
0	0	0	0	0	0.24159859	0	-0.016872465	0
0	0	0	0	0	0.23406901	0	0	0
0	-0.19991776	0	0	0	0.17602462	0	0.07080466	0
0	0	0	0	0	0.23851595	0	0	0
0	0	0	0.18784335	0	0	0	0	0
0	8.72E-05	0	0	0	0.24035352	0	0	0
0	0	0	0.19037751	0	0	0	0	0
0	0	0	0.43286541	0	-0.27802074	0	0	0
0	0.39631179	0	0.15983633	0	0	0	0	0
0	0	0	0	0	0	0	0	0
0	-0.19076542	0	0	0	0	0	0	0
0	0	0	0	0	0	0	0	0
0	0	0	0	0	0.25064191	0	-0.044080603	0
0	0	0	0	0	0.25616119	0	-0.035643329	0
0	-0.013631249	0	0	0	0.25263064	0	-0.043874449	0

0	0	0	0	0	0.18555459	0	0	0
0	0	0	0	0	0.1928909	0	0	0
0	0.4255561	0	0	0	0	0	0	0
0	0	0	0	0	0	0	-0.035144379	0
0	-0.22686282	0	0.4327384	0	-0.27804868	0	0	0
0	0	0	0	0	0.25648905	0	0	0
0	-0.021190463	0	0	0	0.25858156	0	0	0
0	0	0	0	0	0.17668256	0	0.042585836	0
0	-0.20023276	0	0	0	0	0	0	0
0	0.61200464	0	0	0	0.26987947	0	0	0
0	-0.21780761	0	0	0	0.18578028	0	0	0
0	-0.20538769	0	0	0	0	0	-0.034205152	0
0	0	0	0	0	0	0	-0.028152865	0
0	0.39601442	0	0	0	0.15134955	0	0	0
0	0	0	0	0	0	0	0	0
0	-0.006510916	0	0	0	0	0	0	0
0	0	0	0	0	0.17353511	0	0	0
0	0	0	0	0	0.17374267	0	0	0
0	0	0	0	0	0	0	0.066585665	0
0	0	0	0	0	0	0	0.062258697	0
0	0	0	0	0	0	0	0.054487705	0
0	0	0	0	0	0	0	0	0
0	0	0	0.25575917	0	0	0	0.050037495	0
0	0	0	0.5145117	0	-0.26153932	0	0	0
0	0	0	0.2596415	0	0	0	0	0
0	0	0	0.25886388	0	0	0	0	0
0	0	0	0.24305517	0	0	0	0.070502365	0
0	0	0	0	0	0	0	0	0
0	0.53551364	0	0	0	0	0	0	0
0	-0.089601749	0	0	0	0	0	0	0
0	0	0	0	0	0	0	0	0
0	0	0	0	0	0.24260521	0	0.049644526	0
0	0	0	0.15181762	0	0	0	0	0
0	0	0	0	0	0	0	0	0
0	-0.10375679	0	0	0	0	0	0	0
0	0	0	0	0	0	0	0.024331955	0
0	-0.088477458	0	0	0	0	0	0	0
0	-0.079468746	0	0	0	0	0	0.024895109	0
0	-0.24692018	0	0.15162895	0	0	0	0	0
0	0	0	0	0	0.24648951	0	0	0
0	0	0	0	0	0	0	0	0
0	0	0	0	0	0.24506561	0	0	0
0	0	0	0.15028063	0	0	0	0	0
0	0	0	0.492035	0	-0.25478663	0	0	0
0	0	0	0	0	0	0	0.015367695	0
0	0	0	0	0	0	0	0	0
0	0	0	0	0	0	0	0.033977868	0

0	0	0	0.25309574	0	0	0	0	0
0	0	0	0	0	0	0	0.02556756	0
0	0	0	0	0	0.13823751	0	0	0
0	0	0	0	0	0	0	0	0
0	0	0	0	0	0.23065246	0	0.069338621	0
0	0	0	0.48205703	0	-0.28073019	0	0.1134247	0
0	0	0	0.22759357	0	0	0	0.098243714	0
0	0	0	0.46095902	0	-0.32980591	0	0	0
0	-0.24881284	0	0.15034156	0	0	0	0	0
0	0	0	0	0	0	0	0.046483962	0
0	0	0	0	0	0	0	0	0
0	0.46721867	0	0	0	0	0	0	0
0	0	0	0	0	0	0	0	0
0	0	0	0.1400905	0	0	0	0.04314597	0
0	0.37694765	0	0.13681944	0	0	0	0	0
0	-0.24766275	0	0	0	0.13803727	0	0	0
0	0	0	0	0	0.13701816	0	0	0
0	-0.24665219	0	0.4611171	0	-0.33012923	0	0	0
0	-0.23460942	0	0	0	0	0	0	0
0	0	0	0	0	0	0	0	0
0	0	0	0	0	0	0	0.005093464	0
0	0	0	0	0	0	0	0	0
0	0	0	0	0	0	0	0	0
0	-0.23314048	0	0.14004958	0	0	0	0.044280403	0
0	0	0	0.13435259	0	0	0	0	0
0	-0.15755313	0	0	0	0	0	0	0
0	-0.15330979	0	0	0	0	0	0.004916474	0
0	-0.15283922	0	0	0	0	0	0	0
0	0	0	0	0	0	0	0	0
0	-0.24944527	0	0	0	0.13705964	0	0	0
0	0	0	0	0	0.12738378	0	0.041466163	0
0	0.39274991	0	0	0	0	0	0	0
0	0	0	0	0	0	0	0.030535752	0
0	0.37626948	0	0	0	0.12463454	0	0	0
0	0	0	0	0	0.23996184	0	0	0
0	0	0	0	0	0	0	0	0
0	0	0	0	0	0.21527159	0	0.096183771	0
0	-0.246167	0	0.13419403	0	0	0	0	0
0	0.39079613	0	0	0	0	0	0	0
0	0	0	0	0	0	0	0	0
0	0	0	0	0	0	0	0.042406398	0
0	0.046012685	0	0	0	0	0	0	0
0	-0.2369406	0	0	0	0	0	0	0
0	0.39864956	0	0	0	0	0	0.025680528	0
0	0	0	0	0	0	0	0	0
0	0	0	0	0	0	0	-0.009783907	0
0	0	0	0	0	0	0	0	0

0	0.38707473	0	0	0	0	0	0	0
0	0	0	0	0	0	0	0.002264272	0
0	-0.23437655	0	0	0	0.12732393	0	0.042597534	0
0	0	0	0	0	0.12162818	0	0	0
0	0	0	0.22289988	0	0	0	0	0
0	0	0	0	0	0	0	0.01332089	0
0	-0.22224058	0	0	0	0	0	0.043488707	0
0	-0.22959603	0	0	0	0	0	0	0
0	-0.2287812	0	0	0	0	0	0.003745139	0
0	0	0	0.49527928	0	-0.3246912	0	0	0
0	-0.24682019	0	0	0	0.12145897	0	0	0
0	-0.22725292	0	0	0	0	0	0.014058466	0
0	-0.25237901	0	0.22256882	0	0	0	0	0
0	0	0	0.2028736	0	0	0	0	0
0	0.37449722	0	0.17631187	0	0	0	0	0
0	-0.24903551	0	0.49552968	0	-0.32505675	0	0	0
0	0	0	0.18414185	0	0	0	0.025027288	0
0	0	0	0	0	0	0	0.009702676	0
0	-0.25236265	0	0.2035262	0	0	0	0	0
0	0	0	0.46011566	0	-0.24797432	0	0	0
0	0	0	0	0	0.20671788	0	0	0
0	-0.24164542	0	0.18446095	0	0	0	0.026237109	0
0	0	0	0	0	0	0	0.018718128	0
0	0	0	0.5102006	0	-0.34971199	0	0	0
0	0	0	0.51352121	0	-0.35249124	0	0	0
0	0	0	0	0	0	0	0	0
0	0.37371905	0	0	0	0.16646848	0	0	0
0	0	0	0	0	0	0	0.017152302	0
0	0.37505149	0	0.54975113	0	-0.36612833	0	0	0
0	0	0	0	0	0.18888941	0	0	0
0	-0.25283725	0	0	0	0.20637694	0	0	0
0	0	0	0.52468203	0	-0.35354984	0	-0.025059543	0
0	0.37620593	0	0.16607939	0	0	0	0	0
0	0.37707376	0	0.16642846	0	0	0	0	0
0	0	0	0	0	0.17297505	0	0.02116323	0
0	-0.24741376	0	0.51028246	0	-0.34999527	0	0	0
0	-0.24658175	0	0.51425438	0	-0.35304318	0	0	0
0	-0.25273908	0	0	0	0.1895391	0	0	0
0	0.36772158	0	0.17722277	0	0	0	-0.027001411	0
0	0.36905335	0	0	0	0	0	0	0
0	-0.25506769	0	0.52561688	0	-0.35428046	0	-0.024431276	0
0	0	0	0.1648214	0	0	0	0	0
0	0	0	0.16553864	0	0	0	-0.000586161	0
0	-0.24361442	0	0	0	0.1732769	0	0.022367693	0
0	0	0	0.16631419	0	0	0	-0.004924922	0
0	0	0	0	0	0	0	0.009046263	0
0	0	0	0	0	0	0	0	0

0	0	0	0	0	0	0	0	0
0	0.36829027	0	0	0	0	0	0	0
0	0.37157576	0	0	0	0	0	0	0
0	0	0	0.19685907	0	0	0	0	0
0	0.38011343	0	0	0	0	0	0.034416686	0
0	-0.24687653	0	0.16482829	0	0	0	0	0
0	-0.2467769	0	0.16531758	0	0	0	0.001097298	0
0	0.37547782	0	0	0	0.1525514	0	0	0
0	0.37690394	0	0	0	0.15282994	0	0	0
0	-0.24756017	0	0.16650871	0	0	0	-0.00419803	0
0	0	0	0	0	0	0	0	0
0	0	0	0	0	0	0	-0.030460541	0
0	0	0	0	0	0	0	0.02509125	0
0	-0.045964715	0	0	0	0	0	0	0
0	0	0	0	0	0	0	0.021625384	0
0	0	0	0	0	0	0	0	0
0	-0.25033934	0	0	0	0	0	0.010674313	0
0	-0.25333933	0	0	0	0	0	0	0
0	-0.25357328	0	0	0	0	0	0	0
0	0.52031325	0	0	0	0	0	0	0
0	0.36548334	0	0	0	0.16505657	0	-0.031534181	0
0	0	0	0	0	0.15115895	0	0	0
0	0	0	0	0	0.15201946	0	-0.000952601	0
0	0.52027746	0	0	0	0	0	0	0
0	0	0	0	0	0.15275521	0	-0.00588162	0
0	-0.24372055	0	0	0	0	0	0.026361626	0
0	-0.24743353	0	0	0	0	0	0.022431329	0
0	-0.25305774	0	0	0	0	0	0	0
0	0	0	0	0	0	0	0	0
0	0	0	0.49318532	0	-0.24927002	0	0	0
0	0	0	0.23997602	0	0	0	-0.038750023	0
0	0	0	0.22610098	0	0	0	0	0
0	0	0	0	0	0.18212964	0	0	0
0	0	0	0	0	0	0	-0.003157312	0
0	-0.097624525	0	0	0	0	0	0	0
0	0	0	0.2320972	0	0	0	0	0
0	-0.24755976	0	0	0	0.15115719	0	0	0
0	-0.24762786	0	0	0	0.15178375	0	0.000724892	0
0	-0.24846928	0	0	0	0.15292429	0	-0.005157824	0
0	-0.10022316	0	0	0	0	0	-0.002133412	0
0	0	0	0	0	0	0	-0.013337564	0
0	0.03130446	0	0.25574267	0	0	0	0	0
0	0	0	0.25383584	0	0	0	0	0
0	-0.10277714	0	0	0	0	0	-0.01268806	0
0	0	0	0	0	0	0	0.012842626	0
0	0	0	0	0	0.21324122	0	0	0
0	0	0	0	0	0.23014404	0	-0.042054838	0

0	0	0	0	0	0.22029023	0	0	0
0	0.025961642	0	0	0	0.24530463	0	0	0
0	0	0	0	0	0.24336457	0	0	0
0	0.47712103	0	0	0	0	0	-0.086795044	0
0	0	0	0	0	0	0	-0.071307602	0
0	-0.14825234	0	0	0	0	0	-0.072018629	0
0	0.45482761	0	0	0	0	0	0	0
0	0.37012482	0	0	0	0	0	0	0
0	0.13115919	0	0	0	0	0	0	0
0	0.36979573	0	0	0	0	0	0	0
0	0.36793162	0	0	0	0	0	0	0
0	0.36895975	0	0	0	0	0	0.00219577	0
0	0.36985659	0	0	0	0	0	-0.004165896	0
0	0	0	0	0	0	0	0	0
0	0	0	0.48951686	0	-0.34279866	0	0.087521495	0
0	0	0	0	0	0	0	0	0
0	0	0	0	0	0	0	0.004858665	0
0	0	0	0	0	0	0	0.001865928	0
0	0	0	0	0	0	0	-0.002238601	0
0	0	0	0.20509271	0	0	0	0	0
0	0	0	0	0	0	0	0.061534276	0
0	0	0	0.15905223	0	0	0	0.06601181	0
0	0.35576026	0	0	0	0	0	-0.028039533	0
0	-0.25134031	0	0	0	0	0	0.00628736	0
0	-0.25360991	0	0	0	0	0	0	0
0	-0.25372259	0	0	0	0	0	0.00345086	0
0	0	0	0	0	0	0	0.075601713	0
0	0	0	0.15991543	0	0	0	0.094178436	0
0	-0.25424896	0	0	0	0	0	-0.001349809	0
0	0	0	0.15638231	0	0	0	0.076485938	0
0	0	0	0.4903097	0	-0.33445143	0	0	0
0	0	0	0	0	0	0	-0.01523363	0
0	0	0	0	0	0	0	0	0
0	0	0	0	0	0	0	0	0
0	0	0	0	0	0	0	0.059183679	0
0	0	0	0	0	0	0	0	0
0	0	0	0.27420526	0	0	0	0	0
0	0	0	0	0	0.18817357	0	0	0
0	0	0	0	0	0.14503651	0	0.065925212	0
0	0	0	0.16086341	0	0	0	0	0
0	0	0	0.47945633	0	-0.32607602	0	0	0
0	0	0	0	0	0.14619075	0	0.091745881	0
0	0	0	0.27956637	0	0	0	0	0
0	0	0	0	0	0.14251159	0	0.076130315	0
0	-0.056149919	0	0.28113605	0	0	0	0	0
0	0	0	0.49707725	0	-0.20951757	0	0	0
0	0	0	0.27147056	0	0	0	0	0

0	0	0	0.29333049	0	0	0	0	0
0	0	0	0.29098544	0	0	0	0.000763832	0
0	0	0	0.17368318	0	0	0	0	0
0	0	0	0.27497206	0	0	0	0	0
0	0	0	0.48574749	0	-0.20365408	0	0	0
0	-0.050051744	0	0.27648793	0	0	0	0	0
0	0	0	0.28527488	0	0	0	0.011118345	0
0	0	0	0.46209622	0	-0.3266099	0	0	0
0	0	0	0	0	0.14695754	0	0	0
0	0	0	0	0	0.26190306	0	0	0
0	0	0	0.44981462	0	-0.25492698	0	0	0
0	0.068442636	0	0.21065864	0	0	0	0	0
0	0	0	0.20929993	0	0	0	0	0
0	0	0	0	0	0.2674148	0	0	0
0	0	0	0	0	0.15838882	0	0	0
0	0	0	0.20395732	0	0	0	0	0
0	0	0	0.47699059	0	-0.31608441	0	0	0
0	0	0	0.20385677	0	0	0	0	0
0	0	0	0.19917885	0	0	0	-0.00618733	0
0	-0.058184999	0	0	0	0.26902601	0	0	0
0	0	0	0.46860448	0	-0.2143563	0	0.042772954	0
0	0	0	0.52316514	0	-0.31975327	0	0.068868306	0
0	0	0	0	0	0.25966291	0	0	0
0	0	0	0.19094199	0	0	0	0	0
0	0.43440753	0	0.16662573	0	0	0	0	0
0	0	0	0.24773444	0	0	0	0.046876048	0
0	0.5960027	0	0.24210027	0	0	0	0	0
0	0	0	0	0	0.28168465	0	0	0
0	0	0	0.25045605	0	0	0	0.041338946	0
0	-0.18820101	0	0.47709859	0	-0.31626185	0	0	0
0	0	0	0	0	0.27952256	0	8.88E-05	0
0	-0.022411404	0	0.25241727	0	0	0	0.041438744	0
0	0	0	0.18836615	0	0	0	0	0
0	0	0	0.19298134	0	0	0	0	0
0	0	0	0	0	0.26349178	0	0	0
0	0	0	0.46777455	0	-0.2350467	0	0	0
0	-0.052375489	0	0	0	0.26505579	0	0	0
0	-0.18305924	0	0.19116556	0	0	0	0	0
0	0	0	0.44662733	0	-0.21012985	0	0	0
0	0	0	0.17121579	0	0	0	0.015021394	0
0	-0.027124534	0	0.44673674	0	-0.20825	0	0	0
0	0.065359761	0	0	0	0.19910953	0	0	0
0	0	0	0	0	0.27444008	0	0.009743899	0
0	0	0	0.44259117	0	-0.20241145	0	0	0
0	-0.19119071	0	0.18879898	0	0	0	0	0
0	0	0	0.2243659	0	0	0	0.059280403	0
0	-0.19105646	0	0.19261412	0	0	0	0	0

0	0	0	0	0	0.19771514	0	0	0
0	0	0	0.23771993	0	0	0	0	0
0	0	0	0	0	0	0	0	0
0	0.007512661	0	0.23804802	0	0	0	0	0
0	0	0	0.23671902	0	0	0	-0.002297004	0
0	0	0	0.23638209	0	0	0	0	0
0	0	0	0.21042447	0	0	0	0.067666682	0
0	0	0	0.45995136	0	-0.22739254	0	0	0
0	0	0	0.23906875	0	0	0	0	0
0	-0.021311412	0	0.24049358	0	0	0	0	0
0	-0.18379673	0	0.17151466	0	0	0	0.016072229	0
0	0	0	0.52216872	0	-0.30360367	0	0	0
0	0	0	0	0	0.18761265	0	-0.008561737	0
0	0	0	0	0	0.19058605	0	0	0
0	0	0	0	0	0.18909788	0	0	0
0	0.43358334	0	0	0	0.15681797	0	0	0
0	0	0	0.23555831	0	0	0	0.008464168	0
0	0.007472997	0	0.23781258	0	0	0	0	0
0	0	0	0.23632034	0	0	0	0	0
0	0	0	0	0	0.17789629	0	0	0
0	0.59216061	0	0	0	0.23323422	0	0	0
0	0	0	0	0	0.174381	0	0	0
0	0	0	0.50735462	0	-0.30949134	0	0	0
0	0	0	0	0	0.23695711	0	0.044618848	0
0	0	0	0	0	0.17668781	0	0	0
0	0	0	0	0	0.24044858	0	0.038047201	0
0	0	0	0	0	0	0	-0.039475522	0
0	-0.02657076	0	0	0	0.24245044	0	0.038132889	0
0	-0.18459644	0	0	0	0.17810965	0	0	0
0	0	0	0	0	0.16009722	0	0.011198748	0
0	0	0	0.2242315	0	0	0	0	0
0	0	0	0.45163125	0	-0.23265195	0	0.035179108	0
0	0	0	0.51651269	0	-0.34858577	0	0.05988614	0
0	-0.1914699	0	0	0	0.17480873	0	0	0
0	0.62644811	0	0.22311931	0	0	0	0	0
0	0	0	0.5211512	0	-0.35294349	0	0.053168275	0
0	-0.1911903	0	0	0	0.17631485	0	0	0
0	0	0	0.51425313	0	-0.277696	0	0	0
0	0.010337853	0	0	0	0	0	0	0
0	0.41231715	0	0.24163216	0	0	0	0	0
0	0	0	0	0	0	0	0	0
0	-0.18566271	0	0	0	0.16038278	0	0.012243938	0
0	0	0	0	0	0.22609759	0	0	0
0	0	0	0	0	0.22845876	0	0	0
0	0.004857404	0	0	0	0.22598506	0	0	0
0	0	0	0	0	0.22521377	0	-0.002606665	0
0	0	0	0	0	0.22427352	0	0	0

0	0.018642218	0	0.22674484	0	0	0	0.034815112	0
0	0	0	0.2250609	0	0	0	0.034242703	0
0	-0.02327506	0	0	0	0.22992369	0	0	0
0	0	0	0	0	0.20756669	0	0.058836837	0
0	0.006336631	0	0.43842834	0	-0.21861449	0	0	0
0	0	0	0.4388438	0	-0.22073212	0	0	0
0	0	0	0	0	0.19567572	0	0.066013762	0
0	-0.21081512	0	0.51438107	0	-0.27771599	0	0	0
0	0	0	0.24397693	0	0	0	0	0
0	0	0	0.23744281	0	0	0	0	0
0	0	0	0	0	0.22428492	0	0.007789993	0
0	0	0	0.24104392	0	0	0	-2.57E-06	0
0	0.004952715	0	0	0	0.22606214	0	0	0
0	0	0	0	0	0.2245176	0	0	0
0	0	0	0.17074117	0	0	0	0.060731877	0
0	0	0	0.50647827	0	-0.33861631	0	0	0
0	0	0	0	0	0	0	-0.053335579	0
0	-0.2109203	0	0.24430875	0	0	0	0	0
0	-0.21124908	0	0.23771362	0	0	0	0	0
0	-0.20976934	0	0.24114471	0	0	0	0.001566776	0
0	0.62676694	0	0	0	0.2124425	0	0	0
0	0	0	0	0	0.2073695	0	0	0
0	0.015430257	0	0	0	0.21559867	0	0.032746175	0
0	0	0	0	0	0	0	0.07275479	0
0	0	0	0	0	0.21417043	0	0.032204642	0
0	0	0	0	0	0	0	0.076905091	0
0	0	0	0.49614247	0	-0.27213441	0	0	0
0	0	0	0	0	0	0	0.070126552	0
0	0	0	0	0	0	0	0	0
0	0.41115605	0	0	0	0.2286532	0	0	0
0	-0.030311605	0	0	0	0	0	0	0
0	0.40543726	0	0.22838066	0	0	0	0	0
0	-0.21790056	0	0.4966278	0	-0.27211545	0	0	0
0	0	0	0	0	0.15664338	0	0.060669723	0
0	0	0	0.22934015	0	0	0	0.020124561	0
0	0	0	0	0	0.23085056	0	0	0
0	0	0	0.23279348	0	0	0	0	0
0	0	0	0	0	0.22351326	0	0	0
0	0	0	0	0	0.22807622	0	-0.000648128	0
0	-0.21032041	0	0.22986883	0	0	0	0.020866145	0
0	-0.21596289	0	0.23330908	0	0	0	0	0
0	-0.21205097	0	0	0	0.23118117	0	0	0
0	-0.21268557	0	0	0	0.223779	0	0	0
0	-0.21113227	0	0	0	0.22815969	0	0.000917013	0
0	0	0	0	0	0	0	0	0
0	0.40463489	0	0	0	0.21633609	0	0	0
0	0	0	0	0	0.21713605	0	0.018757504	0

0	0	0	0	0	0.21947872	0	0	0
0	0	0	0	0	0	0	-0.078254382	0
0	-0.21158873	0	0	0	0.21765489	0	0.019499244	0
0	-0.089368731	0	0	0	0	0	-0.07894908	0
0	-0.21716933	0	0	0	0.21999062	0	0	0
0	0	0	0.4768585	0	-0.25503652	0	0	0
0	0.62326251	0	0.25033444	0	0	0	0	0
0	0.053593668	0	0.22810172	0	0	0	0	0
0	0	0	0.22789858	0	0	0	0	0
0	0.62260579	0	0.25141865	0	0	0	0	0
0	0	0	0.47069557	0	-0.28923199	0	0.073850792	0
0	0	0	0.22759429	0	0	0	-0.013143854	0
0	0	0	0.22624446	0	0	0	0	0
0	0.42410851	0	0.18644025	0	0	0	0.07351548	0
0	0	0	0.486304	0	-0.23930529	0	-0.006787718	0
0	0	0	0.48799844	0	-0.2434801	0	0	0
0	0	0	0.48725066	0	-0.24116683	0	0	0
0	-0.001003046	0	0.48751261	0	-0.24129088	0	0	0
0	0	0	0.20554803	0	0	0	0.060730059	0
0	0	0	0.49419576	0	-0.25269032	0	0	0
0	0	0	0.49079437	0	-0.24175712	0	-0.013793137	0
0	0.6104113	0	0.26258293	0	0	0	-0.039140526	0
0	0	0	0.49488849	0	-0.24898619	0	0	0
0	-0.001594669	0	0.49442297	0	-0.24669874	0	0	0
0	-0.1985453	0	0.47117604	0	-0.28939342	0	0.075032518	0
0	0	0	0.50334935	0	-0.24691393	0	-0.037399151	0
0	0.60388733	0	0.26809112	0	0	0	0	0
0	0	0	0.24950135	0	0	0	-0.009130389	0
0	0	0	0.25069803	0	0	0	-0.00819953	0
0	0.001285908	0	0.25114388	0	0	0	-0.008256045	0
0	-0.013314847	0	0.50338503	0	-0.24496126	0	-0.037189859	0
0	0	0	0.50180895	0	-0.24004724	0	-0.031354242	0
0	0	0	0.24895974	0	0	0	0	0
0	0.003455402	0	0.25062577	0	0	0	0	0
0	0	0	0.24574862	0	0	0	-0.021348646	0
0	-0.19695933	0	0.20594548	0	0	0	0.061860752	0
0	0	0	0.49917349	0	-0.29923305	0	0	0
0	0.61145924	0	0.54135079	0	-0.26821014	0	0	0
0	0.5119675	0	0	0	0	0	0	0
0	0	0	0.25116701	0	0	0	-0.016622986	0
0	-0.001102831	0	0.25291475	0	0	0	-0.015838307	0
0	0.013829783	0	0.24825939	0	0	0	0	0
0	0	0	0.24714365	0	0	0	0	0
0	0	0	0.51268083	0	-0.2509882	0	0	0
0	-0.019345209	0	0.51304572	0	-0.24926122	0	0	0
0	0.43329054	0	0	0	0	0	0	0
0	0	0	0.4948402	0	-0.3121604	0	0.046185747	0

0	0	0	0.26104131	0	0	0	-0.031312465	0
0	0	0	0.46075642	0	-0.26866276	0	0	0
0	0.40582636	0	0.1976431	0	0	0	0	0
0	-0.012172092	0	0.26302593	0	0	0	-0.031665638	0
0	0	0	0.21016945	0	0	0	0.031054116	0
0	0.055566464	0	0	0	0.21382184	0	0	0
0	0.42239377	0	0	0	0.17628319	0	0.069903973	0
0	0	0	0.19648642	0	0	0	0	0
0	0	0	0	0	0.21357269	0	0	0
0	0.076127513	0	0	0	0	0	0	0
0	0.62061237	0	0	0	0.23819466	0	0	0
0	0	0	0	0	0	0	0	0
0	0	0	0.20481768	0	0	0	0	0
0	0	0	0	0	0	0	0	0
0	0	0	0	0	0.21316514	0	-0.014006443	0
0	0	0	0	0	0	0	0.002823035	0
0	0	0	0	0	0	0	0	0
0	0	0	0	0	0.19321818	0	0.058481597	0
0	0	0	0	0	0.21141915	0	0	0
0	0.43178851	0	0	0	0	0	0	0
0	-0.21746651	0	0.46086164	0	-0.26853417	0	0	0
0	0.61980005	0	0	0	0.23909616	0	0	0
0	0	0	0.48642534	0	-0.30521393	0	0	0
0	0	0	0.48830346	0	-0.30506636	0	0	0
0	0	0	0	0	0	0	0	0
0	0	0	0.19205756	0	0	0	0.043723535	0
0	-0.1856728	0	0	0	0	0	0	0
0	0	0	0.18998754	0	0	0	0.045046641	0
0	0	0	0	0	0	0	-0.005410114	0
0	0.39574024	0	0.43255614	0	-0.27722422	0	0	0
0	-0.18789257	0	0	0	0	0	0.004245807	0
0	-0.18907328	0	0	0	0	0	0	0
0	-0.19883972	0	0	0	0.19360408	0	0.05960836	0
0	0.60614128	0	0	0	0.25174162	0	-0.043984903	0
0	0	0	0	0	0	0	0	0
0	0	0	0	0	0	0	-0.010474224	0
0	0	0	0	0	0.23739628	0	-0.009833972	0
0	0	0	0	0	0.23906123	0	-0.008505939	0
0	-0.001509291	0	0	0	0.23908843	0	-0.008970922	0
0	0	0	0	0	0.23666792	0	0	0
0	0.000833739	0	0	0	0.23838329	0	0	0
0	0.4221753	0	0	0	0	0	0	0
0	0	0	0	0	0.23310553	0	-0.023159935	0
0	0.40474068	0	0	0	0.18609739	0	0	0
0	0	0	0	0	0.23896046	0	-0.018009648	0
0	0.59852822	0	0	0	0.25766914	0	0	0
0	-0.004144323	0	0	0	0.24076847	0	-0.017242281	0

0	0.012030781	0	0	0	0.23539282	0	0	0
0	0	0	0	0	0.23422842	0	0	0
0	0	0	0.19234746	0	0	0	0	0
0	0.41694721	0	0	0	0	0	-0.03509636	0
0	0.03458246	0	0	0	0	0	0	0
0	0	0	0	0	0	0	0	0
0	0	0	0	0	0	0	-0.012639478	0
0	-0.19259272	0	0	0	0	0	0	0
0	-0.1936311	0	0	0	0	0	-0.009609219	0
0	0	0	0	0	0	0	-0.020914593	0
0	0	0	0	0	0.19667169	0	0.028980492	0
0	0	0	0	0	0.25018053	0	-0.034541755	0
0	0	0	0	0	0.17992561	0	0	0
0	-0.016993808	0	0	0	0.25219971	0	-0.034910098	0
0	0	0	0	0	0.18962573	0	0	0
0	0.61313222	0	0	0	0	0	0	0
0	-0.2045059	0	0	0	0	0	-0.019971517	0
0	0	0	0	0	0.1731597	0	0.044680697	0
0	0	0	0	0	0.17743755	0	0.042130457	0
0	0	0	0	0	0	0	-0.027781743	0
0	-0.004146899	0	0	0	0	0	-0.028028024	0
0	0	0	0	0	0.17546642	0	0	0
0	0	0	0	0	0	0	0.066473231	0
0	0	0	0.52161655	0	-0.27030016	0	0.051131825	0
0	0	0	0.20236174	0	0	0	0	0
0	0	0	0.25738939	0	0	0	0.048812711	0
0	0	0	0.25600302	0	0	0	0.04996682	0
0	0	0	0.51566567	0	-0.26036055	0	0	0
0	0	0	0.51504834	0	-0.26092514	0	0	0
0	-0.25126071	0	0.20204147	0	0	0	0	0
0	0	0	0	0	0	0	0	0
0	0	0	0.26100869	0	0	0	0	0
0	0	0	0.49177894	0	-0.34507242	0	0	0
0	0	0	0.50770341	0	-0.26921432	0	0.071681287	0
0	-0.11916777	0	0	0	0	0	0	0
0	0.53031948	0	0	0	0	0	0	0
0	0.5157736	0	0	0	0	0	0	0
0	0	0	0	0	0.18581476	0	0	0
0	0	0	0	0	0	0	0	0
0	0.37630675	0	0.15231524	0	0	0	0	0
0	0.53190915	0	0	0	0	0	0	0
0	0	0	0.25008449	0	0	0	0.067220911	0
0	0.54051841	0	0	0	0	0	0.024404671	0
0	0	0	0	0	0	0	0.029755423	0
0	-0.2471848	0	0.49188016	0	-0.34536849	0	0	0
0	-0.1144525	0	0	0	0	0	0	0
0	0	0	0	0	0	0	0	0

0	0	0	0	0	0	0	0.003887923	0
0	-0.1000648	0	0	0	0	0	0.030150067	0
0	0	0	0.48260557	0	-0.33741142	0	0	0
0	-0.088928642	0	0	0	0	0	0	0
0	-0.088292375	0	0	0	0	0	0.004929647	0
0	-0.25174341	0	0	0	0.18547794	0	0	0
0	0	0	0	0	0.24405511	0	0.048561404	0
0	0	0	0	0	0.24200821	0	0.049806238	0
0	0	0	0.15246129	0	0	0	0	0
0	0	0	0.1516998	0	0	0	0.006281727	0
0	0	0	0	0	0	0	0.015416924	0
0	0	0	0.17202823	0	0	0	0	0
0	-0.084068927	0	0	0	0	0	0.01613148	0
0	0.37402875	0	0.1504781	0	0	0	0	0
0	-0.24910217	0	0.48308643	0	-0.33784348	0	0	0
0	0	0	0.4762697	0	-0.34135182	0	0.045001354	0
0	0	0	0.49897648	0	-0.25055352	0	0	0
0	0.37568639	0	0	0	0.13873664	0	0	0
0	0	0	0	0	0	0	0.017232752	0
0	-0.24730874	0	0.15245726	0	0	0	0	0
0	-0.24448999	0	0.15148196	0	0	0	0.008001502	0
0	0	0	0.49451676	0	-0.27187794	0	0.099452725	0
0	0	0	0	0	0	0	0	0
0	0	0	0.14994248	0	0	0	0.019131518	0
0	0	0	0	0	0.24707175	0	0	0
0	-0.25176966	0	0.17246772	0	0	0	0	0
0	0.37674525	0	0.46070552	0	-0.32900478	0	0	0
0	0	0	0	0	0	0	0.016918198	0
0	0	0	0	0	0	0	0.041853537	0
0	0.38786156	0	0	0	0	0	0	0
0	0	0	0	0	0	0	0.02792264	0
0	0.38923477	0	0.1406274	0	0	0	0.043459793	0
0	-0.23284063	0	0.47680306	0	-0.34193415	0	0.046135278	0
0	0	0	0	0	0.13810023	0	0.006027833	0
0	0	0	0	0	0.13878254	0	0	0
0	0	0	0	0	0.23655005	0	0.066583221	0
0	-0.24245377	0	0.14998924	0	0	0	0.019964111	0
0	0	0	0.4626109	0	-0.33465727	0	0	0
0	0	0	0	0	0	0	0.048058501	0
0	0.37339661	0	0	0	0.13722515	0	0	0
0	0	0	0	0	0	0	0	0
0	0	0	0	0	0.15706968	0	0	0
0	0.46185454	0	0	0	0	0	0	0
0	0.46674822	0	0	0	0	0	0.004842825	0
0	0	0	0	0	0	0	0.006282981	0
0	0.46776726	0	0	0	0	0	0	0
0	0	0	0	0	0	0	0	0

0	0	0	0.14386934	0	0	0	0.047431591	0
0	0.37703849	0	0.13478392	0	0	0	0	0
0	-0.24800746	0	0	0	0.13877176	0	0	0
0	-0.24532045	0	0	0	0.13786887	0	0.00773479	0
0	0	0	0	0	0.13657676	0	0.018632032	0
0	-0.24629897	0	0.46276545	0	-0.33497795	0	0	0
0	-0.25212068	0	0	0	0.15751965	0	0	0
0	0	0	0	0	0	0	0	0
0	0.38575641	0	0	0	0	0	0	0
0	-0.23227287	0	0	0	0	0	0.007786662	0
0	0.3880055	0	0	0	0.12792173	0	0.041775489	0
0	-0.23454267	0	0	0	0	0	0	0
0	0	0	0	0	0	0	-0.00029011	0
0	0	0	0	0	0	0	0.006307061	0
0	-0.23221606	0	0.14399347	0	0	0	0.048744322	0
0	0.62655073	0	0	0	0	0	0	0
0	0	0	0	0	0	0	0.022059198	0
0	-0.155989	0	0	0	0	0	0	0
0	-0.24325608	0	0	0	0.13660307	0	0.019464971	0
0	-0.15734801	0	0	0	0	0	0.00081267	0
0	-0.15146197	0	0	0	0	0	0.006707051	0
0	0.40012549	0	0	0	0	0	0.04263981	0
0	0.39308279	0	0	0	0	0	0	0
0	0	0	0	0	0.13047196	0	0.044846792	0
0	0.051154118	0	0	0	0	0	0	0
0	0	0	0.52670678	0	-0.31049759	0	0	0
0	0.39351891	0	0	0	0	0	0.002554181	0
0	0	0	0	0	0	0	0	0
0	0	0	0	0	0	0	0.029392662	0
0	0.37656922	0	0	0	0.12206771	0	0	0
0	0	0	0	0	0	0	-0.006158999	0
0	-0.22968287	0	0	0	0	0	0.022816761	0
0	0.37072989	0	0.22333352	0	0	0	0	0
0	0.39553708	0	0	0	0	0	0.014444511	0
0	0.04446527	0	0	0	0	0	-0.00688136	0
0	0	0	0	0	0	0	-0.0075312	0
0	0	0	0	0	0	0	0.003468302	0
0	-0.23363428	0	0	0	0.13057687	0	0.046153516	0
0	0.37415636	0	0.4951273	0	-0.32398068	0	0	0
0	0	0	0.51219066	0	-0.31574874	0	0	0
0	-0.25243386	0	0.52676111	0	-0.31089257	0	0	0
0	0	0	0.22348309	0	0	0	0	0
0	0	0	0.22291349	0	0	0	-0.000906397	0
0	0.37060206	0	0.2022336	0	0	0	0	0
0	0	0	0.5049863	0	-0.32603711	0	0.025069492	0
0	-0.22813449	0	0	0	0	0	0.004770649	0
0	-0.25237435	0	0.51289169	0	-0.31579603	0	0	0

0	0.38142744	0	0.18473383	0	0	0	0.025307603	0
0	0	0	0.20387067	0	0	0	0.011393034	0
0	-0.25266871	0	0.22348746	0	0	0	0	0
0	-0.25213344	0	0.22253874	0	0	0	0.000781692	0
0	-0.24183304	0	0.50568035	0	-0.32642055	0	0.026277539	0
0	0.37044002	0	0	0	0.20713731	0	0	0
0	-0.2486194	0	0.20459337	0	0	0	0.012253464	0
0	0.3759409	0	0.509973	0	-0.34891019	0	0	0
0	0.37599237	0	0.51237279	0	-0.35116174	0	0	0
0	0.37021427	0	0	0	0.1882458	0	0	0
0	0	0	0	0	0.2070169	0	0	0
0	0	0	0	0	0.20675011	0	-0.001467292	0
0	0.36803666	0	0.52462164	0	-0.35275148	0	-0.024717887	0
0	0.37949843	0	0	0	0.1735675	0	0.021440701	0
0	0	0	0	0	0	0	0.019586731	0
0	0	0	0.50970007	0	-0.34997772	0	0	0
0	0	0	0.51033017	0	-0.3498569	0	0.000904439	0
0	0	0	0.51285339	0	-0.35182865	0	-0.00291845	0
0	0.13257449	0	0.1973602	0	0	0	0	0
0	0	0	0	0	0.18968943	0	0.009649728	0
0	0	0	0.19717579	0	0	0	0	0
0	-0.25303679	0	0	0	0.20701511	0	0	0
0	-0.25277014	0	0	0	0.20637238	0	0.000224311	0
0	0.37658334	0	0.1650827	0	0	0	0	0
0	0.37616232	0	0.1660859	0	0	0	-0.000198731	0
0	0.37597574	0	0.16641292	0	0	0	-0.003483622	0
0	-0.24716195	0	0.50991432	0	-0.35019031	0	0	0
0	-0.24663987	0	0.5106539	0	-0.35041008	0	0.002582949	0
0	-0.24727733	0	0.51375434	0	-0.3525474	0	-0.002191146	0
0	-0.24953354	0	0	0	0.19040458	0	0.010510953	0
0	0.57434277	0	0	0	0	0	0	0
0	0.36933945	0	0	0	0	0	0	0
0	0.37191661	0	0	0	0	0	0.009413003	0
0	0.36904765	0	0	0	0	0	0	0
0	0	0	0.45174056	0	-0.26079882	0	0	0
0	0	0	0.16481425	0	0	0	0.000284108	0
0	0.13470359	0	0	0	0.18321334	0	0	0
0	0.3786537	0	0	0	0	0	0.025308526	0
0	0.37549275	0	0	0	0	0	0.022695252	0
0	0.3697733	0	0	0	0	0	0	0
0	0	0	0	0	0	0	0.010051614	0
0	0	0	0	0	0	0	0.009046487	0
0	0	0	0	0	0	0	0	0
0	0	0	0	0	0	0	-0.031393806	0
0	0	0	0.19713325	0	0	0	-0.017567644	0
0	0	0	0	0	0.18298452	0	0	0
0	0	0	0.19822224	0	0	0	0	0

0	0	0	0.19761656	0	0	0	0	0
0	-0.047531741	0	0	0	0	0	-0.031276123	0
0	-0.24633973	0	0.16477967	0	0	0	0.001757977	0
0	0.37590484	0	0	0	0.15141736	0	0	0
0	0.37532415	0	0	0	0.1525641	0	-0.000571296	0
0	0.37507299	0	0	0	0.15286377	0	-0.004438176	0
0	0	0	0	0	0	0	0.01924359	0
0	0.5223722	0	0	0	0	0	0	0
0	-0.24978307	0	0	0	0	0	0.011480733	0
0	-0.25032775	0	0	0	0	0	0.01067471	0
0	-0.25331755	0	0	0	0	0	0	0
0	0.51950668	0	0	0	0	0	-0.003057799	0
0	0	0	0.47639911	0	-0.25582217	0	0	0
0	0	0	0.4791476	0	-0.24348886	0	-0.038000344	0
0	0	0	0	0	0.15115462	0	7.19E-05	0
0	0.51725612	0	0	0	0	0	-0.011884278	0
0	0	0	0.4792112	0	-0.25220809	0	0	0
0	0.064688173	0	0.23269426	0	0	0	0	0
0	-0.24683682	0	0	0	0	0	0.020174889	0
0	0.62647744	0	0.25424314	0	0	0	0	0
0	0	0	0.2317851	0	0	0	0	0
0	0	0	0.22450283	0	0	0	-0.030293376	0
0	0	0	0.22850261	0	0	0	-0.032905855	0
0	0	0	0	0	0	0	0.000122436	0
0	0.050804165	0	0.23979475	0	0	0	0	0
0	0.03617825	0	0.2480213	0	0	0	-0.040316833	0
0	0	0	0.22033453	0	0	0	0	0
0	0.027846743	0	0.49424611	0	-0.24375174	0	0	0
0	0	0	0.24619548	0	0	0	-0.039945446	0
0	0	0	0.49395033	0	-0.24539733	0	0	0
0	0	0	0.23846366	0	0	0	0	0
0	0	0	0	0	0.18252743	0	-0.018266507	0
0	0	0	0	0	0.18326185	0	0	0
0	0	0	0	0	0.18278442	0	0	0
0	-0.09731364	0	0	0	0	0	0.001148613	0
0	-0.24709066	0	0	0	0.15111043	0	0.00154138	0
0	0.06290355	0	0	0	0.21984429	0	0	0
0	0	0	0	0	0.21175586	0	-0.031971378	0
0	0	0	0	0	0.21888546	0	0	0
0	0	0	0	0	0.21664052	0	-0.035135605	0
0	0	0	0	0	0.20658111	0	0	0
0	0.62646412	0	0	0	0.24377516	0	0	0
0	0.04766459	0	0	0	0.22765363	0	0	0
0	0.031391661	0	0	0	0.23721511	0	-0.043460291	0
0	0	0	0	0	0.22629361	0	0	0
0	0	0	0	0	0.23535544	0	-0.043084846	0
0	0.47162526	0	0	0	0	0	-0.071797445	0

0	0	0	0.20351169	0	0	0	0.066461626	0
0	0.62638848	0	0	0	0	0	0	0
0	0	0	0.50329411	0	-0.30529848	0	0	0
0	0	0	0.49950159	0	-0.34575294	0	0.06746357	0
0	0.37194852	0	0	0	0	0	0.005615186	0
0	0.36997273	0	0	0	0	0	0	0
0	0.13337968	0	0	0	0	0	0	0
0	0.12957137	0	0	0	0	0	-0.006956141	0
0	0.13092997	0	0	0	0	0	0	0
0	0.36947875	0	0	0	0	0	-0.00102996	0
0	0.3685868	0	0	0	0	0	0.002202528	0
0	0	0	0	0	0	0	0	0
0	0	0	0	0	0	0	-0.007469782	0
0	0	0	0	0	0	0	0	0
0	0	0	0.49393982	0	-0.34293036	0	0.078310917	0
0	0	0	0.48883269	0	-0.33537137	0	0.094635114	0
0	0	0	0	0	0	0	0.004842755	0
0	0	0	0	0	0	0	0.063381668	0
0	0	0	0.2059529	0	0	0	0	0
0	0	0	0.17779378	0	0	0	0.082666772	0
0	0	0	0	0	0.18628267	0	0.066186811	0
0	0	0	0.15821529	0	0	0	0.066876859	0
0	-0.25169584	0	0	0	0	0	0.006269412	0
0	0	0	0.49021571	0	-0.33452599	0	0	0
0	0	0	0	0	0	0	-0.013908515	0
0	0	0	0	0	0	0	-0.014932105	0
0	0	0	0	0	0	0	0	0
0	0	0	0.48540854	0	-0.21505323	0	0	0
0	0	0	0.26701182	0	0	0	0	0
0	-0.040750772	0	0.26832446	0	0	0	0	0
0	0.56321676	0	0.28061099	0	0	0	0	0
0	0	0	0.27661918	0	0	0	0	0
0	0	0	0.27408204	0	0	0	0.001743478	0
0	0	0	0	0	0.1620457	0	0.08156989	0
0	0	0	0.50091646	0	-0.22574829	0	0	0
0	0	0	0.48108656	0	-0.3144802	0	0	0
0	0	0	0	0	0.14406618	0	0.06691715	0
0	0	0	0	0	0.18878927	0	0	0
0	-0.059799897	0	0.50043553	0	-0.22365619	0	0	0
0	0	0	0.28187359	0	0	0	0	0
0	0.57057942	0	0.27516898	0	0	0	0	0
0	0	0	0.27969206	0	0	0	-0.005719214	0
0	-0.057359447	0	0.28342092	0	0	0	0	0
0	0	0	0.47382697	0	-0.20615163	0	0	0
0	0	0	0.4982192	0	-0.20841139	0	0	0
0	-0.05733399	0	0.28124392	0	0	0	-0.004747876	0
0	0	0	0.49742023	0	-0.21000296	0	0.002032444	0

0	0	0	0.2648794	0	0	0	0	0
0	0	0	0.48822019	0	-0.21767113	0	0	0
0	-0.037571889	0	0.26605664	0	0	0	0	0
0	0	0	0.27053588	0	0	0	0.015157525	0
0	-0.053452355	0	0.48804233	0	-0.2159366	0	0	0
0	0	0	0.29342236	0	0	0	-0.001106725	0
0	0	0	0.27500699	0	0	0	0.006892058	0
0	0	0	0.48871602	0	-0.20709162	0	0.012489102	0
0	0.626538	0	0.20972296	0	0	0	0	0
0	-0.048470703	0	0.27652744	0	0	0	0.007409272	0
0	0.064771404	0	0.44740058	0	-0.24221161	0	0	0
0	0	0	0	0	0.2542624	0	0	0
0	0	0	0.44775785	0	-0.24396468	0	0	0
0	-0.0421294	0	0	0	0.25561686	0	0	0
0	0.56117506	0	0	0	0.26848011	0	0	0
0	0	0	0	0	0.26418309	0	0	0
0	0	0	0	0	0.26178949	0	0.001175542	0
0	0.43429561	0	0.47678057	0	-0.31535327	0	0	0
0	0	0	0.49304584	0	-0.30822261	0	0	0
0	0	0	0.45218121	0	-0.25385368	0	0	0
0	0	0	0.45231883	0	-0.25419383	0	0	0
0	0	0	0.44822571	0	-0.25401906	0	-0.005135214	0
0	0.079473567	0	0.20443409	0	0	0	0	0
0	0.068667287	0	0.20974482	0	0	0	-0.006966651	0
0	0.070416421	0	0.20928286	0	0	0	0	0
0	0.43931328	0	0.19147692	0	0	0	0	0
0	0	0	0.20427574	0	0	0	0	0
0	0	0	0.2083982	0	0	0	-0.006844896	0
0	0	0	0.20865844	0	0	0	0	0
0	0	0	0.49051173	0	-0.30864054	0	0	0
0	0.59747783	0	0.25154083	0	0	0	0.041215539	0
0	0	0	0.49415391	0	-0.30813848	0	0	0
0	0.56819118	0	0	0	0.26372637	0	0	0
0	0.43130044	0	0.19342248	0	0	0	0	0
0	-0.18341554	0	0.49334147	0	-0.30830006	0	0	0
0	0	0	0	0	0.2695964	0	0	0
0	0.4315464	0	0.18763347	0	0	0	0	0
0	0	0	0	0	0.26758892	0	-0.006518823	0
0	0	0	0.48262244	0	-0.31663852	0	0.01494338	0
0	0	0	0	0	0.2526694	0	0	0
0	0	0	0.46068699	0	-0.21689505	0	0.048190335	0
0	0	0	0.20373054	0	0	0	-0.008885487	0
0	-0.059298793	0	0	0	0.27119193	0	0	0
0	0	0	0.20404959	0	0	0	-0.008800945	0
0	0	0	0.2048127	0	0	0	0	0
0	0	0	0.46799281	0	-0.2219784	0	0.04243359	0
0	0.59273267	0	0.44633449	0	-0.20867749	0	0	0

0	-0.038923846	0	0	0	0.25389047	0	0	0
0	0	0	0.53135253	0	-0.31399035	0	0.060542601	0
0	-0.059555433	0	0	0	0.26917746	0	-0.00555524	0
0	-0.025761964	0	0.46816745	0	-0.22015016	0	0.042525543	0
0	-0.1911097	0	0.49094576	0	-0.30864234	0	0	0
0	-0.19096094	0	0.4940549	0	-0.30841619	0	0	0
0	0	0	0.19824255	0	0	0	0	0
0	0	0	0	0	0.25866367	0	0.014116743	0
0	0.62653109	0	0	0	0.19815226	0	0	0
0	0.43847227	0	0.17177794	0	0	0	0.01521085	0
0	0	0	0.52339225	0	-0.31956434	0	0.068617884	0
0	0	0	0.19600848	0	0	0	0	0
0	0.62731736	0	0.23695816	0	0	0	0	0
0	0	0	0.19123497	0	0	0	0.001895181	0
0	0	0	0.24398651	0	0	0	0.045791741	0
0	-0.013760027	0	0.24544426	0	0	0	0.046221455	0
0	-0.18393772	0	0.48312338	0	-0.316845	0	0.015991038	0
0	0.59822423	0	0.23998195	0	0	0	0	0
0	0	0	0	0	0.28183174	0	-0.001662815	0
0	0	0	0.19570678	0	0	0	0	0
0	0	0	0.18864025	0	0	0	0.002949308	0
0	0	0	0	0	0.26347876	0	0.00540881	0
0	0	0	0.1929722	0	0	0	0.000653547	0
0	0	0	0.46835963	0	-0.23447431	0	0	0
0	0	0	0.46808058	0	-0.23641456	0	0	0
0	0.003142493	0	0.46759146	0	-0.23421517	0	0	0
0	0	0	0.46766364	0	-0.23487465	0	-0.000717268	0
0	-0.18581379	0	0.19871576	0	0	0	0	0
0	-0.051140527	0	0	0	0.26504654	0	0.005917785	0
0	-0.18476718	0	0.19575049	0	0	0	0	0
0	-0.18240028	0	0.19160818	0	0	0	0.002885104	0
0	0.081585159	0	0	0	0.19006528	0	0	0
0	0	0	0	0	0	0	-0.03083296	0
0	0	0	0.44448805	0	-0.20987181	0	0	0
0	0.06569162	0	0	0	0.19794373	0	-0.009375635	0
0	0.069673661	0	0	0	0.19610788	0	0	0
0	-0.02460531	0	0.44400209	0	-0.20792074	0	0	0
0	-0.19175564	0	0.19558057	0	0	0	0	0
0	0	0	0.22210462	0	0	0	0.060680135	0
0	0.6263953	0	0.23635151	0	0	0	0	0
0	0	0	0	0	0.18985329	0	0	0
0	-0.19005703	0	0.18915079	0	0	0	0.003818671	0
0	0.032717625	0	0	0	0	0	0	0
0	0	0	0	0	0.19656325	0	-0.009245848	0
0	-0.19038814	0	0.19258443	0	0	0	0.002176758	0
0	0	0	0	0	0.19544104	0	0	0
0	0.43776761	0	0	0	0.17842947	0	0	0

0	0	0	0	0	0	0	0	0
0	0.007270476	0	0.2391871	0	0	0	0	0
0	0	0	0.23807588	0	0	0	-0.003683095	0
0	0	0	0.23756859	0	0	0	0	0
0	0.007109522	0	0.2380963	0	0	0	-0.001549418	0
0	0	0	0.23645717	0	0	0	-0.00244061	0
0	0	0	0.46200411	0	-0.23038925	0	0.010247205	0
0	0	0	0.46018792	0	-0.22848256	0	0	0
0	0.003264977	0	0.45975449	0	-0.22651623	0	0	0
0	0.5933	0	0	0	0.24155761	0	0.03791781	0
0	0.43125307	0	0	0	0.17364032	0	0	0
0	0.43117403	0	0	0	0.17713628	0	0	0
0	0	0	0.52152151	0	-0.3042306	0	0	0
0	0	0	0	0	0.19039838	0	-0.010218778	0
0	0	0	0	0	0.18937227	0	-0.009517071	0
0	0	0	0	0	0.18983871	0	0	0
0	0.43661323	0	0	0	0.16065788	0	0.011384991	0
0	0.62679644	0	0	0	0	0	0	0
0	3.1676924	0	0.4382927	0	-0.21946786	0	0	0
0	0	0	0	0	0.18328778	0	0	0
0	0.009947813	0	0.23748491	0	0	0	0.009444505	0
0	0	0	0.23601441	0	0	0	0.008692188	0
0	0	0	0	0	0.17969039	0	0	0
0	0	0	0	0	0.17783581	0	-0.000403179	0
0	0.62509996	0	0	0	0.22538488	0	0	0
0	0.59624883	0	0	0	0.22939585	0	0	0
0	0	0	0	0	0.23269824	0	0.043445576	0
0	0.41175049	0	0.51404855	0	-0.27688414	0	0	0
0	0.62644663	0	0.22548246	0	0	0	0.034048287	0
0	0	0	0	0	0.17919444	0	0	0
0	-0.016285753	0	0	0	0.23419052	0	0.043866211	0
0	0	0	0	0	0.17449223	0	0.001257691	0
0	0	0	0	0	0.17668934	0	0.000231598	0
0	0.00857775	0	0	0	0	0	-0.039344943	0
0	0	0	0	0	0	0	-0.039508583	0
0	-0.18665555	0	0	0	0.18375661	0	0	0
0	0	0	0.51534246	0	-0.3498992	0	0.062315248	0
0	-0.1850893	0	0	0	0.17942373	0	0	0
0	-0.18446489	0	0	0	0.17819623	0	0.000589111	0
0	0.014761112	0	0.45120942	0	-0.22911794	0	0.036205165	0
0	0	0	0.45144857	0	-0.23108062	0	0.035641106	0
0	-0.19183165	0	0	0	0.17906047	0	0	0
0	-0.19083278	0	0	0	0.17500048	0	0.002106266	0
0	0	0	0.51567057	0	-0.27624719	0	0	0
0	-0.19065751	0	0	0	0.17628366	0	0.001742339	0
0	0.41174552	0	0.2441791	0	0	0	0	0
0	0.62563933	0	0	0	0.22505879	0	0	0

0	0	0	0.51226189	0	-0.27988326	0	0	0
0	0.41126308	0	0.2380375	0	0	0	0	0
0	0	0	0.51440461	0	-0.27785928	0	0.001054368	0
0	0.4124036	0	0.24162957	0	0	0	0.000295065	0
0	0	0	0	0	0.22648509	0	-0.003889318	0
0	0.004645793	0	0	0	0.22704677	0	0	0
0	0	0	0	0	0.22537778	0	0	0
0	0	0	0	0	0.22438241	0	-0.003045077	0
0	0.004298493	0	0	0	0.22606307	0	-0.002160985	0
0	0	0	0	0	0.20494491	0	0.060435789	0
0	-0.21146805	0	0.51589412	0	-0.27613721	0	0	0
0	-0.21197623	0	0.51245049	0	-0.27980119	0	0	0
0	0	0	0.24014816	0	0	0	0	0
0	-0.21001962	0	0.51472712	0	-0.27809163	0	0.002620827	0
0	0	0	0.2440486	0	0	0	-0.002680061	0
0	0	0	0.23742609	0	0	0	0.000688888	0
0	0.007142767	0	0	0	0.22570368	0	0.008489616	0
0	0	0	0	0	0.22418689	0	0.007749244	0
0	0.62642279	0	0	0	0.21431005	0	0.03199282	0
0	0	0	0	0	0	0	-0.052570521	0
0	-0.027211345	0	0	0	0	0	-0.052742082	0
0	-0.2120338	0	0.2406097	0	0	0	0	0
0	0.58926738	0	0	0	0	0	0	0
0	-0.21134117	0	0.24434537	0	0	0	-0.001367867	0
0	-0.21058344	0	0.23765665	0	0	0	0.002241435	0
0	0.40486803	0	0.49531398	0	-0.27149787	0	0	0
0	0	0	0	0	0	0	0.072570699	0
0	0	0	0.50060593	0	-0.27586982	0	0.021270149	0
0	0.41071734	0	0	0	0.23105316	0	0	0
0	0	0	0.49847515	0	-0.27070512	0	0	0
0	0.41253008	0	0.22916099	0	0	0	0.021175678	0
0	0.40973006	0	0	0	0.22410062	0	0	0
0	0.41104505	0	0	0	0.2286576	0	-0.000354656	0
0	0.40675747	0	0.23282236	0	0	0	0	0
0	-0.21051487	0	0.50123276	0	-0.27596949	0	0.022010776	0
0	-0.21669059	0	0.49896408	0	-0.27067826	0	0	0
0	0	0	0.23248333	0	0	0	0.018870381	0
0	0	0	0	0	0.22610641	0	0	0
0	0	0	0	0	0.23094689	0	-0.003176616	0
0	0	0	0	0	0.22350612	0	0.000223124	0
0	-0.20971535	0	0.23298657	0	0	0	0.019625974	0
0	-0.21342756	0	0	0	0.22656607	0	0	0
0	-0.21262298	0	0	0	0.23123809	0	-0.001864285	0
0	-0.2121579	0	0	0	0.22372334	0	0.001772024	0
0	0.41125479	0	0	0	0.21695753	0	0.019814696	0
0	0.40554366	0	0	0	0.21952085	0	0	0
0	0.53067572	0	0	0	0	0	-0.078728168	0

0	0	0	0	0	0.2190704	0	0.017982252	0
0	0.626282	0	0.22845526	0	0	0	0	0
0	0.050034027	0	0.46959385	0	-0.24690797	0	0	0
0	0.61884084	0	0.4875333	0	-0.24189606	0	0	0
0	-0.2112235	0	0	0	0.21956623	0	0.018735597	0
0	0.42376738	0	0.4705125	0	-0.28845888	0	0.074129084	0
0	0	0	0.47117826	0	-0.24872646	0	0	0
0	0.61815259	0	0.49325127	0	-0.24677636	0	0	0
0	0	0	0.47456863	0	-0.25247253	0	-0.011690068	0
0	0	0	0.47620491	0	-0.25561812	0	0	0
0	0	0	0.4820094	0	-0.28154617	0	0.061872749	0
0	0.42534864	0	0.20611858	0	0	0	0.061003519	0
0	0.60646454	0	0.50282056	0	-0.24527616	0	-0.037306603	0
0	0.62090659	0	0.25058941	0	0	0	-0.009150254	0
0	0.6233494	0	0.24966595	0	0	0	0	0
0	0.051379146	0	0.2282527	0	0	0	-0.009764583	0
0	0.054847297	0	0.22663632	0	0	0	0	0
0	0.62658978	0	0.24627328	0	0	0	0	0
0	0	0	0.22806354	0	0	0	-0.010453852	0
0	0.61884651	0	0.25165569	0	0	0	-0.015178727	0
0	0	0	0.22655891	0	0	0	0	0
0	-0.19736131	0	0.48252806	0	-0.28167143	0	0.063002032	0
0	0	0	0.22671693	0	0	0	-0.012563957	0
0	0.60038201	0	0.51239983	0	-0.24954189	0	0	0
0	0	0	0.48677306	0	-0.24201121	0	-0.007940174	0
0	0	0	0.4862034	0	-0.23948048	0	-0.006522432	0
0	-0.002787086	0	0.48642111	0	-0.23997695	0	-0.00706935	0
0	0	0	0.48899542	0	-0.24837333	0	-0.020010844	0
0	0	0	0.48779394	0	-0.24361813	0	0	0
0	-0.000934693	0	0.48730906	0	-0.24141828	0	0	0
0	0.010578541	0	0.49356518	0	-0.25089245	0	0	0
0	0	0	0.49419172	0	-0.25267952	0	0	0
0	0	0	0.49123799	0	-0.24501593	0	-0.015013019	0
0	0.60756104	0	0.26213291	0	0	0	-0.031650144	0
0	-0.005093247	0	0.4909741	0	-0.24295626	0	-0.014248295	0
0	0	0	0.50690894	0	-0.3027657	0	0.031692098	0
0	0.40508162	0	0.46047895	0	-0.26781367	0	0	0
0	0	0	0.50126503	0	-0.24520535	0	-0.0303335	0
0	0	0	0.49190851	0	-0.30247498	0	0	0
0	-0.01574728	0	0.50147609	0	-0.24339438	0	-0.030690732	0
0	0.62649577	0	0	0	0.21412081	0	0	0
0	0	0	0.24933046	0	0	0	-0.008949134	0
0	0.00137585	0	0.2509624	0	0	0	-0.008054597	0
0	0.012335915	0	0.24707884	0	0	0	-0.021055573	0
0	0	0	0.24595609	0	0	0	-0.021306005	0
0	0	0	0.49780648	0	-0.29974672	0	0	0
0	0.62639089	0	0	0	0	0	0	0

0	0.43662384	0	0	0	0	0	0	0
0	0.43350063	0	0	0	0	0	0	0
0	0	0	0.19568918	0	0	0	0.038789972	0
0	0.43432473	0	0	0	0	0	0.003075022	0
0	0.42338504	0	0	0	0.1937943	0	0.058767993	0
0	0	0	0.49632833	0	-0.31090308	0	0.044512801	0
0	0	0	0.49484759	0	-0.31215233	0	0.046176475	0
0	0	0	0.2058481	0	0	0	0.033559701	0
0	0.057039221	0	0	0	0.21206837	0	0	0
0	0.053191148	0	0	0	0.21404908	0	-0.010537817	0
0	0.07850937	0	0	0	0	0	0	0
0	0	0	0	0	0.21381503	0	-0.0112166	0
0	0	0	0.19775768	0	0	0	0	0
0	0	0	0	0	0.21194256	0	0	0
0	0.61810585	0	0	0	0.23852852	0	-0.009848655	0
0	0.076062954	0	0	0	0	0	-0.000276793	0
0	0.62070433	0	0	0	0.23739493	0	0	0
0	0	0	0	0	0	0	0	0
0	0	0	0	0	0	0	0.001816709	0
0	0	0	0	0	0	0	0	0
0	0	0	0	0	0.21201302	0	-0.013253998	0
0	0	0	0	0	0	0	0.003490716	0
0	0.62666258	0	0	0	0.23335646	0	0	0
0	0.43015382	0	0	0	0	0	0	0
0	0.42900714	0	0	0	0	0	-0.009357412	0
0	0	0	0	0	0	0	-0.000912164	0
0	0.61576838	0	0	0	0.23949725	0	-0.016567944	0
0	0	0	0.48921798	0	-0.30412383	0	0	0
0	0.62658545	0	0	0	0	0	0	0
0	0	0	0	0	0	0	-0.003713113	0
0	-0.18543213	0	0	0	0	0	0	0
0	-0.1847494	0	0	0	0	0	0.00316987	0
0	0	0	0.19116288	0	0	0	0.044232582	0
0	-0.18761112	0	0	0	0	0	0.004735037	0
0	0.60271952	0	0	0	0.25129666	0	-0.034884705	0
0	0.41771077	0	0	0	0	0	-0.020790574	0
0	0	0	0	0	0	0	-0.008566054	0
0	0	0	0	0	0.23712184	0	-0.009531555	0
0	-0.001368619	0	0	0	0.23879176	0	-0.008647459	0
0	0.010475304	0	0	0	0.23426469	0	-0.02292358	0
0	0	0	0	0	0.23309649	0	-0.02316213	0
0	0	0	0	0	0.17894833	0	0.038494554	0
0	0.033481145	0	0	0	0	0	-0.011586546	0
0	0	0	0	0	0	0	-0.011935245	0
0	0	0	0	0	0.19047288	0	0.03256987	0
0	-0.19463961	0	0	0	0	0	-0.007671148	0
0	0.61549731	0	0	0	0	0	-0.028110039	0

0	0	0	0	0	0.18096568	0	0	0
0	0	0	0	0	0.17409116	0	0.044072591	0
0	0	0	0.50674008	0	-0.31135936	0	0	0
0	0.37207709	0	0.20273839	0	0	0	0	0
0	0	0	0.52218755	0	-0.26934104	0	0.049989069	0
0	0	0	0.52149183	0	-0.27046327	0	0.051209377	0
0	-0.25128969	0	0.5068115	0	-0.31175853	0	0	0
0	0	0	0.20469437	0	0	0	0	0
0	0	0	0.20224228	0	0	0	0.005699772	0
0	0.50012569	0	0	0	0	0	0	0
0	0	0	0.25750898	0	0	0	0.048780245	0
0	0	0	0.5160824	0	-0.25987236	0	0	0
0	0.37610675	0	0.49152375	0	-0.34433366	0	0	0
0	-0.25211999	0	0.20468664	0	0	0	0	0
0	-0.24900691	0	0.20189696	0	0	0	0.007424129	0
0	0.37161807	0	0	0	0.18619362	0	0	0
0	0	0	0	0	0	0	0	0
0	0	0	0	0	0	0	0.003791982	0
0	0.50570274	0	0	0	0	0	0	0
0	0	0	0.5112055	0	-0.26614644	0	0.068636058	0
0	0.51949227	0	0	0	0	0	0.029746921	0
0	0	0	0.49280241	0	-0.3462566	0	0.00773528	0
0	0	0	0.49212298	0	-0.34481937	0	0	0
0	0.53112926	0	0	0	0	0	0	0
0	-0.11848556	0	0	0	0	0	0	0
0	-0.11785902	0	0	0	0	0	0.004888527	0
0	0.53139593	0	0	0	0	0	0.003990953	0
0	0	0	0	0	0.18793531	0	0	0
0	0.37374976	0	0.48194897	0	-0.33653519	0	0	0
0	0	0	0	0	0.18567623	0	0.00529898	0
0	0	0	0.48514813	0	-0.32007014	0	0	0
0	0	0	0	0	0	0	0.017033356	0
0	0.53638759	0	0	0	0	0	0.016454707	0
0	0.37831226	0	0.15219214	0	0	0	0.006682227	0
0	0.37621187	0	0.15269877	0	0	0	0	0
0	-0.10993772	0	0	0	0	0	0.017658646	0
0	-0.24431201	0	0.49312952	0	-0.34680889	0	0.009445492	0
0	-0.24754795	0	0.49233328	0	-0.34503806	0	0	0
0	0	0	0.48632748	0	-0.34156933	0	0.020954351	0
0	0	0	0	0	0	0	0.005153778	0
0	0.37115843	0	0.17159197	0	0	0	0	0
0	-0.25175606	0	0.48567867	0	-0.32014627	0	0	0
0	-0.25250744	0	0	0	0.1879202	0	0	0
0	-0.087223282	0	0	0	0	0	0.006171002	0
0	-0.24959483	0	0	0	0.18530327	0	0.007018818	0
0	0.38954139	0	0.47610249	0	-0.34062506	0	0.045309212	0
0	0	0	0	0	0.24336497	0	0.04874955	0

0	0	0	0.17332196	0	0	0	0.025575227	0
0	0	0	0.15229595	0	0	0	0.005518769	0
0	0.38045028	0	0.15011939	0	0	0	0.020211377	0
0	-0.24215965	0	0.48694046	0	-0.34214761	0	0.021783331	0
0	0	0	0.47604038	0	-0.33851458	0	0.047861326	0
0	-0.24357071	0	0.17380688	0	0	0	0.026468387	0
0	0.37749281	0	0	0	0.13859319	0	0.006424687	0
0	0.37531827	0	0	0	0.13904476	0	0	0
0	0	0	0	0	0	0	0.018684714	0
0	-0.24514754	0	0.15224849	0	0	0	0.00699045	0
0	0.37695246	0	0.46235445	0	-0.33396734	0	0	0
0	0	0	0	0	0	0	0.043603309	0
0	0.38984524	0	0	0	0	0	0.006591684	0
0	0.3880786	0	0	0	0	0	0	0
0	0.37080456	0	0	0	0.15663161	0	0	0
0	0.39013061	0	0.14438167	0	0	0	0.047709294	0
0	-0.23221255	0	0.47655802	0	-0.3389168	0	0.049170865	0
0	0	0	0	0	0.13859972	0	0.005396959	0
0	0	0	0	0	0.15817231	0	0.024362707	0
0	0.37964962	0	0	0	0.13676016	0	0.019711967	0
0	0.46372648	0	0	0	0	0	0	0
0	0.46180036	0	0	0	0	0	-0.000118559	0
0	0.46925126	0	0	0	0	0	0.007284428	0
0	0	0	0	0	0	0	0.006779026	0
0	-0.24429804	0	0	0	0.15865491	0	0.025266847	0
0	-0.24588591	0	0	0	0.13854172	0	0.006866652	0
0	0.39307796	0	0	0	0	0	0.023059097	0
0	0.62650254	0	0	0	0	0	0	0
0	0.38870834	0	0	0	0.13098305	0	0.045125913	0
0	0	0	0	0	0	0	0.001363067	0
0	-0.2320693	0	0	0	0	0	0.008092011	0
0	0.37083457	0	0.52641561	0	-0.30979623	0	0	0
0	0.62634646	0	0	0	0	0	-0.007865211	0
0	-0.15534524	0	0	0	0	0	0.002405934	0
0	0.39444609	0	0	0	0	0	0.004090511	0
0	0.050528819	0	0	0	0	0	-0.002588335	0
0	0	0	0.52693144	0	-0.31028437	0	0	0
0	0	0	0.52676166	0	-0.31056098	0	0.000374363	0
0	0	0	0	0	0	0	-0.003383881	0
0	0.37058898	0	0.51125322	0	-0.3154361	0	0	0
0	0.37103829	0	0.22344256	0	0	0	0	0
0	0.37069653	0	0.22332062	0	0	0	-0.0005009	0
0	0.38053087	0	0.50486737	0	-0.3253296	0	0.025324703	0
0	0	0	0.51495714	0	-0.31746054	0	0.012374782	0
0	-0.25267691	0	0.52716078	0	-0.31051385	0	0	0
0	-0.25181181	0	0.5270598	0	-0.31122826	0	0.002071925	0
0	0	0	0.22353265	0	0	0	-0.001367104	0

0	0.37437694	0	0.20330826	0	0	0	0.012384468	0
0	-0.24832858	0	0.51584157	0	-0.3176247	0	0.01323579	0
0	-0.252644	0	0.22348453	0	0	0	8.41E-05	0
0	0.37008613	0	0	0	0.2071593	0	-0.001064587	0
0	0.37049283	0	0	0	0.20698614	0	0	0
0	0.3763128	0	0.50926496	0	-0.34928302	0	0	0
0	0.37633633	0	0.51015456	0	-0.3491034	0	0.001283155	0
0	0.37627969	0	0.51212976	0	-0.35096845	0	-0.001489011	0
0	0.62649206	0	0.19773766	0	0	0	0	0
0	0.12901431	0	0.43262887	0	-0.24080948	0	0	0
0	0.37345304	0	0	0	0.18911984	0	0.010650199	0
0	0	0	0	0	0.20708958	0	-0.001715409	0
0	0	0	0.43410087	0	-0.24249453	0	0	0
0	0	0	0.50995826	0	-0.35029574	0	0.001870318	0
0	0.13305848	0	0.19829505	0	0	0	0	0
0	0.13046869	0	0.19748611	0	0	0	-0.009148371	0
0	0.1320958	0	0.19796019	0	0	0	0	0
0	0	0	0.19731192	0	0	0	-0.009699101	0
0	0	0	0.19810214	0	0	0	0	0
0	0	0	0.19796424	0	0	0	0	0
0	-0.25311808	0	0	0	0.20702608	0	-0.000265375	0
0	0.37590779	0	0.16506707	0	0	0	0.00097568	0
0	0.57284288	0	0	0	0	0	-0.030671764	0
0	-0.24614167	0	0.51036658	0	-0.35074237	0	0.00334074	0
0	0.62647871	0	0	0	0.1835502	0	0	0
0	0.37266251	0	0	0	0	0	0.010718346	0
0	0.36936584	0	0	0	0	0	0	0
0	0.37181064	0	0	0	0	0	0.009395958	0
0	0	0	0.44874176	0	-0.2574831	0	-0.016151807	0
0	0	0	0.45235269	0	-0.26012339	0	0	0
0	0	0	0.45249928	0	-0.26080514	0	0	0
0	0.13246492	0	0	0	0.18340476	0	-0.009779805	0
0	0.3759936	0	0	0	0	0	0.020187086	0
0	0.13514161	0	0	0	0.1840541	0	0	0
0	0.13442495	0	0	0	0.18357112	0	0	0
0	0	0	0	0	0	0	0.010052589	0
0	0	0	0.19883859	0	0	0	-0.018778573	0
0	0	0	0.19799656	0	0	0	-0.018062418	0
0	0	0	0	0	0.18318772	0	-0.01032084	0
0	0	0	0	0	0.18381543	0	0	0
0	0	0	0	0	0.18353261	0	0	0
0	0	0	0.19885858	0	0	0	0	0
0	0.62669983	0	0.2309382	0	0	0	0	0
0	0.37599966	0	0	0	0.1514093	0	0.000788814	0
0	0.061485588	0	0.47281708	0	-0.24577619	0	0	0
0	0.52250572	0	0	0	0	0	0.000638572	0
0	0.62647858	0	0.24656621	0	0	0	-0.040203948	0

0	0.6264756	0	0.49359491	0	-0.24464322	0	0	0
0	0.62646304	0	0.23885745	0	0	0	0	0
0	0	0	0.47356229	0	-0.24747233	0	0	0
0	0	0	0.46917233	0	-0.25001749	0	-0.029029148	0
0	-0.24976267	0	0	0	0	0	0.011481036	0
0	0	0	0.47035932	0	-0.24675085	0	-0.031896191	0
0	0	0	0.47094719	0	-0.25629113	0	0	0
0	0.062774128	0	0.23094368	0	0	0	-0.028923216	0
0	0.047395216	0	0.47774737	0	-0.2434014	0	0	0
0	0.072022567	0	0.22592704	0	0	0	0	0
0	0.032652379	0	0.47982358	0	-0.23678339	0	-0.039421658	0
0	0	0	0.47804914	0	-0.24506607	0	0	0
0	0	0	0.47967824	0	-0.2385051	0	-0.039057919	0
0	0	0	0.23002597	0	0	0	-0.029203403	0
0	0	0	0.22523801	0	0	0	0	0
0	0.051378219	0	0.23623534	0	0	0	-0.033243366	0
0	0	0	0.23490942	0	0	0	-0.033170126	0
0	0	0	0.21984324	0	0	0	-0.02803417	0
0	0	0	0	0	0.18403124	0	-0.019322819	0
0	0	0	0	0	0.18330102	0	-0.018709045	0
0	0	0	0	0	0.18381626	0	0	0
0	0.62754139	0	0	0	0.21804604	0	0	0
0	0.62625693	0	0	0	0.2267117	0	0	0
0	0.62646322	0	0	0	0.23573826	0	-0.043337364	0
0	0.060951655	0	0	0	0.21818443	0	-0.030702285	0
0	0.071060494	0	0	0	0.21237019	0	0	0
0	0	0	0	0	0.21722478	0	-0.030971377	0
0	0	0	0	0	0.21164275	0	0	0
0	0.04838489	0	0	0	0.22406666	0	-0.035503429	0
0	0	0	0	0	0.20630615	0	-0.029297303	0
0	0	0	0	0	0.22271142	0	-0.035421959	0
0	0	0	0.51261096	0	-0.31645706	0	0.067730044	0
0	0	0	0.20327429	0	0	0	0.066616969	0
0	0.62644646	0	0	0	0	0	0	0
0	0.62662473	0	0	0	0	0	-0.007834873	0
0	0.62634107	0	0	0	0	0	0	0
0	0	0	0.49735697	0	-0.32681003	0	0.083734222	0
0	0	0	0.49912887	0	-0.34629862	0	0.068409087	0
0	0	0	0.50364239	0	-0.30490794	0	0	0
0	0.37159566	0	0	0	0	0	0.005611862	0
0	0.13220835	0	0	0	0	0	-0.004829244	0
0	0.13318892	0	0	0	0	0	0	0
0	0.1294116	0	0	0	0	0	-0.00673347	0
0	0	0	0	0	0	0	-0.005513906	0
0	0	0	0	0	0	0	0	0
0	0	0	0	0	0	0	-0.007246086	0
0	0	0	0	0	0.18576601	0	0.066519429	0

0	0.57849428	0	0.26793166	0	0	0	0	0
0	0	0	0.48967273	0	-0.2272614	0	0	0
0	-0.044409108	0	0.4889526	0	-0.22518751	0	0	0
0	0	0	0	0	0	0	-0.013679327	0
0	0.55954772	0	0.50047487	0	-0.22422794	0	0	0
0	0	0	0.48658287	0	-0.21384855	0	0	0
0	0	0	0.26940516	0	0	0	0	0
0	0	0	0.48590213	0	-0.21577734	0	0.003081847	0
0	-0.041884587	0	0.27068777	0	0	0	0	0
0	0	0	0.26711959	0	0	0	-0.003319611	0
0	0.56235267	0	0.28245413	0	0	0	0	0
0	0.56178417	0	0.2807388	0	0	0	-0.005686098	0
0	-0.041362245	0	0.26840078	0	0	0	-0.002324956	0
0	0	0	0.27665372	0	0	0	-0.000387532	0
0	0	0	0.50217513	0	-0.22473624	0	0	0
0	0.58273598	0	0.26512798	0	0	0	0	0
0	0	0	0.5002176	0	-0.22493039	0	-0.004710895	0
0	0.56716807	0	0.48699529	0	-0.21621158	0	0	0
0	-0.06096062	0	0.50173522	0	-0.22270267	0	0	0
0	-0.060727514	0	0.49987082	0	-0.22299705	0	-0.00374101	0
0	0	0	0.47746348	0	-0.21707997	0	0	0
0	-0.040996848	0	0.47695893	0	-0.21535946	0	0	0
0	0	0	0.28217777	0	0	0	-0.007980195	0
0	0.57221133	0	0.27521052	0	0	0	0.007872204	0
0	0	0	0.47732927	0	-0.21078393	0	0.016607772	0
0	-0.059184715	0	0.28369282	0	0	0	-0.007063119	0
0	0.57711048	0	0	0	0.25520262	0	0	0
0	0	0	0.49825347	0	-0.20846501	0	0.000222729	0
0	0.62652879	0	0.44739436	0	-0.2431798	0	0	0
0	0	0	0.26457056	0	0	0	0.011447205	0
0	-0.034526565	0	0.2657336	0	0	0	0.012101571	0
0	0	0	0.49004581	0	-0.21949084	0	0.008090177	0
0	-0.051648899	0	0.48998135	0	-0.21786498	0	0.008599794	0
0	0.43894675	0	0.49284829	0	-0.30747535	0	0	0
0	0.62651242	0	0.20481821	0	0	0	0	0
0	0.62652499	0	0.20879728	0	0	0	-0.007117061	0
0	0.62652006	0	0.20803156	0	0	0	0	0
0	0	0	0	0	0.25655201	0	0	0
0	0.075821676	0	0.44072719	0	-0.24180352	0	0	0
0	0.064970574	0	0.44555107	0	-0.24111602	0	-0.005898746	0
0	0.066998219	0	0.44598295	0	-0.2423611	0	0	0
0	0	0	0	0	0.25440773	0	-0.003976502	0
0	-0.043182688	0	0	0	0.25787975	0	0	0
0	0.43141742	0	0.49391624	0	-0.30743041	0	0	0
0	0.43162591	0	0.48951782	0	-0.30837841	0	0	0
0	0	0	0.442367	0	-0.24363393	0	0	0
0	0	0	0.44594871	0	-0.24289467	0	-0.005774467	0

0	0	0	0.44700789	0	-0.24405017	0	0	0
0	-0.042907472	0	0	0	0.25572992	0	-0.002990306	0
0	0.56039121	0	0	0	0.27019576	0	0	0
0	0.55952973	0	0	0	0.26864877	0	-0.006471464	0
0	0	0	0	0	0.26426005	0	-0.000843917	0
0	0.59411339	0	0.46764038	0	-0.22049597	0	0.042316748	0
0	0.43833703	0	0.48247403	0	-0.31590857	0	0.015129415	0
0	0.58135445	0	0	0	0.25295269	0	0	0
0	0	0	0.49725797	0	-0.30572533	0	0	0
0	0	0	0.49597988	0	-0.30693077	0	0	0
0	0	0	0.45044421	0	-0.25227484	0	-0.007471902	0
0	0	0	0.49367326	0	-0.30847138	0	0.002490194	0
0	0	0	0.45095496	0	-0.25264114	0	-0.007329559	0
0	0	0	0.45273197	0	-0.25374728	0	0	0
0	0.079059238	0	0.20492987	0	0	0	0	0
0	0.078662769	0	0.20450256	0	0	0	-0.003451653	0
0	0.069727284	0	0.20905997	0	0	0	-0.006476632	0
0	0.43702502	0	0.19781289	0	0	0	0	0
0	0.60575064	0	0.24488289	0	0	0	0.045779385	0
0	0.43741869	0	0.19646597	0	0	0	0	0
0	0	0	0.49542944	0	-0.30679327	0	0	0
0	0.439774	0	0.19179057	0	0	0	0.002065479	0
0	0	0	0.20490876	0	0	0	0	0
0	0	0	0.20435969	0	0	0	-0.004126181	0
0	0	0	0.20842239	0	0	0	-0.006862434	0
0	0	0	0.49133371	0	-0.30911627	0	0.003809818	0
0	0	0	0.49440041	0	-0.30841662	0	0.001866126	0
0	0.62296755	0	0.46762656	0	-0.23484883	0	0	0
0	-0.18592895	0	0.49770715	0	-0.30570189	0	0	0
0	0.43080363	0	0.19571602	0	0	0	0	0
0	0.56951585	0	0	0	0.26371417	0	0.006392428	0
0	-0.18479923	0	0.49593708	0	-0.30715302	0	0	0
0	0	0	0	0	0.26997001	0	-0.008651054	0
0	-0.18262591	0	0.49421119	0	-0.30863895	0	0.003480816	0
0	0.43164999	0	0.19342879	0	0	0	0.000988426	0
0	0.43281668	0	0.18799868	0	0	0	0.003924711	0
0	0	0	0.46164947	0	-0.22224768	0	0.046973008	0
0	3.1479547	0	0.23723177	0	0	0	-0.002945441	0
0	0	0	0	0	0.25231417	0	0.010341324	0
0	-0.017018634	0	0.46133512	0	-0.22043964	0	0.047397292	0
0	-0.061277606	0	0	0	0.27152802	0	-0.00774262	0
0	0	0	0.20518529	0	0	0	-0.009606872	0
0	0	0	0.53043545	0	-0.31555253	0	0.062069184	0
0	-0.036198015	0	0	0	0.25351678	0	0.010986979	0
0	0.59491493	0	0.44395746	0	-0.20839695	0	0	0
0	-0.19162558	0	0.4954545	0	-0.30695141	0	0	0
0	0.62668707	0	0	0	0	0	0	0

0	-0.18970938	0	0.49194767	0	-0.30922253	0	0.0046599	0
0	0.62650419	0	0	0	0.19040401	0	0	0
0	-0.18992614	0	0.49450377	0	-0.30892129	0	0.00338359	0
0	0.62651879	0	0	0	0.19697458	0	-0.009524143	0
0	0.62653449	0	0	0	0.19482665	0	0	0
0	0	0	0.19812627	0	0	0	-0.002989571	0
0	0	0	0.19830302	0	0	0	0	0
0	0	0	0.19603445	0	0	0	-0.001285572	0
0	0.62728054	0	0.23779215	0	0	0	0	0
0	0	0	0.19575849	0	0	0	-0.001541899	0
0	0.62361585	0	0.45913612	0	-0.22683772	0	0	0
0	0	0	0.46866451	0	-0.23584437	0	0	0
0	0.002918629	0	0.46818724	0	-0.23369876	0	0	0
0	0	0	0.46805106	0	-0.23396066	0	-0.002038397	0
0	0	0	0.46789726	0	-0.23618741	0	-0.001289177	0
0	0.003039207	0	0.46753428	0	-0.23414407	0	-0.00040202	0
0	-0.18638653	0	0.19863393	0	0	0	-0.002122797	0
0	-0.18563952	0	0.19825826	0	0	0	0	0
0	-0.18472244	0	0.1957475	0	0	0	0.000152942	0
0	0.029910854	0	0	0	0	0	-0.029895604	0
0	0	0	0	0	0	0	-0.030254333	0
0	0.080640942	0	0	0	0.19016857	0	-0.00407453	0
0	0.081373414	0	0	0	0.19033056	0	0	0
0	0.068853318	0	0	0	0.19589253	0	-0.007901646	0
0	-0.19182917	0	0.19558845	0	0	0	-0.000236373	0
0	0.62624422	0	0.23602307	0	0	0	0.009447681	0
0	0	0	0	0	0.18997706	0	-0.004739044	0
0	0	0	0	0	0.19025971	0	0	0
0	0.43617511	0	0	0	0.18285698	0	0	0
0	0	0	0	0	0.19521509	0	-0.008281059	0
0	0.43710469	0	0	0	0.18014989	0	0	0
0	0.43771508	0	0	0	0.17839442	0	-0.000231462	0
0	0.006493371	0	0.23931749	0	0	0	-0.002981116	0
0	0.62704863	0	0	0	0	0	-0.038925393	0
0	0	0	0.23773612	0	0	0	-0.003874339	0
0	0.60322489	0	0	0	0.23362078	0	0.043430381	0
0	0.006061125	0	0.46180577	0	-0.22899111	0	0.010832369	0
0	0	0	0.46210528	0	-0.23080119	0	0.010094891	0
0	0.4307313	0	0	0	0.17920101	0	0	0
0	0.43192876	0	0	0	0.17383737	0	0.002245583	0
0	0.43132314	0	0	0	0.17712727	0	0.000487648	0
0	0	0	0	0	0.1903077	0	-0.010173083	0
0	0.62641606	0	0.45101946	0	-0.23023206	0	0.035450604	0
0	0	0	0	0	0.18177616	0	0	0
0	0	0	0	0	0.18314743	0	-0.004152703	0
0	0	0	0	0	0.17973274	0	-0.001663863	0
0	0.41119911	0	0.51528787	0	-0.27564878	0	0	0

0	0.6250272	0	0	0	0.22610894	0	0	0
0	0.41043907	0	0.5120521	0	-0.27907082	0	0	0
0	0.62390266	0	0	0	0.2254896	0	-0.00306302	0
0	0.41215695	0	0.51423492	0	-0.27708436	0	0.001347657	0
0	0	0	0	0	0.17926006	0	-0.001769292	0
0	-0.18587956	0	0	0	0.18172102	0	0	0
0	-0.18754004	0	0	0	0.18364621	0	-0.00328478	0
0	-0.18516001	0	0	0	0.17942215	0	-0.000231276	0
0	0	0	0.51358027	0	-0.27854291	0	0	0
0	-0.19197627	0	0	0	0.17907839	0	-0.00046561	0
0	0.41058031	0	0.24039027	0	0	0	0	0
0	0	0	0.51547205	0	-0.27600395	0	-0.001537154	0
0	0.62642975	0	0	0	0.22422113	0	0.008496518	0
0	0.41110281	0	0.24423485	0	0	0	-0.002080591	0
0	0	0	0.51250017	0	-0.28017838	0	0.001855506	0
0	0.41145299	0	0.2380025	0	0	0	0.000979814	0
0	0.59235956	0	0	0	0	0	-0.052837799	0
0	0	0	0	0	0.22558996	0	-0.004373815	0
0	0.00373918	0	0	0	0.22721738	0	-0.00349298	0
0	-0.21273317	0	0.51386693	0	-0.27836452	0	0	0
0	-0.21153687	0	0.51586573	0	-0.27610239	0	-0.000224148	0
0	-0.21096093	0	0.51288555	0	-0.28033988	0	0.003403338	0
0	0	0	0.24023435	0	0	0	-0.002005723	0
0	-0.21224927	0	0.24063968	0	0	0	-0.000706831	0
0	0.4123359	0	0.49997786	0	-0.27540609	0	0.022319302	0
0	0.4060347	0	0.49779086	0	-0.26997597	0	0	0
0	0	0	0.50204274	0	-0.27468102	0	0.020228738	0
0	0.40918547	0	0	0	0.22634694	0	0	0
0	0.41306188	0	0.23249297	0	0	0	0.019844324	0
0	0.40982344	0	0	0	0.23112863	0	-0.002575659	0
0	0.40988324	0	0	0	0.2240849	0	0.000515756	0
0	-0.21002012	0	0.50265577	0	-0.27479247	0	0.020982705	0
0	0	0	0	0	0.22621684	0	-0.002331044	0
0	-0.21374066	0	0	0	0.22661819	0	-0.001033713	0
0	0.41155188	0	0	0	0.21908631	0	0.018957883	0
0	0.62647344	0	0.47089887	0	-0.24790614	0	0	0
0	0.42495288	0	0.48182192	0	-0.28076624	0	0.062144182	0
0	0.62651979	0	0.22653862	0	0	0	0	0
0	0.6264887	0	0.22861142	0	0	0	-0.010829846	0
0	0.051351571	0	0.46855476	0	-0.24743679	0	0	0
0	0.048110147	0	0.4681872	0	-0.24533577	0	-0.008545791	0
0	0.61681357	0	0.48630538	0	-0.24042481	0	-0.007962767	0
0	0.61892658	0	0.4870988	0	-0.24218613	0	0	0
0	0.62666113	0	0.4925348	0	-0.25191736	0	0	0
0	0	0	0.46966675	0	-0.24703404	0	-0.009233658	0
0	0	0	0.47025142	0	-0.24924754	0	0	0
0	0.61484658	0	0.48996681	0	-0.24320171	0	-0.013579632	0

0	0	0	0.47415286	0	-0.25309322	0	-0.011007026	0
0	0.60397857	0	0.50086801	0	-0.24367632	0	-0.030671926	0
0	0.62661581	0	0.2451154	0	0	0	-0.020775998	0
0	0.62113754	0	0.25002191	0	0	0	-0.008535397	0
0	0.052703715	0	0.22695103	0	0	0	-0.008806585	0
0	0	0	0.22690158	0	0	0	-0.009611206	0
0	0	0	0.49810409	0	-0.30975071	0	0.040083621	0
0	0	0	0.48667812	0	-0.2421566	0	-0.007687381	0
0	0.009199869	0	0.4884942	0	-0.24685119	0	-0.019795002	0
0	-0.002669794	0	0.48631923	0	-0.24011992	0	-0.006799692	0
0	0	0	0.48905727	0	-0.24858087	0	-0.020037603	0
0	0	0	0.50419988	0	-0.30517432	0	0.034644328	0
0	0.62652284	0	0	0	0.21192552	0	0	0
0	0	0	0.49248917	0	-0.30189407	0	0	0
0	0.6264831	0	0	0	0.21437319	0	-0.011593311	0
0	0.62646521	0	0	0	0	0	0	0
0	0.62642696	0	0	0	0	0	-0.001218532	0
0	0.4370274	0	0	0	0	0	0	0
0	0.4373897	0	0	0	0	0	0.002014073	0
0	0.43475287	0	0	0	0	0	0.00405374	0
0	0	0	0.1963447	0	0	0	0.038331944	0
0	0	0	0.49524759	0	-0.3115248	0	0.045472285	0
0	0.054767196	0	0	0	0.21246151	0	-0.009380603	0
0	0.079004624	0	0	0	0	0	0.002002486	0
0	0	0	0	0	0.21236906	0	-0.010178167	0
0	0.62663351	0	0	0	0.23225838	0	-0.022618935	0
0	0.61836996	0	0	0	0.23782397	0	-0.009121883	0
0	0	0	0	0	0	0	0.002710942	0
0	0	0	0	0	0	0	0.001229536	0
0	0.42811046	0	0	0	0	0	-0.007664494	0
0	0.62656801	0	0	0	0	0	-0.011433472	0
0	-0.18428696	0	0	0	0	0	0.003806011	0
0	0	0	0	0	0.17937183	0	0.038188193	0
0	0.37200982	0	0.50642889	0	-0.31070585	0	0	0
0	0	0	0.5077828	0	-0.31017693	0	0	0
0	0	0	0.50765856	0	-0.3124499	0	0.006997505	0
0	0.37149983	0	0.20467528	0	0	0	0	0
0	0.37388308	0	0.20261685	0	0	0	0.006133673	0
0	0	0	0.52201787	0	-0.26956692	0	0.050092034	0
0	-0.25209662	0	0.50800056	0	-0.31040842	0	0	0
0	-0.24863287	0	0.50796418	0	-0.31311907	0	0.008719173	0
0	0	0	0.20454032	0	0	0	0.003882046	0
0	0.50101208	0	0	0	0	0	0	0
0	0.50118354	0	0	0	0	0	0.003921157	0
0	0.37849786	0	0.49259102	0	-0.34557299	0	0.00813025	0
0	0.37598349	0	0.49171015	0	-0.34421391	0	0	0
0	-0.25046152	0	0.20447765	0	0	0	0.005337763	0

0	0.371106	0	0	0	0.18791789	0	0	0
0	0.37323012	0	0	0	0.18605035	0	0.005765514	0
0	0.51028468	0	0	0	0	0	0.017996063	0
0	0	0	0	0	0	0	0.004742073	0
0	0.37117679	0	0.48442294	0	-0.31977517	0	0	0
0	0	0	0.49301037	0	-0.34593299	0	0.007058213	0
0	0.53270978	0	0	0	0	0	0.005674671	0
0	-0.11686529	0	0	0	0	0	0.0057916	0
0	0	0	0.4901162	0	-0.32376722	0	0.026665079	0
0	0.3807505	0	0.48584705	0	-0.34089011	0	0.022028819	0
0	0	0	0	0	0.18776984	0	0.003670713	0
0	0.37803831	0	0.1525161	0	0	0	0.006278591	0
0	0.37934545	0	0.17290989	0	0	0	0.026433362	0
0	-0.24490732	0	0.49340359	0	-0.34637981	0	0.008528636	0
0	-0.24320624	0	0.49082676	0	-0.32399235	0	0.027568618	0
0	-0.25091455	0	0	0	0.18768891	0	0.005123852	0
0	0.39013421	0	0.47587023	0	-0.33781155	0	0.048140132	0
0	0.37734423	0	0	0	0.13881853	0	0.006143339	0
0	0.37861012	0	0	0	0.15776134	0	0.02523686	0
0	0.39033938	0	0	0	0	0	0.007400923	0
0	0.46422812	0	0	0	0	0	0.001875266	0
0	0.62652163	0	0	0	0	0	-0.003312777	0
0	0.37089328	0	0.52640116	0	-0.30981174	0	0	0
0	0.3710301	0	0.52651962	0	-0.30991274	0	0.000779644	0
0	0	0	0.5269375	0	-0.31029222	0	5.24E-05	0
0	0.37466343	0	0.5142278	0	-0.31727901	0	0.013372703	0
0	0.37060597	0	0.22348266	0	0	0	-0.000602155	0
0	-0.25221499	0	0.52735769	0	-0.3107708	0	0.001502951	0
0	0.62648109	0	0.43393964	0	-0.24177499	0	0	0
0	0.37015603	0	0	0	0.20703381	0	-0.000944402	0
0	0.37705087	0	0.50960401	0	-0.34970692	0	0.002597348	0
0	0.62648508	0	0.19788079	0	0	0	-0.010058694	0
0	0.62644683	0	0.19864582	0	0	0	0	0
0	0.12949463	0	0.43351898	0	-0.24076936	0	0	0
0	0.62644818	0	0.19800468	0	0	0	0	0
0	0.12718638	0	0.43139266	0	-0.23943283	0	-0.007994831	0
0	0.12860634	0	0.43297268	0	-0.24062831	0	0	0
0	0	0	0.43499579	0	-0.24247021	0	0	0
0	0	0	0.43278799	0	-0.24102926	0	-0.008544055	0
0	0	0	0.43451866	0	-0.24221926	0	0	0
0	0.13085219	0	0.19848286	0	0	0	-0.009706408	0
0	0.12969927	0	0.19827976	0	0	0	-0.009763443	0
0	0.13264213	0	0.1987826	0	0	0	0	0
0	0	0	0.19830277	0	0	0	-0.010252011	0
0	0	0	0.19830732	0	0	0	-0.01046141	0
0	0	0	0.19877304	0	0	0	0	0
0	0.62647574	0	0	0	0.18376379	0	-0.010681301	0

0	0.62643679	0	0	0	0.18436444	0	0	0
0	0.62644252	0	0	0	0.18357484	0	0	0
0	0.3726929	0	0	0	0	0	0.010720259	0
0	0	0	0.44935508	0	-0.2564968	0	-0.017278638	0
0	0	0	0.44952503	0	-0.25741297	0	-0.016638162	0
0	0	0	0.45309065	0	-0.26025009	0	0	0
0	0.62668461	0	0.47200847	0	-0.24677501	0	0	0
0	0.13281732	0	0	0	0.18431374	0	-0.010286342	0
0	0.13193156	0	0	0	0.18396762	0	-0.010212336	0
0	0.13492168	0	0	0	0.18431742	0	0	0
0	0.62665898	0	0.22922548	0	0	0	-0.028668506	0
0	0.62644739	0	0.47768762	0	-0.24431308	0	0	0
0	0.62653475	0	0.47923074	0	-0.23767255	0	-0.039264804	0
0	0.62664105	0	0.22463067	0	0	0	0	0
0	0	0	0.19957915	0	0	0	-0.019193786	0
0	0	0	0	0	0.18409053	0	-0.010822812	0
0	0	0	0	0	0.18395584	0	-0.010903862	0
0	0	0	0	0	0.18426407	0	0	0
0	0.62646686	0	0.23528684	0	0	0	-0.033412231	0
0	0.059684083	0	0.46594071	0	-0.24047428	0	-0.027750743	0
0	0.068803801	0	0.46533066	0	-0.24510913	0	0	0
0	0	0	0.46661885	0	-0.24211015	0	-0.028022684	0
0	0	0	0.46629619	0	-0.24680108	0	0	0
0	0.04799585	0	0.46874302	0	-0.23774449	0	-0.032232338	0
0	0	0	0.46518047	0	-0.2508789	0	-0.026689144	0
0	0	0	0.46906527	0	-0.23942519	0	-0.032155391	0
0	0.069355896	0	0.22525089	0	0	0	-0.0256804	0
0	0	0	0.22455565	0	0	0	-0.026072737	0
0	0	0	0	0	0.18469224	0	-0.019691702	0
0	0.62666895	0	0	0	0.21643052	0	-0.030432943	0
0	0.62664716	0	0	0	0.21104397	0	0	0
0	0.62645618	0	0	0	0.22309864	0	-0.035665147	0
0	0.068320617	0	0	0	0.21183788	0	-0.027044953	0
0	0	0	0	0	0.21110477	0	-0.027430691	0
0	0	0	0.51245833	0	-0.31671653	0	0.067996952	0
0	0.62643955	0	0	0	0	0	-0.005448288	0
0	0.62640756	0	0	0	0	0	0	0
0	0.62634346	0	0	0	0	0	-0.007551535	0
0	0.13206309	0	0	0	0	0	-0.00469445	0
0	0	0	0	0	0	0	-0.005373499	0
0	0.57481672	0	0.4891191	0	-0.22575867	0	0	0
0	0.57771762	0	0.26988694	0	0	0	0	0
0	0.57762406	0	0.26803945	0	0	0	-0.003279953	0
0	0	0	0.49094319	0	-0.22616822	0	0	0
0	-0.045501194	0	0.49027732	0	-0.2241753	0	0	0
0	0	0	0.48942523	0	-0.22694977	0	-0.002229181	0
0	0.55872431	0	0.50150404	0	-0.22345634	0	0	0

0	-0.044741059	0	0.48877702	0	-0.22496569	0	-0.001273841	0
0	0.55837948	0	0.49977862	0	-0.22341125	0	-0.004677834	0
0	0	0	0.48673714	0	-0.21409388	0	0.001009287	0
0	0	0	0.26967745	0	0	0	-0.005752684	0
0	-0.043184388	0	0.27091651	0	0	0	-0.004806606	0
0	0.57930813	0	0.47634607	0	-0.21567613	0	0	0
0	0.56039362	0	0.28274472	0	0	0	-0.007552588	0
0	0	0	0.501216	0	-0.22348966	0	-0.006909381	0
0	0.58584004	0	0.26479264	0	0	0	0.012399203	0
0	0.56902378	0	0.48902996	0	-0.21824511	0	0.009062257	0
0	-0.062495914	0	0.50090452	0	-0.22161991	0	-0.005997348	0
0	0	0	0.47988944	0	-0.21990735	0	0.01269358	0
0	-0.037680356	0	0.47949996	0	-0.21831854	0	0.013337082	0
0	0.57640132	0	0	0	0.25705082	0	0	0
0	0.57607664	0	0	0	0.2553516	0	-0.003939834	0
0	0.62650326	0	0.44212936	0	-0.24285423	0	0	0
0	0.62651324	0	0.44551762	0	-0.24206718	0	-0.006049177	0
0	0.62651276	0	0.44569403	0	-0.24336769	0	0	0
0	0.43690804	0	0.49645743	0	-0.30535013	0	0	0
0	0.43737169	0	0.49572178	0	-0.30617456	0	0	0
0	0.43955636	0	0.49351586	0	-0.30774085	0	0.002662986	0
0	0.62651231	0	0.20491118	0	0	0	-0.0044817	0
0	0.62647926	0	0.20493077	0	0	0	0	0
0	0.62652174	0	0.20781513	0	0	0	-0.006411187	0
0	0	0	0	0	0.25688225	0	-0.00628907	0
0	0.075479558	0	0.44098752	0	-0.2416442	0	0	0
0	0.075298867	0	0.44038461	0	-0.24140769	0	-0.002244419	0
0	0.43093499	0	0.49497235	0	-0.30631827	0	0	0
0	0.066445392	0	0.44480512	0	-0.24133652	0	-0.005199569	0
0	-0.044614703	0	0	0	0.2581618	0	-0.005351615	0
0	0.4320545	0	0.49417813	0	-0.30774244	0	0.002127382	0
0	0.43306338	0	0.49054672	0	-0.30897522	0	0.004792441	0
0	0	0	0.44269674	0	-0.24340991	0	0	0
0	0	0	0.44192137	0	-0.24311781	0	-0.002917801	0
0	0	0	0.44573155	0	-0.24294361	0	-0.005587347	0
0	0.55828134	0	0	0	0.27055275	0	-0.008227149	0
0	0.60249224	0	0.46111495	0	-0.22078492	0	0.046958768	0
0	0.58414202	0	0	0	0.25256443	0	0.011292454	0
0	0	0	0.49691404	0	-0.30545039	0	-0.001904447	0
0	0	0	0.49704171	0	-0.30579616	0	0	0
0	0	0	0.49597647	0	-0.30692636	0	-3.54E-05	0
0	0	0	0.45129285	0	-0.25195426	0	-0.008048866	0
0	0.07808411	0	0.20507822	0	0	0	-0.003902838	0
0	0.43644206	0	0.1977294	0	0	0	-0.002159034	0
0	0.43680778	0	0.19834017	0	0	0	0	0
0	0.43711335	0	0.1964871	0	0	0	-0.00104147	0
0	0	0	0.49540278	0	-0.30675903	0	-0.000207435	0

0	0	0	0.20508899	0	0	0	-0.004689333	0
0	0.62288501	0	0.46803754	0	-0.23448606	0	0	0
0	0.62262897	0	0.46744045	0	-0.23461726	0	-0.001306745	0
0	-0.18562409	0	0.49710208	0	-0.30591096	0	0	0
0	-0.18620876	0	0.4975227	0	-0.30555446	0	-0.00103712	0
0	0.43050863	0	0.19574808	0	0	0	-0.000946417	0
0	0.62666065	0	0	0	0	0	-0.029781836	0
0	-0.18439022	0	0.49611851	0	-0.3073671	0	0.00139971	0
0	3.1239296	0	0.23793363	0	0	0	-0.003734381	0
0	-0.19128398	0	0.49558894	0	-0.30712635	0	0.001097768	0
0	0.62649834	0	0	0	0.19053777	0	-0.005104763	0
0	0.62647914	0	0	0	0.19028606	0	0	0
0	0.62651842	0	0	0	0.1946184	0	-0.007826904	0
0	3.132976	0	0.46094505	0	-0.22959256	0	0.011284259	0
0	0	0	0.19842572	0	0	0	-0.003178881	0
0	0	0	0.46830336	0	-0.23535892	0	-0.002660482	0
0	0.002457907	0	0.4679405	0	-0.23337072	0	-0.001779604	0
0	-0.18621618	0	0.19832762	0	0	0	-0.001930705	0
0	0.080292875	0	0	0	0.19052365	0	-0.004349602	0
0	0	0	0	0	0.19048761	0	-0.005129464	0
0	0.43528228	0	0	0	0.18274539	0	-0.003316342	0
0	0.4365694	0	0	0	0.18180993	0	0	0
0	0.43668919	0	0	0	0.18018571	0	-0.001422751	0
0	0.43036204	0	0	0	0.17924611	0	-0.001175156	0
0	0	0	0	0	0.18192378	0	-0.003371904	0
0	0.4098838	0	0.51321472	0	-0.27792347	0	0	0
0	0.41091055	0	0.51516352	0	-0.27549763	0	-0.000937825	0
0	0.62358351	0	0	0	0.22629563	0	-0.003972553	0
0	0.4110801	0	0.51232599	0	-0.27941068	0	0.002144211	0
0	-0.1865136	0	0	0	0.18181335	0	-0.002124737	0
0	0	0	0.5137443	0	-0.27853885	0	-0.000729005	0
0	0.41015214	0	0.24045092	0	0	0	-0.001409091	0
0	-0.21256792	0	0.51389844	0	-0.27842121	0	0.000543389	0
0	0.41276028	0	0.50150996	0	-0.27411966	0	0.021207371	0
0	0.40866076	0	0	0	0.22642923	0	-0.001735119	0
0	0.62650403	0	0.46962021	0	-0.24864216	0	0	0
0	0.62647131	0	0.46935456	0	-0.24617515	0	-0.00961061	0
0	0.62650901	0	0.22687941	0	0	0	-0.009563498	0
0	0.62683502	0	0.48755333	0	-0.24794067	0	-0.01952594	0
0	0.049536531	0	0.46742213	0	-0.24600476	0	-0.007497673	0
0	0.61706335	0	0.48604144	0	-0.24079909	0	-0.007278632	0
0	0	0	0.469002	0	-0.24766733	0	-0.008298445	0
0	0	0	0.4975843	0	-0.30859477	0	0.039647841	0
0	0.62651003	0	0	0	0.21234978	0	-0.010130617	0
0	0.62646845	0	0	0	0	0	0.001295433	0
0	0.43796476	0	0	0	0	0	0.003151271	0
0	0.37158853	0	0.50729395	0	-0.30975162	0	0	0

0	0.37426967	0	0.50740049	0	-0.31184807	0	0.007427742	0
0	0	0	0.50843087	0	-0.31105563	0	0.005302091	0
0	0.37289798	0	0.20450276	0	0	0	0.004634092	0
0	-0.24999494	0	0.5088272	0	-0.31152725	0	0.00675517	0
0	0.5024862	0	0	0	0	0	0.005246185	0
0	0.37828902	0	0.49268217	0	-0.3454236	0	0.007797323	0
0	0.3797111	0	0.48953335	0	-0.32358757	0	0.027533614	0
0	0.37240455	0	0	0	0.18772343	0	0.004428434	0
0	0.37105898	0	0.52649847	0	-0.30993285	0	0.000819168	0
0	0.62643913	0	0.43481716	0	-0.24174862	0	0	0
0	0.6264749	0	0.43257931	0	-0.24025368	0	-0.008904891	0
0	0.62643821	0	0.43405743	0	-0.24170433	0	0	0
0	0.62644533	0	0.198855	0	0	0	-0.010592045	0
0	0.62643718	0	0.19834616	0	0	0	-0.010406042	0
0	0.62641764	0	0.19881579	0	0	0	0	0
0	0.12756956	0	0.43225919	0	-0.23931362	0	-0.008541009	0
0	0.12914891	0	0.43379894	0	-0.24063526	0	0	0
0	0.12652475	0	0.43175711	0	-0.23909847	0	-0.008532202	0
0	0	0	0.43364708	0	-0.24090895	0	-0.009085206	0
0	0	0	0.43323807	0	-0.24058044	0	-0.009227608	0
0	0	0	0.43536051	0	-0.24223833	0	0	0
0	0.13015932	0	0.19916232	0	0	0	-0.010238011	0
0	0	0	0.19917827	0	0	0	-0.010928679	0
0	0.62643776	0	0	0	0.18464962	0	-0.011163929	0
0	0.62643041	0	0	0	0.18399673	0	-0.010848428	0
0	0.62626068	0	0	0	0.18432864	0	0	0
0	0	0	0.45006622	0	-0.25647747	0	-0.017684228	0
0	0.62664645	0	0.46522646	0	-0.24152957	0	-0.027488182	0
0	0.62662997	0	0.46501009	0	-0.2461292	0	0	0
0	0.62645254	0	0.46865153	0	-0.23863596	0	-0.032397923	0
0	0.62661166	0	0.22396511	0	0	0	-0.025647344	0
0	0.13235475	0	0	0	0.18477734	0	-0.010646323	0
0	0.066297942	0	0.46019912	0	-0.24052316	0	-0.024467124	0
0	0	0	0	0	0.18475248	0	-0.01133095	0
0	0	0	0.46107572	0	-0.24213335	0	-0.024853213	0
0	0.62661789	0	0	0	0.21052274	0	-0.027001563	0
0	0.62640366	0	0	0	0	0	-0.005299886	0
0	0.57407725	0	0.4901911	0	-0.22490679	0	0	0
0	0.57423032	0	0.48881163	0	-0.22537035	0	-0.002228617	0
0	0.57627264	0	0.27014135	0	0	0	-0.005313883	0
0	0	0	0.49034251	0	-0.22533102	0	-0.004640622	0
0	-0.04649555	0	0.48973469	0	-0.22344474	0	-0.0037007	0
0	0.55705732	0	0.5006041	0	-0.22228485	0	-0.006484705	0
0	0.58267399	0	0.47894775	0	-0.21870934	0	0.013642864	0
0	0.57482767	0	0	0	0.25735975	0	-0.005857556	0
0	0.62648843	0	0.44163768	0	-0.24228312	0	-0.003284089	0
0	0.62646851	0	0.44214759	0	-0.24284157	0	0	0

0	0.62649935	0	0.44454863	0	-0.24237304	0	-0.00513456	0
0	0.43663519	0	0.49626153	0	-0.3051807	0	-0.001060989	0
0	0.43682444	0	0.49659343	0	-0.30530295	0	0	0
0	0.43745319	0	0.4957547	0	-0.30623962	0	0.000203797	0
0	0.62647281	0	0.20510688	0	0	0	-0.004637129	0
0	0.074830606	0	0.44061888	0	-0.24116435	0	-0.002615226	0
0	0.43105541	0	0.49501936	0	-0.30637974	0	0.000386556	0
0	0	0	0.44222031	0	-0.24278982	0	-0.003398062	0
0	0	0	0.49663608	0	-0.30531671	0	-0.001814918	0
0	0.43602678	0	0.19844065	0	0	0	-0.002608491	0
0	0.62228867	0	0.46773043	0	-0.23406849	0	-0.002251687	0
0	-0.18579393	0	0.49703002	0	-0.30581485	0	-0.000568358	0
0	0.62647218	0	0	0	0.1905106	0	-0.005077673	0
0	0.43573102	0	0	0	0.18193277	0	-0.002801898	0
0	0.40983122	0	0.51319276	0	-0.27789242	0	-0.000161195	0
0	0.62648312	0	0.46837042	0	-0.24705889	0	-0.008247721	0
0	0.37331931	0	0.50802366	0	-0.31073602	0	0.006054937	0
0	0.62642787	0	0.43342628	0	-0.24013436	0	-0.009425594	0
0	0.62643027	0	0.43276433	0	-0.24007284	0	-0.0091724	0
0	0.62622331	0	0.43490512	0	-0.24171861	0	0	0
0	0.62641379	0	0.19921904	0	0	0	-0.010868009	0
0	0.12698189	0	0.43255877	0	-0.23902455	0	-0.008998301	0
0	0	0	0.43402845	0	-0.24050651	0	-0.009686103	0
0	0.6264109	0	0	0	0.18479642	0	-0.011270727	0
0	0.62660183	0	0.45990323	0	-0.24155686	0	-0.024427634	0
0	0.572945	0	0.48964589	0	-0.22414726	0	-0.004208132	0
0	0.62646146	0	0.44167893	0	-0.24223159	0	-0.003347275	0
0	0.43645051	0	0.49644397	0	-0.30510004	0	-0.001245999	0
0	0.62639588	0	0.43355877	0	-0.23999932	0	-0.00962408	0

SUPPLEMENTARY TABLE 5

Model	BIC	Loglike	H2r	SE	bsnp_rs2938864	SE	bsnp_rs1001646
cov0	0	-219.893	0.4180209	0	0	0	0
cov1	0.4018	-216.571	0.4185549	0	-0.089374734	0	0
cov6	0.4686	-216.605	0.4191203	0	0	0	0
cov2	1.0516	-216.896	0.4213974	0	0	0	-0.083996826
cov5	2.4807	-217.611	0.4212418	0	0	0	0
cov4	3.1226	-217.932	0.4244336	0	0	0	0
cov10	3.1693	-217.955	0.4115142	0	0	0	0
cov9	3.8533	-218.297	0.4117738	0	0	0	0
cov11	4.0549	-218.398	0.4151934	0	0	0	0
cov8	5.7948	-219.268	0.4184404	0	0	0	0
cov7	5.8007	-219.271	0.4184467	0	0	0	0
cov1_10	6.4846	-216.09	0.415162	0	-0.073655875	0	0
cov6_7	6.5391	-216.117	0.4198951	0	0	0	0
cov6_8	6.551	-216.123	0.4198837	0	0	0	0
cov1_8	6.5687	-216.132	0.4193109	0	-0.087034435	0	0
cov1_7	6.5754	-216.136	0.4193131	0	-0.08703092	0	0
cov6_10	6.6052	-216.151	0.4155828	0	0	0	0
cov5_10	6.6913	-216.194	0.4151843	0	0	0	0
cov2_10	6.8247	-216.26	0.4171334	0	0	0	-0.06814853
cov3	6.915	-219.828	0.4191744	0	0	0	0
cov1_9	6.9164	-216.306	0.4158491	0	-0.076979755	0	0
cov1_3	6.9416	-216.319	0.4209892	0	-0.092707934	0	0
cov3_6	6.9959	-216.346	0.4216259	0	0	0	0
cov2_6	7.0079	-216.352	0.4203444	0	0	0	-0.039097963
cov6_9	7.0206	-216.358	0.4163222	0	0	0	0
cov2_8	7.0999	-216.398	0.4220827	0	0	0	-0.082280415
cov2_7	7.1056	-216.401	0.4220849	0	0	0	-0.082282256
cov1_6	7.1776	-216.437	0.4188528	0	-0.049186229	0	0
cov1_2	7.2002	-216.448	0.4196684	0	-0.061874931	0	-0.032085332
cov2_9	7.2824	-216.489	0.4177545	0	0	0	-0.070812972
cov1_11	7.3016	-216.499	0.4196789	0	-0.10512834	0	0
cov4_10	7.3238	-216.51	0.4180482	0	0	0	0
cov1_5	7.4038	-216.55	0.4180409	0	-0.10196064	0	0
cov4_6	7.4122	-216.554	0.4204224	0	0	0	0
cov5_9	7.4231	-216.559	0.4156576	0	0	0	0
cov1_4	7.4325	-216.564	0.4190855	0	-0.084872118	0	0
cov6_11	7.4798	-216.588	0.4196407	0	0	0	0
cov5_6	7.4816	-216.589	0.4194446	0	0	0	0
cov2_3	7.6128	-216.654	0.4238906	0	0	0	-0.087246966
cov2_5	7.8785	-216.787	0.4216609	0	0	0	-0.066069871
cov4_9	8.0023	-216.849	0.4184662	0	0	0	0
cov2_11	8.0875	-216.892	0.4210488	0	0	0	-0.080995493
cov2_4	8.0962	-216.896	0.4214575	0	0	0	-0.083169304
cov5_8	8.4052	-217.051	0.4219813	0	0	0	0
cov5_7	8.4142	-217.055	0.4219836	0	0	0	0

cov10_11	8.7649	-217.23	0.4108629	0	0	0	0
cov8_11	9.2645	-217.48	0.4158519	0	0	0	0
cov7_11	9.2665	-217.481	0.415858	0	0	0	0
cov7_10	9.3347	-217.515	0.4121184	0	0	0	0
cov8_10	9.3352	-217.515	0.4121186	0	0	0	0
cov5_11	9.3594	-217.528	0.419718	0	0	0	0
cov9_11	9.3868	-217.541	0.4112449	0	0	0	0
cov4_8	9.3961	-217.546	0.4245315	0	0	0	0
cov4_7	9.4044	-217.55	0.4245328	0	0	0	0
cov4_5	9.4485	-217.572	0.4222968	0	0	0	0
cov3_10	9.4824	-217.589	0.4132819	0	0	0	0
cov3_5	9.4985	-217.597	0.4218089	0	0	0	0
cov4_11	9.6052	-217.651	0.4212334	0	0	0	0
cov9_10	9.8623	-217.779	0.4121068	0	0	0	0
cov8_9	9.9827	-217.839	0.4123983	0	0	0	0
cov7_9	9.9828	-217.839	0.4123979	0	0	0	0
cov3_4	10.1639	-217.93	0.4246093	0	0	0	0
cov3_9	10.2699	-217.983	0.4134228	0	0	0	0
cov3_11	10.485	-218.09	0.4175235	0	0	0	0
cov3_8	12.5787	-219.137	0.4165256	0	0	0	0
cov3_7	12.5799	-219.138	0.4165101	0	0	0	0
cov1_3_10	12.6657	-215.658	0.4174896	0	-0.075005828	0	0
cov1_9_10	12.7062	-215.679	0.4163334	0	-0.079134542	0	0
cov1_8_10	12.7734	-215.712	0.4159889	0	-0.072468902	0	0
cov1_7_10	12.7767	-215.714	0.4159854	0	-0.072434118	0	0
cov3_6_10	12.786	-215.718	0.4179203	0	0	0	0
cov6_7_10	12.8128	-215.732	0.4164822	0	0	0	0
cov6_8_10	12.824	-215.737	0.4164701	0	0	0	0
cov7_8	12.8395	-219.268	0.4184422	0	0	0	0
cov6_9_10	12.8497	-215.75	0.4167972	0	0	0	0
cov5_8_10	12.9103	-215.781	0.4160543	0	0	0	0
cov5_7_10	12.9133	-215.782	0.4160506	0	0	0	0
cov2_3_10	12.9737	-215.812	0.4195679	0	0	0	-0.069869332
cov5_9_10	13.0157	-215.833	0.4162511	0	0	0	0
cov2_8_10	13.0427	-215.847	0.41795	0	0	0	-0.067622445
cov2_7_10	13.0447	-215.848	0.4179466	0	0	0	-0.067598863
cov2_6_7	13.1005	-215.876	0.4211105	0	0	0	-0.038227899
cov2_6_8	13.1063	-215.879	0.4211094	0	0	0	-0.0384701
cov1_8_9	13.1711	-215.911	0.4167217	0	-0.075796676	0	0
cov1_7_9	13.1752	-215.913	0.4167172	0	-0.075759501	0	0
cov6_7_9	13.1898	-215.92	0.4172761	0	0	0	0
cov6_8_9	13.2011	-215.926	0.4172636	0	0	0	0
cov1_3_9	13.2098	-215.93	0.4180549	0	-0.078222526	0	0
cov2_6_10	13.233	-215.942	0.4169756	0	0	0	-0.035574049
cov2_9_10	13.2336	-215.942	0.4182235	0	0	0	-0.071357612
cov3_6_9	13.3114	-215.981	0.418544	0	0	0	0
cov1_6_7	13.3146	-215.983	0.4196571	0	-0.044125777	0	0

cov1_6_8	13.3175	-215.984	0.4196453	0	-0.04483622	0	0
cov2_5_10	13.3182	-215.985	0.4167126	0	0	0	-0.036486406
cov1_2_10	13.3271	-215.989	0.4163078	0	-0.049080418	0	-0.029081383
cov5_6_10	13.3327	-215.992	0.4157814	0	0	0	0
cov1_2_8	13.3474	-215.999	0.4204846	0	-0.058433777	0	-0.033340998
cov1_2_7	13.3538	-216.002	0.4204875	0	-0.058409947	0	-0.033363863
cov3_5_10	13.3588	-216.005	0.4165399	0	0	0	0
cov4_6_10	13.3623	-216.007	0.4173616	0	0	0	0
cov1_6_10	13.3792	-216.015	0.4155379	0	-0.044222735	0	0
cov1_4_10	13.3845	-216.018	0.4165388	0	-0.057203095	0	0
cov1_10_11	13.416	-216.033	0.4162008	0	-0.087828166	0	0
cov1_5_10	13.4284	-216.04	0.4153531	0	-0.048591946	0	0
cov2_8_9	13.4643	-216.058	0.4186158	0	0	0	-0.070298262
cov2_7_9	13.467	-216.059	0.4186115	0	0	0	-0.070273239
cov2_3_6	13.4885	-216.07	0.4230296	0	0	0	-0.040907623
cov2_3_9	13.5392	-216.095	0.4200678	0	0	0	-0.072459579
cov4_6_7	13.5452	-216.098	0.4206765	0	0	0	0
cov5_6_7	13.5453	-216.098	0.4202674	0	0	0	0
cov4_6_8	13.555	-216.103	0.4206875	0	0	0	0
cov5_6_8	13.555	-216.103	0.4202673	0	0	0	0
cov3_6_7	13.5799	-216.115	0.4201477	0	0	0	0
cov6_7_11	13.5806	-216.116	0.4197406	0	0	0	0
cov6_7_8	13.5831	-216.117	0.419897	0	0	0	0
cov3_6_8	13.5906	-216.121	0.4201702	0	0	0	0
cov6_8_11	13.5925	-216.122	0.419727	0	0	0	0
cov1_5_8	13.593	-216.122	0.4189387	0	-0.095795888	0	0
cov5_8_9	13.5989	-216.125	0.4165625	0	0	0	0
cov1_5_7	13.5994	-216.125	0.4189383	0	-0.095857823	0	0
cov1_8_11	13.6001	-216.126	0.4196324	0	-0.09213401	0	0
cov5_7_9	13.6026	-216.127	0.4165581	0	0	0	0
cov1_3_8	13.6038	-216.127	0.4196911	0	-0.087898443	0	0
cov1_7_11	13.607	-216.129	0.4196326	0	-0.092099769	0	0
cov1_3_7	13.6106	-216.131	0.4196923	0	-0.087896699	0	0
cov1_4_8	13.6132	-216.132	0.4193959	0	-0.086297596	0	0
cov1_7_8	13.6136	-216.132	0.4193106	0	-0.087035356	0	0
cov1_4_7	13.62	-216.135	0.4193946	0	-0.086324186	0	0
cov2_6_9	13.6217	-216.136	0.4177269	0	0	0	-0.036691746
cov6_10_11	13.6263	-216.139	0.4160271	0	0	0	0
cov1_3_5	13.6716	-216.161	0.4202413	0	-0.1315831	0	0
cov1_3_6	13.6879	-216.169	0.421389	0	-0.050433462	0	0
cov4_5_10	13.7056	-216.178	0.4159196	0	0	0	0
cov1_2_3	13.7103	-216.181	0.4222502	0	-0.063671747	0	-0.03399479
cov5_10_11	13.7338	-216.192	0.4153138	0	0	0	0
cov1_2_9	13.7411	-216.196	0.4170114	0	-0.051286979	0	-0.03033212
cov2_4_10	13.7528	-216.202	0.4179088	0	0	0	-0.050281324
cov1_6_9	13.7843	-216.218	0.416286	0	-0.045093564	0	0
cov4_9_10	13.7913	-216.221	0.4190957	0	0	0	0

cov4_8_10	13.8039	-216.227	0.4183032	0	0	0	0
cov4_7_10	13.8069	-216.229	0.4182984	0	0	0	0
cov1_9_11	13.8362	-216.244	0.416934	0	-0.091831322	0	0
cov4_6_9	13.8369	-216.244	0.4178915	0	0	0	0
cov5_6_9	13.861	-216.256	0.4164791	0	0	0	0
cov1_4_9	13.8625	-216.257	0.4169706	0	-0.063304519	0	0
cov2_10_11	13.8685	-216.26	0.4170212	0	0	0	-0.067154841
cov2_5_9	13.8876	-216.269	0.4174493	0	0	0	-0.042845262
cov2_6_11	13.9067	-216.279	0.4216911	0	0	0	-0.044779839
cov2_5_8	13.9142	-216.283	0.4224016	0	0	0	-0.063838861
cov2_5_7	13.921	-216.286	0.4224031	0	0	0	-0.063884538
cov1_3_11	13.9285	-216.29	0.4215441	0	-0.10264791	0	0
cov1_5_9	13.9343	-216.293	0.4159449	0	-0.064031203	0	0
cov1_3_4	13.9615	-216.306	0.4204431	0	-0.099444163	0	0
cov1_2_6	13.9945	-216.323	0.4199444	0	-0.024104387	0	-0.030874178
cov3_4_6	14.0345	-216.343	0.4218743	0	0	0	0
cov2_4_6	14.0356	-216.343	0.4198613	0	0	0	-0.044444091
cov1_6_11	14.0363	-216.344	0.420128	0	-0.063952035	0	0
cov3_5_6	14.0369	-216.344	0.4215735	0	0	0	0
cov6_9_11	14.0369	-216.344	0.4168071	0	0	0	0
cov3_6_11	14.0388	-216.345	0.4217234	0	0	0	0
cov2_8_11	14.0389	-216.345	0.4209412	0	0	0	-0.071576597
cov2_7_11	14.044	-216.347	0.4209408	0	0	0	-0.071542907
cov2_5_6	14.0489	-216.35	0.4202691	0	0	0	-0.040313205
cov1_2_11	14.0651	-216.358	0.4210217	0	-0.077494733	0	-0.034405283
cov3_4_10	14.0867	-216.369	0.4188852	0	0	0	0
cov2_4_8	14.129	-216.39	0.4216766	0	0	0	-0.088151078
cov2_4_7	14.1343	-216.393	0.4216745	0	0	0	-0.088219648
cov2_3_8	14.1441	-216.398	0.4221897	0	0	0	-0.082486006
cov2_7_8	14.1447	-216.398	0.4220831	0	0	0	-0.082279559
cov2_3_7	14.1499	-216.4	0.4221853	0	0	0	-0.082475437
cov3_5_9	14.1727	-216.412	0.416855	0	0	0	0
cov1_5_6	14.1822	-216.417	0.4183533	0	-0.061579853	0	0
cov1_2_5	14.1946	-216.423	0.4191352	0	-0.075100891	0	-0.032593303
cov1_4_6	14.2119	-216.431	0.4193199	0	-0.045543275	0	0
cov1_2_4	14.2226	-216.437	0.4190996	0	-0.063609896	0	-0.037701456
cov2_4_9	14.2567	-216.454	0.4183595	0	0	0	-0.056962916
cov4_10_11	14.2584	-216.455	0.4169619	0	0	0	0
cov1_5_11	14.3045	-216.478	0.4191604	0	-0.11752785	0	0
cov2_9_11	14.3265	-216.489	0.4176593	0	0	0	-0.069949244
cov1_4_5	14.3331	-216.492	0.4193058	0	-0.10299706	0	0
cov1_4_11	14.3333	-216.492	0.4201856	0	-0.10074533	0	0
cov4_6_11	14.4055	-216.528	0.4212052	0	0	0	0
cov4_5_9	14.4271	-216.539	0.4164981	0	0	0	0
cov4_8_9	14.4504	-216.551	0.418733	0	0	0	0
cov4_5_6	14.452	-216.551	0.420541	0	0	0	0
cov4_7_9	14.454	-216.552	0.4187274	0	0	0	0

cov5_9_11	14.468	-216.559	0.4156393	0	0	0	0
cov5_6_11	14.4736	-216.562	0.4202163	0	0	0	0
cov7_10_11	14.4784	-216.565	0.4117877	0	0	0	0
cov8_10_11	14.4807	-216.566	0.4117869	0	0	0	0
cov2_3_4	14.5534	-216.602	0.4232377	0	0	0	-0.10471801
cov2_3_5	14.5812	-216.616	0.423779	0	0	0	-0.075752093
cov2_3_11	14.603	-216.627	0.4231776	0	0	0	-0.079898222
cov3_10_11	14.6371	-216.644	0.4131904	0	0	0	0
cov2_4_5	14.7743	-216.713	0.4200871	0	0	0	-0.076925156
cov3_4_9	14.8338	-216.742	0.419191	0	0	0	0
cov4_9_11	14.9118	-216.781	0.4172996	0	0	0	0
cov2_5_11	14.9194	-216.785	0.4219215	0	0	0	-0.067341119
cov3_5_8	14.9305	-216.791	0.4192761	0	0	0	0
cov3_5_7	14.9345	-216.793	0.4192592	0	0	0	0
cov7_9_11	15.036	-216.843	0.4122356	0	0	0	0
cov8_9_11	15.0377	-216.844	0.4122353	0	0	0	0
cov5_8_11	15.0506	-216.851	0.4197314	0	0	0	0
cov5_7_11	15.0576	-216.854	0.4197283	0	0	0	0
cov2_4_11	15.1322	-216.892	0.4211009	0	0	0	-0.080320568
cov9_10_11	15.2321	-216.941	0.4115441	0	0	0	0
cov3_9_11	15.3886	-217.02	0.4134846	0	0	0	0
cov4_5_8	15.4451	-217.048	0.4222332	0	0	0	0
cov5_7_8	15.4501	-217.051	0.4219808	0	0	0	0
cov4_5_7	15.4542	-217.053	0.4222347	0	0	0	0
cov4_8_11	15.4545	-217.053	0.4203911	0	0	0	0
cov4_7_11	15.4607	-217.056	0.4203862	0	0	0	0
cov3_4_8	15.9679	-217.309	0.4220654	0	0	0	0
cov3_4_7	15.9733	-217.312	0.4220503	0	0	0	0
cov7_9_10	16.022	-217.336	0.4127375	0	0	0	0
cov8_9_10	16.0223	-217.337	0.4127373	0	0	0	0
cov3_9_10	16.1624	-217.407	0.4139124	0	0	0	0
cov3_5_11	16.2782	-217.465	0.4204642	0	0	0	0
cov3_8_11	16.2896	-217.47	0.4153787	0	0	0	0
cov3_7_11	16.29	-217.47	0.4153642	0	0	0	0
cov7_8_11	16.3071	-217.479	0.4158559	0	0	0	0
cov3_8_10	16.3074	-217.479	0.4127205	0	0	0	0
cov3_7_10	16.3085	-217.48	0.4127159	0	0	0	0
cov4_5_11	16.3178	-217.484	0.4207969	0	0	0	0
cov7_8_10	16.3783	-217.515	0.4121194	0	0	0	0
cov4_7_8	16.4406	-217.546	0.4245312	0	0	0	0
cov3_4_5	16.4775	-217.564	0.4226621	0	0	0	0
cov3_4_11	16.5055	-217.578	0.4217634	0	0	0	0
cov3_8_9	17.0007	-217.826	0.412764	0	0	0	0
cov3_7_9	17.0018	-217.826	0.4127576	0	0	0	0
cov7_8_9	17.0261	-217.839	0.4123995	0	0	0	0
cov1_3_9_10	18.8546	-215.23	0.4187735	0	-0.080621799	0	0
cov1_8_9_10	18.9952	-215.301	0.417213	0	-0.077949941	0	0

cov3_6_9_10	18.9983	-215.302	0.4192481	0	0	0	0
cov1_7_9_10	18.9991	-215.303	0.4172091	0	-0.077913848	0	0
cov6_7_9_10	19.0517	-215.329	0.4177585	0	0	0	0
cov6_8_9_10	19.0633	-215.335	0.4177449	0	0	0	0
cov5_8_9_10	19.2294	-215.418	0.4171745	0	0	0	0
cov5_7_9_10	19.2329	-215.419	0.4171705	0	0	0	0
cov2_3_9_10	19.3546	-215.48	0.4207548	0	0	0	-0.073180253
cov2_3_6_10	19.3763	-215.491	0.4194726	0	0	0	-0.03715567
cov2_8_9_10	19.4462	-215.526	0.4190864	0	0	0	-0.070846924
cov2_7_9_10	19.4485	-215.527	0.419083	0	0	0	-0.07082235
cov2_6_7_10	19.453	-215.53	0.4178663	0	0	0	-0.035035543
cov2_6_8_10	19.4589	-215.532	0.417866	0	0	0	-0.035262173
cov1_2_3_10	19.4812	-215.544	0.4187756	0	-0.048852576	0	-0.030977176
cov2_6_9_10	19.4862	-215.546	0.4181754	0	0	0	-0.035196957
cov2_5_8_10	19.5439	-215.575	0.4175897	0	0	0	-0.036190203
cov2_5_7_10	19.5468	-215.576	0.4175864	0	0	0	-0.036194171
cov5_6_7_10	19.5519	-215.579	0.4166942	0	0	0	0
cov1_3_6_10	19.5525	-215.579	0.4179037	0	-0.044827908	0	0
cov5_6_8_10	19.5568	-215.581	0.4166874	0	0	0	0
cov5_6_9_10	19.5645	-215.585	0.4169983	0	0	0	0
cov1_2_9_10	19.5766	-215.591	0.4173877	0	-0.056217782	0	-0.027011354
cov1_6_9_10	19.5794	-215.593	0.4167632	0	-0.047740805	0	0
cov1_2_8_10	19.5965	-215.601	0.417204	0	-0.046726229	0	-0.030446747
cov1_2_7_10	19.5995	-215.603	0.4172011	0	-0.04667604	0	-0.030464178
cov3_7_8	19.6198	-219.135	0.4165167	0	0	0	0
cov4_6_9_10	19.6198	-215.613	0.4185268	0	0	0	0
cov1_3_8_10	19.6231	-215.615	0.4171241	0	-0.073994785	0	0
cov1_3_7_10	19.6262	-215.616	0.4171259	0	-0.073989316	0	0
cov1_4_9_10	19.628	-215.617	0.4175906	0	-0.063900048	0	0
cov2_5_9_10	19.628	-215.617	0.4178317	0	0	0	-0.037116561
cov1_6_7_10	19.6382	-215.622	0.4164362	0	-0.039865483	0	0
cov1_6_8_10	19.6417	-215.624	0.4164275	0	-0.040532189	0	0
cov1_9_10_11	19.6524	-215.629	0.4173048	0	-0.092304718	0	0
cov1_5_9_10	19.6617	-215.634	0.4165074	0	-0.055519959	0	0
cov3_5_9_10	19.688	-215.647	0.4176216	0	0	0	0
cov4_6_7_10	19.6914	-215.649	0.417764	0	0	0	0
cov1_3_10_11	19.6918	-215.649	0.417849	0	-0.080869125	0	0
cov1_5_8_10	19.6949	-215.65	0.4162176	0	-0.044737172	0	0
cov1_5_7_10	19.6982	-215.652	0.4162151	0	-0.0447077	0	0
cov4_6_8_10	19.6984	-215.652	0.417766	0	0	0	0
cov3_6_7_10	19.7019	-215.654	0.4174888	0	0	0	0
cov1_3_4_10	19.7059	-215.656	0.4176893	0	-0.071841986	0	0
cov3_6_8_10	19.7066	-215.656	0.417498	0	0	0	0
cov1_3_5_10	19.7091	-215.658	0.4174998	0	-0.078301134	0	0
cov3_5_6_10	19.7327	-215.669	0.4178287	0	0	0	0
cov1_4_8_10	19.745	-215.676	0.4169176	0	-0.060726163	0	0
cov1_4_7_10	19.7486	-215.677	0.4169122	0	-0.06071185	0	0

cov2_3_5_10	19.7515	-215.679	0.4189514	0	0	0	-0.046814247
cov3_4_6_10	19.7623	-215.684	0.4186271	0	0	0	0
cov2_6_7_9	19.8045	-215.705	0.4186718	0	0	0	-0.036119987
cov1_8_10_11	19.8098	-215.708	0.4162543	0	-0.076554637	0	0
cov2_6_8_9	19.8103	-215.708	0.4186712	0	0	0	-0.036351214
cov1_7_10_11	19.8133	-215.71	0.4162473	0	-0.076469215	0	0
cov1_7_8_10	19.8181	-215.712	0.415989	0	-0.072460816	0	0
cov3_6_10_11	19.8295	-215.718	0.4178281	0	0	0	0
cov6_7_10_11	19.8529	-215.729	0.4162976	0	0	0	0
cov6_7_8_10	19.8566	-215.731	0.4164838	0	0	0	0
cov6_8_10_11	19.864	-215.735	0.4162837	0	0	0	0
cov2_3_6_9	19.8746	-215.74	0.420106	0	0	0	-0.038248728
cov6_9_10_11	19.8801	-215.743	0.4171443	0	0	0	0
cov2_3_8_10	19.906	-215.756	0.4191069	0	0	0	-0.068974632
cov2_3_7_10	19.9087	-215.757	0.4191057	0	0	0	-0.068966597
cov5_8_10_11	19.9268	-215.766	0.4156495	0	0	0	0
cov5_7_10_11	19.9294	-215.768	0.4156431	0	0	0	0
cov3_5_8_10	19.9528	-215.779	0.4159507	0	0	0	0
cov5_7_8_10	19.9549	-215.78	0.4160544	0	0	0	0
cov4_5_8_10	19.9552	-215.781	0.4160541	0	0	0	0
cov3_5_7_10	19.9556	-215.781	0.4159423	0	0	0	0
cov4_5_7_10	19.9582	-215.782	0.4160481	0	0	0	0
cov2_3_10_11	19.9732	-215.79	0.4189181	0	0	0	-0.063233825
cov1_2_8_9	19.9757	-215.791	0.4179531	0	-0.048949818	0	-0.031679491
cov1_2_7_9	19.9796	-215.793	0.4179492	0	-0.048896266	0	-0.03169794
cov1_2_3_9	20.006	-215.806	0.4193593	0	-0.050933177	0	-0.032248347
cov1_6_7_9	20.0065	-215.806	0.4172378	0	-0.040676084	0	0
cov1_6_8_9	20.0099	-215.808	0.4172288	0	-0.04135771	0	0
cov2_3_4_10	20.0161	-215.811	0.4195023	0	0	0	-0.072832668
cov2_8_10_11	20.0268	-215.816	0.4171495	0	0	0	-0.05976238
cov2_7_10_11	20.0283	-215.817	0.4171425	0	0	0	-0.059701264
cov5_6_7_9	20.0382	-215.822	0.4174424	0	0	0	0
cov4_5_9_10	20.0393	-215.823	0.4168598	0	0	0	0
cov5_6_8_9	20.0443	-215.825	0.4174337	0	0	0	0
cov2_4_8_10	20.0523	-215.829	0.4183346	0	0	0	-0.05767318
cov2_4_7_10	20.0547	-215.83	0.4183294	0	0	0	-0.057701218
cov5_9_10_11	20.0605	-215.833	0.4162436	0	0	0	0
cov1_3_6_9	20.0691	-215.838	0.4185343	0	-0.045577682	0	0
cov2_4_6_7	20.0706	-215.838	0.4201539	0	0	0	-0.049453539
cov2_5_8_9	20.0734	-215.84	0.4183655	0	0	0	-0.042465504
cov2_5_7_9	20.077	-215.842	0.4183611	0	0	0	-0.04247002
cov2_4_6_8	20.078	-215.842	0.420164	0	0	0	-0.049575856
cov2_7_8_10	20.087	-215.847	0.41795	0	0	0	-0.067613768
cov1_3_8_9	20.1035	-215.855	0.4175859	0	-0.076927763	0	0
cov1_3_7_9	20.1076	-215.857	0.4175852	0	-0.076912916	0	0
cov1_2_6_7	20.1131	-215.86	0.4208113	0	-0.017991294	0	-0.032099512
cov4_6_7_9	20.1146	-215.86	0.4183486	0	0	0	0

cov1_2_6_8	20.1161	-215.861	0.4207971	0	-0.018722128	0	-0.032090192
cov4_6_8_9	20.1223	-215.864	0.4183546	0	0	0	0
cov2_6_7_11	20.1268	-215.866	0.4215705	0	0	0	-0.040360048
cov2_3_6_7	20.1319	-215.869	0.4215832	0	0	0	-0.038707441
cov2_6_8_11	20.1326	-215.869	0.4215685	0	0	0	-0.040589655
cov2_3_6_8	20.1362	-215.871	0.4216053	0	0	0	-0.038944811
cov2_5_6_7	20.144	-215.875	0.4210582	0	0	0	-0.038974361
cov2_6_7_8	20.1454	-215.876	0.4211109	0	0	0	-0.038244934
cov2_5_6_8	20.15	-215.878	0.4210662	0	0	0	-0.039129129
cov3_6_7_9	20.1513	-215.879	0.4180147	0	0	0	0
cov2_4_9_10	20.1545	-215.88	0.4190223	0	0	0	-0.052965445
cov3_6_8_9	20.1576	-215.882	0.4180244	0	0	0	0
cov2_5_6_10	20.1623	-215.884	0.4167915	0	0	0	-0.027715606
cov2_6_10_11	20.1648	-215.885	0.418216	0	0	0	-0.040640964
cov1_4_8_9	20.1752	-215.891	0.4174022	0	-0.066984847	0	0
cov1_5_8_9	20.1759	-215.891	0.4168482	0	-0.060017934	0	0
cov1_4_7_9	20.1796	-215.893	0.4173964	0	-0.0669643	0	0
cov1_5_7_9	20.1801	-215.893	0.4168435	0	-0.059987995	0	0
cov1_8_9_11	20.2052	-215.906	0.4170169	0	-0.080375669	0	0
cov1_7_9_11	20.2096	-215.908	0.4170093	0	-0.080293644	0	0
cov1_7_8_9	20.2159	-215.911	0.4167218	0	-0.075790953	0	0
cov1_3_5_9	20.2209	-215.913	0.4181138	0	-0.093992736	0	0
cov1_3_9_11	20.2256	-215.916	0.4184977	0	-0.085500079	0	0
cov1_2_10_11	20.2297	-215.918	0.4175709	0	-0.063218586	0	-0.031204143
cov6_7_9_11	20.2311	-215.919	0.417119	0	0	0	0
cov1_2_6_10	20.2322	-215.919	0.4166594	0	-0.021321816	0	-0.02832671
cov6_7_8_9	20.2336	-215.92	0.4172778	0	0	0	0
cov6_8_9_11	20.2424	-215.924	0.4171045	0	0	0	0
cov2_4_6_10	20.2494	-215.928	0.4173929	0	0	0	-0.02808978
cov2_3_4_6	20.2517	-215.929	0.4217912	0	0	0	-0.065852001
cov1_3_4_9	20.2543	-215.93	0.4181071	0	-0.077365067	0	0
cov2_9_10_11	20.2688	-215.937	0.4178862	0	0	0	-0.068327523
cov4_8_9_10	20.2776	-215.942	0.4193678	0	0	0	0
cov4_7_9_10	20.2809	-215.943	0.4193629	0	0	0	0
cov1_6_10_11	20.2812	-215.944	0.4167304	0	-0.057286331	0	0
cov1_2_5_10	20.289	-215.948	0.4164308	0	-0.027379237	0	-0.027816021
cov5_6_10_11	20.2929	-215.949	0.4166937	0	0	0	0
cov1_4_6_10	20.2983	-215.952	0.4168234	0	-0.030729395	0	0
cov2_5_10_11	20.303	-215.954	0.4175776	0	0	0	-0.040647192
cov3_4_6_9	20.3085	-215.957	0.4191213	0	0	0	0
cov3_5_6_9	20.3116	-215.959	0.4184751	0	0	0	0
cov1_2_4_8	20.3135	-215.96	0.4194762	0	-0.061566576	0	-0.043988436
cov1_2_4_7	20.3192	-215.963	0.419475	0	-0.061554097	0	-0.044062484
cov1_4_10_11	20.3222	-215.964	0.4175226	0	-0.071338517	0	0
cov1_6_7_11	20.3326	-215.969	0.420111	0	-0.050249523	0	0
cov2_4_5_10	20.3338	-215.97	0.416122	0	0	0	-0.042064525
cov1_6_8_11	20.3349	-215.97	0.420106	0	-0.050978903	0	0

cov1_5_6_10	20.3379	-215.972	0.415699	0	-0.022461531	0	0
cov4_5_6_10	20.3389	-215.972	0.4166265	0	0	0	0
cov1_5_6_7	20.3409	-215.973	0.4192989	0	-0.052611846	0	0
cov1_2_4_10	20.344	-215.975	0.4167519	0	-0.046280964	0	-0.022302289
cov1_5_6_8	20.344	-215.975	0.4192889	0	-0.053277555	0	0
cov1_3_6_7	20.3476	-215.977	0.420079	0	-0.044922358	0	0
cov1_3_6_8	20.3494	-215.978	0.4200868	0	-0.045586131	0	0
cov3_6_9_11	20.3563	-215.981	0.4185275	0	0	0	0
cov4_6_10_11	20.357	-215.982	0.4181248	0	0	0	0
cov1_6_7_8	20.359	-215.983	0.4196548	0	-0.044312225	0	0
cov1_4_6_7	20.3595	-215.983	0.4196531	0	-0.044157245	0	0
cov1_4_6_8	20.3624	-215.984	0.4196487	0	-0.044809112	0	0
cov1_5_10_11	20.3639	-215.985	0.4163797	0	-0.063018858	0	0
cov1_2_5_8	20.3664	-215.986	0.4200855	0	-0.067970481	0	-0.033689595
cov1_2_8_11	20.3671	-215.987	0.4209619	0	-0.064707513	0	-0.034183765
cov3_5_10_11	20.3719	-215.989	0.4161829	0	0	0	0
cov1_2_5_7	20.3724	-215.989	0.4200856	0	-0.068010507	0	-0.033714704
cov1_2_7_11	20.3737	-215.99	0.4209619	0	-0.064657473	0	-0.034200862
cov1_2_3_8	20.3785	-215.992	0.4209469	0	-0.059225412	0	-0.033614426
cov1_2_3_7	20.385	-215.996	0.4209481	0	-0.059206476	0	-0.033633171
cov2_3_5_9	20.3852	-215.996	0.4195735	0	0	0	-0.052741955
cov1_2_7_8	20.3923	-215.999	0.4204846	0	-0.058433912	0	-0.033340923
cov3_4_5_10	20.3982	-216.002	0.4168273	0	0	0	0
cov2_3_5_6	20.4016	-216.004	0.4230178	0	0	0	-0.048859672
cov2_3_8_9	20.4036	-216.005	0.4194981	0	0	0	-0.071333674
cov1_2_3_5	20.4043	-216.005	0.4215671	0	-0.10290427	0	-0.036192989
cov2_3_7_9	20.4069	-216.006	0.4194945	0	0	0	-0.071319063
cov1_3_5_6	20.4153	-216.011	0.420628	0	-0.089296204	0	0
cov1_4_5_10	20.4197	-216.013	0.4163229	0	-0.050640825	0	0
cov2_8_9_11	20.4474	-216.027	0.4178345	0	0	0	-0.062431009
cov2_7_9_11	20.4496	-216.028	0.4178272	0	0	0	-0.062371612
cov1_2_3_4	20.46	-216.033	0.4209125	0	-0.071413751	0	-0.057573103
cov2_3_6_11	20.4739	-216.04	0.4237395	0	0	0	-0.044491006
cov1_2_3_6	20.4764	-216.041	0.4226288	0	-0.023857315	0	-0.032761618
cov2_4_8_9	20.4973	-216.052	0.418839	0	0	0	-0.064542066
cov2_4_7_9	20.5002	-216.053	0.4188325	0	0	0	-0.064569101
cov2_7_8_9	20.5087	-216.057	0.4186157	0	0	0	-0.070290498
cov4_8_10_11	20.5378	-216.072	0.4165546	0	0	0	0
cov2_6_9_11	20.5395	-216.073	0.4190304	0	0	0	-0.042043608
cov4_7_10_11	20.5398	-216.073	0.4165459	0	0	0	0
cov2_3_9_11	20.5462	-216.076	0.4194935	0	0	0	-0.066448977
cov3_4_9_10	20.5593	-216.083	0.4199359	0	0	0	0
cov2_3_4_9	20.5718	-216.089	0.4199261	0	0	0	-0.078908743
cov1_3_5_8	20.5754	-216.091	0.4196337	0	-0.11149972	0	0
cov1_3_5_7	20.5812	-216.094	0.4196392	0	-0.11174383	0	0
cov4_5_6_7	20.5841	-216.095	0.4205638	0	0	0	0
cov3_5_6_7	20.5892	-216.098	0.4201574	0	0	0	0

cov4_6_7_11	20.5895	-216.098	0.42059	0	0	0	0
cov4_6_7_8	20.5897	-216.098	0.4206742	0	0	0	0
cov5_8_9_11	20.5897	-216.098	0.4160219	0	0	0	0
cov5_6_7_8	20.5899	-216.098	0.4202662	0	0	0	0
cov5_6_7_11	20.59	-216.098	0.4202227	0	0	0	0
cov3_4_6_7	20.5901	-216.098	0.4206632	0	0	0	0
cov5_7_9_11	20.5929	-216.099	0.4160151	0	0	0	0
cov4_5_6_8	20.5935	-216.1	0.4205705	0	0	0	0
cov2_4_5_8	20.5985	-216.102	0.4201278	0	0	0	-0.080787949
cov3_5_6_8	20.5993	-216.103	0.4201789	0	0	0	0
cov4_6_8_11	20.5993	-216.103	0.4205999	0	0	0	0
cov5_6_8_11	20.5998	-216.103	0.4202248	0	0	0	0
cov3_4_6_8	20.5999	-216.103	0.4206918	0	0	0	0
cov2_4_5_7	20.6041	-216.105	0.4201254	0	0	0	-0.080868557
cov1_4_5_8	20.6134	-216.11	0.4194803	0	-0.096497774	0	0
cov3_5_8_9	20.6145	-216.11	0.4162107	0	0	0	0
cov3_5_7_9	20.6174	-216.112	0.4162	0	0	0	0
cov1_4_5_7	20.6199	-216.113	0.4194794	0	-0.096559295	0	0
cov2_5_6_9	20.6209	-216.113	0.4176175	0	0	0	-0.03182026
cov3_6_7_11	20.6211	-216.114	0.4199943	0	0	0	0
cov1_2_6_9	20.6214	-216.114	0.4174221	0	-0.021244211	0	-0.029479737
cov3_6_7_8	20.6239	-216.115	0.420148	0	0	0	0
cov1_5_8_11	20.624	-216.115	0.419261	0	-0.10109107	0	0
cov6_7_8_11	20.6247	-216.115	0.4197453	0	0	0	0
cov1_2_9_11	20.6297	-216.118	0.4183279	0	-0.066086701	0	-0.032534445
cov1_5_7_11	20.6306	-216.118	0.4192575	0	-0.10112115	0	0
cov3_6_8_11	20.6317	-216.119	0.4200146	0	0	0	0
cov1_3_8_11	20.6355	-216.121	0.4200057	0	-0.092935577	0	0
cov1_5_7_8	20.6379	-216.122	0.4189387	0	-0.09579546	0	0
cov1_3_7_11	20.6424	-216.124	0.4200069	0	-0.092920539	0	0
cov4_5_8_9	20.6433	-216.125	0.4166512	0	0	0	0
cov5_7_8_9	20.6436	-216.125	0.4165627	0	0	0	0
cov1_4_8_11	20.6446	-216.125	0.4197278	0	-0.091332227	0	0
cov1_7_8_11	20.645	-216.126	0.4196328	0	-0.092149003	0	0
cov4_5_7_9	20.6471	-216.127	0.4166444	0	0	0	0
cov1_3_4_8	20.6482	-216.127	0.4196203	0	-0.088969473	0	0
cov1_3_7_8	20.6486	-216.127	0.4196945	0	-0.087912317	0	0
cov1_3_6_11	20.6487	-216.127	0.4220585	0	-0.060464603	0	0
cov1_4_7_11	20.6515	-216.129	0.4197245	0	-0.091325448	0	0
cov1_3_4_7	20.655	-216.131	0.4196197	0	-0.088998378	0	0
cov1_4_7_8	20.6581	-216.132	0.4193949	0	-0.086296686	0	0
cov2_4_6_9	20.6604	-216.133	0.4179209	0	0	0	-0.033205287
cov1_3_4_5	20.6683	-216.137	0.4209663	0	-0.13122426	0	0
cov4_9_10_11	20.6703	-216.138	0.417798	0	0	0	0
cov1_6_9_11	20.6709	-216.138	0.4175357	0	-0.058842096	0	0
cov1_2_3_11	20.6754	-216.141	0.4229671	0	-0.074149829	0	-0.035422002
cov1_3_5_11	20.6829	-216.144	0.4206868	0	-0.13785339	0	0

cov1_3_4_6	20.6985	-216.152	0.4207349	0	-0.05763389	0	0
cov1_4_6_9	20.7477	-216.177	0.4173072	0	-0.034160756	0	0
cov4_5_10_11	20.749	-216.178	0.4160123	0	0	0	0
cov1_2_5_9	20.7674	-216.187	0.4170705	0	-0.041000278	0	-0.02979532
cov1_2_4_9	20.7799	-216.193	0.4172181	0	-0.049947743	0	-0.027188407
cov1_4_9_11	20.7872	-216.197	0.4180075	0	-0.078207609	0	0
cov2_4_10_11	20.7974	-216.202	0.4178528	0	0	0	-0.049838644
cov1_5_6_9	20.8102	-216.208	0.4163594	0	-0.03488434	0	0
cov1_2_6_11	20.8168	-216.211	0.421453	0	-0.038281608	0	-0.033362036
cov5_6_9_11	20.8265	-216.216	0.4173515	0	0	0	0
cov4_6_9_11	20.8276	-216.217	0.4186784	0	0	0	0
cov3_4_10_11	20.8319	-216.219	0.4173181	0	0	0	0
cov4_7_8_10	20.8487	-216.227	0.4183028	0	0	0	0
cov3_4_8_10	20.8488	-216.227	0.4183041	0	0	0	0
cov3_4_7_10	20.8518	-216.229	0.4182976	0	0	0	0
cov4_5_6_9	20.8553	-216.231	0.4174391	0	0	0	0
cov1_5_9_11	20.8557	-216.231	0.4170252	0	-0.079182806	0	0
cov2_5_9_11	20.8839	-216.245	0.4182124	0	0	0	-0.046554873
cov2_4_5_9	20.889	-216.248	0.4167422	0	0	0	-0.049657316
cov7_9_10_11	20.8953	-216.251	0.4125677	0	0	0	0
cov8_9_10_11	20.8974	-216.252	0.4125662	0	0	0	0
cov1_4_5_9	20.9054	-216.256	0.4170669	0	-0.066265724	0	0
cov2_3_5_8	20.9245	-216.265	0.4216049	0	0	0	-0.059035867
cov2_5_8_11	20.9288	-216.267	0.4217084	0	0	0	-0.060143816
cov2_3_5_7	20.9306	-216.268	0.4215961	0	0	0	-0.059017497
cov2_4_6_11	20.9311	-216.269	0.421179	0	0	0	-0.050691859
cov2_5_7_11	20.9351	-216.271	0.4217062	0	0	0	-0.060162774
cov2_5_6_11	20.9516	-216.279	0.4216831	0	0	0	-0.044856921
cov1_3_4_11	20.9534	-216.28	0.4210307	0	-0.10827564	0	0
cov2_5_7_8	20.9591	-216.283	0.4224019	0	0	0	-0.063839657
cov1_2_5_6	20.992	-216.299	0.4194231	0	-0.037159152	0	-0.031372351
cov1_2_4_6	21.0147	-216.31	0.4193369	0	-0.025755523	0	-0.036759286
cov3_9_10_11	21.0413	-216.324	0.4140003	0	0	0	0
cov1_5_6_11	21.0421	-216.324	0.4196207	0	-0.076109686	0	0
cov3_4_5_6	21.0539	-216.33	0.4221668	0	0	0	0
cov1_2_5_11	21.0601	-216.333	0.4204839	0	-0.090623859	0	-0.034904638
cov2_4_8_11	21.0621	-216.334	0.4204311	0	0	0	-0.078150488
cov2_4_7_11	21.0666	-216.336	0.4204246	0	0	0	-0.078191223
cov1_4_6_11	21.0719	-216.339	0.4205624	0	-0.060492664	0	0
cov3_4_6_11	21.0755	-216.341	0.4220604	0	0	0	0
cov1_2_4_11	21.0786	-216.342	0.4203863	0	-0.079960647	0	-0.041115155
cov2_4_5_6	21.0791	-216.343	0.4197894	0	0	0	-0.044889267
cov3_5_6_11	21.0811	-216.344	0.4216386	0	0	0	0
cov2_3_8_11	21.0817	-216.344	0.4211202	0	0	0	-0.071848232
cov2_7_8_11	21.0835	-216.345	0.4209409	0	0	0	-0.071559807
cov2_3_7_11	21.087	-216.347	0.4211112	0	0	0	-0.071805789
cov1_4_5_6	21.129	-216.367	0.4195405	0	-0.063820577	0	0

cov4_8_9_11	21.1326	-216.369	0.4169139	0	0	0	0
cov4_7_9_11	21.1351	-216.371	0.4169049	0	0	0	0
cov2_3_4_8	21.1644	-216.385	0.4219622	0	0	0	-0.091478725
cov2_3_4_7	21.17	-216.388	0.4219589	0	0	0	-0.091508131
cov3_5_9_11	21.1727	-216.389	0.4164462	0	0	0	0
cov2_4_7_8	21.1737	-216.39	0.4216764	0	0	0	-0.088164599
cov2_3_7_8	21.189	-216.398	0.422188	0	0	0	-0.082481248
cov3_4_5_9	21.2042	-216.405	0.417297	0	0	0	0
cov2_3_4_5	21.2306	-216.418	0.4219466	0	0	0	-0.098472078
cov1_2_4_5	21.2389	-216.422	0.4192119	0	-0.076295778	0	-0.031263676
cov1_4_5_11	21.2392	-216.423	0.4203714	0	-0.11824755	0	0
cov2_4_9_11	21.3014	-216.454	0.4183101	0	0	0	-0.056567912
cov3_8_10_11	21.3774	-216.492	0.4126267	0	0	0	0
cov3_7_10_11	21.3782	-216.492	0.4126182	0	0	0	0
cov4_5_6_11	21.4496	-216.528	0.4212298	0	0	0	0
cov4_5_9_11	21.4716	-216.539	0.4164551	0	0	0	0
cov3_4_8_9	21.4822	-216.544	0.4185281	0	0	0	0
cov3_4_7_9	21.4854	-216.546	0.4185189	0	0	0	0
cov4_7_8_9	21.4953	-216.551	0.4187325	0	0	0	0
cov2_3_4_11	21.5202	-216.563	0.4222973	0	0	0	-0.097891782
cov7_8_10_11	21.5219	-216.564	0.4117888	0	0	0	0
cov3_4_9_11	21.5659	-216.586	0.4176482	0	0	0	0
cov2_3_5_11	21.6088	-216.607	0.4233623	0	0	0	-0.073690364
cov2_4_5_11	21.79	-216.698	0.420631	0	0	0	-0.081791464
cov3_5_8_11	21.8157	-216.711	0.4183662	0	0	0	0
cov3_5_7_11	21.8189	-216.712	0.4183473	0	0	0	0
cov3_4_5_8	21.9684	-216.787	0.4195673	0	0	0	0
cov3_5_7_8	21.9721	-216.789	0.4192617	0	0	0	0
cov3_4_5_7	21.9726	-216.789	0.4195467	0	0	0	0
cov3_8_9_11	22.0069	-216.806	0.4128376	0	0	0	0
cov3_7_9_11	22.0077	-216.807	0.4128297	0	0	0	0
cov7_8_9_11	22.0791	-216.843	0.4122371	0	0	0	0
cov4_5_8_11	22.0904	-216.848	0.4199841	0	0	0	0
cov5_7_8_11	22.0953	-216.851	0.4197307	0	0	0	0
cov4_5_7_11	22.0975	-216.852	0.4199826	0	0	0	0
cov3_4_8_11	22.3411	-216.974	0.4194823	0	0	0	0
cov3_4_7_11	22.3446	-216.975	0.419463	0	0	0	0
cov4_5_7_8	22.49	-217.048	0.4222331	0	0	0	0
cov4_7_8_11	22.4992	-217.053	0.4203895	0	0	0	0
cov3_8_9_10	22.9903	-217.298	0.4133597	0	0	0	0
cov3_7_9_10	22.9916	-217.299	0.4133554	0	0	0	0
cov3_4_7_8	23.0112	-217.309	0.4220534	0	0	0	0
cov7_8_9_10	23.0655	-217.336	0.412736	0	0	0	0
cov3_4_5_11	23.2621	-217.434	0.4212967	0	0	0	0
cov3_7_8_11	23.3308	-217.468	0.4153557	0	0	0	0
cov3_7_8_10	23.352	-217.479	0.4127174	0	0	0	0
cov3_7_8_9	24.045	-217.826	0.4127594	0	0	0	0

cov2_3_6_9_10	25.5971	-215.079	0.4207881	0	0	0	-0.03680259
cov1_2_3_9_10	25.7	-215.131	0.4199646	0	-0.05612329	0	-0.028904775
cov2_6_7_9_10	25.7004	-215.131	0.4191274	0	0	0	-0.034657882
cov2_6_8_9_10	25.7069	-215.134	0.4191259	0	0	0	-0.034885436
cov1_3_6_9_10	25.7189	-215.14	0.4192479	0	-0.048427281	0	0
cov5_6_7_9_10	25.7782	-215.17	0.4179737	0	0	0	0
cov5_6_8_9_10	25.7834	-215.172	0.4179658	0	0	0	0
cov1_3_8_9_10	25.8223	-215.192	0.4184334	0	-0.079639796	0	0
cov1_3_7_9_10	25.8256	-215.193	0.4184355	0	-0.079637398	0	0
cov1_6_7_9_10	25.837	-215.199	0.4177213	0	-0.043383741	0	0
cov1_6_8_9_10	25.8404	-215.201	0.417712	0	-0.04405112	0	0
cov1_2_8_9_10	25.8476	-215.204	0.4183363	0	-0.053859212	0	-0.02838089
cov2_5_8_9_10	25.8484	-215.205	0.4187633	0	0	0	-0.036825016
cov1_2_7_9_10	25.8512	-215.206	0.418333	0	-0.053807238	0	-0.028398828
cov2_5_7_9_10	25.8518	-215.206	0.4187593	0	0	0	-0.036828886
cov1_3_9_10_11	25.8876	-215.224	0.4190596	0	-0.085280985	0	0
cov1_3_5_9_10	25.8951	-215.228	0.4187947	0	-0.086341867	0	0
cov1_3_4_9_10	25.8987	-215.23	0.4188576	0	-0.079261882	0	0
cov3_6_7_9_10	25.9235	-215.242	0.4188372	0	0	0	0
cov3_6_8_9_10	25.9281	-215.245	0.4188463	0	0	0	0
cov1_5_8_9_10	25.9296	-215.245	0.4174237	0	-0.051664394	0	0
cov1_5_7_9_10	25.9336	-215.247	0.4174195	0	-0.051633729	0	0
cov4_6_7_9_10	25.9406	-215.251	0.4189893	0	0	0	0
cov3_5_6_9_10	25.9407	-215.251	0.4191483	0	0	0	0
cov4_6_8_9_10	25.9481	-215.255	0.4189934	0	0	0	0
cov1_4_8_9_10	25.9823	-215.272	0.4180273	0	-0.067465295	0	0
cov3_4_6_9_10	25.9841	-215.273	0.4198943	0	0	0	0
cov1_4_7_9_10	25.9864	-215.274	0.4180221	0	-0.06744562	0	0
cov1_8_9_10_11	26.0355	-215.298	0.4174093	0	-0.080973302	0	0
cov3_6_9_10_11	26.0374	-215.299	0.4190628	0	0	0	0
cov1_7_9_10_11	26.0395	-215.3	0.4174021	0	-0.080889683	0	0
cov1_7_8_9_10	26.04	-215.301	0.4172131	0	-0.077943946	0	0
cov2_3_5_9_10	26.0634	-215.312	0.4201053	0	0	0	-0.047427813
cov6_7_9_10_11	26.0856	-215.323	0.4174798	0	0	0	0
cov6_7_8_9_10	26.0952	-215.328	0.4177606	0	0	0	0
cov6_8_9_10_11	26.0971	-215.329	0.4174657	0	0	0	0
cov5_8_9_10_11	26.2245	-215.393	0.4166505	0	0	0	0
cov5_7_9_10_11	26.2275	-215.394	0.416644	0	0	0	0
cov3_5_8_9_10	26.271	-215.416	0.4170529	0	0	0	0
cov4_5_8_9_10	26.2734	-215.417	0.4170549	0	0	0	0
cov5_7_8_9_10	26.2741	-215.418	0.4171746	0	0	0	0
cov3_5_7_9_10	26.2742	-215.418	0.4170447	0	0	0	0
cov4_5_7_9_10	26.2769	-215.419	0.4170495	0	0	0	0
cov2_3_8_9_10	26.2939	-215.428	0.4203161	0	0	0	-0.07231035
cov2_3_7_9_10	26.2968	-215.429	0.4203095	0	0	0	-0.072290037
cov2_3_6_7_10	26.3133	-215.437	0.4190355	0	0	0	-0.036317816
cov2_3_6_8_10	26.3147	-215.438	0.4190449	0	0	0	-0.0364442

cov2_3_9_10_11	26.3177	-215.439	0.4199048	0	0	0	-0.064345472
cov2_3_4_6_10	26.3561	-215.459	0.4191041	0	0	0	-0.049817036
cov1_2_3_6_10	26.3794	-215.47	0.4191642	0	-0.020406759	0	-0.030215025
cov2_5_6_7_10	26.3871	-215.474	0.4176947	0	0	0	-0.027344547
cov2_5_6_8_10	26.3901	-215.476	0.4176934	0	0	0	-0.027469619
cov2_8_9_10_11	26.3907	-215.476	0.4180818	0	0	0	-0.060820675
cov2_7_9_10_11	26.3925	-215.477	0.4180749	0	0	0	-0.060758975
cov2_3_4_9_10	26.3973	-215.479	0.4206962	0	0	0	-0.075856803
cov2_3_6_10_11	26.4002	-215.481	0.419945	0	0	0	-0.039338011
cov2_5_6_9_10	26.4051	-215.483	0.4179825	0	0	0	-0.026990011
cov2_3_5_6_10	26.4197	-215.49	0.4194185	0	0	0	-0.036186332
cov1_2_3_8_10	26.4378	-215.499	0.4184047	0	-0.047795104	0	-0.031027919
cov1_2_3_7_10	26.4408	-215.501	0.4184051	0	-0.047783326	0	-0.031033491
cov2_6_9_10_11	26.4411	-215.501	0.4192808	0	0	0	-0.039726379
cov2_4_5_8_10	26.4454	-215.503	0.4164167	0	0	0	-0.048792607
cov2_4_5_7_10	26.4477	-215.504	0.4164105	0	0	0	-0.048827857
cov2_4_8_9_10	26.4519	-215.507	0.4194902	0	0	0	-0.060374946
cov2_4_7_9_10	26.4546	-215.508	0.4194845	0	0	0	-0.06040052
cov1_2_6_9_10	26.4607	-215.511	0.4177914	0	-0.026520725	0	-0.02617446
cov1_2_3_4_10	26.461	-215.511	0.4183515	0	-0.053527766	0	-0.042636821
cov1_2_6_7_10	26.4728	-215.517	0.4176272	0	-0.015835166	0	-0.029658811
cov1_2_6_8_10	26.4764	-215.519	0.4176178	0	-0.01651608	0	-0.029651188
cov2_6_7_10_11	26.4859	-215.524	0.4182495	0	0	0	-0.036758199
cov2_7_8_9_10	26.4905	-215.526	0.4190865	0	0	0	-0.070838663
cov2_6_8_10_11	26.4918	-215.526	0.4182488	0	0	0	-0.036975983
cov1_2_3_10_11	26.4948	-215.528	0.4192852	0	-0.055650871	0	-0.031918854
cov1_6_9_10_11	26.4965	-215.529	0.4178957	0	-0.060068886	0	0
cov1_2_9_10_11	26.4976	-215.529	0.4185705	0	-0.069336696	0	-0.029016891
cov2_4_6_7_10	26.4979	-215.529	0.4178827	0	0	0	-0.034706479
cov2_6_7_8_10	26.4979	-215.53	0.4178663	0	0	0	-0.03503446
cov1_3_6_7_10	26.4995	-215.53	0.4175314	0	-0.042419173	0	0
cov1_3_6_8_10	26.4996	-215.53	0.4175331	0	-0.042762721	0	0
cov2_4_6_8_10	26.5037	-215.532	0.4178889	0	0	0	-0.034798574
cov2_4_6_9_10	26.5059	-215.534	0.4185644	0	0	0	-0.028165987
cov1_2_3_5_10	26.5197	-215.54	0.4188157	0	-0.055356653	0	-0.031422789
cov1_4_6_9_10	26.5203	-215.541	0.4179207	0	-0.035423353	0	0
cov1_2_5_8_10	26.5386	-215.55	0.41736	0	-0.022540551	0	-0.029057532
cov1_2_5_7_10	26.5418	-215.551	0.4173568	0	-0.02249581	0	-0.029075687
cov5_6_9_10_11	26.5423	-215.552	0.4178098	0	0	0	0
cov1_2_5_9_10	26.5478	-215.554	0.4174945	0	-0.035761819	0	-0.025808666
cov1_5_6_9_10	26.55	-215.556	0.4169079	0	-0.027508852	0	0
cov2_3_5_8_10	26.559	-215.56	0.4181254	0	0	0	-0.039608836
cov3_5_6_7_10	26.5594	-215.56	0.4172047	0	0	0	0
cov2_3_5_7_10	26.5623	-215.562	0.4181203	0	0	0	-0.039603034
cov3_5_6_8_10	26.5627	-215.562	0.4172091	0	0	0	0
cov1_3_6_10_11	26.5657	-215.563	0.4183845	0	-0.051113536	0	0
cov1_5_6_7_10	26.5775	-215.569	0.4166316	0	-0.01571151	0	0

cov1_4_9_10_11	26.5794	-215.57	0.4185125	0	-0.077044247	0	0
cov1_5_6_8_10	26.5807	-215.571	0.4166228	0	-0.016350418	0	0
cov4_5_6_9_10	26.581	-215.571	0.4177194	0	0	0	0
cov2_5_8_10_11	26.5882	-215.575	0.4176666	0	0	0	-0.036594732
cov2_5_7_8_10	26.5884	-215.575	0.4175901	0	0	0	-0.036190729
cov2_3_4_5_10	26.5885	-215.575	0.417801	0	0	0	-0.064650332
cov2_5_7_10_11	26.5912	-215.576	0.4176589	0	0	0	-0.036579152
cov5_6_7_10_11	26.5926	-215.577	0.4168843	0	0	0	0
cov1_3_5_6_10	26.5928	-215.577	0.4179271	0	-0.050295369	0	0
cov1_3_4_6_10	26.596	-215.579	0.4180128	0	-0.043307581	0	0
cov4_5_6_7_10	26.5963	-215.579	0.4167813	0	0	0	0
cov5_6_7_8_10	26.5968	-215.579	0.416694	0	0	0	0
cov5_6_8_10_11	26.5974	-215.579	0.4168775	0	0	0	0
cov1_2_4_9_10	26.5984	-215.58	0.4177888	0	-0.05364706	0	-0.020845631
cov4_5_6_8_10	26.6012	-215.581	0.4167792	0	0	0	0
cov1_5_9_10_11	26.6114	-215.586	0.4174675	0	-0.068915056	0	0
cov1_2_8_10_11	26.624	-215.593	0.4176172	0	-0.051984499	0	-0.031159254
cov1_4_6_7_10	26.6263	-215.594	0.4172541	0	-0.030876844	0	0
cov1_2_7_10_11	26.6274	-215.594	0.4176103	0	-0.051894533	0	-0.031164779
cov2_4_5_9_10	26.6279	-215.595	0.4170971	0	0	0	-0.044019788
cov4_6_9_10_11	26.629	-215.595	0.4191699	0	0	0	0
cov1_4_6_8_10	26.6291	-215.595	0.4172509	0	-0.03146164	0	0
cov2_5_9_10_11	26.6351	-215.598	0.4185119	0	0	0	-0.040410943
cov1_2_4_8_10	26.641	-215.601	0.4172503	0	-0.046398758	0	-0.029629267
cov1_2_7_8_10	26.6411	-215.601	0.4172043	0	-0.046712202	0	-0.030452226
cov1_2_4_7_10	26.6441	-215.603	0.4172459	0	-0.046362224	0	-0.02967682
cov1_3_4_8_10	26.6534	-215.607	0.4174533	0	-0.068227269	0	0
cov3_4_6_7_10	26.6541	-215.608	0.4182602	0	0	0	0
cov1_3_5_8_10	26.6544	-215.608	0.4170091	0	-0.06242597	0	0
cov1_3_4_7_10	26.6567	-215.609	0.4174526	0	-0.06824725	0	0
cov1_3_5_7_10	26.6579	-215.61	0.4170093	0	-0.062524852	0	0
cov3_4_6_8_10	26.6579	-215.61	0.4182722	0	0	0	0
cov1_3_8_10_11	26.6612	-215.611	0.4173613	0	-0.077636564	0	0
cov1_4_5_9_10	26.6632	-215.612	0.4173751	0	-0.057323419	0	0
cov1_3_7_10_11	26.6643	-215.613	0.417364	0	-0.077645554	0	0
cov1_6_7_10_11	26.666	-215.614	0.4168204	0	-0.044773792	0	0
cov1_3_7_8_10	26.6672	-215.614	0.4171306	0	-0.074018902	0	0
cov1_6_8_10_11	26.669	-215.615	0.4168177	0	-0.045472591	0	0
cov3_5_9_10_11	26.6788	-215.62	0.4171687	0	0	0	0
cov1_6_7_8_10	26.6829	-215.622	0.4164356	0	-0.039981254	0	0
cov3_4_5_9_10	26.7309	-215.646	0.4177956	0	0	0	0
cov1_3_4_10_11	26.731	-215.646	0.4180823	0	-0.077525513	0	0
cov1_5_8_10_11	26.7332	-215.647	0.4164524	0	-0.048537615	0	0
cov1_3_5_10_11	26.7359	-215.649	0.4178513	0	-0.083256164	0	0
cov4_6_7_8_10	26.7361	-215.649	0.4177625	0	0	0	0
cov4_6_7_10_11	26.7363	-215.649	0.4177626	0	0	0	0
cov1_4_5_8_10	26.7365	-215.649	0.4164444	0	-0.045307199	0	0

cov1_5_7_10_11	26.7368	-215.649	0.4164451	0	-0.048452648	0	0
cov3_6_7_10_11	26.739	-215.65	0.4172595	0	0	0	0
cov1_5_7_8_10	26.7396	-215.65	0.4162178	0	-0.04472699	0	0
cov1_4_5_7_10	26.7399	-215.651	0.416439	0	-0.04527296	0	0
cov1_3_4_5_10	26.7417	-215.651	0.4179215	0	-0.078763426	0	0
cov2_3_6_7_9	26.743	-215.652	0.4195613	0	0	0	-0.037135233
cov4_6_8_10_11	26.7433	-215.652	0.4177671	0	0	0	0
cov3_6_8_10_11	26.7436	-215.652	0.417267	0	0	0	0
cov2_3_6_8_9	26.7451	-215.653	0.4195727	0	0	0	-0.03729605
cov3_6_7_8_10	26.7457	-215.653	0.4174903	0	0	0	0
cov3_4_5_6_10	26.773	-215.667	0.4180944	0	0	0	0
cov3_5_6_10_11	26.7738	-215.667	0.4179832	0	0	0	0
cov1_4_8_10_11	26.7808	-215.671	0.4172012	0	-0.064911744	0	0
cov1_4_7_10_11	26.7845	-215.673	0.4171909	0	-0.064848749	0	0
cov1_4_7_8_10	26.7898	-215.675	0.416917	0	-0.060726966	0	0
cov2_3_5_10_11	26.7963	-215.679	0.4189665	0	0	0	-0.046888588
cov2_5_6_7_9	26.8059	-215.684	0.4185671	0	0	0	-0.031370102
cov3_4_6_10_11	26.807	-215.684	0.4186772	0	0	0	0
cov2_5_6_8_9	26.8097	-215.685	0.4185653	0	0	0	-0.031499156
cov2_3_4_6_9	26.8105	-215.686	0.4196426	0	0	0	-0.054612323
cov1_2_6_7_9	26.8247	-215.693	0.4184421	0	-0.015710938	0	-0.030792785
cov1_2_6_8_9	26.8282	-215.695	0.418432	0	-0.016409447	0	-0.030783605
cov2_6_7_9_11	26.8339	-215.698	0.4191014	0	0	0	-0.038071065
cov2_6_8_9_11	26.8397	-215.7	0.4191002	0	0	0	-0.038291757
cov2_4_6_7_9	26.842	-215.702	0.4184841	0	0	0	-0.03996826
cov2_4_6_8_9	26.8483	-215.705	0.4184901	0	0	0	-0.04006396
cov2_6_7_8_9	26.8494	-215.705	0.4186719	0	0	0	-0.036126359
cov1_7_8_10_11	26.8546	-215.708	0.4162532	0	-0.076530891	0	0
cov1_2_3_6_9	26.8786	-215.72	0.4198102	0	-0.020189545	0	-0.031389327
cov2_3_8_10_11	26.8822	-215.722	0.4182695	0	0	0	-0.060633616
cov2_3_7_10_11	26.8849	-215.723	0.4182625	0	0	0	-0.060626113
cov2_3_6_9_11	26.8867	-215.724	0.4206883	0	0	0	-0.040960391
cov6_7_8_10_11	26.8968	-215.729	0.4163024	0	0	0	0
cov1_2_3_8_9	26.9	-215.731	0.4188817	0	-0.049660033	0	-0.032224471
cov1_2_3_7_9	26.904	-215.733	0.4188809	0	-0.04963634	0	-0.032234337
cov2_3_5_6_9	26.9133	-215.737	0.4202173	0	0	0	-0.040197665
cov4_8_9_10_11	26.9187	-215.74	0.4174411	0	0	0	0
cov4_7_9_10_11	26.921	-215.741	0.417432	0	0	0	0
cov2_4_5_8_9	26.94	-215.751	0.4170886	0	0	0	-0.056552869
cov2_4_5_7_9	26.943	-215.752	0.4170824	0	0	0	-0.056585872
cov1_2_3_4_9	26.9431	-215.752	0.4188305	0	-0.05708987	0	-0.047182394
cov1_3_6_7_9	26.948	-215.755	0.4180627	0	-0.042520191	0	0
cov1_3_6_8_9	26.9485	-215.755	0.4180648	0	-0.042968239	0	0
cov2_3_7_8_10	26.9507	-215.756	0.4191104	0	0	0	-0.068983445
cov2_3_4_8_10	26.9508	-215.756	0.419119	0	0	0	-0.068267381
cov2_3_4_7_10	26.9535	-215.757	0.4191172	0	0	0	-0.068293881
cov5_7_8_10_11	26.9712	-215.766	0.415648	0	0	0	0

cov3_5_8_10_11	26.9717	-215.766	0.4156446	0	0	0	0
cov4_5_8_10_11	26.9717	-215.766	0.4156591	0	0	0	0
cov3_5_7_10_11	26.9743	-215.768	0.415634	0	0	0	0
cov4_5_7_10_11	26.9743	-215.768	0.4156489	0	0	0	0
cov1_2_5_8_9	26.9914	-215.776	0.4180361	0	-0.0360062	0	-0.031013824
cov1_2_5_7_9	26.9953	-215.778	0.4180315	0	-0.035961005	0	-0.03103205
cov3_5_7_8_10	26.9971	-215.779	0.4159465	0	0	0	0
cov3_4_5_8_10	26.9977	-215.779	0.4159584	0	0	0	0
cov1_2_8_9_11	26.9998	-215.78	0.4184015	0	-0.054703712	0	-0.032454479
cov4_5_7_8_10	26.9998	-215.78	0.4160527	0	0	0	0
cov3_4_5_7_10	27.0005	-215.781	0.4159479	0	0	0	0
cov1_2_3_5_9	27.0006	-215.781	0.4194801	0	-0.069269621	0	-0.033412869
cov1_2_7_9_11	27.004	-215.783	0.418394	0	-0.054611225	0	-0.032465702
cov1_2_3_9_11	27.0063	-215.784	0.4199616	0	-0.059048325	0	-0.03335452
cov2_3_4_10_11	27.0115	-215.786	0.4187766	0	0	0	-0.067664247
cov1_2_4_8_9	27.0154	-215.788	0.4177816	0	-0.050175241	0	-0.034648991
cov1_2_4_7_9	27.0192	-215.79	0.417776	0	-0.050132553	0	-0.034697468
cov1_2_7_8_9	27.0205	-215.791	0.4179533	0	-0.048938943	0	-0.031683749
cov1_5_6_7_9	27.0219	-215.792	0.4173371	0	-0.027926234	0	0
cov1_4_6_7_9	27.0238	-215.792	0.4177989	0	-0.034360047	0	0
cov1_5_6_8_9	27.0252	-215.793	0.4173282	0	-0.028580851	0	0
cov1_4_6_8_9	27.0266	-215.794	0.4177954	0	-0.034965766	0	0
cov1_6_7_9_11	27.0304	-215.796	0.4176596	0	-0.04611507	0	0
cov2_3_4_6_7	27.032	-215.797	0.4209184	0	0	0	-0.057563083
cov1_6_8_9_11	27.0333	-215.797	0.417656	0	-0.046823666	0	0
cov2_3_4_6_8	27.036	-215.799	0.4209396	0	0	0	-0.057788395
cov2_4_8_10_11	27.0442	-215.803	0.41754	0	0	0	-0.051420948
cov2_4_7_10_11	27.0461	-215.804	0.4175305	0	0	0	-0.051424325
cov1_6_7_8_9	27.0512	-215.806	0.4172369	0	-0.040813456	0	0
cov2_5_6_10_11	27.0559	-215.809	0.4182252	0	0	0	-0.032335206
cov1_3_5_6_9	27.0673	-215.814	0.4186199	0	-0.062974125	0	0
cov1_3_6_9_11	27.0679	-215.815	0.4191101	0	-0.053155578	0	0
cov3_5_6_7_9	27.0682	-215.815	0.4177657	0	0	0	0
cov2_4_5_6_7	27.07	-215.816	0.4198134	0	0	0	-0.052166957
cov2_7_8_10_11	27.0707	-215.816	0.4171469	0	0	0	-0.059725244
cov1_2_4_6_7	27.0725	-215.817	0.4197409	0	-0.020836023	0	-0.043184354
cov3_5_6_8_9	27.0731	-215.817	0.4177722	0	0	0	0
cov2_4_5_6_8	27.076	-215.819	0.4198181	0	0	0	-0.052342575
cov1_2_4_6_8	27.0767	-215.819	0.4197242	0	-0.021547847	0	-0.04312251
cov5_6_7_9_11	27.0807	-215.821	0.4175831	0	0	0	0
cov4_5_6_7_9	27.081	-215.821	0.4176217	0	0	0	0
cov5_6_7_8_9	27.0831	-215.822	0.4174422	0	0	0	0
cov4_5_9_10_11	27.0841	-215.823	0.4168332	0	0	0	0
cov5_6_8_9_11	27.0868	-215.824	0.4175757	0	0	0	0
cov4_5_6_8_9	27.0871	-215.824	0.4176195	0	0	0	0
cov2_4_7_8_10	27.0968	-215.829	0.4183336	0	0	0	-0.057692048
cov2_4_6_7_11	27.0972	-215.829	0.4206081	0	0	0	-0.05153083

cov1_2_6_10_11	27.1039	-215.833	0.4180826	0	-0.033921662	0	-0.030597998
cov2_4_6_8_11	27.1047	-215.833	0.4206179	0	0	0	-0.051650856
cov2_3_5_8_9	27.1106	-215.836	0.4186373	0	0	0	-0.044234947
cov1_3_4_6_9	27.1137	-215.837	0.4184869	0	-0.04622149	0	0
cov2_3_5_7_9	27.1144	-215.838	0.418629	0	0	0	-0.044220767
cov2_4_6_7_8	27.1155	-215.838	0.4201536	0	0	0	-0.049451621
cov1_2_6_7_11	27.1164	-215.839	0.4214213	0	-0.024777569	0	-0.033141874
cov2_5_7_8_9	27.118	-215.84	0.4183653	0	0	0	-0.042465671
cov2_5_8_9_11	27.1182	-215.84	0.418336	0	0	0	-0.042306337
cov1_2_6_8_11	27.1188	-215.84	0.4214152	0	-0.025506483	0	-0.033142191
cov2_5_7_9_11	27.1218	-215.841	0.4183279	0	0	0	-0.042291723
cov3_4_6_7_9	27.122	-215.842	0.4186871	0	0	0	0
cov3_4_6_8_9	27.1272	-215.844	0.4187002	0	0	0	0
cov1_2_5_6_7	27.1344	-215.848	0.4204241	0	-0.027278193	0	-0.032436275
cov1_2_5_6_8	27.1376	-215.849	0.4204134	0	-0.027958378	0	-0.032426299
cov1_3_8_9_11	27.139	-215.85	0.4178623	0	-0.081216584	0	0
cov1_3_4_8_9	27.1408	-215.851	0.4178161	0	-0.072747678	0	0
cov1_2_3_6_7	27.142	-215.852	0.4213105	0	-0.018687209	0	-0.032385108
cov1_3_7_9_11	27.1431	-215.852	0.4178622	0	-0.081206255	0	0
cov1_2_3_6_8	27.1437	-215.852	0.4213186	0	-0.019343062	0	-0.032388597
cov1_3_4_7_9	27.1451	-215.853	0.4178135	0	-0.072754527	0	0
cov1_3_7_8_9	27.1479	-215.855	0.4175912	0	-0.076947264	0	0
cov1_3_5_8_9	27.1484	-215.855	0.4175819	0	-0.076520207	0	0
cov1_3_5_7_9	27.1525	-215.857	0.417582	0	-0.076630234	0	0
cov1_2_6_7_8	27.1575	-215.859	0.4208072	0	-0.018182316	0	-0.032098101
cov2_3_6_7_11	27.1589	-215.86	0.4220255	0	0	0	-0.04079089
cov4_6_7_8_9	27.1593	-215.86	0.418348	0	0	0	0
cov4_6_7_9_11	27.1595	-215.86	0.4183482	0	0	0	0
cov2_3_6_8_11	27.1632	-215.862	0.4220467	0	0	0	-0.041014769
cov2_3_5_6_7	27.1633	-215.862	0.4216963	0	0	0	-0.041674823
cov4_6_8_9_11	27.1672	-215.864	0.4183523	0	0	0	0
cov2_3_5_6_8	27.1677	-215.864	0.4217188	0	0	0	-0.041881147
cov1_2_3_5_6	27.1691	-215.865	0.421933	0	-0.063059629	0	-0.034971314
cov2_5_6_7_11	27.1714	-215.866	0.4215384	0	0	0	-0.040670976
cov2_6_7_8_11	27.1717	-215.866	0.4215704	0	0	0	-0.040366258
cov2_3_6_7_8	27.1768	-215.869	0.4215837	0	0	0	-0.038727447
cov2_5_6_8_11	27.1773	-215.869	0.4215431	0	0	0	-0.040825815
cov2_4_6_10_11	27.1867	-215.874	0.4185648	0	0	0	-0.033782016
cov2_5_6_7_8	27.1889	-215.875	0.4210618	0	0	0	-0.038978177
cov3_6_7_9_11	27.1909	-215.876	0.4178292	0	0	0	0
cov2_4_9_10_11	27.1927	-215.877	0.418734	0	0	0	-0.050649234
cov3_6_7_8_9	27.1952	-215.878	0.4180159	0	0	0	0
cov1_2_5_10_11	27.197	-215.879	0.4176792	0	-0.041958934	0	-0.029940877
cov3_6_8_9_11	27.1971	-215.879	0.4178369	0	0	0	0
cov2_3_4_5_9	27.1992	-215.88	0.4183927	0	0	0	-0.071621231
cov2_4_5_6_10	27.203	-215.882	0.4165544	0	0	0	-0.030203281
cov1_2_3_4_6	27.2052	-215.883	0.4212098	0	-0.03037751	0	-0.057139594

cov1_2_5_6_10	27.2068	-215.884	0.4167635	0	-0.002536686	0	-0.027193583
cov1_4_6_10_11	27.2086	-215.885	0.4179552	0	-0.043877042	0	0
cov1_4_8_9_11	27.2088	-215.885	0.4177126	0	-0.071627071	0	0
cov1_5_8_9_11	27.2111	-215.886	0.4171283	0	-0.064569985	0	0
cov3_4_9_10_11	27.2114	-215.886	0.4182062	0	0	0	0
cov1_4_5_8_9	27.2117	-215.886	0.4172171	0	-0.060902923	0	0
cov1_4_7_9_11	27.2133	-215.887	0.4177039	0	-0.071564467	0	0
cov1_5_7_9_11	27.2156	-215.888	0.4171204	0	-0.064492269	0	0
cov1_4_5_7_9	27.216	-215.889	0.4172113	0	-0.060872553	0	0
cov1_4_7_8_9	27.2201	-215.891	0.4174019	0	-0.066985087	0	0
cov1_5_7_8_9	27.2207	-215.891	0.4168482	0	-0.060010819	0	0
cov1_3_5_9_11	27.2412	-215.901	0.4185173	0	-0.099675404	0	0
cov1_3_4_5_9	27.2425	-215.902	0.418683	0	-0.094526801	0	0
cov1_5_6_10_11	27.2455	-215.903	0.4168757	0	-0.03598923	0	0
cov1_7_8_9_11	27.2501	-215.906	0.4170164	0	-0.080363081	0	0
cov2_3_4_6_11	27.252	-215.907	0.4224361	0	0	0	-0.068335014
cov1_2_4_10_11	27.2561	-215.909	0.4178952	0	-0.060505261	0	-0.025604852
cov1_2_4_6_10	27.2564	-215.909	0.4170425	0	-0.019571182	0	-0.022489327
cov1_2_3_4_8	27.2693	-215.915	0.4201992	0	-0.065826872	0	-0.051368217
cov1_3_4_9_11	27.2697	-215.915	0.4185821	0	-0.084259315	0	0
cov1_2_3_4_7	27.275	-215.918	0.4202007	0	-0.065840241	0	-0.051436086
cov6_7_8_9_11	27.2751	-215.918	0.4171236	0	0	0	0
cov2_4_5_10_11	27.2909	-215.926	0.4169471	0	0	0	-0.04967868
cov2_3_4_5_6	27.2951	-215.928	0.4218613	0	0	0	-0.065478024
cov4_5_6_10_11	27.3045	-215.933	0.4174535	0	0	0	0
cov1_3_5_6_7	27.3193	-215.94	0.4200207	0	-0.068665841	0	0
cov1_3_5_6_8	27.3197	-215.94	0.4200242	0	-0.069303669	0	0
cov3_4_8_9_10	27.3225	-215.942	0.4193643	0	0	0	0
cov4_7_8_9_10	27.3225	-215.942	0.4193676	0	0	0	0
cov3_4_7_9_10	27.3258	-215.943	0.4193578	0	0	0	0
cov1_2_3_5_8	27.3272	-215.944	0.4209522	0	-0.085064067	0	-0.035105127
cov1_2_4_8_11	27.3309	-215.946	0.4199593	0	-0.068179333	0	-0.045034828
cov1_2_4_5_10	27.3312	-215.946	0.4162589	0	-0.024664823	0	-0.030594596
cov1_2_3_5_7	27.3325	-215.947	0.4209598	0	-0.085296147	0	-0.035132101
cov1_4_5_6_10	27.3353	-215.948	0.4166239	0	-0.024970773	0	0
cov1_2_4_7_11	27.3369	-215.949	0.4199554	0	-0.068131686	0	-0.045095643
cov3_4_5_6_9	27.346	-215.954	0.4188728	0	0	0	0
cov3_4_6_9_11	27.3519	-215.957	0.4192406	0	0	0	0
cov1_2_4_5_8	27.352	-215.957	0.4194446	0	-0.055300872	0	-0.047280843
cov3_5_6_9_11	27.3527	-215.957	0.4186263	0	0	0	0
cov1_4_5_10_11	27.3576	-215.959	0.4173135	0	-0.064854186	0	0
cov1_2_4_5_7	27.3577	-215.959	0.4194432	0	-0.055300124	0	-0.047350507
cov1_5_6_7_11	27.3583	-215.96	0.4197491	0	-0.05893535	0	0
cov1_2_4_7_8	27.3584	-215.96	0.4194763	0	-0.061564416	0	-0.043998644
cov1_5_6_8_11	27.3608	-215.961	0.4197446	0	-0.059625939	0	0
cov1_3_6_7_11	27.3659	-215.964	0.4205284	0	-0.051010097	0	0
cov1_3_6_8_11	27.3672	-215.964	0.4205391	0	-0.051680508	0	0

cov1_4_5_6_7	27.3707	-215.966	0.4197342	0	-0.05375441	0	0
cov1_4_5_6_8	27.3734	-215.967	0.4197297	0	-0.054414861	0	0
cov1_6_7_8_11	27.377	-215.969	0.420109	0	-0.050441891	0	0
cov1_4_6_7_11	27.3775	-215.969	0.4201192	0	-0.050186227	0	0
cov1_4_6_8_11	27.3798	-215.97	0.4201198	0	-0.050861216	0	0
cov2_3_8_9_11	27.3816	-215.971	0.4186955	0	0	0	-0.063169529
cov2_3_7_9_11	27.3848	-215.973	0.4186898	0	0	0	-0.06314378
cov1_2_5_8_11	27.3852	-215.973	0.4205628	0	-0.074523279	0	-0.034550597
cov1_5_6_7_8	27.3854	-215.973	0.4192975	0	-0.052769103	0	0
cov1_3_4_6_7	27.39	-215.976	0.4199233	0	-0.047040913	0	0
cov1_2_5_7_11	27.3915	-215.976	0.4205595	0	-0.074525647	0	-0.034574966
cov1_3_4_6_8	27.3917	-215.976	0.4199295	0	-0.047714312	0	0
cov1_3_6_7_8	27.392	-215.977	0.4200778	0	-0.045123383	0	0
cov1_2_3_8_11	27.3985	-215.98	0.4214175	0	-0.065449763	0	-0.034448435
cov1_2_3_5_11	27.399	-215.98	0.4221616	0	-0.10975859	0	-0.037246971
cov1_4_6_7_8	27.4039	-215.983	0.4196522	0	-0.044332735	0	0
cov1_2_3_7_11	27.4052	-215.983	0.4214196	0	-0.065422952	0	-0.034464834
cov1_3_5_6_11	27.4062	-215.984	0.4211955	0	-0.095693034	0	0
cov3_4_5_10_11	27.4112	-215.986	0.4164698	0	0	0	0
cov1_2_5_7_8	27.4113	-215.986	0.4200856	0	-0.06797045	0	-0.033690018
cov1_2_7_8_11	27.4119	-215.987	0.4209647	0	-0.064722776	0	-0.03418002
cov1_2_3_4_5	27.4125	-215.987	0.4211307	0	-0.094856734	0	-0.046871128
cov1_2_3_6_11	27.4132	-215.987	0.4234629	0	-0.033947739	0	-0.034374578
cov2_3_5_6_11	27.4207	-215.991	0.4234996	0	0	0	-0.05041183
cov1_2_3_7_8	27.4233	-215.992	0.4209499	0	-0.059239232	0	-0.033612513
cov1_3_4_5_6	27.4249	-215.993	0.4212636	0	-0.089734613	0	0
cov2_3_5_9_11	27.4301	-215.996	0.4195557	0	0	0	-0.052654314
cov1_2_3_4_11	27.4349	-215.998	0.4216109	0	-0.081110246	0	-0.058533873
cov2_3_4_8_9	27.4476	-216.004	0.4194673	0	0	0	-0.073156757
cov2_3_7_8_9	27.4484	-216.005	0.4195001	0	0	0	-0.071338563
cov2_3_4_7_9	27.4509	-216.006	0.4194635	0	0	0	-0.07317494
cov1_2_6_9_11	27.4752	-216.018	0.4189094	0	-0.034497897	0	-0.031837102
cov2_4_8_9_11	27.4849	-216.023	0.4180331	0	0	0	-0.058153548
cov2_4_7_9_11	27.4874	-216.024	0.4180259	0	0	0	-0.058156264
cov2_7_8_9_11	27.4915	-216.026	0.4178322	0	0	0	-0.062398116
cov2_5_6_9_11	27.5117	-216.036	0.419044	0	0	0	-0.036438413
cov2_4_7_8_9	27.5419	-216.051	0.418838	0	0	0	-0.064559411
cov3_4_8_10_11	27.5665	-216.064	0.4166947	0	0	0	0
cov3_4_7_10_11	27.5689	-216.065	0.4166856	0	0	0	0
cov2_3_4_9_11	27.5714	-216.066	0.419253	0	0	0	-0.074061632
cov2_4_6_9_11	27.5805	-216.071	0.4191739	0	0	0	-0.039239062
cov4_7_8_10_11	27.5821	-216.072	0.4165508	0	0	0	0
cov1_3_4_5_8	27.5932	-216.077	0.4202189	0	-0.11257071	0	0
cov1_3_4_5_7	27.5988	-216.08	0.4202279	0	-0.11284604	0	0
cov1_3_5_8_11	27.6065	-216.084	0.4199548	0	-0.11673117	0	0
cov1_3_5_7_11	27.6122	-216.087	0.4199626	0	-0.11702162	0	0
cov1_3_5_7_8	27.6193	-216.09	0.4196414	0	-0.11172243	0	0

cov3_5_8_9_11	27.622	-216.092	0.4158575	0	0	0	0
cov3_5_7_9_11	27.6246	-216.093	0.4158456	0	0	0	0
cov3_4_5_6_7	27.6279	-216.095	0.4204519	0	0	0	0
cov4_5_6_7_8	27.6287	-216.095	0.4205626	0	0	0	0
cov4_5_6_7_11	27.6288	-216.095	0.4205201	0	0	0	0
cov3_5_6_7_8	27.6339	-216.097	0.4201575	0	0	0	0
cov4_5_8_9_11	27.634	-216.098	0.4161203	0	0	0	0
cov4_6_7_8_11	27.634	-216.098	0.4205892	0	0	0	0
cov3_5_6_7_11	27.6341	-216.098	0.4201346	0	0	0	0
cov5_7_8_9_11	27.6341	-216.098	0.4160204	0	0	0	0
cov3_4_6_7_11	27.6344	-216.098	0.4205842	0	0	0	0
cov3_4_6_7_8	27.6346	-216.098	0.4206533	0	0	0	0
cov5_6_7_8_11	27.6347	-216.098	0.4202233	0	0	0	0
cov4_5_7_9_11	27.6372	-216.099	0.4161108	0	0	0	0
cov3_4_5_6_8	27.6377	-216.099	0.4204793	0	0	0	0
cov4_5_6_8_11	27.6382	-216.1	0.4205276	0	0	0	0
cov1_4_6_9_11	27.6409	-216.101	0.4184944	0	-0.048074178	0	0
cov2_4_5_8_11	27.6419	-216.101	0.4200033	0	0	0	-0.07968714
cov1_4_5_8_11	27.6429	-216.102	0.4198367	0	-0.10209528	0	0
cov2_4_5_7_8	27.643	-216.102	0.4201274	0	0	0	-0.080807117
cov2_3_4_5_8	27.6433	-216.102	0.420105	0	0	0	-0.080522416
cov3_5_6_8_11	27.6441	-216.103	0.420154	0	0	0	0
cov3_4_6_8_11	27.6442	-216.103	0.4206104	0	0	0	0
cov2_4_5_7_11	27.6474	-216.104	0.4199971	0	0	0	-0.07972827
cov2_3_4_5_7	27.6489	-216.105	0.4200936	0	0	0	-0.080517204
cov1_4_5_7_11	27.6495	-216.105	0.4198353	0	-0.10213214	0	0
cov1_2_5_6_9	27.6539	-216.108	0.4174648	0	-0.013341321	0	-0.029054363
cov1_2_5_9_11	27.6581	-216.11	0.4183799	0	-0.056291804	0	-0.032013522
cov1_4_5_7_8	27.6583	-216.11	0.4194803	0	-0.096497166	0	0
cov3_5_7_8_9	27.6584	-216.11	0.4162051	0	0	0	0
cov3_4_5_8_9	27.6585	-216.11	0.4163251	0	0	0	0
cov2_4_5_6_9	27.6598	-216.11	0.4173334	0	0	0	-0.034794919
cov3_4_5_7_9	27.6615	-216.111	0.4163073	0	0	0	0
cov1_2_4_6_9	27.6635	-216.112	0.4175398	0	-0.020609923	0	-0.027355072
cov3_6_7_8_11	27.6652	-216.113	0.4199982	0	0	0	0
cov1_3_4_6_11	27.666	-216.114	0.421443	0	-0.066558381	0	0
cov1_5_7_8_11	27.6688	-216.115	0.4192612	0	-0.10110039	0	0
cov1_2_4_9_11	27.6724	-216.117	0.4184381	0	-0.065148105	0	-0.030639498
cov1_3_4_5_11	27.6788	-216.12	0.421424	0	-0.13756916	0	0
cov1_3_4_8_11	27.68	-216.121	0.4199425	0	-0.093845732	0	0
cov1_3_7_8_11	27.6801	-216.121	0.4200121	0	-0.092981376	0	0
cov1_3_4_7_11	27.6869	-216.124	0.4199415	0	-0.093865849	0	0
cov4_5_7_8_9	27.688	-216.125	0.4166498	0	0	0	0
cov1_4_7_8_11	27.6894	-216.125	0.4197286	0	-0.091342014	0	0
cov1_3_4_7_8	27.6929	-216.127	0.419623	0	-0.08899671	0	0
cov1_5_6_9_11	27.699	-216.13	0.4176015	0	-0.049149213	0	0
cov3_8_9_10_11	27.7789	-216.17	0.4134553	0	0	0	0

cov3_7_9_10_11	27.78	-216.171	0.4134496	0	0	0	0
cov1_4_5_6_9	27.7895	-216.175	0.4174289	0	-0.037687461	0	0
cov1_2_4_5_9	27.8116	-216.186	0.4169842	0	-0.039644799	0	-0.031192579
cov1_2_5_6_11	27.8151	-216.188	0.4209256	0	-0.051190704	0	-0.033849562
cov1_2_4_6_11	27.8262	-216.194	0.420764	0	-0.040629857	0	-0.040485769
cov4_5_6_9_11	27.8268	-216.194	0.4182233	0	0	0	0
cov1_4_5_9_11	27.8301	-216.196	0.4181033	0	-0.081163618	0	0
cov2_4_5_9_11	27.8559	-216.209	0.4175083	0	0	0	-0.056783147
cov3_4_7_8_10	27.8936	-216.227	0.4183024	0	0	0	0
cov7_8_9_10_11	27.9386	-216.25	0.4125687	0	0	0	0
cov2_3_5_8_11	27.9511	-216.256	0.4211918	0	0	0	-0.056912593
cov2_3_5_7_11	27.9569	-216.259	0.4211793	0	0	0	-0.056873021
cov2_4_5_6_11	27.958	-216.26	0.4210429	0	0	0	-0.052842358
cov2_3_5_7_8	27.969	-216.265	0.4215981	0	0	0	-0.058993766
cov2_5_7_8_11	27.9736	-216.267	0.4217078	0	0	0	-0.060141119
cov1_4_5_6_11	27.9958	-216.278	0.4207444	0	-0.077997579	0	0
cov1_2_4_5_6	28.0368	-216.299	0.4194432	0	-0.03748922	0	-0.031026104
cov2_3_4_8_11	28.0893	-216.325	0.4207661	0	0	0	-0.082297879
cov2_3_4_7_11	28.0944	-216.328	0.4207575	0	0	0	-0.0822929
cov3_4_5_6_11	28.0981	-216.33	0.4222338	0	0	0	0
cov1_2_4_5_11	28.1047	-216.333	0.4204397	0	-0.089885123	0	-0.035801808
cov2_4_7_8_11	28.1065	-216.334	0.4204286	0	0	0	-0.078156921
cov2_3_7_8_11	28.1265	-216.344	0.4211138	0	0	0	-0.071828276
cov4_7_8_9_11	28.1769	-216.369	0.4169109	0	0	0	0
cov3_4_8_9_11	28.1772	-216.369	0.4169332	0	0	0	0
cov3_4_7_9_11	28.1798	-216.37	0.4169217	0	0	0	0
cov3_4_5_9_11	28.2042	-216.383	0.4168873	0	0	0	0
cov2_3_4_7_8	28.2093	-216.385	0.4219626	0	0	0	-0.091473261
cov2_3_4_5_11	28.2749	-216.418	0.421879	0	0	0	-0.097916584
cov3_7_8_10_11	28.4216	-216.491	0.4126225	0	0	0	0
cov3_4_7_8_9	28.5268	-216.544	0.4185246	0	0	0	0
cov3_4_5_8_11	28.854	-216.708	0.4186496	0	0	0	0
cov3_5_7_8_11	28.8573	-216.709	0.4183519	0	0	0	0
cov3_4_5_7_11	28.8574	-216.709	0.4186273	0	0	0	0
cov3_4_5_7_8	29.0102	-216.786	0.4195499	0	0	0	0
cov3_7_8_9_11	29.0506	-216.806	0.4128319	0	0	0	0
cov4_5_7_8_11	29.1351	-216.848	0.4199832	0	0	0	0
cov3_4_7_8_11	29.3837	-216.972	0.4194681	0	0	0	0
cov3_7_8_9_10	30.035	-217.298	0.4133568	0	0	0	0
cov2_3_6_7_9_10	32.5423	-215.029	0.4203721	0	0	0	-0.035999472
cov2_3_6_8_9_10	32.5438	-215.03	0.4203815	0	0	0	-0.036121667
cov2_3_4_6_9_10	32.5666	-215.041	0.4204046	0	0	0	-0.050433533
cov1_2_3_6_9_10	32.5759	-215.046	0.4204095	0	-0.025685703	0	-0.028059825
cov2_5_6_7_9_10	32.6244	-215.07	0.4189474	0	0	0	-0.026618831
cov2_5_6_8_9_10	32.6277	-215.072	0.4189451	0	0	0	-0.026744194
cov2_3_6_9_10_11	32.6313	-215.074	0.4211232	0	0	0	-0.038364115
cov2_3_5_6_9_10	32.6396	-215.078	0.4207187	0	0	0	-0.035577176

cov1_2_3_4_9_10	32.6668	-215.091	0.4195218	0	-0.061311609	0	-0.041652815
cov1_2_3_8_9_10	32.6668	-215.092	0.4196184	0	-0.0550852	0	-0.0289645
cov1_2_3_7_9_10	32.67	-215.093	0.4196207	0	-0.055077009	0	-0.028970532
cov1_3_6_8_9_10	32.6763	-215.096	0.4189011	0	-0.046454788	0	0
cov1_3_6_7_9_10	32.6764	-215.096	0.4188999	0	-0.046131752	0	0
cov1_2_6_7_9_10	32.7012	-215.109	0.4188168	0	-0.021031418	0	-0.027511035
cov1_2_6_8_9_10	32.7047	-215.11	0.4188068	0	-0.021712206	0	-0.027503324
cov2_4_5_8_9_10	32.7156	-215.116	0.4174908	0	0	0	-0.050883472
cov2_4_5_7_9_10	32.7184	-215.117	0.4174845	0	0	0	-0.050916146
cov1_2_3_9_10_11	32.7233	-215.12	0.4203867	0	-0.061735539	0	-0.029699121
cov1_2_3_5_9_10	32.7339	-215.125	0.420022	0	-0.064685733	0	-0.029483351
cov2_6_7_9_10_11	32.7403	-215.128	0.4193736	0	0	0	-0.035775234
cov1_3_6_9_10_11	32.7404	-215.128	0.4196597	0	-0.053801921	0	0
cov2_4_6_7_9_10	32.7453	-215.131	0.4191193	0	0	0	-0.034823819
cov2_6_7_8_9_10	32.7453	-215.131	0.4191271	0	0	0	-0.034646972
cov2_6_8_9_10_11	32.7467	-215.131	0.419372	0	0	0	-0.035997443
cov2_4_6_8_9_10	32.7518	-215.134	0.4191245	0	0	0	-0.034914279
cov1_3_5_6_9_10	32.7541	-215.135	0.4192867	0	-0.056372551	0	0
cov1_3_4_6_9_10	32.7638	-215.14	0.4192351	0	-0.048609973	0	0
cov3_5_6_7_9_10	32.7782	-215.147	0.4185429	0	0	0	0
cov3_5_6_8_9_10	32.7816	-215.149	0.4185463	0	0	0	0
cov1_5_6_7_9_10	32.7894	-215.153	0.4178974	0	-0.020751392	0	0
cov1_5_6_8_9_10	32.7925	-215.154	0.4178899	0	-0.021401175	0	0
cov1_2_5_8_9_10	32.8	-215.158	0.4184796	0	-0.030869808	0	-0.027076582
cov1_2_5_7_9_10	32.8038	-215.16	0.4184755	0	-0.030823783	0	-0.027098998
cov5_6_7_9_10_11	32.8221	-215.169	0.4180634	0	0	0	0
cov4_5_6_7_9_10	32.823	-215.17	0.4179405	0	0	0	0
cov5_6_7_8_9_10	32.8231	-215.17	0.4179736	0	0	0	0
cov5_6_8_9_10_11	32.8274	-215.172	0.4180555	0	0	0	0
cov4_5_6_8_9_10	32.8283	-215.172	0.4179317	0	0	0	0
cov1_4_6_7_9_10	32.8396	-215.178	0.4184173	0	-0.035595544	0	0
cov1_4_6_8_9_10	32.8424	-215.179	0.4184133	0	-0.036183362	0	0
cov2_3_4_5_9_10	32.8594	-215.188	0.418891	0	0	0	-0.066986959
cov1_3_4_8_9_10	32.8611	-215.189	0.4186418	0	-0.075873749	0	0
cov1_3_5_8_9_10	32.8616	-215.189	0.4183549	0	-0.072155543	0	0
cov1_3_8_9_10_11	32.8639	-215.19	0.4185988	0	-0.082183127	0	0
cov1_3_4_7_9_10	32.8645	-215.19	0.4186415	0	-0.075904269	0	0
cov1_3_5_7_9_10	32.8652	-215.191	0.4183579	0	-0.072321736	0	0
cov2_3_5_8_9_10	32.8653	-215.191	0.4192848	0	0	0	-0.040140353
cov1_3_7_8_9_10	32.8661	-215.191	0.4184411	0	-0.079670758	0	0
cov1_3_7_9_10_11	32.8672	-215.192	0.4186027	0	-0.08220154	0	0
cov2_3_5_7_9_10	32.869	-215.193	0.4192798	0	0	0	-0.040139413
cov1_6_7_9_10_11	32.87	-215.193	0.4180409	0	-0.047471651	0	0
cov1_6_8_9_10_11	32.873	-215.195	0.4180368	0	-0.048174598	0	0
cov1_2_8_9_10_11	32.8813	-215.199	0.4186667	0	-0.05805563	0	-0.028960214
cov1_6_7_8_9_10	32.8817	-215.199	0.4177204	0	-0.043508972	0	0
cov1_2_7_9_10_11	32.8852	-215.201	0.4186597	0	-0.057962088	0	-0.028971152

cov3_4_6_7_9_10	32.8876	-215.202	0.4195462	0	0	0	0
cov3_4_6_8_9_10	32.8915	-215.204	0.4195573	0	0	0	0
cov2_5_8_9_10_11	32.8923	-215.204	0.4186625	0	0	0	-0.036282883
cov1_2_7_8_9_10	32.8924	-215.204	0.4183505	0	-0.05386596	0	-0.028357717
cov1_2_4_8_9_10	32.8925	-215.204	0.4183475	0	-0.053779668	0	-0.028183771
cov2_5_7_8_9_10	32.893	-215.205	0.4187634	0	0	0	-0.036825397
cov2_5_7_9_10_11	32.8957	-215.206	0.418655	0	0	0	-0.036266894
cov1_2_4_7_9_10	32.8961	-215.206	0.4183426	0	-0.053739649	0	-0.02822795
cov1_3_5_9_10_11	32.929	-215.223	0.4190661	0	-0.090167852	0	0
cov1_3_4_9_10_11	32.9312	-215.224	0.4191659	0	-0.083715725	0	0
cov1_3_4_5_9_10	32.9331	-215.225	0.419106	0	-0.086666161	0	0
cov3_6_7_9_10_11	32.9526	-215.234	0.4185141	0	0	0	0
cov3_6_8_9_10_11	32.9572	-215.237	0.4185216	0	0	0	0
cov3_6_7_8_9_10	32.9671	-215.242	0.4188389	0	0	0	0
cov1_5_8_9_10_11	32.9712	-215.244	0.4175913	0	-0.054378106	0	0
cov1_4_5_8_9_10	32.9734	-215.245	0.4175562	0	-0.051994265	0	0
cov1_5_7_8_9_10	32.9744	-215.245	0.4174238	0	-0.051656625	0	0
cov1_5_7_9_10_11	32.9752	-215.246	0.4175838	0	-0.054295625	0	0
cov1_4_5_7_9_10	32.9774	-215.247	0.4175505	0	-0.051959781	0	0
cov4_6_7_9_10_11	32.984	-215.25	0.4188626	0	0	0	0
cov3_4_5_6_9_10	32.9843	-215.25	0.4192881	0	0	0	0
cov3_5_6_9_10_11	32.985	-215.251	0.4192047	0	0	0	0
cov4_6_7_8_9_10	32.9852	-215.251	0.4189875	0	0	0	0
cov4_6_8_9_10_11	32.9916	-215.254	0.4188665	0	0	0	0
cov1_4_8_9_10_11	33.0221	-215.269	0.4182374	0	-0.070590249	0	0
cov1_4_7_9_10_11	33.0263	-215.271	0.4182295	0	-0.070528603	0	0
cov1_4_7_8_9_10	33.0272	-215.272	0.418027	0	-0.067465672	0	0
cov3_4_6_9_10_11	33.0283	-215.272	0.4198114	0	0	0	0
cov1_7_8_9_10_11	33.0803	-215.298	0.4174086	0	-0.080956286	0	0
cov2_3_5_9_10_11	33.106	-215.311	0.419973	0	0	0	-0.046778729
cov6_7_8_9_10_11	33.1294	-215.323	0.4174861	0	0	0	0
cov2_3_8_9_10_11	33.2273	-215.372	0.4192645	0	0	0	-0.06175948
cov2_3_7_9_10_11	33.2302	-215.373	0.419262	0	0	0	-0.061745774
cov4_5_8_9_10_11	33.2686	-215.392	0.4165438	0	0	0	0
cov5_7_8_9_10_11	33.2689	-215.393	0.4166493	0	0	0	0
cov3_5_8_9_10_11	33.2694	-215.393	0.4166542	0	0	0	0
cov4_5_7_9_10_11	33.2716	-215.394	0.4165349	0	0	0	0
cov3_5_7_9_10_11	33.2724	-215.394	0.4166448	0	0	0	0
cov3_4_5_8_9_10	33.3151	-215.416	0.4169456	0	0	0	0
cov3_5_7_8_9_10	33.3154	-215.416	0.4170493	0	0	0	0
cov4_5_7_8_9_10	33.318	-215.417	0.4170545	0	0	0	0
cov3_4_5_7_9_10	33.3183	-215.417	0.4169351	0	0	0	0
cov2_3_4_6_7_10	33.3202	-215.418	0.4188049	0	0	0	-0.046286025
cov2_3_4_6_8_10	33.3215	-215.419	0.4188142	0	0	0	-0.046401138
cov2_5_6_9_10_11	33.3245	-215.42	0.419287	0	0	0	-0.03121261
cov1_2_3_6_7_10	33.326	-215.421	0.4187855	0	-0.017972703	0	-0.03024108
cov1_2_3_6_8_10	33.326	-215.421	0.4187925	0	-0.018279303	0	-0.030257001

cov2_3_5_6_7_10	33.3336	-215.425	0.4187323	0	0	0	-0.032027197
cov2_3_5_6_8_10	33.3346	-215.425	0.4187391	0	0	0	-0.032135901
cov2_3_7_8_9_10	33.3385	-215.427	0.4203146	0	0	0	-0.072308501
cov2_3_4_8_9_10	33.3386	-215.427	0.4203245	0	0	0	-0.071413847
cov2_3_4_7_9_10	33.3415	-215.429	0.4203235	0	0	0	-0.071446435
cov1_2_3_4_6_10	33.3437	-215.43	0.4187018	0	-0.024053274	0	-0.043186344
cov2_3_4_5_6_10	33.3458	-215.431	0.4184561	0	0	0	-0.051093842
cov2_3_6_7_10_11	33.3493	-215.433	0.4193636	0	0	0	-0.037797918
cov2_3_6_8_10_11	33.3507	-215.433	0.4193723	0	0	0	-0.03791589
cov1_2_6_9_10_11	33.3509	-215.434	0.41914	0	-0.038371476	0	-0.028345845
cov2_3_4_9_10_11	33.3551	-215.436	0.4197572	0	0	0	-0.069064988
cov2_4_5_6_7_10	33.3557	-215.436	0.4167782	0	0	0	-0.038215205
cov2_3_6_7_8_10	33.3582	-215.437	0.4190357	0	0	0	-0.036324557
cov2_4_5_6_8_10	33.3586	-215.437	0.4167767	0	0	0	-0.038359167
cov1_2_3_6_10_11	33.3777	-215.447	0.4198015	0	-0.027147163	0	-0.031327158
cov2_3_4_6_10_11	33.3828	-215.45	0.4195509	0	0	0	-0.051584359
cov2_5_6_7_10_11	33.406	-215.461	0.4182572	0	0	0	-0.029344177
cov2_4_8_9_10_11	33.4068	-215.462	0.4184782	0	0	0	-0.052287107
cov2_4_7_9_10_11	33.4089	-215.463	0.4184687	0	0	0	-0.052290177
cov2_5_6_8_10_11	33.4089	-215.463	0.4182571	0	0	0	-0.029464899
cov2_3_4_5_8_10	33.4122	-215.464	0.4170802	0	0	0	-0.056992935
cov1_2_3_5_6_10	33.4127	-215.464	0.4192257	0	-0.028615579	0	-0.030801311
cov2_3_4_5_7_10	33.4154	-215.466	0.4170746	0	0	0	-0.056995993
cov1_2_5_6_7_10	33.4302	-215.473	0.4177564	0	0.005183035	0	-0.028410133
cov2_5_6_7_8_10	33.4317	-215.474	0.4176955	0	0	0	-0.027375052
cov1_2_5_6_8_10	33.4336	-215.475	0.4177498	0	0.004528613	0	-0.028400601
cov2_7_8_9_10_11	33.4347	-215.475	0.4180794	0	0	0	-0.060784256
cov2_3_5_6_10_11	33.4397	-215.478	0.4198962	0	0	0	-0.037714417
cov2_4_5_6_9_10	33.4418	-215.479	0.4176523	0	0	0	-0.030478719
cov1_2_3_4_8_10	33.4437	-215.48	0.418129	0	-0.051648648	0	-0.040236044
cov1_4_6_9_10_11	33.4444	-215.48	0.4189891	0	-0.047831181	0	0
cov1_2_5_6_9_10	33.4446	-215.48	0.4178782	0	-0.009023373	0	-0.025131681
cov1_2_3_4_7_10	33.4464	-215.481	0.4181292	0	-0.051658297	0	-0.040282945
cov2_4_6_9_10_11	33.4654	-215.491	0.4196077	0	0	0	-0.03324181
cov1_2_3_8_10_11	33.4677	-215.492	0.4187889	0	-0.052675368	0	-0.031686957
cov1_2_3_7_10_11	33.4707	-215.493	0.4187919	0	-0.052679045	0	-0.031694361
cov1_5_6_9_10_11	33.4719	-215.494	0.4180244	0	-0.04026613	0	0
cov1_2_5_9_10_11	33.4734	-215.495	0.4186677	0	-0.049238132	0	-0.027836765
cov1_2_3_4_10_11	33.4754	-215.496	0.4188608	0	-0.06021973	0	-0.043485541
cov1_2_3_5_8_10	33.4777	-215.497	0.4183144	0	-0.041123599	0	-0.030585749
cov2_4_5_8_10_11	33.4799	-215.498	0.4166957	0	0	0	-0.051311358
cov1_2_3_5_7_10	33.481	-215.499	0.4183158	0	-0.041224504	0	-0.030600787
cov1_2_3_7_8_10	33.482	-215.499	0.4184096	0	-0.047822305	0	-0.031021555
cov2_4_5_7_10_11	33.4825	-215.499	0.4166862	0	0	0	-0.051314407
cov1_2_4_5_8_10	33.4881	-215.502	0.4164415	0	-0.005391574	0	-0.046226187
cov1_2_4_6_9_10	33.4894	-215.503	0.418126	0	-0.024960727	0	-0.021025225
cov1_2_6_7_10_11	33.4894	-215.503	0.4181647	0	-0.021455711	0	-0.03054214

cov2_4_5_7_8_10	33.4895	-215.503	0.4164178	0	0	0	-0.048794867
cov1_2_4_5_7_10	33.4905	-215.503	0.4164349	0	-0.005267511	0	-0.046319075
cov1_2_6_8_10_11	33.4924	-215.504	0.4181617	0	-0.022156544	0	-0.030542817
cov1_2_3_4_5_10	33.4929	-215.505	0.4181145	0	-0.043045282	0	-0.046279752
cov2_4_7_8_9_10	33.4965	-215.506	0.4194893	0	0	0	-0.060391503
cov1_2_6_7_8_10	33.5175	-215.517	0.4176264	0	-0.015950855	0	-0.029658863
cov1_2_4_6_7_10	33.5176	-215.517	0.4176017	0	-0.015951466	0	-0.030099929
cov1_2_4_6_8_10	33.5213	-215.519	0.4176008	0	-0.016617875	0	-0.029995799
cov1_2_4_9_10_11	33.5273	-215.522	0.4188596	0	-0.066865063	0	-0.023945363
cov1_3_6_8_10_11	33.5296	-215.523	0.4178908	0	-0.047292072	0	0
cov1_3_6_7_10_11	33.5297	-215.523	0.4178879	0	-0.046957692	0	0
cov2_4_6_7_10_11	33.5308	-215.524	0.418264	0	0	0	-0.03645971
cov2_6_7_8_10_11	33.5308	-215.524	0.4182495	0	0	0	-0.03675043
cov1_2_3_5_10_11	33.535	-215.526	0.4193065	0	-0.061082777	0	-0.032279112
cov1_3_5_6_7_10	33.5358	-215.526	0.4174336	0	-0.033547226	0	0
cov1_3_4_6_7_10	33.536	-215.526	0.4177818	0	-0.038512316	0	0
cov1_3_4_6_8_10	33.5361	-215.526	0.4177835	0	-0.038874314	0	0
cov1_3_5_6_8_10	33.5361	-215.526	0.4174377	0	-0.034092314	0	0
cov2_4_6_8_10_11	33.5366	-215.526	0.4182732	0	0	0	-0.03654598
cov2_4_6_7_8_10	33.5428	-215.529	0.4178826	0	0	0	-0.034706018
cov1_3_6_7_8_10	33.5441	-215.53	0.4175324	0	-0.042568095	0	0
cov1_4_5_6_9_10	33.5574	-215.537	0.4177203	0	-0.029699521	0	0
cov4_5_6_9_10_11	33.5629	-215.54	0.418456	0	0	0	0
cov1_2_5_8_10_11	33.5692	-215.543	0.4177377	0	-0.027665681	0	-0.029725995
cov1_2_5_7_10_11	33.5726	-215.544	0.4177306	0	-0.02757246	0	-0.029735942
cov1_2_5_7_8_10	33.5833	-215.55	0.4173603	0	-0.022524368	0	-0.029063151
cov1_2_4_5_9_10	33.5894	-215.553	0.4173113	0	-0.03279782	0	-0.028817357
cov3_5_6_7_10_11	33.6031	-215.56	0.4172929	0	0	0	0
cov2_3_5_8_10_11	33.6038	-215.56	0.4180963	0	0	0	-0.039465383
cov2_3_5_7_8_10	33.6039	-215.56	0.4181248	0	0	0	-0.039603952
cov3_4_5_6_7_10	33.604	-215.56	0.4172685	0	0	0	0
cov3_5_6_7_8_10	33.6043	-215.56	0.4172046	0	0	0	0
cov3_5_6_8_10_11	33.6064	-215.561	0.4172963	0	0	0	0
cov2_3_5_7_10_11	33.6071	-215.562	0.4180903	0	0	0	-0.039452091
cov3_4_5_6_8_10	33.6073	-215.562	0.4172761	0	0	0	0
cov1_3_5_6_10_11	33.6074	-215.562	0.4183932	0	-0.055556111	0	0
cov1_5_6_7_10_11	33.6083	-215.562	0.4169818	0	-0.020499004	0	0
cov1_3_4_6_10_11	33.6085	-215.562	0.4185248	0	-0.049325908	0	0
cov2_4_5_9_10_11	33.6086	-215.562	0.4178175	0	0	0	-0.050526875
cov1_5_6_8_10_11	33.6111	-215.564	0.4169785	0	-0.021177895	0	0
cov1_4_5_9_10_11	33.6148	-215.566	0.4183027	0	-0.070538022	0	0
cov1_4_5_6_7_10	33.6209	-215.569	0.4167858	0	-0.016222142	0	0
cov1_5_6_7_8_10	33.6221	-215.569	0.4166292	0	-0.015864976	0	0
cov1_4_5_6_8_10	33.624	-215.57	0.4167817	0	-0.016871723	0	0
cov2_3_4_5_10_11	33.6272	-215.572	0.4179977	0	0	0	-0.066239108
cov1_3_4_5_6_10	33.6289	-215.573	0.418287	0	-0.05096481	0	0
cov2_5_7_8_10_11	33.6328	-215.575	0.4176646	0	0	0	-0.036585516

cov4_5_6_7_10_11	33.637	-215.577	0.4169707	0	0	0	0
cov5_6_7_8_10_11	33.6375	-215.577	0.4168839	0	0	0	0
cov4_5_6_7_8_10	33.6412	-215.579	0.4167821	0	0	0	0
cov4_5_6_8_10_11	33.6418	-215.579	0.4169688	0	0	0	0
cov1_4_6_7_10_11	33.6537	-215.585	0.4176483	0	-0.035809443	0	0
cov1_4_6_8_10_11	33.6561	-215.586	0.41765	0	-0.036422989	0	0
cov1_2_4_8_10_11	33.6687	-215.592	0.4176509	0	-0.051719114	0	-0.030547339
cov1_2_7_8_10_11	33.6688	-215.592	0.4176162	0	-0.051957338	0	-0.031160737
cov1_4_6_7_8_10	33.6709	-215.594	0.4172538	0	-0.031010278	0	0
cov1_2_4_7_10_11	33.6721	-215.594	0.4176427	0	-0.05163601	0	-0.030581829
cov1_2_4_7_8_10	33.6856	-215.601	0.4172509	0	-0.046391928	0	-0.02964858
cov1_3_4_8_10_11	33.6911	-215.604	0.4177035	0	-0.071899269	0	0
cov1_3_5_8_10_11	33.693	-215.605	0.4172402	0	-0.066131873	0	0
cov1_3_4_7_10_11	33.6944	-215.605	0.4177036	0	-0.071932042	0	0
cov1_3_4_5_8_10	33.695	-215.606	0.4172737	0	-0.063188875	0	0
cov1_3_5_7_10_11	33.6965	-215.606	0.4172419	0	-0.066254997	0	0
cov1_3_4_7_8_10	33.6977	-215.607	0.4174577	0	-0.068272271	0	0
cov3_4_6_7_10_11	33.6981	-215.607	0.418163	0	0	0	0
cov1_3_4_5_7_10	33.6985	-215.607	0.417276	0	-0.063305765	0	0
cov3_4_6_7_8_10	33.6988	-215.608	0.4182586	0	0	0	0
cov1_3_5_7_8_10	33.699	-215.608	0.4170145	0	-0.062572921	0	0
cov3_4_6_8_10_11	33.7019	-215.609	0.4181733	0	0	0	0
cov1_3_7_8_10_11	33.7052	-215.611	0.4173714	0	-0.077711392	0	0
cov1_6_7_8_10_11	33.7107	-215.613	0.4168197	0	-0.044894314	0	0
cov3_4_5_9_10_11	33.7216	-215.619	0.4173442	0	0	0	0
cov2_3_4_6_7_9	33.7244	-215.62	0.4192766	0	0	0	-0.050001016
cov2_3_4_6_8_9	33.7265	-215.621	0.4192886	0	0	0	-0.050151092
cov1_2_3_6_7_9	33.7584	-215.637	0.4193292	0	-0.017190365	0	-0.031328873
cov1_2_3_6_8_9	33.7589	-215.638	0.4193318	0	-0.017636357	0	-0.03132987
cov2_4_5_6_7_9	33.764	-215.64	0.4175997	0	0	0	-0.042973822
cov2_4_5_6_8_9	33.7678	-215.642	0.4175976	0	0	0	-0.043119057
cov1_3_4_5_10_11	33.768	-215.642	0.4182852	0	-0.08379353	0	0
cov1_4_5_8_10_11	33.7744	-215.645	0.4167015	0	-0.049266638	0	0
cov2_3_6_7_9_11	33.7748	-215.645	0.4199537	0	0	0	-0.038925295
cov2_3_6_8_9_11	33.7769	-215.647	0.4199641	0	0	0	-0.039075306
cov1_4_5_7_10_11	33.778	-215.647	0.4166923	0	-0.04917889	0	0
cov1_5_7_8_10_11	33.778	-215.647	0.4164513	0	-0.048509035	0	0
cov4_6_7_8_10_11	33.7811	-215.649	0.4177624	0	0	0	0
cov1_4_5_7_8_10	33.7812	-215.649	0.4164438	0	-0.045294911	0	0
cov3_6_7_8_10_11	33.783	-215.65	0.4172638	0	0	0	0
cov2_3_5_6_7_9	33.7834	-215.65	0.4194307	0	0	0	-0.03530516
cov2_3_5_6_8_9	33.7853	-215.651	0.4194381	0	0	0	-0.035436762
cov2_3_6_7_8_9	33.7879	-215.652	0.4195617	0	0	0	-0.037147972
cov1_2_3_4_6_9	33.7939	-215.655	0.4192394	0	-0.024986836	0	-0.047752159
cov3_4_5_6_10_11	33.8142	-215.665	0.4182469	0	0	0	0
cov1_4_7_8_10_11	33.8256	-215.671	0.4171976	0	-0.06490113	0	0
cov2_5_6_7_9_11	33.8256	-215.671	0.4191121	0	0	0	-0.033321814

cov2_3_4_6_9_11	33.8276	-215.672	0.4201899	0	0	0	-0.056739577
cov2_5_6_8_9_11	33.8294	-215.673	0.4191111	0	0	0	-0.033447736
cov2_3_4_5_6_9	33.8345	-215.675	0.419246	0	0	0	-0.055441213
cov1_2_6_7_9_11	33.8359	-215.676	0.4190226	0	-0.021849737	0	-0.0317459
cov1_2_6_8_9_11	33.8388	-215.678	0.4190192	0	-0.022556988	0	-0.031747163
cov1_2_5_6_7_9	33.8488	-215.683	0.418503	0	-0.005425892	0	-0.030246903
cov2_5_6_7_8_9	33.8506	-215.683	0.4185677	0	0	0	-0.031393096
cov1_2_5_6_8_9	33.8522	-215.684	0.4184956	0	-0.006092802	0	-0.030237741
cov1_2_4_6_7_9	33.8586	-215.687	0.4181946	0	-0.016942539	0	-0.035102528
cov1_2_3_5_6_9	33.8587	-215.687	0.4199664	0	-0.039710001	0	-0.032677549
cov1_2_3_6_9_11	33.8595	-215.688	0.42055	0	-0.028111395	0	-0.032669657
cov1_2_4_6_8_9	33.8625	-215.689	0.4181895	0	-0.017629194	0	-0.035009581
cov1_2_6_7_8_9	33.8694	-215.693	0.4184413	0	-0.015850864	0	-0.030791064
cov2_4_6_7_9_11	33.8714	-215.694	0.418913	0	0	0	-0.041932756
cov2_4_6_8_9_11	33.8777	-215.697	0.4189183	0	0	0	-0.042021128
cov2_6_7_8_9_11	33.8788	-215.698	0.4191014	0	0	0	-0.038068827
cov1_2_3_4_8_9	33.8815	-215.699	0.4185433	0	-0.054668276	0	-0.043942422
cov1_2_3_4_7_9	33.8851	-215.701	0.4185432	0	-0.054667877	0	-0.043988989
cov2_4_6_7_8_9	33.8869	-215.702	0.4184841	0	0	0	-0.03996829
cov1_2_3_8_9_11	33.9257	-215.721	0.4193117	0	-0.05517242	0	-0.032964691
cov2_3_4_8_10_11	33.9268	-215.722	0.4182386	0	0	0	-0.061710702
cov2_3_7_8_10_11	33.9271	-215.722	0.4182682	0	0	0	-0.060652332
cov2_3_4_7_10_11	33.9295	-215.723	0.4182352	0	0	0	-0.06172393
cov1_2_3_7_9_11	33.9297	-215.723	0.4193124	0	-0.055156454	0	-0.032973879
cov2_3_5_6_9_11	33.9301	-215.723	0.4207144	0	0	0	-0.041795692
cov1_2_3_5_8_9	33.9432	-215.73	0.4189359	0	-0.053645079	0	-0.032472424
cov3_4_8_9_10_11	33.9433	-215.73	0.4176004	0	0	0	0
cov2_3_4_5_8_9	33.9437	-215.73	0.4175709	0	0	0	-0.062580368
cov1_2_3_7_8_9	33.9445	-215.73	0.4188864	0	-0.049681662	0	-0.032220342
cov1_2_3_4_9_11	33.9449	-215.731	0.4194273	0	-0.065012234	0	-0.048159481
cov3_4_7_9_10_11	33.946	-215.731	0.4175917	0	0	0	0
cov1_2_3_5_7_9	33.947	-215.732	0.4189378	0	-0.053753702	0	-0.032489891
cov2_3_4_5_7_9	33.9473	-215.732	0.4175626	0	0	0	-0.062574617
cov1_2_4_5_8_9	33.9543	-215.735	0.4171945	0	-0.020163126	0	-0.046943116
cov1_2_4_5_7_9	33.9576	-215.737	0.4171872	0	-0.020044206	0	-0.047030171
cov4_7_8_9_10_11	33.963	-215.74	0.4174374	0	0	0	0
cov1_3_6_7_9_11	33.9736	-215.745	0.4184658	0	-0.047720423	0	0
cov1_3_6_8_9_11	33.9739	-215.745	0.4184703	0	-0.048163981	0	0
cov2_4_5_8_9_11	33.9791	-215.748	0.4172949	0	0	0	-0.058438257
cov2_4_5_7_9_11	33.9822	-215.749	0.4172848	0	0	0	-0.058441603
cov2_4_5_7_8_9	33.9843	-215.75	0.417087	0	0	0	-0.056573693
cov1_2_3_4_5_9	33.9878	-215.752	0.418858	0	-0.058288146	0	-0.046760173
cov1_3_4_6_7_9	33.99	-215.753	0.418207	0	-0.040180177	0	0
cov1_3_4_6_8_9	33.9905	-215.753	0.418209	0	-0.040646399	0	0
cov1_3_5_6_7_9	33.9925	-215.754	0.4180869	0	-0.044631859	0	0
cov1_3_6_7_8_9	33.9926	-215.754	0.4180912	0	-0.042712966	0	0
cov1_3_5_6_8_9	33.9929	-215.755	0.4180937	0	-0.045235586	0	0

cov2_3_4_7_8_10	33.9955	-215.756	0.4191222	0	0	0	-0.068289163
cov1_2_3_5_9_11	34.0071	-215.762	0.4200302	0	-0.075681267	0	-0.034369275
cov3_5_7_8_10_11	34.016	-215.766	0.4156391	0	0	0	0
cov4_5_7_8_10_11	34.0161	-215.766	0.4156558	0	0	0	0
cov3_4_5_8_10_11	34.0166	-215.766	0.4156538	0	0	0	0
cov1_2_5_8_9_11	34.0169	-215.767	0.4184675	0	-0.041884489	0	-0.031777521
cov1_2_3_4_6_7	34.0177	-215.767	0.420504	0	-0.024616378	0	-0.05096845
cov1_2_3_4_6_8	34.0189	-215.768	0.4205113	0	-0.025159209	0	-0.050999308
cov3_4_5_7_10_11	34.0192	-215.768	0.4156418	0	0	0	0
cov1_2_5_7_9_11	34.0212	-215.769	0.4184598	0	-0.041796854	0	-0.031789162
cov1_2_5_7_8_9	34.0361	-215.776	0.418036	0	-0.035994186	0	-0.031017983
cov1_2_4_8_9_11	34.0387	-215.777	0.4182259	0	-0.056125722	0	-0.035652168
cov3_4_5_7_8_10	34.042	-215.779	0.4159526	0	0	0	0
cov1_2_4_7_9_11	34.0428	-215.78	0.4182171	0	-0.056040654	0	-0.035687246
cov1_2_7_8_9_11	34.0446	-215.78	0.4184008	0	-0.054687533	0	-0.032455479
cov1_4_6_7_9_11	34.0472	-215.782	0.418231	0	-0.039804395	0	0
cov1_5_6_7_9_11	34.0474	-215.782	0.4177406	0	-0.033490763	0	0
cov1_4_6_8_9_11	34.0496	-215.783	0.418231	0	-0.040432179	0	0
cov1_5_6_8_9_11	34.0501	-215.783	0.4177395	0	-0.034183349	0	0
cov2_3_4_5_6_7	34.0586	-215.787	0.4205862	0	0	0	-0.05821075
cov1_2_4_7_8_9	34.0601	-215.788	0.4177827	0	-0.050157974	0	-0.034674856
cov2_3_4_6_7_11	34.0607	-215.788	0.4213428	0	0	0	-0.059427631
cov1_4_5_6_7_9	34.0615	-215.789	0.4176234	0	-0.028857455	0	0
cov2_3_4_5_6_8	34.0623	-215.789	0.4206047	0	0	0	-0.058446741
cov1_4_5_6_8_9	34.0645	-215.79	0.4176218	0	-0.029519075	0	0
cov2_3_4_6_8_11	34.0647	-215.79	0.4213623	0	0	0	-0.059647421
cov1_5_6_7_8_9	34.0665	-215.791	0.4173363	0	-0.028065468	0	0
cov1_4_6_7_8_9	34.0683	-215.792	0.4177988	0	-0.034506927	0	0
cov1_3_5_6_9_11	34.0721	-215.794	0.4191476	0	-0.068921921	0	0
cov1_2_4_6_7_11	34.0725	-215.794	0.4203523	0	-0.027938645	0	-0.044485184
cov1_6_7_8_9_11	34.0751	-215.796	0.4176589	0	-0.046257655	0	0
cov1_2_4_6_8_11	34.076	-215.796	0.4203548	0	-0.028685975	0	-0.044405772
cov2_3_4_6_7_8	34.0769	-215.797	0.4209188	0	0	0	-0.057558765
cov2_4_5_6_10_11	34.081	-215.799	0.4178027	0	0	0	-0.038163919
cov2_4_5_6_7_11	34.0815	-215.799	0.4203836	0	0	0	-0.055535677
cov1_2_5_6_10_11	34.0852	-215.801	0.4181644	0	-0.015806702	0	-0.029476099
cov2_4_5_6_8_11	34.0874	-215.802	0.4203849	0	0	0	-0.055703345
cov2_4_7_8_10_11	34.0883	-215.802	0.417536	0	0	0	-0.051424935
cov1_2_3_5_6_7	34.0916	-215.804	0.4213159	0	-0.044638681	0	-0.033871992
cov1_2_3_5_6_8	34.0919	-215.804	0.4213227	0	-0.045214258	0	-0.033883909
cov1_3_4_5_6_9	34.0945	-215.805	0.4191214	0	-0.063846308	0	0
cov1_2_4_5_6_7	34.1061	-215.811	0.4196942	0	-0.012163976	0	-0.047534081
cov1_2_4_5_6_8	34.1107	-215.813	0.4196831	0	-0.013149365	0	-0.047328987
cov3_4_5_6_7_9	34.1113	-215.814	0.4179306	0	0	0	0
cov3_5_6_7_9_11	34.1122	-215.814	0.4178402	0	0	0	0
cov1_3_4_6_9_11	34.1128	-215.815	0.419092	0	-0.053359112	0	0
cov3_5_6_7_8_9	34.1131	-215.815	0.417766	0	0	0	0

cov2_4_5_6_7_8	34.1148	-215.816	0.419814	0	0	0	-0.052192683
cov3_4_5_6_8_9	34.1162	-215.816	0.417941	0	0	0	0
cov1_2_4_6_7_8	34.1171	-215.817	0.4197391	0	-0.020975906	0	-0.043173299
cov3_5_6_8_9_11	34.1172	-215.817	0.417844	0	0	0	0
cov4_5_6_7_9_11	34.1235	-215.82	0.4177624	0	0	0	0
cov5_6_7_8_9_11	34.1256	-215.821	0.4175832	0	0	0	0
cov4_5_6_7_8_9	34.1259	-215.821	0.4176217	0	0	0	0
cov4_5_6_8_9_11	34.1296	-215.823	0.4177605	0	0	0	0
cov1_2_5_6_7_11	34.1368	-215.827	0.4210331	0	-0.03433578	0	-0.033502019
cov1_2_4_6_10_11	34.1374	-215.827	0.4183341	0	-0.032325925	0	-0.026199523
cov1_2_5_6_8_11	34.1393	-215.828	0.4210346	0	-0.035028016	0	-0.033495699
cov1_2_3_5_6_11	34.1408	-215.829	0.4226524	0	-0.069607354	0	-0.03619406
cov2_4_6_7_8_11	34.1421	-215.829	0.4206087	0	0	0	-0.051529142
cov1_2_3_6_7_11	34.1456	-215.831	0.4219207	0	-0.025445949	0	-0.033424162
cov1_2_3_6_8_11	34.1468	-215.832	0.4219311	0	-0.026092842	0	-0.033433204
cov1_2_3_4_6_11	34.1527	-215.834	0.4220311	0	-0.039861913	0	-0.058255011
cov2_3_5_8_9_11	34.1548	-215.835	0.4185661	0	0	0	-0.043863786
cov2_3_5_7_8_9	34.1554	-215.836	0.4186344	0	0	0	-0.044221589
cov2_3_5_7_9_11	34.1586	-215.837	0.4185558	0	0	0	-0.043845188
cov1_2_6_7_8_11	34.1608	-215.839	0.4214192	0	-0.024969036	0	-0.033141628
cov2_5_7_8_9_11	34.1629	-215.84	0.4183344	0	0	0	-0.042298016
cov3_4_6_7_9_11	34.1665	-215.841	0.4186237	0	0	0	0
cov3_4_6_7_8_9	34.1666	-215.841	0.4186852	0	0	0	0
cov1_2_3_4_5_6	34.1673	-215.842	0.4214266	0	-0.053221315	0	-0.047011379
cov3_4_6_8_9_11	34.1717	-215.844	0.4186351	0	0	0	0
cov1_3_4_8_9_11	34.1759	-215.846	0.4181044	0	-0.077017301	0	0
cov1_2_5_6_7_8	34.1789	-215.848	0.4204226	0	-0.027433043	0	-0.032435179
cov1_3_4_7_9_11	34.1801	-215.848	0.4181021	0	-0.077029143	0	0
cov1_3_4_5_8_9	34.1826	-215.849	0.4179887	0	-0.077672199	0	0
cov1_3_7_8_9_11	34.1832	-215.85	0.4178708	0	-0.081279475	0	0
cov1_3_5_8_9_11	34.1839	-215.85	0.4178598	0	-0.080993605	0	0
cov1_3_4_7_8_9	34.1854	-215.851	0.4178198	0	-0.072784374	0	0
cov1_2_3_6_7_8	34.1864	-215.851	0.4213093	0	-0.01888839	0	-0.032385105
cov1_3_4_5_7_9	34.1867	-215.851	0.4179938	0	-0.077803526	0	0
cov1_3_5_7_9_11	34.188	-215.852	0.4178613	0	-0.081135327	0	0
cov1_3_5_7_8_9	34.1928	-215.855	0.4175884	0	-0.076686702	0	0
cov2_3_5_6_7_11	34.1952	-215.856	0.4220587	0	0	0	-0.042916736
cov2_3_5_6_8_11	34.1996	-215.858	0.4220833	0	0	0	-0.043116961
cov2_3_6_7_8_11	34.2037	-215.86	0.4220254	0	0	0	-0.040799664
cov4_6_7_8_9_11	34.2042	-215.86	0.418348	0	0	0	0
cov2_3_5_6_7_8	34.2082	-215.862	0.4216968	0	0	0	-0.041674741
cov2_5_6_7_8_11	34.2163	-215.866	0.4215381	0	0	0	-0.040670447
cov1_2_4_5_10_11	34.2342	-215.875	0.4174175	0	-0.03772838	0	-0.034644755
cov3_6_7_8_9_11	34.235	-215.876	0.4178333	0	0	0	0
cov2_3_4_5_9_11	34.2392	-215.878	0.418561	0	0	0	-0.073006166
cov1_4_5_6_10_11	34.2461	-215.881	0.4177529	0	-0.03825079	0	0
cov1_4_5_8_9_11	34.2462	-215.881	0.4175261	0	-0.065687318	0	0

cov1_2_4_5_6_10	34.2479	-215.882	0.4165539	0	0.000769789	0	-0.030458871
cov1_4_5_7_9_11	34.2507	-215.883	0.417517	0	-0.065610724	0	0
cov1_4_7_8_9_11	34.2537	-215.885	0.4177122	0	-0.071623086	0	0
cov1_5_7_8_9_11	34.256	-215.886	0.4171277	0	-0.064554689	0	0
cov1_4_5_7_8_9	34.2566	-215.886	0.4172172	0	-0.060896126	0	0
cov1_3_4_5_9_11	34.2622	-215.889	0.4191001	0	-0.1002973	0	0
cov1_2_3_4_8_11	34.2861	-215.901	0.4206942	0	-0.072507248	0	-0.052443542
cov1_2_3_4_7_11	34.2919	-215.904	0.4206971	0	-0.07253331	0	-0.05251868
cov2_3_4_5_6_11	34.2966	-215.906	0.4224193	0	0	0	-0.068568925
cov1_2_3_4_5_8	34.3095	-215.913	0.4203143	0	-0.072377772	0	-0.049097189
cov1_2_3_4_7_8	34.3138	-215.915	0.4202038	0	-0.065860981	0	-0.051386775
cov1_2_3_4_5_7	34.315	-215.916	0.42032	0	-0.07257211	0	-0.04910947
cov1_3_5_6_7_11	34.3365	-215.926	0.4204746	0	-0.075028471	0	0
cov1_3_5_6_8_11	34.3366	-215.926	0.4204825	0	-0.075623917	0	0
cov1_2_3_5_8_11	34.3457	-215.931	0.4214387	0	-0.091675295	0	-0.035976156
cov1_3_4_5_6_7	34.3469	-215.932	0.4204995	0	-0.070169159	0	0
cov1_3_4_5_6_8	34.347	-215.932	0.4205065	0	-0.07076533	0	0
cov1_2_3_5_7_11	34.3509	-215.934	0.4214489	0	-0.091970648	0	-0.036006748
cov1_3_5_6_7_8	34.3637	-215.94	0.4200193	0	-0.068846603	0	0
cov3_4_7_8_9_10	34.3674	-215.942	0.4193629	0	0	0	0
cov1_2_4_5_8_11	34.3692	-215.943	0.419929	0	-0.061875499	0	-0.048355284
cov1_2_3_5_7_8	34.371	-215.944	0.4209614	0	-0.085292195	0	-0.035109741
cov1_2_4_5_7_11	34.3753	-215.946	0.4199298	0	-0.061842735	0	-0.048409628
cov1_2_4_7_8_11	34.3758	-215.946	0.4199592	0	-0.068177577	0	-0.045036007
cov1_4_5_6_7_11	34.3867	-215.951	0.4202195	0	-0.060302945	0	0
cov3_4_5_6_9_11	34.3871	-215.952	0.4190228	0	0	0	0
cov1_4_5_6_8_11	34.3888	-215.952	0.4202202	0	-0.060982414	0	0
cov1_2_4_5_7_8	34.3968	-215.957	0.4194447	0	-0.055285773	0	-0.047299285
cov1_2_3_4_5_11	34.4028	-215.96	0.4217283	0	-0.10153457	0	-0.048604881
cov1_5_6_7_8_11	34.4028	-215.959	0.4197495	0	-0.059100057	0	0
cov1_3_4_6_7_11	34.4086	-215.962	0.4203804	0	-0.052944159	0	0
cov1_3_4_6_8_11	34.4098	-215.963	0.4203895	0	-0.053620016	0	0
cov1_3_6_7_8_11	34.4102	-215.963	0.4205776	0	-0.051265496	0	0
cov1_4_5_6_7_8	34.4152	-215.966	0.4197328	0	-0.053920668	0	0
cov1_3_4_5_6_11	34.4154	-215.966	0.4218393	0	-0.096255855	0	0
cov1_4_6_7_8_11	34.4219	-215.969	0.4201188	0	-0.05036782	0	0
cov2_3_4_8_9_11	34.4226	-215.969	0.4186085	0	0	0	-0.06665895
cov2_3_4_7_9_11	34.4258	-215.971	0.4186025	0	0	0	-0.066657966
cov2_3_7_8_9_11	34.4265	-215.971	0.4186961	0	0	0	-0.063172247
cov1_2_5_7_8_11	34.4301	-215.973	0.420563	0	-0.07452821	0	-0.034552121
cov1_3_4_6_7_8	34.4344	-215.975	0.4199228	0	-0.047228412	0	0
cov1_2_3_7_8_11	34.4431	-215.98	0.4214242	0	-0.065495054	0	-0.034449141
cov2_3_4_7_8_9	34.4924	-216.004	0.4194693	0	0	0	-0.073170502
cov1_2_5_6_9_11	34.5101	-216.013	0.4189431	0	-0.02730667	0	-0.031441412
cov1_2_4_6_9_11	34.5199	-216.018	0.4189438	0	-0.034272083	0	-0.031232633
cov2_4_7_8_9_11	34.5292	-216.023	0.4180325	0	0	0	-0.058156472
cov2_4_5_6_9_11	34.5329	-216.025	0.4185837	0	0	0	-0.042818264

cov3_4_7_8_10_11	34.6112	-216.064	0.416691	0	0	0	0
cov1_3_4_5_8_11	34.6228	-216.07	0.4205758	0	-0.11812637	0	0
cov1_3_4_5_7_11	34.6282	-216.072	0.4205888	0	-0.11845998	0	0
cov1_3_4_5_7_8	34.6369	-216.077	0.4202307	0	-0.11282524	0	0
cov1_3_5_7_8_11	34.6501	-216.083	0.4199675	0	-0.11704485	0	0
cov3_5_7_8_9_11	34.6659	-216.091	0.4158425	0	0	0	0
cov3_4_5_8_9_11	34.666	-216.091	0.4159701	0	0	0	0
cov3_4_5_7_9_11	34.6687	-216.092	0.4159539	0	0	0	0
cov3_4_5_6_7_8	34.6725	-216.094	0.4204507	0	0	0	0
cov3_4_5_6_7_11	34.6727	-216.094	0.4204298	0	0	0	0
cov4_5_6_7_8_11	34.6734	-216.095	0.42052	0	0	0	0
cov4_5_7_8_9_11	34.6784	-216.097	0.4161173	0	0	0	0
cov3_5_6_7_8_11	34.6787	-216.097	0.4201359	0	0	0	0
cov3_4_6_7_8_11	34.6789	-216.098	0.4205839	0	0	0	0
cov3_4_5_6_8_11	34.6825	-216.099	0.4204597	0	0	0	0
cov1_4_5_6_9_11	34.6827	-216.099	0.4186169	0	-0.051664469	0	0
cov2_4_5_7_8_11	34.6864	-216.101	0.4199976	0	0	0	-0.079682624
cov2_3_4_5_8_11	34.6868	-216.101	0.4200011	0	0	0	-0.079662414
cov1_4_5_7_8_11	34.6877	-216.102	0.4198378	0	-0.10211133	0	0
cov2_3_4_5_7_8	34.6879	-216.102	0.4200971	0	0	0	-0.080477682
cov2_3_4_5_7_11	34.6923	-216.104	0.419989	0	0	0	-0.079637316
cov1_2_4_5_6_9	34.6975	-216.107	0.4173415	0	-0.011373328	0	-0.031007148
cov1_2_4_5_9_11	34.6989	-216.108	0.418191	0	-0.053254324	0	-0.035419057
cov3_4_5_7_8_9	34.7025	-216.109	0.4163131	0	0	0	0
cov1_3_4_7_8_11	34.7246	-216.12	0.4199477	0	-0.093908588	0	0
cov3_7_8_9_10_11	34.8232	-216.17	0.4134514	0	0	0	0
cov1_2_4_5_6_11	34.8583	-216.187	0.4208118	0	-0.049197786	0	-0.036097061
cov2_3_5_7_8_11	34.9956	-216.256	0.4211843	0	0	0	-0.056862626
cov2_3_4_7_8_11	35.1342	-216.325	0.420763	0	0	0	-0.082274371
cov3_4_7_8_9_11	35.2216	-216.369	0.4169277	0	0	0	0
cov3_4_5_7_8_11	35.8958	-216.706	0.4186328	0	0	0	0
cov1_2_3_4_6_9_10	39.5242	-214.998	0.4199209	0	-0.029766209	0	-0.042227912
cov1_2_3_6_8_9_10	39.5328	-215.002	0.4200571	0	-0.023676717	0	-0.028097893
cov1_2_3_6_7_9_10	39.5329	-215.002	0.420056	0	-0.023352586	0	-0.028097933
cov2_3_4_6_7_9_10	39.54	-215.006	0.4201278	0	0	0	-0.047109217
cov2_3_4_6_8_9_10	39.5414	-215.006	0.4201367	0	0	0	-0.047223774
cov2_3_4_5_6_9_10	39.5426	-215.007	0.4196963	0	0	0	-0.051866159
cov2_3_5_6_7_9_10	39.5606	-215.016	0.4200532	0	0	0	-0.031535374
cov2_3_5_6_8_9_10	39.5618	-215.017	0.4200601	0	0	0	-0.031645037
cov2_4_5_6_7_9_10	39.5769	-215.024	0.4179532	0	0	0	-0.038575029
cov2_4_5_6_8_9_10	39.5802	-215.026	0.4179507	0	0	0	-0.038718687
cov2_3_6_7_9_10_11	39.5842	-215.028	0.4205619	0	0	0	-0.036857989
cov1_2_3_6_9_10_11	39.5854	-215.028	0.4209642	0	-0.03153181	0	-0.029044301
cov2_3_6_8_9_10_11	39.5857	-215.029	0.4205696	0	0	0	-0.036974952
cov2_3_6_7_8_9_10	39.5872	-215.029	0.4203722	0	0	0	-0.035996449
cov1_2_3_5_6_9_10	39.6026	-215.037	0.4204932	0	-0.036011652	0	-0.02878687
cov2_3_4_6_9_10_11	39.6029	-215.037	0.4207119	0	0	0	-0.051641987

cov2_5_6_7_9_10_11	39.6538	-215.063	0.4193785	0	0	0	-0.028165778
cov2_5_6_8_9_10_11	39.6571	-215.064	0.419377	0	0	0	-0.028288516
cov1_2_3_4_8_9_10	39.6606	-215.066	0.4193174	0	-0.059556004	0	-0.039498682
cov1_2_3_4_7_9_10	39.6634	-215.067	0.4193196	0	-0.059574768	0	-0.039552201
cov2_5_6_7_8_9_10	39.669	-215.07	0.4189479	0	0	0	-0.026644847
cov1_2_5_6_7_9_10	39.6692	-215.07	0.4189324	0	-0.001287168	0	-0.026353713
cov2_3_5_6_9_10_11	39.6707	-215.071	0.4210712	0	0	0	-0.036720634
cov1_2_5_6_8_9_10	39.6724	-215.072	0.4189177	0	-0.001940938	0	-0.026343088
cov2_3_4_5_8_9_10	39.679	-215.075	0.4181806	0	0	0	-0.059264733
cov2_3_4_5_7_9_10	39.6826	-215.077	0.4181652	0	0	0	-0.059262943
cov1_2_3_4_9_10_11	39.6908	-215.081	0.4199375	0	-0.066799822	0	-0.042374761
cov1_2_3_4_5_9_10	39.7012	-215.086	0.4193057	0	-0.051917278	0	-0.044927708
cov1_2_3_8_9_10_11	39.7026	-215.087	0.4199178	0	-0.058872562	0	-0.029486545
cov1_2_3_7_9_10_11	39.7057	-215.089	0.4199222	0	-0.058884886	0	-0.02949448
cov1_2_3_5_8_9_10	39.7107	-215.091	0.4195749	0	-0.051979225	0	-0.028761576
cov1_2_3_7_8_9_10	39.7107	-215.091	0.4196257	0	-0.055121203	0	-0.028956987
cov1_3_6_8_9_10_11	39.7114	-215.091	0.419192	0	-0.050138648	0	0
cov1_3_6_7_9_10_11	39.7116	-215.091	0.4191899	0	-0.049822576	0	0
cov1_2_3_5_7_9_10	39.714	-215.093	0.4195872	0	-0.052147342	0	-0.028777069
cov1_3_5_6_7_9_10	39.719	-215.095	0.4188393	0	-0.041469027	0	0
cov1_3_5_6_8_9_10	39.719	-215.095	0.4188515	0	-0.041920291	0	0
cov1_3_4_6_8_9_10	39.7191	-215.095	0.4190255	0	-0.04446664	0	0
cov1_3_4_6_7_9_10	39.7192	-215.095	0.4190242	0	-0.044133337	0	0
cov1_3_6_7_8_9_10	39.721	-215.096	0.4189037	0	-0.046352859	0	0
cov1_2_6_7_9_10_11	39.7252	-215.098	0.4192762	0	-0.025831229	0	-0.028278503
cov1_2_6_8_9_10_11	39.7282	-215.1	0.4192722	0	-0.02653269	0	-0.028279862
cov1_2_4_6_7_9_10	39.7453	-215.108	0.4187519	0	-0.021345739	0	-0.028651627
cov1_2_6_7_8_9_10	39.7459	-215.109	0.4188159	0	-0.021156603	0	-0.027511006
cov1_2_4_5_8_9_10	39.7468	-215.109	0.4175635	0	-0.013496724	0	-0.044480888
cov1_2_4_6_8_9_10	39.7489	-215.11	0.4187463	0	-0.02200643	0	-0.028562518
cov1_2_4_5_7_9_10	39.7498	-215.111	0.4175543	0	-0.013376855	0	-0.044568834
cov2_4_5_8_9_10_11	39.7575	-215.114	0.4176389	0	0	0	-0.052227742
cov1_2_3_5_9_10_11	39.7591	-215.115	0.4204197	0	-0.069309311	0	-0.030192827
cov2_4_5_7_8_9_10	39.7598	-215.116	0.4174894	0	0	0	-0.050904285
cov2_4_5_7_9_10_11	39.7604	-215.116	0.4176327	0	0	0	-0.052227293
cov1_3_5_6_9_10_11	39.7774	-215.124	0.4196774	0	-0.060776846	0	0
cov2_4_6_7_9_10_11	39.7852	-215.128	0.4193647	0	0	0	-0.035959976
cov2_6_7_8_9_10_11	39.7852	-215.128	0.4193737	0	0	0	-0.035761287
cov1_3_4_6_9_10_11	39.7853	-215.128	0.4196677	0	-0.053688568	0	0
cov2_4_6_7_8_9_10	39.7902	-215.131	0.4191189	0	0	0	-0.034816921
cov2_4_6_8_9_10_11	39.7916	-215.131	0.4193696	0	0	0	-0.036046459
cov1_3_4_5_6_9_10	39.7948	-215.133	0.4195292	0	-0.056817653	0	0
cov3_4_5_6_7_9_10	39.8229	-215.147	0.4184857	0	0	0	0
cov3_5_6_7_8_9_10	39.8231	-215.147	0.4185431	0	0	0	0
cov3_5_6_7_9_10_11	39.8231	-215.147	0.4185353	0	0	0	0
cov1_5_6_7_9_10_11	39.8247	-215.148	0.4181863	0	-0.024686436	0	0
cov3_4_5_6_8_9_10	39.8263	-215.149	0.4184935	0	0	0	0

cov3_5_6_8_9_10_11	39.8265	-215.149	0.4185381	0	0	0	0
cov1_5_6_8_9_10_11	39.8275	-215.149	0.4181817	0	-0.025375106	0	0
cov1_5_6_7_8_9_10	39.834	-215.153	0.4178966	0	-0.020888174	0	0
cov1_4_5_6_7_9_10	39.8342	-215.153	0.4179481	0	-0.020919854	0	0
cov1_2_5_8_9_10_11	39.836	-215.154	0.4187765	0	-0.034890481	0	-0.027610734
cov1_4_5_6_8_9_10	39.8372	-215.154	0.4179457	0	-0.021579886	0	0
cov1_2_5_7_9_10_11	39.8399	-215.156	0.4187693	0	-0.034798872	0	-0.027621872
cov1_2_5_7_8_9_10	39.8448	-215.158	0.4184799	0	-0.03085699	0	-0.027081006
cov4_5_6_7_9_10_11	39.867	-215.169	0.4180301	0	0	0	0
cov5_6_7_8_9_10_11	39.867	-215.169	0.4180631	0	0	0	0
cov4_5_6_7_8_9_10	39.8679	-215.17	0.4179402	0	0	0	0
cov4_5_6_8_9_10_11	39.8722	-215.172	0.4180271	0	0	0	0
cov1_4_6_7_9_10_11	39.8723	-215.172	0.4187461	0	-0.039708357	0	0
cov1_4_6_8_9_10_11	39.8747	-215.173	0.418747	0	-0.040330564	0	0
cov1_4_6_7_8_9_10	39.8843	-215.178	0.418417	0	-0.035734651	0	0
cov1_3_4_8_9_10_11	39.9025	-215.187	0.4188164	0	-0.078432374	0	0
cov2_3_4_5_9_10_11	39.9033	-215.187	0.4189656	0	0	0	-0.067598702
cov1_3_5_8_9_10_11	39.9034	-215.187	0.4185172	0	-0.07474905	0	0
cov1_3_4_5_8_9_10	39.9047	-215.188	0.4185264	0	-0.07264342	0	0
cov1_3_4_7_8_9_10	39.905	-215.188	0.4186475	0	-0.075933844	0	0
cov1_3_5_7_8_9_10	39.9057	-215.189	0.4183636	0	-0.072376386	0	0
cov1_3_4_7_9_10_11	39.9059	-215.189	0.4188178	0	-0.078482389	0	0
cov2_3_5_8_9_10_11	39.906	-215.189	0.4191085	0	0	0	-0.039240213
cov1_3_5_7_9_10_11	39.9069	-215.189	0.4185216	0	-0.074944797	0	0
cov1_3_7_8_9_10_11	39.9076	-215.189	0.4186107	0	-0.082272735	0	0
cov1_3_4_5_7_9_10	39.9082	-215.19	0.4185314	0	-0.072822724	0	0
cov2_3_5_7_9_10_11	39.9097	-215.191	0.4191019	0	0	0	-0.039230029
cov2_3_5_7_8_9_10	39.9102	-215.191	0.4192846	0	0	0	-0.040140417
cov1_6_7_8_9_10_11	39.9147	-215.193	0.4180402	0	-0.04760141	0	0
cov1_2_7_8_9_10_11	39.9261	-215.199	0.4186658	0	-0.058035095	0	-0.028961759
cov1_2_4_8_9_10_11	39.9262	-215.199	0.4186683	0	-0.058041501	0	-0.028929045
cov3_4_6_7_9_10_11	39.9273	-215.199	0.4193193	0	0	0	0
cov1_2_4_7_9_10_11	39.9301	-215.201	0.4186601	0	-0.057958443	0	-0.028962778
cov3_4_6_8_9_10_11	39.9312	-215.201	0.4193292	0	0	0	0
cov3_4_6_7_8_9_10	39.9321	-215.202	0.4195445	0	0	0	0
cov2_5_7_8_9_10_11	39.9369	-215.204	0.418661	0	0	0	-0.036274212
cov1_2_4_7_8_9_10	39.9372	-215.204	0.4183478	0	-0.053773027	0	-0.028198778
cov1_3_4_5_9_10_11	39.9667	-215.219	0.4193866	0	-0.090556108	0	0
cov3_6_7_8_9_10_11	39.9966	-215.234	0.4185187	0	0	0	0
cov1_4_5_8_9_10_11	40.0147	-215.243	0.4177389	0	-0.054808181	0	0
cov1_5_7_8_9_10_11	40.016	-215.244	0.4175905	0	-0.054356964	0	0
cov1_4_5_7_8_9_10	40.0182	-215.245	0.4175556	0	-0.051984369	0	0
cov1_4_5_7_9_10_11	40.0188	-215.245	0.4177296	0	-0.054722367	0	0
cov4_6_7_8_9_10_11	40.0286	-215.25	0.4188625	0	0	0	0
cov3_4_5_6_9_10_11	40.0287	-215.25	0.4193438	0	0	0	0
cov1_4_7_8_9_10_11	40.067	-215.269	0.4182368	0	-0.070584196	0	0
cov2_3_4_5_6_7_10	40.2589	-215.365	0.4177866	0	0	0	-0.046969188

cov2_3_4_5_6_8_10	40.2595	-215.365	0.4177908	0	0	0	-0.047097508
cov2_3_4_8_9_10_11	40.2716	-215.371	0.4192302	0	0	0	-0.063139993
cov2_3_7_8_9_10_11	40.2721	-215.372	0.4192677	0	0	0	-0.061773224
cov2_3_4_7_9_10_11	40.2745	-215.373	0.4192273	0	0	0	-0.063158277
cov4_5_7_8_9_10_11	40.313	-215.392	0.4165408	0	0	0	0
cov3_4_5_8_9_10_11	40.3135	-215.392	0.4165486	0	0	0	0
cov3_5_7_8_9_10_11	40.3138	-215.393	0.4166502	0	0	0	0
cov3_4_5_7_9_10_11	40.3165	-215.394	0.416537	0	0	0	0
cov1_2_3_4_6_7_10	40.3207	-215.396	0.4184737	0	-0.021304419	0	-0.040681788
cov1_2_3_4_6_8_10	40.3207	-215.396	0.4184779	0	-0.021604088	0	-0.040686357
cov1_2_5_6_9_10_11	40.3407	-215.406	0.4192109	0	-0.021488474	0	-0.027314563
cov1_2_3_4_6_10_11	40.3422	-215.407	0.4193379	0	-0.0308083	0	-0.044269722
cov2_4_5_6_9_10_11	40.3438	-215.408	0.4188098	0	0	0	-0.037845275
cov1_2_3_6_8_10_11	40.3453	-215.408	0.4192986	0	-0.023603613	0	-0.031077584
cov1_2_3_6_7_10_11	40.3454	-215.408	0.419296	0	-0.023280434	0	-0.031074622
cov2_3_4_5_6_10_11	40.3506	-215.411	0.419002	0	0	0	-0.054082561
cov2_3_4_6_7_10_11	40.3565	-215.414	0.4191277	0	0	0	-0.047691744
cov2_3_4_6_8_10_11	40.3578	-215.415	0.4191362	0	0	0	-0.047803713
cov3_4_5_7_8_9_10	40.3595	-215.415	0.4169405	0	0	0	0
cov2_4_5_6_7_10_11	40.3599	-215.416	0.4173945	0	0	0	-0.041855622
cov2_3_5_6_7_10_11	40.3626	-215.417	0.4191311	0	0	0	-0.033380699
cov2_4_5_6_8_10_11	40.3627	-215.417	0.4173935	0	0	0	-0.041992396
cov2_3_5_6_8_10_11	40.3637	-215.418	0.4191384	0	0	0	-0.033483084
cov2_3_4_6_7_8_10	40.3651	-215.418	0.4188049	0	0	0	-0.046281364
cov1_2_3_5_6_7_10	40.3685	-215.42	0.4187206	0	-0.013540704	0	-0.029941317
cov1_2_3_5_6_8_10	40.3687	-215.42	0.4187244	0	-0.014037617	0	-0.029949977
cov1_2_4_5_6_7_10	40.3689	-215.42	0.4167798	0	0.024180514	0	-0.046526128
cov1_2_3_6_7_8_10	40.3705	-215.421	0.4187852	0	-0.018128649	0	-0.030241376
cov1_2_4_5_6_8_10	40.3739	-215.423	0.416776	0	0.023309398	0	-0.046359621
cov1_2_3_4_5_6_10	40.3783	-215.425	0.4184794	0	-0.014985102	0	-0.04641975
cov2_3_5_6_7_8_10	40.3784	-215.425	0.4187319	0	0	0	-0.032042462
cov2_3_4_7_8_9_10	40.3832	-215.427	0.4203287	0	0	0	-0.07144245
cov1_2_4_6_9_10_11	40.3873	-215.429	0.419355	0	-0.036985824	0	-0.024554931
cov2_3_6_7_8_10_11	40.3942	-215.433	0.4193637	0	0	0	-0.037797936
cov2_4_5_6_7_8_10	40.4001	-215.436	0.4167781	0	0	0	-0.038268597
cov1_2_3_5_6_10_11	40.4135	-215.442	0.4198387	0	-0.034259026	0	-0.031819522
cov1_2_3_4_5_8_10	40.422	-215.447	0.4173965	0	-0.023965677	0	-0.04825658
cov1_2_3_4_5_7_10	40.4252	-215.448	0.4173977	0	-0.023990055	0	-0.048276328
cov2_5_6_7_8_10_11	40.4506	-215.461	0.4182596	0	0	0	-0.029370021
cov1_2_5_6_7_10_11	40.4509	-215.461	0.4182558	0	-0.000498205	0	-0.029256705
cov2_4_7_8_9_10_11	40.451	-215.461	0.4184745	0	0	0	-0.05229095
cov2_3_4_5_8_10_11	40.4534	-215.462	0.4172305	0	0	0	-0.058254532
cov1_2_5_6_8_10_11	40.4537	-215.463	0.4182526	0	-0.001174892	0	-0.029256491
cov2_3_4_5_7_10_11	40.4566	-215.464	0.4172238	0	0	0	-0.058248903
cov2_3_4_5_7_8_10	40.4571	-215.464	0.4170797	0	0	0	-0.056989119
cov1_2_3_4_8_10_11	40.472	-215.472	0.4185286	0	-0.056871363	0	-0.041127756
cov1_2_3_4_7_10_11	40.4746	-215.473	0.4185319	0	-0.056908306	0	-0.04118238

cov1_2_5_6_7_8_10	40.4749	-215.473	0.4177456	0	0.005043704	0	-0.028406413
cov1_4_5_6_9_10_11	40.4819	-215.477	0.4187946	0	-0.042244126	0	0
cov1_2_4_5_6_9_10	40.4851	-215.478	0.4176567	0	-0.005419524	0	-0.028679683
cov1_2_3_4_7_8_10	40.4878	-215.48	0.4181339	0	-0.051692176	0	-0.040259346
cov1_2_3_4_5_10_11	40.5039	-215.488	0.4186222	0	-0.048802305	0	-0.047649982
cov1_2_3_5_8_10_11	40.5081	-215.49	0.4186977	0	-0.04627309	0	-0.031257723
cov1_2_4_5_9_10_11	40.5102	-215.491	0.4183913	0	-0.044876714	0	-0.032672518
cov1_2_3_5_7_10_11	40.5114	-215.491	0.4187014	0	-0.046415324	0	-0.031275248
cov1_2_3_7_8_10_11	40.5117	-215.492	0.4187991	0	-0.052750322	0	-0.031686189
cov1_2_4_5_8_10_11	40.5173	-215.494	0.4168314	0	-0.0106217	0	-0.047039049
cov1_2_4_5_7_10_11	40.5201	-215.496	0.4168201	0	-0.010443083	0	-0.047113528
cov1_2_3_5_7_8_10	40.5222	-215.497	0.4183209	0	-0.041279394	0	-0.030590107
cov2_4_5_7_8_10_11	40.5242	-215.498	0.4166923	0	0	0	-0.051315019
cov1_2_4_5_7_8_10	40.5322	-215.502	0.41644	0	-0.005319585	0	-0.046282452
cov1_2_4_6_7_10_11	40.5339	-215.503	0.4181242	0	-0.021685424	0	-0.031290989
cov1_2_6_7_8_10_11	40.5341	-215.503	0.4181641	0	-0.021577585	0	-0.030542382
cov1_2_4_6_8_10_11	40.5371	-215.504	0.4181205	0	-0.022323183	0	-0.031221991
cov1_2_4_6_7_8_10	40.5623	-215.517	0.4176019	0	-0.016065513	0	-0.030084707
cov1_3_4_6_7_10_11	40.5658	-215.519	0.4181483	0	-0.043022589	0	0
cov1_3_4_6_8_10_11	40.5658	-215.519	0.4181513	0	-0.043376274	0	0
cov1_3_5_6_7_10_11	40.5667	-215.519	0.4177862	0	-0.038328376	0	0
cov1_3_5_6_8_10_11	40.5668	-215.519	0.4177912	0	-0.038850566	0	0
cov1_3_6_7_8_10_11	40.5743	-215.523	0.4178939	0	-0.047103833	0	0
cov2_4_6_7_8_10_11	40.5757	-215.523	0.4182639	0	0	0	-0.036455436
cov1_3_4_5_6_7_10	40.5785	-215.525	0.4176251	0	-0.034256321	0	0
cov1_3_4_5_6_8_10	40.5788	-215.525	0.4176326	0	-0.034797008	0	0
cov1_3_5_6_7_8_10	40.5804	-215.526	0.4174331	0	-0.033700128	0	0
cov1_3_4_6_7_8_10	40.5806	-215.526	0.4177995	0	-0.038662398	0	0
cov1_2_5_7_8_10_11	40.6139	-215.543	0.4177365	0	-0.027633855	0	-0.029727509
cov1_3_4_5_6_10_11	40.643	-215.557	0.4187644	0	-0.056275388	0	0
cov3_4_5_6_7_10_11	40.6477	-215.56	0.4173578	0	0	0	0
cov3_5_6_7_8_10_11	40.648	-215.56	0.4172933	0	0	0	0
cov2_3_5_7_8_10_11	40.6487	-215.56	0.418096	0	0	0	-0.039459401
cov3_4_5_6_7_8_10	40.6489	-215.56	0.4172686	0	0	0	0
cov3_4_5_6_8_10_11	40.651	-215.561	0.4173651	0	0	0	0
cov1_4_5_6_7_10_11	40.6513	-215.561	0.4171573	0	-0.021134192	0	0
cov1_5_6_7_8_10_11	40.6529	-215.562	0.416981	0	-0.020632613	0	0
cov1_4_5_6_8_10_11	40.6541	-215.563	0.4171609	0	-0.021828987	0	0
cov1_4_5_6_7_8_10	40.6656	-215.568	0.4167827	0	-0.016348812	0	0
cov4_5_6_7_8_10_11	40.6819	-215.577	0.4169703	0	0	0	0
cov1_4_6_7_8_10_11	40.6983	-215.585	0.417651	0	-0.035957967	0	0
cov2_3_4_5_6_7_9	40.7034	-215.587	0.4184773	0	0	0	-0.050602961
cov2_3_4_5_6_8_9	40.7049	-215.588	0.4184833	0	0	0	-0.05075802
cov1_2_4_7_8_10_11	40.7135	-215.592	0.4176493	0	-0.051696567	0	-0.030558065
cov1_2_3_4_6_7_9	40.7244	-215.598	0.4189531	0	-0.021466666	0	-0.044397973
cov1_2_3_4_6_8_9	40.7247	-215.598	0.4189496	0	-0.021828913	0	-0.044415923
cov1_3_4_5_8_10_11	40.7331	-215.602	0.4175283	0	-0.067075138	0	0

cov1_3_4_7_8_10_11	40.7352	-215.603	0.4177131	0	-0.071993645	0	0
cov1_3_4_5_7_10_11	40.7365	-215.604	0.4175319	0	-0.067219016	0	0
cov1_3_5_7_8_10_11	40.7374	-215.604	0.4172492	0	-0.066339995	0	0
cov1_3_4_5_7_8_10	40.7395	-215.605	0.417289	0	-0.063330316	0	0
cov3_4_6_7_8_10_11	40.7428	-215.607	0.418163	0	0	0	0
cov2_3_4_6_7_9_11	40.7569	-215.614	0.4196598	0	0	0	-0.051676565
cov2_3_4_6_8_9_11	40.759	-215.615	0.4196702	0	0	0	-0.051817472
cov2_4_5_6_7_9_11	40.7684	-215.62	0.4182048	0	0	0	-0.046580701
cov2_3_4_6_7_8_9	40.7693	-215.62	0.4192767	0	0	0	-0.050000702
cov1_2_3_6_7_9_11	40.7716	-215.621	0.4198915	0	-0.02312767	0	-0.032252038
cov1_2_3_6_8_9_11	40.7718	-215.622	0.4198972	0	-0.023557828	0	-0.032255407
cov2_4_5_6_8_9_11	40.7721	-215.622	0.4181988	0	0	0	-0.046719004
cov1_2_3_4_6_9_11	40.7756	-215.623	0.4199741	0	-0.03284377	0	-0.048973152
cov1_2_3_5_6_7_9	40.799	-215.635	0.4194201	0	-0.023235831	0	-0.031715265
cov1_2_3_5_6_8_9	40.7993	-215.635	0.4194244	0	-0.023778985	0	-0.03172526
cov1_2_4_5_6_7_9	40.8007	-215.636	0.4175986	0	0.012319023	0	-0.047216333
cov1_2_3_6_7_8_9	40.803	-215.637	0.4193287	0	-0.017358907	0	-0.031329191
cov1_2_4_5_6_8_9	40.8056	-215.638	0.4175959	0	0.011426434	0	-0.047052165
cov2_4_5_6_7_8_9	40.8086	-215.64	0.4175995	0	0	0	-0.043017915
cov2_3_5_6_7_9_11	40.8114	-215.641	0.419833	0	0	0	-0.036688174
cov2_3_5_6_8_9_11	40.8134	-215.642	0.4198411	0	0	0	-0.036817629
cov1_4_5_7_8_10_11	40.8192	-215.645	0.4166996	0	-0.049238725	0	0
cov2_3_6_7_8_9_11	40.8197	-215.645	0.4199536	0	0	0	-0.038928932
cov2_3_5_6_7_8_9	40.8282	-215.65	0.4194298	0	0	0	-0.035316026
cov2_3_4_5_6_9_11	40.8351	-215.653	0.419809	0	0	0	-0.058552678
cov1_2_3_4_5_6_9	40.8381	-215.655	0.4192991	0	-0.027380482	0	-0.046884231
cov1_2_3_5_6_9_11	40.8473	-215.659	0.4206485	0	-0.045985403	0	-0.033803191
cov1_2_5_6_7_9_11	40.8619	-215.667	0.4190679	0	-0.011868039	0	-0.031199957
cov1_2_5_6_8_9_11	40.8647	-215.668	0.4190637	0	-0.012551699	0	-0.031199639
cov1_2_4_6_7_9_11	40.8681	-215.67	0.4187709	0	-0.023325655	0	-0.036398993
cov2_5_6_7_8_9_11	40.8704	-215.671	0.4191119	0	0	0	-0.033338364
cov1_2_4_6_8_9_11	40.8714	-215.671	0.418771	0	-0.024024474	0	-0.036323919
cov1_2_6_7_8_9_11	40.8805	-215.676	0.4190217	0	-0.021993105	0	-0.031745774
cov1_2_5_6_7_8_9	40.8934	-215.682	0.4185033	0	-0.00556408	0	-0.030246049
cov1_2_3_4_5_8_9	40.9018	-215.687	0.418096	0	-0.037780945	0	-0.048859428
cov1_2_4_6_7_8_9	40.9033	-215.687	0.4181942	0	-0.017064433	0	-0.035093693
cov1_2_3_4_8_9_11	40.905	-215.688	0.4189927	0	-0.060583567	0	-0.044935189
cov1_2_3_4_5_7_9	40.9057	-215.689	0.4180991	0	-0.037830087	0	-0.048874478
cov1_2_3_4_7_9_11	40.9086	-215.69	0.4189939	0	-0.06060303	0	-0.04498937
cov2_4_6_7_8_9_11	40.9163	-215.694	0.4189129	0	0	0	-0.04192829
cov1_2_3_4_7_8_9	40.9258	-215.699	0.4185483	0	-0.054706916	0	-0.043963256
cov1_2_3_5_8_9_11	40.9686	-215.72	0.419375	0	-0.059559939	0	-0.033243298
cov1_2_3_7_8_9_11	40.97	-215.721	0.4193203	0	-0.05523537	0	-0.032964372
cov2_3_4_7_8_10_11	40.9716	-215.721	0.4182411	0	0	0	-0.061727995
cov1_2_3_5_7_9_11	40.9724	-215.722	0.4193809	0	-0.059721062	0	-0.033263747
cov1_2_4_5_8_9_11	40.9784	-215.725	0.4176361	0	-0.026113363	0	-0.047875437
cov1_2_4_5_7_9_11	40.9822	-215.727	0.4176247	0	-0.025950324	0	-0.047943418

cov2_3_4_5_8_9_11	40.9863	-215.729	0.4176844	0	0	0	-0.063551401
cov1_2_3_5_7_8_9	40.9876	-215.729	0.4189441	0	-0.053814957	0	-0.03247853
cov3_4_7_8_9_10_11	40.988	-215.73	0.4175973	0	0	0	0
cov2_3_4_5_7_8_9	40.9885	-215.73	0.4175689	0	0	0	-0.062570148
cov1_2_3_4_5_9_11	40.9898	-215.731	0.4194195	0	-0.064639522	0	-0.048297166
cov2_3_4_5_7_9_11	40.99	-215.731	0.4176752	0	0	0	-0.063534142
cov1_2_4_5_7_8_9	40.9987	-215.735	0.4171951	0	-0.020102157	0	-0.046988159
cov1_3_4_6_7_9_11	41.0153	-215.743	0.4186161	0	-0.045311427	0	0
cov1_3_4_6_8_9_11	41.0156	-215.743	0.418624	0	-0.045777675	0	0
cov1_2_3_4_6_7_11	41.0163	-215.744	0.4211417	0	-0.031856907	0	-0.052355898
cov1_2_3_4_6_8_11	41.0171	-215.744	0.4211486	0	-0.032379297	0	-0.052375214
cov1_3_5_6_7_9_11	41.0179	-215.745	0.4184962	0	-0.050218632	0	0
cov1_3_5_6_8_9_11	41.0181	-215.745	0.4185012	0	-0.050785497	0	0
cov1_3_6_7_8_9_11	41.0181	-215.745	0.4184656	0	-0.047893571	0	0
cov2_4_5_7_8_9_11	41.0234	-215.747	0.4172916	0	0	0	-0.058441905
cov1_3_4_5_6_7_9	41.0307	-215.751	0.4184158	0	-0.045836983	0	0
cov1_3_4_5_6_8_9	41.0309	-215.751	0.4184239	0	-0.046414895	0	0
cov1_3_4_6_7_8_9	41.0345	-215.753	0.418207	0	-0.040345504	0	0
cov1_3_5_6_7_8_9	41.037	-215.754	0.4180866	0	-0.044809059	0	0
cov1_2_3_4_5_6_7	41.0602	-215.766	0.420588	0	-0.02950993	0	-0.049326816
cov3_4_5_7_8_10_11	41.0609	-215.766	0.4156475	0	0	0	0
cov1_2_3_4_5_6_8	41.0611	-215.766	0.4205995	0	-0.03028113	0	-0.049262044
cov1_2_5_7_8_9_11	41.0618	-215.767	0.4184667	0	-0.041865774	0	-0.031778483
cov1_2_3_4_6_7_8	41.0623	-215.767	0.4205012	0	-0.024812575	0	-0.050954636
cov2_3_4_5_6_7_11	41.0774	-215.774	0.4210487	0	0	0	-0.060812837
cov2_3_4_5_6_8_11	41.0812	-215.776	0.4210656	0	0	0	-0.061045749
cov1_2_4_7_8_9_11	41.0835	-215.777	0.4182247	0	-0.056108858	0	-0.03566017
cov1_4_5_6_7_9_11	41.0861	-215.779	0.4180595	0	-0.034619783	0	0
cov1_4_5_6_8_9_11	41.0886	-215.78	0.4180627	0	-0.035311807	0	0
cov1_4_6_7_8_9_11	41.0918	-215.782	0.418231	0	-0.039957234	0	0
cov1_5_6_7_8_9_11	41.092	-215.782	0.4177419	0	-0.033642838	0	0
cov1_2_3_5_6_8_11	41.093	-215.782	0.4219487	0	-0.052390758	0	-0.034964605
cov1_2_3_5_6_7_11	41.0931	-215.782	0.4219436	0	-0.051874539	0	-0.034956022
cov1_3_4_5_6_9_11	41.0988	-215.785	0.4196606	0	-0.069850291	0	0
cov2_3_4_5_6_7_8	41.1035	-215.787	0.4205873	0	0	0	-0.058221503
cov2_3_4_6_7_8_11	41.1056	-215.788	0.4213426	0	0	0	-0.059420081
cov1_2_4_5_6_7_11	41.1059	-215.789	0.420307	0	-0.019164867	0	-0.048902003
cov1_4_5_6_7_8_9	41.1061	-215.789	0.417622	0	-0.029000816	0	0
cov1_2_4_5_6_8_11	41.1097	-215.791	0.4203108	0	-0.020057199	0	-0.048762608
cov1_2_4_6_7_8_11	41.1172	-215.794	0.4203517	0	-0.02807528	0	-0.044474901
cov1_2_4_5_6_10_11	41.1198	-215.796	0.4178573	0	-0.010651949	0	-0.034939851
cov2_4_5_6_7_8_11	41.1264	-215.799	0.4203833	0	0	0	-0.055548024
cov1_2_3_4_5_6_11	41.1325	-215.802	0.4221459	0	-0.059390079	0	-0.049119978
cov1_2_3_5_6_7_8	41.1361	-215.804	0.4213259	0	-0.044878096	0	-0.033866038
cov1_2_4_5_6_7_8	41.1508	-215.811	0.4196938	0	-0.012303793	0	-0.047522429
cov3_4_5_6_7_9_11	41.1553	-215.813	0.4180067	0	0	0	0
cov3_4_5_6_7_8_9	41.1562	-215.814	0.4179311	0	0	0	0

cov3_5_6_7_8_9_11	41.1571	-215.814	0.4178414	0	0	0	0
cov3_4_5_6_8_9_11	41.1602	-215.816	0.418015	0	0	0	0
cov4_5_6_7_8_9_11	41.1684	-215.82	0.4177625	0	0	0	0
cov1_2_5_6_7_8_11	41.1813	-215.826	0.4210321	0	-0.034499537	0	-0.033497233
cov1_2_3_6_7_8_11	41.19	-215.831	0.421926	0	-0.025662531	0	-0.033430252
cov2_3_5_7_8_9_11	41.1996	-215.835	0.4185627	0	0	0	-0.043853662
cov3_4_6_7_8_9_11	41.2111	-215.841	0.4186233	0	0	0	0
cov1_3_4_5_8_9_11	41.2172	-215.844	0.4182996	0	-0.082397609	0	0
cov1_3_4_7_8_9_11	41.2203	-215.846	0.4181113	0	-0.07709683	0	0
cov1_3_4_5_7_9_11	41.2213	-215.846	0.4183037	0	-0.082570297	0	0
cov1_3_4_5_7_8_9	41.227	-215.849	0.4180011	0	-0.077862695	0	0
cov1_3_5_7_8_9_11	41.2281	-215.85	0.4178703	0	-0.081232331	0	0
cov2_3_5_6_7_8_11	41.2401	-215.856	0.422056	0	0	0	-0.04290922
cov1_4_5_7_8_9_11	41.291	-215.881	0.4175253	0	-0.065676669	0	0
cov1_2_3_4_5_8_11	41.3264	-215.899	0.4208083	0	-0.07902291	0	-0.050182196
cov1_2_3_4_7_8_11	41.3303	-215.901	0.4207032	0	-0.072591611	0	-0.052474266
cov1_2_3_4_5_7_11	41.3319	-215.902	0.4208169	0	-0.079278631	0	-0.050188688
cov1_2_3_4_5_7_8	41.3538	-215.913	0.4203229	0	-0.072592942	0	-0.049060104
cov1_3_4_5_6_8_11	41.3624	-215.917	0.4210008	0	-0.077327594	0	0
cov1_3_4_5_6_7_11	41.3625	-215.917	0.4209937	0	-0.076780582	0	0
cov1_3_5_6_7_8_11	41.3809	-215.926	0.4204772	0	-0.075214522	0	0
cov1_2_3_5_7_8_11	41.3891	-215.93	0.4214535	0	-0.092005364	0	-0.035989662
cov1_3_4_5_6_7_8	41.3913	-215.931	0.4204995	0	-0.070360954	0	0
cov1_2_4_5_7_8_11	41.4141	-215.943	0.4199288	0	-0.061867705	0	-0.048359996
cov1_4_5_6_7_8_11	41.4311	-215.951	0.4202187	0	-0.06047614	0	0
cov1_3_4_6_7_8_11	41.4529	-215.962	0.4203801	0	-0.053137694	0	0
cov2_3_4_7_8_9_11	41.4675	-215.969	0.4186093	0	0	0	-0.066665032
cov1_2_4_5_6_9_11	41.5487	-216.01	0.4187036	0	-0.023282334	0	-0.035731478
cov1_3_4_5_7_8_11	41.6661	-216.069	0.4205937	0	-0.11848444	0	0
cov3_4_5_7_8_9_11	41.7099	-216.091	0.4159608	0	0	0	0
cov3_4_5_6_7_8_11	41.7174	-216.094	0.4204305	0	0	0	0
cov2_3_4_5_7_8_11	41.7313	-216.101	0.4199944	0	0	0	-0.079615635
cov2_3_4_5_6_7_9_10	46.4629	-214.945	0.4190421	0	0	0	-0.047850061
cov2_3_4_5_6_8_9_10	46.4637	-214.945	0.4190465	0	0	0	-0.047977732
cov1_2_3_4_6_8_9_10	46.5127	-214.97	0.4197183	0	-0.027462715	0	-0.03997005
cov1_2_3_4_6_7_9_10	46.5129	-214.97	0.4197175	0	-0.027196219	0	-0.039969946
cov1_2_3_4_6_9_10_11	46.534	-214.98	0.4204749	0	-0.035586557	0	-0.043191655
cov1_2_3_6_8_9_10_11	46.5595	-214.993	0.4204881	0	-0.028123159	0	-0.028813463
cov1_2_3_6_7_9_10_11	46.5597	-214.993	0.4204861	0	-0.027816531	0	-0.028811274
cov2_3_4_5_6_9_10_11	46.561	-214.994	0.4201329	0	0	0	-0.054294052
cov1_2_3_4_5_6_9_10	46.5613	-214.994	0.419729	0	-0.021836644	0	-0.045059428
cov1_2_3_6_7_8_9_10	46.5775	-215.002	0.4200552	0	-0.023520881	0	-0.028097888
cov1_2_3_5_6_8_9_10	46.5777	-215.002	0.4200465	0	-0.023013063	0	-0.028053473
cov1_2_3_5_6_7_9_10	46.5778	-215.002	0.4200447	0	-0.022595481	0	-0.028047162
cov2_3_4_6_7_9_10_11	46.5821	-215.004	0.4203095	0	0	0	-0.047902799
cov2_3_4_6_8_9_10_11	46.5836	-215.005	0.4203178	0	0	0	-0.048016323
cov2_3_4_6_7_8_9_10	46.5848	-215.006	0.4201274	0	0	0	-0.047098478

cov2_4_5_6_7_9_10_11	46.5936	-215.01	0.4184638	0	0	0	-0.041604145
cov2_4_5_6_8_9_10_11	46.5969	-215.012	0.4184619	0	0	0	-0.041742833
cov2_3_5_6_7_9_10_11	46.598	-215.012	0.4203282	0	0	0	-0.032469784
cov2_3_5_6_8_9_10_11	46.5992	-215.013	0.4203312	0	0	0	-0.032575093
cov1_2_4_5_6_7_9_10	46.6043	-215.015	0.4179509	0	0.018019916	0	-0.044761184
cov2_3_5_6_7_8_9_10	46.6054	-215.016	0.4200529	0	0	0	-0.031546914
cov1_2_4_5_6_8_9_10	46.6092	-215.018	0.4179494	0	0.017133658	0	-0.044597465
cov1_2_3_5_6_9_10_11	46.6149	-215.021	0.4210187	0	-0.040822344	0	-0.029675968
cov2_4_5_6_7_8_9_10	46.6214	-215.024	0.4179535	0	0	0	-0.038623969
cov2_3_6_7_8_9_10_11	46.6291	-215.028	0.420561	0	0	0	-0.036852119
cov1_2_3_4_5_8_9_10	46.6514	-215.039	0.4186587	0	-0.034547756	0	-0.046733837
cov1_2_3_4_5_7_9_10	46.6549	-215.041	0.4186602	0	-0.034647989	0	-0.046743469
cov1_2_3_4_8_9_10_11	46.695	-215.061	0.4196351	0	-0.063695237	0	-0.040212915
cov1_2_5_6_7_9_10_11	46.6964	-215.061	0.4193559	0	-0.006106913	0	-0.027088053
cov1_2_3_4_7_9_10_11	46.6976	-215.062	0.4196397	0	-0.063746556	0	-0.040275034
cov2_5_6_7_8_9_10_11	46.6985	-215.062	0.419377	0	0	0	-0.028182604
cov1_2_5_6_8_9_10_11	46.6992	-215.063	0.4193527	0	-0.00678817	0	-0.027084576
cov1_2_3_4_7_8_9_10	46.7042	-215.065	0.4193239	0	-0.059613302	0	-0.039527384
cov1_2_5_6_7_8_9_10	46.7138	-215.07	0.4189314	0	-0.001422485	0	-0.026353095
cov1_2_3_4_5_9_10_11	46.7228	-215.075	0.4197213	0	-0.056650456	0	-0.046055926
cov2_3_4_5_8_9_10_11	46.7238	-215.075	0.4182072	0	0	0	-0.059514968
cov2_3_4_5_7_8_9_10	46.7239	-215.075	0.4181778	0	0	0	-0.05926761
cov2_3_4_5_7_9_10_11	46.7274	-215.077	0.4182009	0	0	0	-0.059513551
cov1_2_3_7_8_9_10_11	46.7463	-215.086	0.4199298	0	-0.05896248	0	-0.029485123
cov1_2_3_5_8_9_10_11	46.7466	-215.087	0.4198751	0	-0.055997386	0	-0.029296195
cov1_2_3_5_7_9_10_11	46.7499	-215.088	0.4198824	0	-0.056206136	0	-0.029318564
cov1_3_4_6_8_9_10_11	46.7539	-215.09	0.4193234	0	-0.048107309	0	0
cov1_3_4_6_7_9_10_11	46.7541	-215.09	0.4193207	0	-0.047783969	0	0
cov1_3_5_6_8_9_10_11	46.7543	-215.09	0.4191389	0	-0.045810446	0	0
cov1_3_5_6_7_9_10_11	46.7544	-215.09	0.4191345	0	-0.045368372	0	0
cov1_2_3_5_7_8_9_10	46.7547	-215.091	0.4195848	0	-0.052204357	0	-0.028768299
cov1_3_6_7_8_9_10_11	46.7561	-215.091	0.4191895	0	-0.049994498	0	0
cov1_3_4_5_6_8_9_10	46.7633	-215.095	0.4189432	0	-0.042256596	0	0
cov1_3_4_5_6_7_9_10	46.7634	-215.095	0.4189377	0	-0.041792834	0	0
cov1_3_5_6_7_8_9_10	46.7635	-215.095	0.4188451	0	-0.041617331	0	0
cov1_3_4_6_7_8_9_10	46.7637	-215.095	0.4190241	0	-0.044297317	0	0
cov1_2_4_6_7_9_10_11	46.769	-215.098	0.4192013	0	-0.026266032	0	-0.029706793
cov1_2_6_7_8_9_10_11	46.7699	-215.098	0.4192755	0	-0.025961675	0	-0.028278734
cov1_2_4_6_8_9_10_11	46.7721	-215.099	0.4192009	0	-0.026950518	0	-0.029618319
cov1_2_4_5_8_9_10_11	46.7817	-215.104	0.4178707	0	-0.017646811	0	-0.045136151
cov1_2_4_5_7_9_10_11	46.785	-215.106	0.4178595	0	-0.017473562	0	-0.045207658
cov1_2_4_6_7_8_9_10	46.79	-215.108	0.4187516	0	-0.021464758	0	-0.028643512
cov1_2_4_5_7_8_9_10	46.7911	-215.109	0.4175589	0	-0.013434894	0	-0.044529256
cov2_4_5_7_8_9_10_11	46.8018	-215.114	0.4176354	0	0	0	-0.052228269
cov1_3_4_5_6_9_10_11	46.818	-215.122	0.4199308	0	-0.061260712	0	0
cov2_4_6_7_8_9_10_11	46.8301	-215.128	0.4193646	0	0	0	-0.035951864
cov3_4_5_6_7_8_9_10	46.8678	-215.147	0.4184859	0	0	0	0

cov3_4_5_6_7_9_10_11	46.8678	-215.147	0.4184762	0	0	0	0
cov3_5_6_7_8_9_10_11	46.868	-215.147	0.4185356	0	0	0	0
cov1_4_5_6_7_9_10_11	46.8693	-215.148	0.418254	0	-0.024940637	0	0
cov1_5_6_7_8_9_10_11	46.8693	-215.148	0.4181848	0	-0.024833318	0	0
cov3_4_5_6_8_9_10_11	46.8712	-215.149	0.4184842	0	0	0	0
cov1_4_5_6_8_9_10_11	46.8721	-215.149	0.4182582	0	-0.02564492	0	0
cov1_4_5_6_7_8_9_10	46.8788	-215.153	0.4179478	0	-0.02105945	0	0
cov1_2_5_7_8_9_10_11	46.8808	-215.154	0.4187757	0	-0.034866287	0	-0.027612069
cov4_5_6_7_8_9_10_11	46.9119	-215.169	0.41803	0	0	0	0
cov1_4_6_7_8_9_10_11	46.9169	-215.172	0.4187459	0	-0.039852286	0	0
cov1_3_4_5_8_9_10_11	46.9462	-215.186	0.4187037	0	-0.075344466	0	0
cov1_3_4_7_8_9_10_11	46.9462	-215.186	0.4188272	0	-0.078549952	0	0
cov1_3_5_7_8_9_10_11	46.9474	-215.187	0.4185298	0	-0.075039556	0	0
cov1_3_4_5_7_8_9_10	46.9487	-215.188	0.4185382	0	-0.072880564	0	0
cov1_3_4_5_7_9_10_11	46.9497	-215.188	0.4187116	0	-0.075571615	0	0
cov2_3_5_7_8_9_10_11	46.951	-215.189	0.4191082	0	0	0	-0.039238697
cov1_2_4_7_8_9_10_11	46.9711	-215.199	0.4186671	0	-0.05802438	0	-0.028937155
cov3_4_6_7_8_9_10_11	46.9719	-215.199	0.4193194	0	0	0	0
cov1_4_5_7_8_9_10_11	47.0596	-215.243	0.4177372	0	-0.054787099	0	0
cov2_3_4_5_6_7_10_11	47.2755	-215.351	0.4182694	0	0	0	-0.049664135
cov2_3_4_5_6_8_10_11	47.2762	-215.351	0.4182735	0	0	0	-0.049785884
cov1_2_3_4_5_6_7_10	47.3024	-215.364	0.4177491	0	0.005477804	0	-0.048493393
cov1_2_3_4_5_6_8_10	47.3034	-215.365	0.4177574	0	0.004841199	0	-0.04845146
cov2_3_4_5_6_7_8_10	47.3035	-215.365	0.4177855	0	0	0	-0.04699941
cov2_3_4_7_8_9_10_11	47.3164	-215.371	0.4192335	0	0	0	-0.063162825
cov1_2_3_4_6_8_10_11	47.3372	-215.382	0.4190068	0	-0.027279403	0	-0.041875089
cov1_2_3_4_6_7_10_11	47.3373	-215.382	0.4190044	0	-0.027012429	0	-0.041873409
cov3_4_5_7_8_9_10_11	47.3579	-215.392	0.4165431	0	0	0	0
cov1_2_3_4_6_7_8_10	47.3654	-215.396	0.4184732	0	-0.021434281	0	-0.040673281
cov1_2_3_4_5_6_10_11	47.3731	-215.4	0.4191087	0	-0.020485091	0	-0.048101392
cov1_2_4_5_6_9_10_11	47.3747	-215.401	0.4188978	0	-0.016176995	0	-0.032946947
cov1_2_4_5_6_7_10_11	47.3875	-215.407	0.4172901	0	0.018466674	0	-0.047579022
cov1_2_3_5_6_7_10_11	47.3885	-215.407	0.4192343	0	-0.01934397	0	-0.030803575
cov1_2_3_5_6_8_10_11	47.3885	-215.407	0.4192392	0	-0.019797271	0	-0.030813906
cov1_2_3_6_7_8_10_11	47.39	-215.408	0.4192983	0	-0.023454278	0	-0.031076738
cov1_2_4_5_6_8_10_11	47.3919	-215.409	0.4172944	0	0.017559141	0	-0.047436114
cov2_3_4_6_7_8_10_11	47.4013	-215.414	0.4191275	0	0	0	-0.047685457
cov2_4_5_6_7_8_10_11	47.4044	-215.415	0.4173911	0	0	0	-0.041913437
cov2_3_5_6_7_8_10_11	47.4074	-215.417	0.4191307	0	0	0	-0.033390091
cov1_2_3_5_6_7_8_10	47.4131	-215.42	0.4187044	0	-0.013705534	0	-0.029938555
cov1_2_4_5_6_7_8_10	47.4136	-215.42	0.4167782	0	0.024090357	0	-0.04651559
cov1_2_3_4_5_8_10_11	47.4511	-215.439	0.4177953	0	-0.029225732	0	-0.049075489
cov1_2_3_4_5_7_10_11	47.4544	-215.44	0.417795	0	-0.029287394	0	-0.049088255
cov1_2_3_4_5_7_8_10	47.4667	-215.447	0.4174038	0	-0.024067004	0	-0.048240727
cov1_2_5_6_7_8_10_11	47.4955	-215.461	0.4182548	0	-0.00063635	0	-0.029256211
cov2_3_4_5_7_8_10_11	47.4983	-215.462	0.4172301	0	0	0	-0.058251768
cov1_2_3_4_7_8_10_11	47.5157	-215.471	0.4185393	0	-0.056974045	0	-0.041163646

cov1_2_3_5_7_8_10_11	47.5524	-215.489	0.4187088	0	-0.046505174	0	-0.031268362
cov1_2_4_5_7_8_10_11	47.5617	-215.494	0.4168497	0	-0.010587093	0	-0.047056709
cov1_2_4_6_7_8_10_11	47.5787	-215.503	0.4181241	0	-0.021802041	0	-0.031282941
cov1_3_4_5_6_7_10_11	47.6089	-215.518	0.4180021	0	-0.039192613	0	0
cov1_3_4_5_6_8_10_11	47.609	-215.518	0.4180122	0	-0.039707976	0	0
cov1_3_4_6_7_8_10_11	47.6104	-215.518	0.4181477	0	-0.043183669	0	0
cov1_3_5_6_7_8_10_11	47.6112	-215.519	0.4177862	0	-0.038486284	0	0
cov1_3_4_5_6_7_8_10	47.6231	-215.525	0.4176237	0	-0.034485557	0	0
cov3_4_5_6_7_8_10_11	47.6926	-215.56	0.4173572	0	0	0	0
cov1_4_5_6_7_8_10_11	47.696	-215.561	0.4171572	0	-0.021273175	0	0
cov2_3_4_5_6_7_9_11	47.7184	-215.572	0.4189638	0	0	0	-0.053351191
cov2_3_4_5_6_8_9_11	47.7199	-215.573	0.41897	0	0	0	-0.053502156
cov1_2_3_4_6_7_9_11	47.7337	-215.58	0.4195419	0	-0.027871556	0	-0.045707187
cov1_2_3_4_6_8_9_11	47.7338	-215.58	0.4195447	0	-0.028229614	0	-0.045718767
cov1_2_3_4_5_6_7_9	47.7469	-215.587	0.4185177	0	-0.005484892	0	-0.049075923
cov1_2_3_4_5_6_8_9	47.748	-215.587	0.4185291	0	-0.006233248	0	-0.049016535
cov2_3_4_5_6_7_8_9	47.7482	-215.587	0.4184764	0	0	0	-0.050627382
cov1_2_3_4_6_7_8_9	47.769	-215.598	0.4189387	0	-0.021667896	0	-0.044352806
cov1_3_4_5_7_8_10_11	47.7774	-215.602	0.4175408	0	-0.067310412	0	0
cov2_3_4_6_7_8_9_11	47.8017	-215.614	0.4196597	0	0	0	-0.051670488
cov1_2_3_5_6_7_9_11	47.8113	-215.619	0.4199972	0	-0.029838776	0	-0.032674472
cov1_2_3_5_6_8_9_11	47.8113	-215.619	0.4200053	0	-0.030317337	0	-0.032698116
cov1_2_4_5_6_7_9_11	47.8116	-215.619	0.4181718	0	0.005871163	0	-0.048409834
cov2_4_5_6_7_8_9_11	47.8131	-215.62	0.4182041	0	0	0	-0.046614281
cov1_2_4_5_6_8_9_11	47.8158	-215.621	0.4181757	0	0.004965408	0	-0.048268025
cov1_2_3_6_7_8_9_11	47.8161	-215.621	0.4198908	0	-0.023309891	0	-0.032250666
cov1_2_3_4_5_6_9_11	47.8204	-215.623	0.4199868	0	-0.033414062	0	-0.048758879
cov1_2_3_5_6_7_8_9	47.8435	-215.635	0.4194195	0	-0.023398422	0	-0.031715357
cov1_2_4_5_6_7_8_9	47.8455	-215.636	0.4175966	0	0.012184016	0	-0.047186843
cov2_3_5_6_7_8_9_11	47.8563	-215.641	0.4198333	0	0	0	-0.036694868
cov1_2_5_6_7_8_9_11	47.9065	-215.666	0.4190671	0	-0.012017486	0	-0.031199377
cov1_2_4_6_7_8_9_11	47.9128	-215.67	0.4187703	0	-0.023450892	0	-0.036390561
cov1_2_3_4_5_8_9_11	47.9257	-215.676	0.4185478	0	-0.04378861	0	-0.049795168
cov1_2_3_4_5_7_9_11	47.9297	-215.678	0.4185482	0	-0.043877562	0	-0.049807481
cov1_2_3_4_5_7_8_9	47.9465	-215.686	0.418104	0	-0.037914893	0	-0.048834432
cov1_2_3_4_7_8_9_11	47.949	-215.688	0.4190024	0	-0.060676665	0	-0.044968208
cov1_2_3_5_7_8_9_11	48.0127	-215.72	0.419388	0	-0.05982267	0	-0.03325491
cov1_2_4_5_7_8_9_11	48.0231	-215.725	0.417633	0	-0.026043429	0	-0.047906117
cov2_3_4_5_7_8_9_11	48.0312	-215.729	0.4176823	0	0	0	-0.063537158
cov1_3_4_5_6_7_9_11	48.0552	-215.741	0.4188569	0	-0.051641864	0	0
cov1_3_4_5_6_8_9_11	48.0552	-215.741	0.4188654	0	-0.052181125	0	0
cov1_2_3_4_5_6_7_11	48.0588	-215.743	0.421225	0	-0.036696744	0	-0.050719385
cov1_2_3_4_5_6_8_11	48.0593	-215.743	0.4212357	0	-0.037398508	0	-0.050673045
cov1_3_4_6_7_8_9_11	48.0598	-215.743	0.4186196	0	-0.045487868	0	0
cov1_2_3_4_6_7_8_11	48.0609	-215.744	0.4211198	0	-0.032032586	0	-0.052341106
cov1_3_5_6_7_8_9_11	48.0624	-215.744	0.4184965	0	-0.050404937	0	0
cov1_3_4_5_6_7_8_9	48.0752	-215.751	0.418409	0	-0.046042177	0	0

cov1_2_3_4_5_6_7_8	48.1047	-215.766	0.4205848	0	-0.029684299	0	-0.049311558
cov2_3_4_5_6_7_8_11	48.1223	-215.774	0.4210483	0	0	0	-0.060819953
cov1_4_5_6_7_8_9_11	48.1307	-215.779	0.4180594	0	-0.034771897	0	0
cov1_2_3_5_6_7_8_11	48.1375	-215.782	0.421942	0	-0.052060724	0	-0.034955567
cov1_2_4_5_6_7_8_11	48.1505	-215.788	0.4203096	0	-0.019310025	0	-0.04889141
cov3_4_5_6_7_8_9_11	48.2002	-215.813	0.4180072	0	0	0	0
cov1_3_4_5_7_8_9_11	48.2614	-215.844	0.4183138	0	-0.082672137	0	0
cov1_2_3_4_5_7_8_11	48.3703	-215.898	0.4208226	0	-0.079337471	0	-0.050144028
cov1_3_4_5_6_7_8_11	48.4069	-215.917	0.4209937	0	-0.076979795	0	0
cov2_3_4_5_6_7_9_10_11	53.4904	-214.936	0.4194247	0	0	0	-0.049932707
cov2_3_4_5_6_8_9_10_11	53.4913	-214.936	0.4194217	0	0	0	-0.050066394
cov1_2_3_4_5_6_7_9_10	53.5074	-214.944	0.4190643	0	-0.003208795	0	-0.046969218
cov2_3_4_5_6_7_8_9_10	53.5077	-214.945	0.4190405	0	0	0	-0.047876036
cov1_2_3_4_5_6_8_9_10	53.5079	-214.945	0.4190751	0	-0.003856378	0	-0.046899722
cov1_2_3_4_6_8_9_10_11	53.5367	-214.959	0.420174	0	-0.032317259	0	-0.040995697
cov1_2_3_4_6_7_9_10_11	53.5369	-214.959	0.4201681	0	-0.03208414	0	-0.041006004
cov1_2_3_4_6_7_8_9_10	53.5575	-214.97	0.4196688	0	-0.027394823	0	-0.039963125
cov1_2_3_4_5_6_9_10_11	53.5683	-214.975	0.4202753	0	-0.026582068	0	-0.046533722
cov1_2_3_6_7_8_9_10_11	53.6042	-214.993	0.4204856	0	-0.027989674	0	-0.02881178
cov1_2_3_5_6_8_9_10_11	53.6044	-214.993	0.4204834	0	-0.027887476	0	-0.028806106
cov1_2_3_5_6_7_9_10_11	53.6046	-214.993	0.4204811	0	-0.027510353	0	-0.028790118
cov1_2_3_5_6_7_8_9_10	53.6223	-215.002	0.4200692	0	-0.022747962	0	-0.028030564
cov2_3_4_6_7_8_9_10_11	53.627	-215.004	0.4203092	0	0	0	-0.0478933
cov1_2_4_5_6_7_9_10_11	53.6298	-215.006	0.4183898	0	0.013045715	0	-0.045639665
cov1_2_4_5_6_8_9_10_11	53.6342	-215.008	0.4183897	0	0.01221878	0	-0.045531333
cov2_4_5_6_7_8_9_10_11	53.6382	-215.01	0.4184628	0	0	0	-0.041641926
cov2_3_5_6_7_8_9_10_11	53.6429	-215.012	0.4203245	0	0	0	-0.032478551
cov1_2_4_5_6_7_8_9_10	53.649	-215.015	0.4179509	0	0.017909706	0	-0.044753106
cov1_2_3_4_5_8_9_10_11	53.6863	-215.034	0.4189792	0	-0.038701867	0	-0.047395008
cov1_2_3_4_5_7_9_10_11	53.6898	-215.036	0.4189779	0	-0.038842051	0	-0.047396473
cov1_2_3_4_5_7_8_9_10	53.6959	-215.039	0.4186667	0	-0.034733901	0	-0.04670484
cov1_2_3_4_7_8_9_10_11	53.7382	-215.06	0.4196476	0	-0.063818159	0	-0.040254776
cov1_2_5_6_7_8_9_10_11	53.741	-215.061	0.4193557	0	-0.00624379	0	-0.027083295
cov2_3_4_5_7_8_9_10_11	53.7687	-215.075	0.4182075	0	0	0	-0.059516708
cov1_2_3_5_7_8_9_10_11	53.7904	-215.086	0.4198904	0	-0.056304831	0	-0.029310625
cov1_3_4_5_6_8_9_10_11	53.7984	-215.09	0.4192525	0	-0.04625171	0	0
cov1_3_4_5_6_7_9_10_11	53.7986	-215.09	0.4192457	0	-0.045807971	0	0
cov1_3_4_6_7_8_9_10_11	53.7987	-215.09	0.4193166	0	-0.047941558	0	0
cov1_3_5_6_7_8_9_10_11	53.7989	-215.09	0.4191347	0	-0.04553613	0	0
cov1_3_4_5_6_7_8_9_10	53.8079	-215.095	0.418938	0	-0.041999498	0	0
cov1_2_4_6_7_8_9_10_11	53.8137	-215.098	0.4192007	0	-0.026387422	0	-0.029684468
cov1_2_4_5_7_8_9_10_11	53.8262	-215.104	0.4178672	0	-0.017563999	0	-0.045172192
cov3_4_5_6_7_8_9_10_11	53.9127	-215.147	0.4184764	0	0	0	0
cov1_4_5_6_7_8_9_10_11	53.9139	-215.148	0.4182539	0	-0.025084976	0	0
cov1_3_4_5_7_8_9_10_11	53.9901	-215.186	0.4187208	0	-0.075671142	0	0
cov2_3_4_5_6_7_8_10_11	54.3202	-215.351	0.4182681	0	0	0	-0.049687891
cov1_2_3_4_5_6_7_10_11	54.3204	-215.351	0.4182738	0	-0.000378465	0	-0.049568509

cov1_2_3_4_5_6_8_10_11	54.321	-215.351	0.4182848	0	-0.001050083	0	-0.049519613
cov1_2_3_4_5_6_7_8_10	54.3471	-215.364	0.417747	0	0.00535231	0	-0.048483602
cov1_2_3_4_6_7_8_10_11	54.3819	-215.382	0.4190045	0	-0.02714948	0	-0.041865086
cov1_2_4_5_6_7_8_10_11	54.4323	-215.407	0.4172901	0	0.018357511	0	-0.047570448
cov1_2_3_5_6_7_8_10_11	54.4331	-215.407	0.4192337	0	-0.019499969	0	-0.030803391
cov1_2_3_4_5_7_8_10_11	54.4957	-215.439	0.4178036	0	-0.029397985	0	-0.04905652
cov1_3_4_5_6_7_8_10_11	54.6534	-215.517	0.4180176	0	-0.039472497	0	0
cov1_2_3_4_5_6_7_9_11	54.757	-215.569	0.4191089	0	-0.012088617	0	-0.050296702
cov1_2_3_4_5_6_8_9_11	54.7576	-215.57	0.419118	0	-0.012771202	0	-0.050253505
cov2_3_4_5_6_7_8_9_11	54.7632	-215.572	0.418963	0	0	0	-0.053369456
cov1_2_3_4_6_7_8_9_11	54.7783	-215.58	0.419542	0	-0.028015543	0	-0.045699108
cov1_2_3_4_5_6_7_8_9	54.7916	-215.587	0.4185184	0	-0.005624095	0	-0.049068744
cov1_2_3_5_6_7_8_9_11	54.8559	-215.619	0.4199995	0	-0.029994462	0	-0.032687232
cov1_2_4_5_6_7_8_9_11	54.8563	-215.619	0.4181718	0	0.005749187	0	-0.048400236
cov1_2_3_4_5_7_8_9_11	54.9703	-215.676	0.4185581	0	-0.044001743	0	-0.049771739
cov1_3_4_5_6_7_8_9_11	55.0996	-215.741	0.4188579	0	-0.051813389	0	0
cov1_2_3_4_5_6_7_8_11	55.1034	-215.742	0.421225	0	-0.036871256	0	-0.050710743
cov1_2_3_4_5_6_7_9_10_11	60.5324	-214.935	0.4195178	0	-0.008233434	0	-0.047882969
cov1_2_3_4_5_6_8_9_10_11	60.5327	-214.935	0.4195274	0	-0.008817478	0	-0.047841082
cov2_3_4_5_6_7_8_9_10_11	60.5352	-214.936	0.4194172	0	0	0	-0.049980092
cov1_2_3_4_5_6_7_8_9_10	60.552	-214.944	0.4190664	0	-0.003372458	0	-0.046942068
cov1_2_3_4_6_7_8_9_10_11	60.5815	-214.959	0.4201745	0	-0.032232398	0	-0.040974907
cov1_2_3_5_6_7_8_9_10_11	60.6491	-214.993	0.4204806	0	-0.027678719	0	-0.028790762
cov1_2_4_5_6_7_8_9_10_11	60.6745	-215.006	0.4183896	0	0.013015993	0	-0.0456547
cov1_2_3_4_5_7_8_9_10_11	60.7306	-215.034	0.4189852	0	-0.038961632	0	-0.047362332
cov1_3_4_5_6_7_8_9_10_11	60.8431	-215.09	0.4192471	0	-0.045982595	0	0
cov1_2_3_4_5_6_7_8_10_11	61.3651	-215.351	0.4182745	0	-0.000511833	0	-0.049559237
cov1_2_3_4_5_6_7_8_9_11	61.8016	-215.569	0.4191093	0	-0.012233087	0	-0.050285544
cov1_2_3_4_5_6_7_8_9_10_11	67.5771	-214.934	0.4195161	0	-0.008464262	0	-0.04786875

SE	bsnp_rs7817754	SE	bsnp_rs2047962	SE	bsnp_rs6469668	SE	bsnp_rs6469667	SE	bsnp_rs7832958
0	0	0	0	0	0	0	0	0	0
0	0	0	0	0	0	0	0	0	0
0	0	0	0	0	0	0	-0.090008998	0	0
0	0	0	0	0	0	0	0	0	0
0	0	0	0	0	-0.074746658	0	0	0	0
0	0	0	-0.069118853	0	0	0	0	0	0
0	0	0	0	0	0	0	0	0	0
0	0	0	0	0	0	0	0	0	0
0	0	0	0	0	0	0	0	0	0
0	0	0	0	0	0	0	0	0	0
0	0	0	0	0	0	0	0	0	0.071097194
0	0	0	0	0	0	0	0	0	0
0	0	0	0	0	0	0	-0.08823132	0	0.062838609
0	0	0	0	0	0	0	-0.088123195	0	0
0	0	0	0	0	0	0	0	0	0
0	0	0	0	0	0	0	0	0	0.059494528
0	0	0	0	0	0	0	-0.073892587	0	0
0	0	0	0	0	-0.066191905	0	0	0	0
0	0	0	0	0	0	0	0	0	0
0	0.017058073	0	0	0	0	0	0	0	0
0	0	0	0	0	0	0	0	0	0
0	0.03384585	0	0	0	0	0	0	0	0
0	0.034285546	0	0	0	0	0	-0.093526646	0	0
0	0	0	0	0	0	0	-0.058720941	0	0
0	0	0	0	0	0	0	-0.077458201	0	0
0	0	0	0	0	0	0	0	0	0
0	0	0	0	0	0	0	0	0	0.063395063
0	0	0	0	0	0	0	-0.044556229	0	0
0	0	0	0	0	0	0	0	0	0
0	0	0	0	0	0	0	0	0	0
0	0	0	0	0	0	0	0	0	0
0	0	0	-0.059911581	0	0	0	0	0	0
0	0	0	0	0	0.014609073	0	0	0	0
0	0	0	-0.015149302	0	0	0	-0.079620791	0	0
0	0	0	0	0	-0.066033481	0	0	0	0
0	0	0	-0.006144401	0	0	0	0	0	0
0	0	0	0	0	0	0	-0.096777989	0	0
0	0	0	0	0	-0.010196972	0	-0.081918171	0	0
0	0.033144344	0	0	0	0	0	0	0	0
0	0	0	0	0	-0.024496802	0	0	0	0
0	0	0	-0.060160966	0	0	0	0	0	0
0	0	0	0	0	0	0	0	0	0
0	0	0	-0.001045563	0	0	0	0	0	0
0	0	0	0	0	-0.073684689	0	0	0	0
0	0	0	0	0	-0.073660141	0	0	0	0.067120417

0	0	0	0	0	0	0	0	0	0
0	0	0	0	0	0	0	0	0	0
0	0	0	0	0	0	0	0	0	0.086894554
0	0	0	0	0	0	0	0	0	0.059898406
0	0	0	0	0	0	0	0	0	0
0	0	0	0	0	-0.061947235	0	0	0	0
0	0	0	0	0	0	0	0	0	0
0	0	0	-0.065256085	0	0	0	0	0	0
0	0	0	-0.065241966	0	0	0	0	0	0.056076182
0	0	0	-0.019029542	0	-0.05831997	0	0	0	0
0	0.041564166	0	0	0	0	0	0	0	0
0	0.007814836	0	0	0	-0.074220678	0	0	0	0
0	0	0	-0.051423364	0	0	0	0	0	0
0	0	0	0	0	0	0	0	0	0
0	0	0	0	0	0	0	0	0	0
0	0	0	0	0	0	0	0	0	0.061118608
0	0.002870123	0	-0.06879869	0	0	0	0	0	0
0	0.038443207	0	0	0	0	0	0	0	0
0	0.038084249	0	0	0	0	0	0	0	0
0	-0.032301246	0	0	0	0	0	0	0	0
0	-0.032710712	0	0	0	0	0	0	0	0.10049296
0	0.045146939	0	0	0	0	0	0	0	0
0	0	0	0	0	0	0	0	0	0
0	0	0	0	0	0	0	0	0	0
0	0	0	0	0	0	0	0	0	0.055392987
0	0.045160904	0	0	0	0	0	-0.075297367	0	0
0	0	0	0	0	0	0	-0.07345581	0	0.058388636
0	0	0	0	0	0	0	-0.073348772	0	0
0	0	0	0	0	0	0	0	0	0.011930236
0	0	0	0	0	0	0	-0.079269695	0	0
0	0	0	0	0	-0.065689945	0	0	0	0
0	0	0	0	0	-0.06565843	0	0	0	0.057887279
0	0.046015351	0	0	0	0	0	0	0	0
0	0	0	0	0	-0.070173361	0	0	0	0
0	0	0	0	0	0	0	0	0	0
0	0	0	0	0	0	0	0	0	0.057972066
0	0	0	0	0	0	0	-0.057661852	0	0.062125181
0	0	0	0	0	0	0	-0.057355327	0	0
0	0	0	0	0	0	0	0	0	0
0	0	0	0	0	0	0	0	0	0.05661169
0	0	0	0	0	0	0	-0.077057307	0	0.059721508
0	0	0	0	0	0	0	-0.07694605	0	0
0	0.042032966	0	0	0	0	0	0	0	0
0	0	0	0	0	0	0	-0.046195469	0	0
0	0	0	0	0	0	0	0	0	0
0	0.0421094	0	0	0	0	0	-0.078770971	0	0
0	0	0	0	0	0	0	-0.047513313	0	0.060767521

0	0	0	0	0	0	0	-0.046744795	0	0
0	0	0	0	0	-0.039939283	0	0	0	0
0	0	0	0	0	0	0	0	0	0
0	0	0	0	0	-0.03445074	0	-0.042841404	0	0
0	0	0	0	0	0	0	0	0	0
0	0	0	0	0	0	0	0	0	0.060188954
0	0.030084009	0	0	0	-0.063329183	0	0	0	0
0	0	0	-0.026130456	0	0	0	-0.053967586	0	0
0	0	0	0	0	0	0	-0.033586984	0	0
0	0	0	-0.020266775	0	0	0	0	0	0
0	0	0	0	0	0	0	0	0	0
0	0	0	0	0	-0.025740617	0	0	0	0
0	0	0	0	0	0	0	0	0	0
0	0	0	0	0	0	0	0	0	0.059212232
0	0.035830827	0	0	0	0	0	-0.060950285	0	0
0	0.043081026	0	0	0	0	0	0	0	0
0	0	0	-0.009444954	0	0	0	-0.081799809	0	0.061270204
0	0	0	0	0	-0.011219693	0	-0.079322087	0	0.063067415
0	0	0	-0.00969484	0	0	0	-0.081522739	0	0
0	0	0	0	0	-0.011537649	0	-0.078958772	0	0
0	0.004168796	0	0	0	0	0	-0.088767217	0	0.059043455
0	0	0	0	0	0	0	-0.085993298	0	0.063767703
0	0	0	0	0	0	0	-0.08827831	0	0.086195329
0	0.004734984	0	0	0	0	0	-0.088738629	0	0
0	0	0	0	0	0	0	-0.085851648	0	0
0	0	0	0	0	0.010141937	0	0	0	0
0	0	0	0	0	-0.065589503	0	0	0	0
0	0	0	0	0	0.010217964	0	0	0	0.058869183
0	0	0	0	0	0	0	0	0	0
0	0	0	0	0	-0.065555692	0	0	0	0.059376667
0	0.006435706	0	0	0	0	0	0	0	0
0	0	0	0	0	0	0	0	0	0.05726914
0	0.006431393	0	0	0	0	0	0	0	0.053602353
0	0	0	-0.001012441	0	0	0	0	0	0
0	0	0	0	0	0	0	0	0	-0.002646347
0	0	0	-0.000971445	0	0	0	0	0	0.05936546
0	0	0	0	0	0	0	-0.048885922	0	0
0	0	0	0	0	0	0	-0.079649249	0	0
0	0.046332619	0	0	0	0.043698699	0	0	0	0
0	0.034843935	0	0	0	0	0	-0.046978152	0	0
0	0	0	-0.011984857	0	-0.055901283	0	0	0	0
0	0.03485564	0	0	0	0	0	0	0	0
0	0	0	0	0	-0.067717156	0	0	0	0
0	0	0	0	0	0	0	0	0	0
0	0	0	-0.020836707	0	0	0	0	0	0
0	0	0	0	0	0	0	-0.036476693	0	0
0	0	0	-0.062485781	0	0	0	0	0	0

0	0	0	-0.056913012	0	0	0	0	0	0
0	0	0	-0.056884216	0	0	0	0	0	0.048164612
0	0	0	0	0	0	0	0	0	0
0	0	0	-0.023279521	0	0	0	-0.059635918	0	0
0	0	0	0	0	-0.027502154	0	-0.052623472	0	0
0	0	0	-0.016743913	0	0	0	0	0	0
0	0	0	0	0	0	0	0	0	0
0	0	0	0	0	-0.035482407	0	0	0	0
0	0	0	0	0	0	0	-0.068765745	0	0
0	0	0	0	0	-0.025187055	0	0	0	0
0	0	0	0	0	-0.025128303	0	0	0	0.063762375
0	0.031453121	0	0	0	0	0	0	0	0
0	0	0	0	0	-0.013239118	0	0	0	0
0	0.036878723	0	0.008785086	0	0	0	0	0	0
0	0	0	0	0	0	0	-0.043026622	0	0
0	0.032928212	0	-0.00400599	0	0	0	-0.090640572	0	0
0	0	0	0.007772254	0	0	0	-0.059772495	0	0
0	0	0	0	0	0	0	-0.048054613	0	0
0	0.03532923	0	0	0	0.003782508	0	-0.096635371	0	0
0	0	0	0	0	0	0	-0.083747301	0	0
0	0.033865213	0	0	0	0	0	-0.095155193	0	0
0	0	0	0	0	0	0	0	0	0
0	0	0	0	0	0	0	0	0	0.0681682
0	0	0	0	0	0.003776918	0	-0.060745723	0	0
0	0	0	0	0	0	0	0	0	0
0	0.026307497	0	-0.056157052	0	0	0	0	0	0
0	0	0	0.007457413	0	0	0	0	0	0
0	0	0	0.007542755	0	0	0	0	0	0.064574106
0	0.001677716	0	0	0	0	0	0	0	0
0	0	0	0	0	0	0	0	0	0.006078451
0	0.001573041	0	0	0	0	0	0	0	0.061963704
0	0.026587202	0	0	0	-0.063390567	0	0	0	0
0	0	0	0	0	0.014155092	0	-0.044336082	0	0
0	0	0	0	0	0.015858116	0	0	0	0
0	0	0	-0.005309984	0	0	0	-0.044281149	0	0
0	0	0	0.00893588	0	0	0	0	0	0
0	0	0	-0.016155204	0	0	0	0	0	0
0	0	0	-0.052305893	0	0	0	0	0	0
0	0	0	0	0	0.014453242	0	0	0	0
0	0	0	0	0	0	0	0	0	0
0	0	0	-0.023302354	0	0.035632618	0	0	0	0
0	0	0	-0.005903071	0	0	0	0	0	0
0	0	0	-0.016579641	0	0	0	-0.087088809	0	0
0	0	0	-0.013875035	0	-0.05411985	0	0	0	0
0	0	0	-0.057100526	0	0	0	0	0	0
0	0	0	-0.018695137	0	0.005908547	0	-0.081878045	0	0
0	0	0	-0.057070942	0	0	0	0	0	0.049497394

0	0	0	0	0	-0.065814132	0	0	0	0
0	0	0	0	0	-0.013230836	0	-0.088204411	0	0
0	0	0	0	0	0	0	0	0	0.074673597
0	0	0	0	0	0	0	0	0	0
0	0.04072743	0	0.021135029	0	0	0	0	0	0
0	0.029136365	0	0	0	-0.015170525	0	0	0	0
0	0.035346287	0	0	0	0	0	0	0	0
0	0.053399503	0	0	0	0	0	0	0	0
0	0	0	0.030145664	0	-0.042265497	0	0	0	0
0	0.02285822	0	-0.056836012	0	0	0	0	0	0
0	0	0	-0.051787643	0	0	0	0	0	0
0	0	0	0	0	-0.025657803	0	0	0	0
0	-0.045727712	0	0	0	-0.076120578	0	0	0	0
0	-0.046122946	0	0	0	-0.076096243	0	0	0	0.10841798
0	0	0	0	0	0	0	0	0	0.076507802
0	0	0	0	0	0	0	0	0	0
0	0	0	0	0	-0.053308721	0	0	0	0
0	0	0	0	0	-0.053219756	0	0	0	0.075412684
0	0	0	-0.000871461	0	0	0	0	0	0
0	0	0	0	0	0	0	0	0	0
0	0.05026086	0	0	0	0	0	0	0	0
0	0	0	-0.004921439	0	-0.069450799	0	0	0	0
0	0	0	0	0	-0.07368717	0	0	0	-0.005001078
0	0	0	-0.004916558	0	-0.069430892	0	0	0	0.066217835
0	0	0	-0.040031345	0	0	0	0	0	0
0	0	0	-0.039956965	0	0	0	0	0	0.072145914
0	-0.043647438	0	-0.067491238	0	0	0	0	0	0
0	-0.04394891	0	-0.067455241	0	0	0	0	0	0.095055741
0	0	0	0	0	0	0	0	0	0.060085017
0	0	0	0	0	0	0	0	0	0
0	0.041931362	0	0	0	0	0	0	0	0
0	0.018350022	0	0	0	-0.055899299	0	0	0	0
0	-0.009049646	0	0	0	0	0	0	0	0
0	-0.009447639	0	0	0	0	0	0	0	0.095136128
0	0	0	0	0	0	0	0	0	0.036794195
0	0.01859377	0	0	0	0	0	0	0	0
0	0.018466363	0	0	0	0	0	0	0	0.042595308
0	0	0	-0.020178433	0	-0.044155553	0	0	0	0
0	0	0	0	0	0	0	0	0	0.032122085
0	0	0	-0.065268809	0	0	0	0	0	-0.014636955
0	0.006046984	0	-0.017775187	0	-0.058995417	0	0	0	0
0	0.019719262	0	-0.045450025	0	0	0	0	0	0
0	0.0112585	0	0	0	0	0	0	0	0
0	0.011084732	0	0	0	0	0	0	0	0.05075143
0	0	0	0	0	0	0	0	0	0.030103788
0	0.04599232	0	0	0	0	0	0	0	0
0	0	0	0	0	0	0	0	0	0

0	0.04599491	0	0	0	0	0	-0.080812924	0	0
0	0	0	0	0	0	0	0	0	0.055350694
0	0	0	0	0	0	0	-0.078854184	0	0.058574299
0	0	0	0	0	0	0	-0.078745081	0	0
0	0	0	0	0	-0.069686968	0	0	0	0
0	0	0	0	0	-0.06965414	0	0	0	0.058043392
0	0.046733672	0	0	0	0	0	0	0	0
0	0.046152175	0	0	0	0	0	-0.046403606	0	0
0	0	0	0	0	0	0	0	0	0
0	0	0	0	0	0	0	0	0	0.058142611
0	0	0	0	0	0	0	-0.04618445	0	0.057956398
0	0	0	0	0	0	0	-0.045900067	0	0
0	0.045872546	0	0	0	0	0	0	0	0
0	0	0	0	0	0	0	-0.05183669	0	0
0	0	0	0	0	-0.039656667	0	0	0	0
0	0	0	0	0	-0.039621051	0	0	0	0.057657989
0	0	0	0	0	-0.033812963	0	-0.042983968	0	0.057982346
0	0.045351531	0	0	0	0	0	-0.034445533	0	0
0	0	0	0	0	-0.03415659	0	-0.042566261	0	0
0	0	0	0	0	-0.035131449	0	-0.04764444	0	0
0	0	0	0	0	0	0	0	0	0
0	0	0	0	0	0	0	-0.035907823	0	0
0	0	0	0	0	0	0	0	0	0
0	0	0	0	0	0	0	0	0	0.056130075
0	-0.032663487	0	0	0	0	0	0	0	0.049650939
0	0	0	-0.025533492	0	0	0	-0.059757854	0	0
0	0.030567882	0	0	0	0	0	0	0	0
0	0.030711776	0	0	0	0	0	0	0	0.026526959
0	0	0	-0.01867985	0	0	0	0	0	0
0	0	0	0	0	-0.043507526	0	0	0	0
0	0	0	0	0	0	0	-0.037134783	0	0.056656825
0	0	0	0	0	0	0	-0.036421093	0	0
0	0	0	0	0	0	0	0	0	0
0	0	0	0	0	-0.024213721	0	0	0	0
0	0.029886335	0	0	0	-0.067318601	0	0	0	0
0	0	0	-0.020070954	0	0	0	-0.058181746	0	0.054569404
0	0.043657525	0	0	0	0	0	0	0	0
0	0	0	0	0	-0.028462559	0	0	0	0
0	0	0	0	0	-0.028456707	0	0	0	0.056247574
0	0	0	-0.020306957	0	0	0	-0.057900359	0	0
0	0.027375975	0	0	0	0	0	-0.074499466	0	0.032722228
0	0.043949271	0	-0.003853652	0	0	0	0	0	0
0	0.027833068	0	0	0	0	0	-0.074458376	0	0
0	0.045913296	0	0	0	0.003360932	0	0	0	0
0	0.040673549	0	0	0	-0.019982486	0	-0.057148049	0	0
0	0	0	-0.014525124	0	0	0	0	0	0
0	0	0	-0.014502427	0	0	0	0	0	0.053134731

0	0.039335097	0	0	0	-0.028768341	0	0	0	0
0	0.041026407	0	-0.01340931	0	0	0	-0.064945932	0	0
0	0	0	0	0	0	0	-0.048937	0	0.059258038
0	0	0	0	0	0	0	0	0	0
0	0	0	0	0	0	0	-0.048645067	0	0
0	0	0	0	0	0	0	0	0	0.05364827
0	0	0	0	0	0	0	0	0	0.010635125
0	0.045544618	0	0	0	0	0	-0.073871157	0	0
0	0	0	0	0	0	0	-0.070786923	0	0.059486849
0	0	0	0	0	0	0	-0.073505727	0	0.084237729
0	0	0	0	0	0	0	-0.070651946	0	0
0	0.043178461	0	0	0	0	0	-0.049023883	0	0
0	0	0	0	0	0	0	-0.08374543	0	0
0	0.029485535	0	0	0	0	0	0	0	0
0	0.029549453	0	0	0	0	0	0	0	0.030251
0	0	0	0	0	-0.060276275	0	0	0	0
0	0	0	0	0	-0.060202778	0	0	0	0.060513591
0	-0.003463149	0	0	0	-0.065991593	0	0	0	0
0	0	0	0	0	-0.065681571	0	0	0	0.014149405
0	0	0	3.69E-06	0	-0.065693091	0	0	0	0
0	-0.003625775	0	0	0	-0.065972267	0	0	0	0.061274283
0	0	0	4.35E-05	0	-0.065695774	0	0	0	0.057895027
0	0.047985214	0	0	0	0	0	0	0	0
0	0	0	0	0	0	0	0	0	0
0	0	0	0	0	0	0	0	0	0.057353464
0	0.042846411	0	0	0	0	0	0	0	0
0	0	0	0	0	0	0	-0.040102554	0	0.057973337
0	0	0	0	0	0	0	-0.039373186	0	0
0	0.047129767	0	0.003407107	0	0	0	0	0	0
0	0	0	0	0	0	0	0	0	0
0	0	0	0	0	0	0	0	0	0.061697985
0	0	0	0	0	-0.026959457	0	-0.052715724	0	0.059449666
0	0	0	-0.009995279	0	-0.061567806	0	0	0	0
0	0	0	0	0	-0.027309104	0	-0.05228765	0	0
0	0	0	-0.011625236	0	0	0	0	0	0
0	0	0	-0.01156576	0	0	0	0	0	0.055870007
0	0	0	0	0	-0.070084814	0	0	0	0
0	0.042274629	0	0	0	0	0	-0.037354522	0	0
0	0	0	0.016369236	0	0	0	-0.059832062	0	0.064633881
0	0	0	0	0	-0.035315522	0	0	0	0
0	0	0	0	0	-0.035278618	0	0	0	0.059034168
0	0	0	0.016179869	0	0	0	-0.059489121	0	0
0	0	0	0	0	0	0	0	0	0.019322937
0	0.02306301	0	0	0	0	0	0	0	0
0	0.023165825	0	0	0	0	0	0	0	0.03488292
0	0	0	0	0	0	0	-0.04596213	0	0.06139627
0	0	0	-0.017044181	0	0	0	-0.064031684	0	0.056494555

0	0	0	0	0	0	0	-0.045179368	0	0
0	0	0	-0.01728758	0	0	0	-0.063739923	0	0
0	0	0	0	0	0	0	-0.061413741	0	0.059820773
0	0.007580664	0	0	0	0	0	-0.058251628	0	0.055216181
0	0	0	0	0	0	0	-0.061112282	0	0
0	0.007953272	0	0	0	0	0	-0.058009727	0	0
0	0	0	0	0	0.002308738	0	-0.05889799	0	0.062063806
0	0	0	0	0	0	0	-0.05763933	0	0.057700276
0	0	0	0	0	0.002045019	0	-0.058452666	0	0
0	0.019910244	0	0	0	0	0	-0.077803182	0	0.041091336
0	0	0	-0.021470289	0	0	0	0	0	0
0	0.020421232	0	0	0	0	0	-0.07774719	0	0
0	0	0	0	0	-0.022547265	0	-0.031991502	0	0
0	0	0	0	0	0	0	-0.055272017	0	0
0	0	0	-0.010832345	0	0	0	0	0	0
0	0	0	0	0	-0.016122326	0	0	0	0
0	0	0	-0.010813895	0	0	0	0	0	0.054936463
0	0	0	0	0	-0.016114605	0	0	0	0.057123066
0	0	0	0	0	0	0	0	0	0
0	0	0	0	0	0	0	0	0	0.054643604
0	0	0	0	0	0	0	0	0	0.00723849
0	0.0457498	0	0	0	0.016012646	0	0	0	0
0	0.040206127	0	0	0	0	0	0	0	0
0	0	0	0	0	0	0	0	0	0
0	0	0	0	0	0	0	-0.074764554	0	0.060671858
0	0	0	0	0	0	0	-0.032404362	0	0
0	0	0	0	0	0	0	-0.077105243	0	0.084408729
0	0	0	0	0	0	0	-0.074624133	0	0
0	0	0	-0.010557649	0	0	0	-0.043971726	0	0
0	0.048742595	0	0.035311082	0	0	0	-0.066528132	0	0
0	0.041708932	0	-0.001038311	0	0	0	0	0	0
0	0	0	0	0	0	0	0	0	0
0	0	0	-0.059491905	0	0	0	0	0	0
0	0	0	-0.059462993	0	0	0	0	0	0.047876346
0	0	0	0	0	0	0	-0.036919849	0	0
0	0	0	0	0	-0.023382951	0	0	0	0
0	0	0	0	0	-0.038652251	0	-0.050227539	0	0
0	0	0	-0.018925685	0	0	0	-0.031452995	0	0
0	0	0	0	0	-0.04491315	0	0	0	0
0	0.038637948	0	-0.011198941	0	0	0	-0.070090715	0	0
0	0.03903163	0	0	0	-0.01342628	0	-0.066551843	0	0
0	0	0	0.016859262	0	0	0	0	0	0
0	0	0	0.016937564	0	0	0	0	0	0.062661789
0	0	0	-0.019835385	0	0	0	0	0	0
0	0	0	0	0	0	0	-0.048748966	0	0.057621981
0	0	0	0.013559905	0	-0.047569462	0	0	0	0
0	0	0	0	0	0	0	-0.048031492	0	0

0	0	0	0	0	-0.0238219	0	-0.031949374	0	0
0	0	0	-0.013502712	0	-0.022514089	0	-0.043303682	0	0
0	0	0	0	0	0.009637595	0	-0.047335579	0	0.06017278
0	0	0	-0.010503427	0	0	0	0	0	0
0	0	0	0	0	0.009594039	0	-0.046575086	0	0
0	0.007106079	0	0	0	0	0	-0.047690477	0	0.054262067
0	0.007410233	0	0	0	0	0	-0.047016042	0	0
0	0.042179931	0	0	0	0	0	-0.078507859	0	0
0	0	0	-0.027528974	0	0	0	-0.061338074	0	0
0	0	0	0	0	0	0	-0.047308119	0	0.04430746
0	0	0	4.66E-05	0	0	0	-0.047515983	0	0.060773766
0	0	0	-4.02E-05	0	0	0	-0.046742437	0	0
0	0	0	0	0	-0.025222746	0	0	0	0
0	0	0	0	0	0.011385134	0	0	0	0
0	0	0	0	0	0	0	0	0	0
0	0.033368306	0	0	0	-0.057084859	0	0	0	0
0	0	0	0	0	0.011461572	0	0	0	0.059494705
0	0	0	0	0	0	0	0	0	0.057148534
0	0.007643839	0	0	0	0	0	0	0	0
0	0.007633565	0	0	0	0	0	0	0	0.053201234
0	0.037196958	0	0	0	-0.02473129	0	0	0	0
0	0	0	0	0	0	0	0	0	-0.000223994
0	0.029475187	0	-0.005166075	0	-0.058951312	0	0	0	0
0	0.04264883	0	0	0	0.023620192	0	-0.074032348	0	0
0	0.022355883	0	0	0	0	0	0	0	0
0	0.048123079	0	0	0	0.046208635	0	0	0	0
0	0.022378414	0	0	0	0	0	0	0	0.038258493
0	0.047384574	0	0	0	0.043878175	0	-0.047170122	0	0
0	0	0	-0.015954861	0	-0.010334569	0	0	0	0
0	0	0	0	0	0	0	0	0	0
0	0	0	0	0	0	0	0	0	0.062998218
0	0.048110036	0	0.036359704	0	0	0	0	0	0
0	0.033582711	0	0	0	0	0	-0.067342398	0	0
0	0.035790197	0	0	0	0	0	-0.045414204	0	0
0	0	0	-0.00672633	0	0	0	0	0	0
0	0	0	-0.006666214	0	0	0	0	0	0.058010632
0	0	0	0	0	0	0	0	0	0.016632287
0	0	0	-0.043023535	0	0	0	0	0	0
0	0	0	0	0	0	0	-0.058466647	0	0
0	0	0	-0.042953292	0	0	0	0	0	0.05835781
0	0.044845784	0	0	0	0	0	0	0	0
0	0.026069191	0	-0.058756164	0	0	0	0	0	0
0	0.04552603	0	0.007413697	0	0	0	0	0	0
0	0.021249294	0	0	0	0.025017964	0	0	0	0
0	0.0215021	0	0	0	0.025256695	0	0	0	0.038249034
0	0	0	-0.005486081	0	-0.006477109	0	-0.079352405	0	0.062059745
0	-0.002345873	0	0	0	-0.012183174	0	-0.078255735	0	0.065222224

0	0	0	-0.009236012	0	0	0	-0.08094134	0	0.061720381
0	0	0	-0.009393574	0	0	0	-0.0818664	0	0.076979361
0	0	0	0	0	-0.058179211	0	0	0	0
0	0	0	0	0	-0.011151216	0	-0.079402835	0	0.076165087
0	0	0	0	0	-0.011036283	0	-0.07892819	0	0.063287056
0	-0.000359603	0	-0.009538942	0	0	0	-0.081690214	0	0.061583676
0	0	0	0	0	-0.058103845	0	0	0	0.063001305
0	0	0	-0.005612922	0	-0.006680346	0	-0.078995532	0	0
0	0	0	0.047824202	0	-0.053457611	0	0	0	0
0	-0.001880045	0	0	0	-0.012318553	0	-0.078094062	0	0
0	0	0	-0.00948631	0	0	0	-0.080659647	0	0
0	0	0	0	0	-0.01135826	0	-0.078563752	0	0
0	0.000114728	0	-0.009665029	0	0	0	-0.081557591	0	0
0	0	0	0.047912855	0	-0.05344395	0	0	0	0.071660651
0	0	0	-0.010942073	0	0.020169607	0	0	0	0
0	-0.011933246	0	0	0	-0.066693173	0	0	0	0
0	-0.012144924	0	0	0	-0.066671865	0	0	0	0.07070332
0	0	0	-0.010939952	0	0.020242403	0	0	0	0.056802281
0	0	0	0	0	-0.014082157	0	-0.03996222	0	0
0	0.004329425	0	0	0	0	0	-0.086444619	0	0.059870093
0	0	0	0	0	0	0	-0.035194407	0	0
0	0.004158655	0	0	0	0	0	-0.088811806	0	0.082199321
0	0	0	0	0	0.010262763	0	0	0	0
0	0	0	0	0	0	0	-0.086079014	0	0.085457337
0	0	0	0	0	0	0	0	0	0
0	0	0	0	0	0.010336589	0	0	0	0.05659799
0	0.004893394	0	0	0	0	0	-0.086373119	0	0
0	0.006347332	0	0	0	0	0	0	0	0
0	0	0	0	0	0.01014069	0	0	0	-0.001781812
0	0.006367585	0	0	0	0	0	0	0	0.051451453
0	0	0	-0.001584815	0	-0.06423104	0	0	0	0
0	0	0	0	0	-0.065582326	0	0	0	0.011790694
0	0	0	-0.00112717	0	0	0	0	0	0
0	0	0	0	0	0	0	0	0	-0.005964117
0	0	0	-0.00155024	0	-0.064226877	0	0	0	0.059096743
0	0.007133844	0	0.001343292	0	0	0	0	0	0
0	0.006513821	0	0	0	0	0	0	0	-0.009755668
0	0.031998365	0	0	0	0	0	-0.049179598	0	0
0	0	0	-0.001089538	0	0	0	0	0	0.057116373
0	0.007152382	0	0.00138098	0	0	0	0	0	0.053125271
0	0	0	-0.001015192	0	0	0	0	0	-0.002940687
0	0	0	-0.004929871	0	0	0	-0.04782635	0	0
0	0.044712983	0	-0.015182682	0	0.056380407	0	0	0	0
0	0	0	-0.05324512	0	0	0	0	0	0
0	0	0	0	0	0	0	-0.039955109	0	0
0	0.032083475	0	0	0	0	0	0	0	0
0	0.044074624	0	0	0	0.042203297	0	0	0	0

0	0.038418001	0	0.010306505	0	0	0	-0.047758082	0	0
0	0	0	-0.015246699	0	0	0	-0.034738797	0	0
0	0	0	-0.011826897	0	-0.057245156	0	0	0	0
0	0	0	0	0	-0.010982215	0	0	0	0
0	0	0	-0.004900136	0	0	0	0	0	0
0	0	0	-0.016323346	0	0	0	0	0	0
0	0	0	-0.020790594	0	0	0	0	0	0
0	0	0	0	0	-0.011122201	0	-0.035711529	0	0
0	0	0	0	0	0	0	-0.046742582	0	0
0	0	0	0	0	-0.031602297	0	-0.059702735	0	0
0	0	0	-0.024757437	0	0	0	-0.067238208	0	0
0	0.036205179	0	-0.041396473	0	0	0	0	0	0
0	0	0	-0.05690669	0	0	0	0	0	0.006436373
0	3.69E-05	0	-0.056909866	0	0	0	0	0	0
0	-3.09E-05	0	-0.056886807	0	0	0	0	0	0.048193047
0	0	0	-0.015425538	0	-0.013931895	0	-0.053068039	0	0
0	0	0	0	0	-0.012834724	0	0	0	0
0	0	0	0	0	-0.039939616	0	0	0	0
0	0	0	0.016533763	0	-0.044822508	0	0	0	0
0	0	0	0	0	0	0	0	0	0.076086742
0	0	0	0	0	0	0	0	0	0
0	0	0	-0.018697178	0	0.00465883	0	0	0	0
0	-0.013192623	0	0	0	-0.02953933	0	0	0	0
0	0	0	0	0	-0.021923903	0	0	0	0
0	-0.013383735	0	0	0	-0.029533348	0	0	0	0.076001933
0	0	0	0.008496977	0	0	0	-0.070033111	0	0
0	0	0	0	0	-0.021839577	0	0	0	0.066438245
0	0	0	0	0	0.000270883	0	-0.06889303	0	0
0	0.034272839	0	0.007879553	0	0	0	0	0	0
0	0	0	0	0	-0.025185664	0	0	0	0.001601023
0	0	0	0	0	0.015374066	0	-0.042765028	0	0
0	0	0	0.009372853	0	0	0	-0.043218975	0	0
0	0.054843421	0	0	0	0	0	0	0	0
0	0	0	0	0	0.013946701	0	-0.047829531	0	0
0	0.034014445	0	-0.011579791	0	0.013237985	0	-0.096063634	0	0
0	0	0	0	0	0.015770967	0	0	0	0
0	0	0	0.00875272	0	0	0	0	0	0
0	0	0	0.008855863	0	0	0	0	0	0.069696657
0	0	0	-0.004969389	0	0	0	-0.047786001	0	0
0	0.032085458	0	-0.004700291	0	0	0	-0.092494167	0	0
0	0	0	0.010578966	0	0	0	0	0	0
0	0	0	0.009899564	0	-0.003171041	0	-0.058359148	0	0
0	0.034924575	0	0	0	0.003261375	0	-0.097217148	0	0
0	0.002956656	0	0	0	0	0	0	0	0
0	0	0	0	0	0	0	0	0	0.012768064
0	0.002813937	0	0	0	0	0	0	0	0.065646449
0	0	0	-0.021501013	0	0.033568558	0	-0.042926824	0	0

0	0	0	-0.0421923	0	0	0	0	0	0
0	0	0	-0.042121891	0	0	0	0	0	0.060560437
0	0.00716106	0	0.010570041	0	0	0	0	0	0
0	0.007097693	0	0.010613998	0	0	0	0	0	0.058596964
0	0.030481666	0	0	0	-0.056014244	0	0	0	0
0	0	0	0.007476111	0	0	0	0	0	0.008705562
0	0.001643954	0	0	0	0	0	0	0	0.004306678
0	0.025656808	0	-0.008045626	0	-0.056575216	0	0	0	0
0	0.040750169	0	0.052368763	0	-0.04231658	0	0	0	0
0	0	0	-0.002211902	0	0.017802515	0	0	0	0
0	0	0	-0.022749481	0	0.034982211	0	0	0	0
0	0	0	-0.016109444	0	0	0	0	0	0
0	0.026624475	0	0	0	0	0	0	0	0
0	0.026449798	0	0	0	0	0	0	0	0.050228357
0	0	0	-0.018011529	0	0.00244336	0	-0.087837222	0	0
0	0	0	-0.013948589	0	-0.053479476	0	0	0	0
0	-0.007982659	0	-0.057812657	0	0	0	0	0	0
0	-0.008096176	0	-0.057785467	0	0	0	0	0	0.056923597
0	0	0	-0.057096277	0	0	0	0	0	0.004174294
0	0.044262604	0	0.023591164	0	0	0	0	0	0
0	0	0	0	0	0	0	0	0	0.046294686
0	0.032900613	0	-0.041838428	0	0	0	0	0	0
0	0.031360403	0	0	0	-0.011900039	0	0	0	0
0	0	0	0.033774529	0	-0.047653827	0	0	0	0
0	-0.035948044	0	0	0	-0.061803979	0	0	0	0
0	-0.036316664	0	0	0	-0.061742753	0	0	0	0.10525083
0	-0.045824022	0	-0.005817963	0	-0.071120498	0	0	0	0
0	-0.046049365	0	0	0	-0.076116533	0	0	0	0.043655788
0	-0.046209958	0	-0.005736254	0	-0.071166462	0	0	0	0.10744361
0	0.018906361	0	0	0	0	0	0	0	0
0	0.018676356	0	0	0	0	0	0	0	0.059271936
0	0	0	0	0	0	0	0	0	0.044528365
0	0	0	-0.004965667	0	-0.049032794	0	0	0	0
0	0	0	0	0	-0.05328798	0	0	0	0.010975497
0	0	0	-0.004935231	0	-0.048972876	0	0	0	0.074508108
0	-0.026557271	0	-0.044718079	0	0	0	0	0	0
0	-0.02686454	0	-0.044650051	0	0	0	0	0	0.093849414
0	0	0	-0.004932109	0	-0.069444536	0	0	0	-0.006032695
0	0	0	-0.040007591	0	0	0	0	0	0.011755813
0	0.019118288	0	0	0	0	0	0	0	0
0	0.019001564	0	0	0	0	0	0	0	0.04228168
0	-0.043876488	0	-0.067475714	0	0	0	0	0	0.03136512
0	0	0	0	0	0	0	0	0	0.03040879
0	0.016551621	0	-0.017126596	0	-0.041389251	0	0	0	0
0	-0.00940035	0	0	0	0	0	0	0	0.047518464
0	0.01848133	0	0	0	0	0	0	0	0.01363637
0	0.011102018	0	0	0	0	0	0	0	0.019153829

0	0.046974927	0	0	0	0	0	-0.052165905	0	0
0	0.046656953	0	0	0	0	0	0	0	0
0	0	0	0	0	0	0	-0.051847891	0	0.058148046
0	0	0	0	0	0	0	-0.051560711	0	0
0	0.046223884	0	0	0	0	0	-0.036834826	0	0
0	0	0	0	0	-0.034497843	0	-0.047806621	0	0.058161788
0	0	0	0	0	-0.034842452	0	-0.04738621	0	0
0	0.032295281	0	0	0	0	0	0	0	0
0	0.032477741	0	0	0	0	0	0	0	0.024825304
0	0	0	0	0	0	0	-0.039463425	0	0.056694602
0	0	0	0	0	0	0	-0.038749492	0	0
0	0	0	0	0	0	0	0	0	0
0	0	0	0	0	-0.0432357	0	0	0	0
0	0	0	0	0	0	0	0	0	0.056040287
0	0	0	0	0	-0.043200203	0	0	0	0.057814269
0	0.044801472	0	0	0	0	0	0	0	0
0	0.047321416	0	0	0	0.005821466	0	0	0	0
0	0.045479273	0	-0.001647478	0	0	0	0	0	0
0	0.02885726	0	0	0	0	0	-0.080014462	0	0.031522031
0	0.029322317	0	0	0	0	0	-0.079976381	0	0
0	0	0	0	0	-0.026935864	0	0	0	0
0	0	0	0	0	-0.026929695	0	0	0	0.056160139
0	0	0	-0.019438366	0	0	0	-0.064027944	0	0.05487501
0	0.04141316	0	0	0	-0.020413779	0	-0.062285559	0	0
0	0	0	-0.019676985	0	0	0	-0.06374296	0	0
0	0	0	-0.012909156	0	0	0	0	0	0
0	0.042151716	0	-0.012452381	0	0	0	-0.071170112	0	0
0	0	0	-0.01289094	0	0	0	0	0	0.053344379
0	0	0	0	0	0	0	0	0	0
0	0.046777024	0	0	0	0	0	-0.077935774	0	0
0	0	0	0	0	0	0	0	0	0.054059822
0	0	0	0	0	0	0	0	0	0.007628622
0	0.039259337	0	0	0	-0.032352879	0	0	0	0
0	0	0	0	0	0	0	-0.074828187	0	0.060241318
0	0	0	0	0	0	0	-0.078909155	0	0.086702893
0	0	0	0	0	0	0	-0.074692129	0	0
0	0	0	0	0	-0.062568652	0	0	0	0
0	0	0	0	0	-0.062495019	0	0	0	0.061523922
0	-0.004064799	0	0	0	-0.070043682	0	0	0	0
0	0	0	0.002127516	0	-0.071515758	0	0	0	0
0	0	0	0	0	-0.069679754	0	0	0	0.011762882
0	-0.004212884	0	0	0	-0.070021376	0	0	0	0.061978879
0	0	0	0.002163642	0	-0.071513345	0	0	0	0.058432495
0	0.030741043	0	0	0	0	0	0	0	0
0	0.03081553	0	0	0	0	0	0	0	0.029236334
0	0.029845048	0	0	0	0	0	-0.046325018	0	0.029959837
0	0.030081094	0	0	0	0	0	-0.046180075	0	0

0	0.049407291	0	0	0	0	0	0	0	0
0	0.051911117	0	0.017580945	0	0	0	-0.050130886	0	0
0	0.046053334	0	0	0	0	0	-0.033203612	0	0
0	0	0	0	0	-0.022071497	0	-0.032280392	0	0.057786179
0	0	0	0	0	-0.022359949	0	-0.031814556	0	0
0	0	0	0	0	0	0	0	0	0
0	0	0	0	0	0	0	0	0	0.062946762
0	0.047740203	0	0.003078874	0	0	0	0	0	0
0	0.044681149	0	0	0	0	0	-0.050393883	0	0
0	0	0	0	0	-0.023536916	0	-0.037047428	0	0
0	0.045515818	0	0	0	-0.002718735	0	-0.044687998	0	0
0	0.031228331	0	0	0	0	0	0	0	0
0	0.031361222	0	0	0	0	0	0	0	0.026667472
0	0	0	0	0	0	0	-0.059861997	0	0
0	0	0	0.030695966	0	-0.056899413	0	0	0	0
0	0	0	0.030769519	0	-0.05690232	0	0	0	0.063110062
0	0	0	-0.012246855	0	0	0	0	0	0
0	0	0	-0.012189588	0	0	0	0	0	0.055927641
0	0	0	0	0	0	0	-0.03478034	0	0
0	0.05164361	0	0.017686146	0	0	0	0	0	0
0	0	0	0	0	0	0	-0.035941902	0	0.057334799
0	0	0	0	0	0	0	-0.035219958	0	0
0	0	0	0	0	0	0	-0.049240719	0	0.05612634
0	0	0	0	0	0	0	0	0	0.017501145
0	0	0	0	0	0	0	-0.04896245	0	0
0	0.043966627	0	0	0	0	0	0	0	0
0	0	0	0	0	0	0	-0.039040887	0	0
0	0	0	0	0	0	0	0	0	0
0	0	0	-0.00046531	0	0	0	-0.046086444	0	0.057871944
0	0	0	0	0	0	0	-0.046185832	0	0.058238237
0	0.029785417	0	0	0	0	0	-0.035943137	0	0.028621205
0	0.029928987	0	0	0	0	0	-0.035581642	0	0
0	0	0	-0.000654574	0	0	0	-0.045762959	0	0
0	0	0	-0.009917815	0	0	0	-0.049737385	0	0
0	0.047482874	0	0	0	0.007017438	0	0	0	0
0	0	0	-0.017212515	0	0	0	-0.033941097	0	0
0	0	0	0	0	-0.026029174	0	0	0	0
0	0	0	0	0	-0.026022428	0	0	0	0.05687811
0	0	0	0	0	-0.038868401	0	-0.054171517	0	0
0	0	0	0	0	-0.022026271	0	0	0	0
0	0	0	0	0	-0.022125612	0	-0.034368527	0	0
0	0.012789492	0	0	0	-0.036082176	0	0	0	0
0	0.014582091	0	0	0	-0.028775589	0	-0.048079735	0	0.044371764
0	0.012754769	0	0	0	-0.036065057	0	0	0	0.045721397
0	0.014860626	0	0	0	-0.028940303	0	-0.04785979	0	0
0	0.043413949	0	0	0	0	0	-0.036019099	0	0
0	0	0	0	0	-0.026384596	0	-0.035363724	0	0.057389136

0	0	0	-0.01829109	0	0	0	0	0	0
0	0	0	0	0	-0.026423734	0	-0.034638627	0	0
0	0	0	-0.011549658	0	-0.02491934	0	-0.048013464	0	0
0	0	0	0	0	-0.040139604	0	0	0	0
0	0	0	0	0	-0.039646439	0	0	0	0.014426147
0	0.047371322	0	0.038229044	0	-0.04799725	0	0	0	0
0	0	0	0	0	-0.040081203	0	0	0	0.057300752
0	0	0	0	0	-0.034794933	0	-0.044686651	0	0.056906078
0	0.04671459	0	0	0	0.005966638	0	-0.034882241	0	0
0	0.044691429	0	-0.002119307	0	0	0	-0.03419493	0	0
0	0	0	-0.001503757	0	-0.032486036	0	-0.043035614	0	0.057711849
0	0	0	0	0	-0.033840491	0	-0.042950101	0	0.053317036
0	0	0	0	0	-0.03513127	0	-0.044274684	0	0
0	0	0	-0.009565495	0	0	0	0	0	0
0	0	0	-0.001586828	0	-0.032754811	0	-0.042622085	0	0
0	0	0	0	0	-0.023748136	0	0	0	0
0	0	0	0	0	0	0	0	0	0
0	0	0	-0.012829129	0	0	0	-0.035560438	0	0.054606205
0	0	0	0	0	0	0	0	0	0.053634956
0	0	0	0.016775558	0	-0.052990201	0	0	0	0
0	0	0	-0.026719359	0	0	0	-0.065929122	0	0
0	0	0	-0.012909492	0	0	0	-0.03486399	0	0
0	0	0	0	0	-0.04740652	0	0	0	0
0	0	0	-0.001260314	0	0	0	0	0	0
0	0	0	0	0	0	0	0	0	0.012507906
0	0	0	-0.001212316	0	0	0	0	0	0.055922088
0	0.027372553	0	-0.006937119	0	0	0	0	0	0
0	0.020819149	0	-0.015689646	0	0	0	-0.062310267	0	0.035883859
0	0.024825727	0	0	0	-0.011579859	0	0	0	0
0	0.027516668	0	-0.006903822	0	0	0	0	0	0.028454914
0	0.024974778	0	0	0	-0.011468437	0	0	0	0.032263685
0	0.021170198	0	-0.015773919	0	0	0	-0.062194013	0	0
0	0.03044089	0	0	0	0	0	0	0	0
0	0	0	-0.014358268	0	-0.010357689	0	0	0	0
0	0.030603083	0	0	0	0	0	0	0	0.02504594
0	0	0	0	0	0	0	-0.038182667	0	0.054172812
0	0.030741646	0	0	0	0	0	0	0	-0.02057145
0	0	0	0	0	0	0	-0.037513468	0	0
0	0.034171989	0	0	0	-0.059231421	0	0	0	0
0	0	0	0	0	0	0	-0.037009673	0	0.046505661
0	0.029513309	0	-0.003167424	0	-0.064627348	0	0	0	0
0	0.04228093	0	-0.004286399	0	0	0	0	0	0
0	0	0	0	0	-0.028256021	0	0	0	0
0	0.044255284	0	0	0	0.002523863	0	0	0	0
0	0	0	-0.020037708	0	0	0	-0.058225977	0	0.064363337
0	0	0	-0.020067596	0	0	0	-0.058168813	0	0.054576286
0	0	0	-0.004039738	0	-0.024526101	0	0	0	0

0	0	0	0	0	-0.028252883	0	0	0	0.054708174
0	0.027672436	0	0	0	0	0	-0.071094651	0	0.033850169
0	0	0	0	0	-0.028464497	0	0	0	0.011070655
0	0	0	-0.0040038	0	-0.02455502	0	0	0	0.055507026
0	0.045095366	0	-0.007720869	0	0.010297515	0	0	0	0
0	0.02252496	0	0	0	0	0	-0.048991273	0	0.038168861
0	0	0	-0.02030134	0	0	0	-0.057884089	0	0
0	0.028120105	0	0	0	0	0	-0.071033737	0	0
0	0.022815002	0	0	0	0	0	-0.048805233	0	0
0	0.027368518	0	0	0	0	0	-0.074547153	0	0.057603896
0	0.040112356	0	-0.00471127	0	-0.016012057	0	-0.05711857	0	0
0	0.039735166	0	0	0	-0.021240751	0	-0.058442446	0	0
0	0	0	-0.014589514	0	0	0	0	0	0
0	0	0	-0.014572456	0	0	0	0	0	0.051310789
0	0	0	-0.014517094	0	0	0	0	0	0.007565274
0	0.039246305	0	0	0	-0.02891057	0	0	0	0
0	0	0	0	0	-0.0137322	0	-0.040236068	0	0.059180587
0	0.040803755	0	-0.013583552	0	0	0	-0.065439713	0	0
0	0	0	0	0	-0.014025664	0	-0.039758363	0	0
0	0.050690247	0	0.022748436	0	0	0	-0.053937569	0	0
0	0	0	0	0	0	0	-0.038810547	0	0.058651124
0	0	0	0	0	0	0	-0.038071443	0	0
0	0	0	0	0	0	0	-0.05239245	0	0.057171337
0	0	0	0	0	0	0	-0.052106536	0	0
0	0	0	0.005454164	0	0	0	-0.050109578	0	0.060241698
0	0	0	0.005256656	0	0	0	-0.049770513	0	0
0	0	0	0	0	0	0	-0.048928801	0	0.057617868
0	0	0	0	0	0	0	0	0	0.007982091
0	0.0430593	0	0	0	0	0	-0.036011798	0	0
0	0.030131586	0	0	0	0	0	0	0	0
0	0.030161186	0	0	0	0	0	0	0	0.033624693
0	0.041375554	0	0	0	0	0	-0.053987076	0	0
0	0	0	0	0	0	0	-0.070879454	0	0.083358652
0	0.023891313	0	0	0	0	0	0	0	0
0	0.0239853	0	0	0	0	0	0	0	0.034868643
0	0.044492907	0	0	0	0.005496121	0	-0.052510243	0	0
0	0	0	-0.043801157	0	0	0	0	0	0
0	0	0	-0.043730914	0	0	0	0	0	0.05952013
0	0	0	0.034278606	0	-0.054660809	0	0	0	0
0	0	0	0.034348584	0	-0.054659795	0	0	0	0.065124083
0	0.050322598	0	0.022755698	0	0	0	0	0	0
0	0.022216867	0	0	0	0	0	-0.039258995	0	0.037106044
0	0.022423375	0	0	0	0	0	-0.038789558	0	0
0	0.029573673	0	0	0	0	0	0	0	-0.010514931
0	0.029110971	0	-0.000806297	0	0	0	0	0	0
0	0.029192065	0	-0.000766728	0	0	0	0	0	0.03044687
0	0	0	0	0	-0.060242894	0	0	0	0.017990264

0	-0.000285729	0	0	0	-0.060337249	0	0	0	0
0	0	0	-0.000172208	0	-0.060127494	0	0	0	0
0	-0.000442071	0	0	0	-0.060296035	0	0	0	0.060900254
0	0	0	-0.000126743	0	-0.060094214	0	0	0	0.060492013
0	0	0	0	0	-0.013802186	0	0	0	0
0	0	0	0	0	-0.013793157	0	0	0	0.057774582
0	-0.003609866	0	0	0	-0.06599392	0	0	0	0.017674516
0	-0.00346861	0	-0.000137345	0	-0.065874428	0	0	0	0
0	0	0	0	0	0	0	0	0	0
0	0	0	2.98E-05	0	-0.065707142	0	0	0	0.014157943
0	-0.003629778	0	-9.78E-05	0	-0.065888778	0	0	0	0.061260458
0	0.047429143	0	0	0	0.019619168	0	0	0	0
0	0	0	0	0	0	0	0	0	0.054608615
0	0.040603346	0	0	0	0	0	0	0	0
0	0.049892392	0	0.005506537	0	0	0	0	0	0
0	0	0	0.004599857	0	0	0	0	0	0
0	0	0	0.004645367	0	0	0	0	0	0.05814336
0	0	0	0	0	0	0	0	0	0.009253832
0	0	0	0	0	-0.013856566	0	-0.039174983	0	0.058381671
0	0	0	-0.008960113	0	0	0	-0.038992657	0	0.056548002
0	0	0	0	0	-0.013889624	0	-0.038440127	0	0
0	0	0	-0.009042721	0	0	0	-0.038271803	0	0
0	0	0	0	0	0	0	-0.041252596	0	0.05521466
0	0.0216829	0	0.026193416	0	0	0	-0.062821161	0	0.046377804
0	0	0	0	0	0	0	-0.040571769	0	0
0	0.022053808	0	0.026225543	0	0	0	-0.062627859	0	0
0	0	0	-0.010313973	0	0	0	0	0	0
0	0	0	-0.010239498	0	0	0	0	0	0.059608018
0	0	0	0	0	0	0	-0.039954301	0	0.045921386
0	0	0	0	0	-0.026287434	0	-0.040245627	0	0
0	0.046669429	0	0	0	0.018896748	0	-0.038743843	0	0
0	0.039973345	0	0	0	0	0	-0.039225998	0	0
0	0.009167509	0	0	0	-0.023731336	0	-0.055973996	0	0.05090436
0	0	0	0.02879051	0	-0.018006327	0	-0.051822055	0	0.067012486
0	0	0	0	0	0	0	0	0	0.024172429
0	0	0	0.017574599	0	0	0	-0.046439003	0	0.063972793
0	0.009515737	0	0	0	-0.023905096	0	-0.055734316	0	0
0	0	0	0.028802243	0	-0.018297066	0	-0.051336296	0	0
0	0	0	0.017446547	0	0	0	-0.045624728	0	0
0	0	0	0	0	-0.027704287	0	-0.053987781	0	0.058636152
0	0	0	-0.003137908	0	-0.024204098	0	-0.052805459	0	0.058883241
0	0	0	0	0	-0.026958028	0	-0.052717446	0	0.059690028
0	0	0	-0.010039219	0	-0.061183176	0	0	0	0
0	0	0	0	0	-0.02805049	0	-0.053568032	0	0
0	0	0	-0.003232364	0	-0.024467485	0	-0.052384441	0	0
0	0	0	-0.011594431	0	0	0	0	0	0.015969328
0	0	0	0.016324992	0	0	0	-0.063541535	0	0.062346467

0	0	0	0	0	0	0	-0.03599315	0	0
0	0	0	0.01614386	0	0	0	-0.063206021	0	0
0	0.006508015	0	0	0	-0.033452748	0	0	0	0
0	0.042555328	0	0.000895256	0	0	0	-0.037463683	0	0
0	0.006437816	0	0	0	-0.0334389	0	0	0	0.05301585
0	0	0	0.016370808	0	0	0	-0.059836222	0	0.06541688
0	0	0	0	0	0	0	-0.047444683	0	0.057496594
0	0	0	0	0	-0.035308174	0	0	0	0.012083989
0	0	0	0	0	-0.035124923	0	0	0	0
0	0	0	0	0	0	0	-0.046729758	0	0
0	0	0	0	0	-0.03506523	0	0	0	0.059201114
0	0.014007058	0	-0.014047582	0	0	0	-0.06684731	0	0.043954425
0	0.014411578	0	-0.0141528	0	0	0	-0.066700391	0	0
0	0	0	0	0	0.010851539	0	-0.045738494	0	0.060731624
0	0	0	0	0	0.010807429	0	-0.044971217	0	0
0	0.022934717	0	0	0	0	0	0	0	0
0	0.020757496	0	-0.004999741	0	0	0	0	0	0
0	0.008244574	0	0	0	0	0	-0.046148237	0	0.053852768
0	0.023059534	0	0	0	0	0	0	0	0.033116892
0	0.008545658	0	0	0	0	0	-0.045478703	0	0
0	0.020862348	0	-0.004971976	0	0	0	0	0	0.036273202
0	0.023199488	0	0	0	0	0	0	0	-0.016355726
0	0.022859132	0	0	0	-0.000406135	0	0	0	0
0	0.023022208	0	0	0	-0.000281431	0	0	0	0.03502662
0	0	0	0	0	0	0	-0.045752424	0	0.045094387
0	0.007395984	0	0	0	0	0	-0.061923471	0	0.053120375
0	0	0	-0.017006071	0	0	0	-0.064082267	0	0.067495202
0	0	0	-0.017039739	0	0	0	-0.064017959	0	0.056501454
0	0.007770547	0	0	0	0	0	-0.061682301	0	0
0	0.012271477	0	0	0	0.008283877	0	-0.063059198	0	0.050722027
0	0	0	-0.017283318	0	0	0	-0.063722049	0	0
0	0.012623423	0	0	0	0.008244529	0	-0.06281751	0	0
0	0.049078948	0	0	0	0.046294102	0	-0.045522682	0	0
0	0	0	0	0	0.001086168	0	-0.061920701	0	0.059838358
0	0	0	0	0	0	0	-0.061402587	0	0.057954457
0	0.007584372	0	0	0	0	0	-0.058225722	0	0.050053742
0	0	0	0	0	0.000831291	0	-0.061501752	0	0
0	0	0	-0.009510869	0	0	0	-0.053059939	0	0
0	0	0	0	0	0.002293439	0	-0.058877368	0	0.0592177
0	0.020131257	0	0	0	0	0	-0.075021115	0	0.042041288
0	0	0	-0.021230807	0	0	0	0	0	0
0	0.019902099	0	0	0	0	0	-0.077849374	0	0.064989458
0	0	0	0	0	-0.02262579	0	0	0	0
0	0.020635595	0	0	0	0	0	-0.074938135	0	0
0	0.045663029	0	0.040352731	0	-0.045078855	0	0	0	0
0	0	0	0.005278158	0	-0.026145064	0	-0.030836795	0	0
0	0.049543681	0	0.037652048	0	0	0	-0.047114905	0	0

0	0	0	0	0	-0.021570182	0	-0.03096693	0	0
0	0	0	-0.018293191	0	0	0	-0.034750051	0	0
0	0	0	-0.010916553	0	0	0	0	0	0
0	0	0	0	0	-0.015909778	0	0	0	0
0	0.037433781	0	-0.041991108	0	0	0	0	0	0
0	0	0	-0.006679053	0	-0.009666741	0	0	0	0
0	0	0	-0.010899773	0	0	0	0	0	0.052896049
0	0	0	0	0	-0.015903738	0	0	0	0.055250936
0	0	0	-0.006650542	0	-0.009685133	0	0	0	0.055888645
0	0	0	-0.010827484	0	0	0	0	0	0.004969434
0	0	0	0	0	-0.016123582	0	0	0	0.007494558
0	0.04382007	0	0	0	0.014963227	0	0	0	0
0	0.044630899	0	-0.010605724	0	0.025448799	0	0	0	0
0	0	0	0	0	-0.023039748	0	-0.035268372	0	0
0	0	0	0	0	0	0	0	0	0.004263001
0	0.046468693	0	0.034427391	0	0	0	-0.071925606	0	0
0	0	0	-0.008575173	0	0	0	0	0	0
0	0	0	-0.009073799	0	0	0	-0.031625412	0	0
0	0.022456831	0	0.027271783	0	0	0	0	0	0
0	0.039695615	0	-0.001583101	0	0	0	0	0	0
0	0.02255003	0	0.027351329	0	0	0	0	0	0.043539805
0	0	0	0	0	0	0	-0.074853875	0	0.08365397
0	0	0	0.019473653	0	-0.057090282	0	0	0	0
0	0.04891618	0	0.033249348	0	0.003212885	0	-0.067980723	0	0
0	0	0	-0.012531733	0	-0.027442142	0	-0.050425204	0	0
0	0.022058082	0	0	0	0.025061283	0	-0.047600631	0	0.039026759
0	0.022354541	0	0	0	0.025236937	0	-0.047116291	0	0
0	-0.000142928	0	-0.059503991	0	0	0	0	0	0
0	0	0	-0.059487554	0	0	0	0	0	0.004128464
0	-0.000193938	0	-0.059479785	0	0	0	0	0	0.048054782
0	0.024712209	0	0	0	0.028737076	0	0	0	0
0	0	0	0.017119934	0	0	0	0	0	0
0	0	0	0.004661263	0	-0.027648169	0	0	0	0
0	0.024981739	0	0	0	0.028985625	0	0	0	0.035563924
0	0	0	-0.0150365	0	-0.009341341	0	-0.03124789	0	0
0	0	0	0.017188434	0	0	0	0	0	0.059520366
0	0.038188859	0	-0.007140939	0	-0.00744799	0	-0.066457591	0	0
0	0.038107524	0	-0.01162132	0	0	0	-0.071265994	0	0
0	0	0	0.022495639	0	-0.008730537	0	0	0	0
0	0.038090317	0	0	0	-0.014701857	0	-0.067831634	0	0
0	0	0	-0.015588008	0	-0.010181673	0	0	0	0
0	0	0	0.022565033	0	-0.008713289	0	0	0	0.06401106
0	0	0	0	0	0.009789936	0	-0.048582825	0	0.056983774
0	0	0	0.016870845	0	0	0	0	0	0.005359886
0	0	0	0	0	0.009751212	0	-0.047874137	0	0
0	0.007031636	0	0	0	0	0	-0.048918577	0	0.051198982
0	0.007309196	0	0	0	0	0	-0.048291792	0	0

0	0	0	-0.008610317	0	0.017536867	0	-0.046690632	0	0.058526339
0	0	0	-0.008717809	0	0.017589921	0	-0.045941251	0	0
0	0	0	0	0	0	0	-0.048540113	0	0.040829102
0	0	0	-9.55E-05	0	0	0	-0.048743654	0	0.057608552
0	0	0	-0.000178267	0	0	0	-0.048022874	0	0
0	0.022891622	0	0	0	0	0	0	0	0
0	0.022877756	0	0	0	0	0	0	0	0.041719142
0	0	0	0	0	0.01158572	0	0	0	0
0	0	0	0	0	0.009620466	0	-0.047146233	0	0.044964645
0	0.00861646	0	0.002888276	0	0	0	-0.047896268	0	0.053267406
0	0	0	0	0	0.01165585	0	0	0	0.056389419
0	0.008930924	0	0.002918169	0	0	0	-0.047236533	0	0
0	0.007125837	0	0	0	0	0	-0.047472163	0	0.036690983
0	0.007553648	0	0	0	0	0	0	0	0
0	0.045405462	0	0	0	0.044445256	0	0	0	0
0	0	0	3.05E-05	0	0	0	-0.047309957	0	0.044316441
0	0.007576388	0	0	0	0	0	0	0	0.050225606
0	0.044548139	0	0	0	0.041978991	0	-0.048935259	0	0
0	0.032759088	0	-0.005178736	0	-0.052693456	0	0	0	0
0	0	0	0	0	0.01138581	0	0	0	0.000770481
0	0	0	0	0	0	0	0	0	-0.004690883
0	0.050516821	0	0.017483695	0	0.032345458	0	0	0	0
0	0.032597937	0	0	0	0	0	-0.047853525	0	0
0	0.040366357	0	0	0	0.021004518	0	-0.076922402	0	0
0	0.007712895	0	0	0	0	0	0	0	-0.008623689
0	0.045970661	0	-0.012980587	0	0.054674219	0	-0.046290649	0	0
0	0.03730285	0	0	0	-0.024561556	0	0	0	0
0	0.045299089	0	0.035777618	0	0	0	0	0	0
0	0.023330855	0	0.00207788	0	0	0	0	0	0
0	0.022405738	0	0	0	0	0	0	0	-0.006021911
0	0.023373739	0	0.00211421	0	0	0	0	0	0.03770776
0	0	0	0	0	0	0	-0.038928961	0	0
0	0	0	-0.005302315	0	0	0	0	0	0
0	0	0	-0.005227875	0	0	0	0	0	0.061932322
0	0	0	0	0	0	0	0	0	0.021469282
0	0	0	0	0	-0.017956877	0	-0.048175765	0	0
0	0	0	-0.006698054	0	0	0	0	0	0.014683034
0	0.00912711	0	-0.041517839	0	0	0	0	0	0
0	0.009025599	0	-0.04148063	0	0	0	0	0	0.050576402
0	0.048154894	0	0.009473502	0	0	0	0	0	0
0	0	0	-0.003903855	0	0	0	-0.057549771	0	0
0	0	0	-0.042983776	0	0	0	0	0	0.019739397
0	0.021698659	0	-0.01151552	0	0.035885952	0	0	0	0
0	0.021990624	0	-0.011561163	0	0.036191965	0	0	0	0.035595643
0	0.021215333	0	0	0	0.025113742	0	0	0	0
0	0.021510526	0	0	0	0.025381301	0	0	0	0.035964254
0	0.021559944	0	0	0	0.025217768	0	0	0	-0.024029847

0	-0.008146317	0	0	0	-0.059941835	0	0	0	0
0	-0.008350677	0	0	0	-0.059896829	0	0	0	0.070290602
0	-0.002435685	0	-0.00552165	0	-0.007447075	0	-0.078244853	0	0.06429059
0	0	0	-0.005483966	0	-0.006411216	0	-0.07943224	0	0.075049404
0	0	0	-0.005483813	0	-0.006297414	0	-0.078959413	0	0.062279425
0	-0.002307834	0	0	0	-0.01210283	0	-0.07834936	0	0.077527318
0	0	0	-0.001785538	0	-0.056638342	0	0	0	0
0	0	0	-0.009191357	0	0	0	-0.081025512	0	0.076848609
0	-0.002237491	0	0	0	-0.012023711	0	-0.078057203	0	0.065261212
0	0	0	0	0	-0.058147454	0	0	0	0.017052523
0	-0.00017124	0	-0.009282313	0	0	0	-0.080898156	0	0.061864275
0	-0.000333704	0	-0.009477332	0	0	0	-0.081767106	0	0.078602785
0	0	0	0	0	-0.010973401	0	-0.079014187	0	0.076147522
0	0	0	-0.001742636	0	-0.056600447	0	0	0	0.062691329
0	-0.001977912	0	-0.005643734	0	-0.00747497	0	-0.078086206	0	0
0	0	0	-0.0056105	0	-0.006504779	0	-0.078607857	0	0
0	0	0	-0.014622527	0	0	0	-0.038217764	0	0
0	0	0	0.047102455	0	-0.052285064	0	0	0	0
0	0	0	-0.011280098	0	0.020606587	0	0	0	0
0	0	0	0.047860464	0	-0.053467705	0	0	0	0.013376508
0	-0.000555519	0	0.047641465	0	-0.053532255	0	0	0	0
0	-0.001761106	0	0	0	-0.012143252	0	-0.077873378	0	0
0	0.000306746	0	-0.009402848	0	0	0	-0.080738064	0	0
0	0	0	0.047166792	0	-0.052230708	0	0	0	0.072166442
0	-0.000728259	0	0.047670665	0	-0.053541283	0	0	0	0.072286638
0	0	0	-0.011283259	0	0.020683458	0	0	0	0.054339944
0	0	0	0	0	-0.008984583	0	-0.034594971	0	0
0	0	0	0	0	-0.010357819	0	0	0	0
0	0	0	-0.010949173	0	0.020173219	0	0	0	-0.004049534
0	-0.012127526	0	0	0	-0.066696632	0	0	0	0.02362923
0	-0.012004826	0	-0.002016669	0	-0.064970876	0	0	0	0
0	0	0	0.006307248	0	-0.018376836	0	-0.038597559	0	0
0	-0.012212627	0	-0.001967604	0	-0.064991523	0	0	0	0.070411157
0	0	0	-0.003312865	0	0	0	-0.034882357	0	0
0	0.004318311	0	0	0	0	0	-0.086528016	0	0.081272056
0	0.035322476	0	0.009273376	0	0	0	-0.049796486	0	0
0	0	0	0	0	0.010258416	0	0	0	-0.005143991
0	0	0	-0.002921186	0	0	0	0	0	0
0	0.04241127	0	-0.015317563	0	0.054977055	0	0	0	0
0	0.006956533	0	0.001171423	0	0	0	0	0	0
0	0.006450864	0	0	0	0	0	0	0	-0.012982253
0	0.007002225	0	0.001215069	0	0	0	0	0	0.051041998
0	0	0	-0.001564259	0	-0.064241564	0	0	0	0.011454167
0	0	0	-0.001135718	0	0	0	0	0	-0.006301164
0	0.007223405	0	0.001359367	0	0	0	0	0	-0.010142249
0	0	0	0	0	-0.010458418	0	-0.039212096	0	0
0	0.027961092	0	0	0	0	0	0	0	0

0	0.027790105	0	0	0	0	0	0	0	0.050421611
0	0	0	-0.017635439	0	0.0057082	0	-0.034859175	0	0
0	0	0	0.002342295	0	-0.013114474	0	0	0	0
0	0	0	0	0	0.015235723	0	-0.04647443	0	0
0	0	0	0.011245337	0	0	0	-0.047069131	0	0
0	0	0	-0.01449573	0	-0.018697231	0	-0.059855358	0	0
0	0	0	-0.0182738	0	0.004651275	0	0	0	0
0	0	0	0.022067222	0	-0.053770517	0	0	0	0
0	-1.57E-05	0	-0.056908023	0	0	0	0	0	0.006451536
0	0	0	0	0	0	0	0	0	0.046197516
0	-0.010934614	0	0	0	-0.026193976	0	0	0	0
0	-0.011116682	0	0	0	-0.026163302	0	0	0	0.076063883
0	0	0	0.016301236	0	-0.011515794	0	-0.065787455	0	0
0	-0.013327679	0	0	0	-0.029571191	0	0	0	0.015187146
0	0	0	0	0	-0.021914073	0	0	0	0.005985826
0	0	0	-0.02072795	0	0.032661252	0	-0.04640332	0	0
0	0	0	-0.000578126	0	0.015882557	0	-0.042743784	0	0
0	0.009819016	0	0.013074572	0	0	0	0	0	0
0	0.009718502	0	0.013114191	0	0	0	0	0	0.061717372
0	0.033611923	0	-0.011577367	0	0.012717514	0	-0.096643112	0	0
0	0	0	0.001479862	0	0.01446864	0	0	0	0
0	0	0	0.008789374	0	0	0	0	0	0.016054764
0	0.002880442	0	0	0	0	0	0	0	0.009717335
0	0	0	-0.042155105	0	0	0	0	0	0.018428271
0	0.001215614	0	-0.041989344	0	0	0	0	0	0
0	0.001068352	0	-0.041945448	0	0	0	0	0	0.059641281
0	0.02955141	0	-0.008043322	0	-0.049200706	0	0	0	0
0	0.007142556	0	0.010566038	0	0	0	0	0	0.002091513
0	0.041099277	0	0.052007784	0	-0.041498339	0	0	0	0
0	0.026459149	0	0	0	0	0	0	0	0.02015551
0	-0.008079223	0	-0.057809147	0	0	0	0	0	0.011945992
0	-0.036055941	0	-0.00566113	0	-0.0569535	0	0	0	0
0	-0.036270934	0	0	0	-0.061796639	0	0	0	0.04414188
0	-0.036412449	0	-0.005579845	0	-0.056962558	0	0	0	0.10430667
0	-0.046136332	0	-0.005748907	0	-0.071175911	0	0	0	0.042545154
0	0.018695097	0	0	0	0	0	0	0	0.026008625
0	0	0	-0.00494891	0	-0.049028857	0	0	0	0.009946761
0	-0.026814924	0	-0.044689517	0	0	0	0	0	0.036390597
0	0.019017558	0	0	0	0	0	0	0	0.012186784
0	0.031300297	0	0	0	0	0	-0.052062106	0	0.028789686
0	0.03154556	0	0	0	0	0	-0.051922724	0	0
0	0.053179391	0	0.018928115	0	0	0	-0.056215691	0	0
0	0.046863018	0	0	0	0	0	-0.03564461	0	0
0	0	0	0	0	-0.023062601	0	-0.037356055	0	0.057971276
0	0	0	0	0	-0.023351919	0	-0.036887489	0	0
0	0.045920286	0	0	0	0	0	-0.054986808	0	0
0	0.046171234	0	0	0	-0.00343579	0	-0.050001401	0	0

0	0.052983238	0	0.019373103	0	0	0	0	0	0
0	0.03288275	0	0	0	0	0	0	0	0
0	0.033055677	0	0	0	0	0	0	0	0.02498568
0	0.031639424	0	0	0	0	0	-0.037894837	0	0
0	0.031517574	0	0	0	0	0	-0.038235085	0	0.027030151
0	0	0	0	0	0	0	-0.038320601	0	0.057324651
0	0	0	0	0	0	0	-0.037599301	0	0
0	0	0	0.034204978	0	-0.06253696	0	0	0	0
0	0	0	0.034274501	0	-0.062536709	0	0	0	0.06389125
0	0.045069242	0	0	0	0	0	0	0	0
0	0.04877357	0	0	0	0.00921355	0	0	0	0
0	0	0	0	0	0	0	-0.053807804	0	0.056962457
0	0.044560132	0	0	0	0	0	-0.038172961	0	0
0	0	0	0.000234471	0	0	0	-0.05189745	0	0.058190598
0	0	0	0	0	0	0	-0.05186193	0	0.060980044
0	0	0	0	0	0	0	-0.053525357	0	0
0	0	0	4.07E-05	0	0	0	-0.051569296	0	0
0	0.048204304	0	0	0	0.008657099	0	-0.037476252	0	0
0	0.046303079	0	0.00025374	0	0	0	-0.036865418	0	0
0	0.015973883	0	0	0	-0.028983006	0	-0.053412457	0	0.043253466
0	0.016264587	0	0	0	-0.029137124	0	-0.053204266	0	0
0	0	0	0	0	-0.024693505	0	-0.03778898	0	0.05738002
0	0	0	0	0	-0.024732849	0	-0.037052673	0	0
0	0	0	0	0	-0.024693273	0	0	0	0
0	0	0	0	0	-0.024681252	0	0	0	0.056752945
0	0	0	0	0	-0.034964052	0	-0.048610242	0	0.057649827
0	0	0	0.000581344	0	-0.035010884	0	-0.047788209	0	0.058266308
0	0	0	0	0	-0.034515343	0	-0.047785081	0	0.05518316
0	0	0	0	0	-0.035305295	0	-0.048192898	0	0
0	0	0	0.000492769	0	-0.035280259	0	-0.047368293	0	0
0	0	0	-0.011073518	0	0	0	-0.038087933	0	0.054924816
0	0	0	-0.011154647	0	0	0	-0.037387588	0	0
0	0.04805951	0	0.04187941	0	-0.053519771	0	0	0	0
0	0.030214411	0	-0.004503137	0	0	0	0	0	0
0	0.028584747	0	0	0	-0.007470725	0	0	0	0
0	0.032203609	0	0	0	0	0	0	0	0
0	0.030405611	0	-0.004462174	0	0	0	0	0	0.026078392
0	0.028820875	0	0	0	-0.007297982	0	0	0	0.028481789
0	0.01240333	0	0	0	-0.03976619	0	0	0	0
0	0.032509985	0	0	0	0	0	0	0	-0.025410653
0	0.032398656	0	0	0	0	0	0	0	0.02378575
0	0.012386747	0	0	0	-0.039742665	0	0	0	0.046222178
0	0	0	0	0	0	0	-0.040331927	0	0.05462121
0	0	0	0	0	0	0	-0.039658009	0	0
0	0	0	0	0	0	0	0	0	0
0	0	0	0	0	0	0	-0.03932825	0	0.045704932
0	0	0	0	0	0	0	0	0	0.054041299

0	0.022725452	0	-0.014650172	0	0	0	-0.068594467	0	0.034482193
0	0.023091865	0	-0.014727541	0	0	0	-0.068486605	0	0
0	0	0	0	0	-0.042594571	0	0	0	0
0	0	0	0	0	0	0	0	0	0.010902759
0	0	0	-0.000304036	0	0	0	0	0	0
0	0	0	0	0	-0.043228087	0	0	0	0.012020218
0	0	0	0	0	-0.042536895	0	0	0	0.058336446
0	0	0	-0.000261432	0	0	0	0	0	0.055995106
0	0.046025173	0	0	0	0.005160154	0	0	0	0
0	0.044158179	0	-0.001997239	0	0	0	0	0	0
0	0.046705509	0	-0.00577978	0	0.011007439	0	0	0	0
0	0.029286179	0	0	0	0	0	-0.0752049	0	0.033118559
0	0.029736922	0	0	0	0	0	-0.075147612	0	0
0	0.028849326	0	0	0	0	0	-0.080067201	0	0.058657712
0	0	0	0	0	-0.026790457	0	0	0	0
0	0	0	-0.002387209	0	-0.02461112	0	0	0	0
0	0	0	0	0	-0.026937366	0	0	0	0.008063931
0	0	0	0	0	-0.026786755	0	0	0	0.055061786
0	0	0	-0.002356173	0	-0.024635003	0	0	0	0.055724498
0	0	0	-0.019112217	0	0	0	-0.062776762	0	0.055558168
0	0.0411138	0	-0.002501856	0	-0.018307437	0	-0.062259951	0	0
0	0.0410643	0	0	0	-0.020880268	0	-0.062760651	0	0
0	0	0	-0.019395657	0	0	0	-0.064085092	0	0.06745083
0	0	0	-0.019352271	0	0	0	-0.062486025	0	0
0	0	0	-0.012961569	0	0	0	0	0	0
0	0	0	-0.012944119	0	0	0	0	0	0.051980213
0	0	0	-0.012903956	0	0	0	0	0	0.00492828
0	0.04252376	0	-0.012161306	0	0	0	-0.070357072	0	0
0	0	0	0	0	0	0	0	0	0.005664376
0	0.040036924	0	0	0	-0.031121137	0	0	0	0
0	0	0	0	0	0	0	-0.074926726	0	0.085373465
0	0.031597639	0	0	0	0	0	0	0	0
0	0.0316389	0	0	0	0	0	0	0	0.033516143
0	0	0	0.001930158	0	-0.064238799	0	0	0	0
0	0	0	0	0	-0.06253734	0	0	0	0.016811672
0	0.000181732	0	0	0	-0.062530235	0	0	0	0
0	0	0	0.001973922	0	-0.064202322	0	0	0	0.061873524
0	3.89E-05	0	0	0	-0.062486864	0	0	0	0.06148993
0	-0.003985213	0	0.001966778	0	-0.071726984	0	0	0	0
0	-0.004196276	0	0	0	-0.070045452	0	0	0	0.015857657
0	0	0	0.002149777	0	-0.071527057	0	0	0	0.012224967
0	-0.004134784	0	0.002004183	0	-0.071736805	0	0	0	0.06226636
0	0.036249899	0	0.013703066	0	0	0	-0.049238436	0	0.026440318
0	0.036485852	0	0.013707991	0	0	0	-0.049113034	0	0
0	0	0	0	0	-0.026930802	0	-0.044491075	0	0
0	0.030452041	0	0	0	0	0	-0.034702625	0	0.028685094
0	0.030598433	0	0	0	0	0	-0.034362611	0	0

0	0.024378377	0	0	0	-0.011639149	0	-0.038967236	0	0.034998355
0	0.024553501	0	0	0	-0.011737759	0	-0.038734735	0	0
0	0.030841172	0	0	0	0	0	0	0	-0.01363696
0	0.03025903	0	-0.001006807	0	0	0	0	0	0
0	0.030367167	0	-0.000961658	0	0	0	0	0	0.029482471
0	0.052497501	0	0.019728519	0	0	0	-0.035026429	0	0
0	0.05102585	0	0.029450119	0	-0.020392543	0	-0.039779645	0	0
0	0.029617301	0	0	0	0	0	-0.048965454	0	0.028591634
0	0.029856375	0	0	0	0	0	-0.048821682	0	0
0	0	0	0	0	0	0	-0.038146673	0	0
0	0.051439389	0	0.005864651	0	0	0	0	0	0
0	0	0	0.023115067	0	-0.037792824	0	-0.02724322	0	0.061861378
0	0.029845809	0	0	0	0	0	-0.046316436	0	0.028236083
0	0	0	0.023137359	0	-0.038116996	0	-0.0267394	0	0
0	0.043725318	0	0	0	0	0	-0.035070685	0	0
0	0.05041984	0	0.017211945	0	0	0	-0.053769766	0	0
0	0	0	0	0	-0.023697653	0	-0.035812142	0	0.055046023
0	0	0	-0.010552553	0	0	0	0	0	0
0	0	0	-0.010477868	0	0	0	0	0	0.060809444
0	0	0	0	0	-0.023975769	0	-0.035368027	0	0
0	0.021420354	0	0.036724715	0	-0.05430201	0	0	0	0
0	0.048231214	0	0	0	0.00947517	0	-0.033872669	0	0
0	0.02139314	0	0.036741861	0	-0.054293465	0	0	0	0.044148632
0	0	0	0	0	-0.024064575	0	-0.034377147	0	0.05797426
0	0	0	0	0	-0.022141616	0	-0.032166033	0	0.043693966
0	0	0	0	0	-0.02410234	0	-0.033645735	0	0
0	0	0	0	0	0	0	0	0	0.023685406
0	0.043275963	0	0	0	-0.005269067	0	-0.047535675	0	0
0	0	0	0.007406335	0	-0.028588453	0	-0.035441386	0	0
0	0.037865987	0	0.013987999	0	0	0	0	0	0
0	0	0	-0.016634035	0	0	0	-0.037056128	0	0
0	0	0	0	0	-0.020070568	0	-0.033417303	0	0
0	0.038056573	0	0.014049419	0	0	0	0	0	0.022786503
0	0	0	-0.008993264	0	0	0	-0.057763846	0	0
0	0.031052497	0	0	0	0	0	0	0	0
0	0.031213988	0	0	0	0	0	0	0	0.024460664
0	0	0	0	0	-0.021404219	0	-0.037493446	0	0
0	0	0	0	0	-0.021355965	0	0	0	0
0	0.049722135	0	0.017571738	0	0	0	0	0	0
0	0.027721657	0	0	0	-0.007050891	0	0	0	0
0	0	0	0.032393457	0	-0.060029703	0	0	0	0
0	0.027886417	0	0	0	-0.006926794	0	0	0	0.030131477
0	0.031390141	0	0	0	0	0	0	0	-0.019320414
0	0	0	0.032442553	0	-0.059995079	0	0	0	0.061735874
0	0	0	0.02860031	0	-0.05246268	0	0	0	0
0	0	0	-0.008013116	0	0	0	-0.034086661	0	0
0	0	0	0	0	0	0	-0.037259983	0	0.054148249

0	0	0	0.030752006	0	-0.056935076	0	0	0	0.018315704
0	0	0	0.028720957	0	-0.052568356	0	0	0	0.062564367
0	0	0	0	0	0	0	-0.036588041	0	0
0	0.050821589	0	0.024502616	0	-0.013264836	0	0	0	0
0	0	0	-0.012219934	0	0	0	0	0	0.01396093
0	0	0	0	0	0	0	-0.03581687	0	0.04717257
0	0	0	0.000673325	0	0	0	-0.036005085	0	0.057452379
0	0	0	0.000536517	0	0	0	-0.035266513	0	0
0	0	0	-0.007774087	0	0	0	0	0	0
0	0.029721805	0	0	0	0	0	-0.036592974	0	0
0	0.029599505	0	0	0	0	0	-0.036922947	0	0.026491469
0	0	0	-0.000421175	0	0	0	-0.049150815	0	0.056050717
0	0	0	0	0	0	0	-0.04925423	0	0.058438763
0	0.045401215	0	0	0	0.006044075	0	0	0	0
0	0.025165213	0	0	0	-0.009258659	0	-0.035506651	0	0.033227088
0	0.027356459	0	-0.005280613	0	0	0	-0.035391103	0	0.030063398
0	0.027508952	0	-0.005277193	0	0	0	-0.035013364	0	0
0	0.025422258	0	0	0	-0.009107264	0	-0.035093847	0	0
0	0	0	-0.000609351	0	0	0	-0.048832211	0	0
0	0	0	-0.000465133	0	0	0	-0.046087201	0	0.058019803
0	0.029796559	0	0	0	0	0	-0.035782219	0	0.015601525
0	0	0	-0.013345661	0	-0.009286435	0	-0.033736172	0	0
0	0	0	-0.010704198	0	-0.029291331	0	-0.054321225	0	0
0	0	0	0	0	-0.025667362	0	0	0	0
0	0	0	0	0	-0.025663745	0	0	0	0.054598418
0	0	0	0	0	-0.026031014	0	0	0	0.012820321
0	0	0	0.005028063	0	-0.026641634	0	0	0	0
0	0.014157923	0	0	0	-0.029452577	0	-0.048852517	0	0.044186297
0	0.012937147	0	0	0	-0.035821886	0	0	0	0
0	0.012771047	0	0	0	-0.03608614	0	0	0	0.001980076
0	0.014546697	0	-0.001116505	0	-0.027805003	0	-0.048103237	0	0.044204431
0	0.014580415	0	0	0	-0.028779869	0	-0.048074628	0	0.043749595
0	0.014448971	0	0	0	-0.029604066	0	-0.048621678	0	0
0	0.012910548	0	0	0	-0.035789437	0	0	0	0.045755105
0	0.014823494	0	-0.001181771	0	-0.027910095	0	-0.047887149	0	0
0	0.044592758	0	0	0	0.004987781	0	-0.036351896	0	0
0	0	0	0	0	-0.026023145	0	-0.036339614	0	0.055123855
0	0.042581739	0	-0.002597712	0	0	0	-0.035730372	0	0
0	0	0	0.021817524	0	-0.061075842	0	0	0	0
0	0	0	0	0	-0.026053705	0	-0.035657305	0	0
0	0	0	-0.01402927	0	-0.010216492	0	0	0	0
0	0	0	-0.002670319	0	-0.023791823	0	-0.035201866	0	0.056890256
0	0	0	0	0	-0.026384658	0	-0.035200401	0	0.044474481
0	0	0	-0.002788024	0	-0.023713481	0	-0.034485318	0	0
0	0.046296494	0	0.03930027	0	-0.050611594	0	0	0	0
0	0.046013899	0	-0.006540757	0	0.011816391	0	-0.034535458	0	0
0	0	0	0	0	-0.040118346	0	0	0	0.01389543

0	0	0	-0.001494076	0	-0.033476499	0	-0.044736193	0	0.056638315
0	0	0	0	0	-0.034814731	0	-0.044657784	0	0.053290533
0	0	0	-0.001502493	0	-0.032514345	0	-0.043001974	0	0.053036252
0	0	0	-0.001577406	0	-0.033737676	0	-0.044328916	0	0
0	0	0	-0.012873834	0	0	0	-0.036615301	0	0.05208664
0	0	0	-0.012950234	0	0	0	-0.035963535	0	0
0	0	0	-0.000937159	0	0	0	0	0	0
0	0	0	0	0	0	0	0	0	0.008744203
0	0	0	-0.012839718	0	0	0	-0.035406981	0	0.042237451
0	0	0	-0.000899299	0	0	0	0	0	0.05349116
0	0	0	-0.001236897	0	0	0	0	0	0.012197793
0	0.027191603	0	-0.007045563	0	0	0	0	0	0
0	0.024791931	0	0	0	-0.011400816	0	0	0	0
0	0.027355092	0	-0.007008979	0	0	0	0	0	0.026957556
0	0.0249832	0	-0.004618393	0	-0.006972849	0	0	0	0
0	0.024969743	0	0	0	-0.011268718	0	0	0	0.030734121
0	0.027543821	0	-0.006909397	0	0	0	0	0	-0.018787528
0	0.0210519	0	-0.015381594	0	0	0	-0.061350897	0	0.036219091
0	0.025150768	0	-0.004635182	0	-0.006830941	0	0	0	0.031237563
0	0.020834571	0	-0.015643736	0	0	0	-0.062370038	0	0.048446596
0	0.025013772	0	0	0	-0.011449828	0	0	0	-0.014581995
0	0.021403799	0	-0.01546283	0	0	0	-0.061219391	0	0
0	0.030632364	0	0	0	0	0	0	0	-0.022870595
0	0	0	0	0	0	0	-0.038054392	0	0.043719519
0	0.033801096	0	-0.003150219	0	-0.05655466	0	0	0	0
0	0.030869777	0	0.017703182	0	0	0	-0.052818105	0	0.033549009
0	0.031160942	0	0.017710123	0	0	0	-0.052654045	0	0
0	0.023047989	0	0	0	0	0	-0.037912799	0	0.037015223
0	0.023253167	0	0	0	0	0	-0.037444161	0	0
0	0	0	0.024665783	0	-0.030498568	0	-0.034916004	0	0.06353379
0	0	0	0.024680256	0	-0.030823442	0	-0.034399957	0	0
0	0.043398664	0	-0.007873272	0	0.009585238	0	0	0	0
0	0	0	-0.004293807	0	-0.024065163	0	0	0	0
0	0.022288651	0	0	0	0	0	-0.05217826	0	0.036465133
0	0.022582315	0	0	0	0	0	-0.051993351	0	0
0	0	0	-0.004258804	0	-0.024095888	0	0	0	0.053869157
0	0	0	0	0	-0.028258657	0	0	0	0.008733602
0	0	0	-0.020037608	0	0	0	-0.058225572	0	0.064363292
0	0	0	-0.004019932	0	-0.024546916	0	0	0	0.010160698
0	0.027662043	0	0	0	0	0	-0.071181409	0	0.056190247
0	0.020138866	0	0	0	-0.004979194	0	-0.045831119	0	0.04037163
0	0.02037484	0	0	0	-0.005082478	0	-0.045567456	0	0
0	0.022526912	0	0	0	0	0	-0.048974991	0	0.034881481
0	0.0512904	0	0.025016608	0	0	0	-0.038319177	0	0
0	0.039178999	0	-0.004692627	0	-0.01728666	0	-0.058401862	0	0
0	0	0	-0.01458498	0	0	0	0	0	0.004794172
0	0	0	0	0	-0.015368544	0	-0.043658477	0	0.056478793

0	0.048857499	0	0.022234832	0	0	0	-0.058397373	0	0
0	0	0	0	0	-0.015650904	0	-0.043207445	0	0
0	0.050116432	0	0.030069629	0	-0.012502144	0	-0.047584431	0	0
0	0	0	0	0	0	0	-0.040230427	0	0.055167493
0	0	0	0	0	0	0	-0.039550161	0	0
0	0	0	0	0	-0.011659746	0	-0.038052015	0	0.058982756
0	0	0	0	0	-0.013785157	0	-0.040149544	0	0.048488117
0	0	0	0	0	-0.011696735	0	-0.037308273	0	0
0	0	0	0.006701025	0	0	0	-0.039457663	0	0.059812039
0	0.048286	0	0	0	0.02233722	0	-0.037600183	0	0
0	0.040372089	0	0	0	0	0	-0.038174413	0	0
0	0	0	0.006569459	0	0	0	-0.038692243	0	0
0	0	0	0	0	0	0	-0.038661071	0	0.046626408
0	0	0	0.005469154	0	0	0	-0.053571762	0	0.058155379
0	0	0	0.005276022	0	0	0	-0.05324149	0	0
0	0	0	0	0	0	0	-0.052396383	0	0.057849473
0	0.032422767	0	0.017849445	0	0	0	0	0	0
0	0.03258	0	0.017907785	0	0	0	0	0	0.029857204
0	0	0	0.005453904	0	0	0	-0.050109225	0	0.060193572
0	0.023728834	0	0	0	0	0	0	0	0
0	0.030742413	0	0.001300016	0	0	0	0	0	0
0	0.03018278	0	0	0	0	0	0	0	-0.005991832
0	0.030787101	0	0.001334139	0	0	0	0	0	0.033314506
0	0.023853661	0	0	0	0	0	0	0	0.03233898
0	0.042120325	0	0	0	0.002737594	0	-0.055474703	0	0
0	0.025996577	0	0	0	0.004181052	0	0	0	0
0	0.010240846	0	-0.04211453	0	0	0	0	0	0
0	0.015542922	0	0.038659162	0	-0.05268205	0	0	0	0
0	0.024017806	0	0	0	0	0	0	0	-0.015139588
0	0.048065495	0	0.022589213	0	0	0	0	0	0
0	0.010151157	0	-0.042076991	0	0	0	0	0	0.050771809
0	0.026176091	0	0	0	0.004316316	0	0	0	0.032682618
0	0.015479339	0	0.03867709	0	-0.052678339	0	0	0	0.051422038
0	0	0	0.026498688	0	-0.038222764	0	0	0	0
0	0	0	0.026607474	0	-0.038315775	0	0	0	0.063050055
0	0	0	-0.04376258	0	0	0	0	0	0.019164061
0	0.022037552	0	0	0	0	0	-0.040367974	0	0.034630335
0	0.022218175	0	0	0	0	0	-0.039936314	0	0
0	0	0	0.035544527	0	-0.05700978	0	0	0	0
0	0	0	0.035591926	0	-0.056971459	0	0	0	0.064084792
0	0	0	0.034327495	0	-0.054675876	0	0	0	0.019575359
0	0.050418646	0	0.021975943	0	0.001507826	0	0	0	0
0	0.020766722	0	-0.003148081	0	0	0	-0.038925331	0	0.03796718
0	0.020982672	0	-0.003138608	0	0	0	-0.03844476	0	0
0	0.023324762	0	0	0	0.002194455	0	-0.039363571	0	0.036000946
0	0.022237362	0	0	0	0	0	-0.03905253	0	0.020279012
0	0.023608711	0	0	0	0.002377331	0	-0.038924979	0	0

0	0.029204946	0	-0.00079134	0	0	0	0	0	-0.01037241
0	0.045060413	0	0	0	0.01840724	0	0	0	0
0	-0.000436345	0	0	0	-0.060334555	0	0	0	0.018378353
0	0	0	-0.000139858	0	-0.060122134	0	0	0	0.017961085
0	-0.000292664	0	-0.000182754	0	-0.060180583	0	0	0	0
0	0	0	0	0	-0.013443173	0	0	0	0
0	0.02385556	0	0.028602304	0	0	0	-0.047298171	0	0.043767815
0	0.024126339	0	0.028643646	0	0	0	-0.046754496	0	0
0	-0.000447585	0	-0.000142775	0	-0.060173805	0	0	0	0.06087993
0	0	0	0	0	-0.013436658	0	0	0	0.055122372
0	0	0	0	0	-0.013803281	0	0	0	0.009427274
0	0	0	0.004930402	0	0	0	0	0	0
0	-0.003614193	0	-0.00011079	0	-0.065899365	0	0	0	0.017654642
0	0	0	0.004966915	0	0	0	0	0	0.055401611
0	0	0	0	0	0	0	0	0	0.005135185
0	0	0	-0.009025443	0	0	0	-0.04014658	0	0.053752371
0	0	0	0	0	-0.01349002	0	-0.040311483	0	0.055717371
0	0	0	-0.009104344	0	0	0	-0.039475419	0	0
0	0	0	0	0	-0.013519058	0	-0.039616398	0	0
0	0.018607851	0	0.033032345	0	-0.011934298	0	-0.057088436	0	0.050543424
0	0	0	0.004619154	0	0	0	0	0	0.0120431
0	0.021422817	0	0.026036957	0	0	0	-0.066278133	0	0.044442684
0	0	0	-0.005136152	0	-0.008909975	0	-0.038869687	0	0.057418922
0	0.018935282	0	0.033112553	0	-0.012043073	0	-0.056818515	0	0
0	0	0	-0.005252262	0	-0.008834781	0	-0.038138923	0	0
0	0.021798356	0	0.026074276	0	0	0	-0.066091769	0	0
0	0	0	0	0	-0.01386829	0	-0.03901231	0	0.045226469
0	0	0	-0.008971881	0	0	0	-0.038823737	0	0.042941597
0	0.044236154	0	0	0	0.017674103	0	-0.040406	0	0
0	0	0	0.017919371	0	0	0	-0.047995295	0	0.059972834
0	0	0	0	0	0	0	-0.041101026	0	0.042834928
0	0	0	0.017798443	0	0	0	-0.047247889	0	0
0	0.021683734	0	0.026197227	0	0	0	-0.062830762	0	0.04817985
0	0	0	0.011686525	0	-0.034513874	0	-0.038261741	0	0
0	0	0	0.030966649	0	-0.021238001	0	-0.055504027	0	0.064281448
0	0	0	0	0	-0.020524578	0	-0.034557075	0	0
0	0	0	0.030986017	0	-0.021535439	0	-0.055046426	0	0
0	0	0	-0.010269336	0	0	0	0	0	0.020901098
0	0.025396766	0	0	0	0.028662845	0	-0.04597483	0	0.036409806
0	0.02566277	0	0	0	0.028819216	0	-0.045524019	0	0
0	0.045681645	0	-0.00925244	0	0.027089525	0	-0.038234941	0	0
0	0	0	0.025040561	0	-0.011549996	0	-0.046875506	0	0.065773589
0	0	0	0.024721893	0	-0.011294863	0	-0.045986701	0	0
0	0.009088416	0	-0.002924736	0	-0.021190963	0	-0.056029667	0	0.0504501
0	0.008803483	0	0	0	-0.024323096	0	-0.056635147	0	0.050738552
0	0.040072499	0	0.000303476	0	0	0	-0.039262994	0	0
0	0.009175291	0	0	0	-0.023712095	0	-0.055997144	0	0.053714265

0	0	0	0.02880612	0	-0.018050976	0	-0.051761236	0	0.059341138
0	0.009430656	0	-0.003001605	0	-0.02129801	0	-0.055792563	0	0
0	0	0	0.017558499	0	0	0	-0.046282778	0	0.051430506
0	0.009160597	0	0	0	-0.024485418	0	-0.056383162	0	0
0	0	0	-0.00313309	0	-0.024952297	0	-0.054075677	0	0.058071631
0	0	0	0	0	-0.027698683	0	-0.053995985	0	0.059664338
0	0	0	-0.003137942	0	-0.02420284	0	-0.052806957	0	0.059094429
0	0	0	-0.003227469	0	-0.025212217	0	-0.053662665	0	0
0	0	0	0	0	0.01108352	0	-0.047238432	0	0.056773315
0	0	0	-0.006752841	0	0	0	-0.035330286	0	0
0	0	0	0	0	0.011055807	0	-0.046541313	0	0
0	0.04582992	0	0	0	0.044162987	0	-0.047525309	0	0
0	0	0	0.016335535	0	0	0	-0.063559779	0	0.065650513
0	0.008189838	0	0	0	0	0	-0.047632464	0	0.050014273
0	0.008457852	0	0	0	0	0	-0.047019818	0	0
0	0.046287785	0	0.037025617	0	0	0	-0.049390808	0	0
0	0.006879326	0	0	0	-0.032791929	0	0	0	0
0	0.006455661	0	0	0	-0.033463134	0	0	0	0.005796138
0	0.00682066	0	0	0	-0.032755061	0	0	0	0.0531075
0	0	0	0	0	0	0	-0.047237043	0	0.040778431
0	0	0	0	0	-0.035106526	0	0	0	0.012309576
0	0.0141565	0	-0.01384189	0	0	0	-0.066216279	0	0.044182767
0	0.014022626	0	-0.014000477	0	0	0	-0.066908607	0	0.056803242
0	0.051800606	0	0.019753737	0	0.030633304	0	-0.046376705	0	0
0	0.014562698	0	-0.013942747	0	0	0	-0.066051597	0	0
0	0.020565892	0	-0.005131877	0	0	0	0	0	0
0	0	0	0	0	0.010834739	0	-0.045553145	0	0.045841833
0	0.020695358	0	-0.005099749	0	0	0	0	0	0.034506331
0	0.02312299	0	-0.007244473	0	0.006778935	0	0	0	0
0	0.023092885	0	0	0	0	0	0	0	-0.019070631
0	0.022824346	0	0	0	-0.000220338	0	0	0	0
0	0.020893563	0	-0.00497742	0	0	0	0	0	-0.015052683
0	0.008264333	0	0	0	0	0	-0.045929825	0	0.036270486
0	0.023311436	0	-0.007271068	0	0.006944977	0	0	0	0.033399326
0	0.023024085	0	0	0	-7.00E-05	0	0	0	0.033152506
0	0.023068148	0	0	0	-0.000259498	0	0	0	-0.016218377
0	0.011221829	0	0	0	0.006713288	0	-0.06532895	0	0.049758783
0	0.011578679	0	0	0	0.00667982	0	-0.065094226	0	0
0	0.007398421	0	0	0	0	0	-0.061907996	0	0.05048873
0	0	0	-0.017005896	0	0	0	-0.064082029	0	0.067487905
0	0.01227168	0	0	0	0.008284372	0	-0.06305991	0	0.050804765
0	0	0	0	0	0.001080909	0	-0.06191246	0	0.058661169
0	0	0	0.007814501	0	-0.029759425	0	0	0	0
0	0.020120809	0	0	0	0	0	-0.075105672	0	0.06385553
0	0.044716545	0	0.041287826	0	-0.047382907	0	0	0	0
0	0	0	-0.014526893	0	-0.009063922	0	-0.034556088	0	0
0	0	0	-0.006979577	0	-0.009154852	0	0	0	0

0	0	0	0.005484383	0	-0.026581883	0	-0.031102959	0	0
0	0	0	-0.006953737	0	-0.009173172	0	0	0	0.053879045
0	0	0	-0.010914481	0	0	0	0	0	0.001884932
0	0	0	0	0	-0.015911119	0	0	0	0.004667087
0	0	0	-0.006667538	0	-0.009678558	0	0	0	0.00598755
0	0.042657293	0	-0.01076337	0	0.024524361	0	0	0	0
0	0.022517645	0	0.027561734	0	0	0	0	0	0
0	0.022653246	0	0.027652845	0	0	0	0	0	0.040264662
0	0.046339099	0	0.035378194	0	-0.001505567	0	-0.071367772	0	0
0	0.025190681	0	0.023113754	0	0.008404968	0	0	0	0
0	0.022601962	0	0.027306202	0	0	0	0	0	-0.015797449
0	0.025390379	0	0.023092102	0	0.008626315	0	0	0	0.039795227
0	0.022085448	0	0	0	0.025235802	0	-0.048853756	0	0.035801278
0	0.022335654	0	0	0	0.02537969	0	-0.048414569	0	0
0	0.024753148	0	0	0	0.028963589	0	0	0	0
0	0.02244151	0	-0.00922862	0	0.033793087	0	-0.046912005	0	0.036896549
0	0.022702819	0	-0.0092952	0	0.034005978	0	-0.046449506	0	0
0	0.025081722	0	0	0	0.02925215	0	0	0	0.032321822
0	0.022070975	0	0	0	0.025051717	0	-0.047394519	0	0.022437555
0	-0.000178044	0	-0.059502695	0	0	0	0	0	0.004299704
0	0	0	0.022801492	0	-0.00880072	0	0	0	0
0	0.025035736	0	0	0	0.028945409	0	0	0	-0.024957013
0	0	0	0.022853961	0	-0.008773605	0	0	0	0.060874066
0	0	0	0.017121461	0	0	0	0	0	0.000755003
0	0	0	-0.009015205	0	0.01806328	0	-0.047937924	0	0.055176401
0	0.0372551	0	-0.007124682	0	-0.008729282	0	-0.067729052	0	0
0	0	0	-0.009123917	0	0.018122963	0	-0.047244923	0	0
0	0	0	0.022520141	0	-0.008747451	0	0	0	0.006469058
0	0.047822545	0	0.018520745	0	0.029683003	0	0	0	0
0	0	0	0	0	0.009773832	0	-0.048389245	0	0.04146034
0	0.008437903	0	0.002688657	0	0	0	-0.049102387	0	0.050292414
0	0.008723381	0	0.002712795	0	0	0	-0.048488484	0	0
0	0.007097562	0	0	0	0	0	-0.048661719	0	0.029945301
0	0	0	-0.008616536	0	0.017522077	0	-0.046488963	0	0.042530511
0	0.043117077	0	-0.013104697	0	0.052910894	0	-0.047999819	0	0
0	0	0	-0.000112252	0	0	0	-0.048533384	0	0.040789979
0	0.024899557	0	0.004245644	0	0	0	0	0	0
0	0.024905406	0	0.004274465	0	0	0	0	0	0.040704933
0	0.022904045	0	0	0	0	0	0	0	-0.001506264
0	0	0	0	0	0.011579789	0	0	0	-0.003778166
0	0.00862917	0	0.002875703	0	0	0	-0.047681757	0	0.036079163
0	0.007657357	0	0	0	0	0	0	0	-0.013033632
0	0.0233885	0	0.002087321	0	0	0	0	0	-0.006408081
0	0	0	0	0	-0.008079541	0	-0.038373562	0	0
0	0	0	-0.00093409	0	0	0	-0.038837569	0	0
0	0	0	-0.005261516	0	0	0	0	0	0.019778816
0	0	0	0.012791216	0	-0.026955762	0	-0.046022917	0	0

0	0.009034359	0	-0.041509525	0	0	0	0	0	0.011723341
0	0.021676433	0	-0.011851767	0	0.036304418	0	0	0	0
0	0.022013475	0	-0.011906234	0	0.036649985	0	0	0	0.033101466
0	0.022049046	0	-0.011571884	0	0.036162721	0	0	0	-0.026932723
0	0.021569395	0	0	0	0.025342724	0	0	0	-0.027412983
0	-0.008358886	0	0	0	-0.059925107	0	0	0	0.026294457
0	-0.008214657	0	-0.002057893	0	-0.058180199	0	0	0	0
0	-0.008417443	0	-0.002004042	0	-0.058181973	0	0	0	0.069992246
0	-0.002397322	0	-0.005518847	0	-0.007369513	0	-0.078338153	0	0.076458203
0	-0.002331098	0	-0.005518463	0	-0.007297878	0	-0.078056703	0	0.064325979
0	0	0	-0.005481705	0	-0.006237749	0	-0.07904917	0	0.07503119
0	0	0	-0.001755656	0	-0.056633071	0	0	0	0.016681849
0	-0.002203289	0	0	0	-0.011949635	0	-0.078156515	0	0.077452378
0	-0.000155221	0	-0.00923337	0	0	0	-0.080986266	0	0.076959806
0	-0.001864476	0	-0.005645705	0	-0.007304063	0	-0.077872635	0	0
0	0	0	-0.017050636	0	0.005803357	0	-0.038341204	0	0
0	0	0	0.04714153	0	-0.052303768	0	0	0	0.017012107
0	-5.95E-05	0	0.04708507	0	-0.052297153	0	0	0	0
0	0	0	-0.011294637	0	0.020614659	0	0	0	-0.007669179
0	-0.000684227	0	0.047633036	0	-0.053558479	0	0	0	0.014017865
0	-0.000218667	0	0.047103541	0	-0.052275017	0	0	0	0.072348066
0	0	0	0.00327483	0	-0.011965947	0	-0.034678057	0	0
0	0	0	0.005668031	0	-0.01550467	0	0	0	0
0	-0.012195631	0	-0.001981036	0	-0.065004882	0	0	0	0.023267266
0	0.007073322	0	0.00119161	0	0	0	0	0	-0.013310456
0	0.027807301	0	0	0	0	0	0	0	0.018724935
0	0	0	0.003712679	0	0.011968907	0	-0.046640223	0	0
0	-0.011077572	0	0	0	-0.026224225	0	0	0	0.016399203
0	0.009757375	0	0.013063792	0	0	0	0	0	0.007317178
0	0.001078872	0	-0.041976956	0	0	0	0	0	0.017469819
0	-0.036366621	0	-0.005590925	0	-0.05700724	0	0	0	0.043061183
0	0.053928192	0	0.021603171	0	0	0	-0.037641654	0	0
0	0.032231716	0	0	0	0	0	-0.036705935	0	0
0	0.032109677	0	0	0	0	0	-0.037047294	0	0.027116558
0	0.038447636	0	0.015275369	0	0	0	-0.055344312	0	0.024860793
0	0.038695862	0	0.015285786	0	0	0	-0.055226477	0	0
0	0.052201609	0	0.032183378	0	-0.022748943	0	-0.044720094	0	0
0	0.025615247	0	0	0	-0.01210697	0	-0.044415774	0	0.03402948
0	0.02580493	0	0	0	-0.012194786	0	-0.044194424	0	0
0	0	0	0.025438547	0	-0.040375412	0	-0.031862804	0	0.06245789
0	0	0	0.025456856	0	-0.040699437	0	-0.031353888	0	0
0	0.03116469	0	0	0	0	0	-0.053577087	0	0.02799983
0	0.044828619	0	0	0	0	0	-0.037257851	0	0
0	0.03141187	0	0	0	0	0	-0.053437249	0	0
0	0.031299625	0	0	0	0	0	-0.052065891	0	0.029551367
0	0.04960004	0	0	0	0.011893779	0	-0.036495791	0	0
0	0.05215351	0	0.018669602	0	0	0	-0.0586795	0	0

0	0	0	0	0	-0.024312769	0	-0.040048623	0	0.055858088
0	0	0	0	0	-0.024593102	0	-0.039600504	0	0
0	0.040499623	0	0.016022916	0	0	0	0	0	0
0	0.040740135	0	0.016097717	0	0	0	0	0	0.020526063
0	0	0	0	0	-0.023123107	0	-0.037257528	0	0.045804785
0	0	0	0	0	-0.022568376	0	-0.036838701	0	0.057924658
0	0.044498228	0	0	0	-0.005332803	0	-0.052094327	0	0
0	0	0	0	0	-0.022606553	0	-0.036107595	0	0
0	0.021879076	0	0.04036949	0	-0.059895518	0	0	0	0
0	0.021863812	0	0.040388325	0	-0.059891127	0	0	0	0.044516739
0	0.051394852	0	0.019283211	0	0	0	0	0	0
0	0.052250825	0	0.025481519	0	-0.011869582	0	0	0	0
0	0.032741828	0	0	0	0	0	0	0	0
0	0.03293571	0	0	0	0	0	0	0	0.023264084
0	0.03125287	0	0	0	-0.003272519	0	0	0	0
0	0.033087094	0	0	0	0	0	0	0	-0.024165831
0	0.031466725	0	0	0	0	0	-0.038713613	0	0
0	0.031362249	0	0	0	0	0	-0.039028066	0	0.02529777
0	0.031516731	0	0	0	-0.003087169	0	0	0	0.026513603
0	0.029084636	0	0	0	-0.004868379	0	-0.037985521	0	0.029454812
0	0.029281471	0	0	0	-0.004757049	0	-0.037632785	0	0
0	0.030403971	0	-0.002685792	0	0	0	-0.037600354	0	0
0	0.030276847	0	-0.002687265	0	0	0	-0.037949835	0	0.027768347
0	0.031553298	0	0	0	0	0	-0.038016417	0	0.011136961
0	0	0	0	0	0	0	-0.039441209	0	0.054591626
0	0	0	0	0	0	0	-0.038767531	0	0
0	0	0	0.001764243	0	0	0	-0.038492142	0	0.057632729
0	0	0	0	0	0	0	-0.038185486	0	0.046342365
0	0	0	0.028998497	0	-0.051492606	0	0	0	0
0	0	0	0.001636599	0	0	0	-0.037754444	0	0
0	0	0	0.029108422	0	-0.051587355	0	0	0	0.062513687
0	0	0	0.035105108	0	-0.064193968	0	0	0	0
0	0.047067445	0	0	0	0.00840602	0	0	0	0
0	0	0	0.034253708	0	-0.062551953	0	0	0	0.019494162
0	0	0	0.035148548	0	-0.064154924	0	0	0	0.06316176
0	0.046411308	0	0	0	0.007821299	0	-0.038702812	0	0
0	0	0	0.000260863	0	0	0	-0.05386378	0	0.057009455
0	0	0	0	0	0	0	-0.053832082	0	0.061100858
0	0.044507219	0	-0.000162909	0	0	0	-0.038154979	0	0
0	0	0	0.000240728	0	0	0	-0.051913311	0	0.061088927
0	0	0	6.91E-05	0	0	0	-0.053540083	0	0
0	0.047721593	0	-0.004480378	0	0.012658575	0	-0.037233666	0	0
0	0.016005346	0	0.00101142	0	-0.029864673	0	-0.05339166	0	0.043405896
0	0.015977902	0	0	0	-0.028973003	0	-0.053424483	0	0.044712263
0	0.016010787	0	0	0	-0.028924143	0	-0.053345913	0	0.043269669
0	0	0	0	0	-0.024399021	0	-0.03859786	0	0.055509694
0	0.016300495	0	0.000951565	0	-0.029962639	0	-0.053188832	0	0

0	0.016307897	0	0	0	-0.029068174	0	-0.053125101	0	0
0	0	0	0	0	-0.024430633	0	-0.037902118	0	0
0	0	0	0	0	-0.024705134	0	-0.037629047	0	0.044453982
0	0	0	-0.000904919	0	-0.023815357	0	-0.037736103	0	0.057211032
0	0	0	0	0	-0.024412038	0	0	0	0
0	0	0	-0.001021954	0	-0.023738586	0	-0.037007605	0	0
0	0	0	0	0	-0.024407741	0	0	0	0.054955018
0	0	0	0	0	-0.024694729	0	0	0	0.009744094
0	0	0	0.000582559	0	-0.035478203	0	-0.048592052	0	0.057754442
0	0	0	0	0	-0.034977717	0	-0.048590276	0	0.055168952
0	0	0	0.000581102	0	-0.035028164	0	-0.047766729	0	0.055290564
0	0	0	0.000495115	0	-0.0357428	0	-0.0481768	0	0
0	0	0	-0.011116266	0	0	0	-0.038963017	0	0.052816637
0	0	0	-0.011193636	0	0	0	-0.038301661	0	0
0	0	0	-0.011084636	0	0	0	-0.037927912	0	0.042026011
0	0.030082783	0	-0.004583571	0	0	0	0	0	0
0	0.04764182	0	0.04228925	0	-0.054524186	0	0	0	0
0	0.028555141	0	0	0	-0.007351053	0	0	0	0
0	0.028683242	0	-0.00302192	0	-0.004461016	0	0	0	0
0	0.030436305	0	-0.004466838	0	0	0	0	0	-0.024241616
0	0.028863812	0	0	0	-0.007276714	0	0	0	-0.021590869
0	0.030288443	0	-0.004539989	0	0	0	0	0	0.02502797
0	0.013330446	0	0	0	-0.038149564	0	0	0	0
0	0.028811562	0	0	0	-0.007163367	0	0	0	0.0274056
0	0.032430244	0	0	0	0	0	0	0	-0.02703091
0	0.028930616	0	-0.003053344	0	-0.004249843	0	0	0	0.027811734
0	0.013316035	0	0	0	-0.038113707	0	0	0	0.046429759
0	0.012403715	0	0	0	-0.039766206	0	0	0	-6.30E-05
0	0	0	0	0	0	0	-0.040194032	0	0.043381045
0	0	0	0	0	0	0	0	0	0.00641653
0	0	0	-4.83E-05	0	0	0	0	0	0
0	0.023281815	0	-0.01391897	0	0	0	-0.066352193	0	0.035268366
0	0	0	-1.28E-05	0	0	0	0	0	0.05403924
0	0.023643717	0	-0.013996314	0	0	0	-0.066228165	0	0
0	0.022744886	0	-0.014592985	0	0	0	-0.068669408	0	0.0501091
0	0	0	0	0	-0.042575572	0	0	0	0.01275838
0	0	0	-0.000287483	0	0	0	0	0	0.009364514
0	0.045378355	0	-0.005903682	0	0.010447346	0	0	0	0
0	0.029275223	0	0	0	0	0	-0.075296214	0	0.05664485
0	0	0	-0.002571609	0	-0.024282193	0	0	0	0
0	0	0	0	0	-0.02679246	0	0	0	0.00639333
0	0	0	-0.00237283	0	-0.024627028	0	0	0	0.007529639
0	0	0	-0.002540114	0	-0.024308965	0	0	0	0.054561061
0	0	0	-0.019076283	0	0	0	-0.062844935	0	0.067273149
0	0.040764889	0	-0.002497248	0	-0.01877984	0	-0.062729718	0	0
0	0	0	-0.012958461	0	0	0	0	0	0.002853833
0	0.029848451	0	0.02953931	0	-0.029390007	0	-0.034029084	0	0.035094311

0	0.030000734	0	0.029590037	0	-0.029533799	0	-0.033778118	0	0
0	0.032384261	0	0.001678907	0	0	0	0	0	0
0	0.031663169	0	0	0	0	0	0	0	-0.007963511
0	0.032446186	0	0.001716541	0	0	0	0	0	0.033115371
0	0	0	0.001961026	0	-0.064233413	0	0	0	0.017226233
0	0.00025918	0	0.001939732	0	-0.064192285	0	0	0	0
0	4.50E-05	0	0	0	-0.062527884	0	0	0	0.016771876
0	0.000115008	0	0.00197801	0	-0.064181729	0	0	0	0.061773665
0	0.037997792	0	0.015903238	0	0	0	-0.035930828	0	0.024363997
0	0.038121018	0	0.015908093	0	0	0	-0.035620002	0	0
0	0	0	0	0	-0.019102076	0	-0.036798664	0	0
0	0.050168523	0	0.019708031	0	0	0	-0.036869241	0	0
0	0	0	0.013306448	0	-0.036299552	0	-0.042248113	0	0
0	0.030342728	0	0	0	0	0	-0.035637832	0	0
0	0.030226843	0	0	0	0	0	-0.035960171	0	0.025878783
0	0.048547758	0	0.031498314	0	-0.024879662	0	-0.043076186	0	0
0	0.035997754	0	0.013642174	0	0	0	-0.05181525	0	0.025114925
0	0.036237979	0	0.013650286	0	0	0	-0.051693442	0	0
0	-0.00411874	0	0.001990995	0	-0.071749565	0	0	0	0.016210518
0	0	0	0.02548821	0	-0.041462431	0	-0.031180339	0	0.058822215
0	0.023240051	0	0	0	-0.013405952	0	-0.041427729	0	0.033909723
0	0	0	0.025507575	0	-0.041769906	0	-0.03070317	0	0
0	0.023421531	0	0	0	-0.013495565	0	-0.041202886	0	0
0	0.036250715	0	0.01370752	0	0	0	-0.049249056	0	0.028208787
0	0.028011223	0	0	0	-0.004878081	0	-0.034484911	0	0.031111186
0	0.028242424	0	0	0	-0.004742123	0	-0.034098335	0	0
0	0	0	0.0302091	0	-0.051911551	0	-0.03548149	0	0.063988875
0	0.030465136	0	0	0	0	0	-0.03453449	0	0.015044207
0	0	0	0.029953803	0	-0.05172709	0	-0.034668195	0	0
0	0.051762885	0	0.025787016	0	-0.011808347	0	-0.034727029	0	0
0	0.024358426	0	0	0	-0.011692303	0	-0.038889379	0	0.026069776
0	0.030381094	0	-0.000987424	0	0	0	0	0	-0.013458959
0	0	0	-0.005826732	0	0	0	-0.037571874	0	0
0	0.029618084	0	0	0	0	0	-0.048965267	0	0.028628358
0	0	0	0.023148329	0	-0.037903728	0	-0.027092717	0	0.044163925
0	0.045716949	0	0	0	0.008407478	0	-0.035616421	0	0
0	0.028396081	0	0.029375268	0	-0.033736219	0	0	0	0
0	0.028470596	0	0.029386915	0	-0.033687291	0	0	0	0.035390208
0	0	0	0	0	-0.023756597	0	-0.035705813	0	0.042933507
0	0	0	0	0	-0.02352006	0	-0.035633343	0	0.055008688
0	0	0	-0.010509131	0	0	0	0	0	0.02033884
0	0.020722602	0	0.03755824	0	-0.056286003	0	0	0	0
0	0	0	0	0	-0.023550812	0	-0.03495351	0	0
0	0.020707079	0	0.037568828	0	-0.056259509	0	0	0	0.043920091
0	0.021405145	0	0.036724579	0	-0.054305014	0	0	0	0.001643099
0	0.0378222	0	0.014284732	0	0	0	0	0	0
0	0.038047252	0	0.014356333	0	0	0	0	0	0.020373644

0	0	0	0	0	-0.024072969	0	-0.034221654	0	0.045050066
0	0	0	-0.012878177	0	-0.009021683	0	-0.036853322	0	0
0	0	0	0.005959268	0	-0.025518693	0	-0.033578398	0	0
0	0.038076916	0	0.014031761	0	0	0	0	0	-0.022569399
0	0.048694279	0	0.025249818	0	-0.014925376	0	0	0	0
0	0.027734673	0	0	0	-0.006679572	0	0	0	0
0	0	0	0.008028715	0	-0.028697026	0	0	0	0
0	0.027944889	0	0	0	-0.006523377	0	0	0	0.027761069
0	0.031242688	0	0	0	0	0	0	0	-0.022714373
0	0	0	0.028793758	0	-0.052263186	0	0	0	0
0	0	0	0.028899192	0	-0.052359303	0	0	0	0.060227997
0	0.02792459	0	0	0	-0.006909712	0	0	0	-0.015726237
0	0	0	0.032430145	0	-0.060023869	0	0	0	0.019122931
0	0	0	0.028679876	0	-0.0525385	0	0	0	0.020353665
0	0	0	0.001150179	0	0	0	-0.037376968	0	0.054334039
0	0	0	0	0	0	0	-0.037130211	0	0.043596534
0	0	0	0.0010357	0	0	0	-0.036726711	0	0
0	0	0	0.000657698	0	0	0	-0.035881701	0	0.047400781
0	0.027118631	0	-0.005388677	0	0	0	-0.036372885	0	0.027938132
0	0.027248992	0	-0.005387482	0	0	0	-0.036024988	0	0
0	0.025162283	0	0	0	-0.008900288	0	-0.036481614	0	0.030966468
0	0.025386833	0	0	0	-0.008768199	0	-0.036102817	0	0
0	0.029607057	0	0	0	0	0	-0.036755243	0	0.014483546
0	0	0	-0.000416733	0	0	0	-0.049164647	0	0.058243545
0	0.025288019	0	-0.003303989	0	-0.005966734	0	-0.035317472	0	0.03249194
0	0.025534231	0	-0.003365608	0	-0.005762405	0	-0.03491048	0	0
0	0.025175764	0	0	0	-0.009263721	0	-0.035336679	0	0.019470461
0	0.02737185	0	-0.005297386	0	0	0	-0.035226086	0	0.01636969
0	0	0	0	0	-0.025670084	0	0	0	0.009389601
0	0.043862374	0	-0.006686924	0	0.010961564	0	-0.036006958	0	0
0	0.014125248	0	-0.001126971	0	-0.028470822	0	-0.048879733	0	0.044013254
0	0.014158929	0	0	0	-0.029453161	0	-0.048851297	0	0.044018845
0	0.01291878	0	0	0	-0.035824944	0	0	0	0.002083664
0	0.014546233	0	-0.001120494	0	-0.027803458	0	-0.048100525	0	0.043564917
0	0.014414117	0	-0.001185353	0	-0.02857052	0	-0.048652642	0	0
0	0	0	-0.003007568	0	-0.023097289	0	-0.036180622	0	0.0545296
0	0	0	0	0	-0.026034076	0	-0.036185876	0	0.042657058
0	0	0	-0.003123179	0	-0.023015321	0	-0.035499817	0	0
0	0	0	-0.002681006	0	-0.023792046	0	-0.035055054	0	0.044409462
0	0	0	-0.001494276	0	-0.033496166	0	-0.044707267	0	0.053012722
0	0	0	-0.012877116	0	0	0	-0.036456615	0	0.039420075
0	0.025714613	0	0.030205368	0	-0.023091709	0	-0.040861779	0	0.040500139
0	0.025929832	0	0.030261793	0	-0.023238229	0	-0.040583104	0	0
0	0	0	-0.000923039	0	0	0	0	0	0.008526442
0	0.032591746	0	0.019968423	0	0	0	-0.039472076	0	0.031516248
0	0.032773771	0	0.01998393	0	0	0	-0.039081559	0	0
0	0.024956418	0	-0.004868641	0	-0.00653728	0	0	0	0

0	0.027383576	0	-0.00701504	0	0	0	0	0	-0.021140771
0	0.025152362	0	-0.004891915	0	-0.006370013	0	0	0	0.029594458
0	0.025008422	0	0	0	-0.011248876	0	0	0	-0.016908166
0	0.025174986	0	-0.004650815	0	-0.006818768	0	0	0	-0.01584405
0	0.021063197	0	-0.015342631	0	0	0	-0.061421697	0	0.048125093
0	0.030597372	0	0.017610365	0	0	0	-0.055898179	0	0.031914141
0	0.030890807	0	0.017618861	0	0	0	-0.055741359	0	0
0	0	0	0.027019845	0	-0.034194282	0	-0.038791194	0	0.06048975
0	0.030872411	0	0.017708737	0	0	0	-0.052824995	0	0.034819707
0	0.022844097	0	0	0	0	0	-0.039300355	0	0.033827718
0	0.023018551	0	0	0	0	0	-0.038877789	0	0
0	0	0	0.02703016	0	-0.034505645	0	-0.038301121	0	0
0	0.04859573	0	0.024940485	0	0	0	-0.040460365	0	0
0	0.026394767	0	0	0	0.006609257	0	-0.038212111	0	0.033685372
0	0.026648962	0	0	0	0.006761567	0	-0.037794019	0	0
0	0	0	0.028263174	0	-0.037648706	0	-0.03909876	0	0.064618042
0	0.02306209	0	0	0	0	0	-0.037731309	0	0.022304559
0	0	0	0.028008001	0	-0.037454284	0	-0.038273831	0	0
0	0	0	0.024693174	0	-0.030589111	0	-0.034792593	0	0.049019096
0	0.018982415	0	0	0	-0.006832074	0	-0.048327102	0	0.039229433
0	0.019227914	0	0	0	-0.006917815	0	-0.048077958	0	0
0	0	0	-0.004277727	0	-0.024083237	0	0	0	0.007689077
0	0.022290181	0	0	0	0	0	-0.05217197	0	0.035332094
0	0.020127162	0	0	0	-0.005018349	0	-0.045774072	0	0.033972773
0	0.04751413	0	0.032215533	0	-0.017277945	0	-0.050985685	0	0
0	0.051487823	0	0.023422158	0	0.003079924	0	-0.03838649	0	0
0	0.045448906	0	0	0	0.021002902	0	-0.039536786	0	0
0	0	0	0	0	-0.01111734	0	-0.039468769	0	0.055581894
0	0	0	0	0	-0.011149926	0	-0.038780675	0	0
0	0	0	0.007194789	0	0	0	-0.040962766	0	0.056325788
0	0	0	0	0	-0.01541006	0	-0.043583579	0	0.047708816
0	0	0	0.007077074	0	0	0	-0.04025597	0	0
0	0	0	0	0	0	0	-0.040078409	0	0.042731382
0	0	0	0	0	-0.011671517	0	-0.037891355	0	0.046028847
0	0.026587946	0	0.027204284	0	-0.02048126	0	0	0	0
0	0	0	0.006687715	0	0	0	-0.039322483	0	0.048943356
0	0.032403197	0	0.01816974	0	0	0	0	0	0
0	0.026688786	0	0.027199642	0	-0.020396426	0	0	0	0.037584157
0	0.032598617	0	0.018237741	0	0	0	0	0	0.027087572
0	0	0	0.005474182	0	0	0	-0.053587124	0	0.060434029
0	0.032603403	0	0.017889086	0	0	0	0	0	-0.019374214
0	0.026017616	0	0	0	0.004553995	0	0	0	0
0	0.0238858	0	0	0	0	0	0	0	-0.018989316
0	0.030797447	0	0.001307547	0	0	0	0	0	-0.006188835
0	0.0262522	0	0	0	0.004730086	0	0	0	0.02991909
0	0	0	0.026733024	0	-0.038066618	0	0	0	0
0	0	0	0.026825073	0	-0.038146936	0	0	0	0.060349632

0	0.015006968	0	0.039299701	0	-0.054218525	0	0	0	0
0	0.026217938	0	0	0	0.004334932	0	0	0	-0.017428749
0	0.01016052	0	-0.042107312	0	0	0	0	0	0.010146709
0	0.015492947	0	0.038658599	0	-0.05269016	0	0	0	0.004887763
0	0.04803076	0	0.022840193	0	-0.000485825	0	0	0	0
0	0.014954602	0	0.039308964	0	-0.054193875	0	0	0	0.051235206
0	0	0	0.026560577	0	-0.038283526	0	0	0	0.016900997
0	0.020529184	0	-0.003278931	0	0	0	-0.040033758	0	0.035508107
0	0.020715579	0	-0.003272126	0	0	0	-0.039584911	0	0
0	0.024033948	0	0.029037644	0	0	0	-0.048886784	0	0.03955292
0	0.024261126	0	0.029064566	0	0	0	-0.048403749	0	0
0	0.023333913	0	0	0	0.002569849	0	-0.040496227	0	0.033323511
0	0.023578626	0	0	0	0.002716668	0	-0.040089701	0	0
0	0.02205154	0	0	0	0	0	-0.040184653	0	0.019692966
0	0	0	0.035579078	0	-0.057004108	0	0	0	0.018026346
0	0.023550575	0	-0.005758101	0	0.007895198	0	-0.039023605	0	0.034704667
0	0.02381181	0	-0.005823762	0	0.008113883	0	-0.038589661	0	0
0	0.02077636	0	-0.003158783	0	0	0	-0.038737722	0	0.022951028
0	0.023353232	0	0	0	0.00218873	0	-0.039169719	0	0.020530805
0	0.025858051	0	0.025589019	0	0.006094556	0	-0.047139874	0	0.041121416
0	-0.00044228	0	-0.000155352	0	-0.060201614	0	0	0	0.018350943
0	0.02621025	0	0.025458843	0	0.006425924	0	-0.046623524	0	0
0	0	0	0	0	-0.013444742	0	0	0	0.005456895
0	0.023857481	0	0.028599481	0	0	0	-0.047105193	0	0.031314066
0	0.017425524	0	0.034696412	0	-0.015195046	0	-0.060018591	0	0.049168712
0	0.017764511	0	0.034772315	0	-0.015287094	0	-0.059762668	0	0
0	0	0	0.004940811	0	0	0	0	0	0.006297853
0	0	0	-0.005519859	0	-0.008163184	0	-0.040003185	0	0.05462438
0	0	0	-0.005633228	0	-0.008085029	0	-0.03932127	0	0
0	0	0	-0.009037225	0	0	0	-0.039973531	0	0.039807974
0	0	0	0	0	-0.013499204	0	-0.040140833	0	0.042275569
0	0.025748817	0	0	0	0.02911688	0	-0.047100711	0	0
0	0.025538458	0	0	0	0.028995035	0	-0.047495584	0	0.032269469
0	0.043216626	0	-0.009394267	0	0.025984866	0	-0.039899698	0	0
0	0.018598951	0	0.033038537	0	-0.011960004	0	-0.057054585	0	0.046606295
0	0.021424373	0	0.026045757	0	0	0	-0.066304153	0	0.048608119
0	0	0	0.025494999	0	-0.011718074	0	-0.04844444	0	0.061786304
0	0	0	-0.005147557	0	-0.008911408	0	-0.038703223	0	0.043761337
0	0	0	0.025266666	0	-0.011556982	0	-0.047666632	0	0
0	0	0	0.017901036	0	0	0	-0.047839763	0	0.047316074
0	0	0	0.009085001	0	-0.028798644	0	-0.034870126	0	0
0	0	0	0.030977846	0	-0.021275838	0	-0.055455883	0	0.058578041
0	0.048593396	0	0.021118849	0	0.027322926	0	-0.048518782	0	0
0	0.025412767	0	0	0	0.02866379	0	-0.045721209	0	0.020875479
0	0	0	0.025022604	0	-0.011548149	0	-0.046719971	0	0.05332608
0	0.008723271	0	-0.002930529	0	-0.021779874	0	-0.056694275	0	0.05028133
0	0.009095929	0	-0.00292454	0	-0.021172405	0	-0.056052176	0	0.053211806

0	0.00881197	0	0	0	-0.024302391	0	-0.056664241	0	0.053934801
0	0.009075476	0	-0.003007205	0	-0.021873259	0	-0.056446685	0	0
0	0	0	-0.003133011	0	-0.02494695	0	-0.05408362	0	0.059070486
0	0	0	0	0	0.011062314	0	-0.047049267	0	0.041489696
0	0.008225344	0	0	0	0	0	-0.047394209	0	0.030964177
0	0.006830527	0	0	0	-0.032793193	0	0	0	0.006063292
0	0.014167746	0	-0.013801147	0	0	0	-0.066290259	0	0.056600263
0	0.0230962	0	-0.007546002	0	0.007269265	0	0	0	0
0	0.020725118	0	-0.00510644	0	0	0	0	0	-0.017791201
0	0.023324051	0	-0.007575755	0	0.007468413	0	0	0	0.031369762
0	0.023357008	0	-0.007289167	0	0.006984606	0	0	0	-0.018151436
0	0.023069402	0	0	0	-4.65E-05	0	0	0	-0.019045763
0	0.011227121	0	0	0	0.006728087	0	-0.065352978	0	0.050848258
0	0	0	-0.006973424	0	-0.009161563	0	0	0	0.002975788
0	0.025238658	0	0.023422578	0	0.0083663	0	0	0	0
0	0.022707022	0	0.027608406	0	0	0	0	0	-0.020569937
0	0.025498352	0	0.023386168	0	0.00864151	0	0	0	0.03651275
0	0.025442295	0	0.023046504	0	0.008626856	0	0	0	-0.019544126
0	0.022699346	0	-0.009701683	0	0.034536356	0	-0.04775249	0	0
0	0.022485386	0	-0.009643524	0	0.034361895	0	-0.048167211	0	0.033485215
0	0.022097166	0	0	0	0.025223156	0	-0.048641006	0	0.018894817
0	0.025137637	0	0	0	0.029212772	0	0	0	-0.029669732
0	0.022455179	0	-0.009242602	0	0.033795907	0	-0.046694422	0	0.01943636
0	0	0	0.022808355	0	-0.008805533	0	0	0	0.001860451
0	0	0	-0.00902803	0	0.018056782	0	-0.047733895	0	0.038811762
0	0.008450804	0	0.002675711	0	0	0	-0.048884075	0	0.032758066
0	0.024918748	0	0.004248324	0	0	0	0	0	-0.0021623
0	0	0	0.007122708	0	-0.014546739	0	-0.038618099	0	0
0	0.022074194	0	-0.011920287	0	0.036624601	0	0	0	-0.030594504
0	-0.008410962	0	-0.002017641	0	-0.058212164	0	0	0	0.024109701
0	-0.00229799	0	-0.005515951	0	-0.00722638	0	-0.078154812	0	0.076387418
0	-0.000185307	0	0.047077496	0	-0.052317425	0	0	0	0.014375905
0	0.031606808	0	0.032265825	0	-0.031504329	0	-0.039098457	0	0.034121633
0	0.031769904	0	0.032314695	0	-0.031637316	0	-0.038858161	0	0
0	0.04081476	0	0.018112748	0	0	0	-0.038187797	0	0
0	0.040718664	0	0.018112484	0	0	0	-0.038468666	0	0.022181188
0	0.051892318	0	0.021576993	0	0	0	-0.039246278	0	0
0	0.03201247	0	0	0	0	0	-0.037792932	0	0
0	0.031913722	0	0	0	0	0	-0.03810053	0	0.024751113
0	0.050177752	0	0.033838756	0	-0.026401852	0	-0.047358745	0	0
0	0.053287574	0	0.026878455	0	-0.010280057	0	-0.037393917	0	0
0	0.032123682	0	0	0	0	0	-0.036866431	0	0.012451421
0	0.031867643	0	0	0	-0.000733157	0	-0.036667912	0	0
0	0.031692975	0	0	0	-0.000831925	0	-0.037008733	0	0.027530615
0	0.038299664	0	0.015236343	0	0	0	-0.056786985	0	0.024114699
0	0.038550281	0	0.015249075	0	0	0	-0.056672396	0	0
0	0.038450099	0	0.015285678	0	0	0	-0.055369661	0	0.029527986

0	0	0	0.027398253	0	-0.043412717	0	-0.035108917	0	0.059924304
0	0	0	0.027414799	0	-0.043724111	0	-0.034623198	0	0
0	0.024827318	0	0	0	-0.013321955	0	-0.046083801	0	0.033283947
0	0.025020847	0	0	0	-0.013401092	0	-0.045866648	0	0
0	0	0	0.030704772	0	-0.050879047	0	-0.037967737	0	0.064039053
0	0.02560023	0	0	0	-0.012146912	0	-0.044357048	0	0.027302422
0	0	0	0.030450576	0	-0.050685353	0	-0.037149532	0	0
0	0.047429689	0	0	0	0.010961013	0	-0.037979237	0	0
0	0	0	0.025468881	0	-0.040476603	0	-0.031725215	0	0.046342125
0	0.031163502	0	0	0	0	0	-0.053587389	0	0.029781208
0	0.031946637	0	0.02987907	0	-0.030408131	0	0	0	0
0	0.032105607	0	0.029869013	0	-0.030278914	0	0	0	0.031870328
0	0.040458062	0	0.016254719	0	0	0	0	0	0
0	0	0	0	0	-0.022111266	0	-0.037889564	0	0.055401486
0	0.040725334	0	0.016337336	0	0	0	0	0	0.018603934
0	0	0	0	0	-0.024357319	0	-0.039970528	0	0.04506362
0	0	0	0	0	-0.022139304	0	-0.037213743	0	0
0	0.04076286	0	0.016079728	0	0	0	0	0	-0.02793069
0	0	0	0	0	-0.022579771	0	-0.036681141	0	0.045177173
0	0.050475442	0	0.02608745	0	-0.01325299	0	0	0	0
0	0.021741046	0	0.040533784	0	-0.060285941	0	0	0	0
0	0.021884776	0	0.040372234	0	-0.059895766	0	0	0	-0.000491163
0	0.021739785	0	0.040544966	0	-0.060255167	0	0	0	0.044463918
0	0.032966716	0	0	0	0	0	0	0	-0.026810465
0	0.031253249	0	0	0	-0.002991475	0	0	0	0
0	0.031539656	0	0	0	-0.002781573	0	0	0	0.024672804
0	0.030185154	0	-0.002782178	0	0	0	-0.038417198	0	0
0	0.030077472	0	-0.002781511	0	0	0	-0.038739304	0	0.026046746
0	0.029243619	0	0	0	-0.004488821	0	-0.038458152	0	0
0	0.029073594	0	0	0	-0.004587454	0	-0.038795772	0	0.027603063
0	0.031545348	0	0	0	-0.003069299	0	0	0	-0.022561175
0	0.031376305	0	0	0	0	0	-0.038845848	0	0.010481794
0	0.029333257	0	-0.001653108	0	-0.003118142	0	-0.037543066	0	0
0	0.029139428	0	-0.001602473	0	-0.003278109	0	-0.037906653	0	0.029103395
0	0.029093528	0	0	0	-0.00487753	0	-0.037818311	0	0.014756303
0	0.030287038	0	-0.002697117	0	0	0	-0.037764849	0	0.012843428
0	0	0	0.002180989	0	0	0	-0.039662416	0	0.054943201
0	0	0	0	0	0	0	-0.039302372	0	0.043277177
0	0	0	0.002057534	0	0	0	-0.038974472	0	0
0	0	0	0.029150412	0	-0.051334899	0	0	0	0
0	0	0	0.02924958	0	-0.051424536	0	0	0	0.060653334
0	0	0	0.00175098	0	0	0	-0.038359435	0	0.046948393
0	0	0	0.029065253	0	-0.051555661	0	0	0	0.017372257
0	0	0	0.035136971	0	-0.064187608	0	0	0	0.018401362
0	0.045903171	0	-0.004613234	0	0.011935418	0	-0.03845964	0	0
0	0	0	0.00026929	0	0	0	-0.053890218	0	0.061227034
0	0.016009262	0	0.001011604	0	-0.029854723	0	-0.053403832	0	0.044879476

0	0.01604444	0	0.001013227	0	-0.029803406	0	-0.053331948	0	0.043419499
0	0.01601431	0	0	0	-0.028915026	0	-0.053358326	0	0.044689777
0	0	0	-0.001185807	0	-0.023246837	0	-0.038525434	0	0.05527524
0	0	0	0	0	-0.024411045	0	-0.038427289	0	0.042386758
0	0.016339197	0	0.000949787	0	-0.029895451	0	-0.053103655	0	0
0	0	0	-0.001303572	0	-0.023161653	0	-0.037836411	0	0
0	0	0	-0.000915611	0	-0.023816575	0	-0.037574667	0	0.044200574
0	0	0	0	0	-0.024414157	0	0	0	0.007042166
0	0	0	0.000583251	0	-0.035494219	0	-0.048571003	0	0.055281965
0	0	0	-0.011127614	0	0	0	-0.038800194	0	0.039658841
0	0.028654935	0	-0.003194095	0	-0.004173225	0	0	0	0
0	0.030317134	0	-0.004546189	0	0	0	0	0	-0.025849062
0	0.028854825	0	0	0	-0.007139853	0	0	0	-0.023233126
0	0.028974345	0	-0.003071665	0	-0.004210195	0	0	0	-0.022395494
0	0.028927611	0	-0.003236744	0	-0.003927225	0	0	0	0.02665595
0	0.013325178	0	0	0	-0.038150434	0	0	0	0.000574908
0	0	0	-3.77E-05	0	0	0	0	0	0.006406627
0	0.023295198	0	-0.013872751	0	0	0	-0.06643626	0	0.049351306
0	0	0	-0.002559397	0	-0.02429591	0	0	0	0.005770346
0	0.028635443	0	0.031265953	0	-0.032805558	0	-0.037051787	0	0.03363595
0	0.028792926	0	0.031312321	0	-0.032934974	0	-0.036810426	0	0
0	0.028628208	0	0.030883462	0	-0.032931527	0	-0.035619097	0	0.036671808
0	0.028931517	0	0.030788527	0	-0.032684144	0	-0.035162596	0	0
0	0.029824208	0	0.029557122	0	-0.02947426	0	-0.03391823	0	0.022717402
0	0.032457339	0	0.001688812	0	0	0	0	0	-0.00821981
0	0.038076661	0	0.01637728	0	0	0	-0.037031515	0	0
0	0.037983964	0	0.016376047	0	0	0	-0.037296084	0	0.0212704
0	0.000120614	0	0.001965292	0	-0.064211804	0	0	0	0.017119621
0	0.038003012	0	0.015891809	0	0	0	-0.035789185	0	0.012710052
0	0.049216481	0	0.026770192	0	-0.013757719	0	-0.036618943	0	0
0	0	0	0.009366377	0	-0.027631334	0	-0.037122419	0	0
0	0	0	0.030500078	0	-0.051623467	0	-0.036802338	0	0.060963251
0	0.028091182	0	0	0	-0.004272966	0	-0.035756779	0	0.028032495
0	0.028279364	0	0	0	-0.004163035	0	-0.035412737	0	0
0	0.030235026	0	0	0	0	0	-0.035773871	0	0.011074079
0	0	0	0.030261947	0	-0.051435972	0	-0.036038876	0	0
0	0.035999255	0	0.013649764	0	0	0	-0.051837345	0	0.028608814
0	0	0	0.02552805	0	-0.041564247	0	-0.031033179	0	0.04287791
0	0.023220065	0	0	0	-0.013450435	0	-0.041357272	0	0.026311443
0	0.02800602	0	0	0	-0.004868975	0	-0.034322179	0	0.017279551
0	0	0	0.030196195	0	-0.051918003	0	-0.035373076	0	0.054963331
0	0.028412946	0	0.029570547	0	-0.033527384	0	0	0	0
0	0.028533983	0	0.029569556	0	-0.033433637	0	0	0	0.032956873
0	0.028505423	0	0.029345255	0	-0.033633427	0	0	0	-0.008612366
0	0	0	0	0	-0.023526639	0	-0.035482092	0	0.042736568
0	0.020713339	0	0.037557751	0	-0.056287134	0	0	0	0.001011538
0	0.038068051	0	0.014340207	0	0	0	0	0	-0.026222165

0	0.027983341	0	0	0	-0.006503785	0	0	0	-0.019268968
0	0	0	0.028837331	0	-0.052289733	0	0	0	0.017169865
0	0	0	0.001137426	0	0	0	-0.037248653	0	0.043975251
0	0.025299137	0	-0.00364504	0	-0.005266453	0	-0.036282871	0	0.030114232
0	0.025510018	0	-0.003698702	0	-0.005085437	0	-0.035918621	0	0
0	0.027127779	0	-0.005398435	0	0	0	-0.036193458	0	0.013484699
0	0.025173332	0	0	0	-0.008904798	0	-0.036309071	0	0.016958578
0	0.02527944	0	-0.003312507	0	-0.005947298	0	-0.035090233	0	0.01666504
0	0.014124791	0	-0.001126133	0	-0.028472724	0	-0.048878584	0	0.043836554
0	0	0	-0.003018195	0	-0.02309821	0	-0.03602265	0	0.041766323
0	0.024487385	0	0.031971015	0	-0.026633002	0	-0.043920824	0	0.038973318
0	0.024708493	0	0.032024382	0	-0.02676392	0	-0.043654436	0	0
0	0.032615811	0	0.0204737	0	0	0	-0.040983296	0	0.027997977
0	0.032761006	0	0.020482277	0	0	0	-0.040630465	0	0
0	0.026944105	0	0.028869506	0	-0.019555554	0	-0.039284433	0	0.038917285
0	0.027294729	0	0.028741238	0	-0.019220398	0	-0.038796	0	0
0	0.025695248	0	0.030219769	0	-0.023158875	0	-0.040773149	0	0.030616302
0	0.032594025	0	0.019939354	0	0	0	-0.039280737	0	0.016933948
0	0.025193054	0	-0.004912587	0	-0.006327995	0	0	0	-0.018297901
0	0.030598392	0	0.017617542	0	0	0	-0.05591932	0	0.035261444
0	0.026495653	0	0	0	0.007225204	0	-0.039634941	0	0.030146342
0	0.026707326	0	0	0	0.007353864	0	-0.039277763	0	0
0	0	0	0.028608601	0	-0.037404396	0	-0.040577551	0	0.061169274
0	0	0	0.027040702	0	-0.034267715	0	-0.038681405	0	0.048073709
0	0	0	0.028373062	0	-0.037220721	0	-0.039811723	0	0
0	0.022861714	0	0	0	0	0	-0.039109102	0	0.018120568
0	0.048650076	0	0.024550083	0	0.000757271	0	-0.040473736	0	0
0	0.026406259	0	0	0	0.006604418	0	-0.038032117	0	0.019116627
0	0	0	0.02825416	0	-0.037656918	0	-0.038965841	0	0.054174977
0	0.01897328	0	0	0	-0.006860712	0	-0.048282029	0	0.034163883
0	0	0	0	0	-0.011124456	0	-0.039303272	0	0.042301738
0	0	0	0.007181922	0	0	0	-0.040823967	0	0.045116896
0	0.026620978	0	0.027430361	0	-0.020293592	0	0	0	0
0	0.026772389	0	0.027423462	0	-0.020170383	0	0	0	0.034764755
0	0.026726654	0	0.02715379	0	-0.020339149	0	0	0	-0.010790253
0	0.032622466	0	0.018220455	0	0	0	0	0	-0.023522634
0	0.026296423	0	0	0	0.00475231	0	0	0	-0.021536115
0	0	0	0.026780245	0	-0.038113427	0	0	0	0.012349187
0	0.014962096	0	0.039297926	0	-0.054225192	0	0	0	0.004938182
0	0.023576674	0	-0.006143931	0	0.008666424	0	-0.04016252	0	0.031875102
0	0.023797846	0	-0.006206285	0	0.0088568	0	-0.039774946	0	0
0	0.026009643	0	0.026049107	0	0.00602735	0	-0.048729751	0	0.036940521
0	0.026308232	0	0.025941034	0	0.006306157	0	-0.048271828	0	0
0	0.020535653	0	-0.003288938	0	0	0	-0.03983776	0	0.020186007
0	0.02404527	0	0.029017517	0	0	0	-0.048696496	0	0.02397808
0	0.023359696	0	0	0	0.002572411	0	-0.040300025	0	0.017493255
0	0.02357567	0	-0.005776093	0	0.007917358	0	-0.038811846	0	0.018302482

0	0.025870395	0	0.025566941	0	0.006108814	0	-0.046959387	0	0.026860945
0	0.017424672	0	0.03469921	0	-0.01520711	0	-0.059999588	0	0.046733385
0	0	0	-0.005531454	0	-0.008164418	0	-0.039830147	0	0.040643415
0	0.02555179	0	0	0	0.028985292	0	-0.047286616	0	0.015405868
0	0	0	0.025476212	0	-0.011716167	0	-0.048284662	0	0.048984714
0	0.008731154	0	-0.002930113	0	-0.02176008	0	-0.05672176	0	0.053432498
0	0.023370498	0	-0.007597197	0	0.007513489	0	0	0	-0.021196439
0	0.025552311	0	0.023341321	0	0.008642146	0	0	0	-0.024325912
0	0.022500105	0	-0.009657584	0	0.034365416	0	-0.047945543	0	0.015656399
0	0.030633868	0	0.033567891	0	-0.034143601	0	-0.041437843	0	0.033004576
0	0.030813913	0	0.033618642	0	-0.034265306	0	-0.041206498	0	0
0	0.032344719	0	0.031486515	0	-0.029414755	0	-0.038186284	0	0.033175231
0	0.031585423	0	0.032281598	0	-0.031579297	0	-0.038999757	0	0.023548575
0	0.032611918	0	0.031372462	0	-0.029149693	0	-0.037752605	0	0
0	0.0407657	0	0.018505699	0	0	0	-0.03938624	0	0
0	0.04069485	0	0.018520767	0	0	0	-0.039629236	0	0.019542012
0	0.040726411	0	0.018126072	0	0	0	-0.038274683	0	0.011449753
0	0.051061272	0	0.027728025	0	-0.011986961	0	-0.039024455	0	0
0	0.031927905	0	0	0	0	0	-0.037916595	0	0.009797757
0	0.031881306	0	0	0	-0.000253674	0	-0.037783846	0	0
0	0.031747765	0	0	0	-0.000331729	0	-0.038083935	0	0.024918675
0	0.031681042	0	0	0	-0.000867785	0	-0.036820486	0	0.014368818
0	0.038301938	0	0.015248643	0	0	0	-0.056822334	0	0.029751285
0	0	0	0.030922944	0	-0.05058507	0	-0.0390642	0	0.061429425
0	0	0	0.030712478	0	-0.050439873	0	-0.038321942	0	0
0	0	0	0.027421931	0	-0.043497812	0	-0.034981974	0	0.045559311
0	0.024816153	0	0	0	-0.013352421	0	-0.046033397	0	0.027442833
0	0	0	0.030691388	0	-0.050875018	0	-0.037848702	0	0.054349614
0	0.031950232	0	0.030031933	0	-0.030247498	0	0	0	0
0	0.032146492	0	0.030012708	0	-0.03008672	0	0	0	0.029938103
0	0.032144119	0	0.029824569	0	-0.030220153	0	0	0	-0.015348785
0	0.040747959	0	0.016320604	0	0	0	0	0	-0.030834964
0	0	0	0	0	-0.022121937	0	-0.037739637	0	0.042380165
0	0.021746591	0	0.040534154	0	-0.060285341	0	0	0	-0.000613179
0	0.031581669	0	0	0	-0.002759541	0	0	0	-0.025343366
0	0.029304648	0	-0.001930632	0	-0.002570103	0	-0.038361138	0	0
0	0.029136961	0	-0.001881101	0	-0.002708315	0	-0.038705931	0	0.027172396
0	0.030082949	0	-0.002791101	0	0	0	-0.038561192	0	0.011543527
0	0.029082858	0	0	0	-0.004592646	0	-0.038612141	0	0.012699235
0	0.029160115	0	-0.001632404	0	-0.003230784	0	-0.037704537	0	0.014624906
0	0	0	0.002160114	0	0	0	-0.039531389	0	0.043963106
0	0	0	0.029206245	0	-0.051390711	0	0	0	0.014571005
0	0.016047213	0	0.001012982	0	-0.029795317	0	-0.053338156	0	0.044876759
0	0	0	-0.001196807	0	-0.023247763	0	-0.038361527	0	0.042034236
0	0.028971916	0	-0.00325833	0	-0.003881804	0	0	0	-0.024142956
0	0.028615613	0	0.031280177	0	-0.032875921	0	-0.036951861	0	0.022824159
0	0.028715369	0	0.031179406	0	-0.032573288	0	-0.036952968	0	0.033521767

0	0.029009229	0	0.031072933	0	-0.032291221	0	-0.03653666	0	0
0	0.028634519	0	0.030867773	0	-0.032924266	0	-0.035486026	0	0.025975566
0	0.037988921	0	0.016362695	0	0	0	-0.03714628	0	0.009200923
0	0	0	0.030486079	0	-0.051618516	0	-0.036686783	0	0.051632406
0	0.028101262	0	0	0	-0.004279596	0	-0.035583932	0	0.014013239
0	0.02857012	0	0.029527809	0	-0.033376762	0	0	0	-0.012255371
0	0.025352248	0	-0.003654744	0	-0.005215426	0	-0.036043404	0	0.014780592
0	0.027061641	0	0.029220621	0	-0.019229015	0	-0.04078169	0	0.035312168
0	0.027358783	0	0.029118334	0	-0.018954883	0	-0.04034358	0	0
0	0.024472156	0	0.03198156	0	-0.026686664	0	-0.043843852	0	0.030676149
0	0.0326218	0	0.020460742	0	0	0	-0.040826066	0	0.015300172
0	0.026952508	0	0.02885182	0	-0.01954368	0	-0.03913922	0	0.027200289
0	0.026514027	0	0	0	0.007226891	0	-0.039462392	0	0.015199343
0	0	0	0.028593139	0	-0.03739948	0	-0.040447854	0	0.050697702
0	0.026813874	0	0.027377962	0	-0.020107891	0	0	0	-0.015021033
0	0.02358679	0	-0.006156317	0	0.008669032	0	-0.039972462	0	0.016726767
0	0.026024766	0	0.026030295	0	0.006040459	0	-0.048547417	0	0.022405156
0	0.03239284	0	0.031729393	0	-0.029120034	0	-0.039305224	0	0.030496143
0	0.03263776	0	0.031637885	0	-0.028886792	0	-0.038928981	0	0
0	0.030629402	0	0.033616595	0	-0.03423102	0	-0.041354745	0	0.023732654
0	0.032338763	0	0.031460326	0	-0.029405614	0	-0.038025051	0	0.02161211
0	0.040702372	0	0.018494664	0	0	0	-0.039470235	0	0.006778752
0	0.031759285	0	0	0	-0.000336868	0	-0.037900118	0	0.009960967
0	0	0	0.030929926	0	-0.050633357	0	-0.03896075	0	0.051364656
0	0.032185091	0	0.029969278	0	-0.03002587	0	0	0	-0.018239645
0	0.029149945	0	-0.001897614	0	-0.002700331	0	-0.038510717	0	0.012142397
0	0.028723509	0	0.031163513	0	-0.032562584	0	-0.036816894	0	0.022509054
0	0.027069877	0	0.029201327	0	-0.019217111	0	-0.040633715	0	0.023239399
0	0.032438813	0	0.031689335	0	-0.029055424	0	-0.039113442	0	0.016653076

SE	bsnp_rs6469674	SE	bsnp_rs13269857	SE	bsnp_rs3020106	SE	bsnp_rs3020119	SE
0	0	0	0	0	0	0	0	0
0	0	0	0	0	0	0	0	0
0	0	0	0	0	0	0	0	0
0	0	0	0	0	0	0	0	0
0	0	0	0	0	0	0	0	0
0	0	0	0	0	0	0	0	0
0	0	0	0	0	-0.098911941	0	0	0
0	0	0	-0.088705327	0	0	0	0	0
0	0	0	0	0	0	0	0.060224668	0
0	0.071095363	0	0	0	0	0	0	0
0	0	0	0	0	0	0	0	0
0	0	0	0	0	-0.054179342	0	0	0
0	0	0	0	0	0	0	0	0
0	0.062315654	0	0	0	0	0	0	0
0	0.059579989	0	0	0	0	0	0	0
0	0	0	0	0	0	0	0	0
0	0	0	0	0	-0.053052461	0	0	0
0	0	0	0	0	-0.085366166	0	0	0
0	0	0	0	0	-0.06112913	0	0	0
0	0	0	0	0	0	0	0	0
0	0	0	-0.040191426	0	0	0	0	0
0	0	0	0	0	0	0	0	0
0	0	0	0	0	0	0	0	0
0	0	0	0	0	0	0	0	0
0	0	0	-0.039044322	0	0	0	0	0
0	0.063424271	0	0	0	0	0	0	0
0	0	0	0	0	0	0	0	0
0	0	0	0	0	0	0	0	0
0	0	0	0	0	0	0	0	0
0	0	0	-0.048607302	0	0	0	0	0
0	0	0	0	0	0	0	-0.020621848	0
0	0	0	0	0	-0.08570356	0	0	0
0	0	0	0	0	0	0	0	0
0	0	0	0	0	0	0	0	0
0	0	0	-0.072957313	0	0	0	0	0
0	0	0	0	0	0	0	0	0
0	0	0	0	0	0	0	-0.009262857	0
0	0	0	0	0	0	0	0	0
0	0	0	0	0	0	0	0	0
0	0	0	0	0	0	0	0	0
0	0	0	-0.074115442	0	0	0	0	0
0	0	0	0	0	0	0	0.004484727	0
0	0	0	0	0	0	0	0	0
0	0.067230184	0	0	0	0	0	0	0
0	0	0	0	0	0	0	0	0

0	0	0	0	0	-0.080250788	0	0.043792752	0
0	0.086723958	0	0	0	0	0	0.066366392	0
0	0	0	0	0	0	0	0.06641078	0
0	0	0	0	0	-0.094522031	0	0	0
0	0.059749103	0	0	0	-0.094450799	0	0	0
0	0	0	0	0	0	0	0.019062538	0
0	0	0	-0.068405057	0	0	0	0.045049336	0
0	0.056239061	0	0	0	0	0	0	0
0	0	0	0	0	0	0	0	0
0	0	0	0	0	0	0	0	0
0	0	0	0	0	-0.10949577	0	0	0
0	0	0	0	0	0	0	0	0
0	0	0	0	0	0	0	0.031515515	0
0	0	0	0.13188862	0	-0.22904692	0	0	0
0	0.060983882	0	-0.084265883	0	0	0	0	0
0	0	0	-0.084339139	0	0	0	0	0
0	0	0	0	0	0	0	0	0
0	0	0	-0.098019731	0	0	0	0	0
0	0	0	0	0	0	0	0.066757152	0
0	0.099912211	0	0	0	0	0	0	0
0	0	0	0	0	0	0	0	0
0	0	0	0	0	-0.064841687	0	0	0
0	0	0	0.20393417	0	-0.25208165	0	0	0
0	0.055387856	0	0	0	-0.050753577	0	0	0
0	0	0	0	0	-0.050850449	0	0	0
0	0	0	0	0	-0.063661453	0	0	0
0	0	0	0	0	-0.049031283	0	0	0
0	0.057871215	0	0	0	-0.04905597	0	0	0
0	0.05923392	0	0	0	0	0	0	0
0	0	0	0.20098127	0	-0.24802852	0	0	0
0	0.057861787	0	0	0	-0.081134109	0	0	0
0	0	0	0	0	-0.081218282	0	0	0
0	0	0	0	0	-0.071875719	0	0	0
0	0	0	0.19011808	0	-0.27214273	0	0	0
0	0.057910293	0	0	0	-0.057086543	0	0	0
0	0	0	0	0	-0.05717481	0	0	0
0	0	0	0	0	0	0	0	0
0	0.061798216	0	0	0	0	0	0	0
0	0.056632114	0	-0.036800519	0	0	0	0	0
0	0	0	-0.036902328	0	0	0	0	0
0	0	0	-0.03501852	0	0	0	0	0
0	0.059204294	0	-0.03504644	0	0	0	0	0
0	0	0	-0.049576542	0	0	0	0	0
0	0	0	0	0	-0.050521089	0	0	0
0	0	0	0.17814263	0	-0.23513084	0	0	0
0	0	0	-0.048383769	0	0	0	0	0
0	0	0	0	0	0	0	0	0

0	0.060506346	0	0	0	0	0	0	0
0	0	0	0	0	-0.070509004	0	0	0
0	0	0	0	0	-0.052983651	0	0	0
0	0	0	0	0	-0.065273569	0	0	0
0	0.060257605	0	0	0	0	0	0	0
0	0	0	0	0	0	0	0	0
0	0	0	0	0	-0.093597858	0	0	0
0	0	0	0	0	-0.059659635	0	0	0
0	0	0	0	0	-0.051211185	0	0	0
0	0	0	0	0	-0.059707812	0	0	0
0	0	0	0	0	-0.053340343	0	-0.018233269	0
0	0	0	0	0	-0.064133757	0	0	0
0	0.059171076	0	-0.044578437	0	0	0	0	0
0	0	0	-0.044670054	0	0	0	0	0
0	0	0	0	0	0	0	0	0
0	0	0	-0.058094418	0	0	0	0	0
0	0	0	0	0	0	0	0	0
0	0	0	0	0	0	0	0	0
0	0.060770449	0	0	0	0	0	0	0
0	0.062624143	0	0	0	0	0	0	0
0	0	0	0	0	0	0	0	0
0	0	0	0	0	0	0	0.003026498	0
0	-0.02338756	0	0	0	0	0	0	0
0	0.058031953	0	0	0	0	0	0	0
0	0.063264275	0	0	0	0	0	0.003068881	0
0	0.058952519	0	0	0	0	0	0	0
0	0.059369828	0	-0.068724553	0	0	0	0	0
0	0	0	0	0	0	0	0	0
0	0.057361506	0	0	0	0	0	-0.006560836	0
0	0	0	-0.068812872	0	0	0	0	0
0	0.05372681	0	0	0	0	0	0	0
0	0	0	0	0	0	0	-0.006520005	0
0	0	0	0	0	0	0	0	0
0	0.059447341	0	0	0	0	0	0	0
0	0.062210622	0	0	0	0	0	0	0
0	0	0	0	0	0	0	0	0
0	0	0	-0.036588764	0	0	0	0	0
0	0	0	0	0	-0.052789115	0	-0.0077686	0
0	0	0	0	0	0	0	0	0
0	0	0	0	0	0	0	0	0
0	0	0	0	0	-0.084829222	0	0	0
0	0	0	0	0	0	0	0	0
0	0	0	0	0	-0.086066098	0	-0.002377053	0
0	0	0	-0.039210911	0	0	0	0	0
0	0	0	0	0	-0.066445334	0	0	0
0	0	0	-0.036901796	0	0	0	0	0
0	0	0	0.16957471	0	-0.25245898	0	0	0

0	0.048176099	0	0	0	-0.082759993	0	0	0	
0	0	0	0	0	-0.082833538	0	0	0	
0	0	0	-0.039456346	0	0	0	-0.019144552	0	
0	0	0	-0.044826843	0	0	0	0	0	
0	0	0	-0.048407874	0	0	0	0	0	
0	0	0	-0.044753298	0	0	0	0	0	
0	0	0	0	0	-0.06103115	0	0.001523234	0	
0	0	0	-0.055981758	0	0	0	0	0	
0	0	0	0	0	0	0	-0.019966904	0	
0	0.063824453	0	0	0	0	0	0	0	
0	0	0	0	0	0	0	0	0	
0	0	0	0	0	0	0	-0.013319096	0	
0	0	0	-0.045194655	0	0	0	0	0	
0	0	0	0	0	0	0	0	0	
0	0	0	0	0	0	0	0	0	
0	0	0	0	0	0	0	0	0	
0	0	0	0	0	0	0	0	0	
0	0	0	0	0	0	0	-0.023459712	0	
0	0	0	0	0	0	0	0	0	
0	0	0	-0.038850781	0	0	0	-0.008522067	0	
0	0	0	0	0	0	0	-0.002287389	0	
0	0.068141542	0	0	0	0	0	0.015801973	0	
0	0	0	0	0	0	0	0.015850757	0	
0	0	0	0	0	0	0	0	0	
0	0	0	0	0	0	0	-0.023048513	0	
0	0	0	0	0	-0.093216901	0	0	0	
0	0.064572919	0	0	0	0	0	0	0	
0	0	0	0	0	0	0	0	0	
0	0.06190896	0	0	0	0	0	0	0	
0	0.057381725	0	0	0	0	0	0	0	
0	0	0	0	0	0	0	0	0	
0	0	0	-0.080014932	0	0	0	0	0	
0	0	0	0	0	0	0	0	0	
0	0	0	0	0	0	0	0	0	
0	0	0	0	0	0	0	0	0	
0	0	0	0	0	0	0	0	0	
0	0	0	-0.052535389	0	0	0	0	0	
0	0	0	0	0	-0.081247273	0	0.014403304	0	
0	0	0	0	0	0	0	-0.020553769	0	
0	0	0	-0.048494359	0	0	0	0.001336363	0	
0	0	0	0	0	0	0	0	0	
0	0	0	0	0	0	0	-0.020546779	0	
0	0	0	0	0	0	0	-0.011561776	0	
0	0	0	-0.072433142	0	0	0	0	0	
0	0.049526387	0	-0.071243692	0	0	0	0	0	
0	0	0	0	0	0	0	0	0	
0	0	0	-0.071320251	0	0	0	0	0	

0	0	0	-0.072854221	0	0	0	0.000345151	0
0	0	0	0	0	0	0	-0.011898295	0
0	0	0	0	0	-0.071768063	0	0.050839651	0
0	0.074434598	0	0	0	-0.071695853	0	0.050809982	0
0	0	0	0	0	0	0	0	0
0	0	0	0	0	0	0	0	0
0	0	0	0	0	0	0	0.011301728	0
0	0	0	0	0	-0.090868967	0	0.050784457	0
0	0	0	0	0	0	0	0	0
0	0	0	-0.080447949	0	0	0	0	0
0	0	0	-0.068925619	0	0	0	0.016035295	0
0	0	0	0	0	0	0	-0.003169114	0
0	0.1078799	0	0	0	0	0	0	0
0	0	0	0	0	0	0	0	0
0	0	0	-0.059598481	0	0	0	0.052444122	0
0	0.076284231	0	-0.059523071	0	0	0	0.05241683	0
0	0.075395257	0	0	0	0	0	0.030149846	0
0	0	0	0	0	0	0	0.030237975	0
0	0	0	0	0	0	0	0.00446264	0
0	0	0	0.17035009	0	-0.24676074	0	0.047494208	0
0	0	0	-0.077615151	0	0	0	0.051632742	0
0	0.066332941	0	0	0	0	0	0	0
0	0.072201695	0	0	0	0	0	0	0
0	0	0	0	0	0	0	0	0
0	0.07211112	0	0	0	0	0	0.042993026	0
0	0	0	0	0	0	0	0.043063478	0
0	0.094665505	0	0	0	0	0	0	0
0	0	0	0	0	0	0	0	0
0	0	0	0.13289585	0	-0.225636	0	0	0
0	0.059942448	0	0.13293466	0	-0.22560231	0	0	0
0	0	0	0.13428778	0	-0.24208997	0	0	0
0	0	0	0	0	0	0	0.026227707	0
0	0.094560454	0	0	0	0	0	0.065368004	0
0	0	0	0	0	0	0	0.065375591	0
0	0.050153525	0	0	0	0	0	0.066396032	0
0	0.042435233	0	0	0	-0.10047858	0	0	0
0	0	0	0	0	-0.10049291	0	0	0
0	0	0	0	0	0	0	0.019618139	0
0	0.027812525	0	0	0	-0.09448103	0	0	0
0	0.07078698	0	0	0	0	0	0	0
0	0	0	0	0	0	0	0	0
0	0	0	0	0	0	0	0.038232637	0
0	0.050519207	0	-0.087755706	0	0	0	0	0
0	0	0	-0.08776603	0	0	0	0	0
0	0.03105425	0	-0.084293961	0	0	0	0	0
0	0	0	0.20794219	0	-0.26682766	0	0	0
0	0.055366876	0	0.20385393	0	-0.24857494	0	0	0

0	0	0	0.20498753	0	-0.26271303	0	0	0
0	0	0	0.20378291	0	-0.24860413	0	0	0
0	0	0	0.20163896	0	-0.24462699	0	0	0
0	0.058039606	0	0.20157401	0	-0.24459009	0	0	0
0	0.058035037	0	0.19075963	0	-0.26852529	0	0	0
0	0	0	0.19069284	0	-0.26854531	0	0	0
0	0	0	0.18203591	0	-0.24984272	0	0	0
0	0	0	0	0	-0.061246789	0	0	0
0	0.058094078	0	0.17886308	0	-0.23177542	0	0	0
0	0	0	0.17880866	0	-0.23181159	0	0	0
0	0	0	0	0	-0.04656552	0	0	0
0	0.057620888	0	0	0	-0.046561792	0	0	0
0	0	0	0	0	-0.063735801	0	0	0
0	0	0	0.19988074	0	-0.24445897	0	0	0
0	0.057629993	0	0	0	-0.06641448	0	0	0
0	0	0	0	0	-0.066496399	0	0	0
0	0	0	0	0	-0.061050932	0	0	0
0	0	0	0	0	-0.06184219	0	0	0
0	0.057680969	0	0	0	-0.061182938	0	0	0
0	0	0	0.20256253	0	-0.26202392	0	0	0
0	0	0	0.20057291	0	-0.2477108	0	0	0
0	0	0	0.20664635	0	-0.25153948	0	0	0
0	0.056112269	0	0	0	-0.049455201	0	0	0
0	0	0	0	0	-0.04955188	0	0	0
0	0.050872936	0	0	0	0	0	0	0
0	0	0	0.19937214	0	-0.25292338	0	0	0
0	0.02684064	0	0	0	-0.059739073	0	0	0
0	0	0	0	0	-0.0598389	0	0	0
0	0	0	0.20132407	0	-0.25464462	0	0	0
0	0	0	0.192043	0	-0.2589237	0	0	0
0	0	0	0	0	-0.047490514	0	0	0
0	0.056378751	0	0	0	-0.04747148	0	0	0
0	0	0	0.20216187	0	-0.24957918	0	-0.01700407	0
0	0	0	0.20252577	0	-0.26007707	0	0	0
0	0	0	0.18947638	0	-0.27968704	0	0	0
0	0	0	0	0	-0.054367378	0	0	0
0	0	0	0	0	-0.064138691	0	-0.007599147	0
0	0.056240658	0	0	0	-0.061705537	0	0	0
0	0	0	0	0	-0.061801366	0	0	0
0	0.054143978	0	0	0	-0.05444647	0	0	0
0	0	0	0	0	-0.057230457	0	0	0
0	0	0	0	0	-0.065609262	0	0	0
0	0.031932787	0	0	0	-0.057386259	0	0	0
0	0	0	0	0	-0.063723706	0	0	0
0	0	0	0	0	-0.069693241	0	0	0
0	0.053145153	0	0	0	-0.054853823	0	0	0
0	0	0	0	0	-0.054941477	0	0	0

0	0	0	0	0	-0.077067439	0	0	0
0	0	0	0	0	-0.066076736	0	0	0
0	0	0	-0.032629833	0	0	0	0	0
0	0.053637321	0	0	0	-0.050622015	0	-0.00520788	0
0	0.058928034	0	-0.032629644	0	0	0	0	0
0	0	0	0	0	-0.050718522	0	-0.005141418	0
0	0.044814715	0	0	0	-0.050768495	0	0	0
0	0	0	0	0	-0.06381818	0	0.001940058	0
0	0	0	0	0	-0.049077394	0	0.003590254	0
0	-0.025883879	0	0	0	-0.049038283	0	0	0
0	0.058987846	0	0	0	-0.049101974	0	0.00362482	0
0	0	0	-0.046057645	0	0	0	0	0
0	0	0	0.19996484	0	-0.24683611	0	-0.006076156	0
0	0.030424081	0	0	0	-0.065892272	0	0	0
0	0	0	0	0	-0.065967512	0	0	0
0	0.060445097	0	0	0	-0.078471444	0	0.008401366	0
0	0	0	0	0	-0.078538255	0	0.008463597	0
0	0.061077093	0	0	0	-0.079951154	0	0	0
0	0.043794345	0	0	0	-0.081149143	0	0	0
0	0.05786244	0	0	0	-0.081134217	0	0	0
0	0	0	0	0	-0.079983331	0	0	0
0	0	0	0	0	-0.081219601	0	0	0
0	0	0	0	0	-0.071678516	0	0.010277121	0
0	0.057360126	0	-0.035730698	0	0	0	0	0
0	0	0	-0.03583241	0	0	0	0	0
0	0	0	-0.048711781	0	0	0	0	0
0	0	0	-0.033203804	0	0	0	0	0
0	0.057700432	0	-0.033183005	0	0	0	0	0
0	0	0	0	0	-0.071267237	0	0	0
0	0.061589945	0	0	0	-0.056059601	0	0.01199192	0
0	0	0	0	0	-0.056147011	0	0.012046484	0
0	0	0	-0.044213238	0	0	0	0	0
0	0	0	0.18900337	0	-0.2706001	0	0	0
0	0.059105641	0	-0.04434857	0	0	0	0	0
0	0.055818417	0	0	0	-0.06019821	0	0	0
0	0	0	0	0	-0.060268683	0	0	0
0	0	0	0.19015727	0	-0.27214024	0	0.000139247	0
0	0	0	-0.04625753	0	0	0	0	0
0	0	0	0	0	0	0	0	0
0	0.059024738	0	-0.05192329	0	0	0	0	0
0	0	0	-0.052009201	0	0	0	0	0
0	0.064227271	0	0	0	0	0	0	0
0	0.0386992	0	0	0	-0.057109574	0	0	0
0	0.035138643	0	-0.043237482	0	0	0	0	0
0	0	0	-0.043337813	0	0	0	0	0
0	0	0	0	0	0	0	0	0
0	0	0	-0.039467324	0	0	0	0	0

0	0.061128476	0	0	0	0	0	0	0	
0	0.056047573	0	-0.039552639	0	0	0	0	0	
0	0	0	0	0	0	0	-0.007378883	0	
0	0	0	0	0	0	0	0	0	
0	0.059493297	0	0	0	0	0	-0.007365725	0	
0	0.054596782	0	0	0	0	0	0	0	
0	0	0	0	0	0	0	0	0	
0	0.00443047	0	0	0	0	0	0	0	
0	0.061734593	0	0	0	0	0	0	0	
0	0	0	-0.040691076	0	0	0	0	0	
0	0	0	0.17916734	0	-0.24160866	0	0	0	
0	0.04021148	0	-0.040859005	0	0	0	0	0	
0	0	0	0	0	-0.059077884	0	0	0	
0	0	0	0	0	-0.049564633	0	-0.017570811	0	
0	0.054968089	0	-0.039850995	0	0	0	0	0	
0	0.057142588	0	-0.04286146	0	0	0	0	0	
0	0	0	-0.039945369	0	0	0	0	0	
0	0	0	-0.042961218	0	0	0	0	0	
0	0.054662349	0	-0.036693603	0	0	0	-0.005849199	0	
0	0	0	-0.036794063	0	0	0	-0.005789578	0	
0	0.049435763	0	-0.03681085	0	0	0	0	0	
0	0	0	-0.044358365	0	0	0	0	0	
0	0	0	-0.04880385	0	0	0	-0.009449173	0	
0	0	0	0	0	-0.051953681	0	-0.020496586	0	
0	0	0	-0.03502503	0	0	0	0.00309752	0	
0	0	0	0	0	-0.050149847	0	0	0	
0	-0.024720417	0	-0.035023706	0	0	0	0	0	
0	0.060172933	0	-0.035052398	0	0	0	0.003134522	0	
0	0	0	0	0	-0.05372413	0	0	0	
0	0	0	0	0	0	0	0	0	
0	0	0	-0.049786779	0	0	0	0	0	
0	0	0	0.17997447	0	-0.23661877	0	0.004692911	0	
0	0.047903794	0	0.168627	0	-0.24859818	0	0	0	
0	0	0	0.16857499	0	-0.24862083	0	0	0	
0	0	0	0	0	-0.049970254	0	-0.020564806	0	
0	0	0	0	0	-0.062078591	0	0	0	
0	0	0	0	0	-0.066249859	0	-0.015076346	0	
0	0	0	0	0	-0.056560452	0	0	0	
0	0	0	0	0	-0.072471591	0	-0.01241626	0	
0	0	0	-0.05039325	0	0	0	0	0	
0	0	0	-0.052270795	0	0	0	0	0	
0	0.062682374	0	0	0	0	0	0	0	
0	0	0	0	0	0	0	0	0	
0	0	0	0	0	-0.05877316	0	-0.017734669	0	
0	0	0	0	0	0	0	-0.009312403	0	
0	0	0	0	0	-0.068842708	0	0	0	
0	0.057353461	0	0	0	0	0	-0.009397954	0	

0	0	0	0	0	-0.060566559	0	0	0
0	0	0	0	0	-0.064452251	0	0	0
0	0	0	0	0	0	0	0	0
0	0	0	0	0	-0.056128046	0	0	0
0	0.059909239	0	0	0	0	0	0	0
0	0	0	0	0	0	0	0	0
0	0.053772371	0	0	0	0	0	0	0
0	0	0	-0.048407899	0	0	0	0.00035967	0
0	0	0	0	0	-0.059624947	0	-0.011383699	0
0	0.016473022	0	0	0	0	0	0	0
0	0	0	0	0	0	0	0	0
0	0.060501044	0	0	0	0	0	0	0
0	0	0	0	0	-0.063105094	0	-0.017909891	0
0	0.05956021	0	0	0	0	0	0	0
0	0.057231234	0	0	0	0	0	-0.009001393	0
0	0	0	0	0	-0.091776149	0	0.009243181	0
0	0	0	0	0	0	0	0	0
0	0	0	0	0	0	0	-0.008959489	0
0	0.053311344	0	0	0	0	0	0	0
0	0	0	0	0	0	0	0	0
0	0	0	-0.061934784	0	0	0	0	0
0	0.060480263	0	0	0	0	0	0	0
0	0	0	0	0	-0.093199361	0	0	0
0	0	0	0	0	0	0	0	0
0	0.038371645	0	-0.050918562	0	0	0	0	0
0	0	0	0	0	0	0	0	0
0	0	0	-0.050992425	0	0	0	0	0
0	0	0	0	0	0	0	0	0
0	0	0	0	0	-0.06252767	0	0	0
0	0.062912183	0	-0.043299267	0	0	0	0.012121397	0
0	0	0	-0.043389617	0	0	0	0.012171533	0
0	0	0	0	0	0	0	0	0
0	0	0	0	0	0	0	-0.012985624	0
0	0	0	0	0	0	0	0	0
0	0.057970929	0	-0.04629567	0	0	0	0	0
0	0	0	-0.046370919	0	0	0	0	0
0	0.042635052	0	-0.044598407	0	0	0	0	0
0	0.05826472	0	0	0	-0.074368985	0	0.025120412	0
0	0	0	-0.035806882	0	0	0	-0.018626671	0
0	0	0	0	0	-0.074435005	0	0.025174336	0
0	0	0	-0.057691057	0	0	0	0.009401233	0
0	0	0	0.16883123	0	-0.2591713	0	0	0
0	0	0	-0.056831254	0	0	0	0	0
0	0.038705756	0	0	0	0	0	0	0
0	0	0	0	0	0	0	0	0
0	0	0	0	0	0	0	0	0
0	0	0	0	0	0	0	0	0

0	0	0	0	0	0	0	0.001353366	0
0	-0.015721377	0	0	0	0	0	0	0
0	0.062942854	0	-0.064999903	0	0	0	0.011617376	0
0	-0.013116359	0	0	0	0	0	0	0
0	0	0	0	0	0	0	0.000729581	0
0	0	0	0	0	0	0	0	0
0	0	0	-0.065071467	0	0	0	0.011678797	0
0	0.061599754	0	0	0	0	0	0	0
0	0.071634096	0	0	0	0	0	0	0
0	0.064345867	0	0	0	0	0	0	0
0	0.061223508	0	0	0	0	0	0.001357904	0
0	0.062843576	0	0	0	0	0	0.000726128	0
0	0.060671393	0	0	0	0	0	0	0
0	0	0	0	0	0	0	0	0
0	0.056898802	0	0	0	0	0	0	0
0	0.070430929	0	-0.064767426	0	0	0	0	0
0	0	0	-0.064797637	0	0	0	0	0
0	0	0	0	0	0	0	0	0
0	0	0	-0.041707872	0	0	0	0	0
0	0	0	0	0	0	0	0.003167649	0
0	0	0	-0.036063032	0	0	0	0	0
0	-0.023176687	0	0	0	0	0	0	0
0	0.056686782	0	0	0	0	0	-0.006678292	0
0	-0.021735787	0	0	0	0	0	0.002969644	0
0	0	0	-0.038315047	0	0	0	-0.021481627	0
0	0	0	0	0	0	0	-0.006637782	0
0	0.058884951	0	0	0	0	0	0.003223805	0
0	0.051610722	0	0	0	0	0	-0.006495816	0
0	0.060723905	0	0	0	0	0	0	0
0	0	0	0	0	0	0	-0.00647312	0
0	0.059085374	0	-0.068684752	0	0	0	0	0
0	0.047647433	0	-0.06873729	0	0	0	0	0
0	0.057205901	0	0	0	0	0	-0.006584399	0
0	0.063284576	0	0	0	0	0	-0.006577538	0
0	0	0	-0.068773807	0	0	0	0	0
0	0.05326781	0	0	0	0	0	0	0
0	0.063353538	0	0	0	0	0	0	0
0	0	0	0	0	0	0	-0.016096143	0
0	0	0	0	0	0	0	-0.006543512	0
0	0	0	0	0	0	0	0	0
0	0.062369993	0	0	0	0	0	0	0
0	0	0	-0.038047287	0	0	0	0	0
0	0	0	0	0	0	0	0	0
0	0	0	0.17836224	0	-0.25560952	0	0.017749679	0
0	0	0	-0.035757203	0	0	0	-0.021642818	0
0	0	0	0	0	0	0	-0.015672912	0
0	0	0	0	0	0	0	-0.010183783	0

0	0	0	0	0	0	0	0	0
0	0	0	-0.041211002	0	0	0	0	0
0	0	0	0	0	-0.085390988	0	-0.001882506	0
0	0	0	-0.043381719	0	0	0	0	0
0	0	0	-0.040647759	0	0	0	0	0
0	0	0	-0.043917246	0	0	0	-0.018767639	0
0	0	0	0	0	-0.066385557	0	0.000738937	0
0	0	0	-0.041173636	0	0	0	0	0
0	0	0	0	0	0	0	-0.025736006	0
0	0	0	-0.049469659	0	0	0	-0.014607826	0
0	0	0	-0.044923368	0	0	0	-0.011832901	0
0	0	0	0	0	-0.088226985	0	0.02527711	0
0	0.041778149	0	0	0	-0.082767441	0	0	0
0	0.048142364	0	0	0	-0.082772603	0	0	0
0	0	0	0	0	-0.082822975	0	0	0
0	0	0	-0.047619119	0	0	0	0	0
0	0	0	-0.044310261	0	0	0	-0.019020996	0
0	0	0	-0.057850353	0	0	0	-0.011175032	0
0	0	0	-0.053904124	0	0	0	0	0
0	0	0	0.17758539	0	-0.24518699	0	0.054833615	0
0	0.075850351	0	0.17761113	0	-0.24513802	0	0.054804423	0
0	0	0	-0.043525173	0	0	0	0	0
0	0.075808477	0	0	0	0	0	0	0
0	0.066453542	0	0	0	0	0	0.008982192	0
0	0	0	0	0	0	0	0	0
0	0	0	0	0	0	0	-0.020201413	0
0	0	0	0	0	0	0	0.009047944	0
0	0	0	0	0	0	0	-0.019931343	0
0	0	0	0	0	0	0	-0.012764193	0
0	0.062232839	0	0	0	0	0	0	0
0	0	0	0	0	0	0	0	0
0	0	0	0	0	0	0	0	0
0	0	0	0.17946478	0	-0.26657245	0	0.054874494	0
0	0	0	0	0	0	0	-0.023380529	0
0	0	0	0	0	0	0	0	0
0	0	0	0	0	0	0	-0.023009104	0
0	0.06962881	0	0	0	0	0	0.016269055	0
0	0	0	0	0	0	0	0.016329089	0
0	0	0	0	0	0	0	-0.023380823	0
0	0	0	0	0	0	0	-0.003306213	0
0	0	0	0	0	0	0	-0.023656303	0
0	0	0	0	0	0	0	0	0
0	0	0	0	0	0	0	-0.001418844	0
0	0.065511333	0	0	0	0	0	0.015935848	0
0	0.055455418	0	0	0	0	0	0.015824047	0
0	0	0	0	0	0	0	0.015974087	0
0	0	0	0	0	0	0	0	0

0	0.060479696	0	-0.061781826	0	0	0	0.027261482	0
0	0	0	-0.061852191	0	0	0	0.027312953	0
0	0.058586106	0	0	0	0	0	0	0
0	0	0	0	0	0	0	0	0
0	0	0	-0.077765774	0	0	0	0.010996205	0
0	0.055921629	0	0	0	0	0	0	0
0	0.057658192	0	0	0	0	0	0	0
0	0	0	-0.079463343	0	0	0	0	0
0	0	0	0	0	0	0	0	0
0	0	0	0	0	0	0	0	0
0	0	0	0	0	0	0	-0.020165721	0
0	0	0	-0.052467456	0	0	0	0.000671897	0
0	0.049988321	0	0	0	-0.0797999	0	0.051992557	0
0	0	0	0	0	-0.079805831	0	0.051997718	0
0	0	0	0	0	0	0	-0.011275257	0
0	0	0	-0.072158894	0	0	0	0.000908439	0
0	0.056802164	0	-0.068609919	0	0	0	0	0
0	0	0	-0.06865726	0	0	0	0	0
0	0.045377084	0	-0.071248587	0	0	0	0	0
0	0	0	0	0	0	0	0.013619908	0
0	0.028416203	0	0	0	-0.071725916	0	0.05084045	0
0	0	0	-0.074843288	0	0	0	0.025921997	0
0	0	0	0	0	0	0	0.00698195	0
0	0	0	0	0	0	0	-0.008872672	0
0	0.10471479	0	0	0	0	0	0.020416155	0
0	0	0	0	0	0	0	0.020470359	0
0	0.10690557	0	0	0	0	0	0	0
0	0.064768235	0	0	0	0	0	0	0
0	0	0	0	0	0	0	0	0
0	0.058949563	0	-0.065006317	0	0	0	0.053219897	0
0	0	0	-0.065005188	0	0	0	0.053225337	0
0	0.032021641	0	-0.059551143	0	0	0	0.052445833	0
0	0.074492287	0	0	0	0	0	0.030155272	0
0	0.064490772	0	0	0	0	0	0.030172757	0
0	0	0	0	0	0	0	0.030241308	0
0	0.093397838	0	0	0	0	0	0.037329502	0
0	0	0	0	0	0	0	0.037381345	0
0	0.072328017	0	0	0	0	0	0	0
0	0.060435538	0	0	0	0	0	0.043016205	0
0	0.042141637	0	0.13371754	0	-0.23257248	0	0	0
0	0	0	0.13368426	0	-0.2325576	0	0	0
0	0.063692875	0	0	0	0	0	0	0
0	0.029706852	0	0.13287945	0	-0.22558289	0	0	0
0	0	0	0	0	0	0	0.025997494	0
0	0.047604834	0	0	0	0	0	0.065344942	0
0	0.028982695	0	0	0	-0.10045525	0	0	0
0	0.031622605	0	-0.087725467	0	0	0	0	0

0	0	0	0.20393409	0	-0.25930144	0	0	0
0	0	0	0.20441329	0	-0.2623693	0	0	0
0	0	0	0.2005586	0	-0.2411425	0	0	0
0	0.057793274	0	0.20048915	0	-0.24107269	0	0	0
0	0	0	0.21075228	0	-0.26634846	0	0	0
0	0	0	0.20319476	0	-0.25839843	0	0	0
0	0.057847556	0	0.20314845	0	-0.25848667	0	0	0
0	0.025206983	0	0.20671223	0	-0.26084015	0	0	0
0	0	0	0.20669723	0	-0.26093595	0	0	0
0	0	0	0.20676705	0	-0.24792789	0	0	0
0	0.056422077	0	0.20678526	0	-0.24792603	0	0	0
0	0.056044165	0	0.20032747	0	-0.24394371	0	0	0
0	0.057803876	0	0.19267666	0	-0.25543381	0	0	0
0	0	0	0.20025335	0	-0.2439695	0	0	0
0	0	0	0.19261017	0	-0.25545205	0	0	0
0	0	0	0.207209	0	-0.26555439	0	-0.006063675	0
0	0	0	0.20839533	0	-0.26533142	0	0	0
0	0	0	0.20766827	0	-0.26688948	0	0	0
0	0	0	0.20384869	0	-0.25541154	0	0	0
0	0.030716076	0	0.20384965	0	-0.25557136	0	0	0
0	0.056174473	0	0.20228909	0	-0.25741848	0	0	0
0	0	0	0.20221744	0	-0.25744597	0	0	0
0	0	0	0.20037449	0	-0.2485689	0	0	0
0	0	0	0.20551189	0	-0.26938422	0	0	0
0	0.054428096	0	0.20029953	0	-0.24857724	0	0	0
0	0.053374377	0	0.20205452	0	-0.25047289	0	0	0
0	0	0	0.20387103	0	-0.26387297	0	0	0
0	0	0	0.20198856	0	-0.25049938	0	0	0
0	0.054067211	0	0.20345397	0	-0.24808892	0	-0.00386717	0
0	0	0	0.2057133	0	-0.26373563	0	0.0039407	0
0	0	0	0.20339047	0	-0.2481256	0	-0.003805015	0
0	0.047782653	0	0.20384393	0	-0.24857589	0	0	0
0	0	0	0.19174829	0	-0.2651755	0	0	0
0	0	0	0.20256855	0	-0.24559983	0	0.005448552	0
0	-0.028166438	0	0.20166664	0	-0.24466167	0	0	0
0	0.059728386	0	0.20250735	0	-0.24556522	0	0.005480756	0
0	0.06146565	0	0.19393723	0	-0.26811386	0	0.011149288	0
0	0	0	0.19388386	0	-0.26813081	0	0.011209702	0
0	0.06180908	0	0.19088805	0	-0.26726317	0	0	0
0	0.058414864	0	0.19100212	0	-0.26883107	0	0	0
0	0.046340267	0	0.19074675	0	-0.26852514	0	0	0
0	0	0	0.19082229	0	-0.26723776	0	0	0
0	0	0	0.19093783	0	-0.26885494	0	0	0
0	0.029434492	0	0.1813443	0	-0.24338873	0	0	0
0	0	0	0.18104436	0	-0.24316312	0	0	0
0	0	0	0	0	-0.055412873	0	0	0
0	0.029579486	0	0	0	-0.055480025	0	0	0

0	0	0	0.18765434	0	-0.25506992	0	0.013839742	0
0	0	0	0	0	-0.05725386	0	0	0
0	0	0	0	0	-0.060870476	0	0	0
0	0	0	0	0	-0.05495204	0	0	0
0	0.057551683	0	0	0	-0.055050814	0	0	0
0	0.062844093	0	0.18495663	0	-0.23640276	0	0.015462594	0
0	0	0	0.18491884	0	-0.23645566	0	0.015516318	0
0	0	0	0.18197365	0	-0.24923184	0	0	0
0	0	0	0	0	-0.060481259	0	-0.007737072	0
0	0	0	0.20119855	0	-0.25466863	0	0	0
0	0	0	0	0	-0.062130317	0	0	0
0	0.026963073	0	0	0	-0.05860901	0	0	0
0	0	0	0	0	-0.058704509	0	0	0
0	0	0	0.19712212	0	-0.24092767	0	-0.015685415	0
0	0.063028412	0	0	0	-0.062256217	0	0	0
0	0	0	0	0	-0.062332827	0	0	0
0	0.055890974	0	0.17941917	0	-0.23559652	0	0	0
0	0	0	0.17936367	0	-0.23561439	0	0	0
0	0	0	0.20330809	0	-0.24732348	0	0	0
0	0	0	0	0	-0.059797682	0	0	0
0	0	0	0	0	-0.046332694	0	0	0
0	0.057052412	0	0	0	-0.046313785	0	0	0
0	0	0	0	0	-0.046367948	0	-0.00591471	0
0	0.040694212	0	0.17884743	0	-0.23178093	0	0	0
0	0.055788728	0	0	0	-0.046365528	0	-0.005908909	0
0	0	0	0	0	-0.062792047	0	-0.00984122	0
0	0	0	0.20485766	0	-0.24863106	0	-0.019453514	0
0	0	0	0.19833337	0	-0.24457444	0	-0.01913356	0
0	0	0	0	0	-0.046712481	0	0	0
0	-0.000282192	0	0	0	-0.046565682	0	0	0
0	0	0	0	0	-0.056314205	0	0	0
0	0.028405597	0	0	0	-0.056343215	0	0	0
0	0.05750393	0	0	0	-0.046768443	0	0	0
0	0	0	0.19947449	0	-0.24707356	0	0	0
0	0	0	0	0	-0.061385794	0	0	0
0	0	0	0.20409691	0	-0.2539327	0	0	0
0	0.056859212	0	0	0	-0.059527947	0	0	0
0	0	0	0	0	-0.05962323	0	0	0
0	0	0	0.20049277	0	-0.26088297	0	-0.013421779	0
0	0	0	0.19944768	0	-0.25518114	0	0	0
0	0	0	0.20524543	0	-0.25886983	0	0	0
0	0.04573404	0	0	0	-0.069392603	0	0	0
0	0	0	0	0	-0.063627003	0	0	0
0	0	0	0	0	-0.069454334	0	0	0
0	0.043861536	0	0	0	-0.063777918	0	0	0
0	0	0	0	0	-0.060786145	0	-0.009933591	0
0	0	0	0	0	-0.057803239	0	0	0

0	0	0	0.19965472	0	-0.25215523	0	-0.01656045	0
0	0.057121896	0	0	0	-0.057798182	0	0	0
0	0	0	0.20137804	0	-0.26017243	0	0	0
0	0.057257895	0	0	0	-0.066630474	0	-0.001202028	0
0	0.043287707	0	0	0	-0.066429467	0	0	0
0	0	0	0	0	-0.073711684	0	0	0
0	0	0	0	0	-0.066701987	0	-0.001143496	0
0	0	0	0	0	-0.061354575	0	-0.003480571	0
0	0	0	0	0	-0.059819377	0	0	0
0	0	0	0	0	-0.062285875	0	0	0
0	0	0	0	0	-0.060979097	0	0	0
0	0.004671349	0	0	0	-0.061059596	0	0	0
0	0.056604681	0	0	0	-0.061484227	0	-0.003477102	0
0	0	0	0.19999827	0	-0.25001617	0	0	0
0	0.057397457	0	0	0	-0.061106492	0	0	0
0	0	0	0.20081417	0	-0.2574649	0	-0.016707123	0
0	0.053618396	0	0	0	-0.049236208	0	-0.007470074	0
0	0	0	0	0	-0.051250415	0	0	0
0	0	0	0	0	-0.049331897	0	-0.007403379	0
0	0	0	0.19428235	0	-0.25906186	0	0	0
0	0	0	0.19769351	0	-0.25126684	0	-0.009606741	0
0	0.054343625	0	0	0	-0.05125432	0	0	0
0	0	0	0.18944216	0	-0.2579285	0	-0.009853553	0
0	0.055897994	0	0	0	-0.049846599	0	0	0
0	0.043677297	0	0	0	-0.049472423	0	0	0
0	0	0	0	0	-0.04992695	0	0	0
0	0.028753524	0	0	0	-0.060757264	0	0	0
0	0	0	0	0	-0.059436586	0	0	0
0	0.032550341	0	0	0	-0.062506067	0	0	0
0	0	0	0	0	-0.060850594	0	0	0
0	0	0	0	0	-0.062572619	0	0	0
0	0.0352471	0	0	0	-0.05957795	0	0	0
0	0.025396347	0	0	0	-0.05958359	0	-0.004649552	0
0	0	0	0.20132365	0	-0.25746926	0	0	0
0	0	0	0	0	-0.059686786	0	-0.00466537	0
0	0	0	0	0	-0.047205129	0	-0.007425151	0
0	0.047130063	0	0	0	-0.059761467	0	0	0
0	0.053881531	0	0	0	-0.047182837	0	-0.007517882	0
0	0	0	0.19278208	0	-0.28055436	0	0.012073327	0
0	0.010159846	0	0	0	-0.047483263	0	0	0
0	0	0	0.18913185	0	-0.27910463	0	0	0
0	0	0	0	0	-0.064971252	0	-0.007826686	0
0	0.054692528	0	0	0	-0.061509483	0	-0.004587352	0
0	0	0	0	0	-0.063309566	0	-0.007485757	0
0	-0.00980092	0	0	0	-0.054361325	0	0	0
0	0	0	0	0	-0.054366816	0	2.09E-05	0
0	0.055498732	0	0	0	-0.061330258	0	0	0

0	0	0	0	0	-0.061606488	0	-0.004518512	0
0	0	0	0	0	-0.057378632	0	0.004595562	0
0	0.045234515	0	0	0	-0.061721733	0	0	0
0	0	0	0	0	-0.061429105	0	0	0
0	0	0	0	0	-0.062953936	0	0	0
0	0	0	-0.038979088	0	0	0	0	0
0	0.054154137	0	0	0	-0.054445191	0	2.88E-05	0
0	0.033087914	0	0	0	-0.05753147	0	0.00461809	0
0	0.037702135	0	-0.039059635	0	0	0	0	0
0	-0.024908158	0	0	0	-0.057235146	0	0	0
0	0	0	0	0	-0.069342572	0	0	0
0	0	0	0	0	-0.069805446	0	-0.003315838	0
0	0.051319385	0	0	0	-0.054734779	0	-0.005400889	0
0	0	0	0	0	-0.054825185	0	-0.00534479	0
0	0.045625152	0	0	0	-0.054862077	0	0	0
0	0	0	0	0	-0.077098985	0	-0.000291637	0
0	0	0	-0.03762697	0	0	0	0	0
0	0	0	0	0	-0.066039752	0	-0.000854973	0
0	0.058914381	0	-0.037729194	0	0	0	0	0
0	0	0	-0.040975133	0	0	0	0	0
0	0	0	-0.032281854	0	0	0	0	0
0	0.058373582	0	-0.032261308	0	0	0	0	0
0	0	0	-0.032486564	0	0	0	-0.006720313	0
0	0.056839768	0	-0.032487434	0	0	0	-0.006711649	0
0	0	0	-0.03095041	0	0	0	0	0
0	0.059859789	0	-0.031011778	0	0	0	0	0
0	0.001642272	0	-0.032629093	0	0	0	0	0
0	0.045709291	0	0	0	-0.050633724	0	-0.005185259	0
0	0	0	-0.045533747	0	0	0	0	0
0	0.033743047	0	0	0	-0.064993437	0	0.012766678	0
0	0	0	0	0	-0.065062871	0	0.012762716	0
0	0	0	-0.045253667	0	0	0	-0.009659423	0
0	-0.023923066	0	0	0	-0.049083234	0	0.003527806	0
0	0.035107259	0	-0.042379199	0	0	0	0	0
0	0	0	-0.042476067	0	0	0	0	0
0	0	0	-0.044348272	0	0	0	0	0
0	0.059432644	0	0.18264366	0	-0.25277615	0	0.02876241	0
0	0	0	0.1826045	0	-0.25280591	0	0.028815182	0
0	0.065057219	0	-0.047197865	0	0	0	0	0
0	0	0	-0.047280047	0	0	0	0	0
0	0	0	-0.043699924	0	0	0	0	0
0	0	0	-0.039452107	0	0	0	0	0
0	0.03678749	0	-0.039493826	0	0	0	0	0
0	0.040795946	0	0	0	-0.065906041	0	0	0
0	0.03062815	0	0	0	-0.065996185	0	0	0
0	0	0	0	0	-0.066066272	0	0	0
0	0.042569905	0	0	0	-0.078479363	0	0.008436693	0

0	0.060692996	0	0	0	-0.078391469	0	0.008345734	0
0	0.060414927	0	0	0	-0.078465376	0	0.008403202	0
0	0	0	0	0	-0.078414344	0	0.008378731	0
0	0	0	0	0	-0.078532933	0	0.00846518	0
0	0.057781862	0	-0.040940731	0	0	0	0	0
0	0	0	-0.04104083	0	0	0	0	0
0	0.043641192	0	0	0	-0.079919817	0	0	0
0	0.061057897	0	0	0	-0.079944942	0	0	0
0	0.054620602	0	-0.035554931	0	0	0	-0.008188568	0
0	0.043791207	0	0	0	-0.081150053	0	0	0
0	0	0	0	0	-0.079978939	0	0	0
0	0	0	-0.042288232	0	0	0	0	0
0	0	0	-0.035654638	0	0	0	-0.008128004	0
0	0	0	-0.047721553	0	0	0	-0.011751668	0
0	0	0	0	0	-0.070684202	0	0.010832482	0
0	0.058135139	0	-0.034334874	0	0	0	0	0
0	0	0	-0.034423674	0	0	0	0	0
0	0.048160252	0	-0.035743772	0	0	0	0	0
0	0	0	-0.038498525	0	0	0	0	0
0	0	0	-0.035826321	0	0	0	0	0
0	0.058115085	0	-0.038489869	0	0	0	0	0
0	0.056281797	0	-0.035829384	0	0	0	0	0
0	0	0	-0.032943974	0	0	0	-0.008229801	0
0	0	0	0	0	0	0	0	0
0	0.054930706	0	-0.032920846	0	0	0	-0.008321307	0
0	0.045767011	0	0	0	0	0	0	0
0	0.059506855	0	0	0	-0.058884295	0	0.011250473	0
0	0	0	0	0	-0.058949834	0	0.01130544	0
0	0.012062181	0	-0.033195164	0	0	0	0	0
0	0	0	0	0	-0.059376314	0	-0.020539095	0
0	0	0	-0.039976379	0	0	0	0	0
0	0	0	-0.045112606	0	0	0	-0.011961751	0
0	0	0	-0.045723727	0	0	0	0	0
0	0	0	0	0	0	0	0	0
0	0.037569713	0	0	0	-0.056084985	0	0.012031978	0
0	0	0	0	0	0	0	0	0
0	0.050268542	0	-0.045896921	0	0	0	0	0
0	0.066690674	0	0	0	0	0	0	0
0	0.063646474	0	0	0	0	0	0	0
0	0	0	-0.044461413	0	0	0	-0.002627072	0
0	0	0	-0.044092713	0	0	0	0	0
0	-0.000240669	0	-0.044212797	0	0	0	0	0
0	0	0	0.18915216	0	-0.27058379	0	0.000545391	0
0	0.058290043	0	-0.044595601	0	0	0	-0.002631603	0
0	0.058525899	0	-0.044222988	0	0	0	0	0
0	0.039947157	0	0	0	-0.060209156	0	0	0
0	0	0	0	0	0	0	-0.007303101	0

0	0	0	0	0	-0.048693519	0	-0.02272558	0
0	0.061938937	0	0	0	0	0	-0.007296638	0
0	0.052977987	0	-0.053382682	0	0	0	0	0
0	0	0	-0.046067756	0	0	0	0	0
0	0	0	-0.05344488	0	0	0	0	0
0	-0.00078375	0	0	0	0	0	0	0
0	0	0	0	0	0	0	-0.011602198	0
0	0.047010774	0	-0.051936248	0	0	0	0	0
0	0.05917264	0	-0.051833392	0	0	0	0.000476651	0
0	0.057227766	0	0	0	0	0	-0.011690033	0
0	0	0	-0.05190868	0	0	0	0.000533514	0
0	0	0	-0.042675993	0	0	0	0	0
0	0.043216397	0	-0.042836545	0	0	0	0	0
0	0	0	0	0	0	0	0	0
0	0.060462266	0	0	0	0	0	0	0
0	0.03341073	0	-0.043100799	0	0	0	-0.00548616	0
0	0.036519097	0	-0.044001561	0	0	0	0	0
0	0	0	0	0	0	0	0	0
0	0	0	-0.043205039	0	0	0	-0.005488494	0
0	0.053368519	0	0	0	0	0	0	0
0	0	0	-0.044096585	0	0	0	0	0
0	0.051271934	0	-0.043252303	0	0	0	0	0
0	0.035341484	0	-0.043333294	0	0	0	0	0
0	0	0	-0.043403788	0	0	0	0	0
0	0.016313234	0	0	0	0	0	0	0
0	0	0	0	0	0	0	-0.007249965	0
0	-0.011009214	0	-0.039461081	0	0	0	0	0
0	0	0	-0.039467635	0	0	0	2.32E-05	0
0	0.052499766	0	0	0	0	0	-0.007230209	0
0	0	0	0	0	0	0	0	0
0	0.05605746	0	-0.039551629	0	0	0	2.90E-05	0
0	0.050111837	0	0	0	0	0	0	0
0	0	0	0	0	0	0	0	0
0	0	0	0	0	0	0	-0.007233605	0
0	0.001869675	0	0	0	0	0	-0.007375601	0
0	0.005165501	0	0	0	0	0	0	0
0	0.05950212	0	0	0	0	0	-0.007254933	0
0	0	0	0	0	-0.052471293	0	-0.017165862	0
0	0.002850457	0	0	0	0	0	0	0
0	0	0	-0.040762342	0	0	0	0.003769765	0
0	0	0	0.18067996	0	-0.24277458	0	0.003904909	0
0	-0.023922524	0	-0.040693601	0	0	0	0	0
0	0	0	0	0	-0.060768806	0	-0.020114047	0
0	0.04118736	0	-0.040927676	0	0	0	0.00380318	0
0	0	0	-0.058219821	0	0	0	0	0
0	0	0	0	0	-0.058842008	0	0	0
0	0	0	0	0	0	0	0	0

0	0	0	0	0	-0.058662029	0	0	0
0	0	0	0	0	-0.05517299	0	-0.019971032	0
0	0.052928787	0	-0.039764439	0	0	0	-0.006017033	0
0	0.055267362	0	-0.04267967	0	0	0	-0.00554871	0
0	0	0	0.18290092	0	-0.26726052	0	0.029077597	0
0	0.055912266	0	-0.042315135	0	0	0	0	0
0	0	0	-0.039858022	0	0	0	-0.005963117	0
0	0	0	-0.042778898	0	0	0	-0.005487742	0
0	0	0	-0.042414697	0	0	0	0	0
0	0.05002824	0	-0.039856601	0	0	0	0	0
0	0.049691687	0	-0.042872627	0	0	0	0	0
0	0	0	-0.043987469	0	0	0	-0.008720483	0
0	0	0	-0.043432321	0	0	0	0	0
0	0	0	0	0	-0.059041266	0	-0.020164234	0
0	0.050428202	0	-0.03669987	0	0	0	-0.005837274	0
0	0	0	0	0	0	0	-0.01125465	0
0	0	0	0	0	-0.054551522	0	-0.019874583	0
0	0	0	0	0	-0.052933547	0	0	0
0	0.043772619	0	0	0	0	0	0	0
0	0	0	-0.049117429	0	0	0	-0.009535671	0
0	0	0	0	0	0	0	0	0
0	-0.023031377	0	-0.035029692	0	0	0	0.003037345	0
0	0	0	0	0	-0.070559627	0	-0.015460883	0
0	0	0	0	0	0	0	0	0
0	0	0	0	0	-0.06545698	0	-0.014602812	0
0	0	0	0	0	0	0	0	0
0	0.038620569	0	0	0	0	0	0	0
0	0.048034603	0	0.16862687	0	-0.24854929	0	0	0
0	0.043800245	0	0.16862045	0	-0.24859652	0	0	0
0	0	0	0.16857673	0	-0.24855677	0	0	0
0	0.036039555	0	0	0	0	0	0	0
0	0.059553526	0	0	0	0	0	-0.009418177	0
0	0	0	0	0	-0.062340178	0	0	0
0	0	0	0	0	0	0	0	0
0	0	0	0	0	-0.059129622	0	0	0
0	0	0	0	0	0	0	-0.009365385	0
0	0	0	-0.05182131	0	0	0	0	0
0	0	0	-0.050333204	0	0	0	-0.002052552	0
0	0.064027729	0	0	0	0	0	0	0
0	0	0	-0.052419428	0	0	0	-0.003333819	0
0	0	0	0	0	-0.061551674	0	-0.017710458	0
0	0	0	0	0	0	0	0	0
0	0	0	0	0	0	0	-0.009413714	0
0	0.057356032	0	0	0	0	0	0	0
0	0.056711959	0	0	0	0	0	-0.009502006	0
0	0	0	0	0	0	0	-0.009270125	0
0	0.050731145	0	0	0	0	0	-0.009340073	0

0	0	0	0	0	0	0	0	0
0	0.05826033	0	0	0	0	0	0	0
0	0.016804979	0	0	0	0	0	-0.00931571	0
0	0	0	0	0	0	0	-0.009314153	0
0	0.057328647	0	0	0	0	0	-0.009403004	0
0	0.041766714	0	-0.04974107	0	0	0	0.012614976	0
0	0	0	-0.049806681	0	0	0	0.012627772	0
0	0.05647191	0	0	0	0	0	-0.009157949	0
0	0.01522116	0	0	0	0	0	0	0
0	0	0	0	0	0	0	0	0
0	0	0	0	0	0	0	-0.009114075	0
0	0.052775784	0	0	0	0	0	0	0
0	0.017566819	0	0	0	0	0	0	0
0	0.050386089	0	0	0	0	0	-0.00894389	0
0	0	0	0	0	0	0	-0.012492252	0
0	0.016468132	0	0	0	0	0	0	0
0	0	0	0	0	0	0	-0.008923246	0
0	0	0	0	0	0	0	-0.012964207	0
0	0	0	0	0	-0.091375762	0	0.009247757	0
0	0.058794284	0	0	0	0	0	0	0
0	0.0618901	0	0	0	0	0	-0.00901393	0
0	0	0	0	0	0	0	0	0
0	0	0	0	0	0	0	-0.018305892	0
0	0	0	0	0	0	0	-0.008816056	0
0	0.061820966	0	0	0	0	0	0	0
0	0	0	0	0	0	0	0	0
0	0	0	-0.061893538	0	0	0	0.000348812	0
0	0	0	0	0	0	0	-0.014689427	0
0	0.037835197	0	-0.050664809	0	0	0	0	0
0	0.044312196	0	-0.05092558	0	0	0	0	0
0	0	0	-0.050734224	0	0	0	0	0
0	0	0	-0.034733746	0	0	0	-0.023866374	0
0	0.061840043	0	-0.044696157	0	0	0	0.011720428	0
0	0	0	-0.044765958	0	0	0	0.011773355	0
0	0.041578036	0	-0.043321323	0	0	0	0.012156622	0
0	0	0	-0.042245164	0	0	0	-0.020738718	0
0	0.043377925	0	-0.046306073	0	0	0	0	0
0	0.050450172	0	0	0	-0.077051385	0	0.026424567	0
0	0	0	0	0	-0.077083524	0	0.026448912	0
0	0	0	-0.056033691	0	0	0	0.010385733	0
0	0	0	-0.036967893	0	0	0	-0.018474901	0
0	0.038658045	0	0	0	-0.074379321	0	0.025157163	0
0	0.036115901	0	0	0	0	0	0	0
0	0	0	0	0	0	0	0	0
0	0.03648883	0	0	0	0	0	-0.006629831	0
0	0	0	0	0	0	0	-0.006648716	0
0	0.062298086	0	0	0	0	0	0	0

0	0.070007202	0	-0.062805432	0	0	0	0.010035424	0
0	0	0	-0.062825218	0	0	0	0.010071869	0
0	0	0	0	0	0	0	0	0
0	-0.013007803	0	0	0	0	0	0	0
0	0	0	0	0	0	0	0.000726355	0
0	-0.012357553	0	0	0	0	0	0	0
0	0.062627621	0	-0.064950234	0	0	0	0.011633343	0
0	-0.015150462	0	0	0	0	0	0.001321854	0
0	0	0	0	0	0	0	0.000458652	0
0	0.045999369	0	-0.065007659	0	0	0	0.011650763	0
0	0	0	0	0	0	0	0.001339399	0
0	-0.017057232	0	0	0	0	0	0	0
0	-0.012882385	0	0	0	0	0	0.000715856	0
0	0	0	-0.065022942	0	0	0	0.011693956	0
0	0.063405784	0	0	0	0	0	0	0
0	0.06181629	0	0	0	0	0	0.000714463	0
0	0	0	-0.039911656	0	0	0	-0.021197451	0
0	0.072117109	0	0	0	0	0	0.002053599	0
0	0.054449439	0	0	0	0	0	-0.007032199	0
0	0.05834228	0	0	0	0	0	0	0
0	0.072107356	0	0	0	0	0	0	0
0	0.064390305	0	0	0	0	0	0.000507111	0
0	0.06096692	0	0	0	0	0	0.001382571	0
0	0	0	0	0	0	0	0.002127398	0
0	0	0	0	0	0	0	0	0
0	0	0	0	0	0	0	-0.006999135	0
0	0	0	-0.039525319	0	0	0	0	0
0	0	0	-0.042249528	0	0	0	-0.021344803	0
0	0.060923127	0	0	0	0	0	0	0
0	0.047118631	0	-0.064728605	0	0	0	0	0
0	0.070135977	0	-0.064693688	0	0	0	0	0
0	0	0	-0.041404361	0	0	0	0	0
0	0	0	-0.064725698	0	0	0	0	0
0	0	0	-0.037059158	0	0	0	0	0
0	-0.021437329	0	0	0	0	0	0.003111374	0
0	0	0	0	0	0	0	-0.01547791	0
0	0.061795595	0	0	0	0	0	-0.006692763	0
0	0	0	-0.039179937	0	0	0	-0.02127813	0
0	0	0	0	0	0	0	-0.01031318	0
0	0.051220501	0	0	0	0	0	-0.006465058	0
0	0.064409853	0	0	0	0	0	-0.006531069	0
0	0	0	0	0	0	0	-0.006442182	0
0	0.0477015	0	-0.06869785	0	0	0	0	0
0	0.063462529	0	0	0	0	0	-0.006602192	0
0	0.063270527	0	0	0	0	0	0	0
0	0	0	-0.039780599	0	0	0	-0.021495872	0
0	0.050194714	0	0.17980276	0	-0.25578864	0	0.05609552	0

0	0	0	0.1797648	0	-0.25575875	0	0.056098513	0
0	0	0	-0.039693737	0	0	0	0	0
0	0	0	-0.04349982	0	0	0	0	0
0	0	0	0	0	0	0	-0.025682307	0
0	0	0	0	0	0	0	-0.026400683	0
0	0	0	-0.048688402	0	0	0	-0.014061287	0
0	0	0	-0.042691459	0	0	0	-0.018767549	0
0	0	0	-0.055649723	0	0	0	-0.014597048	0
0	0.04177743	0	0	0	-0.08276209	0	0	0
0	0.029928507	0	0.17760884	0	-0.24516567	0	0.054834756	0
0	0.075853017	0	0	0	0	0	0.007159225	0
0	0	0	0	0	0	0	0.007218638	0
0	0	0	0	0	0	0	-0.021928558	0
0	0.060833367	0	0	0	0	0	0	0
0	0.060506891	0	0	0	0	0	0.00899559	0
0	0	0	0	0	0	0	-0.022944592	0
0	0	0	0	0	0	0	0	0
0	0.061627363	0	0	0	0	0	0.016944745	0
0	0	0	0	0	0	0	0.01698217	0
0	0	0	0	0	0	0	-0.0014111	0
0	0	0	0	0	0	0	-0.023098416	0
0	0.053683288	0	0	0	0	0	0.01629874	0
0	0.055923878	0	0	0	0	0	0.01594927	0
0	0.042175193	0	-0.061791673	0	0	0	0.027295362	0
0	0.059441061	0	-0.062123403	0	0	0	0.027433787	0
0	0	0	-0.06215177	0	0	0	0.027462923	0
0	0	0	-0.077214447	0	0	0	0.01099629	0
0	0.056521324	0	0	0	0	0	0	0
0	0	0	0	0	0	0	0.001348127	0
0	0.030104938	0	0	0	-0.079762782	0	0.051998477	0
0	0.045015574	0	-0.068591826	0	0	0	0	0
0	0.10377485	0	0	0	0	0	0.020394844	0
0	0.061122294	0	0	0	0	0	0.020420813	0
0	0	0	0	0	0	0	0.020448341	0
0	0.064902265	0	0	0	0	0	0	0
0	0.033289865	0	-0.06496144	0	0	0	0.053227876	0
0	0.064612452	0	0	0	0	0	0.030175609	0
0	0.057461922	0	0	0	0	0	0.037346883	0
0	0.030119211	0	0.13370628	0	-0.2325402	0	0	0
0	0	0	0.20291734	0	-0.25270751	0	0	0
0	0.02838996	0	0.20290172	0	-0.25276356	0	0	0
0	0	0	0.20524518	0	-0.25627584	0	0	0
0	0	0	0.20723921	0	-0.26203843	0	0	0
0	0	0	0.20184807	0	-0.2511553	0	0	0
0	0.057722149	0	0.20179611	0	-0.25120502	0	0	0
0	0	0	0.20287364	0	-0.25772461	0	-0.005527829	0
0	0	0	0.20405718	0	-0.2605375	0	0	0

0	0	0	0.20609049	0	-0.25968375	0	0	0
0	0.025349873	0	0.20316901	0	-0.25633883	0	0	0
0	0	0	0.20315187	0	-0.25642914	0	0	0
0	0.026851988	0	0.20952322	0	-0.25995708	0	0	0
0	0	0	0.20950353	0	-0.25991549	0	0	0
0	0	0	0.20326516	0	-0.24345995	0	0	0
0	0.057048089	0	0.20328544	0	-0.24346037	0	0	0
0	0.063823495	0	0.19729108	0	-0.25532668	0	0	0
0	0	0	0.19722763	0	-0.25534413	0	0	0
0	0	0	0.2033286	0	-0.26053098	0	-0.008180332	0
0	0	0	0.20506009	0	-0.25991294	0	0	0
0	0	0	0.19987184	0	-0.24034815	0	-0.003830685	0
0	0	0	0.2098253	0	-0.26454431	0	-0.008518016	0
0	0	0	0.20056867	0	-0.24107823	0	0	0
0	-0.00283567	0	0.20056175	0	-0.24114711	0	0	0
0	0.056606052	0	0.19980404	0	-0.24028073	0	-0.003828298	0
0	0.057800548	0	0.2004909	0	-0.24106152	0	0	0
0	0	0	0.21147576	0	-0.26411618	0	0	0
0	0	0	0.21079679	0	-0.26633853	0	0	0
0	0	0	0.20416957	0	-0.26216622	0	0	0
0	0.042723989	0	0.2041507	0	-0.26229864	0	0	0
0	0	0	0.20520564	0	-0.2560637	0	0	0
0	0.057117197	0	0.20522597	0	-0.25607846	0	0	0
0	0.056753508	0	0.19905594	0	-0.25226282	0	0	0
0	0	0	0.19901176	0	-0.25231805	0	0	0
0	0	0	0.2029337	0	-0.2582892	0	-0.001655351	0
0	0	0	0.20325565	0	-0.2584853	0	0	0
0	0.002982414	0	0.20319297	0	-0.25840206	0	0	0
0	0.057335446	0	0.20288793	0	-0.25837691	0	-0.001654052	0
0	0.057934306	0	0.20320027	0	-0.25856142	0	0	0
0	0	0	0.20512384	0	-0.24958021	0	0	0
0	0.054663952	0	0.2051301	0	-0.24959024	0	0	0
0	0	0	0.19724778	0	-0.26689127	0	0	0
0	0.026455318	0	0.20590087	0	-0.2607119	0	0	0
0	0.028896444	0	0.2059504	0	-0.26188374	0	0	0
0	0.024197355	0	0.20636679	0	-0.26039518	0	-0.003258978	0
0	0	0	0.20588954	0	-0.26080373	0	0	0
0	0	0	0.205945	0	-0.26194312	0	0	0
0	0.046266816	0	0.19245561	0	-0.25810458	0	0	0
0	0.05026945	0	0.2067647	0	-0.26091889	0	0	0
0	0	0	0.2063519	0	-0.26049283	0	-0.003283403	0
0	0	0	0.19240352	0	-0.2581212	0	0	0
0	0	0	0.20619334	0	-0.24713345	0	-0.006198738	0
0	0.054332576	0	0.20620315	0	-0.2471204	0	-0.006291211	0
0	0.054042886	0	0.19963622	0	-0.2430968	0	-0.005996176	0
0	0.010999009	0	0.20677083	0	-0.24792364	0	0	0
0	0	0	0.19957149	0	-0.24313125	0	-0.005933588	0

0	0	0	0.20242746	0	-0.25609062	0	0	0
0	0.033818985	0	0.20241362	0	-0.25622242	0	0	0
0	0.058303859	0	0.19310788	0	-0.25556633	0	0.001614029	0
0	0.045193092	0	0.20027903	0	-0.24392012	0	0	0
0	0.055992525	0	0.20030973	0	-0.24402065	0	0	0
0	0.045853214	0	0.1926633	0	-0.25543326	0	0	0
0	0	0	0.19305714	0	-0.25559113	0	0.001670459	0
0	0	0	0.20024068	0	-0.24403893	0	0	0
0	0	0	0.20764118	0	-0.2642799	0	-0.005826503	0
0	0	0	0.20686303	0	-0.26560612	0	-0.006172357	0
0	0	0	0.2078363	0	-0.26421489	0	0	0
0	0	0	0.2049944	0	-0.2567369	0	0.006532109	0
0	0.032348836	0	0.20499585	0	-0.25689193	0	0.006552417	0
0	-0.027164739	0	0.20387505	0	-0.25544229	0	0	0
0	0.055064701	0	0.20195728	0	-0.25695698	0	-0.003289408	0
0	0.055736308	0	0.20209146	0	-0.25700605	0	0	0
0	0.048157511	0	0.20227867	0	-0.25742017	0	0	0
0	0	0	0.20189341	0	-0.25699333	0	-0.003225039	0
0	0	0	0.20202288	0	-0.25703874	0	0	0
0	0	0	0.20074149	0	-0.24886469	0	0.002029894	0
0	0	0	0.20523328	0	-0.26892867	0	0	0
0	0	0	0.20529731	0	-0.26921733	0	-0.001230536	0
0	-0.012584591	0	0.20038985	0	-0.24857599	0	0	0
0	0.055114542	0	0.20066648	0	-0.24887312	0	0.002034372	0
0	0.052004674	0	0.20162793	0	-0.24997119	0	-0.004051507	0
0	0	0	0.20156942	0	-0.25000479	0	-0.003996862	0
0	0.048475585	0	0.20204888	0	-0.25047282	0	0	0
0	0	0	0.20415895	0	-0.26421488	0	0.001422248	0
0	0.04844125	0	0.2034478	0	-0.24809138	0	-0.003851101	0
0	0	0	0.19241462	0	-0.26555574	0	0.00255295	0
0	-0.025186244	0	0.20258167	0	-0.24561812	0	0.005382066	0
0	0.033653311	0	0.18755487	0	-0.24830509	0	0.016312692	0
0	0	0	0.18752523	0	-0.24834886	0	0.016315236	0
0	0.061804812	0	0.1941513	0	-0.26839086	0	0.011131614	0
0	0.044761469	0	0.1939283	0	-0.26811251	0	0.011182191	0
0	0.061307743	0	0.19394144	0	-0.26816892	0	0.011184478	0
0	0	0	0.19410262	0	-0.26841402	0	0.011192401	0
0	0	0	0.19388478	0	-0.26814259	0	0.01121716	0
0	0.062086308	0	0.19110949	0	-0.26757021	0	0	0
0	0.046165284	0	0.19087567	0	-0.26722298	0	0	0
0	0.046264719	0	0.1909902	0	-0.26883289	0	0	0
0	0	0	0.19104696	0	-0.2675489	0	0	0
0	0	0	0	0	-0.05298578	0	0	0
0	0.026056526	0	0	0	-0.05305317	0	0	0
0	0	0	0.19809768	0	-0.25192516	0	-0.018717051	0
0	0	0	0	0	-0.055329303	0	0	0
0	0.028463922	0	0	0	-0.055362771	0	0	0

0	0	0	0	0	-0.058214284	0	0	0
0	0.034696378	0	0	0	-0.05829663	0	0	0
0	0.042905164	0	0.18108758	0	-0.24314064	0	0	0
0	0.029708592	0	0.18107937	0	-0.24324442	0	0	0
0	0	0	0.18105511	0	-0.24329734	0	0	0
0	0	0	0	0	-0.056320502	0	0	0
0	0	0	0	0	-0.06118538	0	0	0
0	0	0	0	0	-0.055173892	0	-0.005112836	0
0	0.028206951	0	0	0	-0.055242799	0	-0.005101436	0
0	0	0	0.20105989	0	-0.2437663	0	-0.021478229	0
0	0	0	0.18777537	0	-0.25412915	0	0.014433414	0
0	0	0	0	0	-0.053626617	0	0	0
0	0.001725259	0	0	0	-0.055412097	0	0	0
0	0.06163265	0	0	0	-0.053729994	0	0	0
0	0	0	0	0	-0.059551345	0	-0.012072709	0
0	0	0	0	0	-0.056624915	0	-0.007207553	0
0	0	0	0	0	-0.055274225	0	-0.008814644	0
0	0.060714136	0	0.1851383	0	-0.2394699	0	0.014707492	0
0	0	0	0.18509898	0	-0.23949933	0	0.014761477	0
0	0.054812273	0	0	0	-0.055370282	0	-0.008820277	0
0	0.044165593	0	0	0	-0.066428718	0	0	0
0	0	0	0	0	-0.057639883	0	0	0
0	0	0	0	0	-0.066487573	0	0	0
0	0	0	0	0	-0.055785441	0	0	0
0	0.014109948	0	0	0	-0.054970926	0	0	0
0	0.057702244	0	0	0	-0.0557803	0	0	0
0	0.03930778	0	0.18495119	0	-0.23642231	0	0.015501747	0
0	0	0	0	0	-0.062104014	0	-0.008641661	0
0	0	0	0.2017855	0	-0.2549084	0	0	0
0	0.023143726	0	0	0	-0.056218379	0	0	0
0	0	0	0.20244424	0	-0.25102331	0	-0.018926234	0
0	0	0	0.20217316	0	-0.25414263	0	0	0
0	0	0	0	0	-0.056306798	0	0	0
0	0	0	0.1968214	0	-0.24338395	0	-0.015305594	0
0	0.024808659	0	0	0	-0.058352289	0	-0.006942098	0
0	0	0	0	0	-0.058453788	0	-0.006958733	0
0	0	0	0.20353686	0	-0.25577558	0	-0.019089175	0
0	0	0	0.19727665	0	-0.25186678	0	-0.018780554	0
0	0	0	0	0	-0.058889641	0	-0.009720623	0
0	0.03043786	0	0	0	-0.060308609	0	0	0
0	0.061651894	0	0	0	-0.063000786	0	-0.005411503	0
0	0	0	0	0	-0.06037064	0	0	0
0	0.046018798	0	0	0	-0.058629237	0	0	0
0	0	0	0	0	-0.06306877	0	-0.005348409	0
0	0.062476735	0	0	0	-0.060892713	0	0	0
0	0	0	0.20277894	0	-0.24926838	0	0	0
0	0	0	0	0	-0.04592996	0	-0.009586163	0

0	0.044847995	0	0	0	-0.062251036	0	0	0
0	0	0	0	0	-0.06100096	0	0	0
0	0.053857489	0	0	0	-0.045907778	0	-0.009679234	0
0	0	0	0	0	-0.062720236	0	0	0
0	0.042015646	0	0.17940574	0	-0.23559285	0	0	0
0	0.010170853	0	0	0	-0.046325407	0	0	0
0	0	0	0	0	-0.046117776	0	0	0
0	0.057143115	0	0	0	-0.046144001	0	0	0
0	0	0	0.19793686	0	-0.24654431	0	-0.01857312	0
0	0.026301735	0	0	0	-0.056015457	0	-0.006916298	0
0	0	0	0	0	-0.055993375	0	-0.006889336	0
0	0	0	0	0	-0.046501001	0	-0.005912444	0
0	-0.002316678	0	0	0	-0.046369086	0	-0.005918746	0
0	0	0	0	0	-0.060791813	0	-0.009595138	0
0	0	0	0	0	-0.058563757	0	0	0
0	0	0	0	0	-0.057141804	0	0	0
0	0.029835481	0	0	0	-0.057171634	0	0	0
0	0.032874156	0	0	0	-0.058566072	0	0	0
0	0.055680191	0	0	0	-0.04655872	0	-0.005908198	0
0	-0.00014805	0	0	0	-0.046712483	0	0	0
0	0.013020831	0	0	0	-0.056307964	0	0	0
0	0	0	0.20408225	0	-0.25647142	0	0	0
0	0	0	0.19945293	0	-0.25919648	0	-0.013026505	0
0	0.054577655	0	0	0	-0.059187563	0	-0.006803178	0
0	0	0	0	0	-0.059283468	0	-0.006733972	0
0	0.044113734	0	0	0	-0.059546328	0	0	0
0	0	0	0.19950004	0	-0.25552322	0	0	0
0	0	0	0	0	-0.063715248	0	-0.001881787	0
0	0.045765434	0	0	0	-0.069329015	0	0.000544157	0
0	0.04378258	0	0	0	-0.069390386	0	0	0
0	0	0	0	0	-0.063568465	0	0	0
0	0.000624549	0	0	0	-0.063627922	0	0	0
0	0.043671555	0	0	0	-0.063866474	0	-0.001850087	0
0	0	0	0	0	-0.06938731	0	0.000574573	0
0	0.043685023	0	0	0	-0.063715279	0	0	0
0	0	0	0	0	-0.059116842	0	-0.009731226	0
0	0	0	0	0	-0.057401974	0	-0.006741623	0
0	0	0	0	0	-0.061317361	0	-0.010052831	0
0	0	0	0.19144745	0	-0.25774963	0	-0.013237382	0
0	0.054842265	0	0	0	-0.057390677	0	-0.006832057	0
0	0	0	0.19965918	0	-0.25494698	0	-0.016536304	0
0	0	0	0	0	-0.057572759	0	0	0
0	0.012920797	0	0	0	-0.057792711	0	0	0
0	0.056606372	0	0	0	-0.057555841	0	0	0
0	0	0	0	0	-0.074071966	0	-0.004261493	0
0	0	0	0	0	-0.059205952	0	0	0
0	0.043452288	0	0	0	-0.066640011	0	-0.001172529	0

0	0	0	0	0	-0.061283089	0	-0.003478394	0
0	0.003621835	0	0	0	-0.061360676	0	-0.003475127	0
0	0.004682032	0	0	0	-0.060988026	0	0	0
0	0.056323708	0	0	0	-0.061408135	0	-0.003474773	0
0	0	0	0	0	-0.050974523	0	-0.007509756	0
0	0.051814165	0	0	0	-0.050974542	0	-0.007594787	0
0	0.053469817	0	0	0	-0.049527661	0	-0.007438062	0
0	0.044933338	0	0	0	-0.049248952	0	-0.007445371	0
0	0.012377584	0	0	0	-0.051244625	0	0	0
0	0	0	0	0	-0.04961146	0	-0.007372684	0
0	0.043775481	0	0	0	-0.049855476	0	0	0
0	0.027291202	0	0	0	-0.060612558	0	-0.004803185	0
0	0.031062901	0	0	0	-0.062312753	0	-0.004503062	0
0	0	0	0	0	-0.060709359	0	-0.004813523	0
0	0.031552029	0	0	0	-0.062083122	0	0	0
0	0	0	0	0	-0.062378055	0	-0.004504755	0
0	0.047276041	0	0	0	-0.060773531	0	0	0
0	0	0	0	0	-0.059445463	0	0.001617653	0
0	0	0	0	0	-0.062146453	0	0	0
0	-0.01258536	0	0	0	-0.059432495	0	0	0
0	0.046867931	0	0	0	-0.062491057	0	0	0
0	0.035591767	0	0	0	-0.059586236	0	0.001640573	0
0	0.047934379	0	0	0	-0.059606394	0	-0.00471089	0
0	0.010461414	0	0	0	-0.047197572	0	-0.007427474	0
0	0	0	0.19243485	0	-0.27997177	0	0.012071396	0
0	0	0	-0.035881412	0	0	0	0	0
0	0.033077201	0	-0.035962494	0	0	0	0	0
0	0	0	-0.038746618	0	0	0	0	0
0	0.03669873	0	-0.038787908	0	0	0	0	0
0	0	0	-0.036133424	0	0	0	0	0
0	0.063271893	0	-0.036240838	0	0	0	0	0
0	0	0	0	0	-0.062519474	0	-0.0075869	0
0	0.053853935	0	0	0	-0.061104819	0	-0.004736403	0
0	0	0	-0.038779645	0	0	0	-0.006199956	0
0	0.035994132	0	-0.038862312	0	0	0	-0.00618517	0
0	0	0	0	0	-0.061203893	0	-0.004669157	0
0	0.046018186	0	0	0	-0.061523309	0	-0.004562538	0
0	-0.009800659	0	0	0	-0.05436131	0	6.47E-07	0
0	0.045401163	0	0	0	-0.061347395	0	0	0
0	-0.02237863	0	0	0	-0.057381332	0	0.004536254	0
0	0	0	-0.040117373	0	0	0	0	0
0	0.039967706	0	-0.040220593	0	0	0	0	0
0	0.003289697	0	-0.038978108	0	0	0	0	0
0	0	0	-0.039822162	0	0	0	0	0
0	0	0	0	0	-0.069458361	0	-0.003303005	0
0	0.046558536	0	0	0	-0.054740984	0	-0.00538765	0
0	0	0	-0.038036848	0	0	0	-0.008672002	0

0	0	0	-0.040351704	0	0	0	-0.008902405	0
0	0.056210905	0	-0.038135648	0	0	0	-0.008684134	0
0	0	0	-0.04323114	0	0	0	0	0
0	0	0	-0.031923548	0	0	0	-0.0104561	0
0	0.054884168	0	-0.031900213	0	0	0	-0.010547774	0
0	0	0	-0.036752759	0	0	0	0	0
0	0.010705954	0	-0.037641275	0	0	0	0	0
0	0.058710562	0	-0.036745757	0	0	0	0	0
0	0	0	-0.030191239	0	0	0	0	0
0	0	0	-0.038080317	0	0	0	0	0
0	0	0	-0.044151212	0	0	0	-0.014146398	0
0	0.059496781	0	-0.030211791	0	0	0	0	0
0	0.012034812	0	-0.032273394	0	0	0	0	0
0	0	0	-0.030802633	0	0	0	-0.00672843	0
0	0.057771706	0	-0.030863779	0	0	0	-0.006722232	0
0	-0.000679382	0	-0.03248687	0	0	0	-0.006721499	0
0	0.030168534	0	-0.039338131	0	0	0	0	0
0	0	0	-0.03942752	0	0	0	0	0
0	4.81E-05	0	-0.030950486	0	0	0	0	0
0	0.032631815	0	-0.0421644	0	0	0	-0.007851994	0
0	0.033434262	0	0	0	-0.064820373	0	0.012865447	0
0	0.03964307	0	0	0	-0.065003882	0	0.01274776	0
0	0	0	0	0	-0.064882891	0	0.012874021	0
0	0	0	-0.042266189	0	0	0	-0.007855914	0
0	0	0	-0.044442684	0	0	0	-0.009175447	0
0	0.033017791	0	-0.041387096	0	0	0	0	0
0	0.050668383	0	0.18325364	0	-0.25638142	0	0.030237969	0
0	0.051387595	0	-0.050076307	0	0	0	0	0
0	0.050040745	0	-0.04239297	0	0	0	0	0
0	0	0	-0.042764538	0	0	0	-0.011537951	0
0	0	0	0.18321155	0	-0.25637762	0	0.030261001	0
0	0	0	-0.041453325	0	0	0	0	0
0	0	0	-0.05013667	0	0	0	0	0
0	0.06299199	0	-0.042119481	0	0	0	0	0
0	0	0	-0.042230737	0	0	0	0	0
0	0.040397385	0	0.18263819	0	-0.25278083	0	0.028798174	0
0	0	0	-0.039152497	0	0	0	-0.007889899	0
0	0.03433895	0	-0.039185317	0	0	0	-0.007930675	0
0	0.06401212	0	-0.047794153	0	0	0	-0.004087966	0
0	0	0	-0.047866678	0	0	0	-0.004025761	0
0	0.045603823	0	-0.047212015	0	0	0	0	0
0	0	0	-0.043377948	0	0	0	0	0
0	0	0	-0.039965608	0	0	0	0	0
0	0.03763834	0	-0.040006668	0	0	0	0	0
0	0	0	-0.038925363	0	0	0	0	0
0	0.016812706	0	-0.03945811	0	0	0	0	0
0	0.035612351	0	-0.03891631	0	0	0	0	0

0	0.040855626	0	0	0	-0.066007878	0	0	0
0	0	0	-0.041765025	0	0	0	-0.010925222	0
0	0.042563458	0	0	0	-0.078357221	0	0.00835308	0
0	0.042574403	0	0	0	-0.078474447	0	0.008438118	0
0	0.060667064	0	0	0	-0.078382937	0	0.008346605	0
0	0.055132596	0	-0.040635434	0	0	0	-0.007886223	0
0	0	0	0	0	0	0	0	0
0	0.043353641	0	0	0	0	0	0	0
0	0	0	0	0	-0.078407724	0	0.008379268	0
0	0	0	-0.040733537	0	0	0	-0.007824673	0
0	0.048409616	0	-0.040954518	0	0	0	0	0
0	0.055399572	0	-0.034055541	0	0	0	-0.008343236	0
0	0.043645193	0	0	0	-0.079914813	0	0	0
0	0	0	-0.034145153	0	0	0	-0.008280881	0
0	0.049520193	0	-0.035562495	0	0	0	-0.008174134	0
0	0	0	-0.0355827	0	0	0	-0.008308779	0
0	0	0	-0.038110092	0	0	0	-0.00791875	0
0	0.053478931	0	-0.035582981	0	0	0	-0.008392544	0
0	0.055438597	0	-0.038095397	0	0	0	-0.008007684	0
0	0	0	0	0	0	0	0	0
0	0.046153082	0	-0.034348194	0	0	0	0	0
0	0	0	0	0	0	0	-0.006879986	0
0	0	0	-0.038111607	0	0	0	0	0
0	0.049999049	0	0	0	0	0	0	0
0	0.057140454	0	-0.038094474	0	0	0	0	0
0	0.043825409	0	0	0	0	0	-0.006872333	0
0	0.01316669	0	-0.03849356	0	0	0	0	0
0	0.013616071	0	-0.035820013	0	0	0	0	0
0	0	0	-0.039314208	0	0	0	-0.011172737	0
0	0	0	0	0	0	0	-0.012056178	0
0	0.012389389	0	-0.032935003	0	0	0	-0.008232579	0
0	0.059641523	0	0	0	0	0	-0.012152356	0
0	-0.001804281	0	0	0	0	0	0	0
0	0	0	0	0	-0.058875238	0	-0.021965423	0
0	0	0	0	0	0	0	-0.010087433	0
0	0	0	0	0	-0.056819337	0	-0.02229057	0
0	0.063953647	0	0	0	0	0	-0.010109268	0
0	0.038746369	0	0	0	-0.058894019	0	0.01128832	0
0	0	0	0	0	0	0	0	0
0	0.036048799	0	0	0	0	0	0	0
0	0	0	-0.039225994	0	0	0	0	0
0	0	0	0	0	0	0	0	0
0	0.06538722	0	0	0	0	0	0	0
0	0	0	-0.045598285	0	0	0	0	0
0	0	0	-0.045818761	0	0	0	-0.001633699	0
0	0	0	-0.045050012	0	0	0	-0.011943804	0
0	-0.002820762	0	-0.045719965	0	0	0	0	0

0	0.00768447	0	0	0	0	0	0	0
0	0.04980861	0	-0.045767064	0	0	0	0	0
0	0.012549901	0	0	0	0	0	0	0
0	0.050101599	0	-0.045990448	0	0	0	-0.001601421	0
0	0	0	-0.044340769	0	0	0	-0.002623705	0
0	-0.001029977	0	-0.044459707	0	0	0	-0.0026286	0
0	-0.000211473	0	-0.044092336	0	0	0	0	0
0	0.057712096	0	-0.044470009	0	0	0	-0.002628078	0
0	0	0	0	0	0	0	-0.011739259	0
0	0	0	0	0	-0.050798787	0	-0.022195611	0
0	0.056502871	0	0	0	0	0	-0.011823591	0
0	0	0	0	0	0	0	-0.015127673	0
0	-0.003307582	0	0	0	0	0	-0.007307149	0
0	0	0	0	0	0	0	-0.011573448	0
0	0.049563874	0	0	0	0	0	-0.011641659	0
0	0	0	0	0	0	0	-0.017372283	0
0	0.053065906	0	-0.053201941	0	0	0	0.001398891	0
0	0.047263985	0	-0.053375439	0	0	0	0	0
0	0	0	-0.053259892	0	0	0	0.001436118	0
0	0.016730109	0	0	0	0	0	-0.011605469	0
0	0.046942503	0	-0.051841573	0	0	0	0.000502952	0
0	0	0	-0.042667244	0	0	0	0.001072135	0
0	-0.012871572	0	-0.042670844	0	0	0	0	0
0	0	0	0	0	0	0	0	0
0	0.043454225	0	-0.042827144	0	0	0	0.001100341	0
0	0.034790614	0	-0.043882191	0	0	0	-0.005602766	0
0	0.014902531	0	0	0	0	0	0	0
0	0	0	-0.043980865	0	0	0	-0.005601015	0
0	0.033755079	0	-0.042745938	0	0	0	0	0
0	0.05220567	0	-0.043116785	0	0	0	-0.00553745	0
0	0.033521398	0	-0.04315297	0	0	0	-0.005483702	0
0	0.051360775	0	-0.044011747	0	0	0	0	0
0	0.017578034	0	0	0	0	0	0	0
0	0	0	-0.04281305	0	0	0	0	0
0	0	0	-0.043221531	0	0	0	-0.005487592	0
0	0.051266006	0	-0.043313359	0	0	0	0	0
0	0	0	0	0	0	0	-0.006280441	0
0	0.049143066	0	0	0	0	0	-0.006273003	0
0	0.00263409	0	0	0	0	0	-0.007245209	0
0	-0.011001735	0	-0.039461029	0	0	0	4.95E-07	0
0	-8.30E-05	0	0	0	0	0	0	0
0	0.001178619	0	0	0	0	0	-0.007231444	0
0	0	0	0	0	-0.061181236	0	-0.020559739	0
0	-0.021851264	0	-0.040763768	0	0	0	0.003712366	0
0	0	0	-0.058579433	0	0	0	-0.003766534	0
0	0	0	0	0	-0.057671687	0	-0.019934499	0
0	0.053902141	0	-0.042101235	0	0	0	-0.0057836	0

0	0	0	0	0	-0.058958627	0	0	0
0	0	0	-0.042200439	0	0	0	-0.005726221	0
0	0.05105694	0	-0.039766663	0	0	0	-0.00601167	0
0	0.050631931	0	-0.042687066	0	0	0	-0.005535511	0
0	0.049961619	0	-0.042324965	0	0	0	0	0
0	0	0	-0.043042854	0	0	0	-0.008843668	0
0	0.040565723	0	0	0	0	0	-0.009499016	0
0	0	0	0	0	0	0	-0.009503091	0
0	0	0	0	0	0	0	-0.011505999	0
0	0.040175226	0	0	0	0	0	0	0
0	0.059349192	0	0	0	0	0	0	0
0	0	0	0	0	0	0	0	0
0	0	0	0	0	0	0	-0.00944137	0
0	0.035447452	0	0	0	0	0	-0.009483918	0
0	0.032892534	0	0	0	0	0	-0.009207753	0
0	0	0	0	0	0	0	0	0
0	0.036530486	0	0	0	0	0	0	0
0	0	0	0	0	0	0	-0.009236367	0
0	0.016591065	0	0	0	0	0	0	0
0	0.043792692	0	0.16862211	0	-0.24853756	0	0	0
0	0.060903848	0	0	0	0	0	-0.009435831	0
0	0.060541346	0	0	0	0	0	0	0
0	0	0	0	0	0	0	-0.009379249	0
0	0.058803915	0	0	0	0	0	-0.009416121	0
0	0	0	0	0	0	0	-0.009665795	0
0	0	0	-0.051970034	0	0	0	-0.003312228	0
0	0.054902228	0	0	0	0	0	-0.009751636	0
0	0.057602054	0	0	0	0	0	0	0
0	0	0	0	0	0	0	-0.013039721	0
0	0.015535302	0	0	0	0	0	-0.009417003	0
0	0	0	0	0	0	0	-0.00921209	0
0	0.049824283	0	0	0	0	0	-0.009280323	0
0	0.021172948	0	0	0	0	0	-0.009276345	0
0	0.016010561	0	0	0	0	0	0	0
0	0	0	0	0	0	0	-0.013022169	0
0	0.016828535	0	0	0	0	0	-0.009317685	0
0	0.040768702	0	-0.049188212	0	0	0	0.012979711	0
0	0	0	-0.049250792	0	0	0	0.012993696	0
0	0.043252027	0	-0.049743003	0	0	0	0.01261278	0
0	0.06022066	0	0	0	0	0	-0.009170509	0
0	0.017188441	0	0	0	0	0	0	0
0	0.063236309	0	0	0	0	0	-0.008979125	0
0	0.044154245	0	-0.050671051	0	0	0	0	0
0	0	0	-0.037853416	0	0	0	-0.02372539	0
0	0	0	-0.03501847	0	0	0	-0.023795668	0
0	0.042196312	0	-0.04470506	0	0	0	0.011755646	0
0	0	0	-0.041667503	0	0	0	-0.022294146	0

0	0.038885068	0	0	0	-0.077030266	0	0.026433143	0
0	0.033698579	0	0	0	0	0	-0.007001051	0
0	0	0	0	0	0	0	-0.007030939	0
0	0.062545562	0	0	0	0	0	0	0
0	0.063377354	0	0	0	0	0	-0.006705448	0
0	0.044060003	0	-0.062750989	0	0	0	0.010060857	0
0	0.069699818	0	-0.062732291	0	0	0	0.010040686	0
0	0	0	-0.062751145	0	0	0	0.010076522	0
0	-0.012219672	0	0	0	0	0	0	0
0	0	0	0	0	0	0	0.000436298	0
0	-0.012775808	0	0	0	0	0	0.000705722	0
0	0.046057711	0	-0.064958655	0	0	0	0.011665726	0
0	-0.012244702	0	0	0	0	0	0.000443143	0
0	-0.015131186	0	0	0	0	0	0.001309227	0
0	0.063449121	0	0	0	0	0	0.000489305	0
0	0	0	-0.038369666	0	0	0	-0.021204816	0
0	0.055204994	0	0	0	0	0	0.002075488	0
0	0.072166118	0	0	0	0	0	0.002046103	0
0	0.062063313	0	0	0	0	0	-0.007054035	0
0	0.058289038	0	0	0	0	0	0	0
0	0	0	0	0	0	0	0.002100671	0
0	0	0	-0.03968894	0	0	0	0	0
0	0	0	-0.042532703	0	0	0	-0.021677679	0
0	0.047186436	0	-0.06465621	0	0	0	0	0
0	0.06433612	0	0	0	0	0	-0.006500812	0
0	0.031722633	0	0.17978979	0	-0.25574132	0	0.056100729	0
0	0	0	0	0	0	0	-0.025913637	0
0	0.05968342	0	0	0	0	0	0.007170727	0
0	0.05441033	0	0	0	0	0	0.016954287	0
0	0.042205465	0	-0.062094145	0	0	0	0.027446748	0
0	0.061257268	0	0	0	0	0	0.020398727	0
0	0	0	0.20926163	0	-0.2590203	0	0	0
0	0.026938172	0	0.20600161	0	-0.25562087	0	0	0
0	0	0	0.20598244	0	-0.25557992	0	0	0
0	0	0	0.20411384	0	-0.25116465	0	0	0
0	0.024455037	0	0.20410348	0	-0.25122583	0	0	0
0	0	0	0.20697535	0	-0.26234009	0	0	0
0	0	0	0.20316547	0	-0.25586249	0	0	0
0	0.033703874	0	0.20314489	0	-0.25592659	0	0	0
0	0	0	0.20391462	0	-0.25170572	0	0	0
0	0.062213824	0	0.20386036	0	-0.2517585	0	0	0
0	0	0	0.20237504	0	-0.25204164	0	-0.002962596	0
0	0	0	0.20597064	0	-0.25965704	0	-0.01052972	0
0	0.02759845	0	0.20236344	0	-0.25210292	0	-0.002954262	0
0	-0.000762042	0	0.20291811	0	-0.2527085	0	0	0
0	0	0	0.20814204	0	-0.25886017	0	0	0
0	0	0	0.20427925	0	-0.25490808	0	-0.004938016	0

0	0	0	0.2006992	0	-0.25028752	0	-0.006797537	0
0	0.055607944	0	0.20064768	0	-0.25033484	0	-0.006805941	0
0	0.020961816	0	0.20476833	0	-0.25515788	0	0	0
0	0	0	0.20476663	0	-0.2552526	0	0	0
0	0.012181792	0	0.20183783	0	-0.25116163	0	0	0
0	0	0	0.20198605	0	-0.25108252	0	0	0
0	0	0	0.2028887	0	-0.25938153	0	-0.006443105	0
0	0.057657876	0	0.202002	0	-0.2510937	0	0	0
0	0.044559524	0	0.19773493	0	-0.26002148	0	0	0
0	0	0	0.19769329	0	-0.26004754	0	0	0
0	0	0	0.20501784	0	-0.2578911	0	-0.008033843	0
0	0	0	0.20578062	0	-0.26199599	0	0	0
0	0.023672616	0	0.20253245	0	-0.25551977	0	-0.005420016	0
0	0	0	0.20251649	0	-0.25561466	0	-0.005445041	0
0	0.026965589	0	0.20285887	0	-0.25682576	0	0	0
0	0.049184057	0	0.20321932	0	-0.25641432	0	0	0
0	0.025140382	0	0.20898685	0	-0.25916873	0	-0.005639436	0
0	0	0	0.20897123	0	-0.25913677	0	-0.005616521	0
0	0	0	0.20285293	0	-0.25688361	0	0	0
0	0	0	0.20898755	0	-0.26059781	0	0	0
0	0.029190514	0	0.20902698	0	-0.2606342	0	0	0
0	0.027583162	0	0.20901821	0	-0.25988807	0	0	0
0	0	0	0.20899761	0	-0.25984491	0	0	0
0	0.015864537	0	0.20957455	0	-0.25997818	0	0	0
0	0	0	0.20240791	0	-0.24228304	0	-0.008223931	0
0	0.054303786	0	0.20241756	0	-0.24226977	0	-0.008316283	0
0	0	0	0.2033816	0	-0.24301125	0	0	0
0	0.010991624	0	0.20326901	0	-0.24345581	0	0	0
0	0.062447427	0	0.19937938	0	-0.25395787	0	0	0
0	0.057330196	0	0.20339491	0	-0.24304584	0	0	0
0	0	0	0.19929683	0	-0.25398633	0	0	0
0	0.063085263	0	0.19663796	0	-0.25508458	0	-0.002896046	0
0	0	0	0.20396172	0	-0.25836513	0	-0.007832981	0
0	0.04445081	0	0.19727653	0	-0.25532616	0	0	0
0	0	0	0.19659023	0	-0.25510931	0	-0.00283193	0
0	0	0	0.21051206	0	-0.26259313	0	-0.008196368	0
0	0	0	0.19988329	0	-0.24027683	0	-0.003831896	0
0	-0.004145978	0	0.19987506	0	-0.24035331	0	-0.003837865	0
0	0	0	0.20979385	0	-0.26454607	0	-0.008525131	0
0	-0.002900919	0	0.20057219	0	-0.24108123	0	0	0
0	0.056618273	0	0.19980694	0	-0.24026174	0	-0.003828666	0
0	0	0	0.21102307	0	-0.263259	0	0	0
0	0	0	0.20427755	0	-0.26232494	0	0	0
0	-0.001464412	0	0.20417088	0	-0.26216555	0	0	0
0	0	0	0.20419763	0	-0.26218573	0	0.000163749	0
0	0	0	0.20470978	0	-0.25525161	0	-0.00556832	0
0	0.042859546	0	0.20425789	0	-0.26245412	0	0	0

0	0.042743174	0	0.20418587	0	-0.26232454	0	0.000193306	0
0	0.055230495	0	0.20471797	0	-0.25524995	0	-0.005657519	0
0	0.012937295	0	0.20520929	0	-0.25606248	0	0	0
0	0	0	0.20512697	0	-0.25590936	0	0	0
0	0.054952862	0	0.19845098	0	-0.25140854	0	-0.005371401	0
0	0.056928935	0	0.20513383	0	-0.25589983	0	0	0
0	0	0	0.19838609	0	-0.25144273	0	-0.005306743	0
0	0.047066275	0	0.19904271	0	-0.25226393	0	0	0
0	0	0	0.20299478	0	-0.25837633	0	-0.001655619	0
0	0.002485214	0	0.20293253	0	-0.25829231	0	-0.001651582	0
0	0.002979446	0	0.20325347	0	-0.25848863	0	0	0
0	0.057423817	0	0.20293978	0	-0.25845111	0	-0.001654289	0
0	0	0	0.20453655	0	-0.24878171	0	-0.006282228	0
0	0.052542558	0	0.20453496	0	-0.24878019	0	-0.006367997	0
0	0.012908005	0	0.20512673	0	-0.24957703	0	0	0
0	0.025435054	0	0.20553023	0	-0.2602507	0	-0.003364784	0
0	0	0	0.19686859	0	-0.26666055	0	-0.001652964	0
0	0.027855335	0	0.20562707	0	-0.26143463	0	-0.003169647	0
0	0.028247722	0	0.20571281	0	-0.26137719	0	0	0
0	0.050354205	0	0.20595694	0	-0.26078748	0	0	0
0	0.05009521	0	0.20601428	0	-0.26192302	0	0	0
0	0	0	0.20552055	0	-0.26034614	0	-0.003384957	0
0	0.046463099	0	0.19335314	0	-0.25858622	0	0.003417083	0
0	0	0	0.20562402	0	-0.26149481	0	-0.003186254	0
0	0.050835202	0	0.20641487	0	-0.2604689	0	-0.003331267	0
0	0	0	0.20570715	0	-0.26143284	0	0	0
0	0	0	0.19330711	0	-0.25860403	0	0.003446333	0
0	0.04632915	0	0.19245674	0	-0.25810581	0	0	0
0	0.011248867	0	0.20619695	0	-0.24712877	0	-0.006201305	0
0	0.047669865	0	0.19962914	0	-0.24309919	0	-0.005978265	0
0	0.054035292	0	0.19963308	0	-0.24310868	0	-0.005994697	0
0	0	0	0.20316793	0	-0.25683132	0	0.003819306	0
0	0	0	0.19957076	0	-0.24313453	0	-0.005933166	0
0	0.034621502	0	0.20315541	0	-0.25696253	0	0.003838796	0
0	-0.015655228	0	0.20244835	0	-0.25610586	0	0	0
0	0.045627807	0	0.1931013	0	-0.25556831	0	0.001641293	0
0	0.046685424	0	0.20029829	0	-0.24401728	0	0	0
0	0	0	0.20704546	0	-0.26311178	0	-0.005911361	0
0	-0.023566586	0	0.20500647	0	-0.25675105	0	0.006470284	0
0	0.054562128	0	0.20173525	0	-0.25650002	0	-0.003380122	0
0	0.04871474	0	0.20195077	0	-0.2569608	0	-0.003271273	0
0	0.048253241	0	0.20208313	0	-0.25701059	0	0	0
0	0	0	0.20167459	0	-0.25654166	0	-0.003316287	0
0	-0.011731282	0	0.20075139	0	-0.24886768	0	0.002005746	0
0	0	0	0.20502329	0	-0.26876705	0	-0.001225971	0
0	0.049170675	0	0.20162552	0	-0.24997214	0	-0.004043394	0
0	0	0	0	0	-0.05725449	0	0	0

0	0.034846071	0	0	0	-0.057329879	0	0	0
0	0.033265422	0	0.18758941	0	-0.24811257	0	0.01645317	0
0	0.041506072	0	0.18756093	0	-0.24832231	0	0.016300449	0
0	0	0	0.18756178	0	-0.24815338	0	0.016459333	0
0	0.044694159	0	0.19414555	0	-0.2683939	0	0.01116506	0
0	0.061581305	0	0.19415841	0	-0.26847081	0	0.011181723	0
0	0.044761909	0	0.19392936	0	-0.26812615	0	0.011190858	0
0	0	0	0.19410584	0	-0.26844951	0	0.011214444	0
0	0	0	0	0	-0.052496689	0	0	0
0	0.024181518	0	0	0	-0.05251918	0	0	0
0	0	0	0.2000197	0	-0.25031813	0	-0.021080047	0
0	0	0	0	0	-0.055010772	0	-0.012050509	0
0	0	0	0.19888425	0	-0.25211928	0	-0.020333342	0
0	0.025700603	0	0	0	-0.054900237	0	-0.009105651	0
0	0	0	0	0	-0.054879977	0	-0.009080099	0
0	0	0	0	0	-0.061085989	0	-0.011039194	0
0	0	0	0	0	-0.05276195	0	-0.005012493	0
0	0.024724857	0	0	0	-0.052830773	0	-0.005007327	0
0	0.046097974	0	0.19109875	0	-0.26753204	0	0	0
0	0	0	0	0	-0.053898844	0	-0.011126148	0
0	0	0	0	0	-0.058315411	0	-0.006926299	0
0	0.058594674	0	0	0	-0.053997896	0	-0.011129211	0
0	0.033602425	0	0	0	-0.058397679	0	-0.006915118	0
0	-0.001770783	0	0	0	-0.052985486	0	0	0
0	0	0	0	0	-0.05652408	0	0	0
0	0.030796019	0	0	0	-0.056524894	0	0	0
0	0	0	0	0	-0.057107736	0	0	0
0	0.013640272	0	0	0	-0.055323453	0	0	0
0	0.063609004	0	0	0	-0.057096725	0	0	0
0	0	0	0	0	-0.058950757	0	0	0
0	0.008958514	0	0	0	-0.058223788	0	0	0
0	0.0429794	0	0.18109912	0	-0.24327894	0	0	0
0	0	0	0.20072208	0	-0.24525489	0	-0.021023057	0
0	-3.83E-05	0	0	0	-0.055174759	0	-0.005113699	0
0	0.017725616	0	0	0	-0.053648455	0	0	0
0	0	0	0	0	-0.056719145	0	-0.011765263	0
0	0.03556463	0	0	0	-0.06172715	0	0	0
0	0	0	0	0	-0.061794867	0	0	0
0	0.012132066	0	0	0	-0.055290515	0	-0.008802387	0
0	0	0	0	0	-0.055197467	0	-0.008878282	0
0	0.040512073	0	0.18513286	0	-0.23947405	0	0.014744215	0
0	0.043944737	0	0	0	-0.06674431	0	-0.003283931	0
0	0.054727137	0	0	0	-0.055186235	0	-0.008968866	0
0	0	0	0	0	-0.066801846	0	-0.003256544	0
0	0.042546232	0	0	0	-0.066426782	0	0	0
0	0.020792527	0	0	0	-0.055898235	0	-0.007313729	0
0	0	0	0	0	-0.055989956	0	-0.00734208	0

0	0.012930637	0	0	0	-0.055780013	0	0	0
0	0	0	0.20243783	0	-0.25349991	0	-0.018889851	0
0	0	0	0.2022547	0	-0.25454344	0	0	0
0	0.04539107	0	0	0	-0.056235695	0	0	0
0	0	0	0	0	-0.062124217	0	-0.010275414	0
0	0.028136556	0	0	0	-0.059967491	0	-0.006825122	0
0	0	0	0.19733322	0	-0.25235182	0	-0.019236646	0
0	0	0	0	0	-0.0600275	0	-0.006835384	0
0	0.047192728	0	0	0	-0.058374982	0	-0.007003001	0
0	0.060134602	0	0	0	-0.060546078	0	-0.007094141	0
0	0	0	0	0	-0.060655306	0	-0.00700661	0
0	0.04587894	0	0	0	-0.060291807	0	0	0
0	0.042657403	0	0	0	-0.063009287	0	-0.005375263	0
0	0.042256079	0	0	0	-0.060925232	0	0	0
0	0	0	0	0	-0.045561833	0	-0.00963156	0
0	0.010559854	0	0	0	-0.045922222	0	-0.009588612	0
0	0.054024889	0	0	0	-0.045574247	0	-0.009716422	0
0	0.01005742	0	0	0	-0.04611604	0	0	0
0	0	0	0	0	-0.056833928	0	-0.006970158	0
0	0.02773613	0	0	0	-0.056857348	0	-0.006998712	0
0	0	0	0	0	-0.058162488	0	-0.006736097	0
0	0.03064743	0	0	0	-0.058162168	0	-0.006773243	0
0	0.012007941	0	0	0	-0.05599981	0	-0.006889638	0
0	-0.002196221	0	0	0	-0.046500688	0	-0.005916302	0
0	0	0	0	0	-0.058281535	0	0	0
0	0.032144603	0	0	0	-0.05827798	0	0	0
0	0.013758528	0	0	0	-0.058558971	0	0	0
0	0.013696637	0	0	0	-0.057140824	0	0	0
0	0.045251759	0	0	0	-0.059202318	0	-0.006776597	0
0	0	0	0	0	-0.058485294	0	-0.009795849	0
0	0	0	0	0	-0.063656715	0	-0.001883561	0
0	0.000166548	0	0	0	-0.063716275	0	-0.001881093	0
0	0.043711411	0	0	0	-0.069326525	0	0.000546599	0
0	0.000639817	0	0	0	-0.063568393	0	0	0
0	0.043490095	0	0	0	-0.063804252	0	-0.001852984	0
0	0	0	0	0	-0.057135844	0	-0.006838268	0
0	0.012476855	0	0	0	-0.057397181	0	-0.006744149	0
0	0.054231708	0	0	0	-0.057114384	0	-0.006930631	0
0	0.012489835	0	0	0	-0.05756644	0	0	0
0	0.003631867	0	0	0	-0.061289194	0	-0.003472945	0
0	0.012675489	0	0	0	-0.050963752	0	-0.007513402	0
0	0	0	-0.038981811	0	0	0	0	0
0	0.040145915	0	-0.039074886	0	0	0	0	0
0	0.045003368	0	0	0	-0.049535704	0	-0.007414476	0
0	0	0	-0.035194865	0	0	0	0	0
0	0.031246981	0	-0.035229108	0	0	0	0	0
0	0.029954989	0	0	0	-0.061859465	0	-0.004671721	0

0	0.048115456	0	0	0	-0.060629319	0	-0.004859439	0
0	0	0	0	0	-0.061921373	0	-0.004677915	0
0	0.047649899	0	0	0	-0.062293337	0	-0.004549717	0
0	0.047118877	0	0	0	-0.062068397	0	0	0
0	-0.011932512	0	0	0	-0.059441619	0	0.001593833	0
0	0	0	-0.035704103	0	0	0	-0.006033089	0
0	0.031437328	0	-0.035786216	0	0	0	-0.006026139	0
0	0	0	-0.036516084	0	0	0	-0.011105951	0
0	-0.001273889	0	-0.035881354	0	0	0	0	0
0	0	0	-0.038340377	0	0	0	-0.010142644	0
0	0.033549401	0	-0.038372198	0	0	0	-0.010181417	0
0	0.060227088	0	-0.036623057	0	0	0	-0.011114711	0
0	0	0	-0.038466057	0	0	0	-0.01405335	0
0	0	0	-0.037151753	0	0	0	0	0
0	0.033338421	0	-0.037149239	0	0	0	0	0
0	0	0	-0.037901014	0	0	0	0	0
0	0.01470986	0	-0.038740054	0	0	0	0	0
0	0.064241196	0	-0.037884625	0	0	0	0	0
0	0.014537845	0	-0.0361512	0	0	0	0	0
0	0	0	-0.040313252	0	0	0	-0.007145297	0
0	0.038813207	0	-0.040411312	0	0	0	-0.007135969	0
0	0.04622013	0	0	0	-0.061118545	0	-0.004714054	0
0	0.001133469	0	-0.038779426	0	0	0	-0.006197996	0
0	0.006421052	0	-0.040126271	0	0	0	0	0
0	0	0	-0.043270388	0	0	0	-0.011615247	0
0	0	0	-0.039158712	0	0	0	0	0
0	0	0	-0.037227776	0	0	0	-0.013288618	0
0	0	0	-0.03619555	0	0	0	-0.010161567	0
0	0.055303903	0	-0.036179037	0	0	0	-0.010251307	0
0	0	0	-0.02966981	0	0	0	-0.010721058	0
0	0.008784199	0	-0.038048099	0	0	0	-0.008661911	0
0	0.056006914	0	-0.029683569	0	0	0	-0.010811663	0
0	0.012445794	0	-0.031914192	0	0	0	-0.01045902	0
0	0.012965119	0	-0.036747932	0	0	0	0	0
0	0.037806683	0	-0.042608703	0	0	0	0	0
0	0.010875551	0	-0.030187599	0	0	0	0	0
0	0.027464831	0	-0.039056487	0	0	0	-0.008298217	0
0	0	0	-0.04267763	0	0	0	0	0
0	0	0	-0.039149859	0	0	0	-0.00831645	0
0	-0.002282249	0	-0.030801937	0	0	0	-0.006732989	0
0	0.049268205	0	-0.039349002	0	0	0	0	0
0	0.03033751	0	-0.041081363	0	0	0	-0.007921845	0
0	0.051346991	0	-0.042180279	0	0	0	-0.007903233	0
0	0.039534749	0	0	0	-0.064827944	0	0.012857077	0
0	0	0	-0.04114405	0	0	0	-0.007935162	0
0	0.060293685	0	-0.041813084	0	0	0	-0.008170518	0
0	0	0	-0.041923082	0	0	0	-0.008092095	0

0	0.051205871	0	-0.050349349	0	0	0	-0.002555038	0
0	0.050134787	0	-0.04136596	0	0	0	0	0
0	0.040658616	0	0.18324604	0	-0.25635569	0	0.030245233	0
0	0.046577466	0	-0.050069663	0	0	0	0	0
0	0	0	-0.042866682	0	0	0	-0.011557391	0
0	0	0	-0.05040743	0	0	0	-0.002522517	0
0	0.046199011	0	-0.042147737	0	0	0	0	0
0	0	0	-0.039684521	0	0	0	-0.007940965	0
0	0.035209506	0	-0.039718092	0	0	0	-0.007983656	0
0	0	0	0	0	0	0	-0.012251058	0
0	0.039186333	0	0	0	0	0	-0.012305641	0
0	0	0	-0.038533477	0	0	0	-0.007929018	0
0	0.032976299	0	-0.038529413	0	0	0	-0.007969515	0
0	0.01493559	0	-0.039144904	0	0	0	-0.00789288	0
0	0.046106764	0	-0.047802216	0	0	0	-0.004054075	0
0	0	0	-0.038495775	0	0	0	0	0
0	0.034336502	0	-0.038489019	0	0	0	0	0
0	0.015018399	0	-0.039960518	0	0	0	0	0
0	0.015449864	0	-0.038923722	0	0	0	0	0
0	0	0	0	0	0	0	0	0
0	0.042568691	0	0	0	-0.078350093	0	0.008353572	0
0	0.040605402	0	0	0	0	0	0	0
0	0.049712728	0	-0.040644072	0	0	0	-0.007870809	0
0	0.01248343	0	0	0	0	0	0	0
0	0	0	0	0	0	0	-0.008950479	0
0	0.048610596	0	0	0	0	0	-0.008945539	0
0	0.049146012	0	-0.034061651	0	0	0	-0.008325858	0
0	0	0	-0.03768256	0	0	0	-0.008089237	0
0	0.054335574	0	-0.037662258	0	0	0	-0.008179249	0
0	0.013953558	0	-0.035576203	0	0	0	-0.008311524	0
0	0.013452001	0	-0.038101704	0	0	0	-0.007920857	0
0	0.031976994	0	0	0	0	0	-0.011910565	0
0	0	0	0	0	0	0	-0.01187589	0
0	0	0	-0.038547735	0	0	0	-0.011249618	0
0	0.003952153	0	0	0	0	0	0	0
0	-0.004173006	0	0	0	0	0	-0.006887316	0
0	0	0	0	0	0	0	-0.012098421	0
0	0.01366729	0	-0.038105545	0	0	0	0	0
0	0.061415366	0	0	0	0	0	-0.012200369	0
0	0.012665228	0	0	0	0	0	-0.012057477	0
0	0	0	0	0	-0.057263367	0	-0.022828276	0
0	0.005715008	0	0	0	0	0	-0.010082225	0
0	0	0	0	0	0	0	-0.015805196	0
0	0.015534377	0	0	0	0	0	0	0
0	0.0124546	0	0	0	0	0	0	0
0	0	0	-0.045692901	0	0	0	-0.001640747	0
0	-0.002772214	0	-0.04559461	0	0	0	0	0

0	-0.003210564	0	-0.045814165	0	0	0	-0.00163861	0
0	0.049638878	0	-0.04586002	0	0	0	-0.001608498	0
0	-0.001000584	0	-0.044339132	0	0	0	-0.002625206	0
0	0.015293936	0	0	0	0	0	-0.011745656	0
0	0.01902863	0	0	0	0	0	-0.011572516	0
0	0.047086598	0	-0.053194529	0	0	0	0.00140674	0
0	-0.012444688	0	-0.042662542	0	0	0	0.001047289	0
0	0.031785742	0	-0.04253362	0	0	0	-0.005737681	0
0	0.052317812	0	-0.043893161	0	0	0	-0.00565013	0
0	0	0	-0.042597472	0	0	0	-0.005747756	0
0	0.051569175	0	-0.042720568	0	0	0	0	0
0	0.052204445	0	-0.043127872	0	0	0	-0.005536941	0
0	-0.001098262	0	0	0	0	0	-0.006281561	0
0	0.050947745	0	-0.042106418	0	0	0	-0.005774966	0
0	0.036987395	0	0	0	0	0	-0.009491861	0
0	0.060828878	0	0	0	0	0	-0.009555803	0
0	0	0	0	0	0	0	-0.009506098	0
0	0.059351202	0	0	0	0	0	0	0
0	0.033176905	0	0	0	0	0	-0.009747708	0
0	0	0	0	0	0	0	-0.009710771	0
0	0.016909448	0	0	0	0	0	-0.009443803	0
0	0.061993318	0	0	0	0	0	-0.009290629	0
0	0.017459436	0	0	0	0	0	0	0
0	0.059057447	0	0	0	0	0	-0.00943076	0
0	0.016375042	0	0	0	0	0	-0.009669261	0
0	0.017533386	0	0	0	0	0	-0.009215721	0
0	0.042900131	0	-0.049190873	0	0	0	0.012976778	0
0	0	0	-0.038179372	0	0	0	-0.02415135	0
0	0.063691677	0	0	0	0	0	-0.007087586	0
0	0.045926019	0	-0.062688457	0	0	0	0.010049841	0
0	-0.012112304	0	0	0	0	0	0.000421231	0
0	0.057991552	0	0	0	0	0	0.002058615	0
0	0	0	0.2060931	0	-0.25766184	0	0	0
0	0.033855149	0	0.20607533	0	-0.25772069	0	0	0
0	0.022042463	0	0.20792191	0	-0.25425605	0	0	0
0	0	0	0.20790696	0	-0.25422468	0	0	0
0	0	0	0.20799459	0	-0.25665085	0	-0.010491585	0
0	0.024603829	0	0.20518147	0	-0.2544367	0	-0.007694686	0
0	0	0	0.20516672	0	-0.25440723	0	-0.007672855	0
0	0	0	0.20550307	0	-0.26082936	0	-0.00898956	0
0	0	0	0.20897282	0	-0.26102826	0	0	0
0	0.014664382	0	0.20598808	0	-0.25557893	0	0	0
0	0.027297816	0	0.20592947	0	-0.25573138	0	0	0
0	0	0	0.20589947	0	-0.25570294	0	0	0
0	0	0	0.20359342	0	-0.25053331	0	-0.002837565	0
0	0.023704724	0	0.20358402	0	-0.25059555	0	-0.002836078	0
0	-0.004673895	0	0.2041199	0	-0.25117083	0	0	0

0	0	0	0.20250776	0	-0.25056535	0	-0.009263327	0
0	0.059680444	0	0.20245456	0	-0.25061563	0	-0.009269142	0
0	0	0	0.20231682	0	-0.25510671	0	-0.004765636	0
0	0.032957026	0	0.2023066	0	-0.25518015	0	-0.00475341	0
0	0	0	0.20240873	0	-0.25283756	0	0	0
0	0.006749593	0	0.20315905	0	-0.25586327	0	0	0
0	0.063662936	0	0.20242635	0	-0.25283913	0	0	0
0	0	0	0.20685148	0	-0.25682038	0	-0.010121973	0
0	0.016141358	0	0.20390382	0	-0.25171522	0	0	0
0	-0.001783404	0	0.2023779	0	-0.25204493	0	-0.002965785	0
0	0.032172942	0	0.2032782	0	-0.25867344	0	0	0
0	0	0	0.20325642	0	-0.25871688	0	0	0
0	0.019094114	0	0.20410649	0	-0.25425955	0	-0.005831375	0
0	0	0	0.20121225	0	-0.24983347	0	-0.007565153	0
0	0	0	0.20410644	0	-0.25435871	0	-0.005869818	0
0	0.010813129	0	0.20073098	0	-0.2503248	0	-0.006785589	0
0	0.055118205	0	0.20122704	0	-0.24983551	0	-0.00765807	0
0	0.048493644	0	0.20483206	0	-0.25524093	0	0	0
0	0.01275863	0	0.2019899	0	-0.25108158	0	0	0
0	0	0	0.20464156	0	-0.26039516	0	-0.008516447	0
0	0.044514239	0	0.1975844	0	-0.2599372	0	-0.00065266	0
0	0.045042596	0	0.19773391	0	-0.26002131	0	0	0
0	0	0	0.19754115	0	-0.25995736	0	-0.000626117	0
0	0.05009296	0	0.20257982	0	-0.25559269	0	-0.005491551	0
0	0.025165395	0	0.20225986	0	-0.25597754	0	-0.005369105	0
0	0	0	0.2022567	0	-0.25603262	0	-0.005394386	0
0	0.025884554	0	0.20845925	0	-0.2590905	0	-0.005685689	0
0	0	0	0.20844255	0	-0.2590568	0	-0.005661845	0
0	0.027368197	0	0.20851773	0	-0.25981133	0	-0.005569169	0
0	0	0	0.20848412	0	-0.25978153	0	-0.005540233	0
0	0.049116124	0	0.20292605	0	-0.25686722	0	0	0
0	0.014814315	0	0.20897737	0	-0.25913614	0	-0.005619578	0
0	0.028834518	0	0.20888166	0	-0.26035398	0	0	0
0	0	0	0.20885234	0	-0.26033001	0	0	0
0	0.014703401	0	0.20899021	0	-0.26059499	0	0	0
0	0.014926506	0	0.20900198	0	-0.25984422	0	0	0
0	0	0	0.20255537	0	-0.2417285	0	-0.00831289	0
0	0.011323224	0	0.20241153	0	-0.24227829	0	-0.008226492	0
0	0.05463149	0	0.20254503	0	-0.24173503	0	-0.008398006	0
0	0.060579224	0	0.19874253	0	-0.25306432	0	-0.005664439	0
0	0	0	0.19867265	0	-0.25310683	0	-0.005581217	0
0	0.010691141	0	0.20338453	0	-0.24301061	0	0	0
0	0.045190126	0	0.19935569	0	-0.25396233	0	0	0
0	0.044806649	0	0.19663209	0	-0.2550882	0	-0.002861166	0
0	0	0	0.21004024	0	-0.2617021	0	-0.008244559	0
0	-0.004223795	0	0.19988649	0	-0.24027937	0	-0.003839322	0
0	-0.001479117	0	0.20427898	0	-0.26232432	0	0	0

0	0	0	0.20429469	0	-0.26233056	0	0.00016334	0
0	-0.001425747	0	0.20419861	0	-0.26218487	0	0.000162016	0
0	0	0	0.20460285	0	-0.25504526	0	-0.005606288	0
0	0.01313353	0	0.20471311	0	-0.25525161	0	-0.00557019	0
0	0.042884735	0	0.20428778	0	-0.26247444	0	0.000196438	0
0	0.054975625	0	0.20460315	0	-0.25502287	0	-0.005699339	0
0	0.013019846	0	0.20512987	0	-0.25590645	0	0	0
0	0.047958444	0	0.19844346	0	-0.25141232	0	-0.005351613	0
0	0.002476461	0	0.20299112	0	-0.25837754	0	-0.001652768	0
0	0.013166157	0	0.2045392	0	-0.24877805	0	-0.006285258	0
0	0.027138116	0	0.20537275	0	-0.2608944	0	-0.003277494	0
0	0.050896484	0	0.20558584	0	-0.26032549	0	-0.003434287	0
0	0.050645314	0	0.20568922	0	-0.26146774	0	-0.003234267	0
0	0.050225796	0	0.20577517	0	-0.26140961	0	0	0
0	0	0	0.20536019	0	-0.26093758	0	-0.003302544	0
0	0.045896631	0	0.19335264	0	-0.25858501	0	0.003417583	0
0	0.047673684	0	0.19962713	0	-0.24310897	0	-0.005977035	0
0	-0.014114213	0	0.20318123	0	-0.2568395	0	0.003791288	0
0	0.048833351	0	0.20173	0	-0.25650512	0	-0.003363227	0
0	0	0	0	0	-0.057334189	0	-0.009321523	0
0	0.033382939	0	0	0	-0.057408256	0	-0.009310563	0
0	0	0	0	0	-0.057894013	0	0	0
0	0.036213225	0	0	0	-0.057905278	0	0	0
0	0.012418361	0	0	0	-0.057266848	0	0	0
0	0.041367993	0	0.18759713	0	-0.24813045	0	0.01644227	0
0	0.021130831	0	0	0	-0.051952711	0	-0.009616412	0
0	0	0	0	0	-0.051937913	0	-0.009595471	0
0	0.044696042	0	0.1941489	0	-0.26843108	0	0.011188171	0
0	0.011657829	0	0	0	-0.052493451	0	0	0
0	0	0	0	0	-0.058018064	0	-0.012547395	0
0	0	0	0.200098	0	-0.25085101	0	-0.021634345	0
0	0	0	0	0	-0.056512402	0	-0.009236014	0
0	0	0	0	0	-0.055930945	0	-0.008987424	0
0	0.027768923	0	0	0	-0.055928648	0	-0.009019274	0
0	0.014807836	0	0	0	-0.054870323	0	-0.009082674	0
0	0.060571742	0	0	0	-0.056490764	0	-0.009341495	0
0	-0.003500439	0	0	0	-0.052761828	0	-0.005018742	0
0	0.015974677	0	0	0	-0.053917901	0	-0.011111596	0
0	0.007630982	0	0	0	-0.058320955	0	-0.00691565	0
0	0.013846521	0	0	0	-0.056510399	0	0	0
0	0.009031873	0	0	0	-0.05710811	0	0	0
0	0.033198163	0	0	0	-0.06138042	0	-0.007124289	0
0	0	0	0	0	-0.061445644	0	-0.007116295	0
0	0.044016731	0	0	0	-0.061716994	0	0	0
0	0.012281681	0	0	0	-0.0551903	0	-0.008883048	0
0	0.042947898	0	0	0	-0.066743056	0	-0.00328299	0
0	0.046617794	0	0	0	-0.055914938	0	-0.007385451	0

0	0.047037728	0	0	0	-0.059944241	0	-0.00687987	0
0	0.043086935	0	0	0	-0.060568452	0	-0.007058701	0
0	0.010364736	0	0	0	-0.045558345	0	-0.009633465	0
0	0	0	0	0	-0.057846669	0	-0.006850212	0
0	0.029807044	0	0	0	-0.057838371	0	-0.006890024	0
0	0.014454095	0	0	0	-0.056828754	0	-0.006973407	0
0	0.014008615	0	0	0	-0.058157812	0	-0.006739022	0
0	0.015848648	0	0	0	-0.058251775	0	0	0
0	0.000177363	0	0	0	-0.063656439	0	-0.001883112	0
0	0.012771487	0	0	0	-0.057130025	0	-0.006841184	0
0	0	0	-0.039175155	0	0	0	-0.009581052	0
0	0.038613348	0	-0.039265246	0	0	0	-0.009572781	0
0	0	0	-0.034674023	0	0	0	-0.010756435	0
0	0.027769678	0	-0.034697974	0	0	0	-0.010788475	0
0	0	0	-0.03833354	0	0	0	0	0
0	0.038400059	0	-0.038325477	0	0	0	0	0
0	0.009916966	0	-0.038991731	0	0	0	0	0
0	0.01457784	0	-0.035192519	0	0	0	0	0
0	0.047894602	0	0	0	-0.061834044	0	-0.004724448	0
0	-0.003353081	0	-0.035703585	0	0	0	-0.006038976	0
0	0	0	-0.036591768	0	0	0	-0.0102816	0
0	0.02985368	0	-0.03658435	0	0	0	-0.01031805	0
0	0	0	-0.03733788	0	0	0	-0.010512093	0
0	0.012439249	0	-0.036530844	0	0	0	-0.011093546	0
0	0.06078558	0	-0.037316687	0	0	0	-0.010615655	0
0	0.015700058	0	-0.038334259	0	0	0	-0.01014637	0
0	0	0	-0.038305408	0	0	0	-0.014023869	0
0	0.01457047	0	-0.037146305	0	0	0	0	0
0	0.010450306	0	-0.037899579	0	0	0	0	0
0	0.005081264	0	-0.040318742	0	0	0	-0.007139945	0
0	0.013290766	0	-0.036188653	0	0	0	-0.010164603	0
0	0.011215894	0	-0.029665644	0	0	0	-0.010722019	0
0	0.035059203	0	-0.042298836	0	0	0	-0.00821432	0
0	0	0	-0.042367141	0	0	0	-0.008210414	0
0	0.048390674	0	-0.042591964	0	0	0	0	0
0	0.050633002	0	-0.039067491	0	0	0	-0.00836285	0
0	0.051461663	0	-0.041052254	0	0	0	-0.007982965	0
0	0.048037372	0	-0.041834614	0	0	0	-0.008136234	0
0	0.046339181	0	-0.050343752	0	0	0	-0.0025502	0
0	0	0	-0.038062587	0	0	0	-0.008118896	0
0	0.031552278	0	-0.038053203	0	0	0	-0.008157041	0
0	0	0	0	0	0	0	-0.012244726	0
0	0.036491487	0	0	0	0	0	-0.01229293	0
0	0.015325949	0	-0.039679046	0	0	0	-0.007945039	0
0	0.015563338	0	0	0	0	0	-0.012254961	0
0	0.015815119	0	-0.038527456	0	0	0	-0.007930256	0
0	0.016382455	0	-0.038488833	0	0	0	0	0

0	0.014252644	0	0	0	0	0	0	0
0	0.002439374	0	0	0	0	0	-0.008949187	0
0	0.01398997	0	-0.037676336	0	0	0	-0.008092402	0
0	0.01686415	0	0	0	0	0	-0.011879082	0
0	0.012807157	0	0	0	0	0	-0.012102111	0
0	-0.003163667	0	-0.045688924	0	0	0	-0.001644595	0
0	0.052567297	0	-0.042501279	0	0	0	-0.00579807	0
0	0.060832564	0	0	0	0	0	-0.009558811	0
0	0.017826116	0	0	0	0	0	-0.009714583	0
0	0	0	0.20493108	0	-0.256594	0	-0.007292648	0
0	0.032715152	0	0.20489953	0	-0.25664179	0	-0.007289751	0
0	0	0	0.20641985	0	-0.25760522	0	0	0
0	0.010608629	0	0.20608038	0	-0.25766087	0	0	0
0	0.032777341	0	0.20645669	0	-0.25763561	0	0	0
0	0.019430749	0	0.20708331	0	-0.25295562	0	-0.008258322	0
0	0	0	0.20707376	0	-0.25293161	0	-0.00824735	0
0	0.010737902	0	0.20789448	0	-0.25420388	0	0	0
0	0	0	0.20760814	0	-0.25889616	0	-0.010926508	0
0	0.014951678	0	0.20517281	0	-0.25440649	0	-0.007675982	0
0	0.024735198	0	0.20513785	0	-0.25445668	0	-0.007697991	0
0	0	0	0.20513654	0	-0.25445959	0	-0.007665091	0
0	0.013186255	0	0.20590658	0	-0.25571558	0	0	0
0	-0.005647454	0	0.20359826	0	-0.25053747	0	-0.002847625	0
0	0	0	0.20161725	0	-0.25155078	0	-0.007939571	0
0	0.06105073	0	0.20161839	0	-0.2515373	0	-0.008034056	0
0	0.014391639	0	0.20249898	0	-0.25057405	0	-0.009248566	0
0	0.005861219	0	0.20231596	0	-0.25511124	0	-0.004759088	0
0	0.009695438	0	0.20241011	0	-0.25283449	0	0	0
0	0.030301313	0	0.20264471	0	-0.25778312	0	-0.005670507	0
0	0	0	0.20262548	0	-0.25782756	0	-0.005678158	0
0	0.04723323	0	0.20332246	0	-0.25869733	0	0	0
0	0.049461871	0	0.20416738	0	-0.25433833	0	-0.005915295	0
0	0.013028268	0	0.20122727	0	-0.24984299	0	-0.007569626	0
0	0.045118592	0	0.19758479	0	-0.25993831	0	-0.000653295	0
0	0.050023406	0	0.20232199	0	-0.25600735	0	-0.005441234	0
0	0.026931491	0	0.20835336	0	-0.25948377	0	-0.005631208	0
0	0	0	0.20832077	0	-0.25945729	0	-0.005608844	0
0	0.014511788	0	0.20844095	0	-0.25904833	0	-0.005662376	0
0	0.014908571	0	0.20849522	0	-0.25978658	0	-0.005543491	0
0	0.014462924	0	0.20884108	0	-0.26030405	0	0	0
0	0.010986381	0	0.20254573	0	-0.24171715	0	-0.008310803	0
0	0.046117749	0	0.19872778	0	-0.25307549	0	-0.005623904	0
0	-0.001460558	0	0.20430805	0	-0.26234462	0	0.00016496	0
0	0.013249504	0	0.20460553	0	-0.25504182	0	-0.005609334	0
0	0.050800374	0	0.20542404	0	-0.26090689	0	-0.003351562	0
0	0.010849498	0	0	0	-0.0573449	0	-0.009311837	0
0	0	0	0	0	-0.05729027	0	-0.009355222	0

0	0.033075905	0	0	0	-0.057284897	0	-0.009402955	0
0	0.010695386	0	0	0	-0.057890782	0	0	0
0	0.012071797	0	0	0	-0.051934607	0	-0.009597986	0
0	0.009335612	0	0	0	-0.056508229	0	-0.009237821	0
0	0.014021044	0	0	0	-0.055926723	0	-0.008990086	0
0	0.045212311	0	0	0	-0.061363561	0	-0.007158664	0
0	0.015288488	0	0	0	-0.057837064	0	-0.006872872	0
0	0	0	-0.037763175	0	0	0	-0.010643466	0
0	0.034860619	0	-0.037758109	0	0	0	-0.010691544	0
0	0.008326062	0	-0.039183136	0	0	0	-0.009573402	0
0	0.012700002	0	-0.034670578	0	0	0	-0.010758664	0
0	0.011714637	0	-0.038326275	0	0	0	0	0
0	0.014942351	0	-0.036583792	0	0	0	-0.010286154	0
0	0.010477013	0	-0.037333247	0	0	0	-0.010513978	0
0	0.049784423	0	-0.042276182	0	0	0	-0.008256352	0
0	0.015149147	0	-0.038058959	0	0	0	-0.008122677	0
0	0.014527895	0	0	0	0	0	-0.012246807	0
0	0	0	0.20561549	0	-0.256304	0	-0.008037718	0
0	0.030126938	0	0.20564992	0	-0.2563326	0	-0.00807759	0
0	0.009282542	0	0.20491148	0	-0.25658439	0	-0.00730322	0
0	0.011575771	0	0.20641727	0	-0.2575945	0	0	0
0	0.012759613	0	0.20712574	0	-0.2529851	0	-0.008238673	0
0	0.014958426	0	0.20514023	0	-0.25445763	0	-0.007668894	0
0	0.01007722	0	0.20158002	0	-0.25152574	0	-0.007932876	0
0	0.048178675	0	0.20268934	0	-0.25780169	0	-0.005722208	0
0	0.015026532	0	0.20833329	0	-0.259464	0	-0.005605069	0
0	0.011010652	0	0	0	-0.057285361	0	-0.00935731	0
0	0.012070882	0	-0.037758279	0	0	0	-0.010645587	0
0	0.01378627	0	0.20561083	0	-0.25629203	0	-0.00804866	0

SUPPLEMENTARY TABLE 6

Model	BIC	Loglike	H2r	SE	bsnp_rs2938864	SE	bsnp_rs1001646
cov1	-0.2036	-466.662	0.4141379	0	-0.11730652	0	0
cov0	0	-470.278	0.4191907	0	0	0	0
cov5	1.0534	-467.29	0.415772	0	0	0	0
cov9	1.4954	-467.511	0.4097978	0	0	0	0
cov10	1.5331	-467.53	0.4103697	0	0	0	0
cov6	2.1785	-467.853	0.4148549	0	0	0	0
cov4	2.2903	-467.909	0.4129426	0	0	0	0
cov2	2.3505	-467.939	0.4155531	0	0	0	-0.09337137
cov11	3.5086	-468.518	0.4156561	0	0	0	0
cov5_10	3.9851	-465.242	0.4083374	0	0	0	0
cov5_9	4.1737	-465.336	0.4080858	0	0	0	0
cov1_10	5.0168	-465.757	0.4101466	0	-0.090243553	0	0
cov1_9	5.1213	-465.81	0.4099249	0	-0.089402998	0	0
cov4_10	5.134	-465.816	0.4060856	0	0	0	0
cov4_9	5.2307	-465.864	0.4057511	0	0	0	0
cov1_6	6.4553	-466.477	0.4145425	0	-0.17623011	0	0
cov1_3	6.5112	-466.505	0.4165893	0	-0.1205726	0	0
cov2_10	6.6503	-466.574	0.4104636	0	0	0	-0.064289399
cov2_9	6.6885	-466.593	0.4100647	0	0	0	-0.063355343
cov1_4	6.7364	-466.617	0.4134331	0	-0.10321563	0	0
cov1_11	6.7489	-466.624	0.4143902	0	-0.13161033	0	0
cov1_2	6.7759	-466.637	0.4141951	0	-0.13286559	0	0.0181323
cov1_5	6.7763	-466.637	0.4141993	0	-0.10039068	0	0
cov1_7	6.8217	-466.66	0.4142493	0	-0.11710126	0	0
cov6_10	6.8217	-466.66	0.4100576	0	0	0	0
cov1_8	6.8244	-466.661	0.4140756	0	-0.11741721	0	0
cov6_9	6.8921	-466.695	0.409732	0	0	0	0
cov7	6.9581	-470.243	0.4194849	0	0	0	0
cov3	6.988	-470.258	0.4200829	0	0	0	0
cov8	7	-470.264	0.4193874	0	0	0	0
cov10_11	7.0152	-466.757	0.4096663	0	0	0	0
cov9_11	7.0893	-466.794	0.4093075	0	0	0	0
cov2_5	7.8218	-467.16	0.4151871	0	0	0	-0.033054178
cov5_6	7.8841	-467.191	0.4151072	0	0	0	0
cov3_9	7.9461	-467.222	0.4120364	0	0	0	0
cov3_10	7.9514	-467.225	0.4127024	0	0	0	0
cov5_11	7.9699	-467.234	0.4152379	0	0	0	0
cov5_7	8.0474	-467.273	0.4160849	0	0	0	0
cov4_5	8.0605	-467.279	0.4152839	0	0	0	0
cov5_8	8.0738	-467.286	0.4159326	0	0	0	0
cov3_5	8.0816	-467.29	0.4156191	0	0	0	0
cov4_6	8.3431	-467.421	0.4129384	0	0	0	0
cov9_10	8.471	-467.485	0.4099483	0	0	0	0
cov8_9	8.5213	-467.51	0.4097195	0	0	0	0
cov7_9	8.5225	-467.51	0.4098571	0	0	0	0

cov8_10	8.5588	-467.529	0.4102883	0	0	0	0
cov7_10	8.5605	-467.529	0.4104263	0	0	0	0
cov4_11	8.6678	-467.583	0.4124936	0	0	0	0
cov2_6	8.707	-467.603	0.414715	0	0	0	-0.048961676
cov2_4	8.8387	-467.668	0.4135882	0	0	0	-0.050351059
cov3_6	8.9534	-467.726	0.4170516	0	0	0	0
cov6_11	9.0356	-467.767	0.4145654	0	0	0	0
cov2_11	9.0579	-467.778	0.4148818	0	0	0	-0.071036813
cov2_3	9.1392	-467.819	0.4177068	0	0	0	-0.096189046
cov6_7	9.1862	-467.842	0.4150952	0	0	0	0
cov6_8	9.2058	-467.852	0.4149268	0	0	0	0
cov3_4	9.2976	-467.898	0.4121663	0	0	0	0
cov4_8	9.3065	-467.902	0.412741	0	0	0	0
cov4_7	9.319	-467.909	0.4129098	0	0	0	0
cov2_7	9.3584	-467.928	0.4157835	0	0	0	-0.093020763
cov2_8	9.3771	-467.938	0.4156343	0	0	0	-0.093252023
cov3_11	10.1069	-468.303	0.4183156	0	0	0	0
cov7_11	10.28	-468.389	0.4162667	0	0	0	0
cov8_11	10.3706	-468.434	0.4161681	0	0	0	0
cov5_6_10	10.4687	-464.969	0.4076631	0	0	0	0
cov5_6_9	10.6938	-465.081	0.4073551	0	0	0	0
cov3_5_10	10.7638	-465.116	0.4101721	0	0	0	0
cov2_5_10	10.8867	-465.178	0.4079514	0	0	0	0.025310148
cov5_10_11	10.9651	-465.217	0.4083918	0	0	0	0
cov3_5_9	10.9762	-465.223	0.4098079	0	0	0	0
cov5_8_10	11.0045	-465.237	0.4081493	0	0	0	0
cov1_5_10	11.0047	-465.237	0.4081694	0	0.01073748	0	0
cov4_5_10	11.0128	-465.241	0.4082289	0	0	0	0
cov5_9_10	11.0137	-465.241	0.4083099	0	0	0	0
cov5_7_10	11.0142	-465.242	0.4083372	0	0	0	0
cov2_5_9	11.1023	-465.286	0.4077386	0	0	0	0.022373048
cov5_9_11	11.1534	-465.311	0.4081189	0	0	0	0
cov5_8_9	11.1945	-465.332	0.4079142	0	0	0	0
cov1_5_9	11.1988	-465.334	0.4079698	0	0.006920854	0	0
cov4_5_9	11.1993	-465.334	0.4079122	0	0	0	0
cov5_7_9	11.2027	-465.336	0.4081005	0	0	0	0
cov1_3_10	11.3213	-465.395	0.4130427	0	-0.091748991	0	0
cov1_6_10	11.4034	-465.436	0.4104929	0	-0.16625437	0	0
cov1_3_9	11.4668	-465.468	0.4127147	0	-0.090840976	0	0
cov1_6_9	11.5102	-465.49	0.4102366	0	-0.16512191	0	0
cov1_4_10	11.6008	-465.535	0.4078734	0	-0.054147119	0	0
cov1_4_9	11.7181	-465.594	0.4076425	0	-0.053600561	0	0
cov1_2_10	11.9645	-465.717	0.4101193	0	-0.10988901	0	0.023203897
cov3_4_10	11.991	-465.73	0.4076891	0	0	0	0
cov1_10_11	11.9984	-465.734	0.4103554	0	-0.10169048	0	0
cov1_8_10	12.0285	-465.749	0.4099016	0	-0.090486977	0	0
cov1_7_10	12.0448	-465.757	0.4100865	0	-0.090307056	0	0

cov1_9_10	12.0458	-465.757	0.4101355	0	-0.090181409	0	0
cov1_2_9	12.0778	-465.773	0.4099116	0	-0.10799491	0	0.021907368
cov4_10_11	12.078	-465.774	0.4062505	0	0	0	0
cov4_8_10	12.0859	-465.778	0.4055471	0	0	0	0
cov1_9_11	12.0975	-465.783	0.4101393	0	-0.10144581	0	0
cov3_4_9	12.1031	-465.786	0.4072711	0	0	0	0
cov2_4_10	12.1117	-465.79	0.4053279	0	0	0	0.018278688
cov4_7_10	12.131	-465.8	0.4057442	0	0	0	0
cov1_8_9	12.1345	-465.802	0.4096911	0	-0.089630651	0	0
cov4_9_10	12.1464	-465.808	0.4059225	0	0	0	0
cov1_7_9	12.1496	-465.809	0.4098754	0	-0.089453972	0	0
cov4_6_10	12.1595	-465.814	0.4061927	0	0	0	0
cov4_8_9	12.1873	-465.828	0.4052438	0	0	0	0
cov4_9_11	12.1879	-465.829	0.4059434	0	0	0	0
cov2_4_9	12.2141	-465.842	0.4050333	0	0	0	0.017236046
cov4_7_9	12.2307	-465.85	0.405439	0	0	0	0
cov4_6_9	12.2579	-465.864	0.4058438	0	0	0	0
cov1_7_8	12.568	-466.019	0.4136068	0	-0.11703478	0	0
cov7_8	12.7057	-469.602	0.4188778	0	0	0	0
cov2_3_10	12.9622	-466.216	0.4132714	0	0	0	-0.066130613
cov3_10_11	13.0132	-466.241	0.4127551	0	0	0	0
cov2_3_9	13.0365	-466.253	0.4127695	0	0	0	-0.065182516
cov3_9_11	13.1508	-466.31	0.4122912	0	0	0	0
cov3_6_10	13.1607	-466.315	0.412785	0	0	0	0
cov1_3_6	13.1943	-466.332	0.416869	0	-0.17745906	0	0
cov1_3_8	13.2139	-466.342	0.4175348	0	-0.12626461	0	0
cov3_6_9	13.2693	-466.369	0.4123559	0	0	0	0
cov1_3_7	13.3423	-466.406	0.4173643	0	-0.12504763	0	0
cov1_4_6	13.3833	-466.426	0.4137558	0	-0.16217942	0	0
cov1_6_11	13.4356	-466.452	0.4147469	0	-0.18567788	0	0
cov1_5_6	13.4384	-466.454	0.4145921	0	-0.15961716	0	0
cov1_2_6	13.4446	-466.457	0.4146063	0	-0.18942148	0	0.016243132
cov2_10_11	13.4563	-466.463	0.4100167	0	0	0	-0.046186482
cov1_6_8	13.4819	-466.476	0.4144521	0	-0.17651935	0	0
cov1_6_7	13.4828	-466.476	0.4146131	0	-0.17595789	0	0
cov2_6_10	13.487	-466.478	0.4102634	0	0	0	-0.041769214
cov1_2_3	13.4994	-466.484	0.4166036	0	-0.13465237	0	0.01645946
cov1_3_11	13.5131	-466.491	0.4165982	0	-0.12907928	0	0
cov2_9_11	13.5215	-466.495	0.4096883	0	0	0	-0.046529599
cov1_3_4	13.5318	-466.5	0.416195	0	-0.11566422	0	0
cov1_3_5	13.5399	-466.505	0.4166193	0	-0.12215617	0	0
cov2_6_9	13.5477	-466.508	0.4099321	0	0	0	-0.042306769
cov1_2_4	13.578	-466.524	0.4129789	0	-0.1262843	0	0.040828052
cov6_10_11	13.6384	-466.554	0.409777	0	0	0	0
cov2_9_10	13.6554	-466.562	0.4102421	0	0	0	-0.0635113
cov2_8_10	13.6718	-466.57	0.410318	0	0	0	-0.06437293
cov2_7_10	13.6792	-466.574	0.4104833	0	0	0	-0.064277265

cov1_4_11	13.6918	-466.58	0.4136977	0	-0.11747429	0	0
cov2_8_9	13.7106	-466.59	0.4099254	0	0	0	-0.063433181
cov2_7_9	13.7174	-466.593	0.4100903	0	0	0	-0.063339993
cov6_9_11	13.7242	-466.597	0.4094797	0	0	0	0
cov1_5_11	13.728	-466.599	0.4144603	0	-0.11463948	0	0
cov1_2_11	13.7369	-466.603	0.4144243	0	-0.14501646	0	0.01653912
cov1_2_5	13.7521	-466.611	0.4142646	0	-0.11589427	0	0.018804075
cov1_4_8	13.761	-466.615	0.4132923	0	-0.10308795	0	0
cov1_8_11	13.7642	-466.617	0.4141971	0	-0.13381069	0	0
cov1_4_5	13.7644	-466.617	0.4135149	0	-0.10121547	0	0
cov1_4_7	13.7647	-466.617	0.413493	0	-0.10327149	0	0
cov1_7_11	13.7776	-466.623	0.4143612	0	-0.13195398	0	0
cov1_5_7	13.7994	-466.634	0.4143406	0	-0.099734369	0	0
cov1_2_7	13.8016	-466.635	0.4143001	0	-0.13260636	0	0.018055344
cov1_2_8	13.8036	-466.636	0.4141266	0	-0.13302869	0	0.018179848
cov1_5_8	13.8051	-466.637	0.4141641	0	-0.10054818	0	0
cov5_7_8	13.8311	-466.65	0.4154725	0	0	0	0
cov6_9_10	13.8403	-466.655	0.4099164	0	0	0	0
cov6_8_10	13.844	-466.657	0.4099164	0	0	0	0
cov6_7_10	13.8503	-466.66	0.4100946	0	0	0	0
cov6_8_9	13.9152	-466.692	0.409595	0	0	0	0
cov6_7_9	13.9205	-466.695	0.4097762	0	0	0	0
cov7_10_11	13.9832	-466.726	0.4100364	0	0	0	0
cov3_7	13.9861	-470.242	0.4196453	0	0	0	0
cov3_8	14.0148	-470.257	0.419969	0	0	0	0
cov8_10_11	14.0233	-466.746	0.4098907	0	0	0	0
cov9_10_11	14.0252	-466.747	0.4094893	0	0	0	0
cov7_9_11	14.0582	-466.764	0.4096796	0	0	0	0
cov8_9_11	14.098	-466.784	0.4095318	0	0	0	0
cov7_8_9	14.138	-466.804	0.4090912	0	0	0	0
cov7_8_10	14.1766	-466.823	0.4096693	0	0	0	0
cov3_8_10	14.2105	-466.84	0.4137068	0	0	0	0
cov3_8_9	14.2586	-466.864	0.4129623	0	0	0	0
cov3_7_10	14.4318	-466.95	0.4136158	0	0	0	0
cov3_7_9	14.471	-466.97	0.412876	0	0	0	0
cov6_7_8	14.6967	-467.083	0.4142973	0	0	0	0
cov2_5_6	14.7972	-467.133	0.4149472	0	0	0	-0.025011852
cov2_5_11	14.824	-467.147	0.4149796	0	0	0	-0.028996142
cov2_5_7	14.8243	-467.147	0.415471	0	0	0	-0.032549522
cov2_3_5	14.8336	-467.151	0.4158329	0	0	0	-0.035778147
cov2_4_5	14.8387	-467.154	0.4155352	0	0	0	-0.036964731
cov2_5_8	14.8462	-467.158	0.4153094	0	0	0	-0.032839567
cov5_6_7	14.8855	-467.177	0.4153945	0	0	0	0
cov5_6_11	14.8876	-467.178	0.4149424	0	0	0	0
cov4_5_6	14.8912	-467.18	0.4146236	0	0	0	0
cov3_9_10	14.8951	-467.182	0.4122403	0	0	0	0
cov3_5_6	14.8962	-467.183	0.4157245	0	0	0	0

cov5_6_8	14.9084	-467.189	0.4152268	0	0	0	0
cov5_7_11	14.932	-467.201	0.4155992	0	0	0	0
cov4_5_11	14.9718	-467.22	0.4146819	0	0	0	0
cov5_8_11	14.9728	-467.221	0.4154741	0	0	0	0
cov3_5_11	14.9864	-467.228	0.4157736	0	0	0	0
cov3_5_7	15.0325	-467.251	0.4149824	0	0	0	0
cov4_5_7	15.0637	-467.266	0.4156649	0	0	0	0
cov4_5_8	15.0854	-467.277	0.4154403	0	0	0	0
cov3_4_5	15.0867	-467.278	0.4149825	0	0	0	0
cov3_5_8	15.0876	-467.278	0.4152856	0	0	0	0
cov2_7_8	15.0936	-467.281	0.4151288	0	0	0	-0.093082964
cov4_7_8	15.0984	-467.284	0.4123171	0	0	0	0
cov4_6_11	15.2817	-467.375	0.4127499	0	0	0	0
cov3_4_6	15.3341	-467.402	0.4140158	0	0	0	0
cov2_4_6	15.3366	-467.403	0.4131548	0	0	0	-0.016288701
cov4_6_8	15.3669	-467.418	0.4127964	0	0	0	0
cov4_6_7	15.3715	-467.42	0.41299	0	0	0	0
cov2_3_6	15.4509	-467.46	0.4170499	0	0	0	-0.050508661
cov8_9_10	15.4965	-467.483	0.4098647	0	0	0	0
cov7_9_10	15.4985	-467.484	0.4100024	0	0	0	0
cov2_4_11	15.5666	-467.518	0.4129721	0	0	0	-0.029563056
cov3_4_11	15.664	-467.567	0.4134741	0	0	0	0
cov4_7_11	15.6644	-467.567	0.4128161	0	0	0	0
cov2_6_11	15.6769	-467.573	0.4145231	0	0	0	-0.044539231
cov4_8_11	15.6903	-467.58	0.4126484	0	0	0	0
cov3_6_11	15.7078	-467.588	0.4171072	0	0	0	0
cov2_3_11	15.7105	-467.59	0.4174514	0	0	0	-0.069784663
cov2_6_7	15.7188	-467.594	0.4149349	0	0	0	-0.048771366
cov2_6_8	15.7351	-467.602	0.4147686	0	0	0	-0.048927749
cov3_6_8	15.8133	-467.641	0.4177966	0	0	0	0
cov2_3_4	15.8362	-467.653	0.4147119	0	0	0	-0.05674306
cov2_4_8	15.8646	-467.667	0.4134762	0	0	0	-0.050009919
cov2_4_7	15.8663	-467.668	0.4136648	0	0	0	-0.050592655
cov3_6_7	15.9084	-467.689	0.417569	0	0	0	0
cov7_8_11	15.9933	-467.731	0.4156192	0	0	0	0
cov6_7_11	16.0006	-467.735	0.4149571	0	0	0	0
cov2_7_11	16.0093	-467.739	0.4152753	0	0	0	-0.067847249
cov2_3_8	16.0183	-467.744	0.4184541	0	0	0	-0.099520799
cov6_8_11	16.0431	-467.756	0.414803	0	0	0	0
cov2_8_11	16.0549	-467.762	0.4151442	0	0	0	-0.068997382
cov2_3_7	16.101	-467.785	0.4182308	0	0	0	-0.098439349
cov3_4_7	16.3157	-467.892	0.4119262	0	0	0	0
cov3_4_8	16.3263	-467.898	0.4122164	0	0	0	0
cov5_7_8_10	16.6804	-464.56	0.4075795	0	0	0	0
cov5_7_8_9	16.8703	-464.655	0.4073344	0	0	0	0
cov3_7_11	17.1266	-468.298	0.4181203	0	0	0	0
cov3_8_11	17.1349	-468.302	0.4183806	0	0	0	0

cov1_3_8_10	17.2277	-464.834	0.4140383	0	-0.096451604	0	0
cov1_5_6_10	17.2772	-464.859	0.4084007	0	-0.066267135	0	0
cov3_5_8_10	17.3631	-464.902	0.4109058	0	0	0	0
cov3_5_6_10	17.3934	-464.917	0.4089594	0	0	0	0
cov1_3_8_9	17.4591	-464.95	0.4136322	0	-0.095244757	0	0
cov1_5_6_9	17.4798	-464.96	0.408163	0	-0.069537254	0	0
cov2_5_6_10	17.4831	-464.962	0.4075622	0	0	0	0.00908438
cov5_6_8_10	17.4888	-464.964	0.4074889	0	0	0	0
cov5_6_9_10	17.4913	-464.966	0.4075574	0	0	0	0
cov1_3_7_10	17.4923	-464.966	0.4139979	0	-0.095958955	0	0
cov5_6_10_11	17.4962	-464.968	0.4076384	0	0	0	0
cov4_5_6_10	17.4976	-464.969	0.4076277	0	0	0	0
cov5_6_7_10	17.4978	-464.969	0.4076609	0	0	0	0
cov3_5_7_10	17.5239	-464.982	0.4107984	0	0	0	0
cov3_5_8_9	17.6259	-465.033	0.410467	0	0	0	0
cov3_5_6_9	17.6314	-465.036	0.4085621	0	0	0	0
cov1_7_8_10	17.7084	-465.074	0.4093275	0	-0.089641078	0	0
cov1_3_7_9	17.712	-465.076	0.4135935	0	-0.094767337	0	0
cov5_6_8_9	17.7154	-465.078	0.4071965	0	0	0	0
cov2_5_6_9	17.7157	-465.078	0.4072857	0	0	0	0.006321059
cov1_3_6_10	17.7201	-465.08	0.4133153	0	-0.16703895	0	0
cov4_5_6_9	17.7214	-465.081	0.4072366	0	0	0	0
cov5_6_9_11	17.7222	-465.081	0.4073389	0	0	0	0
cov5_6_7_9	17.7228	-465.081	0.4073666	0	0	0	0
cov2_3_5_10	17.7303	-465.085	0.4096724	0	0	0	0.018199923
cov3_5_7_9	17.7757	-465.108	0.4103628	0	0	0	0
cov1_3_5_10	17.7847	-465.112	0.410465	0	-0.010635467	0	0
cov3_4_5_10	17.7903	-465.115	0.4103554	0	0	0	0
cov3_5_10_11	17.7906	-465.115	0.4101198	0	0	0	0
cov4_7_8_10	17.7906	-465.115	0.405015	0	0	0	0
cov3_5_9_10	17.7923	-465.116	0.4101411	0	0	0	0
cov1_7_8_9	17.8147	-465.127	0.4091068	0	-0.088742769	0	0
cov2_4_5_10	17.8529	-465.146	0.4069462	0	0	0	0.035653784
cov1_3_6_9	17.8687	-465.154	0.4129531	0	-0.16578559	0	0
cov4_7_8_9	17.8908	-465.165	0.4047019	0	0	0	0
cov2_5_10_11	17.8997	-465.17	0.4080184	0	0	0	0.022694531
cov1_4_6_10	17.9065	-465.173	0.4079062	0	-0.1317113	0	0
cov2_5_8_10	17.9068	-465.173	0.4077747	0	0	0	0.025242133
cov1_2_5_10	17.9076	-465.174	0.4080784	0	-0.011377212	0	0.02892871
cov2_5_9_10	17.9151	-465.178	0.4079182	0	0	0	0.025333082
cov2_5_7_10	17.9157	-465.178	0.4079578	0	0	0	0.025312729
cov2_3_5_9	17.9599	-465.2	0.4093791	0	0	0	0.015448191
cov5_8_10_11	17.9705	-465.205	0.4080994	0	0	0	0
cov1_3_5_9	17.9909	-465.215	0.4102148	0	-0.014180019	0	0
cov5_7_10_11	17.9914	-465.216	0.4082927	0	0	0	0
cov5_9_10_11	17.993	-465.216	0.4083452	0	0	0	0
cov4_5_10_11	17.9937	-465.217	0.4083273	0	0	0	0

cov1_5_10_11	17.9942	-465.217	0.4083949	0	-2.88E-05	0	0
cov3_5_9_11	18.0019	-465.221	0.40974	0	0	0	0
cov3_4_5_9	18.0048	-465.222	0.4098902	0	0	0	0
cov1_5_8_10	18.025	-465.233	0.4079992	0	0.010213936	0	0
cov1_4_6_9	18.0264	-465.233	0.4076335	0	-0.13080664	0	0
cov4_5_8_10	18.0303	-465.235	0.407956	0	0	0	0
cov1_5_9_10	18.0328	-465.236	0.4081261	0	0.011035644	0	0
cov1_4_5_10	18.033	-465.237	0.408094	0	0.010434725	0	0
cov5_8_9_10	18.0331	-465.237	0.4081214	0	0	0	0
cov1_5_7_10	18.0337	-465.237	0.4081784	0	0.010756823	0	0
cov4_5_9_10	18.0414	-465.241	0.4081972	0	0	0	0
cov4_5_7_10	18.0419	-465.241	0.4082101	0	0	0	0
cov5_7_9_10	18.0428	-465.241	0.4083096	0	0	0	0
cov1_2_4_10	18.0499	-465.245	0.4061576	0	-0.09007232	0	0.073302427
cov2_4_5_9	18.067	-465.254	0.4067062	0	0	0	0.032869875
cov2_5_9_11	18.112	-465.276	0.4078002	0	0	0	0.019519648
cov1_2_5_9	18.1195	-465.28	0.4079072	0	-0.013852971	0	0.026801994
cov2_5_8_9	18.1239	-465.282	0.4075759	0	0	0	0.022299028
cov2_5_7_9	18.1313	-465.286	0.4077602	0	0	0	0.022383111
cov5_8_9_11	18.1612	-465.301	0.4078416	0	0	0	0
cov5_7_9_11	18.1805	-465.31	0.4080352	0	0	0	0
cov4_5_9_11	18.1806	-465.31	0.4079861	0	0	0	0
cov1_5_9_11	18.1811	-465.311	0.408201	0	-0.004705464	0	0
cov1_3_4_10	18.1935	-465.317	0.4111144	0	-0.068620797	0	0
cov1_2_4_9	18.2104	-465.325	0.4060135	0	-0.088186756	0	0.070337541
cov4_5_8_9	18.2175	-465.329	0.407649	0	0	0	0
cov1_5_8_9	18.2202	-465.33	0.4078124	0	0.006419202	0	0
cov1_4_5_9	18.2251	-465.333	0.4078213	0	0.006375465	0	0
cov1_5_7_9	18.2278	-465.334	0.4079903	0	0.00698642	0	0
cov4_5_7_9	18.2284	-465.334	0.4079021	0	0	0	0
cov1_2_3_10	18.2823	-465.361	0.412968	0	-0.10971754	0	0.021237625
cov1_3_4_9	18.3412	-465.391	0.4107718	0	-0.067713802	0	0
cov1_3_10_11	18.3494	-465.395	0.4130518	0	-0.093437074	0	0
cov1_3_9_10	18.3504	-465.395	0.4130538	0	-0.091808391	0	0
cov3_4_8_10	18.3636	-465.402	0.4087481	0	0	0	0
cov1_2_6_10	18.3654	-465.403	0.4104916	0	-0.18329861	0	0.021085914
cov1_6_8_10	18.4065	-465.423	0.4102029	0	-0.1671115	0	0
cov1_6_10_11	18.4135	-465.427	0.4106315	0	-0.17220344	0	0
cov1_6_7_10	18.4276	-465.434	0.4103706	0	-0.16669267	0	0
cov1_6_9_10	18.4315	-465.436	0.4104513	0	-0.16610167	0	0
cov1_2_3_9	18.4365	-465.438	0.4126605	0	-0.10766192	0	0.019834306
cov1_2_6_9	18.4806	-465.46	0.4102457	0	-0.18106119	0	0.019704792
cov1_3_9_11	18.4932	-465.467	0.4127293	0	-0.093582795	0	0
cov1_6_8_9	18.5151	-465.478	0.4099578	0	-0.16593663	0	0
cov1_6_9_11	18.5166	-465.478	0.4103849	0	-0.17162371	0	0
cov3_4_8_9	18.5322	-465.486	0.4082467	0	0	0	0
cov1_6_7_9	18.5351	-465.488	0.4101251	0	-0.16551859	0	0

cov3_4_7_10	18.5648	-465.502	0.40863	0	0	0	0
cov1_4_8_10	18.5802	-465.51	0.4073688	0	-0.052944057	0	0
cov1_4_10_11	18.5908	-465.515	0.4080912	0	-0.064852679	0	0
cov1_4_7_10	18.6145	-465.527	0.4075989	0	-0.053479187	0	0
cov1_4_9_10	18.6289	-465.534	0.4078257	0	-0.053845942	0	0
cov1_4_8_9	18.7009	-465.57	0.407154	0	-0.052427578	0	0
cov1_4_9_11	18.7026	-465.571	0.4078669	0	-0.064991776	0	0
cov3_4_7_9	18.7233	-465.582	0.4081321	0	0	0	0
cov1_4_7_9	18.7337	-465.587	0.4073838	0	-0.052968093	0	0
cov3_4_10_11	18.8054	-465.623	0.4086488	0	0	0	0
cov3_4_9_11	18.9471	-465.694	0.4082043	0	0	0	0
cov1_2_10_11	18.9549	-465.697	0.4103054	0	-0.11919845	0	0.021969502
cov2_4_10_11	18.9656	-465.703	0.4050159	0	0	0	0.033098725
cov1_2_8_10	18.9747	-465.707	0.4098637	0	-0.11032473	0	0.023418981
cov1_8_10_11	18.9868	-465.713	0.4100236	0	-0.10519467	0	0
cov3_4_6_10	18.9916	-465.716	0.4082052	0	0	0	0
cov1_2_7_10	18.9921	-465.716	0.4100488	0	-0.11001599	0	0.023266248
cov1_2_9_10	18.9936	-465.717	0.4101227	0	-0.10991391	0	0.023211298
cov3_4_9_10	19.0026	-465.721	0.4075332	0	0	0	0
cov2_3_8_10	19.011	-465.726	0.4143776	0	0	0	-0.069678719
cov2_3_4_10	19.0142	-465.727	0.4072921	0	0	0	0.006651946
cov1_7_10_11	19.0175	-465.729	0.4101974	0	-0.10343846	0	0
cov1_9_10_11	19.0273	-465.734	0.4103365	0	-0.10160382	0	0
cov2_4_8_10	19.0445	-465.742	0.4046162	0	0	0	0.021579238
cov1_8_9_10	19.0575	-465.749	0.4098906	0	-0.090425166	0	0
cov1_2_9_11	19.0628	-465.751	0.4101035	0	-0.11792124	0	0.020603939
cov4_8_10_11	19.0642	-465.752	0.4057985	0	0	0	0
cov1_7_9_10	19.0738	-465.757	0.4100754	0	-0.090245061	0	0
cov1_8_9_11	19.087	-465.763	0.4098113	0	-0.10489636	0	0
cov1_2_8_9	19.0897	-465.765	0.4096682	0	-0.10840025	0	0.022103914
cov2_4_9_11	19.0952	-465.768	0.4047936	0	0	0	0.030619794
cov4_6_10_11	19.0954	-465.768	0.4060433	0	0	0	0
cov4_7_10_11	19.0962	-465.768	0.406028	0	0	0	0
cov2_4_7_10	19.0963	-465.768	0.4048532	0	0	0	0.020533713
cov4_9_10_11	19.0968	-465.768	0.406126	0	0	0	0
cov4_8_9_10	19.0986	-465.769	0.4053996	0	0	0	0
cov1_2_7_9	19.1058	-465.773	0.4098518	0	-0.10809955	0	0.021957521
cov3_4_6_9	19.1104	-465.775	0.4077435	0	0	0	0
cov2_4_6_10	19.1107	-465.775	0.4054376	0	0	0	0.024863518
cov1_3_7_8	19.113	-465.777	0.4166536	0	-0.12478411	0	0
cov4_6_8_10	19.1137	-465.777	0.4056219	0	0	0	0
cov1_7_9_11	19.1172	-465.779	0.4099849	0	-0.10314615	0	0
cov2_4_9_10	19.1207	-465.78	0.4051374	0	0	0	0.018888481
cov2_3_4_9	19.1273	-465.784	0.4069059	0	0	0	0.006039369
cov4_7_9_10	19.1436	-465.792	0.4055956	0	0	0	0
cov2_3_8_9	19.1524	-465.796	0.4137881	0	0	0	-0.068588727
cov2_4_8_9	19.1535	-465.797	0.4043493	0	0	0	0.020375798

cov4_6_7_10	19.1582	-465.799	0.405836	0	0	0	0
cov4_6_9_10	19.1735	-465.807	0.406017	0	0	0	0
cov4_8_9_11	19.1752	-465.808	0.4054945	0	0	0	0
cov2_4_7_9	19.2031	-465.822	0.4045861	0	0	0	0.019344982
cov4_6_9_11	19.2049	-465.822	0.4057203	0	0	0	0
cov4_7_9_11	19.2067	-465.823	0.4057253	0	0	0	0
cov4_6_8_9	19.216	-465.828	0.4052897	0	0	0	0
cov2_4_6_9	19.2211	-465.831	0.4051496	0	0	0	0.022807403
cov3_6_8_10	19.2588	-465.849	0.4137546	0	0	0	0
cov4_6_7_9	19.259	-465.85	0.4055047	0	0	0	0
cov2_3_7_10	19.2601	-465.85	0.4143057	0	0	0	-0.069258993
cov2_7_8_10	19.3183	-465.879	0.4097127	0	0	0	-0.063846046
cov6_7_8_10	19.3406	-465.89	0.4092785	0	0	0	0
cov2_7_8_9	19.3566	-465.898	0.4093103	0	0	0	-0.06288628
cov1_6_7_8	19.3614	-465.901	0.4139262	0	-0.16439677	0	0
cov2_3_7_9	19.3916	-465.916	0.4137203	0	0	0	-0.068171784
cov3_8_10_11	19.3956	-465.918	0.4134894	0	0	0	0
cov6_7_8_9	19.4146	-465.927	0.4089618	0	0	0	0
cov3_6_8_9	19.4386	-465.939	0.4132496	0	0	0	0
cov3_6_7_10	19.5141	-465.977	0.4136854	0	0	0	0
cov1_4_7_8	19.5173	-465.979	0.4128806	0	-0.10373747	0	0
cov1_7_8_11	19.5312	-465.986	0.4137137	0	-0.13111525	0	0
cov1_2_7_8	19.5501	-465.995	0.4136595	0	-0.13217718	0	0.017633144
cov1_5_7_8	19.5525	-465.996	0.413694	0	-0.10086186	0	0
cov2_3_10_11	19.5767	-466.008	0.4130177	0	0	0	-0.041259366
cov3_8_9_11	19.5903	-466.015	0.4129539	0	0	0	0
cov3_7_10_11	19.5978	-466.019	0.4134229	0	0	0	0
cov7_8_10_11	19.5983	-466.019	0.4092714	0	0	0	0
cov7_8_9_11	19.6743	-466.057	0.4089068	0	0	0	0
cov3_6_7_9	19.6822	-466.061	0.4131825	0	0	0	0
cov2_3_9_11	19.6976	-466.069	0.4125856	0	0	0	-0.041973142
cov3_7_8	19.7345	-469.602	0.4189563	0	0	0	0
cov3_6_10_11	19.7711	-466.106	0.4127696	0	0	0	0
cov3_7_9_11	19.7821	-466.111	0.41289	0	0	0	0
cov2_3_6_10	19.7963	-466.118	0.4131061	0	0	0	-0.043460357
cov2_3_6_9	19.8942	-466.167	0.4126716	0	0	0	-0.04403977
cov1_3_6_8	19.896	-466.168	0.4178108	0	-0.18324832	0	0
cov3_6_9_11	19.9115	-466.176	0.4123613	0	0	0	0
cov2_3_9_10	19.9718	-466.206	0.4130557	0	0	0	-0.065422467
cov1_3_6_7	20.0178	-466.229	0.4176576	0	-0.18264777	0	0
cov3_9_10_11	20.0322	-466.236	0.4126097	0	0	0	0
cov3_7_8_10	20.0619	-466.251	0.4128406	0	0	0	0
cov1_3_5_8	20.0891	-466.265	0.4186748	0	-0.16947892	0	0
cov3_7_8_9	20.1008	-466.27	0.4120906	0	0	0	0
cov1_3_8_11	20.1613	-466.301	0.4176732	0	-0.14190431	0	0
cov3_6_9_10	20.1822	-466.311	0.4126542	0	0	0	0
cov1_2_3_6	20.1909	-466.315	0.4168946	0	-0.1893813	0	0.014695417

cov1_2_3_8	20.2052	-466.323	0.4175411	0	-0.13979369	0	0.015845848
cov1_3_4_6	20.2087	-466.324	0.4163363	0	-0.17155796	0	0
cov1_3_6_11	20.2105	-466.325	0.4168793	0	-0.18237274	0	0
cov1_3_5_6	20.2231	-466.332	0.4168951	0	-0.17884044	0	0
cov1_2_4_6	20.2386	-466.339	0.4133316	0	-0.18333916	0	0.039320585
cov1_3_4_8	20.2417	-466.341	0.4177056	0	-0.12834396	0	0
cov1_3_5_7	20.2736	-466.357	0.4183007	0	-0.15962375	0	0
cov1_3_7_11	20.3032	-466.372	0.4174923	0	-0.13936239	0	0
cov1_2_3_7	20.3328	-466.386	0.4173712	0	-0.13870522	0	0.015995759
cov1_4_6_11	20.3666	-466.403	0.4139717	0	-0.17154079	0	0
cov1_3_4_7	20.3713	-466.406	0.4174023	0	-0.12550625	0	0
cov1_4_6_8	20.4051	-466.423	0.4135768	0	-0.16225974	0	0
cov1_4_5_6	20.4124	-466.426	0.4137717	0	-0.16177569	0	0
cov1_4_6_7	20.4124	-466.426	0.4137649	0	-0.16217141	0	0
cov2_6_10_11	20.4157	-466.428	0.4100047	0	0	0	-0.035946991
cov1_5_6_11	20.418	-466.429	0.4148053	0	-0.16901492	0	0
cov1_2_5_6	20.4244	-466.432	0.4146652	0	-0.17276309	0	0.016901019
cov1_2_6_11	20.4308	-466.435	0.4147899	0	-0.19732155	0	0.015033307
cov1_6_8_11	20.451	-466.446	0.4145624	0	-0.18782821	0	0
cov1_6_7_11	20.4642	-466.452	0.4147102	0	-0.18614362	0	0
cov1_5_6_7	20.4644	-466.452	0.4146931	0	-0.15896056	0	0
cov1_5_6_8	20.4662	-466.453	0.4145287	0	-0.16001307	0	0
cov1_2_3_4	20.4668	-466.453	0.4153335	0	-0.13035667	0	0.03021889
cov2_7_10_11	20.4703	-466.455	0.4101997	0	0	0	-0.044956302
cov1_2_6_8	20.4709	-466.455	0.4145108	0	-0.18977884	0	0.01630592
cov2_9_10_11	20.4721	-466.456	0.409864	0	0	0	-0.045980649
cov1_2_6_7	20.4723	-466.456	0.4146712	0	-0.1891337	0	0.016197883
cov2_8_10_11	20.485	-466.463	0.410048	0	0	0	-0.045985297
cov2_6_9_11	20.4882	-466.464	0.4097017	0	0	0	-0.036859521
cov2_6_9_10	20.5045	-466.472	0.4101143	0	0	0	-0.041831858
cov1_2_3_11	20.5056	-466.473	0.416609	0	-0.14175299	0	0.01561
cov2_6_8_10	20.5081	-466.474	0.4101088	0	0	0	-0.041835567
cov2_6_7_10	20.5159	-466.478	0.410285	0	0	0	-0.041755256
cov1_2_3_5	20.5285	-466.484	0.4166128	0	-0.13520919	0	0.016428643
cov1_3_4_11	20.5315	-466.486	0.4161559	0	-0.12391262	0	0
cov2_7_9_11	20.536	-466.488	0.4098698	0	0	0	-0.045327672
cov1_3_5_11	20.5421	-466.491	0.4165996	0	-0.12915347	0	0
cov2_8_9_11	20.5503	-466.495	0.4097164	0	0	0	-0.046352453
cov1_3_4_5	20.5527	-466.496	0.4160883	0	-0.12187957	0	0
cov1_2_4_11	20.5539	-466.497	0.4132297	0	-0.13724051	0	0.038706069
cov2_6_8_9	20.5696	-466.505	0.4097857	0	0	0	-0.042373281
cov1_2_4_5	20.5725	-466.506	0.4123669	0	-0.14391844	0	0.049853853
cov2_6_7_9	20.5765	-466.508	0.4099602	0	0	0	-0.042288208
cov5_6_7_8	20.5789	-466.509	0.4146099	0	0	0	0
cov2_5_7_8	20.5952	-466.518	0.4148329	0	0	0	-0.033342485
cov1_2_4_8	20.5964	-466.518	0.4127639	0	-0.12654667	0	0.041642759
cov1_2_4_7	20.607	-466.524	0.41297	0	-0.12630286	0	0.040892025

cov6_7_10_11	20.649	-466.545	0.4099913	0	0	0	0
cov6_9_10_11	20.6604	-466.55	0.4096652	0	0	0	0
cov6_8_10_11	20.6667	-466.553	0.409825	0	0	0	0
cov2_8_9_10	20.6768	-466.558	0.4100962	0	0	0	-0.063593892
cov2_7_9_10	20.6844	-466.562	0.4102611	0	0	0	-0.063499831
cov1_4_8_11	20.6983	-466.569	0.4134138	0	-0.11951531	0	0
cov5_7_8_11	20.7012	-466.571	0.414963	0	0	0	0
cov1_2_5_11	20.7127	-466.576	0.4145047	0	-0.12801802	0	0.017209352
cov1_4_7_11	20.7183	-466.579	0.4136038	0	-0.1181711	0	0
cov1_4_5_11	20.7193	-466.58	0.4137971	0	-0.11514025	0	0
cov6_7_9_11	20.7346	-466.587	0.4096959	0	0	0	0
cov1_5_8_11	20.7468	-466.593	0.4142887	0	-0.11709923	0	0
cov6_8_9_11	20.7524	-466.596	0.4095292	0	0	0	0
cov1_2_8_11	20.7525	-466.596	0.4142325	0	-0.14715068	0	0.016484272
cov1_5_7_11	20.7571	-466.599	0.4144559	0	-0.11470576	0	0
cov1_2_7_11	20.7657	-466.603	0.414397	0	-0.1453331	0	0.016531451
cov1_2_5_7	20.7757	-466.608	0.4143998	0	-0.11519705	0	0.01872097
cov1_2_5_8	20.7807	-466.61	0.414224	0	-0.11609744	0	0.018827431
cov1_4_5_8	20.7897	-466.615	0.4133533	0	-0.10180855	0	0
cov1_4_5_7	20.7922	-466.616	0.4136072	0	-0.10087289	0	0
cov3_5_7_8	20.8092	-466.625	0.414282	0	0	0	0
cov4_5_7_8	20.8478	-466.644	0.4150599	0	0	0	0
cov6_8_9_10	20.8626	-466.651	0.4097744	0	0	0	0
cov6_7_9_10	20.8689	-466.654	0.4099527	0	0	0	0
cov4_6_7_8	20.9908	-466.715	0.412358	0	0	0	0
cov7_9_10_11	20.9951	-466.718	0.4098604	0	0	0	0
cov8_9_10_11	21.0345	-466.737	0.4097116	0	0	0	0
cov7_8_9_10	21.1125	-466.776	0.4092392	0	0	0	0
cov3_8_9_10	21.1568	-466.798	0.4132916	0	0	0	0
cov2_6_7_8	21.3152	-466.878	0.4141629	0	0	0	-0.044378613
cov3_7_9_10	21.3779	-466.909	0.413199	0	0	0	0
cov4_7_8_11	21.417	-466.929	0.4122131	0	0	0	0
cov3_6_7_8	21.4252	-466.933	0.4167467	0	0	0	0
cov6_7_8_11	21.5516	-466.996	0.4141818	0	0	0	0
cov2_4_7_8	21.6249	-467.032	0.4130703	0	0	0	-0.051642139
cov2_7_8_11	21.7327	-467.086	0.4146131	0	0	0	-0.067536119
cov2_3_5_6	21.7895	-467.115	0.4158647	0	0	0	-0.027470687
cov2_5_6_7	21.8018	-467.121	0.4152258	0	0	0	-0.024682275
cov2_5_7_11	21.8105	-467.125	0.4152908	0	0	0	-0.027127718
cov2_3_5_11	21.815	-467.128	0.415902	0	0	0	-0.031610353
cov2_5_6_11	21.8172	-467.129	0.4148558	0	0	0	-0.02362226
cov2_5_6_8	21.8227	-467.131	0.4150576	0	0	0	-0.024899047
cov2_4_5_6	21.8244	-467.132	0.4151063	0	0	0	-0.027125441
cov2_4_5_7	21.8318	-467.136	0.4160031	0	0	0	-0.037772306
cov2_3_4_5	21.8342	-467.137	0.4167239	0	0	0	-0.043550781
cov2_5_8_11	21.8413	-467.141	0.4151495	0	0	0	-0.028012801
cov2_4_5_11	21.8484	-467.144	0.4152305	0	0	0	-0.03199992

cov2_3_5_7	21.8529	-467.146	0.4155773	0	0	0	-0.033219181
cov2_3_7_8	21.8539	-467.147	0.4175039	0	0	0	-0.098328007
cov2_4_5_8	21.8592	-467.15	0.4157666	0	0	0	-0.037352549
cov2_3_5_8	21.8613	-467.151	0.4159236	0	0	0	-0.036725456
cov5_6_7_11	21.872	-467.156	0.415275	0	0	0	0
cov3_5_6_11	21.8821	-467.161	0.4158112	0	0	0	0
cov4_5_6_11	21.892	-467.166	0.4144243	0	0	0	0
cov4_5_6_7	21.9009	-467.17	0.414964	0	0	0	0
cov5_6_8_11	21.9044	-467.172	0.4151238	0	0	0	0
cov3_4_5_6	21.9087	-467.174	0.4152026	0	0	0	0
cov3_5_6_7	21.9144	-467.177	0.4154611	0	0	0	0
cov4_5_6_8	21.9187	-467.179	0.414723	0	0	0	0
cov3_5_6_8	21.9238	-467.182	0.4158197	0	0	0	0
cov4_5_7_11	21.9476	-467.194	0.4151657	0	0	0	0
cov3_5_7_11	21.9552	-467.198	0.4152144	0	0	0	0
cov4_5_8_11	21.9834	-467.212	0.4149646	0	0	0	0
cov3_4_5_11	21.993	-467.216	0.4151552	0	0	0	0
cov3_5_8_11	22.0018	-467.221	0.415517	0	0	0	0
cov3_4_5_7	22.0477	-467.244	0.4145245	0	0	0	0
cov3_4_7_8	22.089	-467.265	0.4112429	0	0	0	0
cov3_4_5_8	22.0983	-467.269	0.4147599	0	0	0	0
cov3_4_6_11	22.2312	-467.336	0.4143094	0	0	0	0
cov2_3_6_8	22.2711	-467.356	0.4178767	0	0	0	-0.052457167
cov3_4_6_8	22.2824	-467.361	0.4147452	0	0	0	0
cov2_3_4_6	22.2914	-467.366	0.4148635	0	0	0	-0.024522974
cov2_4_6_11	22.2954	-467.368	0.4129111	0	0	0	-0.010996555
cov4_6_7_11	22.2989	-467.369	0.4129487	0	0	0	0
cov4_6_8_11	22.3108	-467.375	0.4127601	0	0	0	0
cov3_4_6_7	22.3416	-467.391	0.4144037	0	0	0	0
cov2_3_6_11	22.3557	-467.398	0.4170501	0	0	0	-0.044179033
cov2_4_6_8	22.3621	-467.401	0.4130311	0	0	0	-0.015912653
cov2_4_6_7	22.3641	-467.402	0.4132358	0	0	0	-0.016530387
cov2_3_6_7	22.376	-467.408	0.4176608	0	0	0	-0.052030707
cov2_3_4_11	22.4969	-467.468	0.4149097	0	0	0	-0.038313125
cov2_4_7_11	22.5671	-467.504	0.4132742	0	0	0	-0.029127146
cov2_4_8_11	22.5908	-467.515	0.4131	0	0	0	-0.029380231
cov3_6_8_11	22.6237	-467.532	0.4176956	0	0	0	0
cov2_3_8_11	22.6451	-467.543	0.4180263	0	0	0	-0.073913257
cov2_6_7_11	22.6663	-467.553	0.4148313	0	0	0	-0.043295953
cov3_4_7_11	22.686	-467.563	0.4132931	0	0	0	0
cov3_4_8_11	22.6912	-467.566	0.4135635	0	0	0	0
cov2_6_8_11	22.6969	-467.568	0.4146769	0	0	0	-0.043975247
cov3_6_7_11	22.698	-467.569	0.4174694	0	0	0	0
cov2_3_7_11	22.7081	-467.574	0.4177986	0	0	0	-0.072193475
cov2_3_4_8	22.802	-467.621	0.4155648	0	0	0	-0.063404182
cov2_3_4_7	22.851	-467.646	0.4151275	0	0	0	-0.059913938
cov3_7_8_11	22.853	-467.646	0.4174093	0	0	0	0

cov1_3_7_8_10	23.1751	-464.293	0.4132151	0	-0.095258263	0	0
cov3_5_7_8_10	23.1966	-464.304	0.4100255	0	0	0	0
cov5_6_7_8_10	23.2917	-464.351	0.407018	0	0	0	0
cov1_3_7_8_9	23.3966	-464.404	0.4127997	0	-0.094020859	0	0
cov3_5_7_8_9	23.4496	-464.43	0.4095812	0	0	0	0
cov5_6_7_8_9	23.5134	-464.462	0.4067242	0	0	0	0
cov1_3_6_8_10	23.5499	-464.48	0.4143738	0	-0.17638827	0	0
cov2_5_7_8_10	23.5826	-464.497	0.4072037	0	0	0	0.025229093
cov5_7_8_10_11	23.6625	-464.537	0.4075381	0	0	0	0
cov1_5_7_8_10	23.6989	-464.555	0.4074119	0	0.011283276	0	0
cov4_5_7_8_10	23.7083	-464.56	0.4074631	0	0	0	0
cov5_7_8_9_10	23.7088	-464.56	0.4075456	0	0	0	0
cov1_3_6_8_9	23.7899	-464.6	0.413926	0	-0.17451844	0	0
cov2_5_7_8_9	23.7996	-464.605	0.4069967	0	0	0	0.02229314
cov1_3_6_7_10	23.8025	-464.607	0.4143509	0	-0.17669234	0	0
cov5_7_8_9_11	23.8529	-464.632	0.4072723	0	0	0	0
cov1_5_7_8_9	23.8947	-464.653	0.4072136	0	0.007534443	0	0
cov4_5_7_8_9	23.8963	-464.654	0.4071462	0	0	0	0
cov1_3_6_7_9	24.0317	-464.721	0.4139032	0	-0.17478882	0	0
cov1_3_5_6_10	24.0878	-464.749	0.4105293	0	-0.084791908	0	0
cov3_5_6_8_10	24.173	-464.792	0.4098325	0	0	0	0
cov1_3_5_8_10	24.1805	-464.796	0.4128381	0	-0.061800149	0	0
cov1_2_3_8_10	24.1896	-464.8	0.4139467	0	-0.11428727	0	0.021083913
cov1_2_5_6_10	24.1971	-464.804	0.4083502	0	-0.086007298	0	0.02691916
cov1_3_8_10_11	24.2038	-464.807	0.4141884	0	-0.10915603	0	0
cov1_3_4_8_10	24.211	-464.811	0.4129818	0	-0.083606524	0	0
cov3_4_7_8_10	24.2314	-464.821	0.4078826	0	0	0	0
cov1_3_8_9_10	24.2559	-464.833	0.41408	0	-0.096672792	0	0
cov1_6_7_8_10	24.2712	-464.841	0.4096123	0	-0.15489845	0	0
cov1_4_7_8_10	24.2826	-464.847	0.4068537	0	-0.053021	0	0
cov1_5_6_8_10	24.291	-464.851	0.4081828	0	-0.067331416	0	0
cov3_5_6_7_10	24.2919	-464.851	0.4096176	0	0	0	0
cov1_5_6_10_11	24.2941	-464.853	0.4085327	0	-0.071288162	0	0
cov1_3_5_6_9	24.3024	-464.857	0.4102372	0	-0.087737993	0	0
cov1_4_5_6_10	24.3027	-464.857	0.4082224	0	-0.067129654	0	0
cov1_5_6_9_10	24.303	-464.857	0.4083219	0	-0.065863339	0	0
cov1_5_6_7_10	24.3052	-464.858	0.4083443	0	-0.06658086	0	0
cov1_2_4_6_10	24.3481	-464.88	0.4061946	0	-0.16821667	0	0.073754053
cov1_6_7_8_9	24.3796	-464.895	0.4093626	0	-0.15371702	0	0
cov2_3_5_8_10	24.3848	-464.898	0.4107001	0	0	0	0.006456237
cov3_4_7_8_9	24.3904	-464.901	0.4073761	0	0	0	0
cov3_5_8_9_10	24.3912	-464.901	0.410868	0	0	0	0
cov3_5_8_10_11	24.3915	-464.901	0.4108792	0	0	0	0
cov3_4_5_8_10	24.3918	-464.901	0.4108399	0	0	0	0
cov1_3_5_7_10	24.4016	-464.906	0.4124659	0	-0.052406649	0	0
cov1_4_7_8_9	24.403	-464.907	0.4066284	0	-0.052459848	0	0
cov3_5_6_10_11	24.403	-464.907	0.4090329	0	0	0	0

cov3_5_6_9_10	24.4168	-464.914	0.4088532	0	0	0	0
cov1_2_5_6_9	24.417	-464.914	0.4081371	0	-0.087846699	0	0.024676156
cov2_3_5_6_10	24.4174	-464.914	0.4088526	0	0	0	0.005412409
cov3_4_5_6_10	24.4206	-464.916	0.4091226	0	0	0	0
cov1_3_8_9_11	24.4275	-464.919	0.4137851	0	-0.10881469	0	0
cov1_3_5_8_9	24.4278	-464.919	0.4125396	0	-0.064314998	0	0
cov1_2_3_8_9	24.4314	-464.921	0.4135598	0	-0.11167912	0	0.0193852
cov1_3_4_8_9	24.4409	-464.926	0.4125421	0	-0.082100967	0	0
cov3_5_6_8_9	24.4464	-464.929	0.4093679	0	0	0	0
cov1_2_3_7_10	24.4539	-464.932	0.413911	0	-0.11383599	0	0.021134224
cov1_3_4_7_10	24.4655	-464.938	0.4128201	0	-0.081745159	0	0
cov1_3_7_10_11	24.4781	-464.945	0.4141339	0	-0.1074596	0	0
cov1_5_6_9_11	24.4921	-464.951	0.4083133	0	-0.075373533	0	0
cov1_5_6_8_9	24.4951	-464.953	0.4079589	0	-0.070559623	0	0
cov1_4_5_6_9	24.5013	-464.956	0.4079015	0	-0.070729246	0	0
cov2_5_6_8_10	24.5034	-464.957	0.4073902	0	0	0	0.009018615
cov2_4_5_6_10	24.5036	-464.957	0.4071817	0	0	0	0.013612571
cov2_5_6_9_10	24.506	-464.958	0.40746	0	0	0	0.008997269
cov1_5_6_7_9	24.5082	-464.96	0.4081183	0	-0.069792252	0	0
cov2_5_6_10_11	24.5083	-464.96	0.4075131	0	0	0	0.009976255
cov5_6_8_9_10	24.5114	-464.961	0.4073829	0	0	0	0
cov2_5_6_7_10	24.5122	-464.962	0.4075615	0	0	0	0.009081917
cov1_2_4_6_9	24.5131	-464.962	0.4060158	0	-0.16581487	0	0.07067664
cov4_5_6_8_10	24.5169	-464.964	0.4073824	0	0	0	0
cov5_6_8_10_11	24.5176	-464.964	0.4074858	0	0	0	0
cov5_6_9_10_11	24.5191	-464.965	0.4075363	0	0	0	0
cov4_5_6_9_10	24.5202	-464.966	0.4075115	0	0	0	0
cov5_6_7_9_10	24.5204	-464.966	0.4075546	0	0	0	0
cov1_3_7_9_10	24.5207	-464.966	0.4140371	0	-0.096165255	0	0
cov4_5_6_10_11	24.5251	-464.968	0.4075951	0	0	0	0
cov5_6_7_10_11	24.5253	-464.968	0.407653	0	0	0	0
cov4_5_6_7_10	24.5267	-464.969	0.4076184	0	0	0	0
cov1_3_4_6_10	24.538	-464.974	0.4110073	0	-0.14350591	0	0
cov2_3_5_7_10	24.5384	-464.975	0.4105067	0	0	0	0.009105292
cov3_5_7_10_11	24.552	-464.981	0.4107658	0	0	0	0
cov3_5_7_9_10	24.5521	-464.982	0.4107623	0	0	0	0
cov3_4_5_7_10	24.553	-464.982	0.4107802	0	0	0	0
cov3_5_6_7_9	24.5552	-464.983	0.4091509	0	0	0	0
cov1_3_5_7_9	24.6409	-465.026	0.4121594	0	-0.054777602	0	0
cov3_5_6_9_11	24.6461	-465.029	0.4086314	0	0	0	0
cov2_3_5_8_9	24.652	-465.031	0.410335	0	0	0	0.004122132
cov3_4_5_8_9	24.6529	-465.032	0.4103092	0	0	0	0
cov3_5_8_9_11	24.6535	-465.032	0.4104241	0	0	0	0
cov1_2_7_8_10	24.6578	-465.034	0.4092936	0	-0.1090798	0	0.0229469
cov2_3_5_6_9	24.6591	-465.035	0.4085078	0	0	0	0.002793048
cov3_4_5_6_9	24.6603	-465.036	0.4086089	0	0	0	0
cov1_3_4_7_9	24.6835	-465.047	0.4123797	0	-0.080239555	0	0

cov1_2_3_7_9	24.6838	-465.047	0.4135241	0	-0.11127437	0	0.019470597
cov1_7_8_10_11	24.6881	-465.05	0.4094323	0	-0.10192953	0	0
cov1_3_4_6_9	24.6883	-465.05	0.4106174	0	-0.14220798	0	0
cov1_3_7_9_11	24.691	-465.051	0.4137328	0	-0.10710757	0	0
cov1_2_3_6_10	24.6938	-465.052	0.4132754	0	-0.18252173	0	0.019158254
cov1_2_3_5_10	24.7074	-465.059	0.4102817	0	-0.030159624	0	0.026624199
cov2_4_5_6_9	24.7346	-465.073	0.4068679	0	0	0	0.011267649
cov1_7_8_9_10	24.7373	-465.074	0.409309	0	-0.089538707	0	0
cov2_5_6_8_9	24.7375	-465.074	0.4071361	0	0	0	0.00625721
cov4_5_6_8_9	24.7413	-465.076	0.4070002	0	0	0	0
cov2_5_6_9_11	24.743	-465.077	0.4072556	0	0	0	0.006923407
cov5_6_8_9_11	24.7445	-465.078	0.4071959	0	0	0	0
cov2_5_6_7_9	24.7448	-465.078	0.4073004	0	0	0	0.006328966
cov1_3_6_10_11	24.7468	-465.079	0.4132971	0	-0.16487512	0	0
cov2_3_4_5_10	24.7472	-465.079	0.4090375	0	0	0	0.023711395
cov1_3_6_9_10	24.749	-465.08	0.4132928	0	-0.16696381	0	0
cov4_5_6_9_11	24.7496	-465.08	0.4072151	0	0	0	0
cov4_5_6_7_9	24.7505	-465.081	0.4072352	0	0	0	0
cov5_6_7_9_11	24.7511	-465.081	0.4073617	0	0	0	0
cov4_7_8_10_11	24.7513	-465.081	0.4053075	0	0	0	0
cov2_4_7_8_10	24.757	-465.084	0.4041361	0	0	0	0.020326115
cov2_3_5_9_10	24.7587	-465.085	0.4096381	0	0	0	0.01822254
cov2_3_5_10_11	24.7594	-465.085	0.409678	0	0	0	0.018315992
cov1_2_7_8_9	24.7731	-465.092	0.4090867	0	-0.1070964	0	0.021613608
cov1_7_8_9_11	24.7895	-465.1	0.4092107	0	-0.10160651	0	0
cov2_3_5_7_9	24.7965	-465.104	0.4101427	0	0	0	0.006819633
cov4_7_8_9_10	24.802	-465.106	0.4048606	0	0	0	0
cov3_5_7_9_11	24.8029	-465.107	0.4103137	0	0	0	0
cov3_4_5_7_9	24.8038	-465.107	0.4102547	0	0	0	0
cov4_6_7_8_10	24.8058	-465.108	0.4052567	0	0	0	0
cov1_3_5_10_11	24.8064	-465.109	0.4104731	0	-0.014580181	0	0
cov1_3_4_5_10	24.8116	-465.111	0.4106295	0	-0.010409636	0	0
cov1_3_5_9_10	24.8136	-465.112	0.4104397	0	-0.010463198	0	0
cov3_4_5_10_11	24.8171	-465.114	0.4103037	0	0	0	0
cov3_4_5_9_10	24.8189	-465.115	0.4103238	0	0	0	0
cov3_5_9_10_11	24.8189	-465.115	0.4100827	0	0	0	0
cov1_2_4_5_10	24.8277	-465.119	0.4068262	0	-0.033197425	0	0.051240669
cov1_2_3_6_9	24.8506	-465.131	0.4129234	0	-0.18008087	0	0.017674406
cov1_2_3_4_10	24.8565	-465.134	0.4088331	0	-0.094541402	0	0.060529782
cov2_4_5_8_10	24.8582	-465.135	0.4065166	0	0	0	0.036975558
cov4_7_8_9_11	24.8629	-465.137	0.4049975	0	0	0	0
cov2_4_7_8_9	24.8642	-465.138	0.4038606	0	0	0	0.019145179
cov1_4_6_8_10	24.8665	-465.139	0.4073182	0	-0.13140081	0	0
cov2_4_5_10_11	24.8776	-465.144	0.4070501	0	0	0	0.033538811
cov2_4_5_7_10	24.8791	-465.145	0.4067972	0	0	0	0.036120012
cov2_4_5_9_10	24.8805	-465.146	0.4068898	0	0	0	0.035766605
cov1_3_6_9_11	24.897	-465.154	0.4129407	0	-0.16455648	0	0

cov1_4_6_7_10	24.9061	-465.158	0.4075305	0	-0.13165787	0	0
cov1_2_5_10_11	24.9073	-465.159	0.4082697	0	-0.019884757	0	0.027830371
cov4_6_7_8_9	24.9094	-465.16	0.4049275	0	0	0	0
cov2_5_8_10_11	24.912	-465.161	0.407785	0	0	0	0.021922828
cov2_3_7_8_10	24.9166	-465.164	0.4135104	0	0	0	-0.068794851
cov1_4_6_10_11	24.9235	-465.167	0.4080395	0	-0.13667001	0	0
cov1_2_5_8_10	24.9268	-465.169	0.4078957	0	-0.012036909	0	0.029063843
cov2_5_9_10_11	24.9276	-465.169	0.4079762	0	0	0	0.022681181
cov2_5_7_10_11	24.928	-465.169	0.4079689	0	0	0	0.022524578
cov1_2_3_5_9	24.9294	-465.17	0.410066	0	-0.032433008	0	0.024540299
cov1_4_6_9_10	24.9322	-465.172	0.4078199	0	-0.13132073	0	0
cov2_5_8_9_10	24.9351	-465.173	0.4077391	0	0	0	0.025262995
cov1_2_5_9_10	24.9364	-465.174	0.4080536	0	-0.011176589	0	0.028876163
cov1_2_5_7_10	24.9367	-465.174	0.4080781	0	-0.011384159	0	0.028930649
cov2_5_7_9_10	24.9441	-465.178	0.4079247	0	0	0	0.025335666
cov2_3_4_5_9	24.9747	-465.193	0.4086846	0	0	0	0.021443049
cov1_2_4_8_10	24.9845	-465.198	0.4054003	0	-0.089985394	0	0.076520885
cov2_3_5_9_11	24.9889	-465.2	0.4093699	0	0	0	0.015257834
cov1_4_6_8_9	24.9904	-465.201	0.4070633	0	-0.13048851	0	0
cov4_5_8_10_11	24.9969	-465.204	0.4079257	0	0	0	0
cov5_8_9_10_11	24.9981	-465.205	0.4080501	0	0	0	0
cov1_5_8_10_11	24.9988	-465.205	0.4081478	0	-0.003488737	0	0
cov1_3_5_9_11	25.0084	-465.21	0.4102198	0	-0.019019059	0	0
cov3_6_7_8_10	25.0128	-465.212	0.4128603	0	0	0	0
cov5_7_9_10_11	25.0191	-465.215	0.4082427	0	0	0	0
cov4_5_7_10_11	25.0194	-465.215	0.4081834	0	0	0	0
cov1_3_4_5_9	25.0196	-465.215	0.4102812	0	-0.014116101	0	0
cov1_5_7_10_11	25.0204	-465.216	0.4083103	0	-0.001260128	0	0
cov4_5_9_10_11	25.0215	-465.216	0.4082748	0	0	0	0
cov1_5_9_10_11	25.022	-465.216	0.4083409	0	0.000233132	0	0
cov1_4_5_10_11	25.0228	-465.217	0.4083284	0	-0.000286434	0	0
cov1_4_6_7_9	25.0285	-465.22	0.407275	0	-0.13074396	0	0
cov3_4_5_9_11	25.0305	-465.221	0.4098231	0	0	0	0
cov1_2_4_5_9	25.0311	-465.221	0.4066077	0	-0.036537886	0	0.050102217
cov1_2_3_4_9	25.0336	-465.222	0.4085934	0	-0.09253304	0	0.057942105
cov1_2_4_7_10	25.0354	-465.223	0.4056535	0	-0.090025606	0	0.075506337
cov1_4_6_9_11	25.0399	-465.225	0.4077808	0	-0.13641317	0	0
cov2_3_7_8_9	25.0486	-465.23	0.412915	0	0	0	-0.0676837
cov1_4_5_8_10	25.0518	-465.231	0.4078491	0	0.009621571	0	0
cov1_5_8_9_10	25.0531	-465.232	0.4079576	0	0.010511587	0	0
cov4_5_8_9_10	25.0588	-465.235	0.4079224	0	0	0	0
cov1_4_5_9_10	25.0611	-465.236	0.4080492	0	0.010729513	0	0
cov1_5_7_9_10	25.0619	-465.236	0.4081348	0	0.011063642	0	0
cov1_4_5_7_10	25.0621	-465.237	0.4080927	0	0.010429179	0	0
cov1_2_4_10_11	25.0659	-465.238	0.4063079	0	-0.095690501	0	0.072082704
cov4_5_7_9_10	25.0704	-465.241	0.4081786	0	0	0	0
cov2_4_5_8_9	25.0745	-465.243	0.4062973	0	0	0	0.034124191

cov1_2_4_9_10	25.0787	-465.245	0.406136	0	-0.089912631	0	0.073271278
cov2_4_5_9_11	25.0899	-465.25	0.406824	0	0	0	0.030365364
cov2_4_5_7_9	25.094	-465.252	0.4065796	0	0	0	0.03326829
cov1_2_5_9_11	25.1118	-465.261	0.408111	0	-0.02324113	0	0.025595952
cov2_5_8_9_11	25.1255	-465.268	0.4075749	0	0	0	0.018766272
cov1_2_5_8_9	25.1399	-465.275	0.40774	0	-0.014479034	0	0.026923097
cov2_5_7_9_11	25.1405	-465.276	0.4077582	0	0	0	0.019376797
cov1_2_5_7_9	25.1485	-465.28	0.4079169	0	-0.013815137	0	0.026794628
cov1_2_4_8_9	25.1525	-465.282	0.4052903	0	-0.088057125	0	0.073353483
cov4_5_8_9_11	25.1849	-465.298	0.4075942	0	0	0	0
cov3_6_7_8_9	25.1856	-465.298	0.4123581	0	0	0	0
cov1_5_8_9_11	25.186	-465.298	0.4079602	0	-0.008101704	0	0
cov1_2_4_7_9	25.201	-465.306	0.405542	0	-0.088112455	0	0.07236193
cov3_4_8_10_11	25.2045	-465.308	0.4095463	0	0	0	0
cov4_5_7_9_11	25.2066	-465.309	0.4078525	0	0	0	0
cov1_5_7_9_11	25.2073	-465.309	0.4081204	0	-0.005871585	0	0
cov1_4_5_9_11	25.2079	-465.309	0.4080664	0	-0.005128635	0	0
cov1_3_4_10_11	25.2199	-465.315	0.4111178	0	-0.071223024	0	0
cov1_2_4_9_11	25.2218	-465.316	0.4061827	0	-0.094711447	0	0.068953821
cov1_3_4_9_10	25.2225	-465.317	0.4110975	0	-0.068518547	0	0
cov3_7_8_10_11	25.2283	-465.32	0.412639	0	0	0	0
cov1_4_5_8_9	25.2441	-465.328	0.4075794	0	0.005573374	0	0
cov1_4_5_7_9	25.2542	-465.333	0.4078216	0	0.006376527	0	0
cov1_2_3_9_10	25.3111	-465.361	0.4129991	0	-0.10989472	0	0.021291155
cov1_2_3_10_11	25.3113	-465.361	0.4129771	0	-0.11033416	0	0.021163129
cov3_4_6_8_10	25.3162	-465.364	0.409606	0	0	0	0
cov1_3_4_9_11	25.3651	-465.388	0.4107737	0	-0.071331633	0	0
cov1_2_6_8_10	25.3669	-465.389	0.4101929	0	-0.18437677	0	0.021328998
cov3_4_8_9_10	25.3756	-465.393	0.4085963	0	0	0	0
cov1_3_9_10_11	25.3785	-465.395	0.4130699	0	-0.093483167	0	0
cov1_2_6_10_11	25.3803	-465.396	0.4106087	0	-0.1878668	0	0.020368472
cov2_3_4_8_10	25.384	-465.397	0.4092605	0	0	0	-0.008277264
cov1_2_6_7_10	25.3888	-465.4	0.41036	0	-0.18385583	0	0.02119585
cov1_2_6_9_10	25.394	-465.402	0.4104633	0	-0.18313986	0	0.02101802
cov1_6_8_10_11	25.3992	-465.405	0.4103035	0	-0.17598082	0	0
cov3_4_7_10_11	25.4019	-465.406	0.4094475	0	0	0	0
cov3_4_8_9_11	25.4028	-465.407	0.4090216	0	0	0	0
cov3_7_8_9_11	25.414	-465.412	0.4120974	0	0	0	0
cov1_6_7_10_11	25.4297	-465.42	0.4104596	0	-0.17438173	0	0
cov1_6_8_9_10	25.4346	-465.423	0.4101615	0	-0.16695879	0	0
cov1_6_9_10_11	25.4414	-465.426	0.4105861	0	-0.17207002	0	0
cov1_6_7_9_10	25.4557	-465.433	0.4103292	0	-0.16653974	0	0
cov1_2_3_9_11	25.4644	-465.438	0.4126695	0	-0.10931811	0	0.019637338
cov1_2_6_8_9	25.4842	-465.448	0.4099597	0	-0.18207719	0	0.019926704
cov1_2_6_9_11	25.4919	-465.451	0.4103741	0	-0.18618637	0	0.018910015
cov3_4_6_8_9	25.499	-465.455	0.4090578	0	0	0	0
cov1_6_8_9_11	25.5032	-465.457	0.4100605	0	-0.17534849	0	0

cov1_2_6_7_9	25.505	-465.458	0.4101272	0	-0.18156106	0	0.01980036
cov3_4_6_7_10	25.5294	-465.47	0.4094243	0	0	0	0
cov1_6_7_9_11	25.5333	-465.472	0.4102166	0	-0.17375124	0	0
cov1_4_8_10_11	25.5357	-465.473	0.4075044	0	-0.06781925	0	0
cov2_3_4_8_9	25.5522	-465.482	0.4087757	0	0	0	-0.008477574
cov3_4_7_9_10	25.5768	-465.494	0.4084777	0	0	0	0
cov1_4_7_10_11	25.5847	-465.498	0.4077215	0	-0.066797173	0	0
cov2_3_4_7_10	25.5896	-465.5	0.4089929	0	0	0	-0.005825309
cov3_4_7_9_11	25.5899	-465.5	0.408928	0	0	0	0
cov1_4_8_9_10	25.6082	-465.51	0.4073203	0	-0.052631344	0	0
cov1_4_9_10_11	25.6185	-465.515	0.4080368	0	-0.06455243	0	0
cov1_4_7_9_10	25.6425	-465.527	0.4075509	0	-0.053171056	0	0
cov1_4_8_9_11	25.6497	-465.53	0.4072852	0	-0.067896852	0	0
cov1_4_7_9_11	25.6979	-465.554	0.4075033	0	-0.066884045	0	0
cov3_4_6_7_9	25.7007	-465.556	0.408877	0	0	0	0
cov2_3_4_7_9	25.7479	-465.579	0.408508	0	0	0	-0.005986921
cov2_3_8_10_11	25.7735	-465.592	0.4139939	0	0	0	-0.049303197
cov2_3_4_10_11	25.7799	-465.595	0.4075427	0	0	0	0.021399144
cov3_4_9_10_11	25.8273	-465.619	0.408531	0	0	0	0
cov3_4_6_10_11	25.8307	-465.621	0.4085042	0	0	0	0
cov2_3_6_8_10	25.837	-465.624	0.4141639	0	0	0	-0.046558794
cov1_3_6_7_8	25.9166	-465.664	0.4169193	0	-0.17104272	0	0
cov2_3_4_9_11	25.9314	-465.671	0.4071962	0	0	0	0.019357739
cov1_2_8_10_11	25.9437	-465.677	0.4099738	0	-0.1226582	0	0.021928255
cov2_4_8_10_11	25.9476	-465.679	0.4045334	0	0	0	0.03359306
cov2_3_8_9_11	25.9529	-465.682	0.4134825	0	0	0	-0.049875681
cov3_4_6_9_11	25.9721	-465.692	0.4080468	0	0	0	0
cov1_2_7_10_11	25.9742	-465.693	0.410148	0	-0.12092054	0	0.021950818
cov2_4_7_10_11	25.9817	-465.696	0.4047676	0	0	0	0.033358659
cov2_4_9_10_11	25.9833	-465.697	0.4048808	0	0	0	0.033239167
cov1_2_9_10_11	25.984	-465.697	0.410301	0	-0.11916928	0	0.021958786
cov2_4_6_10_11	25.9947	-465.703	0.4050125	0	0	0	0.033002172
cov2_3_4_6_10	25.9983	-465.705	0.4075527	0	0	0	0.014017481
cov1_2_8_9_10	26.0038	-465.707	0.4098672	0	-0.11035129	0	0.023426927
cov2_3_7_10_11	26.0049	-465.708	0.4139163	0	0	0	-0.048205588
cov2_3_6_8_9	26.0051	-465.708	0.4136479	0	0	0	-0.047150116
cov3_4_6_9_10	26.0082	-465.71	0.4080285	0	0	0	0
cov1_8_9_10_11	26.0157	-465.713	0.4100029	0	-0.10510054	0	0
cov3_6_8_10_11	26.0161	-465.714	0.4136047	0	0	0	0
cov1_2_7_9_10	26.0211	-465.716	0.4100523	0	-0.11004228	0	0.023274207
cov2_3_8_9_10	26.0241	-465.718	0.4141844	0	0	0	-0.069031642
cov2_3_4_9_10	26.0247	-465.718	0.4070926	0	0	0	0.007305665
cov1_3_5_7_8	26.0408	-465.726	0.4176068	0	-0.1599382	0	0
cov1_7_9_10_11	26.0464	-465.729	0.4101777	0	-0.10334923	0	0
cov2_4_6_8_10	26.0471	-465.729	0.4047239	0	0	0	0.02770241
cov1_2_8_9_11	26.0525	-465.732	0.4097757	0	-0.12131505	0	0.020550473
cov2_4_8_9_10	26.0534	-465.732	0.4044255	0	0	0	0.022191123

cov2_4_8_9_11	26.0788	-465.745	0.4043167	0	0	0	0.03108619
cov2_6_7_8_10	26.0804	-465.746	0.4094589	0	0	0	-0.037272771
cov1_3_7_8_11	26.0808	-465.746	0.4167752	0	-0.13834706	0	0
cov4_8_9_10_11	26.0821	-465.747	0.4056651	0	0	0	0
cov1_2_7_9_11	26.0826	-465.747	0.4099499	0	-0.11958879	0	0.020579715
cov4_6_8_10_11	26.0843	-465.748	0.4056319	0	0	0	0
cov2_3_6_7_10	26.0931	-465.752	0.4140984	0	0	0	-0.046533872
cov2_4_6_7_10	26.0981	-465.755	0.4049682	0	0	0	0.026731686
cov2_7_8_10_11	26.1018	-465.756	0.4094258	0	0	0	-0.044237771
cov2_4_7_9_10	26.1052	-465.758	0.4046637	0	0	0	0.021144698
cov1_2_3_7_8	26.1053	-465.758	0.4166635	0	-0.13812924	0	0.01562946
cov2_4_7_9_11	26.1121	-465.762	0.4045534	0	0	0	0.030859802
cov4_6_9_10_11	26.1123	-465.762	0.4058879	0	0	0	0
cov4_7_9_10_11	26.1146	-465.763	0.4058967	0	0	0	0
cov4_6_7_10_11	26.1149	-465.763	0.4058462	0	0	0	0
cov2_3_4_6_9	26.122	-465.766	0.4071647	0	0	0	0.012398902
cov2_4_6_9_11	26.1241	-465.768	0.4047789	0	0	0	0.030257058
cov2_4_6_9_10	26.1245	-465.768	0.4052602	0	0	0	0.024871153
cov4_6_8_9_10	26.1273	-465.769	0.405446	0	0	0	0
cov1_3_4_7_8	26.142	-465.776	0.4167113	0	-0.12547924	0	0
cov2_6_7_8_9	26.1457	-465.778	0.4091371	0	0	0	-0.03782251
cov2_4_6_8_9	26.1636	-465.787	0.4044626	0	0	0	0.025497417
cov2_7_8_9_11	26.1687	-465.79	0.4090863	0	0	0	-0.044610825
cov4_6_7_9_10	26.1719	-465.791	0.4056596	0	0	0	0
cov2_3_7_9_11	26.1749	-465.793	0.4134091	0	0	0	-0.048753288
cov6_7_8_10_11	26.1816	-465.796	0.4091947	0	0	0	0
cov4_6_8_9_11	26.1948	-465.803	0.4053103	0	0	0	0
cov2_4_6_7_9	26.2126	-465.812	0.4047055	0	0	0	0.024548166
cov3_6_8_9_11	26.2202	-465.816	0.4131278	0	0	0	0
cov4_6_7_9_11	26.2248	-465.818	0.4055278	0	0	0	0
cov3_6_7_10_11	26.2478	-465.829	0.4135351	0	0	0	0
cov2_3_6_7_9	26.2503	-465.831	0.4135878	0	0	0	-0.04709191
cov6_7_8_9_11	26.2695	-465.84	0.4088986	0	0	0	0
cov2_3_7_9_10	26.2728	-465.842	0.4141099	0	0	0	-0.068604552
cov3_6_8_9_10	26.2826	-465.847	0.4136476	0	0	0	0
cov1_4_6_7_8	26.3001	-465.856	0.4131282	0	-0.15131181	0	0
cov2_7_8_9_10	26.3221	-465.867	0.4094837	0	0	0	-0.06304758
cov1_6_7_8_11	26.344	-465.877	0.414018	0	-0.17444239	0	0
cov1_5_6_7_8	26.3485	-465.88	0.4140038	0	-0.14844717	0	0
cov1_2_6_7_8	26.351	-465.881	0.4139836	0	-0.17754227	0	0.016161821
cov6_7_8_9_10	26.36	-465.885	0.4091419	0	0	0	0
cov1_2_4_7_8	26.3702	-465.891	0.412382	0	-0.12609107	0	0.039691644
cov3_8_9_10_11	26.4142	-465.913	0.4133464	0	0	0	0
cov3_6_7_9_11	26.4409	-465.926	0.4130604	0	0	0	0
cov1_4_7_8_11	26.4785	-465.945	0.4129869	0	-0.11786583	0	0
cov1_5_7_8_11	26.5173	-465.964	0.4138032	0	-0.11505705	0	0
cov1_2_7_8_11	26.5209	-465.966	0.4137521	0	-0.14422063	0	0.016192391

cov1_2_5_7_8	26.5313	-465.971	0.4137554	0	-0.11593797	0	0.018254942
cov3_6_7_9_10	26.5376	-465.974	0.4135743	0	0	0	0
cov1_4_5_7_8	26.5456	-465.978	0.4129656	0	-0.10199254	0	0
cov2_3_6_10_11	26.5723	-465.992	0.4129843	0	0	0	-0.034217396
cov2_3_9_10_11	26.599	-466.005	0.412896	0	0	0	-0.041126115
cov7_8_9_10_11	26.6093	-466.01	0.4090904	0	0	0	0
cov3_7_9_10_11	26.6165	-466.014	0.4132789	0	0	0	0
cov2_3_6_9_11	26.697	-466.054	0.412572	0	0	0	-0.035350569
cov1_3_5_6_8	26.7729	-466.092	0.4189518	0	-0.22608608	0	0
cov3_6_9_10_11	26.7968	-466.104	0.4126834	0	0	0	0
cov2_3_6_9_10	26.8169	-466.114	0.4129672	0	0	0	-0.043510713
cov1_3_6_8_11	26.8702	-466.141	0.4179325	0	-0.1940744	0	0
cov1_2_3_6_8	26.8952	-466.153	0.4178288	0	-0.19464625	0	0.014078677
cov1_3_4_6_8	26.925	-466.168	0.4178423	0	-0.183605	0	0
cov1_3_5_6_7	26.9474	-466.179	0.418609	0	-0.2176706	0	0
cov1_3_6_7_11	27.0027	-466.207	0.4177683	0	-0.1924432	0	0
cov3_7_8_9_10	27.006	-466.208	0.4124169	0	0	0	0
cov1_2_3_6_7	27.0166	-466.214	0.417676	0	-0.19414386	0	0.014203851
cov1_3_5_8_11	27.0353	-466.223	0.4188225	0	-0.1854009	0	0
cov1_3_4_6_7	27.0465	-466.229	0.4175604	0	-0.18159041	0	0
cov1_3_4_5_8	27.0637	-466.237	0.4178236	0	-0.17135873	0	0
cov1_2_3_5_8	27.091	-466.251	0.4186336	0	-0.17958035	0	0.013472462
cov1_2_3_4_6	27.1478	-466.279	0.4154993	0	-0.18558439	0	0.029541823
cov1_2_3_8_11	27.1601	-466.286	0.4176685	0	-0.15335156	0	0.014223603
cov1_3_4_8_11	27.1895	-466.3	0.417813	0	-0.14357609	0	0
cov1_2_3_6_11	27.2095	-466.31	0.4169014	0	-0.19335626	0	0.014147877
cov1_2_3_5_6	27.2199	-466.315	0.4169037	0	-0.18985005	0	0.01466928
cov1_3_4_6_11	27.2231	-466.317	0.4163145	0	-0.17644609	0	0
cov1_2_4_5_6	27.2246	-466.318	0.4126273	0	-0.20374643	0	0.049391771
cov1_2_3_4_8	27.2254	-466.318	0.417005	0	-0.13785941	0	0.021285657
cov1_3_4_5_6	27.2259	-466.318	0.416198	0	-0.17936046	0	0
cov1_3_5_7_11	27.2331	-466.322	0.4184389	0	-0.17430556	0	0
cov1_2_4_6_11	27.237	-466.324	0.4135214	0	-0.19017999	0	0.037739479
cov1_3_5_6_11	27.2395	-466.325	0.416886	0	-0.18270189	0	0
cov1_2_4_6_8	27.2532	-466.332	0.4130718	0	-0.18395714	0	0.04025898
cov1_3_4_5_7	27.2577	-466.334	0.4175283	0	-0.16137307	0	0
cov1_2_4_6_7	27.2667	-466.339	0.4132685	0	-0.18352331	0	0.039561774
cov1_2_3_5_7	27.2728	-466.342	0.4182607	0	-0.17020686	0	0.014121607
cov1_2_3_7_11	27.3006	-466.356	0.4174882	0	-0.15104866	0	0.014521246
cov1_3_4_7_11	27.3322	-466.372	0.4175031	0	-0.13949086	0	0
cov1_2_3_4_7	27.345	-466.378	0.4166217	0	-0.13601784	0	0.02353374
cov1_4_6_8_11	27.3725	-466.392	0.4136875	0	-0.17365146	0	0
cov1_4_6_7_11	27.3922	-466.402	0.4138649	0	-0.17241232	0	0
cov1_4_5_6_11	27.3955	-466.403	0.414003	0	-0.17077006	0	0
cov1_2_5_6_11	27.4102	-466.411	0.4148552	0	-0.18063343	0	0.015688739
cov1_4_5_6_8	27.4341	-466.423	0.413547	0	-0.1629296	0	0
cov2_6_7_10_11	27.4357	-466.423	0.4101506	0	0	0	-0.03538785

cov2_6_9_10_11	27.4359	-466.423	0.4098795	0	0	0	-0.036076466
cov1_5_6_8_11	27.4367	-466.424	0.4146373	0	-0.17148504	0	0
cov1_2_3_4_5	27.4382	-466.425	0.4147091	0	-0.15375484	0	0.041051651
cov1_4_5_6_7	27.4414	-466.426	0.4137864	0	-0.16169391	0	0
cov2_6_8_10_11	27.4447	-466.428	0.4099905	0	0	0	-0.036005988
cov1_2_6_8_11	27.4464	-466.429	0.4146023	0	-0.1994495	0	0.014978302
cov1_5_6_7_11	27.447	-466.429	0.4147919	0	-0.16923249	0	0
cov1_2_5_6_7	27.4508	-466.431	0.4147596	0	-0.17208849	0	0.016848267
cov1_2_5_6_8	27.4521	-466.432	0.4145957	0	-0.17321176	0	0.01693832
cov1_2_6_7_11	27.4594	-466.435	0.4147548	0	-0.19775803	0	0.015022629
cov1_2_3_4_11	27.4703	-466.441	0.4153098	0	-0.13776662	0	0.029622906
cov2_7_9_10_11	27.4867	-466.449	0.410046	0	0	0	-0.044780595
cov2_8_9_10_11	27.5009	-466.456	0.4098917	0	0	0	-0.045806139
cov2_6_7_9_11	27.5083	-466.46	0.4098475	0	0	0	-0.036300675
cov2_6_8_9_11	27.5172	-466.464	0.4096859	0	0	0	-0.036915216
cov2_5_6_7_8	27.518	-466.464	0.4144653	0	0	0	-0.022035236
cov2_6_8_9_10	27.5256	-466.468	0.40996	0	0	0	-0.04189943
cov2_6_7_9_10	27.5334	-466.472	0.4101351	0	0	0	-0.041818474
cov1_2_3_5_11	27.5346	-466.473	0.4165951	0	-0.14106415	0	0.01564599
cov1_2_4_8_11	27.5526	-466.482	0.4128922	0	-0.14011265	0	0.039673717
cov1_2_4_5_11	27.5546	-466.483	0.4126474	0	-0.15267116	0	0.047038654
cov1_3_4_5_11	27.5547	-466.483	0.4160657	0	-0.12889634	0	0
cov2_5_7_8_11	27.5749	-466.493	0.4146393	0	0	0	-0.027530796
cov5_6_7_8_11	27.5755	-466.493	0.4145099	0	0	0	0
cov1_2_4_5_8	27.5772	-466.494	0.4118852	0	-0.14826146	0	0.05313671
cov1_2_4_7_11	27.5773	-466.494	0.4130868	0	-0.1384898	0	0.039130546
cov4_5_6_7_8	27.5945	-466.503	0.4141853	0	0	0	0
cov1_2_4_5_7	27.5983	-466.505	0.412192	0	-0.1455211	0	0.051070376
cov2_4_5_7_8	27.6	-466.505	0.4153958	0	0	0	-0.038884385
cov3_5_6_7_8	27.6056	-466.508	0.4148608	0	0	0	0
cov2_3_5_7_8	27.6242	-466.518	0.4148775	0	0	0	-0.033623222
cov6_7_9_10_11	27.6713	-466.541	0.4098789	0	0	0	0
cov6_8_9_10_11	27.6887	-466.55	0.4097114	0	0	0	0
cov4_5_7_8_11	27.7171	-466.564	0.4145329	0	0	0	0
cov3_5_7_8_11	27.7227	-466.567	0.4145232	0	0	0	0
cov1_4_5_8_11	27.7273	-466.569	0.4134417	0	-0.11880941	0	0
cov1_2_5_8_11	27.7318	-466.571	0.4143318	0	-0.13038328	0	0.017141059
cov1_2_5_7_11	27.7418	-466.576	0.4144996	0	-0.12804463	0	0.017208482
cov1_4_5_7_11	27.7464	-466.579	0.4136925	0	-0.11626084	0	0
cov3_4_5_7_8	27.8247	-466.618	0.41383	0	0	0	0
cov4_6_7_8_11	27.9394	-466.675	0.4123286	0	0	0	0
cov3_4_6_7_8	27.9523	-466.682	0.4138653	0	0	0	0
cov2_3_6_7_8	27.985	-466.698	0.4168441	0	0	0	-0.047597504
cov2_4_6_7_8	27.9948	-466.703	0.4125618	0	0	0	-0.013686135
cov3_6_7_8_11	28.2578	-466.834	0.4166673	0	0	0	0
cov2_6_7_8_11	28.2784	-466.845	0.4140766	0	0	0	-0.039488312
cov2_4_7_8_11	28.3136	-466.862	0.4126739	0	0	0	-0.029802501

cov3_4_7_8_11	28.4405	-466.926	0.4126247	0	0	0	0
cov2_3_7_8_11	28.4499	-466.93	0.4170693	0	0	0	-0.071757707
cov2_3_4_7_8	28.6138	-467.012	0.4144635	0	0	0	-0.060512861
cov2_3_5_6_11	28.7951	-467.103	0.4159133	0	0	0	-0.025713729
cov2_3_5_6_8	28.803	-467.107	0.4161913	0	0	0	-0.029626983
cov2_3_4_5_6	28.8066	-467.109	0.4164514	0	0	0	-0.033439005
cov2_5_6_7_11	28.8113	-467.111	0.4151532	0	0	0	-0.022506765
cov2_3_5_6_7	28.8182	-467.115	0.4158099	0	0	0	-0.027125314
cov2_4_5_6_7	28.8243	-467.118	0.4155608	0	0	0	-0.028621186
cov2_3_4_5_11	28.8264	-467.119	0.4166061	0	0	0	-0.038262528
cov2_4_5_7_11	28.8293	-467.12	0.4156903	0	0	0	-0.031471403
cov2_3_5_7_11	28.8345	-467.123	0.4156543	0	0	0	-0.029084976
cov2_5_6_8_11	28.8387	-467.125	0.4149995	0	0	0	-0.023119611
cov2_3_5_8_11	28.8427	-467.127	0.415995	0	0	0	-0.032586112
cov2_4_5_6_11	28.8456	-467.128	0.4149565	0	0	0	-0.024971703
cov2_4_5_6_8	28.8483	-467.13	0.4153017	0	0	0	-0.02777241
cov2_3_4_5_7	28.8561	-467.133	0.4164682	0	0	0	-0.040996451
cov2_3_4_5_8	28.8607	-467.136	0.4168702	0	0	0	-0.045042562
cov2_4_5_8_11	28.863	-467.137	0.415488	0	0	0	-0.031705168
cov4_5_6_7_11	28.8871	-467.149	0.4148369	0	0	0	0
cov3_4_5_6_11	28.8943	-467.153	0.4152819	0	0	0	0
cov3_5_6_7_11	28.8986	-467.155	0.4155305	0	0	0	0
cov3_5_6_8_11	28.9103	-467.161	0.4158832	0	0	0	0
cov4_5_6_8_11	28.9143	-467.163	0.4146108	0	0	0	0
cov3_4_5_6_7	28.9299	-467.17	0.4150178	0	0	0	0
cov3_4_5_6_8	28.9348	-467.173	0.41531	0	0	0	0
cov3_4_5_7_11	28.9703	-467.191	0.4147559	0	0	0	0
cov3_4_5_8_11	29.0125	-467.212	0.4149889	0	0	0	0
cov2_3_4_6_8	29.1955	-467.303	0.4160363	0	0	0	-0.031967361
cov3_4_6_8_11	29.2016	-467.306	0.4149001	0	0	0	0
cov2_3_4_6_11	29.2142	-467.313	0.4149606	0	0	0	-0.019873854
cov2_3_6_8_11	29.2229	-467.317	0.4177656	0	0	0	-0.04717776
cov3_4_6_7_11	29.2493	-467.33	0.4145701	0	0	0	0
cov2_3_4_6_7	29.2746	-467.343	0.4155934	0	0	0	-0.029119709
cov2_4_6_7_11	29.3122	-467.362	0.4131153	0	0	0	-0.011115469
cov2_3_6_7_11	29.3139	-467.362	0.4175471	0	0	0	-0.046278112
cov2_4_6_8_11	29.3244	-467.368	0.412925	0	0	0	-0.011005649
cov2_3_4_8_11	29.4875	-467.449	0.4155456	0	0	0	-0.044080003
cov2_3_4_7_11	29.5221	-467.467	0.4151172	0	0	0	-0.040155551
cov1_3_6_7_8_10	29.6748	-464.028	0.4135678	0	-0.16494809	0	0
cov1_3_6_7_8_9	29.9041	-464.143	0.4131154	0	-0.16303599	0	0
cov3_5_6_7_8_10	30.055	-464.218	0.4090904	0	0	0	0
cov1_3_5_7_8_10	30.0813	-464.232	0.4116584	0	-0.051160314	0	0
cov1_2_3_7_8_10	30.1386	-464.26	0.4131302	0	-0.11288158	0	0.020831992
cov1_3_4_7_8_10	30.1486	-464.265	0.4120401	0	-0.081086843	0	0
cov1_5_6_7_8_10	30.1662	-464.274	0.4076022	0	-0.055700373	0	0
cov1_3_7_8_10_11	30.1669	-464.274	0.4133418	0	-0.10593033	0	0

cov1_3_7_8_9_10	30.2038	-464.293	0.4132463	0	-0.095421907	0	0
cov2_3_5_7_8_10	30.2109	-464.296	0.4097325	0	0	0	0.009149402
cov3_5_7_8_9_10	30.2246	-464.303	0.4099833	0	0	0	0
cov3_5_7_8_10_11	30.2253	-464.304	0.4100038	0	0	0	0
cov3_4_5_7_8_10	30.2257	-464.304	0.4100173	0	0	0	0
cov2_5_6_7_8_10	30.2978	-464.34	0.406894	0	0	0	0.011366452
cov5_6_7_8_9_10	30.3147	-464.348	0.4069147	0	0	0	0
cov3_5_6_7_8_9	30.3172	-464.35	0.4086253	0	0	0	0
cov5_6_7_8_10_11	30.3205	-464.351	0.4070151	0	0	0	0
cov4_5_6_7_8_10	30.3206	-464.351	0.4069753	0	0	0	0
cov1_3_5_7_8_9	30.3224	-464.352	0.4113404	0	-0.053460106	0	0
cov1_3_4_7_8_9	30.3682	-464.375	0.4115877	0	-0.079521654	0	0
cov1_5_6_7_8_9	30.3695	-464.376	0.4073721	0	-0.058905263	0	0
cov1_2_3_7_8_9	30.3701	-464.376	0.4127335	0	-0.11025657	0	0.019150093
cov1_3_7_8_9_11	30.3819	-464.382	0.4129307	0	-0.10554634	0	0
cov2_3_5_7_8_9	30.4703	-464.426	0.4093604	0	0	0	0.006861037
cov1_3_5_6_8_10	30.4715	-464.427	0.4129464	0	-0.1370573	0	0
cov3_5_7_8_9_11	30.4776	-464.43	0.4095435	0	0	0	0
cov3_4_5_7_8_9	30.4779	-464.43	0.4094826	0	0	0	0
cov1_3_4_6_8_10	30.5036	-464.443	0.4129736	0	-0.16169886	0	0
cov1_2_3_6_8_10	30.5252	-464.454	0.4143209	0	-0.19163611	0	0.018873274
cov2_5_6_7_8_9	30.5292	-464.456	0.4066335	0	0	0	0.008629467
cov4_5_6_7_8_9	30.541	-464.461	0.4065941	0	0	0	0
cov5_6_7_8_9_11	30.5424	-464.462	0.4067235	0	0	0	0
cov2_4_5_7_8_10	30.5481	-464.465	0.4060675	0	0	0	0.035850811
cov1_3_6_8_10_11	30.5562	-464.469	0.4144844	0	-0.18338952	0	0
cov1_3_6_8_9_10	30.579	-464.48	0.4143785	0	-0.17641321	0	0
cov2_5_7_8_10_11	30.5976	-464.49	0.4072135	0	0	0	0.02268625
cov1_2_5_7_8_10	30.6046	-464.493	0.4073151	0	-0.010615873	0	0.028602156
cov2_5_7_8_9_10	30.6107	-464.496	0.4071642	0	0	0	0.025256228
cov1_3_5_6_7_10	30.678	-464.53	0.4126136	0	-0.12928006	0	0
cov5_7_8_9_10_11	30.6899	-464.536	0.4074845	0	0	0	0
cov4_5_7_8_10_11	30.6907	-464.536	0.4074368	0	0	0	0
cov1_5_7_8_10_11	30.6916	-464.537	0.4075352	0	0.000146316	0	0
cov1_2_4_7_8_10	30.7137	-464.548	0.404936	0	-0.089235794	0	0.074816774
cov1_5_7_8_9_10	30.7267	-464.554	0.4073609	0	0.011633704	0	0
cov1_4_5_7_8_10	30.7275	-464.555	0.4073382	0	0.010993558	0	0
cov1_3_5_6_8_9	30.7304	-464.556	0.4126003	0	-0.13869804	0	0
cov4_5_7_8_9_10	30.7366	-464.559	0.407424	0	0	0	0
cov1_3_4_6_8_9	30.7411	-464.561	0.4124812	0	-0.15950949	0	0
cov1_3_4_6_7_10	30.7441	-464.563	0.4128311	0	-0.16079987	0	0
cov2_4_5_7_8_9	30.7642	-464.573	0.4058381	0	0	0	0.033005157
cov1_4_6_7_8_10	30.7675	-464.575	0.4068406	0	-0.12055507	0	0
cov1_2_3_6_8_9	30.7749	-464.578	0.4138827	0	-0.18832701	0	0.017089371
cov1_2_3_6_7_10	30.7777	-464.58	0.4142931	0	-0.19196052	0	0.018901941
cov1_3_6_8_9_11	30.7906	-464.586	0.4140446	0	-0.18234031	0	0
cov2_5_7_8_9_11	30.8118	-464.597	0.4069959	0	0	0	0.01952335

cov1_3_6_7_10_11	30.8146	-464.598	0.4144466	0	-0.18277112	0	0
cov1_2_5_7_8_9	30.8182	-464.6	0.4071462	0	-0.012991321	0	0.026441868
cov1_3_6_7_9_10	30.8316	-464.607	0.4143549	0	-0.17670681	0	0
cov4_5_7_8_9_11	30.8793	-464.631	0.4070986	0	0	0	0
cov1_2_4_7_8_9	30.8806	-464.631	0.4048146	0	-0.087260051	0	0.071649428
cov1_5_7_8_9_11	30.8807	-464.631	0.4073384	0	-0.004460245	0	0
cov1_4_6_7_8_9	30.8906	-464.636	0.406581	0	-0.11963562	0	0
cov1_4_5_7_8_9	30.9215	-464.652	0.4070576	0	0.006966786	0	0
cov1_3_5_6_7_9	30.9293	-464.656	0.4122585	0	-0.13076196	0	0
cov1_3_4_6_7_9	30.9705	-464.676	0.4123365	0	-0.15856323	0	0
cov1_2_3_4_8_10	31.011	-464.696	0.4110486	0	-0.1033568	0	0.048294965
cov1_2_3_6_7_9	31.0163	-464.699	0.4138614	0	-0.18864557	0	0.017151445
cov1_2_3_5_6_10	31.0246	-464.703	0.4103928	0	-0.10227842	0	0.024798608
cov1_3_6_7_9_11	31.039	-464.71	0.4140036	0	-0.18166826	0	0
cov3_4_7_8_10_11	31.0617	-464.722	0.4087131	0	0	0	0
cov1_2_4_5_6_10	31.1023	-464.742	0.4069542	0	-0.11000683	0	0.050679949
cov1_3_5_6_9_10	31.115	-464.748	0.4104629	0	-0.084436644	0	0
cov1_3_4_5_6_10	31.1168	-464.749	0.4105674	0	-0.084708322	0	0
cov1_3_5_6_10_11	31.1169	-464.749	0.4105302	0	-0.08494144	0	0
cov1_2_3_5_8_10	31.1268	-464.754	0.4126149	0	-0.078131392	0	0.023509403
cov3_4_6_7_8_10	31.1519	-464.767	0.4088984	0	0	0	0
cov1_3_5_8_10_11	31.1589	-464.77	0.4130024	0	-0.074749904	0	0
cov1_2_3_8_10_11	31.174	-464.778	0.4140865	0	-0.12491201	0	0.019803881
cov1_2_3_4_6_10	31.1846	-464.783	0.4086876	0	-0.17085012	0	0.061856526
cov1_3_4_8_10_11	31.1853	-464.784	0.4131148	0	-0.096271286	0	0
cov3_5_6_8_10_11	31.1889	-464.785	0.409876	0	0	0	0
cov3_5_6_8_9_10	31.1973	-464.79	0.4097353	0	0	0	0
cov2_3_5_6_8_10	31.2015	-464.792	0.4098786	0	0	0	-0.001870535
cov3_4_5_6_8_10	31.2019	-464.792	0.4097978	0	0	0	0
cov1_3_4_5_8_10	31.2049	-464.793	0.4126327	0	-0.062770799	0	0
cov1_3_5_8_9_10	31.2093	-464.796	0.4128639	0	-0.062014972	0	0
cov1_2_5_6_8_10	31.2096	-464.796	0.408123	0	-0.087237991	0	0.027084412
cov1_2_3_8_9_10	31.2172	-464.8	0.4140015	0	-0.1146789	0	0.021198191
cov1_2_5_6_10_11	31.2189	-464.8	0.4084515	0	-0.089520381	0	0.0263893
cov1_2_5_6_9_10	31.2241	-464.803	0.4082884	0	-0.085589688	0	0.026783239
cov1_2_5_6_7_10	31.2247	-464.803	0.4082834	0	-0.086408356	0	0.026970036
cov1_2_6_7_8_10	31.2323	-464.807	0.4096007	0	-0.17206848	0	0.021202313
cov1_3_8_9_10_11	31.2324	-464.807	0.4142206	0	-0.10929325	0	0
cov1_3_4_8_9_10	31.2398	-464.811	0.4130127	0	-0.08379826	0	0
cov3_4_7_8_9_10	31.2422	-464.812	0.4077245	0	0	0	0
cov1_2_3_4_7_10	31.2485	-464.815	0.4108078	0	-0.10219867	0	0.050050849
cov3_4_7_8_9_11	31.2511	-464.816	0.4081842	0	0	0	0
cov1_2_3_5_6_9	31.2547	-464.818	0.4101312	0	-0.1038646	0	0.02260549
cov1_2_4_6_8_10	31.2558	-464.819	0.4053462	0	-0.16961863	0	0.077410788
cov2_3_4_7_8_10	31.2561	-464.819	0.4082473	0	0	0	-0.005869011
cov1_4_7_8_10_11	31.2599	-464.821	0.4069691	0	-0.06550287	0	0
cov1_2_3_4_8_9	31.2633	-464.823	0.4107128	0	-0.1008126	0	0.045828492

cov1_6_7_8_10_11	31.2744	-464.828	0.4096967	0	-0.16242505	0	0
cov1_5_6_8_10_11	31.2972	-464.84	0.4082942	0	-0.074885966	0	0
cov1_6_7_8_9_10	31.2992	-464.841	0.4095685	0	-0.15473739	0	0
cov3_5_6_7_10_11	31.3061	-464.844	0.4096661	0	0	0	0
cov1_4_7_8_9_10	31.3103	-464.846	0.4067975	0	-0.05266177	0	0
cov1_2_4_6_7_10	31.3107	-464.846	0.4055802	0	-0.16949012	0	0.076486151
cov1_4_5_6_8_10	31.3125	-464.847	0.4078816	0	-0.068730028	0	0
cov1_2_4_5_6_9	31.3129	-464.847	0.4066907	0	-0.1126523	0	0.049341573
cov3_5_6_7_9_10	31.316	-464.849	0.4095185	0	0	0	0
cov1_5_6_8_9_10	31.3168	-464.849	0.4081048	0	-0.066927812	0	0
cov1_5_6_7_10_11	31.3187	-464.85	0.4084378	0	-0.072847654	0	0
cov1_5_6_9_10_11	31.3196	-464.851	0.4084518	0	-0.070928548	0	0
cov1_4_5_6_10_11	31.3199	-464.851	0.4083619	0	-0.072032723	0	0
cov2_3_5_6_7_10	31.321	-464.851	0.4096157	0	0	0	8.32E-05
cov3_4_5_6_7_10	31.321	-464.851	0.4096335	0	0	0	0
cov3_4_6_7_8_9	31.3277	-464.855	0.408355	0	0	0	0
cov1_4_5_6_9_10	31.3283	-464.855	0.4081362	0	-0.066737625	0	0
cov1_4_5_6_7_10	31.3296	-464.856	0.408113	0	-0.06772023	0	0
cov1_3_5_6_9_11	31.3308	-464.856	0.4102413	0	-0.088737432	0	0
cov1_5_6_7_9_10	31.331	-464.856	0.4082656	0	-0.06617552	0	0
cov1_3_4_5_6_9	31.3311	-464.856	0.4101631	0	-0.087892411	0	0
cov1_2_3_5_7_10	31.3437	-464.863	0.412239	0	-0.069150229	0	0.024120313
cov1_2_6_7_8_9	31.3494	-464.866	0.4093637	0	-0.16975121	0	0.019789677
cov1_2_3_4_6_9	31.3659	-464.874	0.4084038	0	-0.16833632	0	0.059193477
cov1_4_7_8_9_11	31.3746	-464.878	0.4067415	0	-0.065554508	0	0
cov1_2_4_6_9_10	31.3754	-464.879	0.4061358	0	-0.16789206	0	0.073665322
cov1_2_4_6_10_11	31.3766	-464.879	0.4062298	0	-0.16920012	0	0.073487369
cov1_6_7_8_9_11	31.3789	-464.88	0.4094493	0	-0.16179138	0	0
cov1_2_3_5_8_9	31.388	-464.885	0.4123532	0	-0.079385236	0	0.021436825
cov1_3_5_7_10_11	31.3904	-464.886	0.4126155	0	-0.064039762	0	0
cov1_3_5_8_9_11	31.398	-464.89	0.4127061	0	-0.078144632	0	0
cov1_3_4_8_9_11	31.4072	-464.895	0.4126748	0	-0.095611694	0	0
cov2_3_4_5_8_10	31.4072	-464.895	0.4102339	0	0	0	0.010599164
cov1_2_3_8_9_11	31.4078	-464.895	0.4137036	0	-0.1231851	0	0.018008794
cov2_3_5_8_9_10	31.4129	-464.897	0.4106607	0	0	0	0.006478663
cov2_3_5_8_10_11	31.4138	-464.898	0.4106962	0	0	0	0.006334686
cov2_3_4_7_8_9	31.4149	-464.898	0.4077613	0	0	0	-0.00601048
cov3_5_8_9_10_11	31.4195	-464.901	0.4108369	0	0	0	0
cov3_4_5_8_9_10	31.4199	-464.901	0.4107971	0	0	0	0
cov3_4_5_8_10_11	31.4202	-464.901	0.4108142	0	0	0	0
cov2_3_5_6_10_11	31.4235	-464.903	0.4089009	0	0	0	0.007152662
cov3_5_6_9_10_11	31.4273	-464.905	0.408934	0	0	0	0
cov1_3_4_5_7_10	31.4285	-464.905	0.4123244	0	-0.053087893	0	0
cov1_2_4_6_8_9	31.4296	-464.906	0.4052006	0	-0.1670993	0	0.074119062
cov3_4_5_6_10_11	31.4303	-464.906	0.4091916	0	0	0	0
cov1_3_5_7_9_10	31.4306	-464.906	0.4124833	0	-0.052548274	0	0
cov1_2_5_6_8_9	31.4313	-464.907	0.4079273	0	-0.089017616	0	0.024822264

cov1_2_5_6_9_11	31.4345	-464.908	0.408259	0	-0.092210134	0	0.024023871
cov2_3_5_6_9_10	31.441	-464.911	0.4087508	0	0	0	0.00533698
cov3_4_5_6_9_10	31.4442	-464.913	0.4090084	0	0	0	0
cov1_2_5_6_7_9	31.4451	-464.913	0.4080833	0	-0.088172678	0	0.024712966
cov2_3_4_5_6_10	31.4465	-464.914	0.4088652	0	0	0	0.005287351
cov1_2_3_7_10_11	31.4472	-464.915	0.4140337	0	-0.1233601	0	0.019986003
cov1_3_4_5_8_9	31.448	-464.915	0.4122538	0	-0.065595906	0	0
cov1_3_4_7_10_11	31.4495	-464.916	0.4129415	0	-0.093253035	0	0
cov3_5_6_8_9_11	31.4663	-464.924	0.4094108	0	0	0	0
cov2_3_5_6_8_9	31.4726	-464.927	0.4094704	0	0	0	-0.004167224
cov3_4_5_6_8_9	31.474	-464.928	0.4092325	0	0	0	0
cov1_2_3_7_9_10	31.4817	-464.932	0.4139608	0	-0.11420715	0	0.021240127
cov1_2_4_6_7_9	31.4822	-464.932	0.405432	0	-0.16697807	0	0.073212157
cov1_2_3_4_7_9	31.4892	-464.936	0.4104686	0	-0.099676419	0	0.047645688
cov1_3_4_7_9_10	31.4943	-464.938	0.4128461	0	-0.081907805	0	0
cov1_5_6_8_9_11	31.4955	-464.939	0.4080766	0	-0.078948598	0	0
cov1_3_7_9_10_11	31.5067	-464.944	0.4141643	0	-0.10758908	0	0
cov1_4_5_6_8_9	31.5114	-464.947	0.407571	0	-0.072356923	0	0
cov1_4_5_6_9_11	31.5142	-464.948	0.4080596	0	-0.076401654	0	0
cov1_5_6_7_9_11	31.5168	-464.949	0.4082195	0	-0.076909831	0	0
cov2_4_5_6_8_10	31.5187	-464.95	0.4068484	0	0	0	0.014883458
cov2_4_5_6_10_11	31.5259	-464.954	0.4070489	0	0	0	0.015600573
cov2_4_5_6_9_10	31.526	-464.954	0.4070648	0	0	0	0.013647227
cov2_5_6_8_9_10	31.5263	-464.954	0.4072884	0	0	0	0.00893111
cov1_4_5_6_7_9	31.5283	-464.955	0.4078006	0	-0.071332016	0	0
cov2_5_6_8_10_11	31.5309	-464.956	0.4073775	0	0	0	0.009617978
cov2_5_6_9_10_11	31.5315	-464.957	0.4074165	0	0	0	0.009849251
cov2_4_5_6_7_10	31.5322	-464.957	0.4071208	0	0	0	0.013842113
cov2_5_6_7_9_10	31.5351	-464.958	0.4074598	0	0	0	0.008997192
cov2_5_6_7_10_11	31.5371	-464.959	0.4075403	0	0	0	0.010052962
cov4_5_6_8_9_10	31.5393	-464.961	0.4072649	0	0	0	0
cov1_2_4_6_9_11	31.5402	-464.961	0.4060754	0	-0.16760686	0	0.070204333
cov5_6_8_9_10_11	31.5403	-464.961	0.4073811	0	0	0	0
cov4_5_6_8_10_11	31.5457	-464.964	0.4073794	0	0	0	0
cov4_5_6_9_10_11	31.5479	-464.965	0.4074846	0	0	0	0
cov5_6_7_9_10_11	31.5481	-464.965	0.4075493	0	0	0	0
cov4_5_6_7_9_10	31.5492	-464.966	0.4075016	0	0	0	0
cov4_5_6_7_10_11	31.5542	-464.968	0.4076091	0	0	0	0
cov2_3_4_5_7_10	31.5597	-464.971	0.4099976	0	0	0	0.013599537
cov1_3_4_6_9_10	31.5657	-464.974	0.4109446	0	-0.14323433	0	0
cov1_3_4_6_10_11	31.5662	-464.974	0.4110019	0	-0.14226148	0	0
cov2_3_5_7_9_10	31.5666	-464.974	0.410468	0	0	0	0.009127561
cov2_3_5_7_10_11	31.5674	-464.975	0.4105036	0	0	0	0.009029411
cov3_5_6_7_9_11	31.5736	-464.978	0.4091988	0	0	0	0
cov3_5_7_9_10_11	31.5802	-464.981	0.4107249	0	0	0	0
cov3_4_5_7_10_11	31.5811	-464.981	0.4107486	0	0	0	0
cov3_4_5_7_9_10	31.5812	-464.982	0.4107401	0	0	0	0

cov2_3_5_6_7_9	31.5835	-464.983	0.4092039	0	0	0	-0.002160347
cov3_4_5_6_7_9	31.5837	-464.983	0.4090671	0	0	0	0
cov1_2_3_5_7_9	31.597	-464.989	0.4119675	0	-0.070276915	0	0.022062376
cov1_3_5_7_9_11	31.6224	-465.002	0.4123117	0	-0.067297987	0	0
cov2_4_7_8_10_11	31.6361	-465.009	0.4040492	0	0	0	0.03342233
cov1_2_7_8_10_11	31.6462	-465.014	0.4093865	0	-0.11923112	0	0.021723058
cov2_3_7_8_10_11	31.6535	-465.018	0.4131179	0	0	0	-0.047466272
cov1_3_4_7_9_11	31.6605	-465.021	0.4125016	0	-0.092570154	0	0
cov1_3_4_5_7_9	31.6647	-465.023	0.4119375	0	-0.055783802	0	0
cov1_2_3_7_9_11	31.6702	-465.026	0.4136524	0	-0.12165468	0	0.018230945
cov2_3_5_6_9_11	31.6722	-465.027	0.4085567	0	0	0	0.004215152
cov2_3_4_5_8_9	31.6724	-465.027	0.4097982	0	0	0	0.008867727
cov2_3_6_7_8_10	31.675	-465.028	0.4132459	0	0	0	-0.042043943
cov3_4_5_6_9_11	31.6751	-465.028	0.4086756	0	0	0	0
cov2_3_5_8_9_11	31.6803	-465.031	0.4103176	0	0	0	0.003675223
cov3_4_5_8_9_11	31.6805	-465.031	0.4102678	0	0	0	0
cov1_2_7_8_9_10	31.6869	-465.034	0.4092896	0	-0.10904988	0	0.022938092
cov1_2_3_4_5_10	31.6875	-465.035	0.4091486	0	-0.043081835	0	0.042311311
cov2_3_4_5_6_9	31.6881	-465.035	0.4084423	0	0	0	0.003425467
cov1_7_8_9_10_11	31.7168	-465.049	0.4094056	0	-0.10181036	0	0
cov1_3_4_6_9_11	31.7173	-465.05	0.4106157	0	-0.14188266	0	0
cov1_2_3_6_10_11	31.7186	-465.05	0.4132514	0	-0.17992322	0	0.019530899
cov1_2_3_6_9_10	31.7229	-465.052	0.413266	0	-0.18247361	0	0.019138121
cov2_4_6_7_8_10	31.7258	-465.054	0.4042975	0	0	0	0.029563148
cov1_2_3_5_10_11	31.7324	-465.057	0.410289	0	-0.032862606	0	0.026271219
cov1_2_3_5_9_10	31.7365	-465.059	0.4102751	0	-0.030098005	0	0.026613189
cov2_4_5_6_8_9	31.7512	-465.067	0.4065539	0	0	0	0.0124626
cov1_2_7_8_9_11	31.7563	-465.069	0.4091783	0	-0.11784544	0	0.020324295
cov2_4_5_6_9_11	31.7596	-465.071	0.4067699	0	0	0	0.012786605
cov2_4_5_6_7_9	31.7634	-465.073	0.4068254	0	0	0	0.011427143
cov2_4_7_8_9_10	31.7647	-465.073	0.4039399	0	0	0	0.020955225
cov2_5_6_8_9_11	31.7661	-465.074	0.4071254	0	0	0	0.006573235
cov2_4_7_8_9_11	31.7678	-465.075	0.4038266	0	0	0	0.030912755
cov4_7_8_9_10_11	31.769	-465.075	0.4051711	0	0	0	0
cov4_5_6_8_9_11	31.7703	-465.076	0.4069993	0	0	0	0
cov2_5_6_7_9_11	31.7717	-465.077	0.4072881	0	0	0	0.007016602
cov2_3_4_5_9_10	31.7752	-465.079	0.4089835	0	0	0	0.023826977
cov1_3_6_9_10_11	31.7757	-465.079	0.4132641	0	-0.16482857	0	0
cov2_3_4_5_10_11	31.7757	-465.079	0.4090312	0	0	0	0.024332419
cov4_5_6_7_9_11	31.7787	-465.08	0.4072302	0	0	0	0
cov4_6_7_8_10_11	31.78	-465.081	0.4052715	0	0	0	0
cov2_3_5_9_10_11	31.7878	-465.085	0.4096422	0	0	0	0.018312263
cov3_6_7_8_10_11	31.7947	-465.088	0.4127349	0	0	0	0
cov2_3_4_5_7_9	31.8157	-465.099	0.4095651	0	0	0	0.011899363
cov1_2_4_5_8_10	31.817	-465.099	0.4062452	0	-0.038389797	0	0.055421428
cov4_6_7_8_9_10	31.8207	-465.101	0.4050865	0	0	0	0
cov2_3_5_7_9_11	31.8251	-465.103	0.4101273	0	0	0	0.006423626

cov2_3_7_8_9_11	31.8251	-465.103	0.4126017	0	0	0	-0.048012664
cov3_4_5_7_9_11	31.8311	-465.106	0.4102074	0	0	0	0
cov1_3_4_5_10_11	31.8334	-465.108	0.4106345	0	-0.014337581	0	0
cov1_3_5_9_10_11	31.8351	-465.108	0.410443	0	-0.014399639	0	0
cov1_2_4_5_10_11	31.8371	-465.11	0.4070311	0	-0.039283219	0	0.049418821
cov2_3_6_7_8_9	31.8376	-465.11	0.4127342	0	0	0	-0.042615011
cov1_3_4_5_9_10	31.8405	-465.111	0.4106026	0	-0.010226478	0	0
cov2_4_6_7_8_9	31.8447	-465.113	0.4040395	0	0	0	0.027347814
cov3_4_5_9_10_11	31.8455	-465.114	0.4102657	0	0	0	0
cov1_2_4_5_7_10	31.8473	-465.115	0.4065468	0	-0.035754955	0	0.053299091
cov1_2_4_5_9_10	31.8564	-465.119	0.406798	0	-0.032967181	0	0.051191244
cov1_4_6_8_10_11	31.8592	-465.121	0.4074381	0	-0.14026812	0	0
cov2_4_5_8_10_11	31.8775	-465.13	0.4066193	0	0	0	0.033928779
cov1_2_3_6_9_11	31.8779	-465.13	0.4129085	0	-0.17838167	0	0.017912864
cov1_2_3_4_10_11	31.8852	-465.134	0.4088373	0	-0.095467789	0	0.060427315
cov1_2_3_4_9_10	31.8856	-465.134	0.4088298	0	-0.094519949	0	0.060527184
cov2_4_5_8_9_10	31.8856	-465.134	0.406458	0	0	0	0.037107808
cov4_6_7_8_9_11	31.8915	-465.137	0.4049543	0	0	0	0
cov1_4_6_8_9_10	31.892	-465.137	0.4072308	0	-0.13099989	0	0
cov2_4_5_7_10_11	31.902	-465.142	0.4068811	0	0	0	0.03369507
cov1_2_3_4_5_9	31.9022	-465.142	0.408853	0	-0.046194004	0	0.041369385
cov2_4_5_9_10_11	31.9049	-465.143	0.4069917	0	0	0	0.033593412
cov2_4_5_7_9_10	31.9066	-465.144	0.4067404	0	0	0	0.036238281
cov1_4_6_7_10_11	31.908	-465.145	0.4076352	0	-0.13936124	0	0
cov1_2_5_8_10_11	31.9125	-465.147	0.4080285	0	-0.023214047	0	0.027758543
cov2_3_7_8_9_10	31.9282	-465.155	0.4133073	0	0	0	-0.068118192
cov1_4_6_7_9_10	31.9316	-465.157	0.4074431	0	-0.13126059	0	0
cov1_2_5_7_10_11	31.9337	-465.158	0.4081912	0	-0.021020517	0	0.027806847
cov1_2_5_9_10_11	31.9359	-465.159	0.4082384	0	-0.01964365	0	0.027759193
cov2_5_8_9_10_11	31.9398	-465.161	0.4077389	0	0	0	0.021904271
cov1_4_6_9_10_11	31.9488	-465.165	0.4079508	0	-0.13632353	0	0
cov1_2_3_5_9_11	31.9508	-465.166	0.4100704	0	-0.036032521	0	0.024069559
cov1_2_5_8_9_10	31.9556	-465.169	0.4078725	0	-0.011826021	0	0.029013041
cov2_5_7_9_10_11	31.9559	-465.169	0.407925	0	0	0	0.022507143
cov1_2_5_7_9_10	31.9655	-465.174	0.4080514	0	-0.011170824	0	0.028877947
cov1_2_4_8_10_11	31.9713	-465.177	0.4055419	0	-0.10048193	0	0.074845603
cov1_4_6_8_9_11	31.9778	-465.18	0.4071845	0	-0.13994806	0	0
cov3_6_7_8_9_11	31.9902	-465.186	0.4122582	0	0	0	0
cov2_3_4_5_9_11	32.0038	-465.193	0.4086836	0	0	0	0.021618844
cov1_2_4_8_9_10	32.0133	-465.198	0.405376	0	-0.089816418	0	0.076487266
cov1_2_4_5_8_9	32.0217	-465.202	0.4060422	0	-0.041532096	0	0.054098947
cov4_5_8_9_10_11	32.0244	-465.203	0.407869	0	0	0	0
cov1_4_5_8_10_11	32.0248	-465.203	0.4079723	0	-0.004301926	0	0
cov1_4_6_7_9_11	32.0259	-465.204	0.4073821	0	-0.13904397	0	0
cov1_5_8_9_10_11	32.0266	-465.204	0.4080964	0	-0.003187469	0	0
cov1_2_4_7_10_11	32.0328	-465.207	0.4057767	0	-0.099133152	0	0.074061217
cov1_2_4_5_9_11	32.0349	-465.208	0.4068324	0	-0.043213051	0	0.04793548

cov3_6_7_8_9_10	32.0369	-465.209	0.4127548	0	0	0	0
cov1_3_4_5_9_11	32.0372	-465.21	0.4102825	0	-0.018941432	0	0
cov4_5_7_9_10_11	32.0471	-465.214	0.4081291	0	0	0	0
cov1_5_7_9_10_11	32.0482	-465.215	0.4082596	0	-0.00096391	0	0
cov1_4_5_7_10_11	32.0483	-465.215	0.4082018	0	-0.001761378	0	0
cov1_4_5_9_10_11	32.0506	-465.216	0.4082747	0	7.46E-06	0	0
cov1_2_4_5_7_9	32.0513	-465.217	0.4063401	0	-0.038894353	0	0.051980574
cov1_2_3_4_9_11	32.0609	-465.221	0.4086013	0	-0.094589826	0	0.057726817
cov1_2_4_7_9_10	32.0643	-465.223	0.4056316	0	-0.089861068	0	0.075473566
cov1_4_5_8_9_10	32.0799	-465.231	0.4078007	0	0.009922382	0	0
cov1_4_5_7_9_10	32.0902	-465.236	0.4080452	0	0.010724486	0	0
cov2_4_5_8_9_11	32.0913	-465.237	0.4064019	0	0	0	0.030735583
cov1_2_4_9_10_11	32.0947	-465.238	0.4062822	0	-0.095520712	0	0.07203799
cov2_4_5_7_9_11	32.1149	-465.248	0.4066649	0	0	0	0.030507752
cov1_2_5_8_9_11	32.1174	-465.25	0.4078736	0	-0.02654225	0	0.025518037
cov1_2_4_8_9_11	32.1323	-465.257	0.405432	0	-0.099356571	0	0.071596872
cov1_2_5_7_9_11	32.1383	-465.26	0.4080354	0	-0.024350984	0	0.025566948
cov1_2_4_7_9_11	32.1923	-465.287	0.4056666	0	-0.09802228	0	0.070830063
cov1_4_5_8_9_11	32.2085	-465.295	0.4077013	0	-0.009210854	0	0
cov3_4_8_9_10_11	32.226	-465.304	0.4094295	0	0	0	0
cov3_4_6_8_10_11	32.2281	-465.305	0.4097329	0	0	0	0
cov2_3_4_8_10_11	32.2308	-465.306	0.4092776	0	0	0	0.004941891
cov1_4_5_7_9_11	32.2329	-465.307	0.4079308	0	-0.006697289	0	0
cov3_7_8_9_10_11	32.2463	-465.314	0.4124906	0	0	0	0
cov1_3_4_9_10_11	32.2489	-465.315	0.4110979	0	-0.071122938	0	0
cov3_4_6_8_9_10	32.3359	-465.359	0.4094541	0	0	0	0
cov1_2_3_9_10_11	32.3401	-465.361	0.4130018	0	-0.11047862	0	0.021217684
cov2_3_4_6_8_10	32.3453	-465.364	0.4095812	0	0	0	0.000504636
cov1_2_6_8_10_11	32.3663	-465.374	0.4102808	0	-0.19159391	0	0.020318509
cov1_2_6_8_9_10	32.3955	-465.389	0.4101665	0	-0.18422139	0	0.021266769
cov1_2_6_7_10_11	32.3966	-465.389	0.4104376	0	-0.19001082	0	0.020340237
cov2_3_4_8_9_10	32.3973	-465.39	0.4090745	0	0	0	-0.007643553
cov1_2_6_9_10_11	32.4088	-465.395	0.4105768	0	-0.187709	0	0.020285514
cov1_2_6_7_9_10	32.4174	-465.4	0.4103331	0	-0.1836966	0	0.021128373
cov3_4_7_9_10_11	32.4235	-465.403	0.4093309	0	0	0	0
cov2_3_4_7_10_11	32.4241	-465.403	0.4090177	0	0	0	0.007854222
cov1_6_8_9_10_11	32.427	-465.404	0.4102562	0	-0.1758463	0	0
cov3_4_6_8_9_11	32.4276	-465.405	0.4091941	0	0	0	0
cov3_4_6_7_10_11	32.4289	-465.405	0.4095638	0	0	0	0
cov2_3_4_8_9_11	32.4306	-465.406	0.4088389	0	0	0	0.003337558
cov1_6_7_9_10_11	32.4575	-465.42	0.410413	0	-0.17425014	0	0
cov1_2_6_8_9_11	32.4789	-465.43	0.4100498	0	-0.18984989	0	0.018850106
cov1_2_6_7_9_11	32.5088	-465.445	0.4102064	0	-0.18827264	0	0.018877146
cov2_3_4_6_8_9	32.528	-465.455	0.4090974	0	0	0	-0.000798844
cov3_4_6_7_9_10	32.5485	-465.465	0.4092672	0	0	0	0
cov2_3_4_6_7_10	32.5578	-465.47	0.4092989	0	0	0	0.002525757
cov1_4_8_9_10_11	32.5632	-465.473	0.4074449	0	-0.067495876	0	0

cov2_3_4_7_9_10	32.6025	-465.492	0.4088041	0	0	0	-0.005183225
cov1_4_7_9_10_11	32.6122	-465.497	0.4076638	0	-0.066484882	0	0
cov2_3_4_7_9_11	32.6145	-465.498	0.4085803	0	0	0	0.00630056
cov3_4_6_7_9_11	32.6176	-465.5	0.4090266	0	0	0	0
cov2_3_4_6_7_9	32.7296	-465.556	0.4088118	0	0	0	0.001282334
cov2_3_6_8_10_11	32.7393	-465.561	0.4139595	0	0	0	-0.039767324
cov2_3_8_9_10_11	32.796	-465.589	0.4138772	0	0	0	-0.049171155
cov2_3_4_9_10_11	32.8008	-465.591	0.4074078	0	0	0	0.021599454
cov2_3_4_6_10_11	32.809	-465.595	0.4075434	0	0	0	0.021416834
cov1_3_5_6_7_8	32.8432	-465.613	0.417884	0	-0.20654269	0	0
cov3_4_6_9_10_11	32.8516	-465.617	0.4083601	0	0	0	0
cov2_3_6_8_9_10	32.8601	-465.621	0.4140493	0	0	0	-0.046597828
cov1_3_6_7_8_11	32.9026	-465.642	0.4170243	0	-0.18070537	0	0
cov1_2_3_6_7_8	32.9153	-465.649	0.416937	0	-0.18254363	0	0.014210733
cov2_3_6_8_9_11	32.9257	-465.654	0.4134738	0	0	0	-0.040887975
cov1_3_4_6_7_8	32.9456	-465.664	0.4168682	0	-0.1704854	0	0
cov2_3_4_6_9_11	32.9604	-465.671	0.4071854	0	0	0	0.019148499
cov2_4_8_9_10_11	32.9642	-465.673	0.4043883	0	0	0	0.033744331
cov1_2_8_9_10_11	32.9727	-465.677	0.4099673	0	-0.12261515	0	0.021911388
cov2_4_6_8_10_11	32.9766	-465.679	0.4045422	0	0	0	0.033898771
cov2_3_6_7_10_11	32.9787	-465.68	0.4138848	0	0	0	-0.039284685
cov2_4_7_9_10_11	32.9988	-465.69	0.4046275	0	0	0	0.033508486
cov1_2_7_9_10_11	33.0033	-465.693	0.4101425	0	-0.12088516	0	0.021937172
cov1_3_5_7_8_11	33.0074	-465.695	0.4177382	0	-0.1738554	0	0
cov2_4_6_7_10_11	33.0108	-465.696	0.404771	0	0	0	0.033458386
cov2_4_6_9_10_11	33.0122	-465.697	0.4048658	0	0	0	0.032916197
cov2_3_4_6_9_10	33.0145	-465.698	0.4073699	0	0	0	0.014121137
cov2_6_7_8_10_11	33.0187	-465.7	0.4093449	0	0	0	-0.031499847
cov1_3_4_5_7_8	33.0255	-465.704	0.4168396	0	-0.16167574	0	0
cov2_3_7_9_10_11	33.0274	-465.705	0.4137993	0	0	0	-0.048072998
cov1_2_3_5_7_8	33.0417	-465.712	0.4175708	0	-0.17022107	0	0.013720582
cov3_6_8_9_10_11	33.0426	-465.712	0.4135317	0	0	0	0
cov2_4_6_8_9_10	33.0605	-465.721	0.4045473	0	0	0	0.027725329
cov1_2_3_7_8_11	33.0795	-465.731	0.4167743	0	-0.14980477	0	0.014236393
cov2_6_7_8_9_11	33.0949	-465.738	0.409043	0	0	0	-0.032427185
cov2_6_7_8_9_10	33.0989	-465.74	0.4093153	0	0	0	-0.037335496
cov4_6_8_9_10_11	33.1004	-465.741	0.4054722	0	0	0	0
cov2_4_6_8_9_11	33.1079	-465.745	0.4043181	0	0	0	0.03112803
cov1_3_4_7_8_11	33.1098	-465.746	0.4168069	0	-0.13872359	0	0
cov2_4_6_7_9_10	33.1117	-465.747	0.4047911	0	0	0	0.0267545
cov2_3_6_7_9_10	33.1158	-465.749	0.4139798	0	0	0	-0.046573666
cov2_7_8_9_10_11	33.1174	-465.75	0.4092667	0	0	0	-0.0440567
cov1_2_3_4_7_8	33.1195	-465.751	0.4159628	0	-0.13561375	0	0.022687702
cov4_6_7_9_10_11	33.1314	-465.757	0.4056873	0	0	0	0
cov2_4_6_7_9_11	33.1412	-465.762	0.4045451	0	0	0	0.030690909
cov2_3_6_7_9_11	33.155	-465.768	0.4134019	0	0	0	-0.04036165
cov1_2_4_6_7_8	33.162	-465.772	0.4126478	0	-0.17222384	0	0.038676495

cov6_7_8_9_10_11	33.2042	-465.793	0.4090847	0	0	0	0
cov3_6_7_9_10_11	33.2742	-465.828	0.4134582	0	0	0	0
cov1_4_6_7_8_11	33.2813	-465.832	0.4132229	0	-0.16140758	0	0
cov1_4_5_6_7_8	33.3292	-465.856	0.4131375	0	-0.15110395	0	0
cov1_5_6_7_8_11	33.3323	-465.857	0.4140979	0	-0.1585869	0	0
cov1_2_5_6_7_8	33.3352	-465.859	0.4140693	0	-0.16152149	0	0.016773831
cov1_2_6_7_8_11	33.3393	-465.861	0.414062	0	-0.1860426	0	0.015004304
cov1_2_4_7_8_11	33.3468	-465.864	0.4124928	0	-0.13761456	0	0.038029256
cov1_2_4_5_7_8	33.3595	-465.871	0.4115842	0	-0.14582916	0	0.050144363
cov1_2_5_7_8_11	33.5039	-465.943	0.4138494	0	-0.12809828	0	0.016824502
cov1_4_5_7_8_11	33.5071	-465.945	0.4130458	0	-0.11659978	0	0
cov2_3_6_9_10_11	33.5967	-465.989	0.4128849	0	0	0	-0.034309062
cov1_3_4_5_6_8	33.7299	-466.056	0.4179394	0	-0.22975639	0	0
cov1_3_5_6_8_11	33.7461	-466.064	0.4190815	0	-0.23716498	0	0
cov1_2_3_5_6_8	33.7814	-466.082	0.4189281	0	-0.2343539	0	0.01170835
cov1_2_3_6_8_11	33.8748	-466.128	0.4179399	0	-0.20393547	0	0.012807349
cov1_3_4_6_8_11	33.8993	-466.141	0.4179451	0	-0.19421016	0	0
cov1_2_3_4_6_8	33.9112	-466.147	0.4171662	0	-0.19265865	0	0.020695247
cov1_2_3_4_5_8	33.9135	-466.148	0.4163538	0	-0.20878614	0	0.045943656
cov1_3_4_5_6_7	33.9146	-466.148	0.4176716	0	-0.22119585	0	0
cov1_3_5_6_7_11	33.9312	-466.157	0.418728	0	-0.22775845	0	0
cov1_2_3_5_6_7	33.9538	-466.168	0.4185845	0	-0.22633217	0	0.012296291
cov1_3_4_5_8_11	34.0055	-466.194	0.4179448	0	-0.18777697	0	0
cov1_2_3_6_7_11	34.0062	-466.194	0.4177759	0	-0.20249747	0	0.013067801
cov1_2_3_4_6_7	34.0236	-466.203	0.4168076	0	-0.19150239	0	0.022804056
cov1_3_4_6_7_11	34.0312	-466.207	0.4176533	0	-0.19120738	0	0
cov1_2_3_5_8_11	34.0437	-466.213	0.4187794	0	-0.19366146	0	0.011802885
cov1_2_3_4_5_6	34.1106	-466.246	0.4148291	0	-0.21130295	0	0.041109109
cov1_2_3_4_5_7	34.1145	-466.248	0.4160883	0	-0.19814021	0	0.045056674
cov1_2_3_4_6_11	34.1647	-466.273	0.415494	0	-0.18984306	0	0.029149744
cov1_2_3_4_8_11	34.1815	-466.282	0.4171669	0	-0.15144506	0	0.019299506
cov1_3_4_5_7_11	34.2135	-466.298	0.4176408	0	-0.17652283	0	0
cov1_2_4_5_6_8	34.2213	-466.302	0.4120725	0	-0.20942165	0	0.053171742
cov1_2_4_5_6_11	34.2284	-466.305	0.4128397	0	-0.20871045	0	0.047329326
cov1_2_4_6_8_11	34.2352	-466.309	0.4131828	0	-0.19309842	0	0.038708683
cov1_2_3_5_6_11	34.2386	-466.31	0.4168938	0	-0.19297627	0	0.014167001
cov1_2_3_5_7_11	34.2386	-466.31	0.4183923	0	-0.18311366	0	0.012600952
cov1_3_4_5_6_11	34.2423	-466.312	0.4161913	0	-0.18326248	0	0
cov1_2_4_5_6_7	34.2461	-466.314	0.4123615	0	-0.20658917	0	0.051237607
cov1_2_4_6_7_11	34.2593	-466.321	0.4133685	0	-0.19163397	0	0.038203413
cov1_2_3_4_7_11	34.3144	-466.348	0.416771	0	-0.14833205	0	0.021723775
cov1_4_5_6_8_11	34.4013	-466.392	0.413637	0	-0.17479757	0	0
cov1_4_5_6_7_11	34.4213	-466.402	0.4138667	0	-0.17234364	0	0
cov1_2_5_6_8_11	34.4292	-466.406	0.4146888	0	-0.18301374	0	0.015619695
cov1_2_5_6_7_11	34.4393	-466.411	0.414844	0	-0.1808084	0	0.015684151
cov1_2_3_4_5_11	34.4491	-466.415	0.4147254	0	-0.15862099	0	0.039888555
cov2_6_7_9_10_11	34.4562	-466.419	0.4100244	0	0	0	-0.035523714

cov2_6_8_9_10_11	34.4649	-466.423	0.409861	0	0	0	-0.036140668
cov2_3_5_6_7_8	34.5276	-466.455	0.4151871	0	0	0	-0.025056548
cov2_5_6_7_8_11	34.5325	-466.457	0.414406	0	0	0	-0.020155479
cov1_2_4_5_8_11	34.5341	-466.458	0.4120159	0	-0.16160211	0	0.051091008
cov2_4_5_6_7_8	34.5437	-466.463	0.4147072	0	0	0	-0.024888884
cov1_2_4_5_7_11	34.5692	-466.476	0.4123101	0	-0.15753475	0	0.049252041
cov4_5_6_7_8_11	34.5909	-466.486	0.4140796	0	0	0	0
cov2_4_5_7_8_11	34.5925	-466.487	0.4150598	0	0	0	-0.032115458
cov3_5_6_7_8_11	34.5977	-466.49	0.4149269	0	0	0	0
cov2_3_5_7_8_11	34.6001	-466.491	0.4149591	0	0	0	-0.029252607
cov3_4_5_6_7_8	34.6214	-466.502	0.4144275	0	0	0	0
cov2_3_4_5_7_8	34.6255	-466.504	0.4157973	0	0	0	-0.041668407
cov3_4_5_7_8_11	34.7382	-466.56	0.4140707	0	0	0	0
cov3_4_6_7_8_11	34.8823	-466.632	0.414024	0	0	0	0
cov2_3_4_6_7_8	34.9023	-466.642	0.4149432	0	0	0	-0.026427043
cov2_3_6_7_8_11	34.9397	-466.661	0.4167501	0	0	0	-0.042431612
cov2_4_6_7_8_11	34.9586	-466.67	0.4124609	0	0	0	-0.008837659
cov2_3_4_7_8_11	35.2734	-466.828	0.4144549	0	0	0	-0.040459642
cov2_3_5_6_8_11	35.8114	-467.097	0.4162049	0	0	0	-0.027770189
cov2_3_4_5_6_11	35.8158	-467.099	0.4164042	0	0	0	-0.030874108
cov2_3_4_5_6_8	35.8197	-467.101	0.4167878	0	0	0	-0.035711269
cov2_3_5_6_7_11	35.8231	-467.102	0.4158276	0	0	0	-0.025118126
cov2_3_4_5_6_7	35.8353	-467.109	0.4164005	0	0	0	-0.033103917
cov2_4_5_6_7_11	35.8367	-467.109	0.4154144	0	0	0	-0.025661039
cov2_3_4_5_7_11	35.8479	-467.115	0.4163478	0	0	0	-0.03564314
cov2_3_4_5_8_11	35.8531	-467.117	0.4167473	0	0	0	-0.039732448
cov2_4_5_6_8_11	35.8659	-467.124	0.4151818	0	0	0	-0.025325293
cov3_4_5_6_7_11	35.9139	-467.148	0.4150838	0	0	0	0
cov3_4_5_6_8_11	35.9213	-467.152	0.4153726	0	0	0	0
cov2_3_4_6_8_11	36.1503	-467.266	0.4159603	0	0	0	-0.027100995
cov2_3_4_6_7_11	36.2163	-467.299	0.4155137	0	0	0	-0.023830046
cov1_3_5_6_7_8_10	36.5518	-463.952	0.4118367	0	-0.11780964	0	0
cov1_3_4_6_7_8_10	36.6213	-463.987	0.4121	0	-0.149554	0	0
cov1_2_3_6_7_8_10	36.6497	-464.001	0.4135092	0	-0.18023755	0	0.018929956
cov1_3_6_7_8_10_11	36.6877	-464.02	0.4136588	0	-0.17087676	0	0
cov1_3_6_7_8_9_10	36.7038	-464.028	0.4135692	0	-0.16495314	0	0
cov1_3_5_6_7_8_9	36.803	-464.078	0.4114762	0	-0.11927123	0	0
cov1_3_4_6_7_8_9	36.8479	-464.1	0.4116008	0	-0.14730698	0	0
cov1_2_3_6_7_8_9	36.8886	-464.121	0.4130727	0	-0.17690961	0	0.017173742
cov1_3_6_7_8_9_11	36.9123	-464.133	0.4132147	0	-0.16977079	0	0
cov1_2_3_4_7_8_10	36.9364	-464.145	0.4100556	0	-0.10133355	0	0.049532677
cov1_2_3_5_7_8_10	37.0251	-464.189	0.4114397	0	-0.067673353	0	0.023842268
cov3_5_6_7_8_10_11	37.0734	-464.213	0.409133	0	0	0	0
cov1_3_5_7_8_10_11	37.0759	-464.214	0.4117973	0	-0.061918989	0	0
cov3_5_6_7_8_9_10	37.0796	-464.216	0.4089945	0	0	0	0
cov2_3_5_6_7_8_10	37.0835	-464.218	0.4090393	0	0	0	0.001932914
cov3_4_5_6_7_8_10	37.0841	-464.218	0.4091093	0	0	0	0

cov1_2_5_6_7_8_10	37.0859	-464.219	0.4075407	0	-0.07549715	0	0.026927911
cov1_3_4_5_7_8_10	37.1085	-464.231	0.4115289	0	-0.051785122	0	0
cov1_3_5_7_8_9_10	37.1104	-464.232	0.4116662	0	-0.051223213	0	0
cov1_2_3_7_8_10_11	37.1371	-464.245	0.413246	0	-0.12166515	0	0.019776633
cov1_3_4_7_8_10_11	37.1387	-464.246	0.4121537	0	-0.091784546	0	0
cov1_2_3_7_8_9_10	37.1667	-464.26	0.4131747	0	-0.11319577	0	0.020923684
cov1_3_4_7_8_9_10	37.1776	-464.265	0.412057	0	-0.081193986	0	0
cov1_2_3_4_7_8_9	37.1784	-464.266	0.4097044	0	-0.098744712	0	0.047116891
cov1_5_6_7_8_10_11	37.1805	-464.267	0.4076911	0	-0.061807917	0	0
cov1_2_4_6_7_8_10	37.184	-464.268	0.404917	0	-0.15806788	0	0.075712679
cov1_4_5_6_7_8_10	37.1918	-464.272	0.4074034	0	-0.05669651	0	0
cov1_5_6_7_8_9_10	37.1918	-464.272	0.4075216	0	-0.055286426	0	0
cov1_3_7_8_9_10_11	37.1957	-464.274	0.4133649	0	-0.10602832	0	0
cov2_3_4_5_7_8_10	37.2328	-464.293	0.4092439	0	0	0	0.01347606
cov2_3_5_7_8_9_10	37.2388	-464.296	0.4096882	0	0	0	0.00917481
cov2_3_5_7_8_10_11	37.24	-464.296	0.409736	0	0	0	0.009252512
cov3_5_7_8_9_10_11	37.2532	-464.303	0.4099572	0	0	0	0
cov3_4_5_7_8_9_10	37.2537	-464.303	0.4099705	0	0	0	0
cov3_4_5_7_8_10_11	37.2544	-464.304	0.4099962	0	0	0	0
cov1_2_3_5_7_8_9	37.2803	-464.317	0.411155	0	-0.068759825	0	0.021777537
cov1_2_3_4_6_8_10	37.2933	-464.323	0.4110035	0	-0.18238806	0	0.049344987
cov1_2_5_6_7_8_9	37.3067	-464.33	0.4073365	0	-0.077253011	0	0.024668135
cov1_3_5_7_8_9_11	37.3101	-464.331	0.4114829	0	-0.065127482	0	0
cov2_4_5_6_7_8_10	37.3135	-464.333	0.40636	0	0	0	0.017149025
cov2_5_6_7_8_9_10	37.3211	-464.337	0.4067947	0	0	0	0.011283428
cov2_5_6_7_8_10_11	37.3247	-464.339	0.4068789	0	0	0	0.012070932
cov3_5_6_7_8_9_11	37.3391	-464.346	0.4086666	0	0	0	0
cov4_5_6_7_8_9_10	37.3435	-464.348	0.4068618	0	0	0	0
cov5_6_7_8_9_10_11	37.3436	-464.348	0.406913	0	0	0	0
cov3_4_5_6_7_8_9	37.3458	-464.349	0.4085462	0	0	0	0
cov2_3_5_6_7_8_9	37.3463	-464.35	0.4086328	0	0	0	-0.000305285
cov1_3_4_5_7_8_9	37.3467	-464.35	0.4111311	0	-0.054416596	0	0
cov4_5_6_7_8_10_11	37.3494	-464.351	0.4069718	0	0	0	0
cov1_3_4_7_8_9_11	37.3516	-464.352	0.4117023	0	-0.091053626	0	0
cov1_2_4_6_7_8_9	37.3557	-464.354	0.4047643	0	-0.15554544	0	0.072434423
cov1_2_3_7_8_9_11	37.3622	-464.357	0.4128544	0	-0.11990882	0	0.01799845
cov1_5_6_7_8_9_11	37.379	-464.366	0.4074684	0	-0.065867395	0	0
cov1_4_5_6_7_8_9	37.3912	-464.372	0.4070822	0	-0.060286796	0	0
cov1_2_3_5_6_8_10	37.4305	-464.392	0.4127716	0	-0.1514677	0	0.021638101
cov1_3_5_6_8_10_11	37.4802	-464.416	0.4130667	0	-0.14410813	0	0
cov1_3_4_5_6_8_10	37.4891	-464.421	0.4126053	0	-0.13899255	0	0
cov2_3_4_5_7_8_9	37.4901	-464.421	0.4088018	0	0	0	0.011795884
cov2_3_5_7_8_9_11	37.4992	-464.426	0.4093516	0	0	0	0.006635346
cov1_3_5_6_8_9_10	37.5005	-464.427	0.4129282	0	-0.13693615	0	0
cov3_4_5_7_8_9_11	37.5059	-464.429	0.4094462	0	0	0	0
cov1_3_4_6_8_10_11	37.5089	-464.431	0.4130797	0	-0.16874954	0	0
cov1_2_3_4_6_7_10	37.518	-464.435	0.41079	0	-0.18206299	0	0.050953579

cov1_2_4_5_7_8_10	37.5207	-464.437	0.405829	0	-0.034392352	0	0.052376375
cov1_3_4_6_8_9_10	37.5326	-464.443	0.4129581	0	-0.16162662	0	0
cov1_2_3_6_8_10_11	37.5362	-464.444	0.414413	0	-0.19726627	0	0.018089379
cov2_4_5_6_7_8_9	37.5432	-464.448	0.4060655	0	0	0	0.01474688
cov1_2_3_6_8_9_10	37.5541	-464.453	0.4143335	0	-0.19173731	0	0.018916437
cov1_2_3_4_6_8_9	37.5543	-464.454	0.4106211	0	-0.17906763	0	0.046805673
cov2_5_6_7_8_9_11	37.5574	-464.455	0.4066263	0	0	0	0.009053998
cov4_5_6_7_8_9_11	37.5701	-464.461	0.406593	0	0	0	0
cov2_4_5_7_8_10_11	37.5725	-464.463	0.4061377	0	0	0	0.033758571
cov2_4_5_7_8_9_10	37.5752	-464.464	0.4060014	0	0	0	0.035984665
cov1_3_6_8_9_10_11	37.5853	-464.469	0.4144857	0	-0.18339312	0	0
cov1_2_5_7_8_10_11	37.6067	-464.48	0.4074211	0	-0.019449106	0	0.027573213
cov2_5_7_8_9_10_11	37.6252	-464.489	0.4071641	0	0	0	0.022666784
cov1_2_5_7_8_9_10	37.6332	-464.493	0.4072825	0	-0.010335998	0	0.028533626
cov1_2_3_5_6_7_10	37.6335	-464.493	0.4124346	0	-0.14401148	0	0.022177226
cov1_3_5_6_7_10_11	37.6927	-464.523	0.4127169	0	-0.13525803	0	0
cov1_3_4_5_6_7_10	37.6993	-464.526	0.4123316	0	-0.13091806	0	0
cov1_2_3_5_6_8_9	37.7027	-464.528	0.4124576	0	-0.15179046	0	0.019462801
cov1_3_5_6_7_9_10	37.7068	-464.53	0.4125868	0	-0.12910069	0	0
cov1_2_4_7_8_10_11	37.7161	-464.534	0.4050493	0	-0.097594503	0	0.073492042
cov4_5_7_8_9_10_11	37.718	-464.535	0.4073775	0	0	0	0
cov1_5_7_8_9_10_11	37.719	-464.536	0.4074774	0	0.000482866	0	0
cov1_4_5_7_8_10_11	37.7198	-464.536	0.40744	0	-0.000294679	0	0
cov1_2_4_5_7_8_9	37.7266	-464.54	0.405613	0	-0.037486487	0	0.051039175
cov1_3_5_6_8_9_11	37.7331	-464.543	0.4127297	0	-0.14664586	0	0
cov1_3_4_6_8_9_11	37.7405	-464.547	0.4125937	0	-0.16737471	0	0
cov1_3_4_5_6_8_9	37.7416	-464.547	0.4121671	0	-0.14105728	0	0
cov1_2_4_7_8_9_10	37.7424	-464.548	0.4049062	0	-0.089015334	0	0.074773154
cov1_4_5_7_8_9_10	37.7552	-464.554	0.4072827	0	0.011337921	0	0
cov1_3_4_6_7_10_11	37.7553	-464.554	0.4129238	0	-0.16694963	0	0
cov1_2_3_4_6_7_9	37.7682	-464.56	0.410389	0	-0.17871076	0	0.048456562
cov1_4_6_7_8_10_11	37.7705	-464.562	0.4069401	0	-0.12809625	0	0
cov1_3_4_6_7_9_10	37.773	-464.563	0.4128099	0	-0.16070333	0	0
cov1_2_3_6_8_9_11	37.7802	-464.566	0.4139924	0	-0.1947782	0	0.01620127
cov2_4_5_7_8_9_11	37.7869	-464.57	0.4059134	0	0	0	0.030564149
cov1_4_6_7_8_9_10	37.7929	-464.573	0.4067511	0	-0.12015019	0	0
cov1_2_3_6_7_10_11	37.7937	-464.573	0.4143772	0	-0.1967554	0	0.01823736
cov1_2_3_6_7_9_10	37.8066	-464.58	0.4143097	0	-0.19204841	0	0.018937472
cov1_2_5_7_8_9_11	37.8135	-464.583	0.4072555	0	-0.022750072	0	0.025310211
cov1_3_6_7_9_10_11	37.8437	-464.598	0.414446	0	-0.18276941	0	0
cov1_2_4_7_8_9_11	37.8772	-464.615	0.4049309	0	-0.09643407	0	0.070234063
cov1_4_6_7_8_9_11	37.8891	-464.621	0.4066828	0	-0.12777701	0	0
cov1_2_3_5_6_7_9	37.8983	-464.626	0.412113	0	-0.14419675	0	0.02002242
cov1_4_5_7_8_9_11	37.9066	-464.63	0.4071603	0	-0.005227524	0	0
cov1_3_5_6_7_9_11	37.939	-464.646	0.4123709	0	-0.13764478	0	0
cov1_3_4_5_6_7_9	37.9451	-464.649	0.4118873	0	-0.13283997	0	0
cov1_3_4_6_7_9_11	37.9768	-464.665	0.4124363	0	-0.16551357	0	0

cov1_2_3_4_5_6_10	37.9909	-464.672	0.4090687	0	-0.11763213	0	0.042547931
cov1_2_3_4_8_10_11	38.0012	-464.677	0.4112155	0	-0.11340705	0	0.046728595
cov1_2_3_6_7_9_11	38.0278	-464.69	0.413955	0	-0.19424331	0	0.016384356
cov1_2_3_4_8_9_10	38.0396	-464.696	0.4110873	0	-0.10361114	0	0.048317563
cov1_2_3_4_5_8_10	38.0399	-464.696	0.411042	0	-0.10136486	0	0.047715585
cov1_2_3_5_6_9_10	38.0527	-464.703	0.4103437	0	-0.10194566	0	0.024705715
cov1_2_3_5_6_10_11	38.0534	-464.703	0.4103896	0	-0.10174918	0	0.024881132
cov3_4_6_7_8_10_11	38.0745	-464.714	0.4090317	0	0	0	0
cov1_2_4_5_6_8_10	38.0754	-464.714	0.4062667	0	-0.11705161	0	0.055638714
cov3_4_7_8_9_10_11	38.0827	-464.718	0.4085912	0	0	0	0
cov2_3_4_7_8_10_11	38.0835	-464.718	0.4082738	0	0	0	0.008048574
cov1_2_4_5_6_7_10	38.111	-464.732	0.4065451	0	-0.11441955	0	0.053700275
cov1_2_3_5_8_10_11	38.1144	-464.734	0.4127704	0	-0.088999842	0	0.022233592
cov1_2_4_5_6_10_11	38.1289	-464.741	0.4070327	0	-0.11174254	0	0.050028018
cov1_2_4_5_6_9_10	38.1292	-464.741	0.4068885	0	-0.10958933	0	0.050560062
cov1_3_4_5_6_9_10	38.1441	-464.748	0.4104946	0	-0.08436873	0	0
cov1_3_5_6_9_10_11	38.1441	-464.748	0.4104647	0	-0.08462728	0	0
cov1_3_4_5_6_10_11	38.1459	-464.749	0.4105667	0	-0.084857213	0	0
cov1_2_3_5_8_9_10	38.1553	-464.754	0.4126562	0	-0.078519103	0	0.023575833
cov3_4_6_7_8_9_10	38.1721	-464.762	0.4087495	0	0	0	0
cov2_3_4_6_7_8_10	38.178	-464.765	0.4086396	0	0	0	0.005229778
cov1_3_4_5_8_10_11	38.1822	-464.767	0.4127758	0	-0.075988374	0	0
cov1_3_5_8_9_10_11	38.1879	-464.77	0.4130183	0	-0.074865696	0	0
cov1_2_3_8_9_10_11	38.202	-464.777	0.4141315	0	-0.12518796	0	0.019904657
cov1_2_3_4_6_10_11	38.2096	-464.781	0.4086658	0	-0.16831921	0	0.062201076
cov1_2_3_4_5_6_9	38.2125	-464.783	0.4087218	0	-0.12014214	0	0.041535854
cov1_2_3_4_6_9_10	38.2128	-464.783	0.4086407	0	-0.17061935	0	0.06181859
cov3_5_6_8_9_10_11	38.2139	-464.783	0.4097851	0	0	0	0
cov1_3_4_8_9_10_11	38.2143	-464.784	0.4131341	0	-0.096375487	0	0
cov3_4_5_6_8_10_11	38.2179	-464.785	0.4098398	0	0	0	0
cov2_3_5_6_8_10_11	38.218	-464.785	0.4098867	0	0	0	-0.000449801
cov1_2_5_6_8_10_11	38.2226	-464.788	0.4082149	0	-0.093023797	0	0.026313173
cov2_3_5_6_8_9_10	38.2258	-464.789	0.4097823	0	0	0	-0.001922257
cov3_4_5_6_8_9_10	38.2262	-464.79	0.4096935	0	0	0	0
cov2_3_4_5_6_8_10	38.2305	-464.792	0.4099072	0	0	0	-0.00214178
cov1_3_4_5_8_9_10	38.2338	-464.793	0.4126556	0	-0.062957463	0	0
cov1_2_5_6_8_9_10	38.2366	-464.795	0.4080471	0	-0.086751092	0	0.026933783
cov1_2_6_7_8_10_11	38.2411	-464.797	0.4096731	0	-0.17807278	0	0.020366636
cov1_2_5_6_7_10_11	38.2437	-464.798	0.4083589	0	-0.091014821	0	0.026358235
cov1_2_3_4_8_9_11	38.2456	-464.799	0.4108865	0	-0.11181107	0	0.044141991
cov1_2_5_6_9_10_11	38.2457	-464.799	0.408387	0	-0.089131057	0	0.026244141
cov1_2_3_4_7_10_11	38.2475	-464.8	0.4109561	0	-0.11106628	0	0.048662861
cov1_2_5_6_7_9_10	38.2518	-464.802	0.4082239	0	-0.085988995	0	0.02683285
cov1_2_6_7_8_9_10	38.2609	-464.807	0.4095707	0	-0.17189347	0	0.021128651
cov3_4_6_7_8_9_11	38.2659	-464.809	0.4084955	0	0	0	0
cov2_3_4_7_8_9_10	38.2679	-464.81	0.4080515	0	0	0	-0.005205277
cov1_2_4_6_8_10_11	38.2697	-464.811	0.4054475	0	-0.17484201	0	0.07639276

cov1_2_3_4_5_7_10	38.2708	-464.812	0.4107658	0	-0.091013542	0	0.046808416
cov2_3_4_7_8_9_11	38.2754	-464.814	0.4078277	0	0	0	0.006494529
cov1_2_3_4_7_9_10	38.2772	-464.815	0.410836	0	-0.10242393	0	0.050072035
cov1_2_4_6_8_9_10	38.283	-464.818	0.4052879	0	-0.16928268	0	0.077322164
cov1_2_3_5_6_9_11	38.2837	-464.818	0.4101327	0	-0.10418619	0	0.022555739
cov1_4_7_8_9_10_11	38.2871	-464.82	0.4069042	0	-0.065151444	0	0
cov1_2_4_5_6_8_9	38.2877	-464.82	0.4060191	0	-0.1195995	0	0.054217894
cov1_2_3_4_5_8_9	38.292	-464.822	0.410723	0	-0.10344803	0	0.046598119
cov1_6_7_8_9_10_11	38.3022	-464.827	0.4096478	0	-0.16228732	0	0
cov1_4_5_6_8_10_11	38.3178	-464.835	0.4079859	0	-0.076546619	0	0
cov1_2_4_5_6_7_9	38.3226	-464.838	0.406295	0	-0.11696002	0	0.05228152
cov1_5_6_8_9_10_11	38.3226	-464.838	0.408212	0	-0.074529142	0	0
cov1_2_4_6_7_10_11	38.3302	-464.841	0.4056608	0	-0.17368231	0	0.075677612
cov3_5_6_7_9_10_11	38.3309	-464.842	0.4095723	0	0	0	0
cov2_3_5_6_7_10_11	38.3348	-464.844	0.4096268	0	0	0	0.001654194
cov3_4_5_6_7_10_11	38.3352	-464.844	0.4096803	0	0	0	0
cov1_2_4_5_6_9_11	38.337	-464.845	0.4067988	0	-0.11506117	0	0.048421411
cov1_2_4_6_7_9_10	38.338	-464.845	0.4055078	0	-0.16916268	0	0.076401237
cov1_4_5_6_8_9_10	38.338	-464.845	0.4077999	0	-0.06837674	0	0
cov1_2_3_5_7_10_11	38.3408	-464.847	0.4123788	0	-0.078754739	0	0.022998582
cov1_4_5_6_7_10_11	38.3426	-464.848	0.408195	0	-0.074170978	0	0
cov1_5_6_7_9_10_11	38.3442	-464.848	0.408356	0	-0.072494634	0	0
cov2_3_5_6_7_9_10	38.3451	-464.849	0.4095161	0	0	0	3.36E-05
cov3_4_5_6_7_9_10	38.3451	-464.849	0.4095245	0	0	0	0
cov1_4_5_6_9_10_11	38.3453	-464.849	0.4082734	0	-0.071686121	0	0
cov2_3_4_5_6_7_10	38.3501	-464.851	0.409635	0	0	0	-0.000176456
cov1_2_6_7_8_9_11	38.3543	-464.854	0.409438	0	-0.17632368	0	0.018892705
cov2_3_4_6_7_8_9	38.3551	-464.854	0.408158	0	0	0	0.003958099
cov1_4_5_6_7_9_10	38.3552	-464.854	0.4080257	0	-0.067331215	0	0
cov1_3_4_5_6_9_11	38.3595	-464.856	0.4101668	0	-0.088896136	0	0
cov1_2_3_5_8_9_11	38.3671	-464.86	0.4125129	0	-0.091189673	0	0.020057864
cov1_2_3_5_7_9_10	38.3723	-464.863	0.4123034	0	-0.069513751	0	0.024169738
cov1_2_3_4_6_9_11	38.3934	-464.873	0.4083926	0	-0.16680643	0	0.059397971
cov1_2_4_6_9_10_11	38.4038	-464.878	0.4061718	0	-0.16891655	0	0.073379068
cov1_3_4_5_7_10_11	38.4165	-464.885	0.4124538	0	-0.064940167	0	0
cov1_3_4_5_8_9_11	38.4166	-464.885	0.4123981	0	-0.07973788	0	0
cov1_3_5_7_9_10_11	38.4195	-464.886	0.4126237	0	-0.064099542	0	0
cov2_3_4_5_8_9_10	38.4351	-464.894	0.4101786	0	0	0	0.010718309
cov2_3_4_5_8_10_11	38.4363	-464.895	0.4102315	0	0	0	0.010755332
cov1_2_5_6_8_9_11	38.4385	-464.896	0.4080242	0	-0.095697445	0	0.023942958
cov1_2_4_6_8_9_11	38.4388	-464.896	0.4053091	0	-0.17308535	0	0.072985435
cov2_3_5_8_9_10_11	38.4419	-464.897	0.4106548	0	0	0	0.006323268
cov2_3_5_6_9_10_11	38.448	-464.9	0.4088066	0	0	0	0.007048576
cov3_4_5_8_9_10_11	38.4482	-464.9	0.4107667	0	0	0	0
cov2_3_4_5_6_10_11	38.4523	-464.903	0.4088093	0	0	0	0.008065524
cov3_4_5_6_9_10_11	38.4548	-464.904	0.4090824	0	0	0	0
cov1_3_4_5_7_9_10	38.4575	-464.905	0.4123404	0	-0.053208627	0	0

cov1_2_5_6_7_9_11	38.4595	-464.906	0.4081683	0	-0.093680468	0	0.023991023
cov2_3_4_5_6_9_10	38.47	-464.911	0.4087464	0	0	0	0.005371877
cov1_2_3_7_9_10_11	38.4753	-464.914	0.4140773	0	-0.12362592	0	0.020082938
cov1_3_4_7_9_10_11	38.4785	-464.916	0.4129569	0	-0.093335419	0	0
cov1_2_3_4_7_9_11	38.4813	-464.917	0.4106238	0	-0.10947298	0	0.046141407
cov3_4_5_6_8_9_11	38.4938	-464.923	0.409274	0	0	0	0
cov2_3_5_6_8_9_11	38.4939	-464.923	0.4094818	0	0	0	-0.003053491
cov1_2_4_6_7_9_11	38.4979	-464.925	0.4055232	0	-0.17193166	0	0.072291316
cov2_3_4_5_6_8_9	38.5016	-464.927	0.4094166	0	0	0	-0.003642728
cov1_4_5_6_8_9_11	38.5105	-464.932	0.4076748	0	-0.081002235	0	0
cov1_2_3_4_5_7_9	38.5158	-464.934	0.4104399	0	-0.092965917	0	0.045692924
cov1_4_5_6_7_9_11	38.5361	-464.944	0.4078837	0	-0.078647471	0	0
cov2_4_5_6_8_9_10	38.5409	-464.947	0.4067283	0	0	0	0.014928088
cov2_4_5_6_8_10_11	38.5445	-464.949	0.4067897	0	0	0	0.01617274
cov2_4_5_6_9_10_11	38.5487	-464.951	0.4069411	0	0	0	0.015570664
cov2_5_6_8_9_10_11	38.554	-464.953	0.4072794	0	0	0	0.009485226
cov2_4_5_6_7_9_10	38.5546	-464.954	0.4070025	0	0	0	0.013886842
cov2_4_5_6_7_10_11	38.5549	-464.954	0.4070397	0	0	0	0.015620618
cov2_5_6_7_9_10_11	38.5604	-464.957	0.4074421	0	0	0	0.009921781
cov4_5_6_8_9_10_11	38.5682	-464.96	0.4072627	0	0	0	0
cov4_5_6_7_9_10_11	38.5769	-464.965	0.4074951	0	0	0	0
cov2_3_6_7_8_10_11	38.5819	-464.967	0.4130597	0	0	0	-0.035389926
cov1_2_3_5_7_9_11	38.5868	-464.97	0.4121136	0	-0.08083194	0	0.020840941
cov2_3_4_5_7_9_10	38.5876	-464.97	0.4099441	0	0	0	0.013704165
cov2_3_4_5_7_10_11	38.5887	-464.971	0.4099948	0	0	0	0.013843893
cov1_3_4_6_9_10_11	38.594	-464.973	0.410941	0	-0.14204276	0	0
cov2_3_5_7_9_10_11	38.5956	-464.974	0.4104638	0	0	0	0.009018569
cov3_4_5_6_7_9_11	38.6021	-464.977	0.4091137	0	0	0	0
cov2_3_5_6_7_9_11	38.6025	-464.978	0.40922	0	0	0	-0.00090361
cov3_4_5_7_9_10_11	38.6092	-464.981	0.4107035	0	0	0	0
cov2_3_4_5_6_7_9	38.6125	-464.983	0.4091506	0	0	0	-0.001640026
cov1_3_4_5_7_9_11	38.645	-464.999	0.4120704	0	-0.068572319	0	0
cov2_4_7_8_9_10_11	38.6524	-465.003	0.4039032	0	0	0	0.033576411
cov2_4_6_7_8_10_11	38.6573	-465.005	0.4041258	0	0	0	0.035700598
cov1_2_7_8_9_10_11	38.6752	-465.014	0.4093744	0	-0.11915273	0	0.021693837
cov2_3_7_8_9_10_11	38.6754	-465.014	0.4129951	0	0	0	-0.047327475
cov2_3_6_7_8_9_10	38.6984	-465.026	0.4131419	0	0	0	-0.042082324
cov2_3_4_5_6_9_11	38.7006	-465.027	0.4084039	0	0	0	0.005741345
cov2_3_4_5_8_9_11	38.7014	-465.027	0.4098019	0	0	0	0.008559127
cov1_2_3_4_5_10_11	38.7136	-465.033	0.4091664	0	-0.045231323	0	0.041838216
cov1_2_3_4_5_9_10	38.7165	-465.035	0.4091335	0	-0.042986272	0	0.042297323
cov2_4_6_7_8_9_10	38.7405	-465.047	0.4041268	0	0	0	0.029584206
cov1_2_3_6_9_10_11	38.7477	-465.05	0.4132451	0	-0.17989569	0	0.019516713
cov1_2_3_5_9_10_11	38.7614	-465.057	0.410274	0	-0.032780698	0	0.02625561
cov2_3_6_7_8_9_11	38.7622	-465.057	0.4125766	0	0	0	-0.036480486
cov2_4_5_6_8_9_11	38.7787	-465.066	0.4065163	0	0	0	0.013340814
cov2_4_5_6_7_9_11	38.7887	-465.071	0.4067669	0	0	0	0.012794588

cov2_4_6_7_8_9_11	38.7907	-465.072	0.4039023	0	0	0	0.032914858
cov4_6_7_8_9_10_11	38.7972	-465.075	0.4051163	0	0	0	0
cov2_3_4_5_9_10_11	38.8038	-465.078	0.4089822	0	0	0	0.024398447
cov1_2_4_5_8_10_11	38.8058	-465.079	0.4063877	0	-0.048893912	0	0.053882041
cov3_6_7_8_9_10_11	38.8213	-465.087	0.4126621	0	0	0	0
cov2_3_4_5_7_9_11	38.8448	-465.099	0.4095673	0	0	0	0.011696921
cov1_2_4_5_8_9_10	38.8457	-465.099	0.406217	0	-0.038151163	0	0.055366987
cov1_2_4_5_7_10_11	38.8465	-465.1	0.4066701	0	-0.044820528	0	0.051989017
cov1_3_4_5_9_10_11	38.8622	-465.107	0.410605	0	-0.014176084	0	0
cov1_2_4_5_9_10_11	38.8657	-465.109	0.4069988	0	-0.038941846	0	0.049317395
cov1_2_4_5_7_9_10	38.876	-465.114	0.4065152	0	-0.03563248	0	0.053271045
cov1_4_6_8_9_10_11	38.884	-465.118	0.4073434	0	-0.13991116	0	0
cov2_4_5_8_9_10_11	38.9044	-465.129	0.4065527	0	0	0	0.033991569
cov1_2_3_4_9_10_11	38.9143	-465.134	0.4088325	0	-0.095447647	0	0.060420297
cov1_2_3_4_5_9_11	38.9254	-465.139	0.4088728	0	-0.049160807	0	0.040708011
cov2_4_5_7_9_10_11	38.9292	-465.141	0.4068179	0	0	0	0.033754392
cov1_4_6_7_9_10_11	38.933	-465.143	0.4075425	0	-0.13901057	0	0
cov1_2_5_8_9_10_11	38.9411	-465.147	0.4079957	0	-0.022963282	0	0.027683391
cov1_2_5_7_9_10_11	38.9622	-465.158	0.4081593	0	-0.020779277	0	0.027734522
cov1_2_4_8_9_10_11	38.9999	-465.176	0.405511	0	-0.10028381	0	0.074793535
cov1_2_4_5_8_9_11	39.0026	-465.178	0.4061869	0	-0.052865101	0	0.052428432
cov1_2_4_5_7_9_11	39.0437	-465.198	0.4064681	0	-0.048808772	0	0.050538816
cov1_4_5_8_9_10_11	39.0524	-465.203	0.4079144	0	-0.004010701	0	0
cov1_2_4_7_9_10_11	39.0614	-465.207	0.4057489	0	-0.098963915	0	0.07401642
cov1_4_5_7_9_10_11	39.076	-465.214	0.408146	0	-0.001473948	0	0
cov3_4_6_8_9_10_11	39.2507	-465.302	0.4096099	0	0	0	0
cov2_3_4_6_8_10_11	39.2521	-465.302	0.409398	0	0	0	0.00696111
cov2_3_4_8_9_10_11	39.2521	-465.302	0.4091479	0	0	0	0.005143376
cov2_3_4_6_8_9_10	39.3649	-465.359	0.4094234	0	0	0	0.000626403
cov1_2_6_8_9_10_11	39.3947	-465.374	0.4102468	0	-0.19142982	0	0.020231936
cov1_2_6_7_9_10_11	39.4251	-465.389	0.4104042	0	-0.18985132	0	0.020255944
cov2_3_4_7_9_10_11	39.4453	-465.399	0.4088885	0	0	0	0.008056207
cov2_3_4_6_7_10_11	39.4486	-465.401	0.4091098	0	0	0	0.009397701
cov3_4_6_7_9_10_11	39.4512	-465.402	0.4094334	0	0	0	0
cov2_3_4_6_8_9_11	39.454	-465.403	0.408951	0	0	0	0.005050002
cov2_3_4_6_7_9_10	39.5768	-465.465	0.4091357	0	0	0	0.002649771
cov2_3_4_6_7_9_11	39.6406	-465.497	0.4086669	0	0	0	0.00754198
cov2_3_6_8_9_10_11	39.7646	-465.559	0.4138713	0	0	0	-0.039852301
cov1_3_4_5_6_7_8	39.8145	-465.584	0.4169863	0	-0.20997128	0	0
cov1_3_5_6_7_8_11	39.8282	-465.59	0.4179973	0	-0.21649568	0	0
cov2_3_4_6_9_10_11	39.8298	-465.591	0.4073992	0	0	0	0.021427669
cov1_2_3_5_6_7_8	39.8497	-465.601	0.4178589	0	-0.21518992	0	0.012275561
cov1_2_3_4_5_7_8	39.8885	-465.621	0.4154349	0	-0.19775775	0	0.044216659
cov1_2_3_6_7_8_11	39.906	-465.629	0.4170316	0	-0.19077741	0	0.013092012
cov1_2_3_4_6_7_8	39.9256	-465.639	0.41614	0	-0.18012582	0	0.0221408
cov1_3_4_6_7_8_11	39.9315	-465.642	0.4169556	0	-0.17996481	0	0
cov1_3_4_5_7_8_11	39.9885	-465.671	0.4169471	0	-0.17605492	0	0

cov2_4_6_8_9_10_11	39.9933	-465.673	0.4043845	0	0	0	0.033834865
cov2_3_6_7_9_10_11	40.0039	-465.678	0.4137944	0	0	0	-0.039370829
cov1_2_3_5_7_8_11	40.014	-465.683	0.4176968	0	-0.18244273	0	0.012281845
cov2_4_6_7_9_10_11	40.0279	-465.69	0.4046175	0	0	0	0.033382155
cov2_6_7_8_9_10_11	40.0398	-465.696	0.4092233	0	0	0	-0.031626822
cov1_2_3_4_7_8_11	40.0951	-465.724	0.4161041	0	-0.14726291	0	0.020977951
cov1_2_4_5_6_7_8	40.1415	-465.747	0.4117486	0	-0.1952393	0	0.050325008
cov1_2_4_6_7_8_11	40.1554	-465.754	0.4127422	0	-0.18022963	0	0.037335787
cov1_4_5_6_7_8_11	40.3104	-465.832	0.413214	0	-0.1616088	0	0
cov1_2_5_6_7_8_11	40.3248	-465.839	0.4141489	0	-0.17011978	0	0.015626013
cov1_2_4_5_7_8_11	40.3365	-465.845	0.4116961	0	-0.1571834	0	0.048427789
cov1_2_3_4_5_6_8	40.5786	-465.966	0.4164614	0	-0.26737384	0	0.046085747
cov1_3_4_5_6_8_11	40.6997	-466.026	0.4180545	0	-0.24124724	0	0
cov1_2_3_5_6_8_11	40.7591	-466.056	0.4190522	0	-0.2440843	0	0.010396326
cov1_2_3_4_5_6_7	40.7697	-466.061	0.4162218	0	-0.25824647	0	0.045265406
cov1_2_3_4_5_8_11	40.8717	-466.112	0.4165191	0	-0.22196875	0	0.04391349
cov1_2_3_4_6_8_11	40.8921	-466.122	0.4173087	0	-0.20192253	0	0.019099009
cov1_3_4_5_6_7_11	40.8954	-466.124	0.4177761	0	-0.23169264	0	0
cov1_2_3_5_6_7_11	40.9419	-466.147	0.4186975	0	-0.23513324	0	0.011115256
cov1_2_3_4_6_7_11	41.0148	-466.184	0.4169364	0	-0.1997815	0	0.02137546
cov1_2_3_4_5_7_11	41.0848	-466.219	0.4162394	0	-0.2102362	0	0.043221308
cov1_2_3_4_5_6_11	41.1329	-466.243	0.4148306	0	-0.21367082	0	0.040444661
cov1_2_4_5_6_8_11	41.2043	-466.279	0.4121891	0	-0.21824168	0	0.051528465
cov1_2_4_5_6_7_11	41.2394	-466.296	0.412466	0	-0.21443049	0	0.049801823
cov2_3_5_6_7_8_11	41.5372	-466.445	0.4152054	0	0	0	-0.023254229
cov2_3_4_5_6_7_8	41.5482	-466.45	0.4156834	0	0	0	-0.030111173
cov2_4_5_6_7_8_11	41.5599	-466.456	0.4145822	0	0	0	-0.022293867
cov2_3_4_5_7_8_11	41.6126	-466.483	0.4156706	0	0	0	-0.035990028
cov3_4_5_6_7_8_11	41.6135	-466.483	0.4144904	0	0	0	0
cov2_3_4_6_7_8_11	41.8601	-466.606	0.414878	0	0	0	-0.021668499
cov2_3_4_5_6_8_11	42.8316	-467.092	0.4167152	0	0	0	-0.033138583
cov2_3_4_5_6_7_11	42.844	-467.098	0.4163216	0	0	0	-0.03027122
cov1_2_3_4_6_7_8_10	43.401	-463.862	0.4100887	0	-0.17059975	0	0.050351129
cov1_2_3_5_6_7_8_10	43.5071	-463.915	0.4116558	0	-0.13254919	0	0.022189221
cov1_3_5_6_7_8_10_11	43.5672	-463.945	0.4119341	0	-0.12363172	0	0
cov1_3_4_5_6_7_8_10	43.5746	-463.949	0.4115867	0	-0.11928672	0	0
cov1_3_5_6_7_8_9_10	43.5806	-463.952	0.4118067	0	-0.1176105	0	0
cov1_3_4_6_7_8_10_11	43.6334	-463.979	0.4121878	0	-0.15555181	0	0
cov1_3_4_6_7_8_9_10	43.6502	-463.987	0.4120767	0	-0.14944654	0	0
cov1_2_3_4_6_7_8_9	43.6511	-463.987	0.4096938	0	-0.16724699	0	0.047868291
cov1_2_3_6_7_8_10_11	43.6665	-463.995	0.4135881	0	-0.18489563	0	0.018284817
cov1_2_3_6_7_8_9_10	43.6787	-464.001	0.4135229	0	-0.1803114	0	0.018959949
cov1_3_6_7_8_9_10_11	43.7168	-464.02	0.4136556	0	-0.17086795	0	0
cov1_2_3_5_6_7_8_9	43.7719	-464.048	0.4113284	0	-0.13270833	0	0.020029558
cov1_3_5_6_7_8_9_11	43.8136	-464.069	0.4115839	0	-0.12599894	0	0
cov1_3_4_5_6_7_8_9	43.8209	-464.072	0.4111383	0	-0.1211958	0	0
cov1_3_4_6_7_8_9_11	43.8552	-464.089	0.4116956	0	-0.15410793	0	0

cov1_2_3_6_7_8_9_11	43.9008	-464.112	0.4131613	0	-0.18237359	0	0.016426013
cov1_2_3_4_7_8_10_11	43.9401	-464.132	0.4101914	0	-0.10945474	0	0.048262728
cov1_2_3_4_5_7_8_10	43.9572	-464.14	0.4100073	0	-0.089033541	0	0.045967097
cov1_2_3_4_7_8_9_10	43.9652	-464.144	0.4100793	0	-0.10149222	0	0.049546749
cov1_2_4_5_6_7_8_10	43.9812	-464.152	0.4058727	0	-0.10264555	0	0.052789438
cov1_2_3_5_7_8_10_11	44.0273	-464.176	0.411566	0	-0.076524186	0	0.022824238
cov1_2_3_5_7_8_9_10	44.054	-464.189	0.4114606	0	-0.067936342	0	0.023888805
cov3_5_6_7_8_9_10_11	44.0985	-464.211	0.4090422	0	0	0	0
cov2_3_5_6_7_8_10_11	44.1007	-464.212	0.4090542	0	0	0	0.003311299
cov1_3_4_5_7_8_10_11	44.1025	-464.213	0.4116492	0	-0.06274636	0	0
cov3_4_5_6_7_8_10_11	44.1025	-464.213	0.4091506	0	0	0	0
cov1_3_5_7_8_9_10_11	44.105	-464.214	0.4117964	0	-0.061913629	0	0
cov1_2_5_6_7_8_10_11	44.1056	-464.215	0.4076106	0	-0.079960285	0	0.026337216
cov2_3_5_6_7_8_9_10	44.1081	-464.216	0.4089478	0	0	0	0.001883187
cov3_4_5_6_7_8_9_10	44.1086	-464.216	0.409005	0	0	0	0
cov2_3_4_5_6_7_8_10	44.1125	-464.218	0.4089802	0	0	0	0.002546213
cov1_2_5_6_7_8_9_10	44.1129	-464.218	0.4074773	0	-0.075065808	0	0.026787934
cov1_3_4_5_7_8_9_10	44.1376	-464.231	0.411535	0	-0.051830773	0	0
cov1_2_3_7_8_9_10_11	44.1656	-464.245	0.4132822	0	-0.12188698	0	0.019853668
cov1_3_4_7_8_9_10_11	44.1678	-464.246	0.4121609	0	-0.091824536	0	0
cov1_2_3_4_7_8_9_11	44.1758	-464.25	0.4098483	0	-0.10780777	0	0.045726685
cov1_2_4_5_6_7_8_9	44.1934	-464.259	0.4056192	0	-0.10518102	0	0.051366304
cov1_2_4_6_7_8_10_11	44.204	-464.264	0.4049934	0	-0.16214013	0	0.074928125
cov1_2_3_4_5_7_8_9	44.2042	-464.264	0.4096689	0	-0.090905747	0	0.044835648
cov1_4_5_6_7_8_10_11	44.2056	-464.265	0.407481	0	-0.062971609	0	0
cov1_5_6_7_8_9_10_11	44.2058	-464.265	0.407607	0	-0.061446471	0	0
cov1_2_4_6_7_8_9_10	44.2111	-464.267	0.4048553	0	-0.15772787	0	0.075621249
cov1_4_5_6_7_8_9_10	44.2172	-464.27	0.407314	0	-0.056299547	0	0
cov2_3_4_5_7_8_9_10	44.2603	-464.292	0.4091822	0	0	0	0.013608251
cov2_3_4_5_7_8_10_11	44.2615	-464.293	0.4092364	0	0	0	0.013989424
cov2_3_5_7_8_9_10_11	44.2679	-464.296	0.4096907	0	0	0	0.009240259
cov1_2_3_5_7_8_9_11	44.2756	-464.3	0.4112898	0	-0.078534743	0	0.020642636
cov3_4_5_7_8_9_10_11	44.2822	-464.303	0.409945	0	0	0	0
cov1_2_3_4_6_8_10_11	44.3091	-464.316	0.4111178	0	-0.18727989	0	0.048410093
cov1_2_3_4_5_6_8_10	44.3213	-464.323	0.4109896	0	-0.17796374	0	0.048036207
cov1_2_5_6_7_8_9_11	44.3218	-464.323	0.4074159	0	-0.082619063	0	0.023963835
cov1_2_3_4_6_8_9_10	44.3224	-464.323	0.410998	0	-0.18234664	0	0.049340008
cov1_3_4_5_7_8_9_11	44.3334	-464.329	0.4112545	0	-0.066336518	0	0
cov2_4_5_6_7_8_9_10	44.3362	-464.33	0.406246	0	0	0	0.017200468
cov2_4_5_6_7_8_10_11	44.3384	-464.331	0.4062957	0	0	0	0.018588792
cov2_5_6_7_8_9_10_11	44.3483	-464.336	0.4067835	0	0	0	0.011941429
cov3_4_5_6_7_8_9_11	44.3676	-464.346	0.4085857	0	0	0	0
cov2_3_5_6_7_8_9_11	44.3681	-464.346	0.4086481	0	0	0	0.00076445
cov1_2_4_6_7_8_9_11	44.372	-464.348	0.4048499	0	-0.16038024	0	0.071531456
cov4_5_6_7_8_9_10_11	44.3724	-464.348	0.4068597	0	0	0	0
cov2_3_4_5_6_7_8_9	44.3747	-464.349	0.4084911	0	0	0	0.001087407
cov1_4_5_6_7_8_9_11	44.4	-464.362	0.4071664	0	-0.06745598	0	0

cov1_2_3_5_6_8_10_11	44.4443	-464.384	0.4128782	0	-0.15708263	0	0.020886811
cov1_2_3_5_6_8_9_10	44.4596	-464.392	0.4127671	0	-0.15143752	0	0.021631495
cov1_3_4_5_6_8_10_11	44.4968	-464.41	0.4127142	0	-0.14630522	0	0
cov1_3_5_6_8_9_10_11	44.509	-464.416	0.4130431	0	-0.14396674	0	0
cov1_3_4_5_6_8_9_10	44.518	-464.421	0.4125796	0	-0.13884489	0	0
cov2_3_4_5_7_8_9_11	44.5192	-464.421	0.4087998	0	0	0	0.011842904
cov1_2_4_5_7_8_10_11	44.5248	-464.424	0.4059425	0	-0.042687369	0	0.051180483
cov1_3_4_6_8_9_10_11	44.5378	-464.431	0.413052	0	-0.16865958	0	0
cov1_2_3_4_5_6_7_10	44.5382	-464.431	0.410743	0	-0.16948413	0	0.047278635
cov1_2_3_4_6_7_10_11	44.5384	-464.431	0.4108818	0	-0.18604942	0	0.050194943
cov1_2_3_4_6_7_9_10	44.5471	-464.435	0.4107755	0	-0.18193333	0	0.05092333
cov1_2_4_5_7_8_9_10	44.5492	-464.436	0.4057934	0	-0.034099135	0	0.052312352
cov1_2_3_4_6_8_9_11	44.5649	-464.444	0.4107467	0	-0.18485173	0	0.045726956
cov1_2_3_6_8_9_10_11	44.5652	-464.444	0.4144259	0	-0.19732729	0	0.018119979
cov2_4_5_6_7_8_9_11	44.5701	-464.447	0.4060222	0	0	0	0.015779572
cov1_2_3_4_5_6_8_9	44.5834	-464.454	0.4106205	0	-0.17918583	0	0.046840276
cov2_4_5_7_8_9_10_11	44.5993	-464.461	0.4060677	0	0	0	0.033835968
cov1_2_5_7_8_9_10_11	44.635	-464.479	0.4073821	0	-0.019155322	0	0.027485138
cov1_2_3_5_6_7_10_11	44.6526	-464.488	0.4125234	0	-0.14860764	0	0.021567716
cov1_2_3_5_6_7_9_10	44.6625	-464.493	0.4124235	0	-0.1439196	0	0.022157419
cov1_2_3_5_6_8_9_11	44.7107	-464.517	0.4125772	0	-0.15834364	0	0.018596347
cov1_3_4_5_6_7_10_11	44.7133	-464.519	0.4124248	0	-0.1371154	0	0
cov1_3_5_6_7_9_10_11	44.7214	-464.523	0.4126848	0	-0.13507181	0	0
cov1_2_4_5_7_8_9_11	44.7243	-464.524	0.4057317	0	-0.046652305	0	0.049708446
cov1_3_4_5_6_7_9_10	44.7281	-464.526	0.4122991	0	-0.13072043	0	0
cov1_3_4_5_6_8_9_11	44.7429	-464.533	0.4122856	0	-0.14929008	0	0
cov1_2_4_7_8_9_10_11	44.7445	-464.534	0.4050135	0	-0.097361689	0	0.073431526
cov1_4_5_7_8_9_10_11	44.7471	-464.535	0.4073772	0	3.15E-05	0	0
cov1_3_4_6_7_9_10_11	44.7842	-464.554	0.4129026	0	-0.16682701	0	0
cov1_2_3_4_6_7_9_11	44.7844	-464.554	0.410507	0	-0.1835825	0	0.047566963
cov1_2_3_4_5_6_7_9	44.7935	-464.559	0.4103696	0	-0.17040967	0	0.046028208
cov1_4_6_7_8_9_10_11	44.7954	-464.56	0.4068453	0	-0.12774025	0	0
cov1_2_3_6_7_9_10_11	44.8228	-464.573	0.4143887	0	-0.19680874	0	0.018264325
cov1_2_3_5_6_7_9_11	44.9126	-464.618	0.4122145	0	-0.14973964	0	0.019293569
cov1_3_4_5_6_7_9_11	44.9538	-464.639	0.4119902	0	-0.13997931	0	0
cov1_2_3_4_5_6_9_10	45.0187	-464.671	0.4090123	0	-0.11729171	0	0.042479078
cov1_2_3_4_5_6_10_11	45.0192	-464.671	0.4090667	0	-0.11661413	0	0.042757995
cov1_2_3_4_8_9_10_11	45.03	-464.677	0.4112445	0	-0.11357011	0	0.046745834
cov1_2_3_4_5_8_10_11	45.0301	-464.677	0.4112096	0	-0.11161036	0	0.046208332
cov1_2_3_4_5_8_9_10	45.0685	-464.696	0.411079	0	-0.10169945	0	0.047762876
cov1_2_3_5_6_9_10_11	45.0815	-464.703	0.4103414	0	-0.10145669	0	0.024784076
cov2_3_4_6_7_8_10_11	45.0893	-464.707	0.4084746	0	0	0	0.011548568
cov1_2_4_5_6_8_10_11	45.0907	-464.707	0.4063695	0	-0.12227598	0	0.054794303
cov3_4_6_7_8_9_10_11	45.0973	-464.71	0.4089076	0	0	0	0
cov1_2_4_5_6_8_9_10	45.1022	-464.713	0.4061989	0	-0.11665133	0	0.055542914
cov2_3_4_7_8_9_10_11	45.1041	-464.714	0.4081378	0	0	0	0.008260692
cov1_2_4_5_6_7_10_11	45.1316	-464.728	0.4066267	0	-0.11851359	0	0.053006889

cov1_2_4_5_6_7_9_10	45.1378	-464.731	0.4064795	0	-0.11400124	0	0.053579917
cov1_2_3_5_8_9_10_11	45.1432	-464.733	0.4128033	0	-0.089258355	0	0.022288854
cov1_2_4_5_6_9_10_11	45.1556	-464.74	0.4069676	0	-0.11135977	0	0.049883678
cov1_3_4_5_6_9_10_11	45.1731	-464.748	0.4104954	0	-0.084564756	0	0
cov2_3_4_6_7_8_9_10	45.198	-464.761	0.4084841	0	0	0	0.005345488
cov1_3_4_5_8_9_10_11	45.2112	-464.767	0.4127889	0	-0.076075614	0	0
cov1_2_3_4_6_9_10_11	45.2379	-464.781	0.4086178	0	-0.16814023	0	0.06218132
cov1_2_3_4_5_6_9_11	45.2415	-464.783	0.4087331	0	-0.12004342	0	0.041569531
cov3_4_5_6_8_9_10_11	45.2428	-464.783	0.4097396	0	0	0	0
cov2_3_5_6_8_9_10_11	45.243	-464.783	0.4097974	0	0	0	-0.000534501
cov2_3_4_5_6_8_10_11	45.247	-464.785	0.4098373	0	0	0	5.25E-05
cov1_2_5_6_8_9_10_11	45.2493	-464.786	0.4081511	0	-0.092630015	0	0.026159813
cov2_3_4_5_6_8_9_10	45.2549	-464.789	0.4097957	0	0	0	-0.002050658
cov1_2_6_7_8_9_10_11	45.2695	-464.797	0.4096378	0	-0.17790408	0	0.020277652
cov1_2_3_4_5_7_10_11	45.2701	-464.797	0.4109143	0	-0.10007571	0	0.045495044
cov1_2_5_6_7_9_10_11	45.2705	-464.797	0.408295	0	-0.090634012	0	0.026206464
cov1_2_3_4_5_8_9_11	45.2742	-464.799	0.4108968	0	-0.11457122	0	0.044950532
cov1_2_3_4_7_9_10_11	45.2763	-464.8	0.4109801	0	-0.11120709	0	0.048680748
cov2_3_4_6_7_8_9_11	45.285	-464.804	0.408028	0	0	0	0.009680328
cov1_2_4_6_8_9_10_11	45.2966	-464.81	0.4053844	0	-0.17453666	0	0.076285715
cov1_2_4_5_6_8_9_11	45.2979	-464.811	0.4061277	0	-0.12557309	0	0.053162088
cov1_2_3_4_5_7_9_10	45.2996	-464.812	0.4107951	0	-0.091276057	0	0.046845475
cov1_2_4_5_6_7_9_11	45.3391	-464.831	0.4063832	0	-0.12186272	0	0.05142738
cov1_4_5_6_8_9_10_11	45.3429	-464.833	0.4078933	0	-0.076207775	0	0
cov1_2_4_6_7_9_10_11	45.3572	-464.84	0.4055987	0	-0.17338377	0	0.075572756
cov2_3_5_6_7_9_10_11	45.3596	-464.842	0.4095364	0	0	0	0.001567674
cov3_4_5_6_7_9_10_11	45.36	-464.842	0.409576	0	0	0	0
cov2_3_4_5_6_7_10_11	45.3637	-464.844	0.4095684	0	0	0	0.002244678
cov1_4_5_6_7_9_10_11	45.3678	-464.846	0.4081034	0	-0.073829365	0	0
cov1_2_3_5_7_9_10_11	45.3697	-464.847	0.4124027	0	-0.078958668	0	0.023039953
cov2_3_4_5_6_7_9_10	45.3742	-464.849	0.4095291	0	0	0	-9.03E-05
cov1_3_4_5_7_9_10_11	45.4456	-464.885	0.4124598	0	-0.064980234	0	0
cov2_3_4_5_8_9_10_11	45.4641	-464.894	0.4101765	0	0	0	0.010816672
cov2_3_4_5_6_9_10_11	45.4767	-464.9	0.4087018	0	0	0	0.008089724
cov1_2_3_4_5_7_9_11	45.5081	-464.916	0.4105953	0	-0.10291858	0	0.044240699
cov2_3_4_5_6_8_9_11	45.5225	-464.923	0.409368	0	0	0	-0.001898796
cov2_4_5_6_8_9_10_11	45.567	-464.945	0.4066812	0	0	0	0.016132588
cov2_4_5_6_7_9_10_11	45.5778	-464.951	0.4069278	0	0	0	0.015595715
cov2_3_6_7_8_9_10_11	45.6074	-464.966	0.4129732	0	0	0	-0.035474303
cov2_3_4_5_7_9_10_11	45.6166	-464.97	0.409942	0	0	0	0.013909851
cov2_3_4_5_6_7_9_11	45.6312	-464.977	0.4090968	0	0	0	0.000340037
cov2_4_6_7_8_9_10_11	45.6751	-464.999	0.4039773	0	0	0	0.035625036
cov1_2_3_4_5_9_10_11	45.7426	-465.033	0.4091475	0	-0.045255842	0	0.041952478
cov1_2_4_5_8_9_10_11	45.8342	-465.079	0.4063534	0	-0.048634668	0	0.053818582
cov1_2_4_5_7_9_10_11	45.875	-465.099	0.4066276	0	-0.044554785	0	0.051902502
cov2_3_4_6_8_9_10_11	46.2746	-465.299	0.4092693	0	0	0	0.006992481
cov2_3_4_6_7_9_10_11	46.4708	-465.397	0.4089773	0	0	0	0.009429001

cov1_2_3_4_5_6_7_8	46.6758	-465.5	0.4155722	0	-0.2463707	0	0.0444266
cov1_3_4_5_6_7_8_11	46.7966	-465.56	0.4170854	0	-0.22032392	0	0
cov1_2_3_5_6_7_8_11	46.8389	-465.581	0.4179664	0	-0.22386989	0	0.011116927
cov1_2_3_4_5_7_8_11	46.865	-465.594	0.4155786	0	-0.20919511	0	0.042484157
cov1_2_3_4_6_7_8_11	46.9178	-465.621	0.4162599	0	-0.18829274	0	0.02075012
cov1_2_4_5_6_7_8_11	47.1357	-465.73	0.4118475	0	-0.20297858	0	0.048909492
cov1_2_3_4_5_6_8_11	47.5619	-465.943	0.4166077	0	-0.27616472	0	0.044444162
cov1_2_3_4_5_6_7_11	47.7624	-466.043	0.4163537	0	-0.2661884	0	0.043807008
cov2_3_4_5_6_7_8_11	48.5605	-466.442	0.4156152	0	0	0	-0.027559916
cov1_2_3_4_5_6_7_8_10	50.4199	-463.857	0.4100431	0	-0.15706049	0	0.046391077
cov1_2_3_4_6_7_8_10_11	50.4219	-463.858	0.4101753	0	-0.17446478	0	0.049616717
cov1_2_3_4_6_7_8_9_10	50.43	-463.862	0.4100738	0	-0.17052534	0	0.050341817
cov1_2_3_5_6_7_8_10_11	50.5268	-463.911	0.4117391	0	-0.13699907	0	0.021599521
cov1_2_3_5_6_7_8_9_10	50.5361	-463.915	0.411641	0	-0.13243251	0	0.022164524
cov1_3_4_5_6_7_8_10_11	50.5895	-463.942	0.411675	0	-0.12531059	0	0
cov1_3_5_6_7_8_9_10_11	50.5959	-463.945	0.4118999	0	-0.12342655	0	0
cov1_3_4_5_6_7_8_9_10	50.6033	-463.949	0.4115512	0	-0.11907343	0	0
cov1_3_4_6_7_8_9_10_11	50.6622	-463.978	0.4121594	0	-0.15543806	0	0
cov1_2_3_4_6_7_8_9_11	50.6679	-463.981	0.4097954	0	-0.17198789	0	0.046987424
cov1_2_3_4_5_6_7_8_9	50.6756	-463.985	0.4096575	0	-0.15811569	0	0.045186749
cov1_2_3_6_7_8_9_10_11	50.6955	-463.995	0.4135974	0	-0.18493806	0	0.018306351
cov1_2_3_5_6_7_8_9_11	50.7869	-464.041	0.4114247	0	-0.13810449	0	0.019320674
cov1_3_4_5_6_7_8_9_11	50.8305	-464.063	0.4112362	0	-0.1281662	0	0
cov1_2_3_4_5_7_8_10_11	50.9613	-464.128	0.4101433	0	-0.097326529	0	0.044767565
cov1_2_3_4_7_8_9_10_11	50.9691	-464.132	0.4102072	0	-0.10954685	0	0.048274482
cov1_2_3_4_5_7_8_9_10	50.9862	-464.14	0.4100268	0	-0.089209275	0	0.045991898
cov1_2_4_5_6_7_8_10_11	51.0023	-464.148	0.4059437	0	-0.1065816	0	0.052112712
cov1_2_4_5_6_7_8_9_10	51.0078	-464.151	0.405805	0	-0.10221652	0	0.052666335
cov1_2_3_5_7_8_9_10_11	51.0563	-464.175	0.4115811	0	-0.076652754	0	0.022850343
cov2_3_5_6_7_8_9_10_11	51.1259	-464.21	0.4089665	0	0	0	0.003228183
cov3_4_5_6_7_8_9_10_11	51.1276	-464.211	0.4090522	0	0	0	0
cov2_3_4_5_6_7_8_10_11	51.1292	-464.212	0.4089166	0	0	0	0.004706937
cov1_3_4_5_7_8_9_10_11	51.1315	-464.213	0.4116461	0	-0.062725428	0	0
cov1_2_5_6_7_8_9_10_11	51.1322	-464.213	0.4075436	0	-0.079579896	0	0.026200879
cov2_3_4_5_6_7_8_9_10	51.137	-464.216	0.4088709	0	0	0	0.002634316
cov1_2_3_4_5_7_8_9_11	51.2018	-464.248	0.4098132	0	-0.10011076	0	0.043495242
cov1_2_4_5_6_7_8_9_11	51.2105	-464.253	0.4057042	0	-0.10996505	0	0.05053579
cov1_4_5_6_7_8_9_10_11	51.2306	-464.263	0.4073885	0	-0.062647777	0	0
cov1_2_4_6_7_8_9_10_11	51.2309	-464.263	0.4049293	0	-0.16183343	0	0.074820556
cov2_3_4_5_7_8_9_10_11	51.2891	-464.292	0.4091771	0	0	0	0.014063012
cov1_2_3_4_5_6_8_10_11	51.3372	-464.316	0.4111061	0	-0.18299036	0	0.047151099
cov1_2_3_4_6_8_9_10_11	51.3382	-464.316	0.4111053	0	-0.18722482	0	0.048400894
cov1_2_3_4_5_6_8_9_10	51.3504	-464.322	0.4109844	0	-0.17789305	0	0.048027198
cov2_4_5_6_7_8_9_10_11	51.3615	-464.328	0.4061861	0	0	0	0.018563453
cov2_3_4_5_6_7_8_9_11	51.3959	-464.345	0.4084462	0	0	0	0.00281036
cov1_2_3_5_6_8_9_10_11	51.4734	-464.384	0.4128692	0	-0.15701745	0	0.020870411
cov1_3_4_5_6_8_9_10_11	51.5256	-464.41	0.4126831	0	-0.14613208	0	0

cov1_2_4_5_7_8_9_10_11	51.553	-464.424	0.4059008	0	-0.042386152	0	0.051098042
cov1_2_3_4_5_6_7_10_11	51.5588	-464.427	0.4108415	0	-0.17341653	0	0.046520168
cov1_2_3_4_5_6_7_9_10	51.5672	-464.431	0.4107338	0	-0.16920809	0	0.047206377
cov1_2_3_4_6_7_9_10_11	51.5674	-464.431	0.4108656	0	-0.18597872	0	0.05018112
cov1_2_3_4_5_6_8_9_11	51.594	-464.444	0.4107475	0	-0.18507991	0	0.045794326
cov1_2_3_5_6_7_9_10_11	51.6816	-464.488	0.4125002	0	-0.14848579	0	0.021546214
cov1_3_4_5_6_7_9_10_11	51.7419	-464.518	0.4123872	0	-0.13691364	0	0
cov1_2_3_4_5_6_7_9_11	51.8098	-464.552	0.410476	0	-0.17537063	0	0.045164092
cov1_2_3_4_5_6_9_10_11	52.047	-464.671	0.4090006	0	-0.11647587	0	0.042716911
cov1_2_3_4_5_8_9_10_11	52.059	-464.677	0.4112363	0	-0.11183434	0	0.046248465
cov2_3_4_6_7_8_9_10_11	52.1121	-464.703	0.408348	0	0	0	0.011578972
cov1_2_4_5_6_8_9_10_11	52.1172	-464.706	0.4062683	0	-0.12172127	0	0.054560165
cov1_2_4_5_6_7_9_10_11	52.1582	-464.726	0.4065542	0	-0.11810632	0	0.052859682
cov2_3_4_5_6_8_9_10_11	52.2719	-464.783	0.4097359	0	0	0	8.50E-05
cov1_2_3_4_5_7_9_10_11	52.299	-464.797	0.4109348	0	-0.10023891	0	0.04552439
cov2_3_4_5_6_7_9_10_11	52.3885	-464.842	0.4094646	0	0	0	0.002285822
cov1_2_3_4_5_6_7_8_11	53.6694	-465.482	0.4156983	0	-0.25421287	0	0.042990755
cov1_2_3_4_5_6_7_8_10_11	57.441	-463.853	0.4101292	0	-0.16101385	0	0.045706234
cov1_2_3_4_5_6_7_8_9_10	57.4489	-463.857	0.410026	0	-0.15691762	0	0.046362739
cov1_2_3_4_6_7_8_9_10_11	57.4508	-463.858	0.4101555	0	-0.17442022	0	0.049619166
cov1_2_3_5_6_7_8_9_10_11	57.5558	-463.911	0.4117224	0	-0.13687635	0	0.021572594
cov1_3_4_5_6_7_8_9_10_11	57.618	-463.942	0.4116233	0	-0.12508924	0	0
cov1_2_3_4_5_6_7_8_9_11	57.6925	-463.979	0.409759	0	-0.16295258	0	0.044345415
cov1_2_3_4_5_7_8_9_10_11	57.9903	-464.128	0.4101368	0	-0.097451779	0	0.044825327
cov1_2_4_5_6_7_8_9_10_11	58.0288	-464.147	0.4058751	0	-0.10620768	0	0.051965502
cov2_3_4_5_6_7_8_9_10_11	58.1543	-464.21	0.4088182	0	0	0	0.004752373
cov1_2_3_4_5_6_8_9_10_11	58.3662	-464.316	0.41109	0	-0.18285451	0	0.047126342
cov1_2_3_4_5_6_7_9_10_11	58.5877	-464.427	0.4108205	0	-0.173274	0	0.046488079
cov1_2_3_4_5_6_7_8_9_10_11	64.4699	-463.853	0.4101065	0	-0.16085531	0	0.04567329

SE	bsnp_rs7817754	SE	bsnp_rs2047962	SE	bsnp_rs6469668	SE	bsnp_rs6469667	SE	bsnp_rs7832958
0	0	0	0	0	0	0	0	0	0
0	0	0	0	0	0	0	0	0	0
0	0	0	0	0	-0.10743216	0	0	0	0
0	0	0	0	0	0	0	0	0	0
0	0	0	0	0	0	0	0	0	0
0	0	0	0	0	0	0	-0.097285207	0	0
0	0	0	-0.095384573	0	0	0	0	0	0
0	0	0	0	0	0	0	0	0	0
0	0	0	0	0	0	0	0	0	0
0	0	0	0	0	-0.094668927	0	0	0	0
0	0	0	0	0	-0.092669989	0	0	0	0
0	0	0	0	0	0	0	0	0	0
0	0	0	0	0	0	0	0	0	0
0	0	0	-0.081791973	0	0	0	0	0	0
0	0	0	-0.080388594	0	0	0	0	0	0
0	0	0	0	0	0	0	0.065378986	0	0
0	0.033601471	0	0	0	0	0	0	0	0
0	0	0	0	0	0	0	0	0	0
0	0	0	0	0	0	0	0	0	0
0	0	0	-0.019242797	0	0	0	0	0	0
0	0	0	0	0	0	0	0	0	0
0	0	0	0	0	0	0	0	0	0
0	0	0	0	0	-0.019619151	0	0	0	0
0	0	0	0	0	0	0	0	0	0.004951256
0	0	0	0	0	0	0	-0.064495854	0	0
0	0	0	0	0	0	0	0	0	0
0	0	0	0	0	0	0	-0.063140662	0	0
0	0	0	0	0	0	0	0	0	0.021306643
0	0.012092445	0	0	0	0	0	0	0	0
0	0	0	0	0	0	0	0	0	0
0	0	0	0	0	0	0	0	0	0
0	0	0	0	0	0	0	0	0	0
0	0	0	0	0	-0.082305347	0	0	0	0
0	0	0	0	0	-0.082279405	0	-0.032007504	0	0
0	0.046377511	0	0	0	0	0	0	0	0
0	0.047770761	0	0	0	0	0	0	0	0
0	0	0	0	0	-0.094290476	0	0	0	0
0	0	0	0	0	-0.1071673	0	0	0	0.014954426
0	0	0	-0.012713946	0	-0.096461793	0	0	0	0
0	0	0	0	0	-0.10730382	0	0	0	0
0	-0.001805153	0	0	0	-0.10755708	0	0	0	0
0	0	0	-0.055409325	0	0	0	-0.059347286	0	0
0	0	0	0	0	0	0	0	0	0
0	0	0	0	0	0	0	0	0	0
0	0	0	0	0	0	0	0	0	0.003544187

0	0	0	0	0	0	0	0	0	0
0	0	0	0	0	0	0	0	0	0.003374678
0	0	0	-0.071890802	0	0	0	0	0	0
0	0	0	0	0	0	0	-0.058085467	0	0
0	0	0	-0.054245008	0	0	0	0	0	0
0	0.030261737	0	0	0	0	0	-0.10033456	0	0
0	0	0	0	0	0	0	-0.078183321	0	0
0	0	0	0	0	0	0	0	0	0
0	0.029422592	0	0	0	0	0	0	0	0
0	0	0	0	0	0	0	-0.096932815	0	0.011678718
0	0	0	0	0	0	0	-0.097177002	0	0
0	-0.008885514	0	-0.096423857	0	0	0	0	0	0
0	0	0	-0.096034602	0	0	0	0	0	0
0	0	0	-0.095494548	0	0	0	0	0	-0.001529465
0	0	0	0	0	0	0	0	0	0.011653169
0	0	0	0	0	0	0	0	0	0
0	0.040156254	0	0	0	0	0	0	0	0
0	0	0	0	0	0	0	0	0	0.040850321
0	0	0	0	0	0	0	0	0	0
0	0	0	0	0	-0.14078109	0	0.06237101	0	0
0	0	0	0	0	-0.13673623	0	0.060315843	0	0
0	0.030810741	0	0	0	-0.091697398	0	0	0	0
0	0	0	0	0	-0.11288617	0	0	0	0
0	0	0	0	0	-0.10325784	0	0	0	0
0	0.029265825	0	0	0	-0.089721629	0	0	0	0
0	0	0	0	0	-0.094748537	0	0	0	0
0	0	0	0	0	-0.10359498	0	0	0	0
0	0	0	-0.003161205	0	-0.091954689	0	0	0	0
0	0	0	0	0	-0.094540671	0	0	0	0
0	0	0	0	0	-0.094668992	0	0	0	-8.26E-06
0	0	0	0	0	-0.10862797	0	0	0	0
0	0	0	0	0	-0.10121711	0	0	0	0
0	0	0	0	0	-0.09273461	0	0	0	0
0	0	0	0	0	-0.098367783	0	0	0	0
0	0	0	-0.00501431	0	-0.088364051	0	0	0	0
0	0	0	0	0	-0.092664202	0	0	0	0.000611952
0	0.05199801	0	0	0	0	0	0	0	0
0	0	0	0	0	0	0	0.086819528	0	0
0	0.050395034	0	0	0	0	0	0	0	0
0	0	0	0	0	0	0	0.086700747	0	0
0	0	0	-0.044428223	0	0	0	0	0	0
0	0	0	-0.043788624	0	0	0	0	0	0
0	0	0	0	0	0	0	0	0	0
0	0.025855381	0	-0.078004484	0	0	0	0	0	0
0	0	0	0	0	0	0	0	0	0
0	0	0	0	0	0	0	0	0	0
0	0	0	0	0	0	0	0	0	-0.002672803

0	0	0	0	0	0	0	0	0	0
0	0	0	0	0	0	0	0	0	0
0	0	0	-0.073542837	0	0	0	0	0	0
0	0	0	-0.083237232	0	0	0	0	0	0
0	0	0	0	0	0	0	0	0	0
0	0.024636331	0	-0.076712202	0	0	0	0	0	0
0	0	0	-0.095992445	0	0	0	0	0	0
0	0	0	-0.082734494	0	0	0	0	0	-0.014423966
0	0	0	0	0	0	0	0	0	0
0	0	0	-0.081251481	0	0	0	0	0	0
0	0	0	0	0	0	0	0	0	-0.00220199
0	0	0	-0.079262512	0	0	0	-0.004057793	0	0
0	0	0	-0.081779773	0	0	0	0	0	0
0	0	0	-0.07286187	0	0	0	0	0	0
0	0	0	-0.093700427	0	0	0	0	0	0
0	0	0	-0.08128056	0	0	0	0	0	-0.013725655
0	0	0	-0.078549439	0	0	0	-0.00298733	0	0
0	0	0	0	0	0	0	0	0	1.0762661
0	0	0	0	0	0	0	0	0	1.0962312
0	0.051789722	0	0	0	0	0	0	0	0
0	0.062990232	0	0	0	0	0	0	0	0
0	0.050360218	0	0	0	0	0	0	0	0
0	0.060871076	0	0	0	0	0	0	0	0
0	0.050782203	0	0	0	0	0	-0.065998775	0	0
0	0.032297528	0	0	0	0	0	0.063258934	0	0
0	0.06567697	0	0	0	0	0	0	0	0
0	0.049246903	0	0	0	0	0	-0.06459632	0	0
0	0.058816918	0	0	0	0	0	0	0	-0.04894943
0	0	0	-0.020500419	0	0	0	0.066445522	0	0
0	0	0	0	0	0	0	0.063147391	0	0
0	0	0	0	0	-0.01896968	0	0.06509406	0	0
0	0	0	0	0	0	0	0.0645506	0	0
0	0	0	0	0	0	0	0	0	0
0	0	0	0	0	0	0	0.065518495	0	0
0	0	0	0	0	0	0	0.065224673	0	0.003219436
0	0	0	0	0	0	0	-0.031938296	0	0
0	0.033149132	0	0	0	0	0	0	0	0
0	0.031521195	0	0	0	0	0	0	0	0
0	0	0	0	0	0	0	0	0	0
0	0.031332999	0	-0.006401147	0	0	0	0	0	0
0	0.034114186	0	0	0	0.001778805	0	0	0	0
0	0	0	0	0	0	0	-0.030156768	0	0
0	0	0	-0.035582589	0	0	0	0	0	0
0	0	0	0	0	0	0	-0.04301749	0	0
0	0	0	0	0	0	0	0	0	0
0	0	0	0	0	0	0	0	0	0
0	0	0	0	0	0	0	0	0	0.000978735

0	0	0	-0.018924714	0	0	0	0	0	0
0	0	0	0	0	0	0	0	0	0
0	0	0	0	0	0	0	0	0	0.001267433
0	0	0	0	0	0	0	-0.04254608	0	0
0	0	0	0	0	-0.019758598	0	0	0	0
0	0	0	0	0	0	0	0	0	0
0	0	0	0	0	-0.020352054	0	0	0	0
0	0	0	-0.01972012	0	0	0	0	0	0
0	0	0	0	0	0	0	0	0	0
0	0	0	-0.017359134	0	-0.003919609	0	0	0	0
0	0	0	-0.019035102	0	0	0	0	0	0.002322032
0	0	0	0	0	0	0	0	0	-0.001516412
0	0	0	0	0	-0.020083152	0	0	0	0.006182789
0	0	0	0	0	0	0	0	0	0.004663049
0	0	0	0	0	0	0	0	0	0
0	0	0	0	0	-0.019506944	0	0	0	0
0	0	0	0	0	-0.10677501	0	0	0	1.0714152
0	0	0	0	0	0	0	-0.063723574	0	0
0	0	0	0	0	0	0	-0.064568107	0	0
0	0	0	0	0	0	0	-0.064479775	0	0.001780887
0	0	0	0	0	0	0	-0.063205452	0	0
0	0	0	0	0	0	0	-0.063122849	0	0.002135891
0	0	0	0	0	0	0	0	0	0.020033516
0	0.002642684	0	0	0	0	0	0	0	0.01893238
0	0.009565816	0	0	0	0	0	0	0	0
0	0	0	0	0	0	0	0	0	0
0	0	0	0	0	0	0	0	0	0
0	0	0	0	0	0	0	0	0	0.019892861
0	0	0	0	0	0	0	0	0	0
0	0	0	0	0	0	0	0	0	1.1283824
0	0	0	0	0	0	0	0	0	1.1280798
0	0.10227443	0	0	0	0	0	0	0	0
0	0.098424918	0	0	0	0	0	0	0	0
0	0.094164289	0	0	0	0	0	0	0	-0.084920883
0	0.090400999	0	0	0	0	0	0	0	-0.081065912
0	0	0	0	0	0	0	-0.099321072	0	1.1794742
0	0	0	0	0	-0.073576213	0	-0.018886713	0	0
0	0	0	0	0	-0.07851553	0	0	0	0
0	0	0	0	0	-0.082457832	0	0	0	0.013032605
0	0.008240987	0	0	0	-0.079665635	0	0	0	0
0	0	0	0.010776261	0	-0.08863101	0	0	0	0
0	0	0	0	0	-0.082374038	0	0	0	0
0	0	0	0	0	-0.082489627	0	-0.031437696	0	0.013288478
0	0	0	0	0	-0.079632497	0	-0.026551458	0	0
0	0	0	-0.012715754	0	-0.071309958	0	-0.032002296	0	0
0	0.047543255	0	0	0	0	0	0	0	0
0	0.008301957	0	0	0	-0.07900285	0	-0.0354396	0	0

0	0	0	0	0	-0.082394064	0	-0.031737831	0	0
0	0	0	0	0	-0.091868647	0	0	0	0.021076484
0	0	0	-0.014155863	0	-0.081761452	0	0	0	0
0	0	0	0	0	-0.092795611	0	0	0	0
0	0.007309922	0	0	0	-0.091878459	0	0	0	0
0	-0.016854519	0	0	0	-0.10806612	0	0	0	0.030025619
0	0	0	-0.009870354	0	-0.098683299	0	0	0	0.013094029
0	0	0	-0.011580484	0	-0.097348402	0	0	0	0
0	-0.003246079	0	-0.013431705	0	-0.096067131	0	0	0	0
0	-0.009906107	0	0	0	-0.10783678	0	0	0	0
0	0	0	0	0	0	0	0	0	1.0889896
0	0	0	-0.095110287	0	0	0	0	0	1.0569829
0	0	0	-0.053147116	0	0	0	-0.046941296	0	0
0	0.012474936	0	-0.051136675	0	0	0	-0.063528741	0	0
0	0	0	-0.04701098	0	0	0	-0.052056056	0	0
0	0	0	-0.055950888	0	0	0	-0.059165978	0	0
0	0	0	-0.05520006	0	0	0	-0.059424291	0	0.002192387
0	0.032072936	0	0	0	0	0	-0.060079241	0	0
0	0	0	0	0	0	0	0	0	0
0	0	0	0	0	0	0	0	0	0.003244339
0	0	0	-0.053214047	0	0	0	0	0	0
0	0.011862484	0	-0.068269615	0	0	0	0	0	0
0	0	0	-0.069515034	0	0	0	0	0	0.014950554
0	0	0	0	0	0	0	-0.050051738	0	0
0	0	0	-0.070826346	0	0	0	0	0	0
0	0.036567105	0	0	0	0	0	-0.076341705	0	0
0	0.037538501	0	0	0	0	0	0	0	0
0	0	0	0	0	0	0	-0.057921328	0	0.010488018
0	0	0	0	0	0	0	-0.058034365	0	0
0	0.053242342	0	0	0	0	0	-0.10408895	0	0
0	0.011952484	0	-0.047626177	0	0	0	0	0	0
0	0	0	-0.054848418	0	0	0	0	0	0
0	0	0	-0.05381958	0	0	0	0	0	0.003175521
0	0.045578945	0	0	0	0	0	-0.10277565	0	-0.029816263
0	0	0	0	0	0	0	0	0	1.1279097
0	0	0	0	0	0	0	-0.074676003	0	0.020821103
0	0	0	0	0	0	0	0	0	0.022844887
0	0.050989275	0	0	0	0	0	0	0	0
0	0	0	0	0	0	0	-0.076106964	0	0
0	0	0	0	0	0	0	0	0	0
0	0.044006001	0	0	0	0	0	0	0	-0.028413634
0	-0.014483717	0	-0.096266674	0	0	0	0	0	0.011287238
0	-0.00774068	0	-0.096454052	0	0	0	0	0	0
0	0	0	0	0	-0.094086975	0	0	0	1.1021514
0	0	0	0	0	-0.092046882	0	0	0	1.1022612
0	0.034917599	0	0	0	0	0	0	0	0.010387321
0	0.041901946	0	0	0	0	0	0	0	0

0	0.11819315	0	0	0	0	0	0	0	0
0	0	0	0	0	-0.10932591	0	0.09433531	0	0
0	0.072293062	0	0	0	-0.088452248	0	0	0	0
0	0.020561395	0	0	0	-0.13349458	0	0.055198414	0	0
0	0.1133353	0	0	0	0	0	0	0	0
0	0	0	0	0	-0.10398521	0	0.09384561	0	0
0	0	0	0	0	-0.14468834	0	0.058811131	0	0
0	0	0	0	0	-0.14082606	0	0.062328092	0	0
0	0	0	0	0	-0.14070526	0	0.062909501	0	0
0	0.11037051	0	0	0	0	0	0	0	-0.10649965
0	0	0	0	0	-0.14007161	0	0.063607255	0	0
0	0	0	-0.00101871	0	-0.13988094	0	0.062336567	0	0
0	0	0	0	0	-0.14078241	0	0.062371151	0	-0.000108958
0	0.063893009	0	0	0	-0.089140818	0	0	0	-0.059691885
0	0.067944859	0	0	0	-0.086448298	0	0	0	0
0	0.019240318	0	0	0	-0.12982356	0	0.053508065	0	0
0	0	0	0	0	0	0	0	0	1.1012913
0	0.10559421	0	0	0	0	0	0	0	-0.10133174
0	0	0	0	0	-0.13676407	0	0.060270786	0	0
0	0	0	0	0	-0.13940625	0	0.057799788	0	0
0	0.051547448	0	0	0	0	0	0.086011309	0	0
0	0	0	-0.003308473	0	-0.13382774	0	0.06022356	0	0
0	0	0	0	0	-0.13625616	0	0.061137451	0	0
0	0	0	0	0	-0.13673084	0	0.060315764	0	0.000567816
0	0.027216295	0	0	0	-0.10514351	0	0	0	0
0	0.059600063	0	0	0	-0.087185249	0	0	0	-0.054969371
0	0.032975143	0	0	0	-0.082647616	0	0	0	0
0	0.031356194	0	0.004378579	0	-0.095404826	0	0	0	0
0	0.029698681	0	0	0	-0.093788249	0	0	0	0
0	0	0	-0.082159164	0	0	0	0	0	1.0906876
0	0.030817698	0	0	0	-0.091554413	0	0	0	0
0	0	0	0	0	0	0	0	0	1.1012028
0	0	0	-0.024825865	0	-0.099013904	0	0	0	0
0	0.049901406	0	0	0	0	0	0.085831204	0	0
0	0	0	-0.080681376	0	0	0	0	0	1.0912149
0	0	0	0	0	-0.11611994	0	0	0	0
0	0	0	-0.048407836	0	0	0	0.092288435	0	0
0	0	0	0	0	-0.11291427	0	0	0	0
0	0	0	0	0	-0.10603303	0	0	0	0
0	0	0	0	0	-0.11274847	0	0	0	0
0	0	0	0	0	-0.11288505	0	0	0	0.000286557
0	0.026161864	0	0	0	-0.10105298	0	0	0	0
0	0	0	0	0	-0.10480524	0	0	0	0
0	0.032175972	0	0	0	-0.077755492	0	0	0	0
0	0	0	0	0	-0.10379968	0	0	0	-0.004365337
0	0	0	0	0	-0.10313666	0	0	0	0
0	0	0	-0.001905448	0	-0.10157397	0	0	0	0

0	0	0	0	0	-0.1032376	0	0	0	0
0	0.02792697	0	0	0	-0.092229146	0	0	0	0
0	0.029505087	0	0.001958252	0	-0.091379299	0	0	0	0
0	0	0	0	0	-0.10323554	0	0	0	0
0	0	0	-0.047812248	0	0	0	0.092172542	0	0
0	0	0	-0.005015707	0	-0.090451172	0	0	0	0
0	0	0	0	0	-0.1036625	0	0	0	0
0	0	0	-0.002344442	0	-0.10133018	0	0	0	0
0	0	0	0	0	-0.094618465	0	0	0	0
0	0	0	0	0	-0.10360609	0	0	0	0.000392029
0	0	0	-0.003233745	0	-0.091755913	0	0	0	0
0	0	0	-0.003291879	0	-0.091848907	0	0	0	-0.000616527
0	0	0	0	0	-0.094540821	0	0	0	-1.34E-05
0	0	0	-0.076592336	0	0	0	0	0	0
0	0	0	-0.02515643	0	-0.094511959	0	0	0	0
0	0	0	0	0	-0.1121743	0	0	0	0
0	0	0	0	0	-0.10038233	0	0	0	0
0	0	0	0	0	-0.1086371	0	0	0	0
0	0	0	0	0	-0.10862637	0	0	0	0.00091328
0	0	0	0	0	-0.10266296	0	0	0	0
0	0	0	0	0	-0.10167165	0	0	0	-0.003732057
0	0	0	-0.003817425	0	-0.097829139	0	0	0	0
0	0	0	0	0	-0.098066596	0	0	0	0
0	0.043022043	0	-0.028142085	0	0	0	0	0	0
0	0	0	-0.074496423	0	0	0	0	0	0
0	0	0	-0.006814982	0	-0.086893677	0	0	0	0
0	0	0	0	0	-0.098017221	0	0	0	0
0	0	0	-0.004551743	0	-0.094010012	0	0	0	0
0	0	0	0	0	-0.098414143	0	0	0	0.000879948
0	0	0	-0.005084861	0	-0.08830662	0	0	0	-0.000331832
0	0.051538897	0	0	0	0	0	0	0	0
0	0.041442051	0	-0.027968331	0	0	0	0	0	0
0	0.051563272	0	0	0	0	0	0	0	0
0	0.052007091	0	0	0	0	0	0	0	0
0	0.076535167	0	-0.076555916	0	0	0	0	0	0
0	0	0	0	0	0	0	0.085896573	0	0
0	0	0	0	0	0	0	0.087457632	0	0
0	0	0	0	0	0	0	0.085307386	0	0
0	0	0	0	0	0	0	0.087167505	0	-0.005627498
0	0	0	0	0	0	0	0.086917995	0	0
0	0.049929026	0	0	0	0	0	0	0	0
0	0	0	0	0	0	0	0.08580191	0	0
0	0.049697176	0	0	0	0	0	0	0	0
0	0	0	0	0	0	0	0.087312434	0	0
0	0	0	0	0	0	0	0.08506235	0	0
0	0.072646507	0	-0.075195404	0	0	0	0	0	0
0	0	0	0	0	0	0	0.08701786	0	-0.005142261

0	0.068402966	0	-0.076826797	0	0	0	0	0	-0.07727052
0	0	0	-0.046422141	0	0	0	0	0	0
0	0	0	-0.044025359	0	0	0	0	0	0
0	0	0	-0.045544321	0	0	0	0	0	-0.010014706
0	0	0	-0.044501687	0	0	0	0	0	0
0	0	0	-0.045704607	0	0	0	0	0	0
0	0	0	-0.043387034	0	0	0	0	0	0
0	0.06458695	0	-0.075487836	0	0	0	0	0	-0.072923233
0	0	0	-0.044830674	0	0	0	0	0	-0.009393891
0	0.036466078	0	-0.062493598	0	0	0	0	0	0
0	0.03441832	0	-0.062392955	0	0	0	0	0	0
0	0	0	0	0	0	0	0	0	0
0	0	0	-0.094589455	0	0	0	0	0	0
0	0	0	0	0	0	0	0	0	0
0	0	0	0	0	0	0	0	0	0
0	0.028554616	0	-0.07029993	0	0	0	-0.011732036	0	0
0	0	0	0	0	0	0	0	0	-0.003123344
0	0	0	0	0	0	0	0	0	0
0	0.025907105	0	-0.077446266	0	0	0	0	0	0
0	0.11363482	0	0	0	0	0	0	0	0
0	0.023920546	0	-0.083455964	0	0	0	0	0	0
0	0	0	0	0	0	0	0	0	-0.008307849
0	0	0	0	0	0	0	0	0	0
0	0	0	-0.10018533	0	0	0	0	0	0
0	0	0	0	0	0	0	0	0	0
0	0	0	0	0	0	0	0	0	0
0	0	0	-0.076295268	0	0	0	0	0	0
0	0	0	0	0	0	0	0	0	-0.00267232
0	0	0	0	0	0	0	0	0	0
0	0	0	0	0	0	0	0	0	0
0	0	0	-0.092281781	0	0	0	0	0	0
0	0	0	-0.076985664	0	0	0	0.008519696	0	0
0	0	0	-0.074940233	0	0	0	0	0	-0.008714677
0	0	0	-0.098864523	0	0	0	0	0	-0.017123104
0	0	0	-0.073401766	0	0	0	0	0	0
0	0	0	-0.082700535	0	0	0	0	0	0
0	0	0	0	0	0	0	0	0	-0.002608969
0	0.026995323	0	-0.070004556	0	0	0	-0.010324029	0	0
0	0	0	-0.093061712	0	0	0	-0.012914195	0	0
0	0.057355247	0	0	0	0	0	0	0	1.0137717
0	0	0	-0.081695349	0	0	0	-0.002448206	0	0
0	0	0	0	0	0	0	0	0	-0.008102902
0	0	0	-0.095874859	0	0	0	0	0	0
0	0.022871673	0	-0.08163991	0	0	0	0	0	0
0	0	0	-0.082195512	0	0	0	0	0	-0.014363656
0	0.10944418	0	0	0	0	0	0	0	0
0	0	0	-0.097685184	0	0	0	0	0	0

0	0	0	-0.080865643	0	0	0	-0.002964905	0	-0.014096004
0	0	0	-0.079378667	0	0	0	-0.003042499	0	0
0	0	0	-0.075568141	0	0	0	0	0	0
0	0	0	-0.09638419	0	0	0	0	0	-0.016233653
0	0	0	-0.076371904	0	0	0	0.00872596	0	0
0	0	0	-0.074218325	0	0	0	0	0	-0.00849267
0	0	0	-0.080902962	0	0	0	-0.001409066	0	0
0	0	0	-0.091166168	0	0	0	-0.011105511	0	0
0	0.11091384	0	0	0	0	0	-0.068976492	0	0
0	0	0	-0.080077095	0	0	0	-0.001932599	0	-0.013513302
0	0.1056617	0	0	0	0	0	0	0	-0.098255076
0	0	0	0	0	0	0	0	0	1.1156405
0	0	0	0	0	0	0	-0.066767025	0	1.1752618
0	0	0	0	0	0	0	0	0	1.1158316
0	0	0	0	0	0	0	0.052483232	0	1.0242052
0	0.10155366	0	0	0	0	0	0	0	-0.093927459
0	0.11249161	0	0	0	0	0	0	0	0
0	0	0	0	0	0	0	-0.065426048	0	1.1741632
0	0.10643705	0	0	0	0	0	-0.067359777	0	0
0	0.10241932	0	0	0	0	0	-0.068371964	0	-0.094323669
0	0	0	-0.018302873	0	0	0	0	0	1.0709725
0	0	0	0	0	0	0	0	0	1.0671347
0	0	0	0	0	0	0	0	0	1.075056
0	0	0	0	0	-0.0187028	0	0	0	1.0746754
0	0.05935004	0	0	0	0	0	0	0	0
0	0.10762552	0	0	0	0	0	0	0	0
0	0.10437651	0	0	0	0	0	0	0	-0.076479434
0	0	0	0	0	0	0	0	0	1.1442731
0	0	0	0	0	0	0	0	0	1.1437014
0	0.098046309	0	0	0	0	0	-0.066778033	0	-0.089684756
0	0.057342213	0	0	0	0	0	0	0	0
0	0.001281436	0	0	0	0	0	0	0	1.0948644
0	0.059146691	0	0	0	0	0	-0.035418103	0	0
0	0.099598443	0	0	0	0	0	0	0	-0.072071734
0	0.051878855	0	0	0	0	0	-0.032155518	0	0
0	0.050413873	0	0	0	0	0	-0.030295476	0	0
0	0.06441837	0	0	0	0	0	0.063357821	0	0
0	0.057155274	0	0	0	0	0	-0.035344646	0	0
0	0.051634388	0	0	0	0	0	0	0	0
0	0.057975706	0	0	0	0	0	0.063958405	0	-0.049877604
0	0.062757689	0	0	0	0	0	0	0	0
0	0.093528188	0	0	0	0	0	0	0	1.0347072
0	0.093120138	0	0	0	0.045694297	0	0	0	0
0	0.089738794	0	0	0	0	0	0	0	1.0386262
0	0.065298663	0	0	0	0	0	0	0	0
0	0.050682004	0	0	0	0	0	-0.065339732	0	0
0	0.031908872	0	0	0	0	0	0.062537612	0	0

0	0.06509588	0	0	0	0	0	0	0	0
0	0.029291297	0	-0.008441357	0	0	0	0.063894816	0	0
0	0.030881048	0	0	0	0	0	0.062181059	0	0
0	0.032745436	0	0	0	0.001557263	0	0.06325335	0	0
0	0	0	-0.036218699	0	0	0	0.065257555	0	0
0	0.067067164	0	0.002603681	0	0	0	0	0	0
0	0.080929933	0	0	0	0.036527294	0	0	0	-0.0714583
0	0.058540074	0	0	0	0	0	0	0	-0.054943539
0	0.058233161	0	0	0	0	0	0	0	-0.048670109
0	0	0	-0.020206313	0	0	0	0.064265604	0	0
0	0.059124774	0	0.000573898	0	0	0	0	0	-0.04915231
0	0	0	-0.021115325	0	0	0	0.066719108	0	0
0	0	0	-0.020144723	0	-0.000737898	0	0.066415018	0	0
0	0	0	-0.020467804	0	0	0	0.066426657	0	0.00035919
0	0	0	0	0	0	0	-0.02120304	0	0
0	0	0	0	0	-0.019104993	0	0.062851265	0	0
0	0	0	0	0	-0.01963804	0	0.06422806	0	0
0	0	0	0	0	0	0	0.062514288	0	0
0	0	0	0	0	0	0	0.063116576	0	0
0	0	0	0	0	0	0	0.063170561	0	-0.00196768
0	0	0	0	0	-0.019301799	0	0.064885186	0	0.004413017
0	0	0	0	0	-0.018756365	0	0.06520113	0	0
0	0.025346074	0	-0.020952299	0	0	0	0	0	0
0	0	0	0	0	0	0	0	0	0.010116345
0	0	0	0	0	0	0	0.064695312	0	0
0	0	0	0	0	0	0	0	0	0
0	0	0	0	0	0	0	0.064411099	0	0.002982604
0	0	0	0	0	0	0	0	0	0
0	0	0	0	0	0	0	-0.020119613	0	0
0	0	0	0	0	0	0	-0.031078251	0	0
0	0.031258217	0	0	0	0	0	0	0	0
0	0	0	0	0	0	0	-0.03196498	0	0
0	0	0	0	0	0	0	-0.03194005	0	0.001028195
0	0.033337603	0	0	0	0.000655714	0	0	0	0
0	0.028882806	0	-0.00720012	0	0	0	0	0	0
0	0	0	0	0	0	0	0	0	0.009922964
0	0.031548002	0	0	0	8.90E-05	0	0	0	0
0	0	0	0	0	0	0	0	0	0
0	0.032848342	0	-0.011065927	0	0.01099947	0	0	0	0
0	0	0	-0.034466165	0	0	0	0	0	0
0	0	0	0	0	0	0	-0.030174499	0	0
0	0	0	-0.051006732	0	0.024568879	0	0	0	0
0	0	0	0	0	0	0	-0.030159873	0	0.001350907
0	0	0	0	0	-0.076731219	0	-0.038254672	0	1.1104548
0	0	0	0	0	-0.081462053	0	0	0	1.0747
0	0	0	-0.036651476	0	0	0	0	0	0
0	0	0	-0.035668152	0	0	0	0	0	-0.000627095

0	0	0	0	0	0	0	-0.041360186	0	0.011205004
0	0	0	0	0	0	0	-0.042533621	0	0
0	0	0	0	0	0	0	-0.042651269	0	0
0	0	0	0	0	0	0	0	0	0
0	0	0	0	0	0	0	0	0	0.000939202
0	0	0	-0.019991274	0	0	0	0	0	0
0	0	0	0	0	-0.090731978	0	0	0	1.0839786
0	0	0	0	0	-0.020416311	0	0	0	0
0	0	0	-0.019291368	0	0	0	0	0	-0.004282586
0	0	0	-0.016679219	0	-0.004670341	0	0	0	0
0	0	0	0	0	0	0	-0.040888717	0	0.011272431
0	0	0	0	0	-0.019125551	0	0	0	0
0	0	0	0	0	0	0	-0.042170988	0	0
0	0	0	0	0	0	0	0	0	0
0	0	0	0	0	-0.019741817	0	0	0	-0.000228836
0	0	0	0	0	0	0	0	0	-0.001424576
0	0	0	0	0	-0.020793109	0	0	0	0.005927369
0	0	0	0	0	-0.020221931	0	0	0	0
0	0	0	-0.018479017	0	-0.002522321	0	0	0	0
0	0	0	-0.016706183	0	-0.004730442	0	0	0	0.002933583
0	-0.018142309	0	0	0	-0.10774172	0	0	0	1.0905358
0	0	0	-0.009699954	0	-0.098437667	0	0	0	1.0693481
0	0	0	0	0	0	0	-0.063794144	0	0
0	0	0	0	0	0	0	-0.063708241	0	0.001754411
0	0	0	-0.051545438	0	0	0	-0.064210937	0	1.1287694
0	0	0	0	0	0	0	0	0	0.01973936
0	0	0	0	0	0	0	0	0	0
0	0	0	0	0	0	0	0	0	1.1286599
0	0.10195412	0	0	0	0	0	0	0	0
0	0	0	0	0	0	0	-0.063757367	0	1.1462161
0	0.093837368	0	0	0	0	0	0	0	-0.084739752
0	0	0	-0.068639042	0	0	0	0	0	1.0848114
0	0.045072042	0	0	0	0	0	-0.10509448	0	1.1362383
0	0	0	0	0	0	0	-0.079245156	0	1.1727319
0	0	0	-0.052568135	0	0	0	0	0	1.0705232
0	0	0	0	0	0	0	0	0	1.1050442
0	0.012386174	0	0	0	-0.067840927	0	-0.022716655	0	0
0	0	0	0	0	-0.073889882	0	-0.018523406	0	0.012515876
0	0	0	0	0	-0.077584472	0	0	0	0.016949458
0	0.012934774	0	0	0	-0.072825666	0	0	0	0
0	0	0	0	0	-0.072446881	0	-0.016289168	0	0
0	0	0	0	0	-0.073716411	0	-0.018711468	0	0
0	0	0	0.004523416	0	-0.076743043	0	-0.017778686	0	0
0	0	0	0.01465579	0	-0.091090695	0	0	0	0.015487224
0	0.012320457	0	0.017672027	0	-0.088722661	0	0	0	0
0	0	0	0	0	-0.078039244	0	0	0	0
0	0	0	0.006971689	0	-0.083052757	0	0	0	0

0	0.001830656	0	0	0	-0.081851815	0	0	0	0.011356101
0	0.042602471	0	0	0	0	0	0	0	1.0431362
0	0	0	0.012659176	0	-0.089831161	0	0	0	0
0	0.010610499	0	0	0	-0.078853025	0	0	0	0
0	0	0	0	0	-0.079037831	0	-0.024006808	0	0.017397638
0	0.012373163	0	0	0	-0.073807885	0	-0.02970575	0	0
0	0	0	-0.013495461	0	-0.067849472	0	-0.02625803	0	0
0	0	0	-0.010249278	0	-0.07361638	0	-0.031518806	0	0.011352652
0	0	0	0	0	-0.079364982	0	-0.025153891	0	0
0	0.006940241	0	-0.011185359	0	-0.069894	0	-0.034872536	0	0
0	0.001215833	0	0	0	-0.081992917	0	-0.03198786	0	0.012172132
0	0	0	-0.012012677	0	-0.071982868	0	-0.031846021	0	0
0	0.010922285	0	0	0	-0.077880294	0	-0.03674011	0	0
0	0	0	-0.010178631	0	-0.083077455	0	0	0	0.019174167
0	-0.006612668	0	0	0	-0.09342619	0	0	0	0.026506831
0	0	0	-0.011891509	0	-0.082520511	0	0	0	0
0	0.005860418	0	-0.013023085	0	-0.080829642	0	0	0	0
0	0.000733179	0	0	0	-0.092621686	0	0	0	0
0	-0.017094482	0	-0.010340584	0	-0.099190936	0	0	0	0.028291308
0	-0.015775796	0	-0.095950916	0	0	0	0	0	1.0734633
0	-0.010196816	0	-0.011877221	0	-0.097642398	0	0	0	0
0	0.018663388	0	-0.046187464	0	0	0	-0.050093935	0	0
0	0.057713173	0	0	0	0	0	-0.062703956	0	0
0	0.029598694	0	-0.048163439	0	0	0	-0.068298727	0	0
0	0.0184668	0	-0.036437609	0	0	0	-0.054563838	0	0
0	0	0	-0.047693636	0	0	0	-0.043200703	0	0
0	0	0	-0.052057243	0	0	0	-0.04604918	0	0.009101869
0	0	0	-0.053092546	0	0	0	-0.046892901	0	0
0	0.02137262	0	-0.049646571	0	0	0	-0.065937431	0	-0.016309655
0	0.03622531	0	0	0	0	0	-0.048463757	0	0
0	0	0	-0.047652146	0	0	0	-0.05207394	0	0
0	0	0	-0.046575504	0	0	0	-0.052062693	0	0.003269064
0	0.050310389	0	0	0	0	0	-0.061764341	0	-0.035392104
0	0.02170526	0	-0.041054338	0	0	0	0	0	0
0	0	0	-0.051261615	0	0	0	0	0	0.014018643
0	0	0	-0.052419226	0	0	0	0	0	0
0	0.054950568	0	0	0	0	0	-0.081789768	0	0
0	0.054327846	0	0	0	0	0	0	0	0
0	0	0	0	0	0	0	-0.048061045	0	0.016469994
0	0.007327981	0	-0.068214708	0	0	0	0	0	0.009042585
0	0.014189647	0	-0.068287063	0	0	0	0	0	0
0	0	0	0	0	0	0	-0.049052332	0	0
0	0.047429678	0	0	0	0	0	-0.079471912	0	-0.021832942
0	0.047326758	0	0	0	0	0	0	0	-0.019588934
0	0.028374476	0	-0.042281115	0	0	0	0	0	0
0	0.019800841	0	-0.045086269	0	0	0	0	0	-0.013476425
0	0.033622599	0	0	0	0	0	0	0	1.0934264

0	0.10962445	0	0	0	0	0	0	0	0.99019951
0	0.063444945	0	0	0	-0.088597861	0	0	0	1.0403393
0	0	0	0	0	-0.13457515	0	0.054727388	0	1.0523229
0	0.10482282	0	0	0	0	0	0	0	0.99476488
0	0.059146302	0	0	0	-0.086610019	0	0	0	1.0446606
0	0	0	0	0	-0.13055193	0	0.052666967	0	1.054484
0	0.11998075	0	0	0	0	0	0.091136326	0	0
0	0	0	0	0	-0.11224274	0	0	0	1.1020639
0	0	0	0	0	-0.10276635	0	0	0	1.0960304
0	0	0	0	0	-0.10346196	0	0	0	1.1029402
0	0	0	-0.003018467	0	-0.091501197	0	0	0	1.1014897
0	0	0	0	0	-0.093930172	0	0	0	1.1022453
0	0.11497596	0	0	0	0	0	0.090615756	0	0
0	0	0	0	0	-0.10794492	0	0	0	1.1021993
0	0.11290988	0	0	0	0	0	0.091981262	0	-0.11201091
0	0	0	0	0	-0.10061898	0	0	0	1.0962053
0	0	0	0	0	-0.098246868	0	0	0	1.1028118
0	0	0	-0.004834401	0	-0.087904104	0	0	0	1.1012091
0	0.10796693	0	0	0	0	0	0.091409276	0	-0.1066522
0	0.030895163	0	0	0	-0.089586852	0	0.092496209	0	0
0	0.055805574	0	0	0	-0.12135753	0	0.042568144	0	0
0	0.10074246	0	0	0	-0.03462254	0	0	0	0
0	0.1177035	0	0	0	0	0	0	0	0
0	0	0	0	0	-0.11153095	0	0.093306284	0	0
0	0.1176627	0	0	0	0	0	0	0	0
0	0.11088835	0	-0.015425196	0	0	0	0	0	0
0	0.067948948	0	-0.076290715	0	0	0	0	0	1.0255309
0	0.11826198	0	0	0	0	0	0	0	0
0	0	0	0	0	0	0	0.074420601	0	1.0292022
0	0	0	-0.04528974	0	0	0	0	0	1.091615
0	0	0	0	0	-0.10887928	0	0.094792484	0	0
0	0.046137284	0	0	0	-0.12494216	0	0.046345802	0	-0.04318123
0	0	0	0	0	-0.10903904	0	0.093105335	0	0
0	0.030039306	0	0	0	-0.084622448	0	0.091995057	0	0
0	0	0	-0.005160953	0	-0.1043559	0	0.094576775	0	0
0	0	0	0	0	-0.10946365	0	0.094523261	0	0
0	0	0	0	0	-0.10920835	0	0.094490892	0	-0.002643808
0	0	0	-0.08079263	0	0	0	0.092718568	0	0
0	0	0	0	0	0	0	0.074279578	0	1.0293295
0	0.069586548	0	0	0	-0.093334214	0	0	0	0
0	0.064119051	0	-0.074930714	0	0	0	0	0	1.0297409
0	0.072320238	0	0	0	-0.088270545	0	0	0	0
0	0.071639131	0	0	0	-0.089582815	0	0	0	0
0	0.07221283	0	-0.001695442	0	-0.087006457	0	0	0	0
0	0.088250053	0	0	0	-0.04348819	0	0	0	-0.084400317
0	0	0	-0.044583513	0	0	0	0	0	1.0917744
0	0.023267786	0	0	0	-0.12989689	0	0.058850462	0	0

0	0.020484574	0	0	0	-0.13345065	0	0.055729524	0	0
0	0	0	0	0	-0.10577859	0	0.092840904	0	0
0	0.01984052	0	0	0	-0.1360774	0	0.053328662	0	0
0	0.021041798	0	0.003796278	0	-0.13667916	0	0.055159162	0	0
0	0.11283948	0	0	0	0	0	0	0	0
0	0.097646414	0	0	0	-0.030771953	0	0	0	0
0	0.11279715	0	0	0	0	0	0	0	0
0	0.10589924	0	-0.015692875	0	0	0	0	0	0
0	0.051732543	0	0	0	-0.11837313	0	0.041647131	0	0
0	0.10988565	0	0	0	0	0	0	0	-0.10645638
0	0.10225055	0	-0.017061377	0	0	0	0	0	-0.10161166
0	0.10994968	0	0	0	0	0	0	0	-0.11104532
0	0	0	0	0	-0.10371706	0	0.092416175	0	0
0	0	0	0	0	-0.10354174	0	0.094276223	0	0
0	0	0	-0.007473163	0	-0.09685327	0	0.094210931	0	0
0	0	0	0	0	-0.14470611	0	0.058794953	0	0
0	0	0	-0.00948493	0	-0.13825312	0	0.05671552	0	0
0	0	0	0	0	-0.14457569	0	0.059372098	0	0
0	0	0	0	0	-0.10388613	0	0.093969708	0	-0.002124291
0	0	0	0	0	-0.14391345	0	0.060477687	0	0
0	0	0	0	0	-0.14075007	0	0.062868274	0	0
0	0	0	0	0	-0.14468726	0	0.058812449	0	3.05E-06
0	0	0	-0.078685899	0	0	0	0.092483022	0	0
0	0	0	-0.002723689	0	-0.13842227	0	0.062233226	0	0
0	0	0	0	0	-0.140522	0	0.062857086	0	0
0	0	0	0	0	-0.14005029	0	0.064044025	0	0
0	0	0	-0.001254451	0	-0.13959748	0	0.062871988	0	0
0	0	0	0	0	-0.1407068	0	0.062909754	0	-0.000128559
0	0.11043613	0	0	0	0	0	0	0	-0.10656281
0	0	0	-0.00121767	0	-0.13898071	0	0.063592129	0	0
0	0	0	0	0	-0.14002327	0	0.063675642	0	0.000711354
0	0	0	-0.001084264	0	-0.13982668	0	0.062334841	0	-0.000309211
0	0.04108138	0	-0.032742298	0	0	0	0.089868851	0	0
0	0.060044595	0	0	0	-0.096026699	0	0	0	-0.055990806
0	0.063112201	0	0	0	-0.090492667	0	0	0	-0.059575032
0	0.063918211	0	0	0	-0.088968368	0	0	0	-0.059722351
0	0.063871682	0	-0.000465651	0	-0.088743686	0	0	0	-0.059758016
0	0.04209623	0	0	0	-0.12199617	0	0.045444866	0	-0.038679619
0	0.085139764	0	0	0	-0.039759568	0	0	0	-0.080881776
0	0.021558616	0	0	0	-0.12670891	0	0.056587597	0	0
0	0.066189526	0	0	0	-0.089550488	0	0	0	0
0	0.067775601	0	-0.00401938	0	-0.083018768	0	0	0	0
0	0.066981443	0	0	0	-0.088117109	0	0	0	0
0	0	0	0	0	0	0	0	0	1.0999542
0	0.018862637	0	0	0	-0.13113832	0	0.052528781	0	0
0	0.019375815	0	0.001081342	0	-0.13072563	0	0.053490393	0	0
0	0.097337786	0	-0.017337968	0	0	0	0	0	-0.096362282

0	0.10505833	0	0	0	0	0	0	0	-0.1011903
0	0	0	0	0	0	0	0	0	1.093291
0	0.039425396	0	-0.032646589	0	0	0	0.089745869	0	0
0	0.1052067	0	0	0	0	0	0	0	-0.10629147
0	0.051137855	0	0	0	0	0	0.085181581	0	0
0	0.031694883	0	0	0	-0.085704324	0	0	0	0
0	0	0	-0.010347043	0	-0.132396	0	0.055536887	0	0
0	0	0	0	0	0	0	0	0	1.1013456
0	0	0	0	0	-0.13940497	0	0.057773405	0	0
0	0	0	-0.004966419	0	-0.13240206	0	0.060125238	0	0
0	0	0	0	0	-0.13886698	0	0.058911621	0	0
0	0	0	0	0	-0.13666923	0	0.060429509	0	0
0	0	0	0	0	-0.13940108	0	0.057793265	0	0.000655004
0	0.052223458	0	0	0	0	0	0.086552677	0	0
0	0.024682259	0	-0.011636173	0	-0.099362701	0	0	0	0
0	0.051527263	0	0	0	0	0	0.086058886	0	0
0	0	0	-0.003450227	0	-0.13318037	0	0.06111356	0	0
0	0	0	-0.00331867	0	-0.13381924	0	0.060223296	0	-4.79E-05
0	0	0	0	0	-0.13617903	0	0.061249773	0	0.00116233
0	0	0	-0.074104749	0	0	0	0	0	1.0984065
0	0	0	-0.098126348	0	0	0	0	0	1.0873827
0	0.027222491	0	0	0	-0.10499989	0	0	0	0
0	0.027365832	0	0	0	-0.10491028	0	0	0	0
0	0	0	0	0	0	0	0	0	1.099896
0	0	0	0	0	0	0	0	0	1.0928384
0	0.056672176	0	0	0	-0.092317849	0	0	0	-0.052147307
0	0	0	-0.0816017	0	0	0	0	0	1.0911953
0	0.058519401	0	0	0	-0.08905947	0	0	0	-0.054817271
0	0.059489029	0	-0.002733182	0	-0.084852917	0	0	0	-0.055372934
0	0	0	-0.07712826	0	0	0	-0.007975534	0	1.0986119
0	0.03162712	0	0	0	-0.083335116	0	0	0	0
0	0.033435517	0	0.004064495	0	-0.086281278	0	0	0	0
0	0.032945562	0	0	0	-0.082698967	0	0	0	0
0	0.030240518	0	0.004401479	0	-0.097527244	0	0	0	0
0	0.031353802	0	0.004308083	0	-0.095212795	0	0	0	0
0	0.029665175	0	0	0	-0.093704079	0	0	0	0
0	0	0	-0.036895813	0	-0.072272204	0	0	0	0
0	0.049491862	0	0	0	0	0	0.085035484	0	0
0	0.031897928	0	-0.058913742	0	0	0	0	0	0
0	0	0	-0.028295998	0	-0.097112778	0	0	0	0
0	0	0	-0.073375201	0	0	0	0	0	1.0982026
0	0	0	-0.095629135	0	0	0	0	0	1.088152
0	0	0	-0.050820072	0	0	0	0.09361009	0	0
0	0	0	-0.023137822	0	-0.10170266	0	0	0	0
0	0	0	-0.02604146	0	-0.098353724	0	0	0	-0.004397879
0	0	0	-0.025015337	0	-0.098704873	0	0	0	0
0	0.050280263	0	0	0	0	0	0.086137456	0	0

0	0	0	-0.050003084	0	0	0	0.093328876	0	-0.013899737
0	0	0	0	0	-0.10561209	0	0	0	0
0	0	0	-0.076333439	0	0	0	-0.006976037	0	1.0980978
0	0	0	0	0	-0.11698784	0	0	0	0
0	0.10494936	0	0	0	0	0	0	0	1.0099148
0	0	0	-0.048127313	0	0	0	0.091045567	0	0
0	0	0	0	0	-0.10566244	0	0	0	0
0	0	0	0	0	-0.11599436	0	0	0	0
0	0	0	0	0	-0.1163127	0	0	0	-0.002339228
0	0.030991789	0	0	0	-0.080350981	0	0	0	0
0	0	0	-0.048555035	0	0	0	0.092489056	0	0
0	0	0	0	0	-0.112773	0	0	0	0
0	0	0	0	0	-0.10605631	0	0	0	0
0	0	0	0	0	-0.10602982	0	0	0	-9.66E-05
0	0	0	0	0	-0.11274739	0	0	0	0.000280662
0	0.023415288	0	-0.012622578	0	-0.094765326	0	0	0	0
0	0	0	-0.080766403	0	0	0	0	0	0
0	0.025919008	0	0	0	-0.10143978	0	0	0	0
0	0	0	-0.050143128	0	0	0	0.093454962	0	0
0	0	0	-0.004543516	0	-0.10086693	0	0	0	0
0	0	0	0	0	-0.10467894	0	0	0	0
0	0	0	0	0	-0.10250129	0	0	0	0
0	0.030501609	0	0	0	-0.078667202	0	0	0	0
0	0.10202325	0	0	0	0	0	-0.070636215	0	1.0761594
0	0	0	0	0	-0.103682	0	0	0	-0.004419808
0	0	0	-0.002873841	0	-0.10130943	0	0	0	-0.004885998
0	0.032359576	0	0.001609516	0	-0.07917105	0	0	0	0
0	0	0	0	0	-0.10296786	0	0	0	-0.004515035
0	0	0	-0.002017538	0	-0.10134104	0	0	0	0
0	0	0	0	0	-0.10329368	0	0	0	0
0	0	0	-0.001931885	0	-0.10135012	0	0	0	0
0	0	0	-0.049328939	0	0	0	0.093165149	0	-0.013265448
0	0.028166581	0	0.001979034	0	-0.093908544	0	0	0	0
0	0	0	-0.038380105	0	-0.065367881	0	0	0	0
0	0.030668402	0	-0.057379178	0	0	0	0	0	0
0	0	0	-0.07944495	0	0	0	0	0	-0.01693727
0	0	0	-0.04751176	0	0	0	0.090777933	0	0
0	0.10081393	0	0	0	0	0	0	0	1.0140568
0	0	0	-0.004152794	0	-0.099185203	0	0	0	0
0	0	0	0	0	-0.10330358	0	0	0	0
0	0	0	-0.005096239	0	-0.090239098	0	0	0	0
0	0	0	-0.002418643	0	-0.10132682	0	0	0	0
0	0	0	0	0	-0.10368149	0	0	0	0.000397188
0	0	0	-0.002358828	0	-0.10131342	0	0	0	-5.71E-05
0	0	0	-0.075822587	0	0	0	0	0	0
0	0	0	-0.003369308	0	-0.091645813	0	0	0	-0.00063644
0	0	0	-0.028472929	0	-0.092666757	0	0	0	0

0	0	0	-0.076614138	0	0	0	0	0	0
0	0	0	-0.0231583	0	-0.097709989	0	0	0	0
0	0	0	-0.026208304	0	-0.093928755	0	0	0	-0.003802785
0	0	0	0	0	-0.10002416	0	0	0	0
0	0	0	0	0	-0.11298945	0	0	0	0
0	0	0	0	0	-0.1000195	0	0	0	0
0	0	0	0	0	-0.11233264	0	0	0	-0.00194848
0	0	0	0	0	-0.10040399	0	0	0	0.000444647
0	0	0	-0.078450389	0	0	0	0	0	0
0	0	0	-0.006405063	0	-0.097117844	0	0	0	0
0	0.097611781	0	0	0	0	0	-0.069057171	0	1.0790972
0	0	0	0	0	-0.097353846	0	0	0	0
0	0	0	-0.077145019	0	0	0	0	0	-0.01591355
0	0.085470651	0	-0.062056398	0	0	0	0	0	0
0	0	0	-0.004723507	0	-0.097584213	0	0	0	-0.004589775
0	0	0	0	0	-0.097825819	0	0	0	-0.004431697
0	0	0	-0.004078718	0	-0.094163343	0	0	0	0
0	0.04225473	0	-0.028324945	0	0	0	0	0	0
0	0	0	-0.073638623	0	0	0	0	0	0
0	0.043000446	0	-0.028173676	0	0	0	0	0	0
0	0.10373265	0	0	0	0	0	0	0	1.0420217
0	0	0	-0.006346102	0	-0.091882443	0	0	0	0
0	0	0	-0.004549226	0	-0.094012952	0	0	0	1.14E-05
0	0.051556712	0	0	0	0	0	0	0	0
0	0.051363846	0	0	0	0	0	0	0	0
0	0.083271959	0	-0.063753264	0	0	0	-0.019397561	0	0
0	0.040380226	0	-0.028233485	0	0	0	0	0	0
0	0	0	0	0	0	0	0.086542215	0	0
0	0.076574359	0	-0.076002545	0	0	0	0	0	0
0	0.05157986	0	0	0	0	0	0	0	0
0	0	0	0	0	0	0	0.08461878	0	0
0	0.08017693	0	-0.069737372	0	0	0	0	0	0
0	0	0	0	0	0	0	0.086263493	0	-0.006006268
0	0	0	0	0	0	0	0.08596744	0	0
0	0	0	0	0	0	0	0.085496805	0	0
0	0.077391126	0	-0.062182096	0	0	0	0	0	-0.075369595
0	0.080666051	0	-0.06197076	0	0	0	0	0	0
0	0.098926608	0	0	0	0	0	0	0	1.0459222
0	0	0	0	0	0	0	0.085527712	0	-0.009487467
0	0	0	0	0	0	0	0.087557006	0	0
0	0	0	0	0	0	0	0.085406942	0	0
0	0	0	0	0	0	0	0.087266005	0	-0.005628895
0	0.049470053	0	0	0	0	0	0	0	0
0	0	0	0	0	0	0	0.086419717	0	0
0	0	0	0	0	0	0	0.084385071	0	0
0	0.078697462	0	-0.063672792	0	0	0	-0.017639174	0	0
0	0	0	0	0	0	0	0.085252119	0	0

0	0	0	0	0	0	0	0.08613533	0	-0.005478324
0	0.074489853	0	-0.065084532	0	0	0	-0.017797061	0	-0.08089098
0	0	0	0	0	0	0	0.085279351	0	-0.009285651
0	0	0	-0.046556653	0	0	0	0	0	0
0	0.07639331	0	-0.068238514	0	0	0	0	0	0
0	0.068438413	0	-0.076274056	0	0	0	0	0	-0.07724194
0	0	0	-0.0456742	0	0	0	0	0	-0.015796131
0	0.070978579	0	-0.072028502	0	0	0	0	0	-0.078870774
0	0.07267437	0	-0.062094887	0	0	0	0	0	-0.070949076
0	0	0	-0.046500572	0	0	0	0	0	0
0	0	0	-0.044110275	0	0	0	0	0	0
0	0	0	-0.045621557	0	0	0	0	0	-0.010025186
0	0	0	-0.045884788	0	0	0	0	0	0
0	0	0	-0.045001653	0	0	0	0	0	-0.015478606
0	0.070014403	0	-0.065000651	0	0	0	-0.016060029	0	-0.076133151
0	0.067246144	0	-0.070575202	0	0	0	0	0	-0.074583715
0	0.1153901	0	0	0	0	0	0	0	0
0	0.032007932	0	-0.077451056	0	0	0	0	0	0
0	0.03629905	0	-0.062425533	0	0	0	0	0	0
0	0.036116726	0	-0.064555881	0	0	0	0.00484093	0	0
0	0.11397394	0	0	0	0	0	-0.032813303	0	0
0	0.056748016	0	0	0	0	0	0.051351385	0	0.96349381
0	0.030311599	0	-0.075920347	0	0	0	0	0	0
0	0	0	0	0	0	0	0	0	0
0	0	0	-0.09778765	0	0	0	0	0	0
0	0.11082532	0	0	0	0	0	0	0	0
0	0.034034667	0	-0.064555532	0	0	0	0.005082307	0	0
0	0	0	0	0	0	0	0	0	-0.00825546
0	0	0	-0.096284054	0	0	0	0	0	-0.009538696
0	0	0	-0.094529803	0	0	0	0	0	0
0	0	0	0	0	0	0	0	0	0
0	0	0	-0.094662459	0	0	0	0.000326901	0	0
0	0.025433033	0	-0.079059198	0	0	0	-0.015885971	0	0
0	0	0	0	0	0	0	0	0	0
0	0.10738111	0	0	0	0	0	0	0	-0.089879341
0	0.10965426	0	0	0	0	0	-0.030730733	0	0
0	0.028374866	0	-0.070461614	0	0	0	-0.010758904	0	0
0	0	0	0	0	0	0	0	0	0
0	0.11326567	0	0	0	0	0	-0.043891358	0	0
0	0	0	0	0	0	0	0	0	-0.003123791
0	0.11338656	0	0	0	0	0	0	0	0
0	0.023784056	0	-0.083413956	0	0	0	0	0	0
0	0.079838096	0	0	0	0.037138391	0	0	0	0.9924294
0	0	0	0	0	0	0	0	0	-0.00831648
0	0	0	-0.097357152	0	0	0	-0.012126548	0	0
0	0	0	0	0	0	0	0	0	0
0	0	0	-0.10006854	0	0	0	0	0	0

0	0	0	-0.09540071	0	0	0	0	0	0
0	0	0	0	0	0	0	-0.037668041	0	1.1474368
0	0.057096201	0	0	0	0	0	0	0	1.0052408
0	0	0	-0.076178519	0	0	0	0	0	0
0	0	0	0	0	0	0	0	0	-0.008039257
0	0	0	-0.079241444	0	0	0	0.007492957	0	0
0	0.10577992	0	0	0	0	0	-0.032234839	0	-0.098311651
0	0	0	-0.095983842	0	0	0	-0.012295253	0	-0.016578091
0	0	0	0	0	0	0	0	0	1.1277692
0	0	0	-0.098747355	0	0	0	0	0	-0.017135781
0	0.05678615	0	0	0	0	0	0	0	1.0133248
0	0	0	-0.093907536	0	0	0	0	0	-0.00923246
0	0	0	-0.077125081	0	0	0	0.009246816	0	0
0	0	0	-0.074825953	0	0	0	0	0	-0.008902016
0	0	0	-0.078118471	0	0	0	0.008063437	0	-0.008211821
0	0.02420745	0	-0.077746266	0	0	0	-0.013979191	0	0
0	0	0	-0.092522048	0	0	0	0.001155924	0	0
0	0	0	-0.09318218	0	0	0	-0.011897089	0	0
0	0	0	-0.081818293	0	0	0	-0.001417841	0	0
0	0.057822047	0	0.000870247	0	0	0	0	0	1.0135117
0	0	0	0	0	0	0	-0.035890276	0	1.1459475
0	0	0	-0.095266475	0	0	0	-0.010311368	0	0
0	0	0	0	0	0	0	0	0	1.1270921
0	0	0	-0.080988434	0	0	0	-0.00193841	0	-0.014150768
0	0.10289858	0	0	0	0	0	0	0	-0.085830331
0	0	0	0	0	0	0	-0.045847199	0	1.1682574
0	0	0	-0.078597153	0	0	0	0.007732228	0	0
0	0	0	-0.093909643	0	0	0	-0.010495909	0	-0.015756969
0	0.10848681	0	0	0	0	0	-0.043528728	0	0
0	0	0	-0.077473398	0	0	0	0.008290607	0	-0.007988721
0	0.10495775	0	0	0	0	0	-0.042250367	0	-0.086023483
0	0.10156236	0	0	0	0	0	-0.030200819	0	-0.093847952
0	0	0	0	0	0	0	-0.045383017	0	1.1674333
0	0.10540595	0	0	0	0	0	0	0	-0.098048176
0	0.11076237	0	0	0	0	0	-0.068422243	0	0
0	0	0	-0.019497828	0	0	0	0.053680746	0	1.0173791
0	0	0	0	0	0	0	0	0	1.1160994
0	0	0	0	0	0	0	0.050464485	0	1.0185094
0	0	0	0	0	-0.018141004	0	0.052192459	0	1.0229481
0	0	0	0	0	0	0	0.051670579	0	1.0238998
0	0	0	0	0	0	0	-0.066024102	0	1.1749253
0	0	0	-0.034450597	0	0	0	0	0	1.0635856
0	0.11227009	0	0	0	0	0	0	0	0
0	0.10026867	0	0	0	0	0	-0.041895338	0	-0.081511674
0	0	0	-0.018548172	0	0	0	0	0	1.0616283
0	0	0	0	0	-0.018382132	0	0	0	1.0656765
0	0	0	0	0	0	0	0	0	1.0665415

0	0	0	0	0	-0.019396943	0	0	0	1.0733624
0	0.10226381	0	0	0	0	0	-0.067800252	0	-0.094197615
0	0	0	-0.016610068	0	-0.003439901	0	0	0	1.071185
0	0.058371838	0	0	0	0	0	-0.014754976	0	0
0	0.059171298	0	0	0	0	0	0	0	0
0	0	0	0	0	0	0	0	0	1.144294
0	0.10415404	0	0	0	0	0	0	0	-0.076502901
0	0.056434902	0	0	0	0	0	-0.013930632	0	0
0	0.091709207	0	0	0	0.045436745	0	0.063209809	0	0
0	0.059045465	0	0	0	0	0	-0.035092411	0	0
0	0.051773887	0	0	0	0	0	-0.031418846	0	0
0	0.064154214	0	0	0	0	0	0.061097161	0	0
0	0.063917351	0	0	0	0	0	0.062665694	0	0
0	0.064669418	0	0.000485551	0	0	0	0.063315411	0	0
0	0.080277798	0	0	0	0.036843881	0	0.064121572	0	-0.072583703
0	0.057779489	0	0	0	0	0	0.061985627	0	-0.05469545
0	0.093196274	0	0	0	0	0	0	0	1.0355906
0	0.057468098	0	0	0	0	0	0.063257042	0	-0.049620198
0	0.092846023	0	0	0	0.045870336	0	0	0	0
0	0.057203312	0	-0.001436971	0	0	0	0.064059299	0	-0.049371038
0	0.093768773	0	-0.020476174	0	0.064972757	0	0	0	0
0	0.091736583	0	0	0	0.044213566	0	0	0	0
0	0.023448819	0	-0.022659451	0	0	0	0.063516139	0	0
0	0.064793472	0	0	0	0	0	0	0	0
0	0.066442002	0	0.002139437	0	0	0	0	0	0
0	0.030649766	0	0	0	0	0	0.061593897	0	0
0	0.032068884	0	0	0	0.000552895	0	0.062536406	0	0
0	0.02758618	0	-0.008972938	0	0	0	0.062779309	0	0
0	0	0	-0.053470014	0	0.027472099	0	0.066046753	0	0
0	0.060497954	0	-0.008237729	0	0	0	0	0	0
0	0.031102936	0	-0.014091851	0	0.013269851	0	0.064255183	0	0
0	0.080798046	0	0	0	0.036769473	0	0	0	-0.07766003
0	0	0	-0.035345157	0	0	0	0.063513777	0	0
0	0.03099927	0	0	0	0.000391913	0	0.062183316	0	0
0	0	0	-0.037463289	0	0	0	0.065616157	0	0
0	0.081579764	0	-0.018588866	0	0.054067469	0	0	0	-0.075698286
0	0	0	-0.036538593	0	0	0	0.06537929	0	-0.002462557
0	0.07947116	0	0	0	0.034969828	0	0	0	-0.070251748
0	0.0580235	0	0	0	0	0	0	0	-0.054391465
0	0.058628182	0	0.00016414	0	0	0	0	0	-0.05500043
0	0.051832912	0	-0.01141752	0	0	0	0	0	-0.044502054
0	0	0	-0.021288369	0	0	0	0.064317311	0	0
0	0	0	-0.020630397	0	0	0	0.064347336	0	-0.004935222
0	0	0	-0.019512589	0	-0.001440125	0	0.06420486	0	0
0	0	0	0	0	-0.019716379	0	0.062181305	0	0
0	0	0	-0.021720792	0	0.001225755	0	0.066774812	0	0
0	0	0	0	0	0	0	-0.020377281	0	0.007877856

0	0	0	0	0	0	0	-0.0205848	0	0
0	0	0	0	0	-0.018469689	0	0.062854898	0	0
0	0.027621509	0	-0.039775802	0	0.032087022	0	0	0	0
0	0	0	-0.020038365	0	-0.000870611	0	0.06638604	0	0.000472977
0	0	0	0	0	0	0	-0.021290709	0	0
0	0	0	0	0	0	0	0.062509897	0	0
0	0	0	0	0	-0.019051803	0	0.062860691	0	-0.00072316
0	0	0	0	0	-0.019954091	0	0.064025449	0	0.004206781
0	0	0	0	0	-0.019409926	0	0.06433492	0	0
0	0	0	0	0	0	0	0.062536822	0	-0.00187917
0	0.023176278	0	-0.02141131	0	0	0	0	0	0
0	0	0	0	0	0	0	0	0	0.009902364
0	0	0	0	0	0	0	0	0	0
0	0	0	0	0	0	0	-0.019307347	0	0.00783837
0	0	0	0	0	0	0	-0.020209052	0	0
0	0	0	0	0	-0.069101633	0	-0.026668848	0	1.1008021
0	0	0	0	0	0	0	-0.03110189	0	0
0	0	0	0	0	0	0	-0.03107996	0	0.000999152
0	0.030998135	0	0	0	-0.000860287	0	0	0	0
0	0	0	-0.036091096	0	0	0	0	0	0
0	0	0	-0.048562805	0	0.022359993	0	0	0	0
0	0.030261994	0	-0.011138903	0	0.009356959	0	0	0	0
0	0	0	0	0	-0.076233987	0	0	0	1.0816754
0	0	0	0	0	-0.073738368	0	-0.031737024	0	1.1101903
0	0	0	-0.056111692	0	0.030050146	0	0	0	0
0	0	0	-0.035178922	0	0	0	0	0	-0.006342442
0	0	0	-0.010152522	0	-0.067943142	0	-0.03833352	0	1.1083726
0	0	0	-0.052895753	0	0.026590466	0	0	0	-0.004797917
0	0	0	0.015547894	0	-0.09061815	0	0	0	1.0785497
0	0.004603576	0	0	0	-0.074842376	0	-0.040347352	0	1.1077383
0	0.000767875	0	0	0	-0.081207893	0	0	0	1.0739153
0	0	0	0	0	0	0	-0.040899327	0	0.011117035
0	0	0	0	0	0	0	-0.042182832	0	0
0	0	0	-0.010022399	0	-0.082076396	0	0	0	1.0818752
0	-0.007478675	0	0	0	-0.092493362	0	0	0	1.0907946
0	0	0	-0.01932499	0	-0.001353508	0	0	0	0
0	0	0	0	0	-0.019796798	0	0	0	0
0	0	0	0	0	-0.020415014	0	0	0	-8.92E-05
0	0	0	-0.017488651	0	-0.003660732	0	0	0	-0.003785439
0	-0.018379057	0	-0.010205432	0	-0.098982739	0	0	0	1.088611
0	0	0	-0.048777522	0	0	0	-0.052282135	0	1.1268557
0	0.022855845	0	-0.045594285	0	0	0	-0.071191767	0	1.1127025
0	0.049414389	0	0	0	0	0	-0.067508238	0	1.0963969
0	0	0	-0.044418811	0	0	0	-0.058097405	0	1.1255266
0	0.046756396	0	0	0	0	0	-0.083959294	0	1.1274504
0	0	0	0	0	0	0	-0.054878365	0	1.1455808
0	0	0	-0.049959346	0	0	0	0	0	1.0864473

0	0.006326164	0	-0.067516318	0	0	0	0	0	1.0790306
0	0.045961305	0	0	0	0	0	0	0	1.0564817
0	0.018847298	0	-0.044257058	0	0	0	0	0	1.0531538
0	0.015167244	0	0	0	-0.064670665	0	-0.019247992	0	0
0	0.021410712	0	0	0	-0.06321928	0	-0.026044515	0	0
0	0.014706131	0	0.011780205	0	-0.075013652	0	-0.020545503	0	0
0	0	0	0	0	-0.072258182	0	-0.01451828	0	0.015428192
0	0.010942397	0	0	0	-0.068568247	0	-0.022196871	0	0.002392305
0	0	0	0.008517736	0	-0.079891908	0	-0.016394862	0	0.014001473
0	0.015692155	0	0.014138104	0	-0.080811294	0	0	0	0
0	0	0	0.010405371	0	-0.084283903	0	0	0	0.018233126
0	0.006576121	0	0	0	-0.075004903	0	0	0	0.011250542
0	0	0	0	0	-0.072389313	0	-0.015409358	0	0
0	0.01538679	0	0	0	-0.071980085	0	0	0	0
0	0	0	0.002727384	0	-0.07441761	0	-0.015762377	0	0
0	0	0	0.006208989	0	-0.078096275	0	-0.017151915	0	0
0	0.006597515	0	0.016931178	0	-0.090246503	0	0	0	0.009826187
0	0.015700908	0	0.01810861	0	-0.087831904	0	0	0	0
0	0	0	0.008842953	0	-0.083734879	0	0	0	0
0	0	0	-0.010345898	0	-0.070070405	0	-0.024066719	0	0.0154558
0	0.011015856	0	-0.011297903	0	-0.064582557	0	-0.02911395	0	0
0	0.004793513	0	0	0	-0.076925256	0	-0.025845987	0	0.013179042
0	0.01437574	0	0	0	-0.072968213	0	-0.03074718	0	0
0	0	0	-0.012103029	0	-0.068850823	0	-0.025244435	0	0
0	0.000960802	0	-0.010228228	0	-0.073242059	0	-0.031953426	0	0.010474435
0	0.010613828	0	-0.011773065	0	-0.06780782	0	-0.03670509	0	0
0	-0.006852463	0	-0.01034159	0	-0.08455117	0	0	0	0.024771777
0	0.000441118	0	-0.011879796	0	-0.08242627	0	0	0	0
0	0.042127952	0	-0.02818968	0	0	0	-0.057921055	0	0
0	0.032857025	0	-0.044036917	0	0	0	-0.055275569	0	0
0	0.022948934	0	-0.034731891	0	0	0	-0.044058493	0	0
0	0.058312896	0	0	0	0	0	-0.053118686	0	0
0	0.024839179	0	-0.045288168	0	0	0	-0.05228465	0	-0.01171259
0	0.032887317	0	-0.031461007	0	0	0	-0.056478481	0	-0.024379559
0	0	0	-0.046529473	0	0	0	-0.042255897	0	0.009228439
0	0.050967424	0	0	0	0	0	-0.051386416	0	-0.029664815
0	0	0	-0.04761777	0	0	0	-0.043134338	0	0
0	0.034271168	0	-0.037067531	0	0	0	0	0	0
0	0.025730059	0	-0.039785229	0	0	0	0	0	-0.007074661
0	0.11186561	0	0	0	0	0	0.079344966	0	0.91107162
0	0.10691933	0	0	0	0	0	0.078779187	0	0.91641873
0	0.049057333	0	0	0	-0.11765128	0	0.037588729	0	1.0201315
0	0.08722507	0	0	0	-0.044032087	0	0	0	1.013598
0	0.10914787	0	0	0	0	0	0	0	0.98946967
0	0.10152785	0	-0.017009123	0	0	0	0	0	0.99477396
0	0	0	0	0	-0.10831612	0	0.081789028	0	1.0237883
0	0.10923481	0	0	0	0	0	0	0	0.9836356

0	0.10967655	0	0	0	0	0	0	0	0.99005826
0	0.059578252	0	0	0	-0.095516793	0	0	0	1.044075
0	0.063474272	0	0	0	-0.088396793	0	0	0	1.0404299
0	0.062924597	0	0	0	-0.0894987	0	0	0	1.040243
0	0.063435281	0	-0.000210654	0	-0.088418226	0	0	0	1.0403026
0	0	0	0	0	-0.13943792	0	0.050244046	0	1.0563647
0	0	0	0	0	-0.13450303	0	0.055250501	0	1.052122
0	0.045037233	0	0	0	-0.11471273	0	0.036664946	0	1.0251391
0	0	0	0	0	-0.13425846	0	0.055277645	0	1.0523311
0	0	0	-0.001095053	0	-0.13361001	0	0.05469081	0	1.0521153
0	0.084074259	0	0	0	-0.040326332	0	0	0	1.0164964
0	0.096581391	0	-0.017304416	0	0	0	0	0	0.99947628
0	0	0	0	0	-0.10299427	0	0.081273277	0	1.0242448
0	0.10429698	0	0	0	0	0	0	0	0.9941495
0	0.10446137	0	0	0	0	0	0	0	0.98763663
0	0.056200751	0	0	0	-0.091773752	0	0	0	1.0475101
0	0.099269751	0	0	0	-0.041173216	0	0.09327561	0	0
0	0.058316193	0	0	0	-0.088049773	0	0	0	1.0445058
0	0.059044629	0	-0.002499656	0	-0.084477168	0	0	0	1.0442149
0	0.11061293	0	-0.019862787	0	0	0	0.093247074	0	0
0	0.11952854	0	0	0	0	0	0.090318369	0	0
0	0	0	0	0	-0.13417586	0	0.049206501	0	1.0575998
0	0	0	-0.003304412	0	-0.12765337	0	0.052575484	0	1.0538524
0	0	0	0	0	-0.13044134	0	0.05285604	0	1.0544881
0	0	0	-0.025596286	0	-0.097959173	0	0	0	1.0964171
0	0.11960235	0	0	0	0	0	0.089574709	0	0
0	0.11999127	0	0	0	0	0	0.091125988	0	0
0	0	0	0	0	-0.1153697	0	0	0	1.0985786
0	0	0	0	0	-0.10584975	0	0	0	1.1013101
0	0	0	0	0	-0.11207958	0	0	0	1.1021738
0	0.087892492	0	0	0	-0.049306342	0	0.094221725	0	-0.087088707
0	0	0	0	0	-0.10263321	0	0	0	1.0961019
0	0	0	-0.002621351	0	-0.10049601	0	0	0	1.0954758
0	0	0	0	0	-0.10286371	0	0	0	1.0960606
0	0	0	-0.078881326	0	0	0	0	0	1.0800761
0	0	0	0	0	-0.1035415	0	0	0	1.103092
0	0	0	-0.002032167	0	-0.10148037	0	0	0	1.1024746
0	0.095970575	0	0	0	-0.037347043	0	0.092579553	0	0
0	0	0	-0.003111065	0	-0.091257164	0	0	0	1.1015685
0	0.1054353	0	-0.020212211	0	0	0	0.092810624	0	0
0	0.10278654	0	-0.021391419	0	0	0	0.09417888	0	-0.10601161
0	0	0	-0.025790419	0	-0.093483077	0	0	0	1.0965573
0	0	0	-0.049159292	0	0	0	0.080585905	0	1.012722
0	0.11448738	0	0	0	0	0	0.089838585	0	0
0	0.1124558	0	0	0	0	0	0.091158896	0	-0.111921
0	0.11460554	0	0	0	0	0	0.088891274	0	0
0	0	0	0	0	-0.1113591	0	0	0	1.0983995

0	0.11261057	0	0	0	0	0	0.09066989	0	-0.11479068
0	0	0	0	0	-0.10021326	0	0	0	1.1012413
0	0.11291665	0	0	0	0	0	0.091972593	0	-0.11201715
0	0	0	-0.004490964	0	-0.096732697	0	0	0	1.0952546
0	0	0	-0.076580669	0	0	0	0	0	1.0806632
0	0	0	0	0	-0.097697666	0	0	0	1.0953515
0	0	0	-0.048486098	0	0	0	0.080428298	0	1.013116
0	0	0	-0.004249116	0	-0.094138487	0	0	0	1.1018456
0	0.084563349	0	0	0	-0.045602269	0	0.093508987	0	-0.083321218
0	0.097665036	0	-0.021751002	0	0	0	0.093694471	0	-0.10054831
0	0.097766392	0	-0.040768035	0	0	0	0	0	0
0	0.1074766	0	0	0	0	0	0.09062726	0	-0.1064832
0	0.029725264	0	0	0	-0.092365283	0	0.091620184	0	0
0	0.10767518	0	0	0	0	0	0.089939659	0	-0.10985206
0	0.077090709	0	-0.06139536	0	0	0	0	0	1.0302847
0	0	0	-0.039330739	0	-0.075600559	0	0.094239471	0	0
0	0.03080817	0	0	0	-0.089745749	0	0.092642815	0	0
0	0.030993136	0	0.000853822	0	-0.090346123	0	0.092450498	0	0
0	0.030833136	0	0	0	-0.089616048	0	0.092453733	0	0
0	0.098406427	0	0	0	-0.03817536	0	0	0	0
0	0.07585253	0	-0.061022722	0	0	0	-0.023126007	0	1.0409334
0	0.10048855	0	0	0	-0.034093331	0	0	0	0
0	0.11724384	0	0	0	0	0	0	0	0
0	0.029691545	0	-0.06424775	0	0	0	0.090900916	0	0
0	0.11020213	0	-0.015733015	0	0	0	0	0	0
0	0.057625292	0	0	0	-0.11853775	0	0.045717963	0	0
0	0.055670376	0	0	0	-0.12134007	0	0.04307588	0	0
0	0.056385249	0	0	0	-0.12035106	0	0.043095877	0	0
0	0.055770817	0	-0.000878494	0	-0.12059404	0	0.042549868	0	0
0	0.10090498	0	-0.006000416	0	-0.028660065	0	0	0	0
0	0.10082623	0	0	0	-0.034529589	0	0	0	0
0	0	0	0	0	-0.11107877	0	0.093776448	0	0
0	0.11779254	0	0	0	0	0	0	0	0
0	0	0	0	0	-0.11126463	0	0.09237099	0	0
0	0	0	0	0	-0.11162817	0	0.093459594	0	0
0	0	0	0	0	-0.11139296	0	0.093484005	0	-0.003064663
0	0	0	0	0	0	0	0.073518196	0	1.028836
0	0.11771756	0	0	0	0	0	0	0	0
0	0.11096133	0	-0.015364454	0	0	0	0	0	0
0	0.067985387	0	-0.075719496	0	0	0	0	0	1.0260123
0	0.088600966	0	-0.043333649	0	0	0	0	0	-0.093976615
0	0.072346427	0	-0.061305855	0	0	0	0	0	1.0341803
0	0.02896904	0	0	0	-0.086955148	0	0.091142453	0	0
0	0	0	-0.085604672	0	0	0	0.094496056	0	0
0	0.07054395	0	-0.071456361	0	0	0	0	0	1.0239968
0	0	0	-0.045411132	0	0	0	0	0	1.0833961
0	0.093233381	0	-0.039739612	0	0	0	0	0	0

0	0	0	0	0	0	0	0.072822105	0	1.0249376
0	0	0	0	0	-0.10830901	0	0.093199365	0	0
0	0	0	0	0	0	0	0.074523594	0	1.0292409
0	0.048089997	0	0	0	-0.12192839	0	0.049692996	0	-0.042473284
0	0	0	-0.045380986	0	0	0	0	0	1.0917403
0	0	0	-0.084395023	0	0	0	0.094301941	0	-0.020950738
0	0	0	-0.007658301	0	-0.10145074	0	0.095190134	0	0
0	0	0	-0.040768188	0	-0.068663428	0	0.093830412	0	0
0	0.045998884	0	0	0	-0.1249235	0	0.046867455	0	-0.043071504
0	0	0	0	0	-0.10901694	0	0.09498033	0	0
0	0	0	0	0	-0.10873963	0	0.09321347	0	-0.005586814
0	0	0	0	0	-0.10917948	0	0.09328663	0	0
0	0	0	-0.004895823	0	-0.10432847	0	0.093352313	0	0
0	0.046110931	0	0	0	-0.12498712	0	0.04632248	0	-0.043155699
0	0.046152277	0	0.000396865	0	-0.12528699	0	0.046354061	0	-0.043122027
0	0.07134946	0	-0.060935809	0	0	0	-0.021417497	0	1.0439444
0	0	0	-0.005308395	0	-0.104356	0	0.094777312	0	0
0	0	0	-0.006002732	0	-0.10337664	0	0.094839101	0	-0.003796362
0	0.029620982	0	0	0	-0.084835293	0	0.091714364	0	0
0	0	0	0	0	-0.10934551	0	0.09467698	0	-0.00264239
0	0.029843278	0	-0.00171524	0	-0.08311205	0	0.092090916	0	0
0	0.085838774	0	0	0	-0.047143447	0	0	0	-0.082492039
0	0	0	0	0	0	0	0.073398506	0	1.0289898
0	0.028398587	0	-0.062747858	0	0	0	0.090683051	0	0
0	0	0	-0.04474535	0	0	0	0	0	1.0831352
0	0	0	-0.080859387	0	0	0	0.092862072	0	0
0	0	0	-0.080612438	0	0	0	0.09243719	0	0
0	0	0	0	0	0	0	0.072581216	0	1.0247575
0	0.095478037	0	0	0	-0.033859228	0	0	0	0
0	0.088115658	0	0	0	-0.042951612	0	0	0	-0.089057602
0	0.097393331	0	0	0	-0.030308516	0	0	0	0
0	0.10522859	0	-0.016041978	0	0	0	0	0	0
0	0.067443812	0	-0.008586841	0	-0.089144732	0	0	0	0
0	0.11237181	0	0	0	0	0	0	0	0
0	0.069605416	0	0	0	-0.093164528	0	0	0	0
0	0.069449376	0	0	0	-0.093567973	0	0	0	0
0	0.066790075	0	-0.069995671	0	0	0	0	0	1.0281343
0	0.071614929	0	0	0	-0.089482089	0	0	0	0
0	0.072235872	0	-0.001797846	0	-0.086732876	0	0	0	0
0	0.071564391	0	-0.001674809	0	-0.088146869	0	0	0	0
0	0.022586369	0	0	0	-0.13294685	0	0.056740725	0	0
0	0.023138284	0	0	0	-0.12993483	0	0.059260303	0	0
0	0.088376903	0	-0.004145085	0	-0.039359788	0	0	0	-0.085310434
0	0	0	-0.083269474	0	0	0	0.094187447	0	0
0	0.023727227	0	0.003688411	0	-0.13300061	0	0.058802449	0	0
0	0.088306789	0	0	0	-0.043426605	0	0	0	-0.084456085
0	0	0	0	0	-0.1053279	0	0.093282885	0	0

0	0	0	0	0	-0.10550909	0	0.09168159	0	0
0	0.019772577	0	0	0	-0.13600553	0	0.053886851	0	0
0	0.020936365	0	0.003557319	0	-0.13643583	0	0.055682257	0	0
0	0	0	0	0	-0.10566582	0	0.092985104	0	-0.002492863
0	0.019889287	0	0.000246009	0	-0.13622251	0	0.053368404	0	0
0	0.10952765	0	0	0	0	0	0	0	-0.11060412
0	0.09788987	0	-0.008281674	0	-0.022596497	0	0	0	0
0	0.10169334	0	-0.017328386	0	0	0	0	0	-0.1061709
0	0.053242391	0	0	0	-0.11599842	0	0.044244485	0	0
0	0.053041383	0	0	0	-0.1161529	0	0.042842969	0	0
0	0.051610466	0	-0.00340243	0	-0.11542749	0	0.041591496	0	0
0	0.10997227	0	0	0	0	0	0	0	-0.10653949
0	0	0	-0.082066589	0	0	0	0.093979286	0	-0.019893739
0	0.084118576	0	-0.04234786	0	0	0	0	0	-0.088847592
0	0.10231377	0	-0.017006805	0	0	0	0	0	-0.10166317
0	0	0	0	0	-0.10298865	0	0.092513257	0	0
0	0.11000235	0	0	0	0	0	0	0	-0.11108214
0	0	0	-0.009962388	0	-0.093944045	0	0.094850974	0	0
0	0	0	-0.007146791	0	-0.096902405	0	0.092794325	0	0
0	0	0	0	0	-0.1034213	0	0.092525003	0	-0.005504261
0	0	0	-0.012321917	0	-0.13635039	0	0.056066652	0	0
0	0	0	-0.011145991	0	-0.13609392	0	0.058574996	0	0
0	0	0	-0.009743959	0	-0.137962	0	0.057240138	0	0
0	0	0	0	0	-0.14459159	0	0.059357063	0	0
0	0	0	-0.008294968	0	-0.095873632	0	0.094470175	0	-0.003720688
0	0	0	0	0	-0.14418804	0	0.059902258	0	0
0	0	0	0	0	-0.14384396	0	0.060938759	0	0
0	0	0	-0.010007366	0	-0.13791154	0	0.056611296	0	-0.001786947
0	0	0	0	0	-0.14457655	0	0.059372559	0	-1.73E-05
0	0	0	0	0	-0.14384995	0	0.060585415	0	0.001369475
0	0	0	-0.002975562	0	-0.1381229	0	0.062772666	0	0
0	0	0	-0.078371406	0	0	0	0.091973719	0	0
0	0	0	0	0	-0.14050537	0	0.063290859	0	0
0	0	0	-0.002731631	0	-0.13810405	0	0.062768402	0	0
0	0	0	-0.001435283	0	-0.13876428	0	0.064029131	0	0
0	0	0	0	0	-0.14000758	0	0.064103935	0	0.000628847
0	0	0	-0.001333753	0	-0.13953065	0	0.062868677	0	-0.000375009
0	0	0	-0.001117582	0	-0.1390359	0	0.063642154	0	0.000507415
0	0.057718594	0	-0.009307635	0	-0.091486907	0	0	0	-0.055484029
0	0.040997712	0	-0.032867598	0	0	0	0.090000787	0	0
0	0.041519075	0	-0.032652801	0	0	0	0.090186083	0	0
0	0.06006165	0	0	0	-0.095863918	0	0	0	-0.056013991
0	0.059959199	0	0	0	-0.096172949	0	0	0	-0.056004891
0	0.043738163	0	0	0	-0.11942361	0	0.048242183	0	-0.038060033
0	0.063088199	0	0	0	-0.090395835	0	0	0	-0.059600128
0	0.063093051	0	-0.000441035	0	-0.090114531	0	0	0	-0.059637842
0	0.063892667	0	-0.000561201	0	-0.088488317	0	0	0	-0.059802086

0	0.042785997	0	0	0	-0.12084301	0	0.046062038	0	-0.039352433
0	0.042023392	0	-0.002087436	0	-0.12019017	0	0.045412461	0	-0.038998957
0	0.082892908	0	0	0	-0.042946335	0	0	0	-0.079082766
0	0.085011632	0	0	0	-0.039271877	0	0	0	-0.085966733
0	0	0	-0.095489315	0	0	0	0	0	1.0976405
0	0	0	0	0	0	0	0	0	1.0927409
0	0.10669171	0	0	0	0	0	0	0	1.0214001
0	0.096795697	0	-0.017644107	0	0	0	0	0	-0.10133282
0	0.085349046	0	-0.006401013	0	-0.033425665	0	0	0	-0.082304588
0	0.10473341	0	0	0	0	0	0	0	-0.10579394
0	0.021146682	0	0	0	-0.12848128	0	0.055319555	0	0
0	0.063755289	0	-0.009813645	0	-0.084748171	0	0	0	0
0	0.1050682	0	0	0	0	0	-0.037926838	0	1.0418131
0	0.021680063	0	0.000985702	0	-0.12753433	0	0.056568755	0	0
0	0.065683107	0	0	0	-0.090420946	0	0	0	0
0	0.066822284	0	-0.003993537	0	-0.084693966	0	0	0	0
0	0	0	0	0	0	0	0	0	1.0999655
0	0.028053802	0	-0.025499656	0	-0.064707234	0	0	0	0
0	0.018623112	0	-0.001229248	0	-0.13041053	0	0.052327119	0	0
0	0	0	0	0	0	0	0	0	1.0933536
0	0.039538784	0	-0.03262248	0	0	0	0.089827906	0	0
0	0.052035969	0	0	0	0	0	0.085891208	0	0
0	0.05113062	0	0	0	0	0	0.085203019	0	0
0	0	0	-0.093819197	0	0	0	-0.018331264	0	1.1040949
0	0.030699548	0	0	0	-0.086180112	0	0	0	0
0	0.031685796	0	0	0	-0.085721406	0	0	0	0
0	0	0	-0.013037388	0	-0.13057269	0	0.054924542	0	0
0	0	0	0	0	0	0	0	0	1.092243
0	0	0	-0.011620925	0	-0.13071811	0	0.056946257	0	0
0	0	0	-0.010716731	0	-0.13215004	0	0.055462824	0	-0.001263181
0	0	0	-0.098005661	0	0	0	0	0	1.0878414
0	0	0	0	0	-0.1391235	0	0.058361956	0	0
0	0	0	-0.09309885	0	0	0	0	0	1.0974766
0	0	0	-0.07398641	0	0	0	0	0	1.0984985
0	0	0	-0.004970697	0	-0.13229463	0	0.060302388	0	0
0	0	0	0	0	-0.13879343	0	0.059038991	0	0.001631882
0	0.024646448	0	-0.011815311	0	-0.099102665	0	0	0	0
0	0.052201564	0	0	0	0	0	0.086603437	0	0
0	0.025114182	0	-0.012068828	0	-0.09831159	0	0	0	0
0	0	0	-0.003341864	0	-0.13324034	0	0.061167468	0	0.000550302
0	0	0	-0.074758407	0	0	0	0.001656114	0	1.0971208
0	0.027333578	0	0	0	-0.10482594	0	0	0	0
0	0.10435306	0	0	0	0	0	-0.046707021	0	1.0658501
0	0.054064324	0	-0.010503099	0	-0.087178764	0	0	0	-0.051595967
0	0	0	-0.043338454	0	-0.065602377	0	0	0	0
0	0	0	-0.077248473	0	0	0	-0.006984761	0	1.0980856
0	0.056226175	0	0	0	-0.093087529	0	0	0	-0.052223869

0	0.1021768	0	0	0	0	0	0	0	1.0248453
0	0.058415514	0	-0.002704472	0	-0.086741256	0	0	0	-0.055217524
0	0.032086209	0	0.00398969	0	-0.086898268	0	0	0	0
0	0.031578278	0	0	0	-0.083410472	0	0	0	0
0	0	0	-0.035373869	0	-0.073318889	0	0	0	0
0	0.10080365	0	0	0	0	0	-0.035896094	0	1.0441885
0	0.033395408	0	0.004005984	0	-0.08630223	0	0	0	0
0	0	0	-0.091718007	0	0	0	-0.016550474	0	1.1031693
0	0.030199341	0	0.004321429	0	-0.097380559	0	0	0	0
0	0	0	-0.040065211	0	-0.068993471	0	0	0	-0.008121504
0	0	0	-0.036910383	0	-0.07229794	0	0	0	0
0	0	0	-0.050821276	0	0	0	0.091648108	0	0
0	0	0	-0.026174496	0	-0.1009725	0	0	0	0
0	0.050068995	0	0	0	0	0	0.085492715	0	0
0	0.031630444	0	-0.058929944	0	0	0	0	0	0
0	0.031894109	0	-0.058918651	0	0	0	0	0	0
0	0	0	-0.028510002	0	-0.096785121	0	0	0	0
0	0	0	-0.07410895	0	0	0	0.001865799	0	1.096772
0	0	0	-0.050974166	0	0	0	0.093817605	0	0
0	0	0	-0.024358288	0	-0.10141363	0	0	0	-0.00572037
0	0.027098423	0	-0.027314292	0	-0.057960371	0	0	0	0
0	0	0	-0.023289796	0	-0.10145277	0	0	0	0
0	0	0	-0.026244745	0	-0.098031814	0	0	0	-0.004442807
0	0	0	-0.05001101	0	0	0	0.091683587	0	-0.017771754
0	0	0	0	0	-0.10490052	0	0	0	0
0	0.10468466	0	0	0	0	0	0	0	1.0105608
0	0	0	-0.050155875	0	0	0	0.093534814	0	-0.013927214
0	0	0	0	0	-0.10537126	0	0	0	-0.004361124
0	0	0	0	0	-0.10565527	0	0	0	0
0	0	0	0	0	-0.11685809	0	0	0	0
0	0	0	-0.048278022	0	0	0	0.091238902	0	0
0	0.029638289	0	0	0	-0.081065371	0	0	0	0
0	0	0	0	0	-0.1056971	0	0	0	0
0	0	0	0	0	-0.11618948	0	0	0	-0.00239198
0	0	0	0	0	-0.10606381	0	0	0	-9.27E-05
0	0	0	-0.080116592	0	0	0	0	0	0
0	0	0	-0.050173102	0	0	0	0.091379686	0	0
0	0.099644755	0	0	0	0	0	-0.046358153	0	1.0694598
0	0.023539299	0	-0.012742898	0	-0.094468366	0	0	0	0
0	0	0	-0.080789721	0	0	0	0	0	0
0	0	0	-0.044633103	0	-0.058883197	0	0	0	0
0	0	0	-0.004672643	0	-0.1006223	0	0	0	0
0	0	0	-0.00489374	0	-0.097722288	0	0	0	0
0	0	0	-0.049362481	0	0	0	0.091409306	0	-0.017461009
0	0	0	0	0	-0.10257945	0	0	0	0
0	0	0	-0.078890791	0	0	0	0	0	-0.021061958
0	0	0	-0.036551822	0	-0.06675241	0	0	0	0

0	0.10187571	0	0	0	0	0	-0.070092843	0	1.0760575
0	0.030676702	0	0.001531627	0	-0.08002208	0	0	0	0
0	0	0	-0.002994873	0	-0.10108046	0	0	0	-0.004960842
0	0	0	0	0	-0.10304481	0	0	0	-0.004533328
0	0	0	-0.003018882	0	-0.10002232	0	0	0	-0.005119221
0	0	0	-0.002017236	0	-0.10134632	0	0	0	0
0	0	0	-0.041357109	0	-0.062285495	0	0	0	-0.007848392
0	0.030077247	0	-0.057427628	0	0	0	0	0	0
0	0	0	-0.079467468	0	0	0	0	0	-0.016939138
0	0	0	-0.004232868	0	-0.099179114	0	0	0	0
0	0	0	-0.002434012	0	-0.1013099	0	0	0	-6.53E-05
0	0	0	-0.02612687	0	-0.096958323	0	0	0	0
0	0	0	-0.07584605	0	0	0	0	0	0
0	0	0	-0.024301554	0	-0.097424635	0	0	0	-0.005333496
0	0	0	0	0	-0.099315278	0	0	0	0
0	0	0	-0.077807473	0	0	0	0	0	0
0	0	0	0	0	-0.099790715	0	0	0	-0.004283727
0	0	0	-0.07659031	0	0	0	0	0	-0.020433244
0	0	0	-0.007113529	0	-0.090468559	0	0	0	0
0	0.085312627	0	-0.061986284	0	0	0	0	0	0
0	0.086679289	0	-0.059522173	0	0	0	-0.00593449	0	0
0	0.083673484	0	-0.065517101	0	0	0	0	0	0
0	0	0	-0.005241175	0	-0.092749022	0	0	0	-0.005481778
0	0.10350283	0	0	0	0	0	0	0	1.0422641
0	0.042223428	0	-0.028363556	0	0	0	0	0	0
0	0.083002651	0	-0.063907179	0	0	0	-0.018537892	0	0
0	0.051390488	0	0	0	0	0	0	0	0
0	0.083099525	0	-0.064076068	0	0	0	-0.019538317	0	0
0	0	0	0	0	0	0	0.084809932	0	0
0	0	0	0	0	0	0	0.086611202	0	0
0	0	0	0	0	0	0	0.084839766	0	-0.009428565
0	0.079935727	0	-0.069726513	0	0	0	0	0	0
0	0	0	0	0	0	0	0.084693459	0	0
0	0	0	0	0	0	0	0.086333655	0	-0.006006015
0	0.077232406	0	-0.06211253	0	0	0	0	0	-0.075389678
0	0.074515583	0	-0.067680776	0	0	0	0	0	-0.07308748
0	0	0	0	0	0	0	0.085601124	0	0
0	0.081749333	0	-0.059722112	0	0	0	-0.005275926	0	0
0	0.078122581	0	-0.060614109	0	0	0	-0.003673423	0	-0.076228243
0	0.079435887	0	-0.064306725	0	0	0	0	0	0
0	0	0	0	0	0	0	0.085630217	0	-0.009509725
0	0	0	0	0	0	0	0.084575739	0	0
0	0	0	0	0	0	0	0.084601171	0	-0.009217123
0	0.078974384	0	-0.063161802	0	0	0	-0.017417504	0	0
0	0.074217041	0	-0.065241632	0	0	0	-0.016914928	0	-0.080692248
0	0.073617152	0	-0.066697968	0	0	0	-0.018505176	0	-0.080344094
0	0	0	-0.046653643	0	0	0	0	0	0

0	0.070729298	0	-0.072018571	0	0	0	0	0	-0.078666621
0	0	0	-0.045767168	0	0	0	0	0	-0.015834115
0	0.070336205	0	-0.066504394	0	0	0	0	0	-0.069090538
0	0.073281885	0	-0.060806257	0	0	0	-0.003024901	0	-0.071654595
0	0.069566504	0	-0.0658173	0	0	0	-0.016419436	0	-0.0758502
0	0.11518929	0	0	0	0	0	-0.020321517	0	0
0	0.11520666	0	0	0	0	0	0	0	0
0	0.031788122	0	-0.077518563	0	0	0	0	0	0
0	0.032008462	0	-0.077439133	0	0	0	-5.74E-05	0	0
0	0.079364994	0	0	0	0.037363641	0	0.051498582	0	0.94187915
0	0.035893218	0	-0.064746513	0	0	0	0.005459687	0	0
0	0.11381442	0	0	0	0	0	-0.032190517	0	0
0	0.056554372	0	0	0	0	0	0.049412548	0	0.95821235
0	0.056240178	0	0	0	0	0	0.050648888	0	0.96377036
0	0.11063157	0	0	0	0	0	-0.019236412	0	0
0	0.056340213	0	-0.0007589	0	0	0	0.051405887	0	0.96366604
0	0.030304003	0	-0.076068309	0	0	0	0.000692073	0	0
0	0	0	-0.097760487	0	0	0	0	0	0
0	0	0	0	0	0	0	0	0	0
0	0	0	-0.097588989	0	0	0	-0.001019783	0	0
0	0.10708726	0	0	0	0	0	-0.018980078	0	-0.091687398
0	0	0	-0.096258525	0	0	0	0	0	-0.00973896
0	0	0	0	0	0	0	0	0	-0.008258094
0	0.079714993	0	0	0	0.037366039	0	0	0	0.98367646
0	0	0	-0.096221393	0	0	0	-0.000322011	0	-0.009561492
0	0	0	-0.094747723	0	0	0	0.00105089	0	0
0	0.025227835	0	-0.079288351	0	0	0	-0.014930332	0	0
0	0	0	0	0	0	0	-0.027097183	0	1.1466996
0	0.080483615	0	-0.018467298	0	0.054563218	0	0	0	0.987869
0	0.1071956	0	0	0	0	0	0	0	-0.089860982
0	0.078421967	0	0	0	0.035625333	0	0	0	0.99290352
0	0.11315033	0	0	0	0	0	-0.043606758	0	0
0	0	0	-0.097493553	0	0	0	-0.011098927	0	0
0	0.056590734	0	0	0	0	0	0	0	1.0052656
0	0	0	0	0	0	0	-0.026029547	0	1.1452449
0	0	0	0	0	0	0	-0.036840858	0	1.1470374
0	0	0	-0.079405401	0	0	0	0.008229805	0	0
0	0	0	-0.095374521	0	0	0	-0.000136338	0	0
0	0.057354375	0	0.000481234	0	0	0	0	0	1.0051007
0	0	0	-0.096119951	0	0	0	-0.011270559	0	-0.016634827
0	0.10561679	0	0	0	0	0	-0.031595048	0	-0.098180547
0	0	0	0	0	0	0	0	0	1.1278555
0	0.050794661	0	-0.01069025	0	0	0	0	0	1.0162746
0	0	0	-0.078280841	0	0	0	0.008790743	0	-0.008370151
0	0	0	-0.094013525	0	0	0	0.000546415	0	-0.009191096
0	0.1026174	0	0	0	0	0	-0.017928314	0	-0.087500646
0	0	0	-0.035211719	0	0	0	0.052699947	0	1.0111562

0	0	0	0	0	0	0	-0.045396648	0	1.1680346
0	0.10483917	0	0	0	0	0	-0.041958153	0	-0.085969611
0	0	0	-0.019658256	0	0	0	0.051639303	0	1.0115438
0	0	0	-0.019310825	0	-0.000379157	0	0.053663188	0	1.0174181
0	0	0	0	0	-0.017893107	0	0.050200924	0	1.0173469
0	0	0	0	0	-0.018788232	0	0.051338749	0	1.0225893
0	0	0	0	0	0	0	0.049831308	0	1.0185706
0	0	0	-0.033990867	0	0	0	0	0	1.0556504
0	0	0	-0.052143637	0	0.027310164	0	0	0	1.0600783
0	0	0	0	0	-0.019041188	0	0	0	1.0650103
0	0	0	-0.017353605	0	-0.002426069	0	0	0	1.0617904
0	0.058253091	0	0	0	0	0	-0.014319743	0	0
0	0.092414045	0	-0.023584248	0	0.067630025	0	0.064896177	0	0
0	0.091536966	0	0	0	0.045592205	0	0.060928797	0	0
0	0.090519988	0	0	0	0.044151623	0	0.062639046	0	0
0	0.063710659	0	0	0	0	0	0.060575125	0	0
0	0.064255251	0	0.00018857	0	0	0	0.061083547	0	0
0	0.058305164	0	-0.010039521	0	0	0	0.063089333	0	0
0	0.090079582	0	-0.052937114	0	0.090481794	0	0	0	0
0	0.081019972	0	-0.021863653	0	0.057478204	0	0.065747496	0	-0.077594966
0	0.080194184	0	0	0	0.037032024	0	0.06212806	0	-0.077573517
0	0.0790157	0	0	0	0.035485801	0	0.063508809	0	-0.071523569
0	0.093513892	0	-0.021295184	0	0.065924445	0	0	0	0
0	0.057322796	0	0	0	0	0	0.061445787	0	-0.054201595
0	0.050146388	0	-0.013047703	0	0	0	0.063747332	0	-0.044863195
0	0.05686229	0	-0.001705836	0	0	0	0.062101618	0	-0.054103672
0	0.091641946	0	0	0	0.044564612	0	0	0	0
0	0.025873925	0	-0.042853777	0	0.034417434	0	0.064259961	0	0
0	0.078041737	0	-0.050436205	0	0.079152067	0	0	0	-0.079112872
0	0.021980496	0	-0.022948527	0	0	0	0.062500673	0	0
0	0.060515405	0	-0.007667698	0	0	0	0	0	0
0	0.081469893	0	-0.019359802	0	0.055043188	0	0	0	-0.082242172
0	0	0	-0.05938413	0	0.033824036	0	0.066784894	0	0
0	0	0	-0.051606311	0	0.025770304	0	0.064412121	0	0
0	0	0	-0.036985613	0	0	0	0.063553407	0	0
0	0.030507896	0	0	0	-0.00047195	0	0.061592837	0	0
0	0.079500335	0	0	0	0.03536774	0	0	0	-0.076312029
0	0.029368672	0	-0.014086	0	0.01211848	0	0.063202198	0	0
0	0	0	-0.056357562	0	0.030563387	0	0.066496832	0	-0.007288947
0	0	0	-0.036131103	0	0	0	0.063622392	0	-0.006938436
0	0.051926452	0	-0.01088	0	0	0	0	0	-0.050346698
0	0	0	-0.022302064	0	0.002055269	0	0.064405148	0	0
0	0	0	-0.020574368	0	-0.000118906	0	0.06434243	0	-0.004919913
0	0	0	0	0	-0.019088869	0	0.062188178	0	0
0	0	0	0	0	-0.019669858	0	0.062188758	0	-0.000589742
0	0.025642555	0	-0.039023727	0	0.030140095	0	0	0	0
0	0	0	0	0	0	0	-0.019782405	0	0.007766047

0	0	0	0	0	0	0	-0.02068054	0	0
0	0.013566408	0	0	0	-0.062488605	0	-0.031247417	0	1.0914735
0	0	0	0	0	-0.067696333	0	-0.023193514	0	1.1014267
0	0	0	-0.055415649	0	0.029836943	0	0	0	0
0	0	0	0.006164063	0	-0.073449122	0	-0.025120663	0	1.1008163
0	0	0	-0.052299236	0	0.026421784	0	0	0	-0.010462941
0	0	0	-0.01023747	0	-0.064864521	0	-0.031795487	0	1.1080578
0	0	0	0.010982517	0	-0.083304388	0	0	0	1.083597
0	0.007835032	0	0	0	-0.070272817	0	-0.034759787	0	1.1055109
0	0.005785458	0	0	0	-0.073964669	0	0	0	1.0762592
0	0.004352262	0	-0.010057074	0	-0.066239761	0	-0.040311456	0	1.1058234
0	0.005697213	0	0.017512565	0	-0.089889705	0	0	0	1.0732448
0	-0.00771534	0	-0.010205904	0	-0.083735192	0	0	0	1.0888671
0	0.025977846	0	-0.04168788	0	0	0	-0.058821521	0	1.1083663
0	0.033297009	0	-0.029113454	0	0	0	-0.062577592	0	1.0990918
0	0.05001378	0	0	0	0	0	-0.058108629	0	1.095122
0	0	0	-0.044389006	0	0	0	-0.049253995	0	1.1249065
0	0.024865419	0	-0.038870659	0	0	0	0	0	1.0643031
0	0.023195295	0	0	0	-0.060663692	0	-0.022476524	0	0
0	0.016922456	0	0.009939478	0	-0.070953431	0	-0.017669758	0	0
0	0.023876144	0	0.011964353	0	-0.070449452	0	-0.023882788	0	0
0	0.012879207	0	0	0	-0.065796826	0	-0.018358415	0	0.003865157
0	0.013379768	0	0.011748891	0	-0.075660449	0	-0.020079218	0	0.002187002
0	0	0	0.006513625	0	-0.076953482	0	-0.013151059	0	0.016374962
0	0.009859146	0	0.013367858	0	-0.082323536	0	0	0	0.010054894
0	0.018957715	0	0.01457167	0	-0.079959234	0	0	0	0
0	0	0	0.004551683	0	-0.075673029	0	-0.014448757	0	0
0	0.004540048	0	-0.010250433	0	-0.068152612	0	-0.025807817	0	0.011478137
0	0.01407206	0	-0.011788336	0	-0.062874116	0	-0.030710947	0	0
0	0.042952715	0	-0.027766505	0	0	0	-0.048600252	0	0
0	0.033789341	0	-0.030997894	0	0	0	-0.046399309	0	-0.018939083
0	0.086985835	0	0	0	-0.049033684	0	0.081586014	0	0.93489111
0	0.1020427	0	-0.020760819	0	0	0	0.081509656	0	0.9144886
0	0.11141094	0	0	0	0	0	0.078519272	0	0.91122984
0	0.11157353	0	0	0	0	0	0.078070587	0	0.90800697
0	0.11186792	0	0	0	0	0	0.079342074	0	0.91106775
0	0.083647422	0	0	0	-0.045341289	0	0.080879337	0	0.93875303
0	0.096915536	0	-0.021125843	0	0	0	0.081030938	0	0.91992235
0	0.10642873	0	0	0	0	0	0.077994937	0	0.91663928
0	0.10663242	0	0	0	0	0	0.077348752	0	0.91289397
0	0.088022802	0	-0.043007372	0	0	0	0	0	1.000011
0	0.084814444	0	0	0	-0.047673051	0	0	0	1.0147288
0	0.050706801	0	0	0	-0.11511045	0	0.040436041	0	1.019107
0	0.087102126	0	0	0	-0.043535015	0	0	0	1.0070238
0	0.048925042	0	0	0	-0.11763452	0	0.038087842	0	1.0200449
0	0.048453508	0	0	0	-0.11868583	0	0.03703819	0	1.0212275
0	0.049075725	0	0.000480256	0	-0.11806827	0	0.037598392	0	1.0202101

0	0	0	0	0	-0.1105036	0	0.080785401	0	1.0232266
0	0.087341425	0	-0.003800506	0	-0.040246864	0	0	0	1.0126077
0	0.087250307	0	0	0	-0.04400472	0	0	0	1.0135529
0	0.10881831	0	0	0	0	0	0	0	0.98355536
0	0.10101273	0	-0.017255299	0	0	0	0	0	0.98812919
0	0.10922106	0	0	0	0	0	0	0	0.98926223
0	0.10157013	0	-0.016972081	0	0	0	0	0	0.99469272
0	0.083510934	0	-0.042036474	0	0	0	0	0	1.0046639
0	0	0	0	0	-0.10785942	0	0.080548945	0	1.0205886
0	0	0	-0.083207156	0	0	0	0.081620923	0	1.0000287
0	0	0	-0.00521445	0	-0.10325035	0	0.082098397	0	1.0222936
0	0	0	0	0	-0.108457	0	0.081980627	0	1.0238508
0	0.10927466	0	0	0	0	0	0	0	0.983545
0	0.057338327	0	-0.00896722	0	-0.091141955	0	0	0	1.0442767
0	0.059597902	0	0	0	-0.09532798	0	0	0	1.0441789
0	0.059693807	0	0	0	-0.095319349	0	0	0	1.0441463
0	0.062897488	0	0	0	-0.08938882	0	0	0	1.0403271
0	0.06345964	0	-0.000321561	0	-0.088121768	0	0	0	1.040374
0	0.062916199	0	-0.000194289	0	-0.089332127	0	0	0	1.0402092
0	0.081859665	0	0	0	-0.043471205	0	0	0	1.0174801
0	0.097202919	0	-0.045792078	0	0	0	0.093860832	0	0
0	0	0	0	0	-0.10477015	0	0.080291115	0	1.0237323
0	0.083956141	0	0	0	-0.039871265	0	0	0	1.0093111
0	0	0	-0.01214774	0	-0.1312049	0	0.047557178	0	1.0561286
0	0	0	0	0	-0.13933222	0	0.050785147	0	1.0561411
0	0	0	0	0	-0.13883404	0	0.051538684	0	1.0566457
0	0.046382328	0	0	0	-0.11260984	0	0.038971689	0	1.0243278
0	0	0	-0.001336814	0	-0.13332427	0	0.055209419	0	1.0518682
0	0	0	0	0	-0.13424374	0	0.055698171	0	1.0521309
0	0.044968063	0	-0.001986389	0	-0.1129943	0	0.036634205	0	1.0248022
0	0.045134479	0	0	0	-0.11455031	0	0.036752806	0	1.0249657
0	0.084273681	0	-0.006076831	0	-0.034313009	0	0	0	1.0149093
0	0	0	-0.001109129	0	-0.13327862	0	0.055244505	0	1.0521218
0	0.096075336	0	-0.017590805	0	0	0	0	0	0.99226468
0	0	0	-0.080879689	0	0	0	0.081302921	0	1.0010313
0	0.10399529	0	0	0	0	0	0	0	0.98760543
0	0	0	0	0	-0.10253979	0	0.07986503	0	1.0205622
0	0	0	-0.007518231	0	-0.095752283	0	0.081734916	0	1.0220889
0	0.097135927	0	0	0	-0.044385942	0	0.092501928	0	0
0	0.099134717	0	0	0	-0.040727932	0	0.091773692	0	0
0	0.099515859	0	-0.009409335	0	-0.031862385	0	0.093791327	0	0
0	0.053665748	0	-0.010181431	0	-0.086798104	0	0	0	1.0477085
0	0.055948118	0	0	0	-0.092210069	0	0	0	1.0473512
0	0.099209423	0	0	0	-0.041241214	0	0.093313334	0	0
0	0.058220987	0	-0.002477564	0	-0.085926171	0	0	0	1.0440658
0	0.11016636	0	-0.019989046	0	0	0	0.091666477	0	0
0	0.088891493	0	-0.048168487	0	0	0	0.094709718	0	-0.098261117

0	0	0	-0.039088984	0	-0.069717781	0	0	0	1.090997
0	0.11057476	0	-0.019897595	0	0	0	0.093279607	0	0
0	0.11920817	0	0	0	0	0	0.088952425	0	0
0	0	0	-0.012849022	0	-0.12547304	0	0.046396564	0	1.0573354
0	0.11955902	0	0	0	0	0	0.090272014	0	0
0	0.092494798	0	-0.044804607	0	0	0	0.093353731	0	0
0	0	0	0	0	-0.13380068	0	0.049982309	0	1.0577737
0	0	0	-0.003309334	0	-0.12753182	0	0.052774985	0	1.0538516
0	0	0	-0.024145414	0	-0.10060141	0	0	0	1.0946945
0	0	0	-0.025826161	0	-0.097599365	0	0	0	1.0965223
0	0.11960459	0	0	0	0	0	0.089572092	0	0
0	0	0	0	0	-0.10524707	0	0	0	1.0954251
0	0	0	0	0	-0.11523176	0	0	0	1.0986424
0	0	0	0	0	-0.10589562	0	0	0	1.1014124
0	0.085682147	0	0	0	-0.052614462	0	0.09340516	0	-0.085312769
0	0.0878186	0	0	0	-0.048904964	0	0.092994605	0	-0.089848227
0	0.088125375	0	-0.007760643	0	-0.041607174	0	0.094669572	0	-0.088802999
0	0.094020734	0	0	0	-0.040094786	0	0.091840776	0	0
0	0.087802759	0	0	0	-0.049406784	0	0.094275785	0	-0.087002209
0	0	0	-0.078373855	0	0	0	0	0	1.0743557
0	0	0	-0.002755209	0	-0.10024233	0	0	0	1.0955189
0	0	0	0	0	-0.10295109	0	0	0	1.0961943
0	0	0	-0.002645491	0	-0.10028059	0	0	0	1.0954145
0	0	0	-0.040390575	0	-0.062985846	0	0	0	1.0905985
0	0.095834519	0	0	0	-0.036912569	0	0.090898621	0	0
0	0.10498358	0	-0.020366497	0	0	0	0.091067314	0	0
0	0.096300709	0	-0.011783519	0	-0.02576608	0	0.093252542	0	0
0	0	0	-0.078911664	0	0	0	0	0	1.0801581
0	0	0	-0.002122323	0	-0.10147372	0	0	0	1.102608
0	0.10242886	0	-0.021497567	0	0	0	0.092845001	0	-0.10882998
0	0.084208404	0	-0.04720511	0	0	0	0.094145946	0	-0.092905227
0	0	0	-0.049167652	0	0	0	0.078981352	0	1.00845
0	0.10273604	0	-0.021437298	0	0	0	0.094221181	0	-0.10597221
0	0.11417519	0	0	0	0	0	0.088301255	0	0
0	0	0	-0.024104255	0	-0.096574375	0	0	0	1.094538
0	0	0	-0.049314776	0	0	0	0.080795145	0	1.0127414
0	0.11220869	0	0	0	0	0	0.090037142	0	-0.11436279
0	0.11248227	0	0	0	0	0	0.091121662	0	-0.11194701
0	0	0	0	0	-0.099641998	0	0	0	1.094695
0	0.1126095	0	0	0	0	0	0.090671128	0	-0.11478995
0	0	0	-0.076069825	0	0	0	0	0	1.0743801
0	0	0	-0.048519424	0	0	0	0.078712405	0	1.0084809
0	0.08253286	0	0	0	-0.048440778	0	0.092727545	0	-0.081669202
0	0	0	-0.004895049	0	-0.092959149	0	0	0	1.0941683
0	0.08449164	0	0	0	-0.045198537	0	0.092105901	0	-0.086542006
0	0.084886877	0	-0.010107886	0	-0.03563969	0	0.094113026	0	-0.085579141
0	0.097301926	0	-0.021882315	0	0	0	0.092204597	0	-0.10378608

0	0.02557827	0	-0.028886274	0	-0.068650507	0	0.092537938	0	0
0	0.097610011	0	-0.040206404	0	0	0	0	0	0
0	0.10723367	0	0	0	0	0	0.089336908	0	-0.10937949
0	0.097856307	0	-0.04069741	0	0	0	0	0	0
0	0.097082863	0	-0.039670276	0	-0.002413874	0	0	0	0
0	0.029664239	0	0	0	-0.092474399	0	0.091733635	0	0
0	0.029963433	0	0	0	-0.092261625	0	0.091782192	0	0
0	0.079134535	0	-0.057001965	0	0	0	-0.010278278	0	1.0363669
0	0	0	-0.04701637	0	-0.067714437	0	0.095321264	0	0
0	0.076925286	0	-0.061322773	0	0	0	0	0	1.0305074
0	0.074144121	0	-0.067030131	0	0	0	0	0	1.032704
0	0	0	-0.044011711	0	-0.070809658	0	0.095044074	0	-0.01193259
0	0.098304671	0	0	0	-0.037499934	0	0	0	0
0	0	0	-0.038766218	0	-0.075963157	0	0.093666491	0	0
0	0	0	-0.03936909	0	-0.075668216	0	0.094402077	0	0
0	0.030891613	0	0.000728553	0	-0.090392221	0	0.092603055	0	0
0	0.030726991	0	0	0	-0.08978782	0	0.092588988	0	0
0	0.030931948	0	0.000851612	0	-0.090372537	0	0.092409237	0	0
0	0.098533694	0	0	0	-0.038037849	0	0	0	0
0	0.075591444	0	-0.061173423	0	0	0	-0.022286869	0	1.0407121
0	0.074043067	0	-0.064359529	0	0	0	-0.024596588	0	1.0432775
0	0.1006678	0	-0.006706266	0	-0.027423772	0	0	0	0
0	0.10054114	0	0	0	-0.034037344	0	0	0	0
0	0.11731994	0	0	0	0	0	0	0	0
0	0.030573154	0	-0.06423111	0	0	0	0.091596066	0	0
0	0.024559032	0	-0.030764466	0	-0.061813365	0	0.092145235	0	0
0	0.029632776	0	-0.064326978	0	0	0	0.091004261	0	0
0	0.057457532	0	0	0	-0.11858878	0	0.046114815	0	0
0	0.11024984	0	-0.015692153	0	0	0	0	0	0
0	0.057589621	0	-0.000914571	0	-0.11774247	0	0.045700618	0	0
0	0.057749767	0	0	0	-0.11831881	0	0.045819065	0	0
0	0	0	0	0	-0.11053676	0	0.092465641	0	0
0	0.056265558	0	0	0	-0.12030573	0	0.043620031	0	0
0	0.055622643	0	-0.001098289	0	-0.12039225	0	0.043062363	0	0
0	0.056492564	0	0.000526802	0	-0.12065948	0	0.043179123	0	0
0	0.10097996	0	-0.005962778	0	-0.028612808	0	0	0	0
0	0	0	0	0	-0.11120941	0	0.093906831	0	0
0	0	0	0	0	0	0	0.072131253	0	1.0250722
0	0	0	0	0	-0.11097097	0	0.092476819	0	-0.005431233
0	0.09311661	0	-0.039160554	0	0	0	0	0	0
0	0	0	0	0	-0.11136877	0	0.092517501	0	0
0	0.088518984	0	-0.042825359	0	0	0	0	0	-0.097924333
0	0	0	0	0	-0.11149627	0	0.093637373	0	-0.003062933
0	0	0	0	0	0	0	0.073590345	0	1.0288725
0	0.074281074	0	-0.057190265	0	0	0	-0.009647651	0	1.039865
0	0.070285959	0	-0.071445888	0	0	0	0	0	1.0246399
0	0	0	-0.085153505	0	0	0	0.093219856	0	0

0	0.084710786	0	-0.037188853	0	-0.013541059	0	0	0	-0.088862213
0	0.069936178	0	-0.065851181	0	0	0	0	0	1.0361609
0	0.088674672	0	-0.043270911	0	0	0	0	0	-0.094037964
0	0	0	-0.085681885	0	0	0	0.09464829	0	0
0	0.028822028	0	0	0	-0.087025846	0	0.091044108	0	0
0	0	0	-0.045516249	0	0	0	0	0	1.0835161
0	0	0	-0.04834125	0	-0.060871012	0	0.094894874	0	0
0	0.094147131	0	-0.041185481	0	0.00317756	0	0	0	0
0	0	0	0	0	0	0	0.072928266	0	1.0249576
0	0	0	-0.008085893	0	-0.10044165	0	0.093618589	0	0
0	0	0	-0.045334261	0	-0.063976957	0	0.09461437	0	-0.011634834
0	0	0	0	0	-0.10845001	0	0.093383798	0	0
0	0	0	-0.084035886	0	0	0	0.09331003	0	-0.023184852
0	0.047917088	0	0	0	-0.12198046	0	0.05009854	0	-0.042386522
0	0.047625316	0	0	0	-0.12273289	0	0.049319653	0	-0.041947688
0	0.048103428	0	0.000356923	0	-0.12223871	0	0.0496998	0	-0.042420193
0	0	0	-0.039963856	0	-0.069214074	0	0.093040814	0	0
0	0	0	-0.084469569	0	0	0	0.094446745	0	-0.020963577
0	0	0	-0.007818918	0	-0.10142627	0	0.095423894	0	0
0	0.08582998	0	0	0	-0.046495297	0	0	0	-0.086737281
0	0	0	-0.006359415	0	-0.10255249	0	0.093559089	0	-0.006862174
0	0	0	0	0	-0.10887918	0	0.093396511	0	-0.005619209
0	0.045992412	0	0	0	-0.12494139	0	0.046857006	0	-0.043063473
0	0.046006035	0	0.000174966	0	-0.12507526	0	0.046869825	0	-0.04304398
0	0	0	-0.005046138	0	-0.10432803	0	0.093546074	0	0
0	0.046210961	0	0.000515792	0	-0.12530748	0	0.046410303	0	-0.043160388
0	0	0	0	0	0	0	0.071901264	0	1.0248695
0	0.0699649	0	-0.063460991	0	0	0	-0.022523023	0	1.0457308
0	0	0	-0.00615712	0	-0.1033693	0	0.095042762	0	-0.00382454
0	0.029422792	0	-0.001724713	0	-0.083316449	0	0.091809808	0	0
0	0.095382264	0	0	0	-0.033233674	0	0	0	0
0	0.085970128	0	0	0	-0.046977854	0	0	0	-0.082621464
0	0.028928588	0	-0.062725273	0	0	0	0.091096174	0	0
0	0	0	-0.08066777	0	0	0	0.092578423	0	0
0	0.088260721	0	-0.004789288	0	-0.038176645	0	0	0	-0.090154489
0	0.097654872	0	-0.009017092	0	-0.021400119	0	0	0	0
0	0.088142919	0	0	0	-0.042922668	0	0	0	-0.089081216
0	0.067416463	0	-0.008780837	0	-0.088857323	0	0	0	0
0	0.06753332	0	-0.008693838	0	-0.08889526	0	0	0	0
0	0	0	0	0	-0.10477651	0	0.091778902	0	0
0	0	0	-0.082783863	0	0	0	0.092741578	0	0
0	0.069429336	0	0	0	-0.093461305	0	0	0	0
0	0.022470719	0	0	0	-0.13294083	0	0.057175748	0	0
0	0.071534873	0	-0.001781271	0	-0.087952517	0	0	0	0
0	0.022282848	0	-0.001743847	0	-0.13187119	0	0.056500201	0	0
0	0.023571853	0	0.003472504	0	-0.13285756	0	0.059205229	0	0
0	0.088426038	0	-0.004123557	0	-0.039327248	0	0	0	-0.085354631

0	0	0	0	0	-0.10521838	0	0.091786889	0	-0.00535184
0	0.01976316	0	-5.45E-05	0	-0.13596895	0	0.053868626	0	0
0	0.10960227	0	0	0	0	0	0	0	-0.11065556
0	0.10173154	0	-0.017296033	0	0	0	0	0	-0.10619598
0	0.084075441	0	-0.041816922	0	0	0	0	0	-0.093279639
0	0.053121389	0	-0.003432382	0	-0.11302335	0	0.044192077	0	0
0	0.054104123	0	0	0	-0.11452433	0	0.044943519	0	0
0	0	0	-0.081676225	0	0	0	0.092818577	0	-0.022548841
0	0.052843475	0	-0.001006024	0	-0.11555674	0	0.042668994	0	0
0	0	0	-0.010416345	0	-0.092941868	0	0.093077171	0	0
0	0.081761167	0	-0.038679086	0	-0.008081941	0	0	0	-0.085756481
0	0	0	-0.008692234	0	-0.095037952	0	0.093017302	0	-0.007250337
0	0	0	-0.012605497	0	-0.13604126	0	0.056594129	0	0
0	0	0	-0.013202781	0	-0.13500786	0	0.05747606	0	0
0	0	0	-0.011340039	0	-0.1358885	0	0.059022463	0	0
0	0	0	0	0	-0.14412177	0	0.060361848	0	0
0	0	0	-0.010287238	0	-0.13760583	0	0.057130927	0	-0.00185744
0	0	0	-0.011220541	0	-0.13605615	0	0.058537674	0	-0.000313356
0	0	0	0	0	-0.14378479	0	0.061038229	0	0.001281562
0	0	0	-0.002985019	0	-0.13786464	0	0.063203939	0	0
0	0	0	-0.001360086	0	-0.13880583	0	0.064066116	0	0.000380106
0	0.10628152	0	0	0	0	0	-0.025662003	0	1.0397334
0	0.082901105	0	0	0	-0.042325968	0	0	0	-0.083818463
0	0.057696432	0	-0.009484078	0	-0.091208535	0	0	0	-0.055503623
0	0.057868701	0	-0.009469609	0	-0.091082846	0	0	0	-0.055448273
0	0.041421071	0	-0.032778367	0	0	0	0.090303061	0	0
0	0.05993935	0	0	0	-0.096071079	0	0	0	-0.056033478
0	0.043666021	0	-0.002120813	0	-0.11758612	0	0.0482114	0	-0.038384405
0	0.043996782	0	0	0	-0.11898608	0	0.048447113	0	-0.03835295
0	0.063064612	0	-0.000541896	0	-0.089930502	0	0	0	-0.059677437
0	0.042584775	0	-0.001009405	0	-0.12024735	0	0.04589759	0	-0.039344988
0	0.08524196	0	-0.007077031	0	-0.032262462	0	0	0	-0.087604968
0	0	0	-0.095462807	0	0	0	0	0	1.0977342
0	0	0	-0.09405121	0	0	0	-0.007334479	0	1.1032165
0	0	0	0	0	0	0	0	0	1.092771
0	0.10649752	0	0	0	0	0	0	0	1.0216563
0	0.10492017	0	0	0	0	0	-0.037319812	0	1.0417725
0	0.020640597	0	-0.002896182	0	-0.12669889	0	0.05491566	0	0
0	0.063574176	0	-0.009602826	0	-0.08525255	0	0	0	0
0	0.027249663	0	-0.025217177	0	-0.065343418	0	0	0	0
0	0.028037972	0	-0.025511507	0	-0.064722321	0	0	0	0
0	0	0	-0.0939518	0	0	0	-0.017342428	0	1.1035716
0	0.052029089	0	0	0	0	0	0.085904217	0	0
0	0.030689505	0	0	0	-0.086200014	0	0	0	0
0	0.10177872	0	0	0	0	0	-0.024611223	0	1.042403
0	0	0	-0.013637962	0	-0.12964307	0	0.055878452	0	0
0	0	0	-0.011650261	0	-0.130704	0	0.056932762	0	-0.000119074

0	0	0	-0.091827027	0	0	0	-0.006481886	0	1.102399
0	0	0	-0.074918109	0	0	0	0.002364876	0	1.0966902
0	0.025049731	0	-0.012211289	0	-0.098133573	0	0	0	0
0	0	0	-0.042882769	0	-0.065289483	0	0	0	0
0	0.10423887	0	0	0	0	0	-0.046424697	0	1.0658235
0	0.053946529	0	-0.010365281	0	-0.087508669	0	0	0	-0.051629765
0	0	0	-0.04335091	0	-0.065632642	0	0	0	0
0	0	0	-0.03969297	0	-0.06870346	0	0	0	-0.012160654
0	0.032037439	0	0.003939929	0	-0.086918343	0	0	0	0
0	0	0	-0.035321631	0	-0.07347019	0	0	0	0
0	0	0	-0.040133628	0	-0.068911165	0	0	0	-0.008140457
0	0	0	-0.050988696	0	0	0	0.091856346	0	0
0	0	0	-0.026362522	0	-0.10069219	0	0	0	0
0	0.031622865	0	-0.058936054	0	0	0	0	0	0
0	0.025968975	0	-0.026908132	0	-0.058913062	0	0	0	0
0	0	0	-0.024535899	0	-0.10114894	0	0	0	-0.005811675
0	0	0	-0.05017436	0	0	0	0.091887964	0	-0.017838332
0	0	0	0	0	-0.1049461	0	0	0	0
0	0	0	0	0	-0.10541425	0	0	0	-0.004373279
0	0	0	-0.080145823	0	0	0	0	0	0
0	0	0	-0.044113536	0	-0.058680847	0	0	0	0
0	0	0	-0.040927681	0	-0.062084973	0	0	0	-0.012324188
0	0	0	-0.004995781	0	-0.097700027	0	0	0	0
0	0	0	-0.078906452	0	0	0	0	0	-0.021074931
0	0	0	-0.003113205	0	-0.10000735	0	0	0	-0.005156184
0	0.086419571	0	-0.059694359	0	0	0	-0.005373317	0	0
0	0.084432525	0	-0.063799557	0	0	0	-0.0073328	0	0
0	0.083439753	0	-0.065586913	0	0	0	0	0	0
0	0.082791042	0	-0.064309767	0	0	0	-0.018709348	0	0
0	0	0	0	0	0	0	0.084888482	0	0
0	0	0	0	0	0	0	0.084916049	0	-0.00944499
0	0.07427872	0	-0.067751317	0	0	0	0	0	-0.073049621
0	0.075058958	0	-0.066384746	0	0	0	-0.005567709	0	-0.073940402
0	0.077854826	0	-0.06079386	0	0	0	-0.003095629	0	-0.07611243
0	0.080095162	0	-0.062825928	0	0	0	-0.006288477	0	0
0	0.073298387	0	-0.066935315	0	0	0	-0.017653534	0	-0.080114921
0	0.070788214	0	-0.065439509	0	0	0	-0.004544296	0	-0.069784987
0	0.11505341	0	0	0	0	0	-0.01992665	0	0
0	0.08008416	0	-0.021119809	0	0.057295068	0	0.053095069	0	0.93509926
0	0.079281579	0	0	0	0.037548212	0	0.049538445	0	0.93642703
0	0.031783471	0	-0.077636376	0	0	0	0.000560158	0	0
0	0.078105189	0	0	0	0.036008113	0	0.050886864	0	0.94290246
0	0.077015599	0	-0.049720632	0	0.079179086	0	0	0	0.98167467
0	0.056096669	0	0	0	0	0	0.048870329	0	0.95875355
0	0.049490337	0	-0.012032804	0	0	0	0.051121864	0	0.9666335
0	0.05600356	0	-0.001024447	0	0	0	0.049483759	0	0.95843791
0	0.08038216	0	-0.019198701	0	0.055487509	0	0	0	0.97868849

0	0	0	-0.097710888	0	0	0	-0.000281853	0	0
0	0.10694961	0	0	0	0	0	-0.018578033	0	-0.091634886
0	0.078452228	0	0	0	0.036001148	0	0	0	0.9844892
0	0	0	-0.09633901	0	0	0	0.000410744	0	-0.00971086
0	0	0	0	0	0	0	-0.026521414	0	1.1463652
0	0.050884598	0	-0.010183947	0	0	0	0	0	1.008181
0	0	0	-0.054983953	0	0.030491731	0	0.053819124	0	1.0061247
0	0	0	-0.034805971	0	0	0	0.050968634	0	1.006322
0	0	0	-0.019836301	0	0.000360136	0	0.051655171	0	1.0115008
0	0	0	0	0	-0.018511438	0	0.049534501	0	1.0173642
0	0	0	-0.051581206	0	0.02714992	0	0	0	1.0522058
0	0.088710155	0	-0.056154462	0	0.093224543	0	0.064979512	0	0
0	0.092259719	0	-0.024154235	0	0.068338241	0	0.062551352	0	0
0	0.090487867	0	0	0	0.044445262	0	0.060506957	0	0
0	0.077464148	0	-0.053869917	0	0.082688819	0	0.065872946	0	-0.081029403
0	0.090008102	0	-0.052242924	0	0.090213497	0	0	0	0
0	0.058395754	0	-0.009518259	0	0	0	0.060998886	0	0
0	0.080951798	0	-0.022387526	0	0.058167305	0	0.063726139	0	-0.082866547
0	0.079058386	0	0	0	0.035798888	0	0.061665079	0	-0.076390718
0	0.050272616	0	-0.012571491	0	0	0	0.061954938	0	-0.049526722
0	0.078085057	0	-0.049830515	0	0.079004986	0	0	0	-0.084843988
0	0.024673879	0	-0.042379455	0	0.033205784	0	0.06353019	0	0
0	0	0	-0.058722759	0	0.033534395	0	0.064735198	0	0
0	0	0	-0.05579403	0	0.030322897	0	0.064747433	0	-0.011678047
0	0.015305273	0	0	0	-0.06000545	0	-0.027779665	0	1.0910004
0	0.015622736	0	0.0099304	0	-0.068490569	0	-0.02944796	0	1.0900825
0	0	0	0.004407664	0	-0.070878634	0	-0.022259284	0	1.1014
0	0.009158198	0	0.013734225	0	-0.081483689	0	0	0	1.0755039
0	0.007584854	0	-0.010078841	0	-0.061648773	0	-0.034718908	0	1.1035792
0	0.034110322	0	-0.02870777	0	0	0	-0.053446561	0	1.0977993
0	0.025151791	0	0.01025315	0	-0.067072094	0	-0.020914421	0	0
0	0.014780848	0	0.009849896	0	-0.071945861	0	-0.01686103	0	0.003587112
0	0.088314345	0	-0.047223024	0	0	0	0.082072764	0	0.91926434
0	0.084775484	0	0	0	-0.052342023	0	0.080767755	0	0.93668373
0	0.086913667	0	0	0	-0.048645276	0	0.080396324	0	0.93188723
0	0.087195524	0	-0.006959407	0	-0.042129569	0	0.08199645	0	0.93268299
0	0.086886271	0	0	0	-0.049145147	0	0.081645769	0	0.93501105
0	0.10169352	0	-0.02086474	0	0	0	0.080213718	0	0.91136635
0	0.10198599	0	-0.020811684	0	0	0	0.081556499	0	0.91454717
0	0.083637574	0	-0.046280637	0	0	0	0.081525824	0	0.92470613
0	0.11117097	0	0	0	0	0	0.077434155	0	0.90854811
0	0.11143333	0	0	0	0	0	0.078488581	0	0.91119494
0	0.11156809	0	0	0	0	0	0.078076761	0	0.9080142
0	0.081618381	0	0	0	-0.048181755	0	0.080094746	0	0.94040868
0	0.083577664	0	0	0	-0.044948444	0	0.079511699	0	0.93523811
0	0.083947104	0	-0.009311872	0	-0.036164867	0	0.081447257	0	0.93577438
0	0.096559878	0	-0.02125474	0	0	0	0.07957863	0	0.916335

0	0.10619126	0	0	0	0	0	0.076740141	0	0.91345375
0	0.087948651	0	-0.042542562	0	0	0	0	0	0.99451525
0	0.083745067	0	-0.03625076	0	-0.014889672	0	0	0	1.0062358
0	0.08807831	0	-0.042964087	0	0	0	0	0	0.99990325
0	0	0	-0.042579961	0	-0.071238015	0	0.082348381	0	1.0104796
0	0.084835242	0	0	0	-0.047051038	0	0	0	1.0088178
0	0.084915591	0	0	0	-0.047568028	0	0	0	1.0145443
0	0.050539729	0	0	0	-0.11516156	0	0.040828138	0	1.0190527
0	0.049778439	0	0	0	-0.11671615	0	0.03968236	0	1.0208976
0	0.087235429	0	-0.004396915	0	-0.039150994	0	0	0	1.0058142
0	0.050723539	0	0.000446306	0	-0.11549848	0	0.040444518	0	1.0191791
0	0.087099755	0	0	0	-0.043537924	0	0	0	1.0070239
0	0	0	0	0	-0.11008935	0	0.07981285	0	1.0206723
0	0.048335074	0	0	0	-0.11864392	0	0.037549496	0	1.0211023
0	0.048935601	0	0.00026847	0	-0.11786759	0	0.038092326	0	1.0200886
0	0.04821342	0	-0.001188481	0	-0.11798415	0	0.036839437	0	1.0213666
0	0	0	0	0	-0.11060391	0	0.080942387	0	1.0232778
0	0.087360032	0	-0.003792374	0	-0.040234532	0	0	0	1.012576
0	0.10888133	0	0	0	0	0	0	0	0.98339893
0	0.10103075	0	-0.017238614	0	0	0	0	0	0.98809714
0	0.083471598	0	-0.041546437	0	0	0	0	0	0.99849003
0	0	0	-0.04390532	0	-0.064399187	0	0.081925276	0	1.0105842
0	0	0	-0.082858834	0	0	0	0.080660522	0	0.99763048
0	0.080756893	0	-0.037749934	0	-0.00944147	0	0	0	1.0086597
0	0	0	-0.005561632	0	-0.10244876	0	0.080858093	0	1.0189416
0	0	0	0	0	-0.10800208	0	0.080735107	0	1.0206153
0	0	0	-0.083278985	0	0	0	0.081771711	0	1.0000567
0	0	0	-0.005371594	0	-0.10324314	0	0.082305362	0	1.0223129
0	0.05730688	0	-0.009182025	0	-0.09082401	0	0	0	1.0443988
0	0.057637002	0	-0.009317555	0	-0.090311122	0	0	0	1.0445224
0	0.059671397	0	0	0	-0.09520347	0	0	0	1.0442238
0	0.081867316	0	0	0	-0.042895208	0	0	0	1.0109177
0	0.062883971	0	-0.000308161	0	-0.089124257	0	0	0	1.0402737
0	0.09712016	0	-0.045395236	0	0	0	0.092659826	0	0
0	0.0956607	0	-0.043318733	0	-0.005441816	0	0.093939165	0	0
0	0	0	0	0	-0.10433504	0	0.079127893	0	1.0206314
0	0.097184624	0	-0.045806463	0	0	0	0.093874003	0	0
0	0.084174796	0	-0.006708763	0	-0.033226224	0	0	0	1.007464
0	0	0	-0.012414869	0	-0.13091827	0	0.048060106	0	1.0558909
0	0	0	-0.013130716	0	-0.12970478	0	0.049127618	0	1.0564919
0	0	0	0	0	-0.13877121	0	0.051980088	0	1.0564176
0	0.046312891	0	-0.002014032	0	-0.11086563	0	0.038942886	0	1.0239838
0	0.046163131	0	0	0	-0.11297771	0	0.038794607	0	1.0247462
0	0	0	-0.080497283	0	0	0	0.080178178	0	0.99817653
0	0	0	-0.001346682	0	-0.13305394	0	0.055660829	0	1.0518755
0	0.044596778	0	-0.002701083	0	-0.11295452	0	0.036310182	0	1.0252993
0	0	0	-0.007901625	0	-0.094919461	0	0.08032238	0	1.0182241

0	0.097092699	0	0	0	-0.043887778	0	0.091224956	0	0
0	0.097124376	0	0	0	-0.04439838	0	0.092508642	0	0
0	0.099391317	0	-0.009803247	0	-0.03101009	0	0.092276739	0	0
0	0.099055255	0	0	0	-0.04081633	0	0.091819978	0	0
0	0.099438269	0	-0.009445994	0	-0.031914149	0	0.093842297	0	0
0	0.053696214	0	-0.010233692	0	-0.086703705	0	0	0	1.0477667
0	0	0	-0.038750082	0	-0.069450485	0	0	0	1.085423
0	0.11011139	0	-0.020036458	0	0	0	0.091701026	0	0
0	0.084424685	0	-0.041159256	0	-0.015445318	0	0.094872552	0	-0.092403
0	0.088846412	0	-0.047845101	0	0	0	0.093769236	0	-0.10034707
0	0.088849459	0	-0.048191761	0	0	0	0.094694597	0	-0.098215784
0	0	0	-0.039106018	0	-0.06975178	0	0	0	1.0911038
0	0.092433615	0	-0.04435981	0	0	0	0.091957772	0	0
0	0.11923085	0	0	0	0	0	0.088924295	0	0
0	0	0	-0.013554719	0	-0.12437921	0	0.047515803	0	1.0576074
0	0.092536645	0	-0.044869613	0	0.000143958	0	0.093351976	0	0
0	0	0	-0.024346005	0	-0.10031413	0	0	0	1.0946584
0	0	0	0	0	-0.10529948	0	0	0	1.0955192
0	0.085680416	0	0	0	-0.052189283	0	0.092398073	0	-0.08767026
0	0.085644804	0	0	0	-0.05265531	0	0.093430892	0	-0.085276691
0	0.093984352	0	0	0	-0.039581034	0	0.090366403	0	0
0	0.088060222	0	-0.008104017	0	-0.040857454	0	0.093432887	0	-0.091706552
0	0.087712854	0	0	0	-0.049023526	0	0.093054297	0	-0.089758009
0	0	0	-0.039995363	0	-0.062798946	0	0	0	1.0843939
0	0.088023185	0	-0.007807738	0	-0.041676874	0	0.094734679	0	-0.088713507
0	0.096173437	0	-0.012217437	0	-0.024894294	0	0.091550726	0	0
0	0	0	-0.078408454	0	0	0	0	0	1.0744314
0	0	0	-0.002752679	0	-0.10026526	0	0	0	1.0955254
0	0.10236534	0	-0.021551902	0	0	0	0.092874188	0	-0.10877877
0	0.084192971	0	-0.046845759	0	0	0	0.093027755	0	-0.095503026
0	0.081270409	0	-0.042636098	0	-0.010108141	0	0.094255027	0	-0.089060495
0	0	0	-0.049333251	0	0	0	0.079188816	0	1.0084227
0	0.11222863	0	0	0	0	0	0.090012757	0	-0.11437601
0	0.082542081	0	0	0	-0.047980972	0	0.091524164	0	-0.084549261
0	0.084826344	0	-0.010489951	0	-0.034850175	0	0.092695344	0	-0.088970847
0	0.025502406	0	-0.028945106	0	-0.068722529	0	0.092665057	0	0
0	0.025979279	0	-0.028969817	0	-0.068446144	0	0.092745415	0	0
0	0.097675851	0	-0.040152753	0	0	0	0	0	0
0	0.096995289	0	-0.03921933	0	-0.002171161	0	0	0	0
0	0.097197808	0	-0.03965025	0	-0.002309444	0	0	0	0
0	0.029887314	0	0	0	-0.092376193	0	0.091882906	0	0
0	0.075371957	0	-0.06408653	0	0	0	-0.012618542	0	1.0412232
0	0	0	-0.04676752	0	-0.067422328	0	0.094103555	0	0
0	0.078879053	0	-0.057172314	0	0	0	-0.009730496	0	1.0361303
0	0	0	-0.047083908	0	-0.067773654	0	0.095495499	0	0
0	0.073897558	0	-0.067103931	0	0	0	0	0	1.0329963
0	0	0	-0.043781355	0	-0.070626401	0	0.094124598	0	-0.014054299

0	0	0	-0.044050812	0	-0.070876946	0	0.09520756	0	-0.01193676
0	0.098397516	0	0	0	-0.037404144	0	0	0	0
0	0	0	-0.03878904	0	-0.076038699	0	0.093822325	0	0
0	0.030810012	0	0.000723951	0	-0.090427229	0	0.092548167	0	0
0	0.073739301	0	-0.06458726	0	0	0	-0.023781133	0	1.0431115
0	0.10070796	0	-0.006687779	0	-0.027398423	0	0	0	0
0	0.030499202	0	-0.064325886	0	0	0	0.09168925	0	0
0	0.024615007	0	-0.03078522	0	-0.061767196	0	0.092196164	0	0
0	0.05741078	0	-0.00111774	0	-0.11761854	0	0.04609583	0	0
0	0.05760368	0	0	0	-0.11832893	0	0.046235079	0	0
0	0.057574048	0	-0.000949711	0	-0.1177371	0	0.045687223	0	0
0	0	0	0	0	-0.11063859	0	0.092617914	0	0
0	0.056315	0	0.000250078	0	-0.1204541	0	0.043660863	0	0
0	0	0	0	0	0	0	0.07221199	0	1.0250855
0	0.084712136	0	-0.036812429	0	-0.013255046	0	0	0	-0.092900066
0	0	0	0	0	-0.11106686	0	0.092627444	0	-0.005458454
0	0.094069409	0	-0.040670441	0	0.003319494	0	0	0	0
0	0.088576253	0	-0.042782083	0	0	0	0	0	-0.09796209
0	0.071081384	0	-0.063129567	0	0	0	-0.011606105	0	1.0439714
0	0	0	-0.085224637	0	0	0	0.093366365	0	0
0	0	0	-0.047955898	0	-0.060715356	0	0.093478575	0	0
0	0.08480244	0	-0.03717373	0	-0.013457729	0	0	0	-0.088950246
0	0	0	-0.045034761	0	-0.063842601	0	0.093501278	0	-0.014219394
0	0	0	-0.008256138	0	-0.10042368	0	0.093820627	0	0
0	0	0	-0.084105496	0	0	0	0.093453861	0	-0.023224733
0	0.047477766	0	0	0	-0.12274206	0	0.049742367	0	-0.041888598
0	0.047920273	0	0.000150119	0	-0.12211381	0	0.050094457	0	-0.042369657
0	0.047417504	0	-0.001115885	0	-0.12204988	0	0.049165101	0	-0.041926041
0	0	0	-0.006526041	0	-0.10254216	0	0.09375349	0	-0.006928203
0	0.085906388	0	0	0	-0.046415696	0	0	0	-0.086802884
0	0.04603673	0	0.000234228	0	-0.12507831	0	0.046896532	0	-0.043062885
0	0.088279374	0	-0.004780895	0	-0.038164815	0	0	0	-0.090168954
0	0.067477497	0	-0.008847292	0	-0.088693919	0	0	0	0
0	0.022122661	0	-0.001979178	0	-0.13172175	0	0.056899404	0	0
0	0.081779211	0	-0.038242298	0	-0.007874879	0	0	0	-0.090260563
0	0.053701205	0	-0.002182111	0	-0.11319039	0	0.04464609	0	0
0	0	0	-0.013432266	0	-0.13477347	0	0.057920017	0	0
0	0	0	-0.011447934	0	-0.13583031	0	0.058967779	0	-0.000438178
0	0.10614951	0	0	0	0	0	-0.025275734	0	1.0396433
0	0.057812675	0	-0.009623105	0	-0.0908858	0	0	0	-0.055471651
0	0.043560997	0	-0.00234455	0	-0.11755631	0	0.048130007	0	-0.038308521
0	0	0	-0.094160227	0	0	0	-0.006630423	0	1.1027593
0	0.027212378	0	-0.025309816	0	-0.065300833	0	0	0	0
0	0	0	-0.042900336	0	-0.065325348	0	0	0	0
0	0	0	-0.039694956	0	-0.068745636	0	0	0	-0.012172942
0	0.084158776	0	-0.063995488	0	0	0	-0.006771596	0	0
0	0.074780052	0	-0.066584474	0	0	0	-0.004992412	0	-0.073816163

0	0.076598061	0	-0.052533839	0	0.08203877	0	0.053256733	0	0.92871288
0	0.080015535	0	-0.021637682	0	0.057974517	0	0.051110966	0	0.92930901
0	0.078143978	0	0	0	0.036311789	0	0.049074478	0	0.93760395
0	0.077058094	0	-0.049150109	0	0.079039863	0	0	0	0.97371372
0	0.049596941	0	-0.011569099	0	0	0	0.049342764	0	0.96164824
0	0	0	-0.054429929	0	0.030256154	0	0.052096816	0	1.0013842
0	0.088696711	0	-0.055491143	0	0.092919515	0	0.062938416	0	0
0	0.077516673	0	-0.053295617	0	0.082474961	0	0.064107345	0	-0.085505909
0	0.016892085	0	0.008222449	0	-0.065140059	0	-0.026519856	0	1.0898802
0	0.083564388	0	-0.039724085	0	-0.016539643	0	0.08221902	0	0.92603304
0	0.088270342	0	-0.046909525	0	0	0	0.081162059	0	0.91702425
0	0.088272852	0	-0.047252023	0	0	0	0.082104659	0	0.91929878
0	0.084773659	0	0	0	-0.051931386	0	0.079799826	0	0.93413854
0	0.084727625	0	0	0	-0.052394505	0	0.080798744	0	0.93674269
0	0.087131771	0	-0.007293421	0	-0.041400884	0	0.080799279	0	0.9295054
0	0.086798276	0	0	0	-0.048772248	0	0.080460021	0	0.93201288
0	0.087085055	0	-0.007010311	0	-0.042204414	0	0.08206658	0	0.93280255
0	0.10162203	0	-0.020928143	0	0	0	0.08026661	0	0.91142138
0	0.083613594	0	-0.045914452	0	0	0	0.08042486	0	0.92193411
0	0.080400934	0	-0.041246082	0	-0.011098882	0	0.081625501	0	0.92930484
0	0.11118665	0	0	0	0	0	0.07741505	0	0.90852849
0	0.081628749	0	0	0	-0.047738724	0	0.078928703	0	0.93728268
0	0.083887737	0	-0.009684865	0	-0.035394301	0	0.080068345	0	0.9320574
0	0.083747296	0	-0.035908226	0	-0.0146257	0	0	0	1.0006601
0	0.087986124	0	-0.042514301	0	0	0	0	0	0.99445253
0	0.083806547	0	-0.036240593	0	-0.014833855	0	0	0	1.0061235
0	0	0	-0.042361468	0	-0.071048757	0	0.081419974	0	1.008173
0	0	0	-0.042620507	0	-0.071306397	0	0.082516022	0	1.0105218
0	0.084883412	0	0	0	-0.047000874	0	0	0	1.0087379
0	0.049636545	0	0	0	-0.11672637	0	0.040088948	0	1.0207991
0	0.050549361	0	0.000248935	0	-0.11537788	0	0.04083222	0	1.0190936
0	0.049287706	0	-0.002644043	0	-0.1150947	0	0.03931455	0	1.0211979
0	0.087225687	0	-0.004401294	0	-0.039157163	0	0	0	1.005829
0	0	0	0	0	-0.11019012	0	0.079976943	0	1.0206596
0	0.048042679	0	-0.001459175	0	-0.11777944	0	0.037309888	0	1.0212847
0	0.080774206	0	-0.037349137	0	-0.00924717	0	0	0	1.0024169
0	0	0	-0.043611762	0	-0.064265532	0	0.080835854	0	1.0078004
0	0	0	-0.005723263	0	-0.10242957	0	0.081062089	0	1.0189776
0	0	0	-0.082930692	0	0	0	0.080807524	0	0.99762012
0	0.05757436	0	-0.009489063	0	-0.090090608	0	0	0	1.0446158
0	0.095631411	0	-0.043008878	0	-0.005257819	0	0.092737021	0	0
0	0.09709005	0	-0.045419518	0	0	0	0.092684665	0	0
0	0.095633846	0	-0.043328436	0	-0.005463592	0	0.093953086	0	0
0	0	0	-0.01334969	0	-0.12948708	0	0.04954855	0	1.0562417
0	0.045446346	0	-0.003862977	0	-0.1106158	0	0.038265073	0	1.0252119
0	0.097063402	0	0	0	-0.043919558	0	0.091243197	0	0
0	0.099289979	0	-0.009855572	0	-0.031074047	0	0.092334596	0	0

0	0	0	-0.038768457	0	-0.069489177	0	0	0	1.0855186
0	0.08442987	0	-0.040873736	0	-0.015383838	0	0.093922304	0	-0.094520514
0	0.084369875	0	-0.041126232	0	-0.015607046	0	0.094891321	0	-0.092341494
0	0.088809494	0	-0.047873477	0	0	0	0.093800577	0	-0.10032475
0	0.092513745	0	-0.044486362	0	0.000278635	0	0.091953665	0	0
0	0.085629017	0	0	0	-0.052233646	0	0.092414775	0	-0.087615463
0	0.087940973	0	-0.008160429	0	-0.040934665	0	0.093503224	0	-0.09161536
0	0.08128885	0	-0.042328778	0	-0.009961156	0	0.093126153	0	-0.091686702
0	0.025891167	0	-0.02909288	0	-0.068422172	0	0.092942349	0	0
0	0.097078179	0	-0.039206162	0	-0.002094038	0	0	0	0
0	0.075104347	0	-0.064279507	0	0	0	-0.012066461	0	1.0411034
0	0	0	-0.046694006	0	-0.067696722	0	0.094295597	0	0
0	0	0	-0.043810112	0	-0.070706165	0	0.094270573	0	-0.014085586
0	0.057385836	0	-0.001172288	0	-0.11761121	0	0.046075017	0	0
0	0.084773702	0	-0.036801873	0	-0.0132009	0	0	0	-0.092949459
0	0.047224482	0	-0.001341793	0	-0.12192885	0	0.049559786	0	-0.041857833
0	0.076648756	0	-0.051969793	0	0.081830211	0	0.051521512	0	0.92387779
0	0.083569036	0	-0.039490644	0	-0.016372168	0	0.081318358	0	0.92375145
0	0.08350188	0	-0.039733068	0	-0.016593795	0	0.08225969	0	0.92610295
0	0.088217257	0	-0.046970359	0	0	0	0.08122286	0	0.91722107
0	0.084712883	0	0	0	-0.051992114	0	0.079841913	0	0.93417006
0	0.087004033	0	-0.007362764	0	-0.04147731	0	0.080871443	0	0.92964762
0	0.080418396	0	-0.040947499	0	-0.010955253	0	0.080528469	0	0.92647765
0	0.08380272	0	-0.035954011	0	-0.0145536	0	0	0	1.0006501
0	0	0	-0.042383505	0	-0.071134793	0	0.081608072	0	1.0082056
0	0.049099373	0	-0.002879386	0	-0.11496202	0	0.039691674	0	1.0211337
0	0.09556809	0	-0.043020836	0	-0.005304923	0	0.09274072	0	0
0	0.084361834	0	-0.040885841	0	-0.015445529	0	0.093969555	0	-0.094464438
0	0.083493963	0	-0.039505037	0	-0.016440585	0	0.081364191	0	0.92383133

SE	bsnp_rs6469674	SE	bsnp_rs13269857	SE	bsnp_rs3020106	SE	bsnp_rs3020119	SE
0	0	0	0	0	0	0	0	0
0	0	0	0	0	0	0	0	0
0	0	0	0	0	0	0	0	0
0	0	0	-0.14666113	0	0	0	0	0
0	0	0	0	0	-0.14794332	0	0	0
0	0	0	0	0	0	0	0	0
0	0	0	0	0	0	0	0	0
0	0	0	0	0	0	0	0	0
0	0	0	0	0	0	0	0.082414687	0
0	0	0	0	0	-0.12869846	0	0	0
0	0	0	-0.12470181	0	0	0	0	0
0	0	0	0	0	-0.093215419	0	0	0
0	0	0	-0.090398937	0	0	0	0	0
0	0	0	0	0	-0.13024136	0	0	0
0	0	0	-0.12753785	0	0	0	0	0
0	0	0	0	0	0	0	0	0
0	0	0	0	0	0	0	0	0
0	0	0	0	0	-0.11239705	0	0	0
0	0	0	-0.11089541	0	0	0	0	0
0	0	0	0	0	0	0	0	0
0	0	0	0	0	0	0	-0.018816843	0
0	0	0	0	0	0	0	0	0
0	0	0	0	0	0	0	0	0
0	0	0	0	0	0	0	0	0
0	0	0	0	0	-0.10797594	0	0	0
0	-0.002678457	0	0	0	0	0	0	0
0	0	0	-0.10624524	0	0	0	0	0
0	0	0	0	0	0	0	0	0
0	0	0	0	0	0	0	0	0
0	0.013604315	0	0	0	0	0	0	0
0	0	0	0	0	-0.12376497	0	0.057001226	0
0	0	0	-0.12184371	0	0	0	0.055289062	0
0	0	0	0	0	0	0	0	0
0	0	0	0	0	0	0	0	0
0	0	0	-0.15782846	0	0	0	0	0
0	0	0	0	0	-0.16003276	0	0	0
0	0	0	0	0	0	0	0.019709677	0
0	0	0	0	0	0	0	0	0
0	0	0	0	0	0	0	0	0
0	0.007407883	0	0	0	0	0	0	0
0	0	0	0	0	0	0	0	0
0	0	0	0	0	0	0	0	0
0	0	0	-0.083991912	0	-0.065082552	0	0	0
0	-0.004478711	0	-0.1469965	0	0	0	0	0
0	0	0	-0.14640071	0	0	0	0	0

0	-0.004648273	0	0	0	-0.14830015	0	0	0
0	0	0	0	0	-0.14768904	0	0	0
0	0	0	0	0	0	0	0.042499054	0
0	0	0	0	0	0	0	0	0
0	0	0	0	0	0	0	0	0
0	0	0	0	0	0	0	0	0
0	0	0	0	0	0	0	0.026291557	0
0	0	0	0	0	0	0	0.033685158	0
0	0	0	0	0	0	0	0	0
0	0	0	0	0	0	0	0	0
0	0.003362263	0	0	0	0	0	0	0
0	0	0	0	0	0	0	0	0
0	-0.009112157	0	0	0	0	0	0	0
0	0	0	0	0	0	0	0	0
0	0	0	0	0	0	0	0	0
0	0.003963332	0	0	0	0	0	0	0
0	0	0	0	0	0	0	0.089314823	0
0	0	0	0	0	0	0	0.085250146	0
0	0.032816456	0	0	0	0	0	0.084679679	0
0	0	0	0	0	-0.15798006	0	0	0
0	0	0	-0.15287181	0	0	0	0	0
0	0	0	0	0	-0.13709285	0	0	0
0	0	0	0	0	-0.13899289	0	0	0
0	0	0	0	0	-0.13266839	0	-0.013478319	0
0	0	0	-0.13243941	0	0	0	0	0
0	-0.007856658	0	0	0	-0.12928721	0	0	0
0	0	0	0	0	-0.13339701	0	0	0
0	0	0	0	0	-0.12856629	0	0	0
0	0	0	-0.006116926	0	-0.12269001	0	0	0
0	0	0	0	0	-0.12869903	0	0	0
0	0	0	-0.13355278	0	0	0	0	0
0	0	0	-0.1287528	0	0	0	-0.013542493	0
0	-0.00723369	0	-0.12522983	0	0	0	0	0
0	0	0	-0.12770768	0	0	0	0	0
0	0	0	-0.12452945	0	0	0	0	0
0	0	0	-0.12465789	0	0	0	0	0
0	0	0	0	0	-0.10546123	0	0	0
0	0	0	0	0	-0.10091758	0	0	0
0	0	0	-0.10162573	0	0	0	0	0
0	0	0	-0.098241507	0	0	0	0	0
0	0	0	0	0	-0.10548593	0	0	0
0	0	0	-0.10250936	0	0	0	0	0
0	0	0	0	0	-0.094129474	0	0	0
0	0	0	0	0	-0.13760787	0	0	0
0	0	0	0	0	-0.092552484	0	-0.014806199	0
0	-0.010526685	0	0	0	-0.093876932	0	0	0
0	0	0	0	0	-0.093378547	0	0	0

0	0	0	-0.002301921	0	-0.09098212	0	0	0
0	0	0	-0.09106355	0	0	0	0	0
0	0	0	0	0	-0.1253018	0	0.015854768	0
0	-0.022312485	0	0	0	-0.13164204	0	0	0
0	0	0	-0.08982553	0	0	0	-0.015610683	0
0	0	0	-0.13434718	0	0	0	0	0
0	0	0	0	0	-0.13728006	0	0	0
0	0	0	0	0	-0.13112465	0	0	0
0	-0.010052013	0	-0.091010232	0	0	0	0	0
0	0	0	-0.036057589	0	-0.094786709	0	0	0
0	0	0	-0.090528121	0	0	0	0	0
0	0	0	0	0	-0.12827388	0	0	0
0	-0.021612799	0	-0.12882597	0	0	0	0	0
0	0	0	-0.12276013	0	0	0	0.014633439	0
0	0	0	-0.13410601	0	0	0	0	0
0	0	0	-0.12833463	0	0	0	0	0
0	0	0	-0.12606274	0	0	0	0	0
0	-1.0725662	0	0	0	0	0	0	0
0	-1.0761811	0	0	0	0	0	0	0
0	0	0	0	0	-0.12448688	0	0	0
0	0	0	0	0	-0.13619565	0	0.065286541	0
0	0	0	-0.12198866	0	0	0	0	0
0	0	0	-0.13290865	0	0	0	0.063298416	0
0	0	0	0	0	-0.11989172	0	0	0
0	0	0	0	0	0	0	0	0
0	-0.062426881	0	0	0	0	0	0	0
0	0	0	-0.11716583	0	0	0	0	0
0	0	0	0	0	0	0	0	0
0	0	0	0	0	0	0	0	0
0	0	0	0	0	0	0	-0.015074387	0
0	0	0	0	0	0	0	0	0
0	0	0	0	0	0	0	0	0
0	0	0	0	0	-0.11051401	0	0.0280406	0
0	-0.003958275	0	0	0	0	0	0	0
0	0	0	0	0	0	0	0	0
0	0	0	0	0	-0.10505684	0	0	0
0	0	0	0	0	0	0	0	0
0	0	0	0	0	0	0	-0.011455933	0
0	0	0	-0.10857962	0	0	0	0.026325475	0
0	0	0	0	0	0	0	0	0
0	0	0	0	0	0	0	0	0
0	0	0	-0.10347515	0	0	0	0	0
0	0	0	0	0	0	0	0	0
0	0	0	0	0	-0.10891338	0	0.029170165	0
0	0	0	-0.043262263	0	-0.070146676	0	0	0
0	-0.006963439	0	0	0	-0.11288564	0	0	0
0	0	0	0	0	-0.11232997	0	0	0

0	0	0	0	0	0	0	-0.018451707	0
0	-0.006673545	0	-0.11135162	0	0	0	0	0
0	0	0	-0.11081094	0	0	0	0	0
0	0	0	-0.10681906	0	0	0	0.028091882	0
0	0	0	0	0	0	0	-0.018902712	0
0	0	0	0	0	0	0	-0.017783017	0
0	0	0	0	0	0	0	0	0
0	-0.005365281	0	0	0	0	0	0	0
0	-0.009751488	0	0	0	0	0	-0.021181555	0
0	0	0	0	0	0	0	0	0
0	0	0	0	0	0	0	0	0
0	0	0	0	0	0	0	-0.019186309	0
0	0	0	0	0	0	0	0	0
0	0	0	0	0	0	0	0	0
0	-0.002960112	0	0	0	0	0	0	0
0	-0.001469289	0	0	0	0	0	0	0
0	-1.0576795	0	0	0	0	0	0	0
0	0	0	-0.028767686	0	-0.080074011	0	0	0
0	-0.006573248	0	0	0	-0.10843645	0	0	0
0	0	0	0	0	-0.10785159	0	0	0
0	-0.006205081	0	-0.10667078	0	0	0	0	0
0	0	0	-0.10609938	0	0	0	0	0
0	0	0	0	0	-0.12146498	0	0.058862219	0
0	0	0	0	0	0	0	0	0
0	0.005074473	0	0	0	0	0	0	0
0	0.011698256	0	0	0	-0.12240589	0	0.058086654	0
0	0	0	-0.038662615	0	-0.085982736	0	0.056151681	0
0	0	0	-0.11953065	0	0	0	0.057182807	0
0	0.011555594	0	-0.12048431	0	0	0	0.056388123	0
0	-1.1263565	0	-0.1480936	0	0	0	0	0
0	-1.126226	0	0	0	-0.1494006	0	0	0
0	-0.099951089	0	0	0	-0.18149372	0	0	0
0	-0.096021914	0	-0.17754663	0	0	0	0	0
0	0	0	0	0	-0.17816754	0	0	0
0	0	0	-0.17438146	0	0	0	0	0
0	-1.1694059	0	0	0	0	0	0	0
0	0	0	0	0	0	0	0	0
0	0	0	0	0	0	0	0.010310681	0
0	0	0	0	0	0	0	0	0
0	0	0	0	0	0	0	0	0
0	0	0	0	0	0	0	0	0
0	0.005443924	0	0	0	0	0	0	0
0	0	0	0	0	0	0	0	0
0	0	0	0	0	0	0	0.01039817	0
0	0	0	0	0	0	0	0	0
0	0	0	-0.081084036	0	-0.079984532	0	0	0
0	0	0	0	0	0	0	0	0

0	0.005495417	0	0	0	0	0	0	0
0	0	0	0	0	0	0	0.022781234	0
0	0	0	0	0	0	0	0.020182264	0
0	0.013140913	0	0	0	0	0	0.021609737	0
0	0	0	0	0	0	0	0.022568056	0
0	0	0	0	0	0	0	0	0
0	0	0	0	0	0	0	0	0
0	0.005239233	0	0	0	0	0	0	0
0	0	0	0	0	0	0	0	0
0	0.016202509	0	0	0	0	0	0	0
0	-1.0786083	0	0	0	0	0	0	0
0	-1.0596609	0	0	0	0	0	0	0
0	0	0	0	0	0	0	0.019207752	0
0	0	0	0	0	0	0	0	0
0	0	0	0	0	0	0	0	0
0	-0.005878091	0	0	0	0	0	0	0
0	0	0	0	0	0	0	0	0
0	0	0	0	0	0	0	0	0
0	-0.004783292	0	-0.084084243	0	-0.065358664	0	0	0
0	0	0	-0.083931678	0	-0.064897547	0	0	0
0	0	0	0	0	0	0	0.032589471	0
0	0	0	0	0	0	0	0.046544834	0
0	0	0	0	0	0	0	0.04485396	0
0	0	0	0	0	0	0	0.015931263	0
0	0.006717772	0	0	0	0	0	0.043554375	0
0	0	0	0	0	0	0	0.033895893	0
0	0	0	0	0	0	0	0.040991254	0
0	0	0	0	0	0	0	0	0
0	0.002425323	0	0	0	0	0	0	0
0	-0.044814023	0	0	0	0	0	0	0
0	0	0	0	0	0	0	0	0
0	-0.004549886	0	0	0	0	0	0	0
0	0	0	0	0	0	0	0	0
0	0	0	0	0	0	0	0	0
0	-1.0882566	0	0	0	0	0	0.085588479	0
0	0	0	0	0	0	0	0.030253086	0
0	0	0	0	0	0	0	0.037457716	0
0	-0.042127074	0	0	0	0	0	0	0
0	0.012071314	0	0	0	0	0	0.028614029	0
0	0.014652208	0	0	0	0	0	0.036094723	0
0	0	0	0	0	0	0	0	0
0	0	0	0	0	0	0	0	0
0	-0.002313163	0	0	0	0	0	0	0
0	-1.1036374	0	0	0	-0.13049558	0	0	0
0	-1.1031248	0	-0.12646326	0	0	0	0	0
0	0	0	0	0	0	0	0.089135646	0
0	-0.003471741	0	0	0	0	0	0.08937421	0

0	-0.12100396	0	0	0	-0.12865823	0	0	0
0	0	0	0	0	-0.14398527	0	0	0
0	-0.074975102	0	0	0	-0.1540113	0	0	0
0	0	0	0	0	-0.16021389	0	0	0
0	-0.11576747	0	-0.12268646	0	0	0	0	0
0	0	0	-0.13832396	0	0	0	0	0
0	0	0	0	0	-0.16000462	0	0	0
0	-0.007553041	0	0	0	-0.15852614	0	0	0
0	0	0	-0.022606608	0	-0.13602753	0	0	0
0	0	0	0	0	-0.12571201	0	0	0
0	0	0	0	0	-0.1578105	0	0.002547358	0
0	0	0	0	0	-0.15792155	0	0	0
0	0	0	0	0	-0.15798826	0	0	0
0	0	0	0	0	-0.15048653	0	0	0
0	-0.070210235	0	-0.14779105	0	0	0	0	0
0	0	0	-0.15477896	0	0	0	0	0
0	-1.1054199	0	0	0	-0.09545654	0	0	0
0	0	0	-0.11989883	0	0	0	0	0
0	-0.00688779	0	-0.1533532	0	0	0	0	0
0	0	0	-0.15419802	0	0	0	0	0
0	0	0	0	0	-0.11299021	0	0	0
0	0	0	-0.15271574	0	0	0	0	0
0	0	0	-0.15274366	0	0	0	0.001711906	0
0	0	0	-0.15283143	0	0	0	0	0
0	0	0	0	0	-0.14351319	0	0	0
0	0	0	-0.14438755	0	0	0	0	0
0	0	0	0	0	-0.13302956	0	0	0
0	0	0	0	0	-0.13742392	0	0	0
0	0	0	0	0	-0.13770597	0	-0.003112047	0
0	-1.1064854	0	0	0	-0.13292198	0	0	0
0	0	0	-0.00678523	0	-0.13042992	0	0	0
0	-1.1048562	0	-0.092631159	0	0	0	0	0
0	0	0	0	0	-0.1421643	0	0	0
0	0	0	-0.10928254	0	0	0	0	0
0	-1.1063078	0	-0.13013126	0	0	0	0	0
0	0	0	0	0	-0.14029311	0	-0.008027624	0
0	0	0	0	0	-0.11477359	0	0	0
0	-0.007560904	0	0	0	-0.13952933	0	0	0
0	0	0	0	0	-0.13548564	0	0	0
0	0	0	-0.007358443	0	-0.13177439	0	0	0
0	0	0	0	0	-0.13897297	0	0	0
0	0	0	-0.1377292	0	0	0	0	0
0	-0.012612999	0	0	0	-0.13426784	0	-0.015703499	0
0	0	0	-0.12705249	0	0	0	0	0
0	0	0	0	0	-0.13321592	0	-0.014253104	0
0	0	0	-0.010022744	0	-0.12286276	0	-0.013615566	0
0	0	0	0	0	-0.13256603	0	-0.013398429	0

0	0	0	0	0	-0.13265706	0	-0.013483674	0
0	0	0	-0.13320961	0	0	0	-0.00375908	0
0	0	0	-0.13256963	0	0	0	0	0
0	-0.007480201	0	0	0	-0.13372857	0	0	0
0	0	0	-0.11196057	0	0	0	0	0
0	-0.008776847	0	0	0	-0.12914636	0	0	0
0	0	0	-0.008600306	0	-0.12507979	0	0	0
0	0	0	0	0	-0.1331666	0	0	0
0	-0.007862442	0	-0.006207567	0	-0.12319015	0	0	0
0	0	0	0	0	-0.13337703	0	0	0
0	0	0	-0.006502611	0	-0.12217596	0	0	0
0	0	0	0	0	-0.12860611	0	0	0
0	0	0	-0.006117024	0	-0.1226909	0	0	0
0	0	0	0	0	-0.11727147	0	0	0
0	0	0	-0.13684369	0	0	0	0	0
0	0	0	-0.13506973	0	0	0	-0.008843893	0
0	0	0	-0.12928713	0	0	0	0	0
0	-0.006932666	0	-0.13402986	0	0	0	0	0
0	0	0	-0.13349143	0	0	0	0	0
0	-0.011979811	0	-0.13026233	0	0	0	-0.015664125	0
0	0	0	-0.12921825	0	0	0	-0.014206586	0
0	0	0	-0.12856987	0	0	0	-0.013368589	0
0	0	0	-0.12705179	0	0	0	-0.014688584	0
0	0	0	0	0	-0.11112324	0	0	0
0	0	0	-0.11314614	0	0	0	0	0
0	-0.008489708	0	-0.1250874	0	0	0	0	0
0	-0.006991287	0	-0.1280004	0	0	0	0	0
0	0	0	-0.12731456	0	0	0	0	0
0	0	0	-0.12767355	0	0	0	0	0
0	0	0	-0.12455075	0	0	0	0	0
0	0	0	0	0	-0.10618734	0	0	0
0	0	0	-0.10737086	0	0	0	0	0
0	0	0	0	0	-0.10526017	0	-0.00219962	0
0	0	0	0.002195407	0	-0.10759332	0	0	0
0	-0.092187554	0	0	0	-0.15782867	0	0	0
0	0	0	0	0	-0.10166571	0	0	0
0	-0.012890721	0	0	0	-0.10178357	0	0	0
0	0	0	0	0	-0.10036193	0	-0.009407083	0
0	0	0	0	0	-0.10129178	0	0	0
0	0	0	-0.008846302	0	-0.092343701	0	0	0
0	0	0	-0.10212239	0	0	0	0	0
0	0	0	-0.098756337	0	0	0	0	0
0	0	0	-0.10133836	0	0	0	-0.003579534	0
0	-0.012403656	0	-0.099050341	0	0	0	0	0
0	0	0	-0.097715177	0	0	0	-0.01028259	0
0	-0.087776534	0	-0.15284551	0	0	0	0	0
0	0	0	-0.098573104	0	0	0	0	0

0	0	0	0	0	-0.15445663	0	0	0
0	-0.017949662	0	0	0	-0.10716297	0	0	0
0	0	0	0	0	-0.10477337	0	-0.013424802	0
0	0	0	0	0	-0.10640451	0	0	0
0	0	0	-0.008958659	0	-0.096814399	0	0	0
0	-0.01732482	0	-0.10408948	0	0	0	0	0
0	0	0	-0.10187052	0	0	0	-0.014341719	0
0	0	0	-0.14962005	0	0	0	0	0
0	0	0	-0.1033495	0	0	0	0	0
0	0	0	0	0	-0.13227257	0	0.026827104	0
0	0	0	-0.12889325	0	0	0	0.024997614	0
0	0	0	0	0	-0.093481239	0	-0.01339325	0
0	0	0	0	0	-0.13524835	0	0.024831375	0
0	-0.010971062	0	0	0	-0.094827631	0	0	0
0	-0.016780498	0	0	0	-0.093426634	0	-0.01883712	0
0	0	0	0	0	-0.13268637	0	0	0
0	0	0	0	0	-0.094322639	0	0	0
0	0	0	0.000691745	0	-0.09480094	0	0	0
0	0	0	-0.036800454	0	-0.10143735	0	0	0
0	-0.11301213	0	0	0	-0.14685261	0	0	0
0	0	0	0	0	-0.13961789	0	0	0
0	0	0	0	0	-0.092969884	0	-0.016812038	0
0	0	0	-0.003922708	0	-0.088745655	0	-0.01483119	0
0	-0.025125219	0	0	0	-0.14012786	0	0	0
0	-0.010526436	0	-0.002288615	0	-0.091656537	0	0	0
0	0	0	-0.090498569	0	0	0	-0.014299443	0
0	-0.017265124	0	0	0	-0.12736348	0	0.012715693	0
0	0	0	-0.002294905	0	-0.091152062	0	0	0
0	-0.016571501	0	-0.09068642	0	0	0	-0.019597955	0
0	-0.010453116	0	-0.091705257	0	0	0	0	0
0	0	0	-0.13174253	0	0	0	0.022855601	0
0	0	0	0	0	-0.12831574	0	0.019441052	0
0	0	0	0	0	-0.12633068	0	0.014264781	0
0	0	0	0	0	-0.13919647	0	0	0
0	0	0	-0.028329078	0	-0.097615259	0	0.015311376	0
0	-0.022255822	0	-0.035632895	0	-0.096600884	0	0	0
0	0	0	-0.091218643	0	0	0	0	0
0	0	0	-0.12990057	0	0	0	0	0
0	0	0	0	0	-0.13355188	0	0	0
0	-1.0626216	0	0	0	0	0	0	0
0	-0.022058938	0	0	0	-0.13043888	0	0	0
0	0	0	-0.09023146	0	0	0	-0.017570366	0
0	0	0	-0.039538961	0	-0.098636791	0	0	0
0	0	0	-0.13616139	0	0	0	0	0
0	0	0	-0.035767268	0	-0.095951295	0	0	0
0	-0.10861471	0	-0.14242782	0	0	0	0	0
0	-0.024234056	0	-0.1367468	0	0	0	0	0

0	0	0	0	0	-0.12966672	0	0	0
0	0	0	-0.034525223	0	-0.0948179	0	0	0
0	-0.017043517	0	-0.12479741	0	0	0	0.011505216	0
0	0	0	-0.13585189	0	0	0	0	0
0	0	0	-0.12588674	0	0	0	0.018253238	0
0	0	0	-0.12376382	0	0	0	0.013069462	0
0	-0.021468062	0	-0.12812167	0	0	0	0	0
0	0	0	-0.1307448	0	0	0	0	0
0	-0.11002748	0	0	0	-0.14171228	0	0	0
0	0	0	-0.12736826	0	0	0	0	0
0	0	0	0	0	-0.1437949	0	0	0
0	-1.1161578	0	0	0	-0.11426087	0	0	0
0	-1.1751301	0	0	0	-0.10822358	0	0	0
0	-1.1160571	0	-0.11274079	0	0	0	0	0
0	-1.0218394	0	0	0	0	0	0	0
0	0	0	-0.13953156	0	0	0	0	0
0	-0.091649155	0	0	0	-0.15661172	0	0.063286136	0
0	-1.1736665	0	-0.10639216	0	0	0	0	0
0	-0.10528739	0	-0.13705381	0	0	0	0	0
0	0	0	0	0	-0.13859759	0	0	0
0	-1.0697974	0	0	0	0	0	0	0
0	-1.0695627	0	0	0	0	0	-0.018188879	0
0	-1.0716364	0	0	0	0	0	0	0
0	-1.0698257	0	0	0	0	0	0	0
0	0	0	0	0	-0.12364173	0	0.038933318	0
0	-0.087178012	0	-0.15166292	0	0	0	0.061151622	0
0	0	0	0	0	-0.1531426	0	0.063619848	0
0	-1.1257702	0	0	0	-0.1231857	0	0.058837387	0
0	-1.1253442	0	-0.12124601	0	0	0	0.057129072	0
0	0	0	-0.13411123	0	0	0	0	0
0	0	0	-0.12030293	0	0	0	0.036703736	0
0	-1.075965	0	0	0	0	0	0	0
0	0	0	0	0	-0.12320599	0	0.041867597	0
0	0	0	-0.14832821	0	0	0	0.061527633	0
0	0	0	0	0	-0.11711525	0	0	0
0	0	0	-0.11454419	0	0	0	0	0
0	-0.062521019	0	0	0	0	0	0	0
0	0	0	-0.11974751	0	0	0	0.040215652	0
0	0	0	-0.039026954	0	-0.086337399	0	0	0
0	0	0	0	0	0	0	0	0
0	0	0	-0.028186089	0	-0.10861067	0	0.064631232	0
0	-1.1205449	0	0	0	-0.17966803	0	0	0
0	-0.090231814	0	0	0	0	0	0	0
0	-1.1205825	0	-0.17586391	0	0	0	0	0
0	-0.068850238	0	0	0	0	0	-0.020272907	0
0	0	0	-0.02442074	0	-0.096183064	0	0	0
0	0	0	0	0	0	0	0	0

0	-0.062144065	0	0	0	0	0	0	0
0	0	0	0	0	0	0	0	0
0	0	0	0	0	0	0	-0.007922641	0
0	0	0	0	0	0	0	0	0
0	0	0	0	0	0	0	0	0
0	-0.06333686	0	0	0	0	0	0	0
0	0	0	0	0	0	0	0	0
0	0	0	0	0	0	0	-0.018539432	0
0	0	0	0	0	0	0	0	0
0	0	0	0	0	0	0	-0.014616565	0
0	0	0	0	0	0	0	0	0
0	-0.006860959	0	0	0	0	0	0	0
0	0	0	0	0	0	0	0	0
0	0	0	0	0	0	0	0	0
0	0	0	0	0	-0.10613107	0	0.020743388	0
0	0	0	0	0	0	0	-0.015175087	0
0	0	0	0	0	0	0	0	0
0	0	0	0	0	0	0	-0.014172466	0
0	-0.009713757	0	0	0	0	0	-0.017412743	0
0	0	0	0	0	0	0	-0.015552418	0
0	0	0	0	0	0	0	0	0
0	-0.002789749	0	0	0	0	0	0	0
0	0	0	0	0	0	0	0	0
0	0	0	0	0	-0.10970578	0	0.029751531	0
0	-0.004194329	0	0	0	0	0	0	0
0	0	0	-0.03234153	0	-0.078967604	0	0.027459321	0
0	0	0	0	0	0	0	0	0
0	0.001665258	0	0	0	-0.11037831	0	0.028321227	0
0	0	0	-0.10423104	0	0	0	0.01948329	0
0	0	0	-0.030233651	0	-0.07572822	0	0	0
0	0	0	0	0	0	0	-0.010541065	0
0	-0.007106372	0	0	0	-0.10554966	0	0	0
0	0	0	0	0	-0.10498583	0	0	0
0	0	0	0	0	0	0	0	0
0	0	0	0	0	0	0	-0.011933451	0
0	0	0	-0.1077686	0	0	0	0.028018064	0
0	0	0	0	0	0	0	-0.011449305	0
0	0.001472117	0	-0.10845696	0	0	0	0.026575713	0
0	0	0	0	0	0	0	0	0
0	0	0	0	0	0	0	-0.015733333	0
0	-0.006770536	0	-0.1039338	0	0	0	0	0
0	0	0	0	0	0	0	0	0
0	0	0	-0.10338424	0	0	0	0	0
0	-1.098794	0	0	0	0	0	0	0
0	-1.0629395	0	0	0	0	0	0	0
0	-0.008318189	0	0	0	0	0	0	0
0	0	0	0	0	0	0	0	0

0	0	0	0	0	-0.10819878	0	0.031283222	0
0	0	0	-0.023753823	0	-0.085867345	0	0.028961298	0
0	0.002417968	0	0	0	-0.10875882	0	0.029631442	0
0	-0.007006276	0	-0.043347193	0	-0.070555258	0	0	0
0	0	0	-0.043252148	0	-0.070092188	0	0	0
0	-0.012574803	0	0	0	0	0	-0.021480245	0
0	-1.0638318	0	0	0	0	0	0.023886928	0
0	0	0	0	0	0	0	-0.017823603	0
0	0	0	0	0	0	0	-0.019487953	0
0	0	0	0	0	0	0	-0.018515208	0
0	0	0	-0.10609313	0	0	0	0.030224411	0
0	-0.008491381	0	0	0	0	0	-0.020959046	0
0	0.002490722	0	-0.10665833	0	0	0	0.028568491	0
0	-0.009655196	0	0	0	0	0	-0.020127874	0
0	0	0	0	0	0	0	-0.018958402	0
0	0	0	0	0	0	0	-0.018130599	0
0	0	0	0	0	0	0	0	0
0	-0.001716497	0	0	0	0	0	0	0
0	-0.005039875	0	0	0	0	0	0	0
0	0	0	0	0	0	0	0	0
0	-1.0605809	0	0	0	0	0	0	0
0	-1.0574402	0	0	0	0	0	0	0
0	-0.006597271	0	-0.028836072	0	-0.080469969	0	0	0
0	0	0	-0.028747955	0	-0.079970612	0	0	0
0	-1.1275018	0	0	0	0	0	0	0
0	0	0	-0.03677919	0	-0.085557284	0	0.058026701	0
0	0.011397197	0	-0.037567573	0	-0.085728804	0	0.057233189	0
0	-1.1269385	0	-0.08481278	0	-0.065741272	0	0	0
0	-0.099781558	0	-0.080026582	0	-0.10245232	0	0	0
0	-1.1371875	0	0	0	0	0	0	0
0	0	0	-0.08014063	0	-0.099011482	0	0	0
0	-1.0707132	0	0	0	0	0	0.045697753	0
0	-1.1672002	0	0	0	0	0	0	0
0	-1.1544091	0	0	0	0	0	0.027247081	0
0	-1.0684089	0	0	0	0	0	0	0
0	-1.0833106	0	0	0	0	0	0.03801463	0
0	0	0	0	0	0	0	0	0
0	0	0	0	0	0	0	0	0
0	0	0	0	0	0	0	0.013386117	0
0	0	0	0	0	0	0	0.014520556	0
0	0	0	0	0	0	0	0.006339366	0
0	0.004791425	0	0	0	0	0	0	0
0	0	0	0	0	0	0	0	0
0	0	0	0	0	0	0	0	0
0	0	0	0	0	0	0	0	0
0	0.008893314	0	0	0	0	0	0.011914553	0
0	0	0	0	0	0	0	0.009103025	0

0	0	0	0	0	0	0	0	0
0	-1.0715356	0	0	0	0	0	0	0
0	0.007544002	0	0	0	0	0	0	0
0	-0.004208259	0	0	0	0	0	0	0
0	0	0	0	0	0	0	0.013826614	0
0	0	0	0	0	0	0	0.014131414	0
0	0	0	0	0	0	0	0.010951609	0
0	0	0	0	0	0	0	0	0
0	0.009131348	0	0	0	0	0	0.012208783	0
0	0	0	0	0	0	0	0	0
0	0	0	0	0	0	0	0	0
0	0.00324048	0	0	0	0	0	0	0
0	-0.004502518	0	0	0	0	0	0	0
0	0	0	0	0	0	0	0.022844516	0
0	0	0	0	0	0	0	0.020987299	0
0	0.01093563	0	0	0	0	0	0.021687971	0
0	0	0	0	0	0	0	0.022436431	0
0	0.012543128	0	0	0	0	0	0.021810012	0
0	0	0	0	0	0	0	0	0
0	-1.0621841	0	0	0	0	0	0	0
0	0.014236569	0	0	0	0	0	0	0
0	0	0	0	0	0	0	0.024013499	0
0	-0.049862039	0	0	0	0	0	0	0
0	-0.031373244	0	0	0	0	0	0	0
0	0	0	0	0	0	0	0	0
0	0	0	0	0	0	0	0.017377729	0
0	0	0	0	0	0	0	0.021084059	0
0	0.000467448	0	0	0	0	0	0.019305269	0
0	0	0	0	0	0	0	0	0
0	0	0	0	0	0	0	0.023539629	0
0	-0.004813536	0	0	0	0	0	0	0
0	0	0	0	0	0	0	0	0
0	0	0	0	0	0	0	0	0
0	0	0	0	0	0	0	0.037064808	0
0	0	0	0	0	0	0	0.03494394	0
0	0.005761861	0	0	0	0	0	0.033553173	0
0	-0.037053036	0	0	0	0	0	0.030584773	0
0	-0.033681208	0	0	0	0	0	0.038716588	0
0	0	0	0	0	0	0	0.019356252	0
0	0	0	0	0	0	0	0.046424562	0
0	-0.004634251	0	0	0	0	0	0.046616141	0
0	0.007869193	0	0	0	0	0	0.017579023	0
0	0	0	0	0	0	0	0.031999181	0
0	0	0	0	0	0	0	0.039659674	0
0	-0.028247721	0	0	0	0	0	0	0
0	0	0	0	0	0	0	0	0
0	-1.0831007	0	0	0	0	0	0.089328535	0

0	-1.0974429	0	0	0	-0.12756074	0	0	0
0	-1.1010864	0	0	0	-0.15212837	0	0	0
0	-1.0538298	0	0	0	-0.15611569	0	0	0
0	-1.0968136	0	-0.12177582	0	0	0	0	0
0	-1.1006785	0	-0.1460409	0	0	0	0	0
0	-1.0553214	0	-0.15098729	0	0	0	0	0
0	-0.12514348	0	0	0	-0.13742991	0	0	0
0	-1.1032552	0	0	0	-0.14073644	0	0	0
0	-1.1016542	0	0	0	-0.13478635	0	-0.013547864	0
0	-1.1040056	0	0	0	-0.13540332	0	0	0
0	-1.1035332	0	0	0	-0.13041009	0	0	0
0	-1.1037377	0	-0.007480437	0	-0.12314844	0	0	0
0	-0.11974328	0	-0.13149602	0	0	0	0	0
0	-1.102762	0	-0.13526204	0	0	0	0	0
0	0	0	0	0	-0.13481233	0	0	0
0	-1.1012	0	-0.13080027	0	0	0	-0.013518671	0
0	-1.1033876	0	-0.1297162	0	0	0	0	0
0	-1.1029696	0	-0.12636105	0	0	0	0	0
0	0	0	-0.12901543	0	0	0	0	0
0	0	0	0	0	-0.14343718	0	0	0
0	-0.05946104	0	0	0	-0.1683407	0	0	0
0	-0.10366766	0	0	0	-0.13688061	0	0	0
0	-0.12094768	0	0	0	-0.12936917	0	0	0
0	0	0	0	0	-0.14581071	0	0	0
0	-0.12591808	0	0	0	-0.12810859	0	-0.016303728	0
0	-0.11664259	0	0	0	-0.13092559	0	0	0
0	-1.1037541	0	0	0	-0.15609719	0	0	0
0	-0.12106896	0	0.008058391	0	-0.13649647	0	0	0
0	-1.0357614	0	0	0	-0.1020808	0	0	0
0	-1.1030402	0	0	0	-0.1084063	0	0	0
0	-0.009875871	0	0	0	-0.14447386	0	0	0
0	0	0	0	0	-0.1661945	0	0	0
0	0	0	0	0	-0.14342637	0	-0.007525491	0
0	0	0	-0.13750081	0	0	0	0	0
0	0	0	0	0	-0.1435058	0	0	0
0	0	0	-0.016069389	0	-0.12846567	0	0	0
0	0	0	0	0	-0.14411481	0	0	0
0	0	0	0	0	-0.12667269	0	0	0
0	-1.0354012	0	-0.099364955	0	0	0	0	0
0	-0.072386706	0	0	0	-0.15570547	0	0	0
0	-1.1035986	0	-0.15126056	0	0	0	0	0
0	-0.075008433	0	-0.008554117	0	-0.14561887	0	0	0
0	-0.074875603	0	0	0	-0.15431902	0	-0.001676324	0
0	-0.075211674	0	0	0	-0.15393649	0	0	0
0	0	0	0	0	-0.1360123	0	0	0
0	-1.1025757	0	-0.10537818	0	0	0	0	0
0	0	0	0	0	-0.15987747	0	0.009471149	0

0	0	0	-0.021173242	0	-0.13964482	0	0	0
0	0	0	-0.13966177	0	0	0	0	0
0	0	0	0	0	-0.16134115	0	0	0
0	0	0	0	0	-0.16048431	0	0	0
0	-0.12110705	0	-0.12225552	0	0	0	-0.017449832	0
0	-0.10016951	0	-0.12990703	0	0	0	0	0
0	-0.11561787	0	-0.12314481	0	0	0	0	0
0	-0.11132855	0	-0.12510114	0	0	0	0	0
0	-0.054945428	0	-0.1618406	0	0	0	0	0
0	0	0	0	0	-0.12642721	0	0	0
0	0	0	0	0	-0.12821537	0	0	0
0	0	0	0	0	-0.12522694	0	-0.014750106	0
0	0	0	-0.13776627	0	0	0	-0.008843	0
0	-0.009357495	0	-0.13876384	0	0	0	0	0
0	0	0	-0.13772079	0	0	0	0	0
0	-0.007460815	0	0	0	-0.16052732	0	0	0
0	0	0	0	0	-0.16046736	0	0	0
0	0	0	-0.022118975	0	-0.13850615	0	0	0
0	0	0	-0.13842289	0	0	0	0	0
0	0	0	0	0	-0.15992601	0	0.004155403	0
0	-0.007571247	0	-0.022682071	0	-0.13650147	0	0	0
0	0	0	0	0	-0.16000413	0	0	0
0	0	0	-0.12268023	0	0	0	0	0
0	-0.008053117	0	0	0	-0.15840547	0	0	0
0	-0.007221816	0	0	0	-0.15843006	0	0.001085215	0
0	0	0	-0.022228114	0	-0.13623812	0	0.002356246	0
0	0	0	-0.022749781	0	-0.13581718	0	0	0
0	0	0	-0.022607968	0	-0.13603575	0	0	0
0	0	0	0.007511032	0	-0.13301826	0	0	0
0	0	0	0	0	-0.15773686	0	0.002600938	0
0	0	0	0	0	-0.1577482	0	0.002690595	0
0	0	0	0	0	-0.15794071	0	0	0
0	0	0	0	0	-0.11991514	0	0	0
0	0	0	0	0	-0.15286903	0	0	0
0	0	0	0	0	-0.15085517	0	-0.002004636	0
0	0	0	-0.00812177	0	-0.14251798	0	0	0
0	0	0	0	0	-0.15046615	0	0	0
0	0	0	-0.15981967	0	0	0	0	0
0	0	0	-0.12920811	0	0	0	0	0
0	0	0	-0.15439881	0	0	0	0.008128953	0
0	-0.06852774	0	-0.14883466	0	0	0	0	0
0	-0.070794064	0	-0.14765143	0	0	0	0	0
0	-0.070072125	0	-0.14827136	0	0	0	-0.002492031	0
0	-1.1045258	0	0	0	-0.096386545	0	0	0
0	0	0	-0.15532674	0	0	0	0	0
0	0	0	-0.15484344	0	0	0	0	0
0	0	0	-0.12256288	0	0	0	0	0

0	0	0	-0.12036093	0	0	0	0	0
0	-1.1026887	0	0	0	-0.095069085	0	-0.015730883	0
0	0	0	-0.11633771	0	0	0	0	0
0	0	0	-0.11951892	0	0	0	-0.01585888	0
0	0	0	0	0	-0.11357237	0	0	0
0	0	0	0	0	-0.13496406	0	0	0
0	0	0	-0.15474453	0	0	0	0	0
0	-1.1054734	0	-0.00379524	0	-0.091774428	0	0	0
0	-0.006818227	0	-0.15465924	0	0	0	0	0
0	-0.007804297	0	-0.15318286	0	0	0	0	0
0	0	0	-0.15411117	0	0	0	0.002823019	0
0	-0.006786427	0	-0.15332085	0	0	0	0.000333718	0
0	0	0	-0.15415108	0	0	0	0	0
0	0	0	0	0	-0.11335193	0	0.00343674	0
0	0	0	0	0	-0.14458112	0	0	0
0	0	0	-0.004342787	0	-0.10877571	0	0	0
0	0	0	-0.1525692	0	0	0	0.001863204	0
0	0	0	-0.15271869	0	0	0	0	0
0	0	0	-0.15264281	0	0	0	0.001946707	0
0	-1.1083078	0	0	0	-0.12797098	0	0.014739644	0
0	-1.1058502	0	0	0	-0.14091208	0	0	0
0	0	0	-0.007602461	0	-0.13605802	0	0	0
0	0	0	0	0	-0.14346284	0	0.000474587	0
0	-1.1039485	0	-0.093308954	0	0	0	0	0
0	-1.1020314	0	-0.09234645	0	0	0	-0.016505468	0
0	0	0	-0.14610923	0	0	0	0	0
0	-1.1069311	0	-0.036981116	0	-0.096555734	0	0	0
0	0	0	-0.14492792	0	0	0	-0.002799106	0
0	0	0	-0.14429339	0	0	0	0	0
0	-1.1135366	0	0	0	-0.12901153	0	0	0
0	0	0	0	0	-0.1327083	0	-0.006019221	0
0	0	0	0	0	-0.1334232	0	0	0
0	0	0	-0.004511296	0	-0.1286645	0	0	0
0	0	0	0	0	-0.13804234	0	-0.00313017	0
0	0	0	-0.006282326	0	-0.13124959	0	0	0
0	0	0	-0.007685723	0	-0.1301816	0	-0.003227841	0
0	0	0	0	0	-0.13347061	0	0	0
0	0	0	-0.10965504	0	0	0	0	0
0	0	0	0	0	-0.11939611	0	0	0
0	-0.012619513	0	0	0	-0.14350409	0	0	0
0	-1.1078847	0	-0.12540515	0	0	0	0.01351975	0
0	-1.1057254	0	-0.13757091	0	0	0	0	0
0	-0.021185974	0	0	0	-0.11688545	0	0	0
0	0	0	0	0	-0.14265067	0	-0.00433314	0
0	0	0	0	0	-0.14262544	0	0	0
0	0	0	-0.01085269	0	-0.13154219	0	0	0
0	0	0	-0.10946881	0	0	0	0.001954296	0

0	0	0	0	0	-0.11615301	0	0	0
0	0	0	0	0	-0.13475429	0	-0.011676272	0
0	-1.112433	0	-0.12665215	0	0	0	0	0
0	-0.010657355	0	0	0	-0.14138606	0	-0.010091749	0
0	-1.1089874	0	0	0	-0.14550584	0	0	0
0	0	0	0	0	-0.11424841	0	-0.007526889	0
0	-0.007960361	0	0	0	-0.13584831	0	0	0
0	0	0	-0.009570323	0	-0.13092631	0	-0.008160523	0
0	0	0	0	0	-0.14052984	0	-0.008482855	0
0	0	0	-0.12851616	0	0	0	0	0
0	0	0	-0.016538838	0	-0.098786446	0	0	0
0	-0.007568284	0	-0.007442238	0	-0.13223096	0	0	0
0	0	0	-0.005032859	0	-0.13060806	0	0	0
0	0	0	0	0	-0.13549182	0	0	0
0	0	0	-0.007355688	0	-0.13175753	0	0	0
0	0	0	-0.13894302	0	0	0	0	0
0	-0.02488839	0	0	0	-0.12011381	0	0	0
0	0	0	-0.13782574	0	0	0	-0.00078919	0
0	-0.02055027	0	-0.11396676	0	0	0	0	0
0	-0.01342502	0	0	0	-0.13411783	0	-0.01563258	0
0	-0.012670515	0	-0.010812735	0	-0.12369732	0	-0.015860131	0
0	-0.013022965	0	0	0	-0.13304543	0	-0.016632445	0
0	0	0	-0.12674671	0	0	0	-0.007490221	0
0	-1.1717548	0	0	0	-0.13885395	0	0	0
0	0	0	-0.010275736	0	-0.1231734	0	-0.014403409	0
0	0	0	0	0	-0.13312023	0	-0.014208535	0
0	0	0	-0.12718226	0	0	0	0	0
0	0	0	0	0	-0.13277441	0	-0.014589042	0
0	0	0	-0.010237163	0	-0.1225407	0	-0.013523431	0
0	0	0	-0.010063584	0	-0.12290783	0	-0.01355917	0
0	0	0	0	0	-0.13245557	0	-0.013459108	0
0	0	0	-0.11324977	0	0	0	0	0
0	0	0	-0.1333425	0	0	0	-0.003765296	0
0	0	0	-0.12733238	0	0	0	0	0
0	0	0	-0.11486841	0	0	0	0	0
0	0	0	0	0	-0.11917906	0	0	0
0	0	0	-0.11143758	0	0	0	-0.008528244	0
0	-1.1087742	0	-0.14124425	0	0	0	0	0
0	-0.008263776	0	0	0	-0.13335404	0	0	0
0	-0.007476832	0	-0.008573662	0	-0.12543693	0	0	0
0	-0.008797948	0	-0.00682586	0	-0.12243976	0	0	0
0	0	0	-0.00881757	0	-0.12463146	0	0	0
0	0	0	-0.008602434	0	-0.12506082	0	0	0
0	0	0	0	0	-0.13316908	0	0	0
0	0	0	0	0	-0.11666104	0	-0.007794094	0
0	0	0	-0.006524624	0	-0.12219525	0	0	0
0	-0.0120186	0	-0.13810382	0	0	0	0	0

0	0	0	-0.004300985	0	-0.11310313	0	0	0
0	0	0	-0.13747101	0	0	0	-0.005179028	0
0	0	0	-0.13723675	0	0	0	0	0
0	0	0	-0.12863365	0	0	0	-0.013041792	0
0	-0.010265361	0	-0.13611989	0	0	0	-0.010842182	0
0	-0.007416874	0	-0.12960485	0	0	0	0	0
0	0	0	-0.13526622	0	0	0	-0.009225608	0
0	0	0	-0.12926889	0	0	0	0	0
0	-0.023859773	0	-0.11577953	0	0	0	0	0
0	-0.013133653	0	-0.1301005	0	0	0	-0.015576635	0
0	-1.1700077	0	-0.13428215	0	0	0	0	0
0	-0.012934875	0	-0.12745397	0	0	0	-0.017806698	0
0	0	0	-0.11487705	0	0	0	0	0
0	-0.090369938	0	0	0	-0.15243259	0	0.025130108	0
0	0	0	-0.12909858	0	0	0	-0.014144098	0
0	0	0	-0.12718448	0	0	0	-0.015761312	0
0	0	0	-0.12670325	0	0	0	-0.014605777	0
0	0	0	0	0	-0.11083242	0	-0.00358683	0
0	0	0	-0.11253342	0	0	0	-0.009071861	0
0	0	0	-0.002803374	0	-0.10840756	0	0	0
0	-1.1194277	0	0	0	-0.15466175	0	0.06356623	0
0	-0.008193598	0	-0.12750411	0	0	0	0	0
0	0	0	-0.12731433	0	0	0	0	0
0	0	0	0.004898718	0	-0.11094755	0	0	0
0	0	0	0	0	-0.10610436	0	-0.000886247	0
0	-0.096326293	0	0	0	-0.15059898	0	0	0
0	0	0	-0.10702186	0	0	0	-0.005009396	0
0	-0.013269823	0	0	0	-0.10256539	0	0	0
0	-0.092165666	0	-0.036497488	0	-0.12195122	0	0	0
0	0	0	0.001877211	0	-0.10708146	0	-0.002188144	0
0	0	0	0	0	-0.1011588	0	-0.008141203	0
0	-0.094432018	0	0	0	-0.15582007	0	0	0
0	0	0	0	0	-0.10206755	0	0	0
0	0	0	-0.006061059	0	-0.095788215	0	0	0
0	-0.017339058	0	0	0	-0.10128162	0	-0.01356007	0
0	0	0	0	0	-0.1489947	0	0.025381461	0
0	-0.085886795	0	-0.14739372	0	0	0	0.023148211	0
0	-1.1189004	0	-0.14984292	0	0	0	0.06144507	0
0	0	0	0	0	-0.10085829	0	-0.011683728	0
0	-0.012892992	0	-0.008879548	0	-0.093177743	0	0	0
0	0	0	-0.009765642	0	-0.090893707	0	-0.009462916	0
0	0	0	-0.008857402	0	-0.092707171	0	0	0
0	0	0	-0.10192725	0	0	0	-0.00238032	0
0	-0.012739642	0	-0.099592814	0	0	0	0	0
0	0	0	-0.098268675	0	0	0	-0.009121547	0
0	-0.091472127	0	-0.14602699	0	0	0	0	0
0	-0.017135578	0	-0.098621896	0	0	0	-0.014393732	0

0	0	0	-0.099112216	0	0	0	0	0
0	0	0	0	0	-0.14777945	0	0	0
0	0	0	-0.098199768	0	0	0	-0.012514482	0
0	-0.024342541	0	0	0	-0.10674198	0	-0.0191915	0
0	-0.090097495	0	-0.15078896	0	0	0	0	0
0	0	0	-0.036461833	0	-0.11861277	0	0	0
0	0	0	0	0	-0.10602323	0	-0.017185771	0
0	0	0	0	0	-0.15304543	0	0	0
0	0	0	-0.14408854	0	0	0	0.023442725	0
0	-0.017960997	0	-0.009231348	0	-0.098229365	0	0	0
0	0	0	-0.010376398	0	-0.094727232	0	-0.013488196	0
0	0	0	-0.009098119	0	-0.097599838	0	0	0
0	-0.024022511	0	-0.10380938	0	0	0	-0.020047689	0
0	0	0	-0.10309236	0	0	0	-0.018036884	0
0	0	0	-0.14337513	0	0	0	0	0
0	0	0	-0.14817	0	0	0	0	0
0	-0.10505522	0	0	0	-0.14459713	0	0.031501128	0
0	0	0	0	0	-0.1378506	0	0.031289328	0
0	0	0	-0.023816955	0	-0.10896401	0	0.026320003	0
0	0	0	0	0	-0.133919	0	0.028759445	0
0	-0.1134709	0	0	0	-0.13942218	0	0	0
0	-1.0130935	0	0	0	0	0	0	0
0	0	0	-0.13383655	0	0	0	0.028958519	0
0	-0.016724034	0	0	0	-0.094350989	0	-0.017413586	0
0	-0.018089538	0	0	0	-0.13755706	0	0.021677048	0
0	-0.10095028	0	-0.13964681	0	0	0	0.029209386	0
0	0	0	-0.13064343	0	0	0	0.026989388	0
0	0	0	0	0	-0.09389517	0	-0.015387536	0
0	0	0	0	0	-0.13645271	0	0.023161938	0
0	0	0	-0.029916656	0	-0.10605277	0	0.024295537	0
0	0	0	-0.000934173	0	-0.092574204	0	-0.01339989	0
0	0	0	0	0	-0.13533658	0	0.024944214	0
0	0	0	0	0	-0.13517915	0	0	0
0	-0.010971292	0	0.000734308	0	-0.095540369	0	0	0
0	0	0	0	0	-0.14144545	0	0.032536539	0
0	-0.10890453	0	-0.13493149	0	0	0	0	0
0	0	0	-0.031451295	0	-0.10218161	0	0	0
0	-0.016789748	0	-0.004342714	0	-0.089212596	0	-0.018866787	0
0	-0.10189697	0	0	0	-0.14279705	0	0.034038933	0
0	0	0	0.000707968	0	-0.095010479	0	0	0
0	-0.11281621	0	-0.03533131	0	-0.1122769	0	0	0
0	0	0	-0.03808714	0	-0.10238043	0	0	0
0	-1.0641661	0	0	0	0	0	0	0
0	0	0	-0.004124829	0	-0.08896729	0	-0.016840344	0
0	-0.024669824	0	0	0	-0.13657569	0	0	0
0	-0.016503623	0	-0.091354344	0	0	0	-0.018274008	0
0	-0.025142908	0	-0.039665297	0	-0.10136226	0	0	0

0	-0.017781775	0	-0.13400493	0	0	0	0.019717794	0
0	-1.1479393	0	0	0	-0.10565555	0	0	0
0	-1.0597695	0	0	0	0	0	-0.017565125	0
0	-0.01745762	0	-0.029610599	0	-0.098447745	0	0.012112249	0
0	0	0	-0.090900481	0	0	0	-0.016245154	0
0	-0.016752626	0	0	0	-0.12995328	0	0.015962842	0
0	0	0	0	0	-0.13641716	0	0	0
0	0	0	0	0	-0.13558691	0	0	0
0	-1.1190173	0	0	0	-0.11160247	0	0.030193207	0
0	0	0	-0.03960783	0	-0.1004868	0	0	0
0	-1.0619016	0	0	0	0	0	0	0
0	0	0	-0.1329059	0	0	0	0.021220256	0
0	0	0	-0.031061629	0	-0.098217362	0	0.019150558	0
0	0	0	-0.02897537	0	-0.098034588	0	0.013674873	0
0	0	0	0	0	-0.12912429	0	0.017750283	0
0	0	0	-0.13204947	0	0	0	0	0
0	0	0	-0.13205235	0	0	0	0.023233007	0
0	0	0	-0.034686739	0	-0.099937362	0	0	0
0	-0.022110149	0	-0.034921515	0	-0.096603663	0	0	0
0	-1.062669	0	0	0	0	0	0	0
0	-1.1461149	0	-0.10395543	0	0	0	0	0
0	-0.023833508	0	-0.13358221	0	0	0	0	0
0	-1.1185399	0	-0.10965919	0	0	0	0.028426647	0
0	0	0	-0.034794164	0	-0.095955247	0	0	0
0	0	0	-0.13663017	0	0	0	0.030297809	0
0	-1.1595888	0	0	0	-0.10853201	0	0.028265939	0
0	-0.016526447	0	-0.127506	0	0	0	0.014808503	0
0	0	0	-0.13262407	0	0	0	0	0
0	-0.097311966	0	-0.1376346	0	0	0	0.032474287	0
0	0	0	-0.12667512	0	0	0	0.016601017	0
0	0	0	0	0	-0.13976204	0	0.035473274	0
0	0	0	-0.13209604	0	0	0	0	0
0	-1.1586915	0	-0.10638238	0	0	0	0.027206175	0
0	0	0	-0.035705906	0	-0.10885114	0	0	0
0	-0.10990372	0	-0.020418178	0	-0.1218649	0	0	0
0	-1.0177344	0	0	0	0	0	0	0
0	-1.1166578	0	-0.044415793	0	-0.070887693	0	0	0
0	-1.0212565	0	0	0	0	0	-0.015330102	0
0	-1.0194598	0	0	0	0	0	0	0
0	-1.0217696	0	0	0	0	0	0	0
0	-1.1748185	0	-0.027658883	0	-0.081398846	0	0	0
0	-1.0652675	0	0	0	0	0	0	0
0	-0.091676337	0	-0.028750834	0	-0.12847537	0	0.062622915	0
0	0	0	-0.13474769	0	0	0	0.033934678	0
0	-1.0667133	0	0	0	0	0	-0.018481349	0
0	-1.0669035	0	0	0	0	0	-0.017978916	0
0	-1.0688789	0	0	0	0	0	-0.017155672	0

0	-1.06876	0	0	0	0	0	0	0
0	0	0	-0.021070962	0	-0.11811602	0	0	0
0	-1.0695648	0	0	0	0	0	0	0
0	0	0	0	0	-0.12037298	0	0.033675067	0
0	0	0	-0.023126279	0	-0.10104511	0	0.038485264	0
0	-1.1260924	0	-0.037694695	0	-0.086383457	0	0.057982781	0
0	0	0	-0.02859301	0	-0.12516021	0	0.062959988	0
0	0	0	-0.11710443	0	0	0	0.031802178	0
0	-0.090168706	0	0	0	0	0	0	0
0	0	0	-0.016500946	0	-0.10717326	0	0.041700921	0
0	0	0	-0.025844451	0	-0.092020862	0	0	0
0	-0.067800225	0	0	0	0	0	-0.016668518	0
0	-0.06226963	0	0	0	0	0	0	0
0	-0.062681651	0	0	0	0	0	0	0
0	0	0	0	0	0	0	0	0
0	0	0	0	0	0	0	-0.014987031	0
0	-1.1212442	0	-0.081041607	0	-0.099622633	0	0	0
0	0	0	0	0	0	0	0	0
0	-0.096810612	0	0	0	0	0	-0.020422433	0
0	0	0	0	0	0	0	0	0
0	-0.094808611	0	0	0	0	0	0	0
0	-0.08909022	0	0	0	0	0	0	0
0	0	0	0	0	0	0	0	0
0	-0.06830998	0	0	0	0	0	-0.019369769	0
0	-0.069582924	0	0	0	0	0	-0.020225115	0
0	0	0	0	0	0	0	-0.007126694	0
0	0	0	0	0	0	0	0	0
0	0	0	0	0	0	0	-0.008482753	0
0	0	0	0	0	0	0	0	0
0	-0.059167701	0	0	0	0	0	0	0
0	0	0	0	0	0	0	0	0
0	0	0	0	0	0	0	-0.01871749	0
0	0	0	0	0	0	0	-0.012010395	0
0	0	0	0	0	0	0	-0.007893168	0
0	-0.00968854	0	0	0	0	0	0	0
0	0	0	0	0	0	0	0	0
0	0	0	0	0	0	0	0	0
0	0	0	0	0	0	0	0	0
0	0	0	0	0	0	0	-0.01761692	0
0	0	0	0	0	0	0	-0.018535932	0
0	0	0	0	0	0	0	0	0
0	-0.012740319	0	0	0	0	0	-0.017681652	0
0	0	0	0	0	0	0	-0.015805759	0
0	0	0	0	0	0	0	-0.014639862	0
0	0	0	0	0	0	0	-0.014237149	0
0	-0.007019893	0	0	0	0	0	0	0
0	0	0	0	0	-0.10567221	0	0.022359908	0

0	0	0	-0.026490063	0	-0.080420318	0	0.020480267	0
0	-0.008516642	0	0	0	0	0	-0.017237264	0
0	0	0	0	0	0	0	0	0
0	0	0	0	0	0	0	0	0
0	-0.000788658	0	0	0	-0.10617345	0	0.020577154	0
0	-0.009645345	0	0	0	0	0	-0.016515134	0
0	0	0	0	0	0	0	-0.015350461	0
0	0	0	0	0	0	0	0	0
0	-0.002993914	0	0	0	0	0	0	0
0	0	0	0	0	0	0	-0.014629649	0
0	0	0	0	0	0	0	-0.011140004	0
0	0	0	-0.031565077	0	-0.078934065	0	0.029148535	0
0	0.001445758	0	-0.032230484	0	-0.078958293	0	0.027705199	0
0	0	0	-0.10376554	0	0	0	0.021096776	0
0	-0.000818551	0	-0.10427966	0	0	0	0.019313903	0
0	-1.0898177	0	0	0	0	0	0	0
0	-0.007132651	0	-0.030310092	0	-0.076148811	0	0	0
0	0	0	-0.030221848	0	-0.075670854	0	0	0
0	0	0	0	0	0	0	-0.010603352	0
0	-0.014617446	0	0	0	0	0	-0.019187608	0
0	0	0	0	0	0	0	-0.014844176	0
0	0	0	0	0	0	0	-0.011494307	0
0	-1.0657182	0	0	0	0	0	0.014353683	0
0	-1.0949341	0	0	0	0	0	0.012078206	0
0	-0.013006155	0	0	0	0	0	0	0
0	0	0	0	0	0	0	-0.017238195	0
0	-1.0986287	0	0	0	0	0	0	0
0	0	0	0	0	0	0	0	0
0	-1.0641871	0	0	0	0	0	0	0
0	-1.1003066	0	0	0	0	0	0	0
0	-1.0628579	0	0	0	0	0	0	0
0	0	0	-0.023265431	0	-0.085632414	0	0.031061985	0
0	0.002330049	0	-0.023653037	0	-0.0858164	0	0.029406602	0
0	-1.0636003	0	0	0	0	0	0.023948523	0
0	-1.0645061	0	0	0	0	0	0.021857963	0
0	-0.012392918	0	0	0	0	0	-0.021455075	0
0	-0.008346797	0	0	0	0	0	-0.019854926	0
0	0	0	0	0	0	0	-0.017851699	0
0	0	0	0	0	0	0	-0.019417579	0
0	-1.0603674	0	0	0	0	0	0	0
0	-1.1194374	0	0	0	0	0	0.018748995	0
0	-1.1312242	0	0	0	0	0	0	0
0	-1.1324253	0	0	0	0	0	0	0
0	-1.123364	0	0	0	0	0	0	0
0	-1.1511682	0	0	0	0	0	0.028978016	0
0	-1.1311766	0	0	0	0	0	0.017369935	0
0	-1.0733035	0	0	0	0	0	0.035561041	0

0	-1.0700312	0	0	0	0	0	0.047052594	0
0	-1.0759498	0	0	0	0	0	0.040150892	0
0	-1.0668891	0	0	0	0	0	0	0
0	0	0	0	0	0	0	0.010555288	0
0	-0.014940789	0	0	0	0	0	0	0
0	0	0	0	0	0	0	0	0
0	0	0	0	0	0	0	0.009570798	0
0	0	0	0	0	0	0	0	0
0	0	0	0	0	0	0	0	0
0	0	0	0	0	0	0	0.012969833	0
0	0	0	0	0	0	0	0.011817706	0
0	0	0	0	0	0	0	0.014492462	0
0	0.007179105	0	0	0	0	0	0.007848851	0
0	-0.004345474	0	0	0	0	0	0.014534713	0
0	0	0	0	0	0	0	0.005995528	0
0	0.005876063	0	0	0	0	0	0	0
0	0	0	0	0	0	0	0	0
0	-0.005833659	0	0	0	0	0	0	0
0	0.009973655	0	0	0	0	0	0.010578297	0
0	0	0	0	0	0	0	0.013867795	0
0	0	0	0	0	0	0	0.014184852	0
0	0	0	0	0	0	0	0.014441524	0
0	-0.003480966	0	0	0	0	0	0.014045728	0
0	0.006873307	0	0	0	0	0	0.012261269	0
0	0	0	0	0	0	0	0	0
0	-0.006432353	0	0	0	0	0	0	0
0	0	0	0	0	0	0	0.020985937	0
0	0.010578091	0	0	0	0	0	0.021808792	0
0	-0.040024933	0	0	0	0	0	0	0
0	-0.026926383	0	0	0	0	0	0.022068188	0
0	0	0	0	0	0	0	0.021810156	0
0	-0.044566511	0	0	0	0	0	0.018859573	0
0	0	0	0	0	0	0	0.023188275	0
0	0	0	0	0	0	0	0	0
0	0	0	0	0	0	0	0.019260291	0
0	0	0	0	0	0	0	0.020476136	0
0	0.000611637	0	0	0	0	0	0.017503397	0
0	-0.022123284	0	0	0	0	0	0.035953861	0
0	0	0	0	0	0	0	0.036705894	0
0	-1.0230184	0	0	0	-0.13528468	0	0	0
0	-1.0230037	0	-0.12950534	0	0	0	0	0
0	-1.0674546	0	0	0	-0.16481848	0	0	0
0	-1.098465	0	0	0	-0.13799299	0	0	0
0	-1.0966682	0	0	0	-0.12826459	0	0	0
0	-1.0971413	0	0	0	-0.13005576	0	0	0
0	-1.0273812	0	0	0	-0.14455451	0	0	0
0	-1.0950944	0	0	0	-0.12710656	0	-0.013685724	0

0	-1.0973517	0	0.005955968	0	-0.13335414	0	0	0
0	-1.1011037	0	0	0	-0.15452303	0	0	0
0	-1.1012126	0	-0.009467877	0	-0.14283932	0	0	0
0	-1.100912	0	0	0	-0.15237377	0	-0.001335711	0
0	-1.1010795	0	0	0	-0.15211913	0	0	0
0	-1.057737	0	0	0	-0.15863123	0	0	0
0	-1.0536476	0	-0.021902113	0	-0.13484892	0	0	0
0	-1.0679824	0	-0.15844277	0	0	0	0	0
0	-1.0534931	0	0	0	-0.15601548	0	0.001129777	0
0	-1.0538245	0	0	0	-0.15606766	0	0	0
0	-1.0978025	0	-0.13122053	0	0	0	0	0
0	-1.0965632	0	-0.12443386	0	0	0	0	0
0	-1.0273175	0	-0.13887319	0	0	0	0	0
0	-1.0960591	0	-0.12222938	0	0	0	0	0
0	-1.0943139	0	-0.12141559	0	0	0	-0.014809419	0
0	-1.1006887	0	-0.14777336	0	0	0	0	0
0	-0.10462273	0	0	0	-0.14741144	0	0	0
0	-1.1004064	0	-0.14645558	0	0	0	-0.002150057	0
0	-1.1006017	0	-0.14595472	0	0	0	0	0
0	-0.11962113	0	0	0	-0.14055207	0	0	0
0	-0.12505621	0	0	0	-0.13798574	0	0	0
0	-1.0583223	0	-0.15278231	0	0	0	0	0
0	-1.0553028	0	-0.15087509	0	0	0	0	0
0	-1.0552055	0	-0.15094944	0	0	0	0.000392406	0
0	-1.1022101	0	0	0	-0.14432515	0	0	0
0	-0.12831138	0	0	0	-0.13691754	0	-0.010742033	0
0	-0.12515449	0	0.001347558	0	-0.13874007	0	0	0
0	-1.1021634	0	0	0	-0.14215496	0	-0.00773717	0
0	-1.1028576	0	0	0	-0.13748768	0	0	0
0	-1.1033722	0	-0.008714173	0	-0.13218864	0	0	0
0	0	0	0	0	-0.14670983	0	0	0
0	-1.1017874	0	-0.011456283	0	-0.12358697	0	-0.013714467	0
0	-1.1015728	0	0	0	-0.13469973	0	-0.013508824	0
0	-1.1016683	0	0	0	-0.13483783	0	-0.013509623	0
0	-1.098353	0	0	0	-0.12105796	0	0	0
0	-1.104153	0	-0.010094497	0	-0.12564115	0	0	0
0	-1.1039261	0	0	0	-0.13521978	0	0	0
0	-0.100898	0	-0.14044893	0	0	0	0	0
0	-1.1036358	0	-0.007857266	0	-0.12269021	0	0	0
0	-0.11412052	0	-0.13481874	0	0	0	0	0
0	0	0	0	0	-0.13816729	0	0	0
0	-1.1017583	0	-0.1389466	0	0	0	0	0
0	-1.0274049	0	0	0	-0.11668636	0	0	0
0	-0.11957593	0	-0.13182249	0	0	0	0	0
0	0	0	0	0	-0.13536987	0	0	0
0	-0.12333992	0	-0.13103176	0	0	0	-0.011997843	0
0	-1.101597	0	-0.13689593	0	0	0	-0.008499528	0

0	0	0	0	0	-0.1343775	0	-0.009272204	0
0	-1.102244	0	-0.1312901	0	0	0	0	0
0	0	0	0.000803882	0	-0.13559344	0	0	0
0	-1.1010642	0	-0.1306863	0	0	0	-0.013459129	0
0	-1.0979127	0	-0.11678566	0	0	0	0	0
0	-1.1008774	0	-0.12925354	0	0	0	-0.014699781	0
0	-1.0271632	0	-0.113786	0	0	0	0	0
0	-1.1032314	0	-0.12938125	0	0	0	0	0
0	0	0	-0.13990034	0	0	0	0	0
0	0	0	-0.13258475	0	0	0	0	0
0	-0.10934712	0	0	0	-0.13628372	0	0	0
0	0	0	-0.12934354	0	0	0	0	0
0	0	0	0	0	-0.14513803	0	0	0
0	0	0	-0.12862052	0	0	0	-0.010495529	0
0	-1.1065794	0	0	0	-0.15054662	0	0.025813735	0
0	0	0	0	0	-0.1437683	0	0	0
0	0	0	-0.012070517	0	-0.13178119	0	0	0
0	0	0	0	0	-0.14351452	0	0	0
0	0	0	0	0	-0.14341748	0	-0.000280818	0
0	-0.10182698	0	0	0	-0.13851903	0	0	0
0	-1.1238829	0	0	0	-0.1474522	0	0	0
0	-0.10873501	0	0	0	-0.13621644	0	-0.015939122	0
0	-0.12547933	0	0	0	-0.12881943	0	-0.015024505	0
0	0	0	0	0	-0.12846803	0	0	0
0	-0.12155286	0	0	0	-0.13041146	0	-0.016582042	0
0	-0.058776151	0	0	0	-0.16797062	0	0.007791168	0
0	-0.059351051	0	-0.019302695	0	-0.14957394	0	0	0
0	-0.060018526	0	0	0	-0.16802737	0	0	0
0	-0.059590314	0	0	0	-0.16829569	0	0	0
0	-0.1049555	0	0	0	-0.13634627	0	0	0
0	-0.103749	0	0.004439889	0	-0.14117644	0	0	0
0	-0.010296428	0	0	0	-0.14633148	0	0	0
0	-0.12103403	0	0.010745347	0	-0.13982476	0	0	0
0	0	0	0	0	-0.1453405	0	-0.005847468	0
0	0	0	-0.012661325	0	-0.13357338	0	0	0
0	0	0	0	0	-0.14596598	0	0	0
0	-1.0357725	0	0	0	-0.10285528	0	0	0
0	-0.12595524	0	0.00624937	0	-0.13418873	0	-0.016259668	0
0	-0.11670245	0	0.005129917	0	-0.13590715	0	0	0
0	-1.1042063	0	-0.037667569	0	-0.11906878	0	0	0
0	0	0	0	0	-0.13376786	0	0	0
0	-1.106034	0	-0.14563758	0	0	0	0.023846708	0
0	0	0	-0.13875469	0	0	0	0	0
0	-0.028235108	0	0	0	-0.13007591	0	0	0
0	-1.1038327	0	0	0	-0.15467535	0	0	0
0	-1.1002351	0	0	0	-0.10804382	0	-0.016104503	0
0	-0.10417191	0	-0.12988893	0	0	0	0	0

0	-1.0352725	0	0	0	-0.1016565	0	-0.011429188	0
0	-0.013417611	0	0	0	-0.14385062	0	-0.010750524	0
0	-1.0358016	0	-0.009303663	0	-0.093063776	0	0	0
0	0	0	0	0	-0.1658018	0	0.008303398	0
0	-1.1031786	0	-0.01056654	0	-0.098181582	0	0	0
0	0	0	0	0	-0.12919252	0	0	0
0	-0.011332484	0	0	0	-0.14384275	0	0	0
0	0	0	-0.13770933	0	0	0	0	0
0	0	0	-0.019850382	0	-0.14689519	0	0	0
0	-0.009874895	0	-0.016062425	0	-0.12896086	0	0	0
0	0	0	0	0	-0.14360072	0	-0.008870833	0
0	0	0	-0.016789122	0	-0.12720483	0	-0.007618869	0
0	0	0	0	0	-0.14297972	0	-0.007415202	0
0	0	0	0	0	-0.1662084	0	0	0
0	0	0	0	0	-0.16621467	0	0	0
0	-1.1220996	0	-0.14295933	0	0	0	0	0
0	0	0	-0.01657321	0	-0.12748594	0	0	0
0	0	0	0	0	-0.14361386	0	0	0
0	0	0	-0.13739285	0	0	0	-0.001898821	0
0	0	0	-0.01606368	0	-0.12860017	0	0	0
0	0	0	-0.13736808	0	0	0	0	0
0	0	0	0	0	-0.13769584	0	0	0
0	-1.035398	0	-0.099904975	0	0	0	0	0
0	0	0	-0.12409068	0	0	0	0	0
0	-1.099658	0	-0.10513082	0	0	0	-0.016971186	0
0	0	0	-0.011882314	0	-0.11517265	0	0	0
0	0	0	0	0	-0.12651147	0	-0.001693514	0
0	-1.03489	0	-0.098998762	0	0	0	-0.01226611	0
0	-0.098440175	0	-0.13113991	0	0	0	0	0
0	0	0	0	0	-0.13541647	0	-0.01423107	0
0	-0.10566247	0	-0.12937383	0	0	0	-0.017185109	0
0	-0.11666058	0	-0.12471632	0	0	0	-0.017749842	0
0	-0.07192441	0	0	0	-0.156414	0	0	0
0	-0.12061483	0	-0.12270982	0	0	0	-0.016296379	0
0	-0.072412485	0	-0.008783411	0	-0.147094	0	0	0
0	-0.07240728	0	0	0	-0.1557622	0	-0.000482764	0
0	-1.1036566	0	-0.14980322	0	0	0	0	0
0	-0.074902797	0	-0.009056797	0	-0.14545838	0	-0.00181218	0
0	-0.075260109	0	-0.008769458	0	-0.14532829	0	0	0
0	-0.075109957	0	0	0	-0.15424303	0	-0.001664799	0
0	0	0	0	0	-0.16133152	0	0.01041988	0
0	0	0	-0.019498647	0	-0.14094269	0	0.009265072	0
0	0	0	0	0	-0.13564285	0	0	0
0	-0.027176944	0	-0.12585359	0	0	0	0	0
0	0	0	0	0	-0.16014108	0	0.009445647	0
0	0	0	0.002910968	0	-0.13882926	0	0	0
0	-0.009723807	0	-0.14013005	0	0	0	0	0

0	0	0	-0.13916383	0	0	0	-0.007344045	0
0	0	0	-0.020937864	0	-0.14098731	0	0	0
0	0	0	-0.020743937	0	-0.1403155	0	0	0
0	0	0	-0.13978057	0	0	0	0	0
0	0	0	0	0	-0.16133301	0	0	0
0	0	0	0	0	-0.12594528	0	-0.013458921	0
0	-0.10197081	0	-0.12926343	0	0	0	0	0
0	0	0	0	0	-0.12775932	0	-0.015044652	0
0	-0.054355252	0	-0.16146023	0	0	0	0.006519498	0
0	-0.056207961	0	-0.16119246	0	0	0	0	0
0	-0.055459582	0	-0.1617037	0	0	0	0	0
0	0	0	0.010203756	0	-0.13635583	0	0	0
0	0	0	-0.12499681	0	0	0	0	0
0	0	0	-0.12754127	0	0	0	0	0
0	0	0	0.004257179	0	-0.13234829	0	0	0
0	-0.01333505	0	-0.13819147	0	0	0	-0.012051088	0
0	0	0	0.005885529	0	-0.13095338	0	-0.014708826	0
0	-0.011258603	0	-0.1380487	0	0	0	0	0
0	0	0	-0.13720068	0	0	0	-0.008672945	0
0	0	0	-0.13793975	0	0	0	-0.01016936	0
0	-0.009669248	0	0	0	-0.16128613	0	0	0
0	0	0	0	0	-0.16044487	0	0.005552231	0
0	0	0	-0.022952626	0	-0.13817179	0	0	0
0	-0.007483359	0	-0.022197869	0	-0.13895539	0	0	0
0	0	0	-0.13781496	0	0	0	0	0
0	-0.006617078	0	0	0	-0.16041767	0	0.002757817	0
0	0	0	-0.02143921	0	-0.13909238	0	0.003950562	0
0	0	0	0	0	-0.1606208	0	0	0
0	0	0	-0.022127079	0	-0.13849893	0	0	0
0	0	0	0	0	-0.15982236	0	0.004443614	0
0	-0.008117796	0	-0.023018365	0	-0.13604311	0	0	0
0	0	0	-0.12241871	0	0	0	-0.003083506	0
0	-0.00730417	0	-0.022538864	0	-0.13656297	0	0.00087463	0
0	-0.00771623	0	0	0	-0.15830921	0	0.00110316	0
0	0	0	-0.022377759	0	-0.13600608	0	0.002418111	0
0	0	0	-0.022201009	0	-0.13620936	0	0.002483095	0
0	0	0	-0.022758995	0	-0.13583061	0	0	0
0	0	0	0	0	-0.15769848	0	0.002698701	0
0	0	0	0	0	-0.15363765	0	0	0
0	0	0	-0.010468404	0	-0.10978572	0	0	0
0	0	0	0	0	-0.12011512	0	0.002079132	0
0	0	0	-0.008448615	0	-0.14458579	0	0	0
0	0	0	0	0	-0.15290508	0	-0.000302372	0
0	0	0	-0.15940987	0	0	0	0.00704248	0
0	0	0	-0.008714375	0	-0.14232929	0	-0.002135396	0
0	0	0	0	0	-0.15083536	0	-0.002001618	0
0	0	0	-0.008187948	0	-0.14242855	0	0	0

0	0	0	-0.15948369	0	0	0	0	0
0	0	0	-0.15973675	0	0	0	0	0
0	0	0	-0.13047702	0	0	0	0	0
0	0	0	-0.12872311	0	0	0	-0.015460324	0
0	-1.1083665	0	0	0	-0.13811372	0	0.023653576	0
0	-1.1020864	0	0	0	-0.095984285	0	-0.01432226	0
0	-1.1119924	0	0	0	-0.14313085	0	0.032961156	0
0	0	0	-0.12222209	0	0	0	-0.01617654	0
0	0	0	-0.12871087	0	0	0	0	0
0	0	0	-0.1199797	0	0	0	-0.014690844	0
0	0	0	-0.15519965	0	0	0	0.008682794	0
0	-0.068015809	0	-0.1496951	0	0	0	0	0
0	-1.1409773	0	0	0	-0.13687536	0	0	0
0	0	0	-0.15445801	0	0	0	0.008121675	0
0	-0.068610246	0	-0.14906899	0	0	0	-0.001802276	0
0	-0.07065347	0	-0.14812812	0	0	0	-0.002468659	0
0	-1.1045368	0	-0.000832122	0	-0.095578841	0	0	0
0	0	0	0	0	-0.13363417	0	0	0
0	0	0	-0.1553775	0	0	0	0	0
0	-1.1027629	0	-0.005501024	0	-0.089731259	0	-0.015768621	0
0	0	0	-0.11638422	0	0	0	0.000544181	0
0	0	0	0	0	-0.11406875	0	0.004605867	0
0	0	0	-0.001836719	0	-0.11178985	0	0	0
0	-1.1217699	0	0	0	-0.13555438	0	0	0
0	0	0	0	0	-0.13469726	0	-0.004519622	0
0	0	0	-0.001334443	0	-0.1336747	0	0	0
0	-0.009152513	0	-0.15550756	0	0	0	0	0
0	-1.1013727	0	-0.093005006	0	0	0	-0.015199346	0
0	0	0	-0.15468165	0	0	0	0.004273558	0
0	0	0	-0.15485075	0	0	0	0	0
0	-1.1063228	0	-0.040785849	0	-0.10105194	0	0	0
0	-0.006363682	0	-0.15458397	0	0	0	0.001474617	0
0	-1.1078971	0	-0.13456212	0	0	0	0.021684562	0
0	-1.1085937	0	-0.02996442	0	-0.098709508	0	0.014129705	0
0	-0.007692619	0	-0.15314725	0	0	0	0.000367885	0
0	0	0	-0.15398768	0	0	0	0.003167668	0
0	0	0	-0.009229963	0	-0.13554495	0	0	0
0	0	0	-0.003938193	0	-0.10953071	0	0.0034145	0
0	0	0	0	0	-0.14443194	0	0.001702055	0
0	0	0	-0.1525271	0	0	0	0.001970049	0
0	-1.1069171	0	0	0	-0.1285424	0	0.015455128	0
0	0	0	-0.007507073	0	-0.13611228	0	0.000360684	0
0	-1.153835	0	0	0	-0.13991544	0	0.032449306	0
0	0	0	-0.14703049	0	0	0	0	0
0	-0.016571672	0	0	0	-0.13387104	0	0	0
0	-1.1130627	0	-0.03348505	0	-0.096568982	0	0	0
0	0	0	-0.14631681	0	0	0	-0.001594208	0

0	-1.1113643	0	-0.13830925	0	0	0	0.030691754	0
0	0	0	-0.14483187	0	0	0	-0.002783435	0
0	0	0	0	0	-0.13309641	0	-0.005987285	0
0	0	0	-0.005299662	0	-0.12758278	0	-0.006067235	0
0	0	0	0	0	-0.13295348	0	-0.009587499	0
0	-1.138833	0	-0.13245299	0	0	0	0	0
0	0	0	-0.004066939	0	-0.12949693	0	0	0
0	-1.1200089	0	-0.13250351	0	0	0	0	0
0	0	0	-0.00718431	0	-0.13100273	0	-0.003238074	0
0	0	0	0	0	-0.13365184	0	0	0
0	0	0	-0.005607789	0	-0.12804233	0	0	0
0	-0.025634104	0	0	0	-0.11638296	0	-0.01355986	0
0	-0.014262654	0	0	0	-0.1444222	0	-0.006610874	0
0	0	0	-0.10994304	0	0	0	0.003003514	0
0	0	0	0	0	-0.11927355	0	-0.001337485	0
0	0	0	-0.000558273	0	-0.11885486	0	0	0
0	-0.012662429	0	-0.011473061	0	-0.13228173	0	0	0
0	-1.106339	0	-0.12605805	0	0	0	0.014313894	0
0	-0.02121386	0	-0.016966496	0	-0.10048757	0	0	0
0	0	0	0	0	-0.14335368	0	-0.005251457	0
0	0	0	-0.12723191	0	0	0	0	0
0	0	0	-0.011824435	0	-0.13109319	0	-0.004473158	0
0	0	0	-0.011067274	0	-0.13179808	0	0	0
0	0	0	0	0	-0.11571999	0	-0.011716824	0
0	-0.012864252	0	0	0	-0.13514624	0	-0.014779055	0
0	-1.1094196	0	-0.036913313	0	-0.10938113	0	0	0
0	0	0	-0.016815793	0	-0.099900837	0	0	0
0	0	0	0	0	-0.13487927	0	-0.012733414	0
0	0	0	-0.006419776	0	-0.12853822	0	-0.011721158	0
0	-0.010713507	0	-0.010253205	0	-0.13135672	0	-0.010245305	0
0	0	0	-0.017236041	0	-0.09758097	0	-0.007619948	0
0	0	0	-0.128246	0	0	0	-0.006141299	0
0	-0.007957978	0	-0.004968047	0	-0.13104027	0	0	0
0	0	0	-0.009720301	0	-0.13102159	0	-0.008628119	0
0	0	0	-0.005019162	0	-0.130632	0	0	0
0	-0.029597128	0	0	0	-0.11951031	0	-0.014589054	0
0	-0.025323285	0	-0.11354337	0	0	0	-0.014503891	0
0	-1.1529101	0	-0.13486071	0	0	0	0.030910736	0
0	0	0	-0.13889526	0	0	0	0.000487872	0
0	-0.024890123	0	-0.00447914	0	-0.1157733	0	0	0
0	-0.01629461	0	-0.12772029	0	0	0	0	0
0	-0.013508506	0	-0.011360973	0	-0.1230071	0	-0.01579513	0
0	-0.01399313	0	0	0	-0.13259891	0	-0.016772433	0
0	0	0	-0.11288141	0	0	0	-0.012655735	0
0	-0.013042553	0	-0.010341353	0	-0.12304129	0	-0.01670202	0
0	0	0	0	0	-0.11865341	0	-0.012654463	0
0	0	0	-0.12689076	0	0	0	-0.010863892	0

0	-1.171533	0	-0.020013435	0	-0.11940056	0	0	0
0	0	0	-0.12687581	0	0	0	-0.007479397	0
0	0	0	-0.01064519	0	-0.1227101	0	-0.01436173	0
0	0	0	-0.010151337	0	-0.12295414	0	-0.014657076	0
0	0	0	0	0	-0.13250141	0	-0.014678129	0
0	0	0	-0.010238103	0	-0.12254253	0	-0.013521628	0
0	0	0	-0.12751214	0	0	0	0	0
0	0	0	-0.11463397	0	0	0	-0.00297614	0
0	0	0	-0.004390157	0	-0.11492499	0	0	0
0	-0.008275937	0	-0.00894957	0	-0.12469279	0	0	0
0	0	0	-0.008820992	0	-0.12462992	0	0	0
0	-0.013877948	0	-0.13919694	0	0	0	-0.007411921	0
0	0	0	-0.005200649	0	-0.11162038	0	-0.00782877	0
0	0	0	-0.1381272	0	0	0	-0.006041184	0
0	-0.01277929	0	-0.12902627	0	0	0	-0.016130188	0
0	-0.028965534	0	-0.11527943	0	0	0	-0.015749569	0
0	0	0	-0.12875927	0	0	0	-0.01408592	0
0	0	0	-0.11443351	0	0	0	-0.01380633	0
0	-0.014347194	0	-0.12688963	0	0	0	-0.018002841	0
0	-0.090393329	0	-0.024372401	0	-0.12858566	0	0.024611158	0
0	-0.091808958	0	0	0	-0.15073526	0	0.022733701	0
0	-0.088954534	0	0	0	-0.15340487	0	0.026187894	0
0	0	0	-0.12676404	0	0	0	-0.01591031	0
0	-1.1196946	0	-0.029550976	0	-0.12574197	0	0.062884523	0
0	0	0	-0.003293178	0	-0.1076396	0	-0.003614212	0
0	-0.096126753	0	-0.027272908	0	-0.12410977	0	0	0
0	0	0	0.004782113	0	-0.11075429	0	-0.00084562	0
0	-0.096220264	0	0	0	-0.15066957	0	0	0
0	-0.017281396	0	0	0	-0.10207367	0	-0.012283366	0
0	-0.013270058	0	-0.006063957	0	-0.096685078	0	0	0
0	0	0	0	0	-0.10165101	0	-0.010405029	0
0	-0.094239109	0	-0.035145149	0	-0.12142552	0	0	0
0	0	0	-0.006953732	0	-0.094413566	0	-0.008185927	0
0	0	0	-0.006058078	0	-0.096194421	0	0	0
0	0	0	-0.024211839	0	-0.12530413	0	0.024865906	0
0	0	0	0	0	-0.15053538	0	0.027064156	0
0	-0.017361933	0	-0.010211616	0	-0.091382184	0	-0.013623945	0
0	-0.087163014	0	-0.1458482	0	0	0	0.021051383	0
0	0	0	0	0	-0.14793593	0	0.023898464	0
0	-0.084916003	0	-0.14803729	0	0	0	0.023853343	0
0	0	0	-0.010006234	0	-0.091157935	0	-0.011746286	0
0	-0.017067865	0	-0.09917061	0	0	0	-0.013220302	0
0	0	0	-0.0987489	0	0	0	-0.011339425	0
0	-0.091644795	0	-0.14591892	0	0	0	0	0
0	0	0	-0.028040612	0	-0.12054612	0	0	0
0	0	0	0	0	-0.14812684	0	0	0
0	-0.024382804	0	-0.011352804	0	-0.095753606	0	-0.019270464	0

0	0	0	-0.035542961	0	-0.11826035	0	0	0
0	0	0	-0.010991979	0	-0.095383817	0	-0.017262322	0
0	0	0	-0.14530054	0	0	0	0.024773821	0
0	0	0	-0.14319607	0	0	0	0.022240788	0
0	0	0	-0.14354606	0	0	0	0	0
0	-0.10720789	0	0	0	-0.14052491	0	0.024107578	0
0	-0.10504107	0	-0.022766227	0	-0.12234957	0	0.031061273	0
0	0	0	-0.025413884	0	-0.11303146	0	0.030790031	0
0	0	0	0	0	-0.13783547	0	0.031270229	0
0	-1.0145063	0	0	0	0	0	0	0
0	0	0	-0.025473653	0	-0.10919955	0	0.028463893	0
0	-0.11334118	0	-0.021814256	0	-0.11821551	0	0	0
0	-1.0125615	0	0	0	0	0	-0.014775667	0
0	-1.0131126	0	0	0	0	0	0	0
0	-0.10294802	0	-0.13561377	0	0	0	0.022291931	0
0	-1.0129982	0	0	0	0	0	0	0
0	0	0	-0.13402205	0	0	0	0.029187131	0
0	-0.018295957	0	-0.031281329	0	-0.10705577	0	0.021080726	0
0	-0.016727112	0	-0.00135908	0	-0.093031487	0	-0.017423819	0
0	-0.018175299	0	0	0	-0.13730315	0	0.021306193	0
0	0	0	0	0	-0.13759899	0	0.025644675	0
0	0	0	-0.03062163	0	-0.10659514	0	0.022577558	0
0	0	0	-0.001133587	0	-0.092794798	0	-0.015396418	0
0	-1.0612908	0	0	0	0	0	-0.01774446	0
0	0	0	0	0	-0.13637159	0	0.023049075	0
0	0	0	-0.030210348	0	-0.10604057	0	0.024644582	0
0	0	0	-0.031865052	0	-0.10429188	0	0	0
0	-1.1409518	0	0	0	-0.10627625	0	0.020372453	0
0	-1.0638169	0	0	0	0	0	0	0
0	0	0	-0.022733667	0	-0.11922878	0	0.032097756	0
0	-1.063468	0	0	0	0	0	0	0
0	-0.101844	0	-0.014282349	0	-0.12890949	0	0.033899091	0
0	-0.024723123	0	-0.035103894	0	-0.10256912	0	0	0
0	-1.0592535	0	0	0	0	0	-0.016661458	0
0	-1.1395288	0	-0.10429721	0	0	0	0.019103645	0
0	-1.1475676	0	-0.02900081	0	-0.077524752	0	0	0
0	-0.016909754	0	-0.03198956	0	-0.098968747	0	0.015630631	0
0	-0.017792286	0	-0.13396985	0	0	0	0.019670091	0
0	-1.059796	0	0	0	0	0	-0.017554807	0
0	0	0	-0.034974998	0	-0.10170532	0	0	0
0	0	0	-0.022450132	0	-0.11459276	0	0	0
0	-1.1193247	0	-0.032559367	0	-0.07986208	0	0.02957097	0
0	-1.0609489	0	0	0	0	0	0	0
0	0	0	-0.031521147	0	-0.098594085	0	0.017422932	0
0	0	0	-0.13304246	0	0	0	0.021412254	0
0	0	0	-0.13282991	0	0	0	0.023866331	0
0	-1.0142673	0	0	0	0	0	0	0

0	-1.1594515	0	-0.022721084	0	-0.086493651	0	0.028050193	0
0	0	0	-0.014663796	0	-0.12550388	0	0.035329428	0
0	-1.0171148	0	0	0	0	0	-0.01557217	0
0	-1.0177238	0	0	0	0	0	0	0
0	-1.018924	0	0	0	0	0	-0.015142726	0
0	-1.0193053	0	0	0	0	0	0	0
0	-1.0212294	0	0	0	0	0	-0.01440868	0
0	-1.0627338	0	0	0	0	0	-0.016298861	0
0	-1.0660441	0	0	0	0	0	0	0
0	-1.0661	0	0	0	0	0	-0.016897889	0
0	-1.0665458	0	0	0	0	0	-0.018434792	0
0	0	0	-0.019192505	0	-0.10171565	0	0.033465173	0
0	-0.095434923	0	0	0	0	0	0	0
0	-0.09559337	0	0	0	0	0	-0.016828835	0
0	-0.089177223	0	0	0	0	0	0	0
0	-0.067323147	0	0	0	0	0	-0.015885249	0
0	-0.067865075	0	0	0	0	0	-0.016665146	0
0	-0.058642747	0	0	0	0	0	0	0
0	-0.098168648	0	0	0	0	0	0	0
0	0	0	0	0	0	0	0	0
0	0	0	0	0	0	0	-0.015158933	0
0	0	0	0	0	0	0	0	0
0	-0.10174492	0	0	0	0	0	-0.020963432	0
0	0	0	0	0	0	0	-0.014187636	0
0	0	0	0	0	0	0	0	0
0	0	0	0	0	0	0	-0.015016692	0
0	-0.095565861	0	0	0	0	0	-0.019668392	0
0	0	0	0	0	0	0	0	0
0	0	0	0	0	0	0	0	0
0	0	0	0	0	0	0	-0.007710984	0
0	-0.065491456	0	0	0	0	0	-0.019218794	0
0	0	0	0	0	0	0	-0.019222957	0
0	-0.01499264	0	0	0	0	0	0	0
0	0	0	0	0	0	0	-0.01093187	0
0	-0.014730035	0	0	0	0	0	-0.015487876	0
0	0	0	0	0	0	0	-0.007160033	0
0	0	0	0	0	0	0	-0.017908481	0
0	0	0	0	0	0	0	-0.00789742	0
0	0	0	0	0	0	0	0	0
0	0	0	0	0	0	0	-0.013652247	0
0	0	0	0	0	0	0	-0.017393969	0
0	-0.013018851	0	0	0	0	0	-0.017715412	0
0	0	0	0	0	0	0	-0.015803558	0
0	-0.00838414	0	0	0	0	0	-0.016271299	0
0	0	0	0	0	0	0	-0.01437914	0
0	0	0	0	0	0	0	-0.009450929	0
0	0	0	-0.026136048	0	-0.080308976	0	0.02207547	0

0	-0.000899049	0	-0.026522791	0	-0.080441739	0	0.020294118	0
0	-1.0930446	0	0	0	0	0	0	0
0	-1.0879201	0	0	0	0	0	0.008279881	0
0	-0.019242339	0	0	0	0	0	-0.019092311	0
0	-1.0887555	0	0	0	0	0	0	0
0	0	0	0	0	0	0	-0.017165314	0
0	-1.0947219	0	0	0	0	0	0.012120741	0
0	-1.0662857	0	0	0	0	0	0.012698673	0
0	-1.0971539	0	0	0	0	0	0.013081326	0
0	-1.0653151	0	0	0	0	0	0.015326996	0
0	-1.1000598	0	0	0	0	0	0	0
0	-1.06377	0	0	0	0	0	0	0
0	-1.0642904	0	0	0	0	0	0.021856401	0
0	-1.1227204	0	0	0	0	0	0.020943893	0
0	-1.1249244	0	0	0	0	0	0	0
0	-1.1259787	0	0	0	0	0	0.018480366	0
0	-1.1173842	0	0	0	0	0	0.017303618	0
0	-1.0715437	0	0	0	0	0	0.03726205	0
0	-0.013563987	0	0	0	0	0	0.009934216	0
0	0	0	0	0	0	0	0.009790499	0
0	-0.015122904	0	0	0	0	0	0	0
0	0	0	0	0	0	0	0.010728805	0
0	0	0	0	0	0	0	0	0
0	0	0	0	0	0	0	0.008948494	0
0	0	0	0	0	0	0	0.013029622	0
0	-0.005637312	0	0	0	0	0	0.012940473	0
0	0.007842019	0	0	0	0	0	0.007414574	0
0	0	0	0	0	0	0	0.014449904	0
0	-0.005413724	0	0	0	0	0	0.014053637	0
0	-0.034987831	0	0	0	0	0	0.018483289	0
0	0	0	0	0	0	0	0.020037158	0
0	-1.0220523	0	0	0	-0.14711786	0	0	0
0	-1.0206121	0	0	0	-0.13853977	0	0	0
0	-1.0230878	0	0	0	-0.13584303	0	0	0
0	-1.0226628	0	0	0	-0.13486054	0	-0.009037915	0
0	-1.0230166	0	0.000276177	0	-0.13555301	0	0	0
0	-1.022137	0	-0.14032903	0	0	0	0	0
0	-1.020578	0	-0.13297111	0	0	0	0	0
0	-1.0230555	0	-0.12983438	0	0	0	0	0
0	-1.0226058	0	-0.12911576	0	0	0	-0.010263896	0
0	-1.0948218	0	0	0	-0.13554755	0	0	0
0	-1.0976818	0	0	0	-0.13965975	0	0	0
0	-1.0658247	0	0	0	-0.164488	0	0.007030681	0
0	-1.0961956	0	0	0	-0.13743798	0	-0.013158663	0
0	-1.0672608	0	-0.018954713	0	-0.14639034	0	0	0
0	-1.0679646	0	0	0	-0.16514168	0	0	0
0	-1.0674617	0	0	0	-0.16484279	0	0	0

0	-1.0272412	0	0	0	-0.14640233	0	0	0
0	-1.0983089	0	0	0	-0.13765381	0	0	0
0	-1.0984447	0	0.001291485	0	-0.13924273	0	0	0
0	-1.0945781	0	0	0	-0.12781709	0	-0.012408908	0
0	-1.0947328	0	0	0	-0.12962746	0	-0.01397946	0
0	-1.0965329	0	0.008611029	0	-0.13664366	0	0	0
0	-1.097095	0	0.00270855	0	-0.13268476	0	0	0
0	-1.0943175	0	-0.12935414	0	0	0	0	0
0	-1.0270477	0	0	0	-0.14405337	0	-0.008639885	0
0	-1.0216861	0	0	0	-0.1295935	0	0	0
0	-1.0268871	0	0	0	-0.14411868	0	0	0
0	-1.0274422	0	-0.016453114	0	-0.12866404	0	0	0
0	-1.0950317	0	0.004449701	0	-0.13143585	0	-0.013654575	0
0	-1.1008186	0	0	0	-0.15526261	0	0	0
0	-1.1012341	0	-0.009796215	0	-0.14491854	0	0	0
0	-1.101157	0	0	0	-0.15447499	0	0.000408391	0
0	-1.1010245	0	-0.009879526	0	-0.14270808	0	-0.00148389	0
0	-1.1012025	0	-0.009506332	0	-0.1427875	0	0	0
0	-1.1009059	0	0	0	-0.15236501	0	-0.001334388	0
0	-1.0970119	0	-0.13247488	0	0	0	0	0
0	-0.11218426	0	0	0	-0.14608779	0	0	0
0	-1.0271721	0	-0.14022859	0	0	0	0	0
0	-1.0953529	0	-0.13076417	0	0	0	-0.014404385	0
0	-1.0596752	0	0	0	-0.15937842	0	0	0
0	-1.0575326	0	-0.021287744	0	-0.13794268	0	0	0
0	-1.057024	0	0	0	-0.15850069	0	0.003229027	0
0	-1.0666584	0	-0.15810789	0	0	0	0.005780806	0
0	-1.0536408	0	-0.022053416	0	-0.13464336	0	0	0
0	-1.0533735	0	-0.021750263	0	-0.13491417	0	0.000926646	0
0	-1.0679495	0	-0.15836391	0	0	0	0	0
0	-1.0679038	0	-0.15839538	0	0	0	0	0
0	-1.0975657	0	-0.13074599	0	0	0	0	0
0	-1.0534862	0	0	0	-0.15596611	0	0.001137855	0
0	-1.0939972	0	-0.12411068	0	0	0	-0.015126917	0
0	-1.0216313	0	-0.12540774	0	0	0	0	0
0	-1.0937923	0	-0.12187082	0	0	0	-0.01365752	0
0	-1.026939	0	-0.13840062	0	0	0	-0.009941835	0
0	-1.0266092	0	-0.13833317	0	0	0	0	0
0	-0.10292285	0	0	0	-0.14882994	0	0	0
0	-0.10784216	0	0	0	-0.14681783	0	-0.010166945	0
0	-0.10664755	0	0	0	-0.14663363	0	0	0
0	-1.1003461	0	-0.14866435	0	0	0	0	0
0	-1.1005739	0	-0.14789098	0	0	0	-0.000905232	0
0	-0.10456505	0	-0.003139895	0	-0.1443771	0	0	0
0	-1.1003329	0	-0.14636741	0	0	0	-0.002135731	0
0	-0.12281813	0	0	0	-0.14004848	0	-0.010962134	0
0	0	0	0	0	-0.14387426	0	0	0

0	-1.1003699	0	0	0	-0.13568952	0	0	0
0	-0.11959165	0	-0.002613856	0	-0.13801791	0	0	0
0	-0.12789486	0	0	0	-0.13750474	0	-0.009612298	0
0	-1.0603597	0	-0.15361857	0	0	0	0	0
0	-0.12508542	0	0.003804355	0	-0.1416843	0	0	0
0	-0.10682392	0	-0.13976346	0	0	0	0	0
0	-1.05789	0	-0.15268114	0	0	0	0.001963658	0
0	-1.055176	0	-0.15083482	0	0	0	0.000415014	0
0	-1.1016294	0	0	0	-0.14495323	0	-0.004534555	0
0	-1.1023664	0	-0.012365518	0	-0.13222795	0	0	0
0	-0.12831286	0	0.000262581	0	-0.13717275	0	-0.010740346	0
0	-1.1008794	0	0	0	-0.13692426	0	-0.011670792	0
0	-1.1022864	0	-0.010878497	0	-0.13151406	0	-0.007899774	0
0	-1.1029558	0	-0.006551504	0	-0.13114688	0	0	0
0	0	0	0	0	-0.14816219	0	0	0
0	0	0	0	0	-0.14621161	0	-0.008534006	0
0	0	0	0	0	-0.14606979	0	0	0
0	-0.099326998	0	-0.14148274	0	0	0	0	0
0	0	0	-0.004595028	0	-0.14226924	0	0	0
0	-1.0964154	0	0	0	-0.12057213	0	-0.011612009	0
0	-1.1017021	0	-0.011779334	0	-0.12317991	0	-0.013677294	0
0	-1.1018228	0	-0.011527749	0	-0.12368636	0	-0.013586754	0
0	-1.1015509	0	0	0	-0.13459564	0	-0.013587026	0
0	-1.0996957	0	-0.12956884	0	0	0	0	0
0	-0.1045794	0	-0.13989778	0	0	0	-0.011549507	0
0	-0.11774621	0	-0.13437036	0	0	0	-0.012241598	0
0	-0.10346268	0	-0.13961043	0	0	0	0	0
0	-1.0984375	0	-0.005886523	0	-0.11535381	0	0	0
0	-1.1040722	0	-0.010284684	0	-0.12526569	0	0	0
0	0	0	0	0	-0.13773799	0	-0.00950222	0
0	0	0	-0.13768379	0	0	0	0	0
0	-1.0269211	0	0	0	-0.11626257	0	-0.011463684	0
0	0	0	-0.003463966	0	-0.13480898	0	0	0
0	-0.12288219	0	-0.13138124	0	0	0	-0.01099309	0
0	-1.1010918	0	-0.13973279	0	0	0	-0.005341958	0
0	-1.0274522	0	-0.017125742	0	-0.10013464	0	0	0
0	0	0	0	0	-0.13496817	0	-0.008132324	0
0	0	0	0.003269093	0	-0.13854672	0	0	0
0	-1.1000695	0	-0.13081259	0	0	0	-0.013036038	0
0	0	0	-0.000124438	0	-0.13425654	0	-0.009272993	0
0	-1.0958114	0	-0.11637404	0	0	0	-0.012780482	0
0	-1.026641	0	-0.11342481	0	0	0	-0.012406964	0
0	0	0	-0.14096086	0	0	0	0	0
0	-1.1006739	0	-0.12886319	0	0	0	-0.014838049	0
0	0	0	-0.13942747	0	0	0	-0.00990317	0
0	0	0	-0.13918128	0	0	0	0	0
0	0	0	-0.13219825	0	0	0	-0.010749575	0

0	0	0	0	0	-0.14373466	0	0	0
0	-0.11372719	0	0	0	-0.13567546	0	-0.013998671	0
0	0	0	-0.12896934	0	0	0	-0.009481055	0
0	-0.10942124	0	0.006428684	0	-0.14252717	0	0	0
0	-0.10845027	0	0	0	-0.13667618	0	0	0
0	0	0	-0.0090784	0	-0.13636507	0	0	0
0	0	0	0	0	-0.14522093	0	0.001097912	0
0	-1.1150711	0	0	0	-0.14759582	0	0.021667513	0
0	-0.019677343	0	0	0	-0.1443618	0	0	0
0	-1.1068231	0	-0.025216334	0	-0.12587357	0	0.025276863	0
0	-1.1066602	0	0	0	-0.15212581	0	0.027537983	0
0	0	0	0	0	-0.14412286	0	0	0
0	-0.1065281	0	0	0	-0.13782457	0	-0.014464634	0
0	0	0	0	0	-0.14352061	0	-0.003472597	0
0	0	0	-0.013369453	0	-0.13084483	0	0	0
0	0	0	-0.011989359	0	-0.13192497	0	0	0
0	0	0	-0.012095188	0	-0.13173328	0	-0.000364269	0
0	0	0	0	0	-0.14349485	0	-0.000276969	0
0	-0.1019514	0	0.007062058	0	-0.14535659	0	0	0
0	-1.1234698	0	-0.026592424	0	-0.12162466	0	0	0
0	-1.1250913	0	0	0	-0.14816939	0	0	0
0	-0.11023544	0	0	0	-0.13561216	0	-0.016129043	0
0	-0.10877982	0	0.002718528	0	-0.13884832	0	-0.015920204	0
0	-0.12553008	0	0.008916963	0	-0.13749851	0	-0.014955108	0
0	0	0	0	0	-0.12894854	0	0.004486818	0
0	0	0	-0.13742239	0	0	0	0	0
0	0	0	-0.008255674	0	-0.12047448	0	0	0
0	-0.058692494	0	-0.017948021	0	-0.15053046	0	0.007604523	0
0	-0.12158343	0	0.003223582	0	-0.13354178	0	-0.016558564	0
0	-0.058909925	0	0	0	-0.16792377	0	0.007794142	0
0	-0.058915808	0	0	0	-0.1678983	0	0.007727355	0
0	-0.013262923	0	0	0	-0.14575462	0	-0.009040412	0
0	-0.059923506	0	-0.019370592	0	-0.1491859	0	0	0
0	-0.05950886	0	-0.019424232	0	-0.14940202	0	0	0
0	-0.060024001	0	0	0	-0.16800654	0	0	0
0	-0.10502097	0	0.00391692	0	-0.14013861	0	0	0
0	-0.010286216	0	-0.012663588	0	-0.13410737	0	0	0
0	-1.0353484	0	0	0	-0.10245091	0	-0.010149458	0
0	0	0	0	0	-0.14550784	0	-0.007157354	0
0	-0.10904869	0	-0.12937875	0	0	0	-0.015358127	0
0	0	0	-0.01329061	0	-0.13249042	0	-0.005932597	0
0	0	0	0	0	-0.13323949	0	-0.012336636	0
0	0	0	-0.012651534	0	-0.13373661	0	0	0
0	-1.0358104	0	-0.006503807	0	-0.096552143	0	0	0
0	-1.1139755	0	-0.14280169	0	0	0	0.020015985	0
0	-1.1042648	0	-0.036745031	0	-0.11871456	0	0	0
0	-0.031016287	0	0	0	-0.12957845	0	-0.008762043	0

0	0	0	0	0	-0.13596439	0	0	0
0	-1.1060989	0	-0.1468871	0	0	0	0.02521872	0
0	0	0	0.005587675	0	-0.13919261	0	0	0
0	-0.028246161	0	-0.012211878	0	-0.11825942	0	0	0
0	0	0	-0.1387133	0	0	0	-0.00067804	0
0	-1.100398	0	-0.01233293	0	-0.096106898	0	-0.016189928	0
0	-0.019379461	0	-0.13827067	0	0	0	0	0
0	-0.10536769	0	-0.12938377	0	0	0	0	0
0	-1.0353157	0	-0.010428229	0	-0.091547134	0	-0.011494417	0
0	-0.015022306	0	0	0	-0.14316322	0	-0.010954244	0
0	0	0	-0.13804093	0	0	0	0	0
0	-0.013450626	0	-0.017087029	0	-0.12734208	0	-0.010853797	0
0	0	0	0	0	-0.12879624	0	-0.00698535	0
0	0	0	-0.018403093	0	-0.14791878	0	0.008111383	0
0	0	0	0	0	-0.16606516	0	0.008537748	0
0	0	0	0	0	-0.16581992	0	0.008302376	0
0	0	0	-0.13741626	0	0	0	-0.004875888	0
0	0	0	-0.012111234	0	-0.1174726	0	0	0
0	-0.011361608	0	-0.016803165	0	-0.12759227	0	0	0
0	0	0	0	0	-0.13708591	0	-0.012703078	0
0	0	0	0	0	-0.14306021	0	-0.009034629	0
0	0	0	-0.016912366	0	-0.12726095	0	-0.008972805	0
0	0	0	-0.019840668	0	-0.14691297	0	0	0
0	0	0	-0.019826346	0	-0.14692763	0	0	0
0	0	0	-0.017258771	0	-0.12629055	0	-0.007507922	0
0	0	0	0	0	-0.16620293	0	0	0
0	-1.0349336	0	-0.099548876	0	0	0	-0.01109037	0
0	-1.1230101	0	-0.14349004	0	0	0	0	0
0	0	0	-0.016649453	0	-0.12752102	0	0	0
0	0	0	-0.13725872	0	0	0	-0.001904161	0
0	-0.10362438	0	-0.13056789	0	0	0	-0.015874773	0
0	0	0	0.005688222	0	-0.14318934	0	0	0
0	0	0	-0.12434569	0	0	0	0.002718841	0
0	0	0	-0.01206517	0	-0.11482549	0	-0.001763804	0
0	0	0	0	0	-0.13498395	0	-0.014370071	0
0	-0.1077023	0	-0.12866506	0	0	0	-0.017431481	0
0	0	0	0.001391497	0	-0.13676364	0	-0.014221555	0
0	-0.071942564	0	-0.009985531	0	-0.14663998	0	0	0
0	-0.071901329	0	0	0	-0.15637526	0	0.000409649	0
0	-0.01318425	0	-0.13957483	0	0	0	-0.0105238	0
0	-0.030378623	0	-0.12537906	0	0	0	-0.010036067	0
0	-0.072438579	0	-0.008950128	0	-0.14700336	0	-0.000619287	0
0	0	0	-0.019020758	0	-0.14284103	0	0.01020698	0
0	-0.075152684	0	-0.009267594	0	-0.14517133	0	-0.001803081	0
0	0	0	0	0	-0.1613945	0	0.010555167	0
0	0	0	-0.019097525	0	-0.14157909	0	0.009242109	0
0	0	0	0.002556069	0	-0.1381183	0	0	0

0	0	0	-0.1393302	0	0	0	-0.008636096	0
0	0	0	-0.020938588	0	-0.14098793	0	0	0
0	0	0	0.008576845	0	-0.1342937	0	-0.013392494	0
0	0	0	0.002540706	0	-0.13022668	0	-0.015026256	0
0	0	0	-0.12709599	0	0	0	-0.013658013	0
0	-0.054873015	0	-0.16132149	0	0	0	0.00652889	0
0	-0.055321509	0	-0.16100573	0	0	0	0.006089409	0
0	0	0	-0.12460679	0	0	0	-0.008252784	0
0	-0.056203814	0	-0.16122795	0	0	0	0	0
0	-0.015405606	0	-0.13743172	0	0	0	-0.012301874	0
0	0	0	-0.12882422	0	0	0	0	0
0	0	0	-0.13730588	0	0	0	-0.010383808	0
0	-0.009739485	0	-0.02329744	0	-0.13866137	0	0	0
0	-0.008599242	0	0	0	-0.16117932	0	0.003993775	0
0	0	0	-0.022165519	0	-0.13891702	0	0.005369083	0
0	-0.006704888	0	-0.021754995	0	-0.13928346	0	0.002531327	0
0	0	0	-0.023019906	0	-0.13826522	0	0	0
0	0	0	0	0	-0.16047205	0	0.005495666	0
0	0	0	-0.021376729	0	-0.13905635	0	0.004221075	0
0	-0.007846774	0	-0.022873169	0	-0.13610447	0	0.00089324	0
0	0	0	-0.022353531	0	-0.13600086	0	0.002491541	0
0	-1.1327836	0	0	0	-0.13796216	0	0.023651956	0
0	0	0	-0.1299671	0	0	0	-0.014094756	0
0	0	0	-0.009756587	0	-0.14408454	0	0	0
0	0	0	0	0	-0.15357188	0	0.000669615	0
0	0	0	-0.01022298	0	-0.11021563	0	0.002001613	0
0	0	0	-0.008564912	0	-0.14452282	0	-0.000433075	0
0	0	0	-0.15932533	0	0	0	0.00704877	0
0	0	0	-0.15927628	0	0	0	0.006914441	0
0	0	0	-0.008778473	0	-0.1422422	0	-0.002132652	0
0	0	0	-0.15952455	0	0	0	0	0
0	0	0	-0.12816479	0	0	0	-0.015658846	0
0	-1.1086681	0	-0.031627124	0	-0.10727565	0	0.023050873	0
0	-1.1144631	0	0	0	-0.13627285	0	0.02109351	0
0	-1.102122	0	-0.00254542	0	-0.093513155	0	-0.01434164	0
0	-1.1122294	0	-0.023777485	0	-0.11989451	0	0.032502294	0
0	-1.14081	0	-0.02132235	0	-0.11616307	0	0	0
0	0	0	-0.15531594	0	0	0	0.008904114	0
0	-0.068065004	0	-0.1497845	0	0	0	-0.000831055	0
0	0	0	0	0	-0.13342262	0	-0.003833563	0
0	0	0	-0.002172167	0	-0.13153184	0	0	0
0	-1.121301	0	-0.033673381	0	-0.10293428	0	0	0
0	0	0	-0.001262817	0	-0.11284186	0	0.004596223	0
0	0	0	-0.001994642	0	-0.13276769	0	-0.004528024	0
0	-1.1312299	0	-0.13312551	0	0	0	0.021867817	0
0	-0.008414955	0	-0.15540575	0	0	0	0.002742236	0
0	0	0	-0.15469287	0	0	0	0.004251328	0

0	-1.1132676	0	-0.13288678	0	0	0	0.019454505	0
0	-1.1066406	0	-0.030644004	0	-0.098862753	0	0.01513654	0
0	0	0	-0.008862447	0	-0.1357657	0	0.00158223	0
0	-0.021213604	0	0	0	-0.13321445	0	-0.01426166	0
0	-1.1537562	0	-0.014166261	0	-0.12614095	0	0.032310607	0
0	0	0	-0.14708927	0	0	0	-0.000544634	0
0	-0.016570354	0	-0.005595164	0	-0.1284562	0	0	0
0	0	0	0	0	-0.13308038	0	-0.01228032	0
0	0	0	-0.004879487	0	-0.1283709	0	-0.006028395	0
0	0	0	-0.006784641	0	-0.12640977	0	-0.009629955	0
0	0	0	-0.005569552	0	-0.12823678	0	0	0
0	-0.025700897	0	-0.018304612	0	-0.098687478	0	-0.013674778	0
0	-0.014359771	0	-0.013035584	0	-0.13169293	0	-0.006780733	0
0	0	0	-0.000753141	0	-0.11854092	0	-0.001350131	0
0	0	0	-0.12701074	0	0	0	-0.005393579	0
0	0	0	-0.012309188	0	-0.1313336	0	-0.005411851	0
0	0	0	-0.017975638	0	-0.098342654	0	-0.011829244	0
0	-0.012877218	0	-0.006708935	0	-0.1286511	0	-0.014828768	0
0	0	0	-0.006512569	0	-0.12857358	0	-0.012781953	0
0	-0.029614985	0	-0.006184039	0	-0.11351566	0	-0.014635651	0
0	-0.021368323	0	-0.12718382	0	0	0	-0.015572789	0
0	0	0	-0.12703669	0	0	0	-0.013593465	0
0	-0.014034953	0	-0.010795993	0	-0.12214408	0	-0.0168501	0
0	0	0	-0.005816155	0	-0.11300873	0	-0.012696937	0
0	0	0	-0.010440893	0	-0.12238945	0	-0.014749906	0
0	-0.091694763	0	-0.022733063	0	-0.12865259	0	0.022478029	0
0	-0.090154182	0	0	0	-0.15170468	0	0.023659273	0
0	-0.088920381	0	-0.024743145	0	-0.12923476	0	0.025704315	0
0	-0.095997796	0	-0.027297642	0	-0.12417542	0	0	0
0	-0.017298226	0	-0.007405275	0	-0.094891417	0	-0.012335142	0
0	0	0	-0.007197136	0	-0.094670645	0	-0.010455691	0
0	0	0	-0.024791278	0	-0.12631818	0	0.026578918	0
0	0	0	0	0	-0.14923322	0	0.025147044	0
0	0	0	-0.023276696	0	-0.12532682	0	0.023636045	0
0	-0.085939401	0	-0.14652561	0	0	0	0.021714738	0
0	0	0	-0.028141854	0	-0.12081147	0	0	0
0	0	0	-0.1441989	0	0	0	0.023229064	0
0	-0.10715479	0	-0.017289529	0	-0.12370811	0	0.023917169	0
0	-1.0125669	0	0	0	0	0	0	0
0	-1.013974	0	0	0	0	0	-0.014949893	0
0	0	0	-0.02557119	0	-0.11302411	0	0.030974877	0
0	-1.0144714	0	0	0	0	0	0	0
0	-1.0609729	0	0	0	0	0	0	0
0	-1.012608	0	0	0	0	0	-0.01397526	0
0	-1.0115894	0	0	0	0	0	0	0
0	-1.0124316	0	0	0	0	0	-0.014793673	0
0	-1.0608467	0	0	0	0	0	-0.018245951	0

0	-0.018324768	0	-0.031221279	0	-0.10704971	0	0.020980378	0
0	0	0	-0.017624729	0	-0.12045659	0	0.025450485	0
0	-1.0607934	0	0	0	0	0	-0.016957956	0
0	0	0	-0.030746408	0	-0.10657778	0	0.022720391	0
0	-1.1407243	0	-0.02528194	0	-0.081743023	0	0.020100394	0
0	-1.0583828	0	0	0	0	0	-0.016453722	0
0	-1.0140499	0	0	0	0	0	0	0
0	-1.0138486	0	0	0	0	0	-0.013466964	0
0	-1.0171211	0	0	0	0	0	-0.015578376	0
0	-1.0188082	0	0	0	0	0	-0.014174514	0
0	-1.0635232	0	0	0	0	0	-0.016223292	0
0	-0.098805754	0	0	0	0	0	0	0
0	-0.10116673	0	0	0	0	0	-0.017345951	0
0	-0.094507892	0	0	0	0	0	-0.016189669	0
0	0	0	0	0	0	0	0	0
0	-0.10428594	0	0	0	0	0	-0.018945643	0
0	-0.063821696	0	0	0	0	0	-0.015673178	0
0	0	0	0	0	0	0	-0.015650109	0
0	0	0	0	0	0	0	-0.014470821	0
0	0	0	0	0	0	0	-0.013900298	0
0	0	0	0	0	0	0	-0.017249527	0
0	0	0	0	0	0	0	-0.00579825	0
0	-0.019931839	0	0	0	0	0	-0.01531174	0
0	0	0	0	0	0	0	-0.01350173	0
0	-1.091241	0	0	0	0	0	0.009652113	0
0	-1.0918226	0	0	0	0	0	0	0
0	-1.0872519	0	0	0	0	0	0.007858922	0
0	-1.0657889	0	0	0	0	0	0.013824472	0
0	-1.0968924	0	0	0	0	0	0.013090775	0
0	-1.1187148	0	0	0	0	0	0.018085324	0
0	-0.013829031	0	0	0	0	0	0.009115467	0
0	0	0	0	0	0	0	0.009954623	0
0	-1.0177278	0	0	0	-0.14417956	0	0	0
0	-1.0220695	0	0	0	-0.14857262	0	0	0
0	-1.0217342	0	0	0	-0.14663384	0	-0.008307205	0
0	-1.0213815	0	0	0	-0.14654325	0	0	0
0	-1.0220763	0	-0.005095639	0	-0.14219354	0	0	0
0	-1.0202364	0	0	0	-0.13812109	0	-0.009262128	0
0	-1.0206263	0	-0.003866561	0	-0.13479071	0	0	0
0	-1.0178298	0	-0.13802012	0	0	0	0	0
0	-1.0227754	0	0	0	-0.13545344	0	-0.007895337	0
0	-1.023074	0	0.002745371	0	-0.13851149	0	0	0
0	-1.0226663	0	-0.000629411	0	-0.13424877	0	-0.009041875	0
0	-1.0221432	0	-0.14139243	0	0	0	0	0
0	-1.0217702	0	-0.13986752	0	0	0	-0.009676616	0
0	-1.0212397	0	-0.13966674	0	0	0	0	0
0	-1.0201564	0	-0.13259293	0	0	0	-0.010512553	0

0	-1.0226945	0	-0.12946917	0	0	0	-0.009248082	0
0	-1.0929395	0	0	0	-0.13506075	0	-0.01129636	0
0	-1.0954238	0	0	0	-0.13796454	0	0	0
0	-1.0947604	0	0.004029516	0	-0.13946114	0	0	0
0	-1.0230669	0	0	0	-0.14461787	0	0	0
0	-1.0956878	0	0	0	-0.13909443	0	-0.011643268	0
0	-1.0975974	0	0.003947113	0	-0.14347927	0	0	0
0	-1.0656862	0	-0.017737897	0	-0.14725156	0	0.006845747	0
0	-1.0665662	0	0	0	-0.16501436	0	0.007498932	0
0	-1.0959928	0	0	0	-0.13704017	0	-0.013286582	0
0	-1.0658303	0	0	0	-0.16451065	0	0.007029374	0
0	-1.0961935	0	-9.79E-05	0	-0.13734349	0	-0.013159296	0
0	-1.026976	0	0	0	-0.14595992	0	-0.006928197	0
0	-1.067745	0	-0.01888803	0	-0.14676706	0	0	0
0	-1.0672648	0	-0.018923528	0	-0.14643426	0	0	0
0	-1.0680909	0	0	0	-0.16518001	0	0	0
0	-1.0272889	0	-0.013043846	0	-0.1337951	0	0	0
0	-1.0982939	0	0.000965311	0	-0.13858865	0	0	0
0	-1.0944668	0	0.007151111	0	-0.13477706	0	-0.012356717	0
0	-1.0947131	0	0.001113994	0	-0.13070806	0	-0.013972713	0
0	-1.0922413	0	-0.12893881	0	0	0	-0.01263346	0
0	-1.0228732	0	-0.13854148	0	0	0	0	0
0	-1.0214568	0	0	0	-0.12920877	0	-0.006781531	0
0	-1.0947026	0	-0.13085354	0	0	0	0	0
0	-1.0265155	0	0	0	-0.14358018	0	-0.008783383	0
0	-1.0271075	0	-0.017278107	0	-0.12736041	0	-0.008744147	0
0	-1.0217258	0	-0.012475149	0	-0.11752095	0	0	0
0	-1.026935	0	-0.016962286	0	-0.12772362	0	0	0
0	-1.100959	0	-0.011053376	0	-0.14444325	0	0	0
0	-1.1009852	0	0	0	-0.15513091	0	0.001364588	0
0	-1.1012674	0	-0.009726482	0	-0.14495627	0	0.000260187	0
0	-1.0948328	0	-0.13199593	0	0	0	-0.01305275	0
0	-1.101015	0	-0.009916301	0	-0.1426582	0	-0.001482377	0
0	-0.11472154	0	0	0	-0.14560472	0	-0.008220984	0
0	-0.11016357	0	0	0	-0.1469788	0	0	0
0	-1.0268561	0	-0.13979024	0	0	0	-0.008410712	0
0	-0.11216995	0	-0.001331458	0	-0.14479617	0	0	0
0	-1.0950587	0	-0.13023409	0	0	0	-0.014592905	0
0	-1.0595067	0	-0.022324096	0	-0.13770495	0	0	0
0	-1.0588419	0	0	0	-0.15925845	0	0.00445799	0
0	-1.0568815	0	-0.020760825	0	-0.13833224	0	0.003012057	0
0	-1.0666215	0	-0.15802779	0	0	0	0.00578628	0
0	-1.0668293	0	-0.15822053	0	0	0	0.005887901	0
0	-1.0213639	0	-0.12502838	0	0	0	-0.00805064	0
0	-1.0533643	0	-0.021901356	0	-0.13470763	0	0.000935024	0
0	-1.0682184	0	-0.15850432	0	0	0	0	0
0	-1.0261874	0	-0.13782374	0	0	0	-0.010137035	0

0	-0.10577432	0	0	0	-0.14826552	0	-0.008818476	0
0	-0.10291173	0	-0.000664732	0	-0.14818673	0	0	0
0	-0.11003301	0	0	0	-0.14599137	0	-0.010414635	0
0	-0.10777438	0	-0.004116177	0	-0.14283877	0	-0.010191756	0
0	-0.10658029	0	-0.004004264	0	-0.14276123	0	0	0
0	-1.1004009	0	-0.14865342	0	0	0	0.00013068	0
0	-1.098488	0	0	0	-0.13516283	0	-0.011231136	0
0	-0.12278548	0	-0.003732162	0	-0.13642679	0	-0.010990696	0
0	0	0	0	0	-0.14636693	0	0	0
0	0	0	0	0	-0.14348832	0	-0.006649515	0
0	0	0	-0.002141287	0	-0.14179242	0	0	0
0	-1.1004755	0	-0.00710643	0	-0.12881085	0	0	0
0	-0.10986642	0	-0.13929369	0	0	0	-0.009705288	0
0	-0.12790949	0	0.00273819	0	-0.14016718	0	-0.009592518	0
0	-1.059763	0	-0.1534984	0	0	0	0.003223331	0
0	-0.10687888	0	-0.13974078	0	0	0	0	0
0	-1.1016912	0	-0.013407532	0	-0.13186901	0	-0.004702543	0
0	-1.1009883	0	-0.007919503	0	-0.12925662	0	-0.011729687	0
0	0	0	0	0	-0.14770621	0	-0.007142745	0
0	0	0	-0.00206369	0	-0.14616719	0	0	0
0	-0.10270023	0	-0.14094149	0	0	0	-0.010366738	0
0	0	0	0	0	-0.14553196	0	-0.008744395	0
0	0	0	-0.00541159	0	-0.14098097	0	-0.008569649	0
0	-1.0976255	0	-0.12912545	0	0	0	-0.012563066	0
0	0	0	-0.005304852	0	-0.14093932	0	0	0
0	-0.10733143	0	-0.1390143	0	0	0	-0.011841631	0
0	-1.096512	0	-0.00725016	0	-0.11354461	0	-0.011666124	0
0	-1.1017045	0	-0.011783731	0	-0.12318674	0	-0.013669001	0
0	0	0	-0.00438416	0	-0.13347727	0	-0.009520505	0
0	0	0	-0.13731002	0	0	0	-0.008100738	0
0	0	0	-0.1393109	0	0	0	0	0
0	-1.0269613	0	-0.018261412	0	-0.098608962	0	-0.011577962	0
0	0	0	0.00236923	0	-0.13727189	0	-0.008115446	0
0	0	0	-0.14050513	0	0	0	-0.008675079	0
0	0	0	-0.13866956	0	0	0	-0.010160807	0
0	0	0	-0.010103297	0	-0.13396822	0	0	0
0	0	0	0	0	-0.14390782	0	0.001958597	0
0	-0.11376954	0	0.004769879	0	-0.14030784	0	-0.013963554	0
0	-0.11291829	0	0	0	-0.13602876	0	-0.013991572	0
0	-0.1085592	0	0.006317364	0	-0.14279473	0	0	0
0	0	0	-0.008942132	0	-0.13657417	0	0.001024105	0
0	-1.1171207	0	0	0	-0.14918977	0	0.023197619	0
0	-0.022350567	0	0	0	-0.1438288	0	-0.008347361	0
0	-1.1147294	0	-0.022284521	0	-0.12594751	0	0.021412272	0
0	-0.019687835	0	-0.013456802	0	-0.13136113	0	0	0
0	-1.1069122	0	-0.025808177	0	-0.12691523	0	0.027034004	0
0	0	0	0	0	-0.14371356	0	-0.006556182	0

0	0	0	-0.013412743	0	-0.13115746	0	0	0
0	-0.1066056	0	0.005360126	0	-0.14302121	0	-0.014426055	0
0	0	0	-0.013736403	0	-0.13023429	0	-0.003557816	0
0	0	0	-0.012036551	0	-0.13185371	0	-0.000370038	0
0	-1.1247062	0	-0.02679744	0	-0.12215985	0	0	0
0	-0.11026598	0	0.002118146	0	-0.13766419	0	-0.016114062	0
0	0	0	-0.007707989	0	-0.12148694	0	0.004428096	0
0	0	0	-0.13743631	0	0	0	0.000261094	0
0	-0.058853297	0	-0.01808053	0	-0.15034412	0	0.007605246	0
0	-0.058856383	0	-0.01798243	0	-0.15041036	0	0.007527854	0
0	-0.058899288	0	0	0	-0.16793033	0	0.007801211	0
0	-0.013288777	0	-0.0135867	0	-0.13261844	0	-0.009129869	0
0	-0.059924618	0	-0.01934546	0	-0.14920143	0	0	0
0	-1.035379	0	-0.007615397	0	-0.095064962	0	-0.010202773	0
0	0	0	0	0	-0.13539219	0	-0.012278786	0
0	0	0	-0.013433403	0	-0.1325164	0	-0.007249278	0
0	-0.11029659	0	-0.12885211	0	0	0	-0.015362456	0
0	0	0	0.004142636	0	-0.13726438	0	-0.012305651	0
0	-1.115685	0	-0.1440884	0	0	0	0.021283513	0
0	-0.031057044	0	-0.013145077	0	-0.11685255	0	-0.008851004	0
0	-0.022528722	0	-0.13777433	0	0	0	-0.009809944	0
0	0	0	0.004919423	0	-0.14072887	0	0	0
0	0	0	-0.13763802	0	0	0	-0.00799044	0
0	-0.015090508	0	-0.01788942	0	-0.125865	0	-0.011066394	0
0	0	0	-0.012865818	0	-0.11634067	0	-0.007072458	0
0	0	0	-0.018305188	0	-0.14826353	0	0.008334898	0
0	0	0	-0.018382616	0	-0.14794595	0	0.008101876	0
0	0	0	0	0	-0.16610254	0	0.008624604	0
0	0	0	-0.017545219	0	-0.12609856	0	-0.00914403	0
0	0	0	0.004115415	0	-0.14107259	0	-0.012672488	0
0	0	0	-0.019822133	0	-0.14691981	0	0	0
0	0	0	0.000964971	0	-0.13591879	0	-0.014363299	0
0	-0.071928647	0	-0.009919745	0	-0.14667768	0	0.000271111	0
0	0	0	-0.019179796	0	-0.14275793	0	0.010355583	0
0	0	0	-0.12834633	0	0	0	-0.013635323	0
0	-0.055285034	0	-0.16108944	0	0	0	0.006258213	0
0	-0.008725492	0	-0.02273338	0	-0.13910374	0	0.003774992	0
0	0	0	-0.022195477	0	-0.1389241	0	0.005286622	0
0	-1.1326432	0	-0.016846184	0	-0.12157721	0	0.023465789	0
0	0	0	-0.009621674	0	-0.14416638	0	0.000538734	0
0	0	0	-0.1593665	0	0	0	0.00709726	0
0	-1.1141494	0	-0.029815302	0	-0.10737536	0	0.020773048	0
0	0	0	-0.002729055	0	-0.13077399	0	-0.0038683	0
0	-0.021229332	0	-0.007270634	0	-0.12617545	0	-0.014312968	0
0	0	0	-0.007064113	0	-0.12623758	0	-0.012332479	0
0	-0.090031017	0	-0.022832848	0	-0.12953084	0	0.023406421	0
0	0	0	-0.023382919	0	-0.12652568	0	0.024888663	0

0	-1.0095517	0	0	0	0	0	0	0
0	-1.0119714	0	0	0	0	0	-0.015425003	0
0	-1.0139666	0	0	0	0	0	-0.014264144	0
0	-1.0584297	0	0	0	0	0	-0.016308982	0
0	-1.0111953	0	0	0	0	0	-0.013714131	0
0	-1.0136395	0	0	0	0	0	-0.013319121	0
0	-0.10371917	0	0	0	0	0	-0.01527887	0
0	0	0	0	0	0	0	-0.013626763	0
0	-1.0903489	0	0	0	0	0	0.009009916	0
0	-1.0182578	0	0	0	-0.14687931	0	0	0
0	-1.0175095	0	0	0	-0.14380569	0	-0.00644429	0
0	-1.0177287	0	-0.002564009	0	-0.14169056	0	0	0
0	-1.0218048	0	0	0	-0.1481314	0	-0.006909021	0
0	-1.022082	0	-0.002562143	0	-0.14609498	0	0	0
0	-1.0210238	0	0	0	-0.14602065	0	-0.008494298	0
0	-1.0217604	0	-0.005891434	0	-0.14093829	0	-0.008343036	0
0	-1.0214043	0	-0.005732999	0	-0.14099877	0	0	0
0	-1.0202463	0	-0.004817738	0	-0.13344855	0	-0.009294162	0
0	-1.0175738	0	-0.13764282	0	0	0	-0.007897282	0
0	-1.0181884	0	-0.13979289	0	0	0	0	0
0	-1.0227662	0	0.001870484	0	-0.13727219	0	-0.007882001	0
0	-1.021823	0	-0.14095203	0	0	0	-0.008446156	0
0	-1.0208318	0	-0.13916702	0	0	0	-0.009914663	0
0	-1.0935406	0	0	0	-0.13743756	0	-0.011231911	0
0	-1.0929015	0	0.002707393	0	-0.13769113	0	-0.011276131	0
0	-1.0953704	0	0.003291581	0	-0.14115233	0	0	0
0	-1.0228128	0	0	0	-0.14421617	0	-0.006353152	0
0	-1.0231135	0	-0.013778065	0	-0.13129923	0	0	0
0	-1.0956492	0	0.002591527	0	-0.1416048	0	-0.011624052	0
0	-1.0664107	0	-0.017541837	0	-0.14795524	0	0.007304213	0
0	-1.0656902	0	-0.017709227	0	-0.14729205	0	0.006845327	0
0	-1.0668177	0	0	0	-0.16510233	0	0.00770329	0
0	-1.096	0	-0.000504137	0	-0.13655178	0	-0.013290109	0
0	-1.0269875	0	-0.013835942	0	-0.13257928	0	-0.007016535	0
0	-1.0679149	0	-0.019029885	0	-0.14667968	0	0	0
0	-1.0926227	0	-0.13040809	0	0	0	-0.012606597	0
0	-1.0226103	0	-0.13814652	0	0	0	-0.007791378	0
0	-1.0266178	0	-0.017824401	0	-0.12633715	0	-0.00889733	0
0	-1.021488	0	-0.013192274	0	-0.11643791	0	-0.006870962	0
0	-1.1011049	0	-0.010768312	0	-0.14460469	0	0.001218126	0
0	-0.1127637	0	0	0	-0.14646846	0	-0.008199121	0
0	-0.1147025	0	-0.002206184	0	-0.14346397	0	-0.008236012	0
0	-0.11013964	0	-0.001606247	0	-0.14542567	0	0	0
0	-1.0587135	0	-0.021705126	0	-0.13818607	0	0.004252403	0
0	-1.0672246	0	-0.15836747	0	0	0	0.006186216	0
0	-0.10574962	0	-0.001600006	0	-0.1467176	0	-0.008829288	0
0	-0.10995632	0	-0.00504453	0	-0.14111229	0	-0.010444069	0

0	-1.0986029	0	-0.008419616	0	-0.12701011	0	-0.011293568	0
0	0	0	0	0	-0.1460031	0	-0.006573207	0
0	0	0	-0.002935228	0	-0.14357374	0	0	0
0	0	0	-0.002865273	0	-0.14070892	0	-0.006671051	0
0	-0.10997139	0	-0.13924894	0	0	0	-0.009706037	0
0	0	0	-0.002809937	0	-0.14497978	0	-0.007148579	0
0	0	0	-0.006174203	0	-0.13955835	0	-0.008783411	0
0	0	0	-0.13890221	0	0	0	-0.008066342	0
0	0	0	-0.009862155	0	-0.13433645	0	0.001877739	0
0	-0.11298457	0	0.004675108	0	-0.14055719	0	-0.013957334	0
0	-1.1168816	0	-0.022416648	0	-0.12742042	0	0.022949538	0
0	-0.022394087	0	-0.014298237	0	-0.13006481	0	-0.008464311	0
0	0	0	-0.014109637	0	-0.13006936	0	-0.006649597	0
0	-0.058835408	0	-0.018081045	0	-0.15035406	0	0.007617988	0
0	0	0	0.00349597	0	-0.13877918	0	-0.012252204	0
0	0	0	-0.018413038	0	-0.1482052	0	0.008438709	0
0	-1.0091324	0	0	0	0	0	-0.013440649	0
0	-1.0180356	0	0	0	-0.14648295	0	-0.006362927	0
0	-1.0182683	0	-0.003391798	0	-0.14359613	0	0	0
0	-1.0176819	0	-0.003287408	0	-0.14062471	0	-0.006493905	0
0	-1.0217841	0	-0.00328336	0	-0.14495479	0	-0.0069282	0
0	-1.0210705	0	-0.006667077	0	-0.13956751	0	-0.008536545	0
0	-1.0179198	0	-0.13939449	0	0	0	-0.007859932	0
0	-1.0935981	0	0.001863018	0	-0.13925615	0	-0.01122302	0
0	-1.0228832	0	-0.014495923	0	-0.1301984	0	-0.006452461	0
0	-1.0666903	0	-0.017772283	0	-0.1478279	0	0.007525307	0
0	-0.11270134	0	-0.002428722	0	-0.14411157	0	-0.008208607	0
0	0	0	-0.0036167	0	-0.14250389	0	-0.006591289	0
0	-1.0180547	0	-0.004071835	0	-0.14254316	0	-0.00639022	0

SUPPLEMENTARY TABLE 7

Model	BIC	Loglike	H2r	SE	bsnp_rs2938864	SE	bsnp_rs1001646
cov0	0	-227.149	0.372704	0	0	0	0
cov9	4.3123	-225.795	0.3701944	0	0	0	0
cov10	4.3266	-225.802	0.3706997	0	0	0	0
cov3	4.7989	-226.038	0.3668976	0	0	0	0
cov1	4.933	-226.105	0.3716411	0	-0.050935257	0	0
cov2	5.6548	-226.466	0.373105	0	0	0	-0.040725921
cov6	6.4283	-226.853	0.3727161	0	0	0	0
cov11	6.723	-227	0.3713302	0	0	0	0
cov5	6.9273	-227.102	0.3724024	0	0	0	0
cov7	6.9711	-227.124	0.373258	0	0	0	0
cov8	6.9884	-227.133	0.3731517	0	0	0	0
cov4	7.0198	-227.149	0.3727212	0	0	0	0
cov1_5	8.3152	-224.286	0.3736679	0	-0.16803053	0	0
cov1_4	9.3785	-224.818	0.372682	0	-0.11251736	0	0
cov3_9	10.0477	-225.153	0.366488	0	0	0	0
cov3_10	10.0885	-225.173	0.366947	0	0	0	0
cov2_4	10.0975	-225.177	0.3766971	0	0	0	-0.11597477
cov1_6	10.1446	-225.201	0.3694756	0	-0.1566975	0	0
cov1_3	10.228	-225.243	0.3664635	0	-0.044780565	0	0
cov3_8	10.6013	-225.429	0.3595119	0	0	0	0
cov1_10	10.6527	-225.455	0.3706591	0	-0.03233146	0	0
cov1_9	10.6905	-225.474	0.3702914	0	-0.031448139	0	0
cov3_7	10.7043	-225.481	0.3597964	0	0	0	0
cov2_3	10.8526	-225.555	0.3676205	0	0	0	-0.034495066
cov2_10	10.9961	-225.627	0.3713842	0	0	0	-0.022270407
cov2_9	11.0065	-225.632	0.3709333	0	0	0	-0.021593756
cov1_11	11.1863	-225.722	0.374417	0	-0.087805112	0	0
cov7_9	11.1936	-225.725	0.3711301	0	0	0	0
cov7_10	11.2061	-225.732	0.3716466	0	0	0	0
cov8_9	11.2225	-225.74	0.3710301	0	0	0	0
cov8_10	11.2352	-225.746	0.3715463	0	0	0	0
cov4_9	11.2445	-225.751	0.3703674	0	0	0	0
cov4_10	11.2759	-225.767	0.3708727	0	0	0	0
cov9_10	11.3041	-225.781	0.3704024	0	0	0	0
cov5_9	11.3312	-225.794	0.370166	0	0	0	0
cov9_11	11.3319	-225.795	0.3701495	0	0	0	0
cov6_9	11.332	-225.795	0.3702157	0	0	0	0
cov5_10	11.3404	-225.799	0.3706306	0	0	0	0
cov10_11	11.343	-225.8	0.3705754	0	0	0	0
cov6_10	11.343	-225.8	0.3707468	0	0	0	0
cov3_6	11.4854	-225.871	0.3671369	0	0	0	0
cov3_5	11.6158	-225.937	0.366111	0	0	0	0
cov3_4	11.771	-226.014	0.3665118	0	0	0	0
cov3_11	11.7752	-226.016	0.3665301	0	0	0	0
cov1_7	11.8473	-226.052	0.3723412	0	-0.051759031	0	0

cov1_8	11.8741	-226.066	0.3722502	0	-0.051643897	0	0
cov1_2	11.9411	-226.099	0.3714416	0	-0.057149143	0	0.007233494
cov2_5	11.945	-226.101	0.3748664	0	0	0	-0.074179385
cov2_11	12.5583	-226.408	0.3744281	0	0	0	-0.051797692
cov2_7	12.5962	-226.427	0.3737508	0	0	0	-0.04121919
cov2_6	12.6188	-226.438	0.3731879	0	0	0	-0.051167408
cov2_8	12.6188	-226.438	0.3736532	0	0	0	-0.041142179
cov4_6	12.8908	-226.574	0.3735769	0	0	0	0
cov5_6	13.2047	-226.731	0.3735577	0	0	0	0
cov6_7	13.3807	-226.819	0.3733294	0	0	0	0
cov6_8	13.4002	-226.829	0.3732364	0	0	0	0
cov6_11	13.4482	-226.853	0.3727767	0	0	0	0
cov1_5_6	13.4738	-223.356	0.3715963	0	-0.27590385	0	0
cov4_11	13.5897	-226.924	0.3711079	0	0	0	0
cov4_5	13.637	-226.947	0.3724279	0	0	0	0
cov7_11	13.7206	-226.989	0.3717474	0	0	0	0
cov8_11	13.7317	-226.995	0.3716287	0	0	0	0
cov7_8	13.7348	-226.996	0.3731343	0	0	0	0
cov5_11	13.737	-226.997	0.3712633	0	0	0	0
cov5_7	13.8947	-227.076	0.3729549	0	0	0	0
cov5_8	13.9129	-227.085	0.3728523	0	0	0	0
cov4_7	13.9912	-227.124	0.3732534	0	0	0	0
cov4_8	14.0086	-227.133	0.3731504	0	0	0	0
cov1_5_11	14.5841	-223.911	0.376213	0	-0.20424375	0	0
cov1_4_6	14.7485	-223.993	0.3710363	0	-0.21157733	0	0
cov1_3_5	15.0266	-224.132	0.3712428	0	-0.14908887	0	0
cov1_5_7	15.1213	-224.179	0.3747991	0	-0.17125388	0	0
cov1_4_5	15.1278	-224.182	0.3736722	0	-0.16662798	0	0
cov1_5_8	15.1588	-224.198	0.374701	0	-0.17097753	0	0
cov1_5_9	15.2885	-224.263	0.3733551	0	-0.15624113	0	0
cov1_5_10	15.2899	-224.264	0.3734392	0	-0.15642316	0	0
cov1_3_6	15.3209	-224.279	0.3640948	0	-0.15361349	0	0
cov1_2_5	15.3343	-224.286	0.3735985	0	-0.16986473	0	0.00223401
cov1_6_10	15.3593	-224.298	0.3681858	0	-0.1495185	0	0
cov1_6_9	15.3911	-224.314	0.3677414	0	-0.1485251	0	0
cov1_4_11	15.5963	-224.417	0.3754231	0	-0.15063351	0	0
cov1_2_4	15.7766	-224.507	0.3748135	0	-0.078423285	0	-0.059802496
cov1_3_4	15.8193	-224.528	0.3693587	0	-0.096627776	0	0
cov1_4_10	15.8422	-224.54	0.3719981	0	-0.092123936	0	0
cov1_4_9	15.8686	-224.553	0.3717567	0	-0.091821155	0	0
cov1_3_11	15.8887	-224.563	0.3692754	0	-0.093673953	0	0
cov2_4_9	16.3212	-224.779	0.3747829	0	0	0	-0.090528239
cov2_4_10	16.3418	-224.789	0.3751127	0	0	0	-0.090818826
cov1_4_7	16.3739	-224.806	0.3729983	0	-0.11228088	0	0
cov1_4_8	16.3854	-224.811	0.3729148	0	-0.11234886	0	0
cov1_3_10	16.4709	-224.854	0.3667678	0	-0.030974448	0	0
cov1_3_9	16.4786	-224.858	0.3664333	0	-0.030110402	0	0

cov1_3_8	16.5219	-224.88	0.360855	0	-0.038070426	0	0
cov1_6_11	16.5914	-224.914	0.3721102	0	-0.18328808	0	0
cov3_8_9	16.6011	-224.919	0.3617778	0	0	0	0
cov1_3_7	16.6062	-224.922	0.3611628	0	-0.038412251	0	0
cov2_3_4	16.6132	-224.925	0.3727641	0	0	0	-0.095729505
cov3_8_10	16.652	-224.945	0.3621835	0	0	0	0
cov3_7_9	16.6685	-224.953	0.3621103	0	0	0	0
cov3_7_10	16.719	-224.978	0.3625265	0	0	0	0
cov2_3_9	16.7938	-225.015	0.3670913	0	0	0	-0.019796722
cov2_3_10	16.8106	-225.024	0.367503	0	0	0	-0.020526474
cov1_10_11	16.9882	-225.113	0.3732532	0	-0.06775719	0	0
cov2_3_8	16.9956	-225.116	0.3612241	0	0	0	-0.028214733
cov1_9_11	17.0104	-225.124	0.3729129	0	-0.067183128	0	0
cov1_6_7	17.0238	-225.131	0.3702648	0	-0.15875039	0	0
cov3_5_9	17.0288	-225.133	0.3661685	0	0	0	0
cov2_4_11	17.0316	-225.134	0.3778096	0	0	0	-0.12508515
cov3_9_11	17.0395	-225.138	0.3667536	0	0	0	0
cov2_4_5	17.0406	-225.139	0.3764311	0	0	0	-0.11295834
cov3_5_10	17.054	-225.146	0.3665555	0	0	0	0
cov3_9_10	17.0598	-225.149	0.3666231	0	0	0	0
cov1_6_8	17.0634	-225.15	0.3701528	0	-0.15819691	0	0
cov3_4_9	17.0637	-225.15	0.3665878	0	0	0	0
cov3_6_9	17.0678	-225.152	0.366481	0	0	0	0
cov2_3_7	17.0863	-225.162	0.3615323	0	0	0	-0.028500945
cov3_10_11	17.0918	-225.164	0.3671635	0	0	0	0
cov3_4_10	17.1072	-225.172	0.3670103	0	0	0	0
cov3_6_10	17.1079	-225.173	0.366964	0	0	0	0
cov2_4_7	17.1144	-225.176	0.3768029	0	0	0	-0.11570072
cov2_4_8	17.1174	-225.177	0.3767252	0	0	0	-0.11589999
cov2_4_6	17.1177	-225.177	0.376696	0	0	0	-0.11612364
cov1_2_6	17.1621	-225.2	0.3693967	0	-0.15942254	0	0.003379535
cov1_2_3	17.223	-225.23	0.3661577	0	-0.053727762	0	0.010447088
cov3_5_8	17.3619	-225.3	0.3585002	0	0	0	0
cov3_5_7	17.4679	-225.353	0.358788	0	0	0	0
cov3_6_8	17.4834	-225.36	0.3602171	0	0	0	0
cov3_7_8	17.493	-225.365	0.3597373	0	0	0	0
cov2_3_11	17.4959	-225.367	0.3695517	0	0	0	-0.054173866
cov1_7_10	17.5086	-225.373	0.371597	0	-0.032902412	0	0
cov1_8_10	17.5409	-225.389	0.3715029	0	-0.032826571	0	0
cov1_7_9	17.5499	-225.394	0.371216	0	-0.032002869	0	0
cov3_6_7	17.5767	-225.407	0.3605164	0	0	0	0
cov1_8_9	17.5818	-225.409	0.3711225	0	-0.031928125	0	0
cov3_4_8	17.5835	-225.41	0.3592031	0	0	0	0
cov3_8_11	17.5903	-225.414	0.3592683	0	0	0	0
cov2_3_5	17.6435	-225.44	0.3693848	0	0	0	-0.054760935
cov1_2_10	17.6468	-225.442	0.3703304	0	-0.041373114	0	0.010664867
cov1_9_10	17.6671	-225.452	0.3705497	0	-0.031858732	0	0

cov2_5_9	17.6701	-225.454	0.372564	0	0	0	-0.047384594
cov2_6_9	17.6731	-225.455	0.3709479	0	0	0	-0.046320494
cov3_4_7	17.6865	-225.462	0.3594883	0	0	0	0
cov1_2_9	17.6886	-225.463	0.3699913	0	-0.039777868	0	0.009800397
cov3_7_11	17.6927	-225.465	0.3595489	0	0	0	0
cov2_6_10	17.6946	-225.466	0.3714695	0	0	0	-0.045981648
cov2_5_10	17.6961	-225.467	0.3729196	0	0	0	-0.046999165
cov2_3_6	17.7807	-225.509	0.367679	0	0	0	-0.047789621
cov2_9_11	17.8312	-225.534	0.3725635	0	0	0	-0.035446565
cov1_7_11	17.8361	-225.537	0.376394	0	-0.097274302	0	0
cov2_10_11	17.8421	-225.54	0.3729475	0	0	0	-0.035461012
cov2_7_10	17.868	-225.553	0.372314	0	0	0	-0.022514339
cov2_7_9	17.8808	-225.559	0.3718517	0	0	0	-0.021830406
cov1_8_11	17.8927	-225.565	0.3762389	0	-0.096457382	0	0
cov2_8_10	17.8982	-225.568	0.3722167	0	0	0	-0.022478629
cov2_8_9	17.9107	-225.574	0.3717547	0	0	0	-0.021795127
cov4_5_10	17.9329	-225.585	0.3705104	0	0	0	0
cov4_5_9	17.9446	-225.591	0.3700479	0	0	0	0
cov7_8_9	17.9938	-225.615	0.3710332	0	0	0	0
cov2_9_10	17.9957	-225.616	0.3711574	0	0	0	-0.021683022
cov7_8_10	18.0062	-225.622	0.3715454	0	0	0	0
cov4_7_9	18.1478	-225.692	0.3712206	0	0	0	0
cov4_6_9	18.1653	-225.701	0.3709504	0	0	0	0
cov4_8_9	18.1741	-225.706	0.3711216	0	0	0	0
cov4_7_10	18.1753	-225.706	0.3717399	0	0	0	0
cov4_6_10	18.1791	-225.708	0.3714439	0	0	0	0
cov7_9_10	18.1831	-225.71	0.3713527	0	0	0	0
cov4_8_10	18.2021	-225.72	0.3716414	0	0	0	0
cov1_2_11	18.2055	-225.721	0.3743458	0	-0.089541124	0	0.002163468
cov7_9_11	18.2121	-225.725	0.3712295	0	0	0	0
cov5_7_9	18.2122	-225.725	0.3710958	0	0	0	0
cov8_9_10	18.2122	-225.725	0.3712522	0	0	0	0
cov6_7_9	18.2131	-225.725	0.3711539	0	0	0	0
cov4_9_11	18.2167	-225.727	0.3699611	0	0	0	0
cov5_7_10	18.2191	-225.728	0.3715681	0	0	0	0
cov6_7_10	18.222	-225.73	0.3716907	0	0	0	0
cov7_10_11	18.2263	-225.732	0.3716519	0	0	0	0
cov4_10_11	18.2353	-225.736	0.3703934	0	0	0	0
cov5_8_9	18.2411	-225.739	0.3709966	0	0	0	0
cov8_9_11	18.2415	-225.739	0.3711127	0	0	0	0
cov4_9_10	18.2418	-225.74	0.3705498	0	0	0	0
cov6_8_9	18.2419	-225.74	0.3710542	0	0	0	0
cov5_8_10	18.2483	-225.743	0.3714693	0	0	0	0
cov6_8_10	18.251	-225.744	0.3715941	0	0	0	0
cov8_10_11	18.2553	-225.746	0.3715357	0	0	0	0
cov5_9_10	18.3215	-225.779	0.3703665	0	0	0	0
cov9_10_11	18.3231	-225.78	0.37034	0	0	0	0

cov6_9_10	18.3234	-225.78	0.3704322	0	0	0	0
cov5_6_9	18.3512	-225.794	0.3701325	0	0	0	0
cov5_9_11	18.3514	-225.794	0.3701665	0	0	0	0
cov6_9_11	18.352	-225.795	0.3701763	0	0	0	0
cov5_10_11	18.3605	-225.799	0.370608	0	0	0	0
cov5_6_10	18.3605	-225.799	0.3706117	0	0	0	0
cov6_10_11	18.3625	-225.8	0.3706531	0	0	0	0
cov3_6_11	18.4184	-225.828	0.3680007	0	0	0	0
cov3_4_6	18.4256	-225.831	0.3679546	0	0	0	0
cov3_5_6	18.5045	-225.871	0.3672516	0	0	0	0
cov3_4_5	18.525	-225.881	0.3662884	0	0	0	0
cov1_7_8	18.6085	-225.923	0.3722272	0	-0.051782246	0	0
cov3_5_11	18.6112	-225.924	0.3662315	0	0	0	0
cov3_4_11	18.7824	-226.01	0.3664316	0	0	0	0
cov1_2_7	18.854	-226.046	0.3721388	0	-0.058343585	0	0.007658618
cov2_5_7	18.8783	-226.058	0.3755949	0	0	0	-0.074894994
cov1_2_8	18.881	-226.059	0.3720494	0	-0.05817143	0	0.007591603
cov2_5_8	18.9016	-226.069	0.3754882	0	0	0	-0.074802282
cov2_5_6	18.9197	-226.078	0.3750395	0	0	0	-0.068118773
cov2_5_11	18.9606	-226.099	0.375092	0	0	0	-0.075595262
cov2_7_8	19.3612	-226.299	0.3736371	0	0	0	-0.041196644
cov2_7_11	19.4427	-226.34	0.3756442	0	0	0	-0.055270006
cov2_8_11	19.4741	-226.356	0.3754957	0	0	0	-0.05482428
cov2_6_7	19.5625	-226.4	0.3738269	0	0	0	-0.051445595
cov2_6_11	19.5677	-226.402	0.3742848	0	0	0	-0.055290774
cov2_6_8	19.5853	-226.411	0.3737251	0	0	0	-0.051340931
cov4_6_7	19.882	-226.56	0.3739553	0	0	0	0
cov4_6_8	19.8934	-226.565	0.3738746	0	0	0	0
cov4_6_11	19.9044	-226.571	0.3733025	0	0	0	0
cov4_5_6	19.907	-226.572	0.3734997	0	0	0	0
cov1_5_6_11	19.9364	-223.077	0.3740149	0	-0.30187491	0	0
cov1_3_5_6	20.1364	-223.177	0.3689642	0	-0.25691553	0	0
cov5_6_7	20.1515	-226.694	0.374223	0	0	0	0
cov5_6_8	20.1706	-226.704	0.3741358	0	0	0	0
cov6_7_8	20.1776	-226.707	0.3732145	0	0	0	0
cov5_6_11	20.2155	-226.726	0.3732402	0	0	0	0
cov1_5_6_7	20.2291	-223.223	0.3728338	0	-0.28104145	0	0
cov1_5_6_8	20.2833	-223.25	0.3727114	0	-0.28016117	0	0
cov1_5_6_9	20.3003	-223.259	0.3708832	0	-0.25726719	0	0
cov1_5_6_10	20.3065	-223.262	0.3710563	0	-0.25750032	0	0
cov1_4_5_6	20.3537	-223.285	0.3717683	0	-0.27297088	0	0
cov6_7_11	20.394	-226.816	0.3736935	0	0	0	0
cov6_8_11	20.4151	-226.826	0.3735584	0	0	0	0
cov4_5_11	20.4663	-226.852	0.3713399	0	0	0	0
cov7_8_11	20.4878	-226.862	0.3716367	0	0	0	0
cov1_2_5_6	20.4934	-223.355	0.3716425	0	-0.27459322	0	-0.001706696
cov4_7_11	20.6065	-226.922	0.3712808	0	0	0	0

cov4_8_11	20.6097	-226.923	0.3711498	0	0	0	0
cov4_5_7	20.6424	-226.94	0.3727146	0	0	0	0
cov4_5_8	20.6512	-226.944	0.3726115	0	0	0	0
cov5_7_8	20.6558	-226.946	0.3728284	0	0	0	0
cov5_7_11	20.7377	-226.987	0.371676	0	0	0	0
cov5_8_11	20.748	-226.993	0.3715496	0	0	0	0
cov4_7_8	20.7549	-226.996	0.3731272	0	0	0	0
cov1_4_6_10	20.849	-223.533	0.3699818	0	-0.19671822	0	0
cov1_4_6_9	20.8743	-223.546	0.3696564	0	-0.19611338	0	0
cov1_3_5_11	21.0084	-223.613	0.3732898	0	-0.18470284	0	0
cov1_5_7_11	21.0418	-223.629	0.3788193	0	-0.21933591	0	0
cov1_3_4_6	21.0849	-223.651	0.3672891	0	-0.19741682	0	0
cov1_2_4_6	21.0872	-223.652	0.3731811	0	-0.17773852	0	-0.062550344
cov1_5_8_11	21.1097	-223.663	0.3786606	0	-0.21835712	0	0
cov1_4_6_11	21.1561	-223.687	0.3736459	0	-0.23952783	0	0
cov1_3_6_9	21.1681	-223.693	0.3639538	0	-0.14731986	0	0
cov1_3_6_10	21.1697	-223.693	0.3643669	0	-0.14821443	0	0
cov1_3_6_11	21.237	-223.727	0.366968	0	-0.19069866	0	0
cov1_4_5_11	21.3798	-223.798	0.376249	0	-0.20319373	0	0
cov1_5_9_11	21.5702	-223.894	0.3759232	0	-0.19390429	0	0
cov1_5_10_11	21.5743	-223.896	0.3760021	0	-0.19446873	0	0
cov1_2_5_11	21.6025	-223.91	0.3763145	0	-0.20208721	0	-0.002819522
cov1_3_6_8	21.632	-223.925	0.3585886	0	-0.14636042	0	0
cov1_3_4_11	21.6649	-223.941	0.3717297	0	-0.1388324	0	0
cov1_4_6_7	21.7243	-223.971	0.3714449	0	-0.21194367	0	0
cov1_3_6_7	21.7434	-223.98	0.3590252	0	-0.14610997	0	0
cov1_4_6_8	21.7444	-223.981	0.3713431	0	-0.21173853	0	0
cov1_2_4_11	21.8218	-224.019	0.3781969	0	-0.11602368	0	-0.068014223
cov1_3_4_5	21.9031	-224.06	0.3714712	0	-0.14980317	0	0
cov1_5_7_8	21.9091	-224.063	0.3746791	0	-0.17084809	0	0
cov1_6_10_11	21.9136	-224.065	0.3705456	0	-0.17371353	0	0
cov1_6_9_11	21.9308	-224.074	0.3701384	0	-0.17308282	0	0
cov1_3_5_9	21.9978	-224.107	0.3709285	0	-0.13697861	0	0
cov1_3_5_10	22.0012	-224.109	0.3710251	0	-0.1374665	0	0
cov1_4_5_7	22.0053	-224.111	0.374592	0	-0.16955617	0	0
cov1_4_5_8	22.036	-224.126	0.374493	0	-0.16923698	0	0
cov1_2_3_5	22.0423	-224.13	0.3710893	0	-0.1525936	0	0.00443426
cov1_2_4_5	22.0437	-224.13	0.3745298	0	-0.14342161	0	-0.027292141
cov1_3_5_8	22.0457	-224.131	0.3709095	0	-0.14717824	0	0
cov1_3_5_7	22.0458	-224.131	0.3715664	0	-0.15094851	0	0
cov1_4_5_10	22.0783	-224.148	0.373367	0	-0.15209744	0	0
cov1_4_5_9	22.0797	-224.148	0.3732739	0	-0.15227319	0	0
cov1_5_7_9	22.0868	-224.152	0.3745023	0	-0.15858451	0	0
cov1_5_7_10	22.0878	-224.152	0.3745907	0	-0.15870401	0	0
cov1_4_10_11	22.1208	-224.169	0.3746435	0	-0.12992138	0	0
cov1_5_8_9	22.1247	-224.171	0.3744045	0	-0.15835525	0	0
cov1_5_8_10	22.1257	-224.171	0.3744928	0	-0.15848133	0	0

cov1_4_9_11	22.136	-224.176	0.3744236	0	-0.12973205	0	0
cov1_2_5_7	22.1398	-224.178	0.3747156	0	-0.17350997	0	0.002741968
cov1_6_7_10	22.151	-224.184	0.3692839	0	-0.15195006	0	0
cov1_2_5_8	22.1773	-224.197	0.3746195	0	-0.17318398	0	0.002681745
cov1_6_7_9	22.1872	-224.202	0.3688266	0	-0.15091269	0	0
cov1_6_8_10	22.2018	-224.209	0.3691631	0	-0.15134072	0	0
cov1_6_8_9	22.2375	-224.227	0.3687058	0	-0.15030925	0	0
cov1_3_9_11	22.2879	-224.252	0.3690899	0	-0.077839261	0	0
cov1_3_10_11	22.2997	-224.258	0.3693732	0	-0.078457278	0	0
cov1_2_5_9	22.3064	-224.262	0.3732523	0	-0.15864913	0	0.003127199
cov1_2_5_10	22.3076	-224.262	0.3733306	0	-0.15893466	0	0.003309937
cov1_5_9_10	22.3083	-224.263	0.3733772	0	-0.15608466	0	0
cov1_2_3_6	22.3312	-224.274	0.3639318	0	-0.15889	0	0.006554988
cov1_6_9_10	22.3642	-224.291	0.3680083	0	-0.1490436	0	0
cov1_2_6_10	22.3685	-224.293	0.3680016	0	-0.15505955	0	0.006899714
cov1_2_6_9	22.4033	-224.31	0.3675857	0	-0.1532553	0	0.005886075
cov1_3_4_10	22.4103	-224.314	0.3692484	0	-0.080329856	0	0
cov1_3_4_9	22.4226	-224.32	0.3689897	0	-0.079713658	0	0
cov1_4_7_11	22.4236	-224.32	0.3767745	0	-0.15567582	0	0
cov1_2_4_10	22.4435	-224.33	0.3739937	0	-0.067295798	0	-0.050174751
cov1_2_4_9	22.4549	-224.336	0.3738145	0	-0.066539165	0	-0.050920403
cov1_4_8_11	22.4622	-224.34	0.3766333	0	-0.15511794	0	0
cov1_2_3_4	22.4804	-224.349	0.3717999	0	-0.073308948	0	-0.047304106
cov1_3_4_8	22.4915	-224.354	0.365141	0	-0.085688698	0	0
cov1_3_8_11	22.5265	-224.372	0.3647577	0	-0.083000273	0	0
cov1_3_4_7	22.5519	-224.384	0.3655048	0	-0.086612208	0	0
cov1_3_7_11	22.5919	-224.404	0.3651345	0	-0.083835528	0	0
cov1_2_4_7	22.7883	-224.503	0.374973	0	-0.078615111	0	-0.059221614
cov1_2_4_8	22.7942	-224.506	0.374902	0	-0.078527882	0	-0.059488007
cov1_4_7_10	22.8076	-224.512	0.3724835	0	-0.091121959	0	0
cov1_4_8_10	22.8253	-224.521	0.3723999	0	-0.091297455	0	0
cov1_4_7_9	22.8359	-224.526	0.3722282	0	-0.090829577	0	0
cov1_4_8_9	22.8531	-224.535	0.3721455	0	-0.09100569	0	0
cov1_4_9_10	22.8616	-224.539	0.3719575	0	-0.09190814	0	0
cov1_2_3_11	22.9049	-224.561	0.3691347	0	-0.097022406	0	0.004166303
cov2_3_4_9	22.9486	-224.583	0.3715677	0	0	0	-0.074352622
cov2_3_4_10	22.9749	-224.596	0.3718977	0	0	0	-0.07487119
cov1_3_8_9	23.1087	-224.663	0.3621633	0	-0.02814543	0	0
cov1_3_8_10	23.1163	-224.667	0.3624926	0	-0.028992469	0	0
cov1_4_7_8	23.1521	-224.685	0.3728847	0	-0.11209288	0	0
cov1_3_7_9	23.1721	-224.695	0.3625059	0	-0.028263015	0	0
cov2_3_4_8	23.1786	-224.698	0.3673013	0	0	0	-0.081366958
cov1_3_7_10	23.1787	-224.698	0.3628463	0	-0.029118692	0	0
cov2_4_9_11	23.2017	-224.709	0.3761155	0	0	0	-0.10122558
cov1_6_7_11	23.2237	-224.72	0.374153	0	-0.19351473	0	0
cov2_4_10_11	23.2357	-224.726	0.3764042	0	0	0	-0.10116662
cov2_4_5_9	23.2413	-224.729	0.3744138	0	0	0	-0.086701635

cov2_3_4_7	23.246	-224.731	0.3676993	0	0	0	-0.082417899
cov2_4_5_10	23.2473	-224.732	0.3747051	0	0	0	-0.086471023
cov2_4_6_9	23.2513	-224.734	0.3746442	0	0	0	-0.099582908
cov2_4_6_10	23.2844	-224.751	0.3750257	0	0	0	-0.099314233
cov1_6_8_11	23.2958	-224.756	0.3739639	0	-0.19203804	0	0
cov2_4_7_9	23.3138	-224.765	0.3750831	0	0	0	-0.089147423
cov2_4_8_9	23.3255	-224.771	0.375012	0	0	0	-0.089487931
cov2_4_7_10	23.3336	-224.775	0.3754214	0	0	0	-0.089394302
cov2_4_9_10	23.3367	-224.777	0.3748599	0	0	0	-0.090265339
cov2_4_8_10	23.3455	-224.781	0.375351	0	0	0	-0.089739872
cov2_3_9_11	23.3843	-224.801	0.3691418	0	0	0	-0.04044789
cov1_3_7_8	23.3897	-224.803	0.3611126	0	-0.03849966	0	0
cov2_3_8_9	23.3909	-224.804	0.3625659	0	0	0	-0.01817314
cov2_3_4_11	23.4125	-224.815	0.3739772	0	0	0	-0.10753063
cov2_3_8_10	23.4196	-224.818	0.3629453	0	0	0	-0.018919937
cov2_3_10_11	23.4311	-224.824	0.3694975	0	0	0	-0.040637778
cov1_2_3_10	23.454	-224.835	0.3663731	0	-0.041730285	0	0.012693973
cov2_3_7_9	23.4559	-224.836	0.3629069	0	0	0	-0.018268124
cov1_2_3_9	23.4653	-224.841	0.3660581	0	-0.040345838	0	0.012049597
cov2_3_6_9	23.4662	-224.842	0.3671831	0	0	0	-0.044295403
cov3_7_8_9	23.4749	-224.846	0.3620343	0	0	0	0
cov1_3_9_10	23.4819	-224.849	0.3666297	0	-0.030376754	0	0
cov2_3_7_10	23.4841	-224.851	0.3632971	0	0	0	-0.01901781
cov2_3_6_10	23.5129	-224.865	0.3676522	0	0	0	-0.04407528
cov1_2_3_8	23.5145	-224.866	0.3605265	0	-0.047408201	0	0.010916076
cov3_7_8_10	23.525	-224.871	0.3624458	0	0	0	0
cov3_5_8_9	23.5378	-224.877	0.361056	0	0	0	0
cov1_7_10_11	23.5521	-224.885	0.3755399	0	-0.077553947	0	0
cov2_3_4_5	23.5565	-224.887	0.3724617	0	0	0	-0.092726243
cov3_5_8_10	23.5704	-224.894	0.3613797	0	0	0	0
cov1_7_9_11	23.5759	-224.896	0.3751929	0	-0.076940165	0	0
cov1_2_3_7	23.5991	-224.908	0.3608359	0	-0.047708155	0	0.010866518
cov1_6_7_8	23.603	-224.91	0.3700197	0	-0.16461251	0	0
cov3_8_9_11	23.6073	-224.912	0.3620177	0	0	0	0
cov3_5_7_9	23.6091	-224.913	0.3614045	0	0	0	0
cov1_2_6_11	23.6114	-224.914	0.3721346	0	-0.18264772	0	-0.000841074
cov1_8_10_11	23.6123	-224.915	0.3753842	0	-0.076790478	0	0
cov3_6_8_9	23.6195	-224.918	0.3617296	0	0	0	0
cov3_4_8_9	23.6206	-224.919	0.3618294	0	0	0	0
cov3_8_9_10	23.6207	-224.919	0.3618303	0	0	0	0
cov2_3_4_6	23.6242	-224.921	0.3727154	0	0	0	-0.099000735
cov1_8_9_11	23.636	-224.927	0.375037	0	-0.076176939	0	0
cov3_5_7_10	23.6416	-224.929	0.3617376	0	0	0	0
cov3_8_10_11	23.6651	-224.941	0.3623595	0	0	0	0
cov3_4_8_10	23.6722	-224.945	0.3621911	0	0	0	0
cov3_6_8_10	23.6722	-224.945	0.3621783	0	0	0	0
cov3_7_9_11	23.6738	-224.945	0.3623586	0	0	0	0

cov3_6_7_9	23.6873	-224.952	0.3620689	0	0	0	0	0
cov3_4_7_9	23.6878	-224.952	0.3621686	0	0	0	0	0
cov3_7_9_10	23.6879	-224.952	0.3621733	0	0	0	0	0
cov2_3_5_9	23.7135	-224.965	0.3683351	0	0	0	0	-0.034103127
cov3_7_10_11	23.7316	-224.974	0.3627102	0	0	0	0	0
cov3_4_7_10	23.7392	-224.978	0.3625408	0	0	0	0	0
cov3_6_7_10	23.7392	-224.978	0.3625261	0	0	0	0	0
cov2_3_5_10	23.7443	-224.981	0.3686476	0	0	0	0	-0.033930425
cov2_3_8_11	23.7713	-224.994	0.363204	0	0	0	0	-0.044617558
cov2_3_9_10	23.8043	-225.011	0.3672435	0	0	0	0	-0.019857089
cov3_4_5_9	23.845	-225.031	0.3663312	0	0	0	0	0
cov2_3_7_11	23.8569	-225.037	0.3635435	0	0	0	0	-0.045091014
cov3_4_5_10	23.8573	-225.037	0.3667391	0	0	0	0	0
cov2_4_5_11	23.8631	-225.04	0.3781085	0	0	0	0	-0.12592227
cov3_5_9_11	23.8663	-225.042	0.3664562	0	0	0	0	0
cov2_3_7_8	23.8734	-225.045	0.3614816	0	0	0	0	-0.02853453
cov2_3_6_8	23.8735	-225.045	0.361077	0	0	0	0	-0.044566374
cov2_4_7_8	23.9037	-225.06	0.3766826	0	0	0	0	-0.11531561
cov3_5_10_11	23.9042	-225.061	0.3668592	0	0	0	0	0
cov3_5_6_9	23.9541	-225.086	0.3650215	0	0	0	0	0
cov2_3_6_7	23.9722	-225.095	0.3614074	0	0	0	0	-0.044399614
cov2_3_5_8	23.9774	-225.097	0.3624257	0	0	0	0	-0.037359928
cov3_5_6_10	23.9857	-225.101	0.3654991	0	0	0	0	0
cov1_9_10_11	23.9987	-225.108	0.3731131	0	-0.067259187	0	0	0
cov1_2_10_11	24.0007	-225.109	0.3730444	0	-0.072320461	0	0	0.005784636
cov1_2_9_11	24.0251	-225.121	0.3727347	0	-0.071056087	0	0	0.004899126
cov2_4_5_6	24.0278	-225.122	0.3763823	0	0	0	0	-0.11980068
cov2_4_6_11	24.0348	-225.126	0.3780003	0	0	0	0	-0.1215467
cov2_4_7_11	24.035	-225.126	0.3781841	0	0	0	0	-0.12541656
cov3_5_9_10	24.0373	-225.127	0.3663155	0	0	0	0	0
cov3_6_9_11	24.0378	-225.127	0.3671315	0	0	0	0	0
cov1_2_6_7	24.0406	-225.129	0.3701778	0	-0.16184525	0	0	0.003829642
cov2_4_8_11	24.0443	-225.131	0.3780609	0	0	0	0	-0.12531013
cov3_9_10_11	24.0536	-225.135	0.3668616	0	0	0	0	0
cov3_4_9_11	24.0576	-225.137	0.3667187	0	0	0	0	0
cov2_4_5_8	24.0601	-225.139	0.3763801	0	0	0	0	-0.11303862
cov2_4_5_7	24.0605	-225.139	0.3764634	0	0	0	0	-0.11290561
cov2_3_5_7	24.0619	-225.139	0.3628338	0	0	0	0	-0.038374046
cov3_4_9_10	24.0764	-225.147	0.3667126	0	0	0	0	0
cov3_6_9_10	24.08	-225.148	0.3666185	0	0	0	0	0
cov1_2_6_8	24.0803	-225.149	0.3700673	0	-0.16123463	0	0	0.003760515
cov3_4_6_9	24.0812	-225.149	0.3667254	0	0	0	0	0
cov3_6_10_11	24.0843	-225.151	0.3675695	0	0	0	0	0
cov3_4_10_11	24.1096	-225.163	0.3671265	0	0	0	0	0
cov3_4_6_10	24.1208	-225.169	0.3672132	0	0	0	0	0
cov2_4_6_7	24.1346	-225.176	0.3768039	0	0	0	0	-0.11585686
cov2_4_6_8	24.1376	-225.177	0.3767261	0	0	0	0	-0.11604743

cov3_4_5_8	24.1443	-225.181	0.3583985	0	0	0	0
cov3_5_7_8	24.2533	-225.235	0.3587259	0	0	0	0
cov3_4_5_7	24.2553	-225.236	0.3586789	0	0	0	0
cov1_7_8_10	24.299	-225.258	0.3714964	0	-0.033122666	0	0
cov3_5_8_11	24.3108	-225.264	0.3584976	0	0	0	0
cov1_7_8_9	24.3401	-225.279	0.371121	0	-0.03223913	0	0
cov3_5_6_8	24.3711	-225.294	0.3578078	0	0	0	0
cov3_6_7_8	24.3803	-225.299	0.3604232	0	0	0	0
cov3_5_7_11	24.4198	-225.318	0.358787	0	0	0	0
cov2_3_5_11	24.4531	-225.335	0.3702106	0	0	0	-0.062224608
cov3_4_7_8	24.4739	-225.345	0.3594249	0	0	0	0
cov3_7_8_11	24.4822	-225.35	0.3594933	0	0	0	0
cov3_5_6_7	24.4824	-225.35	0.3582935	0	0	0	0
cov3_6_8_11	24.4851	-225.351	0.3607635	0	0	0	0
cov3_4_6_8	24.4913	-225.354	0.3606944	0	0	0	0
cov1_2_7_10	24.4996	-225.358	0.3712596	0	-0.042467242	0	0.011276249
cov2_3_6_11	24.5133	-225.365	0.3694889	0	0	0	-0.055944504
cov1_7_9_10	24.5231	-225.37	0.3714907	0	-0.032434585	0	0
cov1_2_8_10	24.5322	-225.375	0.3711678	0	-0.042331209	0	0.011204559
cov2_5_7_9	24.544	-225.381	0.373511	0	0	0	-0.047635197
cov1_2_7_9	24.5453	-225.381	0.3709071	0	-0.04083143	0	0.010381262
cov2_6_7_9	24.5459	-225.381	0.3719005	0	0	0	-0.046611106
cov1_8_9_10	24.5554	-225.386	0.3713966	0	-0.032358147	0	0
cov2_6_7_10	24.5655	-225.391	0.3724296	0	0	0	-0.046264607
cov2_5_7_10	24.5688	-225.393	0.3738709	0	0	0	-0.047213914
cov2_5_8_9	24.5737	-225.395	0.373413	0	0	0	-0.047609864
cov3_6_7_11	24.5754	-225.396	0.3611035	0	0	0	0
cov1_2_8_9	24.5775	-225.397	0.3708155	0	-0.040697222	0	0.010311642
cov2_6_8_9	24.5783	-225.398	0.37179	0	0	0	-0.046491755
cov3_4_6_7	24.5815	-225.399	0.3610452	0	0	0	0
cov1_7_8_11	24.5839	-225.4	0.3763045	0	-0.097591307	0	0
cov2_5_6_9	24.5979	-225.407	0.3719828	0	0	0	-0.053533867
cov2_6_8_10	24.598	-225.408	0.3723193	0	0	0	-0.046143896
cov2_5_8_10	24.5987	-225.408	0.3737729	0	0	0	-0.047192444
cov3_4_8_11	24.5987	-225.408	0.3591663	0	0	0	0
cov2_7_9_11	24.6008	-225.409	0.3742721	0	0	0	-0.039580302
cov2_7_10_11	24.6155	-225.416	0.3746505	0	0	0	-0.039576017
cov2_5_9_11	24.6281	-225.423	0.3732858	0	0	0	-0.051708997
cov2_5_6_10	24.6314	-225.424	0.3724067	0	0	0	-0.052837796
cov2_8_9_11	24.6424	-225.43	0.3741244	0	0	0	-0.039187873
cov2_6_9_11	24.6466	-225.432	0.3718245	0	0	0	-0.049684772
cov2_8_10_11	24.6567	-225.437	0.3745035	0	0	0	-0.03918254
cov2_3_5_6	24.6604	-225.439	0.3692658	0	0	0	-0.056190989
cov2_5_10_11	24.6611	-225.439	0.3736147	0	0	0	-0.051106199
cov1_2_9_10	24.662	-225.44	0.3702356	0	-0.040788076	0	0.010488144
cov2_7_8_10	24.6636	-225.44	0.3722163	0	0	0	-0.022655206
cov2_6_9_10	24.6715	-225.444	0.3711402	0	0	0	-0.046101042

cov2_6_10_11	24.675	-225.446	0.3722807	0	0	0	-0.049099506
cov2_7_8_9	24.6764	-225.447	0.3717618	0	0	0	-0.021977445
cov2_5_9_10	24.6809	-225.449	0.3726577	0	0	0	-0.046868722
cov3_4_7_11	24.7013	-225.459	0.3594494	0	0	0	0
cov2_9_10_11	24.83	-225.524	0.3727159	0	0	0	-0.035207057
cov1_2_7_11	24.8555	-225.536	0.3763308	0	-0.098794585	0	0.001898432
cov2_7_9_10	24.8675	-225.542	0.3720908	0	0	0	-0.02192505
cov4_5_7_10	24.8887	-225.553	0.3711395	0	0	0	0
cov2_8_9_10	24.8977	-225.557	0.3719935	0	0	0	-0.021889308
cov4_5_7_9	24.9006	-225.559	0.3706764	0	0	0	0
cov4_5_8_10	24.9079	-225.562	0.3710389	0	0	0	0
cov1_2_8_11	24.912	-225.565	0.3761752	0	-0.0979863	0	0.001909241
cov4_5_8_9	24.9198	-225.568	0.3705759	0	0	0	0
cov4_5_9_10	24.9229	-225.57	0.3702803	0	0	0	0
cov4_7_8_9	24.9508	-225.584	0.3711213	0	0	0	0
cov4_5_10_11	24.9524	-225.585	0.370562	0	0	0	0
cov4_5_6_10	24.9524	-225.585	0.3704473	0	0	0	0
cov4_5_9_11	24.9635	-225.59	0.3701135	0	0	0	0
cov4_5_6_9	24.9641	-225.591	0.3699774	0	0	0	0
cov4_7_8_10	24.9779	-225.597	0.3716364	0	0	0	0
cov7_8_9_10	24.9837	-225.6	0.3712539	0	0	0	0
cov5_7_8_9	25.0118	-225.614	0.370994	0	0	0	0
cov7_8_9_11	25.0123	-225.615	0.3711326	0	0	0	0
cov6_7_8_9	25.0139	-225.615	0.3710406	0	0	0	0
cov5_7_8_10	25.0183	-225.618	0.3714624	0	0	0	0
cov6_7_8_10	25.0242	-225.621	0.3715805	0	0	0	0
cov7_8_10_11	25.0264	-225.622	0.3715501	0	0	0	0
cov4_6_7_9	25.0868	-225.652	0.371665	0	0	0	0
cov4_6_7_10	25.0988	-225.658	0.3721689	0	0	0	0
cov4_6_8_9	25.11	-225.663	0.3715806	0	0	0	0
cov4_6_8_10	25.1221	-225.67	0.3720846	0	0	0	0
cov4_7_9_10	25.1424	-225.68	0.3714236	0	0	0	0
cov4_7_9_11	25.1512	-225.684	0.3708712	0	0	0	0
cov4_6_9_10	25.1624	-225.69	0.3711351	0	0	0	0
cov4_8_9_10	25.169	-225.693	0.3713212	0	0	0	0
cov4_7_10_11	25.1709	-225.694	0.3713027	0	0	0	0
cov4_8_9_11	25.1742	-225.696	0.3707406	0	0	0	0
cov4_6_9_11	25.1809	-225.699	0.3707317	0	0	0	0
cov4_6_10_11	25.1927	-225.705	0.3711765	0	0	0	0
cov4_8_10_11	25.1937	-225.705	0.3711709	0	0	0	0
cov5_7_9_10	25.1999	-225.708	0.3713101	0	0	0	0
cov6_7_9_10	25.2022	-225.71	0.3713842	0	0	0	0
cov7_9_10_11	25.2023	-225.71	0.3714259	0	0	0	0
cov4_9_10_11	25.2116	-225.714	0.3701421	0	0	0	0
cov5_7_9_11	25.224	-225.72	0.371328	0	0	0	0
cov6_7_9_11	25.2265	-225.722	0.3715075	0	0	0	0
cov5_8_9_10	25.2291	-225.723	0.3712105	0	0	0	0

cov6_8_9_10	25.2312	-225.724	0.3712807	0	0	0	0
cov8_9_10_11	25.2318	-225.724	0.3713068	0	0	0	0
cov5_6_7_9	25.2322	-225.725	0.3710633	0	0	0	0
cov5_7_10_11	25.2328	-225.725	0.3717791	0	0	0	0
cov6_7_10_11	25.2377	-225.727	0.3719826	0	0	0	0
cov5_6_7_10	25.2392	-225.728	0.371549	0	0	0	0
cov5_8_9_11	25.2547	-225.736	0.3712016	0	0	0	0
cov6_8_9_11	25.2568	-225.737	0.3713685	0	0	0	0
cov5_6_8_9	25.2612	-225.739	0.3709704	0	0	0	0
cov5_8_10_11	25.2637	-225.74	0.3716518	0	0	0	0
cov6_8_10_11	25.2679	-225.742	0.3718438	0	0	0	0
cov5_6_8_10	25.2685	-225.743	0.3714558	0	0	0	0
cov5_6_9_10	25.3411	-225.779	0.370299	0	0	0	0
cov5_9_10_11	25.3417	-225.779	0.3703687	0	0	0	0
cov6_9_10_11	25.3432	-225.78	0.3703669	0	0	0	0
cov5_6_9_11	25.3714	-225.794	0.3701206	0	0	0	0
cov5_6_10_11	25.3805	-225.799	0.3705665	0	0	0	0
cov3_4_6_11	25.388	-225.802	0.3685142	0	0	0	0
cov3_4_5_6	25.3974	-225.807	0.3674863	0	0	0	0
cov3_5_6_11	25.4347	-225.826	0.3678296	0	0	0	0
cov3_4_5_11	25.5225	-225.87	0.3664014	0	0	0	0
cov1_2_7_8	25.6147	-225.916	0.3720167	0	-0.058497241	0	0.007810077
cov2_5_7_8	25.6549	-225.936	0.3754634	0	0	0	-0.074604658
cov2_5_6_7	25.8491	-226.033	0.3757848	0	0	0	-0.06859975
cov2_5_6_8	25.8722	-226.045	0.3756886	0	0	0	-0.068491021
cov2_5_7_11	25.8784	-226.048	0.3761576	0	0	0	-0.078015146
cov2_5_8_11	25.9048	-226.061	0.3760122	0	0	0	-0.077680171
cov2_5_6_11	25.9198	-226.068	0.3755669	0	0	0	-0.069904058
cov2_7_8_11	26.2042	-226.211	0.3755496	0	0	0	-0.055386272
cov2_6_7_8	26.3095	-226.263	0.3737222	0	0	0	-0.053024192
cov1_3_5_6_11	26.3393	-222.768	0.3710574	0	-0.28254722	0	0
cov1_5_6_7_11	26.3704	-222.784	0.3767026	0	-0.31799342	0	0
cov1_5_6_8_11	26.4579	-222.827	0.3765072	0	-0.31617937	0	0
cov2_6_7_11	26.4596	-226.338	0.3755411	0	0	0	-0.057152656
cov2_6_8_11	26.4905	-226.354	0.3753842	0	0	0	-0.056834992
cov4_6_7_8	26.7129	-226.465	0.3738277	0	0	0	0
cov1_5_6_9_11	26.7953	-222.996	0.3732899	0	-0.28411439	0	0
cov1_4_5_6_11	26.8008	-222.999	0.3742191	0	-0.29915645	0	0
cov1_5_6_10_11	26.8062	-223.002	0.3734507	0	-0.28453169	0	0
cov1_5_6_7_8	26.84	-223.018	0.3725892	0	-0.28615343	0	0
cov4_6_7_11	26.9008	-226.559	0.3738009	0	0	0	0
cov4_5_6_7	26.9009	-226.559	0.3738952	0	0	0	0
cov4_6_8_11	26.9113	-226.564	0.3736778	0	0	0	0
cov4_5_6_8	26.9118	-226.564	0.373806	0	0	0	0
cov4_5_6_11	26.9222	-226.57	0.3732735	0	0	0	0
cov1_2_5_6_11	26.9486	-223.073	0.3742013	0	-0.29760524	0	-0.005902721
cov1_3_5_6_9	26.9556	-223.076	0.3682423	0	-0.23774656	0	0

cov1_3_5_6_10	26.9664	-223.082	0.3684374	0	-0.23830996	0	0
cov5_6_7_8	26.9794	-226.598	0.3740568	0	0	0	0
cov1_5_6_7_9	27.035	-223.116	0.3721778	0	-0.26163013	0	0
cov1_5_6_7_10	27.0404	-223.119	0.3723551	0	-0.2618103	0	0
cov1_3_4_5_6	27.0728	-223.135	0.3692776	0	-0.25599192	0	0
cov1_5_6_8_9	27.0914	-223.144	0.3720541	0	-0.26083333	0	0
cov1_5_6_8_10	27.0968	-223.147	0.3722314	0	-0.26101917	0	0
cov1_3_4_6_11	27.1474	-223.172	0.3696988	0	-0.22950634	0	0
cov1_4_5_6_9	27.1487	-223.173	0.3709941	0	-0.25248191	0	0
cov1_4_5_6_10	27.1504	-223.174	0.3711744	0	-0.2524108	0	0
cov1_3_5_6_7	27.1526	-223.175	0.3695851	0	-0.26061019	0	0
cov1_3_5_6_8	27.156	-223.176	0.3687159	0	-0.25547604	0	0
cov1_2_3_5_6	27.1565	-223.177	0.3689463	0	-0.25734912	0	0.000586374
cov5_6_7_11	27.1707	-226.694	0.3740986	0	0	0	0
cov1_4_5_6_7	27.1741	-223.185	0.3728068	0	-0.27826889	0	0
cov5_6_8_11	27.1891	-226.703	0.3739733	0	0	0	0
cov6_7_8_11	27.1936	-226.705	0.3735001	0	0	0	0
cov1_4_5_6_8	27.2213	-223.209	0.3726842	0	-0.27731277	0	0
cov1_2_5_6_7	27.249	-223.223	0.3728651	0	-0.28012118	0	-0.001194781
cov1_2_4_5_6	27.2625	-223.23	0.3726614	0	-0.24923212	0	-0.028185622
cov1_3_6_9_11	27.2756	-223.236	0.3665866	0	-0.18168379	0	0
cov1_3_6_10_11	27.2971	-223.247	0.3669431	0	-0.18221885	0	0
cov1_2_5_6_8	27.3031	-223.25	0.3727444	0	-0.27919661	0	-0.001252557
cov1_5_6_9_10	27.3189	-223.258	0.3709255	0	-0.25699659	0	0
cov1_2_5_6_9	27.3205	-223.259	0.370886	0	-0.25719447	0	-9.91E-05
cov1_2_5_6_10	27.3267	-223.262	0.3710485	0	-0.25769545	0	0.000269829
cov1_2_4_6_11	27.3404	-223.269	0.3764144	0	-0.20534164	0	-0.069695762
cov1_4_6_10_11	27.3454	-223.271	0.3724275	0	-0.22331068	0	0
cov1_3_4_6_10	27.3553	-223.276	0.3669843	0	-0.18570939	0	0
cov1_4_6_9_11	27.359	-223.278	0.3721341	0	-0.22293066	0	0
cov1_3_4_6_9	27.3622	-223.28	0.3666349	0	-0.1848494	0	0
cov4_7_8_11	27.3793	-226.798	0.3711802	0	0	0	0
cov4_5_7_8	27.4049	-226.811	0.3725894	0	0	0	0
cov1_2_4_6_10	27.4585	-223.328	0.3719372	0	-0.17198682	0	-0.049611705
cov1_2_4_6_9	27.4664	-223.332	0.3716789	0	-0.1709101	0	-0.050515397
cov4_5_7_11	27.4852	-226.851	0.3714426	0	0	0	0
cov4_5_8_11	27.4864	-226.852	0.3713152	0	0	0	0
cov5_7_8_11	27.5058	-226.861	0.3715764	0	0	0	0
cov1_2_3_4_6	27.7218	-223.459	0.3697737	0	-0.17409043	0	-0.048812462
cov1_3_4_6_8	27.7491	-223.473	0.3629792	0	-0.18647825	0	0
cov1_4_6_7_10	27.7686	-223.483	0.3706225	0	-0.19673762	0	0
cov1_4_6_7_9	27.7965	-223.497	0.3702837	0	-0.1961168	0	0
cov1_4_6_8_10	27.8004	-223.499	0.3705169	0	-0.1965483	0	0
cov1_5_7_8_11	27.8166	-223.507	0.3787219	0	-0.21919849	0	0
cov1_4_6_8_9	27.8279	-223.512	0.3701792	0	-0.19593342	0	0
cov1_3_4_6_7	27.829	-223.513	0.3634847	0	-0.1871861	0	0
cov1_3_6_8_11	27.8569	-223.527	0.3623314	0	-0.18020643	0	0

cov1_4_6_9_10	27.8635	-223.53	0.3698693	0	-0.1963139	0	0
cov1_3_6_8_9	27.8886	-223.543	0.3602511	0	-0.14335862	0	0
cov1_3_4_5_11	27.8943	-223.546	0.3734958	0	-0.18523175	0	0
cov1_3_6_8_10	27.9056	-223.551	0.3606843	0	-0.14424331	0	0
cov1_3_6_7_11	27.9432	-223.57	0.3628451	0	-0.18073744	0	0
cov1_4_5_7_11	27.9561	-223.576	0.3785683	0	-0.21720561	0	0
cov1_3_6_7_9	27.9637	-223.58	0.3607379	0	-0.14335106	0	0
cov1_4_6_7_11	27.9663	-223.582	0.3750555	0	-0.24534059	0	0
cov1_3_5_7_11	27.9744	-223.586	0.3757174	0	-0.19971587	0	0
cov1_3_6_7_10	27.979	-223.588	0.3611851	0	-0.14426852	0	0
cov1_3_5_9_11	27.9944	-223.596	0.3730145	0	-0.17435503	0	0
cov1_3_5_10_11	28.0012	-223.599	0.3731071	0	-0.17541834	0	0
cov1_3_5_8_11	28.0028	-223.6	0.3749509	0	-0.19495271	0	0
cov1_4_6_8_11	28.0165	-223.607	0.3748823	0	-0.24428284	0	0
cov1_4_5_8_11	28.017	-223.607	0.3784058	0	-0.21616988	0	0
cov1_5_7_9_11	28.0199	-223.608	0.378545	0	-0.20796168	0	0
cov1_5_7_10_11	28.0244	-223.611	0.3786262	0	-0.20851963	0	0
cov1_2_3_5_11	28.0285	-223.613	0.373317	0	-0.18416483	0	-0.000718127
cov1_2_5_7_11	28.0595	-223.628	0.3789403	0	-0.2168183	0	-0.003305284
cov1_2_4_6_7	28.0868	-223.642	0.3734206	0	-0.17847158	0	-0.061656642
cov1_5_8_9_11	28.0878	-223.642	0.3783856	0	-0.20698733	0	0
cov1_5_8_10_11	28.0924	-223.645	0.3784671	0	-0.20754504	0	0
cov1_2_4_6_8	28.0994	-223.648	0.3733327	0	-0.17812834	0	-0.062000615
cov1_2_5_8_11	28.1274	-223.662	0.378781	0	-0.21585148	0	-0.003289898
cov1_3_6_9_10	28.1693	-223.683	0.3641616	0	-0.1476607	0	0
cov1_2_3_6_10	28.1715	-223.684	0.3641269	0	-0.15536332	0	0.008909252
cov1_2_3_6_9	28.1731	-223.685	0.3637354	0	-0.15383658	0	0.008115927
cov1_2_4_5_11	28.2061	-223.701	0.3776418	0	-0.1736761	0	-0.037557541
cov1_2_3_4_11	28.2542	-223.726	0.3745275	0	-0.11460267	0	-0.051887452
cov1_2_3_6_11	28.2569	-223.727	0.3669349	0	-0.19154873	0	0.001114775
cov1_4_6_7_8	28.3343	-223.766	0.3712233	0	-0.21723117	0	0
cov1_4_5_9_11	28.3469	-223.772	0.3758697	0	-0.19018621	0	0
cov1_4_5_10_11	28.3493	-223.773	0.3759572	0	-0.19036828	0	0
cov1_3_6_7_8	28.3501	-223.773	0.3588673	0	-0.15184233	0	0
cov1_3_4_9_11	28.3536	-223.775	0.371319	0	-0.12223614	0	0
cov1_3_4_10_11	28.3563	-223.777	0.3715436	0	-0.12279828	0	0
cov1_6_7_10_11	28.4372	-223.817	0.3729787	0	-0.18498964	0	0
cov1_6_7_9_11	28.4558	-223.826	0.3725665	0	-0.18432211	0	0
cov1_6_8_10_11	28.5184	-223.858	0.3727805	0	-0.18347612	0	0
cov1_6_8_9_11	28.5369	-223.867	0.3723681	0	-0.18281092	0	0
cov1_3_4_8_11	28.5417	-223.869	0.3687778	0	-0.12859482	0	0
cov1_2_4_10_11	28.5671	-223.882	0.3773215	0	-0.10473001	0	-0.059177697
cov1_2_4_9_11	28.5673	-223.882	0.3771638	0	-0.10409093	0	-0.059763937
cov1_3_4_7_11	28.5821	-223.889	0.3692128	0	-0.13007248	0	0
cov1_2_5_9_11	28.5895	-223.893	0.3760001	0	-0.19246329	0	-0.002030797
cov1_5_9_10_11	28.5903	-223.894	0.3759102	0	-0.19402535	0	0
cov1_2_5_10_11	28.5936	-223.895	0.3760746	0	-0.19313201	0	-0.001917999

cov1_2_3_6_8	28.6408	-223.919	0.358408	0	-0.15200103	0	0.007019556
cov1_2_4_7_11	28.6885	-223.943	0.3793181	0	-0.12134282	0	-0.066410823
cov1_2_4_8_11	28.7221	-223.959	0.3791903	0	-0.12068718	0	-0.066627877
cov1_6_7_8_10	28.7475	-223.972	0.3690607	0	-0.15772151	0	0
cov1_2_3_6_7	28.7523	-223.975	0.3588451	0	-0.15172498	0	0.006988859
cov1_6_7_8_9	28.7834	-223.99	0.3686072	0	-0.15669033	0	0
cov1_4_5_7_8	28.7938	-223.995	0.3744729	0	-0.16915538	0	0
cov1_3_5_7_8	28.8358	-224.016	0.3714866	0	-0.1507875	0	0
cov1_3_4_5_9	28.8564	-224.027	0.3710988	0	-0.13563921	0	0
cov1_3_4_5_10	28.8575	-224.027	0.3712066	0	-0.13582182	0	0
cov1_2_3_4_5	28.8701	-224.033	0.3722831	0	-0.13446925	0	-0.019866747
cov1_5_7_8_9	28.8794	-224.038	0.374397	0	-0.15874665	0	0
cov1_5_7_8_10	28.8802	-224.039	0.3744812	0	-0.15885665	0	0
cov1_4_7_10_11	28.8854	-224.041	0.3762241	0	-0.13438941	0	0
cov1_4_7_9_11	28.9019	-224.049	0.3759891	0	-0.13416156	0	0
cov1_6_9_10_11	28.9143	-224.056	0.3703526	0	-0.17329365	0	0
cov1_3_4_5_8	28.9151	-224.056	0.3705896	0	-0.14470105	0	0
cov1_3_4_5_7	28.9227	-224.06	0.3712408	0	-0.14846665	0	0
cov1_2_5_7_8	28.9273	-224.062	0.3745907	0	-0.17323735	0	0.002904157
cov1_4_8_10_11	28.9292	-224.063	0.3760831	0	-0.13393709	0	0
cov1_2_6_10_11	28.9317	-224.064	0.3704485	0	-0.17599113	0	0.003011917
cov1_4_8_9_11	28.9456	-224.071	0.3758487	0	-0.13371091	0	0
cov1_2_6_9_11	28.9501	-224.073	0.3700753	0	-0.17457752	0	0.001973857
cov1_4_5_7_10	28.9522	-224.075	0.3743099	0	-0.15469135	0	0
cov1_4_5_7_9	28.9538	-224.075	0.3742157	0	-0.1548677	0	0
cov1_2_4_5_7	28.96	-224.078	0.3751501	0	-0.15043772	0	-0.022033854
cov1_4_5_8_10	28.983	-224.09	0.3742082	0	-0.1543837	0	0
cov1_4_5_8_9	28.9845	-224.091	0.374114	0	-0.1545595	0	0
cov1_2_4_5_8	28.9861	-224.091	0.3750705	0	-0.14947422	0	-0.022777418
cov1_2_4_5_10	28.996	-224.096	0.3742257	0	-0.12926145	0	-0.027066798
cov1_2_4_5_9	28.9962	-224.097	0.374134	0	-0.12917199	0	-0.0272274
cov1_2_3_5_9	29.0114	-224.104	0.3707335	0	-0.1409419	0	0.00536653
cov1_2_3_5_10	29.0144	-224.106	0.370825	0	-0.14149202	0	0.005529236
cov1_3_5_7_9	29.0161	-224.106	0.3713591	0	-0.13934288	0	0
cov1_3_5_8_9	29.0175	-224.107	0.3707075	0	-0.13577139	0	0
cov1_3_5_9_10	29.018	-224.107	0.3709344	0	-0.136945	0	0
cov1_3_5_7_10	29.0192	-224.108	0.3714841	0	-0.13994888	0	0
cov1_3_5_8_10	29.021	-224.109	0.3708305	0	-0.13641895	0	0
cov1_2_3_5_8	29.0611	-224.129	0.370717	0	-0.15058442	0	0.004561989
cov1_2_3_5_7	29.0616	-224.129	0.3713833	0	-0.15418347	0	0.004334615
cov1_4_5_9_10	29.0976	-224.147	0.3733251	0	-0.15187572	0	0
cov1_2_5_7_9	29.1039	-224.15	0.3743834	0	-0.16145171	0	0.003716372
cov1_2_5_7_10	29.1045	-224.151	0.374466	0	-0.16168639	0	0.003922912
cov1_5_7_9_10	29.1063	-224.152	0.3745323	0	-0.15837733	0	0
cov1_3_8_9_11	29.1369	-224.167	0.365981	0	-0.072834804	0	0
cov1_4_9_10_11	29.1385	-224.168	0.3745728	0	-0.1295885	0	0
cov1_2_5_8_9	29.1418	-224.169	0.3742874	0	-0.16117333	0	0.003652933

cov1_2_5_8_10	29.1425	-224.17	0.3743696	0	-0.16141499	0	0.003858224
cov1_5_8_9_10	29.1442	-224.171	0.3744339	0	-0.15815236	0	0
cov1_3_8_10_11	29.1545	-224.176	0.3662639	0	-0.073558	0	0
cov1_6_7_9_10	29.1561	-224.176	0.369112	0	-0.15147643	0	0
cov1_2_6_7_10	29.158	-224.177	0.3690889	0	-0.15803728	0	0.007563865
cov1_3_7_9_11	29.181	-224.189	0.3663937	0	-0.073445169	0	0
cov1_4_7_8_11	29.189	-224.193	0.3766849	0	-0.15576661	0	0
cov1_2_3_4_10	29.1923	-224.195	0.3713262	0	-0.063263227	0	-0.039182048
cov1_2_3_4_9	29.1973	-224.197	0.3711242	0	-0.06241269	0	-0.039699313
cov1_2_6_7_9	29.1976	-224.197	0.3686601	0	-0.15615787	0	0.006513901
cov1_3_7_10_11	29.1981	-224.197	0.3666875	0	-0.074171855	0	0
cov1_6_8_9_10	29.2069	-224.202	0.3689904	0	-0.15086686	0	0
cov1_2_6_8_10	29.2091	-224.203	0.36897	0	-0.15736273	0	0.007485153
cov1_3_4_8_10	29.2472	-224.222	0.3660948	0	-0.075088856	0	0
cov1_2_6_8_9	29.2481	-224.222	0.3685411	0	-0.15549222	0	0.006438556
cov1_3_4_8_9	29.2492	-224.223	0.3658061	0	-0.074315959	0	0
cov1_2_3_4_8	29.2547	-224.226	0.3678837	0	-0.067300789	0	-0.040565916
cov1_3_4_7_10	29.2915	-224.244	0.3664887	0	-0.07571362	0	0
cov1_3_4_7_9	29.2946	-224.246	0.3661921	0	-0.074934697	0	0
cov1_2_3_9_11	29.3	-224.248	0.368879	0	-0.082562465	0	0.005954218
cov1_3_9_10_11	29.3029	-224.25	0.3691912	0	-0.077822501	0	0
cov1_2_3_4_7	29.3058	-224.251	0.3682987	0	-0.067907946	0	-0.041298252
cov1_2_3_10_11	29.3101	-224.253	0.3691421	0	-0.083633717	0	0.00655142
cov1_2_5_9_10	29.3261	-224.261	0.3732745	0	-0.15853701	0	0.003212284
cov1_3_4_7_8	29.3402	-224.269	0.3654401	0	-0.08658464	0	0
cov1_3_7_8_11	29.3625	-224.28	0.3651094	0	-0.084205137	0	0
cov1_2_6_9_10	29.3744	-224.286	0.3678385	0	-0.15435023	0	0.006589008
cov1_3_4_9_10	29.4281	-224.312	0.3691708	0	-0.079915299	0	0
cov1_2_4_7_10	29.4363	-224.317	0.3742739	0	-0.067270821	0	-0.048788025
cov1_2_4_8_10	29.4481	-224.322	0.3742075	0	-0.067274326	0	-0.049138125
cov1_2_4_7_9	29.4489	-224.323	0.3740845	0	-0.066489311	0	-0.049603746
cov1_2_4_8_9	29.4604	-224.329	0.3740178	0	-0.066496167	0	-0.049945454
cov1_2_4_9_10	29.4619	-224.329	0.3739332	0	-0.066937184	0	-0.050241412
cov1_2_3_8_11	29.5404	-224.369	0.3645653	0	-0.087198483	0	0.005268944
cov1_2_4_7_8	29.5752	-224.386	0.3748505	0	-0.07867479	0	-0.058791858
cov1_4_7_8_10	29.603	-224.4	0.3723814	0	-0.091249638	0	0
cov1_2_3_7_11	29.606	-224.401	0.364946	0	-0.087950283	0	0.005164598
cov1_4_7_8_9	29.631	-224.414	0.3721306	0	-0.090973458	0	0
cov2_3_4_9_11	29.6902	-224.444	0.3728775	0	0	0	-0.086694972
cov2_3_4_10_11	29.7363	-224.467	0.373187	0	0	0	-0.087038387
cov2_3_4_8_9	29.7481	-224.472	0.3678168	0	0	0	-0.06735246
cov2_3_4_8_10	29.7786	-224.488	0.3681294	0	0	0	-0.068055115
cov2_3_4_7_9	29.7957	-224.496	0.3682305	0	0	0	-0.068112057
cov1_6_7_8_11	29.7985	-224.498	0.3739249	0	-0.19949192	0	0
cov2_3_4_7_10	29.826	-224.511	0.3685519	0	0	0	-0.068807121
cov1_4_7_9_10	29.827	-224.512	0.3724418	0	-0.090897787	0	0
cov2_3_4_6_9	29.8344	-224.516	0.3712109	0	0	0	-0.084484768

cov1_4_8_9_10	29.8447	-224.521	0.3723605	0	-0.091077008	0	0
cov2_3_4_5_9	29.8706	-224.534	0.3711737	0	0	0	-0.070600735
cov2_3_4_6_10	29.8768	-224.537	0.3716135	0	0	0	-0.084501598
cov2_3_4_5_10	29.8836	-224.54	0.3714729	0	0	0	-0.070652147
cov2_4_5_6_9	29.9082	-224.553	0.3736233	0	0	0	-0.10359283
cov2_4_5_6_10	29.9218	-224.559	0.3740098	0	0	0	-0.1031318
cov2_4_5_9_11	29.9553	-224.576	0.3763067	0	0	0	-0.10105443
cov2_3_4_9_10	29.9669	-224.582	0.3716253	0	0	0	-0.074229866
cov1_3_7_8_9	29.9696	-224.583	0.3624352	0	-0.028504854	0	0
cov2_4_5_10_11	29.9718	-224.584	0.3765981	0	0	0	-0.10069153
cov1_3_7_8_10	29.9759	-224.586	0.3627701	0	-0.029345655	0	0
cov2_3_4_5_8	30.0187	-224.608	0.3661439	0	0	0	-0.074788702
cov2_3_4_8_11	30.0367	-224.617	0.3686532	0	0	0	-0.092398679
cov2_3_4_7_8	30.042	-224.619	0.3676167	0	0	0	-0.082168036
cov2_3_6_8_9	30.0642	-224.631	0.3626354	0	0	0	-0.042619729
cov2_3_8_9_11	30.0937	-224.645	0.3649196	0	0	0	-0.036324938
cov2_3_4_5_7	30.0945	-224.646	0.3665604	0	0	0	-0.075974975
cov1_2_3_8_9	30.0953	-224.646	0.361791	0	-0.038397488	0	0.012070364
cov1_2_3_8_10	30.1001	-224.648	0.3621093	0	-0.039655675	0	0.012580097
cov2_3_4_7_11	30.1006	-224.649	0.3690759	0	0	0	-0.093592041
cov2_4_7_8_9	30.1203	-224.659	0.3749845	0	0	0	-0.089054622
cov2_3_6_8_10	30.1206	-224.659	0.3630617	0	0	0	-0.042492042
cov1_3_8_9_10	30.1245	-224.661	0.3623178	0	-0.028305425	0	0
cov2_3_6_7_9	30.1348	-224.666	0.3630103	0	0	0	-0.042534327
cov2_4_7_8_10	30.1397	-224.668	0.3753182	0	0	0	-0.089300176
cov2_3_8_10_11	30.143	-224.67	0.3652211	0	0	0	-0.036614857
cov2_4_7_9_11	30.1503	-224.674	0.3768693	0	0	0	-0.10089615
cov2_3_7_9_11	30.1507	-224.674	0.3653039	0	0	0	-0.036662052
cov1_2_3_7_9	30.1587	-224.678	0.3621345	0	-0.038500561	0	0.012052922
cov2_3_4_5_11	30.1614	-224.679	0.3739808	0	0	0	-0.10732735
cov1_2_3_7_10	30.1626	-224.68	0.3624658	0	-0.03975739	0	0.012553689
cov2_3_4_6_8	30.1644	-224.681	0.3669893	0	0	0	-0.08723638
cov2_4_8_9_11	30.1711	-224.684	0.3767514	0	0	0	-0.10094956
cov2_4_7_10_11	30.186	-224.691	0.377156	0	0	0	-0.10082102
cov1_3_7_9_10	30.1871	-224.692	0.3626686	0	-0.028435047	0	0
cov2_3_6_7_10	30.1905	-224.694	0.3634464	0	0	0	-0.042402701
cov2_4_6_9_11	30.1961	-224.696	0.3757775	0	0	0	-0.10446272
cov2_3_7_10_11	30.2001	-224.698	0.3656146	0	0	0	-0.036946517
cov2_4_8_10_11	30.2066	-224.702	0.377037	0	0	0	-0.10087754
cov2_4_9_10_11	30.2199	-224.708	0.3761561	0	0	0	-0.10097125
cov2_4_6_10_11	30.2353	-224.716	0.3761175	0	0	0	-0.10407045
cov2_3_4_6_7	30.2361	-224.716	0.3674188	0	0	0	-0.087933801
cov2_4_6_7_9	30.2393	-224.718	0.374972	0	0	0	-0.098328196
cov1_2_6_7_11	30.2436	-224.72	0.3741861	0	-0.19266122	0	-0.00112352
cov2_4_5_7_9	30.2466	-224.722	0.3746263	0	0	0	-0.085881194
cov2_4_5_9_10	30.251	-224.724	0.3745159	0	0	0	-0.086173349
cov2_4_5_7_10	30.2525	-224.725	0.3749426	0	0	0	-0.085648324

cov2_4_6_8_9	30.2526	-224.725	0.374897	0	0	0	-0.098604139
cov2_4_5_8_9	30.2548	-224.726	0.3745723	0	0	0	-0.086162553
cov2_3_7_8_9	30.2584	-224.728	0.3628398	0	0	0	-0.018421009
cov2_4_5_8_10	30.2608	-224.729	0.3748698	0	0	0	-0.085925569
cov2_4_6_9_10	30.2679	-224.732	0.3747127	0	0	0	-0.099299697
cov2_4_6_7_10	30.2719	-224.734	0.3753599	0	0	0	-0.098024054
cov3_4_5_8_9	30.2754	-224.736	0.3609198	0	0	0	0
cov3_5_6_8_9	30.2796	-224.738	0.3578786	0	0	0	0
cov2_3_6_9_11	30.2846	-224.741	0.3687104	0	0	0	-0.051247826
cov2_4_6_8_10	30.2854	-224.741	0.3752839	0	0	0	-0.098305618
cov2_3_7_8_10	30.2861	-224.741	0.3632248	0	0	0	-0.019163334
cov3_4_5_8_10	30.2976	-224.747	0.3612717	0	0	0	0
cov1_2_6_8_11	30.3157	-224.756	0.3739961	0	-0.19120737	0	-0.001093292
cov2_4_7_9_10	30.3285	-224.763	0.3751706	0	0	0	-0.088845391
cov3_5_6_8_10	30.3285	-224.763	0.3583876	0	0	0	0
cov1_7_8_10_11	30.3295	-224.763	0.3754648	0	-0.078074319	0	0
cov2_4_8_9_10	30.3404	-224.769	0.3750973	0	0	0	-0.089188686
cov2_3_6_10_11	30.3446	-224.771	0.3691178	0	0	0	-0.050835832
cov3_4_5_7_9	30.3517	-224.774	0.3612578	0	0	0	0
cov1_7_8_9_11	30.3533	-224.775	0.3751227	0	-0.077471315	0	0
cov3_5_8_9_11	30.361	-224.779	0.3612599	0	0	0	0
cov3_5_6_7_9	30.3735	-224.785	0.3583933	0	0	0	0
cov3_4_5_7_10	30.3736	-224.785	0.3616185	0	0	0	0
cov1_2_3_7_8	30.3819	-224.789	0.3607805	0	-0.047907113	0	0.010997118
cov2_3_5_8_9	30.3915	-224.794	0.3634208	0	0	0	-0.024848221
cov2_3_5_9_11	30.4023	-224.8	0.369277	0	0	0	-0.042101446
cov2_3_9_10_11	30.4025	-224.8	0.3692005	0	0	0	-0.040359895
cov3_5_8_10_11	30.4057	-224.801	0.3615972	0	0	0	0
cov2_3_8_9_10	30.4099	-224.803	0.3626445	0	0	0	-0.018202846
cov3_5_7_8_9	30.4126	-224.805	0.3613169	0	0	0	0
cov3_5_6_7_10	30.4215	-224.809	0.3589061	0	0	0	0
cov2_3_4_6_11	30.4224	-224.81	0.3741466	0	0	0	-0.10484545
cov2_3_5_8_10	30.4244	-224.811	0.3636951	0	0	0	-0.024874502
cov3_5_7_9_11	30.434	-224.815	0.3616114	0	0	0	0
cov3_5_7_8_10	30.4444	-224.821	0.3616462	0	0	0	0
cov2_3_5_10_11	30.4501	-224.823	0.3695991	0	0	0	-0.041833137
cov2_3_5_7_9	30.4522	-224.825	0.3638543	0	0	0	-0.025642336
cov1_2_3_9_10	30.4662	-224.832	0.3662527	0	-0.040989366	0	0.012472227
cov2_3_7_9_10	30.4745	-224.836	0.3629968	0	0	0	-0.018302326
cov3_5_7_10_11	30.4786	-224.838	0.3619582	0	0	0	0
cov3_7_8_9_11	30.4804	-224.839	0.3622812	0	0	0	0
cov2_3_6_9_10	30.4815	-224.839	0.3672933	0	0	0	-0.044199629
cov2_3_5_6_9	30.4841	-224.84	0.3669544	0	0	0	-0.043097811
cov2_3_5_7_10	30.4852	-224.841	0.3641341	0	0	0	-0.025655124
cov2_3_4_5_6	30.4914	-224.844	0.3720933	0	0	0	-0.10258296
cov3_6_7_8_9	30.4921	-224.844	0.3619732	0	0	0	0
cov3_7_8_9_10	30.4943	-224.846	0.3620951	0	0	0	0

cov3_4_7_8_9	30.4945	-224.846	0.3620822	0	0	0	0
cov2_3_5_6_10	30.5297	-224.863	0.3673779	0	0	0	-0.04258494
cov3_7_8_10_11	30.5376	-224.867	0.3626285	0	0	0	0
cov3_6_7_8_10	30.5449	-224.871	0.3624291	0	0	0	0
cov3_4_7_8_10	30.5452	-224.871	0.3624498	0	0	0	0
cov3_5_8_9_10	30.5558	-224.876	0.36115	0	0	0	0
cov1_7_9_10_11	30.5619	-224.879	0.3754047	0	-0.077046243	0	0
cov1_2_7_10_11	30.5651	-224.881	0.3753365	0	-0.081974684	0	0.005610374
cov1_2_7_9_11	30.591	-224.894	0.3750232	0	-0.080655557	0	0.004699291
cov1_2_6_7_8	30.6198	-224.908	0.3699337	0	-0.16769826	0	0.003818943
cov1_8_9_10_11	30.6221	-224.909	0.3752501	0	-0.076280859	0	0
cov3_6_8_9_11	30.6244	-224.911	0.3622182	0	0	0	0
cov3_4_8_9_11	30.6245	-224.911	0.361966	0	0	0	0
cov1_2_8_10_11	30.6253	-224.911	0.3751723	0	-0.081216948	0	0.005621173
cov3_5_7_9_10	30.6268	-224.912	0.3615072	0	0	0	0
cov3_8_9_10_11	30.6273	-224.912	0.3620504	0	0	0	0
cov3_6_8_9_10	30.6392	-224.918	0.3617784	0	0	0	0
cov3_4_6_8_9	30.6397	-224.918	0.3617092	0	0	0	0
cov2_3_7_8_11	30.6403	-224.919	0.3635079	0	0	0	-0.045244101
cov3_4_8_9_10	30.6403	-224.919	0.3618777	0	0	0	0
cov1_2_8_9_11	30.6512	-224.924	0.374867	0	-0.079896576	0	0.004704898
cov3_6_8_10_11	30.6795	-224.938	0.3626226	0	0	0	0
cov3_4_8_10_11	30.6819	-224.939	0.3623035	0	0	0	0
cov3_4_5_9_11	30.6835	-224.94	0.3666081	0	0	0	0
cov3_6_7_9_11	30.6896	-224.943	0.3625941	0	0	0	0
cov3_4_7_9_11	30.6912	-224.944	0.3623094	0	0	0	0
cov3_4_6_8_10	30.6924	-224.945	0.3621786	0	0	0	0
cov3_7_9_10_11	30.6936	-224.945	0.3624011	0	0	0	0
cov3_6_7_9_10	30.7068	-224.952	0.3621295	0	0	0	0
cov3_4_7_9_10	30.7072	-224.952	0.3622268	0	0	0	0
cov3_4_6_7_9	30.7075	-224.952	0.3620865	0	0	0	0
cov3_4_5_10_11	30.708	-224.952	0.3670326	0	0	0	0
cov2_3_5_9_10	30.73	-224.963	0.3684032	0	0	0	-0.033809931
cov2_3_6_7_8	30.7332	-224.965	0.3613433	0	0	0	-0.04595098
cov3_6_7_10_11	30.7443	-224.971	0.3630064	0	0	0	0
cov3_4_7_10_11	30.7485	-224.973	0.3626568	0	0	0	0
cov2_3_6_8_11	30.7585	-224.978	0.3627821	0	0	0	-0.050463521
cov3_4_6_7_10	30.7593	-224.978	0.3625664	0	0	0	0
cov3_4_5_6_9	30.7759	-224.986	0.3652347	0	0	0	0
cov2_3_5_8_11	30.7915	-224.994	0.3632254	0	0	0	-0.044780989
cov3_4_5_6_10	30.7929	-224.995	0.3657227	0	0	0	0
cov2_4_7_8_11	30.8215	-225.009	0.3780832	0	0	0	-0.12516584
cov3_4_5_9_10	30.8469	-225.022	0.3665173	0	0	0	0
cov3_5_6_9_11	30.8485	-225.023	0.3656642	0	0	0	0
cov2_4_5_7_8	30.8487	-225.023	0.3763412	0	0	0	-0.11249867
cov2_3_6_7_11	30.8489	-225.023	0.3631624	0	0	0	-0.050497366
cov2_3_5_7_8	30.8507	-225.024	0.3627571	0	0	0	-0.03821541

cov2_4_5_7_11	30.8723	-225.035	0.3783969	0	0	0	-0.12617857
cov3_5_9_10_11	30.8763	-225.037	0.366593	0	0	0	0
cov2_3_5_7_11	30.8767	-225.037	0.3636395	0	0	0	-0.045797275
cov2_4_5_6_11	30.8785	-225.038	0.3780242	0	0	0	-0.12808439
cov2_4_5_8_11	30.8795	-225.038	0.3782792	0	0	0	-0.12607409
cov3_5_6_10_11	30.8897	-225.043	0.3661298	0	0	0	0
cov2_3_5_6_8	30.8927	-225.045	0.3608222	0	0	0	-0.043721116
cov2_4_6_7_8	30.9224	-225.06	0.3766916	0	0	0	-0.11678839
cov3_5_6_9_10	30.9588	-225.078	0.3651655	0	0	0	0
cov2_3_5_6_7	30.9924	-225.095	0.3613727	0	0	0	-0.044284556
cov1_2_9_10_11	31.0119	-225.104	0.3729225	0	-0.071637346	0	0.005529431
cov2_4_6_7_11	31.0342	-225.116	0.3784418	0	0	0	-0.12149512
cov3_4_5_7_8	31.0418	-225.119	0.3586174	0	0	0	0
cov2_4_6_8_11	31.0446	-225.121	0.3783147	0	0	0	-0.12149657
cov2_4_5_6_8	31.0461	-225.121	0.3762929	0	0	0	-0.12009994
cov2_4_5_6_7	31.048	-225.122	0.3763812	0	0	0	-0.11980288
cov3_6_9_10_11	31.0522	-225.125	0.3672358	0	0	0	0
cov3_4_6_9_11	31.0567	-225.127	0.3672193	0	0	0	0
cov3_4_9_10_11	31.0718	-225.134	0.3668281	0	0	0	0
cov3_4_6_9_10	31.0937	-225.145	0.3668555	0	0	0	0
cov3_4_5_8_11	31.0954	-225.146	0.3583932	0	0	0	0
cov3_4_6_10_11	31.1026	-225.15	0.3676718	0	0	0	0
cov3_4_5_6_8	31.1546	-225.176	0.3577551	0	0	0	0
cov3_5_7_8_11	31.2019	-225.199	0.3587255	0	0	0	0
cov3_4_5_7_11	31.2093	-225.203	0.3586761	0	0	0	0
cov3_5_6_7_8	31.2594	-225.228	0.3579468	0	0	0	0
cov3_4_5_6_7	31.2706	-225.234	0.3582302	0	0	0	0
cov1_2_7_8_10	31.2894	-225.243	0.3711556	0	-0.042778647	0	0.01138235
cov2_6_7_8_9	31.3018	-225.249	0.3718122	0	0	0	-0.048174833
cov1_7_8_9_10	31.3138	-225.255	0.3713938	0	-0.03266855	0	0
cov2_6_7_8_10	31.3232	-225.26	0.3723342	0	0	0	-0.047824534
cov3_5_6_8_11	31.3307	-225.264	0.3583815	0	0	0	0
cov1_2_7_8_9	31.335	-225.266	0.3708082	0	-0.041165157	0	0.010495795
cov2_5_7_8_9	31.3446	-225.271	0.3734087	0	0	0	-0.047598272
cov2_5_7_8_10	31.3688	-225.283	0.3737646	0	0	0	-0.047179126
cov3_6_7_8_11	31.3839	-225.29	0.3609402	0	0	0	0
cov3_4_6_7_8	31.3907	-225.294	0.3608455	0	0	0	0
cov2_7_8_9_11	31.3933	-225.295	0.3741947	0	0	0	-0.039818853
cov2_7_8_10_11	31.4079	-225.302	0.3745695	0	0	0	-0.039814514
cov2_5_7_9_11	31.4367	-225.317	0.3748163	0	0	0	-0.054099238
cov3_5_6_7_11	31.4398	-225.318	0.3588737	0	0	0	0
cov2_6_7_9_11	31.4597	-225.328	0.3734874	0	0	0	-0.051935136
cov2_3_5_6_11	31.4693	-225.333	0.3703761	0	0	0	-0.06084832
cov2_5_6_7_9	31.471	-225.334	0.3729381	0	0	0	-0.053813495
cov2_5_7_10_11	31.4719	-225.334	0.3751457	0	0	0	-0.053448447
cov2_5_8_9_11	31.4744	-225.336	0.3746778	0	0	0	-0.05386151
cov3_4_7_8_11	31.4895	-225.343	0.3593888	0	0	0	0

cov2_6_7_10_11	31.4902	-225.344	0.3739369	0	0	0	-0.051326379
cov3_4_6_8_11	31.4978	-225.347	0.3610737	0	0	0	0
cov2_6_8_9_11	31.4994	-225.348	0.3733245	0	0	0	-0.051605755
cov2_5_6_8_9	31.5023	-225.35	0.3728375	0	0	0	-0.053732954
cov2_5_6_7_10	31.5032	-225.35	0.3733651	0	0	0	-0.053085738
cov2_5_8_10_11	31.5094	-225.353	0.3750036	0	0	0	-0.053216315
cov1_2_7_9_10	31.515	-225.356	0.3711682	0	-0.041892866	0	0.011102534
cov2_6_8_10_11	31.5296	-225.363	0.3737744	0	0	0	-0.050999199
cov2_5_6_8_10	31.5346	-225.366	0.3732656	0	0	0	-0.05300891
cov2_6_7_9_10	31.5422	-225.369	0.3721058	0	0	0	-0.046384941
cov1_2_8_9_10	31.5476	-225.372	0.3710761	0	-0.041754465	0	0.011030126
cov2_5_7_9_10	31.5534	-225.375	0.3736145	0	0	0	-0.047083518
cov2_6_8_9_10	31.5747	-225.386	0.3719972	0	0	0	-0.046263807
cov2_5_8_9_10	31.5832	-225.39	0.373516	0	0	0	-0.047062034
cov3_4_6_7_11	31.586	-225.391	0.3614491	0	0	0	0
cov2_5_6_9_11	31.5884	-225.393	0.3725995	0	0	0	-0.055648741
cov2_7_9_10_11	31.5993	-225.398	0.3744252	0	0	0	-0.039341705
cov1_2_7_8_11	31.6032	-225.4	0.3762371	0	-0.099207307	0	0.002017982
cov2_5_6_9_10	31.6063	-225.402	0.3720797	0	0	0	-0.053038828
cov2_5_6_10_11	31.626	-225.411	0.3729858	0	0	0	-0.054818346
cov2_5_9_10_11	31.64	-225.418	0.3733683	0	0	0	-0.051186906
cov2_8_9_10_11	31.6409	-225.419	0.3742774	0	0	0	-0.038949317
cov2_6_9_10_11	31.6482	-225.423	0.3719735	0	0	0	-0.049374385
cov2_7_8_9_10	31.6635	-225.43	0.3719988	0	0	0	-0.022071381
cov4_5_7_8_10	31.6898	-225.443	0.3710366	0	0	0	0
cov4_5_7_8_9	31.7021	-225.449	0.3705778	0	0	0	0
cov4_5_7_9_10	31.8779	-225.537	0.3709146	0	0	0	0
cov4_5_8_9_10	31.8973	-225.547	0.370814	0	0	0	0
cov4_5_7_10_11	31.9012	-225.549	0.3713644	0	0	0	0
cov4_6_7_8_9	31.9058	-225.551	0.3715239	0	0	0	0
cov4_5_6_7_10	31.9082	-225.553	0.3710773	0	0	0	0
cov4_5_7_9_11	31.9113	-225.554	0.3709182	0	0	0	0
cov4_6_7_8_10	31.9188	-225.558	0.3720297	0	0	0	0
cov4_5_6_7_9	31.9202	-225.559	0.3706097	0	0	0	0
cov4_5_8_10_11	31.9221	-225.559	0.3712379	0	0	0	0
cov4_5_6_8_10	31.9276	-225.562	0.3709798	0	0	0	0
cov4_5_8_9_11	31.9324	-225.565	0.3707914	0	0	0	0
cov4_5_6_8_9	31.9395	-225.568	0.3705126	0	0	0	0
cov4_5_6_9_10	31.9415	-225.569	0.3701763	0	0	0	0
cov4_5_9_10_11	31.9417	-225.569	0.3703487	0	0	0	0
cov4_7_8_9_10	31.9458	-225.571	0.3713205	0	0	0	0
cov4_7_8_9_11	31.9551	-225.576	0.3707811	0	0	0	0
cov4_5_6_10_11	31.9722	-225.585	0.3705018	0	0	0	0
cov4_7_8_10_11	31.9746	-225.586	0.3712086	0	0	0	0
cov4_5_6_9_11	31.9835	-225.59	0.3700636	0	0	0	0
cov5_7_8_9_10	31.9999	-225.598	0.3712072	0	0	0	0
cov7_8_9_10_11	32.003	-225.6	0.3713262	0	0	0	0

cov6_7_8_9_10	32.0036	-225.6	0.3712689	0	0	0	0
cov5_7_8_9_11	32.0228	-225.61	0.3712379	0	0	0	0
cov5_6_7_8_9	32.0294	-225.613	0.3708545	0	0	0	0
cov6_7_8_9_11	32.0299	-225.613	0.3713188	0	0	0	0
cov5_7_8_10_11	32.0313	-225.614	0.3716852	0	0	0	0
cov5_6_7_8_10	32.0363	-225.617	0.3713426	0	0	0	0
cov6_7_8_10_11	32.0419	-225.619	0.3717931	0	0	0	0
cov4_6_7_9_10	32.0815	-225.639	0.3718645	0	0	0	0
cov4_6_8_9_10	32.105	-225.651	0.3717805	0	0	0	0
cov4_6_7_9_11	32.1067	-225.652	0.371736	0	0	0	0
cov4_6_7_10_11	32.1189	-225.658	0.3721841	0	0	0	0
cov4_6_8_9_11	32.1301	-225.663	0.3716091	0	0	0	0
cov4_6_8_10_11	32.1423	-225.67	0.3720567	0	0	0	0
cov4_7_9_10_11	32.1446	-225.671	0.3710629	0	0	0	0
cov4_8_9_10_11	32.1678	-225.682	0.3709328	0	0	0	0
cov4_6_9_10_11	32.1772	-225.687	0.3708989	0	0	0	0
cov5_7_9_10_11	32.2114	-225.704	0.3715476	0	0	0	0
cov6_7_9_10_11	32.2167	-225.707	0.3716953	0	0	0	0
cov5_6_7_9_10	32.2196	-225.708	0.371247	0	0	0	0
cov5_8_9_10_11	32.2425	-225.72	0.3714205	0	0	0	0
cov5_6_7_9_11	32.2437	-225.72	0.3714133	0	0	0	0
cov6_8_9_10_11	32.2471	-225.722	0.3715651	0	0	0	0
cov5_6_8_9_10	32.2489	-225.723	0.3711534	0	0	0	0
cov5_6_7_10_11	32.2525	-225.725	0.3718637	0	0	0	0
cov5_6_8_9_11	32.2745	-225.736	0.3712814	0	0	0	0
cov5_6_8_10_11	32.2834	-225.74	0.3717317	0	0	0	0
cov3_4_5_6_11	32.3274	-225.762	0.3680499	0	0	0	0
cov5_6_9_10_11	32.3613	-225.779	0.3702854	0	0	0	0
cov2_5_6_7_8	32.6428	-225.92	0.375625	0	0	0	-0.069507748
cov2_5_7_8_11	32.6531	-225.925	0.3760584	0	0	0	-0.077870075
cov2_5_6_7_11	32.8177	-226.007	0.3768157	0	0	0	-0.071656259
cov2_5_6_8_11	32.8456	-226.021	0.3766672	0	0	0	-0.071396742
cov1_5_6_7_8_11	32.9772	-222.577	0.3764746	0	-0.32321234	0	0
cov1_3_5_6_9_11	33.197	-222.687	0.3703675	0	-0.26471032	0	0
cov1_5_6_7_9_11	33.2105	-222.694	0.376048	0	-0.29950002	0	0
cov1_3_5_6_10_11	33.2144	-222.696	0.3705501	0	-0.26561142	0	0
cov2_6_7_8_11	33.2153	-226.206	0.3753779	0	0	0	-0.058502515
cov1_5_6_7_10_11	33.2222	-222.699	0.3762078	0	-0.29989523	0	0
cov1_3_4_5_6_11	33.2791	-222.728	0.3713492	0	-0.28159137	0	0
cov1_5_6_8_9_11	33.2994	-222.738	0.3758496	0	-0.29773852	0	0
cov1_3_5_6_7_11	33.3016	-222.739	0.3735745	0	-0.29820432	0	0
cov1_5_6_8_10_11	33.311	-222.744	0.3760099	0	-0.29814197	0	0
cov1_4_5_6_7_11	33.3327	-222.755	0.3766239	0	-0.31522008	0	0
cov1_3_5_6_8_11	33.3376	-222.757	0.3725981	0	-0.29190329	0	0
cov1_2_3_5_6_11	33.3562	-222.766	0.3711881	0	-0.2798559	0	-0.003799649
cov1_2_5_6_7_11	33.3812	-222.779	0.3769131	0	-0.31337585	0	-0.006413968
cov1_4_5_6_8_11	33.4134	-222.795	0.3764276	0	-0.31329995	0	0

cov1_2_5_6_8_11	33.4688	-222.823	0.3767162	0	-0.31159066	0	-0.006372646
cov1_3_4_6_9_11	33.549	-222.863	0.3689571	0	-0.21593436	0	0
cov1_3_4_6_10_11	33.5587	-222.868	0.3692642	0	-0.21657534	0	0
cov1_4_5_6_9_11	33.6301	-222.903	0.3734301	0	-0.27950478	0	0
cov1_2_4_5_6_11	33.6312	-222.904	0.3756135	0	-0.26987513	0	-0.037117126
cov1_4_5_6_10_11	33.6374	-222.907	0.3735957	0	-0.27960085	0	0
cov1_5_6_7_8_9	33.6483	-222.912	0.3719414	0	-0.26683508	0	0
cov1_5_6_7_8_10	33.6536	-222.915	0.372117	0	-0.26701389	0	0
cov1_2_3_4_6_11	33.719	-222.948	0.3725141	0	-0.20537233	0	-0.052888162
cov4_5_6_7_8	33.7299	-226.463	0.3737345	0	0	0	0
cov4_6_7_8_11	33.7306	-226.464	0.3736185	0	0	0	0
cov1_3_5_6_7_8	33.7665	-222.972	0.3693949	0	-0.2660232	0	0
cov1_4_5_6_7_8	33.7884	-222.983	0.3725683	0	-0.28342413	0	0
cov1_2_5_6_9_11	33.8113	-222.994	0.3734385	0	-0.28116405	0	-0.004320387
cov1_5_6_9_10_11	33.8154	-222.996	0.3733024	0	-0.28402261	0	0
cov1_2_4_6_9_11	33.8205	-222.999	0.3748007	0	-0.19724067	0	-0.058146839
cov1_2_5_6_10_11	33.8228	-223	0.3735887	0	-0.28183738	0	-0.004006355
cov1_2_4_6_10_11	33.825	-223.001	0.3750283	0	-0.19806295	0	-0.057371683
cov1_2_5_6_7_8	33.8599	-223.018	0.3726201	0	-0.28524261	0	-0.001182492
cov1_3_4_5_6_9	33.8674	-223.022	0.3685326	0	-0.23547717	0	0
cov1_3_4_5_6_10	33.8744	-223.026	0.3687388	0	-0.23579837	0	0
cov4_5_6_7_11	33.92	-226.558	0.3737727	0	0	0	0
cov4_5_6_8_11	33.9302	-226.564	0.3736422	0	0	0	0
cov1_4_5_6_7_9	33.9579	-223.067	0.3720858	0	-0.25742019	0	0
cov1_4_5_6_7_10	33.9596	-223.068	0.3722679	0	-0.25734705	0	0
cov1_3_5_6_7_9	33.9682	-223.072	0.3691008	0	-0.24266178	0	0
cov1_2_3_5_6_9	33.9747	-223.076	0.3681693	0	-0.23933505	0	0.002258886
cov1_3_5_6_9_10	33.9753	-223.076	0.3682723	0	-0.23761059	0	0
cov1_3_5_6_8_9	33.9758	-223.076	0.3682291	0	-0.23767281	0	0
cov1_3_5_6_7_10	33.9778	-223.077	0.369355	0	-0.24350656	0	0
cov1_2_3_5_6_10	33.985	-223.081	0.3683533	0	-0.24011856	0	0.002603468
cov1_3_5_6_8_10	33.9865	-223.082	0.3684811	0	-0.23855076	0	0
cov5_6_7_8_11	33.9979	-226.597	0.3738914	0	0	0	0
cov1_3_4_6_8_11	34.0028	-223.09	0.3664842	0	-0.21911123	0	0
cov1_4_5_6_8_9	34.0064	-223.092	0.3719606	0	-0.25651173	0	0
cov1_4_5_6_8_10	34.0082	-223.092	0.3721413	0	-0.2564381	0	0
cov1_2_3_4_5_6	34.0395	-223.108	0.3700973	0	-0.2406622	0	-0.019886517
cov1_5_6_7_9_10	34.0529	-223.115	0.3722295	0	-0.26131131	0	0
cov1_2_5_6_7_9	34.0552	-223.116	0.3721632	0	-0.26200981	0	0.000516847
cov1_3_4_6_7_11	34.0578	-223.117	0.3670652	0	-0.22060906	0	0
cov1_2_4_5_6_9	34.058	-223.117	0.3718911	0	-0.22882303	0	-0.028118703
cov1_2_5_6_7_10	34.0604	-223.119	0.3723293	0	-0.26247442	0	0.000915413
cov1_2_4_5_6_10	34.062	-223.119	0.3720674	0	-0.22910459	0	-0.027826024
cov1_4_6_7_10_11	34.0658	-223.121	0.3741489	0	-0.22936194	0	0
cov1_4_6_7_9_11	34.0807	-223.129	0.3738418	0	-0.22894462	0	0
cov1_3_4_5_6_8	34.0883	-223.132	0.3686158	0	-0.25204026	0	0
cov1_3_4_5_6_7	34.0925	-223.135	0.369476	0	-0.25720613	0	0

cov1_5_6_8_9_10	34.1093	-223.143	0.3721051	0	-0.26051954	0	0
cov1_2_5_6_8_9	34.1115	-223.144	0.3720414	0	-0.26116416	0	0.000450536
cov1_2_5_6_8_10	34.1168	-223.147	0.3722088	0	-0.26163896	0	0.000844949
cov1_4_6_8_10_11	34.1266	-223.152	0.3739716	0	-0.2283251	0	0
cov1_2_4_5_6_7	34.1299	-223.153	0.3733665	0	-0.25930917	0	-0.021824184
cov1_4_6_8_9_11	34.1414	-223.159	0.3736663	0	-0.22791284	0	0
cov1_2_3_4_6_9	34.154	-223.165	0.3686873	0	-0.16779375	0	-0.038241989
cov1_2_3_4_6_10	34.1552	-223.166	0.3689788	0	-0.16890715	0	-0.037623379
cov1_3_6_8_9_11	34.1606	-223.169	0.3638324	0	-0.17630805	0	0
cov1_4_5_6_9_10	34.1649	-223.171	0.3710621	0	-0.25200584	0	0
cov1_2_4_5_6_8	34.1713	-223.174	0.3732719	0	-0.25754997	0	-0.022795299
cov1_2_3_5_6_7	34.1728	-223.175	0.3695708	0	-0.26086755	0	0.000371447
cov1_2_3_5_6_8	34.176	-223.176	0.3686904	0	-0.25594504	0	0.000673869
cov1_3_6_8_10_11	34.1887	-223.183	0.3642118	0	-0.17695072	0	0
cov1_2_4_6_7_11	34.1925	-223.185	0.3775891	0	-0.21136501	0	-0.06802833
cov1_3_6_7_9_11	34.2111	-223.194	0.3643973	0	-0.17706919	0	0
cov1_3_4_6_8_9	34.2317	-223.204	0.3638154	0	-0.17899218	0	0
cov1_3_4_6_8_10	34.2357	-223.206	0.3642234	0	-0.18002425	0	0
cov1_2_4_6_8_11	34.2366	-223.207	0.3774306	0	-0.21022519	0	-0.068289523
cov1_3_6_7_10_11	34.238	-223.207	0.3647899	0	-0.17773371	0	0
cov4_5_7_8_11	34.2547	-226.726	0.371344	0	0	0	0
cov1_3_4_6_7_9	34.2842	-223.23	0.364353	0	-0.17975752	0	0
cov1_3_4_6_7_10	34.2864	-223.232	0.364774	0	-0.18082097	0	0
cov1_3_6_9_10_11	34.2883	-223.232	0.3667062	0	-0.18173161	0	0
cov1_2_3_6_9_11	34.2937	-223.235	0.3664909	0	-0.18398231	0	0.003030752
cov1_2_3_6_10_11	34.314	-223.245	0.3668234	0	-0.18508846	0	0.003790641
cov1_2_5_6_9_10	34.3391	-223.258	0.3709472	0	-0.25703707	0	5.63E-05
cov1_4_6_9_10_11	34.3571	-223.267	0.3722957	0	-0.22289454	0	0
cov1_3_4_6_9_10	34.3655	-223.271	0.3668271	0	-0.18512524	0	0
cov1_4_6_7_8_10	34.3854	-223.281	0.3704096	0	-0.20203273	0	0
cov1_4_6_7_8_9	34.4132	-223.295	0.3700732	0	-0.20141556	0	0
cov1_2_4_6_7_10	34.4163	-223.296	0.3723477	0	-0.17304032	0	-0.047525131
cov1_2_4_6_7_9	34.4262	-223.301	0.3720762	0	-0.17190953	0	-0.048512741
cov1_2_4_6_8_10	34.4406	-223.309	0.3722626	0	-0.17266228	0	-0.047998253
cov1_3_4_6_7_8	34.4489	-223.313	0.3633089	0	-0.1925344	0	0
cov1_2_4_6_8_9	34.4501	-223.313	0.3719931	0	-0.17154341	0	-0.04897383
cov1_2_4_6_9_10	34.4706	-223.324	0.3718073	0	-0.17142802	0	-0.049766961
cov1_2_3_4_6_8	34.4927	-223.335	0.3657963	0	-0.16804481	0	-0.04203143
cov1_3_6_7_8_11	34.5458	-223.361	0.3627044	0	-0.18658241	0	0
cov1_2_3_4_6_7	34.5599	-223.368	0.3663665	0	-0.16843528	0	-0.04300239
cov1_4_6_7_8_11	34.5719	-223.374	0.3748511	0	-0.25074692	0	0
cov1_3_6_7_8_9	34.5762	-223.376	0.3605802	0	-0.14905361	0	0
cov1_3_6_7_8_10	34.5913	-223.384	0.3610245	0	-0.14996995	0	0
cov1_2_4_6_7_8	34.7058	-223.441	0.373189	0	-0.18393769	0	-0.061222619
cov1_4_5_7_8_11	34.7317	-223.454	0.3784729	0	-0.21707671	0	0
cov1_3_5_7_8_11	34.7515	-223.464	0.3756621	0	-0.19983645	0	0
cov1_4_6_7_9_10	34.7828	-223.48	0.370511	0	-0.19632581	0	0

cov1_5_7_8_9_11	34.7991	-223.488	0.3784635	0	-0.20842865	0	0
cov1_5_7_8_10_11	34.8034	-223.49	0.3785404	0	-0.20898453	0	0
cov1_2_3_4_5_11	34.8104	-223.493	0.3747196	0	-0.16470609	0	-0.02792117
cov1_4_6_8_9_10	34.8147	-223.496	0.3704053	0	-0.19613796	0	0
cov1_2_5_7_8_11	34.8345	-223.506	0.3788381	0	-0.2167817	0	-0.003173018
cov1_2_4_5_7_11	34.8618	-223.519	0.3794338	0	-0.19307827	0	-0.029235953
cov1_3_4_5_9_11	34.8658	-223.521	0.3731623	0	-0.17284195	0	0
cov1_3_4_5_10_11	34.8717	-223.524	0.3732646	0	-0.17358866	0	0
cov1_2_3_6_8_11	34.876	-223.526	0.3622598	0	-0.18187685	0	0.002209927
cov1_3_4_5_7_11	34.8826	-223.53	0.3753569	0	-0.19684695	0	0
cov1_2_3_6_8_9	34.8933	-223.535	0.360031	0	-0.14993525	0	0.008195168
cov1_3_6_8_9_10	34.902	-223.539	0.3604355	0	-0.14362385	0	0
cov1_3_4_5_8_11	34.9032	-223.54	0.374594	0	-0.19206849	0	0
cov1_2_3_6_8_10	34.9077	-223.542	0.3604504	0	-0.15134304	0	0.008848159
cov1_2_4_5_8_11	34.917	-223.547	0.3792954	0	-0.19146046	0	-0.029956441
cov1_4_5_7_9_11	34.9208	-223.549	0.3782199	0	-0.20394723	0	0
cov1_4_5_7_10_11	34.9241	-223.55	0.3783057	0	-0.2042195	0	0
cov1_3_5_7_9_11	34.9565	-223.567	0.3755225	0	-0.18932872	0	0
cov1_2_3_6_7_11	34.9624	-223.57	0.362777	0	-0.18232091	0	0.002096402
cov1_3_5_7_10_11	34.9626	-223.57	0.3756313	0	-0.1903147	0	0
cov1_2_3_6_7_9	34.9684	-223.573	0.3605175	0	-0.14992205	0	0.008188369
cov1_3_6_7_9_10	34.9759	-223.576	0.3609388	0	-0.14365056	0	0
cov1_2_3_6_7_10	34.9811	-223.579	0.3609504	0	-0.15137787	0	0.008859693
cov1_4_5_8_9_11	34.9813	-223.579	0.3780529	0	-0.20290082	0	0
cov1_4_5_8_10_11	34.9845	-223.581	0.3781405	0	-0.20313651	0	0
cov1_3_5_8_9_11	34.9859	-223.581	0.3747619	0	-0.18473379	0	0
cov1_3_5_8_10_11	34.9923	-223.584	0.3748693	0	-0.18576274	0	0
cov1_2_3_5_7_11	34.9939	-223.585	0.3758039	0	-0.19850565	0	-0.001771977
cov1_3_5_9_10_11	35.0129	-223.595	0.3729673	0	-0.17470776	0	0
cov1_2_3_5_9_11	35.0146	-223.596	0.3730113	0	-0.17441166	0	8.16E-05
cov1_2_3_5_10_11	35.0214	-223.599	0.3731011	0	-0.1755218	0	0.000151701
cov1_2_3_5_8_11	35.0225	-223.6	0.375022	0	-0.19396503	0	-0.001443991
cov1_6_7_8_10_11	35.0292	-223.603	0.3727724	0	-0.19088482	0	0
cov1_2_5_7_9_11	35.0387	-223.608	0.3786382	0	-0.20623939	0	-0.002435053
cov1_5_7_9_10_11	35.04	-223.608	0.3785346	0	-0.20805858	0	0
cov1_2_5_7_10_11	35.0434	-223.61	0.3787142	0	-0.20691893	0	-0.002303832
cov1_6_7_8_9_11	35.0476	-223.612	0.3723638	0	-0.19022254	0	0
cov1_2_3_4_9_11	35.0559	-223.616	0.3738624	0	-0.10400573	0	-0.04526669
cov1_2_3_4_10_11	35.0638	-223.62	0.3740357	0	-0.10476452	0	-0.044984397
cov1_2_5_8_9_11	35.1067	-223.642	0.3784783	0	-0.20527603	0	-0.002419633
cov1_5_8_9_10_11	35.1079	-223.642	0.3783752	0	-0.20708429	0	0
cov1_2_5_8_10_11	35.1114	-223.644	0.3785544	0	-0.20595528	0	-0.00228835
cov1_2_3_6_9_10	35.1727	-223.675	0.3639383	0	-0.15454275	0	0.008553259
cov1_2_4_5_9_11	35.1747	-223.676	0.3772656	0	-0.16097193	0	-0.037408894
cov1_2_4_5_10_11	35.1787	-223.678	0.3773524	0	-0.16148771	0	-0.03725623
cov1_2_3_4_8_11	35.2055	-223.691	0.3722485	0	-0.10919825	0	-0.047988104
cov1_2_3_4_7_11	35.233	-223.705	0.3727511	0	-0.11036347	0	-0.048857007

cov1_3_4_8_9_11	35.3142	-223.745	0.3694022	0	-0.11722366	0	0
cov1_3_4_8_10_11	35.3204	-223.749	0.369654	0	-0.11796685	0	0
cov1_3_4_7_9_11	35.3396	-223.758	0.3698585	0	-0.11838672	0	0
cov1_3_4_7_10_11	35.3451	-223.761	0.3701204	0	-0.11913175	0	0
cov1_3_4_7_8_11	35.3579	-223.767	0.3691734	0	-0.13031714	0	0
cov1_2_3_6_7_8	35.3591	-223.768	0.3586886	0	-0.15743159	0	0.006958057
cov1_4_5_9_10_11	35.3669	-223.772	0.3758851	0	-0.19003172	0	0
cov1_3_4_9_10_11	35.3693	-223.773	0.3714108	0	-0.12220838	0	0
cov1_2_4_7_10_11	35.385	-223.781	0.3785839	0	-0.1098398	0	-0.05652661
cov1_2_4_7_9_11	35.386	-223.781	0.3784132	0	-0.10914824	0	-0.057181337
cov1_2_4_8_10_11	35.4235	-223.8	0.3784609	0	-0.10927279	0	-0.056816171
cov1_2_4_8_9_11	35.4244	-223.8	0.3782906	0	-0.10858386	0	-0.057466439
cov1_6_7_9_10_11	35.4367	-223.807	0.3727905	0	-0.18456885	0	0
cov1_2_6_7_10_11	35.4556	-223.816	0.3728874	0	-0.18711044	0	0.00280837
cov1_2_4_7_8_11	35.4629	-223.82	0.3792189	0	-0.12163993	0	-0.066009644
cov1_2_6_7_9_11	35.4753	-223.826	0.3725105	0	-0.18563459	0	0.001735978
cov1_6_8_9_10_11	35.518	-223.847	0.3725782	0	-0.1830515	0	0
cov1_2_6_8_10_11	35.5368	-223.857	0.3726886	0	-0.18561351	0	0.002830083
cov1_2_6_8_9_11	35.5564	-223.867	0.3723112	0	-0.18414195	0	0.001760304
cov1_2_4_9_10_11	35.5825	-223.88	0.3772271	0	-0.10420692	0	-0.059315927
cov1_2_5_9_10_11	35.6095	-223.893	0.3759874	0	-0.19256292	0	-0.002086777
cov1_4_7_8_10_11	35.6682	-223.922	0.3761461	0	-0.1348079	0	0
cov1_4_7_8_9_11	35.6846	-223.931	0.3759155	0	-0.13459156	0	0
cov1_3_4_5_7_8	35.7135	-223.945	0.3711622	0	-0.14831284	0	0
cov1_4_5_7_8_10	35.7462	-223.961	0.3742033	0	-0.15486348	0	0
cov1_4_5_7_8_9	35.7477	-223.962	0.3741132	0	-0.1550471	0	0
cov1_2_4_5_7_8	35.7505	-223.964	0.3750243	0	-0.1503371	0	-0.021689996
cov1_6_7_8_9_10	35.7527	-223.965	0.3688905	0	-0.15724918	0	0
cov1_2_6_7_8_10	35.7546	-223.966	0.3688672	0	-0.16378523	0	0.007535919
cov1_2_6_7_8_9	35.7939	-223.985	0.3684418	0	-0.16191609	0	0.006490722
cov1_3_5_7_8_9	35.8106	-223.994	0.3712898	0	-0.13971563	0	0
cov1_3_5_7_8_10	35.8135	-223.995	0.3714089	0	-0.14029913	0	0
cov1_2_3_4_5_9	35.8236	-224	0.3719105	0	-0.12035918	0	-0.019825485
cov1_2_3_4_5_10	35.8254	-224.001	0.3720155	0	-0.12070176	0	-0.019706526
cov1_2_3_5_7_8	35.8514	-224.014	0.3712971	0	-0.15412954	0	0.004478221
cov1_3_4_5_8_9	35.8701	-224.023	0.3703134	0	-0.1312579	0	0
cov1_3_4_5_8_10	35.8717	-224.024	0.3704504	0	-0.1316664	0	0
cov1_3_4_5_9_10	35.8757	-224.026	0.3711356	0	-0.13542543	0	0
cov1_3_4_5_7_9	35.8764	-224.027	0.3709585	0	-0.13485314	0	0
cov1_3_4_5_7_10	35.8776	-224.027	0.3710985	0	-0.13522341	0	0
cov1_2_3_4_5_8	35.8795	-224.028	0.3712966	0	-0.12822982	0	-0.020369162
cov1_2_3_4_5_7	35.8889	-224.033	0.3719284	0	-0.13222338	0	-0.020050508
cov1_2_5_7_8_9	35.8962	-224.036	0.374274	0	-0.16170524	0	0.003834778
cov1_2_5_7_8_10	35.8967	-224.037	0.3743523	0	-0.16192367	0	0.004033354
cov1_5_7_8_9_10	35.8989	-224.038	0.3744259	0	-0.15854654	0	0
cov1_4_7_9_10_11	35.9026	-224.04	0.3761501	0	-0.13402701	0	0
cov1_2_4_5_7_10	35.9088	-224.043	0.3748647	0	-0.13603678	0	-0.021717701

cov1_2_4_5_7_9	35.9093	-224.043	0.3747723	0	-0.13596171	0	-0.02188907
cov1_2_6_9_10_11	35.9329	-224.055	0.3702699	0	-0.17528714	0	0.002632326
cov1_2_4_5_8_10	35.9351	-224.056	0.3747842	0	-0.13510219	0	-0.022454127
cov1_2_4_5_8_9	35.9356	-224.056	0.3746915	0	-0.1350234	0	-0.022625081
cov1_4_8_9_10_11	35.9464	-224.062	0.3760094	0	-0.1335768	0	0
cov1_3_7_8_9_11	35.9658	-224.071	0.3663472	0	-0.073975773	0	0
cov1_4_5_7_9_10	35.9716	-224.074	0.3742678	0	-0.15446273	0	0
cov1_3_7_8_10_11	35.9824	-224.08	0.3666359	0	-0.074691965	0	0
cov1_4_5_8_9_10	36.0023	-224.089	0.3741661	0	-0.15415499	0	0
cov1_2_4_5_9_10	36.0148	-224.096	0.3741743	0	-0.12891745	0	-0.027140631
cov1_2_3_5_7_9	36.03	-224.103	0.3711309	0	-0.14300459	0	0.005238537
cov1_2_3_5_8_9	36.0309	-224.104	0.3704706	0	-0.13958487	0	0.005451722
cov1_2_3_5_9_10	36.0315	-224.104	0.3707435	0	-0.14090263	0	0.005400722
cov1_2_3_5_7_10	36.0327	-224.105	0.3712511	0	-0.14367505	0	0.005396745
cov1_2_3_5_8_10	36.034	-224.105	0.3705892	0	-0.14029031	0	0.005602991
cov1_3_5_7_9_10	36.0362	-224.106	0.3713569	0	-0.13929856	0	0
cov1_3_5_8_9_10	36.0377	-224.107	0.3707139	0	-0.13575817	0	0
cov1_2_3_4_8_9	36.0744	-224.126	0.3681592	0	-0.059493261	0	-0.035660874
cov1_2_3_4_8_10	36.0759	-224.126	0.3683924	0	-0.060360124	0	-0.035383654
cov1_3_4_7_8_10	36.0904	-224.133	0.3664085	0	-0.075891478	0	0
cov1_3_4_7_8_9	36.0936	-224.135	0.366115	0	-0.075132653	0	0
cov1_2_3_4_7_8	36.0982	-224.137	0.3682156	0	-0.068026899	0	-0.040974337
cov1_2_3_4_7_9	36.1134	-224.145	0.3685838	0	-0.059878889	0	-0.036246062
cov1_2_3_4_7_10	36.1141	-224.145	0.3688255	0	-0.060758488	0	-0.035950706
cov1_2_5_7_9_10	36.1232	-224.15	0.3744132	0	-0.16130749	0	0.003829509
cov1_2_3_8_9_11	36.1476	-224.162	0.3657412	0	-0.07793323	0	0.006453336
cov1_3_8_9_10_11	36.156	-224.166	0.3660525	0	-0.072865193	0	0
cov1_2_5_8_9_10	36.1612	-224.169	0.3743173	0	-0.1610311	0	0.003762155
cov1_2_3_8_10_11	36.1637	-224.17	0.3660106	0	-0.079018399	0	0.006933262
cov1_2_6_7_9_10	36.1642	-224.17	0.3689315	0	-0.15733176	0	0.007254985
cov1_2_3_7_9_11	36.1919	-224.184	0.3661567	0	-0.078485963	0	0.006381129
cov1_3_7_9_10_11	36.1997	-224.188	0.3664768	0	-0.073480972	0	0
cov1_2_3_7_10_11	36.2074	-224.192	0.366436	0	-0.079587047	0	0.006876034
cov1_2_3_4_9_10	36.2092	-224.193	0.3712395	0	-0.062755089	0	-0.039249208
cov1_2_6_8_9_10	36.2152	-224.196	0.3688118	0	-0.15665661	0	0.00717594
cov1_2_4_7_8_10	36.2355	-224.206	0.3741654	0	-0.067517191	0	-0.048543224
cov1_2_4_7_8_9	36.2481	-224.212	0.37398	0	-0.066756071	0	-0.049350789
cov1_3_4_8_9_10	36.2634	-224.22	0.3659809	0	-0.0745414	0	0
cov1_3_4_7_9_10	36.308	-224.242	0.3663782	0	-0.075177723	0	0
cov1_2_3_9_10_11	36.3143	-224.245	0.3689763	0	-0.082736497	0	0.006199738
cov2_3_4_5_9_11	36.361	-224.269	0.3727506	0	0	0	-0.085319209
cov1_2_3_7_8_11	36.3763	-224.276	0.3649167	0	-0.08839883	0	0.005263507
cov2_3_4_5_10_11	36.3903	-224.283	0.3730635	0	0	0	-0.085340327
cov2_3_4_5_6_9	36.4331	-224.305	0.3697937	0	0	0	-0.087796829
cov1_2_4_7_9_10	36.4547	-224.316	0.3742146	0	-0.066904564	0	-0.048861814
cov2_3_4_5_6_10	36.4584	-224.318	0.3702162	0	0	0	-0.08761989
cov1_2_4_8_9_10	36.4665	-224.322	0.3741467	0	-0.066906719	0	-0.049211943

cov2_3_4_8_9_11	36.5453	-224.361	0.3694547	0	0	0	-0.079366308
cov2_3_4_7_9_11	36.587	-224.382	0.3699025	0	0	0	-0.080307368
cov2_3_4_8_10_11	36.5912	-224.384	0.3697207	0	0	0	-0.079835939
cov2_3_4_5_8_9	36.5949	-224.386	0.3666348	0	0	0	-0.06099888
cov2_3_4_7_8_9	36.6038	-224.39	0.3681418	0	0	0	-0.068052251
cov2_3_4_5_8_10	36.6119	-224.394	0.3669122	0	0	0	-0.06124123
cov2_3_4_6_8_9	36.6145	-224.396	0.3672393	0	0	0	-0.077880651
cov1_4_7_8_9_10	36.6225	-224.4	0.3723437	0	-0.091045091	0	0
cov2_3_4_7_10_11	36.6331	-224.405	0.3701765	0	0	0	-0.080764596
cov2_3_4_7_8_10	36.6335	-224.405	0.3684596	0	0	0	-0.068742588
cov2_3_4_5_8_11	36.6422	-224.409	0.367698	0	0	0	-0.089328381
cov2_3_4_5_7_9	36.6511	-224.414	0.3670672	0	0	0	-0.061900481
cov2_3_4_6_8_10	36.6622	-224.419	0.3676339	0	0	0	-0.078116106
cov2_3_4_6_7_9	36.6671	-224.422	0.3676906	0	0	0	-0.078509603
cov2_3_4_5_7_10	36.6679	-224.422	0.367352	0	0	0	-0.062134471
cov2_3_4_6_9_11	36.6818	-224.429	0.3725177	0	0	0	-0.090066615
cov2_3_4_9_10_11	36.7104	-224.444	0.372882	0	0	0	-0.086677163
cov2_3_4_6_7_10	36.7143	-224.445	0.3680922	0	0	0	-0.078730697
cov2_3_4_5_7_11	36.718	-224.447	0.3681245	0	0	0	-0.090542443
cov2_3_4_6_10_11	36.7338	-224.455	0.3728852	0	0	0	-0.090049261
cov2_4_5_6_9_11	36.7461	-224.461	0.375285	0	0	0	-0.11208122
cov2_3_4_5_6_8	36.7526	-224.465	0.3640257	0	0	0	-0.089693957
cov2_3_4_8_9_10	36.7682	-224.472	0.3678211	0	0	0	-0.067349258
cov2_4_5_6_10_11	36.7684	-224.473	0.3756469	0	0	0	-0.11147561
cov2_3_4_7_9_10	36.8158	-224.496	0.3682456	0	0	0	-0.068102159
cov1_2_6_7_8_11	36.8184	-224.498	0.3739585	0	-0.19862156	0	-0.001145881
cov2_3_4_5_6_7	36.8533	-224.515	0.3645729	0	0	0	-0.090275823
cov2_3_4_6_9_10	36.8536	-224.515	0.3712536	0	0	0	-0.08436755
cov2_3_4_5_9_10	36.8848	-224.531	0.3712658	0	0	0	-0.070288631
cov2_3_4_5_7_8	36.8893	-224.533	0.3664744	0	0	0	-0.075702361
cov2_3_4_7_8_11	36.8933	-224.535	0.3690073	0	0	0	-0.093451081
cov2_3_6_7_8_9	36.8989	-224.538	0.362946	0	0	0	-0.044079522
cov2_4_5_6_9_10	36.9125	-224.545	0.3737408	0	0	0	-0.10308436
cov2_4_5_7_9_11	36.9159	-224.546	0.376964	0	0	0	-0.10075436
cov2_4_5_6_7_9	36.9197	-224.548	0.3738075	0	0	0	-0.10284253
cov2_4_5_6_8_9	36.926	-224.551	0.3737194	0	0	0	-0.10319608
cov2_4_5_6_7_10	36.9332	-224.555	0.3741954	0	0	0	-0.10237179
cov2_4_5_7_10_11	36.9337	-224.555	0.3772559	0	0	0	-0.10038446
cov2_4_5_8_9_11	36.9348	-224.556	0.3768507	0	0	0	-0.10080868
cov2_4_5_6_8_10	36.9396	-224.558	0.3741074	0	0	0	-0.102728
cov2_3_7_8_9_11	36.9501	-224.563	0.3652492	0	0	0	-0.03690211
cov2_4_5_8_10_11	36.9524	-224.565	0.3771406	0	0	0	-0.10043743
cov2_4_7_8_9_11	36.9541	-224.565	0.3767841	0	0	0	-0.10088889
cov1_2_3_7_8_9	36.9556	-224.566	0.3620596	0	-0.03883486	0	0.012161436
cov2_3_6_7_8_10	36.9557	-224.566	0.3633793	0	0	0	-0.043946829
cov1_2_3_7_8_10	36.9591	-224.568	0.3623858	0	-0.040070517	0	0.01265507
cov2_3_6_8_9_11	36.9648	-224.571	0.3642223	0	0	0	-0.048189807

cov2_4_5_9_10_11	36.9674	-224.572	0.3763892	0	0	0	-0.10053823
cov1_3_7_8_9_10	36.9847	-224.581	0.3625964	0	-0.028675367	0	0
cov2_4_7_8_10_11	36.9894	-224.583	0.3770676	0	0	0	-0.10081827
cov2_3_5_6_8_9	36.9979	-224.587	0.3603439	0	0	0	-0.034426407
cov2_3_7_8_10_11	36.9989	-224.588	0.3655552	0	0	0	-0.037186567
cov3_4_5_6_8_9	37.0119	-224.594	0.3577689	0	0	0	0
cov2_3_4_6_7_8	37.0188	-224.598	0.3672768	0	0	0	-0.088798898
cov2_4_6_7_8_9	37.0243	-224.6	0.3748566	0	0	0	-0.099240269
cov2_3_6_7_9_11	37.028	-224.602	0.3646442	0	0	0	-0.048294633
cov2_3_6_8_10_11	37.0287	-224.603	0.3645863	0	0	0	-0.047900401
cov3_4_5_6_8_10	37.0478	-224.612	0.3582908	0	0	0	0
cov2_4_5_7_8_9	37.052	-224.614	0.3745474	0	0	0	-0.085778423
cov2_3_5_6_8_10	37.0525	-224.615	0.3607917	0	0	0	-0.034156618
cov2_3_4_6_8_11	37.0566	-224.617	0.3685941	0	0	0	-0.092785541
cov2_4_5_7_8_10	37.0577	-224.617	0.3748402	0	0	0	-0.085535699
cov2_4_6_7_8_10	37.0581	-224.617	0.3752453	0	0	0	-0.098943485
cov2_3_5_6_7_9	37.0823	-224.629	0.3609196	0	0	0	-0.035001509
cov2_3_6_8_9_10	37.0844	-224.63	0.3626496	0	0	0	-0.042612836
cov2_3_6_7_10_11	37.0917	-224.634	0.3650164	0	0	0	-0.04799635
cov3_4_5_8_9_11	37.0986	-224.638	0.3611103	0	0	0	0
cov2_3_5_8_9_11	37.1056	-224.641	0.3644776	0	0	0	-0.03286722
cov1_2_3_8_9_10	37.1098	-224.643	0.3619533	0	-0.038773567	0	0.012301434
cov3_4_5_6_7_9	37.1114	-224.644	0.3582724	0	0	0	0
cov2_3_8_9_10_11	37.1138	-224.645	0.3649104	0	0	0	-0.036331261
cov2_3_4_6_7_11	37.1208	-224.649	0.3690668	0	0	0	-0.093653457
cov3_5_6_7_8_9	37.132	-224.654	0.3580345	0	0	0	0
cov3_4_5_8_10_11	37.1322	-224.654	0.3614768	0	0	0	0
cov2_4_7_8_9_10	37.1351	-224.656	0.3750703	0	0	0	-0.088753353
cov2_3_5_6_7_10	37.1362	-224.656	0.3613699	0	0	0	-0.034721055
cov3_4_5_6_7_10	37.1463	-224.661	0.358798	0	0	0	0
cov2_4_6_7_9_11	37.15	-224.663	0.3765448	0	0	0	-0.1038014
cov2_3_5_8_10_11	37.1532	-224.665	0.3647419	0	0	0	-0.032774934
cov2_3_6_7_9_10	37.1548	-224.666	0.3630374	0	0	0	-0.042521683
cov3_4_5_7_8_9	37.1568	-224.667	0.3611721	0	0	0	0
cov2_3_4_5_6_11	37.1597	-224.668	0.3737209	0	0	0	-0.11166243
cov2_3_5_7_9_11	37.1649	-224.671	0.3649246	0	0	0	-0.033699228
cov2_4_7_9_10_11	37.1681	-224.672	0.3769161	0	0	0	-0.10061486
cov2_4_6_8_9_11	37.1704	-224.674	0.3764201	0	0	0	-0.10388638
cov2_3_7_9_10_11	37.1709	-224.674	0.3653039	0	0	0	-0.036662152
cov1_2_3_7_9_10	37.1724	-224.675	0.3623083	0	-0.038903535	0	0.012300216
cov3_4_5_7_9_11	37.1766	-224.677	0.3614525	0	0	0	0
cov3_4_5_7_8_10	37.1782	-224.677	0.361529	0	0	0	0
cov3_5_6_7_8_10	37.1807	-224.679	0.358551	0	0	0	0
cov2_4_8_9_10_11	37.1889	-224.683	0.3767976	0	0	0	-0.1006722
cov2_4_6_7_10_11	37.1903	-224.683	0.376883	0	0	0	-0.10338666
cov3_5_6_8_9_11	37.207	-224.692	0.3585796	0	0	0	0
cov3_4_5_7_10_11	37.2099	-224.693	0.3618267	0	0	0	0

cov2_4_6_8_10_11	37.2105	-224.694	0.3767576	0	0	0	-0.10347651
cov2_3_5_7_10_11	37.2127	-224.695	0.3651948	0	0	0	-0.033592987
cov2_4_6_9_10_11	37.2144	-224.696	0.3758179	0	0	0	-0.10420848
cov3_5_7_8_9_11	37.2336	-224.705	0.3615267	0	0	0	0
cov2_4_6_7_9_10	37.2552	-224.716	0.3750501	0	0	0	-0.098004074
cov2_3_5_7_8_9	37.2557	-224.716	0.3637665	0	0	0	-0.025636658
cov2_4_5_7_9_10	37.2558	-224.716	0.3747548	0	0	0	-0.085336819
cov3_5_6_8_10_11	37.263	-224.72	0.359071	0	0	0	0
cov2_4_5_8_9_10	37.2642	-224.72	0.3746771	0	0	0	-0.085614356
cov2_4_6_8_9_10	37.2687	-224.723	0.3749734	0	0	0	-0.098285987
cov2_3_7_8_9_10	37.2771	-224.727	0.3629277	0	0	0	-0.018454418
cov3_5_7_8_10_11	37.2776	-224.727	0.3618696	0	0	0	0
cov2_3_5_6_9_11	37.281	-224.729	0.3680741	0	0	0	-0.047814535
cov2_3_5_7_8_10	37.2881	-224.732	0.3640427	0	0	0	-0.025650412
cov3_4_5_8_9_10	37.2902	-224.733	0.3610683	0	0	0	0
cov3_5_6_8_9_10	37.2959	-224.736	0.3579932	0	0	0	0
cov3_5_6_7_9_11	37.298	-224.737	0.3591034	0	0	0	0
cov2_3_6_9_10_11	37.3032	-224.74	0.3687627	0	0	0	-0.051154505
cov2_3_5_6_10_11	37.3395	-224.758	0.3684804	0	0	0	-0.047233778
cov1_7_8_9_10_11	37.3397	-224.758	0.3753337	0	-0.077573941	0	0
cov1_2_7_8_10_11	37.3424	-224.759	0.3752583	0	-0.08255186	0	0.005682257
cov3_5_6_7_10_11	37.3531	-224.765	0.3595971	0	0	0	0
cov3_4_5_7_9_10	37.3659	-224.771	0.3614132	0	0	0	0
cov1_2_7_8_9_11	37.3683	-224.772	0.3749497	0	-0.081251093	0	0.004780593
cov3_5_8_9_10_11	37.3798	-224.778	0.361337	0	0	0	0
cov3_5_6_7_9_10	37.3894	-224.783	0.3585156	0	0	0	0
cov2_3_5_8_9_10	37.4112	-224.794	0.3634568	0	0	0	-0.02476939
cov2_3_5_9_10_11	37.4209	-224.799	0.3693196	0	0	0	-0.041889323
cov3_5_7_8_9_10	37.4303	-224.803	0.3614183	0	0	0	0
cov3_5_7_9_10_11	37.4524	-224.815	0.3616972	0	0	0	0
cov2_3_5_7_9_10	37.4718	-224.824	0.3638963	0	0	0	-0.025550732
cov3_4_7_8_9_11	37.497	-224.837	0.3622264	0	0	0	0
cov2_3_5_6_9_10	37.4985	-224.838	0.3670323	0	0	0	-0.042772733
cov3_6_7_8_9_11	37.4988	-224.838	0.3624295	0	0	0	0
cov3_7_8_9_10_11	37.5002	-224.838	0.362321	0	0	0	0
cov3_6_7_8_9_10	37.5117	-224.844	0.3620295	0	0	0	0
cov3_4_6_7_8_9	37.5118	-224.844	0.3618881	0	0	0	0
cov3_4_7_8_9_10	37.514	-224.845	0.3621388	0	0	0	0
cov3_4_7_8_10_11	37.5538	-224.865	0.3625694	0	0	0	0
cov3_6_7_8_10_11	37.554	-224.865	0.3628404	0	0	0	0
cov3_4_6_7_8_10	37.5648	-224.871	0.3623684	0	0	0	0
cov1_2_7_9_10_11	37.5756	-224.876	0.3752166	0	-0.081271754	0	0.005344465
cov2_3_6_7_8_11	37.6191	-224.898	0.3630438	0	0	0	-0.051823859
cov1_2_8_9_10_11	37.6358	-224.906	0.3750605	0	-0.080512008	0	0.005349043
cov3_4_6_8_9_11	37.6437	-224.91	0.3621185	0	0	0	0
cov3_6_8_9_10_11	37.6443	-224.91	0.3622514	0	0	0	0
cov3_4_8_9_10_11	37.6444	-224.911	0.3619987	0	0	0	0

cov2_4_5_7_8_11	37.6552	-224.916	0.3783004	0	0	0	-0.1259322
cov3_4_6_8_9_10	37.6594	-224.918	0.3617544	0	0	0	0
cov2_3_5_7_8_11	37.6603	-224.918	0.3635742	0	0	0	-0.045729327
cov3_4_5_6_9_11	37.6692	-224.923	0.3658655	0	0	0	0
cov3_4_5_9_10_11	37.6873	-224.932	0.3667837	0	0	0	0
cov3_4_5_6_10_11	37.6959	-224.936	0.3663415	0	0	0	0
cov3_4_6_8_10_11	37.6992	-224.938	0.3625514	0	0	0	0
cov3_4_6_7_9_11	37.7093	-224.943	0.3625235	0	0	0	0
cov3_6_7_9_10_11	37.7094	-224.943	0.3626368	0	0	0	0
cov3_4_7_9_10_11	37.711	-224.944	0.3623509	0	0	0	0
cov3_4_6_7_9_10	37.7269	-224.952	0.3621459	0	0	0	0
cov2_3_5_6_7_8	37.7521	-224.964	0.3610666	0	0	0	-0.045032649
cov3_4_6_7_10_11	37.7643	-224.97	0.3629631	0	0	0	0
cov2_3_5_6_8_11	37.7684	-224.973	0.3620566	0	0	0	-0.048061441
cov3_4_5_6_9_10	37.7733	-224.975	0.3654122	0	0	0	0
cov2_4_5_6_7_8	37.8184	-224.998	0.3762354	0	0	0	-0.12108497
cov2_4_6_7_8_11	37.8298	-225.003	0.3782786	0	0	0	-0.12222437
cov3_5_6_9_10_11	37.856	-225.016	0.3657888	0	0	0	0
cov2_3_5_6_7_11	37.8634	-225.02	0.3626256	0	0	0	-0.048702984
cov2_4_5_6_7_11	37.8896	-225.033	0.3783085	0	0	0	-0.12787337
cov2_4_5_6_8_11	37.896	-225.036	0.3781777	0	0	0	-0.12796142
cov3_4_5_7_8_11	37.9925	-225.085	0.358615	0	0	0	0
cov3_4_5_6_7_8	38.0492	-225.113	0.3578876	0	0	0	0
cov3_4_6_9_10_11	38.0711	-225.124	0.3673214	0	0	0	0
cov3_4_5_6_8_11	38.1155	-225.146	0.3583043	0	0	0	0
cov3_5_6_7_8_11	38.2211	-225.199	0.3585108	0	0	0	0
cov2_6_7_8_9_11	38.2235	-225.2	0.3733373	0	0	0	-0.053273347
cov3_4_5_6_7_11	38.2292	-225.203	0.3587908	0	0	0	0
cov2_5_7_8_9_11	38.234	-225.205	0.3747312	0	0	0	-0.054144037
cov2_5_6_7_8_9	38.2442	-225.21	0.3727496	0	0	0	-0.054651395
cov2_6_7_8_10_11	38.2553	-225.216	0.3737858	0	0	0	-0.052664652
cov2_5_7_8_10_11	38.2686	-225.223	0.3750572	0	0	0	-0.053498986
cov2_5_6_7_8_10	38.2768	-225.227	0.373179	0	0	0	-0.05391737
cov2_6_7_8_9_10	38.299	-225.238	0.3720091	0	0	0	-0.047948205
cov1_2_7_8_9_10	38.3051	-225.241	0.3710675	0	-0.042222141	0	0.011214014
cov2_5_7_8_9_10	38.3541	-225.265	0.3735112	0	0	0	-0.047050097
cov2_7_8_9_10_11	38.3923	-225.284	0.3743462	0	0	0	-0.039581406
cov3_4_6_7_8_11	38.3983	-225.287	0.3612091	0	0	0	0
cov2_5_6_7_9_11	38.4115	-225.294	0.3741757	0	0	0	-0.057456287
cov2_5_7_9_10_11	38.4474	-225.312	0.3749067	0	0	0	-0.05354962
cov2_5_6_8_9_11	38.4492	-225.313	0.3740273	0	0	0	-0.057216419
cov2_5_6_7_10_11	38.4508	-225.314	0.3745577	0	0	0	-0.056588982
cov2_6_7_9_10_11	38.4607	-225.319	0.3736391	0	0	0	-0.051617278
cov2_5_6_7_9_10	38.4779	-225.327	0.3730448	0	0	0	-0.053287839
cov2_5_8_9_10_11	38.4852	-225.331	0.3747648	0	0	0	-0.053317459
cov2_5_6_8_10_11	38.4883	-225.332	0.3744091	0	0	0	-0.056353298
cov2_6_8_9_10_11	38.5005	-225.339	0.3734771	0	0	0	-0.051289379

cov2_5_6_8_9_10	38.5094	-225.343	0.3729446	0	0	0	-0.053210764
cov2_5_6_9_10_11	38.598	-225.387	0.3726787	0	0	0	-0.0551368
cov4_5_7_8_9_10	38.6797	-225.428	0.370814	0	0	0	0
cov4_5_7_8_10_11	38.7015	-225.439	0.3712751	0	0	0	0
cov4_5_6_7_8_10	38.706	-225.441	0.3708745	0	0	0	0
cov4_5_7_8_9_11	38.7119	-225.444	0.3708306	0	0	0	0
cov4_5_6_7_8_9	38.7182	-225.447	0.3704061	0	0	0	0
cov4_5_7_9_10_11	38.8882	-225.532	0.3711614	0	0	0	0
cov4_5_6_7_9_10	38.8966	-225.537	0.3708125	0	0	0	0
cov4_6_7_8_9_10	38.9008	-225.539	0.3717231	0	0	0	0
cov4_5_8_9_10_11	38.9095	-225.543	0.3710349	0	0	0	0
cov4_5_6_8_9_10	38.916	-225.546	0.3707148	0	0	0	0
cov4_5_6_7_10_11	38.9213	-225.549	0.3713906	0	0	0	0
cov4_6_7_8_9_11	38.926	-225.551	0.3715413	0	0	0	0
cov4_5_6_7_9_11	38.9314	-225.554	0.3709561	0	0	0	0
cov4_6_7_8_10_11	38.9389	-225.558	0.3719903	0	0	0	0
cov4_5_6_8_10_11	38.9423	-225.559	0.3712553	0	0	0	0
cov4_7_8_9_10_11	38.9489	-225.563	0.3709721	0	0	0	0
cov4_5_6_8_9_11	38.9526	-225.565	0.370821	0	0	0	0
cov4_5_6_9_10_11	38.9611	-225.569	0.3702495	0	0	0	0
cov5_7_8_9_10_11	39.0105	-225.594	0.3714563	0	0	0	0
cov5_6_7_8_9_10	39.0161	-225.596	0.3710397	0	0	0	0
cov6_7_8_9_10_11	39.0205	-225.599	0.3715135	0	0	0	0
cov5_6_7_8_9_11	39.0426	-225.61	0.3711681	0	0	0	0
cov5_6_7_8_10_11	39.0511	-225.614	0.3716202	0	0	0	0
cov4_6_7_9_10_11	39.1016	-225.639	0.3719141	0	0	0	0
cov4_6_8_9_10_11	39.1252	-225.651	0.3717869	0	0	0	0
cov5_6_7_9_10_11	39.2315	-225.704	0.3715587	0	0	0	0
cov5_6_8_9_10_11	39.2626	-225.72	0.3714539	0	0	0	0
cov2_5_6_7_8_11	39.6149	-225.896	0.3766198	0	0	0	-0.07245788
cov1_5_6_7_8_9_11	39.8195	-222.488	0.3758277	0	-0.30481331	0	0
cov1_5_6_7_8_10_11	39.8311	-222.494	0.3759862	0	-0.3052169	0	0
cov1_3_5_6_7_8_11	39.9113	-222.534	0.3734016	0	-0.30373236	0	0
cov1_4_5_6_7_8_11	39.9425	-222.549	0.376404	0	-0.32049273	0	0
cov1_2_5_6_7_8_11	39.988	-222.572	0.3766846	0	-0.31859633	0	-0.006412163
cov1_3_4_5_6_9_11	40.1151	-222.636	0.3706293	0	-0.26240493	0	0
cov1_3_4_5_6_10_11	40.1299	-222.643	0.3708217	0	-0.26304718	0	0
cov1_3_5_6_7_9_11	40.1501	-222.653	0.3730771	0	-0.28107272	0	0
cov1_4_5_6_7_9_11	40.1545	-222.655	0.3759078	0	-0.29532034	0	0
cov1_4_5_6_7_10_11	40.1637	-222.66	0.3760645	0	-0.29547698	0	0
cov1_3_5_6_7_10_11	40.1653	-222.661	0.3733024	0	-0.28207633	0	0
cov1_3_5_6_8_9_11	40.1895	-222.673	0.3720982	0	-0.27490904	0	0
cov1_2_3_4_5_6_11	40.201	-222.679	0.3725508	0	-0.2615199	0	-0.027096175
cov1_3_5_6_8_10_11	40.2055	-222.681	0.3723239	0	-0.27595276	0	0
cov1_2_3_5_6_9_11	40.2161	-222.686	0.3704476	0	-0.26324106	0	-0.00220125
cov1_3_5_6_9_10_11	40.2169	-222.687	0.3703488	0	-0.2648413	0	0
cov1_2_5_6_7_9_11	40.2255	-222.691	0.3762133	0	-0.29625775	0	-0.004753962

cov1_5_6_7_9_10_11	40.2305	-222.693	0.37606	0	-0.29937943	0	0
cov1_2_3_5_6_10_11	40.2337	-222.695	0.3706216	0	-0.26432784	0	-0.00193273
cov1_4_5_6_8_9_11	40.2357	-222.696	0.3756982	0	-0.29342426	0	0
cov1_2_5_6_7_10_11	40.2379	-222.697	0.376362	0	-0.29693384	0	-0.004418796
cov1_4_5_6_8_10_11	40.2447	-222.701	0.3758718	0	-0.29357311	0	0
cov1_2_4_5_6_7_11	40.2451	-222.701	0.3774767	0	-0.29164792	0	-0.028336722
cov1_3_4_5_6_7_11	40.2595	-222.708	0.37342	0	-0.29483411	0	0
cov1_2_3_4_6_9_11	40.2784	-222.717	0.3713917	0	-0.19787356	0	-0.043247697
cov1_3_4_5_6_8_11	40.2879	-222.722	0.3724489	0	-0.28846299	0	0
cov1_2_3_4_6_10_11	40.2949	-222.726	0.3716467	0	-0.19872118	0	-0.042828341
cov1_2_5_6_8_9_11	40.3145	-222.735	0.3760141	0	-0.29452742	0	-0.00471845
cov1_2_3_5_6_7_11	40.3163	-222.736	0.3737944	0	-0.29506159	0	-0.004898295
cov1_2_4_5_6_8_11	40.3188	-222.738	0.3773102	0	-0.2890297	0	-0.029243518
cov1_5_6_8_9_10_11	40.3194	-222.738	0.3758627	0	-0.29762643	0	0
cov1_2_5_6_8_10_11	40.3268	-222.742	0.3761629	0	-0.29520291	0	-0.004384698
cov1_2_3_5_6_8_11	40.3532	-222.755	0.3727978	0	-0.28902072	0	-0.004474095
cov1_2_4_5_6_9_11	40.4636	-222.81	0.3748251	0	-0.2506266	0	-0.036808534
cov1_2_4_5_6_10_11	40.4739	-222.815	0.3749871	0	-0.25113479	0	-0.036507712
cov1_3_4_6_8_9_11	40.5214	-222.839	0.3672241	0	-0.21111883	0	0
cov1_3_4_6_8_10_11	40.5352	-222.846	0.3675858	0	-0.211975	0	0
cov1_3_4_6_7_9_11	40.5494	-222.853	0.367838	0	-0.21265918	0	0
cov1_3_4_6_7_10_11	40.5618	-222.859	0.3682121	0	-0.2135371	0	0
cov1_3_4_6_9_10_11	40.5626	-222.86	0.3690681	0	-0.21595096	0	0
cov1_4_5_6_7_8_9	40.575	-222.866	0.3718587	0	-0.26268612	0	0
cov1_4_5_6_7_8_10	40.5768	-222.867	0.3720376	0	-0.26261482	0	0
cov1_3_5_6_7_8_9	40.5842	-222.87	0.3689176	0	-0.24815612	0	0
cov1_3_5_6_7_8_10	40.5937	-222.875	0.3691696	0	-0.24899475	0	0
cov1_2_4_6_7_9_11	40.5984	-222.877	0.3761829	0	-0.20392666	0	-0.05530237
cov1_2_4_6_7_10_11	40.602	-222.879	0.3764218	0	-0.20481161	0	-0.054448687
cov1_4_5_6_9_10_11	40.649	-222.903	0.3734631	0	-0.2791838	0	0
cov1_2_4_6_8_9_11	40.6523	-222.904	0.3760272	0	-0.20277557	0	-0.055671662
cov1_2_4_6_8_10_11	40.656	-222.906	0.3762652	0	-0.20365349	0	-0.054826933
cov1_2_3_4_6_8_11	40.6563	-222.906	0.3699829	0	-0.19981695	0	-0.048618509
cov1_5_6_7_8_9_10	40.6662	-222.911	0.3719927	0	-0.26651729	0	0
cov1_2_5_6_7_8_9	40.6684	-222.912	0.3719268	0	-0.26721639	0	0.000519482
cov1_3_4_6_7_8_11	40.6734	-222.915	0.3669068	0	-0.22607325	0	0
cov1_2_5_6_7_8_10	40.6736	-222.915	0.3720913	0	-0.26767837	0	0.000915951
cov1_4_6_7_8_10_11	40.6783	-222.917	0.3739531	0	-0.23478232	0	0
cov1_4_6_7_8_9_11	40.693	-222.925	0.3736484	0	-0.23436682	0	0
cov1_2_3_4_6_7_11	40.6944	-222.925	0.3706464	0	-0.20102433	0	-0.04973775
cov1_3_4_5_6_7_8	40.71	-222.933	0.3692944	0	-0.26267272	0	0
cov1_2_4_5_6_7_8	40.7469	-222.952	0.3731196	0	-0.26485351	0	-0.021355715
cov4_5_6_7_8_11	40.7483	-226.462	0.3735684	0	0	0	0
cov1_2_3_5_6_7_8	40.7867	-222.972	0.3693813	0	-0.26627196	0	0.000358432
cov1_2_4_6_7_8_11	40.8077	-222.982	0.3773729	0	-0.21692437	0	-0.067603999
cov1_3_6_7_8_9_11	40.8195	-222.988	0.3642565	0	-0.18288886	0	0
cov1_2_5_6_9_10_11	40.8314	-222.994	0.3734444	0	-0.28112408	0	-0.004291817

cov1_2_4_6_9_10_11	40.833	-222.995	0.3748792	0	-0.19745935	0	-0.057587459
cov1_2_3_4_5_6_9	40.8346	-222.995	0.369352	0	-0.22021938	0	-0.019815595
cov1_2_3_4_5_6_10	40.8427	-223	0.3695473	0	-0.22076894	0	-0.019594378
cov1_3_6_7_8_10_11	40.8461	-223.001	0.3646461	0	-0.18355308	0	0
cov1_3_4_5_6_8_9	40.8852	-223.021	0.3680603	0	-0.23274831	0	0
cov1_3_4_5_6_9_10	40.8856	-223.021	0.368587	0	-0.23519507	0	0
cov1_3_4_5_6_7_9	40.886	-223.021	0.3689234	0	-0.23779536	0	0
cov1_3_4_5_6_7_10	40.8925	-223.024	0.3691869	0	-0.23843129	0	0
cov1_3_4_5_6_8_10	40.8927	-223.025	0.3683222	0	-0.23341366	0	0
cov1_3_4_6_7_8_9	40.9074	-223.032	0.3641788	0	-0.1851068	0	0
cov1_3_4_6_7_8_10	40.9094	-223.033	0.3645977	0	-0.18616778	0	0
cov1_2_4_5_6_7_9	40.9152	-223.036	0.3726415	0	-0.23876829	0	-0.021549632
cov1_2_4_5_6_7_10	40.9188	-223.038	0.3728183	0	-0.23904064	0	-0.021241245
cov1_2_4_5_6_8_9	40.958	-223.057	0.3725439	0	-0.23706192	0	-0.022519944
cov1_2_4_5_6_8_10	40.9616	-223.059	0.3727201	0	-0.23733882	0	-0.022212378
cov1_4_5_6_7_9_10	40.9738	-223.065	0.3721551	0	-0.25693513	0	0
cov1_2_3_5_6_7_9	40.9875	-223.072	0.3690222	0	-0.24397218	0	0.001980805
cov1_3_5_6_7_9_10	40.9875	-223.072	0.3691519	0	-0.24258744	0	0
cov1_2_3_5_6_9_10	40.9942	-223.075	0.3681963	0	-0.23924825	0	0.002351954
cov1_2_3_5_6_8_9	40.9948	-223.076	0.3681395	0	-0.23917758	0	0.002268569
cov1_3_5_6_8_9_10	40.9955	-223.076	0.3682707	0	-0.23760903	0	0
cov1_2_3_5_6_7_10	40.9968	-223.077	0.3692637	0	-0.24502453	0	0.002316779
cov1_2_3_5_6_8_10	41.0052	-223.081	0.3683793	0	-0.24025477	0	0.002595335
cov1_4_5_6_8_9_10	41.0223	-223.089	0.3720295	0	-0.25602758	0	0
cov1_2_4_6_7_8_10	41.0391	-223.098	0.3721225	0	-0.17849281	0	-0.047134489
cov1_2_4_6_7_8_9	41.049	-223.103	0.3718536	0	-0.17736571	0	-0.048120299
cov1_2_3_4_5_6_8	41.0531	-223.105	0.3693249	0	-0.23564236	0	-0.020279139
cov1_2_3_4_5_6_7	41.0597	-223.108	0.3701642	0	-0.24110794	0	-0.019851749
cov1_2_3_4_6_8_9	41.0663	-223.111	0.3661053	0	-0.16435468	0	-0.034749288
cov1_2_5_6_7_9_10	41.073	-223.115	0.3722104	0	-0.26181972	0	0.000697535
cov1_2_3_4_6_8_10	41.0748	-223.116	0.3664536	0	-0.16549553	0	-0.034375636
cov1_2_4_5_6_9_10	41.0752	-223.116	0.371947	0	-0.22851257	0	-0.027997657
cov1_4_6_7_9_10_11	41.0764	-223.116	0.3740161	0	-0.22893017	0	0
cov1_2_3_4_6_7_9	41.1103	-223.133	0.3666949	0	-0.16485228	0	-0.035548959
cov1_2_3_4_6_7_10	41.1172	-223.137	0.3670541	0	-0.16603077	0	-0.035150287
cov1_2_5_6_8_9_10	41.1294	-223.143	0.372088	0	-0.26097689	0	0.000627742
cov1_4_6_8_9_10_11	41.1374	-223.147	0.3738395	0	-0.22788471	0	0
cov1_2_3_4_6_9_10	41.1641	-223.16	0.3688133	0	-0.16822997	0	-0.037743609
cov1_2_3_6_8_9_11	41.178	-223.167	0.3637159	0	-0.1789399	0	0.003489792
cov1_3_6_8_9_10_11	41.178	-223.167	0.3639434	0	-0.17640927	0	0
cov1_2_3_4_6_7_8	41.1842	-223.17	0.3661704	0	-0.17390469	0	-0.042665019
cov1_2_3_6_8_10_11	41.2049	-223.181	0.3640754	0	-0.1800726	0	0.00414221
cov1_3_6_7_9_10_11	41.2278	-223.192	0.3645252	0	-0.17718969	0	0
cov1_2_3_6_7_9_11	41.2287	-223.193	0.3642834	0	-0.17963193	0	0.00339999
cov1_3_4_6_8_9_10	41.2437	-223.2	0.3640203	0	-0.17932646	0	0
cov1_2_3_6_7_10_11	41.2544	-223.205	0.3646553	0	-0.18080124	0	0.004071941
cov1_3_4_6_7_9_10	41.2948	-223.226	0.3645751	0	-0.18013224	0	0

cov1_2_3_6_9_10_11	41.306	-223.231	0.3666044	0	-0.18425817	0	0.003329589
cov1_4_6_7_8_9_10	41.3997	-223.278	0.370299	0	-0.20162287	0	0
cov1_2_4_6_7_9_10	41.4283	-223.292	0.3722195	0	-0.17247898	0	-0.047679939
cov1_2_4_6_8_9_10	41.4526	-223.305	0.372134	0	-0.17210181	0	-0.048152847
cov1_2_3_6_7_8_11	41.565	-223.361	0.3626378	0	-0.18813341	0	0.002053916
cov1_2_3_6_7_8_9	41.5811	-223.369	0.3603614	0	-0.15559528	0	0.008153482
cov1_3_6_7_8_9_10	41.5884	-223.372	0.3607806	0	-0.1493534	0	0
cov1_2_3_6_7_8_10	41.5935	-223.375	0.3607915	0	-0.15704803	0	0.00882252
cov1_2_4_5_7_8_11	41.6398	-223.398	0.3793302	0	-0.19320905	0	-0.028920976
cov1_3_4_5_7_8_11	41.6606	-223.409	0.3753029	0	-0.19697883	0	0
cov1_4_5_7_8_9_11	41.7015	-223.429	0.3781412	0	-0.20443841	0	0
cov1_4_5_7_8_10_11	41.7046	-223.431	0.3782232	0	-0.20471195	0	0
cov1_3_5_7_8_9_11	41.7377	-223.447	0.3754787	0	-0.19001832	0	0
cov1_3_5_7_8_10_11	41.7434	-223.45	0.3755816	0	-0.19098846	0	0
cov1_2_3_5_7_8_11	41.7711	-223.464	0.3757432	0	-0.19870295	0	-0.001659666
cov1_2_3_4_5_9_11	41.7825	-223.469	0.3743856	0	-0.15248329	0	-0.027820854
cov1_2_3_4_5_10_11	41.7893	-223.473	0.3744859	0	-0.15342653	0	-0.02771412
cov1_2_3_4_5_7_11	41.8031	-223.48	0.376412	0	-0.17590531	0	-0.027343097
cov1_2_5_7_8_9_11	41.818	-223.487	0.3785533	0	-0.20676631	0	-0.002350163
cov1_5_7_8_9_10_11	41.8192	-223.488	0.3784516	0	-0.20853661	0	0
cov1_2_3_4_5_8_11	41.8218	-223.489	0.3756714	0	-0.17096183	0	-0.027603949
cov1_2_5_7_8_10_11	41.8224	-223.489	0.3786256	0	-0.20743619	0	-0.002228522
cov1_2_4_5_7_9_11	41.8279	-223.492	0.3790834	0	-0.18013273	0	-0.029057543
cov1_2_4_5_7_10_11	41.8323	-223.494	0.37917	0	-0.18068559	0	-0.028906867
cov1_3_4_5_7_9_11	41.8517	-223.504	0.3750908	0	-0.1845829	0	0
cov1_3_4_5_7_10_11	41.8569	-223.507	0.3752129	0	-0.18534051	0	0
cov1_3_4_5_8_9_11	41.8731	-223.515	0.3743334	0	-0.17993601	0	0
cov1_3_4_5_8_10_11	41.8786	-223.518	0.3744547	0	-0.18073039	0	0
cov1_2_4_5_8_9_11	41.8827	-223.52	0.378943	0	-0.1784914	0	-0.029771456
cov1_3_4_5_9_10_11	41.8857	-223.521	0.3731429	0	-0.1729902	0	0
cov1_2_4_5_8_10_11	41.8872	-223.522	0.3790284	0	-0.17903786	0	-0.029621648
cov1_2_3_6_8_9_10	41.9057	-223.531	0.3602207	0	-0.15043536	0	0.008463867
cov1_4_5_7_9_10_11	41.9409	-223.549	0.378229	0	-0.20384903	0	0
cov1_3_5_7_9_10_11	41.9759	-223.566	0.3754742	0	-0.1894817	0	0
cov1_2_3_5_7_9_11	41.9765	-223.566	0.375571	0	-0.18871729	0	-0.00096639
cov1_2_3_6_7_9_10	41.9795	-223.568	0.360723	0	-0.15047472	0	0.008478954
cov1_2_3_5_7_10_11	41.9826	-223.57	0.375675	0	-0.18976753	0	-0.000878419
cov1_4_5_8_9_10_11	42.0014	-223.579	0.3780642	0	-0.20276745	0	0
cov1_3_5_8_9_10_11	42.0051	-223.581	0.3747074	0	-0.18490877	0	0
cov1_2_3_5_8_9_11	42.006	-223.581	0.3747944	0	-0.18432167	0	-0.000650115
cov1_2_3_5_8_10_11	42.0124	-223.584	0.3748974	0	-0.185408	0	-0.000568433
cov1_2_3_4_7_8_11	42.0131	-223.585	0.3726935	0	-0.1107233	0	-0.048567392
cov1_6_7_8_9_10_11	42.0289	-223.593	0.3725854	0	-0.1904696	0	0
cov1_2_3_5_9_10_11	42.0331	-223.595	0.3729708	0	-0.17466003	0	-7.27E-05
cov1_2_6_7_8_10_11	42.0476	-223.602	0.3726826	0	-0.19297503	0	0.002767082
cov1_2_3_4_8_9_11	42.0495	-223.603	0.372464	0	-0.1014093	0	-0.043310353
cov1_2_3_4_8_10_11	42.0588	-223.608	0.3726586	0	-0.10222935	0	-0.043137329

cov1_2_5_7_9_10_11	42.0588	-223.608	0.3786275	0	-0.20632209	0	-0.002485094
cov1_2_3_4_7_9_11	42.0655	-223.611	0.3729756	0	-0.10234088	0	-0.044028207
cov1_2_6_7_8_9_11	42.0672	-223.612	0.372309	0	-0.19150748	0	0.001699916
cov1_2_3_4_7_10_11	42.0744	-223.615	0.3731782	0	-0.10316868	0	-0.043835936
cov1_2_3_4_9_10_11	42.0752	-223.616	0.3738971	0	-0.10405537	0	-0.04511069
cov1_3_4_7_8_9_11	42.1261	-223.641	0.3698062	0	-0.11886621	0	0
cov1_2_5_8_9_10_11	42.1267	-223.642	0.3784675	0	-0.20535865	0	-0.00246966
cov1_3_4_7_8_10_11	42.1313	-223.644	0.3700625	0	-0.11959525	0	0
cov1_2_4_7_8_10_11	42.1715	-223.664	0.3785002	0	-0.11034499	0	-0.056319968
cov1_2_4_7_8_9_11	42.1726	-223.665	0.3783343	0	-0.10966827	0	-0.056966487
cov1_2_4_5_9_10_11	42.1949	-223.676	0.3772692	0	-0.16097456	0	-0.037397776
cov1_3_4_8_9_10_11	42.3324	-223.744	0.3694973	0	-0.11728805	0	0
cov1_3_4_7_9_10_11	42.3573	-223.757	0.3699658	0	-0.118461	0	0
cov1_2_4_7_9_10_11	42.3999	-223.778	0.3784891	0	-0.10929535	0	-0.056668649
cov1_2_4_8_9_10_11	42.4384	-223.797	0.3783647	0	-0.10873047	0	-0.056958184
cov1_2_6_7_9_10_11	42.4555	-223.806	0.3727141	0	-0.18639788	0	0.002416125
cov1_2_6_8_9_10_11	42.5368	-223.847	0.3725162	0	-0.18489935	0	0.002438072
cov1_3_4_5_7_8_9	42.6724	-223.914	0.3708926	0	-0.13524685	0	0
cov1_3_4_5_7_8_10	42.6735	-223.915	0.3710266	0	-0.13559684	0	0
cov1_2_3_4_5_7_8	42.6814	-223.919	0.3718406	0	-0.13232971	0	-0.019730539
cov1_4_7_8_9_10_11	42.6856	-223.921	0.3760748	0	-0.13445781	0	0
cov1_2_4_5_7_8_10	42.7047	-223.931	0.3747514	0	-0.13648999	0	-0.021389903
cov1_2_4_5_7_8_9	42.7052	-223.931	0.3746615	0	-0.13642942	0	-0.021554979
cov1_2_6_7_8_9_10	42.7609	-223.959	0.3687114	0	-0.16308249	0	0.007228816
cov1_4_5_7_8_9_10	42.7656	-223.961	0.3741641	0	-0.15465051	0	0
cov1_2_3_5_7_8_9	42.8243	-223.99	0.371057	0	-0.14344887	0	0.005340561
cov1_2_3_5_7_8_10	42.8268	-223.992	0.3711716	0	-0.14409104	0	0.005491625
cov1_3_5_7_8_9_10	42.8307	-223.994	0.3712998	0	-0.13969075	0	0
cov1_2_3_4_5_8_9	42.835	-223.996	0.3710163	0	-0.11490771	0	-0.020279563
cov1_2_3_4_5_8_10	42.8373	-223.997	0.3711515	0	-0.11549539	0	-0.020147367
cov1_2_3_4_5_7_9	42.843	-224	0.3716445	0	-0.11872782	0	-0.019963426
cov1_2_3_4_5_9_10	42.8432	-224	0.3719286	0	-0.12020568	0	-0.019786484
cov1_2_3_4_5_7_10	42.845	-224.001	0.3717812	0	-0.11928073	0	-0.019828761
cov1_3_4_5_8_9_10	42.8896	-224.023	0.3703583	0	-0.13114479	0	0
cov1_3_4_5_7_9_10	42.8957	-224.026	0.3710094	0	-0.13472686	0	0
cov1_2_3_4_7_8_9	42.9147	-224.036	0.3684957	0	-0.0601639	0	-0.036034405
cov1_2_3_4_7_8_10	42.9152	-224.036	0.3687323	0	-0.061021949	0	-0.035742874
cov1_2_5_7_8_9_10	42.9155	-224.036	0.3743031	0	-0.16156362	0	0.003944571
cov1_2_4_5_7_9_10	42.9277	-224.042	0.3748347	0	-0.13572584	0	-0.021792112
cov1_2_4_5_8_9_10	42.954	-224.055	0.3747344	0	-0.13476106	0	-0.02252802
cov2_3_4_5_6_8_9	42.9564	-224.056	0.3634307	0	0	0	-0.078946715
cov1_2_3_7_8_9_11	42.9765	-224.066	0.3661073	0	-0.079076957	0	0.006457231
cov1_3_7_8_9_10_11	42.9846	-224.071	0.3664288	0	-0.074010854	0	0
cov1_2_3_7_8_10_11	42.9915	-224.074	0.3663815	0	-0.08016081	0	0.006944023
cov2_3_4_5_6_8_10	42.9922	-224.074	0.3638839	0	0	0	-0.07899611
cov2_3_4_5_6_7_9	43.0421	-224.099	0.364	0	0	0	-0.079524845
cov1_2_3_5_7_9_10	43.05	-224.103	0.3711501	0	-0.14299005	0	0.005279062

cov1_2_3_5_8_9_10	43.051	-224.104	0.3704835	0	-0.13957463	0	0.005479065
cov2_3_4_5_6_7_10	43.077	-224.117	0.364457	0	0	0	-0.079566865
cov1_2_3_4_8_9_10	43.0916	-224.124	0.3682722	0	-0.059746575	0	-0.035441865
cov2_3_4_5_6_9_11	43.0946	-224.126	0.3714178	0	0	0	-0.096909713
cov1_3_4_7_8_9_10	43.1071	-224.132	0.3663008	0	-0.075373689	0	0
cov2_3_4_5_8_9_11	43.1206	-224.139	0.3684106	0	0	0	-0.075792188
cov1_2_3_4_7_9_10	43.13	-224.143	0.3687074	0	-0.060156451	0	-0.03600725
cov2_3_4_5_6_10_11	43.1343	-224.145	0.3718254	0	0	0	-0.096649625
cov2_3_4_5_8_10_11	43.1513	-224.154	0.3686878	0	0	0	-0.075978305
cov1_2_3_8_9_10_11	43.1663	-224.161	0.3658188	0	-0.078066746	0	0.006578704
cov2_3_4_5_7_9_11	43.1742	-224.165	0.3688605	0	0	0	-0.076758663
cov2_3_4_5_7_10_11	43.2049	-224.181	0.3691447	0	0	0	-0.076934162
cov1_2_3_7_9_10_11	43.2102	-224.183	0.3662449	0	-0.078638573	0	0.006523287
cov1_2_4_7_8_9_10	43.2541	-224.205	0.374109	0	-0.067168903	0	-0.048613094
cov2_3_4_5_9_10_11	43.3784	-224.267	0.37281	0	0	0	-0.085067508
cov2_3_4_7_8_9_11	43.3921	-224.274	0.3698255	0	0	0	-0.080325709
cov2_3_4_7_8_10_11	43.4376	-224.297	0.3700952	0	0	0	-0.080783513
cov2_3_4_5_6_9_10	43.4432	-224.3	0.3699067	0	0	0	-0.087471418
cov2_3_4_6_7_8_9	43.4486	-224.302	0.3675496	0	0	0	-0.079367495
cov2_3_4_5_7_8_9	43.4579	-224.307	0.3669759	0	0	0	-0.061816896
cov2_3_4_5_7_8_10	43.4743	-224.315	0.3672566	0	0	0	-0.06204851
cov2_3_4_6_7_8_10	43.4967	-224.327	0.3679508	0	0	0	-0.079594307
cov2_3_4_5_7_8_11	43.5062	-224.331	0.3680528	0	0	0	-0.090384372
cov2_3_4_5_6_8_11	43.5089	-224.333	0.365871	0	0	0	-0.098281596
cov2_3_4_6_8_9_11	43.5184	-224.337	0.3687693	0	0	0	-0.083266156
cov2_3_4_6_7_9_11	43.5641	-224.36	0.3692625	0	0	0	-0.08406247
cov2_3_4_8_9_10_11	43.5646	-224.361	0.3694014	0	0	0	-0.079456687
cov2_3_4_6_8_10_11	43.5722	-224.364	0.3691169	0	0	0	-0.083420206
cov2_3_4_5_6_7_8	43.5992	-224.378	0.3642968	0	0	0	-0.091314411
cov2_3_4_5_6_7_11	43.6032	-224.38	0.3664397	0	0	0	-0.09898344
cov2_3_4_7_9_10_11	43.6066	-224.382	0.369857	0	0	0	-0.080383878
cov2_3_4_5_8_9_10	43.6135	-224.385	0.3667112	0	0	0	-0.060882781
cov2_3_4_6_7_10_11	43.6178	-224.387	0.3696159	0	0	0	-0.084199868
cov2_3_4_7_8_9_10	43.6239	-224.39	0.3681543	0	0	0	-0.068043689
cov2_3_4_6_8_9_10	43.6345	-224.395	0.3672121	0	0	0	-0.077913845
cov2_3_4_5_7_9_10	43.6694	-224.413	0.3671676	0	0	0	-0.061776219
cov2_4_5_6_7_8_9	43.6721	-224.414	0.3736354	0	0	0	-0.10403483
cov2_4_5_6_7_8_10	43.6858	-224.421	0.3740255	0	0	0	-0.10356004
cov2_3_4_6_7_9_10	43.6873	-224.422	0.3676733	0	0	0	-0.078530012
cov2_3_4_6_9_10_11	43.702	-224.429	0.3725233	0	0	0	-0.090052834
cov2_4_5_7_8_9_11	43.7164	-224.436	0.3768821	0	0	0	-0.10074741
cov2_4_5_6_7_9_11	43.7279	-224.442	0.3758518	0	0	0	-0.11137709
cov2_4_5_7_8_10_11	43.7339	-224.445	0.3771703	0	0	0	-0.1003781
cov2_4_5_6_8_9_11	43.7439	-224.45	0.3757269	0	0	0	-0.11151673
cov2_4_5_6_7_10_11	43.7511	-224.454	0.3762186	0	0	0	-0.11077378
cov2_4_5_6_9_10_11	43.7538	-224.455	0.3753751	0	0	0	-0.11154292
cov2_4_5_6_8_10_11	43.7668	-224.462	0.3760889	0	0	0	-0.11093461

cov2_3_6_7_8_9_11	43.8007	-224.479	0.3645264	0	0	0	-0.049617884
cov2_3_5_6_7_8_9	43.8283	-224.492	0.3606035	0	0	0	-0.035700519
cov2_3_5_6_8_9_11	43.8525	-224.504	0.3616554	0	0	0	-0.038963797
cov2_3_6_7_8_10_11	43.865	-224.511	0.3648963	0	0	0	-0.049319788
cov3_4_5_6_7_8_9	43.8713	-224.514	0.3579208	0	0	0	0
cov2_3_5_6_7_8_10	43.883	-224.52	0.3610572	0	0	0	-0.035416864
cov3_4_5_6_7_8_10	43.9068	-224.532	0.3584501	0	0	0	0
cov2_3_4_6_7_8_11	43.912	-224.534	0.368877	0	0	0	-0.094323597
cov2_3_5_6_8_10_11	43.9168	-224.537	0.3620721	0	0	0	-0.038602433
cov2_3_6_7_8_9_10	43.919	-224.538	0.3629673	0	0	0	-0.044069467
cov2_4_5_6_7_9_10	43.9236	-224.54	0.3739298	0	0	0	-0.10231181
cov2_4_5_7_9_10_11	43.9274	-224.542	0.3770526	0	0	0	-0.10021908
cov2_4_5_6_8_9_10	43.9302	-224.543	0.3738353	0	0	0	-0.10266471
cov2_3_5_6_7_9_11	43.9316	-224.544	0.362251	0	0	0	-0.039612411
cov3_4_5_6_8_9_11	43.9401	-224.548	0.3584492	0	0	0	0
cov2_4_6_7_8_9_11	43.9427	-224.55	0.3763774	0	0	0	-0.10450707
cov2_4_5_8_9_10_11	43.9465	-224.551	0.3769443	0	0	0	-0.10027314
cov2_3_5_7_8_9_11	43.9635	-224.56	0.3648466	0	0	0	-0.033756744
cov1_2_3_7_8_9_10	43.9694	-224.563	0.3622318	0	-0.039234589	0	0.012406566
cov2_3_7_8_9_10_11	43.9703	-224.563	0.3652469	0	0	0	-0.03690362
cov2_4_7_8_9_10_11	43.9719	-224.564	0.3768298	0	0	0	-0.10061353
cov3_4_5_7_8_9_11	43.9778	-224.567	0.3613685	0	0	0	0
cov3_4_5_6_8_10_11	43.9831	-224.57	0.3589524	0	0	0	0
cov2_4_6_7_8_10_11	43.9838	-224.57	0.3767163	0	0	0	-0.10409724
cov2_3_6_8_9_10_11	43.9848	-224.571	0.3641963	0	0	0	-0.048214616
cov2_3_5_6_7_10_11	43.9954	-224.576	0.3626673	0	0	0	-0.039244479
cov2_3_5_7_8_10_11	44.0106	-224.584	0.3651131	0	0	0	-0.033654192
cov3_4_5_7_8_10_11	44.0107	-224.584	0.3617398	0	0	0	0
cov2_3_5_6_8_9_10	44.0171	-224.587	0.3603954	0	0	0	-0.034316188
cov3_4_5_6_8_9_10	44.024	-224.59	0.3579327	0	0	0	0
cov3_4_5_6_7_9_11	44.0367	-224.597	0.3589608	0	0	0	0
cov2_4_6_7_8_9_10	44.0405	-224.598	0.3749322	0	0	0	-0.098924845
cov2_3_6_7_9_10_11	44.0481	-224.602	0.3646285	0	0	0	-0.048309352
cov2_4_5_7_8_9_10	44.0614	-224.609	0.3746538	0	0	0	-0.085228663
cov3_5_6_7_8_9_11	44.0617	-224.609	0.3587297	0	0	0	0
cov3_4_5_6_7_10_11	44.0787	-224.618	0.3594664	0	0	0	0
cov2_3_5_6_7_9_10	44.1013	-224.629	0.3609766	0	0	0	-0.034879995
cov3_4_5_8_9_10_11	44.1145	-224.635	0.3612391	0	0	0	0
cov3_5_6_7_8_10_11	44.1174	-224.637	0.3592264	0	0	0	0
cov3_4_5_6_7_9_10	44.1228	-224.64	0.3584439	0	0	0	0
cov2_3_5_8_9_10_11	44.1258	-224.641	0.3644817	0	0	0	-0.032857465
cov3_5_6_7_8_9_10	44.1478	-224.652	0.3581578	0	0	0	0
cov2_4_6_7_9_10_11	44.1678	-224.662	0.3765914	0	0	0	-0.10351936
cov3_4_5_7_8_9_10	44.1711	-224.664	0.361326	0	0	0	0
cov2_3_5_7_9_10_11	44.185	-224.671	0.3649343	0	0	0	-0.033674719
cov2_4_6_8_9_10_11	44.1883	-224.672	0.3764662	0	0	0	-0.1036085
cov3_4_5_7_9_10_11	44.192	-224.674	0.3615902	0	0	0	0

cov3_5_6_8_9_10_11	44.2243	-224.69	0.3586773	0	0	0	0
cov3_5_7_8_9_10_11	44.2521	-224.704	0.3616112	0	0	0	0
cov2_3_5_7_8_9_10	44.2754	-224.716	0.3638078	0	0	0	-0.025547223
cov2_3_5_6_9_10_11	44.2982	-224.727	0.3681253	0	0	0	-0.047554826
cov3_5_6_7_9_10_11	44.315	-224.736	0.3592059	0	0	0	0
cov1_2_7_8_9_10_11	44.3532	-224.755	0.3751413	0	-0.081862898	0	0.005421741
cov3_4_7_8_9_10_11	44.5169	-224.837	0.3622667	0	0	0	0
cov3_4_6_7_8_9_11	44.5171	-224.837	0.3622846	0	0	0	0
cov3_6_7_8_9_10_11	44.5187	-224.838	0.3624701	0	0	0	0
cov3_4_6_7_8_9_10	44.5314	-224.844	0.3619441	0	0	0	0
cov3_4_6_7_8_10_11	44.5729	-224.865	0.3627245	0	0	0	0
cov2_3_5_6_7_8_11	44.6285	-224.892	0.362301	0	0	0	-0.049362111
cov3_4_6_8_9_10_11	44.6636	-224.91	0.3621517	0	0	0	0
cov2_4_5_6_7_8_11	44.6658	-224.911	0.3781357	0	0	0	-0.12900785
cov3_4_5_6_9_10_11	44.67	-224.913	0.3660225	0	0	0	0
cov3_4_6_7_9_10_11	44.7291	-224.943	0.3625662	0	0	0	0
cov3_4_5_6_7_8_11	45.012	-225.084	0.3584354	0	0	0	0
cov2_5_6_7_8_9_11	45.1889	-225.173	0.3739569	0	0	0	-0.058187888
cov2_6_7_8_9_10_11	45.2252	-225.191	0.3734866	0	0	0	-0.052961964
cov2_5_6_7_8_10_11	45.2285	-225.192	0.3743404	0	0	0	-0.057312775
cov2_5_7_8_9_10_11	45.2449	-225.201	0.3748204	0	0	0	-0.053599534
cov2_5_6_7_8_9_10	45.2508	-225.204	0.3728565	0	0	0	-0.054122035
cov2_5_6_7_9_10_11	45.4203	-225.288	0.3742604	0	0	0	-0.056927409
cov2_5_6_8_9_10_11	45.4581	-225.307	0.3741118	0	0	0	-0.056690215
cov4_5_7_8_9_10_11	45.6891	-225.423	0.3710727	0	0	0	0
cov4_5_6_7_8_9_10	45.6939	-225.425	0.3706098	0	0	0	0
cov4_5_6_7_8_10_11	45.7206	-225.439	0.3711536	0	0	0	0
cov4_5_6_7_8_9_11	45.7312	-225.444	0.3707177	0	0	0	0
cov4_5_6_7_9_10_11	45.9084	-225.532	0.3711464	0	0	0	0
cov4_6_7_8_9_10_11	45.921	-225.539	0.37172	0	0	0	0
cov4_5_6_8_9_10_11	45.9297	-225.543	0.3710111	0	0	0	0
cov5_6_7_8_9_10_11	46.0298	-225.593	0.3713431	0	0	0	0
cov1_3_5_6_7_8_9_11	46.7618	-222.449	0.3729111	0	-0.2866907	0	0
cov1_4_5_6_7_8_9_11	46.7671	-222.452	0.3756929	0	-0.3007171	0	0
cov1_4_5_6_7_8_10_11	46.7763	-222.456	0.3758525	0	-0.3008782	0	0
cov1_3_5_6_7_8_10_11	46.7768	-222.457	0.3731357	0	-0.28769271	0	0
cov1_2_5_6_7_8_9_11	46.8345	-222.485	0.3759934	0	-0.30157292	0	-0.004762868
cov1_5_6_7_8_9_10_11	46.8395	-222.488	0.3758406	0	-0.30470202	0	0
cov1_2_5_6_7_8_10_11	46.8468	-222.492	0.3761404	0	-0.30224825	0	-0.004430169
cov1_2_4_5_6_7_8_11	46.8583	-222.497	0.377246	0	-0.29728076	0	-0.027879133
cov1_3_4_5_6_7_8_11	46.8724	-222.504	0.3732564	0	-0.30042903	0	0
cov1_2_3_5_6_7_8_11	46.926	-222.531	0.3736223	0	-0.30057444	0	-0.004922924
cov1_2_3_4_5_6_9_11	47.0387	-222.587	0.3718275	0	-0.24259816	0	-0.026857987
cov1_2_3_4_5_6_10_11	47.0551	-222.596	0.3720132	0	-0.2434957	0	-0.026637712
cov1_2_4_5_6_7_9_11	47.0698	-222.603	0.3767492	0	-0.27221933	0	-0.027947527
cov1_2_4_5_6_7_10_11	47.0812	-222.609	0.3769062	0	-0.27274324	0	-0.027652619
cov1_3_4_5_6_7_9_11	47.0897	-222.613	0.372852	0	-0.27627784	0	0

cov1_3_4_5_6_7_10_11	47.1028	-222.62	0.3730856	0	-0.27708129	0	0
cov1_3_4_5_6_8_9_11	47.1207	-222.628	0.3718777	0	-0.27001165	0	0
cov1_3_4_5_6_8_10_11	47.1346	-222.635	0.3721108	0	-0.27084711	0	0
cov1_3_4_5_6_9_10_11	47.1353	-222.636	0.370632	0	-0.26238666	0	0
cov1_2_4_5_6_8_9_11	47.144	-222.64	0.376578	0	-0.26962704	0	-0.028852617
cov1_2_4_5_6_8_10_11	47.1554	-222.646	0.3767354	0	-0.27014734	0	-0.028557818
cov1_2_3_5_6_7_9_11	47.1677	-222.652	0.3732326	0	-0.27905373	0	-0.003334037
cov1_3_4_6_7_8_9_11	47.1683	-222.652	0.3676809	0	-0.21812746	0	0
cov1_3_5_6_7_9_10_11	47.1702	-222.653	0.3730703	0	-0.28108971	0	0
cov1_4_5_6_7_9_10_11	47.1738	-222.655	0.3759274	0	-0.29505076	0	0
cov1_3_4_6_7_8_10_11	47.1806	-222.658	0.3680526	0	-0.21900337	0	0
cov1_2_3_5_6_7_10_11	47.1833	-222.66	0.3734458	0	-0.28025226	0	-0.003056803
cov1_2_3_4_5_6_7_11	47.1863	-222.661	0.3744588	0	-0.27441491	0	-0.026440061
cov1_2_3_5_6_8_9_11	47.2077	-222.672	0.3722338	0	-0.27313142	0	-0.002923018
cov1_3_5_6_8_9_10_11	47.2096	-222.673	0.3720834	0	-0.27494724	0	0
cov1_2_3_4_5_6_8_11	47.2123	-222.674	0.3735084	0	-0.26783691	0	-0.026777728
cov1_2_4_6_7_8_9_11	47.2174	-222.677	0.375976	0	-0.20947846	0	-0.054922423
cov1_2_4_6_7_8_10_11	47.221	-222.679	0.3762129	0	-0.21036103	0	-0.054071241
cov1_2_3_5_6_8_10_11	47.2241	-222.68	0.3724463	0	-0.27435943	0	-0.002654225
cov1_2_3_5_6_9_10_11	47.2359	-222.686	0.3704288	0	-0.2633354	0	-0.002277384
cov1_2_5_6_7_9_10_11	47.2456	-222.691	0.3762212	0	-0.29620492	0	-0.004715864
cov1_4_5_6_8_9_10_11	47.255	-222.696	0.3757287	0	-0.29314573	0	0
cov1_2_3_4_6_8_9_11	47.2787	-222.707	0.3701716	0	-0.19543819	0	-0.041558942
cov1_2_3_4_6_7_9_11	47.2948	-222.716	0.3708539	0	-0.19672589	0	-0.042503719
cov1_2_3_4_6_9_10_11	47.2965	-222.716	0.3714434	0	-0.19799	0	-0.04300482
cov1_2_3_4_6_8_10_11	47.2969	-222.717	0.3704713	0	-0.19637598	0	-0.041268246
cov1_2_3_4_6_7_10_11	47.312	-222.724	0.3711634	0	-0.19768982	0	-0.042187137
cov1_2_3_4_6_7_8_11	47.3148	-222.726	0.3704676	0	-0.20658835	0	-0.049413156
cov1_2_5_6_8_9_10_11	47.3346	-222.735	0.3760214	0	-0.29447279	0	-0.004680971
cov1_2_4_5_6_9_10_11	47.4833	-222.81	0.3748447	0	-0.25047619	0	-0.036749882
cov1_3_4_5_6_7_8_9	47.5062	-222.821	0.3687513	0	-0.24336236	0	0
cov1_3_4_5_6_7_8_10	47.5126	-222.824	0.3690123	0	-0.24399435	0	0
cov1_2_4_5_6_7_8_9	47.5351	-222.836	0.3724056	0	-0.24441959	0	-0.021085051
cov1_3_4_6_8_9_10_11	47.5379	-222.837	0.3673556	0	-0.21126625	0	0
cov1_2_4_5_6_7_8_10	47.5386	-222.837	0.3725798	0	-0.24469399	0	-0.02077772
cov1_3_4_6_7_9_10_11	47.5649	-222.851	0.3679843	0	-0.212833	0	0
cov1_4_5_6_7_8_9_10	47.591	-222.864	0.3719275	0	-0.26220435	0	0
cov1_2_3_5_6_7_8_9	47.6035	-222.87	0.3688401	0	-0.24945198	0	0.001959033
cov1_3_5_6_7_8_9_10	47.6036	-222.87	0.3689688	0	-0.24808178	0	0
cov1_2_4_6_7_9_10_11	47.609	-222.873	0.3762711	0	-0.20420487	0	-0.05466823
cov1_2_3_5_6_7_8_10	47.6127	-222.874	0.3690794	0	-0.25049643	0	0.00229337
cov1_2_4_6_8_9_10_11	47.6631	-222.9	0.3761145	0	-0.20304607	0	-0.05504902
cov1_2_3_4_5_6_7_8	47.6794	-222.908	0.3699699	0	-0.24691399	0	-0.019415435
cov1_2_5_6_7_8_9_10	47.6863	-222.911	0.3719688	0	-0.26703866	0	0.000699956
cov1_4_6_7_8_9_10_11	47.6889	-222.913	0.3738212	0	-0.23435135	0	0
cov1_2_3_4_6_7_8_9	47.7368	-222.937	0.3665012	0	-0.17031486	0	-0.035231695
cov1_2_3_4_6_7_8_10	47.7436	-222.94	0.3668586	0	-0.17149163	0	-0.034832793

cov1_3_6_7_8_9_10_11	47.8361	-222.986	0.3643821	0	-0.18301117	0	0
cov1_2_3_6_7_8_9_11	47.8371	-222.987	0.3641444	0	-0.18541524	0	0.003352684
cov1_2_3_4_5_6_8_9	47.8509	-222.994	0.3687656	0	-0.21652446	0	-0.020113102
cov1_2_3_4_5_6_9_10	47.8532	-222.995	0.3693983	0	-0.22001706	0	-0.019749139
cov1_2_3_4_5_6_7_9	47.854	-222.995	0.3696076	0	-0.22187371	0	-0.019682609
cov1_2_3_4_5_6_8_10	47.8598	-222.998	0.3690212	0	-0.21744652	0	-0.019866956
cov1_2_3_4_5_6_7_10	47.8618	-222.999	0.3698645	0	-0.22276873	0	-0.019432396
cov1_2_3_6_7_8_10_11	47.8626	-222.999	0.3645135	0	-0.18658221	0	0.004022053
cov1_3_4_5_6_8_9_10	47.9037	-223.02	0.3681321	0	-0.2326029	0	0
cov1_3_4_5_6_7_9_10	47.904	-223.02	0.3690051	0	-0.23765443	0	0
cov1_3_4_6_7_8_9_10	47.9179	-223.027	0.3644004	0	-0.18548154	0	0
cov1_2_4_5_6_7_9_10	47.932	-223.034	0.3727041	0	-0.23845023	0	-0.021412866
cov1_2_4_5_6_8_9_10	47.9748	-223.056	0.3726016	0	-0.23677477	0	-0.022346931
cov1_2_3_5_6_7_9_10	48.0067	-223.071	0.369072	0	-0.24396372	0	0.00208639
cov1_2_3_5_6_8_9_10	48.0144	-223.075	0.3681793	0	-0.23916619	0	0.002358911
cov1_2_4_6_7_8_9_10	48.0511	-223.094	0.3719954	0	-0.17793561	0	-0.047287868
cov1_2_3_4_6_8_9_10	48.0817	-223.109	0.3662463	0	-0.16472767	0	-0.034473362
cov1_2_3_4_6_7_9_10	48.1246	-223.13	0.3668506	0	-0.16527228	0	-0.035245833
cov1_2_3_6_8_9_10_11	48.1951	-223.166	0.3638264	0	-0.17918169	0	0.0036692
cov1_2_3_6_7_9_10_11	48.245	-223.191	0.3644082	0	-0.17991193	0	0.003601862
cov1_2_3_4_5_7_8_11	48.5832	-223.36	0.376354	0	-0.17625766	0	-0.027052449
cov1_2_3_6_7_8_9_10	48.5921	-223.364	0.3605668	0	-0.15615574	0	0.00844229
cov1_2_4_5_7_8_9_11	48.6109	-223.374	0.3789983	0	-0.18086855	0	-0.028756114
cov1_2_4_5_7_8_10_11	48.6151	-223.376	0.3790794	0	-0.18141534	0	-0.028613409
cov1_3_4_5_7_8_9_11	48.6345	-223.385	0.3750508	0	-0.18530125	0	0
cov1_3_4_5_7_8_10_11	48.6394	-223.388	0.3751671	0	-0.18604597	0	0
cov1_4_5_7_8_9_10_11	48.7216	-223.429	0.3781492	0	-0.20435333	0	0
cov1_3_5_7_8_9_10_11	48.757	-223.447	0.3754288	0	-0.19017644	0	0
cov1_2_3_5_7_8_9_11	48.7577	-223.447	0.3755238	0	-0.18944941	0	-0.000899114
cov1_2_3_5_7_8_10_11	48.7634	-223.45	0.3756224	0	-0.19047795	0	-0.000819487
cov1_2_3_4_5_7_9_11	48.7731	-223.455	0.3761489	0	-0.1638466	0	-0.027214347
cov1_2_3_4_5_7_10_11	48.7792	-223.458	0.376268	0	-0.16481101	0	-0.027096038
cov1_2_3_4_5_8_9_11	48.7926	-223.464	0.3754088	0	-0.15903639	0	-0.027478168
cov1_2_3_4_5_8_10_11	48.799	-223.468	0.3755271	0	-0.16003649	0	-0.027362137
cov1_2_3_4_5_9_10_11	48.8021	-223.469	0.3743589	0	-0.15266051	0	-0.027871136
cov1_2_5_7_8_9_10_11	48.838	-223.487	0.3785417	0	-0.20685624	0	-0.002404678
cov1_2_4_5_7_9_10_11	48.8481	-223.492	0.3790831	0	-0.18013536	0	-0.029058655
cov1_2_3_4_7_8_9_11	48.8543	-223.495	0.3729135	0	-0.102883	0	-0.043858382
cov1_2_3_4_7_8_10_11	48.8628	-223.5	0.3731107	0	-0.10369138	0	-0.043669027
cov1_3_4_5_7_9_10_11	48.8718	-223.504	0.3750767	0	-0.18463688	0	0
cov1_3_4_5_8_9_10_11	48.8931	-223.515	0.3743143	0	-0.1800095	0	0
cov1_2_4_5_8_9_10_11	48.9029	-223.52	0.3789431	0	-0.17849131	0	-0.029771496
cov1_2_3_5_7_9_10_11	48.9958	-223.566	0.3755266	0	-0.18880571	0	-0.001075947
cov1_2_3_5_8_9_10_11	49.0251	-223.581	0.3747449	0	-0.18442237	0	-0.000772787
cov1_2_6_7_8_9_10_11	49.0478	-223.592	0.372511	0	-0.19226412	0	0.002376488
cov1_2_3_4_8_9_10_11	49.0695	-223.603	0.3724945	0	-0.10145756	0	-0.043242649
cov1_2_3_4_7_9_10_11	49.0853	-223.611	0.373016	0	-0.10240531	0	-0.043938946

cov1_3_4_7_8_9_10_11	49.1439	-223.64	0.3699117	0	-0.11893911	0	0
cov1_2_4_7_8_9_10_11	49.1866	-223.661	0.378409	0	-0.10983211	0	-0.056443007
cov1_2_3_4_5_7_8_9	49.6407	-223.888	0.3715698	0	-0.11937289	0	-0.01965031
cov1_2_3_4_5_7_8_10	49.6425	-223.889	0.3717003	0	-0.11990041	0	-0.019520968
cov1_3_4_5_7_8_9_10	49.6918	-223.914	0.370942	0	-0.135124	0	0
cov2_3_4_5_6_8_9_11	49.6918	-223.914	0.3653371	0	0	0	-0.087730319
cov1_2_4_5_7_8_9_10	49.7237	-223.93	0.3747056	0	-0.13616265	0	-0.021462941
cov2_3_4_5_6_8_10_11	49.7385	-223.937	0.3657637	0	0	0	-0.087679406
cov2_3_4_5_6_7_9_11	49.7704	-223.953	0.3659272	0	0	0	-0.088431351
cov2_3_4_5_6_7_8_9	49.7752	-223.956	0.363714	0	0	0	-0.080504783
cov2_3_4_5_6_7_8_10	49.8108	-223.974	0.364174	0	0	0	-0.08054651
cov2_3_4_5_6_7_10_11	49.8165	-223.976	0.3663572	0	0	0	-0.088370723
cov1_2_3_5_7_8_9_10	49.8443	-223.99	0.3710751	0	-0.14343508	0	0.005378907
cov1_2_3_4_5_8_9_10	49.8548	-223.995	0.3710509	0	-0.11485081	0	-0.020239075
cov1_2_3_4_5_7_9_10	49.8626	-223.999	0.3716928	0	-0.11869189	0	-0.01989843
cov1_2_3_4_7_8_9_10	49.9313	-224.034	0.3686182	0	-0.060439217	0	-0.035797749
cov2_3_4_5_6_8_9_10	49.9735	-224.055	0.3635274	0	0	0	-0.078808472
cov2_3_4_5_7_8_9_11	49.9751	-224.056	0.3687816	0	0	0	-0.07676094
cov1_2_3_7_8_9_10_11	49.9948	-224.066	0.3661937	0	-0.079226749	0	0.006596817
cov2_3_4_5_7_8_10_11	50.0054	-224.071	0.3690619	0	0	0	-0.076936824
cov2_3_4_5_6_7_9_10	50.0587	-224.097	0.3641033	0	0	0	-0.079379639
cov2_3_4_5_6_9_10_11	50.1091	-224.123	0.3714936	0	0	0	-0.096616291
cov2_3_4_5_8_9_10_11	50.1404	-224.138	0.368442	0	0	0	-0.07572808
cov2_3_4_5_7_9_10_11	50.1939	-224.165	0.3688982	0	0	0	-0.076682146
cov2_3_4_6_7_8_9_11	50.3536	-224.245	0.3690729	0	0	0	-0.084721685
cov2_3_4_5_6_7_8_11	50.3556	-224.246	0.366148	0	0	0	-0.09990369
cov2_3_4_6_7_8_10_11	50.408	-224.272	0.3694264	0	0	0	-0.084863561
cov2_3_4_7_8_9_10_11	50.4117	-224.274	0.3697783	0	0	0	-0.080405191
cov2_3_4_6_7_8_9_10	50.4686	-224.302	0.367528	0	0	0	-0.079393414
cov2_3_4_5_7_8_9_10	50.4763	-224.306	0.3670528	0	0	0	-0.061694096
cov2_4_5_6_7_8_9_11	50.4866	-224.311	0.3756617	0	0	0	-0.11243153
cov2_4_5_6_7_8_10_11	50.51	-224.323	0.3760207	0	0	0	-0.11181556
cov2_3_4_6_8_9_10_11	50.5376	-224.337	0.3687085	0	0	0	-0.083372301
cov2_3_4_6_7_9_10_11	50.5836	-224.36	0.3692109	0	0	0	-0.084153528
cov2_4_5_6_7_8_9_10	50.6758	-224.406	0.3737581	0	0	0	-0.10350051
cov2_3_5_6_7_8_9_11	50.6832	-224.41	0.3619168	0	0	0	-0.040232955
cov2_4_5_7_8_9_10_11	50.728	-224.432	0.3769693	0	0	0	-0.10021574
cov2_4_5_6_7_9_10_11	50.7352	-224.436	0.3759542	0	0	0	-0.11084079
cov2_3_5_6_7_8_10_11	50.7478	-224.442	0.3623387	0	0	0	-0.039855034
cov2_4_5_6_8_9_10_11	50.7512	-224.444	0.375822	0	0	0	-0.11098325
cov3_4_5_6_7_8_9_11	50.8017	-224.469	0.3585936	0	0	0	0
cov2_3_6_7_8_9_10_11	50.8208	-224.479	0.3645069	0	0	0	-0.049636276
cov3_4_5_6_7_8_10_11	50.8442	-224.49	0.3591034	0	0	0	0
cov2_3_5_6_7_8_9_10	50.8472	-224.492	0.3606611	0	0	0	-0.035578175
cov2_3_5_6_8_9_10_11	50.8724	-224.504	0.361679	0	0	0	-0.038902874
cov3_4_5_6_7_8_9_10	50.8826	-224.509	0.3580925	0	0	0	0
cov2_3_5_6_7_9_10_11	50.9514	-224.544	0.3622774	0	0	0	-0.039544284

cov3_4_5_6_8_9_10_11	50.9537	-224.545	0.3585928	0	0	0	0
cov2_4_6_7_8_9_10_11	50.9606	-224.548	0.3764235	0	0	0	-0.10423164
cov2_3_5_7_8_9_10_11	50.9836	-224.56	0.3648551	0	0	0	-0.033735278
cov3_4_5_7_8_9_10_11	50.9933	-224.565	0.3615057	0	0	0	0
cov3_4_5_6_7_9_10_11	51.0497	-224.593	0.3591085	0	0	0	0
cov3_5_6_7_8_9_10_11	51.0785	-224.607	0.3588337	0	0	0	0
cov3_4_6_7_8_9_10_11	51.5369	-224.837	0.3623252	0	0	0	0
cov2_5_6_7_8_9_10_11	52.1975	-225.167	0.374042	0	0	0	-0.057653851
cov4_5_6_7_8_9_10_11	52.7074	-225.422	0.3709081	0	0	0	0
cov1_2_4_5_6_7_8_9_11	53.6857	-222.401	0.3765292	0	-0.27796985	0	-0.027494255
cov1_2_4_5_6_7_8_10_11	53.6971	-222.407	0.3766845	0	-0.27849522	0	-0.027201576
cov1_3_4_5_6_7_8_9_11	53.7052	-222.411	0.3726976	0	-0.28198392	0	0
cov1_3_4_5_6_7_8_10_11	53.7181	-222.417	0.372929	0	-0.2827858	0	0
cov1_2_3_5_6_7_8_9_11	53.7794	-222.448	0.3730679	0	-0.28465139	0	-0.00336839
cov1_3_5_6_7_8_9_10_11	53.782	-222.449	0.3728982	0	-0.28668526	0	0
cov1_4_5_6_7_8_9_10_11	53.7864	-222.451	0.3757194	0	-0.30045365	0	0
cov1_2_3_5_6_7_8_10_11	53.7949	-222.455	0.373279	0	-0.28584438	0	-0.003092884
cov1_2_3_4_5_6_7_8_11	53.8021	-222.459	0.3742819	0	-0.28031897	0	-0.026016049
cov1_2_5_6_7_8_9_10_11	53.8546	-222.485	0.376001	0	-0.30152166	0	-0.004725888
cov1_2_3_4_6_7_8_9_11	53.9175	-222.517	0.3706773	0	-0.20228596	0	-0.042200584
cov1_2_3_4_6_7_8_10_11	53.9345	-222.525	0.3709849	0	-0.20324897	0	-0.041884151
cov1_2_3_4_5_6_7_9_11	54.0185	-222.567	0.3738844	0	-0.2561835	0	-0.026150228
cov1_2_3_4_5_6_7_10_11	54.0333	-222.575	0.3741106	0	-0.25726108	0	-0.025908696
cov1_2_3_4_5_6_8_9_11	54.0471	-222.582	0.3729303	0	-0.24970794	0	-0.026490407
cov1_2_3_4_5_6_9_10_11	54.0588	-222.587	0.3718221	0	-0.24265553	0	-0.026842401
cov1_2_3_4_5_6_8_10_11	54.0626	-222.589	0.3731567	0	-0.25081597	0	-0.026252716
cov1_2_4_5_6_7_9_10_11	54.0896	-222.603	0.3767663	0	-0.27206237	0	-0.02790562
cov1_3_4_5_6_7_9_10_11	54.1098	-222.613	0.3728661	0	-0.27621745	0	0
cov1_3_4_5_6_8_9_10_11	54.1409	-222.628	0.3718895	0	-0.26997278	0	0
cov1_2_4_5_6_8_9_10_11	54.1638	-222.64	0.3765957	0	-0.26948882	0	-0.028801886
cov1_3_4_6_7_8_9_10_11	54.1838	-222.65	0.3678268	0	-0.21830058	0	0
cov1_2_3_5_6_7_9_10_11	54.1879	-222.652	0.373224	0	-0.27906981	0	-0.003356516
cov1_2_3_5_6_8_9_10_11	54.2278	-222.672	0.3722169	0	-0.27315393	0	-0.002965675
cov1_2_4_6_7_8_9_10_11	54.228	-222.672	0.37606	0	-0.20975159	0	-0.054291199
cov1_2_3_4_6_8_9_10_11	54.2978	-222.707	0.370236	0	-0.19558405	0	-0.041404383
cov1_2_3_4_6_7_9_10_11	54.3133	-222.715	0.3709308	0	-0.19689511	0	-0.042330013
cov1_2_3_4_5_6_7_8_9	54.4764	-222.796	0.3694228	0	-0.22777662	0	-0.01924917
cov1_2_3_4_5_6_7_8_10	54.4841	-222.8	0.3696773	0	-0.22866592	0	-0.019000324
cov1_3_4_5_6_7_8_9_10	54.5242	-222.82	0.3688306	0	-0.24320602	0	0
cov1_2_4_5_6_7_8_9_10	54.5519	-222.834	0.3724641	0	-0.24410515	0	-0.020949416
cov1_2_3_5_6_7_8_9_10	54.6228	-222.869	0.3688899	0	-0.24944352	0	0.002064932
cov1_2_3_4_6_7_8_9_10	54.7511	-222.934	0.3666569	0	-0.17073589	0	-0.03492815
cov1_2_3_6_7_8_9_10_11	54.8534	-222.985	0.3642441	0	-0.18572588	0	0.003568996
cov1_2_3_4_5_6_8_9_10	54.8698	-222.993	0.3688266	0	-0.21645316	0	-0.0200426
cov1_2_3_4_5_6_7_9_10	54.8725	-222.994	0.3696765	0	-0.22179877	0	-0.019602711
cov1_2_3_4_5_7_8_9_11	55.5579	-223.337	0.3761002	0	-0.16477434	0	-0.026935971
cov1_2_3_4_5_7_8_10_11	55.5636	-223.34	0.3762137	0	-0.1657206	0	-0.026823479

cov1_2_4_5_7_8_9_10_11	55.6311	-223.374	0.378997	0	-0.18088102	0	-0.028760191
cov1_3_4_5_7_8_9_10_11	55.6545	-223.385	0.3750343	0	-0.18536669	0	0
cov1_2_3_5_7_8_9_10_11	55.777	-223.447	0.3754781	0	-0.18954063	0	-0.001011966
cov1_2_3_4_5_7_9_10_11	55.793	-223.455	0.376123	0	-0.1639205	0	-0.027251568
cov1_2_3_4_5_8_9_10_11	55.8124	-223.464	0.3753781	0	-0.15912577	0	-0.027521944
cov1_2_3_4_7_8_9_10_11	55.8741	-223.495	0.3729521	0	-0.10294349	0	-0.04377005
cov2_3_4_5_6_7_8_9_11	56.5103	-223.813	0.3656262	0	0	0	-0.089295406
cov2_3_4_5_6_7_8_10_11	56.557	-223.837	0.3660583	0	0	0	-0.089228479
cov1_2_3_4_5_7_8_9_10	56.6604	-223.888	0.3716087	0	-0.11930885	0	-0.019605149
cov2_3_4_5_6_8_9_10_11	56.7107	-223.913	0.3653964	0	0	0	-0.087618362
cov2_3_4_5_6_7_9_10_11	56.789	-223.953	0.3659917	0	0	0	-0.088297654
cov2_3_4_5_6_7_8_9_10	56.7918	-223.954	0.3638177	0	0	0	-0.080359174
cov2_3_4_5_7_8_9_10_11	56.9949	-224.055	0.368818	0	0	0	-0.076687013
cov2_3_4_6_7_8_9_10_11	57.373	-224.245	0.3690189	0	0	0	-0.08481799
cov2_4_5_6_7_8_9_10_11	57.4937	-224.305	0.3757588	0	0	0	-0.11188705
cov2_3_5_6_7_8_9_10_11	57.7031	-224.41	0.3619447	0	0	0	-0.04015724
cov3_4_5_6_7_8_9_10_11	57.8146	-224.465	0.3587445	0	0	0	0
cov1_2_3_4_5_6_7_8_9_11	60.6369	-222.366	0.3737161	0	-0.26219327	0	-0.025729772
cov1_2_3_4_5_6_7_8_10_11	60.6515	-222.374	0.3739406	0	-0.26326749	0	-0.025489973
cov1_2_4_5_6_7_8_9_10_11	60.7056	-222.401	0.3765457	0	-0.27783547	0	-0.027443252
cov1_3_4_5_6_7_8_9_10_11	60.7253	-222.411	0.3727153	0	-0.28192749	0	0
cov1_2_3_5_6_7_8_9_10_11	60.7996	-222.448	0.3730576	0	-0.28468056	0	-0.003390597
cov1_2_3_4_6_7_8_9_10_11	60.936	-222.516	0.3707541	0	-0.2024556	0	-0.0420266
cov1_2_3_4_5_6_7_9_10_11	61.0387	-222.567	0.3738896	0	-0.25616986	0	-0.026141705
cov1_2_3_4_5_6_8_9_10_11	61.0673	-222.582	0.3729299	0	-0.24970883	0	-0.026490997
cov1_2_3_4_5_6_7_8_9_10	61.4949	-222.795	0.3694914	0	-0.22769176	0	-0.019182133
cov1_2_3_4_5_7_8_9_10_11	62.5778	-223.337	0.3760728	0	-0.16485313	0	-0.026975085
cov2_3_4_5_6_7_8_9_10_11	63.5289	-223.812	0.3656929	0	0	0	-0.08914694
cov1_2_3_4_5_6_7_8_9_10_11	67.6571	-222.366	0.3737191	0	-0.26214757	0	-0.025735558

SE	bsnp_rs7817754	SE	bsnp_rs2047962	SE	bsnp_rs6469668	SE	bsnp_rs6469667	SE	bsnp_rs7832958
0	0	0	0	0	0	0	0	0	0
0	0	0	0	0	0	0	0	0	0
0	0	0	0	0	0	0	0	0	0
0	-0.071527083	0	0	0	0	0	0	0	0
0	0	0	0	0	0	0	0	0	0
0	0	0	0	0	0	0	0	0	0
0	0	0	0	0	0	0	-0.027508302	0	0
0	0	0	0	0	0	0	0	0	0
0	0	0	0	0	-0.010848369	0	0	0	0
0	0	0	0	0	0	0	0	0	-0.014283318
0	0	0	0	0	0	0	0	0	0
0	0	0	0.000697947	0	0	0	0	0	0
0	0	0	0	0	0.13620756	0	0	0	0
0	0	0	0.083949291	0	0	0	0	0	0
0	-0.055917828	0	0	0	0	0	0	0	0
0	-0.055471206	0	0	0	0	0	0	0	0
0	0	0	0.095281979	0	0	0	0	0	0
0	0	0	0	0	0	0	0.1174768	0	0
0	-0.063535558	0	0	0	0	0	0	0	0
0	-0.11904471	0	0	0	0	0	0	0	0
0	0	0	0	0	0	0	0	0	0
0	0	0	0	0	0	0	0	0	0
0	-0.11740162	0	0	0	0	0	0	0	0.091653334
0	-0.065325258	0	0	0	0	0	0	0	0
0	0	0	0	0	0	0	0	0	0
0	0	0	0	0	0	0	0	0	0
0	0	0	0	0	0	0	0	0	0
0	0	0	0	0	0	0	0	0	-0.024081563
0	0	0	0	0	0	0	0	0	-0.024241663
0	0	0	0	0	0	0	0	0	0
0	0	0	0	0	0	0	0	0	0
0	0	0	0.010637009	0	0	0	0	0	0
0	0	0	0.009521785	0	0	0	0	0	0
0	0	0	0	0	0	0	0	0	0
0	0	0	0	0	-0.001295272	0	0	0	0
0	0	0	0	0	0	0	0	0	0
0	0	0	0	0	0	0	-0.000895206	0	0
0	0	0	0	0	-0.002858301	0	0	0	0
0	0	0	0	0	0	0	0	0	0
0	0	0	0	0	0	0	-0.002420194	0	0
0	-0.067830139	0	0	0	0	0	-0.020797718	0	0
0	-0.073620238	0	0	0	-0.01609066	0	0	0	0
0	-0.073212822	0	-0.007852257	0	0	0	0	0	0
0	-0.069158305	0	0	0	0	0	0	0	0
0	0	0	0	0	0	0	0	0	-0.020996368

0	0	0	0	0	0	0	0	0	0
0	0	0	0	0	0	0	0	0	0
0	0	0	0	0	0.045720289	0	0	0	0
0	0	0	0	0	0	0	0	0	0
0	0	0	0	0	0	0	0	0	-0.018092118
0	0	0	0	0	0	0	0.013655731	0	0
0	0	0	0	0	0	0	0	0	0
0	0	0	0.036223126	0	0	0	-0.052485143	0	0
0	0	0	0	0	0.028646392	0	-0.050239297	0	0
0	0	0	0	0	0	0	-0.027976088	0	-0.016796013
0	0	0	0	0	0	0	-0.027930742	0	0
0	0	0	0	0	0	0	-0.028127429	0	0
0	0	0	0	0	0.13703493	0	0.11903507	0	0
0	0	0	0.016743926	0	0	0	0	0	0
0	0	0	0.038944275	0	-0.04457448	0	0	0	0
0	0	0	0	0	0	0	0	0	-0.009776778
0	0	0	0	0	0	0	0	0	0
0	0	0	0	0	0	0	0	0	-0.40049887
0	0	0	0	0	0.003751699	0	0	0	0
0	0	0	0	0	-0.011065726	0	0	0	-0.014811481
0	0	0	0	0	-0.01101926	0	0	0	0
0	0	0	-0.000276213	0	0	0	0	0	-0.014345349
0	0	0	-7.59E-05	0	0	0	0	0	0
0	0	0	0	0	0.135937	0	0	0	0
0	0	0	0.081306345	0	0	0	0.11218692	0	0
0	-0.029666226	0	0	0	0.11752438	0	0	0	0
0	0	0	0	0	0.13859267	0	0	0	-0.029896351
0	0	0	0.031805558	0	0.1074359	0	0	0	0
0	0	0	0	0	0.13840251	0	0	0	0
0	0	0	0	0	0.12750236	0	0	0	0
0	0	0	0	0	0.12738005	0	0	0	0
0	-0.06563695	0	0	0	0	0	0.12111364	0	0
0	0	0	0	0	0.13610909	0	0	0	0
0	0	0	0	0	0	0	0.13406852	0	0
0	0	0	0	0	0	0	0.13429363	0	0
0	0	0	0.084542152	0	0	0	0	0	0
0	0	0	0.10749561	0	0	0	0	0	0
0	-0.03978237	0	0.067549811	0	0	0	0	0	0
0	0	0	0.073326751	0	0	0	0	0	0
0	0	0	0.073561117	0	0	0	0	0	0
0	-0.075190679	0	0	0	0	0	0	0	0
0	0	0	0.080428213	0	0	0	0	0	0
0	0	0	0.079966745	0	0	0	0	0	0
0	0	0	0.083081388	0	0	0	0	0	-0.010195915
0	0	0	0.083322796	0	0	0	0	0	0
0	-0.054237398	0	0	0	0	0	0	0	0
0	-0.054780062	0	0	0	0	0	0	0	0

0	-0.10228577	0	0	0	0	0	0	0	0
0	0	0	0	0	0	0	0.11149022	0	0
0	-0.090250107	0	0	0	0	0	0	0	0
0	-0.10031294	0	0	0	0	0	0	0	0.071203017
0	-0.038372012	0	0.074294135	0	0	0	0	0	0
0	-0.089816198	0	0	0	0	0	0	0	0
0	-0.087943741	0	0	0	0	0	0	0	0.058500982
0	-0.087463495	0	0	0	0	0	0	0	0.058060809
0	-0.054831647	0	0	0	0	0	0	0	0
0	-0.054350891	0	0	0	0	0	0	0	0
0	0	0	0	0	0	0	0	0	0
0	-0.10749816	0	0	0	0	0	0	0	0
0	0	0	0	0	0	0	0	0	0
0	0	0	0	0	0	0	0.11870034	0	-0.024217401
0	-0.057307397	0	0	0	-0.00718875	0	0	0	0
0	0	0	0.094773608	0	0	0	0	0	0
0	-0.057329483	0	0	0	0	0	0	0	0
0	0	0	0.10981812	0	-0.019809472	0	0	0	0
0	-0.057055107	0	0	0	-0.008464916	0	0	0	0
0	-0.05560918	0	0	0	0	0	0	0	0
0	0	0	0	0	0	0	0.11825057	0	0
0	-0.055249255	0	0.002355806	0	0	0	0	0	0
0	-0.055930902	0	0	0	0	0	0.00038563	0	0
0	-0.10562883	0	0	0	0	0	0	0	0.078361666
0	-0.056575294	0	0	0	0	0	0	0	0
0	-0.055069325	0	0.001429684	0	0	0	0	0	0
0	-0.055429546	0	0	0	0	0	-0.001135362	0	0
0	0	0	0.094807261	0	0	0	0	0	-0.003698955
0	0	0	0.095151961	0	0	0	0	0	0
0	0	0	0.09524729	0	0	0	0.000230528	0	0
0	0	0	0	0	0	0	0.11727911	0	0
0	-0.063817083	0	0	0	0	0	0	0	0
0	-0.12255368	0	0	0	-0.018176859	0	0	0	0
0	-0.1208916	0	0	0	-0.018072129	0	0	0	0.093914937
0	-0.11347579	0	0	0	0	0	-0.013611094	0	0
0	-0.11695189	0	0	0	0	0	0	0	-0.27459167
0	-0.07118263	0	0	0	0	0	0	0	0
0	0	0	0	0	0	0	0	0	-0.026206886
0	0	0	0	0	0	0	0	0	0
0	0	0	0	0	0	0	0	0	-0.025919514
0	-0.11167318	0	0	0	0	0	-0.014073272	0	0.085202222
0	0	0	0	0	0	0	0	0	0
0	-0.12035348	0	-0.006968285	0	0	0	0	0	0
0	-0.11681765	0	0	0	0	0	0	0	0
0	-0.05820278	0	0	0	0.026773422	0	0	0	0
0	0	0	0	0	0	0	0	0	0
0	0	0	0	0	0	0	0	0	0

0	0	0	0	0	0.032654658	0	0	0	0
0	0	0	0	0	0	0	0.035444509	0	0
0	-0.11870136	0	-0.006971289	0	0	0	0	0	0.091256002
0	0	0	0	0	0	0	0	0	0
0	-0.11515287	0	0	0	0	0	0	0	0.091183591
0	0	0	0	0	0	0	0.033637827	0	0
0	0	0	0	0	0.03113006	0	0	0	0
0	-0.066042313	0	0	0	0	0	0.017475605	0	0
0	0	0	0	0	0	0	0	0	0
0	0	0	0	0	0	0	0	0	-0.041076005
0	0	0	0	0	0	0	0	0	0
0	0	0	0	0	0	0	0	0	-0.024886466
0	0	0	0	0	0	0	0	0	-0.02468627
0	0	0	0	0	0	0	0	0	0
0	0	0	0	0	0	0	0	0	0
0	0	0	0	0	0	0	0	0	0
0	0	0	0.045720345	0	-0.04226247	0	0	0	0
0	0	0	0.044558265	0	-0.039699063	0	0	0	0
0	0	0	0	0	0	0	0	0	-0.38132864
0	0	0	0	0	0	0	0	0	0
0	0	0	0	0	0	0	0	0	-0.38153258
0	0	0	0.009273773	0	0	0	0	0	-0.022235603
0	0	0	0.021490596	0	0	0	-0.017531561	0	0
0	0	0	0.009452287	0	0	0	0	0	0
0	0	0	0.008125767	0	0	0	0	0	-0.022602329
0	0	0	0.02134633	0	0	0	-0.018866937	0	0
0	0	0	0	0	0	0	0	0	-0.024283896
0	0	0	0.008306656	0	0	0	0	0	0
0	0	0	0	0	0	0	0	0	0
0	0	0	0	0	0	0	0	0	-0.02454394
0	0	0	0	0	-0.001454762	0	0	0	-0.024112418
0	0	0	0	0	0	0	0	0	0
0	0	0	0	0	0	0	-0.001044752	0	-0.024098287
0	0	0	0.01570666	0	0	0	0	0	0
0	0	0	0	0	-0.003047041	0	0	0	-0.02431755
0	0	0	0	0	0	0	-0.002577934	0	-0.024287894
0	0	0	0	0	0	0	0	0	-0.024264379
0	0	0	0.015266039	0	0	0	0	0	0
0	0	0	0	0	-0.00142463	0	0	0	0
0	0	0	0	0	0	0	0	0	0
0	0	0	0.010316712	0	0	0	0	0	0
0	0	0	0	0	0	0	-0.001070102	0	0
0	0	0	0	0	-0.003014253	0	0	0	0
0	0	0	0	0	0	0	-0.002609265	0	0
0	0	0	0	0	0	0	0	0	0
0	0	0	0	0	-0.001909548	0	0	0	0
0	0	0	0	0	0	0	0	0	0

0	0	0	0	0	0	0	-0.00118196	0	0
0	0	0	0	0	-0.001901093	0	0.000829004	0	0
0	0	0	0	0	-0.001303623	0	0	0	0
0	0	0	0	0	0	0	-0.000474057	0	0
0	0	0	0	0	-0.002496595	0	0	0	0
0	0	0	0	0	-0.003230596	0	0.000502756	0	0
0	0	0	0	0	0	0	-0.001418427	0	0
0	-0.070678223	0	0	0	0	0	-0.03177835	0	0
0	-0.062825253	0	0.014584868	0	0	0	-0.031348733	0	0
0	-0.067275556	0	0	0	0.002011745	0	-0.022448514	0	0
0	-0.071198048	0	0.02350698	0	-0.036274383	0	0	0	0
0	0	0	0	0	0	0	0	0	-0.4086856
0	-0.076890196	0	0	0	-0.021769563	0	0	0	0
0	-0.071291712	0	-0.005198933	0	0	0	0	0	0
0	0	0	0	0	0	0	0	0	-0.021143167
0	0	0	0	0	0.045989053	0	0	0	-0.019014623
0	0	0	0	0	0	0	0	0	0
0	0	0	0	0	0.045965538	0	0	0	0
0	0	0	0	0	0.052220852	0	-0.014147229	0	0
0	0	0	0	0	0.044475333	0	0	0	0
0	0	0	0	0	0	0	0	0	-0.40320047
0	0	0	0	0	0	0	0	0	-0.024405877
0	0	0	0	0	0	0	0	0	0
0	0	0	0	0	0	0	0.013382948	0	-0.017834894
0	0	0	0	0	0	0	0.006593353	0	0
0	0	0	0	0	0	0	0.013349976	0	0
0	0	0	0.035205146	0	0	0	-0.052090944	0	-0.0110538
0	0	0	0.035461321	0	0	0	-0.05221672	0	0
0	0	0	0.036736055	0	0	0	-0.049776179	0	0
0	0	0	0.03940598	0	-0.00528837	0	-0.050483701	0	0
0	0	0	0	0	0.13677198	0	0.11314937	0	0
0	-0.031905526	0	0	0	0.11694411	0	0.12056807	0	0
0	0	0	0	0	0.028983564	0	-0.050993424	0	-0.017481047
0	0	0	0	0	0.029015765	0	-0.050980756	0	0
0	0	0	0	0	0	0	-0.027228589	0	-0.37735469
0	0	0	0	0	0.029944086	0	-0.047537769	0	0
0	0	0	0	0	0.13969856	0	0.1207469	0	-0.033243977
0	0	0	0	0	0.13943854	0	0.12018103	0	0
0	0	0	0	0	0.1191784	0	0.1252193	0	0
0	0	0	0	0	0.1189568	0	0.12501833	0	0
0	0	0	0.026166604	0	0.11335668	0	0.11706558	0	0
0	0	0	0	0	0	0	-0.031220994	0	-0.01815098
0	0	0	0	0	0	0	-0.030798603	0	0
0	0	0	0.037719705	0	-0.029598033	0	0	0	0
0	0	0	0	0	0	0	0	0	-0.39330898
0	0	0	0	0	0.13711061	0	0.11913543	0	0
0	0	0	0.016132286	0	0	0	0	0	-0.003862804

0	0	0	0.01659726	0	0	0	0	0	0
0	0	0	0.037224239	0	-0.043202356	0	0	0	-0.007987687
0	0	0	0.037853392	0	-0.043702442	0	0	0	0
0	0	0	0	0	-0.011215183	0	0	0	-0.40300385
0	0	0	0	0	0.00269798	0	0	0	-0.009203971
0	0	0	0	0	0.00303555	0	0	0	0
0	0	0	-0.000430096	0	0	0	0	0	-0.4006733
0	0	0	0.067177912	0	0	0	0.12539514	0	0
0	0	0	0.067332068	0	0	0	0.12548976	0	0
0	-0.04230109	0	0	0	0.10925387	0	0	0	0
0	0	0	0	0	0.13991097	0	0	0	-0.050690835
0	-0.043237238	0	0.063379019	0	0	0	0.11572524	0	0
0	0	0	0.10589192	0	0	0	0.11424532	0	0
0	0	0	0	0	0.13971158	0	0	0	0
0	0	0	0.081986692	0	0	0	0.10598779	0	0
0	-0.054946431	0	0	0	0	0	0.134442	0	0
0	-0.05433598	0	0	0	0	0	0.13412494	0	0
0	-0.076027527	0	0	0	0	0	0.11321774	0	0
0	0	0	0.033081505	0	0.10600759	0	0	0	0
0	0	0	0	0	0.12851843	0	0	0	0
0	0	0	0	0	0.12876334	0	0	0	0
0	0	0	0	0	0.13606025	0	0	0	0
0	-0.10386231	0	0	0	0	0	0.12041762	0	0
0	-0.052184868	0	0.06317723	0	0	0	0	0	0
0	0	0	0.080127052	0	0	0	0.11295816	0	-0.013664598
0	-0.10105829	0	0	0	0	0	0.11960033	0	0.068641374
0	0	0	0.080447568	0	0	0	0.11262652	0	0
0	0	0	0.1113897	0	0	0	0	0	0
0	-0.026700559	0	0.026732798	0	0.095209382	0	0	0	0
0	0	0	0	0	0.13809658	0	0	0	-0.3966859
0	0	0	0	0	0	0	0.12820305	0	0
0	0	0	0	0	0	0	0.12839573	0	0
0	-0.029764989	0	0	0	0.10856606	0	0	0	0
0	-0.029669715	0	0	0	0.10868458	0	0	0	0
0	0	0	0.026311068	0	0.11439304	0	0	0	-0.024921607
0	0	0	0.026959188	0	0.1136027	0	0	0	0
0	-0.029880117	0	0	0	0.11719443	0	0	0	0
0	0	0	0.04992452	0	0.092249067	0	0	0	0
0	-0.032042392	0	0	0	0.11573479	0	0	0	0
0	-0.02734828	0	0	0	0.11926506	0	0	0	-0.003519457
0	0	0	0.033798002	0	0.094649498	0	0	0	0
0	0	0	0.033544939	0	0.095319369	0	0	0	0
0	0	0	0	0	0.1292379	0	0	0	-0.030450452
0	0	0	0	0	0.12904722	0	0	0	-0.030484095
0	0	0	0.074484832	0	0	0	0	0	0
0	0	0	0	0	0.12908284	0	0	0	0
0	0	0	0	0	0.12889814	0	0	0	0

0	0	0	0.074627572	0	0	0	0	0	0
0	0	0	0	0	0.13847526	0	0	0	-0.029940777
0	0	0	0	0	0	0	0.13607784	0	-0.030892147
0	0	0	0	0	0.13828766	0	0	0	0
0	0	0	0	0	0	0	0.13627729	0	-0.030592511
0	0	0	0	0	0	0	0.13549335	0	0
0	0	0	0	0	0	0	0.13569755	0	0
0	-0.066688125	0	0	0	0	0	0	0	0
0	-0.066233532	0	0	0	0	0	0	0	0
0	0	0	0	0	0.12724663	0	0	0	0
0	0	0	0	0	0.12707721	0	0	0	0
0	0	0	0	0	0.12730199	0	0	0	0
0	-0.065806009	0	0	0	0	0	0.12073814	0	0
0	0	0	0	0	0	0	0.13439955	0	0
0	0	0	0	0	0	0	0.13371587	0	0
0	0	0	0	0	0	0	0.13398106	0	0
0	-0.035409089	0	0.059954953	0	0	0	0	0	0
0	-0.035902407	0	0.059882791	0	0	0	0	0	0
0	0	0	0.082094294	0	0	0	0	0	-0.029773973
0	0	0	0.095049813	0	0	0	0	0	0
0	0	0	0.095492288	0	0	0	0	0	0
0	0	0	0.082382764	0	0	0	0	0	0
0	-0.030634635	0	0.089944831	0	0	0	0	0	0
0	-0.070172532	0	0.059519885	0	0	0	0	0	0
0	-0.10262617	0	0	0	0	0	0	0	0
0	-0.067668413	0	0.060213765	0	0	0	0	0	0.049009561
0	-0.10042002	0	0	0	0	0	0	0	0.051249173
0	0	0	0.10675688	0	0	0	0	0	-0.005989971
0	0	0	0.10709485	0	0	0	0	0	0
0	0	0	0.071692471	0	0	0	0	0	-0.01523558
0	0	0	0.07198972	0	0	0	0	0	0
0	0	0	0.071963533	0	0	0	0	0	-0.014974882
0	0	0	0.072258775	0	0	0	0	0	0
0	0	0	0.073272345	0	0	0	0	0	0
0	-0.075251132	0	0	0	0	0	0	0	0
0	-0.03399133	0	0.062865278	0	0	0	0	0	0
0	-0.0337712	0	0.062635971	0	0	0	0	0	0
0	-0.086334335	0	0	0	0	0	0	0	0
0	-0.085535338	0	0	0	0	0	0	0	0
0	0	0	0.082792698	0	0	0	0	0	-0.38465109
0	-0.083908175	0	0	0	0	0	0	0	0.053064265
0	-0.074023233	0	0.0625445	0	0	0	0	0	0
0	-0.083055927	0	0	0	0	0	0	0	0.052150866
0	0	0	0.079226758	0	0	0	0	0	0
0	0	0	0	0	0	0	0.11211891	0	-0.041999458
0	0	0	0.078920203	0	0	0	0	0	0
0	0	0	0.096770064	0	-0.022578948	0	0	0	0

0	-0.07155794	0	0.063424503	0	0	0	0	0	0.056864514
0	0	0	0.097321724	0	-0.024201083	0	0	0	0
0	0	0	0.076023643	0	0	0	0.018389424	0	0
0	0	0	0.075921062	0	0	0	0.016970288	0	0
0	0	0	0	0	0	0	0.11157606	0	0
0	0	0	0.078697194	0	0	0	0	0	-0.010888734
0	0	0	0.079127751	0	0	0	0	0	0
0	0	0	0.078180002	0	0	0	0	0	-0.011052147
0	0	0	0.080080513	0	0	0	0	0	0
0	0	0	0.078617657	0	0	0	0	0	0
0	-0.060833762	0	0	0	0	0	0	0	0
0	-0.099817072	0	0	0	0	0	0	0	-0.29914466
0	-0.087681952	0	0	0	0	0	0	0	0
0	-0.044395696	0	0.070163143	0	0	0	0	0	0
0	-0.087104699	0	0	0	0	0	0	0	0
0	-0.060269707	0	0	0	0	0	0	0	0
0	-0.054501506	0	0	0	0	0	0	0	0
0	-0.085310679	0	0	0	0	0	0	0	0.055509723
0	-0.05505426	0	0	0	0	0	0	0	0
0	-0.054690757	0	0	0	0	0	0.03510961	0	0
0	-0.087757149	0	0	0	0	0	0	0	-0.29318549
0	-0.054322127	0	0	0	0	0	0	0	0
0	-0.084684221	0	0	0	0	0	0	0	0.054887715
0	-0.054256339	0	0	0	0	0	0.033401408	0	0
0	-0.10264151	0	0	0	0	0	0	0	0
0	-0.087286176	0	0	0	0	0	0	0	-0.29402862
0	-0.094198415	0	0	0	-0.010582829	0	0	0	0
0	0	0	0	0	0	0	0	0	-0.045645947
0	-0.038361814	0	0.088797339	0	-0.019757236	0	0	0	0
0	-0.093988467	0	0	0	-0.011618257	0	0	0	0
0	0	0	0	0	0	0	0	0	-0.045566124
0	-0.10066314	0	0	0	0	0	0	0	0.071316811
0	0	0	0	0	0	0	0.12516984	0	-0.53291413
0	-0.090799858	0	0	0	0	0	0	0	0
0	-0.091806673	0	0	0	-0.010327711	0	0	0	0.061901058
0	0	0	0	0	0	0	0.11153065	0	0
0	0	0	0	0	0	0	0	0	0
0	-0.090409046	0	0	0	0	0	0.001687984	0	0
0	-0.089904463	0	0.000957525	0	0	0	0	0	0
0	-0.090053775	0	0	0	0	0	0	0	0
0	-0.03903732	0	0.07309209	0	0	0	0.005602365	0	0
0	0	0	0	0	0	0	0	0	0
0	-0.091560122	0	0	0	-0.011377907	0	0	0	0.061621591
0	-0.090240547	0	0	0	0	0	0	0	0
0	-0.08976743	0	0.00013736	0	0	0	0	0	0
0	-0.08983685	0	0	0	0	0	0.000207527	0	0
0	-0.088503717	0	0	0	0	0	0	0	0.057660079

0	-0.088073455	0	0	0	0	0	0.001480884	0	0.058646534
0	-0.087552791	0	0.001085166	0	0	0	0	0	0.058350251
0	-0.087706084	0	0	0	0	0	0	0	0.058244867
0	-0.050579059	0	0	0	0.017939792	0	0	0	0
0	-0.087899862	0	0	0	0	0	0	0	0.057503245
0	-0.087372268	0	0.000257924	0	0	0	0	0	0.058027025
0	-0.087463759	0	0	0	0	0	-6.93E-07	0	0.058060867
0	-0.050491021	0	0	0	0.016719976	0	0	0	0
0	-0.10930105	0	0	0	0	0	0	0	0
0	-0.054489888	0	0	0	0	0	0	0	0
0	-0.053483061	0	0.031968099	0	-0.034346753	0	0	0	0
0	-0.10738241	0	0	0	0	0	0	0	0.072415761
0	-0.053023764	0	0.033000325	0	-0.03650891	0	0	0	0
0	0	0	0.1193392	0	-0.033933072	0	0	0	0
0	-0.065213288	0	0	0	-0.022288591	0	0	0	0
0	-0.10516373	0	0	0	0	0	0	0	-0.28915696
0	-0.10970771	0	0	0	0	0	0.02175123	0	0
0	0	0	0.09434481	0	0	0	0	0	-0.36983407
0	-0.06469222	0	0	0	-0.02310914	0	0	0	0
0	-0.06124252	0	0	0	-0.02365454	0	0.021952447	0	0
0	-0.10766319	0	0	0	0	0	0.021126116	0	0.080631784
0	-0.10153635	0	0	0	0.011511921	0	0	0	0
0	-0.060809497	0	0	0	-0.024501498	0	0.021154778	0	0
0	0	0	0	0	0	0	0	0	0
0	0	0	0	0	0	0	0	0	0
0	0	0	0	0	0	0	0	0	0
0	0	0	0.11399916	0	-0.02805723	0	0.01252299	0	0
0	0	0	0.095916748	0	0	0	-0.008344578	0	0
0	0	0	0.093607423	0	0	0	0	0	-0.008667301
0	-0.057003129	0	0	0	-0.00754075	0	0	0	0
0	-0.058229458	0	0	0	0	0	-0.008186179	0	0
0	0	0	0	0	0	0	0.11848055	0	-0.024285238
0	0	0	0.094003534	0	0	0	0	0	0
0	-0.057013637	0	0	0	0	0	0	0	0
0	-0.058199513	0	-0.0020724	0	0	0	0	0	0
0	0	0	0.11022575	0	-0.020077663	0	0	0	0
0	0	0	0.10955582	0	-0.01963799	0	0	0	-0.001063752
0	-0.099155236	0	0	0	0.012421182	0	0	0	0.072204617
0	-0.05499134	0	0.002210463	0	0	0	0	0	0
0	-0.055617808	0	0	0	0	0	0.000227002	0	0
0	0	0	0	0	0	0	0.11803367	0	0
0	-0.054598991	0	0.004289414	0	0	0	-0.002964981	0	0
0	-0.057616851	0	0	0	0	0	-0.009186982	0	0
0	-0.057514425	0	-0.002232388	0	0	0	0	0	0
0	-0.054050455	0	0.004470026	0	0	0	-0.004611387	0	0
0	0	0	0.094771057	0	0	0	0.000241593	0	-0.003699926
0	0	0	0.095117985	0	0	0	0.000227924	0	0

0	-0.12180797	0	0.034638411	0	-0.048034801	0	0	0	0
0	-0.12046072	0	0	0	-0.018188103	0	0	0	-0.27488222
0	-0.12020192	0	0.034283124	0	-0.047623886	0	0	0	0.099562883
0	0	0	0	0	0	0	0	0	-0.39124004
0	-0.12923267	0	0	0	-0.027872266	0	0	0	0
0	0	0	0	0	0	0	0	0	-0.39104298
0	-0.12657605	0	0	0	-0.02398012	0	0.007089924	0	0
0	-0.11152912	0	0	0	0	0	-0.013356931	0	-0.26908263
0	-0.12743181	0	0	0	-0.027565494	0	0	0	0.096086099
0	-0.066031412	0	0	0	0.015362194	0	0	0	0
0	-0.11827283	0	-0.007092594	0	0	0	0	0	-0.2760446
0	-0.11472852	0	0	0	0	0	0	0	-0.27450049
0	-0.12377006	0	0	0	-0.022238983	0	0.005092514	0	0.096771462
0	-0.1138183	0	0	0	0	0	-0.018882433	0	0
0	-0.11060636	0	0.005817479	0	0	0	-0.017957879	0	0
0	0	0	0	0	0	0	0	0	-0.026454309
0	-0.07108919	0	0	0	0	0	0.003346439	0	0
0	0	0	0	0	0	0	0	0	-0.026193635
0	0	0	0	0	0	0	0	0	0
0	0	0	0	0	0.032673582	0	0	0	-0.02471488
0	0	0	0	0	0	0	0	0	-0.026139863
0	0	0	0	0	0	0	0.035520894	0	-0.02480917
0	0	0	0	0	0	0	0	0	0
0	0	0	0	0	0	0	0.033692471	0	-0.024966857
0	0	0	0	0	0.031095503	0	0	0	-0.024821709
0	0	0	0	0	0.032685648	0	0	0	0
0	-0.1120567	0	0	0	0	0	-0.019753475	0	0.083161288
0	0	0	0	0	0	0	0	0	0
0	0	0	0	0	0	0	0.035396347	0	0
0	-0.10848882	0	0.006499714	0	0	0	-0.018922249	0	0.083351513
0	0	0	0	0	0	0	0	0	-0.43885872
0	0	0	0	0	0.02076307	0	0.022277518	0	0
0	0	0	0	0	0	0	0.033572917	0	0
0	0	0	0	0	0.03111274	0	0	0	0
0	-0.11883555	0	-0.004964402	0	0	0	0	0	0
0	0	0	0	0	0	0	0	0	-0.033296366
0	0	0	0	0	0	0	0	0	-0.033035669
0	0	0	0	0	0.027575883	0	0	0	0
0	0	0	0	0	0.019579085	0	0.021299633	0	0
0	0	0	0	0	0	0	0	0	0
0	0	0	0	0	0	0	0.029614123	0	0
0	0	0	0	0	0	0	0	0	0
0	-0.058895463	0	0	0	0.024766855	0	0.003876412	0	0
0	0	0	0	0	0.026328616	0	0	0	0
0	0	0	0	0	0	0	0	0	0
0	0	0	0	0	0	0	0	0	-0.38580934
0	0	0	0	0	0	0	0.03502239	0	0

0	0	0	0	0	0	0	0.028228365	0	0
0	0	0	0	0	0	0	0	0	-0.38560413
0	0	0	0	0	0.031938448	0	0	0	0
0	-0.11714026	0	-0.00490644	0	0	0	0	0	0.091128044
0	0	0	0	0	0	0	0	0	0
0	0	0	0	0	0	0	0	0	-0.041062999
0	0	0	0	0	0	0	0	0	-0.024899895
0	0	0	0.042203303	0	-0.039361596	0	0	0	-0.016719475
0	0	0	0	0	0	0	0	0	0
0	0	0	0.041033995	0	-0.036772757	0	0	0	-0.016690429
0	0	0	0.0427911	0	-0.039839057	0	0	0	0
0	0	0	0	0	0	0	0	0	0
0	0	0	0.041623285	0	-0.037254229	0	0	0	0
0	0	0	0.045267704	0	-0.041038844	0	0	0	0
0	0	0	0.009068331	0	0	0	0	0	-0.37719175
0	0	0	0.045830315	0	-0.043208248	0	0	0	0
0	0	0	0.045772667	0	-0.043636373	0	0.001794719	0	0
0	0	0	0.04470001	0	-0.040954371	0	0	0	0
0	0	0	0.044599177	0	-0.041061675	0	0.00181477	0	0
0	0	0	0.007928997	0	0	0	0	0	-0.37796376
0	0	0	0	0	0	0	0	0	-0.38117657
0	0	0	0	0	-0.001659647	0	0	0	-0.38178658
0	0	0	0	0	0	0	0	0	-0.38176124
0	0	0	0	0	0	0	-0.000330407	0	-0.38109626
0	0	0	0	0	-0.003240396	0	0	0	-0.3823979
0	0	0	0	0	0	0	-0.001879951	0	-0.3801905
0	0	0	0	0	0	0	0	0	-0.38155244
0	0	0	0.019226091	0	0	0	-0.01590303	0	-0.020508222
0	0	0	0.019043518	0	0	0	-0.017232716	0	-0.020712371
0	0	0	0.019580938	0	0	0	-0.016196969	0	0
0	0	0	0.019400163	0	0	0	-0.017525284	0	0
0	0	0	0.00891713	0	0	0	0	0	-0.022497427
0	0	0	0.012540805	0	0	0	0	0	-0.019807345
0	0	0	0.021177132	0	0	0	-0.01755659	0	0
0	0	0	0.00910089	0	0	0	0	0	0
0	0	0	0.012107897	0	0	0	0	0	-0.019677555
0	0	0	0.013022668	0	0	0	0	0	0
0	0	0	0.02192403	0	0	0	-0.015303014	0	0
0	0	0	0.021861076	0	0	0	-0.016155692	0	0
0	0	0	0.012591328	0	0	0	0	0	0
0	0	0	0	0	-0.002096437	0	0	0	-0.024334997
0	0	0	0	0	0	0	-0.001343608	0	-0.024306998
0	0	0	0	0	0	0	0	0	-0.024628773
0	0	0	0.015498759	0	0	0	0	0	0
0	0	0	0	0	-0.004424459	0	0	0	-0.025561384
0	0	0	0	0	0	0	-0.004272071	0	-0.02544807
0	0	0	0	0	-0.002062982	0	0	0	0

0	0	0	0	0	0	0	-0.00136875	0	0
0	0	0	0	0	0	0	0	0	0
0	0	0	0	0	-0.002052187	0	0.000816567	0	-0.024111757
0	0	0	0	0	-0.005667582	0	0	0	-0.025583303
0	0	0	0	0	0	0	-0.005226955	0	-0.025384042
0	0	0	0	0	-0.003432126	0	0.000519939	0	-0.024317886
0	0	0	0	0	-0.004047404	0	0	0	0
0	0	0	0	0	0	0	-0.003930723	0	0
0	0	0	0	0	-0.001906055	0	0.000658116	0	0
0	0	0	0	0	-0.005285198	0	0	0	0
0	0	0	0	0	0	0	-0.004883708	0	0
0	0	0	0	0	-0.003278329	0	0.000356418	0	0
0	0	0	0	0	-0.003098257	0	0.001615384	0	0
0	0	0	0	0	-0.00194043	0	0	0	0
0	0	0	0	0	0	0	-0.000475294	0	0
0	0	0	0	0	-0.001840599	0	0.000933531	0	0
0	0	0	0	0	-0.003010325	0	0.000884057	0	0
0	-0.066128907	0	0.011878864	0	0	0	-0.038546944	0	0
0	-0.064281879	0	0.025227804	0	-0.018522774	0	-0.023847308	0	0
0	-0.07198934	0	0	0	-0.004036791	0	-0.029227931	0	0
0	-0.074345604	0	0.02328222	0	-0.041507853	0	0	0	0
0	0	0	0	0	0	0	0	0	-0.40923159
0	0	0	0	0	0.045623211	0	0	0	-0.3952126
0	0	0	0	0	0.05276509	0	-0.014732804	0	-0.019435963
0	0	0	0	0	0.052764289	0	-0.014779063	0	0
0	0	0	0	0	0.043349438	0	0	0	-0.021234272
0	0	0	0	0	0.043532253	0	0	0	0
0	0	0	0	0	0.050903938	0	-0.017231993	0	0
0	0	0	0	0	0	0	0	0	-0.41227216
0	0	0	0	0	0	0	0.01547983	0	-0.41713159
0	-0.043012825	0	0	0	0.10963725	0	0.11378926	0	0
0	0	0	0	0	0.14083468	0	0.11397816	0	-0.051701088
0	0	0	0	0	0.14056263	0	0.11330852	0	0
0	0	0	0	0	0	0	0.003684185	0	-0.023916322
0	0	0	0	0	0	0	0.003941333	0	0
0	0	0	0.034076942	0	0	0	-0.050628943	0	-0.34364842
0	0	0	0	0	0.12046933	0	0.11896793	0	0
0	0	0	0.027581113	0	0.11181367	0	0.11099749	0	0
0	0	0	0	0	0.12056517	0	0.11870828	0	0
0	0	0	0	0	0.13909455	0	0.12696396	0	-0.52260025
0	0	0	0.035511074	0	0	0	-0.05082344	0	-0.010387363
0	0	0	0.037110004	0	-0.003106537	0	-0.050928684	0	-0.010669794
0	0	0	0.035847803	0	0	0	-0.050600254	0	0
0	0	0	0.037656569	0	-0.003582795	0	-0.050874035	0	0
0	0	0	0.039169989	0	-0.004144005	0	-0.048524324	0	0
0	0	0	0	0	0.13703156	0	0.11343559	0	0
0	-0.032221095	0	0	0	0.098561888	0	0.1268752	0	0

0	-0.032021395	0	0	0	0.098665441	0	0.12659424	0	0
0	0	0	0	0	0.027188847	0	-0.048871125	0	-0.35290705
0	0	0	0	0	0.12101085	0	0.12732309	0	-0.03453512
0	0	0	0	0	0.12070944	0	0.12713877	0	-0.034592704
0	-0.029601932	0	0.020438499	0	0.099901103	0	0.11891442	0	0
0	0	0	0	0	0.12084571	0	0.12670801	0	0
0	0	0	0	0	0.12055307	0	0.12652135	0	0
0	-0.054243564	0	0.059693974	0	0	0	0.10869207	0	0
0	0	0	0.02904694	0	0.091430607	0	0.12353772	0	0
0	0	0	0.029465956	0	0.09054043	0	0.12337865	0	0
0	-0.027454257	0	0	0	0.12028925	0	0.12070235	0	-0.006763161
0	-0.033673067	0	0	0	0.11561223	0	0.12054867	0	0
0	-0.031933242	0	0	0	0.11690059	0	0.12053475	0	0
0	0	0	0	0	0.029402735	0	-0.050078939	0	-0.016970414
0	0	0	0.019576416	0	0.12168236	0	0.11908445	0	-0.029500907
0	0	0	0	0	0.029558086	0	-0.04978449	0	0
0	0	0	0	0	0	0	-0.029773084	0	-0.37653535
0	0	0	0.020452055	0	0.12061649	0	0.11849351	0	0
0	0	0	0	0	0.13975017	0	0.12081612	0	-0.03322638
0	0	0	0.044872898	0	0.097681188	0	0.11731469	0	0
0	-0.065311597	0	0	0	0	0	0.12615762	0	0
0	-0.064752759	0	0	0	0	0	0.12582654	0	0
0	0	0	0	0	0.13949264	0	0.12025385	0	0
0	0	0	0	0	0.11877683	0	0.1252694	0	0
0	0	0	0	0	0.11918612	0	0.1252239	0	0
0	0	0	0	0	0.11893299	0	0.12500638	0	0
0	0	0	0.10946939	0	0	0	0.10750498	0	0
0	0	0	0.068483168	0	0	0	0.11902798	0	0
0	-0.037730575	0	0.052821717	0	0	0	0.12727453	0	0
0	0	0	0.06856671	0	0	0	0.11912341	0	0
0	-0.038341451	0	0.052608381	0	0	0	0.1275049	0	0
0	0	0	0.015790386	0	0	0	0	0	-0.38330117
0	0	0	0.037125112	0	-0.043265703	0	0	0	-0.39510872
0	0	0	0.088680058	0	0	0	0.12516152	0	0
0	0	0	0.089104857	0	0	0	0.1253319	0	0
0	0	0	0.037227718	0	-0.029435398	0	0	0	-0.00237887
0	0	0	0.037837425	0	-0.029637511	0	0	0	0
0	0	0	0	0	0.00227637	0	0	0	-0.3921983
0	-0.033820085	0	0.086467542	0	0	0	0.11656108	0	0
0	-0.073962232	0	0.055230264	0	0	0	0.11588595	0	0
0	0	0	0.064884219	0	0	0	0.12704191	0	-0.020673152
0	0	0	0.065079198	0	0	0	0.12711241	0	-0.020401698
0	0	0	0.065305931	0	0	0	0.12653465	0	0
0	0	0	0	0	0.13940896	0	0	0	-0.42767852
0	0	0	0.065498256	0	0	0	0.1266098	0	0
0	-0.070525918	0	0.056192651	0	0	0	0.11525583	0	0.047972323
0	-0.10406652	0	0	0	0	0	0.11371705	0	0

0	0	0	0.067017803	0	0	0	0.12561809	0	0
0	-0.082602517	0	0	0	0	0	0.13187668	0	0
0	-0.039377451	0	0.025835838	0	0.087723745	0	0	0	0
0	-0.0815919	0	0	0	0	0	0.13155926	0	0
0	-0.10110316	0	0	0	0	0	0.1130187	0	0.050953128
0	0	0	0.023225108	0	0.11852889	0	0	0	-0.046024003
0	-0.07911456	0	0	0	0	0	0.13165032	0	0.044042205
0	0	0	0.079425143	0	0	0	0.10663836	0	-0.031050699
0	-0.026158642	0	0	0	0.12142733	0	0	0	-0.025374497
0	-0.078031605	0	0	0	0	0	0.13135871	0	0.042890983
0	-0.042301457	0	0	0	0.10182802	0	0	0	0
0	-0.042206654	0	0	0	0.10246685	0	0	0	0
0	-0.031290414	0	0	0	0.11758111	0	0	0	0
0	0	0	0.079790929	0	0	0	0.10620871	0	0
0	0	0	0.023974886	0	0.11763814	0	0	0	0
0	0	0	0	0	0.13169896	0	0	0	-0.051067295
0	0	0	0	0	0.13192357	0	0	0	-0.051044192
0	-0.042276721	0	0	0	0.10930074	0	0	0	0
0	0	0	0	0	0.14005848	0	0	0	-0.050724486
0	0	0	0.10473447	0	0	0	0.11474286	0	-0.009338756
0	0	0	0	0	0.13149991	0	0	0	0
0	0	0	0	0	0.1317245	0	0	0	0
0	0	0	0.10518167	0	0	0	0.11448021	0	0
0	0	0	0	0	0.13985851	0	0	0	0
0	-0.054462254	0	0	0	0	0	0.13450934	0	0
0	-0.05451926	0	0	0	0	0	0.13366748	0	0
0	-0.055129008	0	0	0	0	0	0.1340094	0	0
0	0	0	0.058099978	0	0.085015405	0	0	0	0
0	-0.042541025	0	0.087605134	0	0	0	0	0	0
0	-0.076043143	0	0	0	0	0	0.11316409	0	0
0	0	0	0.079584496	0	0	0	0.11923063	0	-0.50393191
0	0	0	0.0346031	0	0.095332836	0	0	0	0
0	0	0	0.034766764	0	0.095110005	0	0	0	0
0	-0.10043844	0	0	0	0	0	0.1258375	0	-0.4233636
0	-0.048288264	0	0.05647977	0	0	0	0	0	0
0	-0.047842892	0	0.056690647	0	0	0	0	0	0
0	0	0	0	0	0	0	0.12948005	0	-0.047573551
0	0	0	0	0	0	0	0.12967564	0	-0.047502693
0	0	0	0	0	0	0	0.12882367	0	0
0	0	0	0	0	0	0	0.1290198	0	0
0	-0.071213883	0	0.058232951	0	0	0	0	0	0
0	0	0	0.10023423	0	0	0	0	0	0
0	0	0	0.10048138	0	0	0	0	0	0
0	-0.068464872	0	0.058968548	0	0	0	0	0	0.029965958
0	0	0	0	0	0.12868776	0	0	0	0
0	0	0	0	0	0.12864678	0	0	0	0
0	0	0	0	0	0.12894281	0	0	0	0

0	-0.10408296	0	0	0	0	0	0.12001252	0	0
0	0	0	0.10856959	0	0	0	0	0	-0.026594337
0	0	0	0.10893593	0	0	0	0	0	0
0	0	0	0	0	0	0	0.14233024	0	-0.52904239
0	-0.10127917	0	0	0	0	0	0.11919616	0	0.068721261
0	0	0	0	0	0	0	0.14252949	0	-0.52886527
0	0	0	0.026243153	0	0.11396023	0	0	0	-0.39116518
0	-0.027021431	0	0	0	0.11900276	0	0	0	-0.36869569
0	-0.02662528	0	0.028470839	0	0.083246339	0	0	0	0
0	-0.026487013	0	0.028710515	0	0.082883931	0	0	0	0
0	-0.024225091	0	0.040391518	0	0.085288276	0	0	0	0
0	0	0	0	0	0.12916317	0	0	0	-0.39349865
0	0	0	0	0	0.12897773	0	0	0	-0.3936243
0	0	0	0.070995236	0	0	0	0	0	-0.034413864
0	0	0	0.071150181	0	0	0	0	0	-0.034321228
0	0	0	0	0	0	0	0.12854794	0	0
0	-0.03297294	0	0.027674415	0	0.089621369	0	0	0	0
0	-0.028347379	0	0.026980177	0	0.093745824	0	0	0	0.002542417
0	0	0	0	0	0.13797192	0	0	0	-0.3969002
0	0	0	0.071331376	0	0	0	0	0	0
0	0	0	0	0	0	0	0.12808368	0	0
0	0	0	0.071485864	0	0	0	0	0	0
0	0	0	0	0	0	0	0.12831312	0	0
0	0	0	0.028284879	0	0.10136802	0	0	0	-0.025237535
0	0	0	0.028026733	0	0.10205093	0	0	0	-0.025233795
0	0	0	0.041656663	0	0.10122336	0	0	0	-0.021662912
0	0	0	0.028925402	0	0.10059391	0	0	0	0
0	0	0	0.028667288	0	0.10127621	0	0	0	0
0	0	0	0.042813906	0	0.099995792	0	0	0	0
0	0	0	0.051742278	0	0.079745952	0	0	0	0
0	0	0	0.05161428	0	0.080211447	0	0	0	0
0	-0.030026374	0	0	0	0.10796095	0	0	0	0
0	-0.02993683	0	0	0	0.1080111	0	0	0	0
0	-0.026671311	0	0	0	0.11080248	0	0	0	-0.004699458
0	-0.031345857	0	0	0	0.10742265	0	0	0	0
0	-0.029757909	0	0	0	0.10852168	0	0	0	0
0	-0.026371805	0	0	0	0.11103699	0	0	0	-0.005008171
0	-0.031062466	0	0	0	0.10769066	0	0	0	0
0	-0.032510064	0	0	0	0.11520871	0	0	0	0
0	-0.02779509	0	0	0	0.11876391	0	0	0	-0.003158798
0	0	0	0.033740388	0	0.094652426	0	0	0	0
0	0	0	0	0	0.12893798	0	0	0	-0.030518809
0	0	0	0	0	0.1286925	0	0	0	-0.030558779
0	0	0	0	0	0.1289716	0	0	0	-0.030475305
0	-0.087114207	0	0	0	0	0	0	0	0
0	0	0	0.074388695	0	0	0	0	0	0
0	0	0	0	0	0.12878792	0	0	0	0

0	0	0	0	0	0.12854972	0	0	0	0
0	0	0	0	0	0.12882205	0	0	0	0
0	-0.086545063	0	0	0	0	0	0	0	0
0	0	0	0	0	0	0	0.13640699	0	-0.030883711
0	0	0	0	0	0	0	0.13570158	0	-0.031046927
0	-0.084446364	0	0	0	0	0	0	0	0.033801579
0	0	0	0.081791392	0	0	0	0	0	-0.4141813
0	-0.028438088	0	0.079551874	0	0	0	0	0	0
0	-0.028748575	0	0.079706348	0	0	0	0	0	0
0	0	0	0	0	0	0	0.13593986	0	-0.030719919
0	-0.083825431	0	0	0	0	0	0	0	0.033241598
0	0	0	0	0	0	0	0.1358229	0	0
0	0	0	0	0	0	0	0.13511903	0	0
0	-0.059130678	0	0.055302878	0	0	0	0	0	0
0	0	0	0	0	0	0	0.1353624	0	0
0	-0.060035026	0	0.055068279	0	0	0	0	0	0
0	-0.05785818	0	0.079905718	0	0	0	0	0	0
0	-0.056242758	0	0.055879283	0	0	0	0	0	0.03539308
0	-0.057200107	0	0.055643571	0	0	0	0	0	0.036372918
0	-0.066741167	0	0	0	0	0	0	0	0
0	-0.066393714	0	0	0	0	0	0	0	0
0	-0.055082856	0	0.080976823	0	0	0	0	0	0.040926522
0	-0.066276045	0	0	0	0	0	0	0	0
0	0	0	0	0	0.12701208	0	0	0	0
0	-0.067258479	0	0.060070885	0	0	0	0	0	-0.31745045
0	-0.099913072	0	0	0	0	0	0	0	-0.32916669
0	0	0	0	0	0	0	0.13405208	0	0
0	-0.035493511	0	0.059825662	0	0	0	0	0	0
0	0	0	0.093287309	0	0	0	0	0	-0.01085277
0	0	0	0.09373349	0	0	0	0	0	0
0	0	0	0.093793587	0	0	0	0	0	-0.010599754
0	0	0	0.094232092	0	0	0	0	0	0
0	0	0	0.09499829	0	0	0	0	0	0
0	-0.10279704	0	0	0	0	0	0	0	0
0	0	0	0.10630147	0	0	0	0	0	-0.37370294
0	0	0	0.071580525	0	0	0	0	0	-0.37606149
0	-0.10058795	0	0	0	0	0	0	0	0.051438858
0	0	0	0.071853779	0	0	0	0	0	-0.37596407
0	-0.040582847	0	0.057715602	0	0	0	0	0	0
0	-0.040165136	0	0.057815112	0	0	0	0	0	0
0	-0.060450665	0	0.056182059	0	0	0	0	0	0
0	-0.060158004	0	0.056153818	0	0	0	0	0	0
0	-0.057616433	0	0.056927599	0	0	0	0	0	0.039447139
0	0	0	0	0	0	0	0.11860751	0	-0.5532704
0	-0.057285227	0	0.056888732	0	0	0	0	0	0.039136054
0	0	0	0.07163464	0	0	0	0	0	-0.015239254
0	-0.036045816	0	0.056394792	0	0	0	0.02256769	0	0

0	0	0	0.071933422	0	0	0	0	0	0
0	-0.033910296	0	0.079083084	0	-0.022352557	0	0	0	0
0	-0.035682955	0	0.056635579	0	0	0	0.021043792	0	0
0	-0.033631017	0	0.079805776	0	-0.023844287	0	0	0	0
0	0	0	0.10794518	0	-0.052821711	0	0.044718556	0	0
0	0	0	0.10853556	0	-0.054518781	0	0.044160167	0	0
0	0	0	0.1077525	0	-0.04045298	0	0	0	0
0	-0.033896849	0	0.062692491	0	0	0	0	0	0
0	-0.083682903	0	0	0	0	0	0	0	-0.30588156
0	0	0	0.10821098	0	-0.041837496	0	0	0	0
0	-0.082840188	0	0	0	0	0	0	0	-0.30710015
0	-0.078696242	0	0.08365858	0	-0.030864461	0	0	0	0
0	-0.077012243	0	0.059712784	0	0	0	0	0	0
0	-0.07127967	0	0.063091605	0	0	0	0	0	-0.30359255
0	-0.087502132	0	0	0	0	0	0.035032562	0	0
0	-0.088544359	0	0	0	0	0	0	0	0
0	-0.076185183	0	0.084031422	0	-0.030141859	0	0	0	0.064812964
0	-0.086612561	0	0	0	0	0	0	0	0
0	-0.085774423	0	0	0	0	0	0	0	0
0	-0.074521287	0	0.060576159	0	0	0	0	0	0.052955223
0	0	0	0.078420906	0	0	0	0	0	-0.36288721
0	-0.087057459	0	0	0	0	0	0.03343794	0	0
0	-0.085748232	0	0	0	0	0	0	0	0
0	-0.084894153	0	0	0	0	0	0.034756848	0	0.055009359
0	0	0	0.07791036	0	0	0	0	0	-0.36337341
0	-0.08817546	0	0	0	0	0	0	0	0
0	0	0	0.076125671	0	0	0	0	0	-0.018102396
0	-0.086091816	0	0	0	0	0	0	0	0.047306824
0	-0.08418246	0	0	0	0	0	0	0	0.053066234
0	-0.047437842	0	0.098059756	0	-0.040866053	0	0	0	0
0	-0.083278779	0	0	0	0	0	0	0	0.052083666
0	-0.076618225	0	0.059764534	0	0	0	0.010949849	0	0
0	0	0	0.076637779	0	0	0	0	0	0
0	0	0	0.075832294	0	0	0	0	0	-0.017880634
0	-0.08327753	0	0	0	0	0	0	0	0.052378703
0	-0.084406144	0	0	0	0	0	0.033156142	0	0.054557721
0	0	0	0.07687559	0	0	0	0.010790569	0	0
0	-0.085679279	0	0	0	0	0	0	0	0.047243624
0	0	0	0.076348108	0	0	0	0	0	0
0	0	0	0.079008504	0	0	0	0	0	0
0	0	0	0.076819075	0	0	0	0.009628565	0	0
0	-0.073923203	0	0.0608476	0	0	0	0.010240056	0	0.058830803
0	0	0	0.074035114	0	0	0	0.018874339	0	-0.011775766
0	0	0	0	0	0	0	0.11217294	0	-0.042007457
0	0	0	0.094560425	0	-0.02131172	0	0	0	-0.008087609
0	0	0	0.096809035	0	-0.023356628	0	0	0	0
0	0	0	0.09512555	0	-0.02296472	0	0	0	-0.008100804

0	0	0	0.074526985	0	0	0	0.018708776	0	0
0	0	0	0.095309868	0	-0.02173861	0	0	0	0
0	-0.085099963	0	0	0	0	0	0	0	-0.2994421
0	0	0	0.095860559	0	-0.023377309	0	0	0	0
0	0	0	0.07574554	0	0	0	0.018281538	0	0
0	0	0	0.073893311	0	0	0	0.017443017	0	-0.01185521
0	-0.092626328	0	0.03780564	0	-0.042967626	0	0	0	0
0	-0.10906659	0	0	0	-0.040626968	0	0.039092961	0	0
0	-0.059268267	0	0	0	0	0	0.022723847	0	0
0	0	0	0.074390651	0	0	0	0.017281045	0	0
0	-0.084483699	0	0	0	0	0	0	0	-0.30046629
0	-0.092170337	0	0.038522454	0	-0.044597508	0	0	0	0
0	0	0	0	0	0	0	0.11162854	0	0
0	0	0	0.078298331	0	0	0	0	0	-0.011045241
0	-0.10828724	0	0	0	-0.040934981	0	0.037821003	0	0
0	0	0	0	0	0	0	0	0	-0.42096971
0	0	0	0.078733739	0	0	0	0	0	0
0	-0.058778272	0	0	0	0	0	0.021365507	0	0
0	-0.090306598	0	0.037478753	0	-0.042435068	0	0	0	0.067259301
0	0	0	0	0	0	0	0	0	-0.42092252
0	-0.10292917	0	0	0	-0.026296282	0	0	0	0
0	-0.10586397	0	0	0	-0.039037854	0	0.037385881	0	0.075097698
0	-0.089816409	0	0.038212272	0	-0.044094759	0	0	0	0.066849329
0	-0.10017263	0	0	0	0	0	0	0	-0.29952856
0	-0.083663257	0	0	0	0.008242525	0	0	0	0
0	-0.059941798	0	0	0	0.002927757	0	0	0	0
0	-0.060646388	0	0	0	0	0	0	0	0
0	-0.10250785	0	0	0	-0.026925474	0	0	0	0
0	-0.087390056	0	0	0	0	0	0	0	0
0	-0.091690075	0	0	0	-0.010518559	0	0	0	-0.29214146
0	-0.10506191	0	0	0	-0.039366808	0	0.036133631	0	0.074279899
0	-0.044194394	0	0.071168808	0	0	0	-0.0065203	0	0
0	-0.083623673	0	0	0	0.007315846	0	0	0	0
0	-0.10048407	0	0	0	-0.025975576	0	0	0	0.062804556
0	-0.09144603	0	0	0	-0.01155852	0	0	0	-0.29294914
0	-0.0596411	0	0	0	0.002103222	0	0	0	0
0	-0.0808434	0	0	0	0.009101886	0	0	0	0.051305359
0	-0.054575388	0	0	0	0	0	0	0	0
0	-0.084974958	0	0	0	0	0	0	0	0.055147812
0	-0.10002351	0	0	0	-0.026615177	0	0	0	0.062577961
0	-0.088314467	0	0	0	0	0	0	0	-0.29391328
0	-0.054447391	0	0	0	0	0	0.034910292	0	0
0	-0.055479745	0	0	0	-0.003364184	0	0.037238059	0	0
0	-0.080778496	0	0	0	0.008153847	0	0	0	0.051228226
0	-0.040773821	0	0.094270602	0	-0.033166575	0	0.020370384	0	0
0	-0.087949856	0	0	0	0	0	0.002184868	0	-0.29452336
0	-0.087527518	0	0	0	0	0	0	0	-0.29339341

0	-0.087435538	0	0.000893022	0	0	0	0	0	-0.29310127
0	-0.055209716	0	0	0	-0.004161638	0	0.036019645	0	0
0	-0.087720761	0	0	0	0	0	0	0	-0.29453874
0	-0.087351929	0	0	0	0	0	0.000694	0	-0.29446298
0	-0.087260601	0	7.23E-05	0	0	0	0	0	-0.29402208
0	-0.093861009	0	0	0	-0.010714738	0	0	0	0
0	0	0	0	0	0	0	0	0	-0.045683919
0	0	0	0	0	0	0	0	0	-0.04562219
0	0	0	0	0	0	0	0	0	-0.045542882
0	0	0	0	0	0	0	0.12495083	0	-0.53295923
0	0	0	0	0	0	0	0	0	0
0	-0.090760251	0	0	0	0	0	-0.003134178	0	0
0	-0.091897705	0	-0.002514826	0	0	0	0	0	0
0	0	0	0	0	0	0	0	0	0
0	-0.091435587	0	0	0	-0.010471488	0	0	0	0.061490716
0	-0.09066442	0	0	0	0	0	0	0	0
0	-0.090224478	0	0	0	0	0	0.001646258	0	0
0	-0.090540948	0	-0.000310435	0	0	0	0.001935266	0	0
0	-0.10692554	0	0	0	0	0	0	0	-0.29806422
0	-0.0897285	0	0.000924245	0	0	0	0	0	0
0	0	0	0	0	0	0	0	0	0
0	-0.090196216	0	0	0	0	0	-0.004226866	0	0
0	-0.091406714	0	-0.002668421	0	0	0	0	0	0
0	-0.061377263	0	0.031893793	0	-0.049344176	0	0	0	0
0	-0.088471234	0	0	0	0	0	-0.003718868	0	0.05684158
0	-0.089564293	0	-0.002438096	0	0	0	0	0	0.057724754
0	-0.089836694	0	-8.12E-07	0	0	0	0.000209211	0	0
0	-0.088328524	0	0	0	0	0	0	0	0.057487739
0	-0.087847602	0	0	0	0	0	0.001431048	0	0.058403784
0	-0.087339194	0	0.001045018	0	0	0	0	0	0.058110072
0	-0.087975931	0	0.000230885	0	0	0	0.001301415	0	0.058599095
0	-0.060655527	0	0.032969425	0	-0.051109127	0	0	0	0
0	-0.050464568	0	0	0	0.017525084	0	0	0	0
0	-0.1073647	0	0	0	0	0	0.023140497	0	-0.30759085
0	-0.087868612	0	0	0	0	0	-0.004807996	0	0.05644281
0	-0.089028528	0	-0.002591998	0	0	0	0	0	0.05757169
0	-0.11016335	0	0	0	0	0	0.011610182	0	0
0	-0.087234006	0	0.000536445	0	0	0	-0.000419869	0	0.057945288
0	-0.057353281	0	0.031529795	0	-0.049951928	0	0.021302411	0	0
0	-0.10914756	0	0	0	0.000283646	0	0	0	0
0	-0.056730934	0	0.032702088	0	-0.051936927	0	0.020687451	0	0
0	0	0	0.093125208	0	0	0	0	0	-0.37717506
0	-0.053032226	0	0.032543028	0	-0.035277052	0	0	0	0
0	-0.066743773	0	0	0	-0.031116925	0	0.014486003	0	0
0	0	0	0.10920121	0	-0.019783109	0	0	0	-0.3680362
0	-0.10813301	0	0	0	0	0	0.010715048	0	0.074518518
0	-0.098817051	0	0	0	0.012179683	0	0	0	-0.29390405

0	0	0	0.1180555	0	-0.033459436	0	0	0	-0.006997966
0	-0.064897919	0	0	0	-0.022558847	0	0	0	0
0	-0.10671535	0	0	0	0.001225304	0	0	0	0.071903975
0	0	0	0.12059398	0	-0.036601351	0	0.004942346	0	0
0	0	0	0.11858989	0	-0.033652414	0	0	0	0
0	-0.06610719	0	0	0	-0.031607855	0	0.01384712	0	0
0	-0.11106064	0	0	0	-0.002346159	0	0.02310607	0	0
0	0	0	0.093999891	0	0	0	0.002282082	0	-0.37200977
0	-0.060975797	0	0	0	-0.02441602	0	0.022425587	0	0
0	-0.10784671	0	0	0	-0.000318192	0	0.02130913	0	0.080809213
0	0	0	0	0	0	0	0	0	0
0	0	0	0.094745961	0	0	0	-0.009341414	0	-0.009714471
0	-0.11977468	0	0.034199207	0	-0.047667348	0	0	0	-0.26835283
0	0	0	0.095105712	0	0	0	-0.009091313	0	0
0	0	0	0.11479808	0	-0.028711709	0	0.012817265	0	0
0	0	0	0.11400438	0	-0.028061733	0	0.012525394	0	1.94E-05
0	-0.057915361	0	0	0	0	0	-0.008144885	0	0
0	-0.057542701	0	0.001956501	0	0	0	-0.009418765	0	0
0	-0.057886842	0	-0.002078264	0	0	0	0	0	0
0	-0.054320128	0	0.004197187	0	0	0	-0.003048802	0	0
0	-0.1283807	0	0.03447545	0	-0.057442898	0	0	0	0
0	-0.056801486	0	0.002332403	0	0	0	-0.01064989	0	0
0	-0.12562236	0	0.034555816	0	-0.053464881	0	0.00672182	0	0
0	-0.12715243	0	0	0	-0.02790665	0	0	0	-0.27523976
0	-0.12664329	0	0.034125979	0	-0.05683201	0	0	0	0.10167526
0	-0.1249843	0	0	0	-0.024752147	0	0.008019247	0	-0.27829374
0	-0.12286964	0	0.03422322	0	-0.051432087	0	0.00471759	0	0.10219855
0	0	0	0	0	0	0	0	0	-0.39191788
0	0	0	0	0	0	0	0.037530934	0	-0.41712105
0	0	0	0	0	0	0	0	0	-0.39097855
0	0	0	0	0	0	0	0.035688902	0	-0.41597393
0	-0.12976142	0	0	0	-0.028633678	0	0.001191795	0	0
0	0	0	0	0	0	0	0	0	-0.39169098
0	0	0	0	0	0.032442502	0	0	0	-0.38163732
0	0	0	0	0	0.030877182	0	0	0	-0.38221484
0	-0.1118681	0	0	0	0	0	-0.018359712	0	-0.26711806
0	-0.10899405	0	0.005177951	0	0	0	-0.017228213	0	-0.26642334
0	0	0	0	0	0	0	0	0	-0.39676992
0	0	0	0	0	0	0	0	0	-0.39661996
0	0	0	0	0	0.025159263	0	0	0	-0.030723262
0	-0.12703689	0	0	0	-0.02699441	0	-0.000895073	0	0.095620819
0	0	0	0	0	0	0	0.026433644	0	-0.030436536
0	-0.065520762	0	0	0	0.017258525	0	-0.004479503	0	0
0	0	0	0	0	0.020728368	0	0.022377872	0	-0.024781996
0	0	0	0	0	0.023862449	0	0	0	-0.030559698
0	0	0	0	0	0.025417649	0	0	0	0
0	-0.11682289	0	-0.00517374	0	0	0	0	0	-0.2756058

0	0	0	0	0	0	0	0.025028813	0	-0.030281521
0	-0.11149211	0	0.004629754	0	0	0	-0.021687412	0	0
0	0	0	0	0	0	0	0.026601761	0	0
0	0	0	0	0	0.020844709	0	0.022182669	0	0
0	0	0	0	0	0.019481068	0	0.021416245	0	-0.024896973
0	0	0	0	0	0.024126803	0	0	0	0
0	0	0	0	0	0	0	0	0	-0.026439647
0	0	0	0	0	0	0	0.025200865	0	0
0	0	0	0	0	0.019605474	0	0.021218917	0	0
0	0	0	0	0	0	0	0.035080552	0	-0.024988153
0	0	0	0	0	0	0	0	0	0
0	0	0	0	0	0.03190607	0	0	0	-0.024837141
0	0	0	0	0	0	0	0.034960862	0	0
0	0	0	0	0	0.03192358	0	0	0	0
0	-0.10944951	0	0.005220233	0	0	0	-0.022906384	0	0.081941793
0	0	0	0	0	0.019035855	0	0.018668792	0	0
0	0	0	0	0	0	0	0	0	-0.033315694
0	0	0	0	0	0	0	0	0	-0.43892291
0	0	0	0	0	0.019805478	0	0.022572318	0	0
0	0	0	0	0	0.018020347	0	0.017898977	0	0
0	0	0	0	0	0.026945895	0	0	0	0
0	0	0	0	0	0	0	0	0	0
0	0	0	0	0	0	0	0.029426614	0	0
0	0	0	0	0	0	0	0	0	-0.38546212
0	0	0	0.042078385	0	-0.039446547	0	0	0	-0.37313125
0	0	0	0.040916807	0	-0.036875634	0	0	0	-0.37283955
0	0	0	0.041733213	0	-0.038117326	0	0	0	-0.016833405
0	0	0	0.042312473	0	-0.038586449	0	0	0	0
0	0	0	0.042285142	0	-0.042301576	0	0	0	-0.018089968
0	0	0	0.018012896	0	0	0	-0.014281045	0	-0.36306134
0	0	0	0.042254244	0	-0.040671629	0	0.001709968	0	-0.016711468
0	0	0	0.041104784	0	-0.039989638	0	0	0	-0.018216465
0	0	0	0.017837372	0	0	0	-0.01562846	0	-0.36238505
0	0	0	0.041074342	0	-0.038073135	0	0.001730264	0	-0.01668198
0	0	0	0.042866768	0	-0.042438927	0	0	0	0
0	0	0	0.042840135	0	-0.041081702	0	0.001620762	0	0
0	0	0	0.041690353	0	-0.040134683	0	0	0	0
0	0	0	0.041664239	0	-0.038489715	0	0.001640796	0	0
0	0	0	0.045338512	0	-0.043136551	0	0.002771662	0	0
0	0	0	0.04541583	0	-0.042345939	0	0	0	0
0	0	0	0.008716647	0	0	0	0	0	-0.37721091
0	0	0	0.012249045	0	0	0	0	0	-0.37410369
0	0	0	0.045840482	0	-0.043978663	0	0.001309723	0	0
0	0	0	0.011818548	0	0	0	0	0	-0.37417941
0	0	0	0.044705476	0	-0.041584112	0	0.001087357	0	0
0	0	0	0	0	-0.002298081	0	0	0	-0.38180235
0	0	0	0	0	0	0	0	0	-0.3814997

0	0	0	0	0	0	0	-0.000627536	0	-0.38073093
0	0	0	0	0	-0.004776546	0	0	0	-0.38402231
0	0	0	0	0	-0.004232918	0	0.00351508	0	-0.3849703
0	0	0	0	0	0	0	-0.002865542	0	-0.38027512
0	0	0	0	0	-0.006013149	0	0	0	-0.38438234
0	0	0	0	0	-0.005627175	0	0.003220891	0	-0.38532697
0	0	0	0	0	0	0	-0.003826889	0	-0.3795661
0	0	0	0.018870042	0	0	0	-0.015900415	0	-0.02076413
0	0	0	0.019227219	0	0	0	-0.016193855	0	0
0	0	0	0.019073304	0	0	0	-0.016481523	0	-0.020827951
0	0	0	0.019012673	0	0	0	-0.017353086	0	-0.020778163
0	0	0	0.019519607	0	0	0	-0.016429152	0	0
0	0	0	0.019458405	0	0	0	-0.01729751	0	0
0	0	0	0.012299618	0	0	0	0	0	-0.019979029
0	0	0	0.012782283	0	0	0	0	0	0
0	0	0	0.021645721	0	0	0	-0.015115667	0	0
0	0	0	0	0	-0.005121709	0	0	0	-0.025810675
0	0	0	0	0	0	0	-0.004287346	0	-0.025527092
0	0	0	0	0	-0.003300566	0	0.001638959	0	-0.024335651
0	0	0	0	0	-0.004741028	0	0	0	0
0	0	0	0	0	-0.003502726	0	-0.001630513	0	-0.025694556
0	0	0	0	0	0	0	-0.003945589	0	0
0	0	0	0	0	-0.003146679	0	0.001474651	0	0
0	0	0	0	0	-0.004711739	0	-0.001673713	0	-0.025719387
0	0	0	0	0	-0.003188412	0	-0.001520451	0	0
0	0	0	0	0	-0.004389398	0	-0.001570333	0	0
0	-0.068998104	0	0.025231172	0	-0.024576126	0	-0.030630946	0	0
0	0	0	0	0	-0.003003327	0	0.001804775	0	0
0	0	0	0	0	0.051130159	0	-0.011949362	0	-0.38349586
0	0	0	0	0	0.042857444	0	0	0	-0.39908226
0	0	0	0	0	0.050689608	0	-0.019977255	0	-0.023352654
0	0	0	0	0	0.050803516	0	-0.019797353	0	0
0	0	0	0	0	0.14023088	0	0.12021416	0	-0.54358029
0	-0.043045825	0	0	0	0.093258009	0	0.11962162	0	0
0	0	0	0	0	0.12367725	0	0.12014628	0	-0.052543831
0	-0.042816108	0	0	0	0.093843516	0	0.11925861	0	0
0	0	0	0	0	0	0	0.006095293	0	-0.41677313
0	0	0	0	0	0.12374618	0	0.11987762	0	-0.052509947
0	-0.04073382	0	0.02001092	0	0.092963861	0	0.11218859	0	0
0	0	0	0	0	0.12346598	0	0.11944049	0	0
0	-0.026347801	0	0	0	0.1222105	0	0.11396114	0	-0.026198517
0	0	0	0	0	0.12354011	0	0.11917515	0	0
0	0	0	0.017193033	0	0.12500021	0	0.11258593	0	-0.048240469
0	-0.03287711	0	0	0	0.11730453	0	0.11369171	0	0
0	-0.042885756	0	0	0	0.10988366	0	0.11397222	0	0
0	0	0	0	0	0.14112248	0	0.11428975	0	-0.051768215
0	0	0	0.018169701	0	0.12382814	0	0.11188255	0	0

0	0	0	0	0	0.14084858	0	0.11361718	0	0
0	-0.049059699	0	0.050073277	0	0	0	0.11993074	0	0
0	-0.048464587	0	0.050410381	0	0	0	0.11965775	0	0
0	0	0	0.030188267	0	0.091650303	0	0.11714675	0	0
0	0	0	0.05232194	0	0.091050357	0	0.11086994	0	0
0	0	0	0.030528905	0	0.091142959	0	0.11693873	0	0
0	0	0	0	0	0.12051344	0	0.13348401	0	-0.52241356
0	0	0	0	0	0.12021286	0	0.13330162	0	-0.5225299
0	-0.044424109	0	0.084582368	0	0	0	0.10935521	0	0
0	0	0	0.037029553	0	-0.004826399	0	-0.04881437	0	-0.34507185
[truncated: 455,557 more chars]
